# Endogenous insensitivity to the Orco agonist VUAA1 reveals novel olfactory receptor complex properties in the specialist fly *Mayetiola destructor*

**DOI:** 10.1038/s41598-018-21631-3

**Published:** 2018-02-22

**Authors:** Jacob A. Corcoran, Yonathan Sonntag, Martin N. Andersson, Urban Johanson, Christer Löfstedt

**Affiliations:** 1 Department of Biology, Lund University, Lund, Sweden; 2 Division of Biochemistry and Structural Biology, Department of Chemistry, Lund University, Lund, Sweden

## Abstract

Insect olfactory receptors are routinely expressed in heterologous systems for functional characterisation. It was recently discovered that the essential olfactory receptor co-receptor (Orco) of the Hessian fly, *Mayetiola destructor* (Mdes), does not respond to the agonist VUAA1, which activates Orco in all other insects analysed to date. Here, using a mutagenesis-based approach we identified three residues in MdesOrco, located in different transmembrane helices as supported by 3D modelling, that confer sensitivity to VUAA1. Reciprocal mutations in *Drosophila melanogaster* (Dmel) and the noctuid moth *Agrotis segetum* (Aseg) Orcos diminish sensitivity of these proteins to VUAA1. Additionally, mutating these residues in DmelOrco and AsegOrco compromised odourant receptor (OR) dependent ligand-induced Orco activation. In contrast, both wild-type and VUAA1-sensitive MdesOrco were capable of forming functional receptor complexes when coupled to ORs from all three species, suggesting unique complex properties in *M. destructor*, and that not all olfactory receptor complexes are “created” equal.

## Introduction

The sense of smell in insects has evolved to detect and discriminate between a vast number of odourants as a prerequisite to find suitable food, mates and oviposition sites, and to avoid dangerous situations and non-preferred habitats. Attractive and repellent odourants are primarily detected by a divergent repertoire of odourant receptors (OR) that has expanded to various degrees in different taxa due to different ecological needs^1–3^. Insect OR complexes are comprised of an olfactory receptor co-receptor (Orco) subunit and a ligand-binding OR subunit in an unknown stoichiometry^4–6^. Insect ORs and Orcos are members of a novel protein family that likely evolved from insect gustatory receptors^7–9^. The ORs and Orcos are seven-transmembrane α-helical proteins similar to G-protein coupled receptors (GPCRs), but in comparison they have an inverted membrane topology with an extracellular C-terminus, and the two protein families share no homology^6,10,11^. *In vivo*, these proteins are expressed in olfactory sensory neurons (OSN) housed primarily in an insect’s antennae, with the Orco subunits generally being co-expressed with a single, neuron-specific OR counterpart^12,13^. The Orco subunit is highly conserved between insect species, whereas ORs are much more divergent both within and across insect species.

Together these proteins function to detect volatile molecules from the environment, however it remains unclear exactly how Orco and OR proteins interact with each other to form a functional complex, both in terms of the nature of the interaction and in terms of the overall stoichiometry of the subunits within the complex (reviewed in ref.^14^). Similarly, it is unclear how signals from the environment are transduced through these complexes in OSNs to cause neuronal depolarisation; there is on-going debate regarding their reliance on ionotropic and metabotropic signalling pathways^15–19^. Experimental evidence suggests that both subunits are required for proper signal detection and transduction *in vivo*^4,6^, however, *in vitro*, signal transduction can still be achieved through co-expression of the OR subunit with a member of the G-protein family^20–22^. In pioneering experiments using cyclic nucleotides^16^, and later using the Orco agonist VUAA1 (N-(4-ethylphenyl)-2-((4-ethyl-5-(3-pyridinyl)-4H-1,2,4-triazol-3-yl)thio)acetamide), it has been clearly demonstrated *in vitro* that the Orco subunits alone can form functional non-specific cation channels^23–26^, and when co-expressed with ORs, that these channels are activated through some direct or indirect mechanism in response to ligand binding by the OR^27,28^. VUAA1 is a non-naturally occurring molecule, which has proven to be an invaluable tool for insect olfactory research due to its ability to directly activate Orco subunits^23^. Since its discovery, VUAA1 has been shown to activate Orcos from diverse insects, including dipterans, lepidopterans, and hymenopterans. Due to its seemingly universal ability to agonise Orco proteins, and therefore ability to modulate insect olfaction, the compound is being pursued for its potential application as a novel pest control tool^23,29,30^. In addition, the compound has opened the door to more fundamental research designed to understand receptor complex ion channel properties and Orco-OR interactions^31,32^.

To date, efforts to solve the 3D structure of the individual subunits or the entire receptor complex have been fruitless due to the challenges associated with crystallising membrane proteins. The lack of homology of Orco and ORs to other proteins whose structures have been experimentally determined limits structural biologists to *de novo* modelling, which unfortunately is inherently difficult. In light of these limitations, and as a rapidly increasing number of OR sequences have become available, a modelling technique that relies on amino acid covariation within proteins has been developed^33,34^. This method is based on large multiple sequence alignments (MSA), in the case of Orcos and ORs, using sequences from a wide range of insect species, and allows the calculation of proximity restraints of amino acid pairs that appear to co-evolve, which guides the subsequent folding of the polypeptide to produce a more accurate theoretical structure. While these models have limitations, they are currently our best estimation of the true 3D structure of insect OR complexes.

Despite not knowing the structure of Orco or ORs, how they interact to form a functional receptor complex, or the exact nature of the signalling pathways they utilise, it is still possible to study the function of these proteins. Since the de-orphanisation (functional characterisation) of the first insect OR in 2001^20^ investigators routinely express Orcos and ORs in various experimental systems with aims to de-orphan and describe the OR repertoire of various insects to address ecological and evolutionary questions, or study the interactions between ligands and their ORs^35–38^. Similarly, in efforts to identify functionally important regions of the proteins, mutational analyses have identified residues that affect OR sensitivity^39^ and selectivity to ligands^40,41^, as well as Orco channel activity and ion selectivity^42–44^.

The highly specialised Hessian fly, *Mayetiola destructor* (Mdes; Diptera, Cecidomyiidae), is a serious gall-inducing pest of wheat (*Triticum* spp.), both in the US and in certain regions of the Old World. Previously, in an attempt to de-orphan sex pheromone-specific ORs in *M. destructor*, it was found that MdesOrco does not respond to the Orco-specific agonist, VUAA1^45^. In that study, candidate sex-pheromone receptors were co-expressed with MdesOrco in HEK293 cells and tested for responsiveness to pheromone compounds^46^. Despite its insensitivity to VUAA1, MdesOrco was shown to be functional as its co-expression was required for responsiveness of MdesOR115 to pheromone compounds. To our knowledge, MdesOrco is the only Orco that has been identified that does not endogenously respond to VUAA1. This insensitivity to VUAA1 is intriguing because of its potential to provide insight into the binding site for VUAA1 as well as OR-complex properties in this organism. Here we undertake a mutagenesis-based approach in combination with 3D modelling to elucidate the molecular basis for this MdesOrco insensitivity to VUAA1. We reveal amino acid residues crucial for VUAA1 responses, and show that these residues are also necessary for odour-evoked responses of OR-Orco complexes in the vinegar fly *Drosophila melanogaster* (Diptera: Drosophilidae) and the moth *Agrotis segetum* (Lepidoptera: Noctuidae), but not in the Hessian fly, suggesting novel OR-Orco interactions in this specialised dipteran.

## Results

### Sequence variation

**Figure 1:**
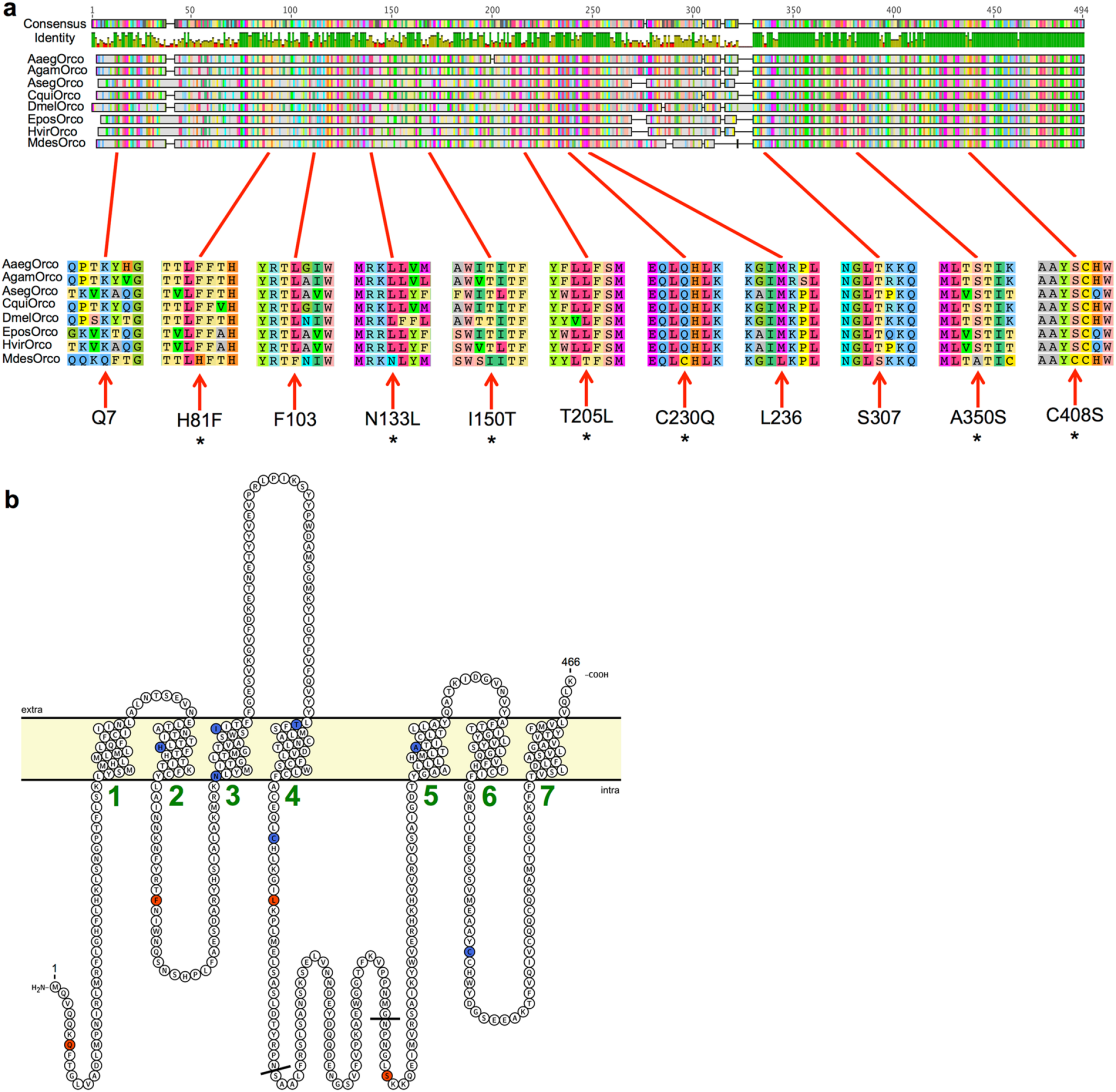
(**a**) MUSCLE alignment of *Mayetiola destructor* Orco (MdesOrco) along with VUAA1-responsive Orcos. Numbered amino acids indicate MdesOrco residues that diverge from those conserved in other Orcos. Asterisks indicate residues that were mutated in this study. Aaeg = *Aedes aegypti*, Agam = *Anopheles gambiae*, Aseg = *Agrotis segetum*, Cqui = *Culex quinquefasciatus*, Dmel = *Drosophila melanogaster*, Epos = *Epiphyas postvittana*, Hvir = *Heliothis virescens*. (**b)** Predicted MdesOrco membrane topology using TOPCONS software^60^, visualised using Protter software^68^. Green numbers represent predicted transmembrane helices. Blue circles indicate the location of the divergent MdesOrco residues that were mutated in this study and red circles those that were not mutated in this study. Residues between black lines are part of the highly divergent IL2 region of MdesOrco.

In order to discern the molecular basis for the insensitivity of MdesOrco to VUUA1, we initially compared the amino acid sequence of MdesOrco to that of several other insect Orcos that have been shown to respond to this compound^23,25,26,47^. Alignment of *Aedes aegypti*, *Anopheles gambiae*, *Agrotis segetum*, *Culex quinquefasciatus*, *Drosophila melanogaster*, *Epiphyas postvittana*, *Heliothis virescens* and *Mayetiola destructor* Orco amino acid sequences revealed an average pairwise identity of 72%. When MdesOrco was removed from the alignment the pairwise identity was 74%, which suggests that the variance of MdesOrco was similar to that of the group as a whole. Overall, MdesOrco sequence deviated the most from the group consensus within the highly variable intracellular loop II (IL2) region (MdesOrco residues 253 to 301), however, interestingly, there were 11 positions outside of this region where the MdesOrco residue was different from that which is conserved in all other species in the alignment (Fig. 1a). Of these 11 divergent residues outside of the IL2 region, six were predicted to be located in intracellular regions and five were predicted to be in transmembrane regions (Fig. 1b). These 11 variant residues are present in the current *M. destructor* genome assembly^48^, a previous transcriptome *de novo* assembly^49^, as well as in Orco sequence derived from cDNA^45^, all however produced from biological material originating from the same laboratory-reared strain of the Great Plains biotype. Hence, to confirm that these 11 MdesOrco variant residues were not a product of decreased selective pressure on laboratory-reared flies, we evaluated their presence in a sibling species, the barley stem gall midge *M. hordei*, as well as in the *M. destructor* Syrian strain through BlastN searches of the SRA database (NCBI). We found that all 11 variant residues were present in the Syrian *M. destructor* biotype Orco, and that 10 of the 11 substitutions (all but S350A) were present in Orco from the sibling species, *M. hordei*.

### Mutagenesis

**Figure 2:**
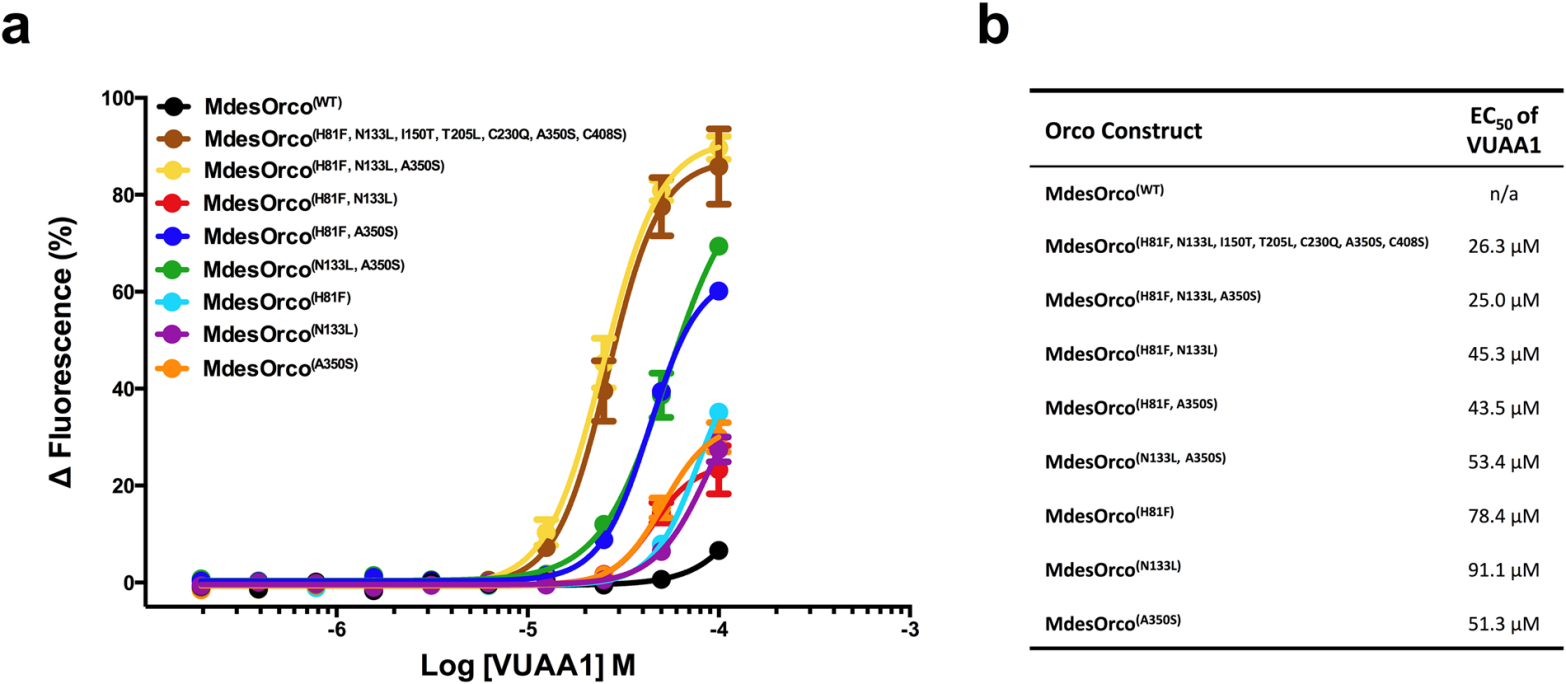
(**a)** Response of TREx/HEK293 cells expressing wild-type or various mutated forms of MdesOrco to different concentrations of the Orco agonist VUAA1. (**b)** EC_50_ values for the responses shown in (**a**). Data represent the mean response (±SEM) of cells from three biological replicates. Note: all data points contain error bars; some are too small to be seen.

We tested the hypothesis that these sequence differences were responsible for MdesOrco VUAA1 insensitivity by generating mutants in which divergent MdesOrco residues were changed to the conserved residues of other Orcos. As a starting point, we engineered an MdesOrco construct with seven point mutations at residues that were predicted to be within transmembrane regions and potentially interact with other helices in the membrane or directly with VUAA1, or in the case of C230 and C408, because of their potential to affect protein tertiary structure through formation of disulfide bridges. We constructed a second MdesOrco mutant in which the divergent IL2 loop region was replaced with that of DmelOrco, and a third mutant in which we combined the seven point mutations and the replacement of the IL2 region in a single mutant. We found that the MdesOrco mutant that contained the seven point mutations responded well to VUAA1 in functional assays using HEK293 cells (Fig. 2). The mutant that had both the point mutations and the IL2 substitution also responded well, however the mutant that contained only the IL2 substitution did not respond well to VUAA1, demonstrating that the sequence variation in this region did not affect MdesOrco sensitivity to the compound (see Supplementary Fig. S1).

We then sought to identify which of the seven mutated residues were responsible for conferring VUUA1 sensitivity to MdesOrco^(H81F, N133L, I150T, T205L, C230Q, A350S, C408S)^. Through sequential reversion of mutated residues back to wild-type (WT) sequence we found that four of the seven mutations, I150T, T205L, C230Q and C408S, had no effect on MdesOrco sensitivity to VUAA1; MdesOrco^(H81F, N133L, I150T, T205L, C230Q, A350S, C408S)^ and MdesOrco^(H81F, N133L, A350S)^ responded similarly to VUAA1 in HEK293 cells. Reversion of any of the three remaining mutations (H81F, N133L, A350S) to WT sequence, alone or in combination, decreased the response of MdesOrco to VUAA1. As previously reported^45^, WT MdesOrco was insensitive to VUAA1 (Fig. 2).

**Figure 3:**
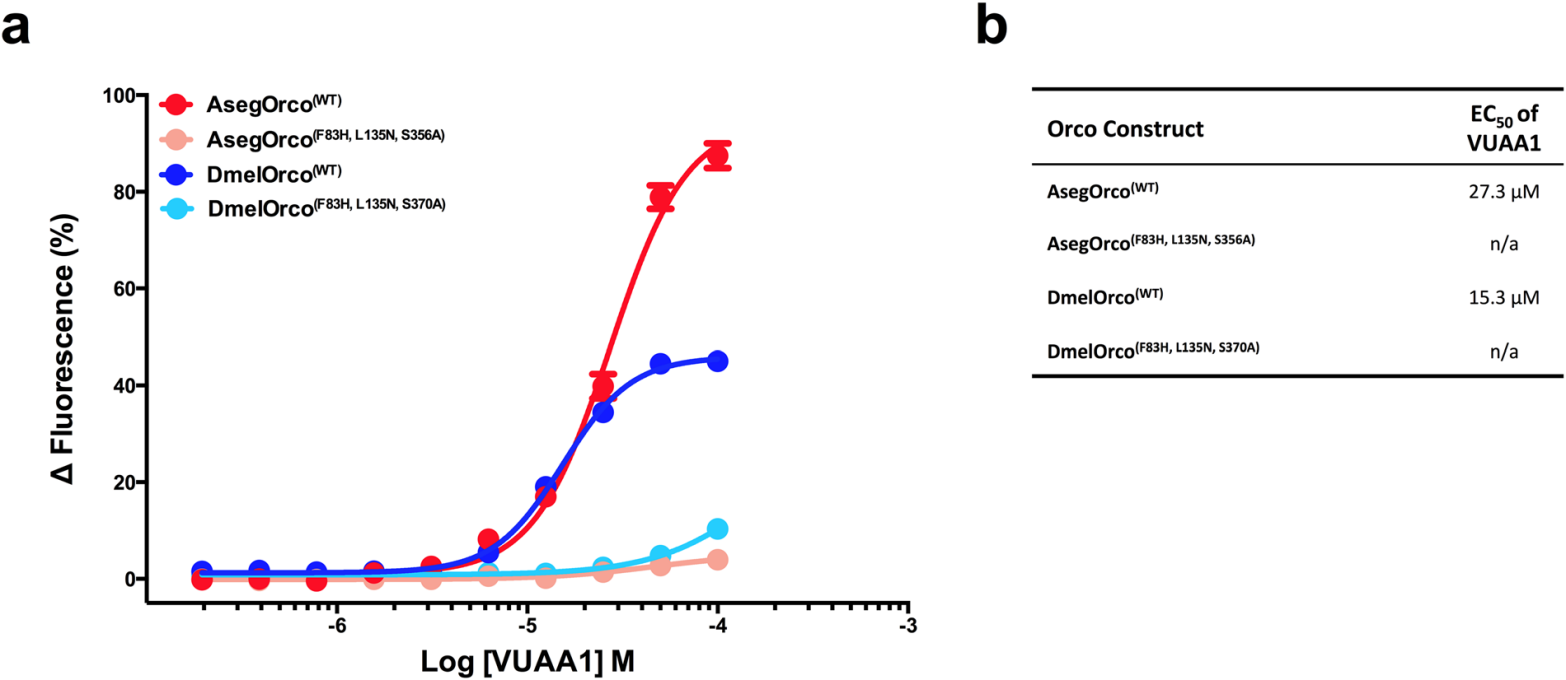
(**a**) Response of TREx/HEK293 cells expressing wild-type or mutated forms of *Agrotis segetum* (Aseg) and *Drosophila melanogaster* (Dmel) Orcos to different concentrations of the Orco agonist VUAA1. (**b**) EC_50_ values for the responses shown in (**a**). Data represent the mean response (±SEM) of cells from three biological replicates. Note: all data points contain error bars; some are too small to be seen.

To verify the effects of these three point mutations on the sensitivity of MdesOrco to VUAA1, we mutated the corresponding three residues in Orcos from two other species, the turnip moth *Agrotis segetum* and *Drosophila melanogaster*, to WT MdesOrco residues and tested them for responsiveness to VUAA1 in HEK293 cells. WT AsegOrco and DmelOrco responded dose-dependently to VUAA1, however the sensitivity of AsegOrco^(F83H, L135N, S356A)^ and DmelOrco^(F83H, L135N, S370A)^ to the compound was greatly diminished (Fig. 3). In summary, the WT forms of AsegOrco and DmelOrco and the mutant MdesOrco^(H81F, N133L, A350S)^ all began to respond to VUAA1 in the 10 µM range, whereas the mutant AsegOrco^(F83H, L135N, S356A)^ and DmelOrco^(F83H, L135N, S370A)^ and the WT form of MdesOrco began responding in the 100 µM range. These results confirmed similar effects of mutating these three residues, in terms of sensitivity to the agonist VUAA1, in three relatively distantly related insect species from two taxonomic orders.

### Phylogenetic analysis

To evaluate the amino acid variation at these three residues within the Insecta, we constructed a phylogenetic tree using the Orco sequences from 187 species (plus the Orco-like protein 2 from the firebrat *Thermobia domestica*^9^ included as an outgroup) retrieved through exhaustive Blast searches (see Supplementary Fig. S2). The presence of F81 and S350 among the most basal insect lineages in our analysis, including *T. domestica*, indicate that these amino acids represent ancestral character states of Orco proteins, dating back to an early point in the radiation of the insects. The residue L133 is not present in *T. domestica*, but appears to have originated in an ancestral Orco protein at least prior to the origin of the Hemiptera. Variation at these three positions is highly limited. Apart from *M. destructor*, the F81H mutation has become fixed independently in only one species of Hymenoptera, whereas one L133V mutation appears to have become fixed independently in an ancestor to two related species of Coleoptera. In contrast, residue 350 is more variable with both serine and threonine being prevalent among Hemiptera and Orthoptera, while the majority of hymenopterans have a threonine. However, the non-conservative F81H, L133N, and S350A changes occurring together is unique to MdesOrco, and none of these individual rare variants are found in any of the other analysed dipteran or lepidopteran species.

### Mutational mechanism of action

**Figure 4:**
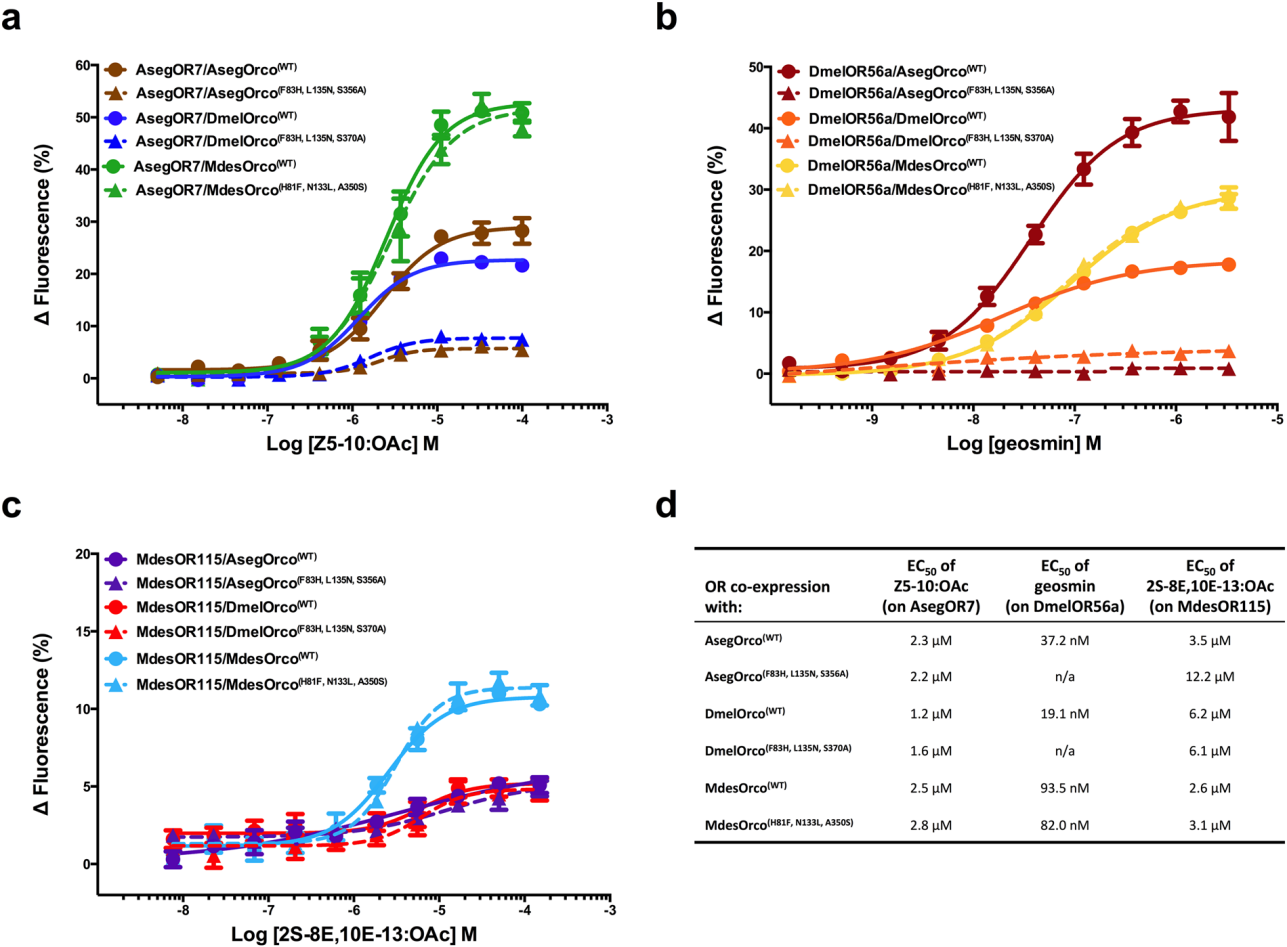
Response of TREx/HEK293 cells expressing *Agrotis segetum* (Aseg) OR7, *Drosophila melanogaster* (Dmel) OR56a or *Mayetiola destructor* (Mdes) OR115 in combination with wild-type or mutated forms of AsegOrco, DmelOrco and MdesOrco to different concentrations of the OR-specific ligands. (**a**) AsegOR7-expressing cells were tested for response to (*Z*)-5-decenyl acetate (Z5–10:OAc), (**b**) DmelOR56a-expressing cells were tested for response to geosmin, and (**c**) MdesOR115-expressing cells were tested for response to (2*S*,8*E*,10*E*)-8,10-tridecadien-2-yl acetate (2S-8E,10E-13:OAc). (**d**) EC_50_ values for the responses shown in (**a**,**b** and **c**). Data represent the mean response (±SEM) of cells from three biological replicates. Note: all data points contain error bars; some are too small to be seen.

While the functional experiments so far validated the effects of mutating three residues on Orco sensitivity to VUAA1, they did not provide insight into the mechanism through which this occurs. It is possible that these mutations affected VUAA1 sensitivity either directly, by altering the binding site of the compound, or indirectly, by inducing structural changes that prohibit either the interaction of VUAA1 with Orco or VUAA1-induced channel activation. To test these hypotheses, we expressed both the WT and mutated forms of AsegOrco, DmelOrco and MdesOrco with a species-specific ligand-binding OR from each of the three species in HEK293 cells and tested their ability to respond to both VUAA1 and the OR-specific ligand. We reasoned that if the response of a given OR to its ligand is altered when co-expressed with WT or mutated forms of Orco, then the mutations are likely inducing structural changes that either prohibit Orco from forming an ion channel or interfere with the interactions between the ligand-binding OR and Orco. Alternatively, if the response of the OR to its ligand is similar when co-expressed with WT or mutated forms of Orco, then the mutations are either affecting the ability of VUAA1 to bind to and/or activate Orco, or are causing a structural change in Orco that affects VUAA1 responsiveness yet neither affects ion channel formation nor interactions between the Orco and OR subunits. We found that when AsegOR7 was co-expressed with WT AsegOrco the cells responded dose dependently to its pheromonal ligand (*Z*)-5-decenyl acetate^36^ (Z5-10:OAc), but when AsegOR7 was co-expressed with AsegOrco^(F83H, L135N, S356A)^ the amplitude of the response was significantly diminished (Fig. 4). Similarly, when DmelOR56a was co-expressed with WT DmelOrco the cells responded dose-dependently to geosmin^50^, but when DmelOR56a was co-expressed with DmelOrco^(F83H, L135N, S370A)^, the response of the cells was abolished. In contrast, MdesOR115 responded equally well to (2*S*,8*E*,10*E*)-8,10-tridecadien-2-yl acetate^45^ (2S-8E,10E-13:OAc) when co-expressed with either WT MdesOrco or MdesOrco^(H81F, N133L, A350S)^. As a control, we tested HEK293 cells that only expressed the OR subunits (no Orco present) and did not see any response to ligands. Similarly, cells expressing Orco and OR proteins did not respond to vehicle controls, and cells not expressing these proteins did not respond to VUAA1 or OR-specific ligands. In all cases, Orco and OR proteins were detected in cell line lysates by western blot, indicating that each version of the protein was expressed in HEK293 cells and present for functional testing (see Supplementary Fig. S3).

Returning to our hypotheses, these results suggested that the three mutations induced a structural change in AsegOrco and DmelOrco that either interfered with ion channel formation or the ability of the Orcos to interact with ORs. In contrast, in MdesOrco the mutations were either affecting the ability of VUAA1 to bind to and/or activate the ion channel or were causing structural changes in MdesOrco that did not affect ion channel formation or interactions between MdesOrco and MdesOR115. While it is theoretically possible, it is unlikely that the binding site for VUAA1 is different on different Orco proteins^25^. Instead, our interpretation of these results is that these mutations are inducing structural changes in all three Orcos, and yet, in contrast to AsegOrco and DmelOrco, these structural changes neither prohibit MdesOrco from forming an ion channel alone nor interfere with its ability to interact with MdesOR115. In other words, MdesOrco is unique, in that the effects of the identities of the residues at these positions are different than they are in AsegOrco and DmelOrco.

To test whether this ability of WT MdesOrco and MdesOrco^(H81F, N133L, A350S)^ to function with MdesOR115 was limited to conspecific Orco-OR co-expression, we co-expressed each version of MdesOrco with AsegOR7 or DmelOR56a and tested them for responsiveness to OR-specific ligands. Similar to what we found when MdesOR115 was co-expressed with WT MdesOrco or MdesOrco^(H81F, N133L, A350S)^, both AsegOR7 and DmelOR56a showed comparable responses to their specific ligands when co-expressed with either version of MdesOrco (Fig. 4). For comparison, we also co-expressed MdesOR115 or AsegOR7 with both versions of DmelOrco, as well as MdesOR115 or DmelOR56a with both versions of AsegOrco and tested them for responsiveness to OR-specific ligands. When AsegOR7 and DmelOR56a were co-expressed with WT forms of DmelOrco or AsegOrco, respectively, they responded well to their respective OR-specific ligand, and when they were co-expressed with DmelOrco^(F83H, L135N, S370A)^ or AsegOrco^(F83H, L135N, S356A)^, respectively, these responses were greatly diminished. Interestingly, when MdesOR115 was co-expressed with both forms (WT and mutant) of AsegOrco or DmelOrco, the response to 2S-8E,10E-13:OAc was comparable between WT and mutant forms of each Orco, yet clearly reduced compared to the response observed when MdesOR115 is co-expressed with either form of MdesOrco.

### Summarisation of functional experiments

To summarise the results of our functional experiments, WT MdesOrco did not respond to VUAA1 but MdesOrco^(H81F, N133L, A350S)^ did, and when either of these proteins were co-expressed with MdesOR115, AsegOR7 or DmelOR56a, the cells responded dose-dependently to the OR-specific ligand. In contrast, WT AsegOrco and WT DmelOrco both responded to VUAA1 but AsegOrco^(F83H, L135N, S356A)^ and DmelOrco^(F83H, L135N, S370A)^ did not. When the WT forms of AsegOrco and DmelOrco were co-expressed with AsegOR7 or DmelOR56a, the cells responded dose-dependently to the OR-specific ligand, however, when AsegOrco^(F83H, L135N, S356A)^ or DmelOrco^(F83H, L135N, S370A)^ were co-expressed with either AsegOR7 or DmelOR56a, the response to the OR-specific ligand was greatly diminished or non-existent. Interestingly, the response of MdesOR115 to its ligand was comparable when expressed with either form of AsegOrco or DmelOrco, yet reduced compared to the response obtained when expressed with MdesOrco. Finally, co-expression of WT MdesOrco, AsegOrco^(F83H, L135N, S356A)^ or DmelOrco^(F83H, L135N, S370A)^ with any ligand-binding ORs did not rescue their insensitivity to VUAA1.

Our interpretation of these results is that mutating the three residues causes a structural change in Orco proteins that ultimately confers VUAA1 sensitivity to MdesOrco or renders AsegOrco and DmelOrco insensitive to the compound. In addition to the effects on VUAA1 sensitivity, these structural changes also disrupt the ability of AsegOrco and DmelOrco to form functional complexes with AsegOR7 and DmelOR56a. However, with MdesOrco, despite the effects of these mutations on VUAA1 sensitivity, they have no effect on the ability of the co-receptor to form functional complexes with MdesOR115, AsegOR7 or DmelOR56a.

### 3D modelling

**Figure 5:**
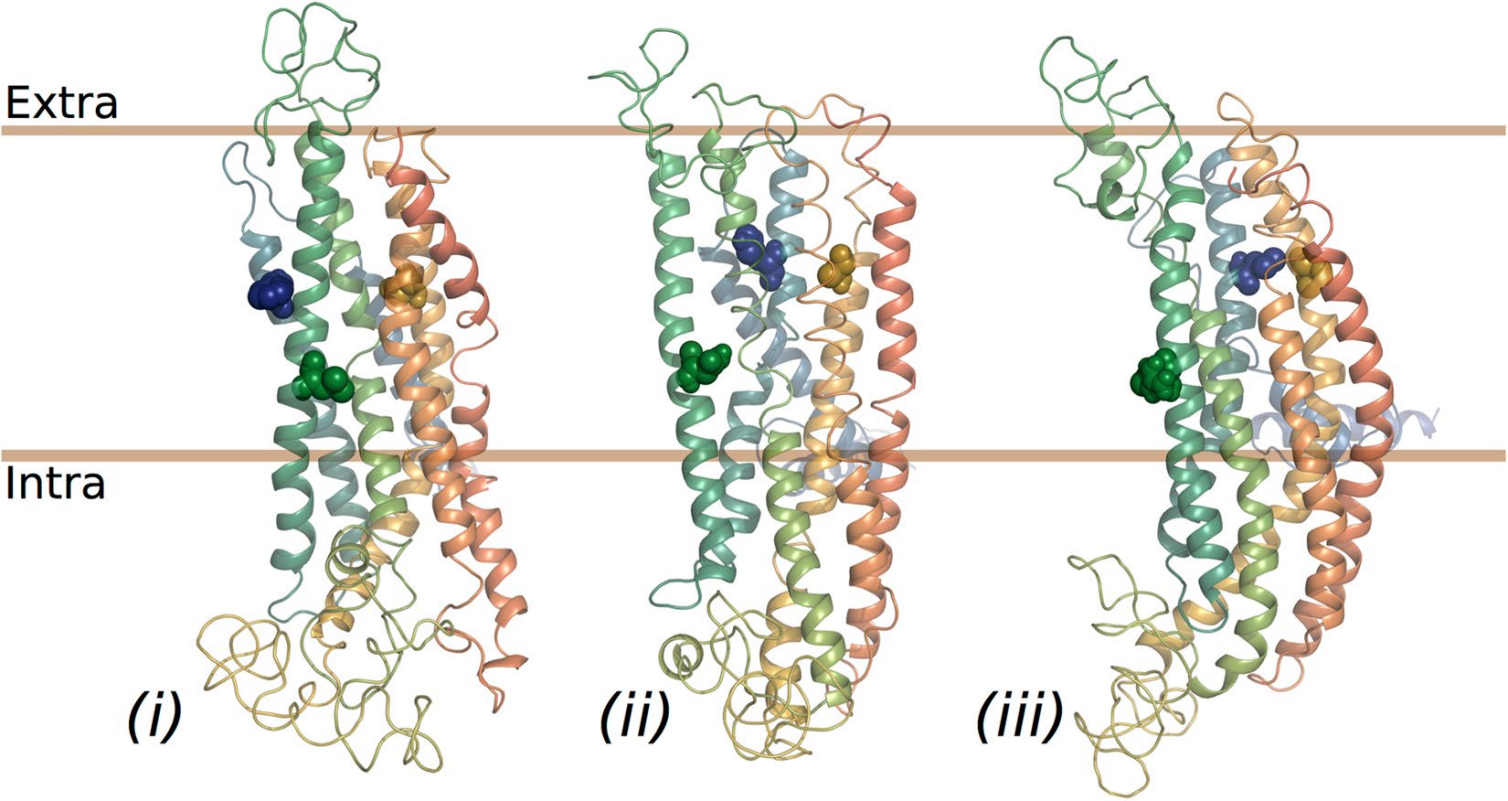
3D models of *Drosophila melanogaster* (Dmel) Orco (**i**), *Mayetiola destructor* (Mdes) Orco (**ii**) and *Agrotis segetum* (Aseg) Orco (**iii**) based on evolutionary coupling. Residues shown in sphere representation represent residues mutated in this study: blue (DmelOrco F83H, MdesOrco H81F, AsegOrco F83H), green (DmelOrco L135N, MdesOrco N133L, AsegOrco L135N), and gold (DmelOrco A370S, MdesOrco S350A, AsegOrco A356S). Note: protein models are coloured from blue (N-termini) to red (C-termini).

Next, in an effort to understand the possible mechanism by which the mutations influence VUAA1 sensitivity, we produced 3D models of the AsegOrco, DmelOrco and MdesOrco proteins using evolutionary amino acid covariation. There are currently no experimentally determined structures of insect Orcos or ORs available, ruling out the possibility of using protein homology modelling. We first produced a model of WT DmelOrco and compared it to a published model of WT DmelOrco that was produced using this method^34^. We found that the DmelOrco model produced by our modified MSA, which included a large number of additional Orco sequences, contained more tightly packed helices than the previously reported DmelOrco model. Also, because ORs and Orcos are believed to have similar tertiary structures – which is the basis for including both in the MSA – we note that our DmelOrco model was more comparable to the reported DmelOR85b model^34^ (see Supplementary Fig. S4). We then produced models of MdesOrco and AsegOrco and found that these models had similar folding and were comparable to both our DmelOrco model and the previously reported DmelOR85b model (Fig. 5, Supplementary Fig. S4). All of our models contain seven transmembrane helices that extend into an intracellular domain containing both helical contributions and loop regions. Helices 5, 6 and 7 consistently formed a helical bundle and the same holds true for helices 2, 3, and 4. Helix 1, which is situated next to helix 2, has less evolutionary couplings to other parts of the protein, giving rise to slight variation between the modelled Orcos. In our analyses we found that DmelOrco residues F83 (helix 2), L135 (helix3) and S370 (helix 5), as well as the homologous residues in MdesOrco and AsegOrco, are all located in similar positions in transmembrane regions of the Orco models for all three organisms.

**Figure 6:**
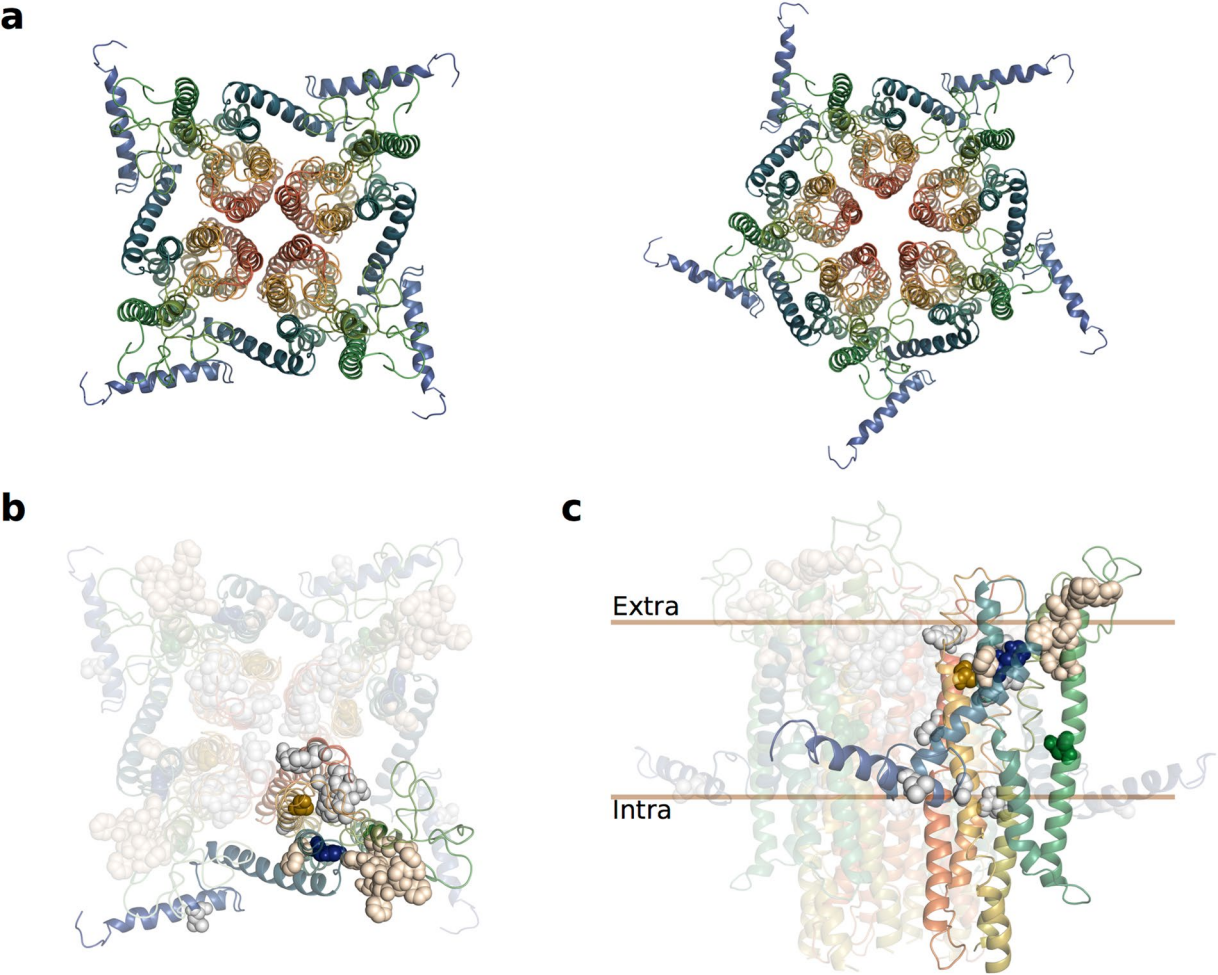
(**a**) 3D models of putative *Mayetiola destructor* (Mdes) Orco tetra- and pentamers, viewed from the extracellular surface. (**b**) Putative tetramer model of MdesOrco multimer, viewed from extracellular surface, with selected residues shown in sphere representation. Blue (H81F), green (N133L) and gold (S350A) spheres represent MdesOrco residues mutated in this study. Grey spheres represent corresponding residues associated with cation selectivity or ion channel function; tan spheres represent residues associated with ligand binding^34^. (**c**) Side view of MdesOrco tetramer with selected residues shown in sphere representation as described in (**b**). Note: protein models are coloured from blue (N-termini) to red (C-termini).

It has been shown previously^23–26^, as well as in the current study, that Orco proteins are capable of forming ion channels by themselves, that is, in the absence of ligand-binding ORs. In an effort to evaluate how these mutations might affect the ion channel we generated models of various MdesOrco homomultimers to see how and where an ion channel might form in the quaternary structure of an Orco homomultimer. From mutational studies, there are indications that helix 7 is likely to be involved in forming the pore^34^. The IL2 region was truncated as this region is likely to be flexible and interfered with monomer packing in the rigid docking. Selecting the top solutions where the relatively charged^14^ helices 5, 6 and 7 were facing the internal (i.e., pore) side of the homomultimer, we found that with three or less Orco subunits forming a functional ion pore did not seem to be possible – otherwise these charged regions of the protein would be exposed to the cell membrane environment. While there is some evidence that certain channelrhodopsins can form functional ion channels with only one, or possibly two, subunits^51^, insect Orcos share no homology to these proteins, have an inverted membrane topology, and have different structural features (e.g., the locations of charged residues), hence a similar stoichiometry for Orcos does not immediately follow. With six or more subunits the resulting pore was likely to be too wide to function as a gated channel. Therefore, the most probable stoichiometry is likely to be 4 or 5 (Fig. 6a).

Within varying degrees of proximity to the three MdesOrco mutations in each subunit are the homologous residues of amino acids that have been found to affect ligand binding, cation selectivity or ion channel activity (reviewed in ref.^34^) (Fig. 6b,c). Most of the mutations that have been shown to affect ligand binding are clustered in the extracellular loop formed between helices 3 and 4, with the exception of residue F84 which lies in the transmembrane region of helix 2. In our models, MdesOrco residues H81 and A350 are adjacent in the membrane, both next to residue F84, and residue N133 (helix 3) is directly adjacent to helix 4. Based on the position of the three mutations relative to those associated with ligand binding, and because it is physically impossible for VUAA1 (maximal length 15 Å) to directly interact with all three residues, it is likely that changing these residues affects ligand binding through, at least in part, induced structural changes, in this case allowing (in the case of MdesOrco^(H81F, N133L, A350S)^) or preventing (in the case of AsegOrco^(F83H, L135N, S356A)^ and DmelOrco^(F83H, L135N, S370A)^) VUAA1 from interacting with its binding site or activating the channel. Similarly, in our MdesOrco models residues H81 and A350 are adjacent to each other and to residues that have been found to affect Orco cation selectivity and/or ion channel activity. A mutation in either of these residues could potentially distort the geometry and function of the ion-conducting pore, allowing (in the case of MdesOrco^(H81F, N133L, A350S)^) or preventing (in the case of AsegOrco^(F83H, L135N, S356A)^ and DmelOrco^(F83H, L135N, S370A)^) VUAA1-induced channel activation. While these data strongly suggest that these mutations are causing structural changes in all three Orcos, it was not possible to determine to what extent each of the individual mutations is contributing to VUAA1 binding or ion channel function. What is interesting here is that, in contrast to MdesOrco, these mutations are also affecting the ability of AsegOrco and DmelOrco to form functional receptor complexes, as measured through OR-dependent  ligand-induced receptor activation. Regardless of the exact mechanisms, this discrepancy between the ability of the Orcos to form functional receptor complexes as a result of these mutation-induced structural changes suggests the existence of fundamental differences in the ability of the Orco proteins to form functional ion channels, both alone and as Orco-OR complexes. Unfortunately, without the ability to describe the exact nature of these structural changes in the Orco proteins, we cannot objectively rationalise the observed differences between the function of WT and mutant forms of MdesOrco compared to AsegOrco and DmelOrco. Nevertheless, these findings are important as they suggest divergence in the structure of orthologous proteins that are thought to be extremely evolutionarily conserved.

## Discussion

Previously it was found that Orco from the Hessian fly, *Mayetiola destructor*, was not activated by the agonist VUAA1^45^. Here we show that three MdesOrco residues (H81, N133 and A350) that differ from the corresponding conserved residues (F81, L133 and S350) of other VUAA1-responsive Orcos are responsible for this lack of sensitivity to the compound. We have found that all three of these deviating residues are present in a different *M. destructor* strain and that two (H81 and N133) are present in the congener *M. hordei*; one hymenopteran, *Megachile rotunda* has substitutions F81H and S350T; two coleopterans, *Rhyzopertha dominica* and *Nicrophorous vespilloides*, have the deviation L133V; and several species have non-canonical residues at S350 (including S350A, S350T, and subsequent substitutions T350A and T350S). Based on our phylogenetic analysis of currently available insect Orco sequences, we believe that residues F81, L133 and S350 represent ancient character states (see Supplementary Fig. S2). Because the mutations observed in *M. destructor* are not present in Orcos from more ancestral (i.e., Culicidae) or more derived (i.e., Drosophilidae) Dipterans^52^, it is likely that they independently became fixed within the Cecidomyiidae family, perhaps even within the *Mayetiola* genus.

Here our interest in understanding the underlying molecular mechanisms causing the MdesOrco insensitivity to VUAA1 led us to discover novel properties of the olfactory receptor complex in *M. destructor*. Our findings suggest that in the lepidopteran, *A. segetum*, and the dipteran, *D. melanogaster*, mutation of three Orco residues to the WT sequence of MdesOrco causes structural changes that significantly diminish the ability of AsegOrco and DmelOrco to form functional receptor complexes with ligand-binding ORs. In contrast, WT MdesOrco is capable of forming functional complexes, not only in our heterologous expression system but also in nature, as the insect utilises its olfactory system for foraging and reproductive purposes^46,53^. We hypothesise that over time, as these three mutations became fixed in Hessian fly populations, other compensatory mutations were simultaneously positively selected allowing MdesOrco to retain its function as a co-receptor due to inherent selection pressure placed on the insect’s olfactory system as a prerequisite for survival and reproduction. Interestingly, in contrast to co-expression of AsegOR7 and DmelOR56a with DmelOrco and AsegOrco, respectively, MdesOR115 did not respond well to its ligand when coupled to AsegOrco or DmelOrco, indicating that this receptor does not efficiently form a functional Orco-OR complex with these Orcos, yet both forms of MdesOrco were capable of forming functional channels with AsegOR7 and DmelOR56a. Indeed, the functionality of inter-specific Orco and OR co-expression has been shown repeatedly^54–57^ and is generally assumed to be universal, due to the highly conserved nature of insect Orcos. Our findings suggest that MdesOR115 and MdesOrco have evolved a unique interaction that may be a result of the structural changes that arose due to mutations in the olfactory receptor co-receptor proteins within this insect family. Interestingly, in our experiments the response of DmelOR56a to geosmin was higher (in maximum magnitude) when it was co-expressed with AsegOrco and MdesOrco compared to DmelOrco, and the response of AsegOR7 to Z5-10:OAc was higher when co-expressed with MdesOrco, and lower when co-expressed with DmelOrco, compared to AsegOrco (Fig. 4). In these studies, the magnitude of the response reflects calcium flux into the cells in response to ligand-induced receptor activation, meaning for example, that DmelOR56a and AsegOrco formed a more effective ion channel compared to DmelOR56a and DmelOrco. While these heterospecific Orco-OR combinations do not occur in nature, they do indicate that DmelOR56a is capable of forming more efficient ion channels than it does with DmelOrco, but it simply cannot because of other selective pressures on their ability to function as a geosmin-detecting receptor complex. These observations further support our conclusion regarding the co-evolution of Orco-OR interactions within species.

Of the 11 divergent MdesOrco residues, mutating three of these had an effect on VUAA1 sensitivity, and in contrast to MdesOrco, mutating these residues in AsegOrco and DmelOrco affected their ability to form functional complexes with ligand-binding ORs. Outside of these 11 sequence divergences between MdesOrco versus DmelOrco and AsegOrco, there are no other obvious critical differences between the proteins. Other conserved regions, such as the potassium channel-like selectivity filter sequence, a calmodulin binding sequence and the proposed “hinge” gating-mechanism^14^ are identical in the Orcos from the three species.

While VUAA1 is not a naturally occurring insect Orco agonist, the lack of sensitivity of Orco proteins to it may be of immediate interest to investigators seeking to identify novel pest control technologies based on this compound^23,58^. To our knowledge, *M. destructor* is the first reported species in which this compound does not activate its Orco. Here we have found that three point mutations, alone or in combination, significantly affect the sensitivity of the molecular target to the compound. This finding is important for two reasons: first, that the compound (and most likely its derivatives^25^) will be ineffective on insects that have divergent residues at these positions, and second, that this insensitivity of MdesOrco could serve as a valuable tool in the development of pest control compounds because it may provide insight into the biochemical interactions between the compound and the target proteins. For example, comparing an experimentally determined 3D structure of a VUAA1-responsive Orco (e.g., DmelOrco) to that of a non-responsive Orco (e.g., MdesOrco) would likely reveal structural differences that may provide insight into the binding site for the receptor agonist and aid in further structure-activity relationship studies.

Previously, several studies have mutated various insect ORs and Orco proteins and found diverse effects on olfactory reception (reviewed thoroughly in ref.^34^). In most cases the effects of these mutations are relatively easy to describe, however, in all cases the underlying mechanisms causing those effects have not been characterised. For example, even in one of the most conceptually simple cases, in which a single point mutation was shown to alter specificity of a pheromone-sensitive OR in *Ostrinia* moths^40^, it is difficult to explain how the observed phenotype arises. In this case, the point mutation could be altering the actual pheromone binding site or simply causing a structural change that indirectly affects pheromone-OR interactions. Here we attempted to characterise such mechanisms by *de novo* modelling of the mutated proteins, but given the uncertainty of the models, the precise location of a VUAA1 binding site and an activation mechanism remain speculative. Similarly, the nature of the interactions between the insect olfactory complex subunits is still unknown. Attempting to describe these interactions poses an increased level of complexity for existing *de novo* modelling techniques. For this reason we purposely limited our modelling efforts to monomers or, in the case of Orco, to homomultimers because of its experimentally proven ability to form homomeric ion channels. However, given the similarity of the modelled structure of the individual Orco and OR subunits (see Supplementary Fig. S4), it is tempting to speculate that OR subunits could easily be interchanged with Orco subunits in our multimeric models (see Fig. 6).

Until the three-dimensional structure of an OR, Orco or perhaps the entire Orco-OR complex is experimentally determined through x-ray diffraction or single particle cryo-electron microscopy, characterising the exact nature of the effects of mutations on insect olfactory receptor complexes, as well as the exact nature of the interactions between the subunits will remain speculative. Nevertheless, the functional data we present here are well founded and allowed us to discover novel olfactory receptor complex properties in *M. destructor*. Despite the fact that the exact underlying mechanisms for this finding could not be determined, our findings can inform future studies of OR-Orco interactions and signalling, and serve as a communication to investigators that not all insect olfactory receptor complexes are “created” equal.

## Methods

### Identification of target residues for mutational studies

Amino acid sequences of VUAA1-responsive insect Orcos were obtained from the NCBI database and used in an alignment with MdesOrco using the MUSCLE alignment tool in Geneious R7 software^59^. Sequences used (accession number): *Aedes aegypti* (XP001651426.1), *Anopheles gambiae* (AAX14774.1), *Agrotis segetum* (KC526964.1), *Culex quinquefasciatus* (DQ231246.1), *Drosophila melanogaster* (NP524235.2), *Epiphyas postvittana* (ACJ12928.2), *Heliothis virescens* (AJ487477.1) and *Mayetiola destructor* (KX661034). The 2D membrane topology of MdesOrco was predicted using TOPCONS software^60^.

### Construct generation and mutagenesis

Gene cloning and construct generation for expression of WT Orcos and ORs in HEK293 cells followed the methods previously described^26^ using previously reported purified plasmids containing WT AsegOrco or AsegOR7^36^, WT DmelOrco or DmelOR56a^38^ and WT MdesOrco or MdesOR115^45^ as templates for cloning reactions. All Orco constructs were modified to contain N-terminal c-myc epitope tags in the pcDNA4/TO (Thermo Fisher Scientific) expression vector. All OR constructs were modified to contain N-terminal V5 epitope tags in the pcDNA5/TO (Thermo Fisher Scientific) expression vector. For generation of mutant Orco constructs, initially three MdesOrco constructs were synthesised (Thermo Fisher Scientific) to contain: (1) seven amino acid substitutions (H81F, N133L, I150T, T205L, C230Q, A350S and C408S) that changed the WT MdesOrco residue to the corresponding residue conserved in other VUAA1-responsive Orcos, (2) a modified intracellular loop II (IL2) region containing WT DmelOrco residues 271–321 in place of WT MdesOrco residues 269–301, and (3) the combination of these seven point mutations and the deletion/insertion event described under (1) and (2). Note that while the variable IL2 region of the Orcos examined in this study spanned MdesOrco residues 253–301, MdesOrco and DmelOrco are identical between residues 253–269 and because of this our substitution began after MdesOrco residue 269. Further mutational work was conducted using MdesOrco^(H81F, N133L, I150T, T205L, C230Q, A350S, C408S)^ in pcDNA4/TO as a template and the Q5 Site-Directed Mutagenesis kit (New England BioLabs) following the manufacturer’s instructions. Residues were mutated back to the WT MdesOrco sequence and tested for their effect on response to VUAA1 in HEK293 cells (described below). WT AsegOrco and WT DmelOrco in pcDNA4/TO were used as templates to sequentially mutate residues to generate AsegOrco^(F83H, L135N, S356A)^ and DmelOrco^(F83H, L135N, S370A)^ constructs using the Q5 Site-Directed Mutagenesis kit following the manufacturer’s instructions, however only constructs containing all three mutations were tested in HEK293 cells.

### Functional characterisation in HEK293 cells

The methods used to generate and functionally test HEK293 cell lines with stable and inducible expression of *A. segetum, D. melanogaster* and *M. destructor* Orco and OR constructs are identical to those previously described^26^. Expression of Orco and OR protein in HEK293 cell lines was verified by western blot using the methods and reagents described previously^45^. Briefly, HEK293 cells stably expressing the Tetracycline Repressor (TREx, Thermo Fisher Scientific) were transfected with MdesOrco constructs and cell lines with stable, inducible expression of MdesOrcos were tested for responsiveness to the Orco agonist VUAA1. TREx/HEK293 cells were then transfected with AsegOR7, DmelOR56a or MdesOR115, and cell lines with stable, inducible expression of each OR were used for further transfections with AsegOrco^WT^, AsegOrco^(F83H, L135N, S356A)^, DmelOrco^WT^, DmelOrco^(F83H, L135N, S370A)^, MdesOrco^WT^ and MdesOrco^(H81F, N133L, A350S)^ to generate cell lines with stable, inducible expression of both the OR and Orco constructs. Cell lines expressing AsegOR7, DmelOR56a or MdesOR115 alone or in combination with each Orco were then tested for responsiveness to VUAA1 (Vitas-M Ltd.) and a previously reported OR-specific ligand ((*Z*)-5-decenyl acetate^36^, geosmin^50^, (2*S*,8*E*,10*E*)-8,10-tridecadien-2-yl acetate^45^, respectively) as described previously. Briefly, cell lines were induced (or not induced as control) to express Orcos and ORs, loaded with the calcium-sensitive fluorophore Fluo-4AM and tested for responsiveness to various concentrations of compounds using a BMG Omega plate reader. Responses of cell lines to compound was measured as a per cent increase in intracellular fluorescence from baseline. Three wells of induced or non-induced cells were tested for response to compound, or dose of compound, in each experiment. Naïve cells were re-plated, induced (or non-induced) and tested for response to compound on three separate occasions to form biological replicates. Dose response data was analysed and EC_50_s were calculated using the non-linear regression curve fit function of GraphPad Prism software, version 6.00 (www.graphpad.com).

### Validation of *Mayetiola Orco* sequences

To verify that the divergent residues in MdesOrco from laboratory-reared individuals of the ‘Great Plains’ biotype was not due to reduced/artificial selection pressures on this particular strain, we analysed raw genomic data from a second *M. destructor* strain as well as its sibling species, *M. hordei*. Wild-type MdesOrco DNA sequence was used as a query in BlastN searches against the SRA database targeted towards Illumina genomic reads of the *M. destructor* Syrian strain (SRX049257) and *M. hordei* (SRX049256). Reads with the highest identity to WT MdesOrco were fetched for mapping. Sequences of 100 bp in length were blasted in each query, but modified in the case of exon/intron boundaries. Intron sequences were removed from reads, allowing them to map to the MdesOrco coding sequence. Reads were mapped to the WT MdesOrco coding sequence using the *de novo* assembly feature of Geneious software.

### Multiple sequence alignment

A multiple sequence alignment (MSA) containing OR and Orco sequences was obtained from Hopf *et al*.^34^ and modified prior to its use for *de novo* modelling. The Orco genes were updated through blast searching of NCBI databases to contain a total of 187 non-redundant sequences. Four Orco sequences derived from automated gene prediction pipelines could be manually corrected (adjusted start for three sequences, removal of an internal insertion in one sequence; indicated in Supplementary File S5), whereas obviously erroneous Orco sequences that could not be manually corrected with confidence were excluded. Our final list of 187 Orco sequences also contain some partial proteins, which all aligned properly and provide information on evolutionary couplings. Our updated MSAs excluded 456 ORs that were included by Hopf *et al*.^34^ because they either interfered with the alignment or could not be properly aligned. The majority of these ORs belong to the hymenopteran-specific radiation of 9-exon ORs, and the remainder represent incorrectly predicted ORs from automated pipelines or partial divergent ORs. Sequences in the MSAs were sorted by pairwise identity using Jalview^61^. All Orco and OR sequences that were used in our MSAs can be found as Supplementary File S5. The MSAs used to generate AsegOrco, DmelOrco and MdesOrco models can be found online as Supplementary Files S6,S7 and S8, respectively.

### Inference of EC from sequence variation and *de novo* folding

Evolutionary constraints were calculated using the EVfold server (evfold.org)^33,34^ using the pseudolikelihood maximisation statistical inference method (Evfold-PLM). The calculations were performed using mainly standard settings. High Conservation Filter Threshold was set to 95. Constraint Rank Weight Function was set to n/i. Transmembrane topology was calculated using PolyPhobius^62^ and validated against TMHMM2^63,64^. PSIPRED^65^ was used to calculate predicted secondary structure. Helices with a reliability index below 3 were set to strand. Predictions were evaluated against the produced models and adjusted where necessary. Coupling of residues leading to distorted tertiary structures was treated as outliers and likewise not included in modelling. Clustering and scoring of the produced models were done by the internal quality assessment protocol of EVfold software. Top ranking models with structural knots, buried or exposed transmembrane helices or other chemically inconsistent traits were rejected in the evaluation process. Pymol^66^ and VMD^67^ were used for visualisation and evaluation of the models.

### Orco phylogeny

To depict where in the insect phylogeny variation among the three residues affecting VUAA1 responses (residues 81, 133, and 350 in the MdesOrco) occur, how frequently alterations to these residues appear, and to predict putative ancestral states, a phylogeny including the 187 insect Orcos covering many taxonomic orders was constructed. Orco sequences were aligned using MAFFT v. 7.017, the subsequent tree was produced using FastTree v. 2.1.5 (both implemented in Geneious 7 software), and then colour coded using FigTree v. 1.4.3.

### Data Availability

The datasets generated during and/or analysed during the current study are available from the corresponding author upon reasonable request. All sequence data used in phylogenetic and multiple sequence alignment analyses can be found online as Supplementary Files. The sources and sequences of wild-type *A. segetum, D. melanogaster and M. destructor* Orco DNA used in functional analyses is described in the methods section.

## Electronic supplementary material

Supplementary Data

Supplementary File S5

Supplementary File S6

Supplementary File S7

Supplementary File S8
