# Supplementary material for: Endogenous insensitivity to the Orco agonist VUAA1 reveals novel olfactory receptor complex properties in the specialist fly Mayetiola destructor
Source: Sci Rep. 2018 Feb 22;8:3489. doi: 10.1038/s41598-018-21631-3 (PMC5823858; doi:10.1038/s41598-018-21631-3)
Supplement: Supplementary file 1 — Supplementary Data [file 41598_2018_21631_MOESM1_ESM.pdf]

Supplementary Data

Endogenous insensitivity to the Orco agonist VUAA1 reveals novel olfactory receptor complex properties in the specialist fly *Mayetiola destructor*

Jacob A. Corcoran, Yonathan Sonntag, Martin N. Andersson, Urban Johanson and Christer Löfstedt

Material included

Figure S1
Figure S2
Figure S3
Figure S4

List of additional supplementary data files

a

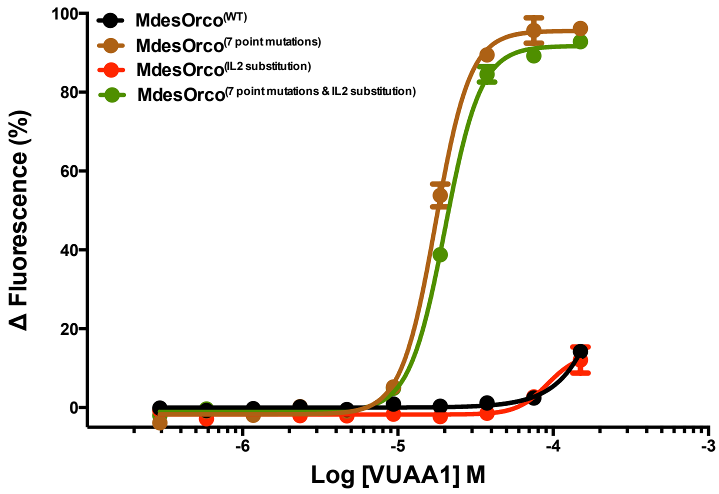

b

Orco Construct	EC_{50} of VUAA1
MdesOrco(WT)	n/a
MdesOrco(7 point mutations)	17.5 μM
MdesOrco(IL2 substitution)	n/a
MdesOrco(7 point mutations & IL2 substitution)	20.1 μM

Figure S1. a) Response of TREx/HEK293 cells expressing wild-type (WT) or mutated forms of MdesOrco to different concentrations of the Orco agonist VUAA1. **b)** EC_{50} values for the responses shown in (a). MdesOrco constructs with seven point mutations have the following differences from wild-type sequence: H81F, N133L, I150T, T205L, C230Q, A350S and C408S. Constructs with the intracellular loop two (IL2) substitution contain WT DmelOrco residues 271-321 in place of WT MdesOrco residues 269-301. Data represent the mean response (± SEM) of cells from three biological replicates. Note: all data points contain error bars; some are too small to be seen.

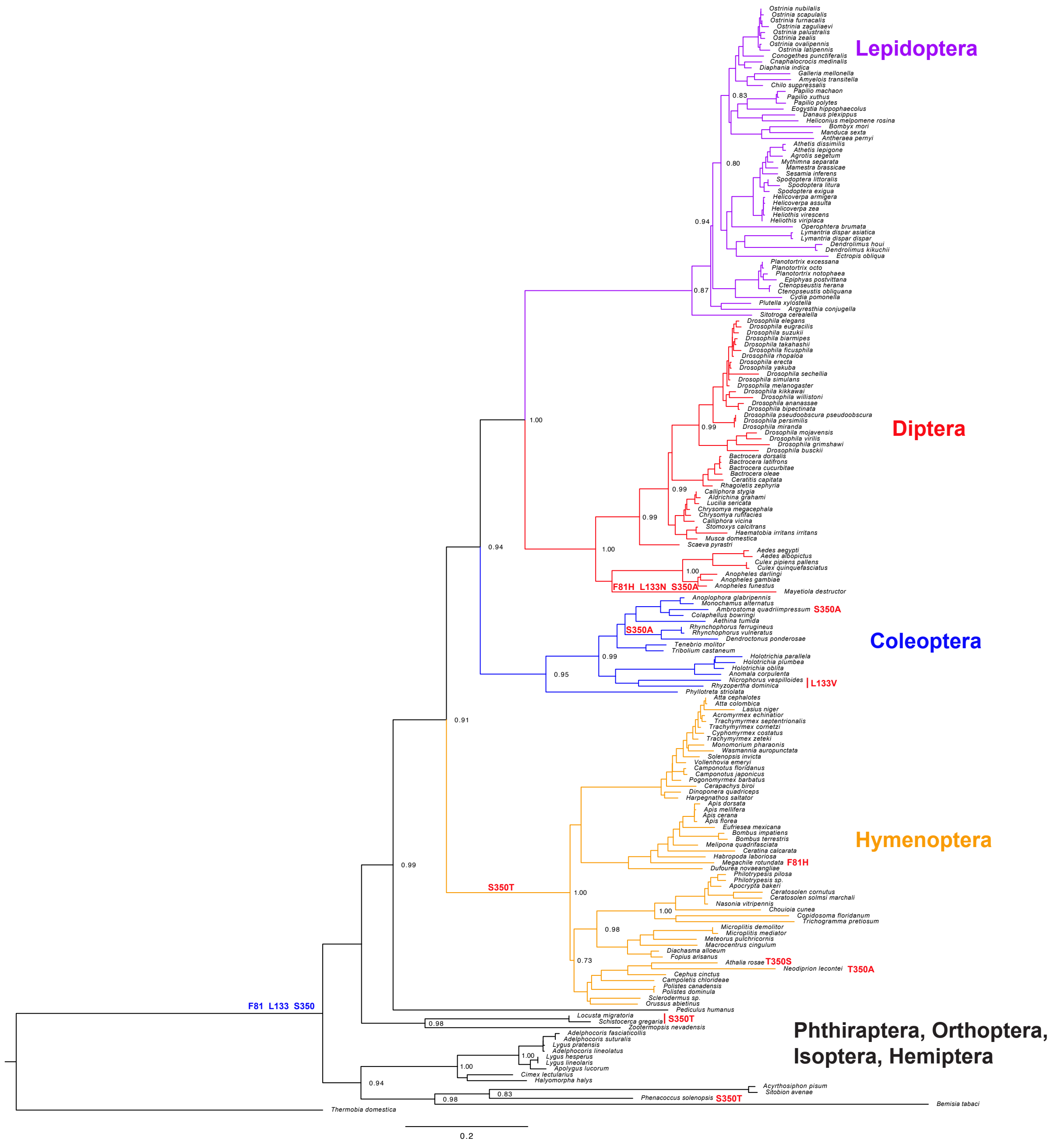

Figure S2. Phylogeny of Orcos from 187 insect species with the Orco-like protein 2 from the primitive firebrat (*Thermobia domestica*) included as an outgroup. The tree was constructed using FastTree from a MAFFT multiple sequence alignment, and rooted and color coded using FigTree. The most parsimonious evolutionary history of the conserved Orco residues 81, 133, and 350 is indicated. Based on occurrence in *T. domestica*, the inferred ancestral states (blue lettering) of residues 81 and 350 are F (phenylalanine) and S (serine), respectively. The highly conserved L (leucine) at position 133 is absent in *T. domestica* (which has divergent sequence in this region), but its presence in all other relatively basal insect orders analyzed here suggests an ancient origin also for this character state. Red lettering on branches or next to species' names represents divergence from the conserved residues at the position indicated. Numbers at nodes represent FastTree support values and are only shown if >0.70, and only on major branches.

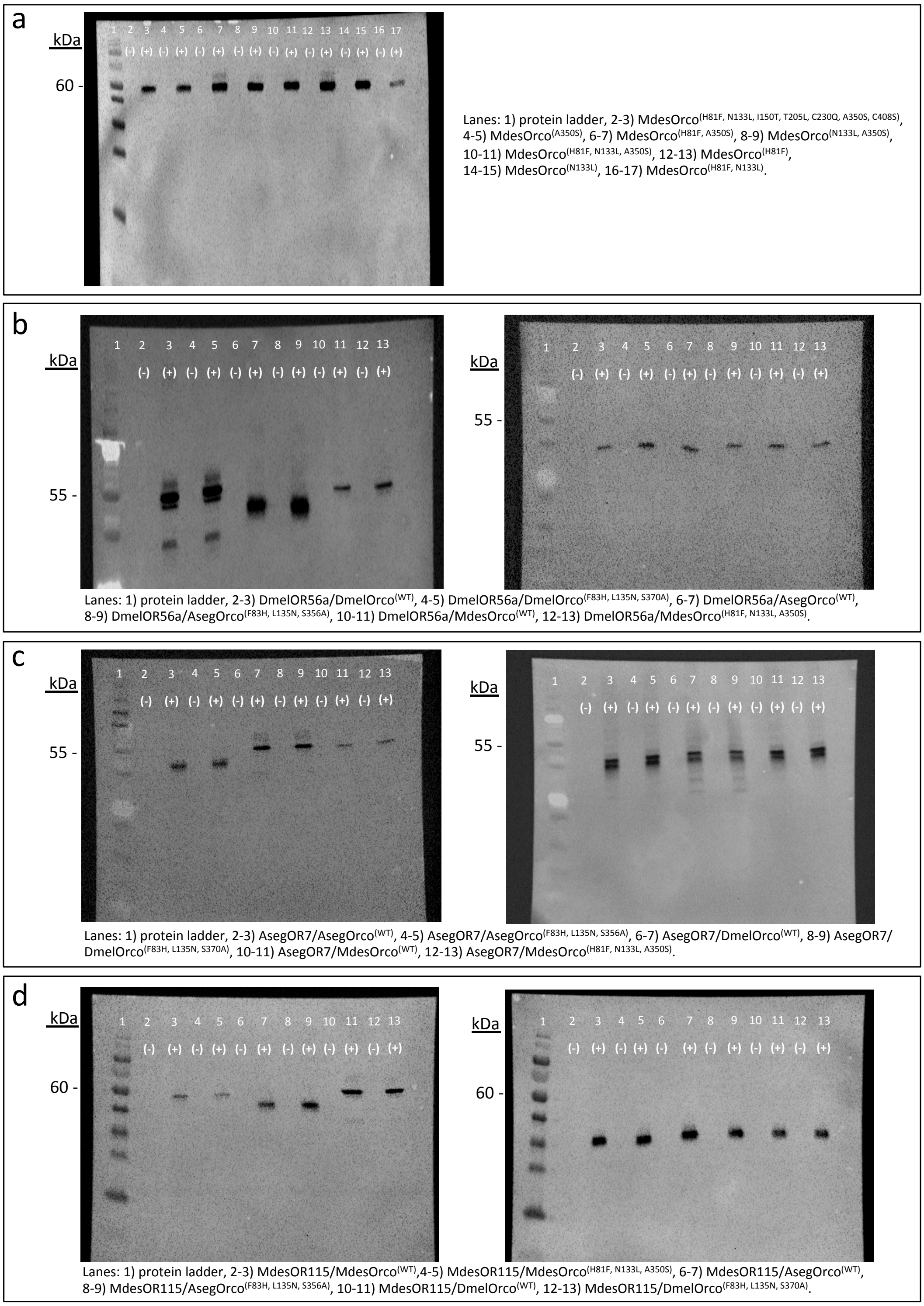

Figure S3. Western blots showing the detection of Orco and olfactory receptor proteins in lysates prepared from TREx/HEK293 cell lines used for functional experiments: **a)** detection of MdesOrco in cell lines expressing various mutated forms of MdesOrco, **b)** detection of Orco (left panel) and DmelOR56a (right panel) in cell lines expressing DmelOR56a in combination with wild-type or mutated Aseg, Dmel or MdesOrco, **c)** detection of Orco (left panel) and AsegOR7 (right panel) in cell lines expressing AsegOR7 in combination with wild-type or mutated Aseg, Dmel or MdesOrco, and **d)** detection of Orco (left panel) and MdesOR115 (right panel) in cell lines expressing MdesOR115 in combination with wild-type or mutated Aseg, Dmel or MdesOrco. Note: (-) indicates non-induced cells, (+) indicates induced cells. SDS/PAGE electrophoresis and western blots for each group of cell lysates (panels a, b, c & d, respectively) were conducted on different days.

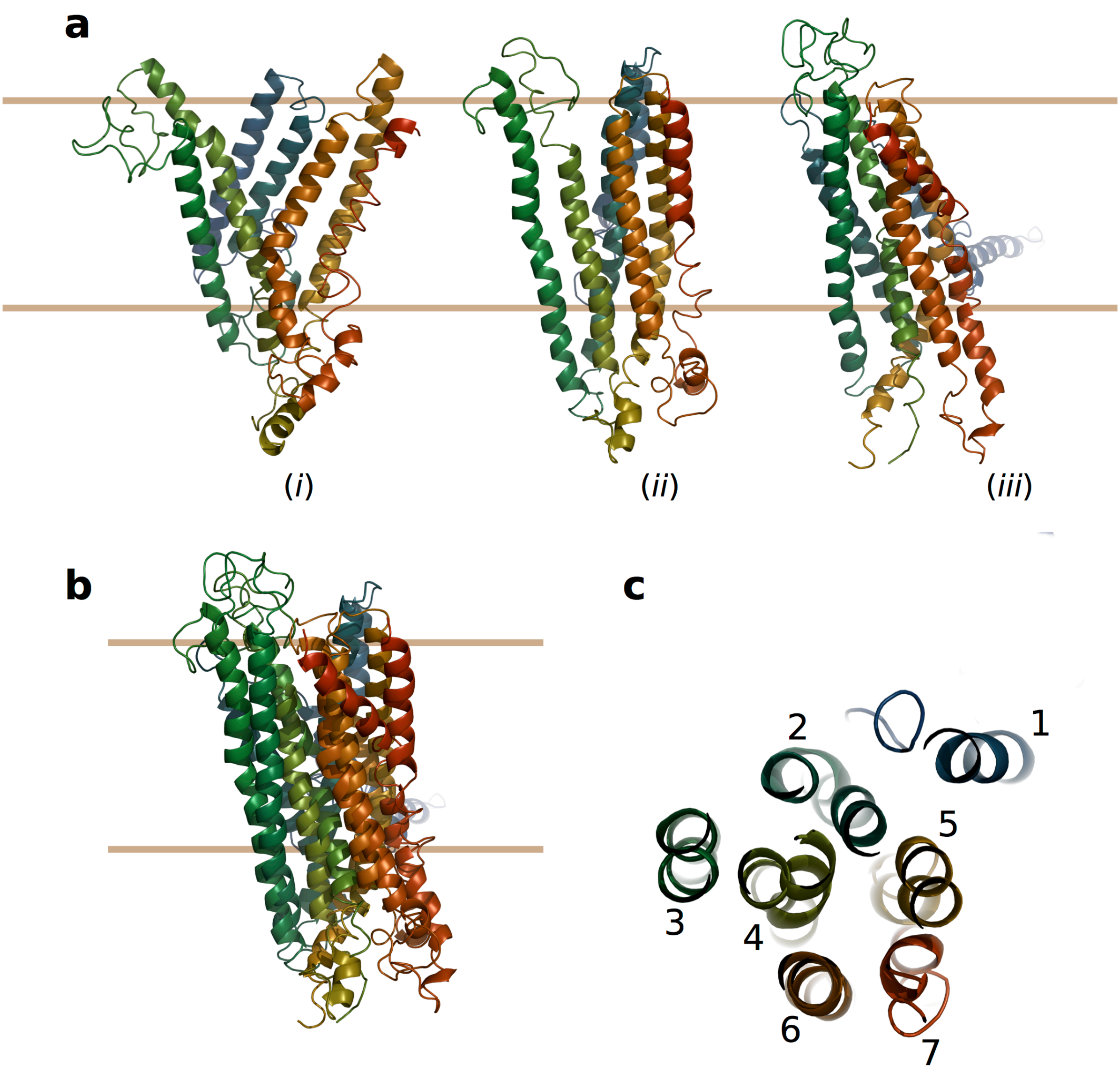

Figure S4. a) Side view of 3D models of *Drosophila melanogaster* (Dmel) Orco (i) and DmelOR85b (ii) as previously published[34], and DmelOrco produced using the modified multiple sequence alignment in the current study (iii). **b)** Overlay of (ii) and (iii). **c)** Cross section of (b) taken from near the intracellular boundary of the membrane, viewed from the extracellular surface. Note: protein models are coloured from blue (N-termini) to red (C-termini).

Additional Supplementary Files:

Supplementary File S5. Orco and OR sequences included in multiple sequence alignments.

Supplementary File S6. Multiple sequence alignment used to generate the AsegOrco models.

Supplementary File S7. Multiple sequence alignment used to generate the DmelOrco models.

Supplementary File S8. Multiple sequence alignment used to generate the MdesOrco models.
