# Supplementary material for: Endogenous insensitivity to the Orco agonist VUAA1 reveals novel olfactory receptor complex properties in the specialist fly Mayetiola destructor
Source: Sci Rep. 2018 Feb 22;8:3489. doi: 10.1038/s41598-018-21631-3 (PMC5823858; doi:10.1038/s41598-018-21631-3)
Supplement: Supplementary file 2 — Supplementary File S5 [file 41598_2018_21631_MOESM2_ESM.doc]

**Endogenous insensitivity to the Orco agonist VUAA1 reveals novel olfactory receptor complex properties in the specialist fly *Mayetiola destructor***

Jacob A. Corcoran, Yonathan Sonntag, Martin N. Andersson, Urban Johanson and Christer Löfstedt

**Supplementary File S5.** Orco and OR sequences included in multiple sequence alignments.

>1_369_Si_gnF.scaffold04648_643308-783672.pep

VLQLTFKILTIVGCWRPQSSFYLSIIYDIYTVFMIILLYTFLVSQFLDIIWNVDNAEDFTENFYATLASVVSCSKMFSLL

VNRKNINMLTNVLVERPYKPSEMDEMRIRYKFDRHIYTNTLCYTILVETTCACITVTSLFTVFKKGNLTYRAWLPYDYYS

SIVFCLTYIHQLISLTAGSLVNVACDSLICGLLAHICCQIEILECRLSKVSNNHETNNHETLRDCVRHHNSILEFAFKLN

NKFRMTIAMQFVVSTLVVCSNLYQMTKSTDINASYLPLLLYMSCMLTQIFIYCWYGNEVKLKSTQLLTNIFAMDWVTMDR

SLKRNLLLIMNRAVVPIEFTSAYVLSMNLDSFVGLLKTSYSAYNILKQV

>1_371_Si_gnF.scaffold01629_62255-192132.pep

TRYLRTNKLLLSLLGLWPMESSFNRNIFYCASFDIILFILVPQFTYLFTRMRDLNDLYDSLPTFLGAHIVVFKLFGLRWQ

TEKLKMLVQHVRYDWCSLMKDSDIWILMEYSEKSRIFTLAYLIFTSVSITSYVTAPITIRLFDIMLGSNVTRPKRLPHPS

EFFLDLEKYYYIILTIIFVGYIAAIITVNATDAIYFALMQHTCGILAILSYRLKNLIVHDKSENIVQCIQLQARTERLIQ

LIESTFAICFFSDIALGIPFYCSACVMAINNRDQPTEALRFLTTCISLSMHFLFLSLPGQKLIDHSSNMHESIYNTEWYN

MPIATRKLLIIMMIKSEKPLMLKMGKVV

>1_372_Si_gnF.scaffold02944_1-38457.pep

KKVTFKEVISIAKFSMFPLWFWPSKNTKLKTFCRKLYNYWCILMGLCLELPSLYGASQHTDDPALLAQTLVMSSTSWHIM

FNCIYQLVYNHRIQSITYEMENFCDLMKPHEEVVIQRYIDKCGVFYGAAIEYYALIVVQIVVLPPLMHQSFPTLVEYPFD

VSYQPLKTIIYLQQSIVGTYMGAQLCLNIFMNLLFWFVTARFAILTEELKDATNINLFKCIKTHQKLLKYATEVTHVLRP

FAFSSICCSTYSIIILFVLLITHQPIALVIQFVGLAITCLAEVYMYVWPGENLINACKEVGEAAFYLLENNDLLQIWKCL

QIIIIRSQKPITISIPCFMPLSLNYFASFISTILSYFTTLRIM

>1_375_Si_gnF.scaffold01629_62255-192132.pep

NSHYNIIRILLGISGLWPFHTRNRRYAIYLTMILVLGTGYIFQILGIVEVWNDSFELINAIPFLFFTTIALGKMICTVYT

LPQIKVLLTRMREYRLSPKSNEETKIQNSHAQYGRKFGYAYTGHSVLYLLSSLSLKIFSNETDELSDKESNYRIGLPHRA

NYIVDVDTYYVPIFIHSAICDFTCTYLLTVFDVLYLTVVEYCCGLFASLRYRLEIALEFNNNKNKLTMSKDKSYSNVVYS

IRRHTESIQFVAIVESIYSLSLSIQMGLVILILSLLLYQVLNDIGNIDRILKPIAYLNGALFNVFFENWQGQKVIDSSEK

VYNTEWYNMPIAARKLLIMLMMKSKTPLMLRLSKMVVLSYITF

>1_379_Si_gnF.scaffold01629_62255-192132.pep

TRYFRINKLFLSLNGLWPYETPYKKLLSQSFSLFSVAYMFVPQVIFLLKHTDDVDEMVEVMPTLTGTVLCLAKMYSLIKN

SKMFKELLQHTQDDWNNLLTKEETQILLRYAEKNRIITLVYSIFTSVSITSYVTAPITIRLFDIMLGSNVTRPKRLPHPS

EFFLDLEKYYYIILTIIFVGYIAAIITVNATDAIYFALMQHTCGILAILSYRLKNLIVHDKSENIVQCIQLQARTERLIQ

LIESTFAICFFSDIALGIPFYCSACVMAINNRDQPTEALRFLTTCISLSMHFLFLSLPGQKLIDHSSNMHESIYNLKWYK

MPIVSQKLLIMIMMRSKKPLTITAGKILVLSHMTFNTV

>1_379_Si_gnF.scaffold05285_6032-497272.pep

LALNFLFYTIGGVWRPIKSKCSICLYSVLNFFSLYLLTFFVLTQLIDTIFIVDNIDDFTTNLSLLLSAIAVYCKAVTATA

RRSEFISLIKMLQEKPCKACNEEEINIQMKYDRLIRQYTMSYSILASFSLTGITIGEVLIALQGELPIRAWIPYDYTSTF

LFWLTSLKLIVAMALSTFVNVATETVILGFCLQICAQFDILICRLRKVIESDEKQENESDEKQENELNSATNKTSRLSEN

IHYHLYIIRFAKMVNKVFSQIVFIQFFVSILVLCTSVYYLSSHKTVTDFIKLGIYTSCMFVQIFMYCWAGNEIILKSIGL

SEAVYKMEWILLTISERKDLLMIMMRSTKPIKFTSSFLVTLSLESYATV

>1_383_Si_gnF.scaffold01629_62255-192132.pep

NHYYYPNKILLSIIGQWPFQSRLEGNIMFAVTFISFVSYAFIEIWGLIAGISDLNIIMENFSPFLVLCYLIINLLYCAFT

KDKLKVFLENIEETWKMKPAGPEKEILQCYAEKSRAFTIQFVIALYVTFVFYTMPSGVARWMYKLFPTNETYRGKFMYRI

EHVLDMDKYFNLLMLHGTIAVFFMVSVAIGVTTVFTLCTLHICALFECIRYNVECIRSTDPVLKDDEMYHDLIACIKSYK

HALKLVSMLVFNIQERYSLTLRNINTKINTFQVLMNLNDAKDIVAPLAIYVAQLTHLFFQFWQAQFLLDYSLAPYESMSN

WYYTSERCKKLLLLIMTRTVSPCRITVGKVATLSIESFGVVNKTTLAFISF

>1_384_Si_gnF.scaffold00330_12820-264637.pep

TLKFMLTLCGIWPGTPYVLLCRIFWVVSMTVILYFHYRYFYWHVRSAEILDLMDCLSSFIAYSKIIIKFFVFWLNERKFI

EVLEVMREDWSDCANNDICMRETVRKAKVSERVTKVMMILHTTTIVTYSIGTILADIDVTNNTTELLFYTKVEFPFDVNT

QRTYRFILAIDFFFLLMCSWCAGIMNTLLLILTIHVAGQINIVRHWLMQLASPENKSKNVPENKSKNVSIATTKIIQKHQ

KIIRFTENIESLYTYIALMLFVSNMIMICLLAFVVVTAPNATEVIMRSLLFYIITNFEAYIFCYAGEYLKNKSREVGFAT

YNCEWYNLKSKDSQILLFIILRSQKQLTLTAGKIMDLTLQSFASIMNASGSYLSMLLAM

>1_389_Si_gnF.scaffold04510_616038-792405.pep

WAIGLNRRMLKIVGLWPEESNHREELLSKLRFLVNVMTIFILIIPALVSLIRVWGDMILMIDNLQFTLPLLITALKVFIM

WYKKGALSPLINMIVKDWMRAKIKEERNVMLKQARIVRLLAICGGIMILFTLLVTVCSFLFGRTLRYVTNFTDPFGKPLP

IQTYYLHDVSTSPKYEITYLIQIISLTISGVSYTAVDNFLGLLILHVCGQMENLHLRLLNLGKNQNGKNQNFQALLKYNV

NDHIRLIRSIHVIDNTFNLMLLGLLFFFGILFCLHGFLIINVHLSLMQFILYISASVAVLMHTCLYCAVGELLTTQSEKI

HRATYEYVWYTLKPKAAKNLTLIMLRAKKPLTITAGKTFPMTMSTFCNLLKTSAGYVSVLLA

>1_394_Si_gnF.scaffold01629_62255-192132.pep

MAEQKDIWQSRYYAIPRLYMTLAGLWPYHPIRDRYLRFVPTFIICSIILIPMLLYVSLAMEDLDDLFECMPTILITIIFS

FKLASLMANSEKIKVCLKTIEDDWLSLNDKEKAILQRHTTYGQNLTIFYAVFMVMTGLLYMFKSIVLIMIEDTSNSTKLA

VTKLPFRVEYGSTIDQYFYPILVHCYLTVYSHVTATVAADSFYFILIQHACGMFSVVGYMLEHIGEDNDTNFDTKPNKIT

KPNKINDNNYKRALYCLRKHLHVIEFAELIESTFTKIFLVSVSLNMIGGSICGIQVLMNLNDAKDIVAPLAIYVAQLTHL

FFQFWQAQFLLDYSLAPYESICRSNWYYTSERCKKLLLLIMTRTVSPCRITVGKVATLSIESFGVVNKTTLAFISF

>1_396_Si_gnF.scaffold10455_73458-77570.pep

TDKKDIWKSRYYLILRTYMTISGLWPYRSLRERCIHFIPTFALCFSILIPQSMYVLIASDINEVIEGLTSALISIAFSWK

VMCIMFDKNVKSCLKTIKKDWLSLKTDVERSILQRHVEYGRYLTTSYAVFMHTIQVSYILKPFLLTLLEINTTNSTKTSA

SKLPFHVEYGVDADQYFYPIAIHCYLAIFAHIFSTIAIDSLYCTLIQHACGMFSIIGHVLEDIGKNNNASLNLNKISDDD

YKKTLDCLRKHLQVLQFTDLIESTFTKILFVSVNLNVICGSVTGIAMIMNLNKGIEIAAPLSVYVAQLVHIFLQFWQAQF

LLDYSIVPCESICRANWYFTSKRCQKLFLLIMTRTVTPCRITAGKIVTLSIESFGSVLKTMMSYFTMLRSF

>1_397_Si_gnF.scaffold02694_2287513-2447739.pep

PYQDPSDYSIQLNRWFLTPVGAWPRSATFREKVISTILIIICYSLISFTVLPCILNILFEETDMHKKLKAIGPLSHWSMG

GMNFFSLLFRNRQIGRCIQHMKIDWQTVKNSEDRQVMLKYAKIGRFVAGICAVFMHGGVFMHSLLQGITPTFEYIGNVSV

SVIVLPCPTYSKFDIGQSPENKIALTIQLMSSIVVNSVTVGACSLAAVFAMHACGQLNVLMRWLYQLDDQKQDDQKQQNT

VQRRLANIVEHHLRVLSFVAQMEELLNQICFVELLGCTFNLCMLGYYVITGWNVIEKTTLMLIYISMCFNIFIFCYIGEV

VTEQCKQVGETAYMTNWYNLHHKTARGLILIISRSSTVIKLTAGKLVHLSIATFGAVMKTSMAYLNMLRTM

>1_398_Si_gnF.scaffold02694_2287513-2447739.pep

TKKNEDDFNYAVQVIRVIMRMIGAWPISSSNVERFAIRLQNIICQFLFAFVIVPTLLLIFLKERDFKRRVRLLGPLLNCL

MGWIKYNLLIYHMREIQSCLKQARQDWRDTVDWGDRKAMLSKAKIGRRFAIFSAAFMYIGGLSYRTLVPLSKGRMLTPMN

TTVRALACPSYFVFDEQATPAYEIVFTLQFFSGLLTYSVTVGAAGLAAFFVLHVCGQLQLLIGKFQRLNDMSEPNDRSFA

DIVEHQIKVKNFLKQVEETMQYVWLVEIVSSTIFLCLTGYNVIMENSNSTAMLTYTVMLTSLTFSIFTNCYVAQLLTNQS

IKFGLMTSTMNWYRLPHKRARTLILIMAVSNIPVKISAGKMIEMSLPTFSNIVRTSMAYFNLLRK

>1_400_Si_gnF.scaffold02694_2287513-2447739.pep

EHYKEDMIYITRLTRHVLSLLGVWPSYSRRRSIRDKAWKYFLISMILLYSVLIGGFFWLIEKRTKVRVQTIPLLFYGFMA

SGKYSNLVFREKNIRRCLKHIEEDYRVITSTEARDTMIESAKIGRRLVTLCAIFMYGSGLSFSILPFAKGKIVTAQNITI

KPLPCPAYFFFDIQISPAYELIFAMQFLSGIVTYSITIALCGLAAVFVMHACGQLKILVDMMKNLVEQQWEEKQEEEKQE

VDRKLARMVEHQIRIRSFLQLVENTLQQACLIELMGCTAIVCLLGYFIIMENSNSIAMCSYFITVTSLMINMFMFCYTGE

QLTVQAERVASTSCELEWYRLPDKKARAIVLVIIMSNMPTKITAGKIMDLSFKTYGDVVKTAVTYFNMLVN

>1_406_Si_gnF.scaffold02694_2287513-2447739.pep

GSDYSLQLTRWFLIPIGAWPRMSTRVKRISSHMHIFVCTSLIAIIMVPCLLYVSLEEKDTEIKLSVIGPLSHWIMGMINY

CLLLTRSNDIRECVLHMEMDWRLVRKIEDRQIMMRQAKIGRFVAGFCAMFMQSGTFLFAVRKSLSTTIVIVGNETVSMHP

MTCPFYNKIDTRFSPANEIMVVVEWLSCFIVNSVTVGACSLDAVFAMHAYGQLNMLFSWLNKLVIDEDKENEDKENKCVE

QRLAIIVEHHLRVLSFISRMETVMRHICLVELLGCTMNMCFLAYYFITNMDSFDKTTSYVIIYLSMAFNIFIFCYIGEIL

TEQCKNVGERAYMINWYELPHKTALGLILVIARSNNIIKMTAGKLFHLSIATFGDVIKTSMVYLNMLRTM

>1_411_Si_gnF.scaffold02694_2287513-2447739.pep

NIKFTYKSNNDYSLQLTRWFLLPIAAWPRPTTTVERISLQAHVFACVFLILIILIPCILYVWLEEKDIQIKLSVMGPLSH

WIMGTINYWLLLAHSDDIRECVQHMETDWRMVRGTDDRDVMLRYAKIGRLVAGFCAVFMQSGTLLFMVAKAVTSMTVIIG

NDTVTMHPMTCPIYSKFDARFSPANEIMILTQILSCFIVNSITVGACSLAAVFAMHAYGQLSMLFSWLNNLVEDEDKENE

FAEQKDKENEFAEQKLATIVEHHLRVLSFISRIENIMQTICLVELVGCTLNMCLLAYYSITNSDFDAKITSYVIVYLSMA

FNIFIFCFIGEILTEQCKNVGEKAYMTNWYELPHKTALGLILIIARSSNVIKITAGKLFHLSIATFGDVIKTSMVYLNIL

RTM

>2_369_Si_gnF.scaffold04648_643308-783672.pep

MKFTLMICALAGCWQPLMWTSLFKHIIYAIFMISSLHIFLFSQFVHMMLNVANSDEFTDALYMMLTILVAGYKQIYLWVD

RKNVMAIINVLIEKPFAPYETHEIAIQEKFEKMIQNNTRRYLTIVVMSISSIILMSVSTDFMKRNLTYKAWIPFDYSSPA

IYFIVYIHQLIAMSTSGIVNVACESLLCGFLLHICCQFEILGYRLTKLTHDQNSLSLRDCVCHHNRIFEYVYTVNNMFAK

IIAIQFAVSMLVVCSNLYRIAMATDYMSFIPLMMYTSAILVQIFILCWFGNEVKLKSLQLVNSIFDIEWPALSNSNKRNL

LLIMKRAMTPVEFTSAYIITMNLDSFVALLKMSYSVFNLLHQT

>2_385_Si_gnF.scaffold00330_12820-264637.pep

ESVNTVCRPVKFGLRMIGVWPSTSYAILRRVFCISSMAVFQTFQYRHLIMHFGDLLLLMDVLSTTMAYTLLMIKLIIFTF

NTRLLNEMIARVIEDWKQRDVCDKYTMTRMAYISRRFSNFIIGLYALSVFLYATGTLLRYRSSNQTDTRELILKLELPFE

IKSTTVYVAVLVTQFIHLTSAASMVGVMNSLLITLVLHVCGQIDIVRQKLSEITRKDVKVKMLIIRHQRIISFSKNIEAF

FSNIALIQFVSNTLVICCLGFLIVISPGGSSMLIKSVFFYIVISMEAFIFCFLGEYLSTKSQKIGDAVYESLWYELDPNQ

NRDILIMIIRSQKHLTLTVGKVMDLSLKQFASIVKASASYVSVLHAM

>2_401_Si_gnF.scaffold02814_302873-346762.pep

VQREYNVNKIFLSRLGLWPFQSKLVRNLLPIVCLILEISYYSFEILMLHDHRDDSQMVFESCYQFVITTAFVVRLWNEVW

NRDKFRRLYEAMNDHWDIFTNEIEVRILKHYSNISRKFTIVYSTMMYLLCSMFIIIPLTPTFLDIILPLNESRPRILAIE

VEFRVDTDEYFVLIFCYTTAIIVVGVSIMVAVDAMHFTCTTHACSLFSIVGEQIENITSTQHEEHELSRKEQVLYQQYVM

CVKKYQIALKFVDILNSTHQTVAVFFLLLICATLSLIGVRIVYVLGQLEEMIRFTFIIVGALLQLLIMCYSGQKLIDESE

NIFHRAYAAKWYLFSPRLKSLLFITLYRSIVPCNLTVGKFCPLSMSTYATVIRTGISYFTAFLSIK

>2_405_Si_gnF.scaffold02694_2287513-2447739.pep

AYQRDSEYSIQLNRWFLKPIGAWPETNTGVDKVLSRSIQFLCHSLIAFTVVPCVLYIMFEPNVYLKLKAFGPMIHWLMGG

ANYCSLLVRSYEIRKCVDHMRVDWRYVGKTRDREVMLQNAKFGRFVSAFCAVFMQGGVCSYSIITTLTPAIIRIGNVTIT

THQLPCPFYNEVDTRYSPANEIVIVLQLLSTVIVNTVTVGACSLAAVFAMHACGQLNILMMELNELVDESEVDESEYNVT

RRKLAIIVEHHLRILNFVSQIEMIMHQICLVELLGCTIDICMLGYYTITELQDTKNLLTYFTIFIAMSCNIFIFCYIAEI

LTNQCRKIGEIAYMTEWYRLHHKIAIDLILIISRSNAVIKITAGKMIQLSIATFANVMKTAFAYVNILRTV

>3_368_Si_gnF.scaffold04648_643308-783672.pep

LDVTFRILMICGCWVPDSWTTPYKRLVYHVYTIFIMLLIHTFMLMDLILTVDNANDFTDNFYMLLAMIVSCCKMFTLLIN

RSNIAMLIDILVRKPCKPVQSDEIEIQQKFDKHVQTNTLVYAFWVETTCLCIAVTSLLTEFRKGRLTFRAWLPFNYSSSL

LFRIVYAHQLISLTAGSVLHVACDGLICGLLVHICCQIEIIECRLRKVAHDQNILRESVLRESVLQHNHVFKFAHLVNEK

FRVTILIQFIVSTLVVCFNLYQFTKSTALRAQYIQLIMYMGCMLTQIFFYCWYGNEVMLKSRHLVHSIFEMEWCELNKHT

KHSLLMIMRRSSKPIELTSAYVISMNLDSFVGLLKTSYSAYNILKQV

>3_379_Si_gnF.scaffold01629_62255-192132.pep

NRYYHFNKIVLSLVGQWPFQSPMKSNVMFATTVLFVFTMITLEFWGLAAGITDLNIIMENASPLLVNCMMIIKFINCVIT

NDKVRQLLERIEKTWKIMHVGHENTILRLYAEESRKSTVRYIIILYSSMILYMTLPVMPSIIASFMSANQTQIYGLLFHV

EAVLDTKKYYYYILLHSYYTTFFMITIPVAVDSMMIAYVQHACGLFQAIGYQLKNVKRPLREDDEHYRTIIDSIVKHKEV

LQFVELLTSSYSISFLLLTILNMAVMAFSAVQAINNRDQPTEALRFLTTCISLSMHFLFLSLPGQKLIDHSSNMHESIYN

LKWYKMPIVSQKLLIMIMMRSKKPLTITAGKILVLSHMTFNTV

>3_387_Si_gnF.scaffold01968_226495-654737.pep

AIELSQLSMTFFGVWPENNTTQKTIMSNIRTFILNIIVWILAIPCMHSLIRIWGDIMSVIDNLQYSLPILMAIMKLVIMW

QKKKDILLLLNMIKDDWLKPKKXKRERCYEKTRATSTYIYNIRIFXMLVSYILVVVLPIFGISMRYLTNKTDPDKLTPLQ

SYYIYDKNKSPFFEVSYIMQSLGLMVAGVTYSSVDSFFGLLVFHVCGQLENLKMRIIHLDKFKNKNFEIALSHSVQDHIR

LIRFINMIDDIFTLMLLSVLLYFGIVFACYGFLLGTIGMSMFRFIYLVSIVLNVSTHMCLFCAVGEILVTKCEALYQAAY

EHKWYTLEPAKAKNLLLIMIRANKSLYITAGKMFPMTMSMFCNVIKTSAGYVSILFAM

>3_401_Si_gnF.scaffold02694_2287513-2447739.pep

ENKEYSIQPIRWLLKPISVWPVTDSSIERILSVVLLIVCIFLIASTLVPCALAIFLETKDLEMKMRDFGPLSNWILASLK

YVSLLTHVGDIHRCIKHIETDWRAVTKLEEQEMMLRNARIGRFIAIFSATIMHSGVFSYGIFRGMTLSAQSTESNNVTVR

PLPFPFYEKVDTTTSPMYEIVFVTQCLSTFVVNSVGVGTCSLTAVFVMHACGQLNILMSLLDNLIDKENEKRNSSQKENE

KRNSSQKFAVIIEHHLKVLSFVSHIDKITNVVCLVEVVGCTLHMCLLGYYCILDQGEMEGMVSYAIILISVIFNIFMFCY

IGEILSEQCGQIGETAYMTNWYLLPGNTALSLVLIILKSSIAVKITAGKMIDLSLSTFGTVVKSALAYLNILRTL

>4_386_Si_gnF.scaffold00330_12820-264637.pep

EFTLRFIGVWPDSSCRFLMRVIWMTAMVISQLLQYWYLFTGSDSLLDVMQAMSLCLSNSLLFFKLSLFWLNGRIICDILA

MMAEDWNECESAHTKLQLMISKAILSHRVSKGSIAAYTATVLLFGVCDVIIQKTAGPEQLVEEKGFIVKMQLPSECSTSP

FYEITMATQFLMQFTLALVAGMLNALIVTLILHIAGQIDIMCHELLEIALRSIVNRHQRIIAFADNVEDLFCYMALMQFL

SNMFVICFLGFVIVISSEANIVIMKIVPYYIVVNLEAFILCFSGEYLSSKSKRIYCAAYNSLWYELKPMESKILFLLIIR

SQKELTMTAGKFIDLSLESFTSIVKASASYVSVLHAM

>5_379_Si_gnF.scaffold01224_363367-449963.pep

AVGWNRFNLSLIGIWPGPLKQLSRCRFIIAGFFMLGFMCIPQSVNLIFIWGNADMMTENLATAIPVANSFMKAFTTWRQR

KALKLLVNFFYQDWYTPKTSAERTNMLKTAKLGRRISIWCTVLTQTMVTIYIVLRLSIIIRIPRSDPARPMLYTAYFPFD

INRSPIFELICVCQILSAYSATVSYTGNDCFISMLVLHICGQFRNLRERLKNLVNESNASNAKTPDKFKKELRRIIKRHE

HLNWFAKTIEDSFNMMMLIQMLSCTVQLCFQGFQVFRIGSVTFQLIFLVSFVAFVLTHLYIYCHIGEMLLVQSTEIGFST

YESNWFNLRGKQAKNLLFIMQRSTTPLTLTAGKFSAFSLQMFSTV

>5_385_Si_gnF.scaffold00330_12820-264637.pep

ELMLTMFGVWPGVSCVVLNRAFWLVTLVINQYLHYWYFHFRVENLFDLMDCLSSLLAHVKLTFKLIVFSLKQRIFVEILT

TTAEDWKDCDNSEIALRETASKTKLSSRICNGLIILHTIAALAYVIGILLADADVTDQTVELPLMMKMEYPFVIDTLHKY

KLVLATQFVFVMVNSWGAGLFNALFLTLTLHVGSQINILLHWLTEVGPTDETKMTKIIQKHQKIINLSENIENLYSYIAL

LQFTSNTVMICSLGFLIVTAPDATEQIIRSLLFYAVTNLEAFIFCFSGEYLSNKSKAVGNAAYNSSWYNMRAKDSRLLLF

IILRSQRQLKLTAGKMTVLSLECFTNIIKASGSYLSVLLAMK

>5_386_Si_gnF.scaffold00330_12820-264637.pep

TLKFMLTLCGIWPGTPYVLLCRIFWVVSMTVILYFHYRYFYWHVRSAEILDLMDCLSSFIAYSKIIIKFFVFWLNERKFV

EILAKMAEDWSDCANNDVGLRETARKAKLSDRITNSIVILHTMTIVLYCIGIILTDVDVTDTSKELPFKLDIPMDINTLF

KYRIVLTVQFLYMMLSSWAAGITNSLLLTLILHTAGQIEIMRHWLAQLVPRKSEDKSEDKHKSITATASKIIQKHQKIIS

FTKNIENLYSYIALLQFVSNTIMICSLGFLIVTASNAVEQIMKSFLFYTITNLEAFIFCYAGEYLNNKSKEIGVAAYNCE

WYDLKCTESRVLLFIILRSQKQLSLTVGKMMDLSLEAFTSIMNASGSYLSVLLAM

>5_389_Si_gnF.scaffold00899_962711-1051965.pep

RNDDVAYAMDPLKFLTVPLGIWPLQKYGIFPLIRSIVSVFSLVVWLITLFLEVNYSNSDAYVKLDQLMLLSCAILSTLKI

VFFRLYADNLICNFFSAVSDYLAIDTEEKRTIMRRHAFIGRMISYSTISLAYVAATMFILLPMLSDENAQVNVSAADLPL

PMTWILEYHFSSLYYTIFIVQYYLLLLNANANVGNDSLFLAIVLHICGQMEFLKTEFTNYGVKSKNLNEKSKNLNEDFLV

LISRHRYLMEHAERLVDVISFVLLVQVLISCIIICVIGLSFIVATHDMMMITKCGSVLSALLLQLFFYSFVSDYLKCQME

DVAHSIYSCDWYSFPLKLMKNVLFVIMRSQQPIQLLAGKFFVVNIETYMTILKTSMSYLSVLRVM

>5_398_Si_gnF.scaffold02694_2287513-2447739.pep

NANHEKDVIDILIWNRWLLRVLGIWPLIYTTIEKILATFSFASCWTVLGLFLVLTSIYTFTDRSIMGEKLKMLGPLGYVF

FSMLKYFFLVIRHKSIRRCIRNLSTDWHKVQEEYHREIMMRDAEKGHLLSKFCIVFMYCGGLSYNTVMPFLSQTHDANEQ

NITIRPMAYGFDIVFNLQLMPIYVFAFSLQCFTGIVMFNITTSVCCLAAMFVAHACGQIDIVIARVESLFKEERERNHIK

FEKCMAIIVQHHVRALKFSASIEDTLRELCLIELVGTTLIMCLVEYSLITENSDSIAIFTYFFLLVSFVFNIFIFCYIGE

LLTEQCSKVGYTSYKVEWYNLPGKIALDLLFMINTSRYPVQITAGRLISLSLASFGNVLKTSVAYLNLLRT

>5_405_Si_gnF.scaffold02694_2287513-2447739.pep

NANHEKDVIDILIWNRWLLRVLGIWPLIYTTIEKILATFSFASCWTVLGLFLVLTSIYTFTDRSIMGEKLKMLGPLGYVF

FSMLKYFFLVIRHKSIRRCIRNLSTDWHKVQEEYHREIMMRDAEKGHLLSKFCIVFMYCGGLSYNTVMPFLSQTHDANEQ

NITIRPMAYGFDIVFNLQLMPIYVFAFSLQCFTGIVMFNITTSVCCLAAMFVAHACGQIDIVIARVESLFKEERNEERNH

IKFEKCMAIIVQHHVRALKFSASIEDTLRELCLIELVGTTLIMCLVEYSLITESXQYSDLYILFSISFICLXNIFIFCYI

GELLTEQCSKVGYTSYKVEWYNLPGKIALDLLFMINTSRYPVQITAGRLISLSLASFGNVLKTSVAYLNLLRT

>5_408_Si_gnF.scaffold02694_2287513-2447739.pep

DWNYSIQLNRWFLKPIGAWPLTLTTMEKIICIVLTIGSAFLICFLLIPCTLCSILVDTDLDTKIKTIGPNSFILMAAVKQ

YILITRSENISKCIRDIRADWDHVALGHREIMMTTAKFGRWLSCVSAIFMYSAGIFFTTMPICARRTQVINNETVRSLSF

PIYRGFDPRATPFFEIAQFTQALAGYVIYTITIGVCSLAAVLVMHACGQFQIVMSKMEDLASEKEKKSASRHKSASRHEG

RLGDIVEHHIRILSFIARTEKLLNEICLVDVIGCTLNICFVGFNMMTENRETLGTMTYLSFVISFTFNIFILCYIGELLA

EQCIEIGIKSYMINWYRIPNKGALGLILIMTMSNATIKLTAGKFMSLSLASFCSVMKASLAYLNLLRTF

>6_378_Si_gnF.scaffold00330_12820-264637.pep

PVEIGLRLTGIWPNSLVLFRMLWTLVMGIVLIFQYHYLLIHFSTEELPNLIDGLSTTLPYNLLFIKMIVLWVNNRIFNDV

LKAMSNDWREYSGMYAMIDKAVLAHRCSKLTIGVYSTAVLLYSTASINFRKQSNDSCRELLIKMELPFNFCESPVYEIVM

WVQFVHLMAVASSIGMLDGLMVTLMLHIGGQIDLMRQEVEEICPNDDRSLINKHQKIIAFTENIESLFSHIALMQFFSNT

IIICCIGFLIVTSMGTDEMLIKTMFFYIAITLEAFIFCFAGEYLSNKSKTIGDAVYESVWYNLKPRDCRVLLFVIMRSQK

RLTITAGKFMELSLQGFTNIVKASASYVSVLHAM

>6_398_Si_gnF.scaffold02694_2287513-2447739.pep

KNYKQDIEYVTKQTNFLLRILGIWPLLDSVVEIACKILLIIICCILVCFESIPSMLYCIFTVEEPRMKLMMIAPTIYSFT

SLTKYGALIVYENEIRKCFRHIKDDWKFIAMSGARDIMMEKVKTARSMFTICCTCLYTAALSYHTIVPLTRGKVITSNNV

TIRPLAYAGYFVFDEQRSPAYEIVFTLQFFGGFVMYSVTIVTYGLAALLVMHACAQMKILMMLMEELVDEKEKNTDEKLV

AVVERQIRIRNFLCLVEDTLQISNLFEILANTTMMCFMGYCILTEDGNTANAYTFVVALASDIVNIFLLCYIGEYIIDTA

EKVAIASCELEWYRLPDRRARTVILLMIMSNAPTKISAGKFVDLSLKTFGDVIKSSMVYLNILRAM

>6_406_Si_gnF.scaffold02694_2287513-2447739.pep

VRYEQDVHYAMQLCRWILKPIGIWHLIHSQSEKFLSFILILACLSGLCFVLVPAGPYILFREKDINIKVKLFGPVGFCLT

SAIKYCFLGARVSAIGRCIKHVESDWRVVRYQDHRKMMLKNSLVGRRLTTLCVIFLYTGGMSYHTIMPLSSGTKTNGSFT

SRPLVYPGYLYVDPQASPAYETIFFMHCLSAMIQYSCTTAACSLAASFATHACGQVQILMTLLDDLVDGKVAKRLSLIAK

HHVRVLRFTADVEEILREICLMELVAATLIICLLEYYCMMENSDAVAILTYFILLISLTFNILIFCYIGELLMEEYGKIG

SAAYEINWYDLPGHKAADLILIITMSHYPPKLTAGKFCDLSLNTFSTVLKTSVAYLNLLRT

>7_388_Si_gnF.scaffold00330_12820-264637.pep

VQIGLRSIGFWPNTPCALLFRAFWILTIGIVQTCQYWWIIIHFRTDNMFYLMDGLSVAIEYTVMSLKLIILWLNSRIFYD

VLAAMAADWREAAISEMDTMMSKANLSRRFSKVIVGLHSIAVVCFGIEVLVSAHTDDYDVDGMETPVRAFTLKLQRPLQF

NESPLYEIVVCLEFLHQLASSTVSGVLNCLLITLILHTSGQIEILCDALRDISFENKNLGFFMKELIVKHQKIIIFSDKI

ERIFSYIALIQFLSSTLLICCVGFTVITSKIDSTALIKAIMFYMTAMVEAFIFCFCGEYLSAKSKMIGDAAYKSLWYDFK

PNESKFILLIILRSQRRLTITAGKIMDLSLEGFTTIVKASASYVSVLHAM

>7_388_Si_gnF.scaffold01968_226495-654737.pep

EWAVKLNRITLDFIGLWPQIADPRKKFMCNFRVLIVFVAVTFGLIPSIHSFIRIYGDIMLMIDNLQFTLPAISCTIRIII

FWWKKEAIIPIMEMITEDWLKSKSAEDRNVMIKRAQSARIILMCVYCITAVGCLFLIVPPGFGMSIRLTPNITDPGRLMP

LQTHYIYDITKRPQYELTYMSQSIYIILAVLSYIGIDHFLGLLVFHLSGQLDILKNRLIYLHKYINYINSHEMLKECIKK

HIRLLRAIAIIEDTYNIILLSLFAYFAILFAFYGFRIMNLALSLTQLVFLLITIFNLFLHICLYCALGEFLVAQCEEIYS

AAYTNKWYSMDPRTVQDLLFLLIRGSKPIYLTAGKMFPMTMTTFCSLVKTSVGYLSVLHTTK

>7_399_Si_gnF.scaffold02694_2287513-2447739.pep

NKYYERDIGNAFAMHRFFFRMIGLWPFAHANSEQLETIVVVFVCFACLIVEAVPTLLYVFMVLTDIRVKLKVMGSAMFTT

VEIMKYVYMLFYKSQMRNCLILVDEDWQNVVSPSDRTSMIEKVKICKRLVVLCAILYSLNIIVRIVIPLSVGKIVTPQNI

TIRPMPHVAYLVLDVQQSPVYEITYIMQLLGGFFKYTIVVTTFSFVTLCAMHFCSQSNILITLINDFVNESRPENVNESR

PENLNKKLSIVVEHQIRIKNFLQLVQSVTQYPSLVEVLGSTVMLCFVGYCIITEEDQNIRLCVYSLILVMFVFNVFIYCY

MGEQIIEHVXQVALTACTLEWYRLPDTQARALILLIIVSETPFKLKAGSFIDLSLRTFGNVVKMAVTYLNFIRKM

>8_385_Si_gnF.scaffold00330_12820-264637.pep

LITRLFWIVTTAFVEYCHYLYFSTHLNSENFFNLVDCFCSFLAHAKVITKLVAFWVNQRKFEETLALITDDWSDYAKNDI

GMRVMTGKAKVSDRITYIILILHTMTIVLYSMGIIIADADVTETIELPFINKLVLPFSINTQHMYRFVLIAEFIHMMLSN

FVAGVYNAILLAMVFHTGGQIDILQCWLAQLQPKNIENKQKSIVVMANKIVLKHQKIIEFSENIESLYTYLAMLLFALNT

LLICTIAFIIVTAADAMEQIIKCILFFTITNLEAFVFCYAGEYLSNKSREVGFATYNCEWYNLKSKDSQILLFIILRSQK

QLTLTAGKIMDLTLQSFASIMNASGSYLSVILAM

>8_387_Si_gnF.scaffold00330_12820-264637.pep

GLRFIGMWPHCVYANVNWWTYIVSVAIVQYFQYSYVFAHFDVSKLSVLVDGLSITLGYSLSFLKLINLWFNRRKLYVILD

TMDKDWSDGIAIQSDVSTMIRYANLSRQCSNVMITTNALSVFFYVIGGPILRSMIQKNDRESSTRELPIKMEFPFKIDHS

PIFELVLVAQFFHDLSVACIIAMLNALLVTLVLHVSGQIDIMRQSFVEIASKKHLKDLINRHQKIIELSDNIEDLFSNIA

LLQFIWNTLVICCIGFVIVISEEGATMITKSLIFYVAITLEAFVFCYAGEYLSAKSKSIGDAAYECLWYDLTPSECRVLL

FLMLRSQKRLTITAGKITDLSLEGFTTVMKASASYMSVLHAM

>9_386_Si_gnF.scaffold00330_12820-264637.pep

IGLRFVGIWPGLPYGTITWSAYMTSLVFALYFQYVYIFGHFDVYNISNLMDALSITLSYSLGFLKLASLWSNRRIFYDIL

LAMEEDWSNVDIYDKRSCIMASNANLSRRCSNVLISINATAALSYSMTSLLRSADFQEDLNVSSRELPIKMEFPFEVDAS

PLFELLAVGQVLHVVSIAALVAMMNCLIITLVLHVSGQIDILRRELLTMCDNGTSQCDSIVKLLIIRHQRIITLSDNIEE

LYSDIALMQFLSNTIVICCIGFTIIGSIGNEVVMLKSVIFYVAVTLEAFIFCFAGEYLSAKSKSIGDAVYGALWYNMTPA

ECRILSFVILRSQKRLTITAGNIMDLTLEGFTSIVKASASYVSVLHAM

>317_Si_gnF.scaffold00330_12820-264637.pep

VFHKVLKDMDNDWRECINVDQHLYMMTIKANISHFFSNVLLSFNVIVATLYLLGDYVIRFVFLTANDNDTVRQLPVKIQF

PFDMQQSPIFELTFVTVFIHTMLQLWTIAIINGLIFTLVFHVSGQIDVICYEFTNISKNTLLHKSSGMLIKRHNRIISFS

KNIETLFSLIALMQVIWNTLVICCLGLVIIVSVHNNTVLVKTGVAYIAMTIETFVICFAGEYLSLKSKSIADATYKSLWY

DMPLRQSKIILFIIMRSQKRLGIHAGKMLDMSFQTFTNV

>339_Si_gnF.scaffold01629_62255-192132.pep

SEMEMYYATNKLFLYRIGVWPYQRRVLKVMIPCFIVIVHISVVISEALLLYDTWGDINVAVDCIVNLILLFTADVKLINM

VVNNRKFRRLLELMNKHWELLNNKIESHILKYYASISQKLTSYYAVYVIVTLTLYLSIPLAPRVLDVVIPLNESRPLIYV

YPAEYRVDKEKYYYPILFHSCLACSITGIILFTVDTTYIVCVLHACSLFTAVRYEYNGFECNLQLINYIHTGEVIRYIFI

LFGTFLHLVCMCIPGQLLINRSAEIFDKAYSSEWYTFSKETRKLLTILLYRSLVPCKLTAGKIFVMSMTMISTV

>346_Si_gnF.scaffold04171_663867-963062.pep

RYYKINRIVLKMLGLWPYQQSYFKQIHQVLFASILLTFILVQLLKLFTMEFNLEFVLNDLSFAIPSIVYLLKYSTFYIQS

QKIKKLMEHIRIDWNALQDEEEIEIIRKNAKIARRYMYAFVVFSSIGLIVFYFMELWPIILDNILPLNESRPRNTFVVTE

YFIDREKYFIPIMLHEMVVVSVGALTIISTGTILMACTQHSCGMLKIVRKIVRFAESILSSFSTFYFIMIGVGISSLTIN

MFQVFIATHNMNELFTTFIFLFAQIVYMYVGNYGGQIITDHYVEVFNTTYNSYWYTVPLRSQKLLLFIMQRTSKNFAFVL

GLFVVSLKGFSTLASMAMSYFTVIYSI

>348_Si_gnF.scaffold04595_2107-23737.pep

MAEDWMAIKLDTERNVMIKQARIARSIMIIGYIFVALACLSLILPSYFGIEVIDTMTNRNKPLPLQTYHYDTDKSPQFEL

TLLIHTLTILFGGIIYLCLDNSLILITFHIRGQLENFRCRLVRLKNFNKVLNNIIITHLRLIRFANNIENIYSLIILISI

LNFSVVFCLCGFLITIVETALAQVYLSTTILLCLLINTFLYCGAGQLIIEQCNKVHYAVCDLEWYKLEARKARNIILLMM

QTSHPFCMTAGNIIPLTMATFVNV

>350_Si_gnF.scaffold02944_1-38457.pep

KKIFSVVRLSLIPVWGWPIRNTKFDMFCVKLQHCFSIIMTIGLVLPLVYGITDNFNNISDLVTQIVVLGGLIHSISNFIF

HMINYQILQKLTFEMVDFCDLMESHEEVIIQRYIDKCVLFHSLSMSIFYFMMFTALLVPSLTDQPFPILTKCPFDTLQQP

LRTIIYLQQTIVGLFVTGALCMNIYMALLIWYATARFEILAEELRKAINELYECIKKHQKLIEYANEITVVARPIAFSTV

CCCMIGVIISFCLVHQPIGIIFQFTTASMTGISEVFMYTWPAEYLIHMCKEVGEAAFYLLENNDLLQIWKCLQIIIIRSQ

KPITISIPCFMPLSLNYFAS

>350_Si_gnF.scaffold04648_643308-783672.pep

MKFTLTVLAVAGCWRPTSWTSLFRYSMYNAYTALIILILYTFATMELIVNADSDTFGDAFFNVVISLLACYKAIILRRNH

DGITILIDSLVKKPFKPMDMSENMIRQKFDKRITNNTLCYLILVFITAVYMIILSLFTDFKNGILMYKAWLPFDCSISVL

FYFAYAHQILSLICIGLVHPTCDNLICGLLLHICCQLEILEYRLSNIANEQKNQKNLRDCVDHHIHILQYVYTVNNMFAK

IIAIQFAVSMLVVCSNLYRIAMATDYMSFIPLMMYTSAILVQIFILCWFGNEVKLKSLQLVNSIFDIEWPALSNSNKRNL

LLIMKRAMTPVEFTSAYIITMNLDSFV

>360_Si_gnF.scaffold04648_643308-783672.pep

GFTFKILSSCGCWIPDSWTSPHRRLMYHVYTVFILLLINTFTLLDIILTVNNPDDFTDNFYMLLAMIVSCFKMFSLLINR

SNIATLTDILINGPCRACDPVEVEIQQRYDKLIETNTLYYMILVELTCASTAVASLLTDYRKEKLTFRAWLPFDYYSSTL

FHFTYFHQLISLTVGSVLHVACDGLICGLLLHICCQLEILSCRLKNIVHNILRNCVIQHNLLFKFAFLLNKKFRFTITFQ

FIVSTLVVCFTLYQLTKTSGKFVELGMYMSCMLTQIFLYCWYGNEVKLKSLQLVNDLFEIEWFTLEQNTRKDLLTIALRG

KIPIEFSSAYVIPMNLDSFVGV

>365_Si_gnF.scaffold00330_12820-264637.pep

IGIWPGASYGTLCWLFYMTTLVVIQYLQYYVYAHLDFNNLTKLMDGLGLTLDYTLTIFKLISLWFNRRIFADILIAMDDD

WKECGTEFHECIMVDKANLAYRCSNVLXYPSTLSLLFYILLKATRVNVSYXEGQLRKFPIQAQFPFDVQETPIYEFVDVV

LFFHVLETATVIAMLNALILTLVLHVSGQIDIMCQELKEIRSTSKSNSISSLVARHQKIISLSKNIENYFSFVALLQFVW

NTFVICAIGFMVVISTSKSGILIQFIIPFCAVTMEAFVFCFAGEYLRTKSKAVGDAAYEAVWYDLPTSECRILLFVILRS

QKQLTITAGKVMDLTLEDFTSIVKASASYVSVLHAM

>365_Si_gnF.scaffold00330_12820-264637.pep3

VLIFQYHYLLIHFSTEELPNLIDGLSTTLPYNLLFIKMIVLWVNNRIFNDVLKAMSNDWREYSGMYAMIDKAVLAHRCSK

LTIGVYSTAVLLYSTASINFRKQSNDSCRELLIKMELPFNFCESPVYEIVMWVQFVHLMAVASSIGMLDGLMVTLMLHIG

GQIDLMRQEVEEICPNDDRSLINKHQKIIAFTENIESLFSHIALMQFFSNTIIICCIGFLIVTSMGTDEMLIKTMFFYIA

ITLEAFIFCFAGEYLSNKSKTIGDAVYESVWYNLKPRDCRVLLFVIMRSQKRLTITAGKFMELSLQGFTNSMKASASYVS

VLYAM

>365_Si_gnF.scaffold00330_12820-264637.pep4

MSNDWREYSSMCVMIDKAVLAHRCSKLTISVYSIAVLLYSIASVNFRRQADDVCRELLIKMKFPFEFCKSPIYEIVVCMQ

FVDLMAISSAIGVLDALMVTLMLHIGGQIDLIRQEVEGISFQNNRSLITRHHKIITFSENIESLFSHIALMQFISNTMII

CCIGFLIVTSLGTDEMLVKTVFFYIAITMEAFIFCFAGEYLSNKSKTIGDAAYESIWYNLKPQHCRVLLFMIMRSQKRLT

ITAGKFMDLSLEGFTNSLKASASYISVLYAM

>366_Si_gnF.scaffold04648_643308-783672.pep

VMQFPLKILTVVGCRPPISWSSKRMVYNVYTILIFLPLFSFMLPQFMDIILNVDNADDFMDTFYVLVALLIACCKMFSLL

LNRKNIEMLIEALLEKPFRPLEPDEIEIREKYNNIVWTNSIIYTVLIELTCGSMNLTSLLTDFRRGKLAYREWIPYKWSD

TVYYFTYFRQIISLTVASIVNVACDILICGLLLHIYCQIEILECRLKKSLRGDLGECVRLHDRIYKYARTMNEKFRLIIT

VQFIASMLVVCSSLYRLAKTTLSPKYIPLMLYTICMCIQILLYCWFGNEVKLKSIQFSDEIFGMDWVTADKKARQSLILI

MNRSLLPIEFSSAHIITVNLDSFVKFLKTSYSIYNLLTQV

>366_Si_gnF.scaffold05285_6032-497272.pep

LSLNFLLYTVCGIWRPVESNGAKLLYSVFTFIVVFSQYFLMLGQFMDIILVVDNIDDFATNVLMFLTMIAVCCKATVVIV

RRNAIFNLVQRLLKAPYKPQDEAEVAVQIKFDKFIRSCSIRYLLLASCSVTGLTLGSVLNAVHGRLPYRIWLPWDYNIPL

IFWILAIYQILTVVFAATINVGTETLVFGLFIQTCAQLEIFENRVKKFITNEIGISECVHHHLSIYKYAKMVNILFNQVL

FVQFFASILVLCSSVYYLSLHITEFSGIAFLLMYTICMFVQIFVYCWSGNEVILKSMSTGDAIYRMDWPLLSSNDRKGLL

MIMIRGTIPIKFSSSFLVILSLQSYSTV

>367_Si_gnF.scaffold01224_363367-449963.pep

YACGWNRYTMTILGIWPANRSLSQASSYIVLAPVLIMLCFICVPQSANLPYVWSDFDLLVDNLSTGVTITISMLKTIIFW

SNGGSLRTLLLYMSRDWNTIVDKRNRKTMLGIANFSRKLSIRSTVLVVTVVIAFVVSRFIEIRRSGRILFFNVRLPYNAT

ISPIYELTLVGQFGGSISAAVSYTAVDTFIATLVLHVCGQLSNLRYELTNLCANTNTKAEFQMKLGNIVRKHEHLNRFAE

TIEDCFNMMLLLQMLGCSVQLCFQCLQAFMQMYRHFGISHSSMTYVSLRIXLNLTFQSTQIAYAAYDCSWYNLSAREARS

LMNIMYRARSPLQISAGGFCCFNRELYSEILKRSVAYMSCILAM

>367_Si_gnF.scaffold02944_1-38457.pep

ISFLKVDLTFACCWPVPITKSQKIRDKLFRFLCCLNGILMSIFLIYTLSNKCDNMILIMKVGCELSAFLQIPVQITLFTF

QSDRLQIIICEMEDYIQQAKSEERNVFQQYINKCKLFYGTTLCWITTTATIMIFGPLVLSQPFPIEVEYPFAVDKQPLKT

IIYLHHIMVVYQSCAQVCSNVFVALLLWFVAARFEILSHKFQKITSISEFEFINCVQLHQRLLRYAKEVTMSIRYIALST

IGFSTIAVVFSGLTFLSRFPPSVKIQYIIICSTSLTKVLLCAWPADHLMRTSSNIAEAAYNSLWYNQSIKTRKIMLYTLL

RCQQAVIISVPGLLKLSFQHYASYVTTAFSYLTTFR

>367_Si_gnF.scaffold04648_643308-783672.pep

ALTFKIMAICGCARLDSTSYKRLVYHVYTIFVMLLIHTFMLSQLVDLIMIVDNSDDFTDNFYVLLAMIVSCCKMFALLVN

RSNIKMLIEILTSKPFKPVEPDELKIRQKFEKLIQSNTLHYTILVETTCLSVAVTSLLTEYRKGNLTFRGWLPFDYTSPQ

LFPLVYAHQLISFTMGSVHHVACDSLICGFLVHICCQIEILEYRLRKSALRECVLHHNHIFKFASIVNEKFRLTIFIQFV

VSTLVMCFNLYQFTKSTALKTKYMQLILYTCSMLSQIFFYCWYGNEVKLRSRQLINNVFEMEWFKFNENGQKALLMIVRR

AAVPIEFTSASVISMNLDSFVGLLKTSYSAYNILKQ

>367_Si_gnF.scaffold05285_6032-497272.pep

AKKMLSLAGLWPYQEKRTRLFRVSLMTVITLSIMIPQLGKFIQCDGDVRCIVIVVPAILFIVVVFVKLYSCQFNSSKIKS

SIDHLFNDWEKLKGAEEYKIMKAHAARARLLSLIYCSFIYVSTALFSSMSLVPRILDVVFPLNTSRRIILPYPAYYFVDD

DQYFYYIYLHMLVASTVAMTGLIAHDSMFFVYIEHICGLFAVVGFCLKTVSRNDRNDRNNVSNNSKNGDKIYNQKTAISI

HIHWRAIWFAEHLEETFSISFVVQMMIVVVVMSITLLQIALQFEDVLETIKSISFIVGQLMHLFCYSMQGQKLMDHSIQL

REEIYNSFWYEIPVKSQRLLLYVMQRSLEPNFLSAGKIFVFSLKSFTTV

>368_Si_gnF.scaffold04648_643308-783672.pep

LDVTFRILMICGCWVPDSWTTPYKRLVYHVYTIFIMLLIHTFMLMDLILTVDNANDFTDNFYMLLAMIVSCCKMFTLLIN

RSNIAMLIDILVRKPCKPVQSDEIEIQQKFDKHVQTNTLVYAFWVETTCLCIAVTSLLTEFRKGRLTFRAWLPFNYSSSL

LFRIVYAHQLISLTAGSVLHVACDGLICGLLVHICCQIEIIECRLRKVAHDQNILRESVLRESVLQHNHVFKFASIVNEK

FRLTIFIQFVVSTLVMCFNLYQFTKSTALKTKYMQLILYTCSMLSQIFFYCWYGNEVKLRSTQLLTNIFAMDWVTMDRSL

KRNLLLIMNRAVVPIEFTSAYVLSMNLDSFVGLLKTSYSAYNILKQV

>368_Si_gnF.scaffold04648_643308-783672.pep8

VLKPTFNLLMIFGCWRPHSSLHKRLAYDVYTSTIFLLVNTFMISQLVDVILTLSNAEDISDNFFTLIALCSACCKLLVLL

INRKSIIMLVDILMDKPCRPSESTEIKILYKFDKSIQINTWRFIHLGIVTVSIVILSSLFMNFRNKKLMYKAWVPFDYSS

AILFYITYTHQMIGMIAAIFLSIGCDTFICGLLFHICCQIEILTYRLKKIISYSSVVLRDCVYQHYHIFKFAVIVNAKFS

LTTTVQFVMSTMMICFSLYHLSKTTSKTKYLETILYMCCML

>368_Si_gnF.scaffold06723_481864-495862.pep

LPVNFYVLRFCGVWKERKDDNIRFISFCYRYAVVILIYEFTISEVIELVRTRDDIEDMTEGLFMALTYVALCFKYGNFLA

RKEEMSMLLDCFRRETCQPRNSEEKTILIKYDRKAKWCVRTFMSISQATCIALILAPIVGPQNTDRPLPFKTYLPYSISG

LYPYLATYLQHVGAIFYGVLLNVSFDSLVYGFTLHVCGQIELLCYRLSKLFKPSHYMISECVRHHLYVHEIVRRIQSLFV

WTVTVLFIFSLVTLCTSIFQMSKKKILSVGFLSLILYLGSMLFQVFFYCWYGNELQLKSKSIVDAIYSSDWTVASTRDRR

SLLFVMAISQKGLKLSYYGIFNLALDTFTWV

>369_Si_gnF.scaffold04648_643308-783672.pep

LMFTFKVLTICGCWPPNSWTSRCKRILYDIYTILIVLLINTFTLLDLILIVDNADDFAENFYITLAMFVSCCKMFSLLRN

RNNIAMLIDILMKKPCRPTEHDEIEIRQKFDKLVQTNTFYYATMVESTCAFALVTSVFKDYRKHRLAFRAWLPFNYSSPM

LFRIAYAHQAISLTVGSVLHVACDSIICGLLMHICSQLEILECHLKKVINKLRECVIQHTYIFQFALMVNEKFRFTITVQ

FLVSMLVVCFNLHQLTQTNVLSAKYIQIVLYMFCMLTQISFYCWYGNEVKLKSQQLVSNVFGMEWFTLDYHVQKNLLIIM

TRCIIPIEFTSAYVISMNLESFVSLLKTSYTVYNVLQQMR

>370_Si_gnF.scaffold00330_12820-264637.pep

RLVRFGLHVYGIRPYVTSTVVFRLYWIIMLSTAQVFQYRYVVMHMDDFSEYMDGVSSAMASSLLYIKLIILWTHERTLFD

ILQMIAVDWKNCVLTDCSLRITTNKAKLSHRFSNWIIGXQLTAIVLYSCGVLAVNAGNVQRMNVXARKHILKMKLPFQID

TSPIYMLVTILEFLHLXMGGCGISMVNSLIVTLILHIGGQIDIHRDWLLNAFFKMKALITKHQQIIMFXENIKNLYTYIA

LMLFVSDILIICCLGFIIVTSIGDDQMLVKSILFYVVINLEAFIFCYAGEYLSVKSKSIGDAAYESLWYDLSPSENRILL

FLIMRSQKQLTITVGKFMNLSLQQFANV

>371_Si_gnF.scaffold01629_62255-192132.pep

TRYLRTNKLLLSLLGLWPMESSFNRNIFYCASFDIILFILVPQFTYLFTRMRDLNDLYDSLPTFLGAHIVVFKLFGLRWQ

TEKLKMLVQHVRYDWCSLMKDSDIWILMEYSEKSRIFTLAYLIFTSVSITSYVTAPITIRLFDIMLGSNVTRPKRLPHPS

EFFLDLEKYYYIILTIIFVGYIAAIITVNATDAIYFALMQHTCGILAILSYRLKNLIVHDKSENIVQCIQLQARTERLIQ

LIESTFAICFFSDIALGIPFYCSAXNIDRILKPIAYLNGALFNVFFENWQGQKVIDSSEKVFDSAYNLKWYKMPIVSQKL

LIMIMMRSKKPLTITAGKILVLSHMTFNTV

>371_Si_gnF.scaffold06792_1626879-1704318.pep

WATRLNRILLNIVGIWPNAHKNADKFFSNVRAAFTAIVLLFATIPGIHSLVRTWGDLMSMIDNLQFTLPITMTIIKLIDI

WWKKTDLLMAINMIAEDWIKDKTNKERCIMIKQAQNARIITMVAYSFMFVGSIFVIILPCFGKSIRYITNVTDPVKILPL

QAHYIYDKNQSPYFEITFVAQSFVILICIASYTGVDNLLGLLVFHLCGQLEILKEKLIKIKNYNEGIALIVEEHIRLIKC

FRIIESTYTLLLLGLLMYFGIIFCLYGFLILAIHMSLMRFMYLVSVAINICGHMCLFCAVGEMLISKCDDLYRAAYEHKW

YKLDPKKAKILVLIMIRANKPLYITAGKMFPMTMSMFCN

>372_Si_gnF.scaffold01629_62255-192132.pep

TRYLRTNKLLLSLLGLWPMESSFNRNIFYCASFDIILFILVPQFTYLFTRMRDLNDLYDSLPTFLGAHIVVFKLFGLRWQ

TEKLKMLVQHVRYDWCSLMKDSDIWILMEYSEKSRIFTLAYLIFTSVSITSYVTAPITIRLFDIMLGSNVTRPKRLPHPS

EFFLDLEKYYYIILTIIFVGYIAAIITVNATDAIYFALMQHTCGILAILSYRLKNLIVHDKSENIVQCIQLQARTERLIQ

LIESTFAICFFSDIALGIPFYCSACVMVMTDTENIGRLMPYGSYLSGLVLNTFFENWQGQKIIDCNEKVFNAAYNLKWYK

MPIVSQKLLIMIMMRSKKPLTITAGKILVLS

>372_Si_gnF.scaffold05285_6032-497272.pep

HYSSIAKKMLSLAGLWPYQEKRTRLFRVSLMTVITLSIMIPQIAYQFTCKTNLQCIFEAMTSYLLTVVAFVKVYTFQFNT

HKIKGLTQHLFVDWKRLETPKEYEIMKLYAKNSRRFCMVYAVYYTVATFTFMSMTFIPFVFDVVWPLNESRPVLPPYPGY

YFVDNREYFFKIYCHSLISWEIIMVGIVAHDCMFVTYVEHVCSKFALVGFHFENLFCNDEEIKITDNSDDTYRKRIKLFV

HEHWEALKFAETLEDTFVVPFAVQILIVTVGISVTLLQEGEVLESIRYVVYVIGQLIHLFFLSFEGQKLIDHSLQTGDKI

CNSAWYEVSVRSQRLIMLVMMKSTRPSFLSAGKIYVFSLESFTTVMR

>373_Si_gnF.scaffold00330_12820-264637.pep

SPLKIGLQLLGLWPGISYSIIYWSSFMLSMIVMQYFQYLYIFNHIKELLNLVDSMPAALDYSLTIFKLISLWLHRRVLHE

ILTAMDNDWRECINVDWQLYVMQVKANISHICCNAILSFNAIATLLYFLGNYIIHIMFLTEDYNDTLRQLPFKTQLPYET

QQSPLFEFVFAILLLHVILHSSTVGIVNGLIFTLVLHVGGQIDIICQKFKNTSENTLLSKTSVPMLIERHNRIISASDNI

EKLFSFIALMQVLWNTLVICSLGFAFTFFNGANFALVKTIFAYFGVIMEAFVICFAGEYLSHKGTSITNATYETLWYDMP

PNQCKIIMFIMMRSQKQLAISAGKMLDMSFETFTSVI

>373_Si_gnF.scaffold00330_12820-264637.pep7

LLMYMTLIVTVQYYQYYVVAHFDLNNIPLLMDCLGLTLAHSLCLLKLSTLWWNRRTFYYIVKTMDEDWKERNVNDFYAST

MVGMANLSRRCSIAMISFNAFGAFFFTIGEHMLHSLVANKGDHKPRVLPLKMQVPFDVSKSPIFEYFLLGQFLYEVVLAS

VVAMINSLLVSLILHVSGQIDIMRQEINEISHGKYDYDSSTFLMDIKGLICKHQKIITLSKKIESLFTYIALMQLLWNTL

IICCSGFMLILGLSNNKVLIKTAFLYFAKALEVFVFCYAGEFLSYKNKSICDAVYESLWYNLMPSNCRVLLVIMIRSQKQ

LTITAGKILDLTLDGFMSV

>373_Si_gnF.scaffold05285_6032-497272.pep

LTLNFLMYTFGGIWRPIESNAANSLYNIFTCIVLIMEYFLVITQFLDILLVVDNVDDFVINSLMFMSIINVVSKATVVVV

RRNAIINLVQRLLKGLCKPQDEDEITIQTKFDQFIRSWSIKYIILASSSLTGVTIGSILNVMHGQLPYRAWLPWDYNNVP

VFWVISIHQIIALIFATFINVGTETLVFGLFIQTCAQFEIFENRLHKLISSSNKDKTEISKCVCHHLSIYKYAKSVNVIF

NQILFIQFSCSILILCTSVYYLSIHIAELSGVASLIVYTICMFVQIYIYCWSGNEVILKSMNTGNAIYHMDWPLLSVSEK

KELLLIMKRSTIPIKFTSSFLITLSLQSYSNVSN

>373_Si_gnF.scaffold06792_1626879-1704318.pep8

IFIKMFFNYNILFIIFHLEDLLMAINMIAEDWIKDKTSKERCIMIKQARNVRIITIVCCFFMFLASSLVVILPCFGMTVR

YITNVTDPFKILPLQTHYIYDKNQSPYFEITFVAQFLVALMCVTSYTGVDNLLGLLIFHLCGQMDILKEKLINIKQFKNY

KQFKNYNDGVALIVKEHIRLIKCFCIIESTYTLLLLGQLIYFGILFCLYGFLILVVKHMSMRFMYLVSVAINICGHMCLF

CAVGEMLLSKCDDLYRAAYEHKWYKLDPKKAKILVLIMIRANKPLYITAGKMFPMTMSMFCNLIKTSAGYVSILLAM

>374_Si_gnF.scaffold02944_1-38457.pep

LDQVITFLKIDLLFACCWPLPRTKGQIIRDKIFRYLSIVNGAFIVIELIYSINNHLDNVSLIMQLACALGIFCEIPLQIF

LFTQQHDRLQNVICEMEDYYKQANAEEKDIFQQYINKYIFLYGTTLSLTAASLAGSLIVPLIQSRMFPLEIEYPFHVDYQ

PMKTIIYFHQALGMYQVYCQVSANVFLALLLWFTTARFEILANKFRIITKYSDWITCIQEHQETLRYAKEMSNSIAHVIL

ASLGISTAALVFGGVTFLSRFPPSVKIQYIIICSTSLTKVLLCAWPADHLMRTSSNIAEAAYNSLWYNQSIKTRKIMLYT

LLRCQQAVIISVPGLLKLSFQHYASYVTTAFSYLTTFR

>374_Si_gnF.scaffold03663_75123-126571.pep

RNYRINKVLLSCVGQWPYQTNRSSNAIIIIMVSLAGTQFIAKICGLFSIDNADVFIDSFSPLIVDLACGVKLITCILKAT

EIKALFNQIQSDWQLLTTPSYIKVLNNYAQNGRTFTIIYASVFYSALILFMLIPLQPLLLGPSSNDTSRPALLHQVEYYI

DMEKYYFPILIHGYITAVICVSIAIAADTMYVIVVQHVCGLFMIIGQQLENAIKEDNTDDKPYGNIISSIRAHKRALRFA

SLIEATFSPMFLVVAGFNMVIISMTGVTAVSNMDKPEEFFRQITFSCALLVHLFFESFQAHRLIDHSTYIHTSLINLAWY

ETSFRTRKLLIFMLMKTREPCVLTAGKMFVISMDTFSTV

>376_Si_gnF.scaffold00330_12820-264637.pep8

WPDSAYATLYWLIYMATMVIVQYYQYAYVVRHFDDIPLLMDCLGLTLAYTLAFLKLFALWWNRRTFYYIVKAMDEDWKEC

NVNDSYASTMVGMADLSRRCSNVMISINALAAFFLSIGEHMLQSMGDANKVDNNPRELPIKMEFPFDVSESPIFECFLIG

QFLYELVLASIVGMMNALLVSLILHVSGQIDIMRQDINEISYGKYIKGLICKHQKIITLSENIESLYTYIALMQLLWNTL

VICCTGFVIIITNDSGTTSIKSVSFYMAITLEVFILCFAGEFLSAKSRSISDAVYESLWYDMPPTNSRILLFVILRSQKR

LTITAGKVVDLTLEGFTSIMKASASYVSVLNAM

>376_Si_gnF.scaffold00330_12820-264637.pep9

WPDSAYPNLYWFSYVTTVAIVQYYQYTYIFVHFDSNNLWLLMDCLSLSLAYSLAFLKLLVLWWNRRIFHYIVKMIDQDWN

EYIINDLHRSIMTSMAGLSRRFANITFSFYAFSAFFLTIGEHLIQSMDDGNQFSNNSRELPIKMEFPFDVSKSPIFECLL

IGQFLYDMVIAFVVGLINALLVASILHVTGQIEIMQQDLIEISNGKIKSLICKHQKIITLSENIETLFTHIALMQVLWNT

LVMCCTGFVIVVIGEDTTNLIKSVSYYIAIIMEVFVYCFAGEFLSAKSKSIGDAVYESLWYNLPPSDSRIILFMMLRCQK

RLTITAGRVFDLTLEGFTSVMKASASYMSVLHAM

>376_Si_gnF.scaffold02648_1900428-1919549.pep

EKITLKKAFATVKLSLFVIWFWPLPLSKRKMLCMKLYQYVCILLTTTVLMSMIYAVVKNFNDLDLFIKSSLGLFPCSHVI

SNILCHLTTYKRLQYVTVEMEKFYTLIKPREEMIVQREYLDKCSKFYGFCIGLFYMSLVGLFVGPIVLDEPLPAPAEFPF

DASQQPLRAITYMHQIVVGMYIASHLCVNAFMALLLWLVSARFKLLTEELRTITNDFAKCIEKHQQLLKYAGEVSLTVRP

FALVTIFFSTVSLIVFGLIFIADVSSSLKIQCVLLSTSALIEVFMYAWPAEHLIHISTNIGQTAFEINWYDESEYFRKNI

QIIILRSQKPILVVLCGLPSLSLRYYASYLSTIFSYFTTMRIM

>376_Si_gnF.scaffold04510_616038-792405.pep15

FRMLFNITLIIFVLTVPTLAQLIKTWGDMIQIIDNLQFIIFNLIAAFKIFIMWYKKEVLSLLINIIINDWMRVKINKERN

VMLKNARITRLLIKDRIFFMLSATSIRMSPAIFKQYFGHMKNLTNLEKSLPIPAHYWYDVSSSPIYELTYLVQTIGSFAC

ALTYSAIDNFLGLLILHVCGQMESLHLRLLNLGKDDFRAVLKYNIKDYLISYRSIEAIDDTFNLLMLGMIGFFNIVTSIL

GFLIINTTHLSITIAWYFSAIVNVLLLSGFYCAIGELLVIQCEKIYNATYECVWYTLSPKTAKDLTLIMLCAKKPFNLTA

GKIIPMTMSTFYSLLKTSMGYISMLFA

>376_Si_gnF.scaffold04648_643308-783672.pep

LQFTLKLCTVSGCWQPLRTMSARIIYDSYRVLLICLISAFTMSQFINIALNIDNFNEISDNIYMMLTVFIATYKLISMWI

SKKHVTTIINIFTEKPFKPLESSEVMIRQKYEKTIRQYAFWYYGLVQITVICIIINAFAMDFMTGNLTYKAWVPFDYRPS

VIFFFVFIHQLIGMIIAAAVNVACDSLVSGLLQEICCQLEILEYRLTKIFHDQNVLLHDCVRHHNRIYECAYMVNGKFAK

IIAIQFAVSMLVVCANLYKLASISLAMIGLLTLILYTCCMLSQIFLYCWFGNELKLKSIGLANSIYNMKWADLENKNKKD

LLLIMRRSMVPIEFSSAVIITLNLDSFGSLLKASYSAYNVLK

>376_Si_gnF.scaffold05285_6032-497272.pep

KNYYDIVCKISSLTGLWPYLKPRARIFRVGLLTVTMLTIFIPQIAYQFTCKTNLQCIFEAMTSYLLTVVAFVKVYTFQFN

THKIKGLTQHLFVDWKRLETPKEYEIMKLYAKNSRRFCMVYAVATFTFMSMTFIPFVFDVVWPLNESRPVLPPYPGYYFV

DNREYFFKIYCHSLISWEIIMVGIVAHDCMFVTYVEHVCSKFALVGFHFENLFCNDEEIKITDNSDDTYRKRIKLFVHEH

WEALKFAETLEDTFVVPFAVQILIVTVGISVTLLQITQQEGEVLESIRYVVYVIGQLIHLFFLSFEGQKLIDHSLQTGDK

ICNSAWYEVSVRSQRLIMLVMMKSTRPSFLSAGKIYVFSLESFTTVMR

>376_Si_gnF.scaffold05901_12517-961344.pep

TLDKVIVFLKVYLTFACCWPLPSTKSQRLVRSAFQCICLTNSIVFVIAAIWTLCKYSDNALMVMKLGCQLSAIVQIPLQM

ILFAMQNKRLQFIVLEMENYYQQAQEYEKKIFQLYVDKCKPFYGSILCWLAMTGISVIFTPLFSSQSFPSEAEYPFDMES

QPLKTIIYAHHILIAYQSVIQVSTNTFPALLLWFVAARFDILSVQFRTMTNIKELMKYTHKELMKYTHEHRLLLRYAREV

TRAIRYVALLCVTFSTGAVIFGYLTFMSHAPLSVKSTFLMIAFCGFVELYMYAWPADNVMSTSSDIASAVYESLWYNNDI

KTRKILIYIILRSQRPVTVSICALPNLSMNYYASYISTVFSYMAFIRA

>378_Si_gnF.scaffold02944_1-38457.pep

LEKIISFLKVDLTFACCWPVPITKSQKIRDKLFRFLCCLNGILMSIFLIYTLSNKCDNMILIMKVGCELSAFLQIPVQIT

LFTFQSDRLQIIICEMEDYIQQAKSEERNVFQQYINKCKLFYGTTLCWITTTATIMIFGPLVLSQPFPIEVEYPFAVDKQ

PLKTIIYLHHIMVVYQSCAQVCSNVFVALLLWFVAARFEILSHKFQKITSISEFINCVQLHQRLLRYAKEVTMSIRYIAL

STIGFSTIAVVFSGLTFLSRQPLTIKTQFFTVAASALIEVFVCAWPADYLLRTSNDIGHAGYKSSWYNQELSLQKNMLYI

VSRCQHPVTLTVPCMLPLSLNYYASYLSTTFSFLTTFRA

>379_Si_gnF.scaffold01629_62255-192132.pep

RYLRTNKLLLSLLGLWPMESSFNRNIFYCASFDIILFILVPQFTYLFTRMRDLNDLYDSLPTFLGAHIVVFKLFGLRWQT

EKMKVLYEDIEQTWKKKCTSAEKEILQRYAEKSRSFTIRYAIILYSSMILYMTLPVMPSIIASFMSANQTQIYGLLFHVE

AVLDTKKYYYYILLHSYYTTFFMITIPVAVDSMMIAYVQHACGLFQAIGYQLKNVKRYPLREDDEHYRTIIDSIVKHKEV

LQFVELLTSSYSISFLLLTILNMAVMAFSAVQAINNRDQPTEALRFLTTCISLSMHFLFLSLPGQKLIDHSSNMHESICA

TNWYAISLRARRLLNLMLVRCKVPCKMTAGKMRVMSLQSFSGV

>379_Si_gnF.scaffold02694_2287513-2447739.pep23

DYSLQFNRWILKSIGAWPEFRKNSKNILINILRLACHSLMVYTIVSSTLYVLFEEKDLRLRLKAIGPTSHIIMGGINYCS

LLHNNDRIRAFIEHMETDWRMAKREQDREVMLRNARIGRVIAAVCTLIMQVGVISYNIARGMSRITVVIGNKTIETGGLP

CASYNKVDTRLSPVYEVVLAVQCLSTIVVNNVTVGACSLAAVFAMHASGQLNVVMLRLEELVAEKQDLQDLQLKLANIVE

HHLRALRFLSHLEVMMRQICFVELVGCTFNLCMLGYYTISEQESINTIITYVMVLTSMLFNIFIFCFIGELVTDQCKKVG

KAAYMTNWYLLP

>379_Si_gnF.scaffold10542_230568-232960.pep

MRLLGFWPQDTRLLYDLWCGAIFMLISSFTFPITYTVVYAVTDWNSVMHQALEIIPTIPLLARFIFMKMMAKNFRLILCM

MTADWADYRYLTKRNRQIMIYAKRGRRFSILSIILMALALIGFILTPVINMWRNDSLWNVTTRILPHEGLYPFHNKKSPV

YEMLYVAQLLVMFLCAMALATVDCFLYVIVFHVCGQFDILATILKRYDCACLSCIVKRHVHILSFVDIIEKSFRDFLLLQ

LLGYWFSLVLQGRELILHRHSNVGIITCILYVLTIMYYIFIYCYVSECIIEKSEDIGRIAHDLEWQHFPRDSNLPIIAAR

TRIPCKLTVGRFLTLSFPCYNTVMKASASYMSVIHAM

>381_Si_gnF.scaffold00330_12820-264637.pep

LKLGLRMIGMWPDSSCKTFFWLFYMITIVVMQYFQYLYFFAQLGTNNFSKLMDGLSVTLDYTLTFLKLLSLWHNRRIFSD

ILSAMNDDWNDCSTDSHVYVMTSKANLAHRCSNVMLVLNTLSTFFYFIGSFLSHRTISKNGDLREFPMQVQFPFDAATDP

IFELIVLGLFLHVWETATVIALLNSLILTLVLHVSGQIDIMCQGLREISTTQKPLIRSLIVRHQRIISLSNNIDNFFSFV

ALIQFVWNTVVICSIGIMIMISEGKSGLLIQSIIPYIAVTLEAFVFCFAGEYLSTKSKSISDAAYDTIWYDLSISECRIL

LLIIIRSQKRLTITAGKVMDLTLEGFTTVMKASASYMSVLHAM

>383_Si_gnF.scaffold00330_12820-264637.pep

RLVRFGLHVYGIRPYVTSTVVFRLYWIIMLSTAQVFQYRYVVMHMDDFSEYMDGVSSAMASSLLYIKLIILWTHERIFSD

LLQMMSTDWQDYISTRHSSRIMTNAANLARRTSRWIVGMQVASGTFYSVGVLASNANNPEKLETRELILKMELPFNISTE

FIYTAVQSVQFYHLSLVCYGITIVNSLLVTLILHICGQIDILRECLLKVFSKNSAESMRSLIAKHQRIIIFAEHIETLYT

YIALMMLLSDTIIICCLGFIIVISLDSPNILVKSMLFYISMNVEAFIYCFSGEYLSAKSKMIGNAAYDSLWYDFPAKESR

TVLFLIVRSQKRLTITSGKIVDLSLERFTSVVKASLSYISVLLAM

>383_Si_gnF.scaffold00899_962711-1051965.pep3

STSITMSIEMIRHCNGHEDAMDAFLLSSSSLISMVKLLLHRVYWRQKLILVESVIHDWTYVKNSHSRDIMLKYARIGRLG

SSIFFYFGCASVVSFVSSVVLANVDLPWTSGKQTFNETYERKLMLAAYCIFGKDTSFAYCAIEALQFVQIVVNGISQCGN

DGFFFDLTMHMCGQFAILRMNFTKLGCEDFSYRSKLNILLKRHYQLICLSHYLERAFTMIILAQVLMSMIVICVEGFLLL

LSMNDALTAAKHSVFILSLCMQLFLYCFAGQTLEFQSKELACAIYESPWYTFDVSMMKTLPLIILRTAHPQQLTAGKFVA

INFMTFKEILKASASYLSVLRVM

>383_Si_gnF.scaffold01573_601649-632525.pep1

PFKLLTLPLGVWPLQKYNTFSLVRSIVCGVSMTAMMIMLFLEINFGSSDAYVKLDDLMLMSCNILCVLKLLSYRLYADNL

IRNYSSAVKDYLAIDDEWKRIIMRRHAYMGRIICYICILSTYGCSLIWTVMPMLAADGEDIQINVTIENQASELPVPVTF

LGDVHIPGVYFVISSMESFILLLTGTSNCGNDALFFAIVLHVCGQMELLKIEFTKYGKTNKNENENTNKNENENFSLLGS

RHRYLMEHAKLLTDVISFVLLVQVLFSCLIISLIGFQFILAVNDAVMIIKSVSVLASFLFQLFFYSFVGDYLKCQMEDIA

DSIYSSNWYCLSTKLMRNVLFVTMRSQQPVQLLAGKFFIINIRTYMTILKSSLSYLSVLRVM

>383_Si_gnF.scaffold01629_62255-192132.pep

TRYLRTNKLLLSLLGLWPMESSFNRNIFYCASFDIILFILVPQFTYLFTRMRDLNDLYDSLPTFLGAHIVVFKLFGLRWQ

TEKLKMLVQHVRYDWCSLMKDSDIWILMEYSEKSRIFTLAYLIFTSVSITSYVTAPITIRLFDIMLGSNVTRPKRLPHPS

EFFLDLEKYYYIILTIIFVGYIAAIITVNATDAIYFALMQHTCGILAILSYRLKNLIVHDKSENIVQCIQLQARTERLIQ

LIESTFAICFFSDIALGIPFYCSACVMAINNRDQPTEALRFLTTCISLSMHFLFLSLPGQKLIDHSSNMHESIYNLKWYK

MPIVSQKLLIMIMMRSKKPLTITAGKILVLSHMTFNTV

>383_Si_gnF.scaffold04171_663867-963062.pep

DHYHKLNQIFLRILGLWPYGKTKYDHFRATCFFIILVSHTFVQLAQLLIADFSIIVIIRILSEFLPILMCLVKFNMFFLE

TKKILKEMTNNRRILTEAQEIEIIEHYANQGRIFTIIYTCNMTFILYILATIELFPSILDCFWPLNESRTHNMLFLTQYH

ISEGIQYYSCFLYFSISVSIGCFSVICITTMFTVAMLHCCAIFKICSYRIKQSVDEKCSNKRNVIVKRIIRTIELHQNAK

RLFKLCIPNFGIYFFIMVVVGTCSCVANLYRLLYSMDNFPEIMLALGYIITHEIYLVATTYMGQNLVNHADELFNAIYMS

LWYKAPVTIQKLLLFMMQVASKSVVPKVGGIYCSVMESFTTV

>383_Si_gnF.scaffold05118_1422230-1821122.pep

IVVWNKWFLTFLGLWPERVNQLVFIFFTTYMVIYCTMGMNHLIRHSDQPELVIANFTDNVFLTMTLGKMLICRRSSRIMA

TFLKSIEPDFTTRMYDQEKQAYLQYNKLALIFVRLSMPMLAFTSTLYYLRLFYEKWSIMISGNFSYETLPYPVHPFFEIK

DTATYVCVCIYLAIMLPIILCGYGGLDAFVLSMALHICGQFAALSYKINNLLKDHKKDHKNYHRHITNIVLRHRHLIKLA

EILENSFNMICLQQTLGTLVLLCLTMFHMLATYGDNANVVAFTLYAVCVSSTILAYCYTGECLFTESAGLSDAFYNTDWY

NNSPSSTKLVGICMIRSDRPLILTAGKFCILSLNTFTSIVKTSMAYLSMLRNF

>384_Si_gnF.scaffold02814_302873-346762.pep

IEREYSINKIFLSCLGVWPFQSKLARSLIPICYIVYETSYYSIEILMLYDHWKDARIVFDCCYQIVLLTTFVSKLLNEFL

NHDKVQRLIETMDNHWNIFTSESELRTLKDYTNISRKFTIFYSIMMYVALTIYLMPPFIPILFDLIWPLNESRPRVFSLS

IKWRIDMDKYYVPIVCYNSITIMTGIIIMIGVDSIYISRTFHACSLFSIVSEQIENITSTQHEEHELSRKEQVLYQQYVM

CVKKYQIALKFVDILNSTHQTVAVFFLLLICATLSLIGVRIVYVLGQLEEMIRFTFIIVGALLQLLIMCYSGQKLIDESE

NIFHRAYAAEWYNFSPRLKSLLIIILHKSIVPCKLTAGNLFPLSMAVFAAV

>384_Si_gnF.scaffold06723_481864-495862.pep

LPYNFRVLWFCGAWSEKNNNNVRFLSFCYRYAVVILIYEFTISEVIELVRTRDDIEDMTEGLFMALTYVALCFKYGNFLA

RKEEMSMLLDCFRRETCQPRNSEEKTILIKYDRKAKWCVRTFMSISQATCIALILAPIVGPQNTDRPLPFKTYLPYSISG

LYPYLATYLQHVGAIFYGVLLNVSFDSLVYGFTLHVCGQIELLCYRLSKLFKPSHYMISECVRHHLYVHEIVRRIQSLFV

WTVTVLFIFSLVTLCTSIFQMSKKKILSVGFLSLILYLGSMLFQVFFYCWYGNELQLKSKSIVDAIYSSDWTVASTRDRR

SLLFVMAISQKGLKLSYYGIFNLALDTFTWILKTSYSAFNVLQQT

>385_Si_gnF.scaffold00330_12820-264637.pep

QVMKIWLRMFGIWPNMSCVLFCRLFWIVALIVEQILQYRYVVNHFHEFSEIMSILGAIVAYTVFLIKLIIFWVKQRTFNK

ILMMMAIDWEKCSSTKFSMFTMTCNAKRSQRFVNMTAIFYAIAVTLFSSNVLVKHVDDGKPSNVSTRMFILQMDLPFDVN

RRFVYESVIIVQFLHLILCSEAIGLLNALLINLILHVGGQIDILCQNLMEMFPKMIKEIIEKHQKIITFSRYIEDLYSYI

ALVLFISDTLIICFLGFTIVTSSDGMESIMKNLAFYLNMNMEAFIFCFSGEYLSAKSKSIGDAAYDSLWYKSDSRDSRTV

QFLIMRSQNQLTITIGKIMNLSLDRFSSIVKASASYISFLLAM

>385_Si_gnF.scaffold01629_62255-192132.pep

SEMEMYYATNKLFLYRIGVWPYQRRVLKVMIPCFIVIVHISVVISEALLLYDTWGDINVAVDCIVNLILLFTADVKLINM

VVNNRKFRRLLELMNKHWELLNNKIESHILKYYASISQKLTSYYAVYLIVIIIFYLLIPLTPKILDFVVPLNESRPLAYI

YQGEYRVDKEKYYYPILFHSYLATACTMTILFTCDTTYIICVLHACSLFTAIGEQLENITSKAGTTSNNDGEIHTEMQYH

TFIKKNDYKILITCLKKHQLAVENAQTLNSMFLHVTFILLSMNMLVLSIIGIQLINNLENTKETIRYICLTGATFIHLVC

MCIPGQLLIDKSTEILDKTYGSEWYTFSNKTKKLLSVLLYKSLVPCTLTAGKMFVMSM

>385_Si_gnF.scaffold01968_226495-654737.pep

AIELSQLSMTFFGVWPENNTTQKTIMSNIRTFILNIIVWILAIPCMHSLIRIWGDIMSVIDNLQYSLPILMAIMKLVIMW

QKKKDILLLLNMIKDDWLKPKKIKERDVMKKRARLARIFTIFGYFMMLVSYILVVVLPIFGISMRYLTNKTDPDKLTPLQ

SYYIYDKNKSPFFEVSYIMQSLGLMVAGVTYSSVDSFFGLLVFHVCGQLENLKMRIIHLDKFKNKNFEIALSHSVQDHIR

LIRFINMIDDIFTLMLLSVLLYFGIVFACYGFLLGTIGMSMFRFIYLVSIVLNVSTHMCLFCAVGEILVTKCEALYQAAY

EHKWYTLEPAKAKNLLLIMIRANKSLYITAGKMFPMTMSMFCNVIKTSAGYVSILF

>385_Si_gnF.scaffold02694_2287513-2447739.pep8

HRFFFRMIGLWPFAHANSEQLETIVVVFVCFACLIVEAVPTLLYVFMVLTDIRVKLKVMGSAMFTTVEIMKYVYMLFYKS

QMRNCLILVDEDWQNVVSPSDRTSMIEKVKICKRLVVLCAILYSLNIIVRIVIPLSVGKIVTPQNITIRPMPHVAYLVLD

VQQSPVYEITYIMQLLGGFFKYTIVVTTFSFVTLCAMHFCSQSNILITLINDFVNESRPENNESRPENLNKKLSIVVEHQ

IRIKNFLQLVQSVTQYPSLVEVLGSTVMLCFVGYCIITEEDQNIRLCVYSLILVMFVFNVFIYCYMGEQIIEH

>385_Si_gnF.scaffold05118_1422230-1821122.pep

KGDHIEDLFVHLERIFSIGGIWPSKRTYVRFAIYISHYALYLVMAWINLYDVFGNLELMVMNIVETVAYSITFPLMCLIR

CSNLLKLVINVIRKDMVRKFENSEEERIYYNYNYISKVFTYGSVVGMFITVVSLYFRPLVYLLTTNQALRHNDTEPLVLP

YRVHPFLDTSNTHAYILMYLYLFPLIYISVCHMAAICLMVILVFHICGELSILSYRIKHVGEYSEDSEDLIVGRIGSFVR

MHLKIIWLMKSINDTFHLILLDELLGNSIVLAISLYYIIMNLDVTNTCFTFTFFAIIALVMLFGYCLMGDQLTQQCVNVQ

DAYYECNWYEMPPVCKKCLLICMIRSQVMLYLTAGRFYIFSFTSFTDIIRTSLAYLSMLRTL

>386_Si_gnF.scaffold00330_12820-264637.pep

SPLKIGLQLLGLWPGISYSIIYWSSFMLSMIVMQYFQYLYIFNHIKELLNLVDSMPAALDYSLTIFKLISLWLHRRVLHE

ILTAMDNDWRECINVDWQLYVMQVKANISHICCNAILSFNAIATLLYFLGNYIIHIMFLTEDYNDTLRQLPFKTQLPYET

QQSPLFEFVFAILLLHVILHSSTVGIVNGLIFTLVIHVSGQIDIICDEFKNISKSFGMLVERHNKIISFSDNIEQLFSFI

VLMQVVWNTLVICCLGFIFIISNGTGIVLVKTVSGYFVVMVEAFVICFAGEYLSLKSKSVGDAIYETLWYDMPTHQSKII

IFIIMRSQKRLAITAGKMVDMSFETFTSIVKASASYVSVLHAM

>386_Si_gnF.scaffold00899_962711-1051965.pep

MMRSMMRILDLWLLQRNNVVYTSLWFIIFTVEFLTLINVLIEYFKSCDTVKDGLKILRMIESAINSWLNIIFPXFTRKKI

TVNVNSAIEDWSSPSMKKESRLVMMAYARVGRLITSVASIQLTIAGSECFASAFLSNKQKTVTVIDNGTETTXAFVLPST

CLYKGVSYSIFKILFMMQXGFLVLILECVRDSFFFSITVHLYGQLELLRIQFTEISKRYEYEEKSYVNVLGPLIKRYCQL

TALSKNIEDIINIVILICLLIISVIIAVSGVRIILSXQDYKEMIKMLLSNQFYVFTYASDILQTQSESIIYTIYSSTWHE

MSLTMTKDLLFIMMRIQTPLRISAGKFFTLTRITITDILRTV

>386_Si_gnF.scaffold00899_962711-1051965.pep5

FTILPFVMMDLWFNQNTNSNIECIIFFTGSVLGIAKCLCIAANQKKLSMNINAVIDDWISVKNNKKKTIMKKSAAKAKML

TNTLLFSLIITFSFYISGIIFMNRKQIFFMDDSVNVNTSNWIFIIPSGPLSSSITGSQFAIILPIQIIQIGIMAFMIYMA

DTFIFNITIHLTGQLEMLKNKFKIFANELDYQKKFVNLINRFNELTELYQNLEDSFTFLILFQLVITTVLLILIGLLLEI

REQNYIESVTFALGLAFLLTQSLIPSYSSEYLQEESESIFYALYETSWFTLPLALQKDLHFAMMRSRIPFRFTGGKCFFI

NCETMKHILKTAVSYISILR

>387_Si_gnF.scaffold00330_12820-264637.pep

EIGLRFIGMWPDSAYATLYWLIYMATMVIVQYYQYAYVVRHFDDIPLLMDCLGLTLAYTLAFLKLFALWWNRRTFYYIVK

AMDEDWKECNVNDSYASTMVGMADLSRRCSNVMISINALAAFFLSIGEHMLQSMGDANKVDNNPRELPIKMEFPFDVSES

PIFECFLIGQFLYELVLASIVGMMNALLVSLILHVSGQIDIMRQDINEISYGKYIKGLICKHQKIITLSENIESLYTYIA

LMQLLWNTLVICCTGFVIIITNDSGTTSIKSVSFYMAITLEVFILCFAGEFLSAKSRSISDAVYESLWYDMPPTNSRILL

FVILRSQKRLTITAGKVVDLTLEGFTSIMKASVSYVSVLNAM

>387_Si_gnF.scaffold01629_62255-192132.pep

FEHNYRVNKFLLSAIGQWPYQSSKTSHGIVIILVTIVCTQLLAKLCGIVPYIHDMDIMIECLIPIMVDVSGMTKIMNSML

CVNEIRALLDRIRDDFYSLKNSSDSVILQKYANTGKKFSAVYVSVIYMLTVVFMLMPFQPLILQVANATTRPMLHRVEYY

VDMDKYYFPILLHGYVTAIICVTSIVATDAIFLIFMQHACGLFIITGLRIEQAIQDVYLITKDMAYQNLVKCVHDHRSAI

RFADLMEIAYSKHILFHAGLNMMAISVTSFGALTKSDELFELFRLAAVTCAVSFHLCFECINAQKLIDYSGYLHTNLINL

NWYDASPRTKKLVLFMMMKTQPPCVLTAGGMFVLCMETFATIVKTAVSYFTFLRS

>387_Si_gnF.scaffold07837_2033265-2089281.pep

ENYYKLNRFLLSTCGLEPFQSKWSARLIRAFITVVLMSSIIFQISSWFTFEITYEFVVNGVPSLLVTLCNLNSLHLRINV

DKVKILFDRISKDWALQKTRGEIKIMREHAEFSKLFTFCWTILSYVSMVGYCIWLCTPEILNVLMPMNESRPRRQPFNVE

FFLDEERYFILIRSHMCFVLLTIPIVFVSGFTLFMTLTQHVCGMCKLLGSRAERLFSIVQDKKSQIRNKNMTVFIQQHYN

IIQFVDIIETCYTTLFLSDLTGIMIMLSLTLIQILTISDIEGAIRSIGISCATLCQLFVCCYMGQKITDESLSVYEKMCN

STWYNAVVSEQKTLLIILVRRCHPFVITASKFYTMSLQNFGKIFQTSLSYCMFVRQ

>387_Si_gnF.scaffold10535_2141076-2156307.pep

WAIGINRFTLQCVGLWPDEKSSRQKFLANVRAFVIFTTVTVSIIPSIFSLTRVWDDMIAIIDNLQILLPISATAMKIIIM

WLRKEDLTLVVSMVIADWIKKKTEEERDTMMKQARIARRLVQLGCIIMVSAIVIIIIPPCFGYSMRYLTNVTDPGKPLLL

QTYYFQDTTQSPYFEIAFVAQAAAVVMAAFSYTGIDNFLGLIVFHICAQMEILKERFLSLKELKEYKDFNIGLSTNVQNH

LRLIRSIDIIESTFNLMLLALVVYFGIIFCLQGFLIISINVSFPRICWLVSVVINTFVHMLLYCVVGEILISKCESIYHA

VYDFAWYTLKPNEAKNLMLIMIRADKPLYITAGKMFPMTLSTFCSLIKTSAGYISVLLA

>388_Si_gnF.scaffold02694_2287513-2447739.pep

YSLQLTRWFLIPIGAWPRMSTRVKRISSHMHIFVCTSLIAIIMVPCLLYVSLEEKDTEIKLSVIGPLSHWIMGMINYCLL

LTRSNDIRECVLHMEMDWRLVRKIEDRQIMMRQAKIGRFVAGFCAMFMQSGTFLFAVRKSLSTTIVIVGNETVSMHPMTC

PFYNKIDTRFSPANEIMVVVEWLSCFIVNSVTVGACSLDAVFAMHAYGQLNMLFSWLNKLVIDEDKENEDKENKCVEQRL

AIIVEHHLRVLSFISRIENIMQTICLVELVGCTLNMCLLAYYSITNSDFDAKITSYVIVYLSMAFNIFIFCFIGEILTEQ

CKNVGEKAYMTNWYELPHKTALGLILIIARSSNVIKITAGKLFHLSIATFGDVIKTSMVYLNILRTM

>389_Si_gnF.scaffold00899_962711-1051965.pep

RDVTVTLSVHRFALSCVGIWPVRERNIFMDLRWIIAIFLEASTAIPMFAEIYIHCNGAKRSFDWVTPGAAATLALTRLIT

PRIHRGELLEIVTSMVDDWTMQKEKKIRWIMKKYATMSTRVTVLTFIMVAIILGVYIAMAISAVTTKRQHPDNEINVSVS

DEAESQSCVFRSESSHQAFMVIQAMQMFTTCIITFGTTSFFFGLAMHLCAQFDALSIKLTEFRNAHRVISEAVQRHCQLI

RLAECMEESFNANVLMYLFVTSFLMCIDGYMLIASVGDVPTIVHSASILLLMLIQLSFYTFAGDYLETRSTALAYTTYNC

DWYELPASRAKDFQIIIMRASIPHQLTAGKFVVMNMITFKDILKSTASYLSVLRVM

>389_Si_gnF.scaffold01224_363367-449963.pep

IVLFYAIMPTTINTIRAWGNVIRMVEGIATTNFCMLALCKIFGTWYHRKTLRTLMTSIMIDWTSKNNEERNTMLHITRRG

RILCITCYAMMIILISFFINSNLIKIFRNISQPQRTLVYPFNYFYNSQKSPNYEITCLFQLIAGACSCFINSTIDVFISL

LLLHICAQLINLRTALNNLVDKLAESSSFKKGLAEITTRHEHLIRNTKIVNNCYSIILFLHMFATTFQLCFKSFQFFTID

VPIITKIYALLYFIYELMHLYIYCYSAERLLTESVNMMHGAYECKWYDLPSKDAKNLIFMIRRSAIPFRLKAGKFGTFSI

EMFGNTVKTSMGYLSLLLTL

>389_Si_gnF.scaffold01629_62255-192132.pep

QHYKINQLFMSWIGQWPEQSTFKRFLLSVHLLFTVSSQFCLLVLGLVTAWQDWNLVIECSSAIIIHLVCIIKYLNFLFNN

RKFKSLFNQMKSSWKLATFDAQMQILTTYTNEGKTMIKVYTVILYSSMILYMTLPVMPSIIASFMSANQTQIYGLLFHVE

AVLDTKKYYYYILLHSYYTTFFMITIPVAVDSMMIAYVQHACGLFQAIGYQLKNVKRYPLREDDEHYRTIIDSIVKHKEV

LQFVELLTSSYSISFLLLTILNMAVMAFSAVQAINNRDQPTEALRFLTTCISLSMHFLFLSLPGQKLIDHSSNMHESICA

TNWYAISLRARRLLNLMLVRCKVPCKMTAGKMRVMSLQSFSGVMQASMSYFTVLTSV

>389_Si_gnF.scaffold01968_226495-654737.pep

EWAIELNRYSLEFIGLWPKMKTTREKLTANIRVFLLIIMVAFVVIPCIHSLIRVWGDLMSVADNLQFTLPMVSMIMKLII

MWSKKAALAPLLYMIAKDWLRQKSIKERKIMIRCARIPRMIIICGFVIMFASFILLFILPCFGITMRYITNVTDPGKPLP

LQTYYFYDTDASPYFELTFVAQGVTLMVSAMGYTAIDSFFGLLIFHVCGQLETLKGRLTTMNSEKNPNFNHVLADVIMDH

VRLIRCVKIIESTFTLMLLGLFLYFGTLFCLYGFLLVTVNLSIARLVYLLTAFINTFTHMCLYCVVGEFLVIQCDGVYKA

ACEYEWYNLEPSKAKNLLFIMIHTNKPLYITAGKLFPMTMSTFCNLLKTSGGYISVLLAHR

>389_Si_gnF.scaffold05285_6032-497272.pep

DRYYDISKRYLRWIGQWPSQKPKESLFFFIFILFFDANVIVAQVARFFVCDNMQCIFETLPPHLLAVIVPVKIFVYRFNR

RKVRSLNDRLFLDWNMLKTEKERDIMRKYAATGRWYVSIYGLYIYISTVSFTSTSLAPRILDIVFPLNTSRPIMLPYPAY

YYVDENEYFYYIFLHMIISSTIIVTAIVAHDCMFFTYIEHNCALFAIVRYRFDQVSRERSNMEKSAINYPDYMYYMYHKN

VAISVYAHQKALQYAKLLEDTFSIAFAIQLLLITLCLSITLVQLHDSAEALRYIVFIMAQLFHLFCFSFQGQKLINHSLE

TCDDIYHSLWYKIPVKEQRMLLFVMRKSIEATALTAGKIYVFSLENFTTVVQSSMSYFTLLSS

>389_Si_gnF.scaffold10455_73458-77570.pep

TDKKDIWKSRYYLILRTYMTISGLWPYRSLRERCIHFIPTFALCFSILIPQSMYVLIASDINEVIEGLTSALISIAFSWK

VMCIMFDKNVKSCLKTIKKDWLSLKTDVERSILQRHVEYGRYLTTSYAVFMHTIQVSYILKPFLLTLLEINTTNSTKTSA

SKLPFHVEYGVDADQYFYPIAIHCYLAIFAHIFSTIAIDSLYCTLIQHACGMFSIIGHVLEDIGKNNNASLNLNKISDDD

YKKTLDCLRKHLQVLQFTDLIESTFTKILFVSVNLNVICGSVTGIAMIMNLNKGIEIAAPLSVYVAQLVHIFLQFWQAQF

LLDYSIVPCESICRANWYFTSKRCQKLFLLIMTRTVTPCRITAGKIVTLSIESFGSVSKIS

>390_Si_gnF.scaffold01629_62255-192132.pep

NRYYYLNKRLLSVIGQWPFQSRLEGNIMFAVTSLFMFSVTAFEFWGLAAGITDLNIIMENASPLLVNCMMIIKFINCVIT

NDKMKVLYEDIEQTWKKKCTGAEKEILQRYAEKSRSFTIRYAIALYATWLFYSTTPLVITWIYKLLPTNETYTARFLYRM

EHVLDMDKYFNLLMLHGFISVFYIVQVPIALESTFVFCTHHICALLECIRYNIECLQSTDFVLLEKDDKTYHDLVDCIKS

YKHVLKLSDVLASNYAVSFLFLLGNIIICLSFGAAELVMVDNQPDEIVRILASNAAQLAHIYYLSATSQRLTDYSTEFQE

VIYSCKWYQISLRSRHLLRLTLLRSTKPCQIKAGNMFIMSLETFSEVMQASMSYFTVLTSV

>390_Si_gnF.scaffold01968_226495-654737.pep

AIELNRFGLKLVGLWPKIHEDNYTSDLRIVIIFVIITFISGIPLICSLLRVRHDMILVIDNLQITLPLMIVSLKLVVMRW

KRTALLSIINMMEEDWIALKTDAERNVMIKRARTARMIVICGCVLMIMAFTSIIVFPIFGVPFRRLTNITDRDKPLPLQT

YYFYDTDKSPQFEITLVIQAITIFLAAVTYTSVDAFLGLTVLHICGQLENFKYRLDNLISKDFNSALRSNVIIHLRLIRF

AYKMEDTFALMLLGLVFYFGIVFCLYGFLLLTIKISIVRVLYVAVGVITLLVHTFLYCGAGELITHQCEAIYRTLHNLKW

YKLESRNAKSLILLMARTREPFRITAGYIIPLTMATFCSLLKTSAGYISFLLA

>390_Si_gnF.scaffold02659_451514-467235.pep

DKRLQEYRKYQRITKILLIISGCWYMPTKSGKSTRYWPVCVILLMIMFITLLHTAYVFRHKLVNMMKMIGLTISAVSATI

KVSSFLINRSSLINNHKTLNDFYEEELVQNEKIRTIIFSLRATYTLAYTYVIIMIGLIVGIFLSPYIFIIRNLSHFHSAT

NYTLPLSRGYFWTVPDNILCHLHLILETAMVMLSCITTCGVECVFSLFAYQLASTARGMIYRLTNEKFSDLLKTCIKKHQ

KLLRCRNILEKVYGPILLWHIISNAMLLCSLIYDVTTEFKFTFNILMSLTYSLIKLLQMFMYAWNGTVITNAGDNFRNGV

YFGEWPKLDRHVRTNVILTMMQKPMTIYAIFSPVEIVMFTNFVNATISYFFLLQS

>390_Si_gnF.scaffold02694_2287513-2447739.pep

DFNYAVQVTRVILRAIGAWPIPNSNMERITTRLQNLICYFLFAFIIIPGLLRVFLKEHEFKRRIRLLAPLLNCGMGWLKY

NLLVNHAREIKSCLKQARQDWSNTIDEDNRKMMLSTAKIGRRFAIFSAAFMYIGGLSYRTLVPLSKGRMLTPMNITVRAL

ACPSYFIFDGEVSPAYEIVFTLQFFAGLITYSVRVGAAGLAAFFIMHVCGQLRIIIGKLQYLNNMPLADIVEHQIKVKRF

LSHLEVMMRQICFVELVGCTFNLCMLGYYTISENSDAVAILTYFILLISLTFNILIFCYIGELLMEESSQIGSMCYMINW

YQLSPRSVRSLILIIAMSSHPIKLSAGRMVDLSLTTFGTVSHYVY

>391_Si_gnF.scaffold01629_62255-192132.pep

NRYYYLNKRLLSIIGQWPFQSRLKGNIMFAVASFFIFSFSSIQFWGLAAGITDLNIIMENASPLLVNCMMIIKFINCVIT

NDKLKVLYEDIEQTWKKKCTGAEKEILQRYAEKSRSFTIRYAIALYATWLFYSTTPLVITWIYKLLPTNETYTARFLYRM

EHVLDMDKYFNLLMLHGFISVFYIVQVPIALESTFVFCTHHICALLECIRYNIECLQSTDFVLLEKDDKTYHDLVDCIKS

YKHVLKLSDVLASNYAVSFLFLLGNIIICLSFGAAELVMVDNQPDEIVRILASNAAQLAHIYYLSATSQRLTDYSTEFQE

VIYSCKWYQISLRSRHLLRLTLLRSTKPCQIKAGNMFIMSLETFSELLKMTMSYFTMLTSM

>391_Si_gnF.scaffold02694_2287513-2447739.pep

NKYYERDIGNAFAMHRFFFRMIGLWPFAHANSEQLETIVVVFVCFACLIVEAVPTLLYVFMVLTDIRVKLKVMGSAMFTT

VEIMKYVYMLFYKSQMRNCLILVDEDWQNVVSPSDRTSMIEKVKICKRLVVLCAILYSLNIIVRIVIPLSVGKIVTPQNI

TIRPMPHVAYLVLDVQQSPVYEITYIMQLLGGFFKYTIVVTTFSFVTLCAMHFCSQSNILITLINDFVNESRPENLNNES

RPENLNKKLSIVVEHQIRIKNFLQLVQSVTQYPSLVEVLGSTVMLCFVGYCIITEEDQNIRLCVYSLILVMFVFNVFIYC

YMGEQIIEHVSIASCELEWYRLPDRRARTVILLMIMSNAPTKISAGKFVDLSLKTFGDVIKTSVVYLNML

>392_Si_gnF.scaffold02694_2287513-2447739.pep

YQRDIRYVFKLNKWILGSIGIWPVSIRGIGRYASKIAIALGNLALSFAIVPCALHIVYDEKDIIMRLKLSGLLAFCLVSM

IKYCILAIRRPKILRCIEYVKNDWWQVTFRSDRELMLKYANTGRNLTIIGVSLMYGAGIIYFVLPFCSEHKIANQTIRPL

VYPIYSKFQSQISPVYEIVYMAHCMCGYTLYSVTAGTCGLAALFATHACGQIQILISRLEDLLTGKQNPNVHRRIAVIVQ

NHVQIIRFAATVEEVLQEACLIEFTSSICTICILEFLCIVANDTVSLATYLLLFVSFCFNVYILCYIGELLMDKSSQIGS

ICYMINWYQLSSKSARSLVLILAMSSHPIKISAGRMVDLSLMTFGNVLKTSVAYMSFLRTL

>393_Si_gnF.scaffold03952_3260511-3308427.pep

LNVRINMLSGLFPMTANSRFSIGWKIYSAITWLMILTVVIAFFVGFNLVSKMKAISDGMIGTVFTIEVFFMAVRIHICRD

LIVQFIRNMNDILRVQDETMRCAVMTSLELMHSRFLKFYWLSGITTVIIWIGMPLKAVFIKNSFFYEDYRLPFISKQPFS

MEIFLTGGLLLILCSVYVLLKKAAIDIYTLNFVMLMTAQYRYIAVKLEELFREKNSLNEYNDSWAEAEMKAICKHHHTVV

YMSSRLKKILGPNFCLIYLTSILRFSFVGVMISNTLTTVVERVAMVFFAMGEIIQFYILCLNVQKLLDASTEMTDMAFHE

NWYQLRPSIKRKFMLLIFGNNLTCRITSVEKFDISLPSFMK

>394_Si_gnF.scaffold01080.pep

NHNYKADTEYVVKVAKTLLTPVGIWPLYRSMSDKIKNFLQTGVIFILMCFLLIPHVIYTYFNAEDLTREMKVIAAQVFSL

LAIIKFWTMIINRKGIRYCLQQMEIQYRDVECEEDRLVMTRSAKIGRFFTVTYLGLSYGGALPYHIMPLLADRIVKEDNT

TQIPLPYLSNYVFVVEDSPLYEIIFVTQILISSIILSTNCGVYSLIASCVMHSCCLFEVVRRQMETILNDETSNLHRRFG

KVIEHHMEAIKFAEMIEKSLNIVFLCEMVGCTIIICFLEFGVLKEDGNILPLGTYFVLMTSIFVNVYIISDIGDRLKEES

EKVGESSYSIQWYNLPTKIIKNLILITLRSNRPSTLTAAKIFDLSLQGFCQVCKTSAAYFNFIRAV

>394_Si_gnF.scaffold01629_62255-192132.pep

SEMEMYYATNKFFLLRIGGWPYQRKILKVLIPCFFIMVHYSTVATQALLLFDTWGDIDIAVEIMINFVIVFGGTVKLMNI

VLNNDKFRQLLQHMSEHWELFNSEFEHHILKYYASIGQKITSYYGVYVIVTLTLYLSIPLAPRVLDVVIPLNESRPLIYV

YPAEYRVDKEKYYYPILFHSCLACSITGIILFTVDTTYIVCVLHACSLFTAVSKRLENIIGESNIKSDDDENVHTEMHYH

IFMEKSDDDENVHTEMHYHIFMEKHISIGNEYRQLITNAQILNSVFTCATTILLGTNIMVLSAIGVQLINYIHTGEVIRY

IFILFGTFLHLVCMCIPGQLLINRSAEIFDKAYSSEWYTFSKETRKLLTILLYRSLVPCKLTAGKIFVMSMTMISTV

>394_Si_gnF.scaffold02659_451514-467235.pep

DKRLQAYRKYQRITKTLLIISGCWYMPTKSDKSTRYWPVCVFLLMIMYTTMFRTTYLFRHKLANMMKMVGMATSAVSAIV

KVSIFMINRNSLINNHRTLNDFYEEELVQNEKRSIIFSSLRTTYTIGYTYVILLIGLVMAYIVPPYIFIIRNISHFNSIT

NYTLPISRGYFWTVPDNFLYHLHLIVETIIATVSCITACAVENVFGFFAYQLASTMRGMTYRLTNEQFSDLLKTCIEKHQ

KLLRCRDILEKVYGPIIFWHIITNAMLFCSLIYDITTEFKFTFNILMSLTYSLIKLLQMFMYAWNGTVITNAGDNFRNGV

YFGEWPKLDRHVRTNVILTMMQKPMTIYAIFSPVEIVMFTNFVNATISYFFLLQS

>394_Si_gnF.scaffold02694_2287513-2447739.pep

NKNYKRDINYVFELSRFVFRLLGIWPYARKTETIERLVVILVSYILLTCELVPAILYMAIVQKETRARLKVVATVIFTMV

AMAKYGQLVFSRDRVRSCLAQVEDDWRNVADSRNRDVMTEKAKTGRRLLVICAIFMYSTGVSFRTIIPLSRGKIVTEQNI

TIRHLPCPNYFVFDVQLSPAYEIVFLMQFFSGVVKCTVTTAVCGLAGLCVMHVCAQLEILMVLMNNLVNERENNLVNERE

LKNVNERLAIIVKHQIKARNFLQLVQNTIQYTSLLEVIGCTIIVCLLGYFVIMEDNNSIALCSYLIGLTSISFNIFIFCF

IGEQLSTKGEKVALTACTLEWYRLPDAKARSLILIMIMSNLPTKIRGGKFMDLSLRTFGNVVKTAVTYFNML

>395_Si_gnF.scaffold00330_12820-264637.pep

IGLRVIGVWPDSSYTVLRRAFWMITLAMQTFQYRYFVIHVRTDDLSHLMDGLSTTMSYSLLLLKLTIFWIKRRIFYDILA

MMARDRSECVSEWAVGLMSKTIDISHRSSNLIIGLYSMSVFLYGAGVLVAHPDEPEDQLTTMPPRELFLKMELPFESNAS

PAYELVMITQFFHQLAAATIVGVLNALIVSLILHVGGQIDIMCRGLVEISSGDDTFTIKALIRQHQRIIALSSDIERVFS

YIALMQFLWNTLVICCLGFLIVTSIGDDQMLVKSILFYVVINLEAFIFCYAGEYLSVKSKSIGDAAYESLWYDLSPSENR

ILLFLIMRSQKQLTITVGKFMNLSLQQFANIIKSSASYISVLHA

>395_Si_gnF.scaffold01629_62255-192132.pep

QHMRINQMLMCLIGQWPYQENWEKFLIQLVFVPAVFAQAVLQGGGMITAYFAGDIDAFMESSSPFVISLMCVCKHINYTY

NHDQMKRLAFTMVDDWKIYSKLSHEYDILCRNYAMGRKVTIAYAVSLYGSMTPFLVVPVVLNTASYMGLYNISDGRPLMF

RTEYFIDSEKYYYPLLVHSYIGTLGFVSIVVAIDSMLVFHVQHECSMCEILGYRLARIVAEDNLYPNKEEAISYDHIKNC

VIIHNHIIEYAKRIENANTTSYFFQLGFNMMGMTFTIFQAVVKLSDPNEALRYASFTVCLISVLFLESWPGQQLSDYTDK

IFAFITNGRWYQSSLRVRKVISIMLMRSYKPIKITAGKLYTLNLANFSAVVRTSFSYFTVLCSM

>395_Si_gnF.scaffold02814_302873-346762.pep

REYHVNKIFLSHVGLWPLQNKFIKNLIPISLFVLHLSNFPFEILMLYDHWEDKQLIFETFHFTTSLVMFIAKLFNEFWNY

DKVQRLYQAMENHWNTFTNEFEVRILKDYSSQSRKIIIIYSTITTYIMPSFTPILLDIISPLNESRPRIFSMSFEWRIDM

DKYYVPIVCYNTITLVTGIIICIGIDSMYITRIFHACSLFSIVSEQIENITSTQHEEHELSRKEQVLYQQYVMCVKKYQI

ALKFVDILNSTHQTVAVFFLLLICATLSLIGVRIVYVLGQLEEMIRFTFIIVGALLQLLIMCYSGQKLIDESENIFHRAY

AAEWYNFSPRLKSLLIIILHKSIVPCKLTAGNLFPLSMAVFAAVIRTGISYFTAFLSIK

>396_Si_gnF.scaffold02694_2287513-2447739.pep

RYEQDVHYAMQLCRWILKPIGIWHLIHSQSEKFLSFILILACLSGLCFVLVPAGPYILFREKDINIKVKLFGPVGFCLTS

AIKYCFLGARVSAIGRCIKHVESDWRVVRYQDHRKMMLKNSLVGRRLTTLCVIFLYTGGMSYHTIMPLSSGTKTNGSFTS

RPLVYPGYLYVDPQASPAYETIFFMHCLSAMIQYSCTTAACSLAASFATHACGQVQILMTLLDDLVDGKVAKRLSLIAKH

HVRVLRFTADVEEILREICLMELVAATLIICLLEYYCMMENSDAVAILTYFILLISLTFNILIFCYIGELLMEEYSKIGS

AVYEINWYDLSGNKALDLVLLVVMSHHPPKLTAGKIINLCINIFGVVFKTPVVYLNLLR

>397_Si_gnF.scaffold03952_3260511-3308427.pep

PLNAKLNKFSGNLLPMTDSPFPIGWRIYSAVILLIEAIQTSAIIPGILYKEKTLRDATVGLVITIEVFFLLRQMHVYRDL

LTQLIQKLNQILRTEDKTMKSIVSSTLKPADIPFKFYCMAGVGSMTVWCSISIALIFKKKYFFYEDYRISIVLSEQPFTM

EIFLLGNSIILIASVFIFIRKVALDVYMIHIVLLVTAQYRYIAVKLSTIFRENTLQSHDQKMKVLCRHHCVLVQITLMLK

ELLSLNMSLIYLTNVFIFCFLDVMLISASKDFLEGNMVVMYITGNLVELYILCSCVNQLLDASKQMTNSAFHEKWYQFGP

SVKRTFMLMMLGNNLECKLSMCDKFNLSLPSFMAILNQSYSIALLFLRVK

>398_Si_gnF.scaffold03952_3260511-3308427.pep

NSRVNMLSGNLLPIANSRFSVFWRAHSAIVWLIELIHTIALIFGLILSPTEKSLKDGTVCVVVAVEAFFMLSRLYSRRKM

IEEMIQKMNDILQDTDEIMADIVKSAIRPIIKPFIIYGVTSAMSIAIWTVQPVALALEKSTFFYVDYNLPTAFSAEPFSS

HVLISSSIFMTIGSVYLFLKKFGVDVYMMHLVLMLTAQYRYIAAKLTILFQDIRNCHDESQNKHDESQNKYHSAKNRWTK

RELRKLCQHQNTVLHMSFILKKLLSVNFSLLYVNNVFRFCFIGIMMTTVSLSFAEGISVTFFAMGSLLQFFLLCSSVQTL

SDASTEITDKAFDEGWHQFGPSMKRTFILLIMANNLECRIAAIEKFNLSLPSFMTIMNQSYSIALLFLR

>399_Si_gnF.scaffold01629_62255-192132.pep

ERKNIWQSHFYIVPRAYMSLIGIWPYHAFRVRCLLFVPMFTFSLTIIVPQLLYLLIAAANLDDVFSCTPSMWITIIFSFK

LGWLMVNNRKLKTCLKTMEDDWLSLNTDVEKSILQRHTTYGRYITLTYGVFMQGVGILLILKSVVVMLLEDTSDATVSTL

VAEAKLPLRVEYGEALDRYLYPMAIHTYLAVFSHISITIAVDSCYIALIRHACGMFAIVGHTLEHIGKDNKVKDDNYNRA

LDCLRKHLHVIQFAELIESTFTNIFLVSVCLNMIGGSMIGIQVVLNLNDAKDIVEPLAIYIAQLIHLFLQFWPAQFLIDY

SVLPYESVCRSNWYYTSGRCRKLLFLIMNRSVLPCRITAGKVVPLTIENFGTVLKTMMSYFTMMRSF

>399_Si_gnF.scaffold02814_302873-346762.pep

VQREYKINKIFLSFLGIWPFQNKLARNLIPIFCLAYIISYYSLEILMLYDHWKDARIVFDCCYQIVLLTTFVSKLLNEFL

NHDKVQRLYQAMENHWNTFTNEFEVRILKDYSSQSRKIIIIYSTMMYLLCSMFIIIPLTPTFLDIILPLNESRPRILAIE

VEFRVDTDEYFVLIFCYTTAIIVVGVSIMVAVDAMHFTCTTHACSLFSIVGEQIENITSTQHEEHELSRKEQVLYQQYVM

CVKKYQIALKFVDILNSTHQTVAVFFLLLICATLSLIGVRIVYVLDQIEEMIRFMCIITGALLQLMIVCYSGQKLMDESQ

NIFHRAYAAEWYNFSPRLKSLLIIILHKSIVPCKLTAGNLFPLSMAVFAAVIRTGISYFTAFLSIK

>399_Si_gnF.scaffold03952_3260511-3308427.pep

FNVRLNLLSGLLPMTDKFSFSLFWKVYCAFVWLLELVYMISLILAYFLVPNKIALSDGFLFVIEEVGFVIDYSRILIQKT

LVQQLIRKLNEALSIEDENMQRIVTTNLKQIKNPFIYYLVTGTGSVIFWCCTTLPLILERNTFYHADYPAVFSKEPFSVG

VFLLGNMMIMVCNTYVISVDVYTAHLISLITAQYQYISSRLVLIFRNNKHPNNRSSREGNSSSREGNSEVDSFTTKEIKN

LCRQHVNVMHITLMLKKLISVNIFLIYLINVFRFCFIGLMVNKISESYLVGCFVILYASGSVTQFYVLCACFQRLSEASS

KVTDLAFHKNWYQFGISIKRTFLLLILGNTLECKLAAYDKYNLSLSSFMTVMNQAYTVALLFLRMK

>399_Si_gnF.scaffold07305.pep

FNVRLNLLSGLLPMTDKSSFSLFWKVYCAFVWLLELVYMISLILAYFLNEATLSDSFDGLFVIEEVGFVIDYSRILTQKT

LVQKLIRNLNKALSIKDENMRRIVTTNLKSIKNPFIYYLVTGTGSLIFWCCTTLPLILERNTFYHADYPAVFYKEPVFLL

GNMMIMVCNIYLFLKVISVDVYTAHLISLITAQYQYISSRLVLIFQNNKHPNNRSSREGNSTATEIKNLCRQHVNVMHIT

LMLKKLISVNIFLIYLINVFRFCFIGLIATTMSESYLKICFVFLYASGSATQFYVLCACFQRLSESSSEITNEAFHENWN

QLNLSVKRTFLLMMMASNIEIKLSLFDRFNLSLPSFMSVLNQAYSVALLILRM

>400_Si_gnF.scaffold00899_962711-1051965.pep

VWNDDIAYEFSTHRTLMQILGIWPLQKKTVFTIIHWSVIIILQLLSIILIYMDLTEKYGDTGRTMDAIIYISSTVCLILK

YSCLAANQRKLAKNINAASDDWLCAKNNEEAYKIMKKHSFESRVYTFVMLYSTYTCGSLYIMSVVVVNIKEIFFQAQMNA

SNEDRAYIFPCGSFGDNMTTLQYTMFTIFQIVLLLVVCTAQSITDSFYISLTLHIAGQLKILETKFKNLSSKPDNQLYYR

KQFIKLVNRHCELRDHNQNVEDTFHLVILYQLVTVTLLLALSGIRILIYNGIYFESAKTGLIINYLFMQSLLYCWGGDFI

QRGSTGIFHAMYTTSWYTLPLTLMKDMNFALMRSARSFQLTGGKFFYVNRMAMVYVLRTAASYISFLR

>400_Si_gnF.scaffold02814_302873-346762.pep

VQREYNVNKIFLSRLGLWPFQSKLVRNLLPIVCLILEISYYSFEILMLHDHRDDSQMVFESCYQFVITTAFVVRLWNEVW

NRDKFRRLYEAMNDHWDIFTNEIEVRILKHYSNISRKFTIVYSTMMYLLCSMFIIIPLTPTFLDIILPLNESRPRILAIE

VEFRVDTDEYFVLIFCYTTAIIVVGVSIMVAVDAMHFTCTTHACSLFSIVGEQIENITSTQHEEHELSRKEQVLYQQYVM

CVKKYQIALKFVDILNSTHQTVAVFFLLLICATLSLIGVRIVYVLDQIEEMIRFMCIITGALLQLMIVCYSGQKLMDESQ

NIFHRAYAAEWYNFSPRLKSLLIIILHKSIVPCKLTAGNLFPLSMAVFAAVIRTGISYFTAFLSIK

>400_Si_gnF.scaffold04171_663867-963062.pep

TTADSYYTVNRYLLLIIGLWPYQNFGFRVIVITFLTIVLTSGVIVQFTTFVTKEYSTDLLLRILAYSMPWITHLLRYNIL

CLNAKKTQDLIERVRINWNELNNARELEIIKKYSAFGKLITLVSTWVINLGVFGVIIMTVLVNYVLNVPTSANESRQREF

PAEIECFVDHQKYFAPLLVYMFFVVWSSLTVLAASETLFMSYTQHACGLFEIVNCRIEQSLNRGTPQNLTSAEKNSIICQ

GIISAIDIHKKAIEFVEMLKDNFKWVFFTALPLTVLSLSINLYRLSRQVTSDEETITTLLFATGQFGYLFFCNYLGQEVI

DHSGDIFHKTYNIQWYMAPLKAQKLLLLVMQRSMQYCTITIGGLFIPSLEGFASLSSMSLSYFMVIYSI

>402_Si_gnF.scaffold00899_962711-1051965.pep

MTDKRWNDDIADVFYVHRVFLKVYGLWPLQTQTVFTKIRWGLCMTAQFTILPFVMMDLWFNQNTNSNIECIIFFTGSVLG

IAKCLCIAANQKKLSMNINAVIDDWISVKNNKKKTIMKKSAAKAKMLTNTLLFSLIITFSFYISGIIFMNRKQIFFMDDS

VNVNTSNWIFIIPSGPLSSSITGSQFAIILPIQIIQIGIMAFMIYMADTFIFNITIHLTGQLEMLKNKFKIFANELDYQK

KFVNLINRFNELTELYQNLEDSFTFLILFQLVITTVLLILIGIRILIYNGIYFESAKTGLIINYLFMQSLLYCWGGDFIQ

RGSTGIFHAMYTTSWYTLPLTLMKDMNFALMRSARSFQLTGGKFFYVNRMAMVYVLRTAASYISFLR

>402_Si_gnF.scaffold02694_2287513-2447739.pep

SNQKKYHGYSLQLNRWFLILIGAWPQINASSKIFILIQVLFCWSVVASMTVPCLIYVLFEKISIKTKLKALGPLIHRAMG

IVNYCVLLKRSGDISKLIRHMEADWSLIQKTDNCEVMLQHAKLGRFITIICGVIMQGGVFLFSLARAMKTVPITVDNQTF

MTHPMTCPIYSKIDTRFSPVNEVALALQFVSMFIMNCSSVGVCSLAAVFAIHACGQLNVLYAWLHKLVEEQEKSKQTTKR

KLEKSKQTTKRKLAAIVEHHLRVLSFVSLIEDIMNTVSLAQLMGCTTVMCLIGYYMIMATLDAANIISCVFTYLSYGFNI

FIFCYIGEIVTEKCKYVGEMAYMTNWHDLHHKTARGLVLIILQSSNVVKITAGKLINLSFITFGDVLKTSVAYLNLLRT

>402_Si_gnF.scaffold06890_748328-817847.pep

YQSVNITRIFMKMVGLWYVETPKDRLLLRAAFGYAIWAIVFAILVMGVDLYHCLGDFYAVTTNLCATLLLVMVLVKLGSF

MFYRDKIMNLIHFAERNFWNATHGETDTQILKQYDKLGMFLIYTFTFIVTVATFNYIFAPFFEPQEKNETKKILPFKLWF

DFPYHSPYYEITYVIQSLSTIHSGICTFCFDNFVSTFNIHAAAQLNILAHRVKVVVIENCIEDTMDQKLSLETDATVFKK

LHNCIQQHLTLIRYVRNMQRVFATMLLGQLLFSSVIICFGGFQMLAADVIIRKCIFASYFVGGVLQLLIYTWTCNDIIVQ

STSISDAAYNSKWYLLGRSVKKGLIMMMIRARRPCELTAGPFAVMSLDLFTSILSTAMSYFTLLRQM

>403_Si_gnF.scaffold02694_2287513-2447739.pep

RDNDYSLQFNRWILKSIGAWPEFRKNSKNILINILRLACHSLMVYTIVSSTLYVLFEEKDLRLRLKAIGPTSHIIMGGIN

YCSLLHNNDRIRAFIEHMETDWRMAKREQDREVMLRNARIGRVIAAVCTLIMQVGVISYNIARGMSRITVVIGNKTIETG

GLPCASYNKVDTRLSPVYEVVLAVQCLSTIVVNNVTVGACSLAAVFAMHASGQLNVVMLRLEELVAEKQDLQDLQLKLAN

IVEHHLRALRFLSHLEVMMRQICFVELVGCTFNLCMLGYYTISEQESINTIITYVMVLTSMLFNIFIFCFIGELVTDQCK

NVGEKAYMTNWYELPHKTALGLILIIARSSNVIKITAGKLFHLSIATFGDVIKTSMVYLNMLRTM

>404_Si_gnF.scaffold02814_302873-346762.pep

VQREYNVNKIFLSRLGLWPFQSKLVRNLLPIVCLILEISYYSFEIVTLYMHRHNGQIIFESLYQIVVSLAFITKLLNQLW

NRDKFRRLYEIMEDHWNIFTNEMDVRILKNYSHISHKFTILYSTLTYTMMSLFIMIPSLGPMFLIVLPLNKSRPRNIALY

SEYGIDRDKYFFPIFLHTSIVIIFGVSIMVAVDSMHIVCTAHACSLFQIIGRQIENIISNMNTDNKMNKLERYPNEEYKI

EYKILNEKIIYREYITCIKKHQLTLEYVIVLNDTHKIVGFSFLFLIGVCFSLLGIRIVYVLDQIEEMIRFMCIITGALLQ

LMIVCYSGQKLMDESQNIFHRAYAAEWYNFSPRLKSLLIIILHKSIVPCKLTAGNLFPLSMAVFAAVIRTGISYFTAFLS

IK

>405_Si_gnF.scaffold02694_2287513-2447739.pep

SNFEDDIRYTVQVHRLILGLIGVWPYLERKWTRFLRGLLRTMCCFLLSFNLIPWLLYMILIMDTFKGRLKMLGALFFYIM

VPAMYCALMLREDRIKECIRHVQEDWRNVRNANDRRIMLDKARAGRFILISTTLFLLTSGFTYRLIPIMSSKITIGNVTI

RPLVQNYYIFFDPQKSPAYEIVFSMHLVTGIVVYIVTTSVCGITALFTMHACGQLKLLAAWLENLPKEAAVARRLATIVM

HHVRIRKFLHQIQGVVGEMCFIEFIGSTLILCLLGYYVVTGRNDALSSMTYAIMLVSFTFNIFILCYIGEVLNTQGSEVN

TTCCTIDWYCLPSKEARYLILVIAMARYPTKLTAGKVINLSYSSFSAVVRTAMAYLNLLRTV

>407_Si_gnF.scaffold02694_2287513-2447739.pep

PNKNYKQDIEYVTKQTNFLLRILGIWPLLDSVVEIACKILLIIICCILVCFESIPSMLYCIFTVEEPRMKLMMIAPTIYS

FTSLTKYGALIVYENEIRKCFRHIKDDWKFIAMSGARDIMMEKVKTARSMFTICCTCLYTAALSYHTIVPLTRGKVITSN

NVTIRPLAYAGYFVFDEQRSPAYEIVFTLQFFGGFVMYSVTIVTYGLAALLVMHACAQMKILMMLMEELVDEKEKNTDEK

LVAVVERQIRIRNFLCLVEDTLQISNLFEILANTTMMCFMGYCILTEDGNTANAYTFVVALASDIVNIFLLCYIGEYIID

TADKVAWKTNMLEWYRLPTERTRDMVLVILVSHMPSKLTVGKFIVLSLKTFGDVMKSAVVYFNILRTV

>408_Si_gnF.scaffold02694_2287513-2447739.pep

NIKFTYKSNNDYSLQLTRWFLLPIAAWPRPTTTVERISLQAHVFACVFLILIILIPCILYVWLEEKDIQIKLSVMGPLSH

WIMGTINYWLLLAHSDDIRECVQHMETDWRMVRGTDDRDVMLRYAKIGRLVAGFCAVFMQSGTLLFMVAKAVTSMTVIIG

NDTVTMHPMTCPIYSKFDARFSPANEIMILTQILSCFIVNSITVGACSLAAVFAMHAYGQLSMLFSWLNNLVEDEDKENE

FAEEDKENEFAEQKLATIVEHHLRVLSFISRIENIMQTICLVELVGCTLNMCLLAYYSITNSDFDAKITSYVIVYLSMAF

NIFIFCFIGEILTEQCKNVGEKAYMTNWYELPHKTALGLILIIARSSNVIKITAGKLFHLSIATFGDVIKSALAYLNMLR

T

>408_Si_gnF.scaffold06535.pep

YKRNNNYSLQWTRWILKPIGVWPELPSAIEKILSKILRLTCHTLIALTVIPSILYIIFEEKDIELKLKAVGPASHWLMGG

VNYCSLLYQKGQIRKSIKHMETDWRMAKRQCDQEMMLKNARAGRIIAGVCALIMQGGLFTYNVARGTSPILVAIGNETVT

MNRLPCPSFNKVDTRFTPVFEIVFTLQLLSTLVVNNTIVGACGLAAVFAMHACGQLSVVMSRFEELVEEKREEKRHNVIQ

RKLANLVEHHLRTLRFLSRMEIIMRQVCFVELTGCTFNLCMLGYYAITQEESTNTIIAYFVIFTSMTFNIFIFCYIGDLV

TEQCKKVGEAVYMTNWYKLPQKTVLSLILIILRSSIVIKMTAGNLVHMSISTFGDVMKTSIAYLNMLRTL

>413_Si_gnF.scaffold02694_2287513-2447739.pep

KQNEAYISDFRYAVQISVWLLKPIGVWPLFNSRLKIALHKALMVIGTFIVLFMVVPWTVYIVKKKLDVFLIIRTICPLLF

STTTSVRYILLLWNQDRLKFCMEHMADDWRCATIAKDRDVMLANARDGRTFGIISMVFMFSCGGLYYTLPIVMPNPINEN

NVTVRLHPSPSELLFDTQASPVYEIVYFLQILSGYTIYSAFSGTCSLIANFVTHVSGQCDLLTTIFEETVDGGEHNSGSI

ENRGSIENRIATAITRHMRLLRLVSDVSNLFTEICLVEFINASCNICLIIYYIVTDNEPFMPTFMFGFGLMSIIFNLYMV

CYIGDLLKERCQQVGNACYAIEWYRMPYKNAMRLLMPIAMSQYPTTLTAGKMVTMTVTTFSDILKTSMAYFNLLRE

>415_Si_gnF.scaffold02694_2287513-2447739.pep

KYNEYSIQIIRWILKAINAWPRPASIVEKIHSDFARFMCYFLIIAVMVPNGLSMFLDKQPYADKLQNFGPFTFWAIAIVN

YSCLLIHIDDIRDCVEHVKVDWRIIKGLEDRKVMLKTARLGRFFAGFCAVFMHCGVFSYNIVQGTLKTALQMENGSVTVR

TLPYPFYHKLNAYYSPAYECVFFMQFLSSFIVNSVTVATCSLAAVFVMHACGQIKIMISWLENFIDNRNEEGTSMRTSMR

QKYAIIVNHHLRIISFVSRIEKIMNIICLVELLGCTMHICCLGYYCMMDSGDKRSILSHGMVLCSITFNIFIFCYIGEIL

SEQGEQIGKSAYMTNWHLLPGKSAQGIILIILRSNTVLKITAGKIVQLSFATFGDVIKSALAYLNMLRT

>426_Si_gnF.scaffold02814_302873-346762.pep6

LHDHRDDSQMVFESCYQFVITTAFVVRLWNEVWNRDKFRRLYEAMNDHWDIFTNEIEVRILKHYSNISRKFTIVYSTMMY

LLCSMFIIIPLTPTFLDIILPLNESRPRILAIEVEFRVDTDEYFVLIFCYTTAIIVVGVSIMVAVDAMHFTCTTHACSLF

SIVGEQIENITSTQHEEHELSRKEQVLYQQYVMCVKKYQIALKFVDILNSTHQTVAVFFLLLICATLSLIGVRIVYVLGQ

LEEMIRFTFIIVGALLQLLIMCYSGQKLIDESENIFHRAYAAKWYLFSPRLKSLLFITLYRSIVPCNLTVGKFCPLSMST

YATVIRAAMSYFTAFLSF

>AaOR1

QRVAFWIWKILGIWATDDESPLYRAYRRIYHFFFTGIYLFSMFTSSFFTENSEELWVEILFILPTEIAMLTKTIITVYKF

ETIHRLLQTTVSKELQPTCPKHGKEYDRFFDRFSKVMLMYYFCSVCAWTHLGFLFDDRLKLPFFNWFFWDRDHLNNYYIL

FAYQMIGMMGHCSLNVSGDMNIAYLLSIAGQQLDLLSCKFASLIQHFAREIERTVSWCVFAQICASGITICAIVFRLSAI

IDHLGTSIPMFFYMVSMLTQIFLPGYFGNDVTLKSQKLTNALYTSKWYQLAMNDRKDLKMMTLRTSESIRLKAGGFFNFN

LEAFTSTLNTAYSVYAVLNS

>AaOR2

FWAYLRKPKWYSYLLGCVPVTVLNVFQFMNLFHGSGDMNKIIIDGYFTVLYFNLVLRTSFLMGNRGKFETFLEGIADEYA

VLEKQNDIRPLLDQLTRRARILSKSNLWLGAFISACFVTYPLFSPDSGLPYGVYIPVDVHASPIYEIVFVLQIYLTFPAC

CMYIPFSSFYCTCALFGLVRIAALKRSLEKIHEYNTSLFARIKECLQYHKDIIKYVSDLNELVTYIFLLELLSFGMMLCA

LLFLLSISNQLAQMVMIGSYIFMILSQMYALYWHSNEVREQSLEIGDSLYNSAWLDFDNSVKKKIILMLARAQRPLAIKI

GNVYPMTLEMFQSLLNASYSYFTLLRRV

>AaOR3

LWLQEKLRKSRWKYLRYRFAISCMCIVVIVPKIFLYRDDIRLVLRGISELIFQANIALKVVIFVWNQDHFEQMLALLRKW

FEKTFSDSEPNKALLRCNRVNDLFAKIYFTYLAVVVNIFNIGPLIHSTFIYLTFDRENSTEPVELTTHMEQEFYGFDIRT

NFLHYMGFTACSILAYFSAAYIMAVEAGFIYCSCKSCSQCSQCFKSSSKWELEDIIAMHDDAYRCLELLDKNTTFASMVQ

VINCVLMWCLMGVYLTYNVNYSAVNVLVLFGLVTFETYARCLLGTEVSQKSFDVYQAVYNFTWHETPVPIQKNLLQVLQR

AQKKVGLTMVGFC

>AaOR4

QSLEFDQTFGFIAKVLQMIGYAPYPTTFASRLKSSAGFVVCFLMLTYCVFGQIINIGLQTDVEEVAIQVSSTGFCIIGLA

KMYSLSYNRAILSWLIADFRVKWNAGELTDKDRSIRDGTLRPTVAITTVAALGNIIMVSAFNFQPVVEMIYGRVVTGEWV

KLFPYVIWFPFNSTHGAIYYLVYLFEVYSGVIVAVGNVGFNCIFCLLTSHLSMQLKLLCSWIEDMVEVEDEKGVQSKKKK

KLYRIVRYHQDLIRGRNALQSMFSTTLFLNFSASSVLMCMQLYLITTAGITLMVKFTLFMLCILMEIFILCYYGEEILAN

SSSIAAGAFNSNWYQSNPRFGKNLIPIIQQGQRPMVLTAWKFWPITIRTFSAILQTSWSYFTLLKTV

>AaOR5

SIEFEQTFGFIAKVLQMIGYAPYPTTFASRLKSSAGFVVCFLMLTYCVFGQIINIYRQTDQVEEVAIQVSSTGFCIIGLA

KMYSLSYNRAILSWLIADFRVKWNAGELTDKDRSIRDGTLRPTVAITTVAALGNIIMVSAFNFQPVVEMIYSRVVTGEWV

KLFPYVIWFPFNPTHGAIYYLVYLFEVYSGVIVAVGNVGFNCIFCLLTSHLSMQLKLLCSWIEDMVEVEDKKKLYRIVRY

HQDLIRGRNALQSMFSTTLFLNFSASSVLMCMQLYLITTAGITLMVKFTLFMLCILMEIFILCYYGEEILVNSSSIATGA

FNSNWYQSNPRFGKNLIPIIQRGQRPMVLTAWKFWPITIRTFSAILQTSWSYFTLLRTV

>AaOR6N

YPVSIKSALKRNAVFLMAFTLLFYTAFGELVYLVERDFSFLEITFQAPCLGYCTIGLMKMLILAVKRNTIAELVQSLQEE

WNKSVRSLEHQSICDDVMKPAIRFTTIVAVVNIVMGLAFTLLPIPEMIYYYSTHGKWVRQLPFLIWWSFDAYSGFVYYFI

YPLYVVIGFSGIIIHMGFDCLFCILSAHLCVHLRILKFDLENLTYGLDSSSENMSIKLNPKLFYIVEKHQNILECHDRMN

KIFNFALFYNFFVSSFIICIQGFMVTAASGYTLIKFALFLASFLVELFLLCFYGHHIVESSVLVAEAAYNCLWYNTNHQF

RTIILQMVNKGQIPLSLMAWKIWPVNMNTFANILSASWSYFTLIRTV

>AaOR8

VKFESFIRVPEIFFDMIGITRYGEDTWKARLKQAFFWSSYANTIFCLIIEHIYFIKNFTNFLELTALAPCIGFTALSIVK

IMTIKLNEAKLNGILDRLSDLFPRSHLDQDRYRTYNYNLESQMVMKSFSILYMILIWIFNLLPLVSMLVNYISTGILEKE

LPYFMWYWYDWHKAGYYEITFFHQNWGAFDSAVFNLSTDLLFCAIILLICLQFDILAYRLRHDYKELEQCVKLHQSVVEL

SNQLEGIFSPSILVNFVGSSVIICLVGFQATSNISAFDLFKFILFLISSLVQVFLLCYYGNKLIEASSQIGYCAFEGTWY

MADLRYQKSLLFVMTRAGQWQKLTAMKFSVVSLASYSAILSTSFSYFTLLKT

>AaOR9

VWRFWSFILKHDYMRYISIIPVTAMTVLMFTDLYRAWGNIGEVIINAYFAVLYFNAVLRVLILVHYHEEYESFLEKIADV

YREIISDEKTKEMVQLFTKRARVMSVSNLGLGAFISACFVVYPLFTGERRLPYGMHIPVNKFESPLYEILYVMQAVLTFP

GCCMYIPFTSFFASTSLFGLIQIKSLQYRLENFKQNGTGKSNKEQRTGKSNKEQRSQLEAIISDHQRVIAYVGELNGLVT

YICLVELLSFGMMLCALLFLLVIIEHYAQLIIVVSYIFMIISQIFAFYWHANEVREESMAIGEAAYSGPWIELDQASKKK

LLLVILRSQVPLEISVGNVYPMTLEMFQSLLNASYSYFTLLKR

>AaOR10

VWRFWSFVLKHDAMRYISIIPVTVMTFFMFTDLCRSWGNIQELIIKAYFAVLYFNAVLRTLILVKDRKLYENFMQGISNV

YFEISHIDDHQSLLKSYTVRARMLSISNLALGAIISTCFVVYPIFTGERGLPYGMFIPLDSFRSPHYEIIYIVQVVLTFP

GCCMYIPFTSFFASTTLFGLVQIKTLQRQLQTFKDNINSQDKEKDNINSQDKEKVKAKVVKLIEDHKRIITYVSELNSLV

TYICFVEFLSFGMMLCALLFLLNVIENHAQIVIVAAYIFMIISQIFAFYWHANEVREESMNLAEAAYSGPWVELDNSIKK

KLLLIILRAQQPLEITVGNVYPMTLEMFQSLLNASYSYFTLLRRV

>AaOR11

LRLTINGLKYYGILLYKSQPFKELNCFRGVCFTASMLAFNVTQYVDLYQVWGNIAEMTANAATTLLFTTTIVRILHFYWN

RARFNNAIKVADEGVQHLLRAPEKEIFWDNVKYMNRLTAAFWICALVTANTMCVYALVQYQTLKSMDLFNSTEPFDPPLI

LRSWYPTDNIVDSFATIYLIQLYIMYVGQLIVPCWHVFMVSLMLYARTALMALNYKLAHLEQYARCNVRKEIVECIQQLH

KIFEYTRELEALTRGAMFMDFVVFSVLLCALLFEASSTNSFVQIFIDICYIMTMTAILFLYYWHANEIHYQANLLSSSAF

MNDWYNYPRSVNRHLITFICYSNKPLDMKAYIVSMSLDTFLAILRASYSYFTILKQ

>AaOR12C

LRLTINGLKYYGILLYKSQPFKKLNCFRGVCFTASMLAFNVTQYVDLYQVWGNIAEMTANAATTLLFTTTIVRILHFYWN

RARFNNAIKVADEGVQHLLRAPEKEIFWDNVKYMNRLTAAFWICALVTANTMCVYALVQYQTLKSMDSFNSTEPFDPPTI

LRSWYPTDNIVDSFATIYLIQLYIMYVGQLIVPCWHVFMVSLMLYARTALMALNYKLAHLEQYACNVRKEIVECIQQQHK

IFEYTRELEALTRGAMFMDFVVFSVLLCALLFEAS

>AaOR13

QMLRFIGLWGDRRQVVRYLLVLFSEFIFLIGPKALLGSDKEGFDSTARNIGELIFLVEVCISIGIFASRRASFERLIVVL

ENILRRKWPRNLQDEIYRFHRRMEFFARAYALYIGFLLFLYNCVPIGSTIVKLIRFDESERSDFMLVVELQFFFDIRRNV

HYAIYMAFCFVAVSCSAYQSTLKGSVIVVVTQYGSKLFELISKRIDAMRSIERADRELREIVKLHGMALEYVQHLESTIS

FVMINQIMNCIFIWCLMMFYVSTNFGPNAANVMLLFLVLMGEMVVYCLNGTALSEQAAGVGHAIYNYPWYKESVAMQKNM

QLMIQRAQRPTGITAAKFYFVNIERLGLVTQASYSYYLILKN

>AaOR14

MPLALRLLETYGLRGEKRKFLLFQVMIFWEMLMIVIPKIFLGYRSQDLVIRGLSELLFQLHIMIRICIFAWHRFKFEGLV

AIIRRVYKKICSSEGDSTMKADLNVMINKQCKGYFLYIMGCVSLFTVAPLFQSLGKFITNRRGNATENVEYITMMEQEFY

GLDIRGNFSHYLIYVALGSIAFFTSASFFAVTGVIMNCVRYTMPMFRLIVVRLNKLHEEQHIREELRETINLHVDALRCV

KKLEKIANVAMVIQIVDCVLIWISMILYMRNNLSVDAISLMVLFVVLTGETYALCDLLTQLTNESLAVTRAIIDCQWYSL

PLDIQKSLSFVLFRAQRKEGITAAKFFFMDVERFGRVAQTSYSIYVVLK

>AaOR15

EAAMPLALRLLETYGLRGGKRKFLQFQVTILWELLMIVIPKIVFGYRSQDLVIRGLSELLFQLHIMIRISIFAWHRFKYE

SLIDIIRKVYRKGGDPTSKSIILKFNQMINKQSKGYFLYIMGCVSLFSVAPVVQSVIIFMANQSRNGTEKAEYVTMMEQE

FYLDIRGNFHYAIYVALAGLAHYYSASFFAVTGVIIICGVRCTILTFKLINVRLSKLHEQDIRDELREIIDLHVDALRCI

QLLEQIANLAMVIQIIDCVLIWISMILYMRNNLGVDAISLMVLFVALTGETYALCDLLTQLTSESLAVTRAIIDCQWYSL

PLDVQKSLSFVLFRAQRKEGITAAKFFFMDIERFGSVAQTSYSIYVVLK

>AaOR16

QKTYDDMMHQLNSVAQKYLGMDTLHLEFSFVNRRFIFLLAIMSTFLYADVESAVLAGDVGEVAYNIAVLGIGLQGFAKFD

AYVYRKESMHTLVWQISAFLNKKKMFDGLNEIVTANVAIMVLLKRFYIGLYGFVFVSMSSLGLISSLSSGERQLSFGFQF

SFDTSNWVGYLATYIYQVAGILMVVISSCCNDILIVVLYITAMGMYDCMMFDLRELSKLSQMEKSSANKREKSSANKRIA

EERIKSVIQQHQEVLEFLELSNETFSLYFLMSLVCMTAAIAILLVALVWNRWYAGLVICFAASSQIFALSLLGTLLLVKS

EELIDEVYSITWYDMDLPVQRSLKLFLLMSQHVKEISYRFGVMNMETYVQSHKMIYSFFTMLVT

>AaOR17

DMAHQLNSIAQKYLGMDTLHLEFSFVNRRFIFLLAIMSTFLYADVESAVLAGDVGEVAYNIAVLGFGLQGFAKFDAYVYR

KESMHTLVWQISAFLNKKKMFDGLNEIVTANVAIMVLLKRFYIGLYGFVFVSMSSFGLISSLSSGERQLSFGFQFSFDTS

NWVGYLATYIYQMAGILMVAISSCCNDILIVVLYITAMGMFDCMMFDLRELSKLSQMEKSSANKREKSSANKRIAEERIK

SVIQQHQEVLEFLELSNETFSLYFLMSLVCMTAAIAILLVALVWNRWYAGLVICFAASSQIFALSLLGTLLLVKSEELID

EVYSITWYDMDLPVQRSLKLFLLMSQHVKEISYRFGVMNMETYVQSHKMIYSFFTMLVT

>AaOR18C

YILLLIIMSTFLYADVEAAALAEDIGGFSYNIAVLGFGLQGFAKFDAYVYRKKSMSELLWSVSRFLGENKGNNRLNPLLV

DNVSVIVMIKRFYFKLYGLVFVTVSTFGMITSLINRERSLSFGFQFSFDTSQWAGFTFTYCYQVISSMMVVISSCCNDIL

IGAVYVNAMTMYDCIMSDLRELSKMSEMEQTPGNKRMAEDRMKSIIQQH

>AaOR19

ILLLIIMSSFLYADVEAAALAEDIGGFSYNIAVLGFGLQGFAKFDAYVYHKKSMSELLWSASRFLGENKGNDRLNPLLVD

NVSVIVMIKRFYFKLYGLVFVTVSTFGMITSLINRERSLSFGFQFSFDTSQLVAFTFTYCYQVICSMMVVISSCCNDILI

GAVYVNAMTMYDCIMSDLRELSKMSEMEQTPVNKRQTPVNKRMAEDRMKSIIQQHQQLMQFLDRANEVYSSYFLMSLASM

TGTIAVLLTALVFVRWYPAIVICFAASFQIFSLSLLGTLLLIKGEELVEQVYDINWYNLDLKVQKSVKLLLLMSQHYKEI

SFRFGVMNMETYVKSNKMIYSFFTMLVTTK

>AaOR21

YNAIIGANMFKSKSIVLTFPFALAVCMHCIYVYLFTSSMYYYRDDIEKILINVTTIGFSIQMLAKLYTFLYGRQNMVELH

KLNLIYFENNHFGSETVKEALFKNAKFTYVVLQLVVIYLFTLWILITAPFLLYSVISSKRLLPFVFEWSHSENWAYSVNF

VIQAICFFYVVVGTYSTDATFIVYLLTGCGQIDAIGAMLQDLNTSEQQITEQITRIVKLHQHLMLYMSDLESKFSGYFLT

TLGVLSFIMIVSMCALILINWATGLLLVLIATCQLLFVCSLGTYWQIKCDKLLVDVWSLKWYRLSVRNQKSFLLLLNGAQ

APLNLTAIFTPLDMSAYLSIHRTLYSICMLLIQF

>AaOR22P

NYRGTFAVFRVFMLICGVNFFDEDFMVGPVNMFRFLGPMLTGYYSLVACFIHLIRYLGDTDITILSLDALFSAFEVLIKV

GGMALKRKLGARLMKTILEDRSYEDGEIERFTFLKYHTLARKLMYITIISYPFTALMLLSYPVLAGKLDEYVLPVGYSIP

FNYKQHPWYTINYLITIAQMAWCALAFIGCDGPFYLYVCYSSCKLEILKSYTEKIGETNDIRTLMRKIIKIHTQVLEFLR

DCSNFYNEIYLTQVLFSIAHICVSLFHVQLKWKNSSYGMLATNVAKMWIXFVTVVVTKSNELSEAMYTNRWYQWSKKDLK

AVQFMLANAYRNVGFSIGGFGFLSYDAFAEIMKTAYSCNAFLHNM

>AaOR23

IFLVCDLTLYIVVNCWCLTVFWGQLTDVVFCLVTMGIAVQGFAKIANYTDDRLYELHVYNVARFDRVRDYPEARESLQTT

AVLCKVFIKIFSYLFMMLTTFIPVYTIVYSITSRSLQLPFGFFFPWDHTQLFGYIINLSYHFLQIYEASYGLLATDTCFL

FFIIHAMGQLDVIIIYLKKLDELALKFKNDEELYQLLNDITEKHQEHVEYMSKMDSLLKPGFFVNFSCMIAETVASLYVQ

SETDGIWYPGLIVVLLCIVQLFIACALGTIYSTKNDQLIDEIYNISWYAMPIPAQKSLALILNSSQHPVVLSDGFDAIDL

FAFVQIYKKIYTYFTMLQSF

>AaOR24

YAHQIKTPNMISKISGLNVFSEDFTVPNKFLFGILLLLGFYFYINAASAYEMRNDTEDLINSLTTFGIATQAASKLVIFI

IFRKDLNWLHKYTEQLYREECNPRTRELLTDNVFLLSVILKTMMVGYGFTSFSLDVAPMLVLAFTGSKLLPFGFYIPIDR

FSWFGYIINYMVQIILTVFVTSEDMGPDCIYMIISMNAFTQIDLIIDSLKEVNRHEVDDHIIKIIQRHQEHLKYLRTVEI

IFRMIFFASFVSLSSVLILSLFAVVTLGWYQGIVFILFVSYQLFFGCFLGTFLEFKNEQLQREIYTISWYKLSIKNQKSL

RFLLQSAQEPVNWTLIFARLNIPTYLQVYKTIYSIFTMLLTVR

>AaOR25

FRFIRRALSVAGCDIFEENWRPSAWTFIVLLFASIFPYFAVVFLLNHHDDMERLAESVAIFITSLDGIYGLLEFIVNRNK

WNEVMKNIHSRRFQYKSKTISELFDLYYYRNYQFCKVLYSAYISSAVSILLAPFVFPIPEQYDLPAACTISIEPAEPYFY

PVNYIFQAIVIFSVQHVLIAQCLSLVTGIMSACCQIRALKIKIDELNEQIADVRESLGEIIYLHQCTKEFIVIIQRKYGV

VYLSMYMVCGGIVCMCLNVIAQNIFTSATLLTMAGVFSVFVHCFFGNLLLIENDSLPDKIYALDWHELDIAQQKSLKLLL

ENAQPDSLLHGILMPLNMSTFVSIMKAAFSYYSIL

>AaOR26

MATNVKLCQLCGLWGDIAYRSHVWRAVLFSFCLIFWYLVPSFMFMISEEPSFIVLMKPILELFAITMFVLRISNHVLCRE

TLYECYRDLQQAYDQFAGHEDVGSIVRHVRRSAELLTKIYFSMVYFQAATYGVVPAITTIVRYSFGNEAVELPSTVLEAD

FVFDHKANFWTWLPTMMTSIAVQYGMLTFSSNECLFWNMLHHVSCLYKIVYQEIGRLNEYKNPDKNPDEFKRQLAIIVEV

HEVCFRTSRRLESVLSPAMALLYFSCIFQTCYVMLVVSVIDDLFLLASMAFILQYTVFLIFSFSMLGTELMDASSLISEA

IYNTKWYEWAAPERRLLLFMLMRSDRVVAISAAKFFHLNRATFGVAMKTAFSYFTLMQR

>AaOR27

MSANVRLCRAVGLWYDLTHWRFTWQPIFVILSVFWFMIPTVAFMIQREKIFAVQLKPILEIVEIGMIVFRTTAHWYGRRS

LTNCFDDLHKAFEQFSVHEDIRRTLRHLQRSASYLVKIYVFVVLFQALSYGPLATFITIVRYCRSDETLVLTSPVLEADY

VFDHLSSSAWLPSSLISVSVQFMMVISITASECLLWNLLHHVSCLFRIVRYEISRLDSDRKTFRKQFVEIISAHNTAYRC

ARRLESILSPVVGMLYCSCIFQTCYVLFVTSVVDDPMLMASMIFILQYTTFLIFSFSMLGTELMGESALVSEAIYTTRWY

EWSVDKRRLVLFVQMRADRITGITASKFFYLTRPTFGTAMKTAFSFFTIIRSL

>AaOR28

RSSSLEYDSFFRLPKIFGLLNGVVYNDEKPSSKKAKNVYFWISLMHSILVAVLELVYLAKSVEDFVFIMSLVPLVGHGIL

AIVKLSVQKYYHKEINSILISLKDIYPSTLDDNITKDYSKKILYMKLFVIFYLVTLIFFNIVPFAPVLHTYFTTGVFEKT

LPFFIYYWYDWRRPILYELTFIEQIWVSTASVVANMNIDLMLCSLILQISMHFDVLSDRLSVLQHNQHNDHKELTKCVER

HSVLLDLCLRVENIFSRSMLASFLLSSVIICLTGFQVFAQDSINKAIPYATFLFLHMVDVYLLCYYGNLMMEKSLDVSNY

AYESLWYLGNRPFQKSILIILERGQRAQTLTAMKFIVINLTCFKTILSTSFSYFTLLK

>AaOR30

NLTIAYLRMVGYWSHPDRPFRPTQAICLIGVLFWIVIPELVYIMRQEPNFVTFVRNLAEILIIGIAVPQASIALVHRPLI

EETYSEIQSSLETVSTDPYRDIQRVIRKLKTIFKGYIGGEVALAGPYFLSIPVTIILKYFITGALPPLRGVFEADYLFDY

QANWLWFITVVINLTSMSYVILFLVSSHCISWSLLHKVSGLLKIISLKIGRLNEFVEDVREELTEIIELHEVAYRSARAL

EKSLNVFMLMLYGMCILNLCVTMVSLSLPNNDRDLLLKMLVILVYILFHIFVYSMLGTELMYSSTLVAEAFYGTHWYMRS

VSEQRIILFALTRSQKMVMLTTGKYFPVNRTTFGMALRTALSYFAVLRQV

>AaOR31

NGRDREKFLRVQLLCLALIGIKRHETVSSRTIFHVCFISMVIMDLATILFALEHANDIALVCDCLGPTFTAYLGIVKQYC

LSAHRVELWNIIETLRRLKDYAGTSEIESIERNNKIDRFLATAYLMSASATGSLFIIAALAKGCYKLIFQNIIEWGFPLS

LSFPFKTSHPIVFGVFFVWSSAAIYIVVFCSVSSDASFGGLASNVVVHFKLLQKRLQDATDENLKQLIEYHSLLLNLSRK

IMSSFRVIIINNLLVASVLLCVLGFQLVMFLGSTLMLIYLMYVTAIVIQITFFAYYGSLLSHESEEVSSSIYCSNWYEAS

PKTRRILLQCLMRAQVPVNTKAGFMVASLPTLRAILNSAGSYVALLLSF

>AaOR34

FIPSQRVAFWIWKILGIWATDDESPFYRAYRRIYHFFFTGIYLFSMFTSSFFTENSEELWVEILFILPTEIAMLTKTIIT

VYKFETIHRLLQTTISKEFQPTCPKHGKEYDRFFDRFSKVMLMYYFCSVCAWTHLGFLFDDRLKLPFFNWFFWDRDHLNN

YYILFAYQMIGMMGHCSLNVSGDMNIAYLLSIAGQQLDLLSCKFASLIQHFAREIERTVSWCVFAQICASGITICAIVFR

LSAIIDHLGTSIPMFFYMVSMLTQIFLPCYFGNDVTLKSQKLTNALYTSKWYQLAMNDRKDLKMMTLRTSESIRLKAGGF

FNFNLEAFTSTLNTAYSVYAVLNS

>AaOR35C

GELISSVRVILWIYRILGLSRENNQSVRYRIYRWVLNIPFLFAYLFAIISALHEENSEILWKDTIFIILTEASMFVKVVT

TYYRFQDTFQLLQTSVSEEFSPRCPCEQERHRRVLRHLNGALMGYLTVSVITACSTAIHIFEGMHKLPTFSWFPYGPDHG

LNYLLIASYQVSGMVMHCALNVSGDIQITYLLAIAGIQLDFLKRRFEDLKEDYLVHLQRRNNRHMQLVEQFVQDIE

>AaOR36

LDDESDYFRQMEWAMVVAGIKLPSQNSTYQRLFDCYRIIMLLQFSIWVVYVAYTEWNSPGELIGVVAFCLALVMILSRAV

LMRVYLKDLLKVRDGLKNRLNNHHSEGRVRSYRLIRRFFMVLEWIYLVDIILYAFGINEERQYSVPDNLRRLSKRSKLGF

DIFICSNHFIFSSVYASILTIMNTVFMGFSTELENIVSECDGIFEIYEESSTNIEMRKNLLFWSALKNEISLIAERYSVL

TEHVATVRNLLKVSFLLIFYTEIVFIGCALFYVKLIGITMNTVIVVSYVAAILLECYWFCRLADNINDTNIAIGFALYNL

DWPIQYLEIRASLLVMMTRSQQNLGITCGGMFEMSAQAFDELMKMIYSCLMFLLSI

>AaOR37

GELISSVRVILWIYRILGLSRENNQSVRYRIYRWVLNIPFLFAYLFAIISALHEENSEILWKDTIFIILTEASMFVKVVT

TYCRFQDTFQLLQTSVSEEFSPRCPCEQERHRRVLRHLNGALMGYLTVSVITACSTAIHIFEGMHKLPTFSWFPYGPDHG

LNYLLIASYQVSGMVMHCALNVSGDIQITYLLAIAGIQLDFLKRRFEDLKEDLIHHNRHMQFVQDIERVYSPATFTQFCV

SAITICATAFRISSIRENAGVAIGMMMYLLSMTVEIYLPCYYGNEITRKSQRLTNALYSCEWYRFDSETRRTVKMLMIRT

NKPMMLKAGRFFQYSLDTFGTTLNSAYSLFAVLQN

>AaOR38P

PFQLQKRLFRVLGYYPGDERLVHWGMLLVLFFHYWSQVMLIYIGEGDLQPALEGICPTPSRFGGILKCCILIWKRKQLKQ

LLDTLKGWFDREEPREKKINQWATYWGYQFTYWELMFTHLTCVFYCLLPVAAMLFHFVKQPDEPRIYILPFKLPFDYCKS

PVFEITYIIMCYIAYPPIFMMAGGDGLFIGVCLLISSQYRIVQRELEALGQSTAEENDHIFEQLKLIARHNRTIDTTEEM

SRLFLQNVFASFTIAAIKIGIACITVMKAEGLNKLIFVWYSLGILTEIYLYSYGGTQLMEESEKLSRTAYDFPWYRYRKN

VRQIIQMMMLRAQKPSRVDVPFFEASVVTFSTILRTAGSYVALMQT

>AaOR42

VKENPYESFERILYWQHLVLKVMGVDGFGPNFRRSALTYFIVFLANLFFVISLIDLVLFRQDVFNFTFVAVTIFYALIGL

GRLAVLVRHLVAPTVLVQSMKTVYQDAVRDPMETVVLQKYTNQLRQCVIFYTVIFMGGVVLTALLPLPIYWWSGDKILPF

GVVLPFDPESSDGYQLNYMYQVSCMLWTPPGLIASQNFYFALSFAIGIQYDVLVLKLKALDKNSEIRIKLIKVVRYQQRL

VQFISNLEDLYSYQTFLEVACNAMQIVMTLFVLHIEFWFPGILIMLVSTFQLFLSCMLGTMNDVKSDLFIQEVYDISWHA

MPKQEQKMLKFMLTKSQRMQKLSCGGMMAINMNLFLAVYKKIYSIFMMLQNL

>AaOR44

YSYEMYDHNLIYVRWLADVCGVDMMVENYKINYRTVATLFLISFTVVNLGYSCWFHYPNWHVIMELMMVFSFCLQGANKF

YNAYIHRHFFILMYGRLRNLHYKYQHHRENNAQLLLLMQRIHLISKGIFVLFGLGACSYFIYPVFNYWRYRRLELLISVR

LPIDADSHYGYIITMAYQIFMMVAAVFGMAAADSAILLFVCSLAGFVDVFKNELRELDMMLVQNPRDESKIRRKVREICV

QHFTVIEYESDLDERYFTTCFVQVVSTTIGLSGALFLAYIVRYIPGFALMLVLTAQLLEFCLLGTVLYVKNEEITEAIYG

TSWHLMEKPQQRCFALMLHKSQNFVEMTVGGLAPLNMETFVAIMKSIYSYFTMLISF

>AaOR46

KTIIKLLHTFGLWSQPYQNHKPWKPLWIIILPTLFLVGPEVAFFIRNHSSFTKATRAAIESVELTNVAIMAMNHLIHRSA

LEQSYKELQLALKIMSYDSHIDVQAAKKIIRISSKVYITFQMIISIGYAMSIPMLTVYHFAKTGKLPPLYGIFEADFIFD

VTHNWAWLLILIVTIVTFLSICLVLAVINSLHWGLLLHVTGFFKIVYMKILRIDEISDKQSRQSRHKEMIDIVQLQELAY

RNARILEQSLNQIMLLQFGMCVVTVCLTMLTLTLANDDKDLLIKMGIILVFIFAHLLVYSLLGTELITASSSIADAMYGT

QWYEWTISEQRNVLFILLRSQRMTALTIGKFFYINRSTLGKILQTTYSHFTVLRQM

>AaOR47

IIKLLKFCGFWGRPYQKFSLWQPLCHIAVLVVCLLAPGVIFIVRNSSNFASAISAAIESMGFINTILLGTTMLYHRSALE

NAYGDIRIALRIGKSSSIVQRNIEFLEKSTNFLFKGYTVFQSVVGTGYALTIPSLTVVYYVQTGQWPPLHGIFEADFFFD

FTTNWLWVLVIAVGMFAMLCLISVLVIVSSFNWSFLHYIIGLFKLVHIRISRLNAFANPQSMELIEIVKLQELVYRCART

AEDTLNLFLLTQFGTCVVAICLTMMTLTLASNDQDLLIKMILMLAYILFNIFVYSMLGEELIATSTSLAEAAYGTQWYEW

SIPEQRNILFIVRRSQKTAALTTGKFFAVNRSTFAATLQAAYSNFTVLRQM

>AaOR50

MQFIIRLLQILGFWTQPFQKRSTAKPLGYMVLFFVWLLLPEIIFIVRQEPTFAIVARNAVECLLIANVILLIGSTIVHQS

KLEESYGNMRFALDTIASNVDSKSTVTHLGSSTDRYFKVYVGFEGIITLVYALANPVLTLTQYIQSGELPPLHAIIESDF

YFDFTSNWLWLLVVIVGGILLCFLSVDIVSINSLHWSLIHHATALFKIVGQRLSYLNTFSDEKSQSRDEKSQSRELTDII

KMHEIILRSVRLLEEVINIYMLVQFGTCIIMLCIALIVLILSIDDRDLLVKMVLMLSYVLSHIMLYSLLGTELISASDSV

ADAVYDVPWYQWTVSEQRKVLFVLGRSQRMTALTAGKFFYINRDSFGKTLQTTYSYFTVMKQM

>AaOR54P

SASLKLCKWLGLWHDANLDKPCWQTVFLILCLLFWYILPYMYIVRGEKMLQDLLKPILEVFSMSVIVLRCLIHMINRQSV

QECFADLQNAISKFKNSPYEQRILRHLLKSADYIVKFYVSIVFVQAGLYGFLSAALTTFKYCTSNEIIQLPMDADYVFDH

TVNYWIWLPVTIVSLIEYLMLGSISAQECLFWNLLHHISSLFKIVHLEIARLDQYKDPEFKERLAFIVSIHEVCFRSARC

MEKVLSPLLALXWYCACIQTCYLLFFISMVNDVVVVASMIFVLQYVVFLIFSFSMLGAELMEESARVSVAIYKTQWYNRM

AAERRLLLFMKMRADRPVGITAVKFFYVNRSTFAEAMKSAFSFFTIMQQF

>AaOR55

ASLKLCRWLGLWHDVNLDKPCWQTVFISFCLLFWYILPGCLYITRGGRMLQYLLKSILEVFSMCVIVLRCVVHMINRKTV

QNSFVELEDAISTFENSPYEVRQMLRHLKSADYLVKIYVSIVFIQASIYGLVPAILTTYRYCNSNETVQLPSAVMEADYV

FDHSTNWIWLLVTIISLLVEYLLLGTFSSQECLFWNLLHHVSSLFKVIRLEIARLDQYTDPKKQYTERLASIVSTHEVCY

RCARSLEIVLSPLLAVLYCTCIIQTCYLLFVISMIDDLVVIASMIFVLQYIVFLIFSFSMLGAELTEESALVSEAIYNSN

WYMRMPAERRLLLFMKMRADRPVGITAAKFFYVNRSTFAEAMKTAFSFFTIMQQF

>AaOR56

ASLKLCKWLGLWHEVNLSSPCWQTVFIMCSILFWFILPGCLYITRGEKTLRDLLKSILEVFAMSVIVSRLMVHMFNRKKL

QACFVDLREAISTFENYPHEQRILRHLLKSADYLVKIYVSIVFIQASVYGVVPAVLTTYQYCTSDEIIRLPMDADYIFDH

TTSWIWMLVTIVSLIVEYLMLGSVSAQECLFWNLLHHTSCLFKMVCLEIARLDQYTDPNQFRERLACIVPIHEVCFKCAR

CLENVLNPLLALWYCTCIVQTCYLLFAISMIDDIVVIASMMFVLQYTVFLIFSFSMLGAELMEESARVSEAVYNTHWYMR

KATESRLLLFIMMRTNRPVGIRAAKFFFVNRSTFADAMKTAFSYFTIMQR

>AaOR57P

SLKLCKWFGLWNESNLNRPCWQTVFVIFCLLFWYILPYLYIARGEKTLQTLLKPIIEIFAMSVITIRCIYHIFNRKNLQE

CFVDLQEAISTFKNCPYERRTLKHLHKSAHYVVKIYVSIVFIQASVYGVLPAALITYQYCTSDEMIQLPSAVMDADYVFD

HTVNWNWILVTFVSVIVQYLMLGSVSAQECLFWNLLHHVSCLFKIVRLEIARLDQYTDPNQFTERLASIVSTHEVCFKCA

RCLENVLNPLLALWYCTCIVQTCYLLFVVSMIDDMVVIASMIFVLQYTIFLIFSFSMLGAELMEESARVSDAVYNSNWYM

RMPAERRLLWFMKLRSDRPVGITAVKFFYVNRSTFAEAMKTAFSLFTIMRR

>AaOR63

SQDFFDEMINFLRVFLTFCGSDVLLMEKFRWNARTWLCFWTLVSFAITLFYTIVFRSDDIYAILDTLSYSGIAIQGAFKM

HGALSRVKLFQQKYLSLKALHARFSKEPDNNMALNKCVLTITYIFRFFLLIYAAGGLAFFVIPLYVLFVYQKVVLILHVE

IPFDPDVFSGYVITTAYQVMMIALAIAGILAADMAIMILVLHIVGIVDIFANKLKELDRMYDKQQIHEKVTEICVMHREI

IKYEEDLDECYHTTVFVQVLTSVACLSLALFVVYMTNDWTRAMFLAATFFQLLEFCILGTALTLKNDQARVAIYHTKWYL

LTTSDQRRMQFVLHRSQNAVEMTIGGVALLNMETFVAIIKTIYSYFTMLVT

>AaOR65

AIMAQGSAEELQNSFDIAKKTTYLVGINPFQADREITARFIFSALLMLAIYVFCCYTLWVLGSEWQTSLEVFLVLMLNFL

GTNKIWLGFMRVYDYYLLFVSTEEIYKKFDEDIRNRKMVRILVAMLKSMVFIYTLSGSLIFLLMTTVVLLTKEKVLLLRV

YIPFVDHTTPGYVITTAFHMLMIAFCVNGYLASDSVFVSTILPIVGYTNSLRKEIDNFNASDNFNASLDEIERNEEEITE

QLMRIVKLHQMIVEYECQAVKFFKDSNLVQVSLQAGSLLVLVFMGLILHYLPAMCATLAVLFELTLYCSLGTVITTKNNQ

MMVDIYAINWHLLPKSQRMLVVFMLHRAQNGKNLYVGNFAPMNMTTYVQILKTVYTFLAMLIT

>AaOR67

IMAQGSAEELQKSFDIAKKTTYLVGVNPFQADREITARFIFSALLMLAIYVFCCYTLWVLGSEWQTSLEVFQVLMLNFLG

TNKIWLGFMRVHDYYLLFVSTEEIYKKFDEDIRNRKMVRILVAMLKSMVVIYTLSGSLIFLLMTTVVLITKEKVLLLRVY

IPFVDHTTPGYVITTAFHMLMIAFCVNGYLASDSVFVSTILPIVGYTNSLRREIDNFNASNASLDEIERNEEEITEQLMR

IVKLHQMIVEYECQSVKFFKDSNLVQVSLQAGLLLVLVFMGLILHYLPAMCATLAVLFELTLYCSLGTVITTKNNQMMVD

IYAINWHLLPKSQRMLVVFMLHRAQNGKNLYVGNFAPMNMTTYVQILKTVYTFLAMLITM

>AaOR68P

GSYIMTQMVIYFWCNFWTVYKYRHDIIHVMEVLNCTGIAFQLSAKFFIAMNNKSKFRRLLQTIEDNLYTRYPDRSTEMVF

MFARKYHILLRILTVLYCSTLFVFAILSLYYSEGELIPLFMFEVSYVDWHTVWGYLLTNFVQNILQSENASQNKEKIIEL

WRECLVEYQTIIEYLIDIESFNGGMSLVLVFTGVFVMCDNLVLCALTDWYASYLFLIICFIQLTIYFAMGNAVELKSDAL

DVCVVNFPWRLLKINYQKEYLFLICRMQN

>AaOR70

IDILRLLSHPVGVWNPDQFLTFGSYSIVTMVIYFWCNFWTVFKYRHDIIHVMEVLNCAGIAFQLSVKFFIAMNNKFLIRG

LLQTIENNLYERYPTSSEGEIVFIFARKYNILLKMLAVLYCSTLLVFALYPLYIYYSEGKLIPLFMFSYVDWHTVWGYLL

TNFVQVVTYLMGLFGMILADGLLVLLVVHGLVYIEVFMIHLRDLAKMLQSENVENEEKIMELWRECLVEHQTIIEYFTDI

ETVNGGMCLILVFTGVFAICDNLVLCALTDWYASYLFLLICFVQLTIYFAVGNAVELKSDALDISVVNFPWHLLKIDNQK

EYLFLICQMQRPIILTVYGFSNLNLEAYMTILKALYQFAMMILNF

>AaOR71

MPFQLRCMELIGLIGPKGRFYRFVLAFGWGTFVILLPKSVLGSSELDAIIKGFAELLFEGNLFIAVASLVPKLPLVKRLL

HVLSEIFRQATHDKRDYALICEQNSKIDKFCKFYFIYCCFGPFVFCIPAMVTSYVRYFGTTNNNGSEHLRFELPMEQEFY

WPIRTNACYHLFLTLSLSAYCVCSYMSVIKVSTLLIMIKYCSLVYRLVAIRIRELGKDTDEDERTKMVKVKEVVEMHEKA

LEATDLVEKVINIPIAMQFMACILFWCMTMVYVSTNINFNLFNVMVLFWLSLIETYGYSYLGTELSEDAKAVGHAVYDLP

WYEDSAQLQRYYRLMIQRSQQNIGVTAAKFFIVGIEKFGKVVNLSYSYYLVLK

>AaOR76

VDMKTCVRRHQKLLEMVMLFRDKLKLYLLLQIYFFSITFCCVMLVIQLKTGDNQMFYTLINLTTALLCLLLFGLFCDYLD

LKVAEISDQVFGSKWSERDRSMKRNLLMILMRSQKKIKFTCGDIYAMSIVTCMNVINMCYSAFTLLMNM

>AaOR79

DVIRRSVERHQRLLELVNLFRAKIKPYFLIAMGLYLFLVTFSCFLLVVQEGDFQALQFNVFNAAISIVTIIMFGVICDMV

EDRVRRIGDQVYESEWPLKYRLQKSSLMMIIARSQKKVGFTCGDIYQMSTITSMQVLKLCYTAFTMLWN

>AaOR82P

YFWLADALCLISEQRPPQTKHFSIRVLWTTLSILHQYSCFAIQLIKCYQQSPLVKKKIAAIVNLMIPLGVAVRGMCLAYH

REAVLKLKKYINSKSCQREDVGSLELRSQQYRKVNRNLAVLHCVTTASSVLWXALTIGLHGDDVFKIPFKSSGWIGKTID

VVYALLLIPWCFTLWYSPTQFVPILSFFNTELQIVVAQFESLELTLEHRKQFWVDLDTAFKNAIFHHSSSISHFELLRKI

SGLNFFVFLCSSAVIITINIIPIIMNPSFEHLPILLCSIEYASETYLCCTMFESLRNENRRIARYVYGIDWLCARKSIKQ

NALILHQRINKDFAIKAGGMFPLTVEMFATMMKSVYSLLTLLLQ

>AaOR83P

YFWLADALCLISEQRPPQTKHLTIHVLWTALSVLHQYCCFVIQMIKCYQKSPDKEMFTSMVNLVFPFGVAVLRGICLAYH

REAVQKLKKYINSKTCQRDDAGSFELRRQQYQKVNRNLAAFHCITTANSVSWALTIGLHGDDVFKIPFKSSGWIGKTIDV

VYALLLIPWCFTLWYSPTQFVPILSFFNTELQIVVAQFESLISHFELLRKISGLNFFVFLCSSAVIITINIIPIIMNPSF

EHLPILLCSIEYASETYLCCTMFESLRNENRRIARYVYGIDWLCARKSIKQNALILHQRINKDFAIKAGGMFPLTVEMFA

TMMKSVYSLLTLLLQ

>AaOR87

FKNLQQHIPQKIMRHIELLEKIQDFKPLVELFFFVLYFHAMALIGTYIFVILQTDFSIGSAMILMALTAYFAKCYFWCWV

ISSFQEVNELIALNIMQLITHLKYIELRTMLMIMMIRTQSSAQFSCGGLSIISVELFGNLLNVSYSVITFLLNI

>AaOR98

SAFWNDLAADIYKIFKKHQAITGKIQLIRPLLEAGFFVLYYCFLLTIGALLYVVMEGGISASSSIIALGGIGGLFECYWW

TSMVDSMQETNELFGDCNFQLLAYDQIKKLQKSLMIFWTTTSNPPPIKCMGMVSISILSVVNLANNSYSMLTFLIEM

>AaOR109

YWNHLRILKNIIGKTFFFVHYFAIFSIGTSGYAIHNVGFNTLSAISLASTLIFLLEYYLLCLWIDKLQDVADSLGSTIYS

REYHSKYFGLRASLMITWINTQHAFSMDCMGLFKISTSSFTSLIDTAYTMLMFLTNV

>AaOR110

EYWNHLRILKNIIGKTFFFVHYFAIFSIGTSGYAIHNVGFNTLSAISLASTLIFLLEYYLLCLWIDKLQDVADSLGSTIY

SREYHSKYFGLRASLMITWINTQHAFSMDCMGLFKISTSSFTSLIDTAYTMLMFLTNV

>AaOR111

RKVRIFANSLFVFQIVVTILQMVHNNNNVEQIVLSITKLVGMIAICVKWSLLIFQTRQICTVLNFITSNQLDSGDEAYDE

LEYKEFNRSACTMMRIIYAMTIANAVLLLVPSAATKQALALPPPLSNYGKLVSCIVYLFSTQLLFLGTVPFLSNMACIGM

LIMGMRFKLKILAHRYMGMRFKLKILAHRYFRMNQPVVSSEKHFARMERDVKEVLNYQTEYRKHFETLKQFVEKAFFIAH

FYALYSLGTCFYLSHKTGFNVLSLTLISLSVAYILKYYLWCHLVESLQDVANSIGDLIYEHCVHTQYMGMKTSLIIIWMN

TKNGYAISCMGMLDISTKTFVSFLNAVYSVMMFLINV

>AaOR112P

RMDREVRTVVDQQMEYCRQLQTLKNLVEKSFFIVHYYSLYSIGSCFFVARELGLNVLTGVIYASVFAYLLKHYLWCHLVD

SLQDVADTIGDVIYSREYHAQYVQLKASLMIIWLNTIDGVSFQCMGMINISTATFVDLINIIYSVLTFLINL

>AaOR113

MDREVRIVLDQQMEYWRQLESLKRLVEKSFFIVHYYSLYSIGTCFFVARDLGVNVLAVAIYASAFAYLTKHYLWCHLVDS

LQDVADTIGDEIYSRKYHQQYVQMKSSLIIVWHNTINGVSMKCMEMIEITTVTFVEMINIVYSVLTFLINM

>AaOR114

FKILANRFRSILSQPFVINGTDWEKMNSELRDTLKQHLEFWCHFKALKKLVGETFFLVHYFSIMSIGALCYICQAIGVNF

LSFVVMATLAMFLMEYYMFCHFVDSFQDVANCIGEHIFQIAIHSHYIGFRTALMIIWMNTRREVSMDCMGLFNISTAAFL

HVLNIAYTVLTFLIQM

>AaOR115

LLLGMQANFKVLANRFHSILSQPFVINGTDWEMINHELKDTVKHHLEFWRHFKALKSLVGETFFLVHYFSIMSIGALCYI

CQDIGVNFLSFVVLATLAMFLVEYYMFCHFVDSFQDIANCIGEHIFQIAIHSHYIGFRTALMIIWLNTRRGVSMDCMGLF

NISTVAFLHVLNIAYTVLTFLIQM

>AaOR119

LLLLGGIHSESTTWAHERKIRIICRCLFAYHAVAFVLQLNDALYEEKKAALVVWELMKVIFIFVAYLKVVLVVQLKGSIA

TLRQFIRSNHICSGDIEYDELEQNKFNKIVRITIQVVFVLIIIDTLILSVPNFSNNDLLKLPHLLALTGMPSYILKILLT

SCLGISVIPKYFACTACVGAVLIGMRTRLRILAHRFEHISQQDFTSEEKCVNRDIQEALAQHLEYWSHLKAMKIMVSKTF

LKVHYFSIVAIGSLIYVCCAMGVNGVTFVIGAGTVSFLMEYYLLCHLVDLLQDEADSIGYHIYNSELRSEYVQLRTTLMI

VWINTRNGISLNCLGLFEITTFTFVTLIDAAYSVLMFLIKM

>AaOR123

LMIAGIPIESPTTNSWKRTIPRISNVISALQLALTIGQFDGNADVLVVAWYLLRILGLGSCFVKFLMLNHFAEKIREVQN

FIVNRPCDSGDNQHDASIREKFGKSVKRILVAIMTMVAMDMVVISIPSSQRTKLLGIPTYFLFGSFCYKSVQFLYGFFIP

LIWIPMYLSYPLVLGMLLTGLRTEMQILKHSFEHLGHQTKDKRHWLQLKHDIGGLLNQQLLLHRHLRTVRHLVGFGFFIA

YYFAVFFIGAILFITKMHDIAFVVFLISLFMVVLIECYCWCHLVDSLDDVADGIGQSIYELCAHSDFVAMRTSLMIIWMN

TCNSPQVKCLGIFRICSEKFVNLCNASYTVFTFFI

>AaOR124

WERPVRYLVHLISAYHSVVMILQAAHVVAERNDVMDTAFCLIKIVGMGSAYIKILLLTYHADSVDKVEHFIRSRPMSSGE

DQYDSTVRGKFLRSTLVMIRWVLGVLIVDEILFAFPNSQRNKLFKLPPAMSLGTGITGWLANFLFVNWMPLIWLSKYLCC

TTKLGVLLMGLRVEFKILTHKLEQITRQAKSIESVEDHVEDHCKFLKELRRIDSSFVGNGIFMIYYYALFFIGTMLYVTH

HQGFEFYSLTFASSVVVTLLECYWWCQLVDSFQDDAESMGNELYDICAHRTYVEMRTSLMIIWINARHSLAIDCVGIFSI

STAIFVQMLNTSYSVLMFLINM

>AaOR129P

LTANALYQQDWPGQYRSVKTMMLIVIMHSQRPFRFTCGGLYKMSVPVFTTMIETLYFAVTFMMR

>AaOR131P

VEDHCKFLKELRRIDSSFVGNGIFMIYYYALFFIGTMLYVTHHQGFEFYSLTFASSVVVTLLECYWWCQLVDSFQDDAES

MGNELYDICAHRTYVEMRTSLMIIWINARHSLAIDCVGIFSISTAIFVQMLNTSYSVLMFLINM

>AAR14938.1_seven_transmembrane_G_protein-coupled_receptor_[Anopheles_gambiae]

MQVQPTKYVGLVADLMPNIRLMQASGHFLFRYVTGPILIRKVYSWWTLAMVLIQFFAILGNLATNADDVNELTANTITTL

FFTHSVTKFIYFAVNSENFYRTLAIWNQTNTHPLFAESDARYHSIALAKMRKLLVLVMATTVLSVVAWVTITFFGESVKT

VLDKATNETYTVDIPRLPIKSWYPWNAMSGPAYIFSFIYQIYFLLFSMVQSNLADVMFCSWLLLACEQLQHLKGIMRSLM

ELSASLDTYRPNSSQLFRAISAGSKSELIINEEKDPDVKDFDLSGIYSSKADWGAQFRAPSTLQTFDENGRNGNPNGLTR

KQEMMVRSAIKYWVERHKHVVRLVSAIGDTYGPALLLHMLTSTIKLTLLAYQATKIDGVNVYGLTVIGYLCYALAQVFLF

CIFGNRLIEESSSVMEAAYSCHWYDGSEEAKTFVQIVCQQCQKAMTISGAKFFTVSLDLFASVLGAVVTYFMVLVQLK

>AAS49925.1_putative_chemosensory_receptor_2,_partial_[Mamestra_brassicae]

MTKVKAQGLVSDLMPNIKLMQAAGHFLFNYHSKNAGMSNLLRKVYASAHAILIIIHFACMGINMAQYSDEVNELTANTIT

VLFFTHTIIKLGFFALNAKSFYRTLAVWNQSNSHPLFTESDARYHQIALTKMRRLLYFICGMTCLSVVSWITLTFFGESV

RLITSKETNETLTEVVPRLPLKAWYPFDAMGGTMYIIAFAFQVYWLLFSMAIANLMDVMFCSWLIFACEQLQHLKAIMKP

LMELSASLDTYRPNTAELFRASSTEKSEKIPDTVDMDIRGIYSTQQDFGMTLRGAGGRLQNFGQQIPNPNGLSPKQEMLA

RSAIKYWVERHKHVVRLVASIGDTYGTALLFHMLVSTITLTLLAYQATKINGINVYAFSTIGYLSYTLGQVFHFCIFGNR

LIEESSSVMEAAYSCQWYDGSEEAKTFVQIVCQQCQKAMSISGAKFFTVSLDLFASV

>AAT71306.1_odorant_receptor_Or83b_[Drosophila_melanogaster]

MTTSMQPSKYTGLVADLMPNIRAMKYSGLFMHNFTGGSAFMKKVYSSVHLVFLLMQFTFILVNMALNAEEVNELSGNTIT

TLFFTHCITKFIYLAVNQKNFYRTLNIWNQVNTHPLFAESDARYHSIALAKMRKLFFLVMLTIVASATAWTTITFFGDSV

KMVVDHETNSSIPVEIPRLPIKSFYPWNASHGMFYMISFAFQIYYVLFSMIHSNLCDVMFCSWLIFACEQLQHLKGIMKP

LMELSASLDTYRPNSAALFRSLSANSKSELIHNEEKDPGTDMDMSGIYSSKADWGAQFRAPSTLQSFGGNGGGGNGLVNG

ANPNGLTKKQEMMVRSAIKYWVERHKHVVRLVAAIGDTYGAALLLHMLTSTIKLTLLAYQATKINGVNVYAFTVVGYLGY

ALAQVFHFCIFGNRLIEESSSVMEAAYSCHWYDGSEEAKTFVQIVCQQCQKAMSISGAKFFTVSLDLFASVLGAVVTYFM

VLVQLK

>AAW52583.1_putative_chemosensory_receptor_2_[Spodoptera_exigua]

MMTKVKAQGLVSDLMPNIKLMQAAGHFLFNYHSENGGMTGLLRKIYASTHAILITIHFACMGINMAQYSDEVNELTANTI

TVLFFTHTIIKLGFFALNSKSFYRTLAVWNQSNSHPLFTESDARYHQIALTKMRRLLYFICGMTVLSVVCWVALTFFGES

VRLITSKETNETLTEVAPRLPLKAWYPFNAMSGTMYIIAFAFQVYWLLFSMAIASLMDVMFCSWLIFACEQLQHLKAIMK

PLMELSASLDTYRPNTAELFRASSTEKSEKIPDTVDMDIRGIYSTQQDFGMTLRGAGGRLQNFGQQNNNPNGLTPKQEML

ARSAIKYWVERHKHVVRLVASIGDTYGTALLFHMLVSTITLTLLAYQATKINGINVYAFSTIGYLSYTLGQVFHFCIFGN

RLIEESSSVMEAAYSCQWYDGSEEAKTFVQIVCQQCQKAMSISGAKFFTVSLDLFASVLGAVVTYFMVLVQLK

>AAX14773.1_odorant_receptor_Or83b_[Helicoverpa_zea]

MTKVKAQGLVSDLMPNIKLMQMAGHFLFNYHSENAGMSNLLRKIYASTHAILIFIHYACMGINMAKYSDEVNELTANTIT

VLFFAHTIIKLAFFALNSKSFYRTLAVWNQSNSHPLFTESDARYHQIALTKMRRLLYFICGMTVLSVISWVTLTFFGESV

RMVTNKETNETLTEVVPRLPLKAWYPFNAMSGTMYIVAFAFQVYWLLFSMAIANLMDVMFCSWLIFACEQLQHLKAIMKP

LMELSASLDTYRPNTAELFRASSTEKSEKIPDTVDMDIRGIYSTQQDFGMTLRGAGGRLQNFGQQNPNPNGLTPKQEMLA

RSAIKYWVERHKHVVRLVASIGDTYGTALLFHMLVSTITLTLLAYQATKINGINVYAFSTIGYLSYTLGQVFHFCIFGNR

LIEESSSVMEAAYSCQWYDGSEEAKTFVQIVCQQCQKAMSISGAKFFTVSLDLFASVLGAVVTYFMVLVQLK

>ABB29301.1_putative_odorant_receptor_Or7_[Culex_quinquefasciatus]

MNVQPTKYQGLVADLMPNIRLMQGVGHFLFRYVTGPIFIRKLYSWWNLTMILLQFFSIAANLVMNTGDVNELTANTITTL

FFVHSVTKFVFFAVNAEGFYRTLGIWNNPNAHPLFAESDARYHSIALAKMRKLLVMVMTTTVLSVVAWITITFFGDSVKG

VLDKETNETYIVEIPRLPIKAWYPWDAMSGAGYVFSFIYQAYFLLFSMCQANLADVLFCSWLLFACEQLQHLKGIMRPLM

ELSASLDTYRPNSAALFRAISAGSKSKLILNEEKDPDSKDFDLSGIYSSKADWGAQFRAPSTLQTFENGMNGEKGNPNGL

TRKQEMMVRSAIKYWVERHKHVVRLVSAIGDTYGAALLLHMLTSTIKLTLLAYQATKIDGLNVYGLTVIGYLVYALAQVF

LFCIFGNRLIEESSSVMEAAYSCHWYDGSEEAKTFVQIVCQQCQKAMTISGAKFFTVSLDLFASVLGAVVTYFMVLVQLK

>ABM05966.1_olfactory_receptor_[Microplitis_mediator]

MMKTKHQGLVADLMPNIRLMQISGHFMFNYYGEGKKLMHKIYCSVHLFLILLQFGFVAINLVKEKEDVDDLTANTITILF

FLHTLIKIVYFAARSKLFYRTLAIWNNPNSHPLFAESNARYHSIALTKVRRLLFCVGAATVATTISWTTLTFFEDPHVER

LNKETNETYIEEIPRLLVRSWYPFDARHGVAHIGMLIYQIYWLFICTVDANSIDVLFCSWLLFACEQLQHLKAIMKPLME

LSATLDTVVPNSGELFKAGSADHLRDNDGVPAEPAMNGDNMLDMDLRGIYSNRQDFTATFRPTAGTQYNGGVGPNQLTKK

QEMLVRSAIKYWVERHKHIVRLVTAIGDAYGVALLFHMLITTITLTLLAYQATKVNGVNVYAASTIGYLLYSLGQVFLFC

IFGNRLIEESSSVMEAAYSCHWYDGSEEAKTFVQIVCQQCQKAMSISGAKFFTVSLDLFASVLGAVVTYFMVLVQLK

>ABU45983.2_odorant_receptor_Or83b_[Helicoverpa_assulta]

MMTKVKAQGLVSDLMPNIKLMQMAGHFLFNYHSENAGMSNLLRKIYASTHAILIVIHYACMGINMAKYSDEVNELTANTI

TVLFFAHTIIKLAFFALNSKSFYRTLAVWNQSNSHPLFTESDARYHQIALTKMRRLLYFICGMTVLSVISWVTLTFFGES

VRMVTNKETNETLTEVVPRLPLKAWYPFNAMSGTMYIVAFAFQVYWLLFSMAIANLMDVMFCSWLIFACEQLQHLKAIMK

PLMELSASLDTYRPNTAELFRASSTEKSEKIPDTVDMDIRGIYSTQQDFGMTLRGAGGRLQNFGQQNPNPNGLTPKQEML

ARSAIKYWVERHKHVVRLVASIGDTYGTALLFHMLVSTITLTLLAYQATKINGINVYAFSTIGYLSYTLGQVFHFCIFGN

RLIEESSSVMEAAYSCQWYDGSEEAKTFVQIVCQQCQKAMSISGAKFFTVSLDLFASVLGAVVTYFMVLVQLK

>ABY51615.1_candidate_odorant_receptor_2_[Apocrypta_bakeri]

MKFKHQGLVADLLPNIRVMQGVGHFMFNYYSEGKKFPHRIYCIVTLLLLLLQYGMMAVNLMMESDDVDDLTANTITMLFF

LHPIVKMIYFPVRSKIFYKTLAIWNNPNSHPLFAESNARFHALAITKMRRLLFCVAGATIFSVISWTGITFIEDSVKRIT

DPETNETTIIPIPRLMIRTFYPFNAMSGAGHVFALIYQFYYLVISMAVSNSLDVLFCSWLLFACEQLQHLKAIMKPLMEL

SATLDTVVPNSGELFKAGSADHLRESQGVQPSGNGDNVLDVDLRGIYSNRQDFTATFRPTAGTTFNGGVGPNGLTKKQEM

LVRSAIKYWVERHKHVVRLVTAVGDAYGVALLLHMLTTTITLTLLAYQATKVNGVNVYAATVIGYLLYTLGQVFLFCIFG

NRLIEESSSVMEAAYSCHWYDGSEEAKTFVQIVCQQCQKAMSISGAKFFTVSLDLFASVLGAVVTYFMVLVQLK

>ABY51616.1_candidate_odorant_receptor_2_[Philotrypesis_pilosa]

MKFKQQGLIADLLPNIRVMQGVGHFMFNYYSEGKKFPHKIYCIVTLLLLLMQYGMMAVNLMMESDDVDDLTANTITMLFF

LHPIVKIIYFLVRSKIFYKTLAIWNNPNSHPLFAESNARLHALAVTKMRRLLFCVAGATIFSVISWTGITFADESVKRII

DAETNETTVIPIPRLMIRTFYPFNAMSGAGHVFAFIYQFYYLIISMAVSNSLDVLFCSWLLFACEQLQHLKAIMKPLMEL

SATLDTVVPNSGELFKAGSADHLRESQGIQPSGNGDNVLDVDLRGIYSNRQDFTATFRPTAGTTFNGGVGPNGLTKKQEM

LVRSAIKYWVERHKHVVRLVTSVGDAYGVALLLHMLTTTITLTLLAYQATKVNGVNVYAATVIGYLLYTLGQVFLFCIFG

NRLIEESSSVMEAAYSCHWYDGSEEAKTFVQIVCQQCQKAMSISGAKFFTVSLDLFASVLGAVVTYFMVLVQLK

>ABY51617.1_candidate_odorant_receptor_2_[Philotrypesis_sp._BL-2007]

MKIKHQGLVADLLTNIRVMQGVGHFMFNYYSGGKKFPHKIYSVVTLLLLLIQYGMMAVNLMMESDDVDDLTANTITMLFF

LHPIVKMIYFLVRSKIFYKTLAIWNNPNSHPLFAESNARFHALAVTKMRRLLFCVAGATIFSVISWTGITFVDESVKRII

DAETNETTIIPIPRLMIRTFYPFNAMSGAGHVFAFIYQFYYLIISMAVSNSLDVLFCSWLLFACEQLQHLKAIMKPLMEL

SATLDTVVPNSGELFKAGSADHLRESQGIQPSGNGDNVLDVDLRGIYSNRQDFTATFRPTAGTTFNGGVGPNGLTKKQEM

LVRSAIKYWVERHKHVVRLVTSVGDAYGVALLLHMLTTTITLTLLAYQATKVNGVNVYAATVIGYLLYTLGQVFLFCIFG

NRLIEESSSVMEAAYSCHWYDGSEEAKTFVQIVCQQCQKAMSISGAKFFTVSLDLFASVLGAVVTYFMVLVQLK

>ACC86853.1_odorant_receptor_Or83b_[Bactrocera_dorsalis]

MQPSKYVGLVADLMPNIRLMKYSGLFMHNFTGGSGLFKKIYSSVHLVLVLVQFLLILVNLALNAEEVNELSGNTITVLFF

THSITKFIYLAVSQKNFYRTLNIWNQVNSHPLFAESDARYHAIALAKMRKLFTLVMLTTVASAVAWTTITFFGESVKFAF

EKETNSTITVEIPRLPIKSFYPWNAGAGMFYIISFAFQCYYLLFSMVHANLCDVLFCSWLIFACEQLQHLKGIMKPLMEL

SASLDTYRPNSAALFRSLSANSKSELINNEEKEPTDLDISGVYSSKADWGAQFRAPSTLQTFNGMNGTNPNGLTRKQEMM

VRSAIKYWVERHKHVVRLVAAIGDTYGGALLLHMLTSTIMLTLLAYQATKITGVNAYAFTTIGYLGYALAQVFHFCIFGN

RLIEESSSVMEAAYSCHWYDGSEEAKTFVQIVCQQCQKAMSISGAKFFTVSLDLFASVLGAVVTYFMVLVQLK

>ACD40044.1_odorant_receptor_[Phyllotreta_striolata]

MMKKVKVTGLVADLMPNIRLMQASGHFMFNYHADNSGVSHLLRKVYSCMHLVLVLIQYACILVNLALNPDDVNELTANTI

TVLFFTHCITKFVYFAVNVEFFYRTIAVWNQPNSHPLFVESDARYHCIGVMKMRRLLFLVNMSTSLDTIAWTTVTFFRES

VANLQDNDTENGTINAHVPRLPIKAFYPWNAESGITYYVTLFFQINCFYFSTEIRNKLVPRFCSWLIFACEQLHHLQAIM

KPLMELTASMDTYVPNSAILFRAPSATSHEQVIDNNQKNEELDLKGVYNTRQEMGANFRSGALQNFGQGGGGVGPNGLTK

KQELMVRSAIKYWVERHKHVVRLVTAIGDAYGVALLLHMLTSTVMLTLLAYQATKINGVDTYAATVIGYLVYALAQVFHF

FIFGNRLIEESSSVMEAAYSCHWYDGSEEAKTFVQIVCQQCQKAMSISGAKFFTISLDLFASVLGATVTYFMVLVQLK

>ACF21678.1_putative_odorant_receptor_Or83b_[Haematobia_irritans_irritans]

MTSMQPTKYVGLVADLMPNIRLMKYSGLFMHAFTGGSALLKNVYSSIHLVLIIIQFGLILVNMALNADEVNELSGNTITA

LFFTHSITKFVYLAVNQKNFYRTLNIWNQPNTHPLFAESDARYHSIALAKMRKLFFCVMLTTVLSAVAWITITFFGESVK

FANDKETNSTITVPIPRLPIKSFYPWDASHGMFYMISFGYQVYYLFFSMVHSNLCDVIFCSWLIFACEQLQHLKGIMKPL

MELSASLDTYRPNSAALFRSLSANSKSELINNEEKEPVNDLDMSGIYNTKADWGAQFRAPSTLQTFNGINGANPNGLTKK

QEMLVRSAIKYWVERHKHVVRLVVAIGDTYGAALLLHMLTSTIKLTLLAYQAIKITGVDVYAFTVIGYLGYALAQVFHFC

IFGNRLIEESSSVMEAAYSCHWYDGSEEAKTFVQIVCQQCQKAMWIWGAKFFTVSLDLFVSVLGAVVTYFMGLVQLK

>ACJ06648.1_G_protein_coupled_receptor_SlOR83b_[Spodoptera_littoralis]

MTKVKAQGLVSDLMPNIKLMQAAGHFLFNYHAENGGMSGLLRKIYASTHAILITIHFACMGINMAQYSDEVNELTANTIT

VLFFTHTIIKLGFFALNSKSFYRTLAVWNQSNSHPLFTESDARYHQIALTKMRRLLYFICGMTVLSVVSWVTLTFFGESV

RLITSKETNETLTEVAPRLPLKAWYPFNAMSGTTYIIAFAFQVYWLLFSMAIANLMDVMFCSWLIFACEQLQHLKAIMKP

LMELSASLDTYRPNTAELFRASSTEKSEKIPDTVDMDIRGIYSTQQDFGMTLRGAGGRLQAFGQQNNNPNGLTPKQEMLA

RSAIKYWVERHKHVVRLVASIGDTYGTALLFHMLVSTITLTLLAYQATKINGINVYAFSTIGYLSYTLGQVFHFCIFGNR

LIEESSSVMEAAYSCQWYDGSEEAKTFVQIVCQQCQKAMSISGAKFFTVSLDLFASVLGAVVTYFMVLVQLK

>ACJ12928.2_odorant_receptor_2_[Epiphyas_postvittana]

MGKVKTQGLVSDLMPNIKLMQTVGHFLFNYSDETGGMSMLLRKVYASTHAVLIVINFLCMAVNMAQYSDEVNELTANTIT

VLFFAHTVIKLLFFALNSKNFYRTLAVWNQSNSHPLFTESDARYHQLALNKMRRLLYFIGTVTVMAVVSWITITFFGESV

RLIADKESNDTLTEPAPRLPLKAWYPFNAMSGTMYIVAFVYQIYWLLFSMAIANLMDVMFCSWLIFACEQLQHLKAIMKP

LMELSASLDTYRPNSSELFRASSTEKSEKVPDPVDLDIRGIYSTQQDFGMMLRGAGGRLQNFNNPNPNNPNGLTQKQEML

ARSAIKYWVERHKHVVRLVASIGDTYGTALLFHMLVSTITLTLLAYQATKIDGLNVYAFSTIGYLSYTLGQVFHFCIFGN

RLIEESSSVMEAAYSCQWYDGSEEAKTFVQIVCQQCQKAMSISGAKFFTVSLDLFASVLGAVVTYFMVLVQLK

>ACT37280

KKDGLIKDLWPNIRLIQMSGLFISEYYEDYSLFRKIYSWITTIIIYSQFIFIVMFMVTKSYDSDQLAAGVVTTLFFTHSM

IKFMYFSTGTKSFYRTLSCWNNTSPHPLFAESHSRFHAKSLSRMRQLLIIVSIVTIFTTISWTTITFFGESVWKVPNPET

NQTMYVPVPRLMLHSWYPWDSSHGLGYIVAFVLQFYWIFITLSHSNLMELLFSSFLVHACEQLQHLKEILNPLIELSATL

DSSVHNPAEIFRASSAKNQSEYGTKGENETNRKGPNNLTSNQEVLVRSAIKYWVERHKHVVKYVSLITECYGSALLFHML

VSTVILTILAYQATKINGVNVFAFSTIGYLMYSFAQIFMFCIHGNELIEESSSVMEAAYGCHWYDGSEEAKTFVQIVCQQ

CQKPLIVSGAKFFNVSLDLFASVLGAVVTYFMVLVQLK

>ACT37280.1_atypical_seven-span_transmembrane_receptor_[Sitobion_avenae]

MGYKKDGLIKDLWPNIRLIQMSGLFISEYYEDYSGLAVLFRKIYSWITTIIIYSQFIFIVMFMVTKSYDSDQLAAGVVTT

LFFTHSMIKFMYFSTGTKSFYRTLSCWNNTSPHPLFAESHSRFHAKSLSRMRQLLIIVSIVTIFTTISWTTITFFGESVW

KVPNPETFNQTMYVPVPRLMLHSWYPWDSSHGLGYIVAFVLQFYWIFITLSHSNLMELLFSSFLVHACEQLQHLKEILNP

LIELSATLDSSVHNPAEIFRASSAKNQSINGIDRDYNGSFVNEITEYGTKGENETNRKGPNNLTSNQEVLVRSAIKYWVE

RHKHVVKYVSLITECYGSALLFHMLVSTVILTILAYQATKINGVNVFAFSTIGYLMYSFAQIFMFCIHGNELIEESSSVM

EAAYGCHWYDGSEEAKTFVQIVCQQCQKPLIVSGAKFFNVSLDLFASVLGAVVTYFMVLVQLK

>ACU31808.1_candidate_odorant_receptor_2_[Ceratosolen_cornutus]

MKFQHQGLVADLLPNIRVMQSVGHFVFNYYSEGKKFPHKIYCIVTLLLLLTQYALMAVNLMMESGDVDDLTANTITMLFF

LHPVVKLIYFPVRGKIFYKTLAIWNNPNSHPLFAESNARYHSLSITKMRRLLFCVAATTVFSVLAWTSITFMDESVKRII

DPETNETTVVPIPRLMIRTFYPWNAINGAGHVFSFIYQFYYLFFSMAISNSLDVLFCSWLLFACEQLQHLKAIMKPLMEL

SATLDTVVPNSGELFKAGSADHLRDTQGVQPSGNGDNVVDVDVRGIYSNRQDFTATFRPTAGTTFNGGVGPNGLSKKQEM

LVRSAIKYWVERHKHVVRLVTSVGDAYGVALLLHMLSTTITLTLLAYQATKVNGANVYAATVIGYLLYTLGQVFLFCVFG

NRLIEESSSVMEAAYSCHWYDGSEEAKTFVQIVCQQCQKAMSISGAKFFTVSLDLFASVLGAVVTYFMVWVQLK

>ADB89179.1_odorant_receptor_2_[Ostrinia_nubilalis]

MTKVKAQGLVSDLMPNIKLMQAAGHFLFNYHSDNSGMTTLLRKVYSSVHAFLIVINYLCMAANMAQYSEEVNELTANTIT

VLFFAHSVIKMLFFAVNSKSFYRTLAVWNQSNSHPLFTESDARYHQLALTKMRRLLYFICGVTVLAVMSWITITFFGESV

RMIANKETNETLTEPAPRLPLKTWYPFDAMSGTMYVVAFVYQVYWLFFSMAIANLMDVMFCSWLIFACEQLQHLKAIMKP

LMELSASLDTYRPNTAELFRASSTEKSEKMPDTVDMDIRGIYSTQQDFGMTLRGAGGRLQNFGQPNPNNPNGLTQKQEML

ARSAIKYWVERHKHVVRLVASIGDTYGTALLFHMLVSTITLTLLAYQATKINGINVYAFSTIGYLSYTLGQVFHFCIFGN

RLIEESSSVMEAAYSCQWYDGSEEAKTFVQIVCQQCQKAMSISGAKFFTVSLDLFASVLGAVVTYFMVLVQLK

>ADK97803.1_odorant_receptor_Or83b_[Bactrocera_cucurbitae]

MQPSKYVGLVADLMPNIRLMKYSGLFMHNFTGGSGLFKKIYSSVHLVLVLVQFLLILVNLALNSEEVNELSGNTITVLFF

THSITKFIYLAVSQKNFYRTLNIWNQVNSHPLFAESDARYHAIALAKMRKLFTLVMLTTVASAVAWTTITFFGESVKFAF

EKETNSTITVEIPRLPIKSFYPWNAGAGMFYIISFAFQCYYLLFSMVHANLCDVLFCSWLIFACEQLQHLKGIMKPLMEL

SASLDTYRPNSAALFRSLSANSKSELINNEEKEPTDLDISGVYSSKADWGAQFRAPSTLQTFNGMNGTNPNGLTRKQEMM

VRSAIKYWVERHKHVVRLVAAIGDTYGGALLLHMLTSTIMLTLLAYQATKITGVNVYAFTTIGYLGYALAQVFHFCIFGN

RLIEESSSVMEAAYSCHWYDGSEEAKTFVQIVCQQCQKAMSISGAKFFTVSLDLFASVLGAVVTYFMVLVQLK

>ADM35103.1_olfactory_receptor_Or83b_[Holotrichia_plumbea]

MMQFKPQGLVADLMPNINLMKFAGHFMLNYYSDNGGALHTLRLGFCFGHLFLMLVQFGFTFGNLVQQSDDVNDLAANTIT

VLFFTHCIVKFIYFGVRQKLFYRTLGIWNQSNSHPLFLESNNRYHQLALTKMRRLLIVVMIGTIGSWIAWTTITFFGDSV

HTTKDPNNENETITEEVPRLLIRAWYPWDAMAGIPYYISLVYQIYYVGFSMLHSNLLDSLFCSWLIFASEQLQHLKEIMK

PLMELSATLDTYVPKSADLFRAPSASSQDKLTESDYNARNEDAHMRAMYSTHQEMGVTYRSGQLQDFSSGGIGPNGLTKK

QELMVRSAIKYWVERHKHVVRLVTAIGDAYGIALLLHMLTSTITLTLLAYQATKIDGVNKYALTVLGYLFYALAQVFHFC

IFGNRLIEESSSVMEAAYSCHWYDGSEEAKTFVQIVCQQCQKAMSISGAKFFTISLDLFASVLGATVTHFMVLVQLK

>ADN88092.1_odorant_receptor,_partial_[Aldrichina_grahami]

IKLMKYSGLFMHAFTGGSPLLKKVYSSIHLVLILAQFVFILVNMALNADEVNELSGNTITALFFTHCVTKFIYLAVNQKN

FYRTLNIWNQVNSHPLFAESDARYHSIALAKMRKLFFLVMLTTVVSAVAWITITFFGESVKFAFDKETNSSITVEIPRLP

IKSFYPWDASQGLFYTISFALQGYYLLFSMVHSNLCDVLFCSWLIFACEQLQHLKGIMKPLMELSASLDTYRPNSAALFR

SLSANSKSELIINEEKEPPSDLDMTGIYSTKADWGAQFRAPTTLQTFNGVNGGNPNGLTKKQEMMVRSAIKYWVERHKHV

VRLVAAIGDTYGAALLLHMLTSTIKLTLLAYQATKITGVNVYAFTVIGYLGYALAQVFHFCIFGNRLIEESSSVMEAAYS

CHWYDGSEEAKTFVQIVCQQCQKAMSISGAKFFTVSLDLFASVLGAVVT

>ADQ13177.1_olfactory_receptor_OR83b_[Helicoverpa_armigera]

MMTKVKAQGLVSDLMPNIKLMQMAGHFLFNYHSENAGMSNLLRKIYASTHAILIFIHYACMGINMAKYSDEVNELTANTI

TVLFFAHTIIKLAFFALNSKSFYRTLAVWNQSNSHPLFTESDARYHQIALTKMRRLLYFICGMTVLSVISWVTLTFFGES

VRMVTNKETNETLTEVVPRLPLKAWYPFNAMSGTMYIVAFAFQVYWLLFSMAIANLMDVMFCSWLIFACEQLQHLKAIMK

PLMELSASLDTYRPNTAELFRASSTEKSEKIPDTVDMDIRGIYSTQQDFGMTLRGAGGRLQNFGQQNPNPNGLTPKQEML

ARSAIKYWVERHKHVVRLVASIGDTYGTALLFHMLVSTITLTLLAYQATKINGINVYAFSTIGYLSYTLGQVFHFCIFGN

RLIEESSSVMEAAYSCQWYDGSEEAKTFVQIVCQQCQKAMSISGAKFFTVSLDLFASVLGAVVTYFMVLIQLK

>ADT82677.1_putative_odorant_receptor_2_[Spodoptera_litura]

MMTKVKAQGLVSDLMPNIKLMQAAGHFLFNYHAENGGMTGLLRKIYASTHAILITIHFACLGINMAQYSDEVNELTANTI

TVLFSTHTIIKLGFFALNSKSFYRTLAVWNQSNSHPLFTESDARYHQIALTKMRRLLYFICGMTVLSAVSWVTLTFFGES

VRLITSKGTNETLTEVAPRLPLKAWYPFNAMSGTTYIIAFAFQVYWLLFSMAIANLMDVMFCPWLIFACEQLQHLKAIMK

PLMELSASPDTYRPNTAELFRASSTEKSEKIPDTVDMDIRGIYSTQQDFGMTLRGAGGRLQTFGQQNNNPNGLTPKQEML

ARSAIKYWVERHKHVVRLVASIGDTYGTALLFHMLVSTITLTLLAYQATKINGINVYAFSTIGYLSYTLGQVFHFCIFGN

RLIEESSSVMEAAYSCQWYDGSEEAKTFVQIVCQQCQKAMSISGAKFLTVSLDLFASVLGAVVTYFMVLVQLK

>AEA30004.2_odorant_receptor_co-receptor_[Chrysomya_megacephala]

MQANLQPTKYVGLVADLMPNIKLMKYSGLFMHAFTGGSALLKKVYSSIHLVLILMQFIFILVNMALNADEVNELSGNTIT

ALFFTHCVTKFIYLAVNQKNFYRTLNIWNQVNSHPLFAESDARYHSIALAKMRKLFFLVMLTTVASAVAWITITFFGESV

KFAFDKETNSSITVEIPRLPIKSFYPWDSSQGMFYIISFAFQGYYLLFSMVHSNLCDVLFCSWLIFACEQLQHLKGIMKP

LMELSASLDTYRPNSAALFRSLSANSKSELIINEEKEPPTDLDMSGIYSSKADWGAQFRAPTTLQTFNGVNGGNPNGLTK

KQEMMVRSAIKYWVERHKHVVRLVAAIGDTYGAALLLHMLTSTIKLTLLAYQATKITGVNVYAFTVIGYLGYALAQVFHF

CIFGNRLIEESSSVMEAAYSCHWYDGSEEAKTFVQIVCQQCQKAMSISGAKFFTVSLDLFASVLGAVVTYFMVLVQLK

>AEA30005.2_odorant_receptor_1_[Lucilia_sericata]

MQSNLQPTKYVGLVADLMPNIKLMKYSGLFMHAFTGGSPLLKKVYSSIHLVLILAQFMFILVNMALNADEVNELSGNTIT

ALFFTHCVTKFIYLAVNQKNFYRTLNIWNQVNTHPLFAESDARYHSIALAKMRKLFFLVMLTTVASAVAWITITFFGESV

KFAFDKETNSSITVEIPRLPIKSFYPWDASQGIFYTISFAFQGYYLLFSMVHSNLCDVLFCSWLIFACEQLQHLKGIMKP

LMELSASLDTYRPNSAALFRSLSANSKSELIINEEKEPPSDLDMTGIYSTKADWGAQFRAPTTLQTFNGVNGGNPNGLTK

KQEMMVRSAIKYWVERHKHVVRLVAAIGDTYGAALLLHMLTSTIKLTLLAYQATKITGVNVYAFTVIGYLGYALAQVFHF

CIFGNRLIEESSSVMEAAYSCHWYDGSEEAKTFVQIVCQQCQKAMSISGAKFFTVSLDLFASVLGAVVTYFMVLVQLK

>AEA76288.1_odorant_receptor_2_[Argyresthia_conjugella]

MMTKTKTQGLVSDMMPNIRLMRAGHFLFNYYNESGGMSMLLRKIYASFHAVMLVIHFMCMAMNMAKYSDDVNELTANTIT

VLFFAHSCIKVLYFALNAKSFYRTLAVWNQSNIHPLFTESDARYHQLALTKMRRLLYFICGITALAVISWVTLTFFGESV

RFITDKETNETLTEPAPRLPLKAWYPFDAMAGPMYIFAFVFQVYWLLLSMSVCNLMDVMFCSWLIFACEQLQHLKAIMKP

LMELSASLDTYRPNTAELFRAGSAEKQEKTPDPVALDIRGIYSTQQDFGMTLRGAGGRLQNFNQPVANNPNGLTQKQEML

ARSAIKYWVERHKHVVRLVASIGDTYGTALLFHMLVSTITLTLLAYQATKIDGLNVYAFSTIGYLSYTLGQVFHFCIFGN

RLIEESSSVMEAAYSCQWYDGSEEAKTFVQIVCQQCQKAMSISGAKFFTVSLDLFASVLGAVVTYFMVLVQLK

>AEE69033.1_olfactory_receptor_Or83b_[Holotrichia_oblita]

MMKFKPQGLVADLMPNIKLMKFAGHFMLNYYAENSGAVHTLRLGFCFGHLFLMLLQFGFTFGNLVQESDDVNDLAANTIT

VLFFTHCIVKFIYFGVRQKLFYRTLGIWNQSNSHPLFLESNNRYHQLALTKMRRLLIIVMIGTIGSWIAWTTITFFGDSV

HNRKDPNNENETITEEIPRLLIRSWYPWDAMSGIPYYVSLIYQIYYVGFSMLHSNLLDSLFCSWLIFACEQLQHLKEIMK

PLMELSATLDTYVPKSADLFRAHSASSQDKLTESDYNARNEDAHMRAMYSTHQEMGVTYRSGQLQEFSSGGIGPNALTKK

QELMVRSAIKYWVERHKHVVRLVTAIGDAYGIALLLHMLTSTITLTLLAYQATKIDGVNKYALTVLGYLFYALAQVFHFC

IFGNRLIEESSSVMEAAYSCHWYDGSEEVKTFVQIVCQQCQKAMSISGAKFFTISLDLFASVLGATVTYFMVLVQLK

>AEG88961.1_odorant_receptor_Or83b_[Holotrichia_parallela]

MMQFKPQGLVADLMPNIKLMKFAGHFMLNYYAENSGAVHTLRLGFCFGHLFLMLLQFGFTFGNLVQESDDVNDLAANTIT

ILFFTHCIVKFIYFGVRQKLFYRTLGIWNQSNSHPLFLESNNRYHQLALTKMRRLLIIVMVGTIGSWIAWTTITFLGDSV

HTRKDPSNENETITEEIPRLLVRSWYPWDAMSGIPYYITLVYQVYYVGFSMLHSNLLDSLFCSWLIFACEQLQHLKEIMK

PLMELSATLDTYVPKSADLFRAPSASSQDRLMDSDYNARNEDVHMKTMYSTHHEMGVTYRSGQLQDFSGGIGPNGLTKKQ

ELMVRSAIKYWVERHKHVVRLVTAIGDAYGIALLLHTSASTITLTLLAYQATKIDGVNKYALTVLGYLFYALTQVFHFCI

FGNRLIEESSSVMEAAYSCHWYDGSEEAKTFVQIVCQQCQKAMSISGAKFFTISLVLFASVLGATVTYFMVLVQLK

>AET06156.1_odorant_receptor_2,_partial_[Planotortrix_excessana]

MMGKVKTQGLVSDLMPNIKLMQAVGHFLFNYTDENGGMSMLLRKIYASTHAVLIVVNFLCMAVNMAQYSDEVNELTANTI

TVLFFAHTVIKLLFFALNSKNFYRTLAVWNQSNSHPLFTESDARYHQLALNKMRRLLYFIGSVTIMAVVSWITITFFGES

VRLIADKESNDTLTEPAPRLPLKTWYPFNAMSGTMYIVAFVYQIYWLLFSMAIANLMDVMFCSWLIFACEQLQHLKAIMK

PLMELSASLDTYRPNTSELFRASSTEKSEKVPEPVDMDIRGIYSTQQDFGMLLRGAGGRLQNFNNPNPNNPNGLTQKQEM

LARSAIKYWVERHKHVVRLVASIGDTYGTALLFHMLVSTITLTLLAYQATKIDGLNVYAFSTVGYLSYTLGQVFHFCIFG

NRLIEESSSVMEAAYSCQWYDGSEEAKTFVQIVCQQCQKAMSISGAKFFTVSLDLFASVLGAVVTYFMVLVQ

>AET06159.1_odorant_receptor_2,_partial_[Planotortrix_notophaea]

MMGKVKTQGLVSDLMPNIKLMQAVGHFLFNYTDENGGMSMLLRKVYASTHAVLIVVNFLCMAVNMAQYSDEVNELTANTI

TVLFFAHTVIKLLFFALNSKNFYRTLAVWNQSNSHPLFTESDARYHQLALNKMRRLLYFIGTVTIMAVVSWITVTFFGES

VRLIADKESNDTLTEPAPRLPLKTWYPFNAMSGTMYIVAFVYQIYWLLFSMAIANLMDVMFCSWLIFACEQLQHLKAIMK

PLMELSASLDTYRPNTSELFRASSTEKSEKVPEPVDMDIRGIYSTQQDFGMMLRGAGGRLQNFNNPNPNNPNGLTQKQEM

LARSAIKYWVERHKHVVRLVASIGDTYGTALLFRMLVSTITLTLLAYQATKIDGLNVYAFSTVGYLSYTLGQVFHFCIFG

NRLIEESSSVMEAAYPCQWYDGSEEAKTFVQIVCQQCQKAMSISGAKFFTVSLDLFASVLGAVVTYFMVLVQ

>AEX28371.1_olfactory_co-receptor,_partial_[Schistocerca_gregaria]

MQKPHGLVADLWPLIRMVQYSGHWMLEYSGGLTALRAIYSSVVSVLVVTQFALMAVNPIQRSGDVNELAANTITVLFFLH

PITKFAYFAVRSKAFYRTLATWNQSNNHPLFAESQARFHQLSVVRMRRLVMYVVSVTALSVVSWTSITFMGDSTREVADP

DNANETITEEVPRLMISTWYPFDASSGMGYMLAFVYQLYWLTATLMHSNLMDVMFCCWLIYACEQLVYLKEIMKPLMELS

ATLDTVVPHTSELFRAASTLPTNEPLYGMGPDMSNGVTDGMTIRGIYSSQRDFSGFNRRSAALSTVREADSGGAVTSAGG

IGPNGLSKRQEMLVRSAIKYWVERHKHVVRFVGNIGDAYGAALLLHMLTTTVTLTLLAYQATKIDSVDVYAASVLGYLFY

TLGQVFLFCVFGNRLIEESSSVMEAAYSCHWYDGSEEAKTFVQIVCQQCQ

>AFC91712.1_putative_odorant_receptor_co-receptor_ORco_[Cydia_pomonella]

MMGKVKSQGLVSDLMPNIKLMQMSGHFLFNYTEETGGMSLLLRKIYAAMHAFLILLNFVCMGINMAQYSEEVNELTANTI

TVLFFAHTIIKLAFFAINSKSFYRTLAVWNQSNSHPLFTESDARYHQLSLDKSRRLLYFICGTTCLSVVSWVTLTFFGES

VRLIADKESNDTLTEPAPRLPLKAWYPFDTMSGSMYIMAFVYQIYWLLFSMLIANLLDVMFCSWLIFACEQLQHLKAIMK

PLMELSAALDTYRPNTAELFRASSTEKSEKVPEPTDIDIRGIYSTQQDFGMMLRGAGGRLQNFNSTNPNPNGLTQKQEML

ARSAIKYWVERHKHVVRLVASIGDTYGTALLFHMLVSTITLTLLAYQATKIDGLNVYAFSTVGYLRYTLGQVFHFCIFGN

RLIEESSSVMEAAYSCQWYDGSEEAKTFVQIVCQQCQKAMSISGAKFFTVSLDLFASVLGAVVTYFMVLVQLK

>AFH96943.1_odorant_receptor_co-receptor_[Chrysomya_rufifacies]

MQANLQPTKYVGLVADLMPNIRLMKYSGLFMHAFTGGSALLKKVYSSIHLMLILIQFIFILVNMALNADEVNELSGNTIT

ALFFTHCVTKFIYLAVNQKNFYRTLNIWNQVNSHPLFAESDARYHSIALAKMRKLFFLVMLTTVASAVAWITITFFGESV

KFAFDKETNSSITVEIPRLPIKSFYPWDASQGMFYIISFAFQGYYLLFSMLHSNLCDVLFCSWLIFACEQLQHLKGIMKP

LMELSASLDTYRPNSAALFRSLSANSKSELIINEEKEPPTDLDMSGIYSTKADWGAQFRAPTTLQTFNGGNPNGLTKKQE

MMVRSAIKYWVERHKHVVRLVAAIGDTYGAALLLHMLTSTIKLTLLAYQATKITGVNVYAFSVIGYLGYALAQVFHFCIF

GNRLIEESSSVMEAAYSCHWYDGSEEAKTFVQIVCQQCQKAMSISGAKFFTVSLDLFASVLGAVVTYFMVLVQLK

>AFH96944.1_odorant_receptor_co-receptor_[Musca_domestica]

MQANLQPTKYTGLVADLMPNIKLMKYSGLFMHAFTGGSALLKNVYSSIHLVLIVLQFIFILVNMALNADEVNELSGNTIT

ALFFTHCITKFVYLAVNQKNFYRTLNIWNQPNSHPLFAESDARYHSIALAKMRKLFFLVMLTTVASAVAWITITFFGESV

KFATDKETNSTITVPIPRLPIKSFYPWDASSGMFYMISFGYQAYYLLFSMVHSNLCDVLFCSWLIFACEQLQHLKGIMKP

LMELSASLDTYRPNSAALFRSLSANSKSELIQNEEKEPVNDLDMSGIYSTKADWGAQFRAPSTLQTFNGINGGNPNGLTK

KQEMMVRSAIKYWVERHKHVVRLVAAIGDTYGAALLLHMLTSTIKLTLLAYQATKITGVNVYAFTVIGYLGYALAQVFHF

CIFGNRLIEESSSVMEAAYSCHWYDGSEEAKTFVQIVCQQCQKAMSISGAKFFTVSLDLFASVLGAVVTYFMVLVQLK

>AFI25169.1_odorant_receptor_83b_[Heliothis_viriplaca]

MMTKVKAQGLVSDLMPNIKLMQMAGHFLFNYHSENAGMSNLLRKIYASTHAILIFIHYACMGINMAKYSDEVNELTANTI

TVLFFAHTIIKLAFFALNSKSFYRTLAVWNQSNSHPLFTESDARYHQIALTKMRRLLYFICGMTVLSVISWVTLTFFGES

VRMVTNKETNETLTEVVPRLPLKAWYPFNAMSGTMYIVAFAFQVYWLLFSMAIANLMDVMFCSWLIFACEQLQHLKAIMK

PLMELSASLDTYRPNTAELFRASSTEKSEKIPDTVDMDIRGIYSTQQDFGMTLRGAGGRLQNFGQQNNNPNGLTPKQEML

ARSAIKYWVERHKHVVRLVASIGDTYGTALLFHMLVSTITLTLLAYQATKINGINVYAFSTIGYLSYTLGQVFHFCIFGN

RLIEESSSVMEAAYSCQWYDGSEEAKTFVQIVCQQCQKAMSISGAKFFTVSLDLFASVLGAVVTYFMVLVQLK

>AFP54145.1_odorant_receptor_coreceptor_[Amyelois_transitella]

MINNKVKAQGLVSDLMPNIKLMQASGHFLFNYYSDNSGMSMLLRKIYSSVHAILIVINYVCMVVNMAQYSDEVNELTANT

ITVLFFAHTVIKLLFFALNSKSFYRTLAVWNQSNSHPLFTESDSRHHQLALTKIRRLLYFICSMTVFSVVSWVTLTFFGE

SVRLIANKETNETISEPAPRLPLKTWYPFDAMGGSMYIIAFAFQVYWLFFSMITANLMDVMFCSWLIFACEQLQHLKAIM

KPLMELSASLDTYRPNTAELFRVSSTEKSEKVPDPVDMDIRGIYATQQDFGMTLRGAGGRLQTFGQQNNNPNGLSQKQEM

LARSAIKYWVERHKHVVRLVTSIGDTYGTALLFHMLISTITLTLLAYQATKIDGINVYAFSTIGYLSYTLGQVFHFCVFG

NQLIEESSSVMEAAYSCQWYDGSEEAKTFVQIVCQQCQKAMSISGAKFFTVSLDLFASVLGAVVTYFMVLVQLK

>AFQ94048.1_olfactory_receptor_2_[Chilo_suppressalis]

MMAKVKAQGLVSDLMPNIKLMQAAGHFLFNYHSDNSGMSTLLRKIYSSVHAILIVINFLCMAVNMAQYSDEVNELTANTI

TVLFFTHTVIKLLFFAVNSKSFYRTLAVWNQSNSHPLFTESDARYHQLALTKMRRLLYFICTVTVLSVVSWVTITFFGES

VRLIANKETNETLTEPAPRLPLKAWYPFDAMSGTMYIIAFAYQVYWLLFSMAIANLMDVMFCSWLIFACEQLQHLKAIMK

PLMELSASLDTYRPNTAELFRASSTEKSEKVPDPVDLDIRGIYSTQQDFGMTLRGGGGRLQTFGQQNTNNPNGLSQKQEM

LARSAIKYWVERHKHVVRLVASIGDTYGTALLFHMLVSTITLTLLAYQATKIGGINVYAFSTVGYLSYTLGQVFHFCIFG

NRLIEESSSVMEAAYSCQWYDGSEEAKTFVQIVCQQCQKAMSISGAKFFTVSLDLFASVLGAVVTYFMVLVQLK

>AFX73447.1_olfactory_co-receptor_[Lygus_hesperus]_

MQKVKMHGLVGDLWPNIRLMQLTGHWLLEYHEETGGMARLLRLAYCWMTTFSVYIQYAFLVCFLILETYNADEMAAVTIT

TLFFLHSVTKFTYFAFRSKYFYRTLGAWNQVNSHPLFAESNARHRATALSRMRKLLMVIGCVTILAVFSWTTVTFLDDPV

WDKTDPDNVNETISVEVPQLMVYAWYPWDAKYGMTYFMTFAFQLYWLFITLAHSNLLDVLFCCFVIFACEQLKHLKEILQ

PLMELSAALDSVVPNSGDLFKAGSAGSDVALIGNGENGNGNDFDVRGIYSSQRDFSGFQGGITNGGTVGPNGLTKRQELL

VRSAIKYWVERHKHVVKFVSSIGDTYGSALLLHMLTSTVTLTLLAYQATKIEGVDVYAASTIGYLVYTLGQVFVFCIHGN

ELIEESSSVMEAAYSCHWYDGSEEAKTFVQIVCQQCQKSLTVSGAKFFTVSLDLFASVFGAVVTYFMVLVQLK

>AFX73448.1_olfactory_co-receptor_[Lygus_lineolaris]

MQKVKMHGLVGDLWPNIRLMQLTGHWLLEYHEETGGMARLLRLAYCWMTTFSVYIQYAFLVCFLILETYNADEMAAVTIT

TLFFLHSVTKFTYFAFRSKYFYRTLGAWNQVNSHPLFAESNARHRATALSRMRKLLMVIGCVTILAVFSWTTVTFLDDPV

WDKTDPDNVNETISVEVPQLMVYAWYPWDAKYGMTYFMTFAFQLYWLFITLAHSNLLDVLFCCFVIFACEQLKHLKEILQ

PLMELSAALDSVVPNSGDLFKAGSAGSDVALIGNGENGNGNDFDVRGIYSSQRDFSGFQGGITNGGTVGPNGLTKRQELL

VRSAIKYWVERHKHVVKFVSSIGDTYGSALLLHMLTSTVTLTLLAYQATKIEGVDVYAASTIGYLVYTLGQVFVFCIHGN

ELIEESSSVMEAAYSCHWYDGSEEAKTFVQIVCQQCQKSLTVSGAKFFTVSLDLFASVFGAVVTYFMVLVQLK

>AGF29886.1_odorant_co-receptor_[Conogethes_punctiferalis]

MMNKVKALGLVSDLMPNIKLMQAAGHFLFNYHSDNSGMAMLLRKIYASVHAFLIVIHYLCMAVNMAQYSEEVNELTANTI

TVLFFAHSVIKLLFFALNSKSFYRTLAVWNQSNSHPLFTESDARYHQLSLTKMRRLLYFICGVTVLAVVCWVTITFFGES

VRMIANKETNETLTEPAPRLPLKAWYPFDAMGGTMYVVAFVFQVYFLFFSMAIANLMDVMFCSWLIFACEQLQHLKAIMK

PLMELSASLDTYRPNTAELFRASSTEKSEKVPDPVDMDIRGIYSTQQDFGMTLRGAGGRLQNFGGNPTNNPNGLTQKHEM

LARSAIKYWVERHKHVVRLVASIGDTYGTALLFHMLVSTITLTLLAYQATKINGINVYAFSTIGYLSYTLGQVFHFCIFG

NRLIEESSSVMEAAYSCQWYDGSEEAKTFVQIVCQQCQKAMSISGAKFFTVSLDLFASVLGAVVTYFMVLVQLK

>AGG91643.1_odorant_receptor_[Ostrinia_furnacalis]

MMTKVKAQGLVSDLMPNIKLMQAAGHFLFNYHSDNSGMTTLLRKVYSSVHAFLIVINYLCMAANMAQYSEEVNELTANTI

TVLFFAHSVIKMLFFAVNSKSFYRTLAVWNQSNSHPLFTESDARYHQLALTKMRRLLYFICGVTVLAVLSWITITFFGES

VRMIANKETNETLTEPAPRLPLKTWYPFDAMSGTMYVVAFVYQVYWLFFSMAIANLMDVMFCSWLIFACEQLQHLKAIMK

PLMELSASLDTYRPNTAELFRASSTEKSEKMPDTVDMDIRGIYSTQQDFGMTLRGAGGRLQNFGQPNPNNPNGLTQKQEM

LARSAIKYWVERHKHVVRLVASIGDTYGTALLFHMLVSTITLTLLAYQATKINGINVYAFSTIGYLSYTLGQVFHFCIFG

NRLIEESSSVMEAAYSCQWYDGSEEAKTFVQIVCQQCQKAMSISGAKFFTVSLDLFASVLGAVVTYFMVLVQLK

>AGI62937.2_olfactory_coreceptor_[Macrocentrus_cingulum]

MKFKQQGLVADLMPNIRLMQISGHFMFNYYSDGKKFMHKIYCSVHLFLILLQFALCGLNLAMEADDVDQLTANTVTVLFF

LHAIVKIGYFGVRSKLFYRTLAIWNNPNSHPLFAESNARYHSIALTKMRRLLFCVGAATVLTIIAWTGITFAENPVRILT

DKVTNETTTIELPRLMVRSWYPFNAKSGMAHIGMLIFQFYWLTITMVDCNSLDVLFCSWLLFACEQLQHLKAIMKPLMEL

SATLDTVVPNSSELFKAGSADHLRDTAGSVPSATQPNGESMLDLDLRGIYSNRQDFTATFRPTAGTQFTGGVGPNGLTKK

QEMLVRSAIKYWVERHKHVVRLVTAIGDAYGVALLFHMLITTISLTLLAYQATKVNGLNVYAATTIGYFSYALAQVFLFC

IFGNRLIEESSSVMEAAYSCHWYDGSEEAKTFVQIVCQQCQKAMSISGAKFFTVSLDLFASVLGAVVTYFMVLEQLK

>AGS41440.1_odorant_receptor_co-receptor_[Agrotis_segetum]

MMTKVKAQGLGSDLLPNIKLMQAAGHFLFNYHSENAGMSNLLRKIYASTHAILITIHFGCMAVNMAQYSDEVNELTANTI

TVLFFTHTIIKLSFFALNSKSFYRTLAVWNQSNSHPLFTESDARYHQIALTKMRRLLYFICGMTCLSVVFWITLTFFGES

VRLITNKETNETLTEPVPRLPLKAWYPFNAMSGTMYIVAFAFQVYWLLFSMAIANLMDVMFCSWLIFACEQLQHLKAIMK

PLMELSASLDTYRPNTAELFRASSTEKSEKIPDAVDMDIRGIYSTQQDFGMTLRGAGGRLQNFGQQNSNPNGLTPKQEML

ARSAIKYWVERHKHVVRLVASIGDTYGTALLFHMLVSTITLTLLAYQATKINGINVYAFSTIGYLSYTLGQVFHFCIFGN

RLIEESSSVMEAAYSCQWYDGSEEAKTFVQIVCQQCQKAMSISGAKFFTVSLDLFASVLGAVVTYFMVLVQLK

>AGY14565.1_putative_odorant_receptor_[Sesamia_inferens]

MMTKVKAQGLVSDLMPNIKLMQAAGHFLFNYHSENAGMSNLLRKIYASVHAILICINFACMGINMAQYSDEVNELTANTI

TVLFFTHTIIKLAFFALNSKSFYRTMAVWNQSNSHPLFTESDARYHQIALTKMRKLLYFICGMTCLSVVSWVTLTFFGES

VRLITSKETNETLTEVAPRLPLKAWYPFNAMGGTTYIIAFAFQVYWLLFAMAIANLMDVMFCSWLIFACEQLQHLKAIMK

PLMELSASLDTYRPNTAELFRASSTEKSEKIPDTVDMDIRSIYSTQQDFGMTLRGAGGRLQNFGQQNSNPNGLTPKQEML

ARSAIKYWVERHKHVVRLVASIGDTYGTALLFHMLVSTITLTLLAYQATKINGINVYAFSTIGYLCYTLGQVFHFCIFGN

RLIEESSSVMEAAYSCQWYDGSEEAKTFVQIVCQQCQKAMSISGAKFFTVSLDLFASVLGAVVTYFMVLVQLK

>AHA50096.1_odorant_receptor_OrCO,_partial_[Lymantria_dispar_dispar]

MSTLLRKIYAGIHTFLILLNFICLGINAAQYADEVNELTANTITVLFFTHTLIRLLFFAMNSKNFYRTLAVWNQSNSHPL

FTESDARYHQISLTKMRRLLYFICGMTVLSVISWVTITFFGESVRLITSKETNETLTEPAPRLPLKAWYPFNAMSGTMYV

LAFIFQIYWLLFSMAIPNLMDVMFCSWLIFACEQLQHLKAIMKPLMELSASLDTYRPNTAELFRASSTEKSERVQDPTDL

DIRGIYSTQQDFGMTIRGTGGRLQNFNQGGNNPNGLSKNQEMLARSAIKYWVERHKHVVRLVASIGDTYGTTLLFHMLVS

TITLTLLAYQATKINRIDVYAFSTVGYLGYTLGQVFHFCIFGNRLIEESSSVMEAAYSCQWYDGSEEAKTFVQIVCQQCQ

KAMSISGAKFFTVSLDLFASVLGAVVTYFMVLVQLK

>AHA50097.1_odorant_receptor_OrCO,_partial_[Lymantria_dispar_asiatica]

MSTLLRKIYAGIHTFLILLNFICLGINAAQYADEVNELTANTITVLFFTHTLIKLLFFAMNSKNFYRTLAVWNQSNSHPL

FTESDARYHQISLTKMRRLLYFICGMTVLSVISWVTITFFGESVRLITSKETNETLTEPAPRLPLKAWYPFNAMSGTMYV

LAFIFQIYWLLFSMAIPNLMDVMFCSWLIFACEQLQHLKAIMKPLMELSASLDTYRPNTAELFRASSTEKSERAQDPTDL

DIRGIYSTQQDFGMTIRGTGGRLQNFNQGGNNPNGLSKNQEMLARSAIKYWVERHKHVVRLVASIGDTYGTTLLFHMLVS

TITLTLLAYQATKINRIDVYAFSTVGYLGYTLGQVFHFCIFGNRLIEESSSVMEAAYSCQWYDGSEEAKTFVQIVCQQCQ

KAMSISGAKFFTVSLDLFASVLGAVVTYFMVLVQLK

>AHC72290.1_olfactory_co-receptor_protein_[Apolygus_lucorum]

MQKVKMHGLVGDLWPNIRLMQLTGHWLLEYHEENGGMLRLLRMAYCWMTTFSIYIQYAFLVCFLILETYNADEMAAVTIT

TLFFLHSVTKFTYFAFRSSYFYRTLGAWNQVNSHPLFAESNARHRATALSRMRKLLMIIGTVTILAVFGWTTVTFLDEPV

WDKTDPDNVNETISVEIPQLMVYAWYPWDARYGMTYFMTFVFQLYWLFITLAHSNLLDVLFCCFVIFACEQLKHLKEILQ

PLMELSAALDSVVPNSGDLFKAGSAGSDIALIGNGENGNDFDVRGIYSSQRDFSGFQGGVVNGGTVGPNGLTKRQELLVR

SAIKYWVERHKHVVKFVSSIGDTYGSALLLHMLTSTVTLTLLAYQATKIEAVDVYAASTIGYLVYTLGQVFVFCIHGNEL

IEESSSVMEAAYSCHWYDGSEEAKTFVQIVCQQCQKSLTVSGAKFFTVSLDLFASVFGAVVTYFMVLVQLK

>AHC72291.1_olfactory_co-receptor_protein_[Lygus_pratensis]

MQKVKMHGLVGDLWPNIRLMQLTGHWLLEYHEETGGMARLIRIAYCWMTTFIVYLQYAFLVCFLTLETYNSDEMAAVTIT

TLFFLHSVTKFTYFAIRSKYFYRTLSAWNQVNSHPLFAESNARHRAAALSRMRKLLMIIGVVTILAVFGWTTVTFLDDPV

WDKTDPDNVNETISVEIPQLMVYAWYPWDAKTGMTYFMTFALQLYWLFITLAHSNLLDVLFCCFVIFSCEQLKHLKEILQ

PLMELSAALDSVVPNSGDLFKSGSAGSNIALISNGDGGNDFDVRGIYSSQRDFSGFQGGMTNGTTVGPNGLTKRQELLVR

SAIKYWVERHRHVVKFVTSIGDTYGTALLLHMLTSTVTLTLLAYQATKIEGVDVYASTTIGYLVYTLGQVFVFCIHGNEL

IEESSSVMEAAYSCHWYDGSEEAKTFVQIVCQQCQKSLTVSGAKFFTVSLDLFASVFGAVVTYFMVLVQLK

>AHC72292.1_olfactory_co-receptor_protein_[Adelphocoris_suturalis]

MQKVKMHGLVGDLWPNIRLMQLTGHWLLEYHEETGGMARLIRIAYCWMTTFIVYLQYAFLVCFLILETYNSDEMAAVTIT

TLFFLHSVTKFTYFAIRSKYFYRTLSAWNQVNSHPLFAESNARHRAAALSRMRKLLMIIGVVTILAVFGWTTVTFLDDPV

WDKTDPDNVNETISVEIPQLMVYAWYPWDAKTGMTYFMTFALQLYWLFITLAHSNLLDVLFCCFVIFSCEQLKHLKEILQ

PLMELSAALDSVVPNSGDLFKSGSAGSNIALISNGDGGNDFDVRGIYSSQRDFSGFQGGMTNGTTVGPNGLTKRQELLVR

SAIKYWVERHRHVVKFVTSIGDTYGTALLLHMLTSTVTLTLLAYQATKIEGVDVYASTTIGYLVYTLGQVFVFCIHGNEL

IEESSSVMEAAYSCHWYDGSEEAKTFVQIVCQQCQKSLTVSGAKFFTVSLDLFASVSLNKTHITILLY

>AHC72294.1_olfactory_co-receptor_protein_[Adelphocoris_fasciaticollis]

MQKVKMHGLVGDLWPNIRLMQLTGHWLLEYHEETGGMARLIRIAYCWMTTFVVYLQYAFLVCFLILETYNSDEMAAVTIT

TLFFLHSVTKFTYFAIRSKYFYRTLSAWNQVNSHPLFAESNARHRAAALSRMRKLLMIIGVVTILAVFGWTTVTFLDDPV

WDKTDPDNVNETISVEIPQLMVYAWYPWDAKTGMTYFMTFAPQLYWLFITLAHSNLLDVLFCCFVIFSCEQLKHLKEILQ

PLMELSAALDSVVPNSGDLFKSGSAGSNVALISNGDGGNDFDVRGIYSSQRDFSGFQGGMTNGTTVGPNGLTKRQELLVR

SAIKYWVERHRHVVKFVTSIGDTYGTALLLHMLTSTVTLTLLAYQATKIEGVDVYASTTIGYLVYTLGQVFVFCIHGNEL

IEESSSVMEAAYSCHWYDGSEEAKTFVQIVCQQCQKSLTVSGAKFFTVSLDLFASVSLNKTHITILLY

>AHJ37468.1_olfactory_receptor_2_[Apis_mellifera]

MKFKQQQGLIADLMPNINLMKATGHFMFNYYTDSSTKHIHKIYCIVHLVLILMQFGFCGINLMMESEDVDDLTANTITML

FFTHSVVKLVYFAVRSKLFYRTLGIWNNPNSHPLFAESNARYHQIAVKKMRILLLAVIGTTVLSAISWTTITFIGDSVKK

VIDPVTNETTYVEIPRLMVRSWYPYDPSHGMAHILTLIFQFYWLIFCMADANLLDVLFCSWLLFACEQIQHLKNIMKPLM

EFSATLDTVVPNSGELFKAGSAEQPKEQEPLPPVTPPQGENMLDMDLRGIYSNRTDFTTTFRPTAGMTFNGGVGPNGLTK

KQEMLVRSAIKYWVERHKHIVRLVTAIGDAYGVALLLHMLTTTITLTLLAYQATKIHAVDTYAASVVGYLLYSLGQVFML

CIFGNRLIEESSSVMEAAYSCHWYDGSEEAKTFVQIVCQQCQKAMSISGAKFFTVSLDLFASVLGAMVTYFMVLVQLK

>AHL20247.1_odorant_co-receptor_[Aedes_albopictus]

MHVQPTKYHGLVLDLMPNIRLMQGFGHFLFRYVSGPVLIRKLYSWWNLIMILLQYFAIMGNLVMNTGDVNELTANTITTL

FFTHSVTKFIYVAVNSEHFYRTLGIWNQPNSHSLFAESDARYHSIALAKMRKLLVMVMVTTVLSVVAWITITFFGDSVKN

VFDKETNETYTVEIPRLPIKALYPWDAMSGVPYFFSFVYQAYFLLFSMCQANLADVMFCSWLLFTCEQLQHLKGIMRPLM

ELSASLDTYRPNSAALFRAASAGSKAELILNEEKDPDTKDFDLNGIYNSKADWGAQFRAPSTLQTFNDNNGMNGNPNGLT

KKQELMVRSAIKYWVERHKHVVRLVSAIGETYGAALLLHMLTSTIKLTLLAYQATKIDALNVYGLTVIGYLVYALAQVFL

FCIFGNRLIEESSSVMEAAYSCHWYDGSEEAKTFVQIVCQQCQKAMTISGAKFFTVSLDLFASVLGAVVTYFMVLVQLK

>AID61201.1_odorant_receptor,_partial_[Calliphora_stygia]

MQSNLQPTKYVGLVADLMPNIKLMKYSGLFMHAFTGGSPLLKKVYSSIHLVLILAQFIFILVNMALNADEVNELSGNTIT

ALFFTHCVTKFIYLAVNQKNFYRTLNIWNQVNSHPLFAESDARYHSIALAKMRKLFFLVMLTTVASAVAWITITFFGESV

KFAFDKETNSSITVEIPRLPIKSFYPWDASQGMFYTISFALQGYYLLFSMVHSNLCDVLFCSWLIFACEQLQHLKGIMKP

LMELSASLDTYRPNSAALFRSLSANSKSELIINEEKEPPSDLDMTGIYSTKADWGAQFRAPTTLQTFNGVNGGNPNGLTK

KQEMMVRSAIKYWVERHKHVVRLVAAIGDTYGAALLLHMLTSTIKLTLLAYQATKITGVNVYAFTVIGYLGYALAQVFHF

CIFGNRLIEESSSVMEAAYSCHWYDGSEEAKTFVQIVCQQCQKAMSISGAKFFTVSLDLFASVLGAVVTYFMVLVQLK

>AII01046.1_odorant_receptor_[Dendrolimus_houi]

MMNKTKTQGLVSDLMPCIKLMQAVGHFLFNYYDENTGMSALIRKIYAGTHAFIIVVHFLFMGINMAKYSDEVNELTANTI

TMLFFTHSLIKLLFFALNSKSFYRTLAAWNQCNSHPLFVESDARYHQLALSRMRRLLYFVGGMTIVSVMSWVILTFFGES

VRYITSKETNETLTEPAPRLPLKAWYPFNAMSGSMYVIAFVLQIYWLLFAMSIANLMDVMFCSWLIFACEQLQHLKAIMQ

PLMELSAALDTYRPNTAELFRVNSGGEKVPDATDLDIRGIYSTQQDFGMAIRGAGGRLQNFAQQKANPNGLSQKQEMLAR

SAIKYWVERHKHVVRLVTSIGDTYGVALLFHMLVSTITLTLLAYQATKINGVNVYAFSTIGYLSYTLGQVFHFCIFGNRL

IEESSSVMEAAYSCQWYDGSEEAKTFVQIVCQQCQKAMSISGAKFFTVSLDLFASVLGAVVTYFMVLVQLK

>AII01079.1_odorant_receptor_[Dendrolimus_kikuchii]

MMTKVKTQGLVSDLMPCIKLMQIVGHFLFNYYDENTGMSALVRKIYAGTHAFIIVVHFLFMGINMAKYSDEVNELTANTI

TMLFFTHSLIKLLFFALNSKSFYRTLAAWNQCNSHPLFVESDARYHQLALSRMRRLLYFVGGMTIVSVISWVILTFFGES

VRYITSKETNETLTEPAPRLPLKAWYPFNAMSGSMYVIAFVLQIYWLLFAMSIANLMDVMFCSWLIFACEQLQHLKAIMQ

PLMELSAALDTYRPNTAELFRVNSGGEKVADATDLDIRGIYSTQQDFGMAIRGAGGRLQNFAQQKANPNGLSQKQEMLAR

SAIKYWVERHKHVVRLVTSIGDTYGVALLFHMLVSTITLTLLAYQATKINGVNVYAFSTIGYLSYTLGQVFHFCIFGNRL

IEESSSVMEAAYSCQWYDGSEEAKTFVQIVCQQCQKAMSISGAKFFTVSLDLFASVLGAVVTYFMVLVQLK

>AII15784.1_odorant_receptor_co-receptor_[Sitotroga_cerealella]

MMTKVKTQGLVTDLMPNINLMRMFGHFVFNYLPESNGMSMLLRKIYASVHAVLIFVHFVCVGVNMAKYADEVNELTANTI

TVLFFTHTIIKLNYFAINSRSFYRTLAVWNQSNSHPLFTESDARYHQLAVSKNRKLLYFICSTTCISVVCWVTITFFGES

VYLIMDKETNETMTTPAPRLPLKAWYPFNAMSGTMYIVMFGFQVYWLLFSMMLANLMDVLFCSWLVFACEQLQHLKAIIK

PLMELSASLDTYRPNTAELFRASSTEKSEKVPDPVDLDIRGIYSTQQDFGVTLRGAGGRLQNFGQNPPNPNGLTQKQEML

VRSAIKYWVERHKHVVRLVSSIGDTYGTALLFHMLVSTITLTLLAYQATKIDGLNVYAFSTIGYLSYTLGQVFHFCIFGN

RLIEESSSVMGAAYSCQWYDGSEEAKTFVQIVCQQCQKAMSISGAKFFTVSLDLFASVLGAVVTYFMVLVQLK

>AIO10777.1_odorant_receptor_co-receptor_[Anopheles_funestus]

MQVQPTKYVGLVADLMPNIRLMQASGHFLFRYVTGPILIRKVYSRWTLIMVLMQFFAILGNLASNADDVNELTANTITTL

FFTHSVTKFIYFAVNSENFYRTLGIWNQTNSHPLFAESDARYHSIALAKMRKLLVLVMATTILSVVAWVTITFFGESVQN

VFDKETNETYKVVIPRLPIKSWYPWNAMSGPAYIFSFIYQIYFLLFSMVQSNLADVMFCSWLLLACEQLQHLKGIMRPLM

ELSASLDTYRPNSAALFRAISAGSKSELIINEEKDPDVKDFDLSGIYSSKADWGAQFRAPSTLQTFDENGRNGNPNGLTR

KQEMMVRSAIKYWVERHKHVVRLVSAIGDTYGPALLLHMLTSTIKLTLLAYQATKIDGVNVYGLTVIGYLCYALAQVFLF

CIFGNRLIEESSSVMEAAYSCHWYDGSEEAKTFVQIVCQQCQKAMTISGAKFFTVSLDLFASVLGAVVTYFMVLVQLK

>AIT69913.1_olfactory_receptor_co_[Ctenopseustis_herana]

MMGKVKTQGLVSDLMPNIKLMQAVGHFLFNYTDESGGMSMLLRKIYASTHAVLIVVNFLCMAVNMAQYSDEVNELTANTI

TVLFFAHTVIKLLFFALNAKNFYRTLAVWNQSNSHPLFTESDARYHQLSLNKMRRLLYFIGSVTIAAVVCWITITFFGES

VRLIADKESNDTLTEPAPRLPLKAWYPFNAMSGTMYIVAFVYQIYWLLFSMAIANLMDIMFCSWLIFACEQLQHLKAIMK

PLMELSASLDTYRPNTSELFRASSTEKSEKVPEPVDMDIRGIYSTQQDFGMMLRGAGGRLQNFNNSNPGNPNGLTQKQEM

LARSAIKYWVERHKHVVRLVASIGDTFGTALLFHMLVSTITLTLLAYQATKIDGLNVYAFSTVGXLSYTLGQVFHFCIFG

NRLIEESSSVMEAAYSCQWYDGSEEAKTFVQIVCQQCQKAMSISGAKFFTVSLDLFASVLGAVVTYFMVLVQLK

>AIT72022.1_olfactory_receptor_[Ctenopseustis_obliquana]

MMGKVKTQGLVSDLMPNIKLMQAVGHFLFNYTDESGGMSMLLRKIYASTHAVLIVVNFLCMAVNMAQYSDEVNELTANTI

TVLFFAHTVIKLLFFALNAKNFYRTLAVWNQSNSHPLFTESDARYHQLSLNKMRRLLYFIGSVTIAAVVCWITITFFGES

VRLIADKESNDTLTEPAPRLPLKAWYPFNAMSGTMYIVAFVYQIYWLLFSMAIANLMDIMFCSWLIFACEQLQHLKAIMK

PLMELSASLDTYRPNTSELFRASSTEKSEKVPEPVDMDIRGIYSTQQDFGMMLRGAGGRLQNFNNSNPSNPNGLTQKQEM

LARSAIKYWVERHKHVVRLVASIGDTFGTALLFHMLVSTITLTLLAYQATKIDGLNVYAFSTVGYLSYTLGQVFHFCIFG

NRLIEESSSVMEAAYSCQWYDGSEEAKTFVQIVCQQCQKAMSISGAKFFTVSLDLFASVLGAVVTYFMVLVQLK

>AIX97092.1_olfactory_receptor_1_[Monochamus_alternatus]

MFNYHADNSGALHTLRLLYSCMHLVFCLFQFGCIFGNLVVEKDDVNYLAANTITVLFFTHCITKFVYFALRSKLFYRTLG

IWNQSNSHPLFVESNNRYHALSLKKMRTLLICVSATTVLSAAAWTGITFVEESVHNIKDPNNENETITEEIPRLLIKSWY

PWDAMSGMAYYGSLVFQIYYVLFSLTHANLMDSLFCSWLIFACEQLQHLKEIMKPLMELSASLDTYVPKSADLFRAPSAK

SQDNYIESDYNTKNEELNLKGIYNTRQELGGNFRSGALQTFGQGGVGPNGLTKKQELMVRSAIKYWVERHKHVVRLVTAI

GDAYGVALLLHMLTSTVMLTLLAYQATKINGVNTYAATTIGYLVYSLAQVFHFCIFGNRLIEESSSVMEAAYSCHWYDGS

EEAKTFVQIVCQQCQKAMQISGAKFFTISLDLFASVLGAVVTYFMVLVQLK

>AIX97139.1_olfactory_receptor_4_[Rhyzopertha_dominica]

MMTFKVQGLVADLMPNIRLMQAVGHFMLNYHADNSGALHTLRLSYCFMHLFLLLTQYGFIFGNLVKERGDVNDLAANTIT

VLFFMHCITKFVYFAIRSKLFYRTLGIWNQSNSHPLFVESNNRFHAIALTKMRRVTIVVVAGTLVSAISWISVTFVGDSV

HHIKDPNNVNETITEEIPRLPIKTWYPWNAMSGMPYYLSLGYQIYYVLFSMFHANLLDVLFCCWLIFACEQLMHLKEIMK

PLMELSASLDTYHPKSADLFRAVSANSQDNLVDHDYNTKNSEEDLKGYYNTHQELGGHFRSGALQMFGQGGGGIGPNGLT

KKQELLVRSAIKYWVERHKHVVRLVTAIGDAYGIALLLHMLTSTIMLTLLAYQATKIDGVNTYAASVLGYLFYALAQVFL

FCIFGNRLIEESSSVMEAAYSCHWYDGSEEAKTFVQIVCQQCQKAMSISGAKFFTISLDLFASVLGAVVTYFMVLVQLK

>AIY24336.1_odorant_receptor_Or83b_[Chouioia_cunea]

MMKMKQVGLVADLMPNIRITQAVGHWLFNYYSEGMRFPHKIYCMVTLFLMLFQFGTMALNLVKESDDVDQLTANTITVLF

FMHPIVKVVYLAARAKIFYKCLGVWNNPNSHPLFAESNQRYHALALSKMRKLLFCVCGAVTFSVICWTGITFFDDAVRKI

HDKETNETTIIPLPRLMIRSAYPWNAMSGAAHIFSMIYQFYYLVITMGICNMFDVLFCSFLLFACEQLQHLKAIMKPLME

LSATLDTVVPNSGELFKAGSADHLRESSGIQPSSNGENVLDVDVRGIYSNRQDFTATFRPTAGTTFNGGVGPNGLTKKQE

MLVRSAIKYWVERHKHVVRLVTVVGDAYGVALLLHMLTTTITLTLLAYQATKVNGVNVYAATTIGYLLYTLGQVFLFCVF

GNRLIEESSSVMEAAYSCHWYDGSEEAKTFVQIVCQQCQKAMSISGAKFFTVSLDLFASVLGAVVTYFMVLVQLK

>AJF20962.1_olfactory_coreceptor_[Operophtera_brumata]

MMTKVKYQGLVSDLLPNIKLMQAAGHFLFNYHDENAGMSTILRKIYSSTHAFLISIHFVLMVINMAKYADEVNELTANTI

TILFFLHTIIKMLFFAVNSKSFYRTLAVWNQSNSHPLFTESDARYHQLALTKMRRLLYFICVMTVVSLICWVTLTFFGES

VRLISSKDTNETLTEEIPRLPLKAWYPFDAMSGSMYIFAFVFQIYWLLFSTSISNFMDVMFCSWLIFACEQLQHLKGIMK

PLMELSASLDTYRPNTAELFRASSTEKSEKVPDPVDIDIRGIYSTQQDFGMTLRGAGGRLQTFGENNNPNNLTQKQEMLA

RSAIKYWVERHKHVVRLVGSIGDTYGTALLFHMLVSTITLTLLAYQATKINGVNVYAFSTLGYLCYTLGQVFHFCIFGNR

LIEESSSVMEAAYSCQWYDGSEEAKTFVQIVCQQCQKALSISGAKFFTVSLDLFASVLGAVVTYFMVLVQLK

>AJF23826.1_olfactory_receptor_Orco_[Planotortrix_octo]

MMGKVKTQGLVSDLMPNIKLMQAVGHFLFNYTDENGGMSMLLRKIYASTHAVLIVVNFLCMAVNMAQYSDEVNELTANTI

TVLFFAHTVIKLLFFAMNSKNFYRTLAVWNQSNSHPLFTESDARYHQLALNKMRRLLYFIGSVTIMAVVSWITITFFGES

VRLIADKESNDTLTEPAPRLPLKTWYPFNAMSGTMYIVAFVYQIYWLLFSMAIANLMDVMFCSWLIFACEQLQHLKAIMK

PLMELSASLDTYRPNTSELFRASSTEKSEKVPEPVDMDIRGIYSTQQDFGMLLRGAGGRLQNFNNPNPNNPNGLTQKQEM

LARSAIKYWVERHKHVVRLVASIGDTYGTALLFHMLVSTITLTLLAYQATKIDGLNVYAFSTVGYLSYTLGQVFHFCIFG

NRLIEESSSVMEAAYSCQWYDGSEEAKTFVQIVCQQCQKAMSISGAKFFTVSLDLFASVLGAVVTYFMVLVQLK

>AJF94638.2_odorant_receptor_co-receptor_[Ambrostoma_quadriimpressum]

MMKFKVSGLVADLMPNIRLIQASGHFMFNYHADNSGALHALRLGYSCLHLVLCLVQFGCTFGNLVIERNDVNDLAANTIT

VLFFTHCITKFVYFAVRSKLFYRTLGIWNKANSHPLFLDSNNRYHALSLKKMRTLLICVMTTTILSASAWTAITFVGDSV

HNVKDPDNDNETITEEIPRLLIKSWYPWNAMSGTAYYVSVSFQIYYVFFSLAHSNLMDSLFCSWLIFACEQLQHLKEIMK

PLMELSASLDTYVPKSADLFRAPSANSQDNLIENEYNEKNEGLNLKGVYNTRQEMGANFRSGALQTFGQGGGGVGPNGLS

KKQELMVRSAIKYWVERHKHVVRLVTAIGDAYGVALLLHMLTATVMLTLLAYQATKIDGVNKYAATVIGYLVYSLAQVFH

FCIFGNRLIEESSSVMEAAYSCHWYDGSEEAKTFAQIICQQCQKALSISGAKFFTISLDLFASVLGAVVTYFMVLVQLK

>AJO62219.1_olfactory_co-receptor_ORco_[Tenebrio_molitor]

MMKFKVSGLVADLMPNIRLIQASGHFMLNYHADNSGAVHTLRLGYCIMHLIFMLLQYGCNFVNLIFERGDVNDLAANTIT

VLFFTHCITKFVYFAARSKLFYRTLGIWNQPNSHPLFVESNNRYHALALKKMRRLLYIIIIWTSFSAIAWTSITFVGDSV

HNIKDPDNENMTITEEIPRLLVKAWYPWNAMSGMPYYITLVFQVYYVFFALSHANLLDSLFCSWLIFACEQLQHLKEIMK

PLMELSASLDTYVPKSADLFRAPSATSQDNLIENDYNTKNEDLKGVYSTRQELGGHFRGGALQNFGGVGGGVGPNGLTKK

QELMVRSAIKYWVERHKHVVRLVTAIGDAYGVALLLHMLTSTIMLTLLAYQATKITGVDKYAATVIGYLLFALAQVFHFC

IFGNRLIEESSSVMEAAYSCHWYDGSEEAKTFVQIVCQQCQKAMSISGAKFFTISLDLFASVLGAVVTYFMVLVQLK

>AKC58535.1_odorant_co-receptor_[Anomala_corpulenta]

MMQFKPQGLVADLIPNIKLMQFSGHFMLNYYAETTGAVHTLRLGFCFGHLFLLLLQFGFTFGNLVQQSDDVNDLAANTIT

VLFFTHCITKFVYFAVRQKLFYRTLGIWNQSNSHPLFLESNNRYHQLALTKMRRLLIVIMIGTIGSWIAWTTITFFGDSV

HTRKDPNNENETITEEVPRLLVRSWYPWDAMSGAAYYVSLVYQIYYVGFSMLHSNLLDSLFCSWLIFACEQLQHLKEIMK

PLMELSATLDTYVPKSADLFRAPSASSQDNLVDSDYNQSNEDANLRNLYTTHQEMGVTYRSGNLQEFSSGGIGPNGLSKK

QELMVRSAIKYWVERHKHVVRLVTAIGDAYGIALLLHMLTSTIMLTLLAYQATKIDGVNKYALTVIGYLLYALAQVFHFC

IFGNRLIEESSSVMEAAYSCHWYDGSEEAKTFVQIVCQQCQKAMSISGAKFFTISLDLFASVLGATVTYFMVLVQLK

>AKO69815.1_olfactory_receptor_co-receptor_[Campoletis_chlorideae]

MMKFKQQGLVSDLMPNIRLMQISGHFMFNYYADGMKFMHKIYCVVHLVLVLVQFGLCLVNMALESGDVDDLTANTITVLF

FAHSIVKIGYFAVRSKLFYRTLAIWNNPNSHPLFAESNARYHAIALTKMRRLLFAVSGATILSVLCWTGITFVGDSVKKV

VDPVTNETTMVEIPRLMLRSYYPFDASHGMAHILTLVYQFYWLVFTMFDANSIDVLFCSWLLFACEQLQHLKQIMKPLME

LSATLDTVVPNSNELFKAGSAEHLRDNSNNVQPPPSAQGENMLDLDLRGIYSNRQDFTATFRPTAGTQFNGGVGPNGLTK

KQEMLVRSAIKYWVERHKHVVRLVTAIGDAYGFALLLHMLITTITLTLLAYQATKVNSVDVYAATTIGYLLYSLGQVFLF

CIFGNRLIEESSSVMEAAYSCHWYDGSEEAKTFVQIVCQQCQKAMSISGAKFFTVSLDLFASVLGAVVTYFMVLVQLK

>AKW50880.1_odorant_receptor_coreceptor_[Ectropis_obliqua]

MMAKSKSVGLVSDMMPNIRLMQWAGHFLFNYYDENSGMNMLLRKVYACVHAFLISLHFIFMCINMTQYSGEVNEFTANTI

TVLFFAHTLIKLVVFAFNSKNFYRTLAVWNQSNSHPLFTESDARYHQQALTKMRKLLYMICAVTGGAVISWVTITFFGES

VRMITNKETNETLTEPAPRLPLKAWYPFDAMSGSMYIFAFVFQTYWLIFSLGIANLLDLMFCSWLIFACEQLKHLKAIMK

PLMELSASLDTYRPNTAELFRISSSLNSEKMPDTTDADIRGIYATQQDFGMTLRGAGGRLQNFVANPNNPNGLSQKQEML

ARSSIKYWVERHKHVVRLVASIGDTYGTALLFHMLVSTITLTLLAYQATKINGMNVYAFSTIGYLSYTLGQVFHFCIFGN

KLIEESSSIMEAAYSCQWYDGSEEAKTFVQIVCQQCQKAMSISGAKFFTVSLDLFASVLGAVVTYFMVLVQLK

>ALD51504.1_odorant_receptor_co_[Locusta_migratoria]

MQKPHGLVADLWPLIRMVQYSGHWMLEYSGGKALRAIYSSAVSLLVVTQFALMAVNLIQRSGDVNELAANTITVLFFLHP

VTKFGYFAVRSKAFYRTLATWNQSNSHPLFAESQARFHQLSVVRMRRLVMYVVSVTALSVVSWTSITFMGDSTREVTDPD

NANETITEEVPRLMISTWYPFDASSGMGYMLAFVYQLYWLTATLMHSNLMDVMFCCWLIYACEQLVHLKEIMKPLMELSA

TLDTVVPHTSELFRAASTLPTNEPLYDAGNGAADGLTIRGIYSSQRDFSGFNRRSAALSTVREADAGGAVSSAGGIGPNG

LSKRQEMLVRSAIKYWVERHKHVVRFVGNIGDAYGAALLLHMLTTTVTLTLLAYQATKIDSVDVYAASVLGYLFYTLGQV

FLFCVFGNRLIEESSSVMEAAYSCHWYDGSEEAKTFVQIVCQQCQKSLMISGAKFFTVSLDLFASVLGAVVTYFMVLVQL

K

>ALG36144.1_odorant_receptor_1,_partial_[Sclerodermus_sp._MQW-2015]

MMKFKQQGLVADLMPNIRLMQISGHFLFNYYNDGGTKFMHRIYCCVHLFLIVLQYGLMGVNLILESGDVDDLTANTITML

FFTHSIVKVIYFAVRSKLFYRTLGIWNNPNSHPLFAESNARYHSIALTKMRRLLFCVGAATVFSVLAWTGITFLEDPWKK

VIDPVTNETTLVEMPRLMLRAWYPFDVKHGMSHVLILIFQFYWLLFAMTDANSLDVLFCSWLLFACEQLQHLKQIMKPLM

ELSATLDTVVPNSSELFKAGSADHLRDTQGTQPALPQPAQGENMLDLDLRGIYSNRQDFTATFRPTAGMTFNGGVGPNGL

TKKQEMLVRSAIKYWVERHKHVVRLVTSIGDSYGIALLLHMLATTITLTLLAYQATKVHAVDVYAATVIGYLLYSLGQVF

LFCIFGNRLIEESSSVMEAAYSCHWYDGSEEAKTFVQIVCQQCQKAMSISGAKFFTVSLDLFASVLGAVVTYFMVLVQLK

>ALJ33155.1_ORCO_[Athetis_dissimilis]

MMTKVKAQGLVSDLMPNIKLMQAAGHFLFNYHSENAGMSNLLRKVYASTHAILIVVNFACMGINMAQYSDEVNELTANTI

TVLFFTHTIIKLVFFALNSKSFYRTLAVWNQSNSHPLFTESDARYHQIALTKMRRLLYFICGMTCLAVVSWITLTFFGES

VRLITNKETNETLTEVAPRLPLKAWYPFNAMSGTMYMIAFGFQVYWLLFSMAIANLMDVMFCSWLIFACEQLQHLKAIMK

PLMELSASLDTYRPNTAELFRASSTEKSEKIPEAVDVDIRGIYSTQQDFGMTLRGAGGRLQNFGQQNANPNGLTPKQEML

ARSAIKYWVERHKHVVRLVASIGDTYGTALLFHMLVSTITLTLLAYQATKINGINVYAFSTIGYLSYTLGQVFHFCIFGN

RLIEESSSVMEAAYSCQWYDGSEEAKTFVQIVCQQCQKAMSISGAKFFTVSLDLFASVLGAVVTYFMVLVQLK

>ALM30348.1_odorant_receptor_[Galleria_mellonella]

MMTKVKAQGLVTDLMPNIKLMQAAGHFLFNYHSDNSGMSMLLRKVYSSVHAVLIVVNYVCMAINMAQYSDEVNELTANTI

TVLFFAHSVIKLLFFALNSKSFYRTLAIWNQSNSHPLFTESDARYHQLALTKMRRLLYCICGVTVLSVASWVTLTFFGES

VRFIANKETNETMTEPAPRLPLKAWYPFNTMSGTMYIAAFALQIYWLLFSMAIANLLDVMFCSWLIFACEQLQHLKAIMK

PLMELSASLDTYRPNTAELFRASSTEKSEKVPDPVDLDIRGIYSTQQDFGITLRGAGGKLQTFGQPTPNNPNGLTQKQEM

LARSAIKYWVERHKHVVRLVASIGDTYGTALLFHMLISTITLTLLAYQATKIDGVNVYAFSTLGYLTYTLGQVFHFCIFG

NRLIEESSSVMEAAYSCQWYDGSEEAKTFVQIVCQQCQKAMSISGAKFFTVSLDLFASVLGAVVTYFMVLIQLK

>ALR72547.1_odorant_receptor_ORco_[Colaphellus_bowringi]

MMKFKVSGLVADLMPNIRLIQASGHFMFNYHADNSGALHALRLGYSCMHLVFCLFQFGCTFGNLVVERDNVNDLAANTIT

VLFFTHCITKFVYFAVRSKLFYRTLGIWNQANSHPLFVESNNRYHALALKKMRTLLVCVMATTVLSASAWTGITFVGDSI

HHIKDPDNENETIIEEIPRLLVKSWYPWDAMSGTAYYASLIFQIYYVFFSLAHANLMDSLFCSWLIFACEQLQHLKEIMK

PLMELSASLDTYVPKSADLFRAPSANSQDNLIENDYNAKNEEINLKGIYNTRQELGINFRSGALQTFGQGGGGVGPNGLS

KKQELMVRSAIKYWVERHKHVVRLVTAIGDAYGVALLLHMLTSTVMLTLLAYQATQIGGVNKYAATVIGYLVYSLAQVFH

FCIFGNRLIEESSSVMEAAYSCHWYDGSEEAKTFVQIVCQQCQKAMSISGAKFFTISLDLFASVLGAVVTYFMVLVQLK

>ALT31679.1_odorant_receptor_co-receptor_[Cnaphalocrocis_medinalis]

MMTKVKAQGLVSDLMPNIKLMQAAGHFLFNYHSDNAGMSTLLRKIYASAHAVLIVIHYLCMAVNMAQYSEEVNELTANTI

TVLFFAHSVIKLLFFAINSKSFYRTLAVWNQSNSHPLFTESDARYQQLALTKMRRLLYFICGVTVLAVVSWITLTFFGES

VRLIANKETNETLTEPAPRLPLKAWYPFDAMSGTMYVVAFVYQIYWLLFSMAMANLLDVMFCSWLIFACEQLQHLKAIMK

PLMELSASLDTYRPNTAELFRASSTDKSEKVPDPVDMDIRGIYSTQQDFGMTLRGAGGRLQNFGTNGSNPNGLTQKQEML

ARSAIKYWVERHKHVVRLVASIGDTYGTALLFHMLVSTITLTLLAYQATKINGINVYAFSTIGYLSYTLGQVFHFCIFGN

RLIEESSSVMEAAYSCQWYDGSEEAKTFVQIVCQQCQKALSISGAKFFTVSLDLFASVLGAVVTYFMVLVQLK

>ALX17413.1_Orco_[Pediculus_humanus]

MGKYKPHGLVADLWPNVRLMQLSGHFLFEYHDNNSAINVLARKIYSWVHLVLILINYSAIIANLAMESDDVNALAANSIT

VLFFAHCVTKFLHFAIRRQKFYRVLGTWDTQNSHPLFAESHARHRALAIRKSRKLLMIAVVGTLMTIVGWTGSTFFGEST

KTISDPESGNQTVTVEIPRLMLRAWYPWDSSSSSYYLLTFLFQLYWIIFTLFHSNLVDILFCSWLIFACEQLLHVKEIMK

PLIDLSSSLDTFNPNSADLFKMTNTNNDSENLYDSKNERSMNIRGIYSNIRELKGNRISVVNPVGTMNGIGPNGLTKKHE

MLVRSAIKYWVERHKHIVRYVSSIGEAYGLALLMHMLTSTITLTLLAYQATKIDSFDLYAMAVLGYLIYSLAQVFVFCTY

GNQLIEESSSVVEAAYSCQWYDGSEEAKTFVQIVCQQCQKALSISGAKFFTVSLDLFASVLGAVVTYFMVLVQLK

>AmOr1

IHYKSDAEYTVHVAKTLLTLIGIWPRRNTFIDNVKFYVQIGIVFFLMCFLLLPHVIYTYFDCENLTKYMKVIAAQIFSLL

AIIKFWTIIINREEIRFWLMEMEIQYRDVECEEDRLVMMNTAKIGRFFTIVYLSLSYTGALPYIILPLISERIVKEDNTT

QIPLPYLSDYVFVIEDSPIYEMTFVLQIFISSIILSTNCGTYSLIASITMHCCGLFEVTNRKIKTLCNNRDLHDRVIDIV

QSHLKAIEYSARVGESLSIVFLSEMLGCTIIICFLEFGVIMEDHKTLSTVTYFVLMTSIFVNVFIISFIGDRLKQESERI

RETSYFIPWYDFPTEVAKNIKTIILRASRPSSLSGAKILELSLQAFCDVCKTSAAYFNFLRAM

>AmOr3

HYRSDAEYTVRVAKILLTMVGIWPRRNTFSNNVKFYVQTTIVFFLMCFLLLPHVIYTYFDCENLTKYMKVIAAQVFSLLA

IIKIWTILINRNEIRFCLMEMEVQYRDVECEEDRLVMMNTAKIGRIFTIVYLFLGYGGALPYVILPLISERIVKADNSTQ

IPLPYLSDYVFVIEDSPTYEITFVVQMFTSFLIMSLNYGIYSLIASITMHCCGLFEVTNRRIETILKNNRDLRGRIADII

QSHLKAIEYSALVGKSLSIVFLSEMLGCTIIICFLEFGVIVEDHKTFSMVTYFVLVTSMFVNVFILSFIGDRLKQESERI

GQTSYFLPWYEFPTEIAKNIRIIILRASRPSSLSGAKMLDLSLRVFCDVFKTSAAYLNFLRTM

>AmOr4

KHTEKDLKQAFYVQTFLKIIGAWPIAIESSSKIQKWFIISFYLFLQICIVAPCILDVFLKEKNGSRRINLFMLLISTLNQ

VFKYVITLNRANELRIAIHEIKKDWLTATPEDRFIFVMNSRIGQRIMLIMAFIMYISGLGYMVLPLLKGKIVLPNNVTIR

LLPCPTYFTFNELVSPYYEMIFMLQLLARFFIYTVLNSTVGISLMLSLHMCSLLKILTRKMADLTDGEKIMQQRIVDIIE

YQTRIKRFLSNTELITQYFCFYDIGCSTCLICFIGYSIIVENHNIASTVIYFSGLVTCTLMIYIICYIGQLLLDESNNLA

QTCITLNWYRFPKKKARYLILMIIMSNYPIKLTAAKVVDVSLTTFTDVMKAAVGYLNMLRE

>AmOr5

HTEKDLKQAFYAQSFLKIVGVWPIPIPLSSKIRNWFITFFSLFLQICIVGPCILVMFLKEKNGKRKINLFKLLTNTLNQL

FKYIITLNRANELAIAMNEIKNDWLTATSEDRWIFTANSKMGQKVMLIVAVTVYSSGLGYMLLPILKGKIVLPNNVTIRL

LPCPTYFTFNELVSPYYEMIFMLQLLAGFFSYTVLNGTVGISLMLSLHMCSLLKILTRKMANLTDRSITSENIIQEKIVE

IVEYQTKIKRFLGNAELITEYFCFYDIGCNMCLMCFIGYSAILENHNIAAIVVHFMLLGTCIFIIYIVCYIGQLLLDESN

NLAQQCITLSWYHFPTRKARCLILMIIMSNYPVKLTAAKVVDVSLTTFTDVMKAAMGYLNMLRE

>AmOr6

EKDLKQAFYAQPFLKIIGAWPILISLSSKIQKWFIISFSISLQMCIVVPCILVMFLKEKNGRRKINLFMLLTNILNQVFK

YVITLNRANELRIAIHEIKKDWLTATPEDRFIFVTNSRIGQRIMLIIAVITYSSGLGYMVLPLLKGKIVLANNVTIRLLP

CPTYFTFNELVSPYYEMIFMLQILAGVFVYTVLSGTIGISLMLSLHMCSLLKILRRKMIDLADGSITSSENTMQKRIVDI

VEYQTKIKRFLGNTELITQYFCFYEISCNTCLICFIGYCIILENSNVVAIVVHFMLLGTCILVTYIVCYIGQLLIDESNN

LARTCITLNWYHFPTRKARCLILIIIMSNYPVKLTAAKVVDVSLTTFTDVMKAAMGYLNMLRE

>AmOr7

EKDLKQAFYAQPFLKIIGAWPIVISLSSKIRKWFIISFSISLQMCIVVPCILVMFLKEKNGRRKINLFMLLTNILNQVFK

YVITLNRANELRIAIHEIKKDWLTATPEDRFIFVTNSRIGQRIMLIIAVIMYSSGLGYMVLPLLKGKIVLPNNVTIRLLP

CPTYFTFNELVSPYYEIIFMLQILAGFFIYTVLSGTIGISLMLSLHMCSLLKILRRKMIDLADGSITSSENTMQKRIVDI

VEYQTKIKRFLGNTELITQYFCFYEISCNTCLICFIGYCIILENRNVIAIVVHFMLLGTCIFVTYIVCYIGQLLLDESNN

LARTCITLNWYHFPTRKARCLILIIIMSNYPVKLTAAKVVDVSLTTFTDVMKAAMGYLNMLREV

>AmOr8

NAREGINHTFWFAYPLSRMLGYWPLNVPSSSKILNSFTIFFSYLLPLIVLIPGLLYVFLKERNGRRKVKMLMPHINSIAQ

MTKYTIILRRTKELGKLLDEIKKDWSTATQENRRIFSERASIEHKLTMIVAITIYGGGFLYRAILPLSKGRIVLPNNVTI

RLLPCPGYFGLDEQVTPNYEIIFTLQVLGGFVTHTAVCGIKSACLMVCMHMCGLLRILTNKLTDLTNDNDNDNDERVVQE

KIVHIVEYQTRIKEFLNHVDQFVPYVYLIEIFVGVLITCILGYCIIVEDSDAMAIIAYVALQTTCVFGTFSICYVGQLLV

DESESVRQACKTLKWYRLPTKKARSLILLIIMSNYPIKVTAGRLVDVSLVTFTSIIKSAVGYMNILQQV

>AmOr9

AREGINHTLWFAYPLSKMVGCWPLNISTFSKIFNAFIIFISYLLSLIVLVPGLLYLFLKEKNGRRKIKMLMPLMSTIAQM

TKYTILLRRMKEFNKLLDEIKKDWSTATQENRQIFSAKASIEHKLTTVIAITIYGGGIFYRMILPLSKGRIVLPNNVTIR

LLPCPGYFGLNVQITPNYEIIFTLQILGGFVIYTALCGVKSSCLMLCMHMCGLLRILTNKVMELTSDKNKVMELTSDKDE

KVVQEKIVYIVQYQTRIKEFYNYVDQFVPYVYFIEMIVGVLITCVLGYCIIVEDSDAMAIIAYVVLQVTCVFGTFSICYA

GQLLVDESENVRQACNTLKWYRLPTKKARSLILLIIMSNYPLKVTAGRIVDVSLVTFTSIIKSAVGYMNILQQI

>AmOr10

NAKEGLRHTFWFAYPFSRMLGHWPLSVSSSSKILNSFIIFISYLLQMIVVIPSLLYVILKEKNPKKKIKLLMPHLNSIVQ

MIKYTILLRQMKLIDKLLDEIKKDWSIATEENRRIFSRTASVEHKLTSIIAITIYSGGFFYRMILPFSKNKIVSNNMTIR

LLPCPGYFGLDEQVSPNYEIIFILQVFGGFVIYTAVCSTKSICLMLCMHMCGLLRILTNKVMELTNDNDNKVMELTNDND

ERVVQEKIVHIVEYQMKIKEFLKQIDQFVPTIYLFEVFIQVLIMCIIGYCIIMEESNGMGLITYVIVQMTCLIGSFSVCY

VGQLLIDESENIRQAFIALKWYQLPVKKSRSLILLIIISNYPIKVTAGKIIDLSLVTFITIIKTAVSYMNMLQQI

>AmOr11

NAKEGLKHTFWFAYPFSRTLGYWPLVSPSATKFFNSFTIFTLYFLELIVLIPGLLYVLQVKNPRTKIKLLMPHLNSIAQM

AKYTIILQRAKEFSKLLDEIKKDWLLATEENRQIFSERASIEHKLTTVIVVTMYGGGFFYRTILPLSKGKILLPNNMTVR

LLPCPSYFGLNEQATPNYEIIFTLQVLGGFIIYTVLCGTKSACLMLCLHMCGLLKILTNKVMDLTNDSDEMDLTNDSDEQ

VVQEKIVHIVEYQTRIKEFLNQLDQFVPAIYLIEVVIQVLIICIIGYCIIMEDSNAMAMVIYVVFQVTCVIGTFSVCYVG

QLLLDESENIRQAYNTLNWYRLPVKKARSLILLILMSHYPIKVTAGRIMDLSLVTFTSIIKSAVGYMNMLRTV

>AmOr12

NAKDGIRHTFWFAYPFSRMLGYWPLSVSSSAKISNYFIIFLSYLLTLIFMVPGLLYIFLKVKNGRSRIKLLMSHINGIVQ

MAKYTILLRKTKEIAKLLDEIKKDWMTASEENRQIFSTRASIEHKLTMVVVVTMYGGGFFYRAILPLSKGKIVLSNNVTI

RLLPCPGYFGFDEQVSPNYEIIFTLQVLGGFVIYTAVCGTKSICLMLCLHMCGLLKILTNKVMELTNDTNKVMELTNDKD

EKVVQEKIAHIVDYQTRIIEFLNDLNQFVPSVYFFEIILEVLIICIIGYCLITEDNNTMATVIFVIFQITCFIGTFAVCY

AGQLLVDESENVRQACSTLNWYRLPVKKARSLILLILMSNYPIKVTAGRIVDVSLVTFTSIIKNSVGYMNILQQV

>AmOr13

YSLKLVYPLLKILGAWPKSSPSSSTILKCCLISICYLIQLMVLIPGILYIFLKEANLGGKIKMFVPHMNGITQVSKYTIL

LRQIKEFNIILKEVKRDYSLATDKNMWIFTTRAYIGHKMMIAIAIAMYSSGVGYMILPFLKGRILLPDNTTVRLLPCPGY

YMFNEQVTPNYEIIFTIQVLGGFLNYTTLCGTTGITTMLCLHMCSLLEILINKMNDLTCQEIIVRKKLADIVEYQMKIID

FLNHVEQLTSYLYFCEILEYVCGACVIGYCLITENSNAAALIVYFILEFLCIFCTLTICYIGQLLIDESDKVRQISVTLD

WYRLPVNEARGLILVIIMSNYPIKVTAGKIVDISLITFTDIVKTSVGYLNILRTV

>AmOr14

AENGMRHTVWFAYPLLRILGAWPNRVSSSSKIFNWYLIFTCYTLQLIVLVPGFLHVFLKEKNGRKKMKMMIPQVNGYLQL

CKYSLVLRWTNKLRVLLNEMKEDWLNTTEEDQLIFRAKASFGHRVMSMIAIVTYSAGLGYRTILPLSKGRILLPNNTTKR

LLPCPGYFVFNEQVSPIYEIIFIIQVLGGLLTYTIMCGTIGMCVMFCLHSSSLLRILLNKIYQLTKQVVHEKIVDIVKYQ

TKVKGFLKNVEQLTTYLFLLEIMVETSIGCVIGYNVVTEDSNAAAMIIHLMMQVSTISCTFIMCYVGQTLIDEGNNVRRM

SITLDWYRFPVKEARNLILVIIMSSYPVKLTAGKVVDISLATFTDIIKTTVGYLNMLQKV

>AmOr15

AEDGMRHTIWFAYMLLGKLGAWPNRATSFSRTRNCILIFMCYSVQLIILIPGLLHFFLKEKDSRKKVKILIPLINGYLQL

CRYSLVLRSANKLCHLLNEMKKDWMNISEEDRLIFRRKASIGHRLMSVVAIIMYSAGLGYRTFIPLSKGRILLPDNTTIR

LLPCPGYYIFNEQITPNYEIVFTLQVIGGLLSYTIMCGTTSMCAMLCLHATSLLRILVKKINELTKQQPDINESAVHMKI

TDIVRYQTKIKQFLNDVEHITTYLFLLEIIDETGIGCVIGYCAITEDSDATAAIIYLLLEASVFGVTFTMCYVGQILIDE

GNNVRRMSITIDWYRFPAKEARNLILVIIMSSYPVKLTAGKVVDISLSTYTDIIKATVGYLNMLRKV

>AmOr16

KAEEDLKYATRFVKPILATIGAWPISSSTSLKALQRLGHIFTYFLFFLIMIPTLAYVFLKEKNSKVRLKLMGPIINCSMQ

FFKYTIIIWRRKEIQEGLHAIRHDWIQATEEERLIFRSKMKIGRRVVLIAAFTMYGGGLCYTILPLLKGTVITADNITIR

PLPCPSYFIINEQQSPIYEILFVLQVMAGMAIYAVISGTCGISALLVLHACSMLRILVNKIKKLVNKSDMSELQRKIMDI

VEYQMKIKRFLKNIETVTEYICLIEMIGGTCLMCLVGYCILMENTNTMAVVVYITLQISIIFCVFILCYIGQMLVDENYI

VSQASSTINWYRLSIKNMRCLILIIAMSNYPMKLKAAKMMEMSLTTFTDVMKMSMGYLNILRE

>AmOr17

KAEEDLKYATRFVKPIMGMIGAWPISPSTSLKVLQRLRHIFTYFLFFLIMIPTLMYVFLKEKNNKVRLKLMPPIINCSIQ

CFKYTIILWRRKEIQEGLYAIKHDWIKATEEERLIFRSKAKIGRRVVLVVAFTMYGGGLCYMILPLLKGTIVTANNTMIR

ALPCPSYFFLNEQQSPIYEILFVLQIIAGIAIYAVICGFCGIFALLVLHAWSMLRILVNKIKKLVDKSDMSELQRKIMDI

VEYQMKIKRFLKNIETITEYICLIEMIGSTCMICLVGYCILMENTNTMAIVIYITIQISIIFCIFILCYIGQLLVDENYI

VSQASSTINWYRLSIKNMRCLILIIAMSNYPMKLKAAKMMEMSLITFTDIMKVSMGYLNILRE

>AmOr18

ANYKNDLSFNVRLNVWTLRTIGTWPRSPSWLETLEHVCLNLFCYELLAFILIPCSIYIILEIKDFYNQLKLGSALSFFLM

AVMKYCVFIIREDDIRKCVELIENDWKNVRYQEDRKIMLENASFSRRLIVICGTFMYGGVIFYYIALPLTRAKIVEEGGN

LTYRRLVYPFPVDARHSPINEICYTIQLLSGFVAHNITVAACGLAALLAIHACGQLQILMSWLEKLVDGRKNDNENKNDN

ENLDQRLANIVKQHVRIINFIALTEDLLHEISLIEVVGCTLNICFLGYYSMMESKQPVSGVTYIILLISVTFNIFIFCYI

GQLLAEQTVKVGEKSYMIDWHRMPWKKSLAIPLMISMSHSTTKITAGNIIELSISSFGDVIKTSVAYLNMLRTF

>AmOr19

SYNASYKNDLFFNVQLNVWTLRTIGTWPKSLSWLETIEHVCLCFLNYVLLAFILIPGVMYFLLEMKDFYDQMKLGSALSF

FLMAVMKMCVFIIRENDIRKCIECIEDDWKNVKYQEDRKIMLENASFSRRLIVICGAFMYGGVVFYYIALPFTRAKVVEE

GGNLTYRRLVYPFPKDARRTPANELLYTIQLLSGFVAHNITVAACGLAALLAMHACGQLQILMSWLEKLVDGRENDENDD

ENLDQRLVNIVEQHVRIINFITLTEDLLREISLVEVVGCTINICFLGYYSMMEWDTEHLIRYIILLTSVTFNIFIFCYIG

ELLAEQTVKVGEKFYMIDWYRMPWKKSLAISLIISISRSTTKITAGNIIELSISSFGAIIKTSFAYLNILRTL

>AmOr20

EYQKNVNLSIQYNRWLLKPMGLWPNSYTSKDYPYWLINIVCYCLISFLFIPCTLYLFLEIEDFYGKLKQFGPLIFCMMAF

VKYYYLIFHKTDIRECVERIKWDWRNITYAKDREIMIMYANFGRKLVMVCTFFMYSGFAFYYIAIPISVGRVKTDNLTFV

PLVFPFRFIVDTRYSPTNEIVFSIQLMAGALMHGITSAACSLVATFAVHACGQMQVLMNWLQHLIDGRLDMDEDERLDGR

IADVIRQHVRVLKFLALTEKTLQQISFTEFLGCTLDICLVGYYVIMESNDVTSVITYIILLISLTFNIFIFCYIGEIVAE

ECRKIGEISYMIEWYRLMGNKKLFCILIIAMSNSSIKLTAGNIVNLSISTFTDVVKTAVTYLNVLQK

>AmOr21

KIDQDYKSNVNLSIKYSRRISKMIGLWPIFDSTIHKFLRMLYNTICYCLLMFMIVLGWMYIAFEVKNIYDGLKFVSLMSF

CMLSITKYHLINIHKDDVRECVKRIEWDWKNISYSEDREIMLMNANFGKRLIIVTTTVTYSGFVFFYIAIPMKIGKIPAP

DANISFIPTMFPFPKYIADVRYSPINEIVFFFQFMCGFLVHGVTSSACSLAAIFTVHACGQIQVMMIWLEHLIEGRLVDQ

RIAKIVSQHVRILKFLSLIEKILQQVSYMEFLECTVNVCLLGYCAIIESNHLTEVVTYVIILITIIFNIFVFCYIGELLA

DQSRKIGEVTYMIEWYRLSGKKKLCCVLIIAMSNSSMKLTAGNLIELSMSTFSDVVKTSFAFLNVLRTL

>AmOr22

QTNHDYKRNVNLSIQWSRWILKPIGLWPNSSSTTGKYLYRLINVICYSLISFLSIPCSLYVILEVEDIYNRIKLFGPLSF

CVMAFLKYHLLILHKDNISECIKRIEWDWKNITYSKDIEIMITNANFGRRLVIICTFFMYSGFAFYYIAVPISVGKILAE

DDNITFIPLVFPFRFIIDTRYSFINEIVFSIQLIAGALMHTITTAACSLAAIFAVHACGQMQVLSNWLKHLINGRSDMYN

SDMYNNVDSRIASIVSQHVRILKFLALTEKALQQVSFVEFLGCMLNICLLGYYVITESSHLTSAITFFILLISLTFNIFI

FCYIGELVAEQCKKIGEISYMVDWYRLEGNKKLCFVLIIAMSNSSIKLTAGNMVELCLTTFSDIVKTAVAFLNVLRTL

>AmOr23

KINQDIKNNINFSIKYSRLILKMIGLWPIFDSTIHKYLQWLYNVICYSLIMFIIISGWIYISLEVENIYDRLKFVSLMSF

CMLSITKYHLINIHKDDVRECVKRIEWDWKNISYSEDREIMLMNANFGKRLIIVTTTVTYSGFVFFYIAVPMKIGKIPAP

DANISFIPTMFPFPKYIADVRYSPINEIVFLAQFICGFLLHGITSSVCSLAAILTVHACGQIQVMMVWLKHLIDGRLDNS

IDQRIATIVNQHVRILKFLSLIEKILQQVSYMEFLECTMNVCLLGYCAIMESNHLTEVITYLILLITIIFNIFIFCYIGE

LLANQSRNIGEVTYMIEWYQLFGKKKLCCVLIIAMSNSSTKLTAGNLIELSMSTFSDVIKTSFAFLNVLRTL

>AmOr24

TKTDHDYKRNVNLSIQWSRWILKPIGLWPNSSSTTGKYLYRLINVICYSLISFLSIPCSLYVILEVEDIYNRIKLFGPLS

FCVMAFLKYHLLILHKDNISECIKRIEWDWKNITYSKDREIMITNANFGRRLVVICTFFMYSGFAFYYIAVPISVGKIPA

EDDNITFIPLVFPFRFIIDTRYSFTNEIVFCIQLVAGVLLHTITTAACSLAAIFAVHACGQMQVLSSWLKHLINGRSDMR

SDMYNNVDSRIASIVNQHVRILKFLALTEKALQQVSFVEFLGCMLDICLLGYYVIMESSHLTSAITFFILLISLTFNIFI

FCYIGELVAEQCKKVGEISYMVDWYRLEGNKKLCFVLIIAMSNSSIKLTAGNMVELCLTTFSDIVKTAVAFLNVLRTL

>AmOr25

DGKKANLSIQWNRWLLTPIGAWPNLRSRIGKCYSLLISIICYGLIGFMLVSCSMFLMVEIKKVYNRIKMIGPLSFFLMTF

MKYYLLLLHENDIREGIECIEWDWKNMKHQEDRNIMIEYANYGRKLVLICTFFMYSAFAFYYLVLPFSVGKIEDGNLTFI

QLPFPSSSLADIRYSPYNEIVLSVQILTGVVMHAITSAACSIAAVFAVHACGQMQVLMNWLDHLVDGRSDMSDMSKAIDD

RIANIVIQHDRILKFLALTEKALQQISFVEFLGCTANMCLLGYYLIVEWNPKEIILYVALIISITFNIFIFCYIGDGVAE

QCQKVGEMAYMIEWYRLTGKKKLCCILIIAMSNSSVKFTAGNMVELSIYTFSDVVKTSVAFLNMFRAL

>AmOr26

MPVSYARDYEYSIQVNRWLLKPIGAWPNLTTRTEKLLVKLLNFICHSLIIFTVMPCIMYIFYEDESLKTRMKAIGPTSHW

LMGELNYCCLLMRAKEIVYCIEHIKYDWKTVRRARDRELMIKNAKLGRFIACIAALCMHSGIMSYTVITGFKKITFQIGN

DSYSMYRLPCPFYTNLDVRFSPMNEIVFALQLLSGFISTSVTVGACGLAAVLAMHACGQFNVVMIRSDKLVKDNNEKKQD

KDNNEKKQDEQTLHKKLGFIVEHHLRTLSLVWYMEKVMNMICLVELVGCTMNMCILKYYFLTEKSKTILGIYAIVYASMV

FNIFIFCYIAEIVTEQGKKVGEKFYMTEWYQLPHKTALGLVLIISRSSMVIKITAGKLIQISIATFAAVFKASFAYLNMI

RT

>AmOr27

AITEEIKTNSDYSLQLNRWFLKPIGAWPLFSTKFEKTVSLILNIICYAIVILCATPSLMQIILAEESFYLKLKTLGPVSH

WFVSTVNYTALLMKSKDIRYCFEHMEADWQTIKRMEDQQTMLKNAKFGRYVAASCAIFMQGGILCFCFVTILTTETIQVG

NETRVLHVLPCAVYKKVNVEENSINIFMLCFQFVAAAIANSSTVGIFSLAAVLAAHAYGQLSVVMVWITEFVNQSRNQKK

TSRNQKKTDDFKEIGIIVERHLRVLNFITYLENIMNRIYFLELFRCTMIICIVGYYILTEEKNVQNLTTYFMMLLSICFN

IFIICYIGEILTEQCMKIGEVVYMTDWYYLPDKTILNLILIILRSTVVVQITAGKLFNMSIYTFGDVLKTAFAYLNLLRQ

M

>AmOr28PSE

KTDPDTNSDYCLQLNRWFLKPIGAWPSFPTKHERIISFLLNVSCYSSLLFTLIPCLLHMLLEDESFYLKMKVLGSLAHWF

VGTMNYTTLLLRGKEIRLCVEHIRTDWQTVTREEDQQVMLKNAKFGRYVAAVSAAILQSGVNCXCCMTISRTELIQIGNE

TRIVHVLPCAVYRKIDVTHSPNSELIIASQFLSGFIVNSSTAGIFSLAAILGAHACGQLSVVMTWITEFVNKSKNKSKKR

EKMIFREIGLIVEHHLRTLNFISCIEETINRIIFLEVFRCCLHICCLGYYILMEDYDKRSMIIYFMLFVSVCFNIFIICY

IGEILAEESMKVGEVVYMTDWYYLPDKTILDLTLIIARSSVVVQITAGKLIHMSIQTFTDVIKTGFAYLNLLRQV

>AmOr29

TQDDYKRKTNLSIQWNRWLLTPIGAWPNLRSRIGKCYSLLISIICYSLIGFMLVSCSIFLMVEINNIYNKLKMVGPLSFF

VMTIMKYYFLLFHENDIREGIERIEWDWKNVKHQEDRNIMITYANYGRKLAFICFFFMLCAFIFYFLIQPFGGGKIVDGN

LTFIQLPFPISILIDVRDSPYNEIMLSIQILTGIVMNAIRSAICSVAAVFAIHACGQMQVLMNWLNHLVEGRSDMSSDMS

KKIDDRIANIVIQHDRILKFLALTERALQQISFVEFLGCTANMCLLGYYLIVEWNPIVSFTYIAIIASITFNIFIFCYIG

ELVAEQTEKVGEVAYMIEWYRIRGKKKLCCVLIIAMSNSSIKFTAGNMVELSIYTFSDVVKTSVAFLNMLRAL

>AmOr42

SLNEYSIQVNRWLSKTIGVWPFTSSKFEKIMTKILIIVCSIIALFVTIPSMLHFILVKEDIITKLKMTGPIIYCIGGGLN

YAILLFLRDDIRYCIEHIEADWKTITRTGDRQVMFKNAKIGRIISGCIGSFLQFSTISYCTVFGVFKQTIKIGNESMEIH

VLPFPTYIPVDTNLEHGIVLGFQYLTACIMTATIIIAFSLATVFACHAVGQLTIMVTWIEEFVNRPQEEKKEKKNMRINE

ISVIIEHHLRILSFLERTEHLLNPIYFMEMFKNILTTCMLSYCILVEGHDIKVLSAYSFTITNIILSLFLICYISEVLNE

KCKEIGNIVYMTNWYRLSDKDILNLIMIIIRSSVEYKMTAGKIIDMSVITFSNIIKTIFAYLNILRQV

>AmOr45

GSLNEYSIQLNRWLSKTIGVWPLSSSKFEKIMTKILIFLCCIIALFVIIPSLLHFTLVKEDIISKLKTLGPIGYCFGGGL

NYAILLLRKNDIRYCIEHMKADWKAITRTDDQQIMLKNAKIGRIISCCFAAFMQFSTVIFCAVGVFKRTIKISNESMEIY

VLPFPTYIPVDVNPGHNIVLGFQFLAGYITTGTVIIAFSFATVFACHAVGQLTIMITWIEEFVNRPQEENKNENKNVRVE

EISVIIEHHLRILSFLERTEHLLSPICFMEMFKNILTICMLSYCILAGHDIRALSAYASAVMNISLGTFLICYVGEILTE

KCKEIGNMVYMTNWYRLPKKDILNLIMIITRCSMEYKMSAGKMIDMSVITFGNIVKTIFAYLNILRQM

>AmOr67

MKTTSNKDFTYAMIPLKFLSWPVGTWPFQVHEIFSISRTIFSISLLLLMVVILQVELYLDRSNAENNLDALLLINCGILA

VAKVMCFRIRPIGLVSNFSSAIKDYNELNSEENRVIMRRHAYMSRVACASLISCSFIASTLFMTVPMLTGDKKDIINVTE

KSIIKYPIPSKNALAIINMPLSFMVFIVEYMMLLFTSTGNLGSDSLFFGIVFHLCGQVEILKLKYNKLSNTNERTMESNT

NERTMEHIILLTKRHIYLLNLSKMLNETVSSILVIQLFSSCVLICTTGFQLILTFGNVVLTIKILAEISILLIQLFAYSY

VGEYLKTQTEGIGNSVYFCTWYDMPKNVSKDIIFIIMKSQRPVLLTAGKFFVINMETYMSILKTSMSYLSVLRVM

>AmOr69

RTIYHLLTSCACWRPPFSPLKNFAYTVYYCYVILLIYGATFCQFVDLLLIVETEDEFCDNFYLTLAIFISCHKMYSMLVN

RENIILVNRMLESEPFQPETEEEMDMRDKCDKQARLNAIYYAILVELSVMSLSFGGLLKAESHKLPYRMWLPYNYTSLSA

HIFIYTQQVVSLIVSAMIHVACDSFIWALLMHICSQIEIFNCRLRKIKHEKNEVTKLCIHYHNLIYRLATTINEQFKMVI

FVQFTVSTLTICVNLYILMGTQITFERIMQLAIYSSCMLTQIYIFCWYGNEVKLKSLDISNMIFELDWPDLDNTTKRDLL

MIMMRASYPIEMTSVHVITMNLDSFVILLKTSYSAYNLLQS

>AmOr70

QALQWTRFLLSVCGCMPPTSSSFKKSLYNIYTCVIWLLILSLVSTQILDIIINVKNKNEFIENFYITLVVFVTSCKMTII

LRYRKNILSLMDDLQHEPFSPMTHEENEIRTKFNKMNERTSICYTILVLVSATWIFVRSFFTDFKKRKLTFRAWLPYDYS

ELLPFALSYAHQATTSMFCSCQNISCDTLFAGFLVQIYCQFEILEERLKNVQQDESNYSKQCVKHYHQIYKFSRTLNEKF

KVILFLQFCAIAFILCFNLYRMTTITMIPKLLEASLYLIRVLVQILYYCWFSNEVKLKSLEVPGMIFKSDWTSWDDKTKK

IFLIIMTRATQPFEFTSGYLVTLNLEFFVALIKASYSVFNLLQRTK

>AmOr71

ILRWTFLLFALCGCFPPSSTRLKRYLYKIYAVFSFVALNSFLLSQILDMVYNVKGTDDFSDNFSVTVVVFVTCFKLITIL

TRRENILLLCNTLKQEPLSPINTEEFEIFLKFEKLTDWNTLGYFILLMSSSLCILMGSLLANFKIRKLAFRTWLPYDYST

ASAFLLAFAYQVVVATVCTFACVASDTLYSGLLIHISCQFEILEHRLKNIGSDKNYTMMKQCVRHHNHIYKYGEMVNDAF

QSIMFFQFCTSLSMICFNFYRIMQIEMDSRYVGTILYMVCSLMQIFYYCWFSNEVKLKSLELSDMIFRSNWTSLNNNVQR

AILLVMRRSMKPIEFTSIYIVSVNLDSFMTLLKSSYSAFSVLQQSR

>AmOr72

LRWTFKLFVATGYFLSPKSPRKRFLYNVYTVVVTLFLLSFLLTLIMQIVFNVRTADELSENFGITITVFTTICKFINLLF

RRGIIISLLDLLQKEPFLPMDIEEIKIHTKYNKLIEKVSIFYTLQNVSCLVALIGATLITDFKKKKLTFEAWIPFNYTAS

WLFSLTFIHQCGCAVVTSFGISIFDTLFAGLLLQVCCQLDTLVYRLQNIKEDAQSLKYCARQHELIYRFTELMNKLFSSI

LCLQFLISAVAICFSVYRVIYTKTDSQFAGAIIFVFSALIQIFYFCWHGDIAKYKSLEIPDMIFNSNWPNLSNEAKKILL

IIMARSLTPVEVVSAHIIPLNLESFKRLIKATYSAYNMLQQTK

>AmOr73

KLSLSFALLTYGGYWRPTKWPASSYKYHLYNIYSAFMIFLLYFITFCTCVLISKNLKTMSEKFSLCISVFGVSLKVANLF

LQRGKIINIMNSLTKENSIPRDEQEEIIQRRNDNYARKVTIYCEILNESAVFFATVGQYKRFINTRTLPVSDWIPYDLSS

TELYIISLLYQTVGLLICANASVGNETLIAGLMIQAGVQFEIFCHRAQNLPSLVLTVFAETVNTVFQYMIFLQFTISSVV

LCLSIYKFSTVDPLSMNFVWSGFYLCCMLMQVYLYCWFGNEVTLKSNKVSDAIYEMDWTILPSNVMKDLLLVIARSKKPV

KITSGQIFILSTESFMKIMKISYSSFNILKN

>AmOr74FIX

DVSLKVSQFLLKSAGIWVIGNDAEERQRKFAVFYTLAALIYGIYVNAVDIYHNLDNLAHCVFLTCNMMCILLGLFKCFVI

SFFRIEFSRIVSYAQKHFWRLDYDYDEKILFGECQKFCRLWIIVVSMISQSSLAFYIITPIYENIGKNKSERILPFKMWV

DLPLSVTPYYEIMFVIQLLAVEQIGIAYVCSDFFLCILNLHALYQFRMMQQELSKIWSAIEQQTTSVAVALKKCIRRHQS

LIEFCNKLEQVFTFPILSHVVVFSLLMCFDTYEILLADIPTLKRLIFLCHMVASFIHIIFFTYICHGLMEESGNVGLATY

SGWWTTLPMMLRKDIRIIMMKSMRPCYLSRSGFFPMSLETSTALVSSTMSYFTLMR

>AmOr75

SIIWTSFLMKIVGLWLATDRNEQRQRDFALIYTVGTLFISICIAFRDIYYSWGNFSNSVFICCNILYVAIVLLKISVLYA

HREEFFNLIAFTQKNFWRLYDDPQELLIITGCKKLCNFSIVLIFCAQGTCAGYMVTPLIENIGKNESDRALPFNLWIDFP

VGLSPYFELLFILQILCVYHVATCYICFDNLLCIVNLHVAGQFRILQHRLKNLGNAIPRYEKCCYERLKDCVVQHQTLIE

YCKRLEDIFTVMVLGQVMFLAVVICLVGFQLFLADTSASKKASLVLNLGGTFFQLLIFTYSCDNLIRQSVNVGNAVFSGP

WVNLPMSVRKNLIIVIMRSQKICCLTAGKFFPVSLETSTAVLSTAISYFTLLKQ

>AmOr76

DLSITVTAFYMKIAGFWTSTNYVEERRRNVTMSYTLFAILFAATTEARDLYFSWGNFSDSIYVACNIITVSLVLIKLLTS

FIYNEELLGIIRYAKTNFWHSNYDTCEKSIMNKCQRTCNYLVFVFTFFAQGTVLGFILRPILVNRGKNESDRILPFNMWL

ELPLSITPYFEVMFFVQVVFVYHVCVCYHCFDSLLCILNLHTASQFRILQHRFANTCNEKRGKRDEDEESALSFYEYYSK

LKAYIRQHQALIEYCKKLEQVFNSIVFGQVLLFSLLMCLDGYLILMEETPFGRRVTFTFHITGCMCQLLMFTYSCDCLIR

DSMDIADAAYNCSWSFLPMMIRRDLMFVITRSRTPCCLTACGFFAVSLETYTKVLSTAISIFTILK

>AmOr77CTE

ASMTMTAAFMKLVGLWTAKNRREQRARKFALIYTVAAMLFALWIEFTDFYYSFGDFSTCLFNTCNIIYITMPLLKIFVIV

LNKKDFFHLIFYTEKHFYKDNYDEHEQRIFTNCRRQCIIFVCFLTFSTKGTLVCYIVSPLVENIGKNQSERALPFNMWVN

LPLSTSPYYEIIFTIQVLSLYHIGVGYFCFDNLLCVLNLQLAGQFQILQYKMANIVDLLKEKNEKRIINTSYFAKRIINT

SYFAKKCYEAFKKCIREHQALIAYCEKLEKVFSLIILCQVLTFSLIICLDGYQII

>AmOr78

SENQLDVSITLSTFFLRNIGLWMSDDPGEQRRMRILLVYTVWILLLGMIINGRDLYFTFLYNGDILYALTNNVTMVMGLI

KIYIILLYKGKFLNLIVHMQQNFWNVNYDYHEKEILDDCRKTCIFFVSSLTTIGICAMLSYLMTPFAIRSGNNESERMLP

FNMWLDMPLSKTPYYEITFLIQAMCVYYIGISNFCFDTVFCIMAVHLAGQFRILQYRFTKLCDTDNQICNQICKKNLILE

EQMQKFHEKFKKYVRRHQALIDYHQKLENVYTTIMLSQVLLFSVLICLFGYQVLLATASLARRSIFIFLLMGAMFLLFMF

TFSCNGVMEQSDNVAVGTYSALWTVMPMMLRKDLIMVIMRSRRVCCLTANRFFPISLETYTKILSTAVSYFTLL

>AmOr79

RLDISINLSTFLLKNVGVWMSHDPGEQRRMRMLLVCTVWMLLLGIVINTRDLYFTMLYNGDILYVVTNNITLIISLVKIC

NIIIYKGKFLNLIVDMQENFWNVDYDYHEKEILDDCKKICIFFISSVTTIGICAIISYLMTPFVAQSGSNESERMLPFNV

WITFPVTRTPYYEIIFFIQAICLYYIGISSFCFDNIFCIMAVHLAGQFRILRYRLTKLCEQEIYEKDSTLTKQMHKFYEQ

FKECVRHHQALIDYHQNLENVYTIITLGQVLVFSVLICLFGYQVFVATASFARRSIFVFMLNGSMFLLFMVTYSCNGVTE

HSDNVAIGAYSALWTIVPMMLRKDLIMVIKRSRRVCCLTANGFFPVSLETYTKILSTAVSYFTLLNN

>AmOr80

TESQLDISINLSTFFLKNVGIWMSDNPNEQRRIKMLFLYTIWNLLFGTVVNSRDLYFTLLYDGDILYVTTNNITMIMGMV

KICIILIYKKKFLNLIVYMQQNFWNVNYDHREKQILDDCRKTCIFFVSCVTIMAICAMICYIMIPFIAQSGSNESERMLP

FNMWINLPISRTPYYQITFLIQATCVYYVGISYFCFDNIFCIMAVHLAGQFRILRYRFTKLCDMEYGIKENSQKENSQSI

LSKQMHKFYEKFRKCVQHHQALIDFYQNLENVYTMITFGQVLVFSVLICLFGYQVLVATISFARRFIFVFMLNGSMFLLF

MVTYSCNGVIEHSDNVAVGAYSALWTIMPMDLRKDLIIVIRRSRRVCCLTANGFFPVSLETYTKILSTALSYFTLLSN

>AmOr83FIX

DNQLDISISLSTFFLKNVGVWMPDNSDEQRRMKMLFLYTIWMLFCGTIISTRDLYFTLLYNGDILYAMTNTITTIMALIK

ICIILTYKGKFLNLIVYMQQNFWNVDYDCQEKEILDDCRKTCIFFISSVTTIGMCTVMSYLTTPVITQSGSNESERMFPF

NIWINLPITRTPYYQIIFFVQGVSVYYIGISYFCFDNIFCIMAVHLAGQFRILRYRLMTLCDTEPETREKDSRSYKFYEQ

FKKCVRYHQALIDYYQNLENVYTIITLGQVLVFSVLICLFGYQVFVAAASTARRFIFVFLLSGSMFLLFMFTYSCNDVME

HSDNVAIGAYSALWTILPMMLRNDLIMVIKRSRRVCYLTANGFFPVSLETYTKILSTAVSYFTLLNN

>AmOr84

IIWTSFLMKIVGLWLAADRDEQRRRDFALIYTVGALFIIVCIGFRDIYFTWGNFSDSVYISCNNLYLMIVVLKVGVLYAH

KMEFFDLVTFTRNNFWRSYPDPEEELILAECKRICTIFVVVISFCAQGTCTGYMITPIIANVGRNESDRELPFNLWVDLP

VGLSPYFEILFTVQILCVYHVGVCYICFDNLLCIVNLHVAGQFRILQHRLRNLNESYRANVCHAKLRSCVIRHQTLTKYC

KQLENIFTIIVLGQVLFLALVICLVGFQLFLMDTPASRKVSLTLNFAGTLCQLLMFTYSCDDLIRESVNVGNAVFSGPWA

ELPMVVRKNLIIVVARSHRVCCLTAGKFFPVSLETSTAVLSTAMSYFTLLR

>AmOr90

KELDISVNLSSFFLRSIGLWIGDGSTNERRRKGMLAYTIWCTFFSTIISSRDLYFTWIYNGDILYALTNYMSVMMILLKI

CVIVVHKSEFINLILYMQRYFWNVNYDSREKEILNGCKKTCAFFVSTVTFIGICAILSYLTTPFTARIGNNESERILPFN

MWVNLPLSQTPYYELLFLIQIITLYYIGICYFCFDNVFCIMAIHLTGQFRILGYRFAKLCNIEHEMREKDTVLSKHVHTC

YEKFKEYVRYHQALINFYTKLENVYTMIILGQVIVFSALICLFCYQVLLANAPSARRSIFIFLLIGAMSLLFMFTYSCDG

VIEQSDNVAVGAYSALWTIMPMMLRNDLIMVIERSRRVCCLTANGFFPVSLETYTTILSTAVSYFTLLRN

>AmOr91

VSVSLTSIFMKLVGLWMAADQYEQRLRNISVTYNLVAILFALYLQTTDIYYSWGNFSACLFSVSNTLSLILPLLKIFILL

SNKEDFFRLIVYMQRNFLQGNYDDHERKIVFGCKRKCTFFICFFTFFTMATIVSYIAGPIIGNIGKNESDRVLPFNMWIN

LPLSMTPYFEITFTLQVLSLYQIGVSYFCFDNFLCIMNLHLAGQFKVLQYRISTIADRVIEKEEKKEKADRVIEKEEKKE

KLIIDSLYFSNKCYTTFKKYIRQHQALIAYCRKLEVVFNWIVLEQVLMFSLLICLDGYQILMANGDIKTRLTFSFHILAC

LCQLLMFSYSCDCIIRESVSVATAAYGGPWTLLPMMMRKDLIIVIMRASIPCCLSGKGYFIVSLETYTSVLSTAASYFTL

LRN

>AmOr92PSE

DVAVSLTSIFMKFVGIWMXQYQQRMRNIMVAYNVIAIFFALWIQTMDMYHSWGNIRACLFSTSNTLSLILPLLKIFILLC

HKQDFFRLVLYMKRNFLXNYDDHERKIVIGCNQKCTFFICFFTFLTIATTASYMVIPLIVNIGKNESDRVLPFNMWVNLP

LSMTPYFEISFVLZVLSLYQIAVSYFCFDNFLCIMNFHVAGQFKVLQHRISTIADLTIKTEEKKEIADLTIKTEEKKEKL

IIDSLHFSNKCYTTFKKYIRQHQTLIAYCRKIEVVFNWIVLEQVLMFSLLICLDGYQILMADEDIKTRSIFSFHILSCLC

QLLMFSYSCDCILRESVSVATAAYEGPWTLPPMMMRKDLIVVIMRASIPCCLSGKGYFIVSLETYTSVLSTAASYFTLLR

N

>AmOr93PSE

XIFMKLVGIWMAGNGZEXITLFTAIIFSGYNLZMYFILGDFSACLFFISIILSSIMLLLKIIILFSHREDFFHLILYMKR

NFLXNYDDHERKMIGCNZKCTSSSVSSRFSRWRPLLLTSSVRLLVKNIGKNESDRILPFNMWVNLLLNITSYFEITYTLQ

XFSLYHIGVSYFCFDNFLCIMNLHVAGQFQVLQYRISNIIYXRFNEKKEKQHQALIYYCRKLEEVFNLIVLEQVLMFSLL

ICLDGYQILMADGDVKTRLIFSFHILGFLCQLLMFSYSCDCIIRWTLXPLLSMTSSRRMIRKDLILVIMRSNVPCYLTGR

GFFIVSLEMYXVLSTA

>AmOr94

SFSLATFFLRVVGFWLASSRLEEWFGNATVMYSIITIIFSMWVQMRGLYFSWGDFGVCTFIVCNSLGLVMDLLKILVVFV

HKKKFLGLIAYMQKNFWHLDYDQRENSIIADARQLCVYFVCVFSFFSQSTVFSYMFMPMISNIGKNESDRILIFNMWLDL

PLSMSPYFEIIYVIQALCLYQVGICYLCVDNMFCIMCLHLASQFRILQYRLANVSNVEDENMNSSNRCYAILKNCIRYHQ

ALIQFSITLEEIFTIITLGQVLIFSTLICFVGYQVLLVNMTLSWRISFLCFLITNMCQLWMFTYSCDCMTRESVNVASAV

YCIPWTRIPMMIRKDLQFIVVRSRRACCLTGCGFFDISLETYTKIMSTAMSYFTILKQ

>AmOr95

DFSINLSSIFIKLMGIWMANDQSEKYVRNVTILYSIIALLFGLWLQITDMYYSWGDFSECIFSMCNMLSIAAPLLKLITL

VVHREDFFYLILYLQRKFLHGDYNDYERNIVLNCKRKCTFFTCSLTFTTLATVVSYVINPLVANIGRNESDRVLPFNIWI

DLPLTITPYYEITFVLEVISLYHIGVSYFCFDNFLCIMNLHVAGQFQVLQYRISNIIDSIDKEKKEKDSCYFASKYYAIF

KKCIRQHQALIAYCRKLEEVFNLIVLEQVLMFSLLICLDGYLVLMADTSTTTRLIFGLHITVCLCQLLMFTYSCDCIIRE

SLSVATAANRGPWPMIPMMMKKDLILVIMRSGTPCCLTGRGFFVVSLETYTNVLSTAASYFTLLKQ

>AmOr96

AFTVASFYLRVVGFWLTTSRLEEWFRIGVVGYTILAITFSAWVQIRGLYFNWGDFSACTYIACDGLGLVMDFFKIFSLFI

YEKKFLGLMVYMQKNFWHYNYDEKEDLIVKDTKRITAYFVCILTFSSLSSIFTYMFRPLLTNIGRNETDRILIFNMYLDL

PLSISPYYEIAYTIQIAALNQAGSCYFCFDNIFCILCLNVACQFRILQYRIANVKGNPDANKNSSDECYKAFKNYVQQHQ

ALLDFCETLEEIFTIIVIGQILMFSILFCFLGYQVILADLTPSYRISFISYLFAGMCQLWMFTYSCDCITQESAKIASAA

YASPWINLPMMLRQDLQIVVMRSRRACCLTACGFFPISLETYTKIMSTTMSYFTLLKQ

>AmOr97PSE

SYYKTFKKYLKFLGQYPNQSRWNKEFNTNVMICSLISFLIPGRVYISIVEKNLNALMEIIPIVFATISCAIKLLNHRINK

KNFDKLFDLMSKEWEMKNDRNQTCILDEFTKQGNKFAEIYKNVLLSALLLFLLLPLFPSFLDIVFPLNETRQQFQIFKMK

YFVNEDEYFYPIYFHSVWSSFVIIMITVTIDSLYMLIIHHASGLFAMCGYQIAKATECNDRHNENHNENELFRQCVMTHN

KAYKFFEIMNKSSRNSYFLQISLTIIGISIIAVQIVMYLHKPEEAFRISLFLIAAQFHLFIITLTGQVIADQSSKLSNNM

YCTTWYRMPPNVQKIFHIIQIKSSKPCKLTAGGILELNLENFGIALKTCMSYFTIFLSL

>AmOr98

YYKVLKKYLQFLGQDPYQECKYRNIITIIMLISMIAIFIPTTFEIYVHDKNTDAVMECLPNLCASLSSVVKILNVHFNRE

NFNKLLEFVVKEWDELKLNELHILEEITIQGSKIAHLYRNTLLSFLILFLLVPMYFPILDMIDALNQTRSRQQLLRVNYM

FNADDYFFYVYLQLAWGAIVIVMIVITVDSLYIIIIHHVCGLFAVCSYEIQKTVKDLTEKCSYKELKNCVIKHKKAIKFY

NILNNSSQLSYLLQIGINIMGISTTAFQLAVNDTRPQEAIRNAVFCGANQFHLFVLSLPGQILLDHCAELSNTIYCSMWY

KLPVKIQKMFNIMLMRSKKSCALTVYGLYELNMENFGTTFKACISYFTMMLSLK

>AmOr99

PYYKMMEKYIRFLGQDPRQKDEFRNIIVFILIISIASIVIPTTLELYIRNKDVDGVIECIPHFIASSISAVKLLNLHFNR

QNYNILFHFVTKKWQQLKSTYELNALDETIMQGKKMAQLYRNTLFSFLILFLVVPLVSPILDIVHPLNQTRSRQQLLRVN

YIFDIDDYFFYVYLQLAWGSIIVVLTIIAADWFYILIIHFNSGLFAVCGVQVLEATMLFAVCGVQVLEATMNSNLISKDA

FSENSSYEKFRTCVIMHNEVIEFYNILNENCQYSYLIQVGLNMLGMSTTAVQTVINLDRPDVAIRSAVFFGADQFHLFLL

SLPGQILLDHCADFANAIYDSTWYGTSLEIQKMLYMMQIRSKKLCALTAGGLYDMNIENFGITFKTCMSYFTMIMSFK

>AmOr100PSE

YYKSLKKWLILSGLYPPKNIIIILVAISIISVTLPLAIYTSLHAKNIDAMFECLPSLGVCIVAMFKLQNIYNNSENFKKL

FTFVAKQWYQLKLNNEIRILEEIIMQGNKMAQIYKNTLLLSMTIFFFVPLIFPILDIVYPLNETRPRQQLYRVNYFFNHE

DYFFYVYFQLVWSSFVCVIVIIIFDWLYILIIHHNSGMFAVCGYQIQKIFAEKIHIYEQFKNCLIVHSEAIQFFSILDES

SRNTYLFLVGTNIMATSISAVQVVLNLDKLEVAIKSAVFLIAAQFHLFILSIPGQILLNHYSNLKNNIFMSSWYNMPIEV

QKMFYVMQIRCKKPCSLTACGLYEMNMENFGTALKTCMSYITMILSLK

>AmOr101

PYYKMMEKYIRFLGQDPRQKNEFRNIIVFILVISIASILIPTTLELYIRNKDMDGVIECIPHFIASSISAVKLLNLHFNR

QNYNILFHFVIKKWQQLKSIYELNALDETIMQGKRMAKLYRNTLFSFLILFLLVPLVSPILDIVHPLNQTRSRQQLLRVN

YIFDTDDYFFYIYLQLAWGSIIVVLTIIAADWFYILIIHFNSGLFAVCGVQVLEATMNNLVSKDAFSENSSYEKFRTCVI

MHNEVIEFYNILNENCQYSYLIQVGLNMLGMSTTAVQTVINLDRPDVAIRSAVFFGANQFHLFLLSLPGQILLDHCADFA

NAIYDTTWYGTSLEIQKMLYMMQIRSKKLCALTAGGLYDMNIENFGITFKTCMSYFTMIMSLK

>AmOr102

YYKMVEKYFQLLGQDPRLKNEFRNFIVTVVVISISGNIVPTSIELYTCDKNMDAVIEGLPHFIAATISAVKILNVYFYRE

NFDKLFQFVTNEWNKLKLNNELHILDKTIIRGNRTAHLYRSALLIALVLFLLIPLISPMLDVFLPLNETRPRQQLLKVNY

LFNDDDYFFYVYLQLAWGSIIVVVTSVAVDSLLILIIHHCSGLFTVCGYQVQKVISNAKSFNGTVLNNYYTYEQIKNCVI

MHDEAIQFYNILNESNRNSYLIQVGLNMLAISATAVQAVVNLDRPEEAIRSAVFCGANQFHLFVLSLPGQVLLDHCSEFS

NNIYSCIWYRAPVRIQKVLYIMQIRSKKLCTLSAGGLYEMNIENFGITFKTCMSYFTMIMSLK

>AmOr103

YYKMVEKYFQLLGQDPRLKNEFRNFIVTVVVISISGNIVPTSIELYTCDKNMDAVIEGLPHFIAATISAVKILNIYFYRE

NFDKLFQFVASEWDKLKLNNELHILDNTIIQGNKMAQLYRSALLTALILFLLIPLLSPILDIVLPLNETRPRQQLLKVNY

LFFNDDNYFFYVYLQLAWGSIMVVVTIVAVDSLLILIIHHCSGLFTVCGYQVQQVTGNAKSLNKIVSNNYNYTYEQIRNC

VITHDEAIQFYNILNESNRNSYLIQVGLNMLAISATAVQAVVNLDRPEEAIRSAVFCGANQFHLFVLSLPGQVLLDHCSE

FSNNIYNCIWYRVPVRIQKVLYVMQIRSKKLCTLSAGGLYEMNIENFGTTFKTCMSYFTMIMSLK

>AmOr104

YYKLLEKYMKFLGQDPRQRDGFRNIIVIVMVASISGILIPTSLELYTRDKNMDAVIECLPHLIAAATSVVKLLNIHFNRE

NFKKLFEFITKEWEKFELNNQFHVLEEITIKGSKMAQLYRNTLLSFMVLFLLVPLIFPFLDIVHPLNETRPRQQLFRVNY

LFNHNDYFFYIYLQLAWGSIIVVMIIVTVDSLYMIIIHHSSGMFAMCGYKVQEATKYQNLFNDENYTYEQLKNCITIHNK

ALQFYNILNESSRNSYLIQVGLNMMGISVTAVQTVVNLDRPEEAIRTAVFLGAEQFHLFVISLPGQVLLDHCTELANNIY

SSTWYRIPVKIQKVLHMMQIRSKKPCSLTAGGLYEMNMENFGITFKTCMSYFTMLMSLK

>AmOr105

QEYNAFDIAYYKTLKLYLTICGINPYQNNSISIIIIIMIISVCMSFLCPTSIQLWESNKDFDNIIQNIPQVITVIASMIK

ILNIYSNKMQFKNLFYSLAQDWKLLESKEELIMLDKFTQYGSKLALLYRRTLLTFLVIFLFLPLCNPILDVILPLNETRS

RQNIFNVNYILDNYEYFYIVYMHLSCSAVIIVIIIISVDSLYISIIYHACGLFAACGYQIQKLTKVHYQIQKLTKVHTIE

KNGPNISNIDYEEFKQCVIMHYKCLQLYDVLEKCCRNLYLIQMGLNIMIISVTCVEVVVFLDRPKEAIRAIIYVIAQQFH

LYAISLPGETLLNQSSKLADKIYDSEWYKIPMKVQKVLHIMQIRSNKPCILTAAGLYEMKIESFGITIKTCMSYFMMFLS

LR

>AmOr106

ERYLKINKIYSIIVGMWPNQKRKTIPRIFVELIAILAHLTQGGNMVLFFSLTLAMDQIPFLIAAILLMIKYNNFIINEQK

FKELFVSILNDWQKKKTHEEEMILEKYADKSLFFILIYVVNAYFCTVLFLILPLTPILLDIFIPLNESRPRVQMYPAYYY

IENEADYYYPILIFSIVSLLTAMCVYIATDTTLVYVVQHACGLLTLAGYRFRNSLNDLYSMRYVVQHACGLLTLAGYRFR

NSLNDLYSMRKDSKMDEKIYRRMCYAIKTHKRALAYLTKIEDFYSMNIFAQVGASILCLTVTLMKIATIKWSMETNQYYG

FVIAQVVHIFFLTAQGQFVIDSHDNVYRDMYEPYWYNVQYKIQAMFVLILRRNLNPPLLTAGGLMQLNLNTFAQVVKTSV

SYFTVLKSV

>AmOr108

EDQYLRINKFFGQLVGVWPYQERFTKFCIRLTIFAIIILTLTTQIYQVIVFCTLDALSNQLPYLNALFILLFKQYNYILN

EDKLRDLLNDIIFDRLMVRSKKELEILNMYSRRATTLCIFYEVIVIFSAIMFIMIPTIPPILNIIMPLNESRDREFIYPT

YFFIDEEKYYYPILTYMATVILIVSSVYLACDTNLVQIVHHGCALLAISGYHFKHAVDDMKFSNDETYKKVKQSIKAHKT

AVEYVDKIDACHIYYFLLIIGMIVLAFTGTFLKLSTMEIEIRFFTFCGYTVAQLTHLFFLTIMGQFLINANDEIFNTIYE

AHWYNGSSRTQSLYVLVLRKCLNPPTLTGGGLIVLNLDSFVQILKLSFSYYTVFRS

>AmOr109

DQYLKINKFFGQLVGVWPYQKKFFKTCIRFITFTIMIFSLATQISRVIVFYSLDVLSDQLPYINAGIVTLFKQYNYILNE

DKLRELLHDIVSDRLIERSKEELEILEMYSRRTTALCALYKVMVYSCAFMFLVIPTIPPILNIVAPLNVSRSREFIYPTY

YFVDEQKYYYPILTHMIAVILVLSSVYLACDTNLVQIVHHGCALLAISGYHFKHAVDDVKDETYVKIRQSIKAHKTAVQY

VDKIDACHIHYFLLVIGMIVLAFTGTFLKLSTMEVGIRFFTFCAYTIAQLIHLFFLTIMGQFLINANEETFKTIYEADWY

NGSSKMQSLYVLVLRKCLSPPKLTGGGFVALNLDSFVQILKASFSYYTVFRS

>AmOr110FIX

NQYLKINKFFGQLVGVWPFQERFIKTCMRFIVSVIMLLDLATQISRVIVFYSFDVFSDQIPYLNAAIICLFKEYNYVLNE

NKLRELLNDIISDRLIRRSKKELEILELYSRKATTLCILYKVMVYSCAFMFLVIPTIPPILNIVAPLNVSRSREFIYPTY

YFVDEQKYYYPILMHMIAAILVLSSIYLACDTNLVQVVHHGCALLAISGYHFKHAVDDVKICDEDETYTRVRHSIKAHRT

AVEYVDKIDACHIYYFLLTIGMIVLTFTGTFVKLSTMEMGIRFFTFCAYIAAQLTHLFFLTIMGQFLINANEEIFRTIYE

ARWYNGSSKTQSLYVLVLRKCLTFPKLTGGGLIILNLNSFVQILKASFSYYTVFRS

>AmOr111

DQYLKINKFFGQLVGVWPYQQRFIKFCIRFITSVIVVLTLAAQISRVIIFYSIDVLSDQLPYLDVGFVLLFKQYNYILNE

DKLRELLNEIISDRLIKRSKEELEILEIYLKRARVLSTVYEVSIFFCGFMFLLIPSIPPILNIISPLNESRGRELIYPSY

YFVDEEKYYYPILMHMIAVALILTSVYVACDTYLVYIVHHGCALLAISGYRFKHAVDDIDETYTKVRQSIKAHKMAVEYV

DKIDACHIHYFLLIIGMIVLAFTGTFVKLSSMEVNVRFFTFCAFTVGQLTHLFFLTIMGQFLINANEEIFKTIYEARWYN

GSSRTQSLYILVLRKCLSPPKLTGGGLVALNLDSFLQILKASFSYYTVFRS

>AmOr112

NFLKVNKIFGLITGVWPYQNYRSKMAERFISVTVMMSGFVTQFAYLVLNPTMDKIATNLPYSIASFGTFVKMGNYFLDET

KLTTILNHIFEDWATIKSKEEYEIMYKYSRRGLFITISYFLHIGVTETFMLILPMVPPILDIIVPLNVSRKRVFLYPAYF

WLDDEKYYVLLLGHMIITLLMICFIFCACDTNYVYAVQHACGLLAIAKYRFKNVCKNLKEEIKYKSICESIKAHQHALKY

LKLIENSYHTYLFVSMGLLIMAISVSLLEVANGKNGSRELVQATFLFAQLFHTFILTVQGQFVINELQDVYESIYESPWY

TFSPRIRSLYVLSLRSCLNFPTLTAGGLIVLNLQSFAEIIKAAVSYYTVMQT

>AmOr114

MKFIGIWPEERKWNQASNYLVLIPFLMILCFICAPQTINLTIISNDFNLVIENLSMGITITLSLLKTIAFWINGKPLKSL

LNCMANDWIKVTSKTEQETMARIASITRNTIIKSTVMCHTVVAFYVFLRYISMKYNENKLLFRAYFPYDTTVSPNYELTI

LGQFVAALYAATSYTAVDTFVAMLILHVCGQLSGIKNELSRLPTYDSRLPTYDKKDLKRRLKEIVQKHEYVNRFAETIEN

CFNVMLLIQILGCTVQLCFQCFQAIMSEYLFFQLMFLLVYVFYVMLQLYLYCYVGERLSVESMEIVNAAYNTEWYTLPTN

ITKMLIIVMCRAKSPLTVTAGRFCSFTLQLFSEVLKTSMRYLSVLYAVK

>AmOr115

FAMGWNRFNLTLLGVYPEPRSRNSRLMSLIFWFTTLVTFTFICAPQTANLILKSTSLDEVIENLSINIPIVFALIKQIVL

RYYKKALTELLGEMLADWSGPIGDQDRETMLRNARLSRAISIVCSTLTYFMLLAFVSLQVWSNAENASETDLGGLLHPAT

FPYETSKSPNYEITWLGQLMGTVLTAICYSCFDTFLAVLVLHLCGQLTVLGTALEDLVNRNDYKTFEQRLSSIVNRHNHL

SRFAVIVEDCFNITLLVQTLICTAMFCLTGYRMITSDVPIVGIIFFIIHVIYTMLHLFIYCYVGETLLGQSTGIGLSTYH

CNWYDLPSRRAVLLMIVIRRANVSFQITAGKFSPFSLEFFNAVLKTSAGYLSVLLAMK

>AmOr116

KKINSNKHLQNNLSIIYYIGLWPDRVKYKYLYNLYTICSLIFVGIIIVSEIIYIIINWGKIEIMMTGLTILMTNSTYAAK

VIYIICRYERIKNLVDITNSEIFNRDNDKYKHIISYYNWQGIFHHIAYQGFASICIFSYSCIPLQSAFSGKSKQLPIAGW

YPYNVTSTPIFEIACLHQVLVILINCINNIAIDTLITGFIIITCCQLTILKKNNINIEKSPSKIYNKFYENLKHCVKHSI

IIFDFTKQIQDIFGIIIFFQLFVNCIIVCLAAFNLSQIKNYITEFFGSLLYICCMIYQIFIYCWHGNELYLHSMKICLSA

YKNNWWNNNKNFNYALLIIMIRTQIPLIIIVGKVMELSLQNFLLILRTSYSIFTLLKTF

>AmOr117

MKKPFNKSIDYYILPNKIFCSIAGMWPIDEKSSSKIFAYVRLIFGLIIVNSFFIPQIIIIVMNWKNIKIIAGIGCVLTTI

TQVLFKMIYLIARREKTYSLYYKIRNLWNSSNDSKERPYEEFAYWARIFSIIFYSSCMCNVFTFSIAAAIDYFKFEYNAN

NTENNRHLPFIVWYGTDISASPSFEIVFFYQIISSSICASVISGLDTSLMTIILHVSGQFKLINIWINNIGIEINCNPNY

MRKLKVDLIKCIRHHQQLIHVVNNVNNLFTPIIFIQLLTSGIEICLSGYAVLDNNSANADLLKFISYFISMGIQLLLWCW

PGEILIQESQEIGHVIYNIPWYNLPPIYQKYLYFMIVRSQQYCRITALTFQTLSICTLSNVFNTSVSYFTLLRQM

>AmOr118

YSLRIFGIWPDSPYPKLKIITWIIILPTFLVFQYWYCITHIKGLIDLLDGLSLTLSNTLVFIKLIVIWFHKRTFYEILMS

MKEDLNNNKHATENKRIIMDKSMLSSRISNFLISYFAITFFLYSGVALVIFDEDQGKFLVRMEFPFIATISPRYEIILIT

QFIFESFIVYGAATSIALIAALILYVGSQIDLFCQNLNLTFHSYKKRESQDTIKDIIVRHQKIIQLSKNIETIFTYISLC

QFVSNMLVICFISFVLTVSEQTIVLIMKCLPYYIAVNCEAFILCYTGEYITSKSENINKAVYNFLWYNLKPRDVRIMLMI

ILRSQKQLTLTAGKFICLSLEAFANMLKASASYVSVLYA

>AmOr120

NQTNTMNIRNYIFINQLVLKFVGFYPINILRYVICISCIMFIVIPQIIMIYINWNDLNIVMETGSTLLTILLAALKSIVW

IFNRKKLEFFIEFMLTDYWKIIETNVFEYLQEYAIYAKNITKGYFFSMCNALLFFFSLPIIETLTKNENLNNFTIKNFPF

AASYPITFYKFPFYEIAYISQILATSICCLMMLAIDSLIATALLHTCGHFTVLKENLKNLDTYIYDLTKTNLKTNSKTNL

KTNSKYINKNLYEIKTQIIYIIKHHQLVLWFCDNMEKNFHLILFLQAITSSLIICFVGFQISIALTERSKFLESFSHLIV

SLFQLLLFCFPGDILIRQSFNISIAAYSMQWYQLPTFIKDEICMIILRSQRPSFITAGKLYIMHLENFTAILSTAFSYFM

MLQSF

>AmOr121

HTSESKKYSKDYEWAVRLNRFSLNVICLWPVEENMRKQSWTKLHIMTCFMLITFVCTIPCLCALKQCNNLMEVTDNLAYS

IPLIITTIKFIVVSSKKKVLSLIVNMVAKDWAKLKTDHEKDIMIRRARIARIINIFGYILICILIWLLMILPRFGITIRY

VTNETDAKKLFPLPSYYIFDVSETPYFEIMYALQSISLLIAAFCYAGVDNFFGILILHICGQLTNLRFQLANIKESEALI

AIVKDHIRLIRVIELLKMFVEQIINLIITIFKYFFKCLKMYIEEEQFSLFRIIYLICNFTNTFLQTFLYFMAGQMLVTQS

EEVHNAAYECEWVSLKYTKAKSLIIIMARSKKPLYLTAGKLFPVTMLTFCNILKISLSYISFLLT

>AmOr124CTE

FDNQYRTYRIILKIVGLWPYDNSIYVRIQRICVLIYFLIGVLVQIFSFVKSEISLRNCIVTFSTTFPTLLFCLRYIYCLT

LFSYAKLLFDDICTEEHLLQDTTEIQIQTKYLDISSHIIYIFC

>AmOr125

DVFDKQYRIYRIILKIVGLWPYDKSIYVWIQRICLSMYFLIGVIFQIIVLVKSEITLRNYIVTLSAIFPLLLFFIRYIYY

ITMFPYAKLLFDDIRTEEYLLEDETEIQIQTRYLDISSHIIYIFCCMTFAFIAAAIIFLVNLIILDLRNSLNEFRFYFDL

LFFFDDQSAYIKIFLILNFMNTLFGLLSITSTESLTNIFSYYVCRQFNIVNYRIRKIIEDLSTPNLSKLLYKALTAMDDR

MEILGSTLIVIYHLMIAFYNNHCGQLIIDSNLGIFNELFASTWYRIPLKAQKLLLFMILRSSMDCELRLSGLFTPSYAGL

TSMMSSSFSYCTVIYSI

>AmOr127

FDNQYRTYRIILKIVGLWPYDNSIYVRIQRICVLIYFLIGILIQIFSFVKSEISLRNCIVTFSTTFPTLLFCLRYIYCLT

LFSYAKLLFDDICTEEHLLQDTTEIQIQTKYLDISSHIIYIFCWLSFICAAASCIFIVNPVILDVIMPLNKFRLHYSVIF

LSNDRRKCIDIFLVLNSIIIFFGLLSLICSELFTNIVSYYICRQFHIVSYRIRKIITNLSMSNLLYNALITMDNRVEIFC

STIVVTYHLMTALYNNHYGQLIINSNHDIFNELCASTWYRIPLKAQKLLLFMILRSSMGCEICLSGLFTPSYAGLTSMMS

SSFSYCAVIYSI

>AmOr128

FDNQYRTYRTVLKIVGLWPYDNSIYVRIQRICVLIYFLIVVLVQIFSLVKSEISLRNCIVTFSTTFPTLLFCLRYIYCLT

LFSYAELLFDNVHTEEHLLEDTTEIQIQTKYLDISSHIIDIFCWMSFICVASTCIFMLNPVILDVIMPLNKFRLHFSLIF

LSNDRRTYIDIFMVLNLIILIFGLLSIVCSESLTNIFSYYIYRQFDIVSYRIQKIIADLSMPNLYKALITMDNRMEIFGC

ILVVAYHLMIAFYSNYCGQLIIDSNLGIFNELYASTWYRIPLKAQKLLLLMMLRSTVGCELHLSGLFTPSYAGFTSMMSS

SFSYCAVIYSI

>AmOr129FIX

FDNQYRTYRIILKIIGLWPYDNSIYVWIYRLCLLIYFLVVVLVQIFSLAKSEISLRNCIVTLSTTFPTLLYCLRYIYCLT

LFSYTELLFDNIRTEEHILQDMTEIQIQTKYLDISSHIIDIFCFICVAATWIFILNPVTLDVIMPLNKSRIHFSLIFLSN

DRRTYIDIFMVLNLIILIFGLLSLICSESLTNIFSYYVCRQFDIVSYRMRKIIVNLSLYNALIIMNNRMEIFGSALMVMY

HLMIAFYNNHCGQLIIDSNFGIFKELYASTWYRIPLKAQKLLLFMMFKSSVGCELRLCGLFTASYAGFTSMMSSSFSYCA

VIYSI

>AmOr130

FDNQYRIYRIILKIIGLWPYDNSIYVWIQRLCLLSYFFANIIFQIVSLLRSEITLQNSILILSITCPLVLFLLRYIGSIA

CFPTIKIVFKHIRTEENIVQDSIESQIRMKLIDDSHHIINIFFWMTYTTIVIFIIYVSYPIILDFMIPLNESRTHFIYYI

TTFSHNQSIYLDILDFNFMFTGIFGLLSVACSESITGIYSYYICILLKIVSYRIQKIIMNATLIKKNQLEMLFCFTLVAI

HLVIIFLNNYNGQIVMNSSQELFDELYNSMWYFMPLKAQKILLLIMLQSTTKHAFNILGLFTPCYAGFSTMLSSSFSYFT

LMYSI

>AmOr131

FDKYYHTYRIVLKIIGLWPYNNSVYVWIQRLCISALFLGNIIFQILSLIRSEITLRNCILILSTTCPLIIILLRYISFII

FFPMVKLLFHHICVEENAVQDLIEIQIRMKYIGNSRHMIEILLRVTFLTITLFSIFLLYFVTMDFIMPLNEFHRHILLYV

TLFSVNRTIYFYILYLNFLFVITFGLLSLICTESIVGLYSYHTGMLFKIISYRIRKIITYLTMFNVSSKQVNAITIKKDQ

LEILITLIIFANHLMIMFLCNYNGQILINSNEEFFHELYIPVWYFVPLKVQKILLLIMIRSSMACIFHIFGVFIPCYVGF

TTMLSTSFSYFTLIYSI

>AmOr133CTE

DSIEAQIRTKYISDSRHMIEILLWMAYATITLYSILGLCPIIFIILLNESPIRMLHYVTLLSVNGTIYFYILCLDFLFII

IFGLLSMICTETIVGIYIYHTSILFKIISHRIQKIIAYLNMFNLLSNQIESKLAELYLVNAITIKKDQLEILISLIIFVN

HLVIMFLCNHTAQILINNNEEFFHELYISVWYSVPLKVQKILLLIMIRSSMACIFHICGVFVPCHAGFTTMLSTSFSYFT

LMYSI

>AmOr134

FDKHYYSYRTVLKIIGLWPYNNSIYVWIQRLWISALFLGNIIFQIVLLLRSKITVRNCILILSTTCPLIIISLRYICFIL

FFPMIKYLFHHMRMEENIIQDSIEAQIRMKYIGDSRHMIEIFLWMAYANITLYSILGLYLIIFIMPLNESPIRMLHYVTL

FSVNGTIYFYILCLDFLFVIIFGFLSIICTETIIGIYIYHTGVLFKIISHRIQKIITYLTIIDLSSKQIDSKLAELLVNA

ITIKKDQLEILISLIIFTNQLVFIFLCNHTAQILINNSEEFFYELYISVWYFVPLKIQKILLLIMIRSSTACMFHIFGVF

VSCHAGFTTMLSTSFSYFTLMYSI

>AmOr136

FDKHYHTYRTLMKIVGLWPYNNSIYVWIQRLWFLMFFFGNIIFQIMSLLTSAITLQNCVLIFSTTCPLIIVLFRYIGLIL

FFPTIKLLFHHMCMEEAMIQDSIEAQIRRKYIDDSCYMIDIFFWMTYVGIALCSILLLCPITLDFIMPLNESRTRIVHYV

TIFSDKSIIYMDILCLNYMLLAILVILSATCTESILGLYSYHTSIMFKIIGHRIQKIVKYLTMFNLSSKQIDSKLAEAIV

IKKDELEILISFIFFTTQLVITFLNNNCNQILIDNSQELFIELYISMWYFVPLKVQKILLLIMIRSSTACMINILGVFTP

CYIGFSKMLSTSFSYFTLMHSI

>AmOr138NTE

TLRNCILILSLIFPLTIILVRYISCIIFFSMIKLLFHHMRMERNMIQDSTEIKIRKKYINDSCHMINIFFZIIYGIAVLS

IIFILYPMTLDFIMPLNKTRIHIIHYITIFPYNRTIYLDILSLNFMFVGIFGSLSLACTESIFGLYCFHANILFKIISYR

IQKIVTYLTMFNLSSKQIDMKLTELVNAIIVKKDQLEMLISFMILMAQLIITFLCNYNNQILIDNSQELLDELYISAWYF

VPLKVQKILLLIMIRSSTSCTFHILGVFIPCYTGFSKILSTSFSYFTMIYSI

>AmOr139PSE

LVNMILIRKDQLEIFMFFIFLVSQMMIMFICNYSSQILIDNSQELLYDLYISMWYFVPLKVZKILLLIMIQSSITYMISI

LGVFILCHIGFSTMLNTSFSYFTLIXST

>AmOr140NTE

LYSVWGNYDAVIECMPPIISIFQSASMYFNGIFNTKKIKNILLFIKNDHKYYINRPENIILQKYDLQGKKITFYYILYVY

TTLFVYLLLPTIPLIIDFITSSNHSQKRNFLFELDYGMDKQQYFYYISIHSYIGTAIVANLIASCDTMYMLYAQHAYALF

AIVSYELKTIHNTNNLINVTDHHLLNVTDHHLLEKYKNITLLSKDEKKVYRKLFICIKNHQNAIKYSNLLESLFTKSILV

QLFFNVLCLSITGVETVIKLGNLSEMMRFGSFTFAQAVHIFFLCLPGQRLLNHSEELHVSACEVTWYIFPKKYQNLYKFL

LARSLIFSKLTAFKVTTLSMQTFLAIIQTAMSYFTVLLST

>AmOr141

DEKTKREFDKTIDLNLFLLKLCGIVPCGDGFARNILAWLAFSCLTIYSISYVHEFITNTTNLTTALESVAMIISIVGGHA

RYTILLWFRDICQTMLNVCEIFWSNLKPHEKKIVQSYTRKTTRLTRWYLASCVLTIAFYAFLVLFGSLFDQSKDFEHNDS

SLVPKRHLPYAFFLDVQKTPWYEIVYAVQLIGMFNVGFTCVGVDTVGALFILIICGYFDTIQSRIENLHSFDTSLSSSLL

NILSRKITTAKMSDIKTEASNSVQRNLRMCVIHHQLLLRFCEDIEHLTSGMFFIQVIASTYNISLVGFKLLEDTPDKFKY

ITQLIILIIQLFLCNWPADLLLSKSIDISRATYSMPWYGYSYNLQKITNILMIRSQKAVRLTAGKFIGLSLETFASMIST

AASFFTMVRSM

>AmOr142

KAILFTKLSVALTCSWPPSPTKAQHLFFNALWCIAFLTSVMLFLPLLAAIYVYRKHPVILGKTVSLTAAVAQVTIKMIIC

RLQQKRFQMLYSEMENFCKQATNEEKIILQRYVDRYKYFHSFYILWSFLTTIFVICGPLYTVQTFPTHAIYPFSVRRHLY

KGLIFFHQSLVGFQVSSGMAIDTQIALLLRYATARFEILGIQFNNAKSDEFDACIKKHDELLRYSREIRQSIKFLILATN

GTTVIAVIFGSLNLIANQPLILKALYAIVVFSASVELFMYAWPADSLMHMTMKMATKVYNMDWYGKDIRTQRKILFIILR

SQKYESFGINGIVPLSLSYYGKYLYTSLSYFNALRI

>AmOr143

LYILELIGTWPINPSKRRIIFRNIFWIFSILNVILLMTSLMLAVVYFRNDILMSLKTASEMAALLEVVLDLILCKWNNSE

FQVLIEEVKSFVEMANEYEIKILQGYVNRYKKFFSTVSMGYISTAISFSLMPLFSAQKLPADGWLPFSTEPFGIYCIIYF

NHVYCILQTAFCIFVDFTIVILFSFPAAKLDVLRSKLRHVNNYDNNYDTLVSCIKEHQKIIGFVEDTKATVETLLFKTNV

TMGSTVMCGAFPLLNNQSLAAISQFLPLVLSGILHLYVIAWPADDLRESSVQFSNSISDIQWLGQSNKMKSCVIFMMMRS

QKAFLIRMSNLLPLSLEYCSNFITTVSSYFMAMRTM

>AmOr144

IRQVLHILELTGTFTCTWPINPSKKYIIIRNILWTFTILNVIFLTISLMLAIFHFRSNIPKSMKTASEMAALLEVVLDLV

LCKWNNSELQVLIEEVKSFLEMASEYEIKILQGYINRYKKFFSTVSMGYILPASSFILMPLFSAQELPAEGWLPFSIEPL

GIYCVVYVNHIYCILQTSFCIFVDFTIVILFSFPAAKLDVLRSKLRHVNNYNNYDMLVSCIKEHQKILGFVEDTNATVET

LLFKTNVTMGSTVICGAFPLLNNQSLDVVTQFLPLVLSGMLHLFVISWPADDLRESSIQFAESINDIQWLGQSKKMKSCV

IFMMIRSQKLFLIRMSSLLPLSLEYCSNFVTTVSSYFMAMRTM

>AmOr145

VFYILELTGTFTCAWPINPSKTYIIIRNILWIFTILNVIFLAISMIFAIFHFRSDIPKSMKTASEMAALLEVALDLALFK

WNNSELQILIEEVKSFLEIADEYEIKILQGYINRYKKFFSTVSMGYILPASSFILTPLLSDKELPTEGWLPFSIEPLGIY

CAVYVNHVYCILQTLSFCIFVDFTIVILFSFPAAKLDVLGSKLQNVNNYNNYDMLVSCIKEHQKILGFVENSNATVETLI

FTNITMGSIVICGAFPLLNNQSLDVVTQFLPLILTGMLHLFVIAWPADDLRESSIQFAESINDIQWLGQLKKMKSCVIFM

MIRSQKLFLIRMSSLLPLSLEYCSNFVTTISSYFMAMRTM

>AmOr146

EKAIAFTQFIVSLSCCWPLPSTKLQTRCFKIIRSLLFLNSLLLFFPLLYFVYVNRNDNTTFCKAMSLSLAVVQVPLLSSF

CITQYDRFQRLIKEMKFCCENANSYERQVFQGYAKSYATFYGVSAIWFYWCALIVVVGTLFISDPFPTNAEYPFPVHFEP

VRSIVFVQQALVGFQCSAHLCVNIFCALLLLFAAARFEILMNELRAVENIESLIKCIEKYYAIRRYAEEVVNSARYTTLI

TLCICGVESVFGGIIFIGRQPFTVKLQFLTLSATTLLAVFMCAWPADYLMDVSENTMRAVYESEWYKRSLKLQKFVLFAT

IPQTPVILKVRCIIPFSLNYYCSFITNVLSMFTALR

>AmOr148

WLSVALSLCWPLPATRKQIVCIKILQIGAIISAFMVLLPLIYAIHLNIHNLINLFKCICLLICVFQNIIQTIICFIKYDV

LQRVVEEMMTCVKEEQLYKVLCIYVKKCNIFYGGTIVLTYGAATVFVLGPTFLPISFPWETEYPFQINDTSRNIIYIHQF

FFTYQCAAHICLSLFGALLLWFAAARFECLVEELQKITNIDMLIVCFKKLLLLRRYAEEVVSCIRFLVFYAIAVGTFMLT

LSGIIMIINSPILVKIQFIIICMSSLMEIYMYAWPADHMQDASINILRSAYNSIWYEQSLDMQKDLLIILMYQRPVILSI

NVLLPLTLRYYCSYVANAFSVFTALRAV

>AmOr149FIX

QMTFKKIINIIWFSVALTFCWPLSATKIQIFVFRILQIISIINAFMLIVPLSYSIYLHYDDIAIIFQSLAILVGLSQMII

QTVILFIKYNFLQRVVEEMIICIKEAQQYERKIFCKYIENCNIFYGSSLTITYLVVIIYIMGPIVLPTPFPVDTEYPFHV

NSTIIKIIIYLQQSLLIFQCAGHLCISIFCALLLWFTAARFECLIVELQKITNILIICIKKQLRLRRYARNVVNSFRFMI

VYAIGVSTFALILYGIIMIVKAPLIMKIESVTLSFVLLLQIYIYAWPADHMKDMSINVSKSVYNIIWYKQTLRMQKDLLN

VLIYQRPITFSVDCILPLSLRYYCSYVSNIFSIFTAV

>AmOr151

YITWVSVALTLCWPLPAGKIQVFMFKALQIISIINAFILLLPLLYSVYLHFDDVIIVSKCVAVSIGLTQVITQTIICFAK

YDSLQHVIEEMIICIKAAQQYEEKIFHKYIEKCYTFYACSITCMYLTATAFIIGPAFSPASFPIDAEYPFQINYTSVKII

IYLQQTLVGFQCAAHVCLSIFGALLLWFTAARFECLAVELQKITNILIACVKKQLRIRRYAKKVVISFRFIILYAIAVST

FVLILDGIIMIMKVSLIVKVQFITLSLTMLTEIYIYAWPADYMKDMSTNVSKSVYNITWYKQTLRMQKDVLNILVYQQPI

IFSVNCILPLSLRYYCSYLSNAFSIFTAIRV

>AmOr152

EKTIQIIWFSVAITFCWPISLSKTQVFIFKILQIISIINVFMLLLPLLYSVYLHFNDIIIVSKSIALSVGLIQVIVQTII

CFIKYDSLQHVVEEMIIYVKEAQQYEKKIFHKYIEKCHIFYGCSIACIYLTATVFVIGPVFSSASFPADAEYPFQVNSTS

MKIIIYLQQSLIAFQCAGHACLSIFGALLLWFVSARFECLAVELQKTTDLIVCVKKQLHIRRYARRVVISFRFIILCAMG

VSIFSLTLGGIIMITKSPFIVKVQFITLILTLLTEIYMYAWPADHMKDMSINVSKSVYNTIWYEQTLRMQKNLLNILMYQ

QPIILSINCILPLSLRYYCSYLSNAFSIFTAIR

>AmOr153PSE

XYAKKMINWFRFIIFNAIGLSILVFTLASIILIMISICMYIVVCSCMYNFINKNYYMYIWPADYMTDKSINVSRKIYDSM

XYKQMLKMQKNLLKXLIFQRPVXIYRLZLLSLILRYYCLYLSNVFSIFTALH

>AmOr154

NVIYIIRLSVAICCCWPRPFTKNQIFAFKVLQISTIISAFMVFLPLLYSIYLHNDNIIHVFKCICLSIGITQLIVQTLIC

FIKHNSLQRVVEEMVNCVKQAQQSEIEIFYKYIEKCKIFYGSSIAFSYLAATAFMLGPAILPISFPLEAEYPFHVNESLI

TIIIYMHQSLVSYQCSANVCVSIFGALLLWFTVARFECLIEEFQKCSNIMMIACIKKQLQLKRYAEEIINCFRYIVLYGI

AVTTFALILCGIILLMNIPLIVKIQFVIICITIMTEVYMYAWPADYVKNMSINISRSVYELSWYEQTIEMQKNFLNVLVY

QKPVIFSISCIVPLSLRYYCSYLSNVFSIFTTLR

>AmOr156

LKRVIYITWLSVALCFCWPVSASRNQIIVFRFFQIFTIISSCLGSLPMFHSIYLHQDDIVIVAKSISIMVVLIQLIVQTT

ICAIKHDTLQHIIEEMITYMKEAKQYEKKIIQKYVSKCYILYGSAIIISYLTTTIFILGPIFLPISLPFYTEFPLSLNNT

AVYIIIYFHQCFFAYQCSATVCLSIFGALLLWFVVIKFECLIMKIQNISNKDDMMVICIKKQLQIRRYAKEIANCFRHII

FYTIIATSFNMILAGIILIMNPLLVIKIQFMITCFTALIEVYLYAWPAQYMDDMSKNVSISAYNLKWYEQTSEMQQNILI

MLIFQKPISLSINFLMPLSLRSYCAYLSNAFSIFTALR

>AmOr157

YLVWLSVAMTFCWPLPPARKRIVGMKVLLIISIVNGCAVILPMLYWIHLHLDDIISLFKCICVALCLVQYVAQTIVCLVK

YDTLQRVVDEMMGLIEERRMYEILRAYASKCNTLYGASIASIYVCGTSFIFAPLFLPNPFPFETEYPFHVNTTTRIFIIY

ASHVLVIFQGTAHMCLCMFGALLLWFTTARFECLIGELRGVLVVCLEKHSRLKRYAEEVVSCIRFLVFHAILLGTFVLTL

CGIVLIINSPLIVKAQFIIICVCILLEIYLYALPADYMYDMSMNISRSVYDSIWYEQRLDLQKALLTVLAFQKPIAVSIN

VLLPLTIRYYCSYVSNALSIFAALRTV

>AmOr159NTE

RLEHAMDTYKGQNEIEIYYIICTKLIKAIEVYKLAVKFVFFCQIFFFFQGNFNLHFPLLKVVYMLQQLENIYKLCINLIL

LIRKFCFLFLITYLGQNIENHSNEVFEKCYDSLWYTAPVATRKLLLIIMINIMKPCQCKFGGLFKGNIEGFAQIIRICIS

YFMSLYST

>AmOr160

VISWSKRLLGLSGLWPDNRNDVRFFLYITYVVIFTWLEIVTLVQNIHDLEKTLKNITLSFPTILIVLKAVMFRMNMHLVL

PLLTVVKRDVNEYRSAEERRTVVWYNVAATLFSTSSALSLFFVPTLFYAKPIIGCLLSKYNNCTLPFELPMKVNNVYEIT

KLQTYALFCVYLIPTSTLLTIGATGADSLLVTLTFHLCSQLSIVAYRMRNVKIYFPKMKALVERHTELLRLANILANTFS

SLMFVQTLGLIFSLCIVVYQLLMTSGEDMNTIHFIIYSCAVILLAFCYCFLGECLINESSEVQMACYFTNWYDLPEQYTR

SLIFCIARAQKPLYLTAGKFYVFSLETFAVIVKASMAYLSVLKS

>AmOr161

EFRNEEYDQLIKPIMITGKIISIWPLAESRITITFRRFHLFCMFFLVIVMSVAVTADVVHNIDDLDEATECALICTAFYL

CVVRLLVYSFHQKDMLYVVNTMKEDWLSSSDQDRLIYAEKTMFAFRLAKYFITTVAITIVMFMSVPILEIYVIGNSDKVL

PFRGYFFINQTVSPIFEFLYLFNVTAGGFGGSMIAGATSFNLVVIIHGSGKFAVLRRRMEALNDNVIRHQQAIKFADTLE

RIINLLALGQFVISTGLICFAGFQITSMMEDKGRLMKYSTFLNSAILELFMFSFSGNGLIDESEGIGESAYNSGWIGSRF

CRSVQIMMMRSKIPSKITAAKFYSMSLESFSAVLSTSFSYFTVLTATK

>AmOr162PSE

MKRLMDIMQEDWKFHARLRNEYEILCEHYAIARKITTSFVAFLLGLTTPFGAMPLLLNIGDALGLCNISDDRPLAFRVEY

FVDVDKYYYLLLVHSSIGTLGYTVIVLAINSIIIVYVLHECGLCEILRESNNMICLRFAKILEDTNTTSYLLQLGFNMIC

ISFTQFQAIINIEDTPKVLRYVSITIALLCDLLFVSWTGQQLSNSTERIFEYTTNGKWYQSSISCRKLLAIMLSKSIAPL

RLTACKLYTLNLESFTTIAKTSVSYTMVLCSL

>AmOr163

EVCLRLIGVWPYSSYRIMQRIFWTIIMGNSTVFQLWYCYFKTADLFDLLDGITLTLSNTVTFFKLIILWFNYRTIHNILT

IVFEDWNNRALTDKKKQLMVDNTRLSSRISNFLFGIYSVTCILYSASIALISDDIDNTNNELILNNKKLLLKMKLPFDFT

IFPLYEFVIVAQFVFECFVALTAGMLMAFSAALVLHIGSQIDITCQELIEIPRHLKNIIVKHQRILRLSENVKYLFLYTS

LIQFLSNILVICFLGFILVNAEQESTIFIKCFPYYIAANCEAFILCYTGEYLMFKNESIVHAAYDTLWYNLNPRDSRIVL

LILIQAQRKLILSAGNFVTLSVQTFASMQKVSASYISILMT

>AmOr164FIX

PVEISLRLIGAWPNSSCQILKYIWTIVMSIFLIFQYSYCIIHIKTATLIDILDCLSITCSNTLLLLKFIIIWFHKRVLFE

SLIIIAEDWDNCKFEWNMEIMMQKAILSYRIAKLMLIIFICSIFMYAVSTFFGPDIGASHSDQKKFLLKMEFPFEATVSP

LYEIIITIQLVMQFMFATMAGMFMTIIATFVLHIASQLDIICDRLSEILDEHKEQEQELRIRIIKKLIAKHQRTLNLSEN

IENIFTFISLSQFFFNILVICFVNFILVTSEQAPTVISKCFPYYIALNFEALILCYTGEYLSSKSENISWIAYNSNWYEL

SIYEIRVLLLLIMRSQKPLTLTIGKYMKLSLETFANMLKISASYASVLYAL

>AmOr166JOI

VKYGLYFAASWPGASFSILHKFFWTIIFCTLHISQYSYLIMHYKYDALTEIIDNISICLPHSLVCIKLFTAWTQNTLIRN

ILLSMEEECQKYAIMDTDNLISKTAYLSYRLTSTIICTCVASTVCYAIGIFSHQEVNVTSSRELLLKMNLPFDTNKSPIY

EFVVIIQYFYQVSAAFVFGVFAAFLLMIVLHVGCQIDIMCQTLMKTTHRDDQKKLKFFIKRHQEIILLAEKIEKFFTYIA

LSQLISNTLITCCLGYLIVITLGNNIILIKYIMFYVAVCSEAFIYCFAGEYLSIKSKLIADTAYEFLWYNMNPNESRLLI

PIILRAQRGFTFTFGKFATLSMESFTAIMKASGSYMSVLLAM

>AmOr167JOI

PIVIGLRLIGIWPKSSYEIIVRFMWVIIMMCAQIFQYQYIINHIGFDNLADLIDSVSTTLPYSLLCFKLISFWTKREIFE

NILIGMYHDWTNAFATDFIVEDMIKKTELAYYCSNLILSIYAIAVFLYVGVFLELSHDHDQENRSNLSPELLIKMDLPFT

YDESPIYEYVFIVQFIQLFFIASSIAVLDALIITLIFHIGGQIEILHKTLKNISINDEKPESSRIIIKSLIDRHYRIIIG

SEYIESLFSYIALMQLICNTLIICCIGFLIVVALNSNKLLIRISFFYIAITLEAFIFSIAGEYLSNKSLSVSISAYESPW

YLLSPKNRGVMILLMVRSQRRLTITAGKFMDLSMQGFANVLKASVSYVSILYAM

>AmOr168

LPMTNNEKSSVISKIYFVIVWIIQLMYLASCTLGLFNVSWERALKDGTVNMVLLLEVIILNVYLHSRKKLLRELIGKLNQ

ILINEDEIFRNVTISTTKMLEKPSRIYIIVNVISIIVWISSPLIKLFQKDEFYHEDFVMPAVFSNQPFSTGVFISGVFLQ

LFGGEYLLFRKISLDLYTMHLNLLITSQYKYLRIKFATILKENGESAKSAKDNDKTIRQEMKLLIRHFETVIEMTGILKK

LLSPNIGILYLNYVFRFCFLSFMFATTLSEKLTYTIIVSYTTGALIQFYILCYCIQDLFEASTSIADDVVYEKWYSYDVR

FQRVILMISLANELKCKISNFQNIDLTLPSFMSILNQAYSICLLFLKTK

>AmOr169

RLNTFVNAVSGILPITDKKRLSIVLKIYSILVWTIELSYLAACILGLFNVSRERALKDSTVNIVISLEVFVLIVYLHNRE

NLLRELIGKLNCLLIVDDETLRDVTIGTVKPLEKPLRVYIIASVGSLMIWASLPLAKIFRKSEFYYTDYQVPAVISEPFP

IGVFIGGVALQIFGSAYTLLRKVSLDLYTMHLILLITAQYKYLRIKFAAILEQETPQNVPCEYDKMVKQEMKLLTRHFEI

VVEMTVMLKKLLSPNIGILYINYVFRFCFLSFMLATSSGMHFEKCLLVSYTIGALIQFYILCYCIQQLLEASTTVADDVV

HEKWYLHDVKFQHIILMITLANKLKCKLSSFRNIDLTLPSFMSILNQAYSVCLLFLKA

>AmOr170

LPMTNNEKLSTILKIYFVVAWIIELIYVAASFLGLFNVSGEKALKDGTVNIAISFEVIVFNIYLHSRKKLLHKLIGKLNH

LLITEDEIFRSVIIDTVKPLEMPLKIYVIASVASLMIWILSPLIKLFQKDEFYYEDFIMPAVFSQQPFSNDVFICGIFLQ

LLGGEDTIIRKISLDIYTIYLCLLITAQYKYLRIKFAIILKEEREITKDHYKRNDNVRQEMKLVTRHFETVIETTTILKK

LISPNIGFLYLSYVFRFCFLSFMFAMTTAKYFEKCLLASYTIGALIQFYILCYCIQRLFEASSSIADDVVYEKWYYYDVR

FQRVILMISLSNELKCKISNFQNIDLTLPTFMSILNQAYSVCLLFLKA

>AmOr171

RLYKKYASFVKLFLLIGGICPITRELNVIYRYIPIWAIFSCFIELCAVGNSSLQNVENIPLLTASLILIGTILNVITKTS

CFFIHRKKLHQVNDIFNSILEEILNEIYIKSIVLFYLQIIYRLIYVQTVLMFVTTIIYSMKPMIIKLFHDANITNVQYPL

PLFGTFPWKINSMLIWQLHYFFDVNILWFIFSVSVSVDAFFGFCMFRICVILRFLSFEFRSSIDDKNKRNKEKDDKNKRN

KEKSYQQIFRECVEKHVLLLKCRNIIQEVYGPIILLVTITNALSMCSIIFQLFQVNGINIKIGTFMIYLMLKLIQTFLYS

WPGDVIFTESEFLRRNVYCSCWYDKNTSFAKYFLLVLAQRPIVLKACSLVQVTMDLLAKIMNTTISYYFLLETM

>AmOr172PSE

QYYNINRILMISVGLWPYERTIYSKMLNILIFFFLSTAIFIQVMSFIMLKMDINLTLNSLSYTSCTCICIMKYFNCLFHI

KDIKNFLDEIKNDWNSLRNIEELRIIHEYSKTIKKITICFVIIIVPLQLVFFLNVFGNNILDILIPLNHTRPRTVPIEIY

YFIDQQKFFFFFGMHLNIITSFGGLVYIAIETISMGMIQHLCGLLKITSFRISHTFVANIPKISSTERSIIIRK

>AMQ13062.1_odorant_receptor_7_[Culex_pipiens_pallens]

MNVQPTKYQGLVADLMPNIRLMQGVGHFLFRYVTGPIFIRKLYSWWNLTMILLQFFSIAANLVMNTGDVNELTANTITTL

FFVHSVTKFVFFAVNAEGFYRTLGIWNNPNAHPLFAESDARYHSIALAKMRKLLVMVMTTTVLSVVAWITITFFGDSVKG

VLDKETNETYIVEIPRLPIKAWYPWDAMSGPGYVFSFIYQAYFLLFSMCQANLADVLFCSWLLFACEQLQHLKGIMRPLM

ELSASLDTYRPNSAALFRAISAGSKSELILNEEKDPDSKDFDLSGIYSSKADWGAQFRAPSTLQTFENGMNGEKGNPNGL

TRKQEMMVRSAIKYWVERHKHVVRLVSAIGDTYGAALLLHMLTSTIKLTLLAYQATKIDGLNVYGLTVIGYLVYALAQVF

LFCIFGNRLIEESSSVMEAAYSCHWYDGSEEAKTFVQIVCQQCQKAMTISGAKFFTVSLDLFASVLGAVVTYFMVLVQLK

>ANW12106.1_olfactory_receptor_protein_1_[Phenacoccus_solenopsis]

MQKLRKQGLVADLWPNIRLMQLSGLFITQYYEDNSTLMRLIRKIYSWITAILVFTQYILLVIWALTESYDADQRAAYSVT

ILFFTHPLIKFIYFSTKTNRFYRTLSAWNNANSHPLFAESNARHRASTLARMRKLLMYVGSITIFATVAWTVITFIGESV

RTVPDPESENGTITIEAPRLMVPAWYPWDVMGGLTYYLTLVYQFYWLFITMSHANLCDILFCSLVLHSCEQLKHLKEIMG

PLIELSAALDTQVPNAEALFRVPSSGSKAPLMENEEYDNYNTNYNNYRNNALSTVAGGGGSGPNGLTKKQEILVRSAIKY

WVERHKHIVRYVASVSEMYGMALLFHMLITTFTLTLLAYQATKIDGVNVYAFSTIGYLVYTLGQIYTFCIYGNELIDESS

SVMEAAYSCSWYDGSEEAKTFVQIVCQQCQKALSVTGAKFFTVSLDLFASVLGAVVTYFMVLIQLN

>AOE48007.1_putative_odorant_receptor_ORco_[Athetis_lepigone]

MMTKVKTQGLVSDLMPNIRLMQAAGHFLFNYHSENAGMSNLLRKVYASTHAILIVINFACMGINMAQYSDEVNELTANTI

TVLFFTHTIIKLSFFALNSKSFYRTLAVWNQSNSHPLFTESDARYHQIALTKMRRLLYFICGMTCLAVVSWITLTFFGES

VRLITNKETNETLTEVAPRLPLKAWYPFNAMSGTMYMIAFGFQVYWLLFSMAIANLMDVMFCSWLIFACEQLQHLKAIMK

PLMELSASLDTYRPNTAELFRASSTEKSEKIPEAVDVDIRGIYSTQQDFGMTLRGAGGRLQNFGQQNANPNGLTPKQEML

ARSAIKYWVERHKHVVRLVASIGDTYGTALLFHMLVSTITLTLLAYQATKINGINVYAFSTIGYLSYTLGQVFHFCIFGN

RLIEESSSVMEAAYSCQWYDGSEEAKTFVQIVCQQCQKAMSISGAKFFTVSLDLFASVLGAVVTYFMVLVQLK

>AOE48068.1_putative_odorant_receptor_ORco_[Scaeva_pyrastri]

MQQQTKYIGLVADLMPNIKLMKYSGLFMHNYTSGSSFFKKVYSCFHFTLILLQFFSILANMALNADEVNELSGNTITALF

FTHCITKFIFFAVNQKQFYRTLNIWNQVNSHPLFAESDARYHSIALAKMRKLFVLVMLTTVLSVVAWTTITFFGESVKFA

RDMETNETITVEIPRLPIKAFYPWDASSGMFYMISFVYQVYFLLFSTTQSNLCDVLFCSWLIFACEQLQHLKGIMKPLME

LSATLDTYRPNSAALFRSLSANSKSELIINEEKEPVDTLDMSGVYSTKADWGAQFRAPSTLQTFNGTNGMSNPNGLTKKQ

EMMVRSAIKYWVERHKHVVRLVAAIGDTYGAALLLHMLTSTIKLTLLAYQATKINGVNVYAFTVIGYLSYSLAQVFHFCI

FGNRLIEESSSVMEAAYSCHWYDGSEEAKTFVQIVCQQCQKAMTISGAKFFTVSLDLFASVLGAVVTYFMVLVQLK

>AOG12930.1_odorant_receptor_[Eogystia_hippophaecolus]

MMAKVKAQGLVSDLMPNIKLMQMAGHFLFNYHSDNSGMSTLLRKIYASVHAVFIVTQYFAMVANMAMYSDEVNELTANTI

TVLFFAHSIIKLIFFALNSKSFYRTLAIWNQSNSHPLFTESDARYHQLALTKMRRLLYFICGVTILSVFSWVTITFFGES

VYMLVNKETNETLTEPAPRLPVKAWYPFNAMSGTMYIVAFVLQVYWLLIAMAIANLMDVMFCSWLIFACEQLQHLKAIMK

PLMELSASLDTYRPNTAELFKVSSTEKSEKVPDPVDLDIRGIYSTQQDFGMTLRGAGGRLQTFGQNLNNPNGLTQKQEML

ARSAIKYWVERHKHIVRLVASIGDTYGTDLLFHMLVSTITLTLLAYQATKINGLNVYAFSTLGYLGYTLGQVFHFCIFGN

RLIEESSSVMEAAYSCQWYDGSEEAKTFVQIVCQQCQKAMSISGAKFFTVSLDLFASVLGAVVTYFMVLVQLK

>AOH73455.1_olfactory_receptor_1_[Adelphocoris_lineolatus]

MQKVKMHGLVGDLWPNIRLMQLTGHWLLEYHEETGGMARLIRIAYCWMTTFVVYLQYAFLVCFLILETYNSDEMAAVTIT

TLFFLHSVTKFTYFAIRSKYFYRTLSAWNQVNSHPLFAESNARHRAAALSRMRKLLMIIGVVTILAVFGWTTVTFLDDPV

WDKTDPDNVNETISVEIPQLMVYAWYPWDAKTGMTYFMTFALQLYWLFITLAHSNLLDVLFCCFVIFSCEQLKHLKEILQ

PLMELSAALDSVVPNSGDLFKSGSAGSNIALISNGDGGNDFDVRGIYSSQRDFSGFQGGMTNGTTVGPNGLTKRQELLVR

SAIKYWVERHRHVVKFVTSIGDTYGTALLLHMLTSTVTLTLLAYQATKIEGVDVYASTTIGYLVYTLGQVFVFCIHGNEL

IEESSSVMEAAYSCHWYDGSEEAKTFVQIVCQQCQKSLTVSGAKFFTVSLDLFASVFGAVVTYFMVLVQLK

>AOO35283.1_olfactory_co-receptor_[Rhynchophorus_ferrugineus]

MNTFKVAGLVADLMPNIRLIQASGHFMLNYHADNSGALHGLRLGYCCMHLLFVLLQFGCIFGNLVKEKDNVNDLAANTIT

ILFFTHCLTKFVYFAVRSKLFYRTLGIWNQANSHPIFIESNNRYHALALKKMRNLLYIIMIGTIFSASAWTGITFMGDSV

HYIKDPNNENETISEEIPRLLIKSWYPFDAMSGMPYYIALVFQVYYVLFSLLHANLLDSLFCSWLIFACEQLQHLKEIMK

PLMELSASLDTYVPKSADLFKAPNSASSQDNLIENEYNSKNDELNLKGVYSTRQELGNLTFRSGALQTFGQGGGGVGPNG

LTKKQELMVRSAIKYWVERHKHVVRLVTAIGDAYGVALLLHMLTATIMLTLLAYEATKIDGVNVYAATTIGYLLYSLAQV

FHFCIFGNRLIEESSSVMEAAYSCHWYDGSEEAKTFVQIVCQQCQKALSISGAKFFTISLDLFASVLGAVVTYFMVLVQL

K

>AOO35284.1_olfactory_co-receptor_[Rhynchophorus_vulneratus]

MNTFKVAGLVADLMPNIRLIQASGHFMLNYHADNSGALHGLRLGYCCMHLLFVLLQFGCIFGNLVKEKDNVNDLAANTIT

ILFFTHCLTKFVYFAVRSKLFYRTLGIWNQANSHPIFIESNNRYHVLALKKMRNLLYIIMIGTIFSASAWTGITFMGDSV

HYIKDPNNENETISEEIPRLLIKSWYPFDAMSGMPYYIALVFQVYYVLFSLLHANLLDSLFCSWLIFACEQLQHLKEIMK

PLMELSASLDTYVPKSADLFKAPNSASSQDNLIENEYNSKNDELNLKGVYSTRQELGNLTFRSGALQTFGQGGGGVGPNG

LTKKQELMVRSAIKYWVERHKHVVRLVTAIGNAYGVALLLHMLTATIMLTLLAYEATKIDGVNVYAATTIGYLLYSLAQV

FHFCIFGNRLIEESSSVMEAAYSCHWYDGSEEAKTFVQIVCQQCQKALSISGAKFFTISLDLFASVLGAVVTYFMVLVQL

K

>AOT85634.1_odorant_receptor_co-receptor_Orco_[Mayetiola_destructor]

MQVQQKQFTGLVADLMPNIRLMRFLGHFLHKLSNGPTFLSKLYSMMHLMLMLLQFFCIIINLALNTSEVNELTANTITTL

HFTHTITKFCYLAINNKNFYRTFNIWNQSNSHPLFAESDARYHSIALAKMRKNLYMITGLTMGTVASWSIITFFGESVKG

VFDKETNETYYVEVPRLPIKSYYPWDAMSGMKYIGTFVFQVYYLTFSMLACNLTDVLFCSWLCFACEQLCHLKGILKPLM

ELSASLDTYRPNSAALFRSLSANSKSELVNNDEYDQQDENGSVFVPKAEWGGTFKVPPNMGNPNGLSKKQEIMVRSAIKY

WVERHKHVVRLVSAIGDTYGAALLLHMLTATICLTLLAYQATKIDGVNVYAFTTIGYLVYSLGQVFHFCIFGNRLIEESS

SVMEAAYCCHWYDGSEEAKTFVQIVCQQCQKAMTISGAKFFTVSLDLFASVLGAVVTYFMVLVQLK

>ApOr1

KKDGLIKDLWPNIRLIQLSGLFISEYYDDYSLFRKIYSWITAIIIYSQFIFIVIFMVTKSNDSDQLAAGVVTTLFFTHSM

IKFVYFSTGTKSFYRTLSCWNNTSPHPLFAESHSRFHAKSLSRMRQLLIIVSIVTIFTTISWTTITFFGESVWKVPDPET

NQTMYVPVPRLMLHSWYPWDSSHGLGYIVAFVLQFYWIFITLSHSNLMELLFSSFLVHACEQLQHLKEILNPLIELSATL

DSSVHNPAEIFRANSAKNQSGTKGENEPNRKGPNNLTSNQEVLVRSAIKYWVERHKHVVKYVSLITECYGSALLFHMLVS

TVILTILAYQATKINGVNVFAFSTIGYLMYSFAQIFMFCIHGNELIEESSSVMEAAYGCHWYDGSEEAKTFVQIVCQQCQ

KPLIVSGAKFFNVSLDLFASVLGAVVTYFMVLVQLK

>ApOr2

QKFCIRWSVFFDSSSSRIETVLRTIQFSTIMITSGMTMTSVLIADNKKALESFTYFVICVFMLAIITFAIRTKRFNRAML

LMVVDEFPGYNRPMPKRKMAAIRTSYGDFTMKVIVSYLTLVLFEIPATAMVPLAAASLTDVKLGSQSTQMVVLWFPADTS

QVGMYAVSYVIQFLIVVTVKFIITGIMCSFSFFVSQMISEFQILSAYVEHAVEIVEDQSADKTTEQKLLDHVKNCVMLHD

RLIYFKDQLNESYGYIILLELMFSTLYFCLSAFNMIFVGNRFVMIKGLLTLSNYLAELFIFCMYGSMVEDAHMGLLRASY

SVAWYAQPVRFRQSLTMVMSRTQTPLQLTVGKVFIANLPLFLSVLKVSYSGVNALR

>ApOr3

YWLTLLLLNGINVYPGHGNRVVRIAAAAYPWMICAFWAFITTSVTTSLALRATSYQEAVEMLTYITGSSSTLALFAIGVH

NRPGLHRMLDAVRRDFWDDGRRPAADVLFSRFVRTYGAILPIANVMMCMTPVIWAARNGDIESPAALIFRMWTPWTRLTT

ARYAVVYAAQFVVSLSVLTSISGMVFAMVLFVTEMQVQVDTLVDAVQDLRRAAFDGLVKCIKHHQALITYFNRFKSYFNL

LFIVDILYIMVMTCLCASSVLMANGFSAFHIKMMSLLIIVVSQFFFYCLIGEQFSTMNQQIGDCVYFKLVKCKDPMLSRA

GLLVILRTQKPLQLTAMGIYTASLFTFTVTMRSAYAGLNVLYN

>ApOr4

KKNQRKFYQTLMTLAFFLDTSQYRYISRFVKQFYIFDWMVLVSVAAAFTILEGNYRMPFVMELIQYMIVGFYFTSIFVVF

IIKKEAIMSNYNCIQTKFIQWSNKRALHSNAAYKRNIKTVKSLSIPLAILSLSIALGPLISTINDIGKLPLDNRAHFVLF

WPTIVDTNKLSMYGIIYTLQVIFTIILYISVLSFNLGYMVFLNELITQFEMLLNGYKMDKQFQTLFIDCIRHHQIIIKFL

DDLKSYFKWMILIEIIVVQVILAILIYNLTKVNASLGYKVKIAGSILFNLLPICFHCHIGEVVLSLHTRLSNHIYNMPWY

DMPNKNKQLIVIMLQRTQRDLTLSSALFSSASRSLISKVIKQVYTILNVLLK

>ApOr5

QTIGEAFADNDEQKFKYAIIQHQKLLSYFNTMKKVFSKPILLSMSFNAIYFGLTTSFVIQARGYINQAIISICIASSAVI

NITIYTFYGSELMDLHDKILHVLFDNAFFYVSKSFKSSILIMITRVTIPLKFTVGYIFTINLNLLLKILKMSYTVLNVLL

S

>ApOr7

LVVTLFFAISAFLSIVYSEEDLSNRVYGFLWLFVEIHVFGLIVVRLYHQSQCRDMYDRSLLIEQGIPKNYRRTIAMVIAY

YCIMSTVHVTVPMLYTISSDSAQVGDPFAFPFADVLPIKTTNPTAYVCKYIVYAFPVYLTHLECCFMNVTFMYFTGVVKR

HFQILDQQVQEAVANEEDEQKLKIAIEYHQESLKFFKEMETVYEKPLIMTIEFCGLYIGLTGYIMIQCIIHPIILGLCIA

SSTASLITISIYCICGSNMYDLHDGILNSLFEQSCYSRNKSLKHLILMMMKRATIPLELKAGSIFKINSNLLVKILKFTY

TVFNLLLT

>ApOr8

DEQKLKIAIIHHQQVLKFFKDMKTVYEKPLLLTIEGFGLYIGLCCCAIIQVQGFVDQIILCMASCVAGFMTISLYCICAS

NMYDLQNGILNSLYEHRCYSRNKSFKRLNLIMMTRATIPLEFNVYSLFIVNLNLLVKILKLTYSVLNVLLT

>ApOr10

EDEQKLKIAIKHHQQVLKYFEDMKTVYEKPILMTIEFCGLYVGLTSCFVIQVQGFIHQIILCIVSSIACLMTIIIYCIYA

SNMYALHNGILNALFEHSCYSRNKSFKRIILIMMTRATIPLEIKAGSVFTINLNLLVKILKFAYTVFNVLLS

>ApOr11

RVVFFSCCEFTVSLFLAILTYLSLVYSKEDLSMRFYDLLLLIVEIVVCSLIVIRVYHQSQHRDMYQRSQKVGIPENYRRK

ITTVIKYHLVMSNVIVVIPVLCTISLDWVRMGDPFTFPSIDVLPIKTTNVTVYVCKYILYALPTYFAHLEMCFMNVTFMY

STGAVKGHFQILEERVEEAMATQDEEKLKIAIKYHQQTLKFFKDMKTVYEKSLLIAIEVSMLYIGLSGCTMIQVFVDPII

LGLCMGSCVSTFMTISIYCICASNMYDLHDGILNAIFEQQCFSRNKSFKQLVLMMMTRATVSLEFRVYSIFTINLNLLVK

ILKFTYTLLNVLLT

>ApOr13

DEEKLKIAIKYHQQALKFFKDMKTVYETPILITIEVSILYIGLSGCTMIQVFVNPIILGLCIVSCVSTFITISIYCICAS

NMYDLHDGILNAVFEHSCYSRNKSFKRLILMMMTRATVSLEFRVYSMFTINLNLLVKILKLTYTLLNVLLT

>ApOr15P_NC

EEKLNIAIKHHQQALKFFKDMKTLYEKSLLIAIRIYTLYAGLSGCTMIQVNNNVFDILKLTYTLL

>ApOr17

RLFKAICLHQILDKGGNRYYRLAFMVVMWVSLSVQIIQLVGLYFAVNDLQRFAFTTTVIFNALLCLSKGYVLVVNADRLR

ASLEVARYEFTSCGARNQRLVRRSRAVLSTILRTFAVLSWVTCFIWALTPLFAMDEYLQTNADGTVSRYRVTIYNVWLPV

VYNETTVWSLVYAVEVIACFVNVFSWLLFDSYVVTMCFTFNAQFRTVSASCTTIGHHSDSFRSPPSDDNNTFNCYDELIN

RIKDNQSIIKIYDDFFEILQPAILFQIIGGSYSVITLIFLTSLTMGFSIPVLKVFFGFLSVTFELFLYCYVFNHIETEKC

NMNFGLYSSNWTAMDLKFKKTLLFAMTNSSHRRVMKVTPMSIINLEMFANVMNMSYSIVSVLLNSR

>ApOr18

KAICLHQILDNGGNRFCKLVLMAFMSVSLSVQIMQLVGLYFAVNDLQRFAFTTTTLSYAFLCMTKDYVLLAHADRLRDSL

EVARFEFTSCGARDQRVVRRSRAVLSMVLRTFAMLSWSTCVIWALVPLFMMDEYLQVTNADDTVSYRVTIFNMWLPVVYN

ATPIWSLIYMVEVIACLFTSFSWLLFDSYVVTMCVTFNAQLRTVSASCATIGHSNCYDELIIHIKDNQNIIKKYDDFFEI

IQPVVLFQIIAGSYSVITLIFLTALSGWSIIPVLKVFFGFLSLTFELFLYCYVFNHIETEMNFGLYSSNWTAMDLKFKKT

LLFAMVNSAHRRVMKVTPTSIINLEMFANVMNMAYSIVSVLLNSR

>ApOr19C

MLFKAIGLYQLLCRGGYSVRSRRALMTALGLSFALHSFQVPYLYYALNDLQRFAYMAAVIIYGMMCSFKGYVLVTNADRL

WLVLNAADYGYTGCGHRDPSRLRRCRATLSALLRTFVALSYGTLIVWIVLPFFVDEYTGITNSDGTVTRYRTTIHNMQYP

IVYNSRPVWALIYVTELYVCIVNVFIWSLFDCYLVTMCFVLNAQFHTMIESNHYSDLISHIQDNQNLIKMFDVFFEVVRP

VVLVQIANGSYSVISLIFLTALMPVLSAAFLKFICGLISLTIELFIFCYGFNHIE

>ApOr20

MLFKAIGLYQLLCRGGYSVRSRRALMTALGLSFALHSFQVPYLYYALNDLQRFAYMAAVIIYGMMCSFKGYVLVTNADRL

WLVLNAADYGYTGCGHRDPSRLRRCRATLSALLRTFVALSYGTLIVWIVLPFFVDEYTGITNSDGTVTRYRTTIHNMQYP

IVYNSRPVWALIYVTELYVCIVNVFIWSLFDCYLVTMCFVLNAQFHTMIESNHYSDLISHIQDNQNLIKMFDVFFEVVRP

VVLVQIANGSYSVISLIFLTALMPVLSAAFLKFICGLISLTIELFIFCYGFNHIETAKSVLNFGIYSSNWTEMDLTFKKT

MLLTMMNSSHKRAMKVSPNSAVGLEMFARVMNMSYSTVSVLLNSR

>ApOr21

AKVIVGLTLVLQSMQVCRLYLARHDITMFAYIGVMIINGLMCLLKGYMVAAKADQMSATLTAANYAFTKCGGRDPSKLRL

CRARLSAILRTFVGLSFGTLIVWLTMPWFMASDYDDQPIWGVVYVIESIILTVNVFCWTSFDCYLVTMCFVFEAQFCTMS

TGYETLGRRRTGAKSSAPQKLNNVKISDVNYDDLTSHIRDNQNIIKQYDAFFDVVRPMVLIQIANGMYSIIMLIFLTLVT

SIFSAPILKFVCGLASLTIELYIYCYGFNHIEDGKSTVNFGLYSSNWTEMDLKFKKTLLLAMLNSAHKRVMKVSPNSIVN

LEMFTGVMNMSYSIVSVLL

>ApOr22

KLFKAIGMYQLLHGLNSDLCRKTAMMVVGLTVGLQLMQVFRLYLARHDIPMFANMAMLVVYGFMCLLKGYTLANHADRIC

ITLEVARYAFTDCGRRDPSLMRRCRARLSTILRTFVGLSFGTLVVWLVMPWFLASEYDGKPLIWAVVYVVESIILTVNVF

CWTSFDCYLVTMCFVFEAIFRTMSSGYEKVGQVKANVYDDLISHIKDNQKIVEKYKTFFEIVTPTVLLQIADGSYTIITM

IFLISIANGNSIMILKYVCGLVSLTIELYIFCYAFNYIEDGRSTVNFGLYSCDWTDKDLKFKKTVLLAMMNSANKQVMKL

SPNSIVNLAMFSRVMNMSYTIVSTLLS

>ApOr24C

NSIINLKFMKITGFYQLIRSDCSKFNMNIFKMLFIAQLSITTIMCLYSIYSCVNDVNQVFNYSIMIFATNFAIYKYYFII

KNAKTIWNFAHNMMSTNLLCYKDHTKEVYKVARTRCSTITLISLALWSSILLYWSLLPFSNNNTYLKVKFEDGVHYYRLN

ALGVVYPVDIFYNKYFHVFYIIEIVLLILWCKMMWVFDILMISVCISIEYQLKTIAESYRSLGLNHNELMSNKKSTMSSY

RSLGLNHNELMSNKKSTMSVEAISDLEVLIQDQQNIYEKMKNMYQILKPFTFIQVAAESFQIILQSCMILKFSLSLILFL

KFLLPGITYSCHLFLTCYLFSFVNEQKESMNFALYSSNWTDMSIKFKKLLLLTMMNNAENLKMKISMKRMVNMEMFA

>ApOr25

SKNEDNFMINMRLMKKTGFYQLLDSRSKVFGHNVFKCMSVVQILSSVAFIVANIYYFSDDINTVMMYSMLITSDVLSILK

LYYILQNSDTIWNCIQMTSIDDLSYKYHDRRILEEGRSKSTSYSILIMFMWLNLIVSWSLGPLFVTNYFLIVEQNDEIYR

YRFNIMNFAFPTDRFYNDNFMIYYGIEFITLVLWCHCTMNFDVLLLSMNITFKYQLKTISNSFKSMFDFKSDFKSLIYDQ

QRVIENMKNIYRVFRPVVLTQLASESLIIMLLSCIIMLNSLLSALNLRIFAAISTFLFHIYVICYLFDDVNEQKDSMNLA

LYSSDWTTSDLQHKILLLHAMMNNAENLRLQVTRNKIVNFQMFTYIQSYNYSL

>ApOr28C

EDKLMANTKLLKTIGLYQILNSSCKVFGYNIFKCITIIEAAAVILGCILNAFFCLSDLYVTTRYFTIGVMCAINTFKLCC

IIGYSDTIWNCLHIITSVKYLSYKYHSKRMLKGGQKKSKLILIFYLVVWIPVYVNWIFMPIVLQNSYLKVEAGNLIYHYR

TNILSLVYPTDKFYNDHFLTFYTMEFIIMLVGIHGAFMFDMFLILMCTTIACYLKTVANSFSTLGNVEDHFLIRYNETKI

INAFKIIIRDQQKVIENMNNFCKVIRPVILFQLAAVSSVIILLSIIIIMDNGFPLKSFTLIATLITYTLELYLICYLLND

VNEQ

>ApOr29

LMENIQLLKTIGLYQILDHSPKVFGYNVFKCVAVIEILTATVYAILNIFYCLSDINEATRYFTLCLIASVPTFKLSYIIG

YSDTIWNCLHITSAEYLSYKYHSRCILEVGRKKLKQFLILFVILWIVVFIAWILTPFIVQNSYLRVEARNMTYHYRTNIL

NLVYPADKFYNANFIMYYNIELTISIVWAHSTIIFDILLISMCITIEYYLKTIANTFSTLGNVENQLDDTKIINDFKIII

QDQQKVIENMKNIYKVIRPVILLQIVAESSIIILLSSITIMNSLSNFRFITSIFIYILHIYFICYFLNDVNEQKDSMNFA

LYSGDWSGKSLKYKKMILYAMMNSTDQMKLQVTKTRVVNLELFTSVMRTTYTVISVL

>ApOr30

KLMSNIPLLKMIGFYQILNSRSKVFGCNIFKCIATIEACILIAGVILNAFYFLSDINEATSYFTMGVMVSVATCKLFYII

GYSDTIWNCLHITSVEYLSYEYHSRCMIDVGRQKLKSILIISMVVWITTSIGWLLTPLIIQNSYLRVEKNEIYHYRTNIM

NLVYPTDTFYNDHFIMFYIIEFIVPIGFIHCTLIFDILLISMCSTIACYLKTIANSFSTLGNVENHCMGHEMKTLNNFKI

IIRDQQKVIQNMKNIYKVIRPVILLQISAATSIIILLSSMTIMNSLISPLNFKFMSTMLTYAMHMYLVCYLLNDINEQID

SLNFALYSGDWTSKSLKYKKMILLAMMNSAIKLKMQVTMTRIVNLELFAGVMRTTYSIISVF

>ApOr31

NDHTCTINLNILKQCGFYQIFDNSKKIFGWNVYRISFIALTVITQCLINCGFLFELEDTTDNIDLFLIIFSNSYFCLKVV

ILIINRKKFLELLDVTDLIFLKSKQCRNNIKILCKHRIRTLQLTNLYFMFCIFVIIEWIIFPIMINSFIAHKTENRRLEN

VVNRRYPVDVNTYKYYILFYVFEIIIGVKTVYLVLMVDILLLSIGWAIIIQYEVLAEAFKNIGYNENLQKDHDHDDYKYF

KSILFDQQQYKYFKSILFDQQQLDSKVKLYFPIVKPIVLMHVAINSVLFIMLSNSFLMVTYKIVNLFKIGTGILYICLQL

FLYCHLFDNINLKRKSVNLGIYSCNWTKMDLKFKKLLLLTMQINANYITIKASTKTIVNLPIFANVLMTSYNIVSVMVK

>ApOr34C

VAINLELMKRSRFYHFNPNGTKIFNCNAYRLLLFLYIVNCIVVFTLGFFVEMDDFTDLFVAIFVLINFFLCYWRICVFMY

NVNAIYDVLSVSRFDLLKSKHCCKNVNVLNDYRDRTIKITNYFFLFSSTVMSQWIIYPLVVIAFTMPEDEYGRFQNIMNL

RYPVSTYTYQYYFIFYLMEVMVAIFTMYAMIFPDILLMSVCWAIIAQQEVLTQAFKNIGH

>ApOr36

NNYTKIFNCNVYCLILFIYIVNCMVVYSIGFFIEMDDIISDVDVFLVVFNFFFCSWRICIILSKSHIICDVLNVAKFNFL

TSKQCFKHLNALYDYRDKTIKITNYFFVFSVIVLIQWIIFPIIVITFMESDVENSRSPNIMNLRFPVSTQTYQYYFIFYL

MEVAIAAFPIYVIMVTDTLILSFSLVTISQQEVINRAFKSIGYEENSQSKYEENSQSKYYEDFKSILGDQIQLNLKIKSY

YSIVKPIILANVAMSSTSFIIVTYVLIVVSNQILTIIKLGSSAIFICGQFFLYCYLLDSMNLKKESVNFALYSCDWTKMD

IKFKKLLLLTMRMNANNFMIRASPRKVVNLQMFANVISMSYNIISVML

>ApOr37

DHEVAINLALFKRYQFYQIFNPNGSKLLYKLTNVMFIVAVTTYNIFSAMCFFTDTVDTIDSVDLLLMIFIYSIILLKISV

LLFNADQIWELFDLTRFDFLTSRQCRKNVGILCKYRDRSITITNLYQNYSTMVFIIWMITPLVLNTFVVVGGPNQRYHNI

FNMQYPVSANYNQYYYLFYLMEIAMGIFVLNYSMIVDNFLISLCWVIIAQYEVITTAFEKIGNDCELTTLQNEKNNNTLQ

NEKNNNSFEAYEDLKSILMDQNKLYIKLKSFYRVVWIIVIFLIIIDSVLLIILTYSFVMISFSIFNILKISTAFFVFVIQ

LYLYCYLFDVLNDKKESVNFGLYCCDWTKMDLRFKKLLLLATKNNANTLKIKSTPNKIVNLQLFSSVMTTAFNIVTVMLK

>ApOr39

VAFNLSTYKQLGYYQLLDKGPHILYRTILKIFLLIVQFITIVMGFFIEMEDTSNSFELIIILTNCSLSSLKIYTLISNSK

IIWDLFDLTRIDFLRCSRHSKLKNFVKRCKKSTTITKWIARSFLVGLILWLMGPFIANEEHTEPNTVHRHKNIINIKFPV

TMKTYNYYFVFYLMEVAVGFCIVYGSVLIDAYLMSFCWIISAQYQSVTKAFATFGYNGSPKDIYKDFKSIIIDHQNIYLK

MKSFYAVVRPITLIHVFAYSCSLIMYAYVIVTILFIIAEIMKIVMTVSNVTMEVFIFCYLFELIDNKKEDVNFGLYSCNW

TGMDIKFKQLLLMSMMNNANRFKLKASPDVTINRPFFANVIHTCFKIVSVLIQT

>ApOr40

TTDKKYAFNLTLFKTIGYYQMVDNSKKIFGFNIYNVINITLVIFTSIMTLSGFFYKTDNIENNLQMLFYLSCIGLGNLKI

AITVYNADAIWKLFNVAHESFLSNKYCKHDKYKLNNCGKQFARIFPWYFFMFIMTAFAWSVVPIVVNNHAASNETQNNEN

AYMTNIANMRYPITVKTYTFYKGFYALEFIMVWYSAYGLVVFDLFIVALLQLLATHYEIISSAYENFKYKAENEDKLRKE

EIQKELVSIIFDCQTIYRKLETLYGFSRPIVLVYMVGDAIGMITMPFLIVMSSIFNTNVMAFSWTLFVVGIQSYMYCSLL

QNLNERKEDVNFGLYGCDWTSLDIEIKKLILLAMMNSSNNLKMKVTSTKFIDLPMFASIVRSSYSVTSVLIN

>ApOr41

IYDSNFTLFKLIGVYQMVDHSQKIFGFNVFHFVSMVFIIFTTSMTILGLFYNNSDVDTIFIMFYTVCITIGNLKVMIIIF

KARQLRNMLEITDESFLSNTFYKRNYYKIVKCGGQLSKFFNLYFSFLLITLTSYAIVPIVLNAHFIDGTTQNTETIQKIN

IVNLKYPFTVETYAFFKIFYASECIMLFYIGFGVFALNLFSMTILVIISAQYKLLASAFEVLEYRVNDEDDSLSDEKLLE

TFISIVSDNQIIHKKIKMLYDIIRPVGLIQLMADALGMICMPYLIVVYSLFNPETMKFVFTFGFAGVQSYMYCSLFQRVT

DRREEVNFGLYCCDWTGMDIHMKKLILFTMMNSSNKLKMNLTTNKCINLPLLSTIIRLSYRISSVMIN

>ApOr42

GHNVFHVTVMVMIGFTVVCLPFGLYYWANDVTQCIFLLITIVNFSFGCFKAFTLVRHSDDICRCLDVTRFDFSSIMSDPD

SARFFRKCRDASSTFTGWFAASSHFVLLVWTLLPFVVVGKGVEINNRDGSTSYYHFNPYNMYFLVSSETYRLHLVFHLVE

WAFGLCFVLIMVAFDTFMVTLCVAITCQMRGIGNAYSKLGHDCATASNVCSDGGIESKSNNEYLRDLKLIIKDHQAVLGK

MNDFYKIVGPVILPQLIVASFTIIFVSFIITRNYFNGMLLTKMCCFPIFFYQIYYTCHAFGNLSHQKNVMNFALYSSDWT

QMEIKFKKLLLLAMQMHANKLDMKLTDKLVINLELFTRVINMCYSIFSVLVN

>ApOr43

EKQYIFNMKLARIMGLYQILFNSTSFFGYNIYHVVTVFFVSFTFAISPIGLLYLRNDIIAIMYYMGCISNFLLSCFKMVN

ILYHSKDIWKCIDVTSFNYILYKHYDRNVFKNWQTRSIRITYIYIVIALFAFFCWIFSPCIMNKSVIAIRNIDGSYSKYR

MNIFNLYLIANETYNKNFYIFYVIEIIISICYVYFTIVFDVLMLLVCFAISYQLETISNTIKSLGHEIYTRDNIRSGNSI

KLKEKEKHGILYNDLITIMTDHQNVLKKLNDFYNIFRSITLTQIFIASSSHVFIWFIAAMSNADSILSFKLFIVLPLINF

QLFMTCSLFGTINEKKDSIIFALYSSNWTNMDLKSKKMILFNLINNASQLKMKFTNTKIVNLEMFSHTMRFCYSIFSMLI

N

>ApOr44

DSIFNLKLAKIFGFYQILDTETVTFLHNVYYGIFVFLIVYQWLLSAIVFLVNNSNIIQDMFYFGFTVNMLYGNYKMYIIL

NRSKVIWDCLSITKFDFTSYGVQGRHTLNVWRNLSIKYTNIYVMFYITVSILCVGFPVVFSNSFIIIKNHDGLSSAYRLL

VNLFLFISEETYTHFYVFYIVESLCLIINTLFIIIFDTIVNTLAIALTGQLQMISNAFESVGHKSLHFPNDNVDNKIKLP

NENIKYMDHYKDLKTLIIDHQNILKKYDEFLSIFRPTMLLQVFVVSCSIIFLWFIFLTSEDDFTQYMAAIFGIPFCTFQM

YMSCFVFNTLNIKKDSITFALYSSNWTEMDMKFKKLILLTMRMNAHQQKLQYTKTKIINMEIFYHTMRVCYTIVNVMINC

K

>ApOr45

IFNQKLAKIFGFYQILDTKTVTFLHNVYYRIFVFLIVYECLLSAIVILYNNNNIVQAMFYFGFVVNMLYGSYKMYIILSR

SKVIWDCLSITKFDFTSYGVQGRHTLNVWRNLSIKYTNIYAIFYLTISILCVASPVVFSNSFIIIKNHDGLSNAYRLGLI

NLYLFVSEETYAYFYVFHIVESLGLVINTLFIIIFDTIVNTLAFALIGQLQTISTAFESVGHKSLHFPNNNIDNKNKLPN

ENIKYMDHYKDLKTLIIDHQNILKKYDEFLSIFRPTMLLQVFVVSSSIIFLWFIFLTSEDDFTQYMAAIFGIPFCTFQMY

MSCFVFNTLNIKKDSITFALYSSNWTEMDMKFKKLILLTMMNNAHHQKLQYTRTRIINMEIFFQTMRVCYTIVNVMISCK

>ApOr47

NEKHHVFNIRLANLIGLYQTLDPETVKFRRNVYQIFVAFVALYLLVISFAGCLHLWTNTATSLLDLLITTNSFYASYKMW

IVVYRSNEIWDCLSITRYGFTSLNNRKWNHDILDRWRARSVRYTSLLAGAYFLTIVFYVGCPLVFGAAVIPIKNQDGSIG

YRLNVINLYLFVDETYNEYYNTFFFIEALFIVGLIITCLLFDTLLLTLCLGICCQIQMICSAFESVNHNSPSSAIDNNDE

KQIISNEHDLIHDELITIIINHQAVIKKFELFLTIFDRVMLSHIFVSSISLIILWFNLIMSSGDTTLKTIVAIPSFLFQI

FMVCYLFEDIHNQKDSIVYALYSSNWTEMDMKCKRLILLTMQLNQKKLRFTRTKIVNLEMFFKTTGHCYTVVSVLMN

>ApOr48

NEKHHVFNIRLANLIGLYQTLDPETVKFRRNVYQIFVAFVALYLLVISFAGCLHLWTNTATSLLDLLITTNSFYASYKMW

IVVYRSNEIWDCLSITRYGFTSLNNRKWNHDILDRWRARSVRYTSLLAGAYFLTMVFYVGCPLVFGAAVIPIKNQDGSIG

YRLNVINLYLFVDETYNEYYNTFFFIEALFIAGLVITYLLFDTLLLTLCLGICCQIQMICSAFESVNHNSPSSAIDNKDE

KQIISNEHDLIHDELITIIINHQAVIKKFELFLTIFDRVMLSHIFVSSISLIILWFNLIMSSGDTTLKTIVAIPPFLFQI

FMACYLFEDIHNQKDSIVYALYSSNWTEMDMKCKRLILLTMQLNQKKLRFTRTKIVNLEMFFKTMGHCYTVVSVLMN

>ApOr50C

NEKHHVFNIRLANLTGLYQILDPGAKCRGRNVYQIFVAFIALYILVVSFVDCLHWTNNTSMSLLNFFVATNSFYACYKMW

IVIYRSNDIWDCLSITRYDFTSLSNRKRIHGILDRWRARSVWYTSLLAGLYFSTMVIYMGSSLAFCNVLIPIKNHDSSFG

NYRLILNLYFFTDETYNEYYNTFFVVEAWLSVAVTIFYILFDILFVSLCLAICCHMQMIFTAFSSVNHKSLSSSIDNTVE

KHIISNEHELIYDELITIIIDHQAVIKKFELFLNIFERVMLSHIFVSSISLIILWFNLIMRSGDTTIKTIVAIPSFLFQI

FMACYLFENVHNQKDSIRYALYSSNWTEMDMKCKKLILLTMQMNQKTLRFTRTRIVNLEMFF

>ApOr51

EKNHVFNIRLAKLTGLYQMLDPGTTKFLRNVYQMFVALFLLYLLVSAVGCLHLWTYDTSMSILDFFLAINSFYACYKMCI

IFYRSDDIWECLSITRYGFTSLRKRNGHGDVLDRWRARSVWYTSSMAGAYCFSFVFYIRCHLIFGDAIIPVKNNYRWNVL

NLSFLSDETYNEHYNTFFVIEALFIVVITIFYLIFDILFLTLCMAICCQMQMICDAFKSVNHKSLSAIDNTDEKQIITSE

RDLIHDELITNIINHQAVIKKFELFLTIFERVMLSQIFVSSISLIILGFNLIMSSGDTTVKTIVAIPSFLFQIFMACYLF

ENIHDQKDSIKFALYSSNWTEMDMKCKQLILLTMQLNQKKLRFSRTKIVNMEMFFKTMGHCYSVLSVLIN

>ApOr52

NENNHVFNIRLAKLIGLFQILNPGSKFLGRNVYHIVVAINMLFVCIVAFASGVYYWSDVLVGVDYGWKGITALFLTYKMW

KVVYHSNDIWDCLTITRYDFTSQNLRDRQILDRWRERSVWITNTMAIAYLMSLVILLSGSLMFRHDTLTVKNHDGSVGNY

RQNIMNLYFIVDETYNAHYKTFYFIEMLFTVGGGTLFTAFDVLLVTLCLAISCQFQVVNAKFESVGYKSKISDNKDEKQN

ISNEHDLIYDELISIIKDHQEVIKKYYELLTIFKRLMLLHVFYSSISLIVIWFIFIMSFFVTTMKIICLIPSFSFHLYMT

CSLFDNLHKQKDSIIFALYSSNWTEMNMKCKKLILLTMKMNQKKLKFTRTKIVNLELFYNIIRSCYNVVWFLIN

>ApOr53

NNHVFNIRLAKLTGLFQVLSESIKFLGQNVYHIVVTVILLYMGIISVSGLYYWADNILLSVDYGWKGITALFSTYKMWNV

VYHSNGIWDCLAITRYDFTSHGLRNRHILDLWRERSVWITNTLTIIYVSTTVLFAGSSLMFHDNISTVKNHDGSVGYHQN

LFNLYLIVDETYNAHYETFYFIETLFAVGLATLFIAFDLLLVTLCLTVSCQMQVVNVAFESVGNKPLIDNADEKKNISNE

HDVIYDELISIIMDHQAVMKKYNDLLRRFKRPMLLQVFYSSTSLIVIWFCFLMSAASEVTTIKIICSIPSISFQLFLVCS

LFDNLHKQKDAIIFALYSSNWTEMNMKCKKLILLTMKMNQKKLKFTRTKIVNLELFCIIIHNCYSVVSVLI

>ApOr54

DQNHVFNVTLAKLIGVYQTLDPKTTKYRINVHRIVMAFIILYIAVILLSGAYYWTNNMPLSVDCYWKGIVSYTMCYSMWL

IVHYSNDIWNCLSITCYGLTSNSLRDRHILDGWRELSVLITTILTFVYFMSAIIFYISSLALSNDLLPVKNHEGLVRNYR

YNLFNLYLFVSEETYVHYNIFYMVEALGVVSLLISFFVFDILLVTFCLAITCQMQMICAAFESVGHKSLANDLSIDSRDE

KKEITNKHDLIYDELKTIIMDHQEVMKKYDMFLTLFKRVLLIQMVVFSVAFIITWFCFIMSKSPTIFIIKIFCGIPPNVF

KLFATCYLFEKLHNQKNSIVFALYSSNWTEMDMKCKKLILLTIKMNNKKLKFTTTKIVSLEMFFKTMRDSYSVLSVLIN

>ApOr56P

KNVHHMVLIFITLFMCVISVILSVSVVYYWTENIPLSIDYIWKSFFSLYMCYSMWVIVHCSNDIWNCLSITCYGFTSHSL

RDRHILDRWREQSVLLTTILTVTYVTAVIIYVVGSLALSDIQSVKNHVGSVGYRHNLINLYLFVDGMYNAHYNIFYMVEA

LCTISILIAFFVFDFLLVTLCLAVCCQMEMIXSVFESVGHKSLGDDEIKETPNEQDLMYDKLKTIIMDHQAVMRIYDEFI

SIFERVLLTLVVVLSIMFIVLWFCFIMSRSSGIFIIKMFCAIPPYLFKLFAVCYLFGNLHDKKDSIIFALYSSNWTEMDM

KFKKLTLLTMKMAHKKLKFTRTKIVNLEMFFKAMGHCYAIISVLVN

>ApOr57P_C

NDQNHIFNITLAKLIDLYQTLDPETTKRGKNVYHIVVAFIRYTLYWCNITSVYYWTDNMPLSIDNFWKGIISICICXPMC

VIVHYSNGVCRSRGVTSHSLQDRHNILDRWRELSVLSTTILAVAYLTTMIIYFFSSLELSNDMLQVKNRDGSVSNYSFVI

NLYLFVSNETYAHYNMFYMVEALFCACIVIALFVFDFFLVTLCLAIRCQMQMVCTAFESVGHKS

>ApOr58P_C

LAKCWISRPCNFVKNVYHMVLIFIMLFMCVISVSLVYYXTNNMYNIWKSFLSLYTCTYVCCSMWVIVHYPNDIWNCLLIT

CYGFISHSLXDRHIILDRWRELSVLLMTILTVAYLTKMIIYFVSSLELSSNILQVKNRDGSVSSYRYNLLNLYLFVGDMY

NAHYNMSYMVEALFCVCIVIALFVFYFLLVTLCLVIHCQXMI

>ApOr60C

EKNHLINIKLAKITGLHQLLDYRGQNIYHVVMSCVSLYMCFISMILLLSGLYYWTGNIPISMNYFFKSVTTFYLIYKMWF

LVRHSNYIWNCLSITCFDFTSFSNRHRHILDHWRDRLAWFTTTYATIYFTTTVSYLAITLAFSENKSPVKSHDGSIGYYR

QNVLNLYLIVDESYNAHYYMFYFVEALFGTFIGLFFFIFDFLLVTLCFSMCCQMQIICSAFESVGHKSLRVQH

>ApOr61C

EKNHFFNIKLAKMTGLYQMLDKTVTYRGRNIYHIGMACVLLYMCLFLLSNLYYWTVNIPISMDYFWKAETTLYVIYKMWF

VIHHSNDIWNCLSITRFDFTSFTNRKRHILECWRERLAWFITIYATMYFTATVSYCAITLAFNENKSPYRQNVMNLYLIV

DETYNAHYYMFYFIEALFGIILGLLIFIFDFLLATLCFSMCCQMQIICSMFESVGHKSLHDH

>ApOr62C

FFNIKLAKIVGLYQMLDPKTKYRGRNIYHIGMACVLLYMCLFLMIYILYYWTNIPISMDYFWKAEITLYVIYKIWFVVQH

SNDIWNCLFITRHDFTSFGNQNRDILDYWRDRLAWLTIVYATMYFMAMFSYLAITLVFSDEKSLVKNHGGSIGYYRQNAM

NLYLIVDQTYNAHYYIFYFVEASFGIFIALLFFIFDFLLVTLCFSMCCQMQIICSAFESVGHKSLHDH

>ApOr65P

FFNIKLAKMTGLYQMLDPKTKYRGRNIYHIGVACVLLYMCLFLLSNLYYWTVNIPISMDYFWKAESTMFIMYKAWFVVHY

SDDIWNCLSITRYDFTSFSNRNRHVLDHWRERVSWSTIIYGIVYFTTCVSYLIMTLAFSQIKSPVKNHDGSIGSYRQNSM

NLYLIVDETYNTHYYMFYFVEALFTIFLGFFPFIFDYLLATLCFSMCCQMQIICSMFELVGHKSLHDRDENIMISPNEHK

LIYDELITIIRDHQAVMKKYEDILTLYRRVMLSHIFTSSLLVILLWFTFIMSNTSDVIVKKLFCVIPSALFQIYMLCYLF

GNIHDQMNIYSHCTYSSNRTELEXKCKKLILLTMKLNNKKLNFTRTNIVNLEMFFKTMGYCYTVTSVLV

>ApOr68N

TYNAHYYMFYFVEASFGILIALLIFIFDFLVVTLCFSMCCQMKIICSAFESVGHKSLDGNIMISPYEHELIYDELITIIM

DHQLVMKKYEDFLTLFRRVMLSHIFVSSFLVIAVWFTFIMSKTSDIIVKKMFCAIPSILFQIYMVCYLFGNIHNQKDSII

FALYSSNWTEMDMKCKKLILLTMNNSQKKLKFTRTKIVNLEMFFKTMGNCYTVISVLVN

>ApOr69

EKNHFFNIKIAKLTALYQMLDPETKFRGRNIYHIVTACVLVYMCLISLSGVYYWTGNIPISMDYFWKSVSAFYIIYKTWI

IIRNSNDIWNCLSITRYDFTSFSDRNRHILDRWRDRLTWFTTIYATMYFTAAVTYLAITLAFGENKSSVKSHDGSIGYYR

QNVMNLYLIVDETYNAHYYIFYFIEALFAAFIGLFFFIFDFLLVTLCFSMCCQMQIICSAFESVGHKSLRVENIKISPKE

HDLIYDELKKIIMDHQLVMKKYEDFLKLFRRVMLLHIFVSSLSVILLCFTLIMSKTSEIILKKLFCLIPSILFQIYMVCY

LFGNIHDQKDEIIFALYSSNWTKMDMKCKKLILLTMNAHKKLKFTRTKIVNLEMFFKTMGHCYTVISVLVN

>ApOr71P

IKLAKLLGLYQMLDPGAVKFRRNIYHIVTSCLLLYACLESILSGLYYCTNITVIMDYFWKAVSTIYIIYKMRINIHHSND

VWYCLSITRYDLTSLTDRKKHILDRWRERLVWLTNIYVIMYFMTLVIYLVITLTSIENQTSNEHNLIYDELKTIIMDHQV

VMKKYEDFSTLFRRVMLFFVSSFTVILLWFTFIMSFSNTSDVIIIKMICEIPSILFQIYMMCYLFCNINDQKDEIIFALY

SSNWTKMDMKCKKILLTMQLNNANQMKTKFTRTKIVNLEIIFKAMHIGYTVILVLV

>ApOr72

TNHVFNIKLAKLLGLYQILDPVAKFRGRNIYHIVTSCLLLYACLISILSGLYYCTNIPVIMDYFWKSVSTIYTIYKMWII

IHYLNDVWNCLSITRYDLTSLTDRNRHILDRWRERLAWLTNIYATTYFTTLVIYFVITLAFSEGKLTVKNRDGSVGYYRQ

NIMNLYLISDDTYNAHYYTFYIIEASFIVFMTFYFLIFDILLLTLCFGMCCQMEIICSAFKSVGHKSINDIKNQTSNVHD

LIYDELKTIIMDHQVVMKKYEDFLTLFRRVMLLQIFVSSFSVILLWFTFIMSKTSEVMVTRMFCLIPSTLFQIYIMCYLF

GNLYDQKDEIIFALYSSNWTEMDMKCKKLILLTMQLNANQIKLKFTRTKIVNLEMFFKTMGHCYTVISVLVN

>ApOr73P_C

VFNIKLAKLLELYQMLDPGAVKRGRNIYQIVTWCLLLYACLISILSSLFYCTNIPVSMDYFWKTITTXIHYSNDVWNCSS

ITRYDFTSLTNRKRHILDRWRECLSWLTIIYAIMYFTSIVIYFVITLAFSECKSTVKDRDGSSGYYRQNPLNLXSITDDT

YNAHYYTFYFIEASFVTFMILYFLIFNILLVTLCFGMCSQMQIICSAFESVGQKSFR

>ApOr74P

VFNIKLAKLLGLYQMLDPGAVKRGRNIYQIVTSCLLLYACLISILSGLYYCTNIPVSMDYFWRAVTVIYIIYKTWIIIHY

SNDVWNCLSITRYDLTSLTDRNRHILDRWRERLSWLTNIYAIMYFTSIVIYFVITLAFSECKSTVENRDGSGGYYRQNVM

NLYLISDDTYNAHYYMFYFIEASFIAFMTLYFLIFDILLVTLCFGMCCQMQIICSAFESVGHKSFDDIKNETSNAHDLIY

DELKTIIMDHQVVMKKYEDFLTLFXRVMLLHIFVSSFSVIFLWFTFIMSTTSDVIVTKLFCLIPSILFQIYMMCYLFGNI

NDQKDEIIFALYSSNWTEMDMKCKKLILLTMQLNANQIKLKFTRTKIVNLEMFFKTMGHCYTVISVLVN

>ApOr75P_C

NETNHVFNIKLAKLLGLYQMLDPGALIREQNIYHIVTSCLLLYARLISILSGLYYCTGITVSMDYFWKSAMTIYVIYKTQ

IIIHYSNDVWNCLSITRYDFTSLTDRNRHIPDRWRERLAWLTNIYDIPYFTTIVIYFVITLEFSEGKSIVKNRDGSVXYY

HEKAVNLYLIVDETYNTNYYMFYFSGSSSIAFMXIYFLIFDILLVTLCFGMCCQMEIICSAFKSVGHKS

>ApOr76C

KNHIFNIKLAKLTGLYQMLDPETTKWGQNIYHVVMSCILLFMLIPMLSGLYYWTVNIPISMDFFWKSVSIFYIIYKTWVI

IRNSNDIWNCLSITSYDFTSFSNRNRHILDDWRDRLARFTTIYAILYFTGTVSYFASTLALSEGKTPVKNIDGSIGHYRQ

NVMNFYLIASADTYSHYYMFYFVEALFLALFATCFLIFDILLVTLCFGMCCQMEIICSAFELVGHKSLNTAFNEHDLIYD

EEIKTIIMDHQVVMKKYEDFLRLFQPMMLLHIFISSFSVISLCFTFIMSRTSDVIVKKMFCSIPPALFQIYMVCYLFGNI

HDQKDSIIFALYSSNWTEMDMKCKKLILLTMNAHKKLKFTRTKIVNLEMFF

>ApOr77

KNHIFNVKLARLTGLYQMLDPDTKCRGRNIYHVVMSCVCLISMILMISGLYYWMVNVPISIDYFWKSVSTFYIIYKTWII

IRHSNDIWNCLSITRHDFTALTDQNRHILERWRKRLAWLTTIYAIMYTMSVVSYLVFTLAFNEGKTPVKNHDGSIGYYRQ

NVMNLYLIVDETYNAHYYTFYFIEALFLGLIGLFYLIFDILLVTLCFGMCCQMQIICSAFESVGHKSNGNTNITPNEHDL

IWDELRTIIMDHQAVMEFSININKMYKLFSCLNCIKFSTLIANSFSNDYRFKTSEVIVKKMFCSIPPILFQIFMVCYLFG

NLHEQKDSIIFALYSSNWTEMDMKCKKLILLTMNANQKKLKFTRTKIVNLEMFFKTMGDCYTVISVLVN

>ApOr78

KNHIFNIKLARLTGLYQMLDPDTKCRGRNIYHVVMSCVLLYMCLISISGVYYWTVNIPISIDYFWKSVSTFYTIYKTSII

IRHSNDIWNCLSITRLDFTTFSNRNRQVLDRWRERLSWLTTIYAIIYTMSVVSYLVFTLVFNEGKTPVKNHDGSIGYYRQ

NVMNFYLMVDETYNAHYYKFYFIEALFAAFMGFFFFIFDFLLVTLCFSMCCQMRIVCSAFESVGHKSEHDLIWDELRTII

MDHQAIMEKYKDFLSLFRRVMLAHIFISSLSVIALWFTFIMSKTMFCAIPTILFQIFMVCYMFGKLHEQKDSIIFALYSS

NWTEMDMKCKKLLLLTMNANQKKLKFTRTRIVNMEMFFKTMGNCYTVISVLVN

>ApOr79F

HIFNIKLARLTGLYQMLDPNTKCRVRNIYYVVMSCVLLYMCLISFSGLYYWTVNIPISIDYFWKSVSTFYTIYKTWIIIR

HSNDIWNCLSITRHDFTTFSNRNRHVLDRWRERVSWSTTIYAIIYTMSMVGYLVFTLAFDEGKTPVKNHDGSIGYYRQNV

MNLYLIVDETYNAHYYTFYIIEALFVGFLGLFFFIFDFLLVTLCFSMCCQMQIVCSAFESVGHKSNGNTKITPNEHDLIW

DELRTIIMDHQAVMEKYKDFLSLFRRVMLVHIFISSITVIALWFTFIMSKTSEVIVIKTFCSTPPVMFQIFMVCYLFGKL

HEQKDSIIFALYSSNWTEMDMKCKKLILLTMNANQKKLKFTRTRIVNLEMFFKTMGNCYTVISVLVN

>AQN78403.1_olfactory_receptor_1_[Meteorus_pulchricornis]

MMKTKYQGLVADLMPNIRLMQISGHFMFNYYGEGKKFMHKIYCSIHLFLILLQFALCGLNLAMESDDVDVLTANTVTLLF

FSHTVIKIIYFAFRSKLFYRTLAIWNNPNSHPLFAESNARYHSIALTKMRRLLFCVGAATVASIISWVVLTLVEDDPVRE

IVDKVTNETTIIPLPRLPVRSFYPFDARHGVAHIAMFAFQLYWLIITMFNANSIDVLFCSWLLFACEQLQHLKAIMKPLM

ELSATLDTVVPNSSELFKAGSADHLRDTTGTVPSATQPNGDNMLDLDLRGIYSNRQDFTATFRPTDRTQYNGGVGPNGLT

KKQEMLVRSAIKYWVERHKHVVRVVTAIGDAYGVALLFHMLITTITLTLMAYQATKVNGINVYAASTIGYLLYSLGQVFL

FCIFGNRLIEESSSVMEAAYSCHWYDGSEEAKTFVQIVCQQCQKAMSISGAKFFTVSLDLFASVLGAVVTYFMVLVQLK

>AQQ73487.1_olfactory_receptor_2_[Heliconius_melpomene_rosina]

MMTKIKTQGLVSDLMPNIKLMQAAGHFLFNYHSDNSGMSTLLRKVYSSVHAFLIMIHYLCMAANMAKYSEEVNELTANTI

TVLFFTHSIIKLAFFAITSKNFYRTLAVWNQSNSHPLFTESDARYHQLSLNKMRRLLYFISGMTIFSVVCWVTITFFGES

VRFLVDKETNDTLTEPVPRLPLKAWYPFDAMGGTMYIIAFAFQIYWLLFSMAMANLLDVMFCSWLIFACEQLQHLKAIMK

PLMELSASLDTYRPNTAELFKVSENSEKIPDSTDLDIRGIYSTQQDFGMNLRGAGGRLQTFGQQNNNPNGLTQKQEMLAR

SAIKYWVERHKHIVRLVSSIGDTYGTALLFHMLVSTITLTLLAYQATKINGLNVYAFSTVGYLSYTLAQVFHFCIFGNKL

IEESSSVMEAAYSCQWYDGSEEAKTFVQIVCQQCQKAMSISGAKFFTVSLDLFASVLGAVVTYFMVLVQLK

>BAG71415.1_olfactory_receptor-2_[Mythimna_separata]

MMTKVKAQGLVSDLMPNIKLMQAAGHFLFNYHSENAGMSNLLRKIYASTHAILIIVHFACMGINMAQYSDEVNELTANTI

TVLFFTHTIIKLGFFALNSKSFYRTLAVWNQSNSHPLFTESDARYHQIALTKMRRLLYFICGMTCLSVVTWITLTFFGES

VRMITSKETNETLTEVVPRLPLKAWYPFNAMSGTMYIVAFAFQVYWLLFSMAIANLMDVMFCSWLIFACEQLQHLKAIMK

PLMELSASLDTYRPNTAELFRASSTEKSEKIPDAVDMDIRGIYSTQQDFGMTLRGAGGRLQNFGQQNANPNGLTPKQEML

ARSAIKYWVERHKHVVRLVASIGDTYGTALLFHMLVSTITLTLLAYQATKINGINVYAFSTIGYLSYTLGQVFHFCIFGN

RLIEESSSVMEAAYSCQWYDGSEEAKTFVQIVCQQCQKAMSISGAKFFTVSLDLFASVLGAVVTYFMVLVQLK

>BAG71418.1_olfactory_receptor-2_[Diaphania_indica]

MMTKVKAQGLVSDLMPNIKLMQAAGHFLFNYHSDNSGMSTLLRKVYSSAHAFLIVIHYLCMAVNMAQYSEEVNELTANTI

TVLFFAHSVIKLVFFAINSKSFYRTLAVWNQSNSHPLFTESDARYHQLSLTKMRRLLYFICGVTVLSVISWVTLTFFGES

VRLIANKETNETLTEPAPRLPLKAWYPFDAMSGTMYVVAFVYQVYWLLFSMAIANLMDVMFCSWLIFACEQLQHLKAIMK

PLMELSASLDTYRPNTAELFRASSTEKSEKVPDPVDMDIRGIYSTQQDFGMTLRGAGGHLQNFGSNGNNPNGLTQKQEML

ARSAIKYWVERHKHVVRLVASIGDTYGTALLFHMLVSTITLTLLAYQATKINGINVYAFSTIGYLSYTLGQVFHFCIFGN

RLIEESSSVMEAAYSCQWYDGSEEAKTFVQIVCQQCQKAMSISGAKFFTVSLDLFASVLGAVVTYFMVLVQLK

>BAH57973.1_olfactory_receptor_[Ostrinia_scapulalis]

MMTKVKAQGLVSDLMPNIKLMQAAGHFLFNYHSDNSGMTTLLRKVYSSVHAFLIVINYLCMAANMAQYSEEVNELTANTI

TVLFFAHSVIKMLFFAVNSKSFYRTLAVWNQSNSHPLFTESDARYHQLALTKMRKLLYFICGVTVLAVMSWITITFFGES

VRMIANKETNETLTEPAPRLPLKTWYPFDAMSGTKYVVAFVYQVYWLFFSMAIANLMDVMFCSWLIFACEQLQHLKAIMK

PLMELSASLDTYRPNTAELFRASSTEKSEKMPDTVDMDIRGIYSTQQDFGMTLRGAGGRLQNFGQPNPNNPNGLTQKQEM

LARSAIKYWVERHKHVVRLVASIGDTYGTALLFHMLVSTITLTLLAYQATKINGINVYAFSTIGYLSYTLGQVFHFCIFG

NRLIEESSSVMEAAYSCQWYDGSEEAKTFVQIVCQQCQKAMSISGAKFFTVSLDLFASVLGAVVTYFMVLVQLK

>BAH57974.1_olfactory_receptor_[Ostrinia_latipennis]

MNQVKAQGLVSDLMPNIKLMQAAGHFLFNYHSDNSGMTTLLRKVYSSVHAFLIVINFLCMVANMAQYSEEVNELTANTIT

VLFFAHSVIKMFFFAVNSKSFYRTLAVWNQSNSHPLFTESDARYHQLALTKMRRLLYFICGVTVLAVISWITITFFGESV

RMIANKESNDTLTEPAPRLPLKTWYPFDAMSGTMYIVAFVHQVYWLFFSMAIANLMDVMFCSWLIFACEQLQHLKAIMKP

LMELSASLDTYRPNTAELFRASSTEKSEKMPDTVDMDIRGIYSTQQDFGMTLRGAGGRLQNFGQSNPNNPNGLTQKQEML

ARSAIKYWVERHKHVVRLVASIGDTYGTALLFHMLVSTITLTLLAYQATKINGINVYAFSTIGYLSYTLGQVFHFCIFGN

RLIEESSSVMEAAYSCQWYDGSEEAKTFVQIVCQQCQKAMSISGAKFFTVSLDLFASVLGAVVTYFMVLVQLK

>BAJ23260.1_odorant_receptor_2,_partial_[Ostrinia_zealis]

QGLVSDLMPNIKLMQAAGHFLFNYHSDNSGMTTLLRKVYSSVHAFLIVINYLCMAANMAQYSDEVNELTANTITVLFFAH

SVIKMLFFAVNSKSFYRTLAVWNQSNSHPLFTESDARYHQLALTKMRRLLYFICGVTVMSVISWIIITFFGESVRMIANK

ETNETLTEPAPRLPLKTWYPFDAMSGTMYVVAFVYQVYWLFFSMAIANLMDVMFCSWLIFACEQLQHLKAIMKPLMELSA

SLDTYRPNTAELFRASSTEKSEKMPDTVDMDIRGIYSTQQDFGMTLRGAGGRLQNFGQPNPNNPNGLTQKQEMLARSAIK

YWVERHKHVVRLVASIGDTYGTALLFHMLVSTITLTLLAYQATKINGINVYAFSTIGYLSYTLGQVFHFCIFGNRLIEES

SSVMEAAYSCQWYDGSEEAKTFVQIVCQQCQKAMSISGAKFFTVSLDLFASVLGAV

>BAJ23262.1_odorant_receptor_2,_partial_[Ostrinia_palustralis]

QGLVSDLMPNIKLMQAAGHFLFNYHSDNSGMTTLLRKVYSSIHAFLIVINYLCMAANMAQYSDEVNELTANTITVLFFAH

SVIKMLFFAVNSKSFYRTLAVWNQSNSHPLFTESDARYHQLALSKMRRLLYFICGVTVLAVISWITITFFGESVRMIANK

ETNETLTEPAPRLPLKTWYPFDAMSGTMYVVAFVYQVYWLFFSMAIANLMDVMFCSWLIFACEQLQHLKAIMKPLMELSA

SLDTYRPNTAELFRASSTEKSEKMPDTVDMDIRGIYSTQQDFGMTLRGAGGRLQNFGQPNPNNPNGLTQKQEMLARSAIK

YWVERHKHVVRLVASIGNTYGTALLFHMLVSTITLTLLAYQATKINGINVYAFSTIGYLSYTLGQVFHFCIFGNRLIEES

SSVMEAAYSCQWYDGSEEAKTFVQIVCQQCQKAMSISGAKFFTVSLDLFASVLGAV

>BAJ23264.1_odorant_receptor_2,_partial_[Ostrinia_ovalipennis]

QGLVSDLMPNIKLMQAAGHFLFNYHSDNSGMTTLLRKVYSSVHAFLIVINYLCMAANMAQYSEEVNELTANTITVLFFAH

SVIKMLFFAVNSKSFYRTLAVWNQSNSHPLFTESDARYHQLALTKMRRLLYFICGVTVLAVISWITITFFGESVRMIANK

ETNDTLTEPAPRLPLKTWYPFDAMSGTMYVVAFVYQVYWLFFSMAIANLMDVMFCSWLIFACEQLQHLKAIRKPLMELSA

SLDTYRPNTAELFRASSTEKSEKMPDTVDMDIRGIYSTQQDFGMTLRGAGGRLQNFGQSNPNNPNGLTQKQEMLARSAIK

YWVERHKHVVRLVASIGDTYGTALLFHMLVSTITLTLLAYQATKINGINVYAFSTIGYLSYTLGQVFHFCIFGNRLIEES

SSVMEAAYSCQWYDGSEEAKTFVQIVCQQCQKAMSISGAKFFTVSLDLFASVLGAV

>BAJ23265.1_odorant_receptor_2,_partial_[Ostrinia_zaguliaevi]

QGLVSDLMPNIKLMQAAGHFLFNYHSDNSGMTTLLRKVYSSVHAFLIVINYLCMAANMAQYSEEVNELTANTITVLFFAH

SVIKMLFFAVNSKSFYRTLAVWNQSNSHPLFTESDARYHQLALTKMRRLLYFICGVTVLAVFSWITITFFGESVRMIANK

ETNETLTEPAPRLPLKTWYPFDAMRGTMYVVAFVYQVYWLFFSMAIANLMDVMFCSWLIFACEQLRHLKAIMKPLMELSA

SLDTYRPNTAELFRASSTEKSEKMPDTVDMDIRGIYSTQQDFGMTLRGAGGRLQNFGQPNPNNPNGLTQKPEMLARSAIK

YWVERHKHVVRLVASIGDTYGTALLFHMLVSTITLTLLAYQATKINGINVYAFSTIGYLSYTLGQVFHFCIFGNRLIEES

SSVMEAAYSCQWYDGSEEAKTFVQIVCQQCQKAMSIPGAKFFTVSLDLFASVLGAV

>BAO48211.1_odorant_receptor_co-receptor_[Camponotus_japonicus]

MMKKQQQGLVADLYPNIRVMKMFGHFVFNYYDDNSSKYLHKVYCCVNLFLLLLQFGLCAVNLIIESADVDDLTANTITLL

FFTHSIVKIVYFAVRSKYFYRTWAIWNNPNSHPLFAESNARYHAIALKKMRLLLFLVGATTVLTAIAWTILTFFEHPIRK

LVDPVTNETTIIELPQLLVRSFYPFNASRGIKHILVLVYQFYWVLFMLINANSLDVLFCSWLLFACEQLQHLKQIMKPLM

ELSATLDTVVPNSSELFKAGSADHLRESENNQSQLPAPPQGDSMLDLDLRNIYSNRQDFTATFRPTAGMTFNGGVGPNGL

TKKQEMLVRSAIKYWVERHKHIVRLVTAVGDAYGFALLLHMLTTTITLTLLAYQATKVNGINVYAASTIGYVLYTFGQVF

LFCIFGNRLIEESTSVMEAAYSCHWYDGSEEAKTFVQIVCQQCQKAMSISGAKFFTVSLDLFASVLGAVVTYFMVLVQLK

>BmOR-1

KPQNFQYMKILRFNLKIICAWPEKQNEIRSLGHSIHRVILPIQSVVCLACGILYIHFHFNEFFILASTFITVMMNLATCS

RTALVMLFERYLVRFITVMHLFNFQKNSDYAYKLCTFVNRMSHFYTLYVFSMFMGLGLFNLLPLYNNYVSGAFSDPYGPN

VTFFHSVYFAFPFDYSHNRGYIIMALFNSYVSVTCSIGLVMFDLLMCLMVMHVWGHLKILSHNLINFPRGPTNVETYTEE

ESKEVFARLRECIKHYGTVDDFANDMSETFGVILLVYYGFHQVSLCMLLLECSDLSTKAMLRYGPLTLIMIQQLIQISII

FELLGSVADRIPDAVYQLPWECMDVKNRRVVYGFLRRTQNPVRFKAMGMLDVGVQTMASILKTSISYFVMLRTV

>BmOR-5

EFTYIKFLKSFCKIMDFWPEREEKNSKTRIFRLRYILVLQFCFTLVAGVLYLTNSVGTFYDLGHTIITVLMNVVSLSRLI

LRCFKKYDVQQFINKIHLYHYRNDSEYAMKIHTVVHKISHNMTYIFSFCIIFGTVTFNLTPIFNNIGSDAYKNPRPDNVT

LQQCVYYALPFDYTGNKWYLLVAIFNVQKTFFCTSLFILFELSLSLMIICLWGHLRIFIHNLNHIPAPEYTKEERQEVDD

TLKKCIQHHTLIIGFVRIMSETYGLAVLIYYAFQQVVGCLLLLQCSQMELKTVTRFGFLTLVLNQQLIQISVIFELLGYM

SDKLQDAVYCVPWEYMDTSHRKMVYMMFRQSQIPLQLKAMNMLSIGVKTMVSILKTSVTYYLILKTV

>BmOR-6

WWGYTFPKYVGWFYHLQCNVVRLFGKCVVVSQILFIILNYQTIDKSVFIIAITITPLGALVGIKAESAKAECYVNLMKHI

HSIYRKNENNEFVKKKVIQIERVSRFTAYFLILIAINCLSWMLKPTLHNIKHFEEIMNKSMEFQYYIYFWTPLDYKYNRD

YIIIHTLCIYLGATAVTVIVTFDIFNFIAVFHVVAHIQILKNNVKSNWSDDFNESEKYLVSILEYHAYIIRIFGEVQSAF

GLNVASNYLQNLIEDGLFLYQIMNGEKENVLMYGLMIILYLGGLIFLSIVLEEIRRQNYDLCEYVYALPWEGMSLENQKI

FVVFLQRTQPDLEFETVCGMKAGVKPAFSIVKSMFSYYVMIN

>BmOR-10

SFLFVPSKVLTLCGVWPVEKTSIFSLIYRSIMLSSQFCFLVFNGIYIGLMWGDLKAVSDALYMFFTQTTCCSKAIGFYFN

FMKIKRIVASMDDVLFTAMSIEDQATIFSHSRTVNKLYKGVLGFTGFTLVQWTVLSLIGSGRTLPFEMWVPTDISKSPNY

EITFVVELWMMVISAALFMSVDTITVATMMFSCAQLDIIMKKTQQIQEIPLSPDLSSRNRSELHSELHEKNNGILIDCIK

QHQAIVRFSELCEGTFQVHSFFHLGGIVFMICVIGFRMAGESPVSAQFWAALSYLVIILGQLYLYCWCANELTTKSEQLR

DKLYLTPWYDQDVKFKRNLCIAMECMAKALTFRAGSYIPLSRAMFVSILRSSYSYFAFLNQ

>BmOR-11

HSHFETSLNKIKVLFKYSGMNLENTTNTYEFLHRWVYILNHAWTLAAVTFICIGISNGQNFIEMTCIAPCVAMTVLAVSK

SFFHYINENAVKSLLENLITDFERTKSVQRTEIVATEKQLLNMVINVLYVLNCSMILVFDMTPLIIIAIKYWTTNKFVRL

LPYLDIFVFVPYKFEYWVMAYILQIWAECIVLLFIGAADCLFFTCCTYIRIHFRLLQYDFERLTSSRRELRDDEDFRETY

TNLVKRHQGLIESSSILEMIYSKSTLSNFVLSSLVICLSAFNVTVIVNDVTIVMTYLIFLAMSLMQVYFLCFFDMLMSAS

EEVGNAVYNCSWYTEKASTGKDLLFTITRS

>BmOR-12

ITDVFSLNFIFWKFLGLWGKSAPSKYNMAYTVFYLFASLFVYDIFLTLNLIHTPRKLETLVRETMFYFNHLVAVTKILMM

FIMRKKILVIFDLLDCEEFKPNDENSQEIMKRKTDFYYIYWRIVAVTSNLSCFMLVIGPLIKMLIWKIELGLPVKFYFMS

DELRNKYFVIWYIYQSFGIYNQMVNNLNLDTFNCGMLWMAVGQLQILKTKFVNLKDLKSRDDMQIERLRKYLTHYEIILK

YCATVQDILNITIFVQLGMSSIVICVGLCGFVAMPSNTETAIFMSSYLITMTMQIFVPSWMGTQISFECGELMSAAYCCE

WIPRSKLFKRSLILFVERAKTPVRITGLKIFTLSLDTFTSIMKTTYSFFTLIRQL

>BmOR-13

CFEINWKFWKFLGIWSENKPHRYYKYYSKIFITFFVILYDVLYTINFYFVPRQLDLIIGEMLFYLTELSVLSKVFTFIIM

RHKLKIIFEILESDAFQTDTEEELKILHRAKVFIKRYWKIVALVSITANLTHISSPLLKNLIFKVELVLPVCSYSFLSES

FLKFEYPLYFYQIVGIHFHMLYNLNIDTYFLGLMILIIAQLDILNVKFRNLKSGKDHTQLICLICILQSVPVEYYIFLAT

YMFIMIIQIMVPCCFGSRIMDKSILLSSAIYNCDWTSNSKDFKINMRLFVERANKPLSITGGKMFSLSLATFTS

>BmOR-15

IFKPNVFFWKMFGIWADRKSSKTYKYYSFVFLFITLIMYNSLLAINLLYTPLKIELLIREVIFCFTEITVTTKVLMILFK

RNKILDAFDLLNKNEFRGNSEESSAIIQKNNSAYKTYWKLYAILSNFAYSSQVLGPLIVKLIWKTKLELPICNYYFLNEE

RHDFFSGWYIYQSFGMYGHMMYNVNIDTFISGLLMMAVTQLKIIQTKLLSLKLNEILKHYELVLKYCSTVQSILDVAMFV

QFGVASAIICVAMCGLIMVRSSTETLLFMVTYLFAMTLQIFVPAWMGTQLHFQSQELVFAAYNSEWIPRCQSFKRSIIIF

VERAKIPITITGLKMFPLSLATFTSIMKTAYSFFTLIRNM

>BmOR-16

MPVSPERSPHYHLGYSFQLVTICMSAYMYFGVDSVAFSSVIFGCAQIGVIKDKIMSINISRYNRKTLIECVKHHQAVISF

TELVEDTYNSYLLFQLVGSVGIICGLAQCPITIPIAILCYLSVMISQLFVCCWCGHELSATSEELHTILYNCAWYDQDVK

FKRDLNFMMARARRPILLRAGYYISLSRQSFVSILRMSYSYFAVLNQT

>BmOR-22

SGFWYQKTRNDKTLLYKIYSCVLFFTYGFMTVLEIMAATMGDFPDDEKRDSVTFASSHTLIMIKFISIIKNKELLKTLNR

KMMMICEAHEEQTLMDEMYRIVKINVVAYCVAVYGSVTFFVFEGLRKFYDGSHFVTIVTYYPSKDDDTLASIFRIATTLV

LLVMMLSMIISVDTYTMAYLIMYKYKFITLRHYFKRLRENVDELKLAQGLVEGIKMHNELLSLSKDIHKAFGTVMALQLC

QSSGSAVSLLLQIAVTMYL

>BmOR-25

FEKALRSANFYMRVIGIPTDIRDGNRTLRNRWFYCINFLWLNTDVAGEVKGLLNGSSTLIENTYLIPCLTLCILGNVKTF

FTIKYANHIIDLVAILKDLEIKNNAARKETEIVKERLKFLTTSNKFLLFVIGTGIIAFGIGPLMLTASIYFSSGDMKLKL

PFLIWYPFDSSDIRYWPFVYVHQVWSACIACCAVYGPDCFYFTSCTFIHIHFIHLQNDITNVIVESSHQAFLELTNRHKD

LIRCVNLLEIIYSKSTLVNVVSSSLLICVTGFNVMVTFCWFAAPFASFLALGLVQTYLLCYYGDTIMCSSTEVSDAVYNS

TWYGTNISQMRDYLFVMKRAQKPCKLTAYGFSDVNLRTFSRILSTAWSYFALLIT

>BmOR-26

SVAPHLRRLRQVGFCQLDPTSQSRLMHRVYHRLVLAATVLYIFEQLTYAYQARNDMERLSRVLFLMLCHLTCIAKQFVFH

SDADKINQLVVGLDDALCNQPVETHRLLLLETSRRAARLLMLYSGCAVSTCILWAVFPLLDQLRGRTVEFAFWIPIDYRH

NFQFAVVLAYAFYSTSLVAVANTTMDAFIATVLYQCTTQLRILRMNFESLPERAYALSRKTRQDYHTVTRQDYHTVTHEL

LVDCLLHYKKITETCNLLEQIFGKAILVQFGVGGWILCMAAYQIVDLSVLSIEFASMILFISCILTELFLYCYYGNEVST

ESERLVTSIYSMEWVGARLGFQRGLLVLLERARRPVRPAAGLVIPLSLQTFLKIIKSSYTFYAVLRQTK

>BmOR-27

LGPTLKGLKYWGMWQSGGIKRILYNSIHAFATFFVITQYVELWIIRNNVELALRNLSVTMLSTVCVVKAGTFVCWQKYWS

GIIGFVSNLEKEKNDAATQAAIVKYIKYSRRVTYFYWSLVTATVFTVILAPLVGFLSSPERELIANGTLPYPEIMSSWVP

FDRSRGFGYWVTALVHTLICFYGGGVVANYDSNAVVLMSFFAGQMKLLSINCSRLFDDGNEVISNNEA

>BmOR-28

NLFFFCSSVIFKTMKYCVDYKIVYKEYNKTHDFKRLMVHEMKLKYDIQSVPPFHCMFAYNFLQVCVLILNYSGFDGSFCI

ASIRLCMKLKLVVYKVQKAFAESKSVSE

>BmOR-29

LGPNFWLINKTGLLLPKTNFGKLAYILVHEIVTFFVVTQYVELYVIRSDLDLVLTNLKISMLSIVCIVKVNTFVFWQTSW

REVLEYVNDKFERNQTDETRGKIIETYTKYCRRLTYFYWSLVFTTFLTTTNTPLMRYWSSPIFREHRNGTEDFPHIFSSW

MPFDKNHSPGSYCTIVWHVLLCAYGAAIMAAYDTCIVVIMVFFGEKLNLLRERCKKMLAND

>BmOR-32

KVAIFCFIVTYMLQVMELYWSKGDQEKLFECFSILSFCGMGVMKLVILRVYHQRWRFLLNQVSYDSDNDNDDNEIVTFIT

KYTDKFKRTSSILIKMYASTLVIYVLSPFVEYIFRQFRGDLNIAYPHILPAWTPLDEFSVTGYLIMVSFETVACIYCVFV

HVAFDLTCVGLMIFACGQFYLLRYRSERIG

>BmOR-35

LLRAITLNIDSRHTARIPFICYVMTVVITLSYFYVFLAWFVFVETRDYLAAMVVLSLGISSEIGTLKFFYTFIYIKKVQR

IVREYLECDHMVVPESRFADNVLKTMRNVKKRAILYWVVVIGNGVVYVTKPLFMSGRHHMEDRYIVYPMFESPNYEVAYF

LMMFGLCFICYPPANVTVFLIVVVGYTEAQMIALGEEMLRIWEDAVYNNKYHTVGALTNSSEKNKIINQRLTEIIKMHTT

NIQLLRQVEFVFRSAIAMGYVFLVLGLIAELLGGLENTYLQIPFALIQVLVDCYTGQKVMDASSLFEQAVYDCKWENFDK

SNMKTVLLILQNSQKSMRLSVGGITVLGFSCMMSVMKSIYSAYATLRT

>BmOR-43

SEGIAMAAYESSWILWPVDMQKDLLIVITAAQKPMKLSAGGMAVLSVQTYSQTLYNGYSIFAVLN

>BmorOR-24

DRSIKKIESYFRWMGINIRSGNNNKKDVFKIRCIYFINFLLNTDVLGAIFWFRQGKTFTEVTYNAPCLTFSFLANFKMLS

LIFYEKTVHELIAALQKHFLRQNCAEELKMLKDEKNFLHAVFKGSKIVNYASILTFGCSPLVLIASNYYKTGRMDYLLPL

IVLYPFDVDNITVWPIIYVRQIWSVITAVIGVCATDYLFYTFCVYISTQFRLLGHSIERVVPRLNGNLRMKFVENLKWHQ

ELIRAASLLEQIYTKSTLYNFVTSSVIICLTGFNVAVVEDFAVILSFLFFLFMSLLQIILLCFFGDKLMKSSTNISDAVY

NSKWYLTEKNVGKVLLMVQIRSQRACRLTAYGFAEVNLRAFMKILSTAWSYFALLQSL

>BmorOR-50

ALTLTLNTLSWAGLILRDDTKTQRIIMKVYGGLVFLYFVFTAYVQIADLVVIWGNIDFMTETSLILFMQLAVSAKVLTLM

LKSKKIMEVTNEADAILNSEKKVEGQRIIASIDKNTTLFLKYYGFFVAFTIICWFMGENTSTFFIRSKYPFNELKSPGRE

FAFVHQCIVVIFTGSFDFNVDIIIISLVAVCRCRLKLVALSLRNLCNKRNLITSDEEKVITERLRNIISQHKRALDAAEA

IKHYLSGALLVQLMVSIVVICTTAYQLAVKKSTTMQSLTMAGYLFGTSLEVFLFCYQGEFLRESSEEIADAAYECPWYTL

TRPLKKTLLIIMTRAQRPATLTAGGFVTLDITEYMAIMKASYSFFTVLQQV

>CAD31851.1_chemosensory_receptor_2_[Heliothis_virescens]

MMTKVKAQGLVSDLMPNIKLMQMAGHFLFNYHSENAGMSNLLRKIYASTHAILIFIHYACMGINMAKYSDEVNELTANTI

TVLFFAHTIIKLAFFALNSKSFYRTLAVWNQSNSHPLFTESDARYHQIALTKMRRLLYFICGMTVLSVISWVTLTFFGES

VRMITNKETNETLTEVVPRLPLKAWYPFNAMSGTMYIVAFAFQVYWLLFSMAIANLMDVMFCSWLIFACEQLQHLKAIMK

PLMELSASLDTYRPNTAELFRASSTEKEKIPDTVDMDIRGIYSTQQDFGMTLRGAGGRLQNFGQQNPNPNGLTPKQEMLA

RSAIKYWVERHKHVVRLVASIGDTYGTALLFHMLVSTITLTLLAYQATKINGINVYAFSTIGYLSYTLGQVFHFCIFGNR

LIEESSSVMEAAYSCQWYDGSEEAKTFVQIVCQQCQKAMSISGAKFFTVSLDLFASVLGAVVTYFMVLVQLK

>CAD88205.1_putative_chemosensory_receptor_2_[Antheraea_pernyi]

MMTNVKTQGLVTYLLPNIKLLQLAGHFLFNYHADNSGMATLLRRVYATVHAILIIIHYVCMGVNMAHYSDEVNELTANTV

TVLFFAHTIIKLLFFAISSKSFYRTMAVWNQSNSHPLFTESDARYHQIAVTKIRRLLYFVCGMTVFSVLSWIILTFFGES

VRLIANKETNETLTEPAPRLPLKAWYPFDAMGGSMYVLAFIFQIYWLLFSMAIANLLDVLFCSLLIFACEQLQHLKAIMK

PLMELSAALDTYRPNTAELFRVSNDKSEKVPDSVDLDIRGIYSTQQDFGMTLRGTGGKLQNFGNTPSNPNGLTQKQEMLA

RSAIKYWVERHKHIVRLVGSIGDTYGTALLFHMLVSTITLTLLAYQATKVNGINVYAFSTIGYLGYTLGQVFHFCIFGNR

LIEESSSVMEAAYSCQWYDGSEEAKTFVQIVCQQCQKAMTISGAKFFTVSLDLFASVLGAVVTYFMVLVQLK

>CAD88246.1_putative_chemosensory_receptor_2,_partial_[Calliphora_vicina]

QLQHLKGIMKPLMELSASLDTYCPNSAALFRSLSANSKSELIINEEKEPPNDLDMTGIYSTKADWGAQFRAPTTLQTFNG

VNGGNPNGLTKKQEMMVRSAIKYWVERHKHVVRLVAAIGDTYGAALLLHMLTSTIKLTLLAYQATKITGVNVYAFTVIGY

LGYALAQVFHFCIFGNRLIEESSSVMEAAYSCHWYDGSEEAKTFVQIV

>CfOr2

NYQRDMRYIFKPCSWILGSIGIWPITFRGIGQHVSKIALVLCNFALGFAIVPCILHIIYDEKDLNIRLKLSGLLGFCLTA

MMKYCVLAIRRPKILRCIEHVKNDWWQVVKFNSDRELMMKYAATGRRLSIISTTSMYIAGFIYHTILPFCTVHKAGNETI

RPLVYPTYSEYQTQISPIYEIVYLAHCICGYTMYSVTAGSCGLAAIFVTHACGQIDVITSRLEDLSRGKNFQQSSDVQQS

SDVNQRIAAIVSGHVRILRFCAAVDEILQEVCLLEFASSIFTMCLPEYYCIVDSDTVGLTTYFLLFVSFCFNMFILCYIG

ELLMTKSSQIGSVCFMIDWYQLPTKAVRSLVLVIAISNHPIKISVGRMIDLSLATFGNV

>CfOr3

SYHRDIKYVLKLNNWILCTIGILPFTTRRIGRHVFKILIAFYNFIISFTIVPCALHIIYDQKDITIRLKLFGLLAFCLTA

MTKYCILTIRLPKILYCIEFVKSDWWQVTFRSDREQMLKYAAAGRKLTIILTSFMYSSVIYYLILPFFSEHIINNETVRP

LVFPIYSKFQFQISPVYEIVYVAHCMCEYTLCSVTVGTCGLAALFVTHACGQIEVILSRLEDLVNGKNFMQNQNPNIHRR

IAAIVKSHVRVVRFAAVVEEVLQEVCLVELSSSLCTICLLEYYCIVDDDRISLATYFILFVSFCFNVYMLCYIGELLMEK

SSQIGHICYMINWYQISPKFARSLILIIAMASHPIKISAGRIADLSLLTFVNILKTTLAYLSFLRTL

>CfOr4

SYHRDIQYVLKLNNWILCTIGILPFTTRRIGRHVFKILIAFCNFIISFAMVPCALHIIYDQKDITIRLKLFGLLAYCITA

MTKYCILTIRRPKILYCIEFVKKDWWQVTFRSDREQMLKYAAAGRKLTIILTSFMYSSVIYYLILPFFSEHIINNETVRP

LVFPIYSKFQFQISPVYEIVYVAHCMCEYTLCSVTVGTCGLAALFVTHACGQIEVILSRLEDLVNGKNFMQNNPNIHRRI

AAIVKSHVRVVRFAAVVEEVLQEVCLVELSSSLCTICLLEYYCIVDDDRISLATYFILFVSFCFNVYMLCYIGELLMEKS

SQIGHICYMINWYQISPKFARSLILIIAMASHPIKISAGRIADLSLLTFVNILKTTLAYLSFLRTL

>CfOr5

FNYREDIQYVFKLNNWILGSLGIWPIATRGIRQHASKIAITLCNLALAFAIVPCALHIIYDEKDIIMRLKLFGLLAFCLT

AMTKYCILAIRRPKILRCIEYVKSDWWKVTFKTDHTIMLKYATTGRNLTIIGASFMYTAGIIYIILPFCSEHKINNQTIR

PLVYPTYSKFQSQISPIYEIVYVAHCMCGYTIYSVTAGACGLAALFATHACGQIQIIISRLENLLEGENFKQSPNVHNFK

QSPNVHQRIAAIVKNHVRVVRFAAVVEEVLQEVCLVEFSSSVCTICLLEYYCILDEDDRIGLATYSLLLVSFCFNVYILC

YIGELLMEKSSQIGYICYMINWYQLSPKTARSFILMIAMASHPIKISAGRMADLSLSTFGNV

>CfOr6

DSSYRRNIWNVFKLNNWILGLIGIWPVTIRGIGRHAYKIAIAVCNFTFSFALVPCALHIIYDQKDIIIRLKIGGLLIFGF

AAMIKYCILAIRRPKIHRCIEYMKSDWWQVTFKSDRKVMLKFAAISRNLTMIGASFMYTAGIIYYLIPFFFEHKVNNQTV

RPLVFPIYSKFQFQISPIYEIVYAAHCMCGYIIYSVTSGTYGLAALFATHACGQIEIIVSRLEDLLSGESKQSSKIHQRI

AAIVKDHVRVVRFTIVVEEVLQEVCLVEFISSICTICLLEYACIVQDNKFGLATYSLFLVSFCFNLYILCYIGELLMEKS

SHIGYICYMINWYQLSPKSTRSLILIIAIGSHPIKISAGGMVDLSLLTFGSVLKASMAYLSFLRTL

>CfOr7

SYRRDIRSVFKLNNWILGSIGIWPVTIRGIRRHVYKIAIAVCNLTFSFALVPCALHIIYDEKDIIIRLKIGGLLLFCLIS

MIKYCILAIRRPKILRCIEYMKSDWWQVTFKSDREVMLKYAAIGRNLTIIGASFMYTAGIIYYLIPFFFEHKVNNQTIRP

LVFPIYSKFQFQISPIYEIVYVAHCMCGYMIYSVTAGACGLAALFATHACGQIEMIVSRLEDLVNGKQSPKIHQRIAAIV

KDHVRIVKFAIVVEEVLREVCLVEFISSVCTICLLEYSCIVQDNKLSLANYFMFFVSLCFNIYILCYIGELLMEKSSQIG

YTCYMINWYQLSPKSSRSLILIIAIASHPIKLSAGGMVDLSLLTFGNVLKTSVAYLSFLRTL

>CfOr9

VHYEHDIRYTMQLCRWVLKPIGIWHLIYSRNEKLISLALIIMCFSALCFVLIPSGLHTLFYEKDINIKVKLFGPVGFCLT

STIKYCYLGARGAAFGKCIRHVENDWWAVQYQDHRKMMLKNALVGRRLTMLCVIFLYTGGLSYHTILPLSSRQVSGNVTH

RPLTYPGYLFFDPEASPVYEIVFCIHCLFALITYNITTAACSLAAIFVTHVCGQLQILITLLDDLVEGKRNNNTTVEQRN

NNTTVEQRLGNLTRHHMRILKFSDNIEKVLREICLMELVTSTLIICLLEYYCLTENSDATAILTYFILLISFTFNILIFC

YIGELLVQQYSKIGSAVYNINWYDLSGNKALSLVLIIMMSHYPPKLTAGKFFDLSIYTFGVVLKTSVVYLNLLRTV

>CfOr10

NVHYEDDIRFTMQLCCWILKPIGIWHFVYSQYKKVLSIMLIFACFSVLCLVLVPSAPHTLLREKDINNKLKFIGPVAFCL

TSAIKYCFLGMRGTAIGRCIEHIEHDWQVIQYQNHRKIMLRNALVGRRLTMLCVIFLYTGGMSFHTIMPLSSRTKTNGSY

TDRPLVYPGYIYFDSQASPAYEFVFLLHCLSAVIQYSATTAACSLAAVFATHACGQLQILMTLLDDLVDGKENKNTTVGK

RLILIENKNTTVGKRLILITRHHMRVLRFTTDVEKVLHEICLIELVAATLIICLVEYYFMTENNNAVAILTYFILLISVP

FNFLIFCYIGELVVEEYSKIASAAYEVNWYDLSGHKALDLILIITMSHYPPKFTAGKFCDLSLNTFSTV

>CfOr11

DGWNYSIQLNRWFLKPVGAWPLTLTTMEKISCVILSMISCFLICFLLVPCTLGTILVDSDLDMKIRMIGPISFFLMAVVK

QYILIARSERITECIRHIRADWNRITLNREDRQIMLDNAKFGRWLSFVSAVFMYSGGFFYTTLMPLCAKRTEIIDNETVR

LLSFPIYRGLDPRTSPSFEIAQFTQTLAGYAIYTLTIGVCSLAAVFVMHACGQFRILMLKLENLADGKERKSGKTPEERL

SDIIQYHIRILSFITRTEELLNEIFFVDVVGCTLNICFLGFNMMTEEHRETGTMTFCSLLISFTFNIFILCYIGELLAEQ

CTQIGIKSYMINWYYLPNKGALGLILVMSMSNTTLKLTAGKFMELSLASFCSIMKAAMAYLNLLRTF

>CfOr12

TYLVDNEYSIQIIRWILKAINLWPRSTSIVEKALSKFLIFVCYFLMITTMIPSGLSIFMSQETFEDKLRSFGPLTFWFMA

MINYSCLLMHVDDISSCIKHVKTDWRLIKKIEDRQLMLRNAKIGRFIAGFCAVFMHSGVFSYNVARGFSKDVLYMENSSV

IVRALPYPFYSKLNAHFSPAYEFVFFLQCFSTFVVNSITVAACGLAAVFVMHACGQLKILMSWLDNLVDGQNEERHERHS

MRQRFAIIVKHHLRVLSFVSRTERITNIICLVELVGCTMHICLLGYYCIMDQDNKQNIISYCIILTSVTFNIFIFCYIGE

ILSEQGEQVGKSAYMTNWYLLPGKTALGLVLIILRSNTALKITAGNIVQLSFSTFGDV

>CfOr13

EDKEYSIQPIRWLLRPISIWPVSNSIKERILSMVLLLLCIFLIISTLIPCALAIFLETKDVEAKVHDFGPLSNWALASLK

YCSLLMHVGDIRRCIEHIESDWRAVTKIEEREIMLKSARIGRFIAIFSAIFVHSGVFSYSIFQAMTLNKTIADNVSVHSL

PFAFYDKLDTTRSPAYEIMFMIQCLSTFVVNSIVIASCCVTSVFVMHACGQLKILMSLLDNFIDENEKRDFSQQRDFSQQ

KFAVVVEHHLKVLSFVSHIEKITNVVCLVEIGGCTMHMCLLGYYCILEQDSKEGIVAYVIILISVTFNIFIFCYIGEILS

EQCDQIGETAYMTNWYLLPGNSALGFVLIILRSSIVVKITAGKMIELSLSTFGIVIKSALAYLNILRTL

>CfOr14

IKLTHKNNNDYSLQLTRWFLIPIAAWPQKCSTTEKISLLAHVLACLFLIVIIMVPCLLYVSLEERDIQIKLSAMGPLSHW

IMGIINYWFLLTRSDDIRECVRHMEMDWKLVRRIDDQDMMLRYAKIGRFIAGFCAVFMQSGTLLFVVAKAMTSITILVGN

VTTSMHPMTCPIYTKFDTRFSPANEIMLVVELLSCFIVNSITVGACSLAAVFAMHAYGQLNMLFSWLNNLVMDENKGNEY

AEQKAEQKLAAIVEHHLRVLSFISRMENIMQNICLVELVGCTMNMCLLAYYSITNSDFDAKIMSYIVVYVSMAFNIFIFC

YIGEILTEQCKNVGEKAYMTNWYDLPHKTALGLVLIIARSSNVIKITAGKLFQLSIATFGDVIKTSVVYLNILRTM

>CfOr15

SIKFTYKNNNDYSLQLTRWFLTPIAAWPRICSTIDRVSLQAHILSCLSLITIIMVPCLLYVSLEEKDIQIKLSVMGPLSH

WIMGTINYWLLLMRSEDIRECVRHMETDWKLVRRIDDQEVMLRYAKIGRFIAGFCAVFMQSGTLLFVVAKAMTSITILVG

NVTTSMHPMTCPIYTKFDTRFSPANEIMLAVELLSCFIVNSITVGACSLAAVFAMHAYGQLNMLFSWLNKLVADEENENE

YADQKLAAIVEHHLRVLSFISRMENIMQNICLVELVGCTINMCLLAYYFIT

>CfOr16

TNIQLTYKNNNDYSLQLARWFLMPLGIWSRISTKAEKFISYVHILVCSFLMTIVTVPFFLYVWLEEKDIEIKLSMIGPLS

HWIMGMINYCLLLAYTNDIRKCVQHMEMDWRLIKNNEDQQVMLQQAKVGRFVSGICAIFMQSGTFLFAIVKSLTTTIVIV

GNETISMRLMACPIYSKFDTRFSPANEIMQVIEILSTFIVNSVTVSICSLDAIFAMHAYAQLTVLFSWLNKLVDKNNENN

ENNFAGRRLAIIVEHHLRVLSFISRMESIMQNICLVELLGCTMNMCLLTYYFITNTLDVAKLMSFVIIYLSMAFNIFVFC

YIGETLTEQCNNVGEKVYMINWYELPHETALGLVLIIARSNNVIRLTAGKFFQLSVATFGDV

>CfOr17

TKELHDHSVQLNRWFLKPIGAWPRSTSSSEKAVSRALIFVCYFLIAFTVIPCALNIVLEEKDVELKLRAIGPLSHWLMGG

MNYCSLLLRSADIHRCMRHMEMDWRIIRRSQHREIMVRNAKLGRFVAGFCAIFMHGGVFSYSIVSGMTTVMVPIDDNRSV

PMLQLPCPSYSKFDARFSPANEIVLIMQLFSCFIVNSTTVGACSLAAVFAMHACGQLDILTLCLDKLVEGEKKSDTVQRR

LADIVDHHLRVLRFIARIEDVMHQICLVELVGCTFNLCMLGYYSITWKIDAKSIAAYIIVYISMSFNIFIFCYIGEILTQ

QCKKVGETAYMTDWYRLPHKTALGLILIISRSSSVIKITAGKLIQLSVATFSDVIKTSLVYLNILRTV

>CfOr18

RSDEYHNYSIQLNRWFLKPIGAWPESRTTDRILSRIIQITCYILIAFTVVPCMLYFYFDEQELDIKMNSVGPVSHWIMNG

INYSSLLWRGKDIRRCIEHMESDWCTVSRIEDRVVMLKYARFGRSVAGFCAVFMHCGVFSYSVVNSLSPMIAIIDNQTVI

MRRLPCPFYSKMDTSRDPINEIVLAMQFLSGFIANSITVGACSLAAVFATHACGQFAVLYSWLSELVDEEEEKEEEKRSV

ECKLANIVEQHLRVLNFLSSFEKIMNQICLVELVGCTLNLCLLGYCSIKEARNTKTIATYSILFISMSFNIFIFCYIGEL

ITEQCKKVGEMAYFTDWYRLPHKTALGMILIISRSSAVIKITAGKLIQLSLITFGDVIKTSAAYLNILRTV

>CfOr19

VEVYRQDNDYSLQLNRWFLKSIGAWPESTSMIKNILIKILQLTCHSLIAFTVIPSILYILFEEKDIRLKLKAIGPTSHCL

MGGINYCSLLHHNDRIRKSVEHMESDWRMMKKKQDREVMLKNARVGRVIAGICALIMQGGVLCYNIARGMSRIIVIVGNK

TITTGRLPCPSFNKVDTRISPIYEVVLVLQCLSTFVVNNVTISACGLAAVFAMHASGQLDIVMLRLEELVDKKQELTLAN

VVEHHLRALKFLSRMEAILRQICFVELVGCTFNLCMLGYYTITEEESMNTIITYIMVLTSMMFNIFIFCFIGELVANQCK

KVGEAAYMINWYYLPHKTVLGLILIILRSR

>CfOr20

YEKDIQLSIQLNRWILKPIGVWPKSASWIEKYGYMLINVMCTSLIGFLFIPCAVYITLEVEDTYNTLKLSGPLSFCLMAV

IKYSSLIFRENDIRRGIEYIKNDWMNTRYYEDRIIMIRNAKFGRRLVVLCAFFMYGGAVFYYLAMPFSNGKVTESDSNLT

YQPLVYPVAVIVDARYSPVSEIFFWVQCLSGFIAHSITAGACSVAAVFAMHAYGRMEVLMQWIEHLVDGREDLNLDDRLS

MIVQQHVRILHFISLTEKILREISVVEITGCTLNMCFLGYYVITENKEPARYITYIVLLISLTFNIFIFCYIGELIAEQC

KKISEISYMIDWYRLPKRKGLALVLIIAMSNSSVKFTAGNFFELSLSSFGDVVKTSVAYLNMLRTL

>CfOr21

DYKRDVQLSIQLNRWILKPIGVWPKSESWIEKYIYMLVNVICTSLIGFLFIPCATFAALEVKDTYDTLKLSGPLSFCIMA

VIKYSSLIFRENDIRRGVEYIENDWMNTRHFEDRIIMIRNAKFGRRLVKICALFMYGGAVFYYLAMPFSNSKVMDSDRNL

TYRPLVFPVAVIVDVRYSPISEIFFWVQCLSGFIAHSITAGACSVAAVFAMHACGRLEVLMQWIEHLVDGREDLDERLSM

IVQQHIRILRFISLTDKVLREISIVEIAGCTLNMCFLGYYTIMESNDPARYITYIVLLLSFIFNIFIFCYIGELIAEQCK

KIGEVSYMIEWYRLPGRKSLALVLIIAMSRSSIKFTAGNYFELSLCTFSDVVKTSVGYLNMLRTL

>CfOr22

HNAKYEDDIRYTVQVHRLILGLIGVWPIFKKSRKRFLKGFVRAMCCLLLSFNLIPWALYMFLILDTFKSRLKMTGALCFY

IMVPTMYCTLILREDSIRKCMKHMEKDWQNVKDENDRKIMLDRAKAGRFILICATLFLFASGFTYRLIQPIFRGKIIVNG

NVTIRPLVQHYYIFFDPQRSPAYEIVFSIHLLIGIFIYIIMASVCGVTALFTMHACGQLEMLSTWLENLLNESHVIARRL

AAIILHHIRIRRFLQHIQHLIGEMCFIEIIGSTLVLCLLGYYVITGQNDALSFLTYAIMLVSFTFNIFILCYIGEILNIQ

ANKVYITCCTLDWYWLPSEQARYLILIIAMANYPTKLTAGKVIDLSFSSFG

>CfOr25

ANENHFNYAVQVTRVILRMIGAWPIPKSNAKKIAIRLQNAFCYLLFAFILVPGLLLVFLKERDYKRRVKLIGPLLNCWMG

CMKYSLFIYHAKEIQPCLEQVQQDWQSTVNWNDQKAMLSKARIGRKFAIFSAVFVYIGGLSIRTIVPLSKGRMLTPMNTT

VRALSCPSYFVFDEQASPAYEIVFTLQFFAGLLTYSVTCGAAGLAAFFIMHVCGQLSVLIAKLQHFTDMTEPEDRFLANI

VEHHIKVKNFLKQVEDAMQFIWLVELVGSTAILCFVEYYVIMESDSTATMFTHFVMLISFIISIFTNCYVGQLLTDQSIK

FGLKTSTINWYHLSYQRARSLILIIAISNIPAKISAGGMIEISLCTFSNIITTSMTYFNLLRK

>CfOr26

NEKDMHISLQLNRWILKSIGAWPKSAKTLERCVYLLLNVICSSLIGFLSIPCAVYMLLVDDAYHIVKIFGHLNFCLIAIV

KYFLLILREDDICRGIEHIKNDMNTRYYDERMIMIRSAKFSHRLVVICSFFMYGGXVFYYLAVPFINGKITEDXNLTYRP

LVYPVAVIVDVRYNPVSEIFFMQCLSGFVTHSIIAGACSVAPVFVMHIYGRLEILVFYGIVDERLSIIVQQHIGILNFVS

LMNKTLREISIVEITELNMCFLGYYTITESSRSARYITYIVLLLSLTFNTNIFLFFCKKIGEVSYMIEWYRLPGKESLAL

VLIIAVXRSSIKFTAGNYFELSLSISADVVRTSVSL

>CfOr27

SVCCLLPLICTIPWVLYMFLILDTFKSRLKMTGTHCFYIMVPTMYCTLILREDSIRKCVKHMEKNWXCQSDVNDRKIMLE

RAGCFILICTXLYFSNGFTYRLIPIFRGKIIVNXDVPIRPLVHGHYYIFDLQXGPAYEIMFSIHLLIDIFVYVIIANVSG

VIALFTMHACNHVTARRLAAIILYHIRIHRFLQHIQHLIGEMYFIEIIGSTFVLCLLGYYVITGRNDAHSFLTYAIMFIS

FTFNIFILSYIGEILNNQRNKVYITCCTLDWYLINKLVILXSNCNYPLKLTASKVIDLSFDSF

>CfOr29

RNDIRRTVQVTRYVLNLIGVWPSQDNSSFRAWIKMFRILCQILLYFIFVPGVLKMFLKERNTRRRLKMIGPMCNCLMAVL

KHAVLICRGDRIKDCIRHIEEDWRKLNLAEDRRIMMGNSRIGRSLAILCVAFVYGSGFSYRTIMPLSRGVIITLQNVTIR

PMGFDGYYVFDPQKTPAYEIVFIIQFLSGFVQYSVTSGTCSLAALLVLHACGQLKILIARMKNLTQIKDKNANQKLAAVV

KQHIRIKSFLNKVEEILQYTCLIEVIGCTFILCLLGYYIIMETKNAVSMTTYTILLLTFIFNIFILCFIGELLTDQSMKM

YVTSCTLDWYRIPHKTARGLTLMIAVSSVPIKITAGKFMDLSLNSFGAIVRTSVAYLNILRT

>CfOr40

YYQDDITFITQLTRNVLSTLGVWPPLNRKRERIHKFLLICITYALLYSVLIPGFLFWFFEKRTHVKIQMIPLLLFGFMTV

TKYGNLIFRERQIKRCLKHIEEDWRNVINMNARNMMIESAKTAKRLVALCGAFMYSSGLSFRLVPFVKGKIINAQNITIR

PLPCPGYFSFNSQASPSYEIIFAIQFLSGLVTFSITTGVCGLAAIFVMHACGQLKILIELMRYLVENQQENQQEREDANK

KLAAVVEHQIRIRYFLQLVEHAMQQICLIELIGGTTIICTLGYCIIVEKSNTIAMCSYCFTLTCMMINMFLFCYTGEQLT

AQAEKVAIASCELEWYRLPDKNARGIVLVMIISNLPTKITAGKIMDLSFKTYGDVIKTSMTYLNVLRNV

>CfOr41

YYQDDITFITQLTRNVLSTLGVWPPLNRKRERIHKFLLICITYALLYSVLIPGFLFWFFEKRTHVKIQMIPLLLFGFMTV

TKYGNLIFRERQIKRCLKHIEEDWRNVINMNARNMMIESAKTAKRLVALCGVFMYSSGLSFRLILPFAKGKIINAQNITI

RPLPCPGYFFFNSQITPNYEMIFAIQFLSGLVTYSITTGACGLAAVFVMHACGQLRILIELMRHLVKDQWQKRREDANKK

LAAVVKHQIRIRYFLQLVEHAMQQICLIELIGGTTIICTLGYCIIVEWEKSNTIATCSYLXCMMINMFLFCYTGEQLTTQ

AEKVAIASCELEWYRLPDKSARGIVLLMIISNLPTKVTAGKVMDLSFKTYGDVIKTSVTYFNMLRNV

>CfOr42

NEHYQDDIIFITQLTRNILSTLGIWPSLSTTRERIYKFLLICISYTLLYSVLIPGFLFWFFEKRTHVKIQVFPLLLFGFM

AISKYANLILREGQIKRCLKHIEEDWRNVTNMNARNTMIESAKTGRRLVALCGAFMYSSGLSFRLVLPFAKGKIINAQNI

TIRPLPCPGYFSFNSQASPSYEIIFAIQFLSGLVTFSITTGVCGLAAIFVMHACGQLKILIELMRHLVEDQWQEREDRED

ANKKLAAVVEHQIRIRYFLQLVEHTMQQICLIELMGCTTIVCILGYCIIVEKSNTIATCSYFMSIISMMINMFLFCYTGE

QLTAQAEKVASASCELEWYRLPDKNARGIVLVMIISNLPTKITAGKIMDLSFKTYGDVVKTAVSYFNMLRNV

>CfOr43

NQHYQDDIMYITQLTRQVLSLLGVWPSLNKRKSISERAWKFLLISSILLYCVLIGLLFWLIEKRPRVRVQTIPLIFYGFM

ATGKYSILVCSEGRIRRCLKHIEEDWKFLISMHARDSMIESAKIGRRLVTLCAAFMYGSGLSFSILPFAKGKIVSAQNVT

IKPLPCPAYLFFDIQVSPIYETVFAIQFLSGIVTYSITIGICGLSAVFVMHACGQLKILMDLMRNLVEIQWEEDQELNRK

LAAMVEHQIRIRNFLRLVEHTMQQACLIELMGCTTIVCLLGYFIIMENSNSIAMCSYFITLTSLMINMFMFCYTGEQLTV

QAERVANTSCELEWYRLPDKKARGIVLVIIMSNLPTKVTAGKIMDLSFKTYGDVVKTAVTYFNMLLNV

>CfOr44

ESYISDFYYAIQISLCLLKPIGAWPLDDDKTSRALHKLSMMIATFLLIFTIVPWIMQIIKEKWSVFLILRTICPLLFMLT

VFTSYILLLWHQDEFKFCIDHVADDWRCGIIVEDRNIMLANAKVGRRFGIVSVAFMFSGGMLYGMLPMVLPNMVNANNVT

VRLHPSPCEFLFDSKTSPAYEIVYFLQLLSGCTAYSAFCGICSLMAHFVTHVCGQCDVLMAIFEETVDGGKHNDGSIEDG

SIENRIATAVTRHLHLLRLVSNISNLFTEICLVEFVNASCSICLICYYIVTDNESFIQIFMYLFALASIVFNIFIFCYIG

DLLKERCQKVGTACYAIEWYRMSSKKAIDLIIPIMISRYPATLTAGKMMTMTLMTFSDILKTSMAYFNLLRE

>CfOr45

KQNKNHISDFYYAVQISFRLLKPIGAWPLRQTKIEVIIHGLSIAVATFLQFFSVASWITCIITTKWSLYEILRTACPLIF

TFTVFLRYLLLLLNQNKIKSCIDQIAEDWRNVTIIEDREIMLANAKSGRYFGIISIAFMFGSGIPYNCMPLVLPPIVTED

NITIRLLPNPFYEIAYVLVALSCITAYTTFCGICSLTAKFVTHVCGQCDILMHVFEELIDGGNVDQRISTAIIHHLRILK

FVSDVDKVLNGICLAEFINASCNICLLGYYVIMDQESMLQIFVYFVAFISITFNIYIFCYIGERLVDRCQQVGIKCYMIE

WYRLPQNKARNLMFPIIMSNYPVELTAGKMVKLTMNSFSNILRTSMAYLNLLREV

>CfOr46

DFYYAAQVSFWLLKPIGAWPLEQTKIEIIIYSLSIVLAMFFQLFMIIPWIICIVTAKWSMYEILRTACPLIFSITVFLRY

LLLLFRRDEIRSCIDHVVEDWRNATIIEDRKIMLANAKSGRSFGIISAAFMFGSGIPYTCMPLVLPSVVTEDNVTIRSLP

NPSELLFDNQVSPIYEIVYVLETLSCCTLYTVFCGTCSLTAKFVTHACGQCEILMYIFEDVIDGGRNQGTIDQRISFAIT

HHLRIFRFVSEVEKILNEICLAEFLNASCNICLLGYYVIMDNHESMQIFVYFFAFVSITFNIYIFCYIGEQLVDRYQKIG

VKCYIIEWYRLPENKARNLIFPMIMSNYPIELTAGKMTTMTISSFSNILKMSMTYLNLLRE

>CfOr47

NYRSDAEYVVKVSKILLTPVGVWPLYKSTFDKMKYVLQTSFMFSLMCFLLVPHIIYTFFDAKDLTRYMKVIAAQVFSLLG

IVKFWTMIINRDDIKHCLQQMEIQYRDVESEEDRSVMVKHAKIGRQFTIMYLGLLYGGALPYIIMPLVADKIVNEDNITQ

LPLPYLSDYVFVVENSPFYEVLFVTQIVFSTIILSTNCGVYSLIATCVMHACCLFEISRRHMETFLTNDLHKRFGQIIMH

HMRALRFTEMIEKSFNFVFLSEMVGCTIIICFLEYGVLKEDNQMLGTIIYFILVISILVNVFTLSSIGDRLKEESIKIGE

TSYFINWYALPAKNVNGLVMVMIRSNRPSTLTAGKIFDVSLQGFCDVCKTSAAYLNFIR

>CfOr48

NNNYRSDTEYVMKVAKTLLTPVGIWPLYRSTSDKMKNFLQTGIIFGLMCFLLIPHVIYTFFDAEDLTKYMKVIAAQVFSL

LAIIKFWTMIINREGIRYCLQQMEIQYRDVECEEDRLVMTKSAKIGRLFTVTYLGLSYGGALPYIIMPLLEERVVKADNT

TQIPLPYLSDYIFVVDNSPFYEILFVSQILISSIILSTNCGVYSLIATCVMHCCCLFEVVRRQMETVLSNGTDNLHKRLG

QIIQHHMQAIRFAEMIEKSLNIVFLCEMVGCTIIICFLEFGVLKEEGKILNMGTYFVLMTSIFVNVYIISAIGDRLKEES

EKVGESSYFIEWYNLPTKIIADLILVMVRSGRPSTLTAAKIFDLSLQGFCEVCKTSAAYFNFIRAM

>CfOr49

SKRWKDDIAYVTTSYKLVSWPIGVWPLQVYNFYSLLRSILSTCFAAIVVIIPPIEMYMGCTSAGQNVDCIMLSFCGSLAV

LKIILFRIYASNLINNYKSALNDYLTIGNIKERIIMRKHAFIAKIVSFPLLCFSYFCCIVYTLTPFMNHDENNQNVTAEE

YPIPSKCTMKYFHAPINMYKIFVIIQAISLIVVTNANLGSDALFINVTLHVCGQMKILRSHFANLDQIYDYFNKLIQRHI

YLIGMIRELAEVISLILLAELFIISICICIMGFQFIIADKDSVMIGQSLMAQSVFLVKLSVYSFIGNYLKSLMEDIGYSI

YQIAWYEFPIKLMRNLVFIFMQTEAPYMFQAGNFILINLTTLVSILKTSFSYLSVLRMM

>CfOr50

RWKNDIAYAMTPFKLIAWPIGVWPLQNYNFYSLLRNIFLTLCGLMVILPSIELCMGCTDAEQNVDCIMLICCGMLGVLKM

IWFRIYANSLIDNYSSAMNDYLTIENVDERAIMRKHAFIGRIVSCPMLCLSYFSCMIYGLIPFLGYDEGNNITRSMNEDA

ILKYAIPSGCTMEYFHAPASMYKAFCLIQVVAMILSTNAHIGNDALFLNITLHICGQVKILRDHFVDFVHDRFNALIQRH

CYLIMLTRKLADMISFILLIELFIISILLCVMGLQFMIANNDTVMMTKSLMVLCTFLSQLTVYSLIGNYLKSQMEDIRFS

IYQSAWCDFPAKLMKNLIFIFMQTEYPVALQAGNFIMINLSTYMSILKSSFSYLSVLRVM

>CfOr51

NKEMAHEFSLYRRTMWPLGSWPLDHDRNFAKYRALLVIIIQSIMVIYISIGYNKDGILSIIVDQLVLASCSMLSIIKITL

IRLHRDDLMKNLCNAADNWTCIARQEHRQVMLRYTNLGRFVFFFQMGSAYVVVVSLAFGPLLSFAMSSSLQNVTRFEEQM

ELPHEMTCPSDVPIVCYGMYLLQTIQLMFTAMGNVGSDVFLFGICMHLCGQLEILSLELLQFHKGMKNRYSTRMKMMAST

RMKMMALTERHCLLLDLADSIVSTLDTILIAQLILHASLICLLGLQLIVSVHDFAVVGTSIMSFNVLMIQLFLYSYMGET

LSSKTEAISQAAYLNDWYDLPRNIVRDLCFIIARANVPVHIRAGKFYNIDFNSFKNVLKASVSYFSVLQ

>CfOr52

WNKEMAYEFSLYRRIMWPVGSWPFDRNNNFAKFRALFIVITQTIMVIYISIGYNKNAVLNIIVDQFVLASCGILTIIKIT

LIRLHRDDLMKNLCNAADNWTCIARQEHRQVMLRYTNLGRFVFFFQMGSAYIVIAPLIVGSLLSLATSFSLQNVTTSEDQ

MQLPQEMVCPSNVPVVCYGMYLLQSVQLISTSTGNVGSDVFLFAVCMHLCGQLEILGLELLRFHKEKEKENRYWKRMKMI

TLIDRHCLLLNLAKDIVYLLDVILIAQLILHALLICLIGLQLIVSIHDFFLVWRSIMSFNILMIQLFLYSYMGETLSSKT

QAISQAAYLSEWYDLPTNIMRDLYFIIARANVPVRIRAGKFYNIDLNSFKNVLKASVSYFSVLQ

>CfOr53

WNEDAVYALSSYKALAWFVGTWPIEDNTLYSKLRWLFAIVSEILLVITLLMEVYLACENSGDPIDTYVVTASAMLVIVKL

TLLRLQRSTLSTNLFSAIQDWCSVEDAKSRDIMIQHARMARIISLSLFYSGFFAFMLYMLLPVVTANERTFYLPTSCLFE

SVTSLQYVLITFYQVVQLFIAYAGNCCTEGIFVGITLHLCGQLELLMIDFQQISRRRHKQKKFVVRHRRLLRLTETIEDT

YNIIILTQIFTSAILICITGFGLIESVHDTIMTTKSIVIMIVMLLQSFIYSFAGDNLRDQSEALSFAVYDSNWCDFSTDD

IRDLTFIMIKTNIPIRLTAGKFFYVTRATFTDILKTAVSYLSALRVM

>CfOr54

MKTHWNNGMDYGFREIRVLMCMFGMWPLQQNNLVCTFRWILTFIIESFTVTSVMIDYFKNCDDIKDSLELFLIIEASSHA

LINIILARIYKKRIAINVSSAIDDWSLSMKKQSYVTMMEYARLGRIIILSQLMIGIICSFLYFPIAFIRSKQQVATIGNK

TLSLWIFVFPTSCLFKDISYSTYKTIFVMQILQGFIMYVSECIGDSFFFAITMHLCGQLELLRISFVEVGRKRNFLGQWI

RRHYELIILARNIEDAFNLNLLIRLSIITVFIAISGMRIIVSHQDYTDVMKSLLFVQYYIIQSFLFTHTSDVLRNKSESI

ISTIYDSTWYEFSSTAMKDLILIMMRTNIPLQLSAGKFFYITRSTVTDILKTALTYISFLQA

>CfOr56

MKTHWNNGMDYGFREIRAIMCMLGIWPLQQNDLVCTFRWILIFIVESFTVTSVLIDSFKNCGDINDNLELFLIIEACFHA

WLNVFLARLYKKKIATNVSSAIDDWSLSMKKQSYVTMMEYARLGRIIILFQLMIGIICAFLYFPIAFIRSKQQVAISEIK

LYLYGIFALPTSCLFKDISYSIYKAIFVMQILQGFIIYMSECVGDGFFFAITMHLCGQLELLRVNFVEVGRKIYRNFLGQ

WIRRHYELIILARNIEDAFNLNLLIRLSIITVFIAISGMRVIVSHQEYIDVMKSLLFIQYFIIQSFLFTHTGDVLRNKSE

SIVSAIYDSTWHEFSSTTMKDLILIMMRTNVPLQLSAGKFFYITRSTTTDILKTALTYISFLQA

>CfOr57

HWNNGMDYGFRTIRVMMCMLGIWPLQQNDLVCTFRWILIFIVETSMLIDSFKNYGDKEQSEFFLLIEACFYAWLNIILAR

IYKKRIAINVNSAIDDWSLSIKKQSYVIMMKYALGQIITLSQLMIGIIYAFLYSTAVFIGNKQQIVVTIGNDTVFIIHXI

SSYLFKEISYSTYKAIFVIHILQRLMXISERDSFFFAITMHLCGQLELLRINFVEVERKYQNFLRPWIRRHHELIILARN

IEDXFNLNLLIRLSIITVFIAISGMRIIVSYQDYTDAMKSLLFVQYFIIQSLFTHTGDVLRNKSESIVSAIYDSTWHEFS

XMKNLILIMMRMNIPLQFNAGKFFYITRSTTTDILKTALTYISFLQA

>CfOr58

WNAETAYVLTIYKYLLGIIGLWVLDEENVFSRIRWFISTMVEMTATISLSLEVIRHCHGYEDAFEAFLSASSSVISILKL

LLHRVNWRDKLILVQAIVHDWTYVKNPHSRDIMLKYARTGRLFSVMFYVGCASCVFLFSLFIFANLDLPWISLEQQNYNK

TSERRLLLSTYCVFETYTSLAYGFVEVLQTLQIFVNCISQCGNDGFFFDLTMHVCGQFEVFRVDFVEISSKQSSSKQSLS

RNKLGLLLKRHHRLIDLAHHLQKAYSLVILSQLLMSVMLLCIEGFQLILTIHNRFATMKHFLYIVVLLIQLFLYCFAGQT

LECQSQGLAYAIYETPWYNFNVSVIKDFPLMILRAAHPHQLTAGKFLAVNFDSFKEILKASASYLSVLRVM

>CfOr59

AKRWSDDFAYAFSIHRIFLKIYGLWPLQEQTLFTKIRYVFCVTAQFMILPFVTLDLMWNNENAGTGIESILYFVSTVLGM

IKHVCIAIGQKKLSINLDAAIDDWLSTKENEETRKIMKKYAARARILTLMLLYSGGGCFSIYMSAIVFINLKQIFFTDPL

SADANTTYWMLLVPSGPLATSITGSQYVILLIFQIVQTSLVCSTQCVIDSFFFNITLHLAGQVEVLKNKFKIFANDSNTN

DSNTEANYRKKFVSLVDRHGELMKFYQNLEDTFHLLILVQLVMVTIMLALIGLRINLCEKDHVEAAKSIVVLNYLLMESL

VLTYGGDFLQRESEGIFYALYATSWFTLPVKLMKDLHFAMMRSSIPFRLTGGKFFYVNRETMMYILKTAASYVSVLR

>CfOr60

RNWNDETHRAINVYEKVLGIIGVWPLNAGEPKSIVRCSIAILIQISTIGSLSLEAYRQCLGTEDMMEAFLMDLSSVVSLS

KLLVIRLTWQHTYILVTSLIDDWSISWDARRREVMTRYTNVGRVLTILYLGYASGMSFLFMAIPFDDLIPWLNASKANNN

ATVMPTYFLATYCVFGSLSGIVHSCVLLLQAAQIFVNATSHCGNDGFFFGLTMHLCGQFEVLEMDFADIDERVCKRRMRM

LIGRHCRLIKLADSLEYAFSMAIFAQTLMSILLLCVEGMQLLISLNDNIAAIKHIVLILTMLVQLYLYCYAGNQLESISE

RLAYSVYDSPWYDFDVKIMKNLPMVMLRGVIPHQITAGKFLPMNLFSFKEILKATGSYLSVLR

>CfOr61

ITTLLVPKLALSCVGIWPVKKKDFFMDLRWIIAVFLEASTVLPMFTEIYLHCNGTKKSFDSLTTGAAAMLALTRLITPRI

HREELLEIVTSMTDDWATQKDKRVRWIMKKYATMSTRVTTLTFILVGIIVSVYTSMAISAITGKKHGNEIENANTSREDR

IRESCVFRSESSRQMFMIVQAMQMLITGISTFGTTSFFFGLAMHLCAQFDALCVQLSEFRVNQAHRAIAEAVQRHCQLIR

LANCMEESFNANILMYLFVTTTLMCIDGFMLIVSLGNLSMIIHNSSVLLLMLIQLSFYTFAGDCLEMRSTALSYATYDCD

WYELPTNVARDFQIILMRASIPHQLTAGKFLPMNMIMFKDILKSTASYLSVLRVM

>CfOr62

ERWTNDFAYAMTPFKLITWPIGVWPLQVYDIYSLLRCILGTFCASLVVILPSMEIYMGCTDVEQNIDCLMIICCGFLGAL

KITWFRIYANSLIINYNSALNDYLTIDNTKDRDIMRKHAFVGRILCSSLLILTYCCCLIYGIIPILNYDISNRINITNED

MILEYALPSRCALKYFNFPSSMYKIFCLIETVVMILASTTNLGNDALFLNITLHICGQVNILRIRFINFDRIYDRFNVLV

ERHRYLITLARELANLISFVLLIELFIISILLCIMGFQFIYAINNTVMMGKSLVVQMLFLTQLTLYSFIGNYLKSQMEDI

GLSIYQSAWYSFPKKLARNVIFILLQTKYPVALQAGNFIIVNLSTYVSILKSSFSYLSVLR

>CfOr63

DDVACAMTPFKLITLPIGVWPLQVYDIYSLLRCILGTFCASLVVILPSMEIYMGCTDVEQNIDCLMIMCCGFLGVLKTTW

FRIYANSLIINYNSAINDYLTVDNTKDRDIMRKHAFIGRTLCSSSLTIAYISCLTYGIIAILNYDMLIRNRTNEDTTLEY

VIPSRCTLEYLNFPTYMYHIFCLVETVLILLATTTNLGNDAMFLNIILHVCGQVNILRSHFLNFDIYDRFGVLVKRHCYL

ITLARELANLISFVLLIELFTISILLSIMGFQFILAVNNTVMVGKSFMVLSAFLTQLTLYSFIGNYLNSEMEEIGLSIYQ

SIWYNFPRKMAKNVIFILMQTKSSVALQAGNFIEVNLSTYVSILKTSFSYLSVLRIM

>CfOr64

RERWKDDITYAITPLKLITWPIGVWPLQVYDIYSLLRCALGICCASLVVILPFMEIYMGCTDVEQNIDCLMIICCGFLGA

LKTTWFRIYANSLIINYNSALNDYHTIVNTKDRDIMKRHAFIGRTICSSLLTIAYICCLTYGLIPILDYDISNRINKTNE

DTTLEYVIPSRCTLEYFNFPTYMYTIFCLIETVVMLLAATTNLGNDALFLNIILHVCGQANILRIRFINFDVRIHDRFNE

LIQRHHYVIMLARKLADLISFVLLIELFIISILLCIVGFQLIFAVNDTVMIGKNLIILSGVLTQLTLYSFIGNYMKSEME

EIGLSIYQSAWYNFPRKLVKSVIFILMQTKSPVALQAGNFIVVNLSTYVSILKTSFSYLFVLRIM

>CfOr65

ERGKDDIAYAIIPFKLITWPIGVWPLQVYDIYSLLRCVLSTCCVSLVVILPSMELYMGCTDVGQKIDCLMLICCGILGVL

KMTWFRVYPNSLIINYNSALNDYLTIENTKERDIMRRHAFIGRILCFSLLAGAYIGGLAYAIIPFVNYAKGNQINITNED

VILEYALPSRCALEYFNFPISMYKISCLIQMIVLILAPTTNFSNDALFLNIILHVCGQVNILRNRFIKFDVTSSRISSRI

DNRFNELIQRHRHVIMLARELADLISFVLLIELFIISILLCIMGFQLIFAVHDTVMISRSSFILSVFLIQLTLYSFIGNY

LKSEMEEIGHSIYQSVWYNFPRKLIKSVIFVLMQTQSPVALQAGNFIVINLSTYVTILKSSFSYLSVLRIM

>CfOr66

REQWKDDVAYAITPFKLITWPIGVWPLQVYDIYSLLRCALGICCASLIVILPSMEIYMGCTNVDRNIDCLLLICCGILGV

LKTTWFRIYANSLIINYDSALNDYLTIDNIKERLIMRKHAFVGRILCSSLLTLVYFSCLIYGIVPILNHDINNQINATNE

DMTLEYAIPSRCALEYFHFPTSMYKIFSLVETVVMLLAATANLGNDALFLNIILHVCGQANILKIRFINFDVQIYDRFNK

LIQRHRYVIMLARKLADLMSFVLLIELFIISILLCIVGFQLIFAVNDTVMIGKSSIVLSGVLTQLTLYSFIGNYLKSEME

EIGLSIYQSAWYSFPRKLIKSVIFILMQTKSPVALQAGNFIVVNLSTYVSILKTSFSYLSVLRIM

>CfOr67

TMTREQWKDDVAYAITPFKFIAWPIGVWPLQVYNIFSLLRCVLGICCASLLVILPSMEMYMGCNNVERNIDCLLLICCGI

LGVLKTTWFRINANNLIINFNSAINDYQTINNIKERDIMKKHAFIGRILCFFFLTIAYCSGLTYTLIPSLNCDKGNQINI

TNEDMQYNRPSDLEYLIGISNLPIYIIYSVIENIVLILATTANLGNDALFLNITLHVCGQVNILRIRFIKFDVRICDRFN

ELIQRHHYVIMLARELADLINFILLIELFIISILLCIMGFQLILALKFNNIVGKSCLLLSGLITQLTLYSFIGNYLKSEM

EDIGLSLYQSTWYNFPKKITRNVVFILMRAKAPVALQAGSFIVINLSTYVSILKTSFSYLSVLRIM

>CfOr68

NDDIAYVFSTHRALMKIVGIWPLQKKTRFTIMQRSLAIFMKLMIFSFLFMELTGNHRDASTSIETILYFACTMILTLKNF

CIIANQRKLARNIDGAISDWLSAKNDEESYKIMKEYAFKSKMFTSVILYSGFICTALYIFAVIFINVKQRFFQDTYISGT

MEWVFLIPSGDLSKIITGSQYLILIIFQNFQLLILCLMQCVSDSFYINITLHITGQLKILKAKFKTFASKPDNVENNRKH

NVENNRKHLSKLVNRHCKLAELNKNIEDTFHLIILFQLVIVTLLLALLGLRIIFSNNDYIELAKSVLVLNFMFMEALVYC

SGGDLIQRESEDIFRAMFMTSWFTLPATLMKDLRFAMMRSSYPFRLTGGKFFYVNRETIIYVLKTAASYVSVLR

>CfOr69

ERWKDDIAYAMTPFKLLTWPIGVWPLQVYNIYSLIRCVLATCCMSIIVTLPTMEIHMGCTDAGQNIDSIMLIFCGILGVL

KTVCFRIYAENLTNNYGSARNDYLTIRNTEHRAIMRRHAFMGRLLSCFMVCFSYFSVTVYSLIPLLGDDEDNQMNVTDED

IVLEYPMPSRCALEYFSVPESLYKIICLFEFIVLILTCTCNLGNDSLFLNITLHVCGQVKILKASFIDFDSSQVYDRFNA

LIKRMSYLIDMAKELANAISFVLLMQLFLSSILLCIMGFQFILAMNDIVMMGKSLTVLCTFLTQLTVYSFVGDYLKSQME

EVGLFIYQSVWYDLPGKLSKNLIFIIMRAQSPVKLQAGNFIVVNLATYMSILKTSMSYLSVLRVM

>CfOr70

NNRWKDDIAYAMTPLKLITWPIGVWPLQVYDIYSLLRCGFGTCCAILVVILSSMEIYMGCTDVEQNIDCLMIICCGLLGV

LKTTWFRIYPNSLITNYDSALNDYLTIEDTKERDIMKKHAFVGRFLCCSLLGISYFNCLIYGIVPILDYDMNNQINITNE

DMTLEYAIPSRCALEYFNFPMSMHKISCLVETVIIILSTTTNLGNDILFLNIILHVCGQVNILRVHFINFDIYNRFNALI

RRHRYLIALVKELADLISFILLIELFIISVLLCIMGFQFIFAVKNIAMVGKSLIALSLFLSQLSLYSFIGNYLKTEMEEI

GFSIYQSAWYSFPKKLARNVIFILMQTKSPVALQAGNFIAVNLSTYVSILKTSFSYLSVLRIM

>CfOr71

EQWKEDIAYAMTPVKLFTWPIGVWPLQVYNIYSLLRCIVSTCFASLVVILPAMEICMGCTDVTQSIDCLMVICCGLLGVL

KLIWFRHYANSLIVNYNSALNDYLTIDDTNERKIMRKHAFMGRILCSCLMSLAYLPNLVAGIAPILVYDNQINITNESIA

LDYAMPSKCALEFFHFPASMFKISCLLEIVVMLTGATANIGNDVLFLTITLHVCGQVNILRSHFINFDRICDRFNALIQR

HQELITLARELSDLMSFVLLIELFIISILLCIMGFQIIIMVNDMVMVGKSLMALSAFLIQLSLYSFIGNYLKTEMEEIGL

SIYQSAWYSFPTKLARNIIFILMQTKSPVALQAGNFVVVNLSTYVSILKTSFSYLSVLRIM

>CfOr72

SEQYKNDIAYAMTPCKLMSWPVGVWPLQVYDIYSLLRCVLGTCWTSLIVILPAMEICMGCTDVMQSIECLMTICCGSLGV

LKATWFRIYANSLIMNYSSALNDYLTIDNIKERKIMRKHAFIGRILCTSLMTIAYSSNLLAGISKILAYDSNRINITNES

IILDYVIPSRCALEYLNFPTSMFKIVSLVETAALMLGSTTNLGNDLLFVNITLHVCGQVNILRLNFINFDVRICDLFNAL

IQRHQELTTLARELSDLMNFVLLIELFFISILLCIMGFQIIIMNNDIVMIGQSLIIISAFMIQLSLHSFIGNYLKSEMED

VGLSIYQSAWYSFPTNLQRNVNFILMQTKSPVALQAGNFIVVNLSTYVSILKTSFSYLSVLRIM

>CfOr73

SEQYKNDTAYAMTPFKLITWPIGVWPLQVYDLYSLLRCALGTCCASLVVILPSMEICMGCTDVTQSIECLMIICCGLLGV

LKTTWFRIYANSLIINYSSALNDYLTIDNIKERKIMRKHAFIGRILCSSLMSIAYFSNLISGIVPILDYDSNQINIINES

MTLEYAMPSRCALEYFNFPTNMFKIFCLLETVAMIFASTTNLGNDALFLNITLHVCGQVNILRLNFINFDRICDRFNALI

QRHQELITLARELSDLMSFVLLIELFIISILLCIMGFQIIIMDNDIIMIGKSLMTLSAFLIQLTLYSFIGNYLKSEMEEI

GLSIYQSAWYSFPTKLQRNVNFIVMQTKSPVALQAGNFIVVNLSTYVSILKTSFSYLSVLRIM

>CfOr74

EQWKDDITYAMTPIKMITWPIGVWPLQVYDIYSLLRCIVSTCCASLIVILPSMELYMGCTDVEQNIDCLTIICCGLLGLL

KTTWFRIYANSLIINYDSALNDYLTIDNAKDRDIMRKHAFIGRILCCSMMGFSYLSCLTYAIIPFFDYVHSNRINMTNED

KMLKYALPSRCALEYFNFPTSMYIISCLIETVVVIFSTTTNLGNDALFLNITLHVCGQVNILRLHFMNFDRIYDRFNVLI

QRHQELITLARELSDLMNFVLLIELFFISILLCIMGFQFIFAVNDIVMIGKSLMTLSAFLIQLTLYSFIGNYLKSEMEEI

GLSIYQSAWHSFPRKLARNIIFILMQTKSPVTLQAGNFIVVNLSTYVSILKTSFSYLSVLRIM

>CfOr75

EQWKHDMVHAMTPFKLITWPIGVWPLQVYNIYSLLRCALATFCASLIVILPSMELYMGCTDIEENIDCLTLICCGLLGVL

KTTWFRIYANSLIINYNSALNDYLTIDNIKERDIMRKHSFVGRILCLSLLVFTYFSCLIYGVTPFLNYNQDNFINLTNED

TVSKYAIPSRCALEYFNFPTSLYKISCLIEAVILIIAATTNLGNDALFLNITLHVCGQVNILRLHFMNFDRIYDRFNALI

QRHCYLIMLVRELADLISFVLLIELFIISILICIMGFQVILAVNDIVMIGKSLMTLSAFLIQLTLYSFIGNYLKSEMEEI

GISIYQSAWYSFPRKLARNVIFILMQTKSPVALQAGNFIVVNLSTYVSILKTSFSYLSVLRIM

>CfOr76

ARERWKDDIAYAMTPFKIITWPIGIWPLQIHNAYSLLRSILSTCCASLAVILPSMELYMGCTDVDQNIASLTIISCGLLG

VLKMAWFRIYAKNLIDNYNSALNDYLTIENTKERDIMRKHAFIGRFLCCSMCGFCYFGCVMYGIIPLLDYDKNNQIKNEN

MILEYGLPSRCALEYFNFPTSMYEISCLFETVIMILAATANVGNDGLFLNITLHICGQINILRIQFINFDVRIYDRFNAL

IVRHQNLITLARELADLISFILLIELFIISILLCITGFQLIFAVSNTVMIGKSLMALSVFLIQLTLYSFIGNYLKSEMEE

IGLSIYQSAWYNFPTKLARNVIFILMQTKSPVMLQAGNYIVINLSTYVNILKTSFSYLSVLR

>CfOr77

RERWKDDIAYAMTPFKIITWPIGIWPFQIYNIYSLLRSALGTCCASLIVILPSMELHMGCTDVEQNITCLTIICCGLLGM

LKMGWFRIYAKNLIDNYNSALNDYLTIKNTKERDIMRTHAFIGRFLCCSMLGFSYFGCVIYGVTPFFYNPDNRINITNRD

TILKYPIPSRCVLEYFNVPTGMYKISCLVQAVILTIAATANFGNDGLFLNITLHVCGQINILRIHFINFDQIYDRFNALI

LRHQNLITLARELADLISFVLLIELFIISILLCITGFQFIFAVNNTVMIGKSLMALSLFLIQLTLYSFIGNYLKSEMEEI

GLSIYQSAWYSFPKKLTRNVIFILMQTKSPVMLQAGNYIVINLSTYVNILKTSFSYLSVLR

>CfOr78

SEQYKNDIAYAMTPFKLITWLIGVWPLQVYDICSLLRCVFGICCAILFVILPSMELYMGCTDMEENIDCLALICCGLLGV

LKTIWFRINANSLIINYNSALNDYLTINNIKERDIMRKHAFIGRILCFSFVVFTYFSCLIYGVTPFLNSNQDNFINITNR

DTVLKYAMPSRCALEYFNFPTSMYRIYCLIEVVILVIAATTNLGNDLLFLNITLHICGQVKILRLRFMNFDVQIYDRFNA

LIQRHCYLIMLARQLTDLISFVLLIELFIISVLICIMGFQVILAINDIVMIGKSSITLSAFLVQLTLFSSIGNYLKSEME

EIGLSIYQSAWHSFPRKLARNVIFILMQVKFPVVLQAGNFIVINLPTYVSILKTSFSYISILRIM

>CfOr79

SEQYKNDIAYAMTPFKLITWLIGVWPLQVYNIYSLLRCVFGICCAILFVVLPSMELYFGWTDIEKNIDCLTLICCGLLGV

LKMTWFRINTNSLIINYNSALNDYQTIDNIKERDIMRKHAFIGKIFCSSLLAFTYFSCLMYGVNSFMNYNQDNRINITNR

DTILKYPIPSKCVLEYFNFPTSMFKIYCLIEIVILVTAATANLGNDVLFLNITLHICGQVNILRLRFMNFDRIYDRFNAL

IQRHCYLIMLARKLTDLISFVLLIELFLIGILICIIGFQLILAVNDIVMIGKSVITLSGFLIQLTLFSSIGNYLKSEMED

IGLSIYQSAWYSFPRKLTRNVIFILMQVKSPVMLQAGSFIIINLPTYVSILKTSFSYISVLRIM

>CfOr87

FCYTCCCYSLVVILSFMEVYLGCTDVEQNIDYCLTICCGLLGVLKTTWFRIYANSLINNYDSALNDYLTIDNTKDRDIMR

KHAFIGRILCCSMMGFSYFSCLIYAITPFLNHDQADQINITNEDKILEYGLPSRCTLEYLNFPTSMYKTVCLVEIFIMIL

ATTTNLGNDALFLNITLHVCGQVNILRIRFVNFDVRIYDRFNVLIKRHRYLITLARELADLISFILLMELFIISILLCIM

GFQLILANNNTVVVGKSLMILSAFLTQLTLYSFIGNYLKSEMEEIGLSIYQSAWYNFPRKFTKNIIFILMQTKYPVALQA

GNFIVVNLSTYVSILKTSFSYLSVLRIM

>CfOr100

ERWKDDIAYAMTPLKLITWPIGVWPLQVYNIYSLLRCVLCTFCACLVAILPSLEIYMGCTDVGQNIDCLMLICCGFLGVL

KTTWFRIYANSLIINYNSALRDYQTIDDTKERDIMRKHAFIGRTIFSSLLIFAYFGCLMFGIVPILNYNMSNQINMTNED

MTLEYVIPSRCALKYFPTSMYTIFCLIETVLITLASTTNFGNDALFLNITLHVCGQIKILRIRFINFDDTSRICDRFNAL

IERHRYLITLTRELANLISFVLQIQLFIISILLCIMGFQFIYAVNDTAMIGKSLVVQILFLTQLTLYSFIGNYLKSEMEE

IGLSIYQSTWYNFPKKLARNVNFILLQTKSPIALQAGNFIVINLSTYVSILKSSFSYLSVLR

>CfOr101

NERWKDDIAYAMTPFKLITWPIGVWPLQVYDIYSLLRCALSTFCASLVVILPSMEIYMGCIDLKQNIDCLMLICCGFLGV

LKMTWFRIYPNSLIISYNSALHDYQTIDDIKERDIMRKHAFIGRIISSFLLSMAYFCCLTYGIISILDYNINNRINVTNE

DTTLEYVIPSRCVLEYFNFPTSMYKICCLVETAIIILVSTANIGNDALFFNITLHICGQVNILRIHFINFDRIYDRFNAL

IQRHQDLISLTRDLADLMSFVFLIELFIISILLCIMGFELILAANNIIRAGNKLFGLSGFLTQLTLYSFIGNYLKSEMEE

IALSIYQSTWYNFPKKLANDVIFILMQTKSAVELQAGNFIAINLSTYVSILKTSFSYLSVLR

>CfOr102

EYAHGWNRYTMMFMGIWPENKNFDRASSYKAVVPILTMFCFICAPQSANLLFIWDDFDLVIENLSMAITITISMLKTAIF

WSKGRSMKILISSMKKDWNMTVDKRERKIMSDIAKITRNLSIRSTIMAEIVVIAYVTYRYIVIRYTGRQLLFRAYFPYNV

SNSPSYELTFFAQIIACMYAAVTYAAVDTFIATLVLHTCGQLANLRQELINLHNCTKTRFQTKLRKIVRKHEYLNSFAET

IEDCFNMMLLIQMVGCSLQLCFQCLQAFMANEFLAQIIFLMIYVVYILLQLYLYCYIGERLLVESTKIAYAAYDCSWYNL

SAYEAKSLIIIMCRAQSPLQITAGRFCSFNRELFSEVLKKSVTYMSCLYA

>CfOr103

LHYAFTLSRQCMRIVGIWPDLDLNVCRRPKIAFIFATCIMTLYVLIPQVLNLLRTSGSVSQMVELFVAPNITFMAICKLI

ITKYHGDKLRILIASSMTDLMTSKNNWERNTMLNLVRTGRKISITYFVIAIAIIIFACYIRLENVFQNIHQPRRYLVYRF

DYIQKSPNYEITCFIQICGAIYAIFGNYSVDSFISILLLHICAQLINLRTALNNLINKLNNKPILNNKPISSSKFKKGLA

AIIVRHEYLIRNVKTINDCYSSVLCIHVLCGSFQLCLVAFQTSTMDNSNVKIIFLAIYISFILTQLYVYCYAAERLLMES

TNMAFGMYECKWYNIPAKDAKDLMLIVYQSAISLKLTAGIFGNFSMELFGIAIKTTMGYLSALLT

>CfOr104

NYALQLSRQCLRLIGVWPDPHISLSDFRRIRFIIAICAVSIYIFTPQAINLIRAWGNVNRMMECFVAANFSMMAISKLVV

TKYHGKKLRTLIASIMTDWMTSNNNSERNKILKLGKNSKNLSFGYFIATVGTTMLAVYVRLEGVFRNIHKPRRHLPYRFD

YIQKTPNYEITCFIQICGGIYTIFGNYSVDNFISILILHICAQLINLQTTLNNLVDKLKNNPASSKLKNNPASSSKFRKG

LTAIIIRHEHLIRSAKTIDDCYSTVLVIHMMGASFQLCLVTFQIFTMDFSPIRTIYLIFFVSLVLMQLYIYCYASERLLT

ENINMAHTAYDCNWYNILAKDARDLMFIVYRSMIPLKLSAGIFGNFSLELFGIAIKTSMGYLSALLTIR

>CfOr105

SYAFTMSRQCLWLLGVWPDPQVSLNIFRPNIFMIVTCILSLYVIVPQLTNMIRAWGDVGRMIEYVASANFGLMALCKLVA

TWYHGETLRTLMTSIVTDWRISRNNWERDAMLNIARRGRSLSFKCCLAATCTVTFYVSFNLIKFYRNMYLPQRSLVYRFA

YPYNIQKSPNYEITFFIQLSGGVYSAIINCSVDCFISILLLHVCAQLINLRTTLNNLVSELANRSISSSSKFKEGLTAIA

IRHQHLIRYAXTSDEIKRYKYLFSLIMKYLNIKFNIFMITDKLDISVKITFLTFYITLVLTHLYIYCYSAERLLTESTNI

AYGVYECKWYNIPAKDAKILMFIVHGSTIPLKLTAGKFGVFSIEMFGTTVKTSMGYLSALLTMK

>CfOr106

NYAFTTSRQCMRLLGIWPDPNLNVFHRSKVGFMLAMCIMSLYVFTPQVINVIRAWGNVSRMVEFFVAANFSLMALCKLII

TRYHGEKLRMIIASIMTDWMTSKSQLEQKTMLKLARSGRSLSFGYFVIVIGTLIAAYYAHIGSIFRNIHQSRRYLIYRFD

YIQKSPNYEITYFIQLCGGTYAIFSNYSVDSFISILLLHMCAQLINLRTTLNNLIDELNNKTFRKGLAAIIIRHEYLIRR

TKTIDDCYSPVLFVHMLSATFQLCLVTFQIFTMDVSFIKIMFFAFYILLVLIQLYIYCYAAERLLTESTNMAYGVYGCKW

YNISAKNAKDLMFIVYRSAISLKLTAGKFGNFSLELFGIAVKTSMGYLSALLTIR

>CfOr107

NYAFTTSRQCMRLLGIWPDPNLNVFRRPKIEFMLATCIMSVYVFTPQIINTIRAWGNISRVVELFVTANFSMMSIGKMII

TRYHGEKLRLLISSMMTDWMTSTSNWERNIMLKLAKTGRRLNFGYFIAAIGTITFAFYVRLENVLQTMHQPRRYLPYRFD

YIQKSPNYEITTFIQICGGAYAVLGNYSVDSFISILLLHICAQLINLQITLNNLIDKLDNKSISSLTFRKGLTAIIIRHE

HLIRILGYWYSCVSVIIYHIMYYFTILLLIKKYYFQMIDNFNISIIKLTIYIFLVLTQLYVYCYAAETLSTESINMAFGV

YNCKWYNIPAKDAKDLMFIVYRSVISLKLTAGIFGNFSVELFGIAVKTSMGYLSALLTIR

>CfOr108

SYAFALSRQCLRMLGVWPDPCIPLSNFRRPSIRFITVTCILSLYVIMLTNMIRAWGNVIHMVEYIASANFSLMALSKLIA

TWYHSETLRTLMTSVMIDWVNSWNNPERNTMLRLARRGRSLSSRYYAFATITVSFYMCFNLLKFYRNIHQPQRRLVYHFV

YPYNSQKSPNYEITFIIQLCGGLCTALINCTVDSFISTLLLHICAQLINLRMALNNLVDELANKSISSSISSSKFKEGLT

AIAIRHIHLIRDARTIDNCYSAVLFAHMLAATFQLCFETFQVYTIDVSTFKMAFLLFYVILVLTQLYIYCYSAERLLTES

SGMAHCVYECKWYNIPAKDAKNLIFIVHGSSIALKLTAGKFGNFSMEMFGTTVKTAMGYLSMLVTIK

>CfOr109

SYAFALSRQCLRMLGIWPDPCLNDFHRPSIRFIIVTCILSLYVIMPQLTNMIRAWGNVIHMVEYIASANFSLMALSKLIA

TWYHSKTLRTLMTSVMIDWINSTNSPERNMMLRLARRGRSLSSRYYALATTGVLFYICLNLLKFYRNIHQPQRRLVYHFV

YPYNSQKSPNYEITFVIQLCGGLCTGLINCTVDSFISILLLHVCAQLINLRTALNNLVDELANKSISSSSSKFKEGLAAI

AIRHKHLIRDAKTIDNCYSAVLFVHMLAATFQLCFETFQVYTIDVSAFKMAFLLFYVILVLTQLYIYCYSAERLLTESSG

MAHCVYECKWYNIPAKDAKNLIFIVHGSSIALKLTAGKFGIFSMEMF

>CfOr119

EWAVKLNRFSLRMIGLWPKTDNNVRKSVYNFRPLIIILMTINALIPCIHSLIKIRTNIMLLIENLQFTLPTLTCIIRLII

FWWKKEAVIWILNMVAEDWLKMKSAQERKVMIKKAQTARIIITCGYCMMMTVFILITVLPISGISMRYSYLSNITAPSTL

LPLPTYHAYDVTRSPQYELTFVLQTVSMVFATMAYTGIDNFLGLLVFHICGQLEILRNRIEHLNKFADIHYTLKNNVEDH

TRLLKAIAVIENIFNIMLLVLFLYFGILFACYGFLVINLNNIHLMYEVCIVINTFSHMCLYCAAGEVLVIQYDQLHYAVY

NYNWYTLDPRNARNLIFLMIRSSKPIYLTAGKVFPMTMATFCNLIKTSASYISVLLTTK

>CfOr139

RIVMLLLPLYGIWPGRSMILIIRVFWVITIAFIEFCHYLYFSTHLNIQNFFNLVDCLCSFVAYAKVLIKLVAFWVNQRKL

VETLTLIMDDWSDCAKSDIGMRVTMHKAKLSDRITNAILILHTMSIFVYCLGVIIADADVTDQTIELPFKLTLPFNINIQ

NTYRLILIAEFVHMIFSNWLLGIVNAILLTLVLHMGGQVEILQSWLSQLVPKKNKEESIVTSTNKIIRKHNKIIQFSENI

EILYTYIALLLFASNTILMCSIAFLIVTAPDATEQILKSILFFWNTNLEAFIFCYAGEYLSNKSRAIEFATYNCPWYNLK

SKDIRILLFIILRSQKELTLTAGKIMDLSLKSFTSIMNASGSYLSVMLAM

>CfOr141

TTKFVLTLFGIWPNINISYVMFCRIFWSVTILIVLFCHYLYFLTYYHSDDVFDLLECFSNFLGFFKFMTKITFFWFNQRI

FYEILMMMTKDWNDCSKSDIEIHEAVNKAKTSNLIANAVIILHIVTVLLYGDIILAKIDVTNRTIKLPHIYKIEVPFNIN

TQRTYKIVLIVELIHVVMCSCGTGILNALLLILVLHVGGQINILHCWLTKLISKENKRENKRKSIAIMMKKIIRKHQKII

YFAKNIDSLYTFIAFMQFVSNTIMICIIGFVIITAPNATKKILKAISYYSVTNIEAFIFCYAGEYLINKSKAIGLAAYNI

AWYELEPEYNRLLLFVIIRAQKQLTLTVGKMTDLSLQCFASV

>CfOr145

AKFMLTLLGIWPDISYVMFYRMFWTVTMLTFLFYHYLYFLAHYHSNDVFDLMDCFSSFLGYSKIIMYFVFFWFNQRIFDE

ILTMMAEDWNDCTESDIEMHETVNKAKMSNHITNVIMTLHVISPVLYGMNIILANVDITDHTVELPHIFKMEIPFNINTQ

CTYKVVLIVELIHLVMCSLSLGVINVLLLILTLHIGGQLNILHRWLAKMIFKENKKENKHKSIAIIMKKIIRKHQKIIYF

AKNVENLYTFIAFMQFISNTIMICIIGFLIVTASNATEKIVRMIPYYSITNLEAFIFCYAGEYLINKSKTIGLVAYNIAW

YELEPKYSRSLLFIMLRAQKHLTLTVGKMKNLSLQCFASVLHIISSYLSVLLAM

>CfOr151

LKLMLTLCGIWPGTSCVIICRAYWIIALATDNICHYLLMHLHSSDLFDLMDCFSSFLTQVKFMTKLIIFWLNERKFAEIL

TIMKEDWNDCCSSDINMRETMCKAKLAGRITNAMFTLHTLTIVGYSIGIFLADVDVTDHQSELPLLLKVTLPIDIKTKRR

YKILLSAQFIHLILSGCGTGLLNALLLTLILHIGGQMDILRCWLNEIVIKKNKEMNKIIRKHQKIINFAEYIENMYTYIA

LLQFTLNTVLICSLGFLIVTAPDATEQIVRTLLFYTVTNLEAFIFCFAGEYLKNKSKAIGNAAYYSAWYEMKPENSRNLI

FVILRAQKQLTLTVGKIMDLSLESFTDIMKASGSYLSVLLAM

>CfOr152

ELLLTAFGVWPGISCVLLYRVFWMITLVINQFFHYRYFHFHVNNLFNLMDCLSSFLAHVKLTFKIIIFSLKQREFIGILT

TMSEDWSCCGDNGIVLHETKRKAKLSSRICNGLIILHTIAAFAYVIGILLADADITDRTTELPLIMKMEYPFVIDTLRKY

RLVLTTQFMFVLACSLGAGLFNALFLTLTLHIGGQINILLRWLTEVRSIRMTKIIQKHQKIISFSEKIENLYSYITLLQF

TSNTVMICSLAFLIVTAPDATEQIMRSLLFYAVTNLEAFIFCFAGEYLSNKSTAIGNAAYNSGWYDMKTADSRILLLIIL

RSQRKLKFTAGKMTDLSLECFTNV

>CfOr153

VKFIFTLCGIWPGISCVLFYRMFWIITMTIAISYLVSYLLAHIYTAELIDLIDCLCTILAHLKVISKCFIFWSNQKTLIE

ILAMMAEDWNDCTDNDITMRETSKKAKLSDRLVNAIFILHTTTVSAYCIGLFLSDMDITDQMIELPFKLKVPFHINTQCV

YRLTIVAKSLHVILCGWVAGMTNVLLLTFTLHAAGQIDILCYWITQLTFCENKLMKKIIQKHKKIVTFSENIECLYTYIA

LVQFVSNTVMICSLGFLIVTAIGSPNQIMKSLLFYTITNLEAFIFCFAGEYMSNKSREIGAAAYNSTWYDLNSKDSRVLL

FVMLRSQKQLTLTAGKMMDLSLESFKNIMSASGSYLSMLLAM

>CfOr154

EIWLRIFGIWPDSSCISLRRLFWIIALALQIFQYQYIIINFYSIEFFEVMGMLGEAMTFSILIIKLVIFWCKQRTFSKIL

TMMAIDWKKSLNTEFSMFVTSNAKLSRRFANVTVALYSMAVIFHSSNIVKHTDENKNTSMRPLVMNMNLPFDLNQTYVYV

LIIIIQFVHLLLCSCATGLLNALLINLILHIGGQVDILCEWLIDIFPIKKKIKKIIKKHQQIIKFSEHIEDLYSNIALAL

FISDTLIICCLGFVMVTSVGTPDIIMRTLLFYFVMNMEAFAFCFAGEYLSTKSNSIGNAAYNSFWYESNSKNNQITLFLI

MRSQKQLVITIGKVMNLSLERFSSIIKASASYISVLLAM

>CfOr155

IEIWLRIFGIWPDSSCISLRRLFWIIALAIQIFHYQYIIIHFYSIEFFEVMGILGETMTFSIIIIKIVTFWCKQRTFCNM

LMMMAIDSEKCSSREFSMSVMTRNAKLSRRFANLTLGLYSMAVILHSSHIIVKHTGDDKISNTSTRALVMDMNLPFDLNQ

TYVYVLIIIIQFAHVLLCSCANGLLNALLINLTLHIGGQIDILCEWLMDIFPIKEKHIKKIIKKHQQIIKFSEYIEDMYS

NIALALFVSDTLIICCLGFVIVTSPDAVKIIMRTLLFYFVMNMEAFAFCFAGEYLSTKSNSIGDAAYNSFWYESNSKNNQ

ITLFLIMRSQKQLTITIGKVTNLSLEQFTSIIKASASYISVLLAM

>CfOr157

ILLRIFGIWPDASHIPLRRVFWTIAIIMEQVLEYKWIVVHFYTNEPFEVMKFLSEAMTYTIMFLKIIIFWVKKRTFVRIL

TMMSIDWENRIIDEVSMITTTHNANLCRRFNNGTIILYTIAVLFHISNVYTNFMDEQTSNTSTRPLVMNMDLPFDLSRTW

VYVSVLIIQFIHLILCAYINGLLNILLINLILHVGGQIDILRKWLMEMFPTERRLSEESSLSKVIKKHKRIITFSEYIEE

MYTNIAMVLFVSDTLIICCLGYILVASIGTPTIIMRTILFYVVSSMEAFIYCFAGEYLTNKTSSIGDAAYNSCWYEGNSR

ESKFIVFLIMRSQKQLTITIGKIMELSMERFTSIMKASASYISILI

>CfOr158

RVVKIGLRTYGIWPYLPSTALCRLLCIVLLSAAQIFQYVLINYHTDSFSNFMDGMSSAMTYSLLFMKLAILWINERTFSD

MLQMMAMDWKNCVLTECSLRITSNKARLSYRFSNWIIGLQMIAITLYSCGVLAVNAGDVQRMNVSAREHILKMKLPFKVN

TFPVYTLVTIFEFFHLMMCGLAISVINSLIITLILHIGGQIDILRDWLLKAFSKNMKMLITKHQRIIMFSENIENLYTYI

ALILFVSDTLIICCLGFIIVTSINTPAILVRSVLYYLVMNLEAFIYCFAGEYLTAKSKMIGDAAYDSLWYDVTSKQSQII

HLIILRSQKRLTITIGKIMDLSLERFTSVLKISASYVSVLLA

>CfOr159

RLVKFSLHIYGIWPYVPSTVLFRLYWIMMLSTQVFQYRYVIVNIYMDDFSELMDGISSAMASSLLYIKLVLLWSNQRIFF

DLLQMMSADWQDRQDTVYNLRIMAKTANAAQRASRWIIGLQIFSVFNYAGGVLANNMDKEEPYKRELILKMELPFNISTN

SIYAAVQSVQFYHLILVAYGITTVNSLLVTLILHVSGQIDILRERLMKVFSKSTVDSEEIQSLLSKHRQIIIFSECIENL

FTYIAFIILLSDTIIICCLGYVIVTSLDMPNILVKSVVFYITINIEAFIYCLSGEYLSAKSTMIANAAYDSLWYNFPSKQ

SRIILFVILRSQKRLTITSGKIMDLSLERFTSVIKASASYLSLLLAM

>CfOr160

RLVRFNLHIYGIWPYVPSTVLFRLYWIIMLSTQVFQYRYVIVNIHMDDFSELMDGIGSAMASSLLYIKLVLLWSNQRIFF

DLLQMMSADWQDRQDTINSRVMTETQNAAQRASRWIIGLQIFAVINYTAGVLANNLDKEEPYKRELILKMELPFNISTNS

IYTAVQSVQFYHLFFVACGITTINSLLVTLILHISGQIDIFRERLMKAFSKSVIDSEEITMQSLIVKHQKIIVFSESIEN

LFTYIAFMILLSDTIIICCLGYVIATSLDMPNILVKSVVFYITINIEAFIYCLSGEYLSAKSKMIGNAAYDSLWYNLPSK

QSRIILFVILRSQKRLTITSGKIMDLSLERFTSVIKASASYLSLLLAM

>CfOr161

SVEFGLRAIGVWPDTSYAILRRILYISSMIVFQIFQYRYLIMGKEDLFILMDVLSITLAYSLVLIKLIIFACNAHLLNEI

ITRIVEDWKRHDVSEEYTMTRIAYLSRRFSNLIITMYMISVFLYATGTLLRYNSNNQTDARELILKMELPFEMKSTSVYI

IVLITQFIHQISAASTTGVLNSLLIILVLHACGQIDIMRHKLSAITQKNIMKTLIIRHQRIISFSKDIEVLYSSIALIQF

VSNTLVICCLGFLIVISPGGSAVLVKSILFYVVICLDAFIFCFIGEYLSTKSRMIGDAAYESLWYESNPNLNRNILLMIV

RSQKHLTLTAGKFMDLSLQEFANIVKASASYVSVLHAM

>CfOr162

SVEFGLRAIGVWPDTSYAILRRILYISSMIVFQIFQYRYLIMGKEDLFILMDVLSATLAYSLLLFKLIVFAFNAHLLNEI

ITRIVEDWKRHDVSEEYTMTRIAYLSRRFSNLIITMYMISVFLYATGTLLRYNSNNQTDARELILKMELPFEMKSTSVYI

IVLITQFIHQMSAASTTGVLNSLLIILVLHACGQIDIVRHKLSAITQKNIMKTLIIRHQRIISFSKDIEVLYSSIALIQF

VSNTLVICCLGFLIVISPGGSAVLVKSILFYVVICLDAFIFCFVGEYLSTKSRMIGDAAYESLWYESNPNLNRNILLMIV

RSQKHLTLTAGKFMDLSLQEFANIVKASASYVSVLHA

>CfOr163

QSVEFGLRAIGVWPDTSYAILRRILCISSIIVFQSFQYRYLHFNENDLFILMDVLSGTLIYSLLFIKLIIFMFNAHLLNE

IITHVVEDWKRHDTFEESIMIRIAYVSRRISNLIITLYAMTVFCYATSTVLRYKIGNQTDARELIFKMELPFEIKSTSVY

IVVLVILFVYQTSAASTTGILNSLLITLVLHVCGQIDIVQRKLHEITRKNIKQNIKQNITESIMKKLIIRHQKIILLSKN

IESLFSGIAFIELFSNTLIICCLGFIIVVSPGGTTVLLKSLVFYIMICLDAFVFCFTGEYLSTKSRMIGNAAYESLWYES

NPNLNRNVLIMIVRSQKHLQLTAGKFMDLSLQQFTNIVKASASYVSVLHAM

>CfOr164

QSVEFGLRAIGVWPDTSYAILRRILCISSIIVFQSFQYRYLHFNEDDLFILMDVLSGTLTYSLLFIKLIIFTFNAHLLNQ

IITHIVEDWKRHDISEENIMTRIACVSRRISNLIITLYAMTVFCYATSTVLRYKIGNQTDARELIFKMELPFEIKSTSVY

IVVLVILFVYQTSAASTAGMLNSLLITLVLHVCGQIDIVQRKLHEITRKNIKQNIKQNITESIMKKLIIRHQKIILFSKN

IEGLFSSIAFIELFSNTLIICCLGFIIVVSPGGTTVLLKSLVFYIMVCLDAFVFCFTGEYLSIKSRMIGNAAYESLWYES

NPSLNKNVLLMIVRSQKHLQLTAGKFVDLSLQQFTNIVKASASYVSVLHAM

>CfOr165

STEFGLRAIGVWPDTSYAILRRILCISSMAVFQTFQYQYLFMHFEEEDLYILMDVLSGTVAYSLLLIKLIIFAFNAHLLS

EIIAHIVEDWKERDVSEEYTMTRIAYISRRLSNLIIIMYAMTVFLYATSTVLRYKSTNQTDTRELILKMELPFEMKSTSV

YIAVLVIQFVHQTSAASTEGVINSLLITLVLHTCGQINIVQQKLSEITQTNNIMKTLIIRHQKIISFFKNIERIFSNIAL

VQFVSNTLVICCLGFLIVVSPNGTTVLVKSVLFYIAINLDAFIFCFVGEYLSTKSRLIGDAAYNSLWYDSNLNQHRNVLL

MIMRSQKHLQLTAGKFVDLSLQQFANIVKASASYVSVLHAM

>CfOr166

FGLRVIGVWPNTSYAILRRILYISSMAMIQIFQYRYLIMHFGDLFTLMDVLSATLAYSLLFIKMIIFTFNVHILNKIIAC

IIEDWKIRDISEEYTMTKVAYISRQFSNLIITMYAMSVFVYATGTLFRYKSSNQTDTRELILKMELPFEIKNTLMYIAVL

ITQFIHQVSGASMEGVFISLLITLVLHVCGQIDIVRQKLNKITRKNIENMKTLIVRHQKIISFSKNIESLYSGIALMQFV

SNILVICCLGFLIVISPGGSTMLVKSLFFYTVVCIDAFILCFLGEYLSTKSRMIGDAAYESLWYESNPNQKRDVLLMIMR

SQKYLTLTVGKFVNLSLQQFSNIVKASASYVSVLLAM

>CfOr167

EFGLRAIGVWPDTSFAALRRILCISSVAVFQIFQYRYLILHFGDLFILMDVLSATLAYSLLFIKLIIFTFNAHMLDKIIA

CIVEDWKIRDISEEYIMTRIAYISRRFSYLLITMYAMSVILYAAGTLLKYKSNNQTDTRELILKMELPFEIKSTSVYIIV

LFTQFVHQTSAASTIGVLNSLLITLVLHVCGQIDIVRQRLNEITRKNIKMKTLIVRHQRIISFSKNIESLFSSIALVQFV

SNTLVICCLGFLIVISPGGSTMLVKSVLFYVVICLDAFIFCFVGEYLSTKSRMIGDAAYESLWYESNPNQNRDVLLMIIR

SQKHLTLTVGKFVDLSFQQFANIVKASASYVSVLHAM

>CfOr168

FGLRIIGVWPDTSYAILRRILYISSLAMFQIFQYLIMHFGEEDIFTLMDVLSVTLAYSLLFIKLIIFTFNAHLLNDIIAR

IVEDWKKDDVSEKHTMTRVAYISRRFSNLIITMCAIAVIVYAIGSLIRYKSGNQTNARELLVKMELPFEIKSTSVYIAVL

VIQLVHQTSAASIEGMLNSLLVTLVLHTCGQINIVQQKLNKITQTNNVMKTLIIRHQKIISFFKNIERIFSNIALVQFVS

NTLVICCLGFLIVVSPNGTTVLVKSVLFYIAINLDAFIFCFVGEYLSTKSGMIGDAVYESLWYQSNPSQNRDVLLMIIRS

QKHLTLTVGKFMDLSLQQFANIVKASASYVSVLHAM

>CfOr169

EFGLRVIGIWPDTSYAILRRILCISSLAMFQIFQYRYLIMHFGDIFIFMDVLSVTLAYSLLFIKLIIFTFNAHLLKEIIA

RIVEDWKRHDVSEKHTMTRVAYISRRFSNLIITMCAIVEFIYATSTLIKYKSSNQTNARELLVKMELPFEIKSTSVYIAV

LVIQLVHQTSVASTEGMLNSLLITLVLHVCGQIDIMQQKLNKVTRKNKEQDAKNKEQDAAKNIMKRLIVRHQKIISFAKD

IENLFSTITLIHFISNTLVICCLGFLIVISPGGTTILVKSVLFYAIICLGPFIFCFVGEYLSTKSGMIGDAAYESLWYQS

NPSQNRDILLMIIRSQKHLTLTVGKFVDLSLQQFANIIKASASYVSVLHAM

>CfOr170

QSVEFGLRVIGVWPHTSCAILRRILCLSSLAIFQIFQYRYLIMHFGDIFILMDVLSITLAYSLMFIKLIIFTFNAHLLHE

IIACVVEDWKRHDVSEKYTMARVAYFCYRLSNLIITIYAMSVFVYASGALIRYKSSNQTDARELLVKMELPFEIKSTSIY

IAILITQFIHQMSTASTEGVLNSLLISLVLHVCGQIDIVQQKLNEITRQSMKKVIVRHQKIISFCNNIEGLFSNIALIHF

VSNILVICCLGFLIVISPGGSIVLVKSVLFYVAICLGAFIFCFVGEYLSTKSRMIGDAAYKSLWYESNSNQHRDVLIMIL

RSQKQLTLTVGKFVDLSLQQFTDIVKASASYVSVLHAM

>CfOr171

FGLRAIGIWPHTSYAILRQILCISSMAIFQTFQYLFMHFDEEDLFILMDVLSGTISYSLVFLKLIIFAFNTHLLNEIIVH

VIEDWKKRDVSEEYTMTRIAYISRRFSNLIIIIYAMSVLLYAIGTLFRYKNSNQNDARELILKMELPFEIKNTSVYIAVL

ITQFVHQTSAASTEGVLNSLLITLVLHACGQIDIVRQKLREITRKNIEQDAQDATESIMKMLIIRHQKIISFSKNIENLF

SIIALIHFVSNTLIICCLGFLIVVSPGGTMVLAKSVFFYLAICLDAFIFCFVGEYLSIKSRMIGDAAYESLWYESNSNQN

RDVLLMMIRSQKHLTLTVGKFADLSLQQFANIVKASASYVSVLHAM

>CfOr172

NGKDSMRAVCRSVEFGLRAVGVWPGTSYAILRRFFCIFSMGVFQTFQHLIMHISEKNLPLLMDVLSATITYSLLLVKLII

FAFNASLLNKIITVMVDDWKEREISDNYTMTRIAYISRRISNFIIVSHALSVFLYATGTLLRHRNDNQTDTRELILKMEL

PFAIDSTSIYVAVLVIQFVHQTSAASMAGVMNSLLITLVLHVGGQIDIVREKLSKISRKSIERSTSKTLIVRHQRIISLS

KNIEALFSNIALIQFVSNTLVICCLGFIIVISPGESAVLVKSVLFYILISLEAFVLCFVGEHLSMKSEMIGDAAYESLWY

ELNPNQNRDIFFIILRSQKHLTLTVGKVMDLSLKQFASIVKASASYMSVLHAM

>CfOr173

MQSMTTVSRSVEFGLRAIGVWPGTSCAILFEVLSISSMVIFQIFQYRYAIVNFGDFTLLMDALSVTFAYTLLLIKMIIFA

LNARLLNKIIEHVVKDWEECNILDKYTMTRMAYISRRFSNMIIFSHTISVFLYATGALLKQKDDNQTDARELIVKMELPF

EIESTPIYVTILITQFLHQSSAAAMVGVLNSFLITLVLHACGQIDIVRQKLSEITRKNDERDATQATQSIMKTLIVQHQR

IIAFSKNIEALFSNIALIQFVSNTLVICCLGFLIVISPDGSMMLIKSVFFYIVMSLEAFIYCFVGEYLSTKSEMIGDAVY

KSNWYELSPSQNRDILLMIIRSQKHLTLTIGKVADLSLKQFADIVKASASYVSVLHAM

>CfOr174

IGLRMIGVWPDSSYAFLHRAFWMITLMMQTFQYRYFVVHIRTDDLSHLMDGLSTTMSYSLLLLKLTIFWINRRIFHDILM

MMARDRSECATDWAVCSMSRTIYVSHRSSNLIIGLYSMSVFLYGTGVLVAHADETDDDEDVQLTVPARELFLKMELPFES

NVSPVYEVVMVTQFFHQLAAATIVGVLNALIVSLILHVGGQIDIMCRGLEEISSDDDVFIKTLIRRHQRIIALSADIETL

FSYIALMQFLWNTLVICCLGFLIVTSIGDTTMLIKSLFFYVVITLEAFIFCYAGEYLSAKGRMIGDAAYEAKWYNLSPTQ

SRILLLLILRSQRKLTITIGKFMDLSLERFTTIIKASGSYVSVLHAM

>CfOr175

FGFRFVGIWPGLPYGTFTWYTFMTSIVIAMYFEYVYIFDHFDIDDISNLIDALSIALACSLSFLKLISLWSHRRIFYDIL

LAMDEDWSDVLNHDRSMLHTMSSANLSRRCSNVLISINATAAVCYAATSFTRRSISLEENFNDSLRVLPLKMQFPFEVNA

SPFFELLAVAQFFHVVSVAALTATINCMIITLVLHVSGQIDILRRDLLAICCDEDSQRDSIITSIRDSIITSIKHLITRH

QRIITFSDNIEELYSDIALMQFLSNTVVICCIGFTIISSDGGTEVLLKSAIFYVAVTLEAFIFCFVGEYLSAKSKSIGDA

AYESLWYYMTPADCRIILFVILRSQKRLTITAGNVMDLSLEGFTTVMKASASYMSVLHAM

>CfOr176

LKIGLQCLGVWPNVPYSTVYWFLVMLSTLIVQYFQYLYVFSHFKELSNLVDGLSSTLESSLMFIKVASLWKHRRILHQLL

AAMDNDWRECNDVHQHLNIMTIKAGISHFCSNAMFSFNTFASVLYLLGDYVIRFVYLTKDYNNSLRQFPVKAQFPFETEQ

SPIFELLVLGLFLHVMSDSFTIAIVNGLIFSLVFHMSGQIDIICQSFRTISKSILGTLIERHNRVVLFSEQIGKLFSFIT

LMQIAANTLVICCIGFLITISESGFFVLLKAILAYVAIMIEAFIICFAGEYLSVKSKCISDAAYKSLWYDLPPREGKAIS

FMILRSQKRLVITAGKITNLSLETFAKIIKASASYVSVLHAM

>CfOr177

LKIGLQLLGMWPDVPYSAVYWLMFMSSMLIIQYFQYVMGHLKMNELSDLVDSLPATLDYTLTLFKMISLWIHRRVLHKIL

IAVDNDWHECVNVEQHFHVMTIKASISHFCSNAMLSFNAIAGVLYVLSDYMIHFVSLVDDYNDTLRQLPIKIELPFEYEQ

SPIFELLVVILFLHTMLHVCAVAILNGLIFTLVLHASGQIDIICQEFRNISDKTSLYDSSKVISFSNNIEKLFSFIALMQ

VVWNTLVICCLGFIIVISGTSVMILIKTIFAYFAIMIEAFIICFAGEYLSLKSKSIADAAYETFWYNMPSSQSKVMTFII

MRSNKRLAITAGKMTDMSFEAFSSMIRASASYISVLHAM

>CfOr178

LKIGLGFIGMWPGSSYGTHLWLFYMATLLAMQYFQYYVFVHLNKNDFSKLMDGLSVTLDYTLTFLKLLSLWHNRRIFSDI

LDAMEDDWNNQVTDSHVYVMTSKANLAHRCSKAMMILNSLATIFYFVGSYLSHRIISAGEEPREFPIQMQFPFDQADSPI

FELIYLATFFHVWETATVIAMLNSLILALVLHVSGQIDIMCQELREISSTKKSHRSLIERHQKIISLSNNIDNFFSFVAL

IQFVWNTIVICSIGFMIVISEGKSGILIQSIIPYVAVTLEAFVFCFAGEYLSSKSRSIGDAVYEIHWYELSTNECQILLL

IIVRAQKQLTITAGKVMDLTLEGFTTVMKASASYISVLHAM

>CfOr179

LLRMGLRVIGMWPDSSYGMFCWLFYMTTLLIVQYFQYSYVYAHLEFDNLIKLMDGLGITLDSTLTFMKMISLWFNRRIFV

DILVAMDNDWKDATDLHKCVMINKANLAYRCSNAMIFVNIVATVLYFIDSYARIRIFSKDGQYPKFPIQIQLPLKAHETP

VFDFIVLGLFFHVLETAIAIAVLNSLILTLVLHVSGQIDIMCQELKEIPSIFKSKKKSLIERHQKIISLSNNIENYFSLI

ALLQFVWNSFVSCCLGFMISFDENKSGVFTQFIIPYLAVSAEAFIFCFAGEYLSTKSRSISDAAYETVWYDLSISECRIL

LFLILRSQKRLPITAGKVMDLTLESYTTVMKASASYISVLHAM

>CfOr180

LLRIGLRAIGMWPGSSYGTFWWLFYMTTLIIMQYFQYSYVYAHLDFGNLTKLMDGLGLTLDYTLTILKLISLWFNRRIFA

DILTAIDDDWKDRPTDLRQCVMMDKANLAHRCSNAIVSVNAVATVLYFIDSHVRRHTISKDGQHREFPIQVQFPFETHET

PIFEFVVLGLFFHVLETATVIAILNSLILTLVLHVSGQIDIMCQELKEISSTLKSKSLIERHQKIISLSNNIENYFSFIA

LLQFIWNSFVICCLGFMVIIVESKSGIFIQFMMPYLAVSIEAFVFCFAGEYLSTKSKSIGDAAYEAVWYDLSTSECRILL

FLILRSQKRLTITAGKVMDLTLESFTTIMKASASYISVLHAM

>CfOr181

IGLRFIGLWPDSTYATFYWFSYMTSIVIVQYYQYYVLGHLEINDVWLLMDCLSLTLAYTLAFFKLLVLWWNRRIFYRIVK

DMDEDWKNCIVNSSYMSTMMTMADLSRRFSNIVFTVNGIGAFFLSIGEHLLQSMDDANRINNSSRELPLKMEFPFDVSES

PIFECFLIGQFIYDLVIAFVVGLMNALLVTLILHVSGQIDIMRQDLAEISKHNYDHNHNTFVIIIKDLICKHQRIIALSE

NIENLYTEIALMQILWNTLVICCTGFFIIITTKDISTLIKSVNYYIAITLEAFIFCYAGEFLSAKSMSISDAIYESLWYN

VSPNDSRILLFMMLRCQKRLTITAGKIIDLTLNGFASIMKASASYISVLNAM

>CfOr182

RPVEIGLRFIGMWPDSAYATLCWLMYMVTMVIVQYYQYAYVFAHFDLNNIPLLMDCLGLTLAYTLAFFKLLALWWNRRRF

YFILAAMDRDGRECDINDSYASMMINVADVSRRCSSVMISINALAAFFLSIGEHLLHSLNDVNRVDNNSRELPIKMEFPF

DVSESPIFECFLIGQFLYELLLASIVGMVNALLVSLILHVSGQIDIMRQDISEISNKYDPIKTLICKHQKIITLSENIEN

LFSYIALMQLLWNTLVICCTGFVIIISDESATTSIKSVSFYIAITLEVFILCFAGEFLSAKSKSISDAVYNSLWYNMPPS

DSRILLFVILRSQKRLTITAGKVVDLTLEGFTSIMKASASYVSVLNAM

>CfOr183

LRFIGMWPHCIYANINWWTYIASVAVVQYFQYSYIHFDISDLSIIIDGLSITLGYSLSFLKLINLWFNRRKLHVILDTMD

KDWNDGIAIHSDVSTMIRHANLSRQCSNVMITTNALSVFFYTIGGPILRSVIHKNDQETITRELPIKMEFPFNVDNSPIF

ELVLVVQLFHDLSVACIIAMLNALLVTLVLHISGQIDIMRQGLLEIPSKNHAIKVLISRHQRIINLSDNIEDLFSNIALL

QFIWNTLVICCIGFLIIISEEGATMITKSLIFYVAITLEAFVFCYAGEYLSAKSKSISDAAYECLWYDLTPSECRILMFL

MLRSQKRLTITAGKMTDLSLEGFTTIMKSSVSYISVLRA

>CfOr184

QIGLRVVGIWPNAPYTLLFRCGWIFTTGIIQTCQYWWIIIHFGTEDLSHLLDGLSVTMEYTVMLLKLIILWLNSRIFYDA

LAAMVSDWKEATINDMHTMTSKANLSRRFSNVIIGLHSVGAFTYGIGVLVSHSDDHDADAIEPVRMQREFTLKMQLPFEC

NESPLYELVMSLEFLHQLASSAVTGILNSLIITLILHASGQIDILCDSLKKISPEKNNRNNRKLAVSITKKLIGKHQKII

IFSNKIEKIFCYIALIQFMSSTLVTCCLGYMIVTSTIDGSALMKAIVFYMAVTVEAFIFCFCGEYLSAKSKMIGDAAYKS

IWYNLKPNDSKLILLIMLRSQRRITITVGKVMDLSLEGFTSIVKASVSYVSVLHAM

>CfOr185

PVEIGLRLTGIWPNSSIFFRLLWSIVMGTGLIFQYRYLLTHFSIEELPNLIDGLSTTLPYTLLFFKLIILWINNRIFNDI

LRTMSNDWYEYSSIYTMIDKANLSHRYSKLVIGVYSTAVLLYSIATIDFRKPINDDCRQLLIKMELPFVFCESPIYEFVA

CVQFVHLMVVASTIGMLDALIVTLMMHIGGQIDIMQQQIREICPKDNEYDRSLIKKHHKIIAFSDNIESLFSSIALMQFV

SNTMIICCIGFLVATSLGTNEMLIKTAFFYIAIVLEAFIFCFSGEYLSNKSRTIGDATYESLWYALKPRDSRILLFVIMR

SQTRLTITAGKFMDLSLEGFTSSLKASASYISVLYAM

>CfOr186

VEFGLRAIGIWPGSPRSMLYRTCWTISLGLAQTFQYIVACIKTNNFLDLVDSISTTLPYSLLCLKLIILWLNQRLFNNIL

TSISRDWRNCDFIACNMRIMMNKAYLSHRCSMLIIGVYSMAVIVYSSVIIELNNIDTDVEEKELLLKMQFPFVYEFSPLR

EIVMFVQFIQLLSHASIIGMLDALIITLILHVSGQVDIVCRGLFELFSGKHEYKEYKSYKDATSAIIRRHQDIIAFSTDI

ENLFSYIALLQFLTNTIVICCIAFAIVTCDQGYALLLKSLFFYIAITLEAFIFCFAGEYLSNKSKSIANTAYEVLWYDAK

PNESRILLILMLRSQKRLTLTIGKFNDLSLEIFMSILKASASYVSVLLAM

>CfOr187

EDLFIHLERIFSIGGIWPFKQTYIRFAIYISYYMLYLIMAYIDLYDVFGNLELMVMNLVETIAYTMTFMVVWLIRCSNLL

KHVINAVRKSIMEQKFENSEERIYYHYNYTSKIFTYGSIAGMLITITLLYFRPLLRFSTSNQVLHNDTQFFMLPYRVHIF

FDTTNNYTYILMYLYLFPMFYVSICHMAAICLLVILVFHICGELSILSYRIKNVRTYSQYSQDTIVDRIRSFVRMHLKII

WMAKSVDNTFNLILLDELLGNSVVLAISMYYVTINISEMATCFTFILFAIIALVMLFGYCLIGDQLTQQ

>CfOr188

SQKTARNRLDQFRELSALHITYLKYIGLWAVDSDSSALLKCLYFAYNKFILTIMLVFMLADICSSFDDLSIVTDDGCIFA

GIVVVFFKVMIFQTRRAQIVRLLRETIDGCDQLCKGDEGEILGKYLLLSRVTFYGFSTLAVFLVIALLFLVPVEDGELPV

RALYPFDTTKYPWHAVGFFVEACTVSIGMTAIIGMDSLHTNLCNLFLVHLGILNEHFKSCSSFKISSSTVEERNREKDSD

EIRRGSSNVFVERFRRSIRNHQRLLAIIDDFNKVFSAGMFVQMLSSTSMICLTGFQAALVVGQRSNICKFSIYLAAAVSQ

LFYICWIGNEVIYQSALLTQSQWLSGWSDLSSKTGRLINLSMIFTKKTLNLKAGVFYVLSMETFIAILKGSYSFFTLLTT

M

>CfOr189

IRMARILMKTIGFWPAETKKGKLLLKGTLVYTLFAFALALWIEATELYLTTGDFYALTYTACTATPVVIILLKLSFFLYH

REELLRMLKYTEDNFWYVQYDAYGSKLLEKIDRKGKILLFTFTFFTQGAVFTYMLAPLVENRGKNESERILICNIWVGIP

TNVSPNFEIIFFFEVVAFIHSGLCFCCFDNLLGLINMHTAGQFNILQHRIETILQKVKKGKVYEVYEEIIECITIHHKLI

WYSEAMERLFMYTTLFQLLVSSVLLCVSGLQIFLGQGTIVRRMIFIAHAIACFFQLFVVTATSNDLIEESRAIGDAAYNA

NWHNDNKGVRNAILMIMKRSMRPCSISAGGFFPVSLETFMTVLSTAVSYFTLLRNF

>CfOr191

TYSRRVRTMLYLGGVLQDRTHSVIRTYIIGFLVILMCLSHSIFLLNFSRDYADNLTLMVKCFGQMSSFIAPALMSACFLI

KREKLLELHETLNDLFERELEQDQETTLAILHAFDRQSYIMFFITTSLILSHVCPPLIFIIYQSVRHIEPKGYRLPFLAK

FPWPINGGFLFYLHFLYHFFIGWWVIFTINSVDSLFGFYAFQISSILHAMSIKLRREVLKICIQIHGRLLQCSYILEDIW

QLIILRMLVTNACVICALIFEASQFTDITMEVFSFIFFIALKLLQTFIYAWNGSSITSASEYFREGIYFSDWPNLDHHIR

ANIIVSMMQKPMIIKTLKISSVNVNMFINIMNTAMSYFFLLQSL

>CfOr192

LLPMTDDNSFSIFCRIYRAFVWSIEVIHAISLIIGMTVVPKEKALKDGIISIVVIMETSFLLASIYTQQKLTIQVVRKMN

EILRNADMIMVDLVKTALKPIILPFIIYGVASVISVTIWTIHPIILIFEKRDTFYFEDYPAFFSPEPFSVHILVIMTTGA

VYLFLRKYSLDVYMMHLVLMLTAQYRYIAIKLTLLFQKPQDDNKNSQKKCHPMTDNKNSQKKCHPMTDQWAEKELRALCQ

HQNNVLFISIMLRKLLSVNFSLLYLNSVFRFCFLGILMTTIPSLTFEGISIVLFAAGSIMQFFLLCSSVQTLSD

>CfOr195

NPLNVRLNMISGNLLPMTNLSFPVLWRIYSFLVWSLAIVQTCITIPGCMYVPKEKALKDSLIAIVVTIEVVFLVVQIHAR

RELMQRLIQKLNNLLRIEDKMMESVVMETLKPMMTPLRFYWMAGIVSIIIWSGVPFTLIFRRDTFFYVDFRMPIAYTKEP

FSTSIFVIGSFSIMISSMYTFTRKVSVDSYMINLILLITAQYKYIALKLSMIFDDDISQNHDSSNKRKHYSKNYYTTNHD

SSNKRKHYSKNYEKQMKSLCQHHNAVIRVTLILRKLLSLNFSLIYVISILRFCGIAIMMISISTTLLEGSLIVMYASGGI

VQLYIICSCVQQLLDASIEITDQAFHEQWYRFGASIKRTFMFMVMANNLELKLSTFEKYNLSLSSFMTILNQSYSVAIIL

FK

>CfOr197

LNVRINIISGLLPLSKDSRFPVMWKMYSIFVWLLEIIQAIVLIPGIIFVPREKALKDGTVTCVVTIEVFFMVMRIQAHRK

LMNQLIRRLNDILCVADETMKNIVTTTLKPMGDPLKFYLLVGWLSVFIWCCLPFLLISEKVAFLYEDYRTPAIFSKQPFS

IDVFLVGSVLLLLSNVYIFLKKVGVDIYMIHLVLLITAQYRYISTKLAIIFRDGNPQSEKDQWVRKEMEALCRHHSSVIH

LSSMLKKLLSLNFSMIYVNSVLRFCFIGIMLSTISSTNFEGSSIVMFASGSIVQFYMLCFCVQQLMDASRKVTDEAFHEK

WYQFGPSVKRTFMLIILGNNLGCKLSMCDKFNLSLPSFMTILNQSYSIALLFLRVK

>CfOr198

LNVRMNIISGLFPMTTDSRFSIGCKIYSIIVWLITISVITLFFLGFTMVSKEKVIADGMISIVFIIEVFFMFIRIHTCKD

LAMQLIQKINDILRAQDETMKCIMMTTLKIAHSSSKFYWINMMINITMWISLPLIVIKKDSFYYEDYRLPFISKEPFSFK

IFVLGTLFLVITSLYIIMKKAAAHFYMMHLVLLITVQYRYIAVKLQKVFQKENSQKNKINSRTKFCSEKRTKFCSEKIDL

WTEREMKSICRHYNIVINFFKLMLMILSNIILFQITVYIISFFVPIYRIYWQLITLFERITIIMFAFGEMLQFYILCSAI

QQLLDASTEITDMAFHEKWYQYGSSIKRTFLLMILSNNLKCKVATMEKFSLSLPSFMTSINQAYSIT

>CfOr199

LNVRLNLFSGLLPMTNNSPFPLLWKMYSMFVWIIELIIGATLIPGCMYVSTEKVLKDGMICFAVFIEMTFLIARIHIYKN

VAHQLIRRLNDILHVADETMMSVVTATLKPVEAPLNFYWSIGVISIIAWTCIPLVLVFKKNLFYYEDYRIPAFSKQPFSL

EIFLLGSVFLMVSAVYMFLQKVGVDVYMIHLVLMTTAQYRYIAMKIAMIFHANDEDNKEYSSELNQRQNDEDNKEYSSEL

NQRQEREFKVLCRHHNSVIHITSLLKELLSLNFSLIYMNSVFRFCFIGIMLSMIPSTTFEGISIIMYASGAVVQLFILCS

CVQQLLDAA

>CfOr203

FQQVFYFLRVLGTIAXTWSPQPRKIKLFLRNFYYCIAIFIYVTTWVSIVINAYKTRNDVDEFMMNLSHTLSLLDATLNSI

LCTIKRKQLQNLVIHIKEFMNISKDREIVILQKYINRHTTFISIVAISFTMAGVTIICAPLFTPKEFPLDTWYPFSVEPL

WLKFICYVTHIXTHTVFCLNVDIIATFFLYSAARLEMLAFEIEQATDEKINRFISKTQETLQHVLFKANFTMAFAVISGG

FPILYLESYVQLPQFISTAIAALQRXYITASAADDLREVSTQLPWSVYGASWIGKTQKMKNDIFIMLQKSQQPCLISLGS

LLPLTLKYYLFIVTTILSYFTTMKA

>CfOr204

FQQIFHLLRVFGTITNTWPPHPGKNKLLLRNFYYCISIFIFATVWIAMLMNAYKTRNDVGELMKNISHMTSIMEAILNSI

LCTVKRKQLQNLVIHIKEFTEILKDCDKIILQKYINRYIAFISTVAISFSMAGITVICAPLFMPLEFPLDVWYPFSVKSL

LLKFILYIMQIFTIAHTVFCLNVDIMIAVFFLYSSARLEMLAFKIKRATDNDHVHVISCIKQHQEIIEFISKTQQTLQHI

LFKTNFTMAFTVISGSFPMLFLQSGVLIPQFISMALGALQRMFITAWAADDLREISTRLSWSIYSASWIGKTRKMKNDIF

IMLQKSRRPCLIFMNGLLPLTLEYYANFVTTVLSYFMTMR

>CfOr205

ILFMKMSVALSCTWPPSPTKSNTILFKACWYICYFCSILLLLPLLSSVYEYRDDPVILAKSVCLSCAVLQVTIKMIVCRI

QYTSFQMLYHDMETFCKQADDKTNITLQRYIDNYKCTYGSYILWCYLTAIGVICGPLFLPQQFPTDAKYPFSVEHHPVKS

IIYLHQSLVGLQASAGMCIDCSVAILLFYSAARLEMLVQEIRNAKSELDACIKLHGEILKYISKMISVVRPLVLTTITMT

TMGVVFGSLNIITEQPTIVKIQYSIVVFSAGVELFMCAFPADNLMHMNSRICLGAYESKWFQRSVSMQRKIVQIIFRSQK

PEVIRINGILPLSLRYYARFLYTSYSYFTAVR

>CfOr208

KLTLENVITFTKLSLVISLSWPLPATKRQVVRFRLLRFLTYMNIWCLFVPLSMTLQDWSDHSDICIKSIPLIAGSAQAFI

EMWICHGQHKHLQLLIAEMESYCKHAIGYEKDILQQYVDRYAMFYATAAMWFYLTAFAVVCSPPFSSDTFPTYAKYPFNV

NYQPLKTVIYVQQSTVGIQMASVLCINILIALLLWFASARFDMLCNELRMISNELTQCIRKHQQVYANDVTHSVRFLVLT

IVGCSAAAILFVGLTLVSLQQPLIIKMQFCVLIFTCLSQVFMCCWPANNLLVASSNIALAAYESLWYSRNVYMQKNLLLI

LLRCQKPVAVTVTCIIPLSLRYFGSYISTAFSYFTTLRVM

>CfOr209

NATLKNVIATVKLSLFITCCWPLPANTIKVICVKLYQYLCMILTLGITVGLCNTIRNHLDDPLIMAQSIMIMCSTIHVIF

NIVSCKINSYRLQLVTYEMENFCELIKSHEEAIIQQYIDRCIYFYGGSIVWVYLTTIVIITGPAALDQTFPTNAEYPFDI

YHQPIKFIIFIYQAFICMQCGSQICMNIFIALLLWFTSMRFKLLSEELRAIVDIHDLIQCIQKHQKLLKYAEEVVSVVRP

FAFLAISISTFALIIIGIILITQGQPLSMKIQCVGLIFGGLAEVFMYTWPAEHLIHISNEVGQTAFNTQWYKLSITLQKN

LQIMIQKSQKPIIVAIPCVMPLSLNYYASYLSTIFSFFTTLR

>CfOr213

ITLEKVIAFLKVDLLFACCWPVSRTKFQIVCDRIFRVISSLHAILLMIELIYTIIYRTESIQMLMQSTCAVGILSEVPLQ

ILLFTLQHDRLQVVIFQIENYYHQAKTEERNVFQKYIDRYIYLYATTLGLITVGLFISFLDPLLRGFDTFPLVIKYPFPI

DRQLLRAIVYCHHMFGIYQIYCQVSSNVFLAFLLWFTSARFEIYSDWKRCISEHQELLRFAQEISLSISYIILLSLGIST

YSLVFGGVSILSRIPLSVKAKFFIVCVSSLLKVLLCAWPADYLMTISSDIGDAAYDSLWYKHGIDSQKMMLYILLRCQRP

IIITVPGLLALTFQHYTSYISTAFSFLTTFR

>CfOr220

LKFSLTVLAVAGCWRPTSWTKTIMYNTYSLSVILTLYTFAITQIMELILNADDADAFGDALFNVITSLLACYKAIVIQKS

HESIITLINNLAETPFKPLDLNENMIQEKFNKRITNNTLCYLVLILITNLYMILLSLFTDFKNGTLMYKAWIPFNYSMSA

LFYPVYIHQLITLMFSGLVHSTCDSIICGLLLHICCQIEILEYRLSNIMNARENLRDCVHHHIHIFESYIYTGSTKSKLL

IQKFLTNARENLRDCVHHHIHIFESYIYTGSTKSKLLIQKFLTFSRIIEKIRVTLIRHNSAKKIVQISFLICRDKSIFYC

NYNEICDKNSLQLSDNIYNTEWTILNNNVKKGLLMIMNRATIPIEFTSANIISMNLESFVMVLKTSYSLFNVLI

>CfOr229

LPMPFALFTYSGYWRPVHFPVNSLKYRMYNIYSAFMFLLLFVFYIVDTFILSASLPEFVNKCYLFLTILGVSCKVIHVFI

CRGKIIELDKMLLEDNCVPRNIEEILIREKFDRYISRLTIACEIFNESSAMFGTLAQFYILLKTRSLPVYNWAPFDLSSI

YVFLPMLIFQCVALMLWANTSVAHETLTSGMMIQICAQFEILCHRARILPTLLMEAEKNSKSDEDLITREKNSKSDEDLI

TREIRDLIYHHLYVYKFAHMVNATFTTMMFIQFSIISLVLCMSVYKLSTITSLFTNFAHKFSYLCSMLVQIFLYCWFGNE

VILKSIDVSTAIYEMDFTKLRVRVMKDLMIIMMRASKPVKISTGYIVTLSTESFMSILKISYSTYNFLK

>CfOr232

LPVSFALFTYTGYWRPGHFPVNSLKYWMYNIYSTFMFILLQLFVFYGIFFISASLEEFVDKCYLFLSIFGVSCKIVHLFI

RRGKIIDLDKMLLKDNCIPRDVEEKLIKKKFDRHARQLTIGCEILNEFTAIFATFAQFYKSLKTRSLPVYNWAPFELSSI

YIFLPILIFQCVGLTLCANSSVAHETLISGMMIQICAQFEILCHRAHKLPALLMEAEKNKIHDLIYHHLYIYKFAHTVNA

AFTMMIFVQFSLISLVLCMSIYKLSTMTSLFTDFAHTFSYLCSMLMQLFLYCWYGNEVTLKSIDVSTAIYEMDWTTLRIR

VMKNLMIIMMRAGTPIKMSSGYIVILSTDSFMSILKISYSTYNFLK

>CfOr235

LSFNFFLYTISGMWRPIESKCSKMLYSMLTCFTMYLLIIFTLTQLLDIILVIENIDDFATTSLLLLSTVSVLFKATAVIT

HRDEIANLIDTLQKKPCKVYTKEESNIQMKFDCTIRSYSIKYTSLTLLSVTGGIIRGLLQILEGQLPFRMWVPYDYTSPF

LLWFTSIQMIVAVIFATFVNLATETTVLGFCLQICAQIEILKHRLQRMMKSSEKKTTSRISLNDESNEIGKLSEYILHHL

CIIRFVKQIIKLFXMYLCIQFFVSILVLCISLYYLYSHVIIIDIISLIIYVLCMF

>CfOr240

DFTFKILMICGCWIPDSWTTPYKRLVYHVYTIFIMLLIHTFMLMDLILTVDNAEDFTDNFYMLLAMIVSCCKMFTLLMNR

SNITMLIDILTRKPCRPDYSDEIEIQQKFDRLIQTNTLYYAILVETTCICIAVTSLLTEFSKGRLTFRAWLPFNYTSPLL

FRIVYIHQLIGLTAGSVLHVGCDGLICGLLVHVCCQIEIMECRLRKIGSNRDNLRKSRDNLRKSVLQHNTVFKLARLINE

KFRLTIVIQFIVSTLVMCFNLYQFTKSTASRAKYMQLIMYMGCMLSQIFLYCWYGNEVKLKSRQLVNNIFEMEWFELDNY

TKQSLLMIMTRGTIPIELTSAYVISMNLDSFVH

>CfOr241

LDFTFKILTICGCGRPDSWTTPYKRLVYYVYTIFIIHTFMLSQLMDLIMTVDNSDDFTDNFYVLLAMIVSCCKMFALLIN

RNNIDMLIDTLMRKPLQPIESDEMEIRQRYERLIQSNTFYYVILVETTCLCVTITSILTEFKKGKLTFRAWLPFDYSSPL

LFLFVYAHQLISFTVGSVHHVACDSLICGLLVHICSQLEILECRIRKILQDCALQHNHIFKFAHMVNDKFRITIFIQFIV

STLVVCFNLYQFTKSTALRAKYMQLILYMCSMLSQIFFYCWCGNEVKLRSRQLVNNVFEMEWFELNENAKKILLLIMRRG

IVPIEFTSASVISMNLDSFVGLLKTSYSAYNILQQT

>CfOr242

LDSTFKFLIICGCWRPDSWTSSCKRVIYHMHTVFILVLINTFTLLDIILTVDNPDDFTDNFYMLLAMIVSCCKMLSMLMN

RKNIAILTNILTEKPCKPLESNEMEIYHKFDKGVQANTIHYAILVETTCVCITLTSLLTDFRKRMLTFRAWLPYDYSLPF

LYYITYAHQLISLIMGSVLHVACDGLICGLLVHICCQIKILESRLRRIARELHDCILQHNRIFDLAFAVNEKFRFTITIQ

FVVSTLVVCFNLYQLTRTTTTKAKYIQLALYMCSMLTQIFFYCWYGNEVK

>CfOr243

ILELTFKVLTICGCWQPESWTKRIIYRVYTFLVILLINSFVLSQFMDIILIVDNTDDFCDNFCVLLPMIIVCYKLFSLLG

SHKNIIKLTDILMKKPCKPLKPNEIKIYYKFDKGMQINTFHYTAMCMVTCVIITITSLLTNFGQKKLTYRAWVPFEYSSM

TLFCILYIHQLIGLTMGALVNVACDSLICGLLVHVCCQYEILTYRLKRIMDGLRNCVQQHCKIFRLAFLVNTNFRMVITI

QFLMSMLVVCFNLYVISLSKLDARCIRLALFMGCMLTQIFVYCWFGNEVRLKSRQFIDNIFEIEWLTLDKNLKKSLIIMM

KRTVMPIEITSAYTISLNLDSFMNVLKTSYSIYNLLQQMK

>CfOr244

NVLEFPFKVLTICGCWQPQSWTSIYKRIMYRMYTILIILIVNSFTFMDIILIVDNTDDFCDNFCILLPMIITCFKLFSLL

ANRKNIIKLIDILTKEPCKPLKLNEIEIYYKFDNSIQLNTFHYTAMCLITWVSITFTSLMTNFGERKLTFRAWVPFEYST

MMLFCLLYIHQLIGLIVGALLNAACDGLICGMMVHICCQFEILTYRLSRIMSDGLRNCVQQHCSILRFAFLVNAKFRIII

TIQFLMSMFEICFNLYQITLSKLDARCIRLALFMSCMLVEVFVYCWYGNEIKLKCLQFVDKIFEMEWLTLDNKLKRSLII

MMARTIIPIEITSAYTISMDLDSFVGVLKTSYSAYNLLQQM

>CfOr245

LEFTFKLLMIFGCWRPNSWTSIYKRIVYYVYSSIIILLLNTFMLMDIILIVDNTDNLSDNLFLFAPIVTTCCKLFILLLN

RKNIIMLINILIEKPCRPLTSNEMKILSKFDKSIQSNTRRYTYLVTITSIFIVLTSLSANFKKRQLTYRAWLPFNYSSMT

SFFLIYIHQLICLIVSGYLNVACDTLICGLLVHICCQIEILTYRLKKIMFYILRDCIRQHYYIFRLAFVINVKFRLTLTI

QFIISMLVICFSLYQLSNRTAKAKYIEMVLYMICMLTQIFFYCWYGNEVKLKSHQMIDDIFDIEWLTLDKNITKSLIIIM

KRAVLPIQITTAYIIPMNLDSFMGLLKMSYSTY

>CfOr246

VLQFTFKILSIVGCWRPESCSSLCMRIYTVFMVILLYTFLISQFLDIIWNVHNTEDFTENFYATLASVVSCSKMLSLLIN

RNNINTLTNILIEKPYRPSDIDEMKIRHKFDRLIYTNTFFYVILVETTCACITMTSLLTEFRKRNLTYRAWLPYNYNSSI

VFCLTYAHQLISLTAGSLVNVACDSLICGLLVHICCQIEILEYRLSKISNGYNNYNNLHDCICHHDGIFNYALKLNKKFR

MTIAMQFIVSTMVVCSNLYQMTKSSTVDASYLPLLLYMSCMLTQIFIYCWYGNEVKLKSIQVVENIFGMNWLTLDKNFKQ

SLLIIMNRASIPIEFTSAYILSMNLDSFVGLLKTSYSVYNLLKQV

>CfOr247

VLQFTFKILTIIGCWQPESWSSCMRTVYDVYTVSIMHTFLITQLLDIIWNVDDAEDFTENFYATLATFVSCSKMFSLLMN

RNNIDTLTNILVEKPYRPLDIEEMIIWHKFDRLVYINTLCYTILIVTTSICFTLTSLLTEFRKRNLTYRAWLPYNYNSSI

VFCLTYAHQLISLIAGSLVNVACDSLICGLLVHICCQIEILEYRLSKISNGYNNYNNLHDCICHHDGIFKYALRLNKKFK

MTIAMQFIVSTMVVCSNLYQMTKSSTVDASYLPLLLYMSCMLTQIFIYCWYGNEVKLKSIQVVDNIFGINWLTLDKNFKQ

SLLIIINRASIPIEFTSAYILSMNLDSFVGLLKTSYSVYNLLKQV

>CfOr248

VLQFTFKILTIIGCWQPESWSSCMRTVYDVYTVSIMHTFLITQLLDIIWNVDDAEDFTENFYATLATFVSCSKMLSLLLN

RNNIDTLTNILVEKPYRPLDIDEMIIWNKFDKLVYINTLCYTILIVTTSICFTLTSLLTEFRKRNLTYRAWLPYDYNSSI

VFCLTYAHQVISLTAGSFITVACDSLICGLLVHICCQIEILECRLNKISNDHNNLNDHNNLHDCIFHHDSIFKYALRLNK

KFKMTIAMQFIVSTMVVCSNLYQMTKSSTVDASYLPLLLYMSCMLTQIFIYCWYGNEVKLKSIQVMDNIFEMNW

>CfOr249

LSLNFLIYTVVGIWRPIDSNVAKLLYNAFTFIVLVLEYFLMLTQFMDIVFVVDNIDDFITNTVMFLSIVGVCFKATIVII

RRNSIISLVHILLKPPYKPRNEDEMAIQMKYDKFIKSCSIMYVLLVISSVTGVTAGSVLNIMQGHLPYRVWLLYDIKVYL

IFWITSIQQILSVILDAIITVGTETLVFGLFLQTCGQFEIFENRLHKLTISKYIHHHLCIYKFAKTINIIFNQIFFVQFF

GSILLICTSVYYVSMHMMESRSATMIIYTCSMFVQIYFFCWSGNEVILKSMNVGDAIYHTEWPLLSISDKKELLMIMMRS

TIPIKFTSSFLITLSLQSFSNILKTSYSAFNILQ

>CfOr250

LSFNFFLYTISGMWRPIESKCSKMLYSMLTCFTMYFLIIFTLTQLLDIILVIENVDDFAMNTSMLLSFMNVLFKAITVIT

HRDKIVNLIEILQKEPCKVYSEKEINIQMKFDCMIRSYSIKYASMCLLSVIGGIIKGLHQILKGHLPIRMWVPYDYTSPF

LLWFTSIQTFVALIFATIVTTATETTILGFCLQICAQIEILKHRLQRMMKSSEKKTSRISLNDESNEIGKLSEYILHHLC

IIRLAKMINKVFSQVIFFQFFASILVLCTSLYHLSSHMTITYIISMIMYVLCMFVQIFVYCWAGNEVILKSTGLSDAVYE

MDWISMPINKQKDLLIIMKRSTRPIIFTSSFLVTLSLESYVNLLKASYAAFNFLRQF

>CfOr251

LSFNFFLYTISGMWRPIESKCSKMLYSMLTCFTMYLLIIFTLTQLLDIILVIDNMDDFAKNSLMFLSIISVLFKATAVIT

RRDEIVNLIETLQKKPCTVYNEEESDIQIKFDCLIRSYSIRYTSLASFSATGAVLGGMFNILEGELPYRMWVPYDYTSPL

LFWFTSIQEVVAVIFGTIVNVATETTVLGFCLQICAQIEILKHRLQTMMKSNKKNKKKEIPISLNNTSNGRSILSEHILH

HLCIIRLAKIINKVFSQVIFVQFFASILVLCTSLYHLSSHMTITDIISLIIYVLCMFVQIFVYCWAGNEVILKSTGLSEA

VYEMDWVLMPISEQKDLLMIMRCSTRPIKFTSSFLVTLSLESYGNLLKTSYSAFNLLQQ

>CfOr252

FSLNFLMYTVGGVWRPIKSNVAKLLYSIFTSIILVLLYFLMLTQFMDILLVVDNIDDFATNTLMFLTIVAVTCKATIVVV

RRNAIIKLVQTLLEPPCKPRNEDEMAIQKKFNKFIRSCSIKYSLLATSSVTGVTVRSVLNVTQGYLPYRIWVPYDTDTSP

MFLITSIQQIITVVFVTIINVGTETLVFGLFLQTCAQFEIFENRLHKLISNNRLHKLISNKIAKYLNPASSNKGRAIISE

YIYHHLSIYKYAKTVNVIFNQVLFVQFFGSILVLCTSVYYLSAHITESESATLIVYTICMFVQIFVYCWSGNEVILKSIS

VGDTIYHMDWPLLSISEKKELLMIMMRSTLPIKFTSSFLITLSLQSYSGILKISYSAFNVLQ

>CfOr253

LSLNFLIYTVVGIWRPIDSNVAKLLYNTFTFIVLVLEYFLMLTQFMDIVLVVDNIDDFATNTLMFLSIVGVCCKATIVVV

RRNSIINLVQILLKAPYKPRNEDEVAIQMKYDKFIKSCSIVYLLLVISSASGVTAGSVLDIMQGHLPYRVWVPYDTNVSP

IFWIMSIQQILSVFFAAIISVGTESLIFGLFLQTCAQFEIFECRLHKLMNNKISKYIHHHLCIYKFAKTVNIIFNQIFFI

QFFGSILLICTSVYYVSTHIMESRSATMXVYTCSMFVQIYFFCWSGNEVILKSINVGDAIYHTEWPLLPISDKKELLMIM

MRSTIPVKFTSSFLITLSLQSFSNILRTSYSAFNILQ

>CfOr254

LSLNFLIYTIGGVWQPIESNSAKLLYNAFTFIVLVLEYFLTLTQFMDIVLVIDNIDDFVANSVTFVSMIGVCCKATTIVI

RRSAIVNLIQVLCSKPCKPQNKDEETIQTKFNEFIRSWSIKYSLLVVSSFTGVIIGSVLNIMQGHLPCRIWLPYDINASP

LFWIIFVQQILSVFFASVINVGTETLIFGLLLQICAQFEIFESRLRKLVARKTIISQYIHHHLSIYKYAKMVNIIFNQVL

FIQFCGSILVLCTCVYYLSAHITEFESATLIIYTICMFIQIYIYCWSGNEVILKSKSVGDAIYHMDWPLLSVNEKKDLLI

IMIRSTIPIKFTSSFLITLSLQSYSNILKTSYSVFNL

>CfOr255

LSLNFFMNTIAGIWRPIDSNTAKLLYNAFTFIVLILEYFLMLTQFMDIVFVVDNIDDFATNTLMFLTIVAICCKATVIII

RRNSIIRLVQKLLKAPYKPQDEDEMAVQTKCDKFIKSWSIKYIVLVIGSVTGITIGSVLPYRVWLPYDTNASLIFWITSI

QQIVSTIFGAIINVGTETLIFGLSLQTCAQFEIYEIRLRKLVTSSSKIRKVAISEYIHHHLCIYRFAKTINIIFNQILFI

QFCGSILILCTSVYYVSTHMMESESATLIVYTFGMFVQIYVICWSGNEVMLKSMSIGDAIYHMDWPLLSTSEKKELLIIM

MRSTIPIKFTSSFLITLSLQSFSSLLRTSYSAFNILQ

>CfOr256

LSLNFFMYTIAGIWRPIDSNVAKLLYNTFTFIVLILEYFQMFTQFMDIILVVDNIDDFATNTLMFLTIVAVCCKATVIVV

RRNSIISLVQILLSTPHKPRNEDEMAIQTKYDKFIKSCSIKYSILVASSLTGLTIGSVLNIMQGXLPYRVWLPYDTNASS

IFWIISIQQIVSVYFGAIISVASDSLIFGFILQICAQFDIFENRLHKLMTNKISNRLHKLMTNKISNYLNQLPTSNIEKA

ISEYVHHHLCIYKFAKTTNIIFNQIFFIQFFGSILLICTNIYYVSTHMMEGSATVIIYTFGMFVQIYFFCWSGNEVILKS

MNVGDAIYHMDWLLLSPREKKELLIIMMRSTIPIKFTSSFLITLSLQSFSSILKTSYSAFNVLQ

>CfOr257

RLSLNFLMYTISGIWRPIESNCAKLLYNVFTTFIIVAVYFLMLTQFMDIVFIVDNIDDFATNSLMFMTIVSVCCKATIAV

IRRNAIIDLVDMLLKDPCKPQNEAEGAIQMKFEEFIRSCSIKYSYLSTSSVTFFTIRSIINITQGRLPFRVWLPYDCNKP

LMFWITFVHQFITSIFATIISVGTDTLICGLFLQTCVQFEIFECRLQLAINTKAGKTMSKYIKHHLSIYNYAKTLNSIFN

QVLFCQFFGSILLLCTSVYYISTHITGSEVATMSMYTVCMFAQIFVYCWSGNEVILKSNSIGNAVYNMNWFLLSINERKE

LLMIMKRSTIPIKFTSSFLITFSLQSYSNVLKTSYSAFNVLQQ

>CfOr258

LPVNFKALQFCGAWKEREDDNVAFLRFCYRYAVVFLIYEFTILDAVEVIRTRDHIHELTEGLFLGLTFLTLSVKYANFLL

RENKVSELLDYLRIKMCQPKNSTEQLIMEEHNRKAKWSTVSFMMISFATGLGFMITPALGLLTKNEHVLPLKSYIPYSVS

NLFIYLATYLQQFITLFYGIMLNVSFDSLIYGFIIHTCAQIELMCHRLTENLNCISSERKSQTNNLNCISSERKSQTNVS

IEECVRHHLLVKILVKKMQELFIWSVMVFFFFSLVIVCTSIFLISKTRLLSFEFLSMLLYLSGMLLQLFYYCWYGNELEL

KSKGIATAIYSSDWTKVTPQDRKSLIFIMINSQKGIMFSYHGIFALSLNTFTWICRTSYSAYNLLQQ

>CfOr259

LPVNFKVLWFCGAWKEQEDDNLSCLHFCYKYAIFFLIYEFTIFEVIELIRMRDRINELTEGLFLASTYITLCLKYANFLL

RKNDVSEVLDCLRVKLCQPRNSTEKIIIETHNRKAKWSTLSFLIMSQATAVGLVMAPILGLGKGEWFLPTKSYVPYSISE

IFPYVATYLQQAAALFYAIMLNVSFDSLVYGFTIHACGQIELICSRLTDNNKASINFQKKSDADNNKASINFQKKSDATI

SIEECIRHHILVHTLVKKIGALFIWTVMILFFFSLIILCTSIFLISKTKLFSIQFLSLTLYFSSMMLQIFFYCWYGNELE

LKSKSIANSIYLSNWTLTTLHERKSLILIMINSQKGLTFSNNKIFALSLDTFTWIFKTSYSAFNILQQV

>CfOr260

LPINFFVFRFCGIWKEHENSNLFAIFCHRYMIAVLIYHLTIFEIIELIRIRNSMEAVMEXFFXXXFTFVSLLKYLNFSMR

QCELHALLSCFRVKICQPRDFAEKLILKQYDRKAKGIVCFYMLMCQTTGLMFMIMPLLIPDEKSLPFKTYIPYSITTLLP

YVLTYLQQSATLIYGILLNVSLDSLAYGFIIHTCGQIELLCYRLTEIFQFLQENNKKNAYAIEIAECAKHHILVYDIIYR

IESLFMWNVAALFFFSLINLCTNIYQMSKKELFGPEFFSFILYLGSMMFQIFTYCWYGNELDLKNKNISSAIYTSNWMTI

STKQRKNLLLMMMMSQKGRILSFYGICALILSSFTWIIKTSYSAFNLLQQ

>CfOr261

LPVNFKVLWFCGAWREQKNDNLGSLHFCYKYAIFFLIYEFTIFEVIELIRMRDRINELTEGLFLASTYITLCLKYANFLL

RKNDVSELLDYLRVKMCQPRNLTEKMIIETHSRKAKWSNLSFLIMCQATTVGFVIAPILGFGKDEWILPTKSYVPYSVSK

ILPYAATYLQQTAALFYAVMLNVSFDSLVYGFTIHACGQIELICCRLTNNIRASVNFGKNSDVTASIEECVRHHILVHTL

VKKIGELFIWTVMVLFFFSLIILCTSIFLISKTKLFSIEFLSLILYFSSIMLQIFFYCWYGNELELKSKNIGNSIYFSNW

TLTTSHERRSLILIMINSQRGLTFSNNRMFALSLDTFTWIFKTSYSAFNLLQQ

>CfOr262

LPVNFKVLWFCGAWKERKNDNFVCLHFCYKYAIFFLIYVFTIFEIIEVIRTRDQIDELTEGLFLASTHITLCLKYTNFLL

RKKDVSELLDCLRVKVCQPKNSTEKMIIKMHIRKAKWSTLSFLIMSYTTAMGFVIAPIILGLSKNEWILPTKSYVPYSTS

ETLPYVATYLQQIASLFYAIMLNVSFDSLVYGFTIHACGQIELICCRLTNNIRGSVNFQKDSDSVNFQKDSDSTASIEEC

VRHHILVHTLVKKVGALFIWTVMVLFFFSLIILCTSIFLISKTKLFSIEFLSLTLYFSSMMLQIFFYCWYGNELELKSKS

IANSIYFSNWTLTTSYERRSLILIMINSQKGLTFSNNKIFALSLDTFTWIFKTSYSAFNLLQQ

>CfOr263

LPLNFRVLWFCGAWREESNNGLFVRFISFCYRYSIVILIYEFTVIELIRTHDHIEDLTEGLFLALTYVALCIKYGNFLAR

QDEVYTLLDCFRGETCQPKNFEEKMILIKYDRKAKWCVRAFMSISQATCIALVLAPIVGPQDTDRPLPFKTYLPYSIVGL

YPYLATYLQHIGAIFYGVLLNVSFDSLVYGFTLHVCGQIELLCYRLSEIFKDYPDMAQYRLNSISQCVRHHLCVHEIVRR

IQSLFVWTVMLLFMFSMVTLCTSIFQMSKKKILSVGFLSLILYLGSMLFQVFFYCWYGNELQLKSKSIGDAIYSSNWTTA

TIQDRRSLLFMMSISQKGLKLSYYGIFSLALGTFTWILKTSYSAFNVLQQT

>CfOr264

LPVNFFVFRFCGIWKEHKDSNIRFAIFCYRYIIAILIYHLTIVEIIELIRIRNDVESVTESLFVVLTFMSLCLKYLNFSV

RQCELRALLDCFRTKICQPKDFAEKSILKQYNRKAKEISCVYMFMCQITGLLFLIMPLLIQDKRSLPFKTYIPYSTTTLL

PYVLTYLHQSIGLIYRILLNVSLDSIVYGFIIHTCGQIELLCHRLRQIFQFLQNNNEKNAINNNEKNAIAIESAIAECVR

HHILVYNIMYKIQSLFMWNVAALFFFSLINLCTNIYQMSKKKVVSLEFFYFNMYLGSLMFQIFTFCWYGNELDLKNKNIS

SAIYTSNWMTISTKQRKNLLLMMMMSQKGRILSFYGICALILSSFTWIIKTSYSAFNLLQQ

>CfOr265

MLKYYSRNQYFLSQMGIWPYQPRMIKILLPCFLVGAEMSVLATQILLLYNTWGDLSMTIEGIITSILLVGATTKLVNVVT

NNKKLQYLLQVMNEHWRLFHSECELHILRYYATIGQKVTKYYSVYINIFVVLFMLIPLMPKVLDVIIPLNESRPVIYVLE

GDWGVDKDKYYFLILLHCYLAAVISTRCMVNVDTMYMMCVLHGCSLFNAIGMSLENILCKTKLVQDMNIEQAVIKEYNSQ

DYCEMIACLRKHQLAIKYTQMLDSTFKSATFLILVLNIMILSLIGLQLINKFGQTQEVIRFGCIAMGSVTHLLSMCLPGQ

LLLDKSIEVFDKAYNAQWYMFSLKTTKLLSVLLYRSVVPCTLSAANMYIMSMTTFSSVMQTAMSYFTTFLSV

>CfOr266

EMEEYYFINKFFLSRIGAWPYQHKVLKVLLPCFLTIVQYSVIATEIILLHDTWGDVDIAVESVIIIIPIVGASTKLINIV

VNNDKFRHLLRIMNEHWSIFNSESERYILKYYAKIGRKVTKYYAVYCCTILVLYLFIPLSPRILDMVIPLNESRPLKYIY

QAEYRVDKDKYYYPILFHAYMSSVITVGIILSIDTMYVICVLHACSLFTAISHRLENIVGQTDVKTDDNENEDYRELMIC

LRKHQLALEHVRILDSTFTHAMFILLSLNVLIMSVIGLQLINKLGHTEEVIRYICVTVGAFTHLVCMCFPGQLLIDRSAE

IFEKAYCSQWYTFSIKSRRLLKILLYRSLVPCTLTAGKMFVMSMTMCSSVMQTAMSYFTTLGSLK

>CfOr267

QSDILRYYHVNKIFMSQIGVWPYQNRAIRILIPTVLTFIDISYVAAEMIRMFDTWGDVDIAVECIISTIIVFACFTKLFN

LSFRINEMRYLFSLIEYHWQVFNNSTDVEILQNYVVFGRKVVIFYSIYVYVSMILYLLMPMSPQILDIMMPLNESRPRKF

LFEVEYRIDREKYYYLILFHSYVAVIGVMSIVVCADTTYIAYVQHGCSLFAAIGYRLEHIVSREYELSQVNQQTVYRELV

ICLRKHQLAIQYARLLESSFMLSTGIQLSCNMLALSLIGIQVISNLDSTEDLIRYLSLCAGAFFHLLWMSLPGQRLMDHS

MKIFDKACRSHWYTFSAESKRLFRILLYRSNVACTLTAGKIYVMSMENYSMVVQTAMSYFMTFSS

>CfOr269

IWQSRYYAIPRFYMTLAGLWPYHSIRNRYLHFVPIFTICFFLLVXKLICVLYIFLYIFIVRKHLKCFLISNFYNIHIYNV

FFYFEIIKSCLKTIENDWLSLNTDNEKAILQRQTAYGRYLTIFYAIFMQLTGFLYILKSVVLIMIDDTSNSTKLAVTKLP

FRVEYGHKIDQYFYLILTHNYLTVFSHVTATVATDTFYFILIQHACGMFSVVGHSLERIGKDSNNSFDSKPDKINDVNYY

KVNYYKVLDCLRKHLHVIEFAELIESTFADILLISISLNMIGGSICGIQVLINLNDAKDIIAPLAIYVAQLTHMFLQFWQ

AQFLLDYSVLPYESICKANWYYTSERCRKLLLLIMNRTILPCRITAGRVVILSIESFGVVLKTSMSYFTMLRSF

>CfOr270

ERRDVWQNRCYIIPRVYMSLIGIWPYHAFRDRCLLFVPMFTFSLTILMPQLLYLLITATNLRDVFSCTPSMWITIIFSFK

LGSLMINNKKLKTCLETIEDDWSSLSSDMERAVLRRHSAYGQYITMTYGVFMQFVGVLLILKSLLVILIEDTSDATITSL

AAESKLPFRVEYGRYLYPMTIHCYLAVFAHISITIAVDTFYIALVWHACGMFAIVGNTLEYIGKDSDNNYSKALECLRKH

LHVIQFAELIESTFSNIFLVSVCLNMIGGGMIGFQVILNLNDAKDIVGPLAIYIGQLIHLFLQFWSAQLLLDYSIVPYQS

ICRSNWYYTSKRCRKLLFLIMNRSVLPCRITAGKIVALSIESFGTVLKTSMSYFTMMRSF

>CfOr272

SRYYMVPRIHMTLVGIWPYNTIGIRYLRFVTMLTFSIGIVVPQILYLLNDSTDIDDVFECVPSITIALMFSLKLVNVMIH

SEKIKVCFKTIEEDWLSLKTDVEKAILRQYTEYGHRMSLSYALIILVPALFHLLKPVITTLMENDIENITKSSISRASKF

PFRVEYGEKLDQYFYVIMVHCCLAVFAHLVATVAVDSFYYTVIQHACGMFSIIGHMLENIGKNDDANLDANLEENNIKDN

NYGIALECLRRHLHVLEFAGLIESIFTNMFLFTISCNMVLGSFSGIQVIMNLNGAGNIATPLSVYFAQLTILFLQFWQGQ

FLLNSNDVPYESICRARWYCTSRKCKKILILIMSRTMLPCKITAGKVVTLSIESFGAVLKTSMSYLMVLRSF

>CfOr273

RQYKYNRILLSLLGQWPFQKKRDRWIVFLAVSFIGITQFISQILALVTLRGDLDATLECVAPLLVNCLCVVKLTNLVYNM

KGIKILLMHIQGDWQSWNIDSEFKILYRFAESGRSITIRLCGGMYAFGSLFPFLAIIPKIIGKNVTSNYSTRPVGFPYHV

EYFVDLDKYYYPVLIHNYLATAIRLTIIVASDTCVIILVQHCCALFSIIRYRMEHIRKAIKELEEDDKVYKNFIHCIRKH

EDAIQFANCLEAIYKQALFVEVGLIVSMMSLSALQATNDTLTPQVAVRHGGYIMAQLIHLFLYCWLGQQIIDHSDHVYTA

IYRGEWYESSSKSKKLLNMIMLRSISPCTLTVGKIMVLSLPSFSAVVRASASYFTVLQSV

>CfOr274

NRYYYINKTLLSLIGQWPFQSRLESNIILVIMLFFSCSLTVLELWGLIAGINNLSIIMENASPLLINSIIFMKLINCLCN

KNKMKNLLEHIEETWKRTQVGPQNKILQNYAEQNRTLIIRYACYKFSELLSSTYATYFLFLLGNVVIASSFSAAELIIAD

LQLDEIIRIVSCNIGLLLHIFYLSLMSQRMTDHSSRFREVIYSCNWYKISQRSKKLLRFTLLRASKPCQIKAGKMFVMSM

ENFSSIIRACMSYVAVLTSL

>CfOr275

NHYYYINKMLLSLIGQWPFQSRMESNVMLVITLFLSCSFTSLQIWGLIAGIRDLNIIMETASPLFISILVLLKLINCLCN

KDKMKHLLKRIEETWKRIQIGPENKILQNHAEQNRTLIIKYASTLYILSTLYSTMPVVVKTLHSLLPTNETYATRFLFRL

DHVLDVDKYFNLLMLNGILGIFFLMSVWVAADGMFILCTQHVCALFETVQYNVKRIQGSDFVIDEPDDEAYHVIISCIKS

YEYALKFSELLSSTYATHFLFLLGNVVVAVSFSVAELIMMDIPLDETVRLTACNIGLLLHIFYLSLMAQQIIDYSDKFQE

VIYSCNWYKISQRSKKLLRFTLLRASKPCQIKAGKMFVMSMENFSSIIRACMSYVAVLTSL

>CfOr276

NHYYYINKMLLSLIGQWPFQSRLEGNIMFVITLFFICSLTILELWGLIAGITDLSVIIENVSPLLVNNSVFIKLINSLYN

NYKMKDLLEHIEETWKMTQTKPERKILESYAERTRTLTIQYAITIYAMWISYSTMPFVIKGIHLLLPSNETYPVKFLYRL

EHVLDVDKYFNLLMLHGIISIFYIVSIPIAVDTMFILYVQHTCALFKNIVHNMKNIRSSDFEMANDETYHIIIKYVKSYK

YALRFSDLLSSTYTTCNFFLLGNVIISLSFSAAELIMVANELDEIVRIIASNMAQLIHIFYLNLISQRLIDHSSRLQEAI

YNCDWYKLSLRSRHLLRFMLMRAIKPCYIRAGKMYVMSLENFSSILQVSMSYFTMLTSM

>CfOr279

NRNYYYDISKRFLWMVGQWPYQKPKTRLSFMALVVIVLANCLFTQIAQIFVCEDTQCIFQTLPPHLLVWNSLVKVLAYRF

NSQKIKDLTDHLFVDWDTLKTQEEREIMKKYADNGRWYSLIYASYCYVSTVSFITTSLVPRIMDIVFPLNTSRPIMLAYP

AHYFVNEEQYFYYISFHMLITALICVTGLIAHDCMFFIYIEHVCGLFAVVGFRFKHALDKQDNLIDCSDNLYHKNVTFSI

HAHREALQFAKLIENAFSVPFAIQLMISTMSISVSLLQFSMQLNDLMEAMRYFVYILAQLFHLFCFSFQGQKLIDHSLEI

CDKIYNSAWYEIPLKGQKLLLITMRKSIEASTLTACKIYVFSLQNFTMVLQTAMSYFTVLASF

>CfOr280

YNNNYYYDINKTFLSIIGQWPYQKPKAKLFFLVFALIFLSNSFVTQMAYMFIHRDNMDCILQILPFFFLMINIIVKVLTY

RLKSEKIKALTDHLFADWNMLETRKEREILRKYAENGRRYTLIYSMYMYVTAISYAITSLMPRIMDIVFPLNTSRPIMLL

WPAYYFVDEKKYYYYIYCDMLIILMVWLAVLIAHDSMFFIYIQHVCGLFAAIGFRFKYLICRFKYLICKRDKKSLIDCPD

DIYHKRIVFSIHAHRKALQFAILIENVFSKSFAIQLAVNTVSISISLLQFSIHLHDMAEAMRYCLYIIAQLFHLFCFSFQ

GQKLINHSLETHNKIYNGVWYEMPVKEQKLLLLVMRKSIEASTVTACKIYIFSLQNFTTVLQSAMSYFMMLASF

>CfOr281

ESNMEYYYNINKRLLSLIGQWPYQKPKEKWAFLILILIIVTNLLITQVAQFFICEDAQCIYQTLPPHMLGIMLLVKIFTF

YFNKQKIKVLTDRLFIDMDMFENQDERKIMKRYAESGRWYTLTYASYVYIATLSFATTALIPRILDIVSPLNTSRPIVLA

YPAYYFVNEEKYFYYIFCHMFVTAGLGLTGLIAHDCMLFAYIEHVCGLFAVIGFRFEHMSYNHDSANHDSAKKNIINYSH

DICYKNIVFSIHAHRKALQFAVLLESTFTVSFAIQILIVTVGMSITLVQFTMQLYNLAEAMRYMVFIVAQMFHLFCLSFQ

GQKLINHSLETCDKIFHSSWYEIPVKAQRLLLMVMRKSIVASTLTAGKIYIFSLESFTMVLQTSMSYFTVLSS

>CfOr282

TQNHYYNIVSKISSLTGVWPFLKPRARLFRVTLMTITIFTIFIPQIAYQFTCNRDLHCIFEGCSSYLLSSIAALKVYTFH

LNNNTIKDLTRHLFVDWKKVGNPEEYEIMKSYARNSRRFSLIYSIYCSMAVFMFMSMSLIPFVLDIVSPLNQSRPILSPY

PGHYFVDIREYFLQIFWHSLVAWQILTTGIIAHDCMYVTFVEHICSMFAVIGFRFERLFHNHDEPNDMYRKKVAFIVRTH

RESLKYAELLEDTFNMPFAAQILMVTIGMSVTLLQISRQDGDLLDLIRYVLYVVGQLIHLFLLSFEGQKLIDHSLQTRDR

IYNSAWYKASIKSQKLLMLVMIKCLRPCVLSAGKIYIFSLESFTMILQTSMSYFTVLASF

>CfOr283

WNYYYSITKRMLLLAGQWPYQRKKERLLRMSLMTLTSLSMIVPQIGKFIQCDKDVQCILTVIPTHLFQLVVIVKLYTCQF

NNSKIKDLTDKVYSDWKSVEIPEECEIMKTYAAKARLFTLIYTSYYFIAAPIFVLITLTPQILDIVLPLNESRPILMPYE

AHYFVDDTEYFFYIFVHTLVGIIILCIAILAHDCMILTCIEHVCGIFAVAGFRFENLAYNENMNNNLDNIYNQKIALSVH

AHWRALQFAELLEDTFSITFIIQILINTAVMSVTLLKIAVQLDDAMEAVRYIAFVIGQLIHLFCFSLQGQRLIDHSLQMR

DKIYNGSWYKIPVKSQKMLLHVMRQCLQPNFLSAGKIYIFSLKSFTTVLQSSMSYFTVLTSF

>CfOr284

ATWNYYYNFVYKISSLVGLWPFLKPRTRIFRVTIFTIALFNISIPQIAYQFTCKKDLHCIFEGSTSYLCTIVIVLKMYTF

QLNINTIKDLTRNLFVDWKKIRNPEEYEIMKSYARNTRRFSIVYTVYCSMAVFTFMSMSLIPFVLDIVSPLNQSRPVLPP

YPGYYFVDIRENFLQIFLHSLIVWQILMSGIMAHDSMYVMFVEHICGMFSVIGFRFERLSYNHDELTENDIYRKKVAFIV

RMHRECLKYSKLLEDTFNMPFAAQMLIATIVMSVTLLQISRQDGELLDLIRYMLYVIGQLIHLFILNFEGQKLIDHSVQT

RDRIYNSAWYKASIKSQKLLMLVMIKCLRPSVLSAGKIYIFSLENFTAVLQTSMSYFTVLASF

>CfOr285

HYYNTIHKISSLSGLWPFLKPRVRIFRVTLFTITVFTIIVPQIAFQFTCKEDLQCTFKSISSYLLTISIMLKVYTFQLNI

NAIKDLTRHLFVNWEKAESPEEYEIMKSYAQSSRRFSLIYSVYCIIAVFIFVSISLIPFVLDIVSPLNQSRPVLLPYPGY

YFVDIHEYFWQIFWHSLVAWQILTIGMIAHDCMYVTFVEHICSMFSVIGFRFEHLIYNYDKTKLNDMYRKKVAFIVRMHR

ESLKYTKLLEDTFNMPLAAQMLMVTAGISLTLLQLSRQDSDLLDLIRYMLYVIGQLIHLFILNFEGQKLIDHSVQTRDRI

YNSAWYKASMKSQKLLMLVMMKCLRLSVLSAGRIYIFSLQNFTMVLQTSMSYFTVLASF

>CfOr286

SNIVYYYDINKRFLSLVGQWPYQTPKEQRSFLILIMIIIINTMVTLVAKFFICKDAQCIYASLPPNMLAVAVLVKIFTFY

FNKQKIKDLTDRLFMNWDMLENQEEREIMRKYTKTGKWYALIYACYIYIGLMLFASIALIPRILDIVFPLNTSRPIELVY

PAYYFVNEEQYFYYIFCHMIISAELSLTAVVATDCMLFVYMEHLCGLFAVIGFRFEHMLYKYNNNNVKKLMINRSDDMYC

KNVMFAVHVHREALQFAILLENTFSLSFGIQMLIAVVGMSISLVQLHDFGEAMRYMLFIGGQLIHLFCYSFQGQKLINHS

TGICDKIYNGSWYEIPITAQRLLLMVMRKGIEASTFTAGKIYVFSLANFTAVLQTSMSYFTVLSSF

>CfOr287

ATWNYYYNFVYKISSLVGLWPFLKPRTRIFRVTVFTITSFTAFIPQIAFQFTCKEDLRCTFKASTSYLYTILIMLKMYTF

QLNINTMKDLTRHLFVDWKKAENPEEYKIMKLYAQNSRRFSLLYLIYCSMGIFIFVSISLIPFVLDIVSPLNQSRPVLLP

YPGYYFVDIREYFLQIFWHSLVAWQILTTGIIAHDCMYVTFVEHICSMFSVIGFRFEHLIYNYDKTKMYRKKVAFIVRMH

RESLKYTKLLEDTFNMPFAAQMLIVTVGMSLTLLQLSRQDSNLLDLIRYMLYVIGQLIHLFILNFEGQKLIDHSVQTRDR

IYNSAWYKASMKSQKLLMLVMMKCLRLSVLSAGRIYIFSLQNFTMVLQTSMSYFTVLASF

>CfOr288

WNYYYSITKKMLLFAGQWPYQKKREKLLRTILLITTELSVMITQVGRVIQCDKNVQCILIGLPTYLVHIVIMVKLIACQF

NSNKIKYLTDQLYFDWKTLENPEEYEIMKTYAAKARLISLTYALYYLICCPLFIFTSLIPKILDIVVPLNESRPIIMPHE

CHYFVSDDTEYFYYIFLHAFISMIILVIPLLAHDCMILTYIEHVCGIFAVAGFRFKNLAHNTGIENNNIENYNCSWYKIP

VKSQRLLLNVMRRSLQPNILSAGRIYIFSLKSFMTVLQSAVSYFTVLASF

>CfOr291

NYYYSITKRMLSLVGQWPYQKRKERLPRMTIVFLTELSVLVTQVGRFIQCGKNLQCILIGIPTYLLHTVITVKLLTCQFY

SSKIKYLTEQLYNDWKNLKSQEEYEIMKTYGARARLISLIYSSYYYISCPLFVLFSLTPKILDKLLPLNESRPILLPHEC

HYFVDHREYFYYIFFHVLISTFIVLTGLLAHDCVILTYIEHVCGIFAVAGFRFENLTHNTDIENDKINVQSKYIQCKTYN

QKTYNQEIALSVHVHWRALQYAEFLKNTFSVTLLIQMFIVIVAMSVTLLQMVVQLENIIETTRYMAFFTGQLIHIFCFSL

QGQRLIDHSLQIHDKIYNCSWYKIPVKSQKLLLNIMRRSSQPNILSAGRIYVFSLKSFMTVLQASMSYFTI

>CfOr296

TRYFRINKLLLSHVGLWPHKSSSNKNIILYCTIAGILXLALPQIAYLFKHATSVNDFYDVLPPLSGACICLMKLISLFHN

FNKFRMLIQHVQYDWRLLENHENFRILMEYLERNRAIPAFISTTATSYITAPLTVPMLDIILALNVTRPKELPHSGEFFV

DLXYYYILFAITCLGYYVSCTSVIAIDTIYFALLQHSXGMLAVLSHRLENCINRVDCNGISKWDKDVENIIQCIQLQIRI

ERLIQLIESTFAMCLILDIGLGVIFQCTARVMVINIHTNTMEIMRYGQLLLLQNSRFFLNNWIGQEIINHSSQIPIAXYN

GMWYQTSLKVKKMFLYLLMKSQKPYRIIMLKLYXISLEDYTMLMKTSVSYITLMVSL

>CfOr297

NRYYYLNKTFLSIIGQWPFQSRLEGNVMLAIAILFICSLTALELLGLIAGITNLNIVMENSSPLFINSLIIVKLINSLCN

KHKMKDLLENIEEIWKMTPIGPESKILRNYAEQNRIFTIQYAVALLSSGIFYSTMPIVVSGIYSFLPTNENYTARFLYRL

EHVLDVDKYFNLLMLHGIISIFYIAYVPIATDCMFILCIQHVCALFEVIRYKINRIRSIFSRNRYKINRIRSSDFVILKP

NIEDDEAYHVIIDCIKSYKHALKLVLLVSFYCFFSFYFKFSDLLSSVYGLIMVDNQLDETIRIVSAIMGLLTHIFYLSLT

SQRLIDHSSELQEAIYSCEWYKISLRSRQLLKFTLMRATKPCQIKAKMYVMSLENFSSILQVSMSYFTMLTSI

>CfOr298

TRYFRINKLLLSHVGLWPHKSSSNKNIILYCTIVGILTLALPQIAYLFKHATSVNDFYDVLPPLSGACICLMKLISLFHN

FNKFRMLIQHVQYDWRLLENHENFRILMEYLERSRMFTMSYLIFISTTATSYITAPLTVPMLDIILALNVTRPKELPHSG

EFFVDLXYYYILFAITCLGYYVSCTSVIAIDTIYFALLQHSCGMLAVLSHRLENCINRVDCNGISKWDKDVENIIQCIQL

QIRIERLIQLIESTFATCLIIDIGLGIIYQCTACVMIVTHTNTMEIMRYGQLLLLQNSRFFLNSWIGQEIIDHSSQIPIA

AYNGMWYQTSLKVKKIFLFLLMKSQKPYRITMAKLYVISLEGYTMLMKTSVSYITLMVSL

>CfOr299

TRYFRINKLFLSFIGLWPYQTSFMKILTLSFAISGVTIMSWPQIAYLLKHLDDLDGVFEVMPTTSGALICAVKIISLTRN

SEKFKVLLRQIYEDWRDLLTNQETQILTRYANNARKFTLVYSISIIGFVFCYAMLPLTAPVLDIISPLNESRPKKMPHAA

EFFVNQDKYYYVLLLNTYMGYIACVSIAVAADTIYVTLVEHICGMYDILCYRLKNLITHDHDKIGQRIRCCIQLHERLLV

FIEMMESTFALFLLFDVGLGFILHTSSCVMFVVRMGRSFEILRYVALVLLQSCRLFFNSWAGQEVTDHSAGISIAAYNGM

WYNAPIEVQKCLILLIARSQKPSQITIAKLYVVNLESFSKVMKTSVSYCTVIISLR

>CfOr341

RNYRVNKILLSAIGQWPYQSSKTNYIITIVITTITCSQLLTKLCSMFSYIHDMEIVIECLTPIMIDISGITKIMNSMLCV

NEMRTLLDQIQADFRSLRNSGDIEILQKYADSGRKFSIIYIYTLYIVMVIFMLTPLPALILHVANETRPMLHRVEYYVDI

DKYYFPILIHGYLTVLICVTSIVATDAMLVIFVQHACGMFIITGSRIKRALQGELVQHACGMFIITGSRIKRALQGELTD

ANLSVVEDKAYRNMIQCVHDHRAAIRFVNLIEAAYSKYILFHTGLNMIAISITGVGAVTKLNDPSELFKLIAVSWALLFH

LCLECLNAQKLMDYSGYLHTSLVNLNWYDASPRTKKLVLFMMMKTHSPCVLTAGGMFVLCMETYATIVKTAVSYFTFLRS

>CfOr342

KRYYKWNRFFLLATGLWPYQSVWSARLNRGISIVIILCMVFVQLNSIFTINITREFMVIVVEVLAVTIGILYHLFAHMRH

IDKYKQLFDHMWQDWALQKTNYEIRIMHQYANITKLVTFYYALLMYGVEVTYFTWLFMPEILDVVSPLNESRPRKQPFDY

NFFIDEEQYFYFIRFLIFIGCTFVPIIFLATSTLFLAFTQHVCAMYKLLGHRAEHLFYVIGSTAEIDLNRKARIKCEKIA

ILVRMHYNIIQFVFLINVYYIFLVRMHYNIIQFVFLINVYYIFNILTDMSSTLCQKLKLFQIYFLICISILNVFVILLIK

NCLVVMISKXKNKWYNSAWYDAVISEQNSLLLMMRRCLHPLVLTACKFYVMSLQSFGMVLQMTISYCMFIK

>CfOr343

EYYYKFNRFLLSVTGLWPYQSKWSACFARTIITVIMLSAIFTQISSVFTSELNMDFVIESLPMFVPTVGNLCQLYSRIFY

VDKIKELLEHMWNDWALEKTDIESKIMHQYAKTTRLVMIYHSLLLYIIVTAMAISMFLPEIFVILPVNKSSQRLEAIHME

FYLDKERYIYLIMSCVCIVLFLVPLVFLASSSLYLVLTQHVCSMCEVLGYRAERLFYIIEDKTKISCKNIVVLVQLHYNV

IEFVNTIETCHTIPFLMDLVGVVITISFWLIQILTIFENVKRACASIGLIIASLCYLAIPNYMGQKVTDMTSSICEKVYN

SAWYYASVSEQKSLLLIMGRRFRPLVLTACKFYAMSLPSFEMILQMGVSYCMFMRKV

>CfOr344

VERYFKLNRFLLFVTGLWPYQSEWSAYLMRIINFAVLLISAFIQMMTILTSELTLEFLTSVLPMILPTLGFMMQMYLRIR

FIDKLKKLFEYMWDDWALQKTHDEIKIMYEHAETTKLWTLGYFSFYYLAITIYSVWLFTPEILDIISPINESRPRIQLSY

MRFELFIDEDRYFYFIRFYICIMSFVTPLISMACSTLFVVFTQHVCAMCELLGYRAERLFCTVGNRETKINCEKITVFVR

LHNNIIEFIGTINSYYTVPCLMDLVGFILLTGITVFQILSAVKIEQAIRSIILTSMLLCHMFMHNYMGDKVTDKSSNVCE

KVYNSAWYDTLILEQKLLLLIIRRRSNPLVLSAWFYIFSLSNFGLILQTVFSYCMFIRQ

>CfOr345

MLRYYSRNQFFLSQIGIWPYQSKMIKILLPCFIISSLLSILVPQVFLLYNSWGDMNMTVEGIVNIILVTGATTKLVNIVT

NNKKLQYLLQVMDEHWRLFHGKCELHILKRYANIGQLITKYYSVYINIFVVLFLSIPLIPKILDVVIPLNQSRPFIYVLE

GDWGVDKEKYYYPIMLHCCLAIIVGTRCLVNIDTMYVVCVLHGCSLFNAIGIRLENILSKAKLMQHEQIMSLEYIKEYYS

KEYYSEDYHEMIACLKKHQLAIKYIYMLDSTFKHATFLILFLNVMILSLVGLQLINKFGQTQEVIRFGCIVMGSITHLLS

MCLPGQLLLDRSIEIFDKAKSAQWYMFSLKTTKLLSILLHRSLVPCTLSAANMYIMSMTTFSSVMQTAMSYFTTFLSV

>CfOr346

MLRYYYRNQIFLSQMGIWPYQHRMIKILLPCFLVLSEASLFVTQILLVHNTWGDMNMAVEGIVNMILLTGATTKLLNIVI

NNKKLQYLLELIHEHWQLFHSESELDILRYYANIAQKVTKYYSVYINIFVAIFMSIPLMPKILDVIIPLNESRPSIYVIE

GDWGVDKEKYYYPIMLHSYLAVIISTRCMVNVDTMYIACVLHGCSLFNAIGNRLENIFDKVKLKNEIQMNKKHSIIKEYN

SEDYHEMIACLKKHQLAIQFVLWKNFIININFIYIXIMIIIYNILNFIYVICNFGQIQEVIRFACLVMGSVTHLVSICLP

GELLLDSSLEVFDKAYNAQWYMFSLKTKRLLSILLHRSLVPCILSAANMYIMSMTTFSSVMQTAMSYFTTFLSV

>CfOr360

NRYYYINKMLLSLIGQWPFQSRMEGIVMLVITLFLSCSFMSLQIWGLIAGINDLNIIMENASPLFIDVFIFMKLINCLYN

KDKMKHLLERIEETWKRIQVGPENKILQNHAEQNRTLIIRYASIMYMLGVFYSAMPIIVKVLYWLLPTNEAYTTRFLFRL

NHVLDVDKYFTLLMLNGILGVLFLMSVWVAADGMFILCTQHVCALFETVQNKVKRIQGSDIVMDEPMDDEAYHVIIDCIK

SYKYTLKFSELLSSTYGTHFLFLLGNVVVAVSFCIAELIMRDIPLDETVRLIACNTGLLLHIFYLSLMAQQIIDYSDKFQ

EVIYSCNWYKISLRSRKLLRFMLLRASKPCQIKAGKMYVMSMENFSSILQTSMSYVAVLTSL

>CfOr388

LREYNINRKFLSCLGFWPFQRKLARYSLPIFCFVLELSYLPFEIITLYMHRRSKQMIFECLYQMTVTIAFLVKLVNQLWN

RSKFQRLYETMENHWNIFTNDFEVQIMKNYSGISQKFTISYSILIYVMSTMFITIPSLGPIFLDIVLPLNESRPRHLALY

AEYGIDQNKYFVGIFLYTTIMIMVGLTIMVAVDTMHIACTAHACSLFQVIGHQIENVSNMREVKLFNEKVIYREYILCLK

KHQLAIEYVDILNDTHKIVGISFSLIIGMIFSLLGIRIVYVLDQVEEIIRFFFIITGALIQLMIVCYSGQKLMDESQNIF

HRAYAAEWYKFSPRLKSLLIIILYRSVVPCKLTAGNLFPLSMVIFATVIRTGISYFTAFLSFK

>CfOr389

REYNVNKIFLSRLGLWPFQSKLVRDLLPTFYLMLEISFYPFEILMVYHHRDDTQMVFEGCYQLVISSAFLVRLWNEIWNR

NKFQCLYEAMNDHWNIFTNDLEVRILKDYSIISQKFTIFYSIMMYLLSSMFIVIPLTPAFLDIVLPLNESRPRILAVEVD

SRIDKDKHFVPLFCYTTAIIVVGISIMVGADTMHFTCTNHACSLFAIIGEQIENIKECCMNKKFKSSNERAIYQEYITCL

KKYQLALKFVDILNSTHQTVAVFFLLLIGATLSLIGIRIVYVLDQMEEMIRFMFIIMGALLQLMIMCYSGQKLIDESQNI

FYRAYAAKWYMYSPRMRSLLIITLYRSNIPCSLTAGKLIPLSMTTYAAVVRAGMSYFTAFLSIK

>CfOr391

NSTWSQYYDIVKRVSSLSGQWPYQRSKTRLLCVCLVTLSTFSMIIPQIAKFTICDGDLRCIFETMTSYMLTSVTLVKLYT

CYFNRCKIKMLIDRLFVDWDELETPEEYEIMKRYAENSRRYSLGYSLYCFSSVCLFMCISLIPQLLDVVLPLNESRPILS

TYPGYYFVDEKKYFFYIFSHAIVAWEIAMAGIVTHDCMLLTYIEHVCSIFAIVGFRFEQLTYRHTDDTCKRIAFSVQTHR

KALKFAELIEDTFSLTLAIQLALNTVMISITLLQITQQKGDFLEAIRYIFYVFGQLIHLFCLSFEGQKLIDHSLQIRDKI

YNSFWYKTSAKSQKMLLFVIRKSFQPMFLSAGKIYIFSMESFTTVVQTSMSYFTILASL

>CfOr392

TTCNHYYNIVYKIASIAGIWPYLKPKTRMFRVTFLTMILLTIFIPQIAYQFTCKEMQCTYQAMTAYLLSIIVMLKMYTFH

LNNRTIKDLTHQLFYDWKTLESTEEYKIMKSYAENSKRFSLLWSVYVFMAVIMFMSMSLIPYMLDIVLPLNESRPILPPY

RGYYFVDIGEYFFQIYWHSIVAWEIVVTGVIAHDCMFVAYVEHVCSMFAIAGFRYEHLFYYHKEKGSNTDDTYDKRVAFL

VYTHREALKYAQLIEDTFTIPFAIQMFIVTIGMSISLLQITQQDDILEAIRYVFYVIGQLIHLFCLSFEGQKLIDHSLQM

RDKIYNSSWYKTSIKLQKLIILVMMKGLRPSFLTAGKIYIFSLESFTTVLQTSVSYLTVLGS

>CfOr399

DRFYGLTRRLMIFLGLWPYQNPKHRKLLLVFVWIIYMKSLLIQYMTFIMHPHDMKLYIEIISFHTLLVLFIIISNIIYLN

STNIKKLLDYIQRDWNLIKDTNELKIIQKYAYKARFYTVFSALIVYPGTSIFISMIFVPDILNILMPLDKPRLRQLPVQV

EVFFDIEIYFYVFFLFFITVVFLGMTILMATETMYMTFIQHACGLFELVSCRLTCALNTNLSGLKTKCKLCTKLLHAFLA

HQHCLEFIKIVQYEFSTSYFILCILGVASLSINMFRFFHAIHNIVEVISTGLFVFAHFCYTFYINYFGQDLIDQSECFFQ

QIYSTQWYTAPICAQKLLFIALQRSAKTSKIVIGSLFVASFEGFATLISMSLSYCMVIYSV

>CfOr400

NEYYGLTRNLMASVGLWPYQKSEHRIVRVFCISCILIISGLVLTTFTTHEYNTTLLIEVFSFTIICIVYVMKYCIVHFNS

NHVKNLFDQIQRDWNLIKDIDELKIIQKYAYKARFYTVFSGLIVYLGTFIFLSMMFVPDILDILMPLDKPRLRQLPFQIE

VFFDTERYFYFIAFLLIIINFLGMTVLMATETMFMAFIQHVCGLFEMFIENMYCAFSISYFFLCAFGVTSLSINMFRFLH

AIHNIFEIISTGLFVFAHLCYTFYVNYLGQDLIDQSECFFQQVYNTQWYTAPLYAQKLLFITLQRSAKSSKLIIGGLFVA

SLLHLKLISMSLSYCMVIYSVR

>CfOr402

DINNYVFINRKVLKFIGLYPTNIVRYIMCCVCMFAIVIPQAMQIYQNWNDLAIVLETSSVLLTILLAILKSLVWISNRRK

MDSFIQYMLTNYWEIMTAHVSANIYAIYVRKGSLYTKRYLFLICNSLIFFFSLPIIEIFVTMIKSTNDNNTSTKHFPFIA

LYPKNYYNFPMYEIIYLSQMVATSLCGLIILGTDTLIATAVFHTCGHFKILHKKIENINTEIDLIVYIEENIRKIKLQII

DIIKHHHLVLWFCDYMEKIFSPMLFLQTLASSLIICLVGFQIATVNTAIIKSIKYVSYLIMALFQLLLFCIPGDALIYES

SMISRTMYTIAWYELSTLFKTEVCLLMLRSQKSSKITAGKFYTMHLENFNAVLNTAISYFMLLRSF

>CfOr403

NEPFASVIRANVMLLRFSGVVSYKDRRTNSQNVLSAIAYGCLFAYAFLYTYEFALHTVYLDTWMESFAMILSLVGGQARF

TLVLLSRSRFQRLLAICEELWTTLNATERKCVRDYVKPTRSLTYYYLFGCAFTIFFYAVASLFMGQHDDSSNATTRTLPY

ACPVQVHRTPYYEIMYALQLSSMINVGLTCAAADTLGPVLILTVCGHFKVLNSRILSLSDRAYSKSSNRVKSDLKNCVHY

HQMVLEFCKEVEKLTNGIFLTQLVGSTYNVSLVGFKLAGEDPDKFKYTTQLSIAMIQLFLCNWPADVLLTESQDVARAMY

FTSWYRFSYQLKRSINIITMRAQKPTQLTAGYIVPLSLQTFASMISSAASFFTMIRSM

>CfOr406

DNRNYYYDVSKKFLWMVGQWPYQKPKTRLSFFALLVTTLIICIITQTAHIFLCKDSECIFETLPAHLLMWNSLVKVCTYQ

FNSQKIKNLTDHLFVDWDILETREEREIMKKYAESGRWSSLIYAWYYYVSTVSFIMTSLIPPIMDILFPLNISRPIILAY

PAHYFINEEQYFYYIFCHMLMTGVICMTGLIAHDCMFFTYIEHVCGLFTVVGFRFEQILYKRNKRKLIDYPDDVYNKNIA

FSVHSYRRALQFAELVEKVFSISFAMQLMIATIGLSVTLLQLHNLMDTTKYFVYIFAQLFHLFCFSFQGQKLIDHSLQIC

YKIYNSAWYEIPVKGQKLLLITMRKSTKASTVTACKIYVYSLQSFTTVLQTAMSYFTVLASF

>cplu_000252_b1

SYAAALLVYLVVGTIQLCIIGYHILVLMGKQHSLMPFFVFIITTYGLISIYCILSENLLAESKKCSEAFWCCQWYEMPQD

CVKDIAYCIMRAQKPLGLTAGAFVVFSNSTLTDVTKTSMGYLSILRNF

>cplu_000774_b1

TEITWYLQPLTTPASPIESDNETVQFLLPYHFYVFYEMNGFHSYVLTYLSHGPHVVISGFGHMTSDCFLIILVFHLSGRL

AVLTERINTLKEE

>cplu_000919_b1

MTCSYYLTTTAVLMGPLVLPEKFPSDAVYPFSVDHPVVATMVYIHQCIVGYQVSAGMTLDCQAALFLWYLSARFEILISE

IENVSSLKEVGDFVKTHQEILRFADRVIRPIRLIVLTTVTMTKVGMISGALVLISDEPFTVKVQFTILVISASINIYVCA

WAADNLLTVSETISEKIFSTTATHSPIIRKIWMTVIHRAQKPITVNIPGFLELSNEYYANFLSTAFSVFTAMHA

>cplu_001022_b1

MFDWGRWISKGIGVWPIAPNDYLFSVTFSYFTAVMILEFVDLYTCLGNFEKVIDNLTENLAFVHIYVRTLMLRVHIDELR

YVITESLNDYKASAFRNSEIKIFMSHINKGKLFAKVVLTFIAMTEITWYLQPLTTP

>cplu_002078_b1

VVDSSETDIVEIPRLLVRSWYPFDARHGVAHIGMLIYQIYWLLVCTVDANSIDVLFCSWLLFACEQLQHLKAIMKPLMEL

SATLDTVVPNSGEASKS

>cplu_002097_b1

CLFFYLQVNGINVYAASTIGYLLYSLGQVFLFCIFGNRLIEEV

>cplu_002266_b1

KEEPEIYKKLKSIIKEHMRLLKMGENIREAFATALLAYLLNGTILLCMIGYQILVNTGPNSDLMQYLFLYLQLFY

>cplu_005941_b1

LNIELIVLQLIGLKSMRNDNIEIYIKSSYKEGFISSVFMLYVYSFWTLLVGYNDISFAAEVITVILSASMCMIKGLTLSL

HRRQLFNILRDFHILWKNARTRKHLRPQIDELINSSKPARYCYMFAAISVGLSYGLRPYYLLVSHYIINSNKSLDYSATV

YPLLYPFPYNSLISYNFCIIYEQCVNYFAVFYWITCDTIFIQLTTHICIHM

>cplu_006402_b1

FVIRFRSCVMHHRRLISHLNEFNDLFNVVLFGQILTSCTLTCVGMFQLVLALEKKRNTFRAATLFALGLVHLIYWCANGD

RIIVENDKLSVSIYQSGWENISKETKGLILNGLIQGLMPMKMKAGAFFIFSMKTYLTVIRTSYSYFAILST

>cplu_006618_b1

ILTKALIFYTSLTEVFWYLKPLVAISEDMIDGNNKTTYILPYEIWWFYEIDSTKGFLFPYLFLLPGPFLATAGSMGAVCL

VIFFVFYISGRMAVLTERIDRLESDQENSDQENTRSELTKIIAEHTKLLEMGDEVTDSFRVILLVYFVSVNFMLCVLG

>cplu_006991_b1

QXITFFSYFSDWYDFPPKYQRLLIPIMMRSKTPCSISAGSIFIL

>cplu_007267_b1

DGQLLGLVTYVILMTSIFVNCFIISFVGERLKEQSIRVGERAYAAHWYSLPKSFAYDLMLIVIRTSQPVTLSTGKVSDLS

LAGFAGVVKTSAAYLNFIRAV

>cplu_007912_b1

LLTTTAVILGPLILPQKFPTDAVYPFSVENRFVSRIVYLHQIIVGYQCSAGMALDCQAAMYLWYLSARFEILSSEIRNVD

SHNHNDLRNYIKKHQKTLMDAKELIRPTRLLALVTVMMTKIGMIFGGIVLISDEPVVIKIQFGILVISTTVNIYVCAWAA

DNLITVSTKLSNAIFETSWRHEPKIRNLLLTVIYRTQKPIIIK

>cplu_008889_b1

KVFSWVRLISKLIGIWPLDPNYYLYNICITYFTYIMITEYIDLYFCLPNFKKVINNLTESLAFTQMYVRGIMLRVHIEKL

QRITAESVKDYDVFKNPDEVYEFLSYVKRGKFFVRAVTIFILSTTTSWFLRPITSSTPATSIIPGDNETAAKFTYILPYK

FHVFYKIND

>cplu_009020_b1

LTIYLPQSTLAYLNWGNMNAVIESLSINGPIFIAIIKIIIFRRYRKVLRVAIDMMARDWGEVKSKEEYTVMLETAKISRI

ISVVSTIITNSLFIAFVFFKIWIGKQLIKRTDLDSRLSDPRLLYPGYLPYNSRLMIFFIPSWIAQCFATIF

>cplu_009436_b1

AKSIFISYTGTIICAATLFSGSREGVMDPPYHLPYSGWFPYNYTNPKIYWATAGLQMYGIYSTAAVDLAFDPLMSAVFCH

LCAQIHILKHRFGVMVS

>cplu_009603_b1

CCLIQVVCLVINQKELVELHTNLDPYFDKLLTNSKLTKIVLKKVKAYRFLSWTLTFCVFTCLAAYIIGPLVYIAHCYFNK

IMITKYPLVYPSGYPWKIPSNFVYKIHFIFETLATSALFFVTCSVDSLFTLYVFQMVAQLREMSYCITHIETEDESQ

>cplu_009998_b1

IAQWINHHNEVLRYSREVHEIFSGAILLNYCLSSTQICMTAYNLANLSASSS

>cplu_010218_b1

SYAKMSTIIRNHDRVLQYSNKLKNVLSEICLVEIAATTILLCISEFSSLRLNGDIINTIPYIIIFGGLSLNMLILCYFSE

ILNSQFSEIGTQLYMT

>cplu_010389_b1

ESPEEIYIFMGYIRKGKFYVKIGASFVFAASTSWFLRPITSSVPATTIIPATNETPAQFIYNLPYKFHIFYEVNDYPAYF

LTYMSWSPFLYISCIGSITSQIM

>cplu_010870_b1

HILGLCKQIEILFNPIIFFTVLFNGIDLCCCIFTLDKEEGHWPKFARSIAHAMTLLIQIIIYCNFAHRATEMTKSITTSI

YNSPWMECDRKVKKLLMITTMRA

>cplu_011994_b1

INCFQWRIRYCIRHHQRIVEIVNRLNDTLSSCLMAQFAVSTMIFCLNGFLQSHFKIDEVDFFLLVGFIQIFYWCRFGNEL

KFQADYLTTSQWMSGWENFNSELRNYITVAMIRTMKPVEIKAGGLFVLSMETFLSILKNSYSVFVLLTTV

>cplu_012123_b1

LGLAYAIRPYCAMLIGYFQKRSLIFDYSMTVYPLNYPFPHDTDLSYNLCLIYEEIVVYLVTLYWVACDITFIQLTTHLRI

HIRALSNNFSMESYDVEE

>cplu_012406_b1

LWESYRFCWYGNEFTHVTNSLTYNQWSSGWEYVKQKLHNLITTTMLQTMHPTEFKAVGLFILSTATFLSVVKSSYSVLAL

LMNM

>cplu_012680_b1

ELPVRALFPFDTRTSPMHEVAFFIQIYSIAFALTNVVTLEFIGLGFIRWTTVQLFILTWNYKNCRTDIKNADHDGSKDCY

LWRFQICIKHHQKLTWIVKELNSVFSSSMMMQLAASATMICLAGFQAVLGSNDKSSFMKFTVYLGATFTQLLYWC

>cplu_013008_b1

LLTAESDKCRDAMYAANWVGDKPFMHSVIIMLSQQPLILQACSFTAVSMDVFMSVLNTTISYFLLLNT

>cplu_013731_b1

KVSFAMALLTYLIVGTIQICIIGYHILVITRQQRNLMPLFTFILTTYFVISIYCILSEHLL

>cplu_013870_b1

PIILDLVLPLNETRQKLLPYPTEYFVDQQENFYILYIHTVIVTATALFFYFAFDLTYAGLVSHACSMFTI

>cplu_014152_b1

SIGTAAFSGHWWKFHKSYNRSLGLLITRFNKSIIFTAGPLMKLSLQLFVSILRMSYSFFTLLKS

>cplu_014272_b1

LPVSFFILKSIGLWQPVHKGFKTLLYSLFTGFSFVILFTDTGCQIVDCFVTCKTIADYADHSFILLT

>cplu_014416_b1

LLFPLIYSVVYFIDDMVVILKSIICGAVIINFLIKFLIVRIYHRKFERLGAALNEFILNASDSSMLVFQKYIDKSWKFQF

LMVCGWNFAAGGIVLGPLVLPQDFPTDAVYPFSVEHP

>cplu_014530_b1

QKYIEKSGFFHVFVSVSMWAVCLAFIVEPILLSHPYPTDTAYPFVVKSEPLRIALYLQQIMALCFIAAALTIDFQVATLL

WFTCVKFDVLSHHFREVSSEREL

>cplu_014564_b1

EWCLNPLIYDCYNIYILKITNYTRSLPFEAFIPWKVDSNKKYIFTFAFQFISGIAASTGMVISDVLNVIFLANISLNLE

>cplu_014959_b1

AYKITALTLRIVGLWPLQSKFEGKFLRFLWCFFITTQMIPQICVCITDFGLETLMSTIPPFTVAIIAGAKMIISTINNNK

IIDLMKIMQQDWNSLKSEVEHEIMKKHVEHGRLLTLFISMLYYNSLMVFLLLPVRPKVMSWLGLSKGPADFAFPYPVNYG

VDQDKYFYVIEAHISFCSALVITLIIAADYVF

>cplu_015170_b1

YDKFKNFIRRLLSLVGAWSTEQSNLFARSLLYLHLSVYVVPAIGVFNFVGVNILNIKVVAKGLAILMGFSTNILKAACIL

INQKDVIELHKFLDSYFDTMVKKPKLSKIVLKGVGSFRRLTIIGTVLTTIVCFCYAAFPVLSVINQWHHHTKPIQ

>cplu_015672_b1

NENTKNNPRKFWSEFSIIVERHEKLNELAKVIEDSFSSILLPQMIICTTTFCFQGFAMISSKVSVLEMLFSIVYVFYTVL

HLFVYCYVGDYLSFESSRIGEAYYKGNWYNLSI

>cplu_015722_b1

FLLLVLTVNAIGLSTIGFRFVAMASTMEDNIQCCAFLFGQVLYIYYLSSFGQKLMDHSAYVFESICAAK

>cplu_015763_b1

CYYVYNCFSNDSDGKYFFGIFTWIALKVLQTFMYAWSGSCLTLESDEFRDSIYACEWYGNKRLMTSIIIILSQRPLILTA

CNFSVVSVEIFQMVINTTVSYFFLLQTL

>cplu_016503_b1

EGMDLSVSIYKMNWYFLTNECKKDLLMMKKRTLKPIKYTSGTLIELSLDSFTNLVKFSYSVYNLLHQ

>cplu_016996_b1

WVLCSMYIICFLHLFLIPREKGSLPFRTVYPFDVTKSPYYEIIMVYQYYCLIYLLAVVLAMDITTIGFIRWSAVQLIALT

SNFKNCN

>cplu_016997_b1

VFFIVMCSQQFLQCITAQQVTIEFENLRNEIYNSDWHSSRTTIMKSMLIIMCKTSRPVVFITGHFVDLSLDSFKNIMKLS

YTIFNVL

>cplu_017074_b1

YMSQRLTDMSAHIHQCIARAKWYENSARSRKLLVLMTLRSQIPCKLTAGKVMELSIETFGVCLKTA

>cplu_017206_b1

PVNTRQYFNLIIIMSQQPQKISAGGVVDLSFVTYLQILKTGFAYLQLLRA

>CpluG2R504O01A4FL9

TFNLVILAQLIMSGILICIMGLQVIIATKNLFAGIKALVVLSSLMSQLFLYSYGGDYLTSQ

>CpluG2R504O01A5V86

YSLGQVFLFCIFGNRLIEESSSVMEAAYSCHWYDGSEEAKTLCLTLL

>CpluG2R504O01AYE76

RSLERQVLKELIKHHQVIIQYSRLSNDVFSPVIFVQYSLSSLILCMCVQSLTK

>CpluG2R504O01AYZEU

NWEGMSQKSKQSWLICMVSGKIPMRMTAGKIYIFSFNGFTGILRSSMAYVSLLRTL

>CpluG2R504O01BDC83

NASPIIRKLWLSVIHRTQKPMTINIPGFLELSNEYYANFLSTAFSVFTAMHA

>CpluG2R504O02B3DPJ

ALEFAELIETTFTESFGIVVGLNLPIMSITAVQIMAETNSIQDIVKNIMFTGAQLVHLFFDCYMSQRLTDMSSHIHY

>CpluG2R504O02B4H4X

ENSKGFKYAIAWNRTSMKIVGLWPEPDDGVVTKLRGWFSACLIILIIYLPQSASVYCYWGNMDAVIECLSINGPVLITIV

KIIIFRYNSKSVAK

>CpluG2R504O02B4IFW

ELVKEHLEIYRLLQCSTIIFGFVIFTQYCVSSTVLCVTVFVFAQVRQFDTHCAMMITYTGCLYFQIYLLCSAGTQMTSQS

QNFVSYIYTFDWNELTVKTKKV

>CpluG2R504O02B5A3Y

LLTLSIFGFVTLPQTVALMKTWGNVTLIGDNIMSNLPVTMAGIKLIKMWTNKKILVTMVKQIENDWYQLNNDTERDVMIK

YAKIPKLMLLCGLINITSSTLFYHILSNFFG

>CpluG2R504O02B6JW9

QLTYLLLLQFSASLTIVCLCGFQIILNLNNTFRVMQFAVFVSIALGELFVFCWYGNEFTHVTNSLTYNQWSSGWEYVND

>CpluG2R504O02B35R8

KLFLFCIFGNRLIEESSSVMEAAYSCHWYDGS

>CpluG2R504O02B94FA

CPQFLEILLVRTSINELTNNMTMLLVMVTACGKIVGILFNRNEIVEILKSLEEKPFKPRDQFEEDIRKKYIHISSLLTRS

YSVYLTIGISGILLTRIPEAKLPDVLPFSSWLPYN

>CpluG2R504O02BNSON

NNNNLSSIDEIIKRHCSLLYVCQQITYLYSPIVMAMIILNGIDLCCTIFAFQQDGRAETSLAQNLPHALLLISQMIIYCN

CAHITSEQIESLTEATYNSRWVDGDKKVAKAIVILMMNAQT

>CpluG2R504O02BOZBW

KGLGYQIQYLFEVFGAYTLAAVTISVDTLFMLYIFQIVGRLRKISYNISHSGDDKNSLSASVAHASVAHYAKLIKYIALL

QKIYGPITLITVVTNAVIICTVLFQLS

>CpluG2R504O02BQHS6

LVGVWKPEQKGVKAALYYFYVTVVTIVNHSFLLSGVLDFELRNIDVVIIIDNLSLLSCLFTVRYKIVTILYYRNMIEEFV

NRFQQKPFKAEDSEEEKIYEKF

>CpluG2R504O02BRQNN

ALYNSPWIKNSKELNKSILIVIQRSQKPATIEVSGVLPLSLPYYATFLSKTFSYFTTLRVM

>CpluG2R504O02BT37D

IASCIRKHNEAIKFTELLENIYCWIFFISVGLETLLMTFSGVQLVSQMGNIDELFRWGPFAFGQLVHVFV

>CpluG2R504O02BWT8E

GLYFTQELLNHCGTSYDITSLFGIIFAAVSGHIKILFLHIYNDNIKFIVTSFINDWWTIKNEKCKKIMHEYSSLNRVLFF

SILTSMFCY

>CpluG2R504O02BZYON

FIFVTGIFSFVTFPQTLALIKVWGDLTLIVDNLIVNLPITTSELKIFILWWNKKVLVELFNEIEKDWYGVTDKLEKEIML

NTQ

>CpluG2R504O02CB3Q1

SAVIEQHATTLRFINKVEKLFQQICFVEVVGCTLNICLLGYYTLLGANDTGGVVTYLLLLTSFVYNIFYIV

>CpluG2R504O02CFBJR

LSVLVGFSTVIVKVACFVINRKDINDLYKILDPYVDELLNKSGISDVILKGITNFRRLCKVAAGFVTVSCSSYAIAPILS

IISQWRHEKRPIRYNLIYPTAYPWEH

>CpluG2R504O02CFCWN

WARVKSEEDRQKMIESVNLGRNITKLCAIFMFSGGVSYHTFMPMWKGSTLNELNETIRPLVYPGNEIFST

>CpluG2R504O02CG00L

FYFFCTLRPENILIDCSTKIYHAAYNSEWINLEKRDMYQIIMIINRAQKPLIVTAGKFAPISLSTFAKLIKTSAGYMSVL

LAVR

>CpluG2R504O02CGYUG

SCQRFQRYFSFSLMVQLGGSLLVICLNGYMLVMFPGDKDNLFRSTAYLLSGFTQLLYWCAFGNQLKIS

>CpluG2R504O03C3D7Y

KKIHYWLMLAVSNIFTVFIVLGFISCDLIFITLLQHICGQFAVLGYRIEHTPTAEILTKNDEKEHMSSQKNDRDVSYQYL

I

>CpluG2R504O03C6YS3

WYDGSEEAKTFVQIVCQQCQKAMSISGAKFFTVSLDLFASVLGAVVTYFMVLVQLK

>CpluG2R504O03CT0FW

IITQKPLAITIFNSSTLSVNNCVTIVDTAISYYILLQSL

>CpluG2R504O03CUPD4

IVKYPLIFPSKYPWQTSSNWAYKLTYLFESLATTSLVLITAGMDSLYLFYIFQI

>CpluG2R504O03CZ0JU

LKNNSLKSLVVCIKMHQDLLKFSELLEKIIQIIFSI

>CpluG2R504O03DBPGW

AKNGISDYRAFHDLTKRLLTVGGLWPYSNSNILYRLLPYIQIFLNLAMALVVFGFVLEHFSNVAVVTRGLSSMTSFASAI

LKVVCL

>CpluG2R504O03DDZ9O

SQVXFSVETHPNYEIIYVHQAFAGILCSSIGSIDCQIAMLLWYSIARLDILSLEMKKINN

>CpluG2R504O04D0AMU

RMVGEWVDYHNDILDLVKFVKSLFSTAIFVQYAASSLLICSIAYTLSHTETRSMN

>CpluG2R504O04D2IV3

DNKTRHFILPYHFHICYKLDDFKTYVLTYLWQSPFAAIICFGISSFNVFLLILVFHVSGRMAVLSGRL

>CpluG2R504O04D6C44

CTCISLSLLLIWPLVLSIIEHRNNLLVVIKSVIVIGGILNYVGKVIVVRIYQKELQLLNSTLEEFVANANAEERKILQKY

VNKCWKF

>CpluG2R504O04D7N4S

FLISNEPLAVKVQFGIMVMSTTANIYVCTWAADYLITVSGSLSEEIFGSSWKHEPKVRKMWSIVLLRSQKPVVINIPGLL

NLSNEYYAAFLSAAFSAFATIR

>CpluG2R504O04D7PBQ

PKKFFFLYTHGAIVTPIALIVLVGFDSLYSGFVLHASSMFNILGRRLENLSESNNTKKNLINR

>CpluG2R504O04D7UIF

YLLTIAIVLITSSVDSLYTYYIFQIIGMLREISYQISTFDEKSSE

>CpluG2R504O04D8LAP

THNQWMSGWEYENNELSNLVTTSMISTLKPLQFKAGGMFALSMPTFLMIVKSSYSTLVLLTTV

>CpluG2R504O04D33

KLLILMTMNSQVPCKLTAAKIMELSMENFGMMMKTCGSYFTMLLSM

>CpluG2R504O04D099O

RLKQCIMHHQKIVKMMTILNDCFSFCVVIQIMASTILICLNGFQILLANDDTDELLLYLQF

>CpluG2R504O04D733J

NNKRLRSFIGQWPYQSRKQSIMFGSIMWSLFILQVIPQVVSVALHYDDEELLFESLVAFYH

>CpluG2R504O04DYGYF

QFRRYMQKYQNLLKYAEIMIAPTRILALSTVLATKQGIIISGIFLISNAPPAVKIQFGLMALSMIINIYVCIWPADYLLT

SSKSLSDEIFGLTWKHDSKTRVLWTIVLRQAQKPVAINISGL

>CpluG2R504O04EB3EX

FFIGAYVHVAFDTLFLRMMFSVVVQISVLEHRLEMIISSIKKFIKNNMNS

>CpluG2R504O04EGAA4

YRVHFLTESFAIFSIILITIAVDNLYSLHVFQMIGFLREISYRMKHFHEDNDDKG

>CpluG2R504O04EGOOH

YYKLAKSFASFIGQWPYQSRFQRLVLEFLLWNLFIIQVVPQVVSLVDNFRDLDVVLELLSAFII

>CpluG2R504O04ELLZL

AVYSSNWQGKKSFMVLIVMCLTQRPLAITACNFSIVSLRMFISVLK

>CpluG2R504O04EMG

QKNNNNLSSINEIIKRHCSLLFVCQQITYLYSPMVMATIIFNGIDLCCSIFAF

>CpluG2R504O04EMJN8

QYHSEIGKLLVTGYTAFLSASATIFIMEPIVPRLYSLFSKSNASVPLRFALPLEYVFEKENHYWLMLAISNTFVVIIIFS

LISCDLIFITLLQHICGQFAVLWHRIENTPTPQNYNKNEKKNS

>CpluG2R504O04EMKK7

KLLKKMIFELCLIEVGASSILLCIDEFCVLRMKKDFANMTSYVMIFFSLSLNILLVCYFSELLEAEFMNVGDKLNSVEW

>CpluG2R504O04EP4Q9

QYTGGVGPNQLTKKQEMLVRSAIKYWVERHKHIVRLVTAIGXAYGVALLFHMLI

>CpluG2R504O04EPD9A

FSFSIIQSIYIVEIFRLIEVRGQLAETLDCFPVFIYHSVVQIKITNSYVNINKVKNLLLRVKDDWTSNLDNSEIELLKSE

NRLYK

>CpluG2R504O04ETUCI

SEFKNSIANMKYYDLSIESQKLLLLMTMRSQIPCKVTAAKIMELSIENFGNMVKTSGSYFTMLMSM

>CpomOR8

SLTSFTSIMNSSYSFFTLLRHM

>CUQ99422.1_Olfactory_receptor_coreceptor_[Manduca_sexta]

MMAKVKTQGLVTDVMPNIKLLQLSGHFLFNYYADNSGMTMLLRKMYSTVHAILIFVQFVCMGVNMAMYADEVNELTANTI

TVLFFAHSIIKLGFLAFTSKSFYRTMAVWNQSNSHPLFTESDARYHQIALTKMRRLTYFICFMTVMSVVSWVTITFFGES

VRMIANKETNETLTEPAPRLPLKAWYPFDTMSGSMYVFVFVFQIYWLLFSMSMANLLDVLFCAWLIFACEQLQHLKAIMK

PLMELSASLDTYRPNTAELFRVSSTDKSEKVPDPVDMDIRGIYSTQQDFGMTLRGTGGKLQNFVQNTVNPNGLTQKQEML

ARSAIKYWVERHKHVVRLVASIGDTYGTALLFHMLVSTITLTLLAYQATKINSINVYAFSTIGYLCYTLGQVFHFCIFGN

RLIEESSSVMEAAYSCQWYDGSEEAKTFVQIVCQQCQKALTISGAKFFTVSLDLFASVLGAVVTYFMVLVQLK

>DanaOr1a__scaffold_13117_residues_4903294_to_4905622_forward_strand.[501_1829].sp

NLWTQRVTFARMGLELQPGKGQRVLRSPLLYGIMVLATSFELCTVCAFMVEHRNQIVLCSEALMHGLQMISSLLKMAIFL

LKSQDLVSLVRMIQDPFQGEDIGLDQWRVQNRRGQLLAAVYFMMCAGTSVSFWVMPLVMTLLRFYNTGEFIPVSSFRVLL

PYDVTQPHIYALDCGLMVFVLMFFCCSTTGVDTLYGWCALGLSSQYRRLGQKLKTLSQNPDPLSELFSEHARLLKLVGFF

NASFKEIAFVEVLVICVLYCSVICQYIMPHTNQNFAFLGFYSMVVTTQLCIYLFGAEQVRLEAEGFARQLYQMPWQGLGP

VHRRLLLIPLQRSQKDTELGAYFFQLGRPLLVWIFRTAGSFTTLLNA

>DanaOr2a__scaffold_12929_residues_2038800_to_2041004_reverse_strand.[501_1705].sp

QLDTHRAVRYHWRVWELTGLMQPDGISRLWYLAYSLALNASVTILFPLSLMARLLFTHNMQNLCENLTITITDIVANLKF

LNVFLVRRKIRSLLKHLDERARQVNHPEELAALNEAVTTAQKGFQYFARIFTFGTILSCVRVAISSKRQLLYPAWFGVDW

ENSWAYVICYGYQLFGLIVQAVQNCASDSYPPAYLCLLTGHMRALELRVRRIGYGTRRGELLDCIRDLMLVHRLKGIIQR

ILSVACMAQFACSAAVQCTVAMHFLYVDNDLSAMILSIVFFVAVTLEVFIICYFGERMRTQSEALCDGFYACNWVDQRPI

FKRNLIFTLARTQKPSLIYAGGYIPLSLETFEQLMRFTYSAFTLLLRA

>DanaOr7a_scaffold_13417_residues_392969_to_395983_reverse_strand.[501_2515].sp

CFRNLFNCFYALGMQAPDGPTRSSTWRRVYRCFSAVMYVWQLILVISYRYIEGMEMTQILTSIQVAFDAVILPAKIVALA

WNLSLLRKAEYYLAQLDGRCRDDEEFHVIDEAGRFCNRLVWFYQICYAIYSTSTFLCSFLLGQPPYALYLPLDWSRSLQF

NIQAWLELLIMNWTCLHQASDDVYAVIYLYVVRVQVKLLARRVRQMGSGDPDEQRQEEHCQELQKCIVDHQTVLKLLGCI

SPVISRTIFVQFLITAAIMGTTMINIFIFANTNTKISSIIYLMAVTLQTAPCCYQATSLMLDNEHLALAICQSRWLGQSA

RFRKMLLFYLHHAQQPITLTAMKLFPINLATYFSIAKFSFSLYTLIKGM

>DanaOr9a___scaffold_13335_residues_2364471_to_2368267_forward_strand.[501_3297].sp

QEEDKVEDQDKSLRIQILVYRCMGIDLWSPTSANDRRLLTVVTMGPLFVFMLPMFLAAHKYITEVSLLSDTLGSTFASML

TLIKFLLFVYYRKQFVGLIFRIRGILEKEISEWPAAREIVDMNESDQLLSLTYTRCFGLAGVFAAIKPFVAIVVGLIRGR

TGETKIHLELPHNGVYPWDLQVVLYYVPTYLWNVMASYSAVTMALCVDTLLFFFTYNVCAIFKIAKHRMIHLILLLHQKG

LQIADHIADQYRPLIFLQFFLSALQICFIGFQVADLFPKQSLYFIAFVGSLLIALFIYSKCGENIKSASLDFGNGIYESR

WTDFAPSTKRALLIAGMRAQRPCQMKGYFFEASMATFSAIVRSAMSYIMMLRSF

>DanaOr13a__scaffold_13250_residues_2313551_to_2716342_forward_strand.[501_402292].sp

KDVRKSLRLQCSWLKINGSWPLSKDGMAYTVWAWYVIASVGVTICYQTGFLMTHLSDIIMTTENCCTTFMGALNFIRLIH

LRLNQTKFRNLIGSFYHDIWIPNITDPIIFADCQKRMRTFVIMTALLLCLIILYCVMPLIELFFNLGLDSHIKPFPYKMV

FPYDPYCSMVYVITYMFTSYAGICVVTTLFAEDSVFGFFITYTCCQFQLLHEKIRCLFMEEIELTANDQELTANDQNEWQ

IYYKRLESIVEKHNKIIRFSKKLEDFFNPILLFNLIISSILICMVGFQIITGKNIFINYIKFLVYIFSAIAQLYILCENG

DTLMSHSSLTAQCLYSCNWESSYTNFQKHISLMIHCSQKPVKITAFKFSTLSLQSFSAILSTSISYFTLLRSL

>DanaOr22aL3__scaffold_12916_residues_14916600_to_14918400_forward_strand.[97_1467].sp

QSRDAFIYLNRGMWVVGWTEPHEKRWTVLYRMWFMLTTLMIIILVPLSMVAEYVQRFRSFGEFLSSLEISVNIYGCSVKC

VATIMGYRKFQEARKILDKLDESCQENAEKVIVRRYVAWGNLVFMIFQVLYSFFVVTNCGGYMLMGSHAWRMYVPFNPNN

NFYMTNLLEFILMNFVVLMEQCTDVCALTYILLGRCHIILLKDRLTRLRDKSEEEHCMELNKCIQDHRLILDYFNVLRPV

FSKTIFIQFLLIGIVLGLSMIYIQFFATFWMGIINLVFMFDVCLETFPFCYVCNLIIDDCQELADSLFQSNWMSADRRYK

STLIYFLHNLQQPIALTAGGVFPICMQTNLSMVKLAFSVVTVIKQF

>DanaOr22aL6__scaffold_12916_residues_14921700_to_14923300_forward_strand.[93_1443].sp

KVSSRDAFVYLDRGMWLVGWTEPLNKRWSLLYKMWSATTTIMLVVLLPIFMVLEYIHNFNTVGEFLSSVEICVNMYGCSF

RSIFTMFGHSRFQVAKKLLDQMDEKCQRSDERTRLRQYVALSNLLFIVYFILYTHYVMLNFTGYLLKGSHAWRIYNPLDT

DENFFSSNMVEFVLMSSVITQALCDDLCPVTYLFVARLHITFLKERLSRLHMDPEKSPEKSEDEHLIDLNNCIRDHRLIL

DYVNALRPVFSKSIFVQFLLIGIVLGLSAINVMFFANFWTGLSTCVFMFDVCLETFPFCYLCDVIMNDCQELADTLFQSN

WMAADRRYKATLVYFLHNLQQPIVLTAGGIFPICMQTNLSMVKLAFSVVTVMKQL

>DanaOr23a__scaffold_12984_residues_64303_to_66461_forward_strand.[501_1659].sp

WAMAFHFLVALAFPMLALSLFSYDSTLENITNFSLTITSLATIVKFGLYTRRLSNLAEMENIIAEMDKRVAGLEQRICHR

LMKKRLHFLSRGFLLISCIVIVSLQMSFLFKDKRSLPYPSWFPLDWTSSWSYIVAVAYQQLVVFVQVFQNYVGDSFPPLA

LFLIAQQCQLLNLRISGIGFQGESLEAELRECIKDQKQLYRLLDLSRSVITWPMFVQFIVIAINVGVTVFGLVFYVETVY

DRIYYVSFLIGLTLETYPLCFYGTNMENSFHELHYAAFRCNWVEQSRSFRSTMLILSERTKMNQLLLAGGLVPIHLSTFQ

ATCKAAYSFFTLMSN

>DanaOr24a__scaffold_12943_residues_4302186_to_4304577_forward_strand.[501_1892].sp

HYFLVPKFVLSLIGLYPEQKRTLPVRLWSFFNFFILSYGCYAEAYYGIHYIPNIATALDALCPVASSILSLVKMIAIWYY

QDELKSLIQRVRFLTEQQRSERKLGYKKKFYVLATRLTTLLLCCGFCTSTSYSVRHLIDNVLRKSRGKEWIYETPFKMIF

PDPLLRLPLYPITYILVHWHGYITVVCFVGADGFFLGFCVYLTVLLMSLRDDVDIEKIPNKKQEAKIVREMEKLVDRHNE

VAELTERLSGVMVEITLAQFVTSSLIIGTSVVDILLFSGLGIIVYVVHTCAVGVEIFLYCLGGSYIMEACSELARSTFSS

HWYVHSVRVQKMALLMIARAQRVLTIKIPFFSPSLETLTSILRFTGSLIAL

>DanaOr30a__scaffold_12943_residues_458346_to_460808_forward_strand.[501_1963].sp

NTLKLMKFWSYLFVHNWRRYMAMAPYMIINSTQYVDIYLSTEPLDFIIRNVYLAVLFTNTVVRGVLLCVQRASYEKFIEM

LKSYYIQLLESDDPINLVEETTRMSNFIGRINLMMGTCTCIGFVTYPLFGSERVLPYGMYLPIDEYKYPFYEVFFVIQAI

MAPMGCCMYIPYTNMVVTFTLFAILMCRVLQHKLRSLEKLKKETQVHREIIWCIRYQLKLSGLVDSMNSLNTHLHLVEFL

CFGAMLCVLLFSLIIAQTIAQTVIVIAYILMIFANSVVLYYVANELFFESFEIAIAAYESNWMDYDVDTQKTLKFLIMRS

QKPLAILVGGTYPMNLKMLQSLLNAIYSFFTLLRRV

>DanaOr33a-2_scaffold_12943_residues_2311525_to_2315692_reverse_strand

RTYWLYLRLLGVEGKYPLRLVVYVLNLFFITFWYPVHLILGLFNKRMVDLFRSLHFTSECFFCSFKFLCFRWKLREIKAI

EKLLKELDQRAESEEDRRFFDQNTRRGAVMLSRCFLGAGISAIITGTAAGLFSSERKLLHPAWYPYDVDATPVFWLTFCY

QVVGSTLAIVQNLSNGIYPPITFCVVAGHVKLLAMRLSRMGHDEKDSKAEVTREDSKAEVTRELVQNIEDHRKLMQIIGL

MRSTLYLSQLGQFISSGINISVTLINILFFAENHFAITYYVVFFVAMLIELFPSCYYGTLMTNEFDKLPYAIFSSKWMEM

NKAYCRSLIILMQFTVAEVEIKAGGIVVIGVNAFFATVRMAYSFFTLGMSL

>DanaOr33b-1_scaffold_12943_residues_2321918_to_2323127_reverse_strand

IYRVYWFYWRLLGLETDFPLRHVLDILITFFVTIWYPIHLIVGVFSERTLGDICKGLPITAACFFCSFKFICFRLKLKEI

KAIEILLKELDQRAVSDDEREYFNKNTQKEANFIWASFLVAYNLSNISAIASVLFGGGHQLLYPGWFPYDVQATLRFWLS

VAYQIAGVSLAIIQNLVNDSYPPMTFCVVAGHVRLLAMRLRRIGHDEKPTKVAVARRKLFESIEDHRKLMKIIDLLRSTM

NISQLGQFMASGINISITLVYILFFAENLFSVTYYAVYFISMVLELFPCCYYGTLISVEMNSLTYAIYSSNWLGMDRKYC

RTLLMFMQLTLTQVQIKAGGVIAIGMNAFFATVRLAYSFFTLAMSLR

>DanaOr33b-2_scaffold_12943_residues_2319070_to_2320279_reverse_strand

YRVYWFYWRLLGLENDFPLRHVLDILITFFVTIWYPIHLIVGVFSERTLGDICKGLPITAACFFCSFKFICFRLKLKEIK

AIEILLKELDQRAVSDDEREYFNKNTQKEANFIWASFLVAYNLSNISAIASVLFGGGHQLLYPGWFPYDVQATLRFWLSV

AYQIAGVSLAIIQNLVNDSYPPMTFCVVAGHVRLLAMRLRRIGHDEKPTKVAVARRKLFESIEDHRKLMKIIDLLRSTMN

ISQLGQFMASGINISITLVYILFFAENLFSVTYYAVYFISMVLELFPCCYYGTLISVEMNSLTYAIYSSNWLGMDRKYCR

TLLMFMQLTLTQVQIKAGGVIAIGMNAFFATVRLAYSFFTLAMSLR

>DanaOr33b-3_scaffold_12943_residues_2310631_to_2311840_reverse_strand

YRVYWFYWRLLGLETDFPLRHVLDILITFFVTIWYPIHLIVGVFSERTLGDICKGLPITAACFFCSFKFICFRLKLKEIK

AIEILLKELDQRAVSDDEREYFNKNTQKEANFIWASFLVAYNLSNISAIASVLFGGGHQLLYPGWFPYDVQATLRFWLSV

AYQIAGVSLAIIQNLVNDSYPPMTFCVVAGHVRLLAMRLRRIGHDEKPTKVAVARKRKLFESIEDHRKLMKIIDLLRSTM

NISQLGQFMASGINISITLVYILFFAENLFSVTYYAVYFISMALELFPCCYYGTLISVEMNSLTYAIYSSNWLGMDSKYC

RTLLMFMQLTLTQVQIKAGGVIAIGMNAFFATVRLAYSFFTLAMSLR

>DanaOr33c__scaffold_12943_residues_2309700_to_2311300_reverse_strand

YRPFWICMKILDPTFFGEPTRLLRVYVVALQFLITFCFPLHLLLNVLLHPSLADLFKNLIMTITCSACSLKHVAQIIHLP

EIKEIDSLITQLDKFIESEDEHEYYRKHVKSNSVRFTRCLYISFGIIYPIFMLGFLVLVASGTGELIIPAYFPFDWKTNY

LYAVAISYQLLGVLVDGLQGLTNDTYTPLTLCILGGHIHMWAMRMSRLGFESDRNQYRRFVAYIEQHKLLMRFHYLTQKT

ISQVQIFQLGGCGGTLCLVVSYVLFYAPDMISLIYNLVFIVVVCVQLFPSCYFASVVAEEVQSLPYAIFSSRWYACSQRH

RRNLLIFTQLTGRGRVMKAGGLVELNLNAFYATLKMAYSLFAVVVRVK

>DanaOr42b__scaffold_13266_residues_19110808_to_19113003_forward_strand.[501_1696].sp

YRAMKFIGWRPPKEGVLRYLYLFWTLMTSTTYLPLGFLGSYMKSFSPGEFLTSLQVCINAYGSSVKVAITYSMLWRLVKA

KDLLDQLDLRCTSMEEREKIHRVVARSNHAFLIFTFVYCGYAGSTYLSSVLSGRPPWQLYNPFDWHDGTKLWMASTLEYI

VMSGAVLQDQLSDTYPLIYTLILRAHMDMLKERIRRLRTDETLSTLSESENYEELVKCVMDHKLILKYCALIKPVISGTI

FTQFLLIGLVLGLTLINVFFFSDIWTGIASFMFIITILLQTFPFCYTCNMIMEDCEALTHAIFQSNWVDANRPYKTTLLY

FLQNVQQPIVFIAGGIFQISMSSNISA

>DanaOr43a__scaffold_13266_residues_3345987_to_3350598_reverse_strand.[1501_3112].sp

SINVRMWRYLAVLYPTPGTDWRKYAFVLPVTAMNFMQFLYLLQSWGDLPAFILNMFFFSAIFNALMRTWLVIIKRVQFEK

FLDQLSVLFHSIRDDSDERNILRQAEDEACHLAILNLSASFLDIVGALISPLFREARAHPFGVALPVNMTRTPVYEIVYL

AQLPTPALLSMMYMPFVSLFAGLAIFGKAMLQILVHRLGQMGGEGQTVEEFRMLCDCIQYHIRVIGYVRRLNDLVTNIVA

AEAIIFGSIICSLLFCLNIITSPTQIISIIMYIFTMLYVLYTYYNRANDLSIENFRVAEAVYNVPWYRGSIRFRKTLLIF

LMQTQHPLEIRVGNVYPMTLAMFQSLLNASYSYFTMLR

>DanaOr43b__scaffold_13266_residues_16134559_to_16138881_reverse_strand.[1501_2823].sp

TLRMSGLNLGEDSGVAWKIWRVISYTYSMVILPLSNYTIHLAEFPPDLLLQSLQLCLNTWCYAFKYFTLVIYTRHLELSN

IQFDEMDKFCVKAEEKRKIRESVAAVNRLYMVFVIVYILYATSSLLDALVHDRVPYNTYFPFNWRIDTQLYIQCFLEYFI

VGYAIFVATATDSYALIFVASLRTHITLLKDRIVSLGQVNSDQDTDTDADSAFRSLVDCVKAHRTMLNFCNAIRPIISGT

IFAQFIICGSILGIIMINMSLFADKSSRFGTVTYIMAVLLQTFPLCFYCNAIVDECNELADALFHSAWWEHDKRYQRVVL

QFLHKLQEPMTFTAMNIFNINLATNINVAKFAFTVYA

>DanaOr45a__scaffold_13266_residues_8335831_to_8340015_reverse_strand.[1501_2685].sp

SYFAVQRRALEIAGFDASSPQLHLKHPIWAGILVLSLISHNWPMAVYALQDLSDLTRLTDNFAVVIQGSLSTFKFLAFVV

KRRRIGALIHQLHQLNQRASESEQQLERIKRENQLDGYVSKAFRNAAYGVIIASAIAPMLMGLLGYIRVGIFRPTTPMEF

NFWLDETQARFFWPIYFWGVLGVAAAAWLAIAADTLFSWLVHNVVVQFQLLELLLKKDQKHKKPHDCQLVTFIHRHRLAL

KLAEDLNHIFAEIVFMQYMLSYLQLCMLAFRFSRSGWSSQVPFRACFLVAVFIQISSYCYGGEYLKQQSLAVSQVVFDCE

WSEMPPGKRLLWKMLIMRSQKPAKISAFMFDVDLPLLLWVARTAGSFLALLRTF

>DanaOr45b__scaffold_13266_residues_174650_to_178292_reverse_strand.[691_2143].sp

LFFVTRYSFGLLGLSFGQKMSWFQLAWLVFNFVNLAHCCQAEFDFGWSHRTSPVDAMDAFCPLACSATTLFKLGWMWWRR

QEVADLINHIRQLTEEQEGRKDSRRDNAQRSTYVMATRSGMLVLTLGSINTGAFVLRSLWEMYARRRQEFKFDMPFRMLF

PDFAHRMPWFPVFYLYSIWSGQVTVYAFAGTDGFFFGFTLYVAFLLQALKSVKESKLCCQQLADIVDRHNEIEKIVERFS

GIMAAPTFVHFVSASVVIATSVIDILLYSGYNIIRYVVYTFTVSSAIFLYCYGGTEMATESLSLGEAAYCSAWYKWDRET

RRRVFLIILRAQRPITVRVPFFAPSLPVFTSVLKFTGSIVA

>DanaOr47a__scaffold_13266_residues_846881_to_849194_forward_strand.[501_1814].sp

QKGTIALLGFDLFGESRKMWLRPYRAVNVFGIATLFPFILAAVLHNIKNVMMMADAMVALLITILGLFKFSMILYLRKDF

KGLIDRFRVMANESRQGEEYAEIIRTANNQDQRVCSIFRTCFLIAWALNSVLPFVRMGLSYWLTGHYEPELPFPCLFPWD

IHILRNYAMSFVFCAFASTGVVLPAVSLDTIFCSFTSNLCAFFKIAQYKVLRFKYQTSLDMCSELNQCYQPIICAQFFIS

SLQLCMLGYLFSITFAQTEGVYYASFIATIIIQAYIYCYCGENLKTESASFEWAIYDSPWHDSSAAICRSLLISMMRAHR

GFRITGYFFEANMEAFSSIVRTAMSYITMLRSF

>DanaOr49a__scaffold_13266_residues_5581229_to_5583587_forward_strand.[501_1859].sp

KKKQRSFEDFSYMAEIMFKSLGYDLMDTSRWRNILMRGYFILCLVSSLYESFFVTYRILEWESPSKVMRQGLHFFYMFSA

QVKFFTFMVYRKKLRVLINNLKDLYPEEEDQRRQYDVNSFYFSRATRSVMYFYYMVMILMAFHPLIQSCIMFAVGLGKSD

FLYLRIFPTQLSFDVRNPKGYILGYIIDLTYSQFIVNVSLGTDLWMMCVSSQISMHFAYLAKVLSSHLPNRERENENRER

ENEDRDFLVSVVRRHQVILRLQKDVNQVFGLLLASNLFTTASLLCCMAFYSVVQGFNLEGISYLMLFLSVAAQFYMVSSY

GQMLIDWSIQIAAAAYEHKWYEGSIRYIKDIAFIMARAQRPAEISARGIIIISFDTFKTLMTITYRFFAVIRQ

>DanaOr49b__scaffold_13266_residues_6198744_to_6201148_forward_strand.[501_1905].sp

IYMNIRILRFWGLLYDKNVRRYICLSLASFLIITQVMYMISTTEGLIGIIRNSYMLVLWINTVLRAYLLLMDHDRYVALI

SNLTKAYYELLSDGYIDEKLTEVNRVGQLMARGNLFFGLLTCLGFGLYPLASAERVLPFGSRIPLNEYATPYYQLWYIFQ

MLITPMGCCMYIPYTSLVVALIMFGIVRCKALQHRLRRKLAAEIVDCIRYQQSIIDYMAQINALTTMVFLFELLAFSALL

CALLFMLIIVNGTSQVIIVCMYINMILAQILALYWYANELREQNLAVAAAAYETEWFTFSFPIRKNILFMMMRAQRPASI

RLGNIRPITLELFQNLLNTTYTFFTVLKR

>DanaOr59a____scaffold_13266_residues_7962974_to_7967173_reverse_strand.[1501_2700].sp

FRSHWTAWRILGLAHHRTKSWRNLYLLYSLVMNVLVTLCYPLHLGMNLFRNGSLTEDILNLTTFATCTACSVKCLIYGYN

IRQVMEMERLLRLLDSRVVGQKQLNIYSQVKVQLRNVLYIFIGIYMPCALFAELMFIFKEERGLMYPAWFPFDWLNSRYY

YMANLYQIGGITFQLIQNYVSDCFPAVALCLISSHVKMLYKRFEEVGEDSEKDSEKDAEKELEACITDHKNLLELFRRVE

AFVSLPMLIQFTVTALNVCIGIAALVFFVTEPMARMYLIFYSMAMPLQIFPTCYFGTDNEYWFGKLHYAAFSCNWHPQNK

SFKRKMMLFVEQALKKSTAVAGGMMRIHLDTFFSTLKGAYSLFTIIIRMR

>DanaOr59b____scaffold_13266_residues_7999841_to_8004148_reverse_strand.[1501_2808].sp

YLYRAMWLIGWIPPKKGILRYVYLFWTCVPFAFGVFYLIISYVKEFKNFGEFLTSLQVCINVYGASVKSTITYIFLWRLK

KTEMLLDALDKRLQNDSDRQKIHDMVARCNYAFLIYSFIYCGYAGSTFLSYALSGRPPWSVYNPFDWRDGISLWIQAIFE

YITMSFAVLQDQLSDTYPLMFTIIFRAHMDVLKDHVRNLRMDPDPERSEAENYQDLVNCVIDHKMILRCCDMIRPIISRT

IFVQFLLIGSVLGLTLINVFFFSNFWKGVASFLFVITILLQTFPFCYTCNLLIDDADDLANTIFQSNWVDAEPRYKATLV

HFMHHVQQPIIFIAGGIFPISMNSNISVAKFAFSIITIVRQM

>DanaOr59c____scaffold_13266_residues_8001563_to_8005833_reverse_strand.[1498_2771].sp

RTAFVMGWLPPKNPAWQRWIYSLWTGTTMLLGLIYLPLGTYVVHFDKFMPNEFLTSLQVDFNCIGNVIKATMTFSQMWRM

QKMNELMAPLDERCVTPSQRQLLHDMVAWVNRMMVFFLAMYLGFSTLNLFTSVFAGKAPWQLYNPIDWRQGWQLWVASIM

EYFVVCIGVMQELLSDTYAIVFISLFRGHLAILRDRIKNLRMDPELSEEENSEEENYKQLVACIQDHRTIVQCTEVIRPL

LSTTIFAQFMLVGIVLGLAAFNILFFPNTFWMILANVSFILAICTETFPCCMLCEYIIEDCSNLTYALFHSNWSTANRRY

KSALIYFLHRVQQPIEFKAGAIFPISVQSNITVAKFAFSIITVVKQM

>DanaOr63a__scaffold_13337_residues_9141173_to_9143967_reverse_strand.[501_2295].sp

KKRNYHRIREMIRLSYTVGFNLMDPSRCGQILRIWTIVLSLSSLASLYGHWHMFRRYIHDIPRIVETVSTAFVFLTSIAK

MWYFLYAHQQIYDLLRRARLPVAKLLRQKVEANMDRCWSSTRRQLLIYLYSCICVTSNYFISSFVTNLYRYFTLPQGSYD

IMLPLPSLYPWERKGPYYHIQMYLETCSLYICGMCAVSFDGVFIVLCLHSLGLIKSLNQMVQYLRCCIIQYQRVASFAEE

INDCYRHITFSQFMLSLFGWGLALFQMSIGSNSSITMIRMTMFLVSAGYQIAVYCYNGQRFTSASEQIGQAFYECEWWTE

SREFRQLIRMMLMRTNRGFRLDVSWFMQMSLPTLMAMVRTSGQYFLLLQNV

>DanaOr65aL1__scaffold_12916_residues_4970956_to_4973306_forward_strand.[501_1955].sp

RAQMKAFGFYMNSEERRKPLLLAWNYFFMFQMMMNMASFTYGLFESLDDIVLLGRDLAFIIAIFFEIFKMIHFSVQVDEI

DKLINGLERCYRWETKGPVRNEVVSIKRWHLLLAAVMGAWLILVISFTFIMISTPFWVESQKLPFHVAYPFDLHKHPAHA

LIFASHGCITFYSLIWVVLADVLAGSIFFELTSDLKILCLQLRFLNMLDKEVNRLALFHQQIICLVDRSNEIFNGALTMQ

LMVIFLLISLSGFEAMVSRHDHEVVAEYLVLMVMALSHLSCWSKFGDLLSQESMEVAVAAYDAYDPTGDKSVHQRLGLII

LRAQKPLSIKSRILPPFTLVNNLAVLNQCYRIFTFLMQT

>DanaOr65aL2__scaffold_12916_residues_4972831_to_4975166_forward_strand.[501_1930].sp

MKAFGFYMNSEAHCKRFLLAWNYFFVAMMSVTMISFCYGLFESLDDIVLLGRDLAFTIAFFFVMFKMIHFWINADKIDQI

IDDLEKFYRWETKSPVQKEILSIKRWHFLVPAALGTWLFLVTSFNLIMVSTPFWVESQKLPFHVAYPFNPSKHPTHALVF

ISQGYITIYSLIWIVFGEFLSGSIYVELTSSLKVLCIELRSLHFDREVNRLALFHQQIICLVDRANQVFNGALTMQLVVN

FLLISLAGFEAMVSRHDSEVVAEYMVLIVMALGHLSCWSKFGDFLSQESMEVAVAAYEAYDPTGNKSVHQRLGLIILRAQ

KPLAIASRFLPPFNLVNNLAVLNQCYRIFTFLMQT

>DanaOr65aL3__scaffold_12916_residues_4974689_to_4977045_forward_strand.[501_1842].sp

HTFAYNRGQMKAMGFYMSAEKRHQPLMVAWHYFFLFQITISLASLGYGILESLDDIVRLGQDMAYTISTFYIYFKMVWFT

IYADDIDEVIESLEKCNRRERKGPGLIRYMKRWIFLLLSLMALWLFLIVSFIMIMISAPFWLESQNLPFHVAYPFDPSKH

PCHIIIFVSQSCIMLYALIWTLFAEQLSVTIYSELTSDLKTLCMELRIVQVYDFAKFHQRIISITDRSNEVFNGAFTMQL

IVNFLLISLSVFEAMVFWHDPKVAAEYLVLMVMALSHLSYWSKFGDMLTQESLEVAVAAYEAYDPTGFRSTHTIIWLIIQ

RAQTPLSVKCRLFPPFNLENNLAVLKQCYGIFNFLLK

>DanaOr67c_scaffold_13337_residues_15153178_to_15155557_reverse_strand.[501_1769].sp

DNTPRTFKDMMRVPVQFYRSIGEDIYAHRSKRSLLLKVYLYAGFINFNVLVIGEIVFFFKSIQNFETIIAVAPCIGFSLV

ADFKQFAMVYHKQTLIRLLDELEDMHPKTLEKQKEYKMSNFEQTMKQVINIFTFLCLAYTTTFSFYPAIKATVKFQLLGY

DTFDRNFGFLIWFPFDATSSLVYWITYWDIAHGAYLAGIAFLCADLLLVVVITQICMHFEYISMRLEEENKEFLIYMIRY

HDKCLKLCEHVNDLYSFSLLLNFLMASMQICFIAFQVTESTVEVIIIYCIFLMTSMVQVFMVCYYGDTLIAASLKVGDAA

YNQKWFQCDKSYCGMLKLLIKRSQKPASIRPPTFPPISLVTYMKVISMSYQFFALLRT

>DanaOr67d-1__scaffold_13337_residues_15189964_to_15193905_forward_strand.[501_1822].sp

SNKKLTPVDRYLRIVRVIRFFVGFCGNDVDPNFKMWWLTYLVLSAIGMFLACTGYIYRGVVIDGDLTVILQAMAMVGSAL

QGLTKLLVTANLAPLLRHIQYGYEDIYREYGAKSEYIKCLERIIKITWRIMISFFCPCLMNLIAVVSFPIFYLVVYKKKI

MVMQFLMPIDEKTDTGYMILSAVHVGLIFFGSFGNYGGDMYLFLFVTNLTLIKDIFCVKLKELNEVVLKKNEYEQMRVML

KKNEYEQMRVMLFDLVTWHQKYQNILLTTRRIYSFVLFVQLSTTCISMLCTIACIFLRVWPAAPIYLLYSATTLYAFCGL

GTAVEISNDVLIREIYSCLWYELPVKEEKIIILMLAKAQTEQHLTAANMCPLSMNTALQLTKGIYSFSMMLLT

>DanaOr67d-2__scaffold_13337_residues_15191800_to_15193600_forward_strand.[372_1606].sp

CARFVGYEVTIPTFKMNWRSYAIIAISVVFVMGSFYIYEGLFIGGDWMVLLQCTCQAGAAFQSIAKIIFYIKDRRFLWEI

LDILDATYEHERCGEHYKRCLQEDFQLIKLWMKGFGILYCIVVLLCVLFPLIYYILYKKKLLMMTFLVPMNPDTTYGYSL

LTSFHFGCMVCGGVGNYAADMYLLFFISSAPVSKNILRCKLHDLDVKLDAKSHNQISSQEIREDMVKIVTWHQKYLRVLE

CSERVFFVIIFVEIVACCVSNLSLLYCILRGDWPSAKVYIFYSISTFMMYCTLGTIVDNSNLDFMDIVYNFRWYDLPLSE

QRIMLFMLRRSQATSGLSVGKMMPLNMQTALQLSKSTYTVLMILLR

>DanaOr69aA____scaffold_13337_residues_16802871_to_16805162_reverse_strand.[501_1792].sp

KLQTVCYYLGSFFMGYQNIGGVVYWCLAKDSIIETAQVFGSLMLTCVGFSKIWCFTRRRHQMELVMAELHDLYPTTRKSF

YRLQHHYDWADLIMKYANLFYFAFYIFYNGSPLVLLLWEYITDDQNLSYKTQANAWYPWKVRGSWGYALAIWVSTMAGNL

GIFLTLAILNILCVCTVQLVMHFDGLATQLLSLDSRLAHQELKYLIRYHRQLIDISDKVNEIFDSIFLTSLICSTLAICM

TSVAVLLLDLAPALKNINGLLAFLVYHFMMSYLGTQINLASEKVLPAAFYNNWYEGDLPYRKMLLILMLRASKPYKWKTI

KLSEVSILNYVDTLKTSYQMYACVRSM

>DanaOr74a_scaffold_13337_residues_8277754_to_8280132_reverse_strand.[501_1879].sp

SWPLEEGSSRWAVWLDKFLIFLGFLVFCEHFHYLIANWQDMDNMLAGMPTYLILVEMQIRSFQLAMHKDQFRVLLQRFYR

EIYVSEKEEPELFSRIQSQMLPTRINSVVYLLALLNFLVVPIQNVVFHRRDMLYKQVYPFDNTWLPVFIPLLVLNFWVGV

IITSMLFGELNVLGELMMHLNARFVQLGQDLRRSASKYRRALTHILRRNAALRDFGEQVEEEFSFRLFIMFLFSAALLCA

LFFKAYTSPINNVAYIVWFLAKFMELLALGMLGSILLQTTDTMGMMYYTADWEQVNRRLMRLLTLAIQLNSKPFFVTGLK

YFRITLTTVLKIIQGAFSYFTFLNSMR

>DanaOr83c_scaffold_13340_residues_18233121_to_18235508_forward_strand.[501_1888].sp

LLGVDILDPVLKFNYRTWTTIFAIINFTVFTLYSMSDNGGDWLVSLKASLMMGGLLHGLGKFLTCIVKQKEMRSLTLFTQ

SIYAEYEKRDIYCSTLDTNIDRIQRIMKGIGYGYVVTNFLMVFTPLAMLVYNDSRMTVMMYEIPLPIHKTFGYALTFLIQ

LVTICVRGFGFYAGDLFVMLGLTQILTFSDILNLKIKDLNSVLSLKAEKERQRLLIEMIKWHQLFTDYCRSVNKLYNPLI

TTQVLAMAYEIMASFCINLGFHSPSAINFVLAAYCMSVYCVMGTKIEFAYDEAYENICNVSWYELSGEQRKIFGMILRES

QSPQNIKLLGVWSLSVRTALQIIKLIYSASMLM

>DanaOr85d__scaffold_13340_residues_4014645_to_4016994_reverse_strand.[501_1850].sp

LLEDFLKYANVFYLSIGMLAYDHKDNSKRKEQLLYWIFIAQMVNLNVVLLIYVFLAISNFLEATMNLSFIGFVVVGDLKI

WHIWRQRAKTTHVVREMEKLHPKRVDQQDEYAIQDYLGSYSRYSIFHFSMHMVLIWTYNLYWAVYWLVCDFWLGIRRFER

MLPYYSWVPWDWSTGNSYYFMYVAQNIGGQACLSGQLAADMLMCALVTILVMHFIRLGRKIEAHVAGFLQAAVVYHQRLL

NLCQDINEIFGVSLLCNFVSSSFIICFVGFHMTIGGKIDNLVMLVLFLFCAMVQVFMITTYAQRLLDASENIGQAVYNQD

WFNADLRYRKMLVLVIKRSQQPSRLKATIFLNVSLVTVSDLLQLSYKFFALLRTM

>DanaOr85e__scaffold_13340_residues_18699561_to_18702841_reverse_strand.[501_2909].sp

MRLQLAMGMVPTPKPWWPKWLRSVYCSIVIFTSLHLGVLFTTLDVLPTGELQAITDALTMTIIYFFTGYGTIYWCVRSRR

LLAYMELINRQYRHHSLAGVTFVSNYESYRLSRNFTAVWILACLLGVITWGVSPLILGIRTLPLLCWYPFDALAPVTYSV

VYATQLFGQILVGLTFGFGGSLFVTLCLLLLGQFDVLYCSLKNLEAHARLLSGDSVKDLSLLQGELLLEDSTNQYAVLKE

HPTDDNVLLEGLKECVRLHRFILHCSIELENLFSPYCLVKSLQITFQLCLLVFVGVSGTREVLRIVNQLQYLGLTVFELM

MFTYCGELLSRHSIRSGDAFWRSGWWKYARHIRQDILIFLVNSRRAVHVTAGKFYVMDVNRLRSVITQAFSFLTLLQK

>DanaOr85f__scaffold_13340_residues_4823200_to_4825544_reverse_strand.[501_1786].sp

EDFAKMPSSLFRLMGYDMLETNRQQKIMMQVYRALCLGSHCVCLGFMIFRMLETINSVSLIMRYATLVTYVVNSDTKYGT

LLQRGAIQSLNNKLAAMYPKTTLDRIYYRVNDHFWSRALSSMIMFYVGSSIMVVVGPIFTSLWSYFFHEQFSYMHCYPYF

IFDPEKHWIYLGIYALEWLHSTQMVISNIAADTWLIFFQVQISMHFRAMIKSIEHFLSKIVDKHDYLVNLQKDLNSIFGG

SLLLSLLSTASVLCTVSVYTLIQGLTLEGVTYVIFISTSVVQMYLVCYHGQQVLDLSAFISHAVYNHNFHNASLSYKKFL

LIIIIRSQKPVELNSMGYLSISLETF

>DanaOr88a__scaffold_13340_residues_20539610_to_20541965_reverse_strand.[501_1856].sp

NTSMLAFLLSAFWNVVFFGFNGWNVLEHLTQNPPIFSITVYFSIRGLMLYLKRKDIIDFVNDLDREWPQNIASQLEKNMD

QTYLSFCQRYKFVQIFSMVGLPMFCSLPVGLFVLTHEGNEVPVSVNEQLLGGWLPFGIRQIPYYFFVWTFDVICTICGVS

FFL

>DanaOr92a__scaffold_13117_residues_595500_to_596900_reverse_strand.[10_1348].sp

KEKREVRTFEDLTRFPMAFYKTIGEDLYSDRDPRRYLLRLYLVLGFLNFNAYVVGEIAYFIVHITTLLEATAVAPCIGFS

FMADFKQFGLTVNRRRLVRLLDDLKAIFPTELEQQRAYHVSYYQKHMNSVMTLFTILCMSYTSSFSFYPAIKSTIKYYFM

GSEIFERNYGFHIQFPYDAETDTVYWFSYWGLAHCAYVAGVSYVCVDLLLITTITQLTMHFSYMADCLEAYEENIKYLHD

LVVYHARALDLSEEVNSIFSFLILWNFIAASLVICFAGFQITASNVEDIVLYFIFFSASLVQVFVVCYYGDEMISSSSRI

GHAAFNQNWLPCSSKYKRILKYIIMRSQKPASIRPPTFPPISFNTFMKVISMSYQFFALLRT

>DanaOr94a__scaffold_13340_residues_12509575_to_12511840_reverse_strand.[501_1766].sp

NKQDRIRSMNLILRVMQLFGLWPWSLDKEEQFLKRNYRYLLHLPITFTFIGLMWLEALVSSNLEQAGQVLYMSITEMALV

VKILSIWHHRTAAWRLMEELKISPDYALRSQIEVDFWRKEQSFFKRFFYIYILISLGVVYSGCTGVLFLDYYELPFAYYV

PFEWKNKRGFRLAYGYDIAGMTLTCISNITLDTLGCYFLFHISLLYRLLGFRLRNLRDIQDDDDIDFGQKLRNIFQLHQN

IKRLSLTCQKIVSPYILSQIILSALIICFSGYRLQHVRANPGQFIAMLQFVSVMILQIYLPCYYGNEITIHANQLTNEVY

HTNWLHCRPPIRKLLNSYMEYLKRPVNVRAGNFFAVGLPIFVKTINNAYSFLALLLNV

>DanaOr94b__scaffold_13340_residues_12507490_to_12509703_reverse_strand.[501_1714].sp

ISAMQLLIVVQRWIGLWPNERGSWLKRIYPLFLHLPFTLTYIALMWLEAITSSDFEEAGQVLYMSVTELALVVKLINIWY

RREHADKFIRDLEYDPAYALRNSDEIRFWQAHQKGFKRIFYWYIGGSLFVALMGYVSVFFQEEYELPFAYFVPFEWRTRE

RYFYAWSYNVVAMTLCCLSNILLDTLGCYFMFQIALTFQLMSLRLQSLKDVPEQKAKPELRRLFQLHAPELRRLFQLHAK

VRILTRECELLVSPYVLSQVVFSAFIICFSAYRLVHMSQRPGLFVTTVQFVAVMIVQIYLPCYYGNEITIHANALTNSVF

GTNWLEYSEGTRKLLNCYLEFLKNPVKVRAGVFFEIGLPIFVKTINNAYSFFALLLKV

>DanaOr98a-2__scaffold_13340_residues_19024762_to_19027055_reverse_strand.[501_1785].sp

FRYFEYAMFWMGWTSKKFKVLYRIVSVFITAWVIYLPIGMLINLIIDTKTPKELLNTLQIFFNGLGTPIKVFFFRIYFWR

FYKVKKILHEMDKRCQTEEEQIEVHRWVVLCNKVYLGYQAMYTGYSLTSFLSAILTGQLIGIYNPFDWRKSQSFWLAALH

ENALIMFSANHTMMSDIYPLLYALILKVHINLLRLRVKKLCEDPHKSDDKSDDENHRDLIKCIQDHRLLKQYADLIRPVI

GSTIFVQFLLIGILLGLSMINLLLFADIWFGLSAAVYIMGLLLQTFPFCYVCDLIRYDCGRLSEAVFHSNWLTSSQKYKR

TLRFFLQNSQKSIAFIAGNIFPISTSTNISVAKLAFTVATFLKQL

>DanaOr98a-3__scaffold_13340_residues_19022903_to_19025152_reverse_strand.[501_1690].sp

EAMHYFEVAMFWVGWAQPTKYQLLYRISTFICLFSTVYLSVGMLISFIFDTDNLSELLTILQLLFNAMGLPIKVLFLRLH

FSRFSDKVKDILGRLDESCSNAEEKREVHRWAVRCNAAYLIYQAIYSSYAISTFFASAFVGTLPWRVYNPFDWRKSWNVW

KAGLIEFVIAIFGITQGLMSDVYALSYALILRAHLKLLRLRVDKLCSDPGKSEEEEEENLGDLIKCIKDHKLINKYYVTI

RPVISGTIFVQFLLIGLCLGLSLINILFSTDIWAALATINYINGLIVQTFPFCYVCDLIKSDCELLERAIFHSNWLEASP

RYKKTLIFFLQRSQKPLAFTAGSIFPISTRT

>DanaOr98b__scaffold_13340_residues_16984316_to_16986520_forward_strand.[501_1705].sp

NFLRLQSIFFSFLGFELQQDKEVSHRYPWRIIFFLLSVATFLPLTIALGISNIQNVEHLTDALCSALVDLVAIFKIGFLL

WLYADLRHLVKRFRCKLQSEGQYGDCEAIILAYNKRDLFISALYCLCFLLAGVSACLMPLLSIFIIYLRTGEVQPELPFP

SVYPWDNKRPLNYLISYLWSVSAAVGVALPTVCVDTLFCALTHNLTALFEIAQLKLMNFMGKSLTRENLVHAFQLYGECL

ELGQSLNGYFRPLIFAKFVVASLHLCVLCFVLSTNLMEPAMLFYVAILGQVSIYCFCGSSVKEESQQFAQAIYESSWQQL

DVKVARSLQFAMMRAQRGCRIDGYFFEANRQTLILIVRSAISSVALLRSL

>DbiaOr7a

ESREAFRNLFNCFYALGMQAPDGPTKSSTWRRIYRCFSVVMYIWQLLVVISYRYMGGMEITQVLTSAQVAIDAVILPAKI

VALAWNLPLLRRAELHLAALDARCRDREEFQLIVEAVRFCNRLVWFYQICYAIYSSSTFVCAFLLGQPPYALYLPLDWQR

SLQFCIQAWIEFIIMNWTCLHQASDDVYAVIYLYVVRMQVQLLARRVERLGRDAKEIFPDERRQEEHCEELQRCIVDHQT

VLQLLGCISPVISRTIFVQFLITAAIMGTTMINIFIFANTNTKIASIIYLMAVTLQTAPCCYQATSLMLDNEKLALAIFQ

CQWLGQSARFRKMLLYYLHRAQQPITLTAMKLFPINLATYFQIAKFSFSLYTLIKGM

>DbiaOr9a

QRQDQSLRIQILVYRCMGIDLWSPTRANDRTWLTLVTMGPLFLFMLPMFLAAHEYITQVSQLSDTLGSTFASMLTLVKFL

LFCYHRRKFVGLIYHIRGILSAEIRPDARAIVDVENESDQMLSLTYTRCFGLAGIFAAIKPFVGIVVSLIRGDEIHLELP

HNGVYPYDLQVVVWYVPTYLWNVMASYSAVTMALCVDSLLFFFTYNVCAIFKIAKHRMIHLLVQVLLLHQKGLRIADHIA

DKYRPLIFLQFFLSALQICFIGFQVADLFPNPQSLYFIAFVGSLLIALFIYSKCGENIKSASLDFGNGLYESNWTDFSPP

TKRALLIAAMRAQRPCQMKGYFFEASMATFSAV

>DbiaOr19a

VDSTKALVNHWRIFRVSGLHPPAKSTLWGRHYRAYSIAWNVVFRFCVWLSFSVNFLQSKSLETFCESLCVALPDTLYMLK

SLNFYLNRGEMLHSHRMLRHLDRRLGCSDDVRIVAEGVAGAEAIFRILGRCVVGILCLGIVYIIMASEPTLMYPSWIPWN

WKDSTAFLMAVIPHTFGLMVTAMEVLNLTTYPCTYLILVSAHTKALAWRVARLGHHEVQQLLVGYIQDHQVILRLVESLQ

RSLSMTCSMQFLSTACGQCTICYFLLFGQVGVMRFTNMLALLLAFTTDTLLLCYMAEQLCQEGESLLVAVYNCNWLDQPV

QFRRLHLLMLKRCQKLPILMAGKIMPISMKTFMIKGAYTMLTLLNEMR

>DbiaOr19b

VDSTKALVNHWRIFRVSGLHPPAKSTLWGRHYRAYSIAWNVVFRFCVWLSFSVNFLQSKSLETFCESLCVALPDTLYMLK

SLNFYLNRGEMLHSHRMLRHLDRRLGCSDDVRIVAEGVAGAEAIFRILGRCVVGILCLGIVYIIMASEPTLMYPSWIPWN

WKDSTAFLMAVIPHTFGLMVTAMEVLNLTTYPCTYLILVSAHTKALAWRVARLGHHEVQQLLVGYIQDHQVILRLVESLQ

RSLSMTCSMQFLSTACGQCTICYFLLFGQVGVMRFTNMLALLLAFTTDTLLLCYMAEQLCQEGESLLVAVYNCNWLDQPV

QFRRLHLLMLKRCQKLPILMAGKIMPISMKTFXXMIKGAYTMLTLLNEMR

>DbiaOr22a

SQDAFIYLDRVMWSMGWAEPENKKWRLLYNVWATFMVALMFILLPISITIEYVHTFLSSLENGVNVYGSSFKSAFTMIGY

KKRQDAKLIMDLLDKRCSRDEERSSVHRFVALANFIDIMYHIFYSTFVVMNFPYFLLKGRHAWRMYYPINCDEQFYISSI

AECLLMTWCIYMDLCTDVCPLIFMLMARCHIKLLKDRLRNLRSEPGKIEYHRLILEYVNALRPVFSGTIFVQFLXXXXXF

FSTFWTGVATCLFMFDASMKSFPFCYLCNMIIDDCQDLANGLFHSNWTSASRRYKSTLIYFLQNLQQPITLTAGGVFPIC

MQTNLAMVKLAFSVVTTIKQF

>DbiaOr22b1

RDAFIYLERVMWTLGWTEPYEKRWAVVYKVFSFMTSIGFFILLCVEYVNRFKSAGEFLSSLEIGVNMCGCSFKNTYTLLG

RRKVRKAKLILDRLDERCLRDEEKSLVHRYVALGNFSYTVYHTCYWVFVITNFIGFLLIGHHAWRMYIPLKSDEHFLISS

VAEFLLMLYVVTMAQCSDVCPTISMLMARCHIILLRNRMMRLRSDPEKDDEDLEELTKCIQDHRLILEYVNTLRPVFSGV

IFVQFLLIGVVLGLSMINVMFFLSFWTGLGTCLFMFDVCMETFPFCYLCNIIIDDCQDLADSLFQSNWTSASRRYKSTLV

YFLHNLQQPIILTAGGIFPICMQTNLSMVKLAFSVVTIIKQF

>DbiaOr22c

FYRIPRISGRLLGLWPQRIGGGRPWHAHLLFVFAFAVVLVGAIGEVSYGCVHLDNLVVALEAFCPGTTKAVCVLKLWVFF

RSNRRWAELLQRLRTMLWQSRREESQRMLVGLATTANRLSLLLLASGTATNTAFNLQPLIMGLYRWMSDLPGQVELPFNI

MLPFAVQPGVFPFTYVLLTASGACTVFTFSFVDGFFVCSCLYICGVFRLVQQDIRRIFADTEAMNAQVRGKLAEVVERHN

AIIDFCTDLTRQFTVIVLMHFLSAAFVLCSTILDIMLNTSSLSGLTYICYIIAALTQLFLYCFGGNHVGESSAAVADVLY

DIEWYKCDARTRKVILMILRRSQRAKTIAVPFFTPSLPALGSILSTAGSYITLLKT

>DbiaOr23a

DYFRYQSQAWRICGALHLSEGNYFSWATVSCIIMFLPGPLLLGALLSYDDPLENNYNLCLTIITMSNFLKFSMYVTQLTK

LVDIQKLIVKLDARVSGKDQIVRREEMSNHIQRLSQLFLITYLILSVGAGVLFIFERERSLPFPMWFPFDWRNSTAYFGA

LVFQQIALLFQDLQNYPCDSFPPIALFLVSEQCQLLIGRISKIGYGSTDVKKNEVKKNEQDLVHCIRDQNKLYRXXXXXR

TMISLPMMIQFLVIGINIAVVLFGLIFYVDTLSDSVFYVCFLSAITLQIYPACYYGTKVQESFAELHYAVFCSNWVDQSV

TFRGLMLIMGERTKAHQILLAGNLVPIHLSTFVACCKGAYSYFTLL

>DbiaOr24a

RHYFAVPKFALSLIGFYPEQKRTLLVKLWSFFNFFILTYGCYAEAYYGIHYIPNIATALDALCPVASSILSLVKMVAIWW

YRDELKSLIERVRFLTEQQRSKRKLGYKMRFYTLATRLTFLLLCCGFSTSTSYSVRHLMDNILRRAHGKDWVYETPFKMM

XXXXXTYILVHWHGYITVVCFVGADGFFLGFCLYFTVLLLCLRDDVGDLLEERSPSEEQEARIVREMEKLVDRHNEVAEL

TERLSGVMVEITLAHFVTSSLIIGTSVVDILLFSGLGIIVYVVYTCAVGVEIFLYCLGGSHIMEACSDLARYTFASHWYG

HSVRVQKMTLLMVARAQKVLTIKIPFFSPSLETLTSV

>DbiaOr33a

WLYWRLLGVEGDYPFRWLLDLAITFFITTWYNVHLILGLYNKPAVEVLRNLYFTTECIFCSFKFICFRWKLSDIREIEGY

LQDLDSRADNEEERRYFNRSPRSVAQTLSKSYLVAAISSIITAIAAGLFSSGRNLMYAGWFPYDVQASLVFWISFTYQAV

GSSMLILENLANDSYPPITFCVVTGHVRLLAMRLSRIGQDEKISMAENKRKNKRKLIEGIKDHRKLMRIISLLRRILHLT

QLAQFLSSGINIAITLINILFFAENKFSMTYYAVFFAAMFIELFPSCYYGTLMKMEFDKLPYAIFSSNWIKKDNDYKRSL

IILMQFMIAPVDIKAGGIVGIDMSAFFATCRLAYSFFTLALSFR

>DbiaOr33b

WRLLGLESQFVLNRLLDVLITVFVTFWYPIHLILGLFLERTLGDVCKGLPITAACFFASFKFVCFRLKLEDIKTIEVLLK

ELDQRALSQGERQFFNQNTKREANLIWKSFIVAYGLSNVSAIASVLFGGGHKLLYPAWFPYDVQASLVFWLSVTYQIVGV

SLAIVQNLANDSYPPMTFCVVAGHVRLLSMRLSRIGQDRKDTTKAVIGKQLVESIEDHRKIVKIVDLLRSTMNMSQLGQF

ISSGVNISITLVNILFFAENNFAVTYYGVYFVSMVLELFPCCYYGTLISVEMSRLTYAIYSSNWIGMDRGHCRTMLIFMQ

LTLTEVQIKAGGMIGIGMNAFFATVRLAYSFFTLAMSLR

>DbiaOr42b

YRAMKFIGWLPPKKGVLRYLYLTWTLMTFVWCTTYLPLGFLGSYMKSFSPGEFLTSLQVCINAYGSSVKVAITYSMLWRL

IKAKGLLDQLDLRCTTMEEREKIHRVVARSNHAFLIFTFVYCGYAGSTYLSSVLSGRPPWQLYNPFDWHDGLMLWVASTL

EYVVMSGAVLQDQLSDTYPLIYTLILRAHLDMLRERIRRLRSDETLEAESYEELVKCVMDHKLILRYCSIIKPVISGTIF

TQFLLIGLVLGLTLINVFFFSDIWTGIASFMFVITILLQTFPFCYTCNLIMEDCESLTHAIFQSNWVDASRRYKTTLLYF

LQNVQQPIVFIAGGIFQISMSSNISVAKFAFSVITITKQM

>DbiaOr45a

SYFAVQRRALEIVGFDPGNPHLRLKHPIWAGILILALISHNWPMAVYALQDLSDLTRLTDNFAVFLQGTLSTFKFLAMMV

KRRRIGSLIHRLHKLNQEASATPSQREKLERENQLDRYVSRSFRNAAHGVIWASAIAPMLLGLWRYIISGEFTPTTPMDF

NFWLDERQPKFFWPIYFWGVLGVSAAAWLAIATDTLFSWLIHNVVVQFQLLELQLAHLTKCIFQHRLALDLAGELSSIFA

EIVFVKYMLSYLQLCMLAFRFSRSGWSAQVPFRATFLLAVIIQLSTYCYGGEYLKQQSLGIAEAVYNSNWPEVPPKRRRL

WQMMIMRAQRPATIFGFMFVVDLPLLLQVTKTAGSFLALLRTF

>DbiaOr45b

FFVTRYSFGLLGLRFGGQLSWLQLSWLVFNFVNLAHCCQAEFVFGWSHLRSPVDAMDAFCPLACSFTTLFKLGWMWWRRQ

EVFELMERIKLLIRQQEMRKDPRRDVAQRSYYLMVTRSGMLVFTMGSITTGAFVLRSLYEMWARRRQEFKYDMPFRMLFP

EFAHRMPWFPIFYLYSTWSGQVTVYAFAGTDGFFFGFTLYMAFLLQALRYDVQDAHICCHRLADIVDRHNEIEKIVEVFS

GIMAFPTFVHFVSASLVIATSVIDILLYSGYNIIRYVVYTCTVSSAIFLYCYGGTEMSTESLSLGEAAYGSAWYKWDRET

RRRVFLIILRAQRPITVRVPFFAPSLPVFTSVIKFTGSIVA

>DbiaOr46aB

DDFYKYQVWYFQILGIWQLPTTDQQRRLQSMRFYLILVILCIMLLLFALELLNNISQVREILKVFFMFATEISCMTKLLH

LKLESRKLGGLVEMMMSQDFAAKTEQERSIMESARRAVVHMRNFYGITSWATASLILLVPCFANYEELPLAMFEWCSIQG

RICYGMQYLFHSISLLPTCVLNITYDSVAFSLLCFLKVQLQMLVLRLEKLGNEKIARELRECAAYYNKIVQFKNLVELFI

KVPGSVQLMCSVLVLVSNLYDMSTMNGDAIFMAKTCIYQLVMLWQIFIVCYASNDVTVQSSRLCHGIYSAQWTGWNRSNR

RILLLMMQRFNSPMVLTFNPTFVFSLEAFGSIVNCSYSYFALLKRV

>DbiaOr47a

SFLQVQKSTIALLGFDLFSADREMWKRPYRAVNVLAIASIFPFILAAVLHNLKHVMLLADAMVALLITILGLFKFSMILY

LRRDFKRLIDKFRLLMANAKQGEEYAEILNVANKQDQRMCTLFRTCFFLAWALNSVLPFVRMSFSYWLSGHAEPELPFPC

LFPWDIHIISYYAPSFIWSTFSSTAVVLPAVSLDTIFCSFTSNLCAFFKIAQYKVVRFKGGSLQESQATLNKVFALYQTS

LDMCNDLNLCYQPIICAQFFISSLQLCMLGYLFTITFAQTEGVYYASFIATIIIQAYIYCYCGENLKTESASFEWAIYDS

PWHENSASICRSLLISMMRAHRGFRITGYFFEANMEAFSSV

>DbiaOr47b

AFNYVRACLSLLCQYPNKKLAKLSLYRWLNWFILCNVMNAFWTMVLALPESQNVIEMGDDLVWISGMALVFTKIFYMHFR

CDEIDELVWDFDYYNRELRPYNDDEEVLGWQRLCYLVESGLYINCFCLVNFFSAAVCLQPLITDGKLPFHSIWPFQWHHP

HMFWFLYIWLSLTSQHNLMSILMVDMCGITTFLQTALNLKLLCIEIKKLGDMARFHEEFCRVVRFHQHIIKVVGKANRAF

NGAFNAQLVASFALISISTFETMVASVDPKMAAKFVLLMVVAFTQLSLWCISGTLVYTQSLEVAQAAFDIDWHTKAPAIQ

RDIGFVICRAQKPLMYVAEPFLPFTLGTYM

>DbiaOr49a

EKQRSYEDYTYMANVMFKTLGYDLFDSPRWQNLLLRSYFVLCVVSNFYEASMVTLRILQWESPSKIMRQGLHFFYMFSAQ

VKFVTFMTYRKRLRLLSNNLKEIYPHDEQKQREYGVNRFYLSRTTRYVLGLYYFTMVLMALGPLIQSCIVYLIGFGKAEF

PYLRIFPTRLSFDSESPLGYVAGYVIDFTYSQFIVNVSLGTDLWMMCISSQISMHFAYLAKLIETEEQDCNFLAIVVTRH

QLILRYHKDVNIVFGLLLASNLFTTASLLCCMAYYTVVQGFNMEGISYMMLFVSVAAQFYMVSSHGQMLIDXSTKIAETA

YESRWFEGSLRYQKKILFLMGRAQRPAEISARGIIIISLDTFKILMTITYRFFAVIRQ

>DbiaOr49b1

YMNIKILRFWALLYDQNIRRYVCIGLASFHIFTQMVYMVSTNEGLTGIIRNSYMVVLWINTVMRAYLLLADHDKYVALIQ

KFTAAYYDLLNLNDISKILFNLNRVGHLMARGNLFFGMLTSMGFGLYPLSSSERVLPFGSRIPLNEYESPYYEMWYVFQM

LITPMGCCMYIPYTSLIVGLIMFGIVRCKALQHRLNQDRDSRLLREEVIACIQYQQSIIEYMDRINELTTMMFLFELMAF

SALLCALLFMLIIVSGTSQLIIVCMYINMILAQILAIYWYANELKEQNLAVATAAYATEWYTFDVPLRKNILFMMMRAQR

PAAILLGNVRPITLELFQNLLNTTYTFFTVLKR

>DbiaOr56a

FRTHLRCFRWYGYVASTDQKRPWISLLRCTVFTASIWLSCTLMLARVFRGYENLNDGATSWATAVQYFTVSIATANAYVQ

REXVIYLLRVAYEDIQNLMFDNREMDLLMDTQVYTRTITLVLWVPSVIAGLIAYSDCIYLPKSVFNMAAVRRGEERPILL

FQLFPFELCDNFVIGYLGPWYALALGITTIPLWHTFITCLMKYVNLKLQILNKRVEEMDITFKEFVDEQLRLRKFIQELQ

YLICVPVMVDFIIFSVLICFLFFALTAGVPGKMDYFFMFIYLFVMAGILWIYHWHATLIVECHDELSLAYFSCGWYDFDI

SLQRMLVFVMMHAQRPMRMRALLVDLNLRTFIDIVRGAYSYFNLLR

>DbiaOr65a

RSFWHTLVAIKSFVFMATMLYGLTESIGDNVQMGRDLAFIIQIFYITFKIFYFLRYGDALDEVVNDLEAFHPWAQQGPHA

VDYRSGKRWYFVLAFFVSTSWALFLVIFLGLLVTSPMWVQNQNLPFHAAFPFQWHTHPTHAIIYLFQCYFAAYALTWLLC

MEGLSISIYVEITFAIEVLCLELLSLHREQLRLETNRLVRLHQKVIRILDRINDVFHGTLIMQMGVNFSLVSLSVLEAME

ARKDPKVVAQFAVLMLLALGHLSMWSLFGDLLSQTSLKVSEAAYEAYDPTGSKEVYRDLCLIIRRAQDPLIMRASPFPSF

NFINYTAILNECFGILTFLLN

>DbiaOr65b

YRSFWHTLVAIKSFVFMATMLYGLTESIGDNVQMGRDLAFIIGVFYITFKIFYFLRYGDALDEVVNDLEAFHPWAQQGPH

AVDYRSGKRWYFVLAFFVSTSWALFLVIFLGLLVTSPMWVQNQNLPFHAAFPFQWHTHPTHAIIYLFQCYFAAYALTWLL

CMEGLSISIYVEITFAIEVLCLELLSLHRRQLRLETNRLVRLHQKVVEILDRINDVFHGTLIMQMGVNFSLVSLSVLEAM

EARKDPKVVAQFAVLMLLALGHLSMWSLFGDLLSQTSLKVSEAAYEAYDPTGSKEVYRDLCLIIRRAQDPLIMRASPFPS

FNFINYTAILNECFGILTFLLN

>DbiaOr65c

YRSFWHTLVAIKSFVFMATMLYGLTESIGDNVQMGRDLAFIIGIFYITFKIFYFLRYGDALDEVVNDLEAFHPWAQQGPH

AVDYRSGKRWYFVLAFFVSTSWALFLVIFLGLLVTSPMWVQNQNLPFHAAFPFQWHTHPTHAIIYLFQCYFAAYALTWLL

CMEGLSISIYVEITFAIEVLCLELLSLHRRQLRLETNRLVRLHQKVVEILDRINDVFHGTLIMQMGVNFSLVSLSVLEAM

EARKDPKVVAQFAVLMLLALGHLSMWSLFGDLLSQTSLKVSEAAYEAYDPTGSKEVYRDLCLIIRRAQDPLIMRASPFPS

FNFINYTAILNECFGILTFLLN

>DbiaOr67a

EVNDFLRLPVIFYNSVGLDPYESEKKPGILFHMYFAFQISNMTFVLAMEILFVVVNNENFLDSCMVMSYIGFVTVGGLKF

VSVLIRKRKMSNLVRQLESFFPEPKDHEKYSVKFFLKKCHRYTKGFGGLYTTLVVVYNLFALSQYSILHLLDSPNAKQVL

PYVDMAPWDYQANWRFYLTYLSQSMAGYMATCGHISGDLMIFAVAMQVIMHFNRLARALKEFQVQEDLRELRHLISYHNE

VLRLTDVMNEVFGIPLLLNFAASSLLVCFVGFQMTVGISPEQFIKLMLILVSALVEIYLLCSFSQMLIDASESVSFAAWD

MNWTKADTRFRKMLIFIALRAQRPVCLKATVFLDVSIQTMSSFLQMSYKFFCAIRTM

>DbiaOr67a2

EVFDVRAFLRLPVMFYSSLGIVPYETAQRPGLWFHLYFVLLMTNCAITGTTSTMISFRKNNDVLEGCILCGYITFTVTGV

LKIIAVVMQKKKMTALVRRLEACSAPFTQVEQEQCFVKSYLKRCHLFTKGFAILLMAMLFTHSLSAIVTYAFQRLWLQSL

DAKQSLPFFNYALWEWRGSWIFYITYLSQSASGYTATCGNMSCDLMIFGMVFQVTMFFDRLSRALREFRTEELRSLVAYH

IKILRLPDLMNAVFGVPLLLNFLASSWLVCLLGFQLTLAFSPEHFCKQVLLLVSALVEIYLLCSFSQMLINAENVRLAVY

EMNWTEFGPRLRKMLILLSVGAQKPICLNGTVILNVSIETMSIFLRVSYKFFCAV

>DbiaOr67b

LGVNIAPRKRSSKYCRVRFVVLIVNLSIIYSLIAFIMEYMISFETYVEAVLLTFQLSVGVVKMFHFQSKVDSCSKLVFST

EDLPKKRELLRAVSLILLDNWLIIDRQVMFFFKIVCMPVLYYTMRPYFQYIYDCYIVCEMTLSYPAIVPYGKFELPSYVI

RFFLLQSGPLWCFFAVFGFNSLFVVLTRYESGLIKVLRFLVQNSTSDVLVPRDLRVKYLQCCVMGMIMVYFVTIALEISL

YNVSAQKVETQSELLFYDWYNCSWYNESKEFKFMIKMMLLFSRRTLVLSVGGFTSLSHKFLVQVFRLSANFFLLLRNM

>DbiaOr67c

EENSARTFMELMRVPVQFYRSIGEDIYAHNPLKSLLLKIYLYAGFINFNLLVIGELVFFYNSIQDFETIIAVAPCIGFSL

VADFKQFAMVKGKKTLIMLLDDLEEMHPKSLKKQKAYRLPDFEKTMKRVINIFTFLCLAYTTTFSFYPAIKASVKFNFLG

YETFDRNFGFLIWFPFDATSSLVYWIMYWDIAHGAYLAGIAFLCADLLLVVVITQICMHFNYISMRLEEKPEEDTENKEF

LIGIIRYHDKCLKLCEHVNDLYSFSLLLNFLMASMQICFIAFQVTESTVEVIIIYCIFLMTSMVQVFMVCYYGDSLIAAS

LRVGDAAYNQKWFQCSKTYCSMLKLLIMRSQKPASIRPPTFPPISLVTYMKVISMSYQFFALLRT

>DbiaOr67d

EQYRKIIRMIRFCVGFCGNDVADPNFRMWWLTYTVIVAIGFFFACTGYTIYVVVIDGDLTVILQAFAMVGSAVQGLTKLL

VTANMASEMREIQNTYEKIYREYGPRGEYAKCLENRIRTTWRLIIGFMVGYIILLGLIIGFPIFYLLVWQEKVLVMQFLM

PLDHTTDGGHLVLTAVHVALIVFGGFGNYGGDMYFFLFVTHVPLIKNIFCVKLEELNEVVVKGNERMRAMLCDLFTWHQL

YSRILQTTKKIYSIVLFVQLSTTCVGLLCTISCIFIKAWPAAPLYLIFAAITLYAFCGLGTLVENSNEDFLSIIYNCLWY

ELPGTEEKLIILMIAKAQKEVCLTAADMAPLSMNTALQLTKGIYSFSMMLMN

>DbiaOr69aB

ENNRNLAKRMIFWFGAVNLVYHNFGCIMYAYYADRSIAELASVGAMLGFTIVGTLNLWKLWTLKPDIEKLMDDFEEMFQL

TKRRPYRSHHYYESYTRFIRNLLIFFTLTIAYYNALPIILMTRELLKESQKLSYRLQSSTWYPWQFQGSPGFLVAVVCQG

FSCQVNLCVLIFSQFLVSFFGIQLEIHFDGLARQLEAIDARHKDQLKSLIRYQKQIFIMADRVNEIFNFTFLISLSISVT

CTSSLAFSVTMFDLGPALKHTFGLLIFLIYNFCMCRNGTHLILQSDKVLPAAFYNNWYEGDLAYRKMLLILMMRATKPYI

WRTYKLAPVSITTYMATLKFSYQMFTCVRSLK

>DbiaOr71a

RPVRYLTGILEWWRLWPKKGRPNWTNWRGYLLHIPFTMLFMVLLWVEAMMSRDIHHTADVLLIGLTTTALGAKMLNNWKY

AHVAQGILNEWSSDLFELKSKQEVDMWKFEHRRYSRVFIFYVMCSAGVIPFIVIQPLFDIPNRLPFWMWIPFDWHQPNLF

WYPFIYEAITIPIVCICNITMDGVNWYMMLHLSLCLRMLGQRLSSLRHDDRKLREDDRKLREKFLELVHVHRRLKRQALD

IEDFISKSTFTQILVSSLIICFTIYSMQMSMQDLPGFAAMIQYLLAMIMQIMLPSIYGNAVIDSANMLTDCMYNSDWPDM

NPRIRRLILMFMVYLNRPMTLKAGGFFHVGLPLFTKVVE

>DbiaOr74a

AWPLEAGRWTVFLDRVLIFLGFLVFCEHNFHYLIANWQDMDNLLTGMPTYLILVEMQIRCFQLAWHKDRFRGLLQRFYEE

IYVAEESEPHLFARIQRQMRATRLNSTVYLLALFNFLLVPVTNVIYHRREMLYKQVYPFDNTKLQFFIPLLGLNFWVGFV

ITSMLFGELNVMGELMMHLNARYVQLGQDLRRSSSYRRALTHVLRRNAALRNFGQRMEEEFSLRIFVMFAFSAGLLCALF

FKAFTNPWGNVAYIVWFLAKFMELLALGMLGSILLKTTDELGMMYYTADWEQVNVKLMKLVTLAIQLNSRPFFITGLNYF

RVSLTAVLK

>DbiaOr83a

VRQTMCIAAMFPFGYYVKGSLLYELFNYFVSVHIAGLFICTIYLNYGQGDLDFFVNCLIQTIIYLWTIMKLYFRRFRPGL

LNAILSNINDEYEPRSAVGFSYVTMAGSYRMSRLWIKTYVYCCYIGTIFWLALPIAYRDKSLPLACWYPFDYTQPIVYEV

VFFLQAMGQIQVAASFASSSGLHMVLCVLISGQYDVLFCSLKNVLATSYVLMGANMAELRQLQAEQSVSDAEYAYSIEKQ

TPLQTAFRQSFVQCIQHHRYIVAALKKMERFYSPIWFVKIGEVTFLMCLVAFVSTKSANSFMRMVSLGQYLLLVLYELFI

ICYFADVVFQNSQRCGEALWRSPWQRHLKEIRSNYLFFILNSRRQFQLTAGKITNLNVERFRGTITTAFSFLTLLQKM

>DbiaOr83c

LLGVDVLAPKLVINYRTWTTIFAIVNYTGFAVFSIINNGGDWAVSLKASLMIGGLIHGVGKFLTCLLKHQDMRRLILYSQ

RIYEEYESRGEYHRTLNSNINRMLGIMRIVRNGYAVAFCLMGILPLAMLMYDGTRVTMMQYRVPLPLENNFCYTVTYLIQ

LVTMVVTGVGFYAGDLFVFLGLTQILTFADMLQLKIHDLNDALELKAENRLIEVIKWHQLFTEYCRKVNGVYYELIATQV

LSMALAMLLSFCINLTSFHMPSAIFFVVSAYGMSIYCILGTILEFGYDQVYDGIYNVNWYELRGDQRKLFALMLRESQFP

YTIQILGVMSLSGRTALQIVKLIYSVSMMMMN

>DbiaOr85b

MKYASFFYTGVGIQPYAKSQKDRIRNRIVFWSNVINLTIVGTCEYVYSAYKEEKFLEAVTVMSYIGFIIVGMSKMFFIRW

KKTAITQMMEEFGDIFPRGKVDEERYNLAKYLRSCSRISLTYSTLYSVLIWTFNLFCIMEYWVYEKWLNIRVVGKTLPYL

MYIPWNYENSWSYYPLLFSQNFAGYTAAAGQISTDLLLCAVATQVIMHFDYLSNTMEHNTMEHHKLSGDWQEDSKFMAKA

VMFHERILRLSEVVNDIFGIPLLLNFMVSSFVICFVGFQMTVGVPPDVVIKLFLFLFSSMSQVYLICHYGQMVADASYGV

SVATYNQNWSHADVRYKRALVIIIARAQNVTFLKATIFLDITRSTMTDLLQISYKFFALLRTM

>DbiaOr85c

MKYAVFFYTSVGIEPYTVPRKISLWSHLIFWTNVLNLSWIVLGEFLYLCVSDGKFIEAVTVMSYIGFVFVGMSKMFFIWW

KKPDLSNLVKDLELIYPEGRAQEKAYRLDRYLRSCSRISITYALLYSVLIWTFNLFSIMQYLVYEKWLKIRVVGQTLPYL

MYFPWNWEGKWSYYVLLVLQNFAGHTSAAGQISTDLLLCAVATQVVMHFDHLARTVENSGDWFENSRFLAKTVHYHQRIL

RLMDVLNDIFGVPLLLNFMVSTFVICFVGFQMTVGVPPDIMIKLSLFLFSSMSQVYLICHYGQLVADASSGLSLAAYKQN

WHRADVRYRRALVFFIARPQRTTYLRATIFMTITRATMTDLLQISYKFFALLRTM

>DbiaOr85d

FLKYANVFYLSIGMLAYDHKESGKRKELLLHWMFIAQMVNLNAVLLIYVFLAIGNFLEATMNLSFIGFVVVGDLKIWHIW

RQRKRLTQVVNELENLHPESLDQQEPYNVKYHLSGYSRYSKFYFGMHLVLIWTYNLYWAVYYLVCDFWLGVRQFERMLPY

YCWVPWDWSTGLSYYLMYVSQNLAGQACLSGQLAADMLMCVLVTLVVMHFIRLSGHILQAVVVYHQRLLHLCQDINEIFG

VSLLCNFVSSSFIICFVGFQMTIGGKIDNLVMLVLFLFCAMVQVFMIATHAQRLIDASEQIGQAVYSHDWFHGDIRYRKM

LVLIVKRAQQPSRLKATIFLNVSLVTV

>DbiaOr85f

SRFPATVFWMMGYDVLGVTRSQRILFRVYHFICLASHSVCVGFMIFRMVETIDNIPLIMRYSTLVTYIINSDTKYATCMQ

RGAIQSLNSKLARIYPKTTLDRIYHRVNDHYWSKSFVYLVIIYIGSSIMVVIGPIITSLISLFNHHGFSYMHCYPYFIFN

PEKHWIYLGIYVLEWLHSTQMVISNIGADIWLIYFQVQINLHFRGIIQSLESVENDYKDREFLSKIVDKQVYLXSMQNDL

NDIFGGSLLLSLLTTASVLCTVAVYTLLQGVSLEGCTYVIFIGTSAMQVYLVCYYGQQVLDLVGQVAHAVYNHDFHNASI

AYKKYLLIIIIRAQQPVELSAMGYLPISLDTF

>DbiaOr88a

SKWPFWVSAILNLVFFTCNGWDIIGHLWLGQPTNQNPPVISITIYFSIRGLMLYLKRNAIVEFVNDLDRECPRDLASQLD

MQMDHTYRYFWKRFHIVQAYGHVGGLAFCLMPLVLFFLTHEGNGSPVAQQEQLLGGWLPLGVRNDPFYLFVWLYDFYCTV

SGISFFITFDNLFNVMQSHLIMHLNHLARQISVLDPRQSLTNEVTNEVQFFSHLKSLVQRQQLLNDFCGRYNEIFKVALL

VSNFVGAGSLCFFLFMFSETSDLLIMGQYIIPAFVLVVFTFEICLRGTLEEASEGLESSLCHQEWYLGSQRYRKFYLIWM

QYSQRTQKLGAFGLVEVNMVHFTDIMQLAYRLFTFLKS

>DbiaOr94a

HKERIESMSLILRVMQVFGLWPWSLWTSGFLKHNYRFLLHLPITFTFIGLMWLEAFISSDLEQAGQVLYMSITEMALVVK

ILSIWHYRTEAWQLMKELRNAPDYELRQQEEKDFWRREQRFFKWFFYIYILISLGVVYSGCTGVLFLEDYELPFAYYVPF

EWRNERRYWFAYGYDMAGMTLTCISNITLDTLGCYFLFHISLLYRLLGLRLRALKNLENLEDEAHFGKKLRGIFILHQKV

RXLTRSCQKIVSPYILSQIILSALIICFSGYRLQHVRDNPGQFIAMLQFVSVMILQIFLPCYYGNEITVYANQLTNEVYH

TDWLNCRPPIRKLLNSYMEHLKKPVTIRAGNFFAVGLPIFV

>DbiaOr94b

AIQTLIVIQRWIGLWEEKEDSLITWLKRIYPFVLHLPLTFTYIALMWYEAITSSDFEEAGQVLYMSITELALVTKLLNIW

YRRNEAANLINELQHDPAYNLRNTEEIKFWQLNQRGFKRIFYWYIGGSLFVAVMGYISVFFQEEYELPFGYYVPFEWRSK

ERYFYAWGYNVVAMTLCCLSNILLDTLGCYFMFQIASLFRLIGMRLEALKNAKKAKPELRNIFQLHAKVRRLTRQCEVLV

SPYVLSQVVFSAFIICFSAYRLVHMKQRPGLFLTTVQFVAVMIVQIFLPCYYGNELTVHANALTNSVFGTNWLEYSVGTR

KLLNCYMEFLKRPVKVRAGVFFEIGLPIFV

>DbiaOr98a

AFKYFEYGMFCMGWHTPVKSKISYYIFATFIFSWCVIYLPIGSISFMKNTFTPSELVTVLQLFFNAVGMPFKVLFFNVYI

SGFYKAKKILSQMDQRCNTLEERTEVHRWVVRCNKAYLIYQMIYTCYTLSTFLSAALSGKLPWRIFNPFDWRESLNFWKA

ALNETALMLCSVTQTLMSDVYPLLYGLILRAHIKLLHLRVDKLCTDPEKNEPEKNEGENQEDLNNCIKDLQLIKEFADTI

RPAIAHTIFVQFLLIGICLGLSMINLLFFADIWSGLATVAYINGLMVQTFPFCFVCDLIKSDCEHLEMAIFHSNWIDTSR

RYKTSLIFFLKNSQKSIAFTAGSVFPISTSSNIKVVKLAFSVVTFVNQ

>DbipOr2a

DTHRAVRYHWRVWELTGLMQPTGISRFWYFIYSLVINASVTILFPLSLVARLFFTHNMQNMCENLTITITDIVANLKFLN

VFLVRRQIRSLLKHLDERARQIHHPEELAALNEAVTIAQKGFQYFARIFTFGTILSCIRVAISSKRQLLYPAWFGVDWEN

SWAYVICYAYQLFGLVVQAVQNCASDSYPPAYLCLLTGHMRALELRVRRIGYGRGRLNSTSTHEELLDCIRDLMLVHQLK

NIIQRILSVACMAQFACSAAVQCTVAMHFLYVDNDLSAMILSIVFFVAVTLEVFIICYFGERMRTQSEALCDGFYACNWV

DQLPVFKRDLIFTLARTQKPSLIYAGGYIPLSLETFEQVMRFTYSAFTLLLRA

>DbipOr7a

CFRNLFNCFYALGMQAPDGPTRSSTWRRIYGCFSAVMYVWQLILVISYRYIEGMEMTQILTSIQVAIDAVILPAKIVALA

WNLSLLRRAEYYLAKLDGRCKDAEEFHMIAEAGRFCNRLVWFYQICYAIYSSSTFLCSFLLGQPPYALYLPLDWSRSVQF

NIQAWIEFLIMNWTCLHQASDDVYAVIYLYVVRVQVQLLARRVRQLGSGDPPESDERRQEEHCQELQKCIVDHQTVLKLL

GCISPVISRTIFVQFLITAAIMGTTMINIFIFANTNTKISSIIYLMAVTLQTAPCCYQATSLMLDNEHLALAICQSRWLG

QSARFRKMLLFYLHHAQQPITLTAMKLFPINLATYFSIAKFSFSLYTLIKGM

>DbipOr10a

VYFFAVPRNSLNLMGYWPGGGKDKKNKNPPLRAYVHFVILAIGVFTEAGMRFLHQEELTLALETFCPAATSAVTLLKMFL

MWRYRQDLASMWCRLRSLLFETGRERPQQEIRLRHSLLAVRINCWPVSAGFLTGSIYNIKPVMIALVLYLQQRSDENVWV

TPYNMTMPPFLLKSPFFPFTYLFTAYTGYVTIFMFGGCDGFYFEFCSHVSALFEILQSETKAIFKLTIDSEEALHLEQQL

RAIVVRHNAIIDLTKSFRQRYAIITLAHFVSAAMVIGFSMVNLLTVGGIGLGVILYVAYTIAAASQLLVYCYGGTLVAES

SLGLARILFACPWNICWPKQRRYVQLLILRSQKALTMAVPFFSPSLAAFASILQTSGSIIALFKSF

>DbipOr13a

LPPQCAWLKLNGSWPLSKDGMTYTVWAWYVIGSVGITICYQTGFLMTHLSDIIMTTENCCTTFMGALNFIRLIHLRVNQN

KFRSLIESFYNDIW

>DbipOr19a

STRPLKLHWLIFRVMGVHAPKGRESLWGRHYRTYSIFWVTFHLCMSMVVNFLMSNSLESFCDSLSVAMPYTIYMLKLVNV

WAARDRLLQTHHILRYLDTRLCSATERRIVRTKGIRRSHRVLWNIFIGVAAVFICGIAYIILSSERTLMYPSWIPWQWKE

SRVFVSTVAAHTGCLAETAVLVYNVATYPCTYLILLSAHTRALACRFARLGHSARETQQQETQQQTHLRLLDYIWDHQML

IRILKSLERSLSKTCFMQFFCMACSQCTICYLILFEKIGFMPLVNMLCLLLAFTCETLLLCYSAELVHQAGNDLLSAVYS

CNWLDQPVLFRRYLVLVLVRCQKPMILYSGIVVPLRMTSFXXXXKGAYSMLTLLSKMR

>DbipOr23a

WAMAINFLVALAFPMLALSLFSYDSPLENITNFSLTITSLATIVKFGLFTRRLSHLGEMEELISEMDKRVAGMEQECYLL

MKKRLHLLSRGFLLISCIVIVSLEMSFFFKDERCLPYPSWFPLDWESSWSFLVAVAYQQLVIFVQVFQNYVGDSFPPLAL

YLIAQQSHLLNLRISRIGFHGQEAELGKCINDQKRLYRLLELSRNVISWPMFVQFIVIAINVGVTVFGLVFFVETVYDRI

YYVSFLIGITLETYPLCYYGTNMEQSFHELHYAAFCCNWAEQSRSFRSTMLILSERTKMNALLLAGGVVPIHLSTFQATC

KAAYSFFTLMS

>DbipOr33a

IYRVYWFYWRLLGLETDFPMRHVLDIIITFFVTIWYPIHLIVGVFSERSLGDICKGLPITASCFFCSFKFICFRSKLKEI

KAIEALLKELDQRAVSDEECEYFDKNSRQEANFIWKSFLVAYNLSNISAIASVLFGGHQLLYPGWFPYDVQATLRYWLSV

AYQVVGVSLAILQNLANDSYPPMTFCLVAGHVRLLAMRLRRIGQETRATQVSVARKLIEGIEDHRKLMKIIDLLRSTMNI

SQLGQFMASGINISITLVYILFFAENLFSITYYAVYFISMVLELFPCCYYGTLISIEMNSLTYAIYSSNWLGMDRKYCRT

LLMFMQLTLTQVQIKAGGTIGIGMNAFFATVRLAYSFFTLAMSLR

>DbipOr43a

GINVRMWRYLAVLYPTPGTNWRKYAFVLPVTAMNFMQFLYLLQSWGDLPAFILNMFFFSAIFNALMRTWLVIIKRVQFEK

FLNQLSVLFHSIREDSDERDILRQAEHEARHLAIVNLSASFLDIVGALISPLFREXVAEAVYNVPWYRGSIRFRKTLLIF

LMQTQHPLEVGVGNVYPMTLAMFRSLLNASYSYFTMLR

>DbipOr43b

TLRMSGLNLGEKSGVAWKIWRVISYTYSMLILPISNYTIHLAEFPPDLLLQSLQLCLNTWCYAFKYFTLVIYTRHLELSN

IQFDEMDKFCVKAEEKRKIRDSVAAVNRLYMVFVVVYILYGTSTLMDALIHDRVPYNTYFPFNWRLSTQLYIQCFLEYFI

VGYAIYVATATDSYALIFVASLRTHITLLKERIISLGIINNTDADSVFTLLVDCVKAHRTMLSFCDAIRPIISGTIFAQF

IICGSILGVIMINMTLFADKSSQFGTVTYIMAVLLQTFPLCFYCNAIVDECNELADALFHSAWWMQEKRYQRVVLQFLQK

LQQPMTFTAMNIFNINLATNINVAKFAFTVYA

>DbipOr45a

SYFAVQRRALEIAGFDASSPKLHLKHPIWAGILVLSLISHNWPMAVYALQDLSDLTRLTDNFAVVIQGSLSTFKFLAFVV

KRRRIGALIHQLHQLNQKASKSEQQLERIKRENRLDRFVSKAFRNAAYGVITASAIAPMLIGLLGFIRVGIFRPTTPMEF

NFWLDETQARFFWPIYFWGVLGVASAAWLAIAADTLFSWLVHNVVVQFQLLELLLHNCQLTTFIHRHRLALKLAEDLNHI

FAEIVFMQYMLSYLQLCMLAFRFSRSGWSSQVPFRASFLVAVFIQISSYCYGGEYLKQQSLAVSQVVFDCEWSEMPPEKR

LLWKMLIMRSQKPAKISAFMFDVDLPLLLWVARTAGSFLALLRTF

>DbipOr45b

LFFVTRYSFGLLGLSFGQKMSWFQLAWLVFNFVNLAHCCQAEFDFGWSHRTSPVDAMDAFCPLACSATTLFKLGWMWWRR

QEVAGLMDHIRQLTEGQERRRDSRRENAQRSCYVMATRSGMLVFTLGSITTGAFVLRSLWEMYVRRRQEFKFDMPFRMLF

PDFAHRMPWFPIFYLYSTWSGQVTVYAFAGTDGFFFGFTLYVAFLLQALKWRESKLCCQQLADIVDRHNEIEKIVERFSG

IMAAPTFVHFVSASVVIATSVIDILLYSGYNIIRYVVYTFTVSSAIFLYCYGGTEMATESLSLGEAAYCSAWYKWDRETR

RRVFLIILRAQRPITVTVPFFAPSLPVFTSVLKFTGSIVA

>DbipOr46aA

YKRQRAFLNIFSLWPPESGYRRTLHQINFIHVIGFWVLLFDLLILHILGNLNRMSEVVKAFFVLATSLGVTIKLISIKWY

NVELDKLFGSLDDEEFRPQGPKERAIYAAACETSRWQRDGYMFLSLAALGMILIPQLIFEWSHLPLPAYSPFDEDPRPGY

WILYTYLFLGETISCLTNIGFDPLCSSLFIFIKCQLDFVAVRFSKLDHESEDHESEDDSTVGEELRKCIRQHMAIVDLTA

QVEKLLTKTISLQVFCSVLVLTANFYAIALLSNEKLVLFKFIVYQAAMLAQIFIMCYYAGEVTQRSSELPHELYKTQWVN

WSRPNRRILLLFMQRLQSNLRIRTINPSLSFDLMLSIVNVSYSYFALLKRV

>DbipOr46aB

DFYKYQVWYFQILGAWRLPITNGQKRLHAFRFGFILVMLWVMLLLFAVELVNNISRMRHILKVFFMFATEMSCMTKLMHL

KSRSRKLAYMVEMMRSKTFSALESEEIEIMESARRVVIKMRNFYGISSLGTASLILLVPFFAGLEELPLTMYEVCNIQGS

LCYWLLFVFHCACLLPTCVLNITYDSEAFSLLTFLRVQLQMLVLRLEKLGSKMDPRDNQRMDPRDNQRIAWELRECARYY

NSIVQLKDLVEEFIKVPGSVQLMCSVLVLVSNLYDMSTINGEGIYMAKTCIYQLVMLWQIFIICYASNEVTIYSSRLCHA

IYSSKWTGWSRINRRMILLMMMRFDSPLLLTLNPTFVFSLEAFGSIVNVSYSYFALLKRV

>DbipOr47a

KGTIALLGFDLFGESRKMWLRPYRAVNVFGIATLFPFILAAVLHNIKNVMLMADAMVALLITILGLFKFSMILYLRKDFK

GLIDRFRLVMANSGQGEEYAEIIRSANQQDQRVCSIFRTCFLIAWALNSVLPFVRMGLSYWLTGHYEPELPFPCLFPWDI

HILRNYAMSFVFCAFASTGVVLPAVSLDTIFCSFTSNLCAFFKIAQYKVLRFKGSTLEESQTTLNRQTSLDMCSELNECY

QPIICAQFFISSLQLCMLGYLFSITFAQTEGVYYASFIATIIIQAYIYCYCGENLKTESASFEWAIYDSPWHDSSAAICR

SLLISMMRAHRGFRITGYFFEANMEAFSSIVRTAMSYITMLRSF

>DbipOr47b

GTKVFYVHLRCNDIDEIIWDLDYYNREVRPHNSDEWQRLCYLAESGFFINCFFLVNFLSAVICLQPLVGQEEKLPFHSIW

PFHWHHPKTYWFLYVWLSVTSHHNLMSILMVDMVGICTFIQTALNLKVLCIEIKRLGEMRADAKFREEFYRVIRYHQHII

KLVDKVNTTFNGSFNAQLMASFAMVSISTFETMVASVDPKMALKFAFLMVVAFVQLSTWCVAGTLV

>DbipOr59a

FRSHWTAWRILGLAHHRLNSWRNLYLLYSLVMNLFITLCYPLHLGMNLFRNGSLTDDILNLTTFATCTACSVKCLIYGYN

IRQVMEMERLLRLLDSRVVGQKQLNIYSQVKVQLRNVLYIFIGIYMPCALFAELMFIFKEERGLMYPAWFPFDWLNSRYY

YMANLYQIGGITFQLLQNYVSDCFPAVALCLISSHVKMLYNRFEEVGEDSEKSEKDAEKELEACITDHKNLLELFRRVEA

FVSLPMLIQFTVTALNVCIGIAALVFFVTEPMARMYFYSMAMPLQIFPTCYFGTDNEYWFGKLHYAAFSCNWHPQNKSFK

RKMMLFVEQALKKSTAVAGGMMRIHLDTFFSTLKGAYSLFTIIIRMR

>DbipOr59b1

YLYRAMWLIGWIPPKKGILRYVYLFWTCVPFAFGVFYLIISYVKEFKNFGEFLTSLQVCINVYGASVKSTITYIFLWRLR

KTEMILDVLDKRLQNDSDRQKIHNMVARCNYAFLIYSFIYCGYAGSTFLSYALSGRPPWSVYNPFDWRDGVSLWIQAIFE

YITMSFAVLQDQLSDTYPLMFTIIFRAHMDVLKDHVRNLRTDPEERSEADNYQDLVNCVIDHKMILRCCDMIRPIISRTI

FVQFALIGSVLGLTLINVFFFSNFWKGVASFLFVITILLQTFPFCYTCNLLIDDADDLANTIFQSNWVDAEPRYRATLVH

FMHHVQQPIIFIAGGIFPISMNSNISVAKFAFSIITIVRQM

>DbipOr59b2

YRAMKFIGWRPPKEGALRYLYLFWTLMTFVWSTTYLPLGFLGSYMKSFSPGEFLTSLQVCINAYGSSVKVAITYSMLWRL

VKAKALLDQLDLRCTSMEEREKIHRVVARSNHAFLIFTFVYCGYAGSTYLSSVLSGRPPWQLYNPFDWRDGTKLWMASTL

EYIVMSGAVLQDQLSDTYPLIYTLILRAHMDMLKERIRRLRCDETLSESENYEELVKCVMDHKLILKXCALIKPVISGTI

FTQFLLIGLVLGLTLINVFFFSDIWTGIASFMFVITILLQTFPFCYTCNLIMEDCEALTHAIFQSNWVDANRPYKTTLLY

FLQNVQQPIVFIAGGIFQISMSSNISVAKFAFSVITITRQM

>DbipOr59c

RTAFVMGWLPPKHPAWQRRIYSLWTGTTMLLGLIYLPLGTYVVHFDKFTPNEFLTSLQVDFNCIGNVIKATMTFSQMWRM

RKMNELIAPLDERCATPAQRQLLHDMVAWVNRIMVFFLAMYLGFSTLNLFTSVFAGKAPWQLYNPIDWRQGWQLWVASFM

EYFVVCIGVMQELLSDTYAIVFISLFRGHLAILRDRIKNLRNDPELSEEESEEENYKQLVACIQDHRTIVQCSEVIRPLL

STTIFAQFMLVGIVLGLAAFNILFFPNTFWMILANVSFILAICTETFPCCMLCEYIIEDCSNLTNALFHSNWSTANRRYK

SALIYFLHRAQQPIEFKAGAIFPISVQSNITVAKFAFSIITVVKQM

>DbipOr65b

IKAIGFYMNTDKCRQPVLVSWHYFFMLQMAISLASLGYGILESLEDIVLLGQDLAYTIGVFYIFFKMVHFSIYADDIDEI

IDGLEECYQWDSQGRGLEESRCMKRRIFFWLTGLMAAWLFMVISFVLIIISTPFWLEAQELPFHVAYPFDPSMHPCHATI

FVSQSCILMYAMIWTLCCEQISVTIFSELTSALKVLCTELRVVHASLFAQEVYDLAKFHQRIIXXXDRSNEIFNGAFTMQ

LIVNFLLISLSVFEAMVSRHDPKVAAEYLVLMVMALGHLSYWSKFGDMLTQESLNVAVAAYEAYDPTGFRSTHTIIWLII

QRAQTPLSVKSRLFPPFNLENNLAVLKQCYGIFNFLLK

>DbipOr67a

IEEFFRLPKIYYRTIGLNPYESKNDPDILSRIQSVVFILNLVFVVVMEVVYIVISFRNFLESCMVMSYIGFSFVGFSKII

VVIVQKEKMTSLFRTLESIFPKPLSAEEDPYDTRQYLRRCLRFTKGFGGLYTFLVFIYNFYPITQYKVLELMGSPNANPV

LPYSITAPWDYRHGWDYYLNYFSEAVAGYTATCGHISADLMIFSVTTQVIMHFSRVIKAFREFDVGDDEALQELRSLVAY

HHHILRITDDVNDVFGIPLLINFAASSLLVCFVGFQMTFGVSPQHFAKLMLMLSSALVEIYLICSFSQRLIEASENVSSA

VYEMNWTGADVRFRRMLIYIAHRSQKPVCLKATVFVDISIETMSIFLRMSYKFFCAIRTM

>DbipOr67c

SEDNTARTFEDMMRVPVQFYRSIGEDIYAHRSKRSLLLKIYLYAGFINFNLLVIGELVFFYNSIQDFETVIAVAPCIGFS

LVADFKQFAMVYHKQTLIQLLDELEDMHPKTLAKQKEYKMSRFEQTMKRVINIFTFLCLAYTTTFSFYPAIKATVKFQFL

GYDTFDRNFGFLIWFPFDATSSVVYWIMYWDIAHGAYLAGIAFLCADLLLVVVITQICMHFEYISMRLEEKENKEFLISM

IRYHDKCLRLCEHVNDLYSFSLLLNFLMASMQICFIAFQVTESTVEVIIIYCIFLMTSMVQVFLVCYYGDSLIAASLKVG

DAAYNQKWFQCDKTYCGMLKLLIKRSQKPASIRPPTFPPISLVTYMKVISMSYQFFALLRT

>DbipOr67d

VDRYLRIIKVIRFFVGFCGNDVNPNFKMWWLTYLVLSAIGLFYACTGYIYRGVVIDGDLTIILQAMAMVGSATQGLTKLL

VTANLAPLLRHIQYSYEDIYREYGAKGEYTECLERRIRITWRLMLSFLCPCLMSMVSIVSFPIFYLVVYKKKIMVMQFLM

PIDENTDGGYMILSAVHVGLIAFGGFGNYGGDMYLFLFVTNLTLIKDIFCIKIKELNEVVLKRTEYEQMRLLLFQMRLLL

FDLVAWHQKYQKILLTTRRIYSIVLFVQLSTTCISMLCTIACVFLHVWPAAPIYLLYAGTMLYAFCGLGTAVEISNDALM

REIYSCLWYELPVKEEKIIVLMLAKAQTESYLTAANMWPLSMNTALQLTKGMYSFTMMMLT

>DbipOr69aA

KLQTVCYYLGSFFMGYQNIGGVVYWCLYAKDIIETAQVFGSLMLTCVGFSKIWCFTRRRNQMEDVMAELHELYPTTRKEH

YRLQHHYDWADLIMKYANLFYFAFYIFYNGSPLVLLLWEYITDDQNLSYKTQANAWYPWKVRGSWGYALAIWVSTMAGNL

GIFLTLAILNILCVCTVQLVMHFDGLATQLLNLDAAHQELKHLIRYHRQLIDISDKTNEIFDSIFLTSLICSTLAICMTS

VAVLLLDLAPALKNINGLLAFLVYHFMLSYLGTQINLASEKILPAAFYNNWYEGDLPYRKMLLILMLRASKPYKWKTIKL

SEVSILNYVDTLKTSYQMYACVRSM

>DbipOr69aB

KLQTVCYYLGSFFMGYQNIGGVVYWCLYAKDIIETAQVFGSLMLTCVGFSKIWCFTRRRNQMEDVMAELHELYPTTRKEH

YRLQHHYDWADLIMKYANLFYFAFYIFYNGSPLVLLLWEYITDDQNLSYKTQANAWYPWKVRGSWGYALAIWVSTMAGNL

GIFLTLAILNILCVCTVQLVMHFDGLATQLLNLDAAHQELKHLIRYHRQLIDISDKTNEIFDSIFLTSLICSTLAICMTS

VAVLLLDLAPALKNINGLLAFLVYHFMLSYLGTQLLFQSEKILPAAFYNNWYEGDLPYRKMLLILMLRASKPYKWKTIKL

SEVSILNYVDTLKTSYQMYACVRSM

>DbipOr71a

LVRPVRLLTAILRVFRLWPKDDSGEDWANWKAYMLQVPFTLLFAAAMWVEVILSDDIQHTSDVLLICLTTTALGAKILNN

WKLSHVAQRLLHEWSTQEQFKLRSLKEVEMFQFEHRRFTRVAISYIMCSFGVIPFICIQPLFDIPNKLPFYMWTPFDWHN

PDLFWYPFIYQVITIPVICICNVTMDAVNWYFMLHLSLCFRILGERLSSLSHEDKNHEDKNVREKFLQLVRLHQDLKRQA

RDIETYVSKSVFTQILVSSLIICFTIYSIQMSKQDFGRFYGMLQYLSCMVMQILIPSIYGNNVTYSADKLPNALYNCDWP

DMSPQMRRFVLMFMSYLNRPVSLRAGGFFAVGLPLFTKTMNQAYSLLALLLNMK

>DbipOr82a

FQLQENCLRAMGHTGGHDNSISDGKLSVKHIVSLAFVVSAQYPLISYIVYNREDMEKVTACLSVVLTNMLTVIKITTFLA

YKQDFWRMIQRFREMHQQXXXXXXXYGYVADANKVASLLGRAYCISCGITGLYFMLGPIVAIITSKWRGTTYIRELPMPM

KFPFNDVNTPGYEVGFVYTVFVTVVVVTYASAVDGLFISFAINLRAHFQTLQRDIENFNYEREVRAALRSVVDYHVELLS

MSKHLRVIYTPIVFGQFFITSLQVGVIIYQLVTRLDSIMDLLVYLSFLGSIMLQLFMYCYGGEIIKVESLKVDIAVRLSD

WHLATTTIRKSLAFIILRSQREVLIKAGFYEASLANFVAICRTAMSLITLIKSI

>DbipOr83a

QTMCIAAMYPFGYYFKVSVLYELFNYFVSVHIAALFMCTIYLNYGKGDLDFFVNCLIQTIIYSWMIMKLYFRRFRPALLD

EIMKYINEKYEPRSAIGFSYVTMDGSYRMSRLWIKTYVYCCYIGTIFWLALPIAYRDKSLPLACWYPFDYTEPGIYEVVF

FLQAVGQIQVAATFASSSGLHMVFCILMSGQYDVLFCSLKNVLATSYLRMGVSLSEMRQLEAEQSVSDAESQYTFQSHLE

QLLQENPKSEDSLDFSRAFRDSFIRCVYHHRYIVSVLKKMEQFYSPIWFLKIGEVTFLMCLVAFVSTKSANSFMRMVSLG

QYLILVLYELFIICYFAEIVYQNSQRCGEALWRSPWQRHSREVRSDYMFFMLNSRRQFQLTAGKITNLNLERFRGTITTA

FSFLTLLQKM

>DbipOr83c

LLGVDILDPVLKFNYRTWTTIFAIINFTVFTIYSIVDNGGDWLVSLKAGLMIGGLTHGTAKFLTCIVRQKEMRSLTLFTQ

DIYDGYEKRNPSSSTLDANIDRLLRFMKGIGYGYMVTNFLMVFTPLAMFAYNDSRMTVLMYEIPLPIQKNFGYFLTFLIH

LVTICVRGFGFYAGDLFVMLGLTQILTFSDILKLKIKELNSVLKLKEEKRQRLLIEMIKWHQLFXXYCRRVNNLYNPLIT

TQVLAMAYEILATFCINLNGLHVPSAINFLLAAYCMSVYCVMGTQIEFSYDEVYENICNVSWYELTGEQRKIFGMTLRES

QSPHNIKLLGVWSLSVRTALQIIKLIYSASMLM

>DbipOr85a

HCVGWNPPSREESNWWIYYITFFVVFMMGFIYMFGLLVTGIKDIADLFTYLQAPVNSVSAIIKTIIIYFMRKRFFKVHEV

MNRMDDLNSGEEDREAIQKCTKDCRKVTIIYQIIYYGYLVGAVLGAIAYNTTPYHLYNPYSPSEKVDLLIGNFMEGLAAF

GILITNLVADVYPIMYVMILRTHIHLLKRRIENLRSDPDKSEDKSEDENYDDLVKCIKAHRLIIEYADLIRPVISNTVFV

QLVCTGVLLGLSTVSITFFDIVDRFVTVYYSMAILSQTFPFCYTCESLLSDCNDLAVVLLHSKWIGAEPRFRKTLVLFMH

QTQAPVSFTACGVVQIGLDTNIKMVKFAFSVFTVVQ

>DbipOr85b2

DDFLKYANKFYLSIGMLAYDHKENSKRKEQLLHWLFIAQMVNLNVVLLIYVFLAIGNFLEATMNLSFIGFVVVGDLKIWH

IWRQRAKTTHVVREMEKLHPKRADQQEGYAIKDYLSSYSRYSIFYFSMHMVLIWTYNLYWAVYWLVCDFWLGIRSFERML

PYYSWVPWDWSTGNTYYFMYVAQNIGGQACLSGQLAADMLMCALVTILVMHFIRLGHQIEALQAAVVYHQRLLNLCQDIN

EIFGVSLLCNFVSSSFIICFVGFQMTIGGKIDNLVMLVLFLFCAMVQVFMITTYAQRLLDAXXXXXXAVYNQDWFNADIR

YRKMLVLVIKRSQQPSRLKATIFLNVSLVTVSDLLQLSYKFFALLRTM

>DbipOr85c

MKYAVFFYASVGIEPYTKSSNRSLSANLLFWANVINLGGITLGELMYLGKSLSDLLEAVTVMSYIGFVFVGMSKMCFIWS

KKRELSSMVEELEQIYPKNIIQEEKYRLQSYLRSCSRISFTYSLLYLILIWTFNLFNMMQYLVYEKLLKSRTVGKILPYP

QYYPWMWEGDFSFYIQFLSQNFAGHTSASGQISTDLLLCAVATQIVMHYDYLERGLTGDWKKDSRFLVKIIRYHQRILRL

TEVINKIFGLPLLLNFMVSTFVICFVGFQMTVGVPPDLLVKLFLFLFSSMCQVYLICHHGQLIADASSGLAIAAYKQDWS

YADVRYRRALVFIIARSQKITYLKATVFMNITRATMTDILQTSYKFFALLRTM

>DbipOr85d

KPILLDDFLKYANKFYLSIGMLAYDHKENSKRKEQLLHWLFIAQMVNLNVVLLIYVFLAIGNFLEATMNLSFIGFVVVGD

LKIWHIWRQRAKTTHVVREMEKLHPKRADQQEGYAIKDYLSSYSRYSIFYFSMHMVLIWTYNLYWAVYWLVCDFWLGIRS

FERMLPYYSWVPWDWSTGNTYYFMYVAQNIGGQACLSGQLAADMLMCALVTILVMHFIRLGHQIEALQAAVVYHQRLLNL

CQDINEIFGVSLLCNFVSSSFIICFVGFQMTIGGKIDNLVMLVLFLFCAMVQVFMITTYAQRLLDASGNIGQAVYNQDWF

NADIRYRKMLVLVIKRSQQPSRLKATIFLNVSLVTVSDLLQLSYKFFALLRTM

>DbipOr85f

SSLFRLMGYDMLEATRLQRIMMQLYRVLCLGSHGVCLGFMFFRLFETIDSVSLIMRYATLVTYVVNSDTKYGTVLQRAAI

QSLNNKLADLYPKTTLDRIYYRVNDHFWSRTLLYLIRFYIGSSIMVVVGPILTSLWLYFFHEQFSYMHCYPYFIFDPEKH

WIYVGIYALEWLHSTQMVVSNIATDMWLIYFQVQICMHFRAMIKSIEHFLSKIVDKHHYLVSLQTDLNSIFGSSLLLSLL

STASVLCTVSVYTLIQGLTLEGVTYVIFIGTSVVQMYFVCYHGQQVLDLSAYISHAVYNHNFHNASLSYKKFLLIIIIRS

QKPVELNAMGYLPISLDTFKQLMSVIYRAITMLRQM

>DbipOr88a

AFWNVIFFGFNGWNVLQTLTQNPPIVSITVYFSIRGLILYLKRKDIVDFVNDLDREWPQDIASQVEKNMDQTYLAFCQRY

RFVQIFSIVGFPIFCMLPVGVFVLTHEASDVPVSLNEQLLGGWLPFGIRQNPFYFLVWTFDVICTLCGVSFFLTFDNLFN

VMQNHLIMHLDDLSKQIDGLNPGDSVTNEKVTNEKVFFANLSALVQRQQLLNELCRRYNNIFKVAFLVSNFLGAGSLCFM

LFILSEASDLLIIVQYMIPTLVLIGFTFEICLRGTQLEEASSRLYSSLARQNWCNGSRKYRKFYLLWIQYSQRTQKLGGF

GLIEVNMVHFTDIMQLAYRLFTFLKS

>DbipOr92a

TFDELTKFPMTFYKTIGEDLYSDRDPNVVRRYLLRFYLVLGFLNFNAYVIAYFIMSTTTLLEATAVAPCIGFSFMADFKQ

FGLTVNRKRLVKLLDDLKEMYPEDKESQRRYQVPYYRKHMNSVMTLFTILCMTYTSSFSFYPAIKSTVKYYLMGSEIFER

NYGFHILFPYDAETDTVYWFSYWGLAHCAYVAGVSYVCVDLLLITTITQLTMHFNFIANDLEAYDGGEHEENIKYLHELV

VYHARALELSEEVNNIFSFLILWNFIAASLVICFAGFQITASNVEDIVLYFIFFSASLVQVFVVCYYGDEMISSSSRIGH

SAFNQNWLPCSTKYKRILKFIIARSQKPASIRPPTFPPISFNTFMKVISMSYQFFALLRT

>DeleOr1a

LWTQRITFACMGLNLQPKKGKVLQTPVLYGIMFLATGFELCTVCAFMVQHRNQIVLCSEALMHGLQMISSLLKMAIFLAK

SHDLVALVQLIQAPFMNRGDLGVSEWRSQNRWGQLMAAVYFMMCAGTSVSFLLMPVALTMIKYYSTGDFAPVSSFRVMLP

YDVTQPNIYAMDCCLMIFVLSFFCCSTTGVDTLYGWCALGLSSQYRRLGLQLKAEHARLLKLVKHFNVSFMEIAFVEILV

ICVLYCAVICQFIMPHTDQNFAFLGFFSMVVTTQLCIYLFGAEQVRLEAEGFSRQLYEIPWQKLSPQHRRLLIFPLQRAQ

RETVLGAYFFELGRPLLVWIFRTAGSFTTLLNA

>DeleOr2a

DTHSAVSYHWRVWELTGLMRPPGIGHLAYRVYSVVLNSVVTVLFPLSLLARLLFTTNMAAMCENLSITITDIVANLKFVN

VYLVRSQIRVLLRRMDGRTRSVGHPAELSALRREVDIAQGTFRTFARIFVFGTILSCVRVVIRPDRELIFPAWFGLDWQH

SRNYVLINLYQLFGLIVQAVQNCASDSYPPAYLCLLTGHMRALELRVRRIGYRREEVYEELVECIRDLMRVHRLREIIQR

ILSVACMAQFVCSAAVQCTVAMHFLYMDHDLTAMIISIVFFTSITLEVFVICFFGDRMRTQSEALCDAFYACNWVEQLPK

FKRALLFTLARTQRPSLIFAGNYIALSLETF

>DeleOr9a

EDQKDQGQGQSLRVQILVYRCMGIDLWSPTRANDRPLLTFVTMGPLFMFMVPMLLAAHEYITQVSLLSDTLGSTFASLLT

LVKFLLFCYHRKEFVGLIHRIRGILDKEISVWPDARQIVEAENQSQMLSLTYTRCFGLAGIFAAMKPFVGIVLSLIRGDD

IHLELPHNGVYPYDLQVVVWYVPTYLWNVMASYSAVTMALCVDTLLFFFTYNVCAIFKIAKHRMIHLEGLIQVLVLHQTG

LQIADNIADKYRPLIFLQFFLSALQICFIGFQVADLFPNPQSLYFIAFVGSLLIALFIYSKCGENINSASLDFGMGLYES

NWTAFAPPTKRALLIAAMRAQRPCQMKGYFFEASMATFS

>DeleOr10a

DQQLSVYFFAVPRLSLDIMGYWPGGTGDRLPRRSIVHFVILSIGVITEWHAGLRFLDQQQITLALETLCPAGTSAVTLLK

MFLMLRYRRDLSTMSDRLRSLLFDLKSERSDQDIRLSHSVKAARINFWPLSTGFFTCTTYNLKPLLIALVLYLQDRFDGF

VWFTPFNMTMPTVLLRSPFFPLTYVFIAYTGYVTVFMFGGCDGFYFEFCAHLSSLFELLQAELRSIFRLEQRMREVIMRQ

NTIIELTNFFRERYTVITLAHFVSAAMVIGFSMVNLLTAGGNSLGALLYVAYTVAALSQLLVYCYGGTLVAESSMELCRV

MSTCPWQLFKPPQRRLVQLMILRSQRPISMAVPFFSPSLATFA

>DeleOr19a

NSTRALVYHWRIFRIIGLHPPPRNTMWGRHYSVVWHTVFHFCLWVSFSVNFLLSNSLETFCESLCVTMPHTLYMLKICNV

YRVHDELLHSHRVFRHLDSRLSCSMEVQIVEEGVARAEFIFKFIVRGILAVLVVGILYIGLASEPTLMYPSWIPWNWTGS

SAYLPTVTLHTSAIIETSVVVLTTSTYPGTYLLLLSAHTKALAWRVASLGHNDLAGYIQDHQIILQLFKSLERSLSMTCF

LQFFCTACAQCTISYFLLFEEIGVMRFANMLFLLVAFTTETLLLCYTAEILCQEGESLLDAVYSCNWLDQSIQFRRLVLL

MLVRCQKPMILVSGVIVPISMKTFLVVVKGAYSMLTLLNEMR

>DeleOr22c

FYRIPRISGRIVGIWPQRIVGVRPWHAHLRFLFALAVVLVGAVGELSYGCVHLDNLVVALEAFCPGTTKAVCVLKLWVFF

RSNRQWAALVQRLRVMLWQSRRAEGQRMLVGLATTANRFSLLLLSSGTMTNTAFNLQPLIMGFYRWMAELPGHIELPFNI

LPGFAVQPGLFPVTYVLLTASGACTVFAFSFVDGFFLCSCLYISGVFRLVQQDIRRIFADTEAMNAEVRHRLAKVVERHN

AIIDFCTDLTRQFTVIVLMHFLSAAFVLCSTILDIMLNTSSLSGLTYICYSIAALTQLFLYCFGGNQVSESAAVADVLYD

IEWYKCDARTRKVILMILRRSQRAKTIAVPFFTPSLPAFGS

>DeleOr24a

RHYFVVPKFALSLIGFYPEQKRTLLIKLWSFFNFFILTYGCYAEYYGIHYVPINIATALDALCPVASSILSLVKMVAIWW

YQDELKSLIQRVRFLTEQQRSKRKLGYKKRFYTLATRLTFLLLCCGFCTSTSYSVRHLLENILRRAHGKEWIYETPFKMM

FPDILLRLPLYPITYILVHWHGYITVVCFVGADGFFLGFCLYFTVLLLCLRDDVGDLLENIEVSLSEQQEARIVREMEIL

VDRHNEVAELTQRLSGVMVEITLAHFVTSSLIIGTSVVDILLFSGLGIIVYVVYTCAVGTEILLYCLGGSYIMEACSDLA

SSTFSSHWYGHSVRVQKMALLMVARAQKVLTIKIPFFSPSLETLTSILRFTGSLIAL

>DeleOr35a

LAWPLALFRLNHIWPLDPSTEKWARYLDKILAVLGCLIFMQHNLRYLRCGNRNMDAFLTGMPTYLILVEAQFRSLHILLH

FEELQKFLQVFYANIYIDPRKEPEMFRRVDGKMLINRCVSAMYGAISGYLISPVFSLINKRKDFLYSMIFPFNSDPLHIF

VPLLLSNVWVGIIIDSMMFGETSLLCELIVHLNGSYLLLKRDLELAIAKQLKELIIKTLRKNVALNKFGQQLEAQYTLRV

FIMFAFAAGLLCALSFKAYTNPMANYIYAIWFGAKTVELLSLGKIGTDLAYTTDSLSSMYYLTHWEKINLRLFKLINLAI

EMNSKPFYVTGLKYFRVSLQTGLKILQAAFSYFTFLTSM

>DeleOr42a

LRCIFLMGVRKPPTHFFLAYVWSFALNFCTFYQPIGFLTGLIIHLSSPGEFLTSLQVAFNAWSCSTKVMIVWALVKHFDE

ANVILDEMDKRITEPGERLQVHRAVSLSNRIFFFFMAVYMVYATNTFLSAILIGRPPYQNYYPYDWRSSLHLGLQAGLEY

FAMAGACFQDVCVDCYPVNFVLVLRAHMSIFADRLRRLGTDPAESEEQRESEEQRYERLVECIQDHKVILRFVDCLRPVI

SGTIFVQFLVVGLVLGFTLINIVLFANLGSAIAALSFMAAVLLETTPFCILCNYLTEDCYKLADALFQSNWIGGKKRYQM

TLMYFLQKLQQPITLMAMNVFPISVGTNISVTKFSFSVFTLVKQM

>DeleOr42b1

YRAMKFIGWLPPKEGVLRYLYLFWTLMTSTTYLPLGFLGSYMKSFSPGEFLTSLQVCFNAYGSSVKVAITYSMLWRLIKA

KSLLDQLDLRCTSMEEREKIHRVVARSNHAFLIFTIVYCGYAGSTYLSSVLSGRPPWQLYNPFDWHDGPKLWVASTLEYV

VMSGAVLQDQLSDTYPLIYTLILRAHMDMLRERIRRLRSDETLSEANSYEELVKCVMDHKLILRYCAIIKPVISGTIFTQ

FLLCGLVLGLTLINVFFFSDLWTGIASFMFVITILLQTFPFCYTCNLIMEDCDSLTHALFQSNWVGASRRYKTTLLYFLQ

NVQQPIVFIAGGIFPISMSSNISVAKFAFSVITITKQM

>DeleOr42b2

YRAMWLIGWIPPKEGVLRYVYLFWTCVPFAFGVFYLIISYVQEFKNFGEFLTSLQVCINVYGASVKSTITYLFLWRLRKT

EMLLDALDRRLNGDSDHQKIHNMVARCNYAFLIYSFIYCGYAGSTFLSYALSGRPPWSVYNPFDWRDGLSLWIQAIFEYI

TMSFAVLQDQLSDTYPLMFTIQFRAHMDILKDHVRNLRTDPKRDPKRSEADNYQDLVKCVMDHKMILRWCDMIRPMISRT

IFVQFALIGSVLGLTLVNVFFFSNFWKGVASLLFVITILLQTFPFCYTCNLLIDDAQELANTIFQSNWVDAEPRYKATLV

HFMHHVQQPIIFIAGGIFPISMNSNISVAKFAFSIITIVRQM

>DeleOr43b

TLRFSGLNVRNDFGVGRKIWRILSFTYNLLILPVSNYTIHLAQFPPDLLLQSLQLCLNTWCFSLKFFTLVIYMPRLELAN

KHFDQMDEFCVRSEEKRKVRDMVAAITRLYLIFVVVYVLYATSTLLDGLLHERVPFNTYYPVNWRVDTQLYVQSFLEYFT

IGYAIVVATATDSYPVIYVAALRTHILLLKDRIVHLGEANSESMFSGLVDCIKAHRTMLSFCEAIQPIISGTIFAQFIIC

GSILGVIMINLVLFADQSTRFGIIIYVMAVLLQTFPLCFYCNAIVDDCNELADALFHSAWWMQDRRYQRTVLQFLQKLQQ

PMTFTAMNIFNINLATNINVAKFAFTVYA

>DeleOr46aA

QKSLLNIFSLWPQDEHWRRICHQVYVHVICFWVLLFDLLLVMHVVANLTDMSEVVRAIFVLATSVGHTTKLLSIKANNVA

LEELFQRLDDDDFRPKGADEEVIFAGASDSSRKLRDFYGTLSLAALSMVLIPQFVLDWSHLPLGTYNPFGENPGPGYWFL

YCYQCLALSVSCITNIGFDSLCSSLFIFIKCQLDILVVRLDKMGQPVEQQLKQNIRYHMTIVELTQIIERLLCKPISVQI

FCSVLVLTANFYAIALLSDQKLALFKYITYQACMLSQIFILCYFAGEVTQRSLELPHELYKTSWVDWSRPNRRIVLLFMQ

RLHSTLRIRTLNPLGFDLMLFSIVNCSYSYFALLKRV

>DeleOr47a

LEVQKSTIAWLGFDLFSENREMWRRPYRALNVFGIATIFPFILAAVLHNWKHVMLLADAMVALLITILGLFKFSMILYLR

RDFKRLIDKFRLLMSNESEQEEYAEILNAANKQDQRMCTLFRTCFFLAWLLNSVLPFVRMGLSYWLTGHAEPELPFPCLF

PWDIHIRRNYVLSFIWSAFASTGVVLPAVSLDTIFCSFTSNLCAFFKIAQYKVIRFKGKNLKESQDTLNKIFALKESQDT

LNKIFALYQTSLDMCTDLNLCYQPIICAQFFISSLQLCMLGYLFSITFAQTEGVYYASFIATIIIQAYIYCYCGENLKTE

SANFEFAIYDSPWHESSTSICRSLLISMMRAHHGFRITGYFFEANMEAFSSV

>DeleOr47b

AFNYARACLSLLCQYPNKKLATLSLYRWINVFIMCNVMNAFVTMVFALPESKNVIEIGDDLVWISGMGLVFTKIFYMRLR

CNEIDELIWDFDYYNRELRPNQRDEEVLGWQRMCYLIESGLYMCFCLVNFFSAAVFLQPLLGEGKLPFHSIWPFQWHHPS

MFWFLYIWLSLTSQHNLMSILMVDMVGISTFLQTALNLKLLCIELRKLGEMKAVARFHEEFCRVIRFHQHIIRMVKKANS

AFNASFNAQLVTSFLLISISTFETMAAALDPKMAAKFVLLLMVVFIQLSLWCIAGTLVYTQSLEVAQAAFEINWHTKAPA

IQRDISFVIFRAQQPLMYEAKPFLPFTLGTYM

>DeleOr56a

FRNHLCGFRLYGYVASTDQKRPWFSLVRCIFFTVSLWMCCALMLARVFRGYENLNDGATSWATAVQYFAMSIATLNAIVQ

RDSVIGLLRVAHADIQNLISEADDREMELLAQVYTRNITLTLCGPSFIAGSMAYLDCIYRTVFLPKSNSSAVQRGEEQPI

LMFFPLEVCDNFVVGYIGAWYALSLGMTAIPMWHTFITCLMKYVNLRLQILNKRVEEMDKQLFRDFVEEHIRIRNLVHEI

QHLIRVPVMLDFIIFSISMCFLFYALALGVSSKMDYFFIGIYIFVMAAILWMYHWHATLIVECNDKLSFAYFSCGWYNFD

VPLQRTLLFVMMHAQRPMNMRALLVELNLRTFIDIMRGAYSYFNLLRS

>DeleOr59a

FFRSHWTTWRVLGVAQFRVQSWRNLYLSYSILLNLMVTLGYPFHLGMSLFRNRTLTEDILNLTTFATCTACSVKCLLYAY

RIKDVLEMERLLRLLDKRVVGPEQREIYGQVRVQLRNVLYIFISIYLPCALFAELSFLFKVERGLMYPAWFPFDWLNSRN

YYIANVYQIVGISFQLLQNYVSDCFPAVVLCLISSHIKMLYKRFEEVGMNPERELEACITDHKHLLKLFRRVEGFISLPM

FIQFTVTALNVCIGIAGLVFFVTEPMARMYFIFYSLAMPLQIFPSCYFGTDNEYWFGRLHYAAFSCNWPTQNRSFKKKMM

LFVEQSLKKNTAVAGGMMRIHIDTFFSTLKGAYSLFTIIIRMR

>DeleOr59b1

YRAMWLIGWIPPKEGVLRYVYLFWTCVPFAFGVFYLIISYVQEFKNFGEFLTSLQVCINVYGASVKSTITYLFLWRLRKT

EMLLDALDRRLNGDSDHQKIHNMVARCNYAFLIYSFIYCGYAGSTFLSYALSGRPPWSVYNPFDWRDGLSLWIQAIFEYI

TMSFAVLQDQLSDTYPLMFTIQFRAHMDILKDHVRNLRTDPKRDPKRSEADNYQDLVKCVMDHKMILRCCDMIRPMISRT

IFVQFALIGSVLGLTLVNVFFFSNFWKGVASLLFVITILLQTFPFCYTCNLLIDDAQELANTIFQSNWVDAEPRYKATLV

HFMHHVQQPIIFIAGGIFPISMNSNISVAKFAFSIITIVRQM

>DeleOr59b2

YRAMKFIGWLPPKEGVLRYLYLFWTLMTSTTYLPLGFLGSYMKSFSPGEFLTSLQVCFNAYGSSVKVAITYSMLWRLIKA

KSLLDQLDLRCTSMEEREKIHRVVARSNHAFLIFTIVYCGYAGSTYLSSVLSGRPPWQLYNPFDWHDGPKLWVASTLEYV

VMSGAVLQDQLSDTYPLIYTLILRAHMDMLRERIRRLRSDETLSEANSYEELVKCVMDHKLILRXCAIIKPVISGTIFTQ

FLLCGLVLGLTLINVFFFSDLWTGIASFMFVITILLQTFPFCYTCNLIMEDCDSLTHALFQSNWVGASRRYKTTLLYFLQ

NVQQPIVFIAGGIFPISMSSNISVAKFAFSVITITKQM

>DeleOr59c

DYYYRITFVLGLTPPKEGLARWIYFLWSAIVMWLGIVYLTYVLHFDRFTPTEFLSSLQVDINCIGNVVKSFVTFSQMWRL

RRMNELIAPLDARCDTPSQRQILHKVVARVNLTVFIFVSMYLGFGFLNVFTSVLAGKAPWQLYNPFDWQNGWQLWIASFL

ECFVVSIGTMQELISDAYPIVFVSLFRGHLAVLKNRIENLRQDPELSEEENYRQEEENYRQLVACIQDHRTIVEXAQVIR

PILSITIFAQFMLVGIDLGLAAISILYFPNTIWTIMANVSFILAICMESFPCCMLCEHLIDDCAHVSDALFHSNWISAEK

RYKSTVIYFLHRVQQPIQFTAGSIFPISVQSNIAVAKFAFTIITIVNQM

>DeleOr63a

KKRNYRSIREMIRIAYTVGFNLMDPSRCGQVLRMWTIVLSLSSLASLYGHWQMLARNIHDIPRIVETVSTAFQFLSSIAK

MWYFLFAHRQIYDLLRRARSDLPVIKTIREKESTMDRYWANTRRQLLVYLYCCICITTNYFINSLATTLYRHFTRPKGSY

EIVLALPSLYPGWAEKGPYYYIQMYLETCSLYICGMSAISFDGVFIVLCLHSVGLIESLNQMIDYLRCCIYQYQRVASFA

EEINDCFRQVTFSQFLLSLFGFGLALFQMSVGSNSSIILFRMTLYLIAGGYQIVVYCYNGQRFTTASEQIGKAFYEVQWY

GESREFRQLIRMVLMRTNRGFRLDVSWFMQMSLPTLMAMVRTSGQYFLLLQNV

>DeleOr65a

YTNSEERRVTRNVWHYYIVIQLAVSLASMCYGVLESIDNIAILGRDLVFIISVIFIFFRLIFFAQYANDVDAVIDALDDI

HHCETKGPGRKEVQATKRFHFLLYMALIVAWFTFVSFILIKISTPFWMESVTLPFHVAWPFDPSKHPAYCLIFVSQSTTL

TYFLVWLGAVENMAVSIFFELTSSLRVLCIELRSLQEGDESLLERELHRLTKFHQRIILLSDHCNNIFNRTFIMQMLVNF

FLVSLSLFEVLVARKNPQVAAEYLIIMLMTLGHLSFWSKFGDMLSEESAEVAEAVYEAYDPTGSINIHQHFRFFILRAQK

PLIMRASPFPPFNLVNYMFILKQCYSILTVL

>DeleOr65b

TSSEERQRPYRSLWHTLVLIQMTIFFASLWYGLTESIGNSVQMGRDLAFIIGTFFIVFKVYYFHWYGDALDEVITDLEAF

HPWSRKGPGAVDIRAGKRWYFLMSFFLGSFWSFFLCIFVVLLMTSPMWVQQQKLPFHAAFPFQWHTHPTHAIIYLFHCLL

MTYAIVWLLCIEGLSVCIYAELTFAIEVLCLELRHLSREEMRLETRRLVRFHQKIIEILDRANDVFHGTLIMQMAVNFLL

VSLSVLKAMEARKDPKVVGQFGVLMVLALGHLSMWSLFGDRLSQESLQVSEAAYEAYDPTGSKEVYRDLCFIIRRAQYPL

IMRASPFPSFNLINYAAILNQCYGILTFLLK

>DeleOr65c

TSSEERQRPYRSLWHTLVLIQMTIFFASLWYGLTESIGNSVQMGRDLAFIIGVFFIVFKVYYFHWYGDALDEVITDLEAF

HPWSRKGPGAVDIRAGKRWYFLMSFFLGSFWSFFLCIFVVLLMTSPMWVQQQKLPFHAAFPFQWHTHPTHAIIYLFHCLL

MTYAIVWLLCIEGLSVCIYAELTFAIEVLCLELRHLSREEMRLETRRLVRFHQKIIRILDRANDVFHGTLIMQMAVNFLL

VSLSVLKAMEARKDPKVVGQFGVLMVLALGHLSMWSLFGDRLSQESLQVSEAAYEAYDPTGSKEVYRDLCFIIRRAQYPL

IMRASPFPSFNLINYAAILNQCYGILTFLLK

>DeleOr67b

LSSILLNNWQIIDRQVMFFFKIVCMPVLYYTMRPYFQYIYDCYFVKDTCEMTLSYPAIVPYGNYEFPSYVIRFFLLQSGP

LWCFFAVFGFNSLFVVLTRYESGLIEVLRFLVQNSTSDVLVPKDQRDKYLQCCVRLFARDKYLQCCVRLFARISSHHFQI

ENLFKYIILVQCSVSSILICMLLYKISTVEVGWVWMGMIMVYFVTIALEITLYNVSAQKVETQSELLFLDWYNCKWYNES

KEFRFMIKMMLLFSRRTFVLSVGGFTSLSHKFLVQVFRLSANFFLLLR

>DeleOr67c

NSVRTFMDMMRVPVLFYRTIGEDIYAYRSTKSLLLKIYLYAGFINFNLLVIGELVFFYKSLQDFETIIAVAPCIGFSLVS

DFKQAAMMTGKESLIMLLEDLEDMHPKTQQKQKAYKLLDFEKTMKRVINIFTFLCLAYTTTFSFYPAIKASVKFNFLGYD

TFDRNFGFLIWFPFDATSNLIYWITYWDIAHGAYLAGIAFLCADLLLVVVITQICMHFSYISRRLEEHDEDRENIEFLVG

IIRYHDKCLKLCERVNDIYSFSILLNFLMASMQICFIAFQVTESTVEVIIIYCIFLMTSLVQVFLVCYYGDMLIAASLKV

GDAAYNQKWFQCSKTYCSMLKLLILRSQKPASIRPPTLPPISLVTYMRVISMSYQFFALLRT

>DeleOr67d

ERYRKVIRMIRFCVGFCGNDVADPNFRMWWLTYTVIGAICFFFACTGYTIYVVVIKGDLTVILQALAMVGSAVQGLTKLL

VTANMAPQMREIQNTYEEIYREYGAKGEYAKCLERRIRTTWQLFIGFMLVYIILLGLIICFPIFYLVVLHKKVLVMQFLM

PFNHNTDGGHLVLTAVHVALITFGGFGNYGGDMYLFLFVTHVPLIKDIFSLKLKEFNEYPRIRAMLCDLLTWHQLYSRIL

QTTKRIYSIVLFVQLSTTCVSLLCTISCIFIKSWPAAPLYLLYAAIILYTFCGLGTLVENSNEDFLTVIYTCLWYELPVK

EEKLLIMMIAKAQKEVCLTAADMAPLSMNTALQLTKGIYSFSMLLMN

>DeleOr69aA

RKPYRLEQKALFVLAVICAMYQIFGVIIYWYRNGRDVTEISEMCGSLMLTTVGFGNIYALIKPRNLIENMFEELEEIYPS

QSDEHYRCQLYYDLAMAIMKIEFMFYMVFYAYYNSAPVLLLLWENLQEGQELSFKTQTNTWFPWKVHGSLGFGGAVLCLI

LGSFVGVGFSIATQNLIVIFTFQLKLHYDGLESKLLHLDSRQPNANQQPNANQQLRDLIAYHSRILHIGDQFNHILNFVF

GTSLVGSTIAICMLSVAISLLDAASAIKYVSGLTAFVLYHFVICYMGTEVT

>DeleOr69aB

VNRTLAQRFFFWFGALNLVYHNIGLIMCARFVDGSVSELTDIGAMLGFTTMGTLNLWKIMRCKPEIEKLMEEFEALFQQA

KKRSYRIQHYYEAHTRLIKKSVRFYAPSICYYNLLPIILMVVELLTDNKQLSYRIQSSAWYPWKVHGSSGFFAAVVCQAF

SCQIDLGIIIFTQFLISFFGIQLEIQFDGLARQLEAIDAKEQLKHLLAYHIKLFNLADRVNHSLNFTFCVSFTVSILSMC

FQGISVIMGDLGPALKHMLGLFVFLVYNFSICRNGTHIXXXSDKVMPAAFYNNWYEGDLAYRKMILILMIRATKPYMWRT

YKLAPVSITTYMAV

>DeleOr71a

PVRRLTGILSWWSLWPSVARPNWNSWQGYLLHVPFTLLFVVLLWVEALMSRDIEHTADVLLICLTTTALGAKTVNNWKYA

HVAQAILTEWSTSDLFELRCKQEVDVWKFEHRRFSRVFICYAMCSVGVIPFVVIKCLFDIPNQLPFWMWMPFDFQQPGLF

WLPFIYQAITIPVICICNITMDAVNWYMMLHLSLCLRMLGKRLSSLRHDDEKLRDDEKLREKFVELVHIHKRLKQQALDI

ETFISKSTFTQILVSSLTICFTIYSVQMSMQDLGKFAGMIQYLLSMIMQIMLPSIYGNGVIDSASVLTQSMYNSDWPDMN

AEMRRLVLMFMVYLNRPMSLKAGGFFRVGLMLFTKTINQSYSLLALLLNM

>DeleOr85f

RLPRFVVGLMGYDMLGVTPTRRILMRFYRFICLASHSVCVGFMIFRMVETIDNVSLIMRYATLVTYVINSDTKYATVLQR

RAIQSLNSKLADLYPKTTLDRIYLQVNDHYWSKPFVYLVTIYIGSSSLVVVGPIITSLISYFTHRGFAFMHCYPYFQFDP

EKHWIYVGIYVLEWLHSTQMVISNIGADIWLLYFQIQIILHFRGIIQSLEDKDRKFLAQIVDKQVYLVELQNELNEIFGG

SLLLSLLTTASVLCTVAVYTLIQGATLEGCSYVLFIGTSTMQVYLVCYYGQQVLDLSAQVAHAVYNHDFHNASIAYKKYL

MIIIIRAQQPVELNAMGYLSISLDTFKQLMSVTYRVITMLRQM

>DeleOr92a

TFDELTSFPMTFYKTIGEDLYSARDTNLVRRYLLRFYLVLGFLNFNAYVIAYFIMSTTTLLEATAVAPCIGFSFMADFKQ

FGLTVNKQRLVNLLDNLKEIYPMDVESQRKYEVPYYQKHMGRVMNLFTILCMTYTSSFSFYPAIKSTIKYYLMGSEIFER

NYGFHILFPYDAETDTVYWFSYWGLAHCAYVAGVSYVCVDLLLITTITQLTMHFNFIANDLEAYDGGELHNLVVYHSRAL

ELSEEVNTIFSFLILWNFIAASLVICFAGFQITASNVEDIVLYFIFFSASLVQVFVVCYYGDEMISSSSRIGHSAFNQNW

LPCSTKYKRILKFIIARSQKPASIRPPTFPPISFNTFMKVISMSYQFFALLRT

>DeleOr98a

AFQYFEYGMSVLGWMAPPKYQFVYSMMAFFLISWCVYYLPIGIISFVIDIKNPSELLTVLQLFFNSVGTPLKILILRVHL

WRFREAKNLLIQMDNRCTAIVERLEVHKWVVNCNKAYLIYICMYIGYTLSTFLSAAFSGVLPWRIYVPFDFRHSTSFWMA

ATHETLLMLFSVSQTLLTDIYPVLYGLMLRVHIRLLRMRVEKLCTDPDKSENENDKSENENMEDLISSIKDHKLIHECAQ

MIHPVIARTILVQFLLIGLPLGFSMINLFYFADLWTGLATVAYINGLMMQTFPFCFLCDLIKSDCELLQIAIFHSNWLNT

SRRYKTSLIFFCLTPKSMLFLQLAQFFPFPRAQTLRMAKLAFSVFTLVSQ

>DereOr2a__scaffold_4644_residues_2106911_to_2109167_forward_strand.[501_1685].sp

QEESELDTHSAVYYHWRVWELTGLMRPPGVANLRYLVYSITVNLVVTVLFPVSLLARLLFTTNMAGFCENLTITITDIVA

NLKFANVYMVRKQIRSLLRLMDARARLVGDPEEISALRKEVNIAQSTFRTFGSIFVFGTILSCIRLVVRPHRELLYPAWF

GVDWMHSRNYVLITIYQLFGLVVQAIQNCASDSYPPAFLCLLTGHMRALELRVRRIGSRKENSTEGQSEEVYQELVECIR

DLMRVHRLREIIQRVLSAACMAQFACSAAVQCSVAMHFLYMEHDHTAMIISIVFFLAVTLEVFVICYFGDRMRTQSEALC

DAFYDCNWVEQLPKFKRELLFTLARTQRPSLIYAGNYIALSLETFEQVMRFTYSVFTLLLRA

>DereOr7a_scaffold_4690_residues_10433363_to_10435763_reverse_strand.[501_1901].sp

EEKAPESRRAFRNLFNCFYALGMQAPDGPTTSSTWRRIYCCFSVVMYVWQLLLVISYRYMGGMEITQVLTSAQVAIDAVI

LPAKIVALAWNLPLLRRAEHHLAALDARCKEQGEFQLVLDAVRFCNYLVWFYQICYAIYSSSTFVCAFLLGQPPYALYLP

LDWQRSMQFWIQAWIEFLIMNWTCLHQASDDVYAVIYLYVVRTQVQLLARRVEKLGRDDTPDERRQEEHCAELQRCIVDH

QTMLQLLGCISPVISRTIFVQFLITAAIMGTTMINIFIFANTNTKIASIIYLMAVTLQTAPCCYQATSLMLDNEQLALAI

FQCQWLGQSARFRKMQLYYLHRAQQPITLTAMKLFPINLATYFSIAKFSFSLYTLIKGM

>DereOr9a___scaffold_4690_residues_4776380_to_4782346_forward_strand.[501_5467].sp

SDEVQGKKEEEKDQSLRVQILVYRCMGIDLWSPTMENDRPWLTFVTMGPLFLFMVPMFLAAHEYITQVSLLSDTLGSTFA

SMLTLVKFLLFCYHRKEFVGLIYHIRAILAKEISPDARDIIEVENQSDQLLSLTYTRCFGLAGIFAALKPFVGIILSSIR

GDEIHLELPHNGVYPYDLQVVMWYVPTYLWNVMASYSAVTMALCVDTLLFFFTYNVCAIFKIAKHRMIHLPEGLVQVLRL

HQKGLQIADHIADKYRPLIFLQFFLSALQICFIGFQVADLFPNPQSLYFIAFVGSLLIALFIYSKCGENIKSASLDFGNG

LYESNWTDYSPPTKRALLIAAMRAQRPCQMKGYFFEASMATFSTIVRSAVSYIMMLRSF

>DereOr10a__scaffold_4690_residues_3845062_to_3847510_forward_strand.[501_1949].sp

DQQLDYFFAVPRLSLNIMGYWPIGDKLPRRALIHFVILAIGVATELHAGMRCLGQQQITLALETLCPAGTSAVTLLKMFL

MLRFRQDLSTMWHRLRCLLFDPNWSEQRDIRLRCSAMAARINFWPLSAGFFTCTTYNLKPVLIALVLWLQNRCEDFVWFT

PFNMTMPQVLLSSPFFPLTYIFTAYTGYVTIFMFGGCDGFYFEFCAHLSALFEVLQAEIESIFRLERKMRSVIIRHNAII

NLARFFRERYTIITLAHFVSAAMVIGFSMVNLLAMGNNGLGALLYVAYTIAALSQLLVYCYGGTLVAESSTELCRTMFAC

PWQLFKPQQRRLVQLLILRSQRPISMAVPFFSPSLATFAAILHTSGSIIALVKSF

>DereOr19a__chrX_residues_11385684_to_11387968_forward_strand.[501_1785].sp

KVDSMKALASHWRIFRIIGVYPPARTTVWGRHYTAYSVVWNVVFNACLWVSFLLQSNSLQTFCESFCLTLPHTIYLLKLT

NVRRMRDGLIRSHQVLRHLDSRLGSSDERQILKAGIDHAMLIFRTILTGVVSTLSVGIIYMAVSSEPTVMYPSWIPWNWR

DSWTYLSTVLLHTSAIFANAATVLNLTTYPGTYLILVSAHAKALALRVSKLGYDTSVILIGYIHDHQSILVLFKSLERCL

SMTCFLQFFSTACAQCTICYFLLFVKVGIMRIMNMMFLMAAITTETLLLCYSAELLCKEGDNLLAAVYSCNWMSQPVKFR

RLLLLMLAHCQKPLILVSGVVVPLSMETFMVVIKGAYTMLTLLTEIR

>DereOr23a__scaffold_4929_residues_2698654_to_2700849_forward_strand.[501_1696].sp

YDYFRLQLNAWRICGALDLSEGRYWSWSVLLCILVYLPAPMLLKGVYSFEDPVENNFSLSLTVTSLSNIMKLSMYVAQLT

KLVEAQSLIGQLDARVSGENQSVRHREMTAHLHRMSKLFQITYALVFIFAAVPFAFKSELSLPMPMWIPFDWKNSVAYIG

VLVLQEIGFFFQIMQCFASDSFPPLILYLISEQCQLLILRISEIGYGSKTLEENEQEENEQELINCIRDQNALYRLLDVT

KSLISYPMMVQFMVIGINIAITLFILIFYVETLYDRIYYLCFLLGITVQTFPLCYYGTMVQESFAELHYAVFCSNWVDQS

ASYRGHMLILAERTKRTQLLLAGNLVPIHLSTYVACCKGAYSFFTLM

>DereOr24a__scaffold_4929_residues_4230966_to_4235452_reverse_strand.[1501_2987].sp

RHYFMVPKFALSLIGFYPEQKRTVLVKLWSFCNFCILTYGCYAEAYYGIHYIPNIATALDALCPVASSILSLIKMVAIWW

YRDELSSLIQRVRFLTEQQKSKRKLGYKKRFYTLATRLTFLLLCCGFCTSTSYSVRHLMDNILRTAHGKDWIYETPFKMI

FPDALLRLPLYPITYILVHWHGYITVVCFVGADGFFLGFCLYFTVLLHCLQDDVVDLLEVENMENMEMSLSEADEIRIVR

EMENLVDRHNEIAELTERLSGVMVEITLAHFVTSSLIIGTSVVDILLFSGLGIIVYVVYTCAVGVEIFLYCLGGSHIMEA

CSNLARCTFSSHWYGHSVRVQKMALLMVARAQRVLTIKIPFFSPSLETLTSILRFTGSLIAL

>DereOr30a__scaffold_4929_residues_9718175_to_9720653_forward_strand.[501_1979].sp

TLKLMKFWSYLFVHNWRRYVAMAPYIIINCTQYVDIYLSTESLDFIIRNVYLAVLFTNTVVRGVLLCVQRFSYERFINIL

KGFYIELLKSDDPAISHLVGTRLSLFISRINLLMGCCTCIGFVTYPIFGSERVLPYGMYLPIDEYKYPYYEIFFVIQAIM

APMGCCMYIPYTNMIVTFTLFAILMCRVLQHKLRSLEKLEQVRGEIIWCIKYQLKLAGFVDSMNALNTHLHLVEFLCFGA

MLCVLLFSLIIAQTIAQTVIVIAYMVMIFANSVVLYYVANELYFQSFDIAIAAYESNWMDFDVDTQKTLKFLIMRSQKPL

AILVGGTYPMNLKMLQSLLNAIYSFFTLLRRV

>DereOr33a__scaffold_4929_residues_13479185_to_13483391_reverse_strand.[1501_2707].sp

ESLYKTYWLYWRLLGVEGDYPFRRLVDFTITFFITILFPVHLILGMYKKPKVQVFRSLHFTSECLFCSYKFFCFRWKLKE

IKAIEGLLQDLDNRAESEEERNYFDRNPSRVARMLSKSYLVAAISAIITATIAGLFSSGRNLMYLGWLPYDFQATLTYWL

SFTYQAVGSSLLILENLANDSYPPITFCVVTGHVRLLVMRLSRIGHDIRVSRSENTKKENTKKLIEGIQDHRKLMQIVRL

LRSVLRLTQLGQFLSSGINISITLINILFFAENHFAMIYYAVFFAAMLIELFPSCYYGTLMTMEFDKLPYALFSSNWLKM

DKRYNRTLIILMQLTLVPVNIKAGGIVGIDMSAFFATVRMAYSFYTLALSFR

>DereOr33b__scaffold_4929_residues_13474925_to_13481762_reverse_strand.[1501_5338].sp

LESHFFLNRLLDMVITAFVTIWYPIHLILGLFMERSLGDVCKGIPITAACFFASFKFICFRIKLSEIKEVEILFKELDQR

ALSQEECEFFNQNTRREANFIWKSFIAAYGLSNISAIASVLFGGGHKLLYPAWFPYDVQASLIYWLSVTYQIAGVSLAIL

QNLANDSYPPMTFCVVAGHVRLLAMRLSIIGQGPEETKETKYSIAKQLIEGIEDHRKLMKIVELLRNTMNISQLGQFISS

GVNISISLINILFFAENHFAVIYYGVYFLSMVLELFPCCYYGTLISVEMNQLTYAIYSSNWMKMDRVYSRTLLIFMQLTL

AEVQIKAGGMIGIGMNAFFATVRLAYSFFTLAMSLR

>DereOr33c__scaffold_4929_residues_13473279_to_13477494_reverse_strand.[1501_2716].sp

CPRTVQLYVVLLNILVTLWFPLHMLLHLLLRPSAADFMKNLTISLTCLACSLKHVAHLYHLPQIVEIESLIGQLDTFIAS

DQEHRYYQDHVYCHARRFTRCLYVSFGMVYAVFLFGVFAQIIGRKWELIYPAYFPFDFENNSLGAVAMGYQVMSLIVEGF

QGLGNDTYTPLTLCFLAGHVHLWSIRMSQLGHFEDETATDHQRLLEYHQRLLEYIEQHKLLVRFHRLVRRTISEVQLVQL

GGCGATLCIIVSYMLFFVGDTISLVYYLVFFGVVCMQLFPSCYFASEVAEEVERLAYAIFSSRWYDQSRDHRFDLLVFTQ

LTNRGWVIMAGGLIELNLNAFFATLKMAYSLFAVVVR

>DereOr35a__scaffold_4929_residues_7365108_to_7369528_reverse_strand.[1501_2921].sp

LSWPLAIFRLNHIWPLDPGQWGRFLDKIMAVAMSLVFVQHNDAELRYLRFSNRNLDAFLTGMPTYLILVEAQFRSLHILM

HFEKLQKFLEKFYANIYIDPRKEPEMFRKVDGRLIINRLVAMYGAVISLYLIAPIFSIINQSKDFLYSMIFPFNSDPLYI

FVPLLLTNVWVGVVIDSMMFGETSLLCELIVHLNGSYLLLKRDLQLAIEKINQLKVLIIKTLRKNVALNHFGQQLEDQYT

VRVFIMFAFAAGLLCALSFKAYTNPMANYIYAIWFGAKTVELLSLGQIGSDLAYTTDSLSSMYYLTHWEQINIRLLKLIN

LAIEMNSKPFYVTGLKYFRVSLQVFSQILQASFSYFTFLTSM

>DereOr42a__scaffold_4929_residues_20666215_to_20669174_forward_strand.[501_2460].sp

LRCVFLMGVRKPPAKFFVAYVLWSFALNFFTFYQPIGFLTGYHLSEFSPGEFLTSLQVAFNAWSCSTKVLIVWVLVKRFD

EANAILDEMDRRITEPEERLQIHRAVSLSNRIFFFFMAVYMIYATNTFLSAIFIGRPPYQNYYPFDWRSSLHLALQAGLE

YFAMAGACFQDVCVDCYPVNFVLVLRAHMPIFEKRLRRLGTSPAESQQESQQQRYERLVECIQDHKVILRFVDCLRPVIS

GTIFVQFLVVGLVLGFTLINIVLFANMGSAIAALSFMAAVLLETTPFCILCNYLTEDCNKLGDALFQSNWIDGEKRYKNA

LMYFLQKLQQPITFMAMDVFPISVGTNISVTKFSFSVFTLVKQM

>DereOr42b__scaffold_4929_residues_20659150_to_20661460_forward_strand.[501_1811].sp

LYRAMKFIGWLPPKQGVLRYVYLTWTLMTFVWCTTYLFLGSYMTQISPGEFLTSLQVCINAYGSSVKVAITYSMLWRLIK

AKNILDQLDLRCTAMEEREKIHRVVARSNHAFLIFTFVYCGYAGSTYLSSVLSGRPPWQLYNPIDWHDGLKLWVASTLEY

MVMSGAVLQDQLSDTYPLIYTLILRAHLDMLRERIRRLRSDETEAESYEELVKCVMDHKLILRYCAIIKPVISGTIFTQF

LLIGLVLGLTLINVFFFSDIWTGIASFMFVITILLQTFPFCYTCNLIMEDCESLTHAIFQSNWVDASRRYKTTLLYFLQN

VQQPIVFIAGGIFQISMSSNISVAKFAFSVITITKQM

>DereOr43b__scaffold_4929_residues_18821071_to_18823645_forward_strand.[501_2075].sp

LRNSGLDVDNDFGIGRKIIRFFSFTYNMVILPISNYVIYRSEFPPELLLQSLQLCLNAWCFALKFFALIVYMNRLELANK

HLDELDEYCVKPAEKRKVREMVAAVSRLYLIFVAVYGFYATSSLVDGLLHNRVPYNTYYPFNWRVDAQLYIQSFLEYITV

GWAIYGSSATDSYPVIYVAALRTHILLLKDRIIYLGDPSNEGLKTLVDCIKAHRTMLNFCDAIQPIISVTIFAQFIICGS

ILGVTMINMVWFADQSTRFGIVTYVMAVLLQTFPLCFYCNAIVDDCNEVADALFHSAWWVQNKRYHRTLIQFLQKLQQPM

TFTAMNIFKINLATNINVAKFAFTVYA

>DereOr45a__scaffold_4929_residues_17425322_to_17429524_reverse_strand.[1501_2703].sp

SYFAVQRRALEIAGFDPSTPQLNLQHPIWAGILMLSLVSHNWPMAFYALQDLSNITRLTDNLAVSLQGVQSTFKFLIIVL

KRRRIGSLIHRLHKLNEAASATPKHLEKIERENQLDRYVSRSFRNAAYGVVCASALAPMLLGLWEYVETGVFTPTTPVDF

NFWLDERKAQFYWPIYAWGVLGVAAAAWWAIATDTLFSWLIHNVVAQYQLLELALEEKDQQNGADCRLVECVRRHRIALD

LAKELSAIFAEIVFVKYMLSYLQLCMLAFRFSRSGWSAQLPFRATFLLAIIIQISSYCYGGEYLKQQSLGIAHAVYNSNW

PKMAPRQRRLWQMVIMRAQRPARIFGFMFDVDLSLLLWVIRTAGSFLALLRTF

>DereOr45b__scaffold_4929_residues_8907843_to_8911150_reverse_strand.[446_1908].sp

LFFVTRYSFGLLGLRFGKELSWLHLSWLVFNFVNLAHCCQAEFVFGWLHLRSPVDAMDAFCPLACSFTTLFKLGWMWWRR

HEVADLMERIRLLIGKQEKREDSRRNVAQRSYYLMVTRCGMLVFTLGSITTGAFVLRSLWEMWVRRHQEFKFDMPFRMLF

HDFAHRMPWFPVFYLYSTWSGQVTVYAFVGTDGFFFGFTLYIAFLLQALRYDIQDAVRESNICCQRLEDIVDRHNEIEQI

VKEFSGIMAAPTFVHFVSASLVIATSVIDILLYSGYNIIRYVVYTFTVSSAIFLYCYGGTEMSTESLSLGETAYSCAWYT

WDREVRRRVFLIILRAQRPMTVRVPFFAPSLPVFTSVIKFTGSIVA

>DereOr46aA__scaffold_4929_residues_8341558_to_8347182_reverse_strand.[1501_4125].sp

QKAFLNIFSLWPQNERLWRIIYVHVIVFWVLLFDLLLVLHVVAHLGYMSEIVKAIFILATSAGHTTKLLSIKANNVPMEE

LFSTLDDEAFRPRGDEEEVIFAAACERSRKLRDFYGALSFAALGMILIPQFALDWSHLPLKTYNPLGENPGPAYWLMYCY

QCLALSVSCITNIGFDSLCTSLFIFIKCQLDVLAMRLDKIGRLSVEQQLKENIRYHMSIVQLSKTVERLLCKPISVQIFC

SVLVLTANFYAIAVLSDERLELFKYVTYQACMLIQIFILCYYAGEVTQRSLDLPHELYKTSWVDWDYRSRRIALLFMQRL

QSTLRIRTLNPLGFDLMLFSIVNCSYSYFALLKRV

>DereOr46aB__scaffold_4929_residues_8341558_to_8347182_reverse_strand.[1501_4125].sp

SQHQTVTDFYKYQVWYFQILGVWQLPTADHQRRFQSMRFGFILVILFIMLLLFSLELLNNISQVREILKVFFLFATEISC

MAKLLHLKLKRRKLAGLVDMMLSPEFGVKSEQERQMLEADRVAVVRMRNFYGIMSLGAAALILIVPCIDNFGELPLTMMK

VCNIEGWICYWLQYLFHSICLLPTCVLNITYDSVAYSLLCFIKVQLQMLVLRLQKLGPVIEPEDNIEPEDNEKIAMELRE

CAAYYNRIVRLKDLVELFIRGPGSVQLMCSVLVLVSNLYDMSTMNGDAIFMVKISIYQLVMLWQIFIICYASNEVTVQSS

RLCHSIYSSQWTGWNRPNRRIVLLMMQRFNSPMLLTFNPTFAFSLEAFGSIVNCSYSYFALLKRV

>DereOr47a__scaffold_4845_residues_18660392_to_18664710_reverse_strand.[1501_2819].sp

SFLQVQKSTIALLGFDLFSDDREMWKRPYRAINVFGIAAIFPFILAAVLHNWMNIMQLADAMVALLITILGLFKFSMILH

LRRDFKRLIDKFRLLMSNEADQGKEAEILDAANGQDQRMCTLFRTCFLLAWALNSVLPFVRMGFSYWQTGHVEPELPFPC

LFPWDIHIIRNYALSFVWSAFASTGVVLPAVSLDTIFCSFTSNLCAFFKIAQYKVVRFRGRSLKESQATLNQVFALYQTS

LEMCNDLNQCYQPIICAQFFISSLQLCMLGYLFSITFAQTEGVYYASFIATIIIQAYIYCYCGENLKTESALFEWAIYDS

PWHESSTSICRSLLISMMRAHRGFRITGYFFEANMEAFSSIVRTAMSYITMLRSF

>DereOr47b__scaffold_4845_residues_18587080_to_18589701_forward_strand.[501_2122].sp

AFNYVRAFLCLLGQYPNKKLASLSLYRCINWFIMCNVMATFWAMFVALPESKNVIEMGDDLVWISGMALIFTKIFYMHLR

CNEIDELIWDFDYYNRELRPHNTDEEVLGWQRLCYVIESGLYINCFCLVFFSAAIFLQPLLGEGKLPFHSVYPFQWHHPY

TFWFLYIWQSLTSQHNLMSILMVDMVGISTFLQTALNLKLLCIEMRNLGDMTDKRFHEEFCRVVRFHQHIIMLVGKANRA

FNGAFNAQLMASFSLISISTFETMAAAVDPKMAAKFVLLMLVAFIQLSLWCVSGTLVYTQSLEVAQAAFDINWHTKSPAI

QRDISFVILRAQKPLMYVAEPFLPFTLGTYMLVLKNCYRLLAVMQ

>DereOr49a__scaffold_4845_residues_17513193_to_17517536_reverse_strand.[1501_2844].sp

FEDFIYMANMMFKTLGYDLFDTSRCSNLLLRGYFVLCTTSSFYEASMVTIRIIQWESSPSKIMRQGLHFFYMLSALVKFV

TFVINRKRLLQLCNHLKELYPHKAQTQRKYEVNRYYLSRATQYVLYVYYFVMVIMALGPLLQSCIMYLLGLGKAEFTYKR

IYPTRLTFDSEKPLGYVVAYVIDFTYSQFIVNVSLGTDLWMMCISTQISMHLGYLAKVLASEQQDCYFLARIVKRHQLML

SLHKDVNQVFGLLLASNLFTTASLLCCIAYYTIVQGFNWEGISYMALFVSVVAQFYMVSSQGQKLIDLSTNIAYAAYESK

WYEGSVRFKKDILLLMTQAQRPLEISARGIIIVSFDSFKILMTITYRFFAVIRQ

>DereOr56a___scaffold_4845_residues_9820235_to_9824974_reverse_strand.[1501_3240].sp

FGMHLRCFQWYGYVASKDQTRPLLSLIRCTILTASIWLSCALMLARVFRGYENLNDGATSYATAVQYFAVSIATFNAYVQ

RDRVISLLRVAHSDIQNLMLEADSQELLFATKAYTRSITLLIWVPSVIAGLMCIYRTLFMPKSVFNVPAVRRGEEQPILL

FQLFPFELCDNFVVGYLGPWYALGLGITTIPLWHTFITCLMKFVNLKLQILKKRVEEMDFKEFVREQLRIRKFIQELQYL

ICVPVMADFIIFSVLICFLFFALTVGVPSKMDYFFMFIYLFVMAGILWIYHWHATLIVECHDELSLAYFSCGWYNFEMPL

QKMLVFMMMHAQRPMKMRALLVDLNLRTFIDIGRGAYSYFNLLRS

>DereOr59a____scaffold_4845_residues_20696554_to_20700745_reverse_strand.[1501_2692].sp

EFFKSHWTAWRVLGVASLRIENWKQLYVCYSILANVLVTLCYPVHLGMLLFRNRTLTEDILNLTTFATCTACSVKCLLYA

YNIKDVLEMERLLRLLDERVAGPVQRGIYGQVRVQLRNVLYVFIGIYLPCALFAELSFLFKEERGLMYPAWFPFDWLHSR

NYYIANAYQIVGITFQLLQNYVSDCFPAVVLCLISSHIKMLYKRFEEVGAEKDLEACITDHKHILELFRRVEAFISLPML

IQFTVTALNVCIGIAALVFFVSEPMSRIYFIFYSMAMPLQIFPSCFFGTDNEYWFGRLHYAAFSCNWHNQERSFKRKMML

FVERSLKRSTAVAGGMMRVHLDTFFSTLKGAYSLFTIIIRMR

>DereOr59c____scaffold_4845_residues_20730247_to_20734522_reverse_strand.[1501_2776].sp

YRIALFMGWTPPKEGLLRWIYCLWTLTTMWLGIVYLCLTYVFDRFTPTEFLTSLQVVINCSGNVIKTCATYSQMWRFRRM

NELMSSLDQRCVTPSQRRTFHRMVARANLIVMLFLSTYLGFCFLNLFTSVFAGKAPWQLYNPFDWRHGWQLWVASVLEYC

VVSIGTMQELISDTYAIVFISLFRCHLAILADRIAHLRQDPRSEWEHYEQLVACIQDHRTIIQCAHIIRPILSITIFAQF

MLVGIDLGLAAISILFFPNTIWTIMANVSFIVAICTESFPFCILCEHLIQDSVHVGNALFHSNWIAADRRYKSAVLYFLQ

RAQQPIQFTAGSIFPISVQSNIAVAKFAFTIITIVNQM

>DereOr63a____scaffold_4784_residues_2983432_to_2986243_forward_strand.[501_2312].sp

LKRRNYRSIREMIRLSYTVGFNLMDPSRCGQVLRIWTIVLSLSSLASLYGHWQMLVRYIHDIPRIGETAGTALQFLASIA

KMWFFLFAHRNMYDLLRKARPVAKIIRKQVESTMNWYWTSTRRQLLIYLYSCICITLNYFINSFVINIYRYFTLPKGSYD

IMLPLPSLYPAWEHKGPYYHIQMYLETCSLYICGMCAVSFDGVFIVLCLHSVGLMKSLNQMIEQATSDLYLRCCIYQYQR

VANFAAEVNDCFRHISFTQFLLSLFNWGLALFQMSVGNNSSITMIRMTMYLVAAGYQIVVYCYNGQRFVTASEEIGNAFY

QVRWYGESREFRHLIRMMLMRTNRGFRLDVSWFMQMSLPTLMAMVRTSGQYFLLLQNV

>DereOr65a____scaffold_4784_residues_9005508_to_9007976_forward_strand.[501_1969].sp

TRDQLKALGFYTNSEERRLPRVVWQYFLLIQLATSMASLFYGVRESVGDIVNLGRDLVFIITTLFICFRLVFLAQYADEV

DAIIDALEDIYHWSVKGPGSREVQETKRLHFLLFMALVVTWFTIILFILLKISTPFWIESQILPFHVAWPFDPTKHPAHF

IIYVAQSTNVLYFMIWLGVAENMGVSIFFELTSALRVLCIELRNLQKLCLGDEDMLSRELCRMINFHQQIILLTERCNHI

FNGAFIMQMLINFLLVSLSLFEVLAAKKDPQVAAEYMVIMLMTLGHLSFWSKFGDMFSEESKQVALAVYEAYDPNGSKSI

HRQFCFFIQRAQKPLVMSAAPFPPFNLENYMLILKQCYSILTVLAN

>DereOr65b____scaffold_4784_residues_9010260_to_9012693_forward_strand.[501_1934].sp

MKAMALYTTTEERQLPYRSTWHILVNIQAVVFFASMCSGLKESIGDHVEMGRDLAFMLGAFFIMFKICYFYWYGDDLDRV

VSELDALHPWAQTGPNAVEYRTGKRWYFVMAFFLASSWSLFLCIFLFLLLTSPMWVHDQNLPFHAAFPFQWHHPIGHVII

YLFQSYFTAYALTWLLCIEGLSVCIYAEVTFAIEVLCLELRQLHRFKVELRMEMNRLVKLHQKIMQILDRTNNVFHGTLI

MQMGVNFSLVSLSVLEAMEARKDPKVVAQFAILMLLALGHLSMWSYFGDMLSQKSLKISEAAYEAYDPTGSREVYRDLCL

IIRRGQEPLIMRASPFPSFNLINYSAILNQCYGILTFLLK

>DereOr65c____scaffold_4784_residues_9012413_to_9014865_forward_strand.[501_1953].sp

FLYTTSKERQLCFRSTWHFLVTIQVSICFLTMCYGVTESLGDKVQVGRDLAFLIGVFFIVFKLYYFHWYGDELDGVVETV

EAFHPWAQKGPGAVDYRTSKRWYLVLAFLLASSWMAFLCIFLLLLITAPMWVHQQILPLHAAFPFQWHYPTSHAIIYLFQ

SWSATYFLIWLVCIEGLSVSIYLELTFAIEVLCLELRHLHRQLRLETNRLVKFHQKIVEILDRTNNVFHGTLIMQMGVNF

SLVSLSVLEAMEARKDPKVVAQFAILMLLALGHLSMWSYFGDMLSQKSLKISEAAYEAYDPSGSREVYRDLCLIIRRGQQ

PLIMRASPFPSFNLINYSAILNQCYGILTFLLK

>DereOr67a-1_scaffold_4784_residues_9516744_to_9519113_forward_strand.[501_1870].sp

KEKIYDVDDFLKLAVNFYNTMGIDPYETGRKWSIWFQLYFAANLINMVYSFFAEAAYLANDSENLLESCMVLSYWTFVII

GLSKICAVMYRKPKLTSLVKQLKSCFPSSAMDQEEYDVKSCLKRTHMYTKGFGVLYTVMYFAHTLIPIFVYFSEKLLLKY

PDAKQNMPFYQWEPWEWRDNWWFYPTYFHQSHAGYTATCGSIAGDLMIFAVVLQVIMHYDRLAKVLRELKNNPNAVDEDL

KKLQSLIANHIDILRLTDFTNDVFGVSLLLNFVASSLLVCLVGFQLTIKFSLQYFGKQVLLLVSVLFEVYLLCSFSQKLM

DASENVGIAAYDMDWQGADKRFKKMLIYISMRAQKPVCLKATIVLDLSMSTVTIFLGMTYKFFCAIRTM

>DereOr67d_scaffold_4784_residues_10260337_to_10262715_forward_strand.[501_1879].sp

RYRKVIRMIRFCVGFCGNDVADPNFRMWWLTYAVIAAIAFFFACTGYTIYVVVINGDLTVILQALAMVGSAIQGLTKLLV

TANNACHMREVQNTFEEIYREYGPKGDYAKCLEKRIRITWTLLIGFMLVYIILLGLIITFPIFYLLILHQKVLVMQFLIP

FDHTTDGGHLILTAAHVVLITFGGFGNYGGDMYLFLFVTHVPLIKDIFCVKLTEFNELVLKRNEVRAMLCDLLVWHQLYT

STLQTTKKIYSIVLFVQLSTSCVGLLCTISCIFMKAWPAAPLYLLYAVITLYTFCGLGTLVENSNEDFLSVIYNCLWYEL

PVKEEKLIILMLAKAQNEVCLTAADMAPLSMNTALQLTKGIYSFSMMLMN

>DereOr69aA____scaffold_4784_residues_12966418_to_12971984_reverse_strand.[1501_5484].sp

QRIVFLLGTICQMVQIAGVLIYWYCNGRDVAQLSEMCSSLTLLLVGFCNVCALTVNRNQIETLLEKLQEIYPGSKKNHYR

CQHYFDLAMTIMRMEFLFYMVFYIYYNSAPVLLLLWEHVHEGYDLSFKTQTNTWFPWKVQGSIGFIMAVLSITVGSFVGV

GFSIVTQNLVCLFSFQLKLHYDGISSQLVSLDHQELRILIAYHCRILQLGDQVNNIMNFVFGSSLVGATIAICMSSVSIL

LLDFASACKYVSGLVAFVLYNFVICYMGTEVNSASGKVLPAAFYNNWYEGDLAYRRMLLILMMRATKPYMWRTYKLAPVS

ITTYMATLKFSYQMFTCVRSLK

>DereOr69aB____scaffold_4784_residues_12966418_to_12971984_reverse_strand

RYQDLVCQAAQLPRYWWNGREVKPNLAKRIIFWIGAVNLLYHNIGCVMFGYFVDGRLGELASVASMLGFTIVGTLNLWKM

LSLKSHFESILDEFEDLFHLTKQISYRTHRYHEEYTRHIRNAVVFHSSAVVYYNSLPIFLMIREHLSNSQQLSYRIQSST

WYPWQTKGSPGFLAAVVCQIFSCQTNISVNMFMQFMVNFFGIQLEIHFDGLARQLETIDARHKDRLKYLIVYHTKLLNLA

DRVNRCFNFTFLISLSVSMISTCFLAFSMTIFDFSTSLKHLLGIMLFITYNFLMCRSGTHLILKSGKVLPAAFYNNWYEG

DLAYRRMLLILMMRATKPYMWRTYKLAPVSITTYMATLKFSYQMFTCVRSLK

>DereOr71a_scaffold_4784_residues_15030803_to_15033043_reverse_strand.[501_1741].sp

RYLTGILKWWRLWPRKESVSRPDWTNWQGYTLHVPFTWLFVVLLWVEAIRSRDIQHTADVLLICLTTTALGAKMINNWKY

AHVAQGILSEWSTYELFELRSNQEVDMWRFEHRRFSRVFIFYCLCSAGVIPFIVIQPLFDIPNQLPFWMWTPFDWHQPVL

FWYPFIYQAITIPVTCICNITMDGVNWYMMLHLSLCLRMLGQRLTKLQHDDKDLRDDKDLRQKFLELVQLHQKLKQQALG

IETFISKSTFTQILVSSLIICFTIYSMQMSLQDLPGFAGMMNYLLAMVMQIMLPTIYGNAVIDSANMLTDSMYNSDWPDM

NSRMRRLVLIFMVYLNRPMTLKAGGFFHMGLPLFTKTMNQAYSLLALLLNM

>DereOr74a_scaffold_4784_residues_16530687_to_16533134_forward_strand.[501_1948].sp

WPLEAGRWTVFLDRVIIFLGFLVFCEFHYLIANRQDMDNLLTGMPTYLILVELQIRCFQLAWHKDRFRALLQRFYAEIYV

SEQLEPHLFARIQRQMLATRVNSSVYLLTLFNFFLVPVTNVIYHRREMLYKQVYPFDNTQLHFFIPLLVLNFWVGFIITS

MLFGELNVMGELMMHLNARYVQLGQDLRRSAQTLLLTHILRRNAALRDFGQRVEEEFTLRIFVMFAFSAGLLCVLFFKAF

TNPWANVAYIVWFLAKLMELLALGMLGSILLKTVDELGMMYYTADWEQVNVKLMKLVTLAIQLNSRPFFITGLNYFRVSL

TAVLKIIQGAFSYFTFLNSMR

>DereOr82a_scaffold_4770_residues_444174_to_446603_forward_strand.[501_1930].sp

YCLRAMAHRDDMDSTEDRALSLKHISSLIFVVSAQYPLISYAAYNRNDMEKVTACLSVVFTNMLTVIKICTFLANRRHFW

EMMRRFRKMHEQTASETHKIGEALDYVAEANKVARAYCLSCGLTGLYFMLGPLVKIGACLWHGTTCDKELPMPMKFPFND

LESPGYEICFLYTVLVTVVVVAYASAVDGLFISFAINLRAHFQTLQAQIENQQDTQARLKSIVDYHVLLHSLSRKLRSMY

TSTVMGQFVITSLQVGVIIYQLVTNMDSVMDLLLYASFFGSIMLQLFIYCYGGEIIKAESLQVDTAVRLSNWHLASPKTR

TALSLIIIQSQKEVLIRAGFFVASLANFVGICRTALSLITLIKSI

>DereOr83a__scaffold_4770_residues_1492131_to_1494694_reverse_strand.[501_2064].sp

TMCIAAMYPFGYRGTLAYELLNYFVSVHIAGLYICTIYINYGQGDLDFFVNCLIQTIIYLWTIMKLYFRRLRPGLLNAIL

SKINDEYEPRSAVGFSFVTMAGSYRISKLWIKTYVYCCYIGTIFWLALPIAYRDGSLPLACWYPFDYTQPIVYEVVFLLQ

AMGQIQVAASFASSSGLHMVLCVLISGQYDILFCSLKNVLASSYVLMGANMAELNQLRAEQSAADAEYTYSVEEESAFRL

SFVRCIQHHRYIVAALKKIEHFYSPIWFVKIGEVTFLMCLVAFVSTKSANSFMRMVSLGQYLLLVLYELFIICYFADIVF

QNSQRCGEALWRSPWQRHLKEVRSDYIFFMLNSRRQFQLTAGKISNLNVDRFRGTITTAFSFLTLLQKM

>DereOr85a_scaffold_4770_residues_17426991_to_17429184_reverse_strand.[501_1813].sp

IDQMGWRLPPRTKPYWWLYYIWTLVVIVLVFIFIPYIMTGIKEFKNFTDLFTYVQVPVNTNASIMKGIIVLFMRRRFSTA

QKMMDAMDTRCTKMEEKVQVHRAAALCNRVVVIYHCIYFGYLSMALMGALVIGKTPFCLYNPVNPDEHFYLATAIESVTM

AGIILANLILDVYPIIYVVILRMHMELLSERIKKLRTEVDKGDDQHYAELVDCVKDHKLIVEYGNILRPMISATMFIQLL

SVGLLLGLAAVSMQFYNTVMERFVSGVYTIAILSQTFPFCYVCEQLSSDCESLTNTLFHSKWIGAERRYRSTMLYFIHNV

QQSILFTAGGIFPICLNTNIKMAKFAFSVVTIVNEM

>DereOr85b_scaffold_4770_residues_17241785_to_17244061_forward_strand.[501_1777].sp

MNYASFFYTVVGIRPYTDGEESKMDRLRYRIVFWSNVINVCFVGYVYSAFKDNQILEAVTVMSYIGFIIVGMSKMFFIRW

KKSAITELINELKDIYPRGSIQAERYNLPMYLGTCSRISLTYSLLYSVLIWTFNLFCIMEYWVYDKWLSIREVGKQLPYL

MYIPWQWQDSWSYYPLLFSQNFAGCTSAAGQISTDLLLCAVASQLVMHFDFLSTSMESMERHEVSGDWKTDSRFLVDIVK

YHERILRLSNTVNDIFGIPLLLNFMVSSFVICFVGFQMTVGVPPDIVVKLFLFLISSMSQVYLICHYGQLVADASHGFSV

ATYNQKWYNADVRYKRALVIIIARSQKVIFLKATIFLDITRTTMTDLLQISYKFFALLRTM

>DereOr85c_scaffold_4770_residues_17240104_to_17242389_forward_strand.[501_1786].sp

MKYAVLFYTSVGIEPYTIPKKFRLWSYLLFWANVINLSFVVLGEILYLGVFDGRFIDAVTVMSYIGFVIVGMSKMFFIWW

KKADLSKLVKELEHIYPTGKAEEEEYRLDSYLRSCSRISITYALLYSVLIWTFNLFSIMQFLVYEKWLKIRVVGQTLPYL

MYLPWNGQENWSYYVLLFLQNFAGYSSATGQISTDLLLCALATQVVMHFDYLARLVENQELDDDWNRNSRFLARAVQYHQ

RILRLMDVLNDIFGIPLLLNFMVSTFVICFVGFQMTVGVPPDIMLKLSLFLFSSLSQVYLICHYGQMIADASSSLSISAY

KQNWHNADIRYRRALVFFIARPQRTSYLQATIYMNVTKSTMTSLLQVSYKFFALLRTM

>DereOr85d__scaffold_4770_residues_17198220_to_17200581_reverse_strand.[501_1862].sp

QKKQKAIPLESFLKYANAFYLSIGMQAYDHKYSQKWKDVLLHWTFIAQMVNLNAVLVIYVFLAIGNFLEATMNLSFIGFV

IVGDLKIWYISRQKKRLTKVVSRLGELHPKRLDQQEPYDMEVHLSGYSRYSKFYFGMHLVLIWTYNLYWAVYYLVCDFWL

GIRQFERMLPYYCWVPWDWSTGFSYYFMYISQNIGGQACLSGQLAADMLMCALVTLVVMHFIRLSAHIECHLQSTVAYHQ

RLIQLCQDINDIFGVSLLSNFVSSSFIICFVGFQMTIGSKIDNLVMLVLFLFCAMVQVFMIATHAQRLVDASEQIGQAVY

NHDWFQADLRYRKMLILIVKRAQQPSRLKATRFLNVSLVTVTDLLQLSYKFFALLRTM

>DereOr88a__scaffold_4770_residues_9481191_to_9483565_forward_strand.[501_1875].sp

QNPPVFSITIYFSIRGLMLFLKRREIVEFVNDLDRECPRDLVSQLDMQMDETYRNFWQRYRFIRIYAHFGGPMFCVVPLA

LFLLTHEGKDTPVAQHEQLLGGWLPFGVRKKPYYLLVWFIDLMCTTCGVSFFITFDNLFNVMQGHLVMHLGHLARKFSAI

DPRQSLTNENQLTNENQFFADLRLLVQRQQFLNGLCRKYNDIFKVAFLVSNFVGAGSLCFYLFMLSETSDAVIIAQYILP

TLVLVGFTFEICLRGTQLEEASEGLESSLRSQEWYLGSQRYRKFYLLWTQYCQRTQKLGAFGLIRVNMVHFTEIMQLAYR

LFTFLKS

>DereOr92a__scaffold_4770_residues_5338755_to_5341189_reverse_strand.[501_1935].sp

TFDELTRFPITFYKTIGEDLYSDRDPNVIRRYLLRFYLVLGFLNFNAYVIAYFIMSTTTLLEATAVAPCIGFSFMADFKQ

FGLTVNRKRLVRLLDDLKEIFPLDVEMQRKYNVSFYRKHMNRVMTLFTILCMTYTSSFSFYPAIKSTIKYYLMGSEIFER

NYGFHILFPYDAETDTVYWFSYWGLAHCAYVAGVSYVCVDLVLIATITQLTMHFNFIANDLEAEENIKYLQDLVVYHARA

LDLSEEVNNIFSFLILWNFIAASLVICFAGFQITASNVEDIVLYFIFFSASLVQVFVVCYYGDEMISSSSRIGHSAFNQN

WLPCSTKYKRILQFIIARSQKPASIRPPTFPPISFNTFMKVISMSYQFFALLRT

>DereOr94a__scaffold_4820_residues_9259223_to_9261436_reverse_strand.[501_1714].sp

HQDRIEAMRLILRVMQLFGLWPWSLEWSGFVRRHYRLLLHLPITFAFIGLMWLEAFISSNLEQAGQVLYMSITQMALVVK

ILSIWHHRTQAWRLMQEFQYAPAYQLHSQEEVDFWRREQRLFRWFFYIYILISLGVVYSGCTGVLFLEDYELPFAYYVPF

EWQNESRYWFAYGYDMAGMTLTCISNITLDTLGCYFLFHISLLYRLLGLRLRELKNMQNMQDDAMFGKELRAIFLLHRRI

RRLTLTCQSIVSPYILSQIVLSALIICFSGYRLQHVRDNPGQFISMLQFVSVMILQIYLPCYYGNEITVYANQLTNEVYH

TNWLESRPPTRKLLTAYMEHLKKPVAIRAGSFFAVGLPIFVRTINNAYSFLALLLNV

>DereOr94b__scaffold_4820_residues_9257218_to_9259433_reverse_strand.[501_1716].sp

AIQTLLVIQRWIGHLKWEKDGALTWLKRIYPLVLHLPFTFTYIGLMWYEAVTSSDFEEAGQVLYMSITELALVTKLLNIW

YRRHEAANLIHELQHDPAFNLRTSEEIRFWQQNQRDFKRIFYCYIGGSLIVAAMGYVSVFFQEDYELPFGYYVPFEWRTR

QRYFYAWGYNVVAMTLCCLSNILLDTLGCYFMFHIASLFRLLGMRLEALKNATEETEEKAKPELRRIFQLHSKVRRLTRK

CEVLVSPYVLSQVVFSAFIICFSAYRLVHMKQRPGLFVTTVQFVAVMIIQIFLPCYYGNELTFHASALTNSVFGANWLEY

SVGTRKLLNCYMELLKRPVKVRAGVFFEIGLPIFVRTINNAYTFFALLLK

>DeugOr1a

WTQRFTFARMGLDLVPKAKGRVLRSPALYGIMFVATGFELCTVCAFMVQHRNQIVLCSEALMHGLQMISSLLKMAIFLAK

SHDLVALIQLIQQPFTVDSLGDSEWKSQNRRGQLLAAIYFMMCAGTSVSFLLMPVALTMLKYHSTGDFAPVSSFRVXLPY

DVTQPHIYALDCCLMVFVLSFFCCSTTGVDTLYGWCALGLSSQYRRLGQQLKWIQEHSDPSLIVEHARLLRLVRRFNASF

MEIAFVEVLVICVLYCSVICQYIMPHTDQNFAFLGFFSMVVTTQLCIYLFGAEQVRLEAEGFSSQLYEIPWQSLSPQQRR

FLLLPLQRAQRDTVLGAYFFELGRPLLVWIFRTAGSFTTLLNA

>DeugOr7a

RRAFRNLFNCFYALGMQAPDGPTKSITWRRIYRYFSVVMYVWQLLLVISYRYMGGMEITQVLTSAQVAIDAVILPAKIVA

LAWNLPLLRRAEHHLAALDARCREQVEFQLILDAIKFCNRLVWFYQISYAIYSSSTFVCAFLLGQPPYALYLPLDWQRSL

QFCIQAWIEFLIMNWTCLHQASDDVYAVIYLYMVRIQVQLLAKRVQNLGRNTKGGEMEIYPDERKQEEHCAELQRCIVDH

QTMLQLLGCISPVISRTIFVQFLITAAIMGTTMINIFIFANTNTKIASIIYLMAVTLQTAPCCYQATSLMLDNEKLALAI

FQCQWLGQSARFRKMLLYYLHRAQQPITLTAMKLFPINLATYFSIAKFSFSLYTLIKGM

>DeugOr9a

QNQEQKQEGQSLRVQILVYRCMGIDLWSPTTINDRPWLTFVTMGPLFMFMVPMFLAAHEYITQVSLLSDTLGSTFASMLT

LVKFLLFCYHRKEFVGLIYLIRGILDKEINPDARAIVVEENQSDQMLSLTYTRCFGLAGIFAAIKPFVGLVISLIRGDEI

HLELPHNGVYPYDLQVVVWYVPTYLWNVMASYSAVTMALCVDTLLFFFTYNVCAIFKIAKHRMIHLLVQVLLLHQKGLQI

ADHIADKYRPLIFLQFFLSALQICFIGFQVADLFPNPQSLYFIAFVGSLLIALFIYSKCGENIKSASLDFGNGLYESNWT

DFSPPTKRALLIAAMRAQRPCQMKGYFFEASMATFST

>DeugOr10a

RDQQLNVYFFAVPKLSLDIMGYWPGNIDNRLPRRSIVHFVILAIGVITEAGLRFLDQQQITLALETLCPAGTSAVTLLKM

FLMLRYRQDLSAMWKRLRSLLFDLKAEQPDQEIRLRHSIMAARINFWPLSTGFFTCTTYNLKPILIAIVLFLQHRSEDFV

WFTPFNMTMPRVLLSSPFFPLTYIFIAYTGYVTIFMFGGCDGFYFEFCAHLSSLFEVLQAEIRSIFKNLEERMRAVIIRH

NAIIELTRFFRERYTIITLAHFVSAAMVIGFSMVNLLAVGNNGLGALLYVAYTIAALSQLLVYCYGGTLVAENSTELCRV

VFSCPWQLLKPSQRLLVQLLILRSQRPMSMAVPFFSPSLATFAAILQTSGSIIALVKSF

>DeugOr19a

KVKADSTRALVNHWRIFRLIGVHPPSKDTMWGRNYTLYSIVWNVTFHLFIWLSFSVNFLLSNSLETFCESLCVAMPHTLY

MLKLFNVYWTRNELLHSNRVFRYLDRRLVSSDEFRIVNEGVQKAEFIFRIISHGVVVIVALAIFYISVASEPTLMYPSWI

PWNWKDSTLCLPTIILHSSAITETALTVLSLSTYPGTYLILVSAHTKALAFRVSKLGYGLNGYIKDHQIILRLFKSLERS

LSMTCFLQFVCTASAQCTICYFLLFEQVGIMRFANMAMLLVAFTTETILLCYTAELLCKEGESLLTAVYSSNWLDQSVRF

RRHLLLMLVRCKKPLILVSGAIVPVSMKTFMLMIKGAYTMLTLLNEMR

>DeugOr19b

KVKADSTRALVNHWRIFRLIGVHPPSKDTMWGRNYTLYSIVWNVTFHLFIWLSFSVNFLLSNSLETFCESLCVAMPHTLY

MLKLFNVYWTRNELLHSNRVFRYLDRRLVSSDEFRIVNEGVQKAEFIFRIISHGVVVIVALAIFYISVASEPTLMYPSWI

PWNWKDSTLCLPTIILHSSAITETALTVLSLSTYPGTYLILVSAHTKALAFRVSKLGYGLNGYIKDHQIILRLFKSLERS

LSMTCFLQFVCTASAQCTICYFLLFEQVGIMRFANMAMLLVAFTTETILLCYTAELLCKEGESLLTAVYSSNWLDQSVRF

RRHLLLMLVRCKKPLILVSGAIVPVSMKTFMLVIKGAYTMLTLLNEMR

>DeugOr22c

FYKIPRLSGRIVGLWPQRISSGRPWHAHLLFVFAFAVVMVGAVGEVTYGCVHLDNLVVALEAFCPGTTKAMCVLKLWVFF

RSNRRWAELIQRLRVMLWQSRREEAQRMLVKLATTANRLSLLLLSSGTMTNTAFNLQPLIMGLYRWFFELPGQIELPFNI

MLPAFALQPGLFPFTYVLLTASGACTVFAFSFVDGFFVCSCLYICGVFRVVQQDIRRIFADTEAMNAEVRDRLAGVVERH

NAIIDFCTDLTRQFTVIVLMHFLSAAFVLCSTILDIMLNTSSLSGLTYICYIIAALTQLFLYCFGGNHVSESSAAVADVL

YDIEWYKCDARTRKVILMILRRSQRAKTIAVPFFTPSLPAFGSILSTAGSYITLLKT

>DeugOr23a1

DYFRNQLIAWRICGAVDLSKGNYWSWAMFLAIFVYLPTPMLMKVLFSYEDPLDNNFNFSLTITSLSNFLKFSIYAAKLTK

ILEIQKLIAQLDARVSGKEQELKHRYFQHTQRMSKMFLVTYAIVFINAAVPFVFESERSLPLPMWIPFDWKNSIAYIAAL

AFQEIGIFFQILQNYPGDSFPPLALFLVSEQCQLLILRISAIGYDSKSLKETESKSLKETEEELVNCIKDQNTLYRXXDE

VHSLISYPMMVQFLVIGVNIAITLFILIFYVQTLGDRIYYVCFLMALTVQTYPLCYYGTMVESSFADLHYAIFCSNWVDQ

SSTYRGYMLILAERTKRRQLLLAGNLVPIHLSTFAACWKGAYSFFTLM

>DeugOr23a2

YFRDQLFIWRICGAMNLSEGNFCSWALLFCVFMYLPTPMLLKVLFSFDSPLDNNFNLCMSITSLSNALKFSIYAAQLKKI

VEIQALIAKLDDRVSGEDQELRHRQMSVHLRNISKVFVVSYSLLLINAAVPFLFNSERSLPIPMWFPFDWKTSTAYIAAV

FFQEVALVFHTIQNFSGDSFPPLALFLVSEQCQLLILRISEIGYGSKTLKANKTLKANEQELVNCIKDQNTLYRLLDIVH

SLISYPMMVQFLVIGFDIAITLINIIFFVETMSDRIFHMSFLLAITLQTYPLCYYGTMVEESFADLHYAIFCSNWVDQSS

TYRGLMLIMSERTKRRQLLLAGNVVPIHLSTFVACCKGAYSFFTLM

>DeugOr24a

RHYFMVPKFALSLIGFYPEQKRTLPVKLWSFFNFFILTYGCYAEAYYGIHYIPNIATALDALCPVASSILSLVKMVAIWW

YQDELKSLIQRVRLLTEQQRSPRKLGYKKRFYTLATRLTFLLLCCGFSTSTSYSVRHLLDNILRRAHGKDWIYETPFKMM

FPDPLLSLPLYPITYILVHWHGYITVVCFVGADGFFLGFCLYFTVLLLCLRDDVGDLLEVANIEETPTAEQEALVVREME

KLVDRHNEVAELTERLSGVMVEITLAHFVTSSLIIGTSVVDMLLFSGLGIIVYVVYTCAVGVEIFLYCLGGSHIMEACSD

LARSTFASHWYGHSVRVQKMTLLMVARAQRVLTIKIPFFSPSLETLTSVLRFTGSLI

>DeugOr33a

TYWRYWRFLGVEGEYPFRRLWDLTMTIFITILYPVHLILGMYDKPKVLIFRSLHFTIECLFCSFKFVFFRWKLAEIKEIE

GLLQDLDKRAGSEEERSYFKENSRVAKMLSKSYLVAAISSIITATVAGLFSSGRNLMYLGWFPYDVQATLNFWTSFTYQA

VGSSLMILENLANDSYPPITFCVVTGHVRLLAMRLSRIGHDEDISSNENTSSNENTSRLIEGVQDHRKLMRIIRLLRSIL

HLPQLGQFLSSGINISISLVNILFFAENNFTMIYYAVFFAAMLIELFPSCYYGTLMMMEFDKLPYAIFSSNWIKMDKGYN

RSLIIFMQLTLIPVDIKAGGIVGIDMSAFFATVRMAYSFYTLAMSFR

>DeugOr33b

WRLLGLESSFILHRLLDILITVFVTVWYPIHLILGLFMERTLVDVCKGLPITAACFFASFKFICFRLKLSEIKTIEVLFK

ELDQRAVSHEECQFFNQNTRREANFIWKSFIVAYGLSNVSAISTVLFGGGHKLLYPAWFPYDVQKSLRFWLSVTYQIAGV

SLQILQNLANDSYPPMTFCVVAGHVRLLAMRLSRIGQDEKESQASEKESQASIGKQLVESIEDHRKLMRIVELLRSTMNM

SQLGQFISSGVNISITLVNILFFAENNFAVTYYGVYFVSMVLELFPCCYYGTLISVEMNRLTYAIYSSNWLGMDRGYCRT

LLIFMQLTLAEVQIKAGGMIGIGMNAFFATVRLAYSFFTLAMSLR

>DeugOr33c

TFFKDSPRLVRLYVVLLHSLVTLWFPLHLLLHLLLHPSPAALLKNLTMSLTCVSCSLKHVAHLYHLPEIESLISQLDTFI

SSEQEHRYYQEHVKCHAKRFTRCLYISFGMVYAVFLLGVIVQIFSGTFDLIYPAYFPFDLERNQLAAVALGYQVFSILVE

GLQGLGNDTYTPLTLCLLAGHLHLWSIRMAQLGFLEKEKESTYNHRRLREYIEQHKLLVRFHHLVARTISQVQLVQLCGC

GATLCIIVSYVLFYVGDTISLVYYLVFFGVVCVQLFPSCYFASEVAEEIQGLPYAVFSSRWYNQPREHRFDLLIFTELTS

QRLVIKAGGLIELNLNAFFATLKMAYSLFAVVVRVK

>DeugOr42a

LMRCIFLMGVRKPPGKFFVVYVLWSFALNFCSTFYQPIGFLTGYISEFSPGEFLTSLQVAFNAWSCSTKVLIVWALVNRF

DEANDILDQMDKRISQPGDRMHVHRAVMLSNRIFFFFMTVYMVYATNTFLSAIFIGRPPYQNYYPYDWRSSLHLALQAGL

EYFAMAGACFQDVCVDCYPVNFVLVLRAHMFIFAERLRRLGTDPFESQDQRYKRLV

>DeugOr42b

LYRAMKFIGWLPPKDGVSRYLYLTWTLMTFVWSTTYLPLGFLGSYMKSFSPGEFLTSLQVCINAYGSSVKVAITYSMLWR

LIKAKSLLDHLDLRCTTMEEREKIHRVVARSNHAFLIFTFVYCGYAGSTYLSSVLSGRPPWQLYNPFDWHDGLKLWVAST

LEYIVMSGAVLQDQLSDTYPLVYTLILRAHLDMLRERIRRLRSDVTEAESYEELVKCVIDHKLILRYCSIIKPVISGTIF

TQFLLIGLVLGLTLINVFFFSDIWTGIASFMFVITILLQTFPFCYTCNLIMEDCESLTHAIFQSNWVDASRRYKTTLLYF

LQNVQQPIVFIAGGIFQISMSSNISVAKFAFSVITITKQM

>DeugOr43a

INVRMWRHLAVLYPSPGTNWRKFAFVLPVTAMNLMQFVYLLRMWGDLPAFILNMFFFSAIFNALMRTWLVIIKRDQFEEF

LNKLCALFHSILESSDERDILRRAESEARSLAILNLSASFLDIVGALISPLFRDERGHPFGVALPLNMTRTPVYEIVYFA

QLPTPLLLSMMYMPFVSLFAGLAIFGKAMLQILVHRLEQFGREEQSEEQSEEVCYQMLTSCIRYHMQVMSYVWELNKLVT

NIVAAEAIIFGSIICSLLFCLNIITSPTQVISIVMYILTMLYVLFTYYNRANEICLENNRVAEAVYNVPWYSASIRFRKT

LLIFLIQTQHPLEIRVGNVYPMTLAMFQSLLNASYSYFTMLR

>DeugOr45a

SYFAVQRRALEIVGFDPSTPQLRLIHPIWAGILILSLVTHNWPMAVYALQDLSDITRVTDNFAVFMQGSLSTFKFLAIVA

KRRRIGSLIHQLHNLNQKASTTLSQLEKIQKENQLDRYVSRSFRNAAYGVIWASAIAPMLLGLLGYIRTGMFTPTTPMEF

NFWLDERNPQYYWPIYFWGVLGVGAAAWLAIATDTLFSWLIHNVVVQFQLLELLLEKSDHNQEKSDHNQEDDSHLIECIY

QHRLALDLAKELSSIFAEIVFVKYMLSYLQLCMLAFRFSQSGWSAQVPFRAAFLVAIIIQLSSYCYGGEYLKQQSLGIAQ

AVYNCNWPEMSPRKRRMWQMMIMRAQRPAKIFGFMFNVDLPLLLWVSRTAGSFLALLRTF

>DeugOr45b

LFFVTRYSFGLLGLRFGQKQSWLHLSWLVFNFVNLAHCCQAEFVFGWSHLRSPVDAMDAFCPLACSATTLFKLGWMWWRH

QEVADLMERVRLLIKEQEKDSRRDRAQRSYYQMVTRCGMMVFTSGSITTGAFVLRSLWEMWVRRHQEFKFDMPFRMLFGE

FAHRMPWFPVFYLYSVWSGQVTVYAFAGTDGFFFGFTLYMAFLLQALRYDVQDRESNLCCQRLADIVDRHNEIEKIVQVF

SGIMAEPTFVHFVSASLVIATSVIDILLYSGYNIIRYMVYTITVSSAIFLYCYGGTEMATESLSLGEAAYSSAWYKWDRE

TRRRVFLIILRAQRPITVRVPFFAPSLPVFTSVIKFTGSIVA

>DeugOr46aA

QKAILNLFSLWPQTERRWRIIHQVNYVHVMGFWVLFFDLLLVIHVVANLSYMSEVVRAIFVLATSAGHTTKLLSVKANNV

ELEKLFKRLDDEDFQPRGVEEELIFTEACERSKKLRDFYGALSLAALSMILIPQFVLDWSQLPLGTYNPFDTSGSPGYWF

LYCYQCLALSVSCFTNIGFDSLCSSLFIFIECQLDILVVRLDKMGRLNNDDNDDSSVEHQLKENIRYHMTIVELTKTVER

LLCNPISVQIFCSVLVLTANFYAIALVSSRLIFIINYITYHAESDFHLVLLCRVGSEVTQRSLDLPHELYKTSWVDWNRS

NLRIVLLFLQRLHSTLRIRTLNPLGFDLMLFSIVNCSYSYFALLKRV

>DeugOr46aB

DFYKYQVWYFEILGVWKLPTTDHQRRFQSMRFGFILVILFIMLLLFALKLLDNISQVREILKVFFMFATEISCMTKLLYL

KLKSRKLAGLVDMMLSTEFSVKTEQERQILASTNVTVVHMRNFYGLMSFITAFMMLLIPCFGNYEELPLTMYEVCEIEGR

ICYWLHYIFHAISLMPTCFLNITYDSMAFSLLCFLKVQLHILVLRLEKLGPVIDPQDNEIDPQDNERIARELRECAAYYN

NIVQFKNLVELFIKVPGSVQLVCSVLVLISNLYDMSTMNGDAIFMAKVCIYQLVMLWQIFIICYASNEVTVQSSKLGHGI

YSSEWTEWNKSNRRIILLMMQRFNSPMLLTFNPTFIFSLEAFASIVNCSYSYFALLKRV

>DeugOr47a

SFLQVQKSTIALLGFDLFSENREMWKRPYRAINVFGIAAIFPFILAAVLHNWKNVMLLADAMVALLITILGLFKFSMILY

LRRDFKRLIEKFRLLMKNEAGGEEYAEILNAANKQDQRMCTLFRTCFFFAWLLNSVLPFVRMGLSYWLAGHAEPELPFPC

LFPWDIHIIRNYAMSFTWSAFASTGVVLPAVSLDTIFCSFTSNLCAFFKIAQYKVVRFRGDSLKESQATLNKVFNKVFAL

YQTSLDMCSDLNLCYQPIICAQFFISSLQLCMLGYLFSITFAQTEGVYYASFIATIIIQAYIYCYCGENLKTESASFEWA

IYDSPWHESSTSICRSLLISMMRAHRGFRITGYFFEANMEAFSSIVRTAMSYITMLRSF

>DeugOr47b

AFNYVRACLSLLGQYPNKKLASLSLYRWINLFIMCNVLNAFCTMVLALESKNVIDMGEDLVWISGMGLVFIKIFYMHLRC

DEIDDIIWDFDYYNRELRPHHTDEEVFGWQRLCYLIESSLYINCFFLVNFFNAAICLQPLLGEGKLPFHSIWPFQWHHPY

MFWFLYIWLAATSQHNLLSILMVDMFGISTFLQTALNLKLLCIEMKKLGEARFHEEFCRVVRFHQHIIKQVEKANRAFNG

AFNAQMMASFSLISISAFETMVAAVDPKMAAKSVLLMVVAFTQLSLWCVSGTLVYTQSLEVAQAAFDIDWHTKALTIQRD

ISFVILRAQKPLIYVAEPFLPFTLGTYMLVLKNCYRLLALMQ

>DeugOr49b

LIYMNIKILRFWALLYDKNIRRYVCIGLASFHIFTQMVYMMSTNEGLIGIIRNSYMLVLWINTVLRAYLLLADHDRYVAL

IQNLTEAYYDLLNDSYISKILAEVNRVGRLMARGNLFFGMLTSMGFGLYPLSSSERVLPFGSRIPLNEYESPYYEMWYVF

QMLITPMGCCMYIPYTSLIVGLIMFGIVRCKALQHRLRQVRDARQLQEEIIACIRYQQSIIEYMDHINELTTMMFLFELM

AFSALLCALLFMLIIVSGTSQLIIVCMYINMILAQILALYWYANELREQNLAVASAAYETEWFTFDIPMRKNILFMMMRA

QRPASILLGNIRPITLELFQNLLNTTYTFFTVLKR

>DeugOr56a

FRTHLRCFRWYGYVASKDQKKPWLSLLRCTIFTASIWLSCALMLARVFRGYENLNDGATSWATAVQYFTVSIATLNAYVQ

RDGVVRLLRVAHEDIQNLMLEADDQEMELLAQAYTQTITLILWVPSVIAGLICIYRTLFLPKSVFNMPAVSRGEEQPILL

FQLYPFEICDNFVVGYLGPLYALALGITTIPLWHTFITCLMKYVNLKLQILNKRVEEMDVSRLNPNFKEFVEEQLRIRKF

IQELQYLICVPVMADFIIFSVLMCFLFFALTVGVPSNMDYFFMFIYLFVMAGILWMYHWHATLIVECHDELSLAYFSCGW

YNFEMPLQRMLVFVMMHAQRPMKMRALLVDLNLRTFIDIVRGAYSYFNLLRS

>DeugOr59a

EFFKSHWTAWRVLGVAQLRVQSWRNLYIAYSIVMNLFVTLCYPLHLGMSLFRNQTLTEDILNLTTFATCTACSVKCLLYA

YNIKDVLEMERLLRLLDERVVGPEQRGIYGQVRVQLRNVLYIFIGIYMPCALFAELSFLFKEQRGLMYPAWFPFDWLHSR

NYYIANFYQIAGISFQLLQNYVSDCFPAVVLCLISSHIKMLYQRFEGVGSKNAEKELEECITDHKHILELFRRVEAFISL

PMLIQFTVTALNVCIGIAGLVFFVSEPMARMYFIFYSLAMPLQIFPSCYFGTDNEYWFGRLHYAAFSCNWHTQGRSFKRK

MMLFVEQSLKKSTAVAGGMMRIHLDTFFSTLKGAYSLFTIIIRMR

>DeugOr59b1

YRAMWLIGWIPPKEGVLRYIYLFWTCVPFAFGVFYLIISYVQEFKNFGEFLTSLQVCINVYGASVKSTITYLFLWRLRKT

EMLLDSLDKRLQNDSDRDRIHNMVARCNYAFLIYSFIYCGYAGSTFLSYALSGRPPWSVYNPFDWRDGLSLWIQAIFEYI

TMSFAVLQDQLSDTYPLMFTIMFRAHMEVLKDHVRNLRTDPDRSEADNYQDLVNCVMDHKTILKCCDMIRPMISRTIFVQ

FALIGSVLGLTLVNVFFFSNFWKGVASLLFVITILLQTFPFCYTCNLLIDDAQELSNTIFQSNWVDAEPRYRATLVHFMH

HVQQPIIFIAGGIFPISMNSNISVAKFAFSIITIVRQM

>DeugOr59b2

LYRAMKFIGWLPPKDGVSRYLYLTWTLMTFVWSTTYLPLGFLGSYMKSFSPGEFLTSLQVCINAYGSSVKVAITYSMLWR

LIKAKSLLDHLDLRCTTMEEREKIHRVVARSNHAFLIFTFVYCGYAGSTYLSSVLSGRPPWQLYNPFDWHDGLKLWVAST

LEYIVMSGAVLQDQLSDTYPLVYTLILRAHLDMLRERIRRLRSDVTEAESYEELVKCVIDHKLILRXCSIIKPVISGTIF

TQFLLIGLVLGLTLINVFFFSDIWTGIASFMFVITILLQTFPFCYTCNLIMEDCESLTHAIFQSNWVDASRRYKTTLLYF

LQNVQQPIVFIAGGIFQISMSSNIVAKFAFSVITITKQM

>DeugOr59c

FYRIALFLGWTPPKEGLLRWIYFLWTATTMWLGIVYLTLTYHFDRFTPTEFLTSLQVDINCIGNVIKSCVTFTQMWRFRR

MIELLVSLDDRCLTPSQRKILSTTVARVNLIVLLFLSTYLGFCLMNLFTSVFAGKAPWQLYNPFDWKNGWQLWIASVLEY

FVVSIGTMQELISDANAIVFISLFRAHLAILKNRIENLRQDPEERENYEQLVSCIQDHRTIIRCSQIFRPVLSITIFAQF

MLVGIDLGLASISILLFPNTIWTIMANVSFIIAICLESFPCCMLCEYLIEDCATLSDAVFHSNWLTADRRYKSTVVYFMH

RVQQPIQFTAGAIFPISVQSNIAVAKFAFTIITIVNQM

>DeugOr65a

HINAYWNRDQLKAMGLYINSERLQMTALIWKYFVLTALIICLASLLYGIPESFGDIVNLGRDLVFTVTVXYICSRLVFFA

QYSDDVDMIIDALEDLHHRMIKGPANREVQAIKRFHILLITALVIWFAVILIFILIKISTPFYIKSQSLPFHVAWPFNPS

KHPSHAIIFLTQSFTLLYFLLWLGFAEVMGVSIFFEMTSLLRVLSIELRNVQCQGDENLLNSELCRLIKFHQQIILLSDR

CNEIYNRAFIIQMLVNFILVSLSLLEVVEARKDPQVATEYLLVMTMTLGHLSFWSKFGDMFSEESEQVALAVYEAYDPTG

SKTINRQYCFFIQRAQRPLQMSASPFPPFNLMNY

>DeugOr65b

TNERQLPYRSIWHTSAIIQMTVLFVSMLYALTESVGDNVQIGRDLAFIIGVFYIIFKMYYFLWYGDALDEVINDLETYHP

WTLKGPSKFRPRKRWFMMGLIVASIWSIFLCIFVVLLITSPIWVQQQNLPFHAAFPFQWHHPISHAVIYLSQSFLVAYGI

TWLLCMEGLSVSIYAEITFAIEVLCLELRNLHQGEQLRMKTNQIVMIHQKIIEILNRINNVFHGTLIMQMSVNFSLVSLS

VLEAMEARRDPKVVAQFAVLMILALGHLSMWSFFGDMISRESLKIAGAAYEAYDPNGSKEVYRDLCLIVRRAQKPLIMRA

SPFPSFNLINYTAILNQCYGILTFLLK

>DeugOr67a

DFLCIPVLFFNIVGIEPYESVKKPSLAFKVFYTLNMINMAIAFITEMVILFRDDENFLESCIVVGYVSFVFVGVLKLFTG

SIRKQKVTILVRQLESCFPSSSEKDQEEYDVKTYLKRCNIFTKGFGGLLTIMCFAHFLIPISLYLVETYVLRSPDTKQYL

PFYELAPWDWHGGWKFYLTYLHQSIAGYTATCGSISCDIMIFAVVFQVIMHYERLAKVLREYKVRNHTEPNGAYHDMVLQ

SLVANHIDILRLTDVVNDVFGVPLLLNFMASSLLVCLVGFQLTVEFSPEYFFKQVLLLSSALVEIYLLCSFSQMVIDASA

DVSSAAYHMDWIQLDTKCRKMLLFICLRAQKPECLKATIVLDLSIATMSXFLGMSYKFFCAIQTM

>DeugOr67b

DKYLQCCVRLFARISSHHNQIENLFKYIILVQCSVSSILICMLLYKISTVEVGWVWMGMIMVYFVTIALEISLYNVSAQK

VETQSELLFYDWYNCSWYNESKEFKFMIKMMLLFSRRTFVLSVGGFTSLSHKFLVQVFRLSANFFLLLRNM

>DeugOr67c

EDSSARTFMELMRVPVQFYRSIGEDIYAHRSTKSLLLKIYLYAGFINFNLLVIGELVFFYNSIQDFDTIIAVAPCIGFSL

VADFKQFAMVRGKKTLILLLDDLEDMHPKTLEKQQQYKLSDFEKIMKRVINIFTFLCLAYTTTFSFYPAIKASVKFNFLG

YDTFDRNFGFLIWFPFDATSSLIYWIMYWDIAHGAYLAGIAFLCADLLLVVVITQICMHFNYISMRLEEPEEDKENIEFL

IGIIRYHDKCLKLCEHVNDLYSFSLLLNFLMASMQICFIAFQVTESTIEVIIIYCIFLMTSMVQVFLVCYYGDSLIAASL

KVGDAAYNQKWFQCSKSYCSMLKLLIMRSQKPASIRPPTFPPISLVTYMKVISMSYQFFALLRT

>DeugOr67d

KVIRMIRFCVGFCGNDVADPNFRMWWLTYTVIGAIGFFFACTGYTIYVVVINGDLTVILQAFAMVGSAVQGLTKLLVTAN

MACQMRDIQNTYEKIYREYGSKGEYVNCLERRIRTTWQLIIGFMLMYIILLGLIIAFPIFYLLIWNEKVLVMQFLMPIDH

TTDGGHLLLTAVHVALITFGGFGNYGGDMYLFLFVTHVPLVKDIFCVKLKEFNEVVERMRSMLVDLFTWHQLYSRILQTT

KRIYSIVLFVQLSTTCVSLLCTISCIFIKAWPAAPLYLLYAAIILYTFCGLGTLVENSNEDFLSVIYNCLWYELPVKEEK

LIIMMIAKAQKEVCLTAADMAPLSMNTALQLTKGIYSFSMMLMN

>DeugOr69aA

DYMKYIDMACWMACIPRYWTGRTRKSYSLAKKSLFIFGVICLIYQIFGQITYLYKNEQDVAVISETFGSLMLTTVGFANI

YALMKNRHQIEIMFEELHKIYPRIRDKHFRCQHYYDMAILIMKIEFMFYMVFYVYYNSAPLFVLYWEYLQEEQDLSFKMQ

TNTWFPWKVQGSIGFGMALLSISLASFVGVGFSIATLNIVCIFTFQLKLHYDGMASQLMDLDSRQSGAHEKLRNLIAYHF

HILKMGDQFNQILNFIFGSSLVGSTIAICMTSVAVLLLDVGSAFKYIIGLIAFVLYHFVICYMGTEV

>DeugOr69aB

RNLAKRIIFLCEAVNLLYHNIGCIMYGYFVVENLAEMAAVGSMLGFTILGTLNLWQILKLKPHVEELLKDFEELFQFAKQ

KPYRTRHYHESYTSYVKIWFIIYTFSVVYYNLQPFILMIWEHLKDSPELSYQIQSGTWYPWRIQGSTGFLVAIICQGFSC

QVIMCSILLSQFLISFFGIQLEIHFDGLASRLKGIDARDQLTYLIVYHSKLFNLADRVNRLFNFTFFVSFSLSIISMCFL

ANCMTMFDLVSGFKHLLGLLIFLIYNFSMCRNGTHLIFQSDKVLPAAFYNNWYEGDLSYRRTLLILMMRATKTYTWRTYK

LTPVSITTYMATLKFSYQMFTCVRSM

>DeugOr74a

WPLEAGRWTVFVDRLLIFMGFLVFCEFHYLIANRQDMGNLLTGMPTYLILMEMQIRCYQLAWHKDRFRALLQRFYAKIYV

SEEMDPHLFARIQKQMLATRANSTVYLLTLFNFLMVPVTNVIYHRREMLYKQVYPFDNTKLYYFIPLLALNFWVGFIITS

MLFGELNVMGEMMMHLNARYIQLGKDLRHSTNVLLEALTQILRRNAVLRDFGERVEEEFSLRIFVMFAFSAGLLCALFFK

AFTVCNPWGNVVYIVWFLAKFMELLALGMLGSILLETTDELGMMYYTSGWEQVNVKLMKLMTLAIQLNSKPFFITGLNYF

RVTLVAVLKIIQGAFSYFTFLNSMR

>DeugOr82a

ELQENCLRAMGHSDDMDSTELRSLSFKHISSLLLVTSAQYPLISYAAYNRNEVEKVTACLSVMLTNFLTVIKITTFLVNR

QAFWDMIRRFRKMQHQAAKQTPRLDYVTKANKQAAFLGKAYCVSCGLTGLYFMLGPIIKMLTSSSHKTIYVRELPMPMKF

PFSDLQSPGYELVFLYTVVVTVIVVAYASAVDGLFISFAINLRAHFRTLQDIIENSSSSEKKVQEGLTSIVRYHQLLILL

SGKLRATYTATVFGQFVITSLQVGVIIYQLVKNMDSVMDLLLYASFFGSIMLQLFIYCYGGEIIKAESLQIDIAFRLSNW

YLASPKLRRSLSVIIQQSQKEILIRAGFFVASLANFVGICRTALSFITVIKSI

>DeugOr83a

LFYEFFNYFVSVHIAGLFICTIYINYGQGDLDFFVNCLIQTIIYLWTIMKLYFRRFRPGLLNAILANINENYEPRSAVGF

SFVTMAGSYRMSKLWIKTYVYCCYIGTIFWLALPIAYRDKSLPLACWYPFDYTQPVVYEVVFFLQAMGQIQVAASFASSS

GLHMVLCVLISGQYDVLFCSLKNVLATTYVLMGANMAELRELQAEQSVSDSQYAYSLEEQTSKDFSTAFRQSFVHCIQHH

RYIVAALKKMESFYSPIWFVKIGEVTFLMCLVAFVSTKSANSFMRMVSLGQYLLLVLYELFIICYFADIVFQNSQRCGEA

LWRSPWQRHLKAVRRDYLFFIMNSRRQFQLTAGKITNLNVERFRGTITTAFSFLTLLQKM

>DeugOr83c

LLGVDVLAPKLEFNYRTWTTIFAIVNYTGFTVFSILNNGGDWGVGLKASLMGGGLFHGLGKFLTCLLKHQDMRRLILYSR

SIYEEYENRGESHRTLNSNIDRLLGIMRIIRNGYVFAFCLMGILPLAMLMYDGTRVTAMQYLIPLPLENNFCYTVTYLIQ

LVTMVVQGVGFYAGDLFVFLGLTQILTFADMLQLKIDELNQALEQKADNRAQVRVGARIYGEEKRQYLDQVYESICNVTW

YELSGDQRKLFGLMLRESQYPHTIRILGVMSLSVRTALQV

>DeugOr85a

IDQMGWRLPPRSKPYWWLYYIWSLMVIVLVFIFIIMTGIKEFKNFTDLFTYVQVPVNTNASIMKGIIVLFMRRRFSLAQK

MMDQMDIRCVKMEEKTQVHRSAALCNRVVVTYHGIYFGYLTMALTGALVIGKTPFCLYNPVNPDNHFYLATAIESVTMAG

IILANLILDVYPIIYVVVLRTHLELLSQRVKNLRSDKDKEDDKDKEDDEHYAELVECVKDHKLIVEYGNTLRPMISATMF

IQLLSVGLLLGLAAVSMQFYNTIMERVVSGVYTIAILSQTFPFCYVCEQLNSDCESLTNTLFHSKWIGAERRYRTTMLYF

IHNVQQSILFTAGGIFPICLNTNIKMAKFAFSVVTIVNEM

>DeugOr85b

KFLKFANFFYTGVGIQPYAKTDKDKLIARIVFMANVINLTYVATSEYVYIAYNDKKLLEAVTVMSYIGFVIVGMSKMFFI

RGKKSAMTEMMKELEEIYPRGKIEEEKYNLPKYLGTCSRISLTYSSLYSVLIWTFNLFSIMEYWIYEKWLKIRVVGKTLP

YLMYIPWIWEDSWSYYPLLFSQNFAGYTAAAGQISTDLLLCAVATQVVMHFDYLSNTMEMEYHELSGNWEEDSRFMADIV

KYHERILRLADVVNDIFGVPLLLNFMVSSFVICFVGFQMTVGVPPDMVIKLFLFLFSSMSQAYLICHYGQMVADASYGLS

VATYNQNWNHADVRYKRALVIIISRAQNVTFLKATIFLDITRSTMTDLLQISYKFFALLRTM

>DeugOr85c

MKYAVFFYTSVGIEPYTKPQKISLWSNLLFWANVINLSVIVFGEVLYLAFSDGKLIDAVTVMSYIGFVIVGMSKMFFIWW

KKPDLSNMVKELEGIYPEGKAQEETYQLERYLKSCSRISITYALLYSVLIWTFNLFSIMQFLVYEKWLKIRVVGQTLPYL

MYFPWNWEGNWLYYLLLFCQNFAGHTSASGQISTDLLLCAVATQIVMHFDHLARVVENENEELSKDWEENSRFLAKTVRY

HQRILRLMDVLNDIFGIPLLLNFMVSTFVICFVGFQMTVGVPPDLMIKLFLFLFSSMCQVYLICHYGQLIADASSGLSIA

AYRQNWHNADIRYRRALVFFIARPQRTTYLKATVFMNITRATMTDLLQISYKFFALLRTM

>DeugOr85d

FLKYANIFYLSIGMLAYDHKDSGRRKEQLLHWMFIAQIVNLNAVLLIYVFLAISNFLEATMNLSFIGFVVVGDLKIWHIW

RQRKRLTQVVNELEHLHPKRLDQQEPYNIGYHLSGYSRYSKFYFGMHLVLIWTYNLYWAVYYLVCDFWLGVRNFERMLPY

YCWVPWDWSTGFSYYLMYVSQNLAGQTCLSGQLAADMLMCALVTLVVMHFIRLSGHIQSVVVYHQRLLHLCHNINEIFGV

SLLCNFVSSSFIICFVGFQMTIGGKIDNLVMLVLFLFCAMVQVFMIATHAQRLIDASEQIGQAAYNHDWFHADLRYRKML

ILIVKRAQQPSRLKATIFLNVSLVTVSDLLQLSYKFFALLRTM

>DeugOr85f

EDFSRFPTFVLWIMGYDMLGITRIRRMLFRLYRFLCLSSHSVCVGFMIFRMIETIDNVSLIMRYATLVTYVINSDTKYAT

VLQRVAIQSLYSKLADLYPKTTLDRIYHRVNDHYWSRLFVYLVIIYIGSSIMVVIGPIITSTISYYTHHGFAYMHCYPYF

LFDPEKHWIYIVIYTLEWLHSTQMVISNIGADIWLLYFQIQINLHFRGIIRSLQRPSVENDEEDRKFLSKIVDKQVYLVX

LQKDLNGIFGGSLLLSLLTTAAVICTVAVYTLIQGPTLEGFTYVIFIATSVMQVYLVCYYGQQVLDLVGEVAHAVYNHDF

HNASIAYKKYLLIIIIRAQQPVELSAMGYLPISLDTFKQLMSVTYRVITMLRQM

>DeugOr88a1

NPPVLSITIYFSIRGLMLFLKRKDIVEFVNDLDRECPRDLASQLDMQMDRTYRNFWQRYRFIRIYAHLGGPIFCLMPVAF

FLLTHEGKESPVLQHEQLLGGWLPFNVRKDPFYFLVWIIDLACTVCGVSFFITFDNLFNVMQGHLIMHLNRLARQFAELD

PRKSLTDEAKFFSEFRFLVQRQQLLNGLCQRYNEIFKVAFLVSNFVGAGSLCFYLFMLSETSDVLIIAQYIIPTMVLVVF

TFEICLRGTQLESASEGLEMSLRSQEWYLGSQRYRKSYLLWMQYCQKTQKLGAFGLIEVNMVHFTEV

>DeugOr88a2

PVLSITIYFSIRGLMLFLKRKDIVEFVNDLDRECPRDLASQLDMQMDKTYRNFWQRYRFIRVYAHLGGPMFCLMPVAFFL

LTHEGRDSPVLQHEQLLGGWLPFNVRKDPFYFLVWIFDLTCTVCGVSFFITFDNLFNVMQGHLIMHLNRLARQFAELDPR

KSLTNETNEAKFFSEFRLLVQRQQLLNGLCQRYNEIFKVAFLVSNFVGAGSLCFYLFMLSETSDVLIIAQYIIPTMVLVV

FTFEICLRGTQLESASEGLEMSLRSQEWYLGSQRYRKFYLLWMQYCQRTQKLGAFGLIEVNMVHFTEIMQLAYRLFTFLK

S

>DeugOr92a
[truncated: 835,737 more chars]
