# Supplementary material for: Endogenous insensitivity to the Orco agonist VUAA1 reveals novel olfactory receptor complex properties in the specialist fly Mayetiola destructor
Source: Sci Rep. 2018 Feb 22;8:3489. doi: 10.1038/s41598-018-21631-3 (PMC5823858; doi:10.1038/s41598-018-21631-3)
Supplement: Supplementary file 4 — Supplementary File S7 [file 41598_2018_21631_MOESM4_ESM.doc]

**Endogenous insensitivity to the Orco agonist VUAA1 reveals novel olfactory receptor complex properties in the specialist fly *Mayetiola destructor***

Jacob A. Corcoran, Yonathan Sonntag, Martin N. Andersson, Urban Johanson and Christer Löfstedt

**Supplementary File S7.** Multiple sequence alignment used to generate the DmelOrco models.

>AAT71306.1_odorant_receptor_Or83b_[Drosophila_melanogaster]

--mttsmqpskytglvadlmpniramkYSGLFMHNFT-----GGSAFMKKVYSSVHLVFLLMQFTFILVNMALNAEEVNELSGNTITTLFFTHCITKFIYLAVNQKNFYRTLNIWNQVNTHPLFAESDARYHSIALAKMRKLFFLVMLTIVASATAWTTITFFGDSVKMVVDHE-TNSSIPVEIPRLPIKSFYPWNASHGMFYMISFAFQIYYVLFSMIHSNLCDVMFCSWLIFACEQLQHLKGIMKPLMELSASLDTY--RPNSAALFRSLSA---NSKSELIHNEEKDPG-------T--DMDMSGIYSSKADWGAQFR-APSTLQSFGGNGGGGNG--LVNGANPNGLTKKQEMMVRSAIKYWVERHKHVVRLVAAIGDTYGAALLLHMLTSTIKLTLLAYQATKINGVNVYAFTVVGYLGYALAQVFHFCIFGNRLIEESSSVMEAAYSCHWYDGSEEAKTFVQIVCQQCQKAMSISGAKFFTVSLDLFASVLGAVVTYFMVLVQLK

>AOT85634.1_odorant_receptor_co-receptor_Orco_[Mayetiola_destructor]

----MQVQQKQFTGLVADLMPNIRLMRFLGHFLHKLS-----NGPTFLSKLYSMMHLMLMLLQFFCIIINLALNTSEVNELTANTITTLHFTHTITKFCYLAINNKNFYRTFNIWNQSNSHPLFAESDARYHSIALAKMRKNLYMITGLTMGTVASWSIITFFGESVKGVFDKE-TNETYYVEVPRLPIKSYYPWDAMSGMKYIGTFVFQVYYLTFSMLACNLTDVLFCSWLCFACEQLCHLKGILKPLMELSASLDTY--RPNSAALFRSLSA---NSKSELVNNDEYDQ-----------QDENGSVFVPKAEWGGTFKVPPNMGN-------------------PNGLSKKQEIMVRSAIKYWVERHKHVVRLVSAIGDTYGAALLLHMLTATICLTLLAYQATKIDGVNVYAFTTIGYLVYSLGQVFHFCIFGNRLIEESSSVMEAAYCCHWYDGSEEAKTFVQIVCQQCQKAMTISGAKFFTVSLDLFASVLGAVVTYFMVLVQLK

>XP_015432571.1_PREDICTED:_odorant_receptor_coreceptor_[Dufourea_novaeangliae]

------MMKFKQQGLVADLMPNIQLMKATGHFMFNYYTD---NSSKFIHKVFCFVHLFLILLQFGLCGINLMLTSDDVDVLTANTITMLFFTHSVVKVLYFAIRSKLFYRTLAIWNNPNSHPLFAESNARFHQLAVKKMRILLMAVMCTTMLSVIAWTTITFIGDSTMKVVDPV-TNETTYVEVPRLMLYSYYPFDPSHGMAHVLTLIFQFYFLLFAMADANLLDVLFCSWLLFACEQIQHLKDIMKPLMEFSATLDTVV--PNSGELFKASSP---SHPAESHEPPPVSD----LQGDNMLDMDLRGIYNQRQDFTATFR--PTAGTAFN------------GTVGPNGLTKKQEMLVRSAIKYWVERHKHTVRLVTAVGDAYGIALLLHMLATTVTLTLLAYQATKINGINVYSASVIGYLLYTLGQVFMLCIFGNRLIEESTSVMEAAYSCHWYDGSEEAKTFVQIVCQQCQKAMSISGAKFFTVSLDLFASVLGAMVTYFMVLVQLK

>XP_003705783.1_PREDICTED:_gustatory_and_odorant_receptor_7-like_isoform_1_[Megachile_rotundata]

------MMKFKQQGLVADLMPNIKLMKASGHFLFNY-AD---GSGKSMQKIYSSVHLVLILMQFAFCGINLVQEREDVDDLTANTITMLHFTHTIVKIIYFAVRSKLFYRTLGIWNNPNSHPLFAESNARYHQIAIKKMRILLLAVMGSTVLSTLSWTILTFIEDPVKKVTDPV-TNETMFVEIPRLMVRSWYPFDASHGMAHVMVLIYQFYWLLFSMASANLLDVLFCSWLLFACEQIQHLKNIMKPLMEFSATLDTVV--PNSGDLFKAGSA---TQNQVPDQEPPPPLTPPA--GDNMLDMDLRGIYNNRQDFTATFR--PTAGMTFN------------GGVGPNGLTKKQEMLVRSAIKYWVERHKHIVRLVTAIGDAYGIALLLHMLTTTITLTLLAYQATKIHGVDTYSASVIGYLLYSLGQVFMLCIFGNRLIEESSSVMEAAYSCHWYDGSEEAKTFVQIVCQQCQKAMSISGAKFFTVSLDLFASVLGAMVTYFMVLVQLK

>XP_017766283.1_PREDICTED:_LOW_QUALITY_PROTEIN:_odorant_receptor_coreceptor_[Eufriesea_mexicana]

------MMKFKQQGLVADLLPNIKLMKATGHFLFNYYTD---SSTKHIHKIYCIVHLILVLMQFGFCGINLMLESDDVDDLTANTITMLFFTHCVVKLIYFAVRSKLFYRTLGVWNNPNSHPLFAESNARYHQKAIKKMRILLLIVMVITVLSAISWTTITFIGDSVKKVIDPV-TNESTYVEIPRLMVRSWYPYDPSHGMAHIFTLILQFYWLIFCMADANLLDVLFCSWLVFACEQIQHLKNIMKPLMEFSATLDTVV--PNSGELFKVSXD--PDPLPLSTP----------PQGENMLDMDLRGIYSNRTDFTTTFR--PTAGMTFN------------GGVGPNGLTKKQEMLVRSAIKYWVERHKHIVRLVTAIGDAYGLALLLHMLTTTITLTLLAYQATKIHAVDTYAASVIGYLVYSLGQVFMLCIFGNRLIEESSSVMEAAYSCHWYDGSEEAKTFVQIVCQQCQKAMSISGAKFFTVSLDLFASVLGAMVTYFMVLVQLK

>XP_017793198.1_PREDICTED:_odorant_receptor_coreceptor_[Habropoda_laboriosa]_

------MMKFKQQGLVADLMPNIKLMKASGHFLFNYYTD---NATKGMHKIYCIVHLVLILIQFGFCGINLMLESDDVDELTANTITMLFFTHSVVKLGYFAVRSKLFYRTFGIWNNPNSHPLFAESNARYHQIAVKKMRILLMAVMGTSVLTVISWTTITFIGDSVKKVVDPV-TNETSFVEVPRLMVRSWYPYDPSHGMAHILTLVFQLYWLIFTIADANLLDVLFCSWLLFACEQIQHLKNIMKPLMEFSATLDTVV--PNSGELFKAGAN--MDHGKDQ-DLPPP-ATP-HPQTDNMLDMDLRGIYSNRTDYTTTFR--ATAGATFN------------GGVGPNGLTQKQEMLVRSAIKYWVERHKHIVRLVTSVGDAYGVALLLHMLATTITLTLLAYQATKIHGVDTYAASVIGYLLYSLGQVFMLCIFGNRLIEESSSVMEAAYSCHWYDGSEEAKTFVQIVCQQCQKAMSISGAKFFTVSLDLFASVLGAMVTYFMVLVQLK

>XP_015126208.1_PREDICTED:_odorant_receptor_coreceptor_[Diachasma_alloeum]

------MMKTKHQGLVADLMPNIRLMQISGHFMFNYYGE---G-KKLMHKVYCSVHLFLIILQFGLCGINLAMESGDVDDLTANTITVLFFLHSVVKVVYFAVRSKLFYRTLAIWNNPNSHPLFAESNARYHSIALTKMRRLLFCVGAATVLSVICWTGITFFEDPHKTITDPI-TNETSTIEIPRLMVRSFYPFDARHGVAHIAMLVFQFYWLMITMVDANSLDVLFCSWLLFACEQLQHLKAIMKPLMELSATLDTVV--PNSSELFKAGSA---DHLRETNGTQPSATP---QQGDNMLDLDLRGIYSNRQDFTATFR--QAAGMQFN------------GGVGPNGLTKKQEMLVRSAIKYWVERHKHVVRLVTAIGDAYGVALLFHMLITTITLTLLAYQATKVNGVNVYAATTIGYLLYSLGQVFLFCIFGNRLIEESSSVMEAAYSCHWYDGSEEAKTFVQIVCQQCQKAMSISGAKFFTVSLDLFASVLGAVVTYFMVLVQLK

>NP_001164465.1_odorant_receptor_1_[Nasonia_vitripennis]

------MMKMKQQGLVADLLPNIRVMQGVGHFMFNYYSE---G-KKFPHRIYCIVTLLMLLMQYGMMAVNLMMESDDVDDLTANTITMLFFLHPIVKMIYFPVRSKIFYKTLAIWNNPNSHPLFAESNARFHALAITKMRRLLFCVAGATIFSVISWTGITFVDESVKRIVDPE-TNETTIIPIPRLMIRTFYPFNAMSGAGHVFALIYQFYYLIISMAISNSLDVLFCSWLLFACEQLQHLKAIMKPLMELSATLDTVV--PNSGELFKAGSA---DHLRDSQGVQPSG------NGDNVLDVDLRGIYSNRQDFTATFR--PTAGTTFN------------GGVGPNGLTKKQEMLVRSAIKYWVERHKHVVRLVTSVGDAYGVALLLHMLTTTITLTLLAYQATKVNGVNVYAATVIGYLLYTLGQVFLFCIFGNRLIEESSSVMEAAYSCHWYDGSEEAKTFVQIVCQQCQKAMSISGAKFFTVSLDLFASVLGAVVTYFMVLVQLK

>AIY24336.1_odorant_receptor_Or83b_[Chouioia_cunea]

------MMKMKQVGLVADLMPNIRITQAVGHWLFNYYSE---G-MRFPHKIYCMVTLFLMLFQFGTMALNLVKESDDVDQLTANTITVLFFMHPIVKVVYLAARAKIFYKCLGVWNNPNSHPLFAESNQRYHALALSKMRKLLFCVCGAVTFSVICWTGITFFDDAVRKIHDKE-TNETTIIPLPRLMIRSAYPWNAMSGAAHIFSMIYQFYYLVITMGICNMFDVLFCSFLLFACEQLQHLKAIMKPLMELSATLDTVV--PNSGELFKAGSA---DHLRESSGIQPSS------NGENVLDVDVRGIYSNRQDFTATFR--PTAGTTFN------------GGVGPNGLTKKQEMLVRSAIKYWVERHKHVVRLVTVVGDAYGVALLLHMLTTTITLTLLAYQATKVNGVNVYAATTIGYLLYTLGQVFLFCVFGNRLIEESSSVMEAAYSCHWYDGSEEAKTFVQIVCQQCQKAMSISGAKFFTVSLDLFASVLGAVVTYFMVLVQLK

>NP_001292395.1_odorant_receptor_coreceptor_[Ceratosolen_solmsi_marchali]

-------MKFKHQGLMADLMPNIRLMQSVGHFLFNYYSE---G-KKFPHKIYCVVTLLLILAQYSLMGVNLMMESDDVDDLTANTITMLFFVHPVVKVIYFPIRSKMFYKTLAIWNNPNSHPLFAESNARYHSLAITKMRRLLFCVAAATVFSVISWTGITFMDESVKRIIDPE-TNETTITPIPRLMIRTFYPWNAMSGAGHVFSLFYQFYYLAIVMAISNSLDVLFCSWLLFACEQLQHLKAIMKPLMELSATLDTVV--PNSGELFKAGSA---DHLRDTQGVQPSG------NGDNIVDVDVRGIYSNRQDFTATFR--PTAGTTFN------------GGVGPNGLSKKQEMLVRSAIKYWVERHKHVVRLVTSIGDAYGIALLLHMLATTITLTLLAYQATKVNGVNVYAATVVGYLLYTLGQVFLFCIFGNRLIEESSSVMEAAYSCHWYDGSEEAKTFVQIVCQQCQKAMSISGAKFFTVSLDLFASVLGAVVTYFMVLVQLK

>ABY51615.1_candidate_odorant_receptor_2_[Apocrypta_bakeri]

-------MKFKHQGLVADLLPNIRVMQGVGHFMFNYYSE---G-KKFPHRIYCIVTLLLLLLQYGMMAVNLMMESDDVDDLTANTITMLFFLHPIVKMIYFPVRSKIFYKTLAIWNNPNSHPLFAESNARFHALAITKMRRLLFCVAGATIFSVISWTGITFIEDSVKRITDPE-TNETTIIPIPRLMIRTFYPFNAMSGAGHVFALIYQFYYLVISMAVSNSLDVLFCSWLLFACEQLQHLKAIMKPLMELSATLDTVV--PNSGELFKAGSA---DHLRESQGVQPSG------NGDNVLDVDLRGIYSNRQDFTATFR--PTAGTTFN------------GGVGPNGLTKKQEMLVRSAIKYWVERHKHVVRLVTAVGDAYGVALLLHMLTTTITLTLLAYQATKVNGVNVYAATVIGYLLYTLGQVFLFCIFGNRLIEESSSVMEAAYSCHWYDGSEEAKTFVQIVCQQCQKAMSISGAKFFTVSLDLFASVLGAVVTYFMVLVQLK

>XP_014601852.1_PREDICTED:_odorant_receptor_coreceptor_isoform_X2_[Polistes_canadensis]

-------MKLKQQGLVADLLPNIRVMQFGGHFMFNYV-E---NSTKFMQKIYCCVHLFLMLLQFGLAGVNLALESGDVDDLTANTITLLFFTHPVVKIIYFAVRSKLFYRVLAIWNNPNSHPLFAESNARYHAIALKKMRTLLFAVGATTVFSVFAWTGITFVGDSVKKVTDPE-TNETTLMEIPRLMLRSWYPYDPSHGMSHVMTLVYQFYWLFFTMMNSNSLDVLFCSWLLFACEQLQHLKQIMKPLMELSATLDTVV--PNSSELFKAGSA---EHLRDTQGTQPPPQPP--PGGESMLDLDLRGIYSNRQDFTATFR--PTAGMTFN------------GGVGPNGLTKKQEMLVRSAIKYWVERHKHVVRLVTAIGDAYGIALLLHMLTTTITLTLLAYQATKVNAVDVYSATVIGYLLYTLGQVFLFCIFGNRLIEESSSVMEAAYSCHWYDGSEEAKTFVQIVCQQCQKAMSISGAKFFTVSLDLFASVLGAVVTYFMVLVQLN

>XP_015184459.1_PREDICTED:_odorant_receptor_coreceptor_[Polistes_dominula]

-------MKLKQQGLVADLLPNIRVMQFGGHFMFNYV-E---NSTKFMQKIYCCVHLFLILLQFGLAGVNLALESGDVDDLTANTITLLFFTHPVVKIIYFAVRSKLFYRVLAIWNNPNSHPLFAESNARYHAIALKKMRTLLFAVGATTVFSVFAWTGITFVGDSVKKVTDPE-TNETTLMEIPRLMLRSWYPYDPSHGMSHVMTLVYQFYWLFFTMMNSNSLDVLFCSWLLFACEQLQHLKQIMKPLMELSATLDTVV--PNSSELFKAGSA---EHLRDTQGTQPPPQPP--PGGESMLDLDLRGIYSNRQDFTATFR--PTAGMTFN------------GGVGPNGLTKKQEMLVRSAIKYWVERHKHVVRLVTAIGDAYGIALLLHMLTTTITLTLLAYQATKVNAVDVYSATVIGYLLYTLGQVFLFCIFGNRLIEESSSVMEAAYSCHWYDGSEEAKTFVQIVCQQCQKAMSISGAKFFTVSLDLFASVLGAVVTYFMVLVQLN

>XP_014224691.1_PREDICTED:_odorant_receptor_coreceptor_[Trichogramma_pretiosum]

------MMKMKQQGLVADLLPNIRVMQFSGHFMFNYYNE----GLKFPHRVFCIVSFLLILVQYSMMGINLAMEVGDPDDMAANTITMLFFIHPIVKTIYFAARSKTFYKTLGIWNNPNTHPLFAESDAHYHSVAVQKMRKLATFVGAATIFTLFAWTTITFFEDSVKTVVDKE-TNETTIIPIPRLPIRAWYPFNSMKGFGHIMAFVYQFYYLAMCLTLSISVDVLFCSWLIFACEQIMHLKAIMKPLMELSATLDTVV--PNSGDLFKAGSD---DHLRDTNGVQPA--------GDGIVDSDVRGIYSNRQDFTATFR--PTAGTNFN------------GNVGPNGLTKKQEMLVRSAIKYWVERHKHVVKLVTAIGDTYGVALLLHMLATTITLTLLAYQATKINGVNPYGATVIGYLLYTLGQVFHFCIFGNRLIEESSSVMEAAYSCHWYDGSEEAKTFVQIVCQQCQKAMSISGAKFFTVSLDLFASVLGAVVTYFMVLVQLK

>XP_012273699.1_PREDICTED:_odorant_receptor_coreceptor_[Orussus_abietinus]

------MMKFKQQGLVADLMPNIRIMQYTGHFMFNYYNDAG-GSIKFIHKIFCSVHLFLILLQFALCGVNLLFESGDVDDLTANTITMLFFTHSVVKLLYFAVRSKLFYRTLGIWNNPNTHPLFAESNARYHAIALTKMRRLLFCVGAATILSALAWTGITFVGDSVKKSIDPV-TNETVIVEIPRLMLRSWYPFDASHGIAHIMILIYQFYWLLFSLADANSLDVLFCSWLLFACEQLQHLKQIMKPLMELSATLDTVV--PNSSELFKAGSA---DHLRENQGSELSAPPP---TSDNMLDLDLRGIYSNRQDFTATFR--PTAGMTFN------------GGVGPNGLTKKQEMLVRSAIKYWVERHKHVVRLVTAIGDAYGVALLLHMLTTTITLTLLAYQATKVNSVDVYAATVIGYLLYTLGQVFLFCIFGNRLIEESSSVMEAAYSCHWYDGSEEAKTFVQIVCQQCQKAMSISGAKFFTVSLDLFASVLGAVVTYFMVLVQLK

>NP_001310774.1_odorant_receptor_coreceptor_[Cephus_cinctus]

------MMKFKQQGLVADLMPNIRHMQFSGHFMFNYYNDTG-GSTKLFHTIYCSIHLFLILLQFGLCCVNLTLERADVDDLTANTITVLFFAHSIIKLAYFAVRSKLFYRTLGIWNNPNSHPLFAESNARYHAIALTKMRRLLAAVGAATILTVCAWTGITFVGDSVKKVTDPV-TNETMTVEIPRLMLRSWYPYDASHGMAHVLTLIYQFYFLLITTMDANSLDVLFCSWLLFACEQLQHLKQIMKPLMELSATLDTVV--PHTNELFKAGST---DHLRDTQGTQPMAPP----PNENMLDMDLRGIYSNRQDFTATFR--TAAGMNFN------------GGVGPNGLTKKQEMLVRSAIKYWVERHKHIVRLVTAIGDAYGVALLFHMLITTVSLTLLAYQATKVNTVDVYAATVIGYVLYTLGQVFLFCIFGNRLIEESSSVMEAAYSCHWYDGSEEAKTFVQIVCQQCQKAMSISGAKFFTVSLDLFASVLGAVVTYFMVLVQLK

>XP_014219015.1_PREDICTED:_odorant_receptor_coreceptor_[Copidosoma_floridanum]

------MMKMKQHGLVADLLPNIRSMQFAGHFMFNYYSE----GKKFPHKMFCFISLFIILIQYVLMGLNLLIESDDVDDMTANTITMLFFAHPIVKTLYFPLFRKVFYKTLAVWNNPNSHPLFAESNARYHALAISKIRKLLFFVMSMTFVSTICWTGITFFEKPVRLRVDKE-TNETISTKLPKLMMRSYYPYDAMGGMNHMLTLLYQFYFLLVTMSLSNSIDTIFCSWLLFACEQLQHLKDIMKPLMELSATLDTVV--PNSGELFKAGSA---DHLRDSSIMPSS------NGDGGVVDSDLRGIYSNRQDFTATFR--PTAGMNFN------------GGVGPNGLTKKQEMLVRSAIKYWVERHKHVVRLTGLIGDAYGVALLLHMLTTTITLTLLAYQATKVNGVNVYAFTVIGYLVYTLAQVFLFCIFGNRLIEESSSVMEAAYSCHWYDGSEEAKTFVQIVCQQCQKAMSISGAKFFTVSLDLFASVLGAVVTYFMVLVQLK

>AQN78403.1_olfactory_receptor_1_[Meteorus_pulchricornis]

------MMKTKYQGLVADLMPNIRLMQISGHFMFNYYGE----GKKFMHKIYCSIHLFLILLQFALCGLNLAMESDDVDVLTANTVTLLFFSHTVIKIIYFAFRSKLFYRTLAIWNNPNSHPLFAESNARYHSIALTKMRRLLFCVGAATVASIISWVVLTLVEDDPVREIVDKVTNETTIIPLPRLPVRSFYPFDARHGVAHIAMFAFQLYWLIITMFNANSIDVLFCSWLLFACEQLQHLKAIMKPLMELSATLDTVV--PNSSELFKAGSA---DHLRDTTGTVPSATQ---PNGDNMLDLDLRGIYSNRQDFTATFR--PTDRTQYN------------GGVGPNGLTKKQEMLVRSAIKYWVERHKHVVRVVTAIGDAYGVALLFHMLITTITLTLMAYQATKVNGINVYAASTIGYLLYSLGQVFLFCIFGNRLIEESSSVMEAAYSCHWYDGSEEAKTFVQIVCQQCQKAMSISGAKFFTVSLDLFASVLGAVVTYFMVLVQLK

>ALG36144.1_odorant_receptor_1,_partial_[Sclerodermus_sp._MQW-2015]

------MMKFKQQGLVADLMPNIRLMQISGHFLFNYYND---GGTKFMHRIYCCVHLFLIVLQYGLMGVNLILESGDVDDLTANTITMLFFTHSIVKVIYFAVRSKLFYRTLGIWNNPNSHPLFAESNARYHSIALTKMRRLLFCVGAATVFSVLAWTGITFLEDPWKKVIDPV-TNETTLVEMPRLMLRAWYPFDVKHGMSHVLILIFQFYWLLFAMTDANSLDVLFCSWLLFACEQLQHLKQIMKPLMELSATLDTVV--PNSSELFKAGSA---DHLRDTQGTQPALPQP--AQGENMLDLDLRGIYSNRQDFTATFR--PTAGMTFN------------GGVGPNGLTKKQEMLVRSAIKYWVERHKHVVRLVTSIGDSYGIALLLHMLATTITLTLLAYQATKVHAVDVYAATVIGYLLYSLGQVFLFCIFGNRLIEESSSVMEAAYSCHWYDGSEEAKTFVQIVCQQCQKAMSISGAKFFTVSLDLFASVLGAVVTYFMVLVQLK

>AKO69815.1_olfactory_receptor_co-receptor_[Campoletis_chlorideae]

------MMKFKQQGLVSDLMPNIRLMQISGHFMFNYYAD----GMKFMHKIYCVVHLVLVLVQFGLCLVNMALESGDVDDLTANTITVLFFAHSIVKIGYFAVRSKLFYRTLAIWNNPNSHPLFAESNARYHAIALTKMRRLLFAVSGATILSVLCWTGITFVGDSVKKVVDPV-TNETTMVEIPRLMLRSYYPFDASHGMAHILTLVYQFYWLVFTMFDANSIDVLFCSWLLFACEQLQHLKQIMKPLMELSATLDTVV--PNSNELFKAGSA---EHLRDNSNN-VQPPPS--AQGENMLDLDLRGIYSNRQDFTATFR--PTAGTQFN------------GGVGPNGLTKKQEMLVRSAIKYWVERHKHVVRLVTAIGDAYGFALLLHMLITTITLTLLAYQATKVNSVDVYAATTIGYLLYSLGQVFLFCIFGNRLIEESSSVMEAAYSCHWYDGSEEAKTFVQIVCQQCQKAMSISGAKFFTVSLDLFASVLGAVVTYFMVLVQLK

>XP_011296908.1_PREDICTED:_odorant_receptor_coreceptor_[Fopius_arisanus]

------MMKTKHQGLVADLMPNIRLMQISGHFMFNYYGE----GKKLMHKVYCSVHLFLIVLQFALCGINLAMESSDVDDLTANTITVLFFLHSVVKVIYFAVRSKLFYRTLAIWNNPNSHPLFAESNARYHSIALTKMRRLLFCVGAATILSVLCWTGITFFEDPHKKIIDPI-TNETTMVEIPRLMVRSFYPFDARHGVAHIAMLVFQFYWLLVCMVDANSLDVLFCSWLLFACEQLQHLKAIMKPLMELSATLDTVV--PNSSELFKAGSA---DHLRETNGTTQPSATP--QQGDNMLDLDLRGIYSNRQDFTATFR--QAAGMQFN------------GGVGPNGLTKKQEMLVRSAIKYWVERHKHVVRLVTAIGDAYGVALLFHMLITTITLTLLAYQATKVNGVNVYAATTIGYLLYSLGQVFLFCIFGNRLIEESSSVMEAAYSCHWYDGSEEAKTFVQIVCQQCQKAMSISGAKFFTVSLDLFASVLGAVVTYFMVLVQLK

>XP_015513389.1_PREDICTED:_odorant_receptor_coreceptor,_manually_corrected_[Neodiprion_lecontei]

------MMKYKQQGLVADLMPNIRLMQFTGHFFFQYYNDAGGSNIKLFHKIYCVVHLILILLQFSLCGLNLFFERDNVEDMTANTITLLFFTHSLSKLTYAGARSKMFYRTLGIWNNPNSHPLFAESNARYHAIALSKNRRLLTSVTAATVFSVLAWTGLTFMGDSVKNIVDKE-TNETTTIEIPRLMLRSWYPYDAGHGVAHVATLIFQFYWVLVCLMSASMLDVLFCSWLLFACEQIQHLKQIMKPLIELSATLDTVV--PHSNELFKAGST---DHLRDNQPPPPPEND--------MLDMDLRGIYSNRQDFTATFR--STTGLGFS------------GGVGPNGLTKKQEILVRSAIKYWVERHKHVVRLVTAVGDTYGFALLIHMLIATITLTLLAYQATKISGFDVYSMGVIGYILYSLGQVFLFCIFGNRLIEESSSVMDAAYSCQWYDGSEEAKTFVQIVCQQCQKAMSVSGAKFFTVSLDLFASVVGAMVTYFMVLMQLG

>AGI62937.2_olfactory_coreceptor_[Macrocentrus_cingulum]

-------MKFKQQGLVADLMPNIRLMQISGHFMFNYYSD----GKKFMHKIYCSVHLFLILLQFALCGLNLAMEADDVDQLTANTVTVLFFLHAIVKIGYFGVRSKLFYRTLAIWNNPNSHPLFAESNARYHSIALTKMRRLLFCVGAATVLTIIAWTGITFAENPVRILTDKV-TNETTTIELPRLMVRSWYPFNAKSGMAHIGMLIFQFYWLTITMVDCNSLDVLFCSWLLFACEQLQHLKAIMKPLMELSATLDTVV--PNSSELFKAGSA---DHLRDTAGSVPSATQP---NGESMLDLDLRGIYSNRQDFTATFR--PTAGTQFT------------GGVGPNGLTKKQEMLVRSAIKYWVERHKHVVRLVTAIGDAYGVALLFHMLITTISLTLLAYQATKVNGLNVYAATTIGYFSYALAQVFLFCIFGNRLIEESSSVMEAAYSCHWYDGSEEAKTFVQIVCQQCQKAMSISGAKFFTVSLDLFASVLGAVVTYFMVLEQLK

>ACU31808.1_candidate_odorant_receptor_2_[Ceratosolen_cornutus]

-------MKFQHQGLVADLLPNIRVMQSVGHFVFNYYSE----GKKFPHKIYCIVTLLLLLTQYALMAVNLMMESGDVDDLTANTITMLFFLHPVVKLIYFPVRGKIFYKTLAIWNNPNSHPLFAESNARYHSLSITKMRRLLFCVAATTVFSVLAWTSITFMDESVKRIIDPE-TNETTVVPIPRLMIRTFYPWNAINGAGHVFSFIYQFYYLFFSMAISNSLDVLFCSWLLFACEQLQHLKAIMKPLMELSATLDTVV--PNSGELFKAGSA---DHLRDTQGVQPSG------NGDNVVDVDVRGIYSNRQDFTATFR--PTAGTTFN------------GGVGPNGLSKKQEMLVRSAIKYWVERHKHVVRLVTSVGDAYGVALLLHMLSTTITLTLLAYQATKVNGANVYAATVIGYLLYTLGQVFLFCVFGNRLIEESSSVMEAAYSCHWYDGSEEAKTFVQIVCQQCQKAMSISGAKFFTVSLDLFASVLGAVVTYFMVWVQLK

>ABY51617.1_candidate_odorant_receptor_2_[Philotrypesis_sp._BL-2007]

-------MKIKHQGLVADLLTNIRVMQGVGHFMFNYYS----GGKKFPHKIYSVVTLLLLLIQYGMMAVNLMMESDDVDDLTANTITMLFFLHPIVKMIYFLVRSKIFYKTLAIWNNPNSHPLFAESNARFHALAVTKMRRLLFCVAGATIFSVISWTGITFVDESVKRIIDAE-TNETTIIPIPRLMIRTFYPFNAMSGAGHVFAFIYQFYYLIISMAVSNSLDVLFCSWLLFACEQLQHLKAIMKPLMELSATLDTVV--PNSGELFKAGSA---DHLRESQGIQPSG------NGDNVLDVDLRGIYSNRQDFTATFR--PTAGTTFN------------GGVGPNGLTKKQEMLVRSAIKYWVERHKHVVRLVTSVGDAYGVALLLHMLTTTITLTLLAYQATKVNGVNVYAATVIGYLLYTLGQVFLFCIFGNRLIEESSSVMEAAYSCHWYDGSEEAKTFVQIVCQQCQKAMSISGAKFFTVSLDLFASVLGAVVTYFMVLVQLK

>ABY51616.1_candidate_odorant_receptor_2_[Philotrypesis_pilosa]

-------MKFKQQGLIADLLPNIRVMQGVGHFMFNYYSE----GKKFPHKIYCIVTLLLLLMQYGMMAVNLMMESDDVDDLTANTITMLFFLHPIVKIIYFLVRSKIFYKTLAIWNNPNSHPLFAESNARLHALAVTKMRRLLFCVAGATIFSVISWTGITFADESVKRIIDAE-TNETTVIPIPRLMIRTFYPFNAMSGAGHVFAFIYQFYYLIISMAVSNSLDVLFCSWLLFACEQLQHLKAIMKPLMELSATLDTVV--PNSGELFKAGSA---DHLRESQGIQPSG------NGDNVLDVDLRGIYSNRQDFTATFR--PTAGTTFN------------GGVGPNGLTKKQEMLVRSAIKYWVERHKHVVRLVTSVGDAYGVALLLHMLTTTITLTLLAYQATKVNGVNVYAATVIGYLLYTLGQVFLFCIFGNRLIEESSSVMEAAYSCHWYDGSEEAKTFVQIVCQQCQKAMSISGAKFFTVSLDLFASVLGAVVTYFMVLVQLK

>XP_017890983.1_PREDICTED:_odorant_receptor_coreceptor_[Ceratina_calcarata]

------MMKFKQQGLVADLMPNIKLMKAFGHFMFNYYED---GSSKHIHKIFCIIHLVLVLMQFGFCGINLMLEADDVDVLTANTITLLFFTHSVVKLIYFAVRSKLFYRTLGIWNNPNSHPLFAESNARYHQVAIKKMRILLMAVMGATCLTAISWTTLTFIEDPVRTIIDKE-TNETTTIQLPRLLVPSWYPFNPSHGVAHILTLIFQLYWVIFCMMDANLLDVLFCSWLLFACEQIQHLKNIMKPLMEFSATLDTVV--PHSGELFKSTSA---ERARD--DLPPPPTPP--PPGENMLDMDLRGVYSNRTDFTATFR--PTAGMNFN------------GGVGPNGLTKKQEMLVRSAIKYWVERHKHIVRLVTAVGDAYGVALLLHMLTTTITLTLLAYQATKIHGVDTYSASVIGYLLYSLGQVFLLCIFGNRLIEESSSVMEAAYSCHWYDGSEEAKTFVQIVCQQCQKAMSISGAKFFTVSLDLFASVLGAMVTYFMVLVQLK

>XP_003402775.1_PREDICTED:_odorant_receptor_coreceptor_[Bombus_terrestris]

------MMKFKQQGLVADLMPNIRLMKATGHFMFNYYTDNS---TKTIHRIFAVVHLILMLMQFGFCGINLIFEKEDVDDLTANTITMLFFTHSVVKVVYFAVRSKLFYRTLGIWNNPNSHPLFAESNSRYHQVAVRKMRILLLAVLVTTMLSAISWTSITFIGDSVKKVIDPI-TNETTYVEIPRLMLRSWYPYNASHGMAHILTLIFQFYWLVFCMADANLLDVLFCSWLLFACEQIQHLKNIMKPLMEFSATLDTVV--PNSGDLFKAGSA---EQPRDH-DPLPPTTPT--APGENMLDMDLRGIYSNRTDFTATFR--PTAGMTFN------------GSVGPNGLTKKQEMLVRSAIKYWVERHKHIVRLVTAIGDAYGVALLLHMLITTITLTLLAYQATKINAVDTYAASVIGYLLYSLGQVFMLCIFGNRLIEESSSVMEAAYSCHWYDGSEEAKTFVQIVCQQCQKAMSISGAKFFTVSLDLFASVLGAMVTYFMVLVQLK

>XP_012253637.1_PREDICTED:_odorant_receptor_coreceptor_[Athalia_rosae]

------MMKYKQEGLVADLMPNIRIMQISGHFMFNYYNDAGGSSIKLFHQIYCVVHLVLILLQFGLCCVNLIQESGDVDDLTADTITILFFAHALIKLGYFAIRSKMFYRTFGIWNNPNSHPLFAESNARYHALALTKMRRLLMAVGITTILSVIAWTGITFVGDSVKTTVDKE-TNETITVEIPRLMLRSWYPFDASHGMAHAIVVGYQFYWLLITMVDSNMLDVLFCSWLLFACEQLQHLKQIMKPLMELSATLDTVV--PHTNDLFKAGST---EHLRDN-EPPPPPPP---NE---LLDLDLRGIYSNRQDFTATFK--SSGGITFN------------GGVGPNGLTKKQEMLVRSAIKYWVERHKHVVRLVTSIGDAYGVALLFHMLVSTVTLTLLAYQATKVHGINVYAASVIGYLLYTLGQVFLFCIFGNRLIEESSSVMEAAYSCHWYDGSEEAKTFVQIVCQQCQKAMTISGAKFFTVSLDLFASVLGAVVTYFMVLVQLN

>KOX76355.1_Gustatory_and_odorant_receptor_7_[Melipona_quadrifasciata]

------MMKFKQQGLVADLMPNIKLMKATGHFMFNYYSDSS---TKRIHQIFCIVHLMLVLTQFGFCGINLMMESDDVDDLTANTITMLFFTHSVVKVVYFAVRSKLFYRTLAVWNNPNSHPLFAESNARYHQIAVKKMRILLLAVMGTTVLSTISWTTITFIGDSVKKVIDPV-TNETTFVEIPRLMVRSWYPYNPSHGIAHILTLVFQFYWLLFCMADANLLDVLFCSWLLFACEQIQHLKNIMKPLMEFSATLDTVV--PNSGELFKAGSA---EHPRET-EPLPPPTPP---QGENMLDMDLRGIYSNRTDFTATFR--PTAGMTFN------------GGVGPNGLTKKQEMLVRSAIKYWVERHKHIVRLVTAIGDAYGVALLLHMLCTTITLTLLAYQATKIHGVDTYSASVIGYLLYSLGQVFMLCIFGNRLIEESSSVMEAAYSCHWYDGSEEAKTFVQIVCQQCQKAMSISGAKFFTVSLDLFASVLGAMVTYFMVLVQLK

>XP_003494153.1_PREDICTED:_odorant_receptor_coreceptor_[Bombus_impatiens]

------MMKFKQQGLVADLMPNIRLMKATGHFLFNYYTDNS---TKNIHRIFAIVHLVLMLMQFGFCGINLFFEKEDVDDLTANTITMLFFTHSVIKVVYFAVRSKLFYRTLGIWNNPNSHPLFAESNARYHQVAVRKMRILLLAVLATTMLSAISWTSITFIGDSVKKVIDPI-TNETTYVEIPRLMLRSWYPYNASHGMAHILTLIFQFYWLVFCMADANLLDVLFCSWLLFACEQIQHLKNIMKPLMEFSATLDTVV--PNSGELFKSGSA---EQPRDH-DPLPPTTPT--APGENMLDMDLRGIYSNRTDFTATFR--PTAGMTFN------------GGVGPNGLTKKQEMLVRSAIKYWVERHKHIVRLVTAIGDAYGVALLLHMLITTITLTLLAYQATKINAVDTYAASVIGYLLYSLGQVFMLCIFGNRLIEESSSVMEAAYSCHWYDGSEEAKTFVQIVCQQCQKAMSISGAKFFTVSLDLFASVLGAMVTYFMVLVQLK

>ABM05966.1_olfactory_receptor_[Microplitis_mediator]

------MMKTKHQGLVADLMPNIRLMQISGHFMFNYYGE----GKKLMHKIYCSVHLFLILLQFGFVAINLVKEKEDVDDLTANTITILFFLHTLIKIVYFAARSKLFYRTLAIWNNPNSHPLFAESNARYHSIALTKVRRLLFCVGAATVATTISWTTLTFFEDPHVERLNKE-TNETYIEEIPRLLVRSWYPFDARHGVAHIGMLIYQIYWLFICTVDANSIDVLFCSWLLFACEQLQHLKAIMKPLMELSATLDTVV--PNSGELFKAGSA---DHLRDN-DGV--PAEPA-MNGDNMLDMDLRGIYSNRQDFTATFR--PTAGTQYN------------GGVGPNQLTKKQEMLVRSAIKYWVERHKHIVRLVTAIGDAYGVALLFHMLITTITLTLLAYQATKVNGVNVYAASTIGYLLYSLGQVFLFCIFGNRLIEESSSVMEAAYSCHWYDGSEEAKTFVQIVCQQCQKAMSISGAKFFTVSLDLFASVLGAVVTYFMVLVQLK

>XP_014295003.1_PREDICTED:_odorant_receptor_coreceptor_[Microplitis_demolitor]

------MMKTKHQGLVADLMPNIRLMQISGHFMFNYYGE----GKKLMHKIYCSVHLFLILLQFGFVAINLVKEKEDVDDLTANTITILFFLHTLIKIIYFAARSKLFYRTLAIWNNPNSHPLFAESNARYHSIALTKMRRLLFCVGAATVATTISWTTLTFFEDPHVERINKE-TNETYIEEIPRLLVRSWYPFDARHGMAHIGMLIYQIYWLFICTVDANSIDVLFCSWLLFACEQLQHLKAIMKPLMELSATLDTVV--PNSGELFKAGSA---DHLRDN-DGVPAEPAM---NGDNMLDMDLRGIYSNRQDFTATFR--PTAGTQYN------------GGVGPNQLTKKQEMLVRSAIKYWVERHKHIVRLVTAIGDAYGVALLFHMLITTITLTLLAYQATKVNGVNVYAASTIGYLLYSLGQVFLFCIFGNRLIEESSSVMEAAYSCHWYDGSEEAKTFVQIVCQQCQKAMSISGAKFFTVSLDLFASVLGAVVTYFMVLVQLK

>XP_006610550.1_PREDICTED:_gustatory_and_odorant_receptor_7-like_[Apis_dorsata]

------MMKFKQQGLIADLMPNINLMKATGHFMFNYYTDSS---TKHIHKIYCIVHL---LEIMLFCGINLMMESDDVDDLTANTITMLFFTHSVVKLVYFAVRSKLFYRTLGIWNNPNSHPLFAESNARYHQIAVKKMRILLLAVIGTTVLSAISWTTITFIGDSVKKVIDPV-TNETTYVEIPRLMVRSWYPYDPSHGMAHILTLIFQFYWLIFCMADANLLDVLFCSWLLFACEQIQHLKNIMKPLMEFSATLDTVV--PNSGELFKAGSA---EQPKEQ-EPLPPVTP---PQGENMLDMDLRGIYSNRTDFTTTFR--PTAGMTFN------------GGVGPNGLTKKQEMLVRSAIKYWVERHKHIVRLVTAIGDAYGVALLLHMLTTTITLTLLAYQATKIHAVDTYAASVVGYLLYSLGQVFMLCIFGNRLIEESSSVMEAAYSCHWYDGSEEAKTFVQIVCQQCQKAMSISGAKFFTVSLDLFASVLGAMVTYFMVLVQLK

>XP_003690663.2_PREDICTED:_odorant_receptor_coreceptor_manually_corrected_[Apis_florea]

------MMKFKQQGLIADLMPNINLMKATGHFMFNYYTDSS---TKHIHKIYCIVHLVLILMQFGFCGINLMMESDDVDDLTANTITMLFFTHSVVKLVYFAVRSKLFYRTLGIWNNPNSHPLFAESNSRYHQIAVKKMRILLLAVIGTTVLSAISWTTITFIGDSVKKVIDPV-TNETTYVEIPRLMVRSWYPYDPSHGMAHILTLIFQFYWLIFCMADANLLDVLFCSWLLFACEQIQHLKNIMKPLMEFSATLDTVV--PNSGELFKAGSA---EQPKEQ-EPLPPITP---PQGENMLDMDLRGIYSNRTDFTTTFR--PTAGMTFN------------GGVGPNGLTKKQEMLVRSAIKYWVERHKHIVRLVTAIGDAYGVALLLHMLTTTITLTLLAYQATKIHAVDTYAASVVGYLLYSLGQVFMLCIFGNRLIEESSSVMEAAYSCHWYDGSEEAKTFVQIVCQQCQKAMSISGAKFFTVSLDLFASVLGAMVTYFMVLVQLK

>NP_001315406.1_odorant_receptor_coreceptor_[Apis_cerana]

------MMKFKQQGLIADLMPNINLMKATGHFMFNYYTDSS---TKHIHKIYCIVHLVLILMQFGFCGINLMMESDDVDDLTANTITMLFFTHSVVKLVYFAVRSKLFYRTLGIWNNPNSHPLFAESNARYHQIAVKKMRILLLAVIGTTVLSAISWTTITFIGDSVKKVIDPV-TNETTYVEIPRLMVRSWYPYDPSHGMAHILTLIFQFYWLIFCMADANLLDVLFCSWLLFACEQIQHLKNIMKPLMEFSATLDTVV--PNSGELFKAGSA---EQPKEQ-EPLPPVTPP---QGENMLDMDLRGIYSNRTDFTTTFR--PTAGMTFN------------GGVGPNGLTKKQEMLVRSAIKYWVERHKHIVRLVTAIGDAYGVALLLHMLTTTITLTLLAYQATKIHAVDTYAASVVGYLLYSLGQVFMLCIFGNRLIEESSSVMEAAYSCHWYDGSEEAKTFVQIVCQQCQKAMSISGAKFFTVSLDLFASVLGAMVTYFMVLVQLK

>AHJ37468.1_olfactory_receptor_2_[Apis_mellifera]

------MKFKQQQGLIADLMPNINLMKATGHFMFNYYTDSS---TKHIHKIYCIVHLVLILMQFGFCGINLMMESEDVDDLTANTITMLFFTHSVVKLVYFAVRSKLFYRTLGIWNNPNSHPLFAESNARYHQIAVKKMRILLLAVIGTTVLSAISWTTITFIGDSVKKVIDPV-TNETTYVEIPRLMVRSWYPYDPSHGMAHILTLIFQFYWLIFCMADANLLDVLFCSWLLFACEQIQHLKNIMKPLMEFSATLDTVV--PNSGELFKAGSA---EQPKEQ-EPLPPVTP---PQGENMLDMDLRGIYSNRTDFTTTFR--PTAGMTFN------------GGVGPNGLTKKQEMLVRSAIKYWVERHKHIVRLVTAIGDAYGVALLLHMLTTTITLTLLAYQATKIHAVDTYAASVVGYLLYSLGQVFMLCIFGNRLIEESSSVMEAAYSCHWYDGSEEAKTFVQIVCQQCQKAMSISGAKFFTVSLDLFASVLGAMVTYFMVLVQLK

>BAO48211.1_odorant_receptor_co-receptor_[Camponotus_japonicus]

------MMKKQQQGLVADLYPNIRVMKMFGHFVFNYYDDNS---SKYLHKVYCCVNLFLLLLQFGLCAVNLIIESADVDDLTANTITLLFFTHSIVKIVYFAVRSKYFYRTWAIWNNPNSHPLFAESNARYHAIALKKMRLLLFLVGATTVLTAIAWTILTFFEHPIRKLVDPV-TNETTIIELPQLLVRSFYPFNASRGIKHILVLVYQFYWVLFMLINANSLDVLFCSWLLFACEQLQHLKQIMKPLMELSATLDTVV--PNSSELFKAGSA---DHLRES-ENNQSQLPAP-PQGDSMLDLDLRNIYSNRQDFTATFR--PTAGMTFN------------GGVGPNGLTKKQEMLVRSAIKYWVERHKHIVRLVTAVGDAYGFALLLHMLTTTITLTLLAYQATKVNGINVYAASTIGYVLYTFGQVFLFCIFGNRLIEESTSVMEAAYSCHWYDGSEEAKTFVQIVCQQCQKAMSISGAKFFTVSLDLFASVLGAVVTYFMVLVQLK

>XP_011867244.1_PREDICTED:_odorant_receptor_coreceptor_[Vollenhovia_emeryi]

-------MMKQQQGLVADLMPNIRVMKIFGHFVFNYYDDNS---SKYLHKVYCCVNLFLLLLQFGLCAVNLIIESADVDDLTANTITLLFFTHSIVKIVYFAIRSKYFYRTWAIWNNPNSHPLFAESNARYHAIALKKMRLLLFLVGATTVLAAVAWTTLTFFEHPIRKLVDPV-TNETTIIQLPQLLVRSFYPFNAGKGITHILILIYQLYWVLFMLINANSLDVLFCSWLLFACEQLQHLKQIMKPLMELSATLDTVV--PNSSELFKAGSA---DHLRES-DNQPPPPIP--PQGDSMLDLDLRNIYSNRQDFTATFR--PTAGMTFN------------GGVGPNGLTKKQEMLVRSAIKYWVERHKHIVRLVTAVGDAYGFALLLHMLTTTITLTLLAYQATKVNGINVYAASTIGYVLYTFGQVFLFCIFGNRLIEESTSVMEAAYSCHWYDGSEEAKTFVQIVCQQCQKAMSISGAKFFTVSLDLFASVLGAVVTYFMVLVQLK

>XP_011632710.1_PREDICTED:_odorant_receptor_coreceptor_[Pogonomyrmex_barbatus]

------MMKGKQQGLVADLAPNIRVMKIFGHFVFNYYDDNS---SKYLHKVYCCVNLFLLLLQFGLCAVNLIIESADVDDLTANTITLLFFTHSIVKIVYFAVRSKYFYRTWAIWNNPNSHPLFAESNARYHAIALKKMRLLLFLVGITTVLAAIAWTILTFFEHPIRKVVDPV-TNETTIIELPQLLVRSFYPFDAGKGITHILVLIYQFYWVLFMLINANSLDVLFCSWLLFACEQLQHLKQIMKPLMELSATLDTVV--PNSSELFKAGSA---DHLRES-ENNQPPPPA--PQGDSMLDLDLRNIYSNRQDFTATFR--PTAGMTFN------------GGVGPNGLTKKQEMLVRSAIKYWVERHKHIVRLVTAVGDAYGFALLLHMLTTTITLTLLAYQATKVNGINVYAASTIGYVLYTFGQVFLFCIFGNRLIEESTSVMEAAYSCHWYDGSEEAKTFVQIVCQQCQKAMSISGAKFFTVSLDLFASVLGAVVTYFMVLVQLK

>XP_011346854.1_PREDICTED:_odorant_receptor_coreceptor_[Cerapachys_biroi]

------MMKMKQQGLVADLLPNIRVMKTFGHFVFNYYNDNS---SKYLHKVYCCVNLFMLLLQFGLCAVNLIVESADVDDLTANTITLLFFTHSIVKICYFAIRSKYFYRTWAIWNNPNSHPLFAESNARYHAIALKKMRLLLFLVGGTTMLAAVAWTVLTFFEHPIRKIVDPV-TNETEIIELPQLLIRSFYPFDAGKGITHVLVLVYQFYWVLFMLIDANSLDVLFCSWLLFACEQLQHLKQIMKPLMELSATLDTVV--PNSSELFKAGSA---DHLRDG-DNPPPPPP---PQSDNMLDLDLRNIYSNRQDFTATFR--PTAGMTFN------------GGVGPNGLTKKQEALVRSAIKYWVERHKHIVRLVTAVGDAYGFALLLHMLTTTITLTLLAYQATKVNGINVYAASTIGYILYTFGQVFLFCIFGNRLIEESTSVMEAAYSCHWYDGSEEAKTFVQIVCQQCQKAMSISGAKFFTVSLDLFASVLGAVVTYFMVLVQLK

>XP_012526645.1_PREDICTED:_odorant_receptor_coreceptor_[Monomorium_pharaonis]

-----MMKMQKQQGLVADLMPNIRVMKIFGHFVFNYYDDNS---SKYLHKVYCCANLFLLLLQFGLCAVNLIIESADVDDLTANTITLLFFTHSIVKVVYFAIRSKYFYRTWAIWNNPNSHPLFAESNARYHAIALKKMRLLLFLVGITTVLTAVAWTTLTFFEHPIRKVVDPI-TNETEIIELPQLLVRSFYPFNAGKGVTHVLVLIYQFYWVLFMLINANSLDVLFCSWLLFACEQLQHLKQIMKPLMELSATLDTVV--PNSSELFKAGSA---DHLRES-ENQPPPPAA--AQGDNMLDLDLRNIYSNRQDFTATFR--PTAGMNFN------------GGVGPNGLTKKQEMLVRSAIKYWVERHKHIVRLVTAVGDAYGFALLLHMLTTTITLTLLAYQATKVNGVNVYAASTIGYVLYTFGQVFLFCIFGNRLIEESTSVMEAAYSCHWYDGSEEAKTFVQIVCQQCQKAMSISGAKFFTVSLDLFASVLGAVVTYFMVLVQLK

>XP_011139767.1_PREDICTED:_odorant_receptor_coreceptor_[Harpegnathos_saltator]

------MMKMKQQGLVADLLPNIRVMKFFGHFVFNYYDDNS---SKYLHKIFCCVNLFLLLLQFALCAVNLIIESADVDDLTANTITLLFFTHSIVKIIYFAVRSKYFYRTWAIWNNPNSHPLFAESNARYHAIALKKMRLLLFLVGATTVLSAIAWTVLTFFEHPIRKLVDPV-TNETTIIELPQLLLRSYYPFDASKGIMHVIVLIYQFYWVLFMLIDANSLDVLFCSWLLFACEQLQHLKQIMKPLMELSATLDTVV--PNSSELFKAGSA---EHLRES-ENQPPPPVP--PQGDSMLDLDLRNIYSNRQDFTATFR--PTAGMTFN------------GGVGPNGLTKKQEMLVRSAIKYWVERHKHIVRLVTAVGDAYGFALLLHMLTTTITLTLLAYQATKVNGVNVYAASTIGYIIYTFGQVFLFCIFGNRLIEESTSVMEAAYSCHWYDGSEEAKTFVQIVCQQCQKAMSISGAKFFTVSLDLFASVLGAVVTYFMVLVQLK

>XP_014483280.1_PREDICTED:_odorant_receptor_coreceptor_isoform_X2_[Dinoponera_quadriceps]

-----MLKMKQQQGLVADLMPNIRIMKIFGHFVFNYYDDNS---SKYLHKIYCCVNLFLLLLQFALCAVNLIIESADVDDLTANTITLLFFTHSIVKIVYFAVRSKYFYRTWAIWNNPNTHPLFAESNARYHAIALKKMRLLLFLVGATTVLSAVAWTVLTFFEHPIRKIVDPV-TNETTVIELPQLLLRSFYPFDASKGIAHVLILAYQFYWVLFMLVDANSMDVLFCSWLLFACEQLQHLKQIMKPLMELSATLDTVV--PNSSELFKAGSA---DHLRES-ENQPPPPVP--PQGDSMLDLDLRNIYSNRQDFTATFR--PTAGMTFN------------GGVGPNGLTKKQEALVRSAIKYWVERHKHIVRLVTAVGDAYGFALLLHMLTTTITLTLLAYQATKVNGVNVYAASTISYILYTFGQVFLFCIFGNRLIEESTSVMEAAYSCHWYDGSEEAKTFVQIVCQQCQKAMSISGAKFFTVSLDLFASVLGAVVTYFMVLVQLK

>XP_011164243.1_PREDICTED:_odorant_receptor_coreceptor_[Solenopsis_invicta]

----MSMMKMKQQGLVADLMPNIRVMKMFGHFVFNYYDDNS---SKYLHKVFCCINLFMLLLQFGLCAVNLIIESADVDDLTANTITLLFFTHSIVKVVYFAIRSKYFYRTWAIWNNPNSHPLFAESNARYHAIALKKMRLLLFLVGGTTVLAALAWTTLTFFEHPVRKIVDPI-TNETEVIQLPQLLVRSFYPFDASKGVTHVLVLIYQFYWVLFMLINANSLDVLFCSWLLFACEQLQHLKQIMKPLMELSATLDTVV--PNSSELFKAGSA---DHLRES-ENNQPPPPP--PQNDNMLDLDLRNIYSNRQDFTATFR--PTAGMTFN------------GGVGPNGLTKKQEMLVRSAIKYWVERHKHIVRLVTAVGDAYGFALLLHMLTTTITLTLLAYQATKVNGINVYAASTIGYVLYTFGQVFLFCIFGNRLIEESTSVMEAAYSCHWYDGSEEAKTFVQIVCQQCQKAMSISGAKFFTVSLDLFASVLGAVVTYFMVLVQLK

>XP_011253641.1_PREDICTED:_odorant_receptor_coreceptor_[Camponotus_floridanus]

-----MMKMKQQQGLVADLYPNIRVMKMFGHFVFNYYDDNS---SKYLHKVYCCVNLFLLLLQFGLCAVNLIIESADVDDLTANTITLLFFTHSIVKVVYFAVRSKYFYRTWAIWNNPNSHPLFAESNARYHAIALKKMRLLLFLVGATTVLTAIAWTVLTFFEHPIRKLVDPV-TNETTIIELPQLLVRSFYPFDASRGIKHILVLIYQFYWVLFMLINANSLDVLFCSWLLFACEQLQHLKQIMKPLMELSATLDTVV--PNSSELFKAGSA---DHLRES-ENNQSQLPAP-PQGDSMLDLDLRNIYSNRQDFTATFR--PTAGMTFN------------GGVGPNGLTKKQEMLVRSAIKYWVERHKHIVRLVTAVGDAYGFALLLHMLTTTITLTLLAYQATKVNGINVYAASTIGYVLYTFGQVFLFCIFGNRLIEESTSVMEAAYSCHWYDGSEEAKTFVQIVCQQCQKAMSISGAKFFTVSLDLFASVLGAVVTYFMVLVQLK

>XP_011700383.1_PREDICTED:_odorant_receptor_coreceptor_[Wasmannia_auropunctata]

------MMKMKQQGLVADLMPNIRIMKIFGHFVFNYYDDNS---SKYLHKVFCCVNLFLLLLQFGLCAVNLMFEAGDVDDLTANTITLLFFTHSIVKLIYFAIRSKYFYRTWAIWNNPNSHPLFAESNARYHAIALKKMRLLLFLVGGTTILAAVAWTTLTFFEHPIRKSVDPI-TNETTITELPQLLVRSYYPFDASKGVTHILILVYQLYWVIFMLINANSLDVLFCSWLLFACEQLQHLKQIMKPLMELSATLDTVV--PNSSELFKAGSG---DHLRESNENNQPPPPAA-NQGDNMLDLDLRNIYSNRQDFTATFR--PTAGMNFN------------GGVGPNGLTKKQEMLVRSAIKYWVERHKHIVRLVTAVGDAYGFALLLHMLTTTITLTLLAYQATKVNGINVYAASTIGYVLYTFGQVFLFCIFGNRLIEESTSVMEAAYSCHWYDGSEEAKTFVQIVCQQCQKAMSISGAKFFTVSLDLFASVLGAVVTYFMVLVQLK

>XP_018396655.1_PREDICTED:_odorant_receptor_coreceptor_[Cyphomyrmex_costatus]

------MMKMKQQGLVADLMPNIRVMKMFGHFVFNYYDDNS---SKYLHKVFCCINLFLLLLQFGLCAVNLIIESTDVDDLTANTITLLFFTHSIVKLVYFAIRSKYFYRTWAIWNNPNSHPLFAESNARYHAIALKKMRLLLFLVGGTTVLAAVAWTTLTFFEHPSRKLVDPI-TNETTIIELPQLLVRSFYPFDARRGVTHILMLIYQFYWVFFMLVNANSLDVLFCSWLLFACEQLQHLKQIMKPLMELSATLDTVV--PNSSELFKAGSA---DHLRESNENNQPPPPSAAAQGDNMLDLDLRNIYSNRQDFTATFR--PTAGMTFN------------GGVGPNGLTKKQEMLVRSAIKYWVERHKHIVRLVTAVGDAYGFALLLHMLTTTITLTLLAYQATKVSGINVYAASTIGYVLYTFGQVFLFCIFGNRLIEESTSVMEAAYSCHWYDGSEEAKTFVQIVCQQCQKAMSISGAKFFTVSLDLFASVLGAVVTYFMVLVQLK

>KMQ91335.1_gustatory_and_odorant_receptor_7_[Lasius_niger]

-----MMKMKQQQGLVADLMPNIRVMKMFGHFVFNYYDDNS---SKYLHKVFCCVNLFLLLLQFGLCAVNLIFESGDVDDLTANTITLLFFTHSIVKIVYFAVRSKYFYRTWAIWNNPNTHPLFAESNARYHAIALKKMRLLLFLVGATTVLTAVAWTILTFFEHPIRKLTDPV-TNETTIIHLPQLLVRSFYPFDASRGVTHILMLIYQFYWVLFMLVNANSLDVLFCSWLLFACEQLQHLKQIMKPLMELSATLDTVV--PNSSELFKAGSG---DHLRDQSEN-QPPPPAP-PQGDSMLDLDLRNIYSNRQDFTATFR--PTAGMTFN------------GGVGPNGLTKKQEMLVRSAIKYWVERHKHIVR----------------------------------------------------------------------------------------------------------------------------------------

>XP_018044486.1_PREDICTED:_odorant_receptor_coreceptor_[Atta_colombica]

------MMKMKQQGLVADLMPNIRVMKMFGHFVFNYYDDNS---SKYLHKIFCCANLFLLLLQFGLCAVNLIIESADVDDLTANTITLLFFTHSIVKIVYFAIRSKYFYRTWAIWNNPNTHPLFAESNARYHAIALKKMRLLLFLVGATTVLTAIAWTTLTFFEHPIRKLVDPI-TNETTIIQLPQLLVRSFYPFDASKGITHILMLIYQFYWVFFMLVNANSLDVLFCSWLLFACEQLQHLKQIMKPLMELSATLDTVV--PNSSELFKAGSA---DHLRESNENNQPPPPSAAAQGDNMLDLDLRNIYSNRQDFTATFR--PTAGMTFN------------GGVGPNGLTKKQEMLVRSAIKYWVERHKHIVRLVTAVGDAYGFALLLHMLTTTITLTLLAYQATKVNGINVYAASTIGYVLYTFGQVFLFCIFGNRLIEESTSVMEAAYSCHWYDGSEEAKTFVQIVCQQCQKAMSISGAKFFTVSLDLFASVLGAVVTYFMVLVQLK

>XP_011057328.1_PREDICTED:_odorant_receptor_coreceptor_[Acromyrmex_echinatior]

------MMKMKQQGLVADLMPNIRIMKMFGHFVFNYYDDNS---SKYLHKIFCCANLFLLLLQFGLCAVNLIIESADVDDLTANTITLLFFTHSIVKVVYFAIRSKYFYRTWAIWNNPNSHPLFAESNARYHAIALKKMRLLLFLVGVTTVLTAVAWTTLTFFEHPIRKLVDPI-TNETTIIQLPQLLVRSFYPFDASRGITHILILIYQFYWVFFMLVNANSLDVLFCSWLLFACEQLQHLKQIMKPLMELSATLDTVV--PNSSELFKAGSA---DHLRESNENNQPPPPSAAAQGDNMLDLDLRNIYSNRQDFTATFR--PTAGMTFN------------GGVGPNGLTKKQEMLVRSAIKYWVERHKHIVRLVTAVGDAYGFALLLHMLTTTITLTLLAYQATKVSGINVYAASTIGYVLYTFGQVFLFCIFGNRLIEESTSVMEAAYSCHWYDGSEEAKTFVQIVCQQCQKAMSISGAKFFTVSLDLFASVLGAVVTYFMVLVQLK

>XP_018347365.1_PREDICTED:_odorant_receptor_coreceptor_[Trachymyrmex_septentrionalis]

------MMKMKQQGLVADLMPNIRVMKMFGHFVFNYYDDNS---SKYLHKIFCCANLFMLLLQFGLCAVNLIIESADVDDLTANTITLLFFTHSIVKVIYFAIRSKYFYRTWAIWNNPNSHPLFAESNARYHAIALKKMRLLLFLVGATTVLTAVAWTTLTFFEHPIRKLVDPI-TNETTIIQLPQLLVRSFYPFDASRGITHILILIYQFYWVFFMLVNANSLDVLFCSWLLFACEQLQHLKQIMKPLMELSATLDTVV--PNSSELFKAGSA---DHIRESNENNQPPPPSAAAQGDNMLDLDLRNIYSNRQDFTATFR--PTAGMTFN------------GGVGPNGLTKKQEMLVRSAIKYWVERHKHIVRLVTAVGDAYGFALLLHMLTTTITLTLLAYQATKVSGINVYAASTIGYVLYTFGQVFLFCIFGNRLIEESTSVMEAAYSCHWYDGSEEAKTFVQIVCQQCQKAMSISGAKFFTVSLDLFASVLGAVVTYFMVLVQLK

>XP_018307006.1_PREDICTED:_odorant_receptor_coreceptor_[Trachymyrmex_zeteki]

------MMKMKQQGLVADLMPNIRIMKIFGHFVFNYYDDNS---SKYLHKVFCCANLFLLLLQFGLCAVNLIIESADVDDLTANTITLLFFTHSIVKVVYFAIRSKYFYRTWAIWNNPNSHPLFAESNARYHAIALKKMRLLLFLVGATTVLAAVAWTTLTFFEHPIRKLVDPI-TNETTIIELPQLLVRSFYPFDASRGITHILMLIYQFYWVLFMLVNANSLDVLFCSWLLFACEQLQHLKQIMKPLMELSATLDTVV--PNSSELFKAGSA---DHLRESNENTQLPPPSAAAQGDNMLDLDLRNIYSNRQDFTATFR--PTAGMTFN------------GGVGPNGLTKKQEMLVRSAIKYWVERHKHIVRLVTAVGDAYGFALLLHMLTTTITLTLLAYQATKVSGINVYAASTIGYVLYTFGQVFLFCIFGNRLIEESTSVMEAAYSCHWYDGSEEAKTFVQIVCQQCQKAMSISGAKFFTVSLDLFASVLGAVVTYFMVLVQLK

>XP_012061929.1_PREDICTED:_odorant_receptor_coreceptor_[Atta_cephalotes]

------MMKMKQQGLVADLMPNIRVMKMFGHFVFNYYDDNS---SKYLHKIFCCANLFLLLLQFGLCAVNLIIESADVDDLTANTITLLFFTHSIVKIVYFAIRSKYFYRTWAIWNNPNTHPLFAESNARYHAIALKKMRLLLFLVGATTVLTAVAWTTLTFFEHPIRKLVDPI-TNETTIIQLPQLLVRSFYPFDASKGITHILMLIYQFYWVFFMLVNANSLDVLFCSWLLFACEQLQHLKQIMKPLMELSATLDTVV--PNSSELFKAGSA---DHLRESNENNQPPPPSAAAQGDNMLDLDLRNIYSNRQDFTATFR--PTAGMTFN------------GGVGPNGLTKKQEMLVRSAIKYWVERHKHIVRLVTAVGDAYGFALLLHMLTTTITLTLLAYQATKVNGINVYAASTIGYVLYTFGQVFLFCIFGNRLIEESTSVMEAAYSCHWYDGSEEAKTFVQIVCQQCQKAMSISGAKFFTVSLDLFASVLGAVVTYFMVLVQLK

>XP_018359353.1_PREDICTED:_odorant_receptor_coreceptor_[Trachymyrmex_cornetzi]

------MMKMKQQGLVADLMPNIRVMKMFGHFVFNYYDDNS---SKYLHKIFCCANLFLLLLQFGLCAVNLIIESADVDDLTANTITLLFFTHSIVKVVYFAIRSKYFYRTWAIWNNPNSHPLFAESNARYHAIALKKMRLLLFLVGATTVLAAVAWTTLTFFEHPIRKLVDPI-TNETTIIQLPQLLVRSFYPFDASKGVTHILMLIYQFYWVFFMLVNANSLDVLFCSWLLFACEQLQHLKQIMKPLMELSATLDTVV--PNSSELFKAGSA---DHLRESSENNQPPPPSAAAQGDNMLDLDLRNIYSNRQDFTATFR--PTAGMTFN------------GGVGPNGLTKKQEMLVRSAIKYWVERHKHIVRLVTAVGDAYGFALLLHMLTTTITLTLLAYQATKVSGINVYAASTIGYVLYTFGQVFLFCIFGNRLIEESTSVMEAAYSCHWYDGSEEAKTFVQIVCQQCQKAMSISGAKFFTVSLDLFASVLGAVVTYFMVLVQLK

>XP_017848171.1_PREDICTED:_odorant_receptor_coreceptor_[Drosophila_busckii]

--MSTSMQPSKYTGLVADLMPNIRAMKYSGLFMHNFT-----GGSSLTKKIYSSIHLVVIVMQLGFILVNMALNAEEVNELSGNTITTLFFTHCIIKFVYLAINQKNFYRTLNIWNQANSHPLFAESDARYHAIALAKMRKLFFLVMLTTFASATAWTTITFFGESVKFAIDHD-TNATITVEIPRLPIKAFYPWDAGKGLFYIISFALQVYYVLFSMVHSNLCDVMFCSWLIFACEQLQHLKGIMKPLMELSASLDTY--RPNSAALFRSLSA---NSKSELIHNEEKDPG--------T-DMDITGIYSTKADWGAQFR-APTTLQSFNSNG---NGN---AMGNPNGLTKKQEMMVRSAIKYWVERHKHVVRLVAGIGDTYGAALLLHMLVSTIMLTLLAYQATKINGVNVYAFTVVGYLGYALAQVFHFCIFGNRLIEESSSVMEAAYSCHWYDGSEEAKTFVQIVCQQCQKAMSISGAKFFTVSLDLFASVLGAVVTYFMVLVQLK

>XP_001994023.1_GH22610_[Drosophila_grimshawi]

--MSTSMQPSKYTGLVADLMPNILTMRYTGLFMHNFT-----GGSVFMKRIFSSVHLVIIVLQFCFILVNMALNAEEVNELSGNTITTLFFTHCIVKFVYLAINQKNFYRTLNIWNQANSHSLFAESDARYHSISLAKMRKLFFLVMLTTVASATAWITITFFGESVKFAMDKE-TNSTITVPIPRLPIKAFYPWDSSHGMFYMISFVLQIYYVLFSMMHSNLCDVMFCSWLIFACEQLQHLKGIMKPLMELSASLDTY--RPNSAALFRSLSA---NSKSELIHNEEKE----------V-DMDMTGIYSTKADWGAQFR-APSTLQSFNPAANG-NG----MASNPNGLSKKQEMMVRSAIKYWVERHKHVVRLVAAIGDTYGAALLLHMLVSTIMLTLLAYQATKINGVNVYAFTVIGYLGYALAQVFHFCIFGNRLIEESSSVMEAAYSCHWYDGSEEAKTFVQIVCQQCQKAMSISGAKFFTVSLDLFASVLGAVVTYFMVLVQLK

>XP_017009993.1_PREDICTED:_odorant_receptor_coreceptor_[Drosophila_takahashii]

--MTTSMQPSKYTGLVADLMPNIRAMKYSGLFMHNFT-----GGSAFMKKVYSSVHLVLLLMQFAFILVNMALNAEEVNELSGNTITTLFFTHCMTKFIYLAVNQKNFYRTLNIWNQVNTHPLFAESDARYHSIALAKMRKLFFLVMLTTVASATAWTTITFFGDSVKMVVDHE-TNSSIPVEIPRLPIKSFYPWNASHGLFYMISFAFQIYYVLFSMIHSNLCDVMFCSWLIFACEQLQHLKGIMKPLMELSASLDTY--RPNSAALFRSLSA---NSKSELIHNEEKDPA--------T-DMDMSGVYSSKADWGAQFR-APSTLQSFGGNGGGGNG--VVNGANPNGLTKKQEMMVRSAIKYWVERHKHVVRLVAAIGDTYGAALLLHMLTSTIKLTLLAYQATKINGVNVYAFTVVGYLGYALAQVFHFCIFGNRLIEESSSVMEAAYSCHWYDGSEEAKTFVQIVCQQCQKAMSISGAKFFTVSLDLFASVLGAVVTYFMVLVQLK

>XP_017114692.1_PREDICTED:_odorant_receptor_coreceptor_[Drosophila_elegans]

--MTTSMQPSKYTGLVADLMPNIRAMKYSGLFMHNFT-----GGSAFMKKVYSSVHLVLLLMQFAFILVNMALNAEEVNELSGNTITTLFFTHSITKFIYLAVNQKNFYRTLNIWNQVNTHPLFAESDARYHSIALAKMRKLFFLVMLTTVISATAWTTITFFGDSVKMVVDHE-TNSSIPVEIPRLPIKSFYPWNASHGLFYMISFAFQIYYVLFSMIHSNLCDVMFCSWLIFACEQLQHLKGIMKPLMELSASLDTY--RPNSAALFRSLSA---NSKSELIHNEEKDPT--------T-DMDMSGVYSSKADWGAQFR-APSTLQSFGGNGGGGNG--LVNGANPNGLTKKQEMMVRSAIKYWVERHKHVVRLVAAIGDTYGAALLLHMLTSTIKLTLLAYQATKINGVNVYAFTVVGYLGYALAQVFHFCIFGNRLIEESSSVMEAAYSCHWYDGSEEAKTFVQIVCQQCQKAMSISGAKFFTVSLDLFASVLGAVVTYFMVLVQLK

>XP_017082279.1_PREDICTED:_odorant_receptor_coreceptor_[Drosophila_eugracilis]

--MTTSMQPSKYTGLVADLMPNIRAMKYSGLFMHNFT-----GGSAFMKKVYSSVHLVFLLLQFAFILVNMALNAEEVNELSGNTITTLFFTHCITKFIYLAVNQKNFYRTLNIWNQVNTHPLFAESDARYHSIALAKMRKLFFLVMLTTVLSATAWTTITFFGDSVKMVVDHE-TNSSIPVEIPRLPIKSFYPWNASHGLFYMISFAFQIYYVLFSMVHSNLCDVMFCSWLIFACEQLQHLKGIMKPLMELSATLDTY--RPNSAALFRSLSA---NSKSELIHNEEKDPT--------T-DMDMSGVYSSKADWGAQFR-APSTLQSFGGNGGGGNG--LVNGANPNGLTKKQEMMVRSAIKYWVERHKHVVRLVAAIGDTYGAALLLHMLTSTIKLTLLAYQATKINGVNVYAFTVVGYLGYALAQVFHFCIFGNRLIEESSSVMEAAYSCHWYDGSEEAKTFVQIVCQQCQKAMSISGAKFFTVSLDLFASVLGAVVTYFMVLVQLK

>XP_016982086.1_PREDICTED:_odorant_receptor_coreceptor_[Drosophila_rhopaloa]_

--MTTSMQPNKYTGLVADLMPNIRAMKYSGLFMHNFT-----GGSAFMKKVYSSVHLVFLLMQFAFILVNMALNAEEVNELSGNTITTLFFTHCITKFIYLAVNQKNFYRTLNIWNQVNTHPLFAESDARYHSIALAKMRKLFFLVMLTTVASATAWTTITFFGDSVKMVVDHE-TNSSIPVEIPRLPIKSFYPWNASHGLFYMISFAFQIYYVLFSMIHSNLCDVMFCSWLIFACEQLQHLKGIMKPLMELSASLDTY--RPNSAALFRSLSA---NSKSELIHNEEKDPA--------T-DMDMSGVYSSKADWGAQFR-APSTLQSFGGNGGGGNG--MVNGANPNGLTKKQEMMVRSAIKYWVERHKHVVRLVAAIGDTYGAALLLHMLTSTIKLTLLAYQATKINGVNVYAFTVVGYLGYALAQVFHFCIFGNRLIEESSSVMEAAYSCHWYDGSEEAKTFVQIVCQQCQKAMSISGAKFFTVSLDLFASVLGAVVTYFMVLVQLK

>XP_002096053.1_uncharacterized_protein_Dyak_GE25284,_isoform_A_[Drosophila_yakuba]

--MTTSMQPSKYTGLVADLMPNIRAMKYSGLFMHNFT-----GGSAFMKKVYSSAHLVFLLMQFTFILVNMALNAEEVNELSGNTITTLFFTHCITKFIYLAVNQKNFYRTLNIWNQVNTHPLFAESDARYHSIALAKMRKLFFLVMLTTVASATAWTTITFFGDSVKMVVDHE-TNSSIPVEIPRLPIKSFYPWNASHGMFYMISFVFQIYYVLFSMIHSNLCDVMFCSWLIFACEQLQHLKGIMKPLMELSASLDTY--RPNSAALFRSLSA---NSKSELIHNEEKDPA--------T-DMDMSGIYSSKADWGAQFR-APSTLQSFGGNGGGGNG--MVNGANPNGLTKKQEMMVRSAIKYWVERHKHVVRLVAAIGDTYGAALLLHMLTSTIKLTLLAYQATKINGVNVYAFTVVGYLGYALAQVFHFCIFGNRLIEESSSVMEAAYSCHWYDGSEEAKTFVQIVCQQCQKAMSISGAKFFTVSLDLFASVLGAVVTYFMVLVQLK

>NP_001315530.1_odorant_receptor_coreceptor_[Drosophila_suzukii]

--MTTSMQPSKYTGLVADLMPNIRAMKYSGLFMHNFT-----GGSAFMKKVYSSVHLVLLLMQFAFILVNMALNAEEVNELSGNTITTLFFTHCITKFIYLAVNQKNFYRTLNIWNQVNTHPLFAESDARYHSIALAKMRKLFFLVMLTTVASATAWTTITFFGDSVKMVVDHE-TNSSIPVEIPRLPIKSFYPWNASHGLFYMISFAFQIYYVLFSMIHSNLCDVMFCSWLIFACEQLQHLKGIMKPLMELSASLDTC--RPNSAALFRSLSA---NSKSELIHNEEKDPA--------T-DMDMTGVYSSKADWGAQFR-APSTLQSFGGNGGGGNG--LVNGANPNGLTKKQEMMVRSAIKYWVERHKHVVRLVAAIGDTYGAALLLHMLTSTVKLTLLAYQATKINGVNVYAFTVVGYLGYALAQVFHFCIFGNRLIEESSSVMEAAYSCHWYDGSEEAKTFVQIVCQQCQKAMSISGAKFFTVSLDLFASVLGAVVTYFMVLVQLK

>XP_001978924.1_uncharacterized_protein_Dere_GG11032,_isoform_A_[Drosophila_erecta]_

--MTTSMQPSKYTGLVADLMPNIRAMKYSGLFMHNFT-----GGSGFMKKVYSSAHLVFLLMQFTFILVNMALNAEEVNELSGNTITTLFFTHCITKFIYLAVNQKNFYRTLNIWNQVNTHPLFAESDARYHSIALAKMRKLFFLVMLTTVASATAWTTITFFGDSVKMVVDHE-TNSSIPVEIPRLPIKSFYPWNASHGMFYMISFAFQIYYVLFSMIHSNLCDVMFCSWLIFACEQLQHLKGIMKPLMELSASLDTY--RPNSAALFRSLSA---NSKSELIHNEEKDPA--------T-DMDMSGIYSSKADWGAQFR-APSTLQSFGGNGGGGNG--MVNGANPNGLTKKQEMMVRSAIKYWVERHKHVVRLVAAIGDTYGAALLLHMLTSTIKLTLLAYQATKINGVNVYAFTVVGYLGYALAQVFHFCIFGNRLIEESSSVMEAAYSCHWYDGSEEAKTFVQIVCQQCQKAMSISGAKFFTVSLDLFASVLGAVVTYFMVLVQLK

>XP_002038406.1_GM10637_[Drosophila_sechellia]

--MTTPMQPSKYTGLVADLMPNIRAMKYSGLFMHNFT-----GGSAFMKKVYSSVHLVFLLMQFTFILVNMALNAEEVNELSGNTITTLFFTHCITKFIYLGCQPEEFLQKLGYMEPGEHASMFAESDARYHSIALAKMRKLFFLVMLTTVASATAWTTITFFGDSVKMVVDHE-TNSSIPVEIPRLPIKSFYPWNASHGMFYMISFAFQIYYVLFSMIHSNLCDVMFCSWLIFACEQLQHLKGIMKPLMELSASLDTY--RPNSAALFRSLSA---NSKSELIHNEEKDPA--------T-DMDMSGIYSSKADWGAQFR-APSTLQSFGGNGGGGNG--MVNGANPNGLTKKQEMMVRSAIKYWVERHKHVVRLVAAIGDTYGAALLLHMLTSTIMLTLLAYQATKINGVNVYALTVVGYLGYALAQVFHFCIFGNRLIEESSSVMEAAYSCHWYDGSEEAKTFVQIVCQQCQKAMSISGAKFFTVSLDLFASVLGAVVTYFMVLVQLK

>XP_016033307.1_uncharacterized_protein_Dsimw501_GD19619_[Drosophila_simulans]

--MTTSMQPSKYTGLVADLMPNIRAMKYSGLFMHNFT-----GGSAFMKKVYSSVHLVFLLMQFTFILVNMALNAEEVNELSGNTITTLFFTHCITKFIYLAVNQKNFYRTLNIWNQVNTHPLFAESDARYHSIALAKMRKLFFLVMLTTVASATAWTTITFFGDSVKMVVDHE-MNSSIPVEIPRLPIKSFYPWNASHGMFYMISFAFQIYYVLFSMIHSNLCDVMFCSWLIFACEQLQHLKGIMKPLMELSASLDTY--RPNSAALFRSLSA---NSKSELIHNEEKDPA--------T-DMDMSGIYSSKADWGAQFR-APSTLQSFGGNGGGGNG--MVNGANPNGLTKKQEMMVRSAIKYWVERHKHVVRLVAAIGDTYGAALLLHMLTSTIKLTLLAYQATKINGVNVYAFTVVGYLGYALAQVFHFCIFGNRLIEESSSVMEAAYSCHWYDGSEEAKTFVQIVCQQCQKAMSISGAKFFTVSLDLFASVLGAVVTYFMVLVQLK

>XP_016948956.1_PREDICTED:_odorant_receptor_coreceptor_[Drosophila_biarmipes]

--MTTSMQPSKYTGLVADLMPNIRAMKYSGLFMHNFT-----GGSAFMKKVYSSVHLVFLLMQFAFILVNMALNAEEVNELSGNTITTLFFTHCMTKFIYLAVNQKNFYRTLNIWNQVNTHPLFAESDARYHSIALAKMRKLFFLVMLTTVASATAWTTITFFGDSVKMVVDHE-TNSSIPVEIPRLPIKSFYPWNASHGLFYMISFAFQIYYVLFSMIHSNLCDVMFCSWLIFACEQLQHLKGIMKPLMELSASLDTY--RPNSAALFRSLSA---NSKSELIHNEEKDPA--------A-DMDMSGVYSSKADWGAQFR-APSTLQSFGGNGGGGIG--VVNGANPNGLTKKQEMMVRSAIKYWVERHKHVVRLVAAIGDTYGAALLLHMLTSTIKLTLLAYQATKINGVNVYAFTVVGYLGYALAQVFHFCIFGNRLIEESSSVMEAAYSCHWYDGSEEAKTFVQIVCQQCQKAMSISGAKFFTVSLDLFASVLGAVVTYFMVLVQLK

>XP_017098675.1_PREDICTED:_odorant_receptor_coreceptor_[Drosophila_bipectinata]

--MTTSMQPSKYTGLVADLMPNIKAMKYSGLFMHNFT-----GGSAFVKKVYSSVHLVLLLLQFVFILVNMALNAEEVNELSGNTITTLFFTHCITKFIYLAVNQKNFYRTLNIWNQVNTHPLFAESDARYHSIALAKMRKLFFLVMLTTVASATAWTTITFFGDSVKMVVDHE-TNSSVPVEIPRLPIKSFYPWNAGHGIFYMISFAFQIYYILFSMIHSNLCDVMFCSWLIFACEQLQHLKGIMKPLMELSASLDTY--RPNSAALFRSLSA---NSKSELIHNEEKDPG--------A-DMDMSGVYSSKADWGAQFR-APSTLQSFGGNGG------IVNGANPNGLTKKQEMMVRSAIKYWVERHKHVVRLVAAIGDTYGAALLLHMLTSTIKLTLLAYQATKITGVNVYAFTVIGYLGYALAQVFHFCIFGNRLIEESSSVMEAAYSCHWYDGSEEAKTFVQIVCQQCQKAMSISGAKFFTVSLDLFASVLGAVVTYFMVLVQLK

>XP_001953343.1_uncharacterized_protein_Dana_GF17712_[Drosophila_ananassae]

--MTTSMQPSKYTGLVADLMPNIKAMKYSGLFMHNFT-----GGSAFVKKVYSSVHLVLLLLQFVFILVNMALNAEEVNELSGNTITTLFFTHCMTKFIYLAVNQKNFYRTLNIWNQVNTHPLFAESDARYHSIALAKMRKLFFLVMLTTVASATAWTTITFFGDSVKMVVDHE-TNSSVPVEIPRLPIKSFYPWNAGHGIFYMISFAFQVYYIIFSMIHSNLCDVMFCSWLIFACEQLQHLKGIMKPLMELSASLDTY--RPNSAALFRSLSA---NSKSELIHNEEKDPG--------A-DMDMSGVYSSKADWGAQFR-APSTLQSFGGNGG------VVNGANPNGLTKKQEMMVRSAIKYWVERHKHVVRLVAAIGDTYGAALLLHMLTSTVKLTLLAYQATKITGVNVYAFTVIGYLGYALAQVFHFCIFGNRLIEESSSVMEAAYSCHWYDGSEEAKTFVQIVCQQCQKAMSISGAKFFTVSLDLFASVLGAVVTYFMVLVQLK

>XP_002017182.1_GL22168_[Drosophila_persimilis]

------MQPSKYTGLVADLMPNIRAMKYSGLFMHNFT-----GGSGFMKKVYSSIHLVMLLMQFIFILVNMALNAEEVNELSGNTITALFFTHCITKFIYLAVNQKNFYRTLNIWNQVNSHPLFAESDARYHSIALAKMRKLFFLVMLTTILSATAWTTITFFGDSVKMVVDHE-TNSSMAVEIPRLPIKSFYPWNAFHGMFYMISFAFQVYYVLFSMIHSNLCDVMFCSWLIFACEQLQHLKGIMKPLMELSASLDTY--RPNSAALFRSLSA---QSKSELIRNEEKDPG--------N-DLDMSGVYSSKADWGAQFR-APTTLQSFGNGNGNGNGGGAANGANPNGLTKKQEMMVRSAIKYWVERHKHVVRLVAAIGDTYGAALLLHMLTSTIKLTLLAYQATKITGVNVYAFTVVGYLGYALAQVFHFCIFGNRLIEESSSVMEAAYSCHWYDGSEEAKTFVQIVCQQCQKAMSISGAKFFTVSLDLFASVLGAVVTYFMVLVQLK

>XP_017023291.1_PREDICTED:_odorant_receptor_coreceptor_[Drosophila_kikkawai]

--MTTSMQPSKYTGLVADLMPNIRAMKYSGLFMHNFT-----GGSAFVKKVYSSVHLVLLLIQFAFILVNMAMNAEEVNELSGNTITTLFFTHCITKFIYLAVNQKNFYRTLNIWNQVNTHPLFAESDARYHSIALAKMRKLFFLVMLTTVASATAWTTITFFGDSVKMVVDHE-TNSSIPVEIPRLPIKSFYPWNASHGMFYVISFAFQIYYVLFSMIHSNLCDVMFCSWLIFACEQLQHLKGIMKPLMELSASLDTY--RPNSAALFRSLSA---NSKSELIRNEEKDPG--------A-DMDMSGIYSSKADWGAQFR-APSTLQNFGGNGGGGNGV-GVNGANPNGLTKKQEMMVRSAIKYWVERHKHVVRLVAAIGDTYGAALLLHMLTSTIKLTLLAYQATKIGGVNVYAFTVVGYLGYALAQVFHFCIFGNRLIEESSSVMEAAYSCHWYDGSEEAKTFVQIVCQQCQKAMSISGAKFFTVSLDLFASVLGAVVTYFMVLVQLK

>XP_017145273.1_PREDICTED:_odorant_receptor_coreceptor_[Drosophila_miranda]

--MTTTMQPSKYTGLVADLMPNIRAMKYSGLFMHNFT-----GGSGFMKKVYSSIHLVLLLMQFIFILVNMALNAEEVNELSGNTITALFFTHCITKFIYLAVNQKNFYRTLNIWNQVNSHPLFAESDARYHSIALAKMRKLFFLVMLTTILSATAWTTITFFGDSVKMVVDHE-TNSSMAVEIPRLPIKSFYPWNAFHGMFYMISFAFQVYYVLFSMIHSNLCDVMFCSWLIFACEQLQHLKGIMKPLMELSASLDTY--RPNSAALFRSLSA---QSKSELIRNEEKDPG--------N-DLDMSGVYSSKADWGAQFR-APTTLQSFGNGNGNGNGGGAANGANPNGLTKKQEMMVRSAIKYWVERHKHVVRLVAAIGDTYGAALLLHMLTSTIKLTLLAYQATKITGVNVYAFTVVGYLGYALAQVFHFCIFGNRLIEESSSVMEAAYSCHWYDGSEEAKTFVQIVCQQCQKAMSISGAKFFTVSLDLFASVLGAVVTYFMVLVQLK

>XP_002072443.2_uncharacterized_protein_Dwil_GK22840_[Drosophila_willistoni]

--MTTPMQPSKYTGLVADLMPNIRAMKYSGLFMHNFT-----GGSGFMKKIYSSVHLVFLFIQFAFILVNMALNAEEVNELSGNTITTLFFTHCITKFIYLAVNQKNFYRTLNIWNQVNTHPLFAESDARYHSIALAKMRKLFFLVMLTTFASATAWTTITFFGDSVKMIVDKE-TNSSIAVEIPRLPIKSFYPWDASKGMFYTISFAFQIYYVLFSMIHSNLCDVMFCSWLIFACEQLQHLKGIMKPLMELSASLDTY--RPNSAALFRSLSA---NSKSELIRNEEKDPG--------A-DMDMTGIYSTKADWGAQFR-APSTLQSFNGGSNGGGGNGV-NGANPNGLTKKQEMMVRSAIKYWVERHKHVVRLVAAIGDTYGAALLLHMLTSTIKLTLLAYQATKITGVNVYAFTVIGYLGYALAQVFHFCIFGNRLIEESSSVMEAAYSCHWYDGSEEAKTFVQIVCQQCQKAMSISGAKFFTVSLDLFASVLGAVVTYFMVLVQLK

>XP_001359364.3_odorant_receptor_co-receptor_[Drosophila_pseudoobscura_pseudoobscura]

--MTTTMQPSKYTGLVADLMPNIRAMKYSGLFMHNFT-----GGSGFMKKVYSSIHLVMLLMQFIFILVNMALNAEEVNELSGNTITALFFTHCITKFIYLAVNQKNFYRTLNIWNQVNSHPLFAESDARYHSIALAKMRKLFFLVMLTTILSATAWTTITFFGDSVKMVVDHE-TNSSMAVEIPRLPIKSFYPWNAFHGMFYMISFAFQVYYVLFSMIHSNLCDVMFCSWLIFACEQLQHLKGIMKPLMELSASLDTY--RPNSAALFRSLSA---QSKSELIRNEEKDPG--------N-DLDMSGVYSSKADWGAQFR-APTTLQSFGNGNGNGNGGGAANGANPNGLTKKQEMMVRSAIKYWVERHKHVVRLVAAIGDTYGAALLLHMLTSTIKLTLLAYQATKITGVNVYAFTVVGYLGYALAQVFHFCIFGNRLIEESSSVMEAAYSCHWYDGSEEAKTFVQIVCQQCQKAMSISGAKFFTVSLDLFASVLGAVVTYFMVLVQLK

>XP_001998414.1_uncharacterized_protein_Dmoj_GI23643_[Drosophila_mojavensis]

--MATSMQPGKYTGLVADLMPNIRAMRYSGLFMHNFT-----GGSIFFKRVYSSVHLVLIVMHIGFILVNLALNAEEVNELSGNTITTLFFTHCIVKFVYLAVNQKNFYRTLNIWNQANTHPLFAESDARYHSIALAKMRKLFFLVMLTTFASATAWTTITFFGDSVKFAVDKE-TNSSITVEIPRLPIKAFYPWDASHGMFYMISFVLQVYYVLFSMVHSNLCDVMFCSWLIFACEQLQHLKGIMKPLMELSASLDTY--RPNSAALFRSLSA---NSKSELIHNEEKEPV--------T-DMDMTGIYSSKADWGAQFR-APSTLQSFNAG-GNGNGNG--MANNPNGLTKKQEMMVRSAIKYWVERHKHVVRLVAAIGDTYGAALLLHMLVSTIMLTLLAYQATKINGVNVYAFTVVGYLGYALAQVFHFCIFGNRLIEESSSVMEAAYSCHWYDGSEEAKTFVQIVCQQCQKAMSISGAKFFTVSLDLFASVLGAVVTYFMVLVQLK

>XP_002056756.2_uncharacterized_protein_Dvir_GJ24719_[Drosophila_virilis]

--MATTMQPSKYTGLVADLMPNIRAMRYSGLFMHNFT-----GGSTFMKRIYSSVHMVVLVMQIGFILANLALNADEVNELCGNTITTLFFTHCIVKFVYLAVNQKNFYRTLNIWNQANTHPLFAESDARYHSIALAKMRKLFFLVMLTTFASATAWTTITFFGDSVKLALDKE-TNSSITVEIPRLPIKAFYPWDASHGMFYMISFALQVYYVLFSMVHSNLCDVMFCSWLIFACEQLQHLKGIMKPLMELSASLDTY--RPNSAALFRSLSA---NSKSELIHNEEKEPV--------T-DLDMTGIYSTKADWGAQFR-APSTLQSFNAG-ANGNGN---MANNPNGLTKKQEMMVRSAIKYWVERHKHVVRLVAAIGDTYGAALLLHMLVSTIMLTLLAYQATKINGVNVYAFTVIGYLGYALAQVFHFCIFGNRLIEESSSVMEAAYSCHWYDGSEEAKTFVQIVCQQCQKAMSISGAKFFTVSLDLFASVLGAVVTYFMVLVQLK

>XP_017044295.1_PREDICTED:_odorant_receptor_coreceptor_[Drosophila_ficusphila]

--MTTSMQPSKYTGLVADLMPNIRAMKYSGLFMHNFT-----GGSAFMKKVYSSAHLVFLLMQFAFILVNMALNAEEVNELSGNTITTLFFTHCITKFIYLAVNQKNFYRTLNIWNQVNSHPLFAESDARYHSIALAKMRKLFFLVMLTTVASATAWTTITFFGDSVKMVVDHE-TNSSIPVEIPRLPIKSFYPWNAGHGIFYMISFAFQIYYVLFSMIHSNLCDVMFCSWLIFACEQLQHLKGIMKPLMELSASLDTY--RPNSAALFRSLSA---NSKSELIHNEEKDPA--------T-DMDMSGVYSSKADWGAQFR-APSTLQSFGGNGNGNGGNGVANGANPNGLTKKQEMMVRSAIKYWVERHKHVVRLVAAIGDTYGAALLLHMLTSTIKLTLLAYQATKINGVNVYAFTVVGYLGYALAQVFHFCIFGNRLIEESSSVMEAAYSCHWYDGSEEAKTFVQIVCQQCQKAMSISGAKFFTVSLDLFASVLGAVVTYFMVLVQLK

>ACC86853.1_odorant_receptor_Or83b_[Bactrocera_dorsalis]

------MQPSKYVGLVADLMPNIRLMKYSGLFMHNFT-----GGSGLFKKIYSSVHLVLVLVQFLLILVNLALNAEEVNELSGNTITVLFFTHSITKFIYLAVSQKNFYRTLNIWNQVNSHPLFAESDARYHAIALAKMRKLFTLVMLTTVASAVAWTTITFFGESVKFAFEKE-TNSTITVEIPRLPIKSFYPWNAGAGMFYIISFAFQCYYLLFSMVHANLCDVLFCSWLIFACEQLQHLKGIMKPLMELSASLDTY--RPNSAALFRSLSA---NSKSELINNEEKEP---------T-DLDISGVYSSKADWGAQFR-APSTLQTF-------NG---MNGTNPNGLTRKQEMMVRSAIKYWVERHKHVVRLVAAIGDTYGGALLLHMLTSTIMLTLLAYQATKITGVNAYAFTTIGYLGYALAQVFHFCIFGNRLIEESSSVMEAAYSCHWYDGSEEAKTFVQIVCQQCQKAMSISGAKFFTVSLDLFASVLGAVVTYFMVLVQLK

>CAD88246.1_putative_chemosensory_receptor_2,_partial_[Calliphora_vicina]

---------------------------------------------------------------------------------------------------------------------------------------------------------------------------------------------------------------------------------------------QLQHLKGIMKPLMELSASLDTY--CPNSAALFRSLSA---NSKSELIINEEKEPP--------N-DLDMTGIYSTKADWGAQFR-APTTLQTF-------NG---VNGGNPNGLTKKQEMMVRSAIKYWVERHKHVVRLVAAIGDTYGAALLLHMLTSTIKLTLLAYQATKITGVNVYAFTVIGYLGYALAQVFHFCIFGNRLIEESSSVMEAAYSCHWYDGSEEAKTFVQIV-----------------------------------------

>AFH96944.1_odorant_receptor_co-receptor_[Musca_domestica]

--MQANLQPTKYTGLVADLMPNIKLMKYSGLFMHAFT-----GGSALLKNVYSSIHLVLIVLQFIFILVNMALNADEVNELSGNTITALFFTHCITKFVYLAVNQKNFYRTLNIWNQPNSHPLFAESDARYHSIALAKMRKLFFLVMLTTVASAVAWITITFFGESVKFATDKE-TNSTITVPIPRLPIKSFYPWDASSGMFYMISFGYQAYYLLFSMVHSNLCDVLFCSWLIFACEQLQHLKGIMKPLMELSASLDTY--RPNSAALFRSLSA---NSKSELIQNEEKEPV--------N-DLDMSGIYSTKADWGAQFR-APSTLQTF-------NG---INGGNPNGLTKKQEMMVRSAIKYWVERHKHVVRLVAAIGDTYGAALLLHMLTSTIKLTLLAYQATKITGVNVYAFTVIGYLGYALAQVFHFCIFGNRLIEESSSVMEAAYSCHWYDGSEEAKTFVQIVCQQCQKAMSISGAKFFTVSLDLFASVLGAVVTYFMVLVQLK

>ADK97803.1_odorant_receptor_Or83b_[Bactrocera_cucurbitae]

------MQPSKYVGLVADLMPNIRLMKYSGLFMHNFT-----GGSGLFKKIYSSVHLVLVLVQFLLILVNLALNSEEVNELSGNTITVLFFTHSITKFIYLAVSQKNFYRTLNIWNQVNSHPLFAESDARYHAIALAKMRKLFTLVMLTTVASAVAWTTITFFGESVKFAFEKE-TNSTITVEIPRLPIKSFYPWNAGAGMFYIISFAFQCYYLLFSMVHANLCDVLFCSWLIFACEQLQHLKGIMKPLMELSASLDTY--RPNSAALFRSLSA---NSKSELINNEEKEP---------T-DLDISGVYSSKADWGAQFR-APSTLQTF-------NG---MNGTNPNGLTRKQEMMVRSAIKYWVERHKHVVRLVAAIGDTYGGALLLHMLTSTIMLTLLAYQATKITGVNVYAFTTIGYLGYALAQVFHFCIFGNRLIEESSSVMEAAYSCHWYDGSEEAKTFVQIVCQQCQKAMSISGAKFFTVSLDLFASVLGAVVTYFMVLVQLK

>XP_018789760.1_PREDICTED:_odorant_receptor_coreceptor_[Bactrocera_latifrons]

------MQPSKYVGLVADLMPNIRLMKYSGLFMHNFT-----GGSGLFKKIYSSVHLVLVLVQFLLILVNLALNAEEVNELSGNTITVLFFTHSITKFIYLAVSQKNFYRTLNIWNQVNSHPLFAESDARYHGIALAKMRKLFTLVMLTTVASAVAWTTITFFGESVKFAFEKE-TNSTITVEIPRLPIKSFYPWNAGAGMFYIISFAFQCYYLLFSMVHANLCDVLFCSWLIFACEQLQHLKGIMKPLMELSASLDTY--RPNSAALFRSLSA---NSKSELINNEEKEP---------T-DLDISGVYSSKADWGAQFR-APSTLQTF-------NG---MNGTNPNGLTRKQEMMVRSAIKYWVERHKHVVRLVAAIGDTYGGALLLHMLTSTIMLTLLAYQATKITGVNVYAFTTIGYLGYALAQVFHFCIFGNRLIEESSSVMEAAYSCHWYDGSEEAKTFVQIVCQQCQKAMSISGAKFFTVSLDLFASVLGAVVTYFMVLVQLK

>XP_017470946.1_PREDICTED:_odorant_receptor_coreceptor_isoform_X1_[Rhagoletis_zephyria]

------MQPSKYVGLVADLMPNIRLMKYSGLFMHNFT-----GGSGLFKKIYSSMHLVLVVVQFLLILVNMALNTDEVNELSGNTITVLFFTHCITKFVYLAVSQKHFYRTLNIWNQVNSHPLFAESDARYHAIALAKMRKLFTLVMLTTVASAVAWTTITFFGESVKFAFDKE-TNSSITVEIPRLPIKSFYPWNAMSGMFYIISFAYQFYYLLFSMVHSNLCDVLFCSWLIFACEQLQHLKGIMKPLMELSASLDTY--RPNSAALFRSLSA---NSKSELINNEEKEP---------T-DLDISGVYSSKADWGAQFR-APSTLQTF-------NG---VNGTNPNGLTRKQEMMVRSAIKYWVERHKHVVRLVAAIGDTYGGALLLHMLTSTIMLTLLAYQATKITGVNVYAFTVIGYLGYALAQVFHFCIFGNRLIEESSSVMEAAYSCHWYDGSEEAKTFVQIVCQQCQKAMSISGAKFFTVSLDLFASVLGAVVTYFMVLVQLK

>NP_001298174.1_odorant_receptor_coreceptor_[Stomoxys_calcitrans]_

--MQANLQPTKYVGLVADLMPNIRLMKYSGLFMHAFT-----GGSALLKNIYSSIHLVLILVQFALILVNMALNADEVNELSGNTITALFFTHSITKFIYLAVNQKNFYRTLNIWNQPNSHPLFAESDARYHSIALAKMRKLFFLVMLTTVASAVAWITITFFGESVKFANDKE-TNSTITVPIPRLPIKSFYPWDASQGMFYMISFGYQVYYLLFSMVHSNLCDVLFCSWLIFACEQLQHLKGIMKPLMELSASLDTY--RPNSAALFRSLSA---NSKSELINNEEKEPV--------N-DLDMSGIYNTKADWGAQFR-APSTLQTF-------NG---INGTNPNGLTKKQEMMVRSAIKYWVERHKHVVRLVAAIGDTYGAALLLHMLTSTIKLTLLAYQATKITGVNVYAFTVIGYLGYALAQVFHFCIFGNRLIEESSSVMEAAYSCHWYDGSEEAKTFVQIVCQQCQKAMSISGAKFFTVSLDLFASVLGAVVTYFMVLVQLK

>NP_001266301.1_odorant_receptor_coreceptor_[Ceratitis_capitata]

------MQPSKYVGLVADLMPNIRLMKYSGLFMHNFT-----GGSGLFKKIYSSMHLVLVLVQFLLILVNLALNAEEVNELSGNTITVLFFTHCITKFIYLAVTQKQFYRTLNIWNQVNSHPLFAESDARYHSIALAKMRKLFTLVMLTTVVSAVAWTTITFFGESVKFAFDKD-TNSSITVEIPRLPIKSFYPWNAGSGMFYIISFAFQCYYLLFSMVHSNLCDVLFCSWLIFACEQLQHLKGIMKPLMELSASLDTY--RPNSAALFRSLSA---NSKSELINNEEKEP---------T-DLDVSGIYSSKADWGAQFR-APSTLQTF-------NG---MNGPNPNGLTRKQEMMVRSAIKYWVERHKHVVRLVAAIGDTYGGALLLHMLTSTIMLTLLAYQATKITGVNVYAFTTVGYLCYALAQVFHFCIFGNRLIEESSSVMEAAYSCHWYDGSEEAKTFVQIVCQQCQKAMSISGAKFFTVSLDLFASVLGAVVTYFMVLVQLK

>ACF21678.1_putative_odorant_receptor_Or83b_[Haematobia_irritans_irritans]

---MTSMQPTKYVGLVADLMPNIRLMKYSGLFMHAFT-----GGSALLKNVYSSIHLVLIIIQFGLILVNMALNADEVNELSGNTITALFFTHSITKFVYLAVNQKNFYRTLNIWNQPNTHPLFAESDARYHSIALAKMRKLFFCVMLTTVLSAVAWITITFFGESVKFANDKE-TNSTITVPIPRLPIKSFYPWDASHGMFYMISFGYQVYYLFFSMVHSNLCDVIFCSWLIFACEQLQHLKGIMKPLMELSASLDTY--RPNSAALFRSLSA---NSKSELINNEEKEPV--------N-DLDMSGIYNTKADWGAQFR-APSTLQTF-------NG---INGANPNGLTKKQEMLVRSAIKYWVERHKHVVRLVVAIGDTYGAALLLHMLTSTIKLTLLAYQAIKITGVDVYAFTVIGYLGYALAQVFHFCIFGNRLIEESSSVMEAAYSCHWYDGSEEAKTFVQIVCQQCQKAMWIWGAKFFTVSLDLFVSVLGAVVTYFMGLVQLK

>XP_014092453.1_PREDICTED:_odorant_receptor_coreceptor_[Bactrocera_oleae]

------MQPSKYVGLVADLMPNIRLMKYSGLFMHNFT-----GGSGFFKKIYSSIHLVLVLVQFLLILVNMALNADEVNELSGNTITVLFFTHSITKFIYLAVTQKNFYRTLNIWNQVNSHPLFAESDARYHAIALAKMRKLFTLVMLTTVASAVAWTTITFFGESVKFAFEKE-TNSTITVEIPRLPIKSFYPWNAGVGMFYIISFAFQCYYLLFSMVHSNLCDVLFCSWLIFACEQLQHLKGIMKPLMELSASLDTY--RPNSAALFRSLSA---NSKSELINNEEKEP---------T-DLDISGVYSSKADWGAQFR-APSTLQTF-------NG---MNGANPNGLTRKQEMMVRSAIKYWVERHKHVVRLVAAIGDTYGGALLLHMLTSTIMLTLLAYQATKITGVNVYAFTTIGYLGYALAQVFHFCIFGNRLIEESSSVMEAAYSCHWYDGSEEAKTFVQIVCQQCQKAVSISGAKFFTVSLDLFASVLGAVVTYFMVLVQLK

>AEA30005.2_odorant_receptor_1_[Lucilia_sericata]

--MQSNLQPTKYVGLVADLMPNIKLMKYSGLFMHAFT-----GGSPLLKKVYSSIHLVLILAQFMFILVNMALNADEVNELSGNTITALFFTHCVTKFIYLAVNQKNFYRTLNIWNQVNTHPLFAESDARYHSIALAKMRKLFFLVMLTTVASAVAWITITFFGESVKFAFDKE-TNSSITVEIPRLPIKSFYPWDASQGIFYTISFAFQGYYLLFSMVHSNLCDVLFCSWLIFACEQLQHLKGIMKPLMELSASLDTY--RPNSAALFRSLSA---NSKSELIINEEKEPP--------S-DLDMTGIYSTKADWGAQFR-APTTLQTF-------NG---VNGGNPNGLTKKQEMMVRSAIKYWVERHKHVVRLVAAIGDTYGAALLLHMLTSTIKLTLLAYQATKITGVNVYAFTVIGYLGYALAQVFHFCIFGNRLIEESSSVMEAAYSCHWYDGSEEAKTFVQIVCQQCQKAMSISGAKFFTVSLDLFASVLGAVVTYFMVLVQLK

>ADN88092.1_odorant_receptor,_partial_[Aldrichina_grahami]

----------------------IKLMKYSGLFMHAFT-----GGSPLLKKVYSSIHLVLILAQFVFILVNMALNADEVNELSGNTITALFFTHCVTKFIYLAVNQKNFYRTLNIWNQVNSHPLFAESDARYHSIALAKMRKLFFLVMLTTVVSAVAWITITFFGESVKFAFDKE-TNSSITVEIPRLPIKSFYPWDASQGLFYTISFALQGYYLLFSMVHSNLCDVLFCSWLIFACEQLQHLKGIMKPLMELSASLDTY--RPNSAALFRSLSA---NSKSELIINEEKEPP--------S-DLDMTGIYSTKADWGAQFR-APTTLQTF-------NG---VNGGNPNGLTKKQEMMVRSAIKYWVERHKHVVRLVAAIGDTYGAALLLHMLTSTIKLTLLAYQATKITGVNVYAFTVIGYLGYALAQVFHFCIFGNRLIEESSSVMEAAYSCHWYDGSEEAKTFVQIVCQQCQKAMSISGAKFFTVSLDLFASVLGAVVT---------

>AEA30004.2_odorant_receptor_co-receptor_[Chrysomya_megacephala]

--MQANLQPTKYVGLVADLMPNIKLMKYSGLFMHAFT-----GGSALLKKVYSSIHLVLILMQFIFILVNMALNADEVNELSGNTITALFFTHCVTKFIYLAVNQKNFYRTLNIWNQVNSHPLFAESDARYHSIALAKMRKLFFLVMLTTVASAVAWITITFFGESVKFAFDKE-TNSSITVEIPRLPIKSFYPWDSSQGMFYIISFAFQGYYLLFSMVHSNLCDVLFCSWLIFACEQLQHLKGIMKPLMELSASLDTY--RPNSAALFRSLSA---NSKSELIINEEKEPP--------T-DLDMSGIYSSKADWGAQFR-APTTLQTF-------NG---VNGGNPNGLTKKQEMMVRSAIKYWVERHKHVVRLVAAIGDTYGAALLLHMLTSTIKLTLLAYQATKITGVNVYAFTVIGYLGYALAQVFHFCIFGNRLIEESSSVMEAAYSCHWYDGSEEAKTFVQIVCQQCQKAMSISGAKFFTVSLDLFASVLGAVVTYFMVLVQLK

>AOE48068.1_putative_odorant_receptor_ORco_[Scaeva_pyrastri]

-----MQQQTKYIGLVADLMPNIKLMKYSGLFMHNYTS----G-SSFFKKVYSCFHFTLILLQFFSILANMALNADEVNELSGNTITALFFTHCITKFIFFAVNQKQFYRTLNIWNQVNSHPLFAESDARYHSIALAKMRKLFVLVMLTTVLSVVAWTTITFFGESVKFARDME-TNETITVEIPRLPIKAFYPWDASSGMFYMISFVYQVYFLLFSTTQSNLCDVLFCSWLIFACEQLQHLKGIMKPLMELSATLDTY--RPNSAALFRSLSA---NSKSELIINEEKEPV---------DTLDMSGVYSTKADWGAQFR-APSTLQTFNGT----NG---M--SNPNGLTKKQEMMVRSAIKYWVERHKHVVRLVAAIGDTYGAALLLHMLTSTIKLTLLAYQATKINGVNVYAFTVIGYLSYSLAQVFHFCIFGNRLIEESSSVMEAAYSCHWYDGSEEAKTFVQIVCQQCQKAMTISGAKFFTVSLDLFASVLGAVVTYFMVLVQLK

>AID61201.1_odorant_receptor,_partial_[Calliphora_stygia]

--MQSNLQPTKYVGLVADLMPNIKLMKYSGLFMHAFT-----GGSPLLKKVYSSIHLVLILAQFIFILVNMALNADEVNELSGNTITALFFTHCVTKFIYLAVNQKNFYRTLNIWNQVNSHPLFAESDARYHSIALAKMRKLFFLVMLTTVASAVAWITITFFGESVKFAFDKE-TNSSITVEIPRLPIKSFYPWDASQGMFYTISFALQGYYLLFSMVHSNLCDVLFCSWLIFACEQLQHLKGIMKPLMELSASLDTY--RPNSAALFRSLSA---NSKSELIINEEKEPP--------S-DLDMTGIYSTKADWGAQFR-APTTLQTF-------NG---VNGGNPNGLTKKQEMMVRSAIKYWVERHKHVVRLVAAIGDTYGAALLLHMLTSTIKLTLLAYQATKITGVNVYAFTVIGYLGYALAQVFHFCIFGNRLIEESSSVMEAAYSCHWYDGSEEAKTFVQIVCQQCQKAMSISGAKFFTVSLDLFASVLGAVVTYFMVLVQLK

>ABB29301.1_putative_odorant_receptor_Or7_[Culex_quinquefasciatus]

----MNVQPTKYQGLVADLMPNIRLMQGVGHFLFRYVT----G-PIFIRKLYSWWNLTMILLQFFSIAANLVMNTGDVNELTANTITTLFFVHSVTKFVFFAVNAEGFYRTLGIWNNPNAHPLFAESDARYHSIALAKMRKLLVMVMTTTVLSVVAWITITFFGDSVKGVLDKE-TNETYIVEIPRLPIKAWYPWDAMSGAGYVFSFIYQAYFLLFSMCQANLADVLFCSWLLFACEQLQHLKGIMRPLMELSASLDTY--RPNSAALFRAISA---GSKSKLILNEEKDPD--------SKDFDLSGIYSSKADWGAQFR-APSTLQTFE------NGMNG-EKGNPNGLTRKQEMMVRSAIKYWVERHKHVVRLVSAIGDTYGAALLLHMLTSTIKLTLLAYQATKIDGLNVYGLTVIGYLVYALAQVFLFCIFGNRLIEESSSVMEAAYSCHWYDGSEEAKTFVQIVCQQCQKAMTISGAKFFTVSLDLFASVLGAVVTYFMVLVQLK

>AMQ13062.1_odorant_receptor_7_[Culex_pipiens_pallens]

----MNVQPTKYQGLVADLMPNIRLMQGVGHFLFRYVT----G-PIFIRKLYSWWNLTMILLQFFSIAANLVMNTGDVNELTANTITTLFFVHSVTKFVFFAVNAEGFYRTLGIWNNPNAHPLFAESDARYHSIALAKMRKLLVMVMTTTVLSVVAWITITFFGDSVKGVLDKE-TNETYIVEIPRLPIKAWYPWDAMSGPGYVFSFIYQAYFLLFSMCQANLADVLFCSWLLFACEQLQHLKGIMRPLMELSASLDTY--RPNSAALFRAISA---GSKSELILNEEKDPD--------SKDFDLSGIYSSKADWGAQFR-APSTLQTFE------NGMNG-EKGNPNGLTRKQEMMVRSAIKYWVERHKHVVRLVSAIGDTYGAALLLHMLTSTIKLTLLAYQATKIDGLNVYGLTVIGYLVYALAQVFLFCIFGNRLIEESSSVMEAAYSCHWYDGSEEAKTFVQIVCQQCQKAMTISGAKFFTVSLDLFASVLGAVVTYFMVLVQLK

>XP_001651426.1_AAEL005776-PA_[Aedes_aegypti]

----MNVQPTKYHGLVLDLMPNIRLMQGFGHFLFRYVN----G-PVLIRKLYSWWNLIMILLQYFAIMGNLVMNTGDVNELTANTITTLFFTHSVTKFIYVAVNSEHFYRTLGIWNQPNSHSLFAESDARYHSIALAKMRKLLVMVMVTTVLSVVAWITITFFGDSVKNVFDKE-TNETYTVEIPRLPIKAWYPWDAMSGVPYFFSFIYQAYFLLFSMCQANLADVMFCSWLLFTCEQLQHLKGIMRPLMELSATLDTY--RPNSAALFRVASA---GSKSELILNEEKDPD--------TKDFDLNGIYNSKADWGAQFR-APSTLQTFGD-----NGI----NGNPNGLTKKQELMVRSAIKYWVERHKHVVRLVSAIGETYGAALLLHMLTSTIKLTLLAYQATKIDALNVYGLTVIGYLVYALAQVFLFCIFGNRLIEESSSVMEAAYSCHWYDGSEEAKTFVQIVCQQCQKAMTISGAKFFTVSLDLFASVLGAVVTYFMVLVQLK

>AHL20247.1_odorant_co-receptor_[Aedes_albopictus]

----MHVQPTKYHGLVLDLMPNIRLMQGFGHFLFRYVS----G-PVLIRKLYSWWNLIMILLQYFAIMGNLVMNTGDVNELTANTITTLFFTHSVTKFIYVAVNSEHFYRTLGIWNQPNSHSLFAESDARYHSIALAKMRKLLVMVMVTTVLSVVAWITITFFGDSVKNVFDKE-TNETYTVEIPRLPIKALYPWDAMSGVPYFFSFVYQAYFLLFSMCQANLADVMFCSWLLFTCEQLQHLKGIMRPLMELSASLDTY--RPNSAALFRAASA---GSKAELILNEEKDPD--------TKDFDLNGIYNSKADWGAQFR-APSTLQTFNDN----NG----MNGNPNGLTKKQELMVRSAIKYWVERHKHVVRLVSAIGETYGAALLLHMLTSTIKLTLLAYQATKIDALNVYGLTVIGYLVYALAQVFLFCIFGNRLIEESSSVMEAAYSCHWYDGSEEAKTFVQIVCQQCQKAMTISGAKFFTVSLDLFASVLGAVVTYFMVLVQLK

>ETN66183.1_putative_olfactory_receptor_manually_corrected_[Anopheles_darlingi]

----MQVQPTKYVGLVADLMPNIRLMQASGHFLFRYVT----G-PILIRKLYSWWTLIMVLLQFFAILGNLATNADDVNELTANTITTLFFTHSVTKFIYFAVNSENFYRTLGVWNQTNTHPLFAESDARYHSIALAKMRKLLVLVMGTTVLSVVAWVTITFFGESVKNVLDKE-TNETYTVEIPRLPIKSWYPWNAMSGPAYIFSFIYQIYYLLFSMVQSNLVDVMFCSWLLLACEQLQHLKGIMRPLMELSASLDTY--RPNSAALFRAISA---GSKSELILNEEKEPE-------PVKDFDLSGIYSSKADWGAQFR-APTTLQTFDENG---------RNGNPNGLSRNQEMMVRSAIKYWVERHKHVVRLVTAIGDTYGPALLLHMLTSTIKLTLLAYQATKIDGVNVYGLTVIGYLCYALAQVFLFCIFGNRLIEESSSVMEAAYSCHWYDGSEEAKTFVQIVCQQCQKAMTISGAKFFTVSLDLFASVLGAVVTYFMVLVQLK

>AAR14938.1_seven_transmembrane_G_protein-coupled_receptor_[Anopheles_gambiae]

----MQVQPTKYVGLVADLMPNIRLMQASGHFLFRYVT----G-PILIRKVYSWWTLAMVLIQFFAILGNLATNADDVNELTANTITTLFFTHSVTKFIYFAVNSENFYRTLAIWNQTNTHPLFAESDARYHSIALAKMRKLLVLVMATTVLSVVAWVTITFFGESVKTVLDKA-TNETYTVDIPRLPIKSWYPWNAMSGPAYIFSFIYQIYFLLFSMVQSNLADVMFCSWLLLACEQLQHLKGIMRSLMELSASLDTY--RPNSSQLFRAISA---GSKSELIINEEKDPD--------VKDFDLSGIYSSKADWGAQFR-APSTLQTFDENG---------RNGNPNGLTRKQEMMVRSAIKYWVERHKHVVRLVSAIGDTYGPALLLHMLTSTIKLTLLAYQATKIDGVNVYGLTVIGYLCYALAQVFLFCIFGNRLIEESSSVMEAAYSCHWYDGSEEAKTFVQIVCQQCQKAMTISGAKFFTVSLDLFASVLGAVVTYFMVLVQLK

>AIO10777.1_odorant_receptor_co-receptor_[Anopheles_funestus]

----MQVQPTKYVGLVADLMPNIRLMQASGHFLFRYVT----G-PILIRKVYSRWTLIMVLMQFFAILGNLASNADDVNELTANTITTLFFTHSVTKFIYFAVNSENFYRTLGIWNQTNSHPLFAESDARYHSIALAKMRKLLVLVMATTILSVVAWVTITFFGESVQNVFDKE-TNETYKVVIPRLPIKSWYPWNAMSGPAYIFSFIYQIYFLLFSMVQSNLADVMFCSWLLLACEQLQHLKGIMRPLMELSASLDTY--RPNSAALFRAISA---GSKSELIINEEKDPD--------VKDFDLSGIYSSKADWGAQFR-APSTLQTFDENG---------RNGNPNGLTRKQEMMVRSAIKYWVERHKHVVRLVSAIGDTYGPALLLHMLTSTIKLTLLAYQATKIDGVNVYGLTVIGYLCYALAQVFLFCIFGNRLIEESSSVMEAAYSCHWYDGSEEAKTFVQIVCQQCQKAMTISGAKFFTVSLDLFASVLGAVVTYFMVLVQLK

>AFH96943.1_odorant_receptor_co-receptor_[Chrysomya_rufifacies]

--MQANLQPTKYVGLVADLMPNIRLMKYSGLFMHAFT-----GGSALLKKVYSSIHLMLILIQFIFILVNMALNADEVNELSGNTITALFFTHCVTKFIYLAVNQKNFYRTLNIWNQVNSHPLFAESDARYHSIALAKMRKLFFLVMLTTVASAVAWITITFFGESVKFAFDKE-TNSSITVEIPRLPIKSFYPWDASQGMFYIISFAFQGYYLLFSMLHSNLCDVLFCSWLIFACEQLQHLKGIMKPLMELSASLDTY--RPNSAALFRSLSA---NSKSELIINEEKEPP--------T-DLDMSGIYSTKADWGAQFR-APTTLQTF-------------NGGNPNGLTKKQEMMVRSAIKYWVERHKHVVRLVAAIGDTYGAALLLHMLTSTIKLTLLAYQATKITGVNVYAFSVIGYLGYALAQVFHFCIFGNRLIEESSSVMEAAYSCHWYDGSEEAKTFVQIVCQQCQKAMSISGAKFFTVSLDLFASVLGAVVTYFMVLVQLK

>XP_011558816.1_PREDICTED:_odorant_receptor_coreceptor_[Plutella_xylostella]

-----MMNKVKAQGLVSDLMPNIKLMQMAGHFLFNYHEENG-GMSMLLRKIYASVHAFLIVIHYLCMLLNMAQYSDDVNELTANTITVLFFAHTVIKLLYFAINSKSFYRTLAVWNQSNSHPLFTESDARYHQLALTKMRRLMYFICAVTVLSVISWVTLTFFGESVRFIPDKE-TNETLTEPAPRLPLKAWYPFDAMSGGMYIVAFAYQVYWLLFAMAIANLMDVMFCSWLLFACEQLQHLKAIMKPLMELSASLDTY--RPNTAELFRANSA----DK-------EKVPD--------PVDMDIRGIYSTQHDFGMTLRGAGGRLQNFGGQ----------QVNNPNGLTQKQEMLARSAIKYWVERHKHVVRLVASIGDTYGTALLFHMLVSTITLTLLAYQATKIDGLNVYAFSTIGYLSYTLGQVFHFCIFGNRLIEESSSVMEAAYSCQWYDGSEEAKTFVQIVCQQCQKAMSISGAKFFTVSLDLFASVLGAVVTYFMVLVQLK

>CAD31851.1_chemosensory_receptor_2_[Heliothis_virescens]

-----MMTKVKAQGLVSDLMPNIKLMQMAGHFLFNYHSENA-GMSNLLRKIYASTHAILIFIHYACMGINMAKYSDEVNELTANTITVLFFAHTIIKLAFFALNSKSFYRTLAVWNQSNSHPLFTESDARYHQIALTKMRRLLYFICGMTVLSVISWVTLTFFGESVRMITNKE-TNETLTEVVPRLPLKAWYPFNAMSGTMYIVAFAFQVYWLLFSMAIANLMDVMFCSWLIFACEQLQHLKAIMKPLMELSASLDTY--RPNTAELFRASST----EK-------EKIPD--------TVDMDIRGIYSTQQDFGMTLRGAGGRLQNFGQ-----------QNPNPNGLTPKQEMLARSAIKYWVERHKHVVRLVASIGDTYGTALLFHMLVSTITLTLLAYQATKINGINVYAFSTIGYLSYTLGQVFHFCIFGNRLIEESSSVMEAAYSCQWYDGSEEAKTFVQIVCQQCQKAMSISGAKFFTVSLDLFASVLGAVVTYFMVLVQLK

>AEA76288.1_odorant_receptor_2_[Argyresthia_conjugella]

-----MMTKTKTQGLVSDMMPNIRLMR-AGHFLFNYYNESG-GMSMLLRKIYASFHAVMLVIHFMCMAMNMAKYSDDVNELTANTITVLFFAHSCIKVLYFALNAKSFYRTLAVWNQSNIHPLFTESDARYHQLALTKMRRLLYFICGITALAVISWVTLTFFGESVRFITDKE-TNETLTEPAPRLPLKAWYPFDAMAGPMYIFAFVFQVYWLLLSMSVCNLMDVMFCSWLIFACEQLQHLKAIMKPLMELSASLDTY--RPNTAELFRAGSA----EKQ------EKTPD--------PVALDIRGIYSTQQDFGMTLRGAGGRLQNFNQP----------VANNPNGLTQKQEMLARSAIKYWVERHKHVVRLVASIGDTYGTALLFHMLVSTITLTLLAYQATKIDGLNVYAFSTIGYLSYTLGQVFHFCIFGNRLIEESSSVMEAAYSCQWYDGSEEAKTFVQIVCQQCQKAMSISGAKFFTVSLDLFASVLGAVVTYFMVLVQLK

>NP_001037060.1_olfactory_receptor_2_[Bombyx_mori]

-----MMTKVKTQGLVTDLMPCIRLLQAAGHFLFNYHADTS-GMNMLLRKIYSSAHAVLIVVHYICMGINMAQYKDEVNELTANTITVLFFAHSIIKLAFFAFNSKSFYRTLAVWNQSNSHPLFTESDARYHQISLSKMRRLLYFICGMTVFSVISWVTLTFFGESVRMIASKE-TNETLTEPAPRLPLKAWYPFKTMSGGGYVFAFIYQIYFLLFSMALANLLDVIFCSWLIFACEQLQHLKAIMKPLMELSAALDTY--RPNTAELFRVSST----DKT------EKVPD--------AVDMDIRGIYSTQQDFGMTLRGAGGKLQNFN------------AENNPNGLTAKQEMLARSAIKYWVERHKHVVRLVASIGDTYGTALLFHMLVSTITLTLLAYQATKINGINVYAFSTIGYLVYTLGQVFHFCIFGNRLIEESSSVMEAAYSCQWYDGSEEAKTFVQIVCQQCQKAMTISGAKFFNVSLDLFASVLGAVVTYFMVLIQLK

>CAD88205.1_putative_chemosensory_receptor_2_[Antheraea_pernyi]

-----MMTNVKTQGLVTYLLPNIKLLQLAGHFLFNYHADNS-GMATLLRRVYATVHAILIIIHYVCMGVNMAHYSDEVNELTANTVTVLFFAHTIIKLLFFAISSKSFYRTMAVWNQSNSHPLFTESDARYHQIAVTKIRRLLYFVCGMTVFSVLSWIILTFFGESVRLIANKE-TNETLTEPAPRLPLKAWYPFDAMGGSMYVLAFIFQIYWLLFSMAIANLLDVLFCSLLIFACEQLQHLKAIMKPLMELSAALDTY--RPNTAELFR-VSN----DKS------EKVPD--------SVDLDIRGIYSTQQDFGMTLRGTGGKLQNFGN-----------TPSNPNGLTQKQEMLARSAIKYWVERHKHIVRLVGSIGDTYGTALLFHMLVSTITLTLLAYQATKVNGINVYAFSTIGYLGYTLGQVFHFCIFGNRLIEESSSVMEAAYSCQWYDGSEEAKTFVQIVCQQCQKAMTISGAKFFTVSLDLFASVLGAVVTYFMVLVQLK

>CUQ99422.1_Olfactory_receptor_coreceptor_[Manduca_sexta]

-----MMAKVKTQGLVTDVMPNIKLLQLSGHFLFNYYADNS-GMTMLLRKMYSTVHAILIFVQFVCMGVNMAMYADEVNELTANTITVLFFAHSIIKLGFLAFTSKSFYRTMAVWNQSNSHPLFTESDARYHQIALTKMRRLTYFICFMTVMSVVSWVTITFFGESVRMIANKE-TNETLTEPAPRLPLKAWYPFDTMSGSMYVFVFVFQIYWLLFSMSMANLLDVLFCAWLIFACEQLQHLKAIMKPLMELSASLDTY--RPNTAELFRVSST----DKS------EKVPD--------PVDMDIRGIYSTQQDFGMTLRGTGGKLQNFVQ-----------NTVNPNGLTQKQEMLARSAIKYWVERHKHVVRLVASIGDTYGTALLFHMLVSTITLTLLAYQATKINSINVYAFSTIGYLCYTLGQVFHFCIFGNRLIEESSSVMEAAYSCQWYDGSEEAKTFVQIVCQQCQKALTISGAKFFTVSLDLFASVLGAVVTYFMVLVQLK

>AKW50880.1_odorant_receptor_coreceptor_[Ectropis_obliqua]

-----MMAKSKSVGLVSDMMPNIRLMQWAGHFLFNYYDENS-GMNMLLRKVYACVHAFLISLHFIFMCINMTQYSGEVNEFTANTITVLFFAHTLIKLVVFAFNSKNFYRTLAVWNQSNSHPLFTESDARYHQQALTKMRKLLYMICAVTGGAVISWVTITFFGESVRMITNKE-TNETLTEPAPRLPLKAWYPFDAMSGSMYIFAFVFQTYWLIFSLGIANLLDLMFCSWLIFACEQLKHLKAIMKPLMELSASLDTY--RPNTAELFRISSS----LNS------EKMPD--------TTDADIRGIYATQQDFGMTLRGAGGRLQNFVA-----------NPNNPNGLSQKQEMLARSSIKYWVERHKHVVRLVASIGDTYGTALLFHMLVSTITLTLLAYQATKINGMNVYAFSTIGYLSYTLGQVFHFCIFGNKLIEESSSIMEAAYSCQWYDGSEEAKTFVQIVCQQCQKAMSISGAKFFTVSLDLFASVLGAVVTYFMVLVQLK

>AQQ73487.1_olfactory_receptor_2_[Heliconius_melpomene_rosina]

-----MMTKIKTQGLVSDLMPNIKLMQAAGHFLFNYHSDNS-GMSTLLRKVYSSVHAFLIMIHYLCMAANMAKYSEEVNELTANTITVLFFTHSIIKLAFFAITSKNFYRTLAVWNQSNSHPLFTESDARYHQLSLNKMRRLLYFISGMTIFSVVCWVTITFFGESVRFLVDKE-TNDTLTEPVPRLPLKAWYPFDAMGGTMYIIAFAFQIYWLLFSMAMANLLDVMFCSWLIFACEQLQHLKAIMKPLMELSASLDTY--RPNTAELFKVS------ENS------EKIPD--------STDLDIRGIYSTQQDFGMNLRGAGGRLQTFGQ-----------QNNNPNGLTQKQEMLARSAIKYWVERHKHIVRLVSSIGDTYGTALLFHMLVSTITLTLLAYQATKINGLNVYAFSTVGYLSYTLAQVFHFCIFGNKLIEESSSVMEAAYSCQWYDGSEEAKTFVQIVCQQCQKAMSISGAKFFTVSLDLFASVLGAVVTYFMVLVQLK

>ALT31679.1_odorant_receptor_co-receptor_[Cnaphalocrocis_medinalis]

-----MMTKVKAQGLVSDLMPNIKLMQAAGHFLFNYHSDNA-GMSTLLRKIYASAHAVLIVIHYLCMAVNMAQYSEEVNELTANTITVLFFAHSVIKLLFFAINSKSFYRTLAVWNQSNSHPLFTESDARYQQLALTKMRRLLYFICGVTVLAVVSWITLTFFGESVRLIANKE-TNETLTEPAPRLPLKAWYPFDAMSGTMYVVAFVYQIYWLLFSMAMANLLDVMFCSWLIFACEQLQHLKAIMKPLMELSASLDTY--RPNTAELFRASST----DKS------EKVPD--------PVDMDIRGIYSTQQDFGMTLRGAGGRLQNFGT-----------NGSNPNGLTQKQEMLARSAIKYWVERHKHVVRLVASIGDTYGTALLFHMLVSTITLTLLAYQATKINGINVYAFSTIGYLSYTLGQVFHFCIFGNRLIEESSSVMEAAYSCQWYDGSEEAKTFVQIVCQQCQKALSISGAKFFTVSLDLFASVLGAVVTYFMVLVQLK

>XP_013142400.1_PREDICTED:_odorant_receptor_coreceptor_[Papilio_polytes]

-----MITKIKTQGLVSDLMPNIKLMQMSGHFLFNYYPENA-GMSTLLRKIYASVHAFLIILQYLCMMANMAQYSDEVNELTANTITVLFFAHSIIKLIFFAINSKSFYRTLAMWNQSNSHPLFTESDARYHQLALTKMRRLLYFICGMTVLSVVSWVTITFLGESVRLITNKE-TNETLTEPAPRLPVKTWYPFNAMGGTMYVIAFIFQVYWLLFSMAIANLLDVMFCSWLIFACEQLQHLKAIMKPLMELSASLDTY--RPNTAELFRVSNT----EKS------EKIPD--------TVDLDIRGIYSTQQDFGMTARGAGGRLQTFGQP----------APNNPNGLTQKQELLARSAIKYWVERHKHVVRLVASIGDTYGTALLFHMLVSTITLTLLAYQATKIDGLNVYAFSTIGYLSYTLGQVFHFCIFGNRLIEESSSVMEAAYSCQWYDGSEEAKTFVQIVCQQCQKAMSISGAKFFTVSLDLFASVLGAVVTYFMVLVQLK

>AGS41440.1_odorant_receptor_co-receptor_[Agrotis_segetum]

-----MMTKVKAQGLGSDLLPNIKLMQAAGHFLFNYHSENA-GMSNLLRKIYASTHAILITIHFGCMAVNMAQYSDEVNELTANTITVLFFTHTIIKLSFFALNSKSFYRTLAVWNQSNSHPLFTESDARYHQIALTKMRRLLYFICGMTCLSVVFWITLTFFGESVRLITNKE-TNETLTEPVPRLPLKAWYPFNAMSGTMYIVAFAFQVYWLLFSMAIANLMDVMFCSWLIFACEQLQHLKAIMKPLMELSASLDTY--RPNTAELFRASST----EKS------EKIPD--------AVDMDIRGIYSTQQDFGMTLRGAGGRLQNFGQ-----------QNSNPNGLTPKQEMLARSAIKYWVERHKHVVRLVASIGDTYGTALLFHMLVSTITLTLLAYQATKINGINVYAFSTIGYLSYTLGQVFHFCIFGNRLIEESSSVMEAAYSCQWYDGSEEAKTFVQIVCQQCQKAMSISGAKFFTVSLDLFASVLGAVVTYFMVLVQLK

>BAJ23265.1_odorant_receptor_2,_partial_[Ostrinia_zaguliaevi]

------------QGLVSDLMPNIKLMQAAGHFLFNYHSDNS-GMTTLLRKVYSSVHAFLIVINYLCMAANMAQYSEEVNELTANTITVLFFAHSVIKMLFFAVNSKSFYRTLAVWNQSNSHPLFTESDARYHQLALTKMRRLLYFICGVTVLAVFSWITITFFGESVRMIANKE-TNETLTEPAPRLPLKTWYPFDAMRGTMYVVAFVYQVYWLFFSMAIANLMDVMFCSWLIFACEQLRHLKAIMKPLMELSASLDTY--RPNTAELFRASST----EKS------EKMPD--------TVDMDIRGIYSTQQDFGMTLRGAGGRLQNFGQP----------NPNNPNGLTQKPEMLARSAIKYWVERHKHVVRLVASIGDTYGTALLFHMLVSTITLTLLAYQATKINGINVYAFSTIGYLSYTLGQVFHFCIFGNRLIEESSSVMEAAYSCQWYDGSEEAKTFVQIVCQQCQKAMSIPGAKFFTVSLDLFASVLGAV-----------

>EHJ65756.1_olfactory_receptor-2,_manually_corrected_[Danaus_plexippus]

-----MITKSENRGLVSDLMPNIKLMQMAGHFLFNYHSDNS-GMSSLLRKCYSSIHAFLIVMHYLCMAANMAKYSEEVNELTANTITVLFFSHSIIKLTFFAITSKNFYRTLAVWNQSNSHPLFTESDARYHQLALTKMRRLLYFICGMSFFSVICWVTITFFGESVVMLVDKE-TNETITEPAPRLPLKAWYPFDAMSGTMYIAAFAFQVYWLLFTIFIANLMDVMFCSWLIFACEQLQHLKAIMKPLMELSASLDTY--RPNTAELFKVSP-----EKS------DKTPD--------PIDMDIRGIYSTQQDFGMTLRGAGGRLQNFGQ-----------NNNNPNGLTQKQEMLARSAIKYWVERHKHIVRLVSSIGDTYGTALLFHMLVSTITLTLLAYQATKINGLNVYAFSTVGYLSYTLGQVFHFCIFGNRLIEESSSVMEAAYSCQWYDGSEEAKTFVQIVCQQCQKAMSISGAKFFTVSLDLFASVLGAVVTYFMVLVQLK

>AII15784.1_odorant_receptor_co-receptor_[Sitotroga_cerealella]

-----MMTKVKTQGLVTDLMPNINLMRMFGHFVFNYLPESN-GMSMLLRKIYASVHAVLIFVHFVCVGVNMAKYADEVNELTANTITVLFFTHTIIKLNYFAINSRSFYRTLAVWNQSNSHPLFTESDARYHQLAVSKNRKLLYFICSTTCISVVCWVTITFFGESVYLIMDKE-TNETMTTPAPRLPLKAWYPFNAMSGTMYIVMFGFQVYWLLFSMMLANLMDVLFCSWLVFACEQLQHLKAIIKPLMELSASLDTY--RPNTAELFRASST----EKS------EKVPD--------PVDLDIRGIYSTQQDFGVTLRGAGGRLQNFGQ-----------NPPNPNGLTQKQEMLVRSAIKYWVERHKHVVRLVSSIGDTYGTALLFHMLVSTITLTLLAYQATKIDGLNVYAFSTIGYLSYTLGQVFHFCIFGNRLIEESSSVMGAAYSCQWYDGSEEAKTFVQIVCQQCQKAMSISGAKFFTVSLDLFASVLGAVVTYFMVLVQLK

>BAJ23260.1_odorant_receptor_2,_partial_[Ostrinia_zealis]

------------QGLVSDLMPNIKLMQAAGHFLFNYHSDNS-GMTTLLRKVYSSVHAFLIVINYLCMAANMAQYSDEVNELTANTITVLFFAHSVIKMLFFAVNSKSFYRTLAVWNQSNSHPLFTESDARYHQLALTKMRRLLYFICGVTVMSVISWIIITFFGESVRMIANKE-TNETLTEPAPRLPLKTWYPFDAMSGTMYVVAFVYQVYWLFFSMAIANLMDVMFCSWLIFACEQLQHLKAIMKPLMELSASLDTY--RPNTAELFRASST----EKS------EKMPD--------TVDMDIRGIYSTQQDFGMTLRGAGGRLQNFGQP----------NPNNPNGLTQKQEMLARSAIKYWVERHKHVVRLVASIGDTYGTALLFHMLVSTITLTLLAYQATKINGINVYAFSTIGYLSYTLGQVFHFCIFGNRLIEESSSVMEAAYSCQWYDGSEEAKTFVQIVCQQCQKAMSISGAKFFTVSLDLFASVLGAV-----------

>BAH57974.1_olfactory_receptor_[Ostrinia_latipennis]

------MNQVKAQGLVSDLMPNIKLMQAAGHFLFNYHSDNS-GMTTLLRKVYSSVHAFLIVINFLCMVANMAQYSEEVNELTANTITVLFFAHSVIKMFFFAVNSKSFYRTLAVWNQSNSHPLFTESDARYHQLALTKMRRLLYFICGVTVLAVISWITITFFGESVRMIANKE-SNDTLTEPAPRLPLKTWYPFDAMSGTMYIVAFVHQVYWLFFSMAIANLMDVMFCSWLIFACEQLQHLKAIMKPLMELSASLDTY--RPNTAELFRASST----EKS------EKMPD--------TVDMDIRGIYSTQQDFGMTLRGAGGRLQNFGQS----------NPNNPNGLTQKQEMLARSAIKYWVERHKHVVRLVASIGDTYGTALLFHMLVSTITLTLLAYQATKINGINVYAFSTIGYLSYTLGQVFHFCIFGNRLIEESSSVMEAAYSCQWYDGSEEAKTFVQIVCQQCQKAMSISGAKFFTVSLDLFASVLGAVVTYFMVLVQLK

>AGG91643.1_odorant_receptor_[Ostrinia_furnacalis]

-----MMTKVKAQGLVSDLMPNIKLMQAAGHFLFNYHSDNS-GMTTLLRKVYSSVHAFLIVINYLCMAANMAQYSEEVNELTANTITVLFFAHSVIKMLFFAVNSKSFYRTLAVWNQSNSHPLFTESDARYHQLALTKMRRLLYFICGVTVLAVLSWITITFFGESVRMIANKE-TNETLTEPAPRLPLKTWYPFDAMSGTMYVVAFVYQVYWLFFSMAIANLMDVMFCSWLIFACEQLQHLKAIMKPLMELSASLDTY--RPNTAELFRASST----EKS------EKMPD--------TVDMDIRGIYSTQQDFGMTLRGAGGRLQNFGQP----------NPNNPNGLTQKQEMLARSAIKYWVERHKHVVRLVASIGDTYGTALLFHMLVSTITLTLLAYQATKINGINVYAFSTIGYLSYTLGQVFHFCIFGNRLIEESSSVMEAAYSCQWYDGSEEAKTFVQIVCQQCQKAMSISGAKFFTVSLDLFASVLGAVVTYFMVLVQLK

>AGF29886.1_odorant_co-receptor_[Conogethes_punctiferalis]

-----MMNKVKALGLVSDLMPNIKLMQAAGHFLFNYHSDNS-GMAMLLRKIYASVHAFLIVIHYLCMAVNMAQYSEEVNELTANTITVLFFAHSVIKLLFFALNSKSFYRTLAVWNQSNSHPLFTESDARYHQLSLTKMRRLLYFICGVTVLAVVCWVTITFFGESVRMIANKE-TNETLTEPAPRLPLKAWYPFDAMGGTMYVVAFVFQVYFLFFSMAIANLMDVMFCSWLIFACEQLQHLKAIMKPLMELSASLDTY--RPNTAELFRASST----EKS------EKVPD--------PVDMDIRGIYSTQQDFGMTLRGAGGRLQNFGGN----------PTNNPNGLTQKHEMLARSAIKYWVERHKHVVRLVASIGDTYGTALLFHMLVSTITLTLLAYQATKINGINVYAFSTIGYLSYTLGQVFHFCIFGNRLIEESSSVMEAAYSCQWYDGSEEAKTFVQIVCQQCQKAMSISGAKFFTVSLDLFASVLGAVVTYFMVLVQLK

>AHA50097.1_odorant_receptor_OrCO,_partial_[Lymantria_dispar_asiatica]

-------------------------------------------MSTLLRKIYAGIHTFLILLNFICLGINAAQYADEVNELTANTITVLFFTHTLIKLLFFAMNSKNFYRTLAVWNQSNSHPLFTESDARYHQISLTKMRRLLYFICGMTVLSVISWVTITFFGESVRLITSKE-TNETLTEPAPRLPLKAWYPFNAMSGTMYVLAFIFQIYWLLFSMAIPNLMDVMFCSWLIFACEQLQHLKAIMKPLMELSASLDTY--RPNTAELFRASST----EKS------ERAQD--------PTDLDIRGIYSTQQDFGMTIRGTGGRLQNFNQ-----------GGNNPNGLSKNQEMLARSAIKYWVERHKHVVRLVASIGDTYGTTLLFHMLVSTITLTLLAYQATKINRIDVYAFSTVGYLGYTLGQVFHFCIFGNRLIEESSSVMEAAYSCQWYDGSEEAKTFVQIVCQQCQKAMSISGAKFFTVSLDLFASVLGAVVTYFMVLVQLK

>ALM30348.1_odorant_receptor_[Galleria_mellonella]

-----MMTKVKAQGLVTDLMPNIKLMQAAGHFLFNYHSDNS-GMSMLLRKVYSSVHAVLIVVNYVCMAINMAQYSDEVNELTANTITVLFFAHSVIKLLFFALNSKSFYRTLAIWNQSNSHPLFTESDARYHQLALTKMRRLLYCICGVTVLSVASWVTLTFFGESVRFIANKE-TNETMTEPAPRLPLKAWYPFNTMSGTMYIAAFALQIYWLLFSMAIANLLDVMFCSWLIFACEQLQHLKAIMKPLMELSASLDTY--RPNTAELFRASST----EKS------EKVPD--------PVDLDIRGIYSTQQDFGITLRGAGGKLQTFGQP----------TPNNPNGLTQKQEMLARSAIKYWVERHKHVVRLVASIGDTYGTALLFHMLISTITLTLLAYQATKIDGVNVYAFSTLGYLTYTLGQVFHFCIFGNRLIEESSSVMEAAYSCQWYDGSEEAKTFVQIVCQQCQKAMSISGAKFFTVSLDLFASVLGAVVTYFMVLIQLK

>XP_013167416.1_PREDICTED:_odorant_receptor_coreceptor_[Papilio_xuthus]

-----MMTKIKTQGLVSDLMPNIKLMQMAGHFLFNYYPENA-GMSILLRKIYASVHAFLIIIQYLCMMANMAQYSDEVNELTANTITVLFFAHSIIKLIFFAINSKSFYRTLAMWNQSNSHPLFTESDARYHQLALTKMRRLLYFICGMTVLSVVCWVTITFFGESVRLITNKE-TNETLTEPAPRLPVKAWYPFNAMSGTMYVVAFIFQVYWLLFSMAIANLLDVMFCSWLIFACEQLQHLKAIMKPLMELSASLDTY--RPNTAELFRVSNT----EKS------EKIPD--------TVDLDIRGIYSTQQDFGMTARGAGGRLQTFGQP----------APNNPNGLTQKQELLARSAIKYWVERHKHVVRLVASIGDTYGTALLFHMLVSTITLTLLAYQATKIDGLNVYAFSTIGYLSYTLGQVFHFCIFGNRLIEESSSVMEAAYSCQWYDGSEEAKTFVQIVCQQCQKAMSISGAKFFTVSLDLFASVLGAVVTYFMVLVQLK

>AFQ94048.1_olfactory_receptor_2_[Chilo_suppressalis]

-----MMAKVKAQGLVSDLMPNIKLMQAAGHFLFNYHSDNS-GMSTLLRKIYSSVHAILIVINFLCMAVNMAQYSDEVNELTANTITVLFFTHTVIKLLFFAVNSKSFYRTLAVWNQSNSHPLFTESDARYHQLALTKMRRLLYFICTVTVLSVVSWVTITFFGESVRLIANKE-TNETLTEPAPRLPLKAWYPFDAMSGTMYIIAFAYQVYWLLFSMAIANLMDVMFCSWLIFACEQLQHLKAIMKPLMELSASLDTY--RPNTAELFRASST----EKS------EKVPD--------PVDLDIRGIYSTQQDFGMTLRGGGGRLQTFGQQ----------NTNNPNGLSQKQEMLARSAIKYWVERHKHVVRLVASIGDTYGTALLFHMLVSTITLTLLAYQATKIGGINVYAFSTVGYLSYTLGQVFHFCIFGNRLIEESSSVMEAAYSCQWYDGSEEAKTFVQIVCQQCQKAMSISGAKFFTVSLDLFASVLGAVVTYFMVLVQLK

>XP_014363049.1_PREDICTED:_odorant_receptor_coreceptor_[Papilio_machaon]

-----MMTKIKTQGLVSDLMPNIKLMQMFGHFLFNYYPENG-GMSTLLRKIYASVHAFLIIIQYLCMMANMAQYSDEVNELTANTITVLFFAHSIIKLIFFAFNSKSFYRTLAMWNQSNSHPLFTESDARYHQLALTKMRRLLYFIFGMTVLSVVCWVTITFFGESVRLITNKE-TNETLTEPAPRLPVKAWYPFNAMNGTMYVVAFIFQVYWLLFSMAIANLLDVMFCSWLIFACEQLQHLKAIMKPLMELSASLDTY--RPNTAELFRVSNT----EKS------EKIPD--------TVDLDIRGIYSTQQDFGMTARGAGGRLQTFGQP----------APNNPNGLTQKQELLARSAIKYWVERHKHVVRLVASIGDTYGTALLFHMLVSTITLTLLAYQATKIDGLNVYAFSTVGYLSYTLGQVFHFCIFGNRLIEESSSVMEAAYSCQWYDGSEEAKTFVQIVCQQCQKAMSISGAKFFTVSLDLFASVLGAVVTYFMVLVQLK

>BAG71418.1_olfactory_receptor-2_[Diaphania_indica]

-----MMTKVKAQGLVSDLMPNIKLMQAAGHFLFNYHSDNS-GMSTLLRKVYSSAHAFLIVIHYLCMAVNMAQYSEEVNELTANTITVLFFAHSVIKLVFFAINSKSFYRTLAVWNQSNSHPLFTESDARYHQLSLTKMRRLLYFICGVTVLSVISWVTLTFFGESVRLIANKE-TNETLTEPAPRLPLKAWYPFDAMSGTMYVVAFVYQVYWLLFSMAIANLMDVMFCSWLIFACEQLQHLKAIMKPLMELSASLDTY--RPNTAELFRASST----EKS------EKVPD--------PVDMDIRGIYSTQQDFGMTLRGAGGHLQNFGS-----------NGNNPNGLTQKQEMLARSAIKYWVERHKHVVRLVASIGDTYGTALLFHMLVSTITLTLLAYQATKINGINVYAFSTIGYLSYTLGQVFHFCIFGNRLIEESSSVMEAAYSCQWYDGSEEAKTFVQIVCQQCQKAMSISGAKFFTVSLDLFASVLGAVVTYFMVLVQLK

>ABU45983.2_odorant_receptor_Or83b_[Helicoverpa_assulta]

-----MMTKVKAQGLVSDLMPNIKLMQMAGHFLFNYHSENA-GMSNLLRKIYASTHAILIVIHYACMGINMAKYSDEVNELTANTITVLFFAHTIIKLAFFALNSKSFYRTLAVWNQSNSHPLFTESDARYHQIALTKMRRLLYFICGMTVLSVISWVTLTFFGESVRMVTNKE-TNETLTEVVPRLPLKAWYPFNAMSGTMYIVAFAFQVYWLLFSMAIANLMDVMFCSWLIFACEQLQHLKAIMKPLMELSASLDTY--RPNTAELFRASST----EKS------EKIPD--------TVDMDIRGIYSTQQDFGMTLRGAGGRLQNFGQ-----------QNPNPNGLTPKQEMLARSAIKYWVERHKHVVRLVASIGDTYGTALLFHMLVSTITLTLLAYQATKINGINVYAFSTIGYLSYTLGQVFHFCIFGNRLIEESSSVMEAAYSCQWYDGSEEAKTFVQIVCQQCQKAMSISGAKFFTVSLDLFASVLGAVVTYFMVLVQLK

>BAH57973.1_olfactory_receptor_[Ostrinia_scapulalis]

-----MMTKVKAQGLVSDLMPNIKLMQAAGHFLFNYHSDNS-GMTTLLRKVYSSVHAFLIVINYLCMAANMAQYSEEVNELTANTITVLFFAHSVIKMLFFAVNSKSFYRTLAVWNQSNSHPLFTESDARYHQLALTKMRKLLYFICGVTVLAVMSWITITFFGESVRMIANKE-TNETLTEPAPRLPLKTWYPFDAMSGTKYVVAFVYQVYWLFFSMAIANLMDVMFCSWLIFACEQLQHLKAIMKPLMELSASLDTY--RPNTAELFRASST----EKS------EKMPD--------TVDMDIRGIYSTQQDFGMTLRGAGGRLQNFGQP----------NPNNPNGLTQKQEMLARSAIKYWVERHKHVVRLVASIGDTYGTALLFHMLVSTITLTLLAYQATKINGINVYAFSTIGYLSYTLGQVFHFCIFGNRLIEESSSVMEAAYSCQWYDGSEEAKTFVQIVCQQCQKAMSISGAKFFTVSLDLFASVLGAVVTYFMVLVQLK

>ACJ06648.1_G_protein_coupled_receptor_SlOR83b_[Spodoptera_littoralis]

------MTKVKAQGLVSDLMPNIKLMQAAGHFLFNYHAENG-GMSGLLRKIYASTHAILITIHFACMGINMAQYSDEVNELTANTITVLFFTHTIIKLGFFALNSKSFYRTLAVWNQSNSHPLFTESDARYHQIALTKMRRLLYFICGMTVLSVVSWVTLTFFGESVRLITSKE-TNETLTEVAPRLPLKAWYPFNAMSGTTYIIAFAFQVYWLLFSMAIANLMDVMFCSWLIFACEQLQHLKAIMKPLMELSASLDTY--RPNTAELFRASST----EKS------EKIPD--------TVDMDIRGIYSTQQDFGMTLRGAGGRLQAFGQ-----------QNNNPNGLTPKQEMLARSAIKYWVERHKHVVRLVASIGDTYGTALLFHMLVSTITLTLLAYQATKINGINVYAFSTIGYLSYTLGQVFHFCIFGNRLIEESSSVMEAAYSCQWYDGSEEAKTFVQIVCQQCQKAMSISGAKFFTVSLDLFASVLGAVVTYFMVLVQLK

>AFP54145.1_odorant_receptor_coreceptor_[Amyelois_transitella]

----MINNKVKAQGLVSDLMPNIKLMQASGHFLFNYYSDNS-GMSMLLRKIYSSVHAILIVINYVCMVVNMAQYSDEVNELTANTITVLFFAHTVIKLLFFALNSKSFYRTLAVWNQSNSHPLFTESDSRHHQLALTKIRRLLYFICSMTVFSVVSWVTLTFFGESVRLIANKE-TNETISEPAPRLPLKTWYPFDAMGGSMYIIAFAFQVYWLFFSMITANLMDVMFCSWLIFACEQLQHLKAIMKPLMELSASLDTY--RPNTAELFRVSST----EKS------EKVPD--------PVDMDIRGIYATQQDFGMTLRGAGGRLQTFGQQ-----------NNNPNGLSQKQEMLARSAIKYWVERHKHVVRLVTSIGDTYGTALLFHMLISTITLTLLAYQATKIDGINVYAFSTIGYLSYTLGQVFHFCVFGNQLIEESSSVMEAAYSCQWYDGSEEAKTFVQIVCQQCQKAMSISGAKFFTVSLDLFASVLGAVVTYFMVLVQLK

>BAJ23264.1_odorant_receptor_2,_partial_[Ostrinia_ovalipennis]

------------QGLVSDLMPNIKLMQAAGHFLFNYHSDNS-GMTTLLRKVYSSVHAFLIVINYLCMAANMAQYSEEVNELTANTITVLFFAHSVIKMLFFAVNSKSFYRTLAVWNQSNSHPLFTESDARYHQLALTKMRRLLYFICGVTVLAVISWITITFFGESVRMIANKE-TNDTLTEPAPRLPLKTWYPFDAMSGTMYVVAFVYQVYWLFFSMAIANLMDVMFCSWLIFACEQLQHLKAIRKPLMELSASLDTY--RPNTAELFRASST----EKS------EKMPD--------TVDMDIRGIYSTQQDFGMTLRGAGGRLQNFGQS----------NPNNPNGLTQKQEMLARSAIKYWVERHKHVVRLVASIGDTYGTALLFHMLVSTITLTLLAYQATKINGINVYAFSTIGYLSYTLGQVFHFCIFGNRLIEESSSVMEAAYSCQWYDGSEEAKTFVQIVCQQCQKAMSISGAKFFTVSLDLFASVLGAV-----------

>ADQ13177.1_olfactory_receptor_OR83b_[Helicoverpa_armigera]

-----MMTKVKAQGLVSDLMPNIKLMQMAGHFLFNYHSENA-GMSNLLRKIYASTHAILIFIHYACMGINMAKYSDEVNELTANTITVLFFAHTIIKLAFFALNSKSFYRTLAVWNQSNSHPLFTESDARYHQIALTKMRRLLYFICGMTVLSVISWVTLTFFGESVRMVTNKE-TNETLTEVVPRLPLKAWYPFNAMSGTMYIVAFAFQVYWLLFSMAIANLMDVMFCSWLIFACEQLQHLKAIMKPLMELSASLDTY--RPNTAELFRASST----EKS------EKIPD--------TVDMDIRGIYSTQQDFGMTLRGAGGRLQNFGQ-----------QNPNPNGLTPKQEMLARSAIKYWVERHKHVVRLVASIGDTYGTALLFHMLVSTITLTLLAYQATKINGINVYAFSTIGYLSYTLGQVFHFCIFGNRLIEESSSVMEAAYSCQWYDGSEEAKTFVQIVCQQCQKAMSISGAKFFTVSLDLFASVLGAVVTYFMVLIQLK

>AAX14773.1_odorant_receptor_Or83b_[Helicoverpa_zea]

------MTKVKAQGLVSDLMPNIKLMQMAGHFLFNYHSENA-GMSNLLRKIYASTHAILIFIHYACMGINMAKYSDEVNELTANTITVLFFAHTIIKLAFFALNSKSFYRTLAVWNQSNSHPLFTESDARYHQIALTKMRRLLYFICGMTVLSVISWVTLTFFGESVRMVTNKE-TNETLTEVVPRLPLKAWYPFNAMSGTMYIVAFAFQVYWLLFSMAIANLMDVMFCSWLIFACEQLQHLKAIMKPLMELSASLDTY--RPNTAELFRASST----EKS------EKIPD--------TVDMDIRGIYSTQQDFGMTLRGAGGRLQNFGQ-----------QNPNPNGLTPKQEMLARSAIKYWVERHKHVVRLVASIGDTYGTALLFHMLVSTITLTLLAYQATKINGINVYAFSTIGYLSYTLGQVFHFCIFGNRLIEESSSVMEAAYSCQWYDGSEEAKTFVQIVCQQCQKAMSISGAKFFTVSLDLFASVLGAVVTYFMVLVQLK

>ADT82677.1_putative_odorant_receptor_2_[Spodoptera_litura]

-----MMTKVKAQGLVSDLMPNIKLMQAAGHFLFNYHAENG-GMTGLLRKIYASTHAILITIHFACLGINMAQYSDEVNELTANTITVLFSTHTIIKLGFFALNSKSFYRTLAVWNQSNSHPLFTESDARYHQIALTKMRRLLYFICGMTVLSAVSWVTLTFFGESVRLITSKG-TNETLTEVAPRLPLKAWYPFNAMSGTTYIIAFAFQVYWLLFSMAIANLMDVMFCPWLIFACEQLQHLKAIMKPLMELSASPDTY--RPNTAELFRASST----EKS------EKIPD--------TVDMDIRGIYSTQQDFGMTLRGAGGRLQTFGQ-----------QNNNPNGLTPKQEMLARSAIKYWVERHKHVVRLVASIGDTYGTALLFHMLVSTITLTLLAYQATKINGINVYAFSTIGYLSYTLGQVFHFCIFGNRLIEESSSVMEAAYSCQWYDGSEEAKTFVQIVCQQCQKAMSISGAKFLTVSLDLFASVLGAVVTYFMVLVQLK

>AAS49925.1_putative_chemosensory_receptor_2,_partial_[Mamestra_brassicae]

------MTKVKAQGLVSDLMPNIKLMQAAGHFLFNYHSKNA-GMSNLLRKVYASAHAILIIIHFACMGINMAQYSDEVNELTANTITVLFFTHTIIKLGFFALNAKSFYRTLAVWNQSNSHPLFTESDARYHQIALTKMRRLLYFICGMTCLSVVSWITLTFFGESVRLITSKE-TNETLTEVVPRLPLKAWYPFDAMGGTMYIIAFAFQVYWLLFSMAIANLMDVMFCSWLIFACEQLQHLKAIMKPLMELSASLDTY--RPNTAELFRASST----EKS------EKIPD--------TVDMDIRGIYSTQQDFGMTLRGAGGRLQNFGQ-----------QIPNPNGLSPKQEMLARSAIKYWVERHKHVVRLVASIGDTYGTALLFHMLVSTITLTLLAYQATKINGINVYAFSTIGYLSYTLGQVFHFCIFGNRLIEESSSVMEAAYSCQWYDGSEEAKTFVQIVCQQCQKAMSISGAKFFTVSLDLFASV---------------

>AHA50096.1_odorant_receptor_OrCO,_partial_[Lymantria_dispar_dispar]

-------------------------------------------MSTLLRKIYAGIHTFLILLNFICLGINAAQYADEVNELTANTITVLFFTHTLIRLLFFAMNSKNFYRTLAVWNQSNSHPLFTESDARYHQISLTKMRRLLYFICGMTVLSVISWVTITFFGESVRLITSKE-TNETLTEPAPRLPLKAWYPFNAMSGTMYVLAFIFQIYWLLFSMAIPNLMDVMFCSWLIFACEQLQHLKAIMKPLMELSASLDTY--RPNTAELFRASST----EKS------ERVQD--------PTDLDIRGIYSTQQDFGMTIRGTGGRLQNFNQ-----------GGNNPNGLSKNQEMLARSAIKYWVERHKHVVRLVASIGDTYGTTLLFHMLVSTITLTLLAYQATKINRIDVYAFSTVGYLGYTLGQVFHFCIFGNRLIEESSSVMEAAYSCQWYDGSEEAKTFVQIVCQQCQKAMSISGAKFFTVSLDLFASVLGAVVTYFMVLVQLK

>ADB89179.1_odorant_receptor_2_[Ostrinia_nubilalis]

------MTKVKAQGLVSDLMPNIKLMQAAGHFLFNYHSDNS-GMTTLLRKVYSSVHAFLIVINYLCMAANMAQYSEEVNELTANTITVLFFAHSVIKMLFFAVNSKSFYRTLAVWNQSNSHPLFTESDARYHQLALTKMRRLLYFICGVTVLAVMSWITITFFGESVRMIANKE-TNETLTEPAPRLPLKTWYPFDAMSGTMYVVAFVYQVYWLFFSMAIANLMDVMFCSWLIFACEQLQHLKAIMKPLMELSASLDTY--RPNTAELFRASST----EKS------EKMPD--------TVDMDIRGIYSTQQDFGMTLRGAGGRLQNFGQP----------NPNNPNGLTQKQEMLARSAIKYWVERHKHVVRLVASIGDTYGTALLFHMLVSTITLTLLAYQATKINGINVYAFSTIGYLSYTLGQVFHFCIFGNRLIEESSSVMEAAYSCQWYDGSEEAKTFVQIVCQQCQKAMSISGAKFFTVSLDLFASVLGAVVTYFMVLVQLK

>AFI25169.1_odorant_receptor_83b_[Heliothis_viriplaca]

-----MMTKVKAQGLVSDLMPNIKLMQMAGHFLFNYHSENA-GMSNLLRKIYASTHAILIFIHYACMGINMAKYSDEVNELTANTITVLFFAHTIIKLAFFALNSKSFYRTLAVWNQSNSHPLFTESDARYHQIALTKMRRLLYFICGMTVLSVISWVTLTFFGESVRMVTNKE-TNETLTEVVPRLPLKAWYPFNAMSGTMYIVAFAFQVYWLLFSMAIANLMDVMFCSWLIFACEQLQHLKAIMKPLMELSASLDTY--RPNTAELFRASST----EKS------EKIPD--------TVDMDIRGIYSTQQDFGMTLRGAGGRLQNFGQ-----------QNNNPNGLTPKQEMLARSAIKYWVERHKHVVRLVASIGDTYGTALLFHMLVSTITLTLLAYQATKINGINVYAFSTIGYLSYTLGQVFHFCIFGNRLIEESSSVMEAAYSCQWYDGSEEAKTFVQIVCQQCQKAMSISGAKFFTVSLDLFASVLGAVVTYFMVLVQLK

>AGY14565.1_putative_odorant_receptor_[Sesamia_inferens]

-----MMTKVKAQGLVSDLMPNIKLMQAAGHFLFNYHSENA-GMSNLLRKIYASVHAILICINFACMGINMAQYSDEVNELTANTITVLFFTHTIIKLAFFALNSKSFYRTMAVWNQSNSHPLFTESDARYHQIALTKMRKLLYFICGMTCLSVVSWVTLTFFGESVRLITSKE-TNETLTEVAPRLPLKAWYPFNAMGGTTYIIAFAFQVYWLLFAMAIANLMDVMFCSWLIFACEQLQHLKAIMKPLMELSASLDTY--RPNTAELFRASST----EKS------EKIPD--------TVDMDIRSIYSTQQDFGMTLRGAGGRLQNFGQ-----------QNSNPNGLTPKQEMLARSAIKYWVERHKHVVRLVASIGDTYGTALLFHMLVSTITLTLLAYQATKINGINVYAFSTIGYLCYTLGQVFHFCIFGNRLIEESSSVMEAAYSCQWYDGSEEAKTFVQIVCQQCQKAMSISGAKFFTVSLDLFASVLGAVVTYFMVLVQLK

>AAW52583.1_putative_chemosensory_receptor_2_[Spodoptera_exigua]

-----MMTKVKAQGLVSDLMPNIKLMQAAGHFLFNYHSENG-GMTGLLRKIYASTHAILITIHFACMGINMAQYSDEVNELTANTITVLFFTHTIIKLGFFALNSKSFYRTLAVWNQSNSHPLFTESDARYHQIALTKMRRLLYFICGMTVLSVVCWVALTFFGESVRLITSKE-TNETLTEVAPRLPLKAWYPFNAMSGTMYIIAFAFQVYWLLFSMAIASLMDVMFCSWLIFACEQLQHLKAIMKPLMELSASLDTY--RPNTAELFRASST----EKS------EKIPD--------TVDMDIRGIYSTQQDFGMTLRGAGGRLQNFGQ-----------QNNNPNGLTPKQEMLARSAIKYWVERHKHVVRLVASIGDTYGTALLFHMLVSTITLTLLAYQATKINGINVYAFSTIGYLSYTLGQVFHFCIFGNRLIEESSSVMEAAYSCQWYDGSEEAKTFVQIVCQQCQKAMSISGAKFFTVSLDLFASVLGAVVTYFMVLVQLK

>ALJ33155.1_ORCO_[Athetis_dissimilis]

-----MMTKVKAQGLVSDLMPNIKLMQAAGHFLFNYHSENA-GMSNLLRKVYASTHAILIVVNFACMGINMAQYSDEVNELTANTITVLFFTHTIIKLVFFALNSKSFYRTLAVWNQSNSHPLFTESDARYHQIALTKMRRLLYFICGMTCLAVVSWITLTFFGESVRLITNKE-TNETLTEVAPRLPLKAWYPFNAMSGTMYMIAFGFQVYWLLFSMAIANLMDVMFCSWLIFACEQLQHLKAIMKPLMELSASLDTY--RPNTAELFRASST----EKS------EKIPE--------AVDVDIRGIYSTQQDFGMTLRGAGGRLQNFGQ-----------QNANPNGLTPKQEMLARSAIKYWVERHKHVVRLVASIGDTYGTALLFHMLVSTITLTLLAYQATKINGINVYAFSTIGYLSYTLGQVFHFCIFGNRLIEESSSVMEAAYSCQWYDGSEEAKTFVQIVCQQCQKAMSISGAKFFTVSLDLFASVLGAVVTYFMVLVQLK

>BAJ23262.1_odorant_receptor_2,_partial_[Ostrinia_palustralis]

------------QGLVSDLMPNIKLMQAAGHFLFNYHSDNS-GMTTLLRKVYSSIHAFLIVINYLCMAANMAQYSDEVNELTANTITVLFFAHSVIKMLFFAVNSKSFYRTLAVWNQSNSHPLFTESDARYHQLALSKMRRLLYFICGVTVLAVISWITITFFGESVRMIANKE-TNETLTEPAPRLPLKTWYPFDAMSGTMYVVAFVYQVYWLFFSMAIANLMDVMFCSWLIFACEQLQHLKAIMKPLMELSASLDTY--RPNTAELFRASST----EKS------EKMPD--------TVDMDIRGIYSTQQDFGMTLRGAGGRLQNFGQP----------NPNNPNGLTQKQEMLARSAIKYWVERHKHVVRLVASIGNTYGTALLFHMLVSTITLTLLAYQATKINGINVYAFSTIGYLSYTLGQVFHFCIFGNRLIEESSSVMEAAYSCQWYDGSEEAKTFVQIVCQQCQKAMSISGAKFFTVSLDLFASVLGAV-----------

>BAG71415.1_olfactory_receptor-2_[Mythimna_separata]

-----MMTKVKAQGLVSDLMPNIKLMQAAGHFLFNYHSENA-GMSNLLRKIYASTHAILIIVHFACMGINMAQYSDEVNELTANTITVLFFTHTIIKLGFFALNSKSFYRTLAVWNQSNSHPLFTESDARYHQIALTKMRRLLYFICGMTCLSVVTWITLTFFGESVRMITSKE-TNETLTEVVPRLPLKAWYPFNAMSGTMYIVAFAFQVYWLLFSMAIANLMDVMFCSWLIFACEQLQHLKAIMKPLMELSASLDTY--RPNTAELFRASST----EKS------EKIPD--------AVDMDIRGIYSTQQDFGMTLRGAGGRLQNFGQ-----------QNANPNGLTPKQEMLARSAIKYWVERHKHVVRLVASIGDTYGTALLFHMLVSTITLTLLAYQATKINGINVYAFSTIGYLSYTLGQVFHFCIFGNRLIEESSSVMEAAYSCQWYDGSEEAKTFVQIVCQQCQKAMSISGAKFFTVSLDLFASVLGAVVTYFMVLVQLK

>AOE48007.1_putative_odorant_receptor_ORco_[Athetis_lepigone]

-----MMTKVKTQGLVSDLMPNIRLMQAAGHFLFNYHSENA-GMSNLLRKVYASTHAILIVINFACMGINMAQYSDEVNELTANTITVLFFTHTIIKLSFFALNSKSFYRTLAVWNQSNSHPLFTESDARYHQIALTKMRRLLYFICGMTCLAVVSWITLTFFGESVRLITNKE-TNETLTEVAPRLPLKAWYPFNAMSGTMYMIAFGFQVYWLLFSMAIANLMDVMFCSWLIFACEQLQHLKAIMKPLMELSASLDTY--RPNTAELFRASST----EKS------EKIPE--------AVDVDIRGIYSTQQDFGMTLRGAGGRLQNFGQ-----------QNANPNGLTPKQEMLARSAIKYWVERHKHVVRLVASIGDTYGTALLFHMLVSTITLTLLAYQATKINGINVYAFSTIGYLSYTLGQVFHFCIFGNRLIEESSSVMEAAYSCQWYDGSEEAKTFVQIVCQQCQKAMSISGAKFFTVSLDLFASVLGAVVTYFMVLVQLK

>AOG12930.1_odorant_receptor_[Eogystia_hippophaecolus]

-----MMAKVKAQGLVSDLMPNIKLMQMAGHFLFNYHSDNS-GMSTLLRKIYASVHAVFIVTQYFAMVANMAMYSDEVNELTANTITVLFFAHSIIKLIFFALNSKSFYRTLAIWNQSNSHPLFTESDARYHQLALTKMRRLLYFICGVTILSVFSWVTITFFGESVYMLVNKE-TNETLTEPAPRLPVKAWYPFNAMSGTMYIVAFVLQVYWLLIAMAIANLMDVMFCSWLIFACEQLQHLKAIMKPLMELSASLDTY--RPNTAELFKVSST----EKS------EKVPD--------PVDLDIRGIYSTQQDFGMTLRGAGGRLQTFGQ-----------NLNNPNGLTQKQEMLARSAIKYWVERHKHIVRLVASIGDTYGTDLLFHMLVSTITLTLLAYQATKINGLNVYAFSTLGYLGYTLGQVFHFCIFGNRLIEESSSVMEAAYSCQWYDGSEEAKTFVQIVCQQCQKAMSISGAKFFTVSLDLFASVLGAVVTYFMVLVQLK

>AJF23826.1_olfactory_receptor_Orco_[Planotortrix_octo]

-----MMGKVKTQGLVSDLMPNIKLMQAVGHFLFNYTDENG-GMSMLLRKIYASTHAVLIVVNFLCMAVNMAQYSDEVNELTANTITVLFFAHTVIKLLFFAMNSKNFYRTLAVWNQSNSHPLFTESDARYHQLALNKMRRLLYFIGSVTIMAVVSWITITFFGESVRLIADKE-SNDTLTEPAPRLPLKTWYPFNAMSGTMYIVAFVYQIYWLLFSMAIANLMDVMFCSWLIFACEQLQHLKAIMKPLMELSASLDTY--RPNTSELFRASST----EKS------EKVPE--------PVDMDIRGIYSTQQDFGMLLRGAGGRLQNFNNP----------NPNNPNGLTQKQEMLARSAIKYWVERHKHVVRLVASIGDTYGTALLFHMLVSTITLTLLAYQATKIDGLNVYAFSTVGYLSYTLGQVFHFCIFGNRLIEESSSVMEAAYSCQWYDGSEEAKTFVQIVCQQCQKAMSISGAKFFTVSLDLFASVLGAVVTYFMVLVQLK

>AET06156.1_odorant_receptor_2,_partial_[Planotortrix_excessana]

-----MMGKVKTQGLVSDLMPNIKLMQAVGHFLFNYTDENG-GMSMLLRKIYASTHAVLIVVNFLCMAVNMAQYSDEVNELTANTITVLFFAHTVIKLLFFALNSKNFYRTLAVWNQSNSHPLFTESDARYHQLALNKMRRLLYFIGSVTIMAVVSWITITFFGESVRLIADKE-SNDTLTEPAPRLPLKTWYPFNAMSGTMYIVAFVYQIYWLLFSMAIANLMDVMFCSWLIFACEQLQHLKAIMKPLMELSASLDTY--RPNTSELFRASST----EKS------EKVPE--------PVDMDIRGIYSTQQDFGMLLRGAGGRLQNFNNP----------NPNNPNGLTQKQEMLARSAIKYWVERHKHVVRLVASIGDTYGTALLFHMLVSTITLTLLAYQATKIDGLNVYAFSTVGYLSYTLGQVFHFCIFGNRLIEESSSVMEAAYSCQWYDGSEEAKTFVQIVCQQCQKAMSISGAKFFTVSLDLFASVLGAVVTYFMVLVQ--

>AFC91712.1_putative_odorant_receptor_co-receptor_ORco_[Cydia_pomonella]

-----MMGKVKSQGLVSDLMPNIKLMQMSGHFLFNYTEETG-GMSLLLRKIYAAMHAFLILLNFVCMGINMAQYSEEVNELTANTITVLFFAHTIIKLAFFAINSKSFYRTLAVWNQSNSHPLFTESDARYHQLSLDKSRRLLYFICGTTCLSVVSWVTLTFFGESVRLIADKE-SNDTLTEPAPRLPLKAWYPFDTMSGSMYIMAFVYQIYWLLFSMLIANLLDVMFCSWLIFACEQLQHLKAIMKPLMELSAALDTY--RPNTAELFRASST----EKS------EKVPE--------PTDIDIRGIYSTQQDFGMMLRGAGGRLQNFNS-----------TNPNPNGLTQKQEMLARSAIKYWVERHKHVVRLVASIGDTYGTALLFHMLVSTITLTLLAYQATKIDGLNVYAFSTVGYLRYTLGQVFHFCIFGNRLIEESSSVMEAAYSCQWYDGSEEAKTFVQIVCQQCQKAMSISGAKFFTVSLDLFASVLGAVVTYFMVLVQLK

>ACJ12928.2_odorant_receptor_2_[Epiphyas_postvittana]

------MGKVKTQGLVSDLMPNIKLMQTVGHFLFNYSDETG-GMSMLLRKVYASTHAVLIVINFLCMAVNMAQYSDEVNELTANTITVLFFAHTVIKLLFFALNSKNFYRTLAVWNQSNSHPLFTESDARYHQLALNKMRRLLYFIGTVTVMAVVSWITITFFGESVRLIADKE-SNDTLTEPAPRLPLKAWYPFNAMSGTMYIVAFVYQIYWLLFSMAIANLMDVMFCSWLIFACEQLQHLKAIMKPLMELSASLDTY--RPNSSELFRASST----EKS------EKVPD--------PVDLDIRGIYSTQQDFGMMLRGAGGRLQNFNNP----------NPNNPNGLTQKQEMLARSAIKYWVERHKHVVRLVASIGDTYGTALLFHMLVSTITLTLLAYQATKIDGLNVYAFSTIGYLSYTLGQVFHFCIFGNRLIEESSSVMEAAYSCQWYDGSEEAKTFVQIVCQQCQKAMSISGAKFFTVSLDLFASVLGAVVTYFMVLVQLK

>AET06159.1_odorant_receptor_2,_partial_[Planotortrix_notophaea]

-----MMGKVKTQGLVSDLMPNIKLMQAVGHFLFNYTDENG-GMSMLLRKVYASTHAVLIVVNFLCMAVNMAQYSDEVNELTANTITVLFFAHTVIKLLFFALNSKNFYRTLAVWNQSNSHPLFTESDARYHQLALNKMRRLLYFIGTVTIMAVVSWITVTFFGESVRLIADKE-SNDTLTEPAPRLPLKTWYPFNAMSGTMYIVAFVYQIYWLLFSMAIANLMDVMFCSWLIFACEQLQHLKAIMKPLMELSASLDTY--RPNTSELFRASST----EKS------EKVPE--------PVDMDIRGIYSTQQDFGMMLRGAGGRLQNFNNP----------NPNNPNGLTQKQEMLARSAIKYWVERHKHVVRLVASIGDTYGTALLFRMLVSTITLTLLAYQATKIDGLNVYAFSTVGYLSYTLGQVFHFCIFGNRLIEESSSVMEAAYPCQWYDGSEEAKTFVQIVCQQCQKAMSISGAKFFTVSLDLFASVLGAVVTYFMVLVQ--

>AIT69913.1_olfactory_receptor_co_[Ctenopseustis_herana]

-----MMGKVKTQGLVSDLMPNIKLMQAVGHFLFNYTDESG-GMSMLLRKIYASTHAVLIVVNFLCMAVNMAQYSDEVNELTANTITVLFFAHTVIKLLFFALNAKNFYRTLAVWNQSNSHPLFTESDARYHQLSLNKMRRLLYFIGSVTIAAVVCWITITFFGESVRLIADKE-SNDTLTEPAPRLPLKAWYPFNAMSGTMYIVAFVYQIYWLLFSMAIANLMDIMFCSWLIFACEQLQHLKAIMKPLMELSASLDTY--RPNTSELFRASST----EKS------EKVPE--------PVDMDIRGIYSTQQDFGMMLRGAGGRLQNFNNS----------NPGNPNGLTQKQEMLARSAIKYWVERHKHVVRLVASIGDTFGTALLFHMLVSTITLTLLAYQATKIDGLNVYAFSTVGXLSYTLGQVFHFCIFGNRLIEESSSVMEAAYSCQWYDGSEEAKTFVQIVCQQCQKAMSISGAKFFTVSLDLFASVLGAVVTYFMVLVQLK

>AIT72022.1_olfactory_receptor_[Ctenopseustis_obliquana]

-----MMGKVKTQGLVSDLMPNIKLMQAVGHFLFNYTDESG-GMSMLLRKIYASTHAVLIVVNFLCMAVNMAQYSDEVNELTANTITVLFFAHTVIKLLFFALNAKNFYRTLAVWNQSNSHPLFTESDARYHQLSLNKMRRLLYFIGSVTIAAVVCWITITFFGESVRLIADKE-SNDTLTEPAPRLPLKAWYPFNAMSGTMYIVAFVYQIYWLLFSMAIANLMDIMFCSWLIFACEQLQHLKAIMKPLMELSASLDTY--RPNTSELFRASST----EKS------EKVPE--------PVDMDIRGIYSTQQDFGMMLRGAGGRLQNFNNS----------NPSNPNGLTQKQEMLARSAIKYWVERHKHVVRLVASIGDTFGTALLFHMLVSTITLTLLAYQATKIDGLNVYAFSTVGYLSYTLGQVFHFCIFGNRLIEESSSVMEAAYSCQWYDGSEEAKTFVQIVCQQCQKAMSISGAKFFTVSLDLFASVLGAVVTYFMVLVQLK

>AII01079.1_odorant_receptor_[Dendrolimus_kikuchii]

-----MMTKVKTQGLVSDLMPCIKLMQIVGHFLFNYYDENT-GMSALVRKIYAGTHAFIIVVHFLFMGINMAKYSDEVNELTANTITMLFFTHSLIKLLFFALNSKSFYRTLAAWNQCNSHPLFVESDARYHQLALSRMRRLLYFVGGMTIVSVISWVILTFFGESVRYITSKE-TNETLTEPAPRLPLKAWYPFNAMSGSMYVIAFVLQIYWLLFAMSIANLMDVMFCSWLIFACEQLQHLKAIMQPLMELSAALDTY--RPNTAELFRVNSG----G--------EKVAD--------ATDLDIRGIYSTQQDFGMAIRGAGGRLQNFAQ-----------QKANPNGLSQKQEMLARSAIKYWVERHKHVVRLVTSIGDTYGVALLFHMLVSTITLTLLAYQATKINGVNVYAFSTIGYLSYTLGQVFHFCIFGNRLIEESSSVMEAAYSCQWYDGSEEAKTFVQIVCQQCQKAMSISGAKFFTVSLDLFASVLGAVVTYFMVLVQLK

>AII01046.1_odorant_receptor_[Dendrolimus_houi]

-----MMNKTKTQGLVSDLMPCIKLMQAVGHFLFNYYDENT-GMSALIRKIYAGTHAFIIVVHFLFMGINMAKYSDEVNELTANTITMLFFTHSLIKLLFFALNSKSFYRTLAAWNQCNSHPLFVESDARYHQLALSRMRRLLYFVGGMTIVSVMSWVILTFFGESVRYITSKE-TNETLTEPAPRLPLKAWYPFNAMSGSMYVIAFVLQIYWLLFAMSIANLMDVMFCSWLIFACEQLQHLKAIMQPLMELSAALDTY--RPNTAELFRVNSG----G--------EKVPD--------ATDLDIRGIYSTQQDFGMAIRGAGGRLQNFAQ-----------QKANPNGLSQKQEMLARSAIKYWVERHKHVVRLVTSIGDTYGVALLFHMLVSTITLTLLAYQATKINGVNVYAFSTIGYLSYTLGQVFHFCIFGNRLIEESSSVMEAAYSCQWYDGSEEAKTFVQIVCQQCQKAMSISGAKFFTVSLDLFASVLGAVVTYFMVLVQLK

>AJF20962.1_olfactory_coreceptor_[Operophtera_brumata]

-----MMTKVKYQGLVSDLLPNIKLMQAAGHFLFNYHDENA-GMSTILRKIYSSTHAFLISIHFVLMVINMAKYADEVNELTANTITILFFLHTIIKMLFFAVNSKSFYRTLAVWNQSNSHPLFTESDARYHQLALTKMRRLLYFICVMTVVSLICWVTLTFFGESVRLISSKD-TNETLTEEIPRLPLKAWYPFDAMSGSMYIFAFVFQIYWLLFSTSISNFMDVMFCSWLIFACEQLQHLKGIMKPLMELSASLDTY--RPNTAELFRASST----EKS------EKVPD--------PVDIDIRGIYSTQQDFGMTLRGAGGRLQTFGE------------NNNPNNLTQKQEMLARSAIKYWVERHKHVVRLVGSIGDTYGTALLFHMLVSTITLTLLAYQATKINGVNVYAFSTLGYLCYTLGQVFHFCIFGNRLIEESSSVMEAAYSCQWYDGSEEAKTFVQIVCQQCQKALSISGAKFFTVSLDLFASVLGAVVTYFMVLVQLK

>ANW12106.1_olfactory_receptor_protein_1_[Phenacoccus_solenopsis]

------MQKLRKQGLVADLWPNIRLMQLSGLFITQYYEDNS-TLMRLIRKIYSWITAILVFTQYILLVIWALTESYDADQRAAYSVTILFFTHPLIKFIYFSTKTNRFYRTLSAWNNANSHPLFAESNARHRASTLARMRKLLMYVGSITIFATVAWTVITFIGESVRTVPDPESENGTITIEAPRLMVPAWYPWDVMGGLTYYLTLVYQFYWLFITMSHANLCDILFCSLVLHSCEQLKHLKEIMGPLIELSAALDTQV--PNAEALFRVPSS---GSKAPLMENEEYD---------------------NYNTNYNNYRN--NALSTVAGG----------GGSGPNGLTKKQEILVRSAIKYWVERHKHIVRYVASVSEMYGMALLFHMLITTFTLTLLAYQATKIDGVNVYAFSTIGYLVYTLGQIYTFCIYGNELIDESSSVMEAAYSCSWYDGSEEAKTFVQIVCQQCQKALSVTGAKFFTVSLDLFASVLGAVVTYFMVLIQLN

>AJO62219.1_olfactory_co-receptor_ORco_[Tenebrio_molitor]

------MMKFKVSGLVADLMPNIRLIQASGHFMLNYHADNS-GAVHTLRLGYCIMHLIFMLLQYGCNFVNLIFERGDVNDLAANTITVLFFTHCITKFVYFAARSKLFYRTLGIWNQPNSHPLFVESNNRYHALALKKMRRLLYIIIIWTSFSAIAWTSITFVGDSVHNIKDPDNENMTITEEIPRLLVKAWYPWNAMSGMPYYITLVFQVYYVFFALSHANLLDSLFCSWLIFACEQLQHLKEIMKPLMELSASLDTYV--PKSADLFRAPSA----TSQDNLIENDYN----------TKNEDLKGVYSTRQELGGHFRG--GALQNFGGVG---------GGVGPNGLTKKQELMVRSAIKYWVERHKHVVRLVTAIGDAYGVALLLHMLTSTIMLTLLAYQATKITGVDKYAATVIGYLLFALAQVFHFCIFGNRLIEESSSVMEAAYSCHWYDGSEEAKTFVQIVCQQCQKAMSISGAKFFTISLDLFASVLGAVVTYFMVLVQLK

>XP_008194693.1_PREDICTED:_odorant_receptor_coreceptor_[Tribolium_castaneum]_

------MMKFKVTGLVADLMPNIRLIQASGHFMLNYHADNS-GALHTLRLGYCCMHLVFVLVQYGCNFVNLVLERGDVNDLAANTITVLFFTHCVTKFVYFAVRSKLFYRTLGIWNQPNSHPLFVESNNRYHGIALKKMRRLLYIIIIWTSFSAIAWTGITFVGDSVHNIKDPENENLTITEPIPRLLVKAWYPWDAMSGMPYYITLVFQIYYVFFSLAHANLLDSLFCSWLIFACEQLQHLKEIMKPLMELSATLDTYV--PKSADLFRAPSA----TSQDQLIENDYN----------EKNEDLKGVYSTRQELGGHFRG--GALQNFGS-----------GGVGPNGLTKKQELMVRSAIKYWVERHKHVVRLVTAIGDAYGVALLLHMLTSTIMLTLLAYQATKITGVDKYAATVLGYLLFALAQVFHFCIFGNRLIEESSSVMEAAYSCHWYDGSEEAKTFVQIVCQQCQKAMSISGAKFFTISLDLFASVLGAVVTYFMVLVQLK

>ADM35103.1_olfactory_receptor_Or83b_[Holotrichia_plumbea]

------MMQFKPQGLVADLMPNINLMKFAGHFMLNYYSDNG-GALHTLRLGFCFGHLFLMLVQFGFTFGNLVQQSDDVNDLAANTITVLFFTHCIVKFIYFGVRQKLFYRTLGIWNQSNSHPLFLESNNRYHQLALTKMRRLLIVVMIGTIGSWIAWTTITFFGDSVHTTKDPNNENETITEEVPRLLIRAWYPWDAMAGIPYYISLVYQIYYVGFSMLHSNLLDSLFCSWLIFASEQLQHLKEIMKPLMELSATLDTYV--PKSADLFRAPSA----SSQDKLTESDYNAR--------NEDAHMRAMYSTHQEMGVTYRS--GQLQDFSS-----------GGIGPNGLTKKQELMVRSAIKYWVERHKHVVRLVTAIGDAYGIALLLHMLTSTITLTLLAYQATKIDGVNKYALTVLGYLFYALAQVFHFCIFGNRLIEESSSVMEAAYSCHWYDGSEEAKTFVQIVCQQCQKAMSISGAKFFTISLDLFASVLGATVTHFMVLVQLK

>AOO35284.1_olfactory_co-receptor_[Rhynchophorus_vulneratus]

------MNTFKVAGLVADLMPNIRLIQASGHFMLNYHADNS-GALHGLRLGYCCMHLLFVLLQFGCIFGNLVKEKDNVNDLAANTITILFFTHCLTKFVYFAVRSKLFYRTLGIWNQANSHPIFIESNNRYHVLALKKMRNLLYIIMIGTIFSASAWTGITFMGDSVHYIKDPNNENETISEEIPRLLIKSWYPFDAMSGMPYYIALVFQVYYVLFSLLHANLLDSLFCSWLIFACEQLQHLKEIMKPLMELSASLDTYV--PKSADLFKAPNSA---SSQDNLIENEYNSK--------NDELNLKGVYSTRQELGNLTFRS-GALQTFGQGG---------GGVGPNGLTKKQELMVRSAIKYWVERHKHVVRLVTAIGNAYGVALLLHMLTATIMLTLLAYEATKIDGVNVYAATTIGYLLYSLAQVFHFCIFGNRLIEESSSVMEAAYSCHWYDGSEEAKTFVQIVCQQCQKALSISGAKFFTISLDLFASVLGAVVTYFMVLVQLK

>XP_019768125.1_PREDICTED:_odorant_receptor_coreceptor_[Dendroctonus_ponderosae]

-----MINKFKVVGLVADLMPNIRLIQASGHFMFNYYADNS-GSLHILRLGYCCMHLFFVLVQYGCIFGNLVKEKDNVSHLAANTITILFFTHCLSKFIYFAARSKLFYRTLGIWNQANSHPIFLESSNRYHALALKKMRSLLYIILFGTIFSASAWTAITFVGESVHFIKDPDNDNETITEEIPRLLIKSWYPFDAMSGMTYYVALVFQIYYVFFSLFQANLLDNLFCSWLIFACEQLQHLKEIMKPLMELSATLDTFV--PKSADLFKSPGSA---TSQDHLIENDFNAK--------ND--DLKGVYSTRQELGNLNFRS-GALQTFGQGG---------GGVGPNGLTKKQELMVRSAIKYWVERHKHVVRLVTAIGDAYGVALLLHMLTATVMLTLLAYEATKIDGLNTYAATTLGYLLYSLAQVFHFCIFGNRLIEESSSVMEAAYSCHWYDGSEEAKTFVQIVCQQCQKSLFISGAKFFTISLDLFASVLGATVTYFMVLVQLK

>AJF94638.2_odorant_receptor_co-receptor_[Ambrostoma_quadriimpressum]

------MMKFKVSGLVADLMPNIRLIQASGHFMFNYHADNS-GALHALRLGYSCLHLVLCLVQFGCTFGNLVIERNDVNDLAANTITVLFFTHCITKFVYFAVRSKLFYRTLGIWNKANSHPLFLDSNNRYHALSLKKMRTLLICVMTTTILSASAWTAITFVGDSVHNVKDPDNDNETITEEIPRLLIKSWYPWNAMSGTAYYVSVSFQIYYVFFSLAHSNLMDSLFCSWLIFACEQLQHLKEIMKPLMELSASLDTYV--PKSADLFRAPSA----NSQDNLIENEYNEK--------NEGLNLKGVYNTRQEMGANFRSG--ALQTFGQGG---------GGVGPNGLSKKQELMVRSAIKYWVERHKHVVRLVTAIGDAYGVALLLHMLTATVMLTLLAYQATKIDGVNKYAATVIGYLVYSLAQVFHFCIFGNRLIEESSSVMEAAYSCHWYDGSEEAKTFAQIICQQCQKALSISGAKFFTISLDLFASVLGAVVTYFMVLVQLK

>ALR72547.1_odorant_receptor_ORco_[Colaphellus_bowringi]

------MMKFKVSGLVADLMPNIRLIQASGHFMFNYHADNS-GALHALRLGYSCMHLVFCLFQFGCTFGNLVVERDNVNDLAANTITVLFFTHCITKFVYFAVRSKLFYRTLGIWNQANSHPLFVESNNRYHALALKKMRTLLVCVMATTVLSASAWTGITFVGDSIHHIKDPDNENETIIEEIPRLLVKSWYPWDAMSGTAYYASLIFQIYYVFFSLAHANLMDSLFCSWLIFACEQLQHLKEIMKPLMELSASLDTYV--PKSADLFRAPSA----NSQDNLIENDYNAK--------NEEINLKGIYNTRQELGINFRSG--ALQTFGQGG---------GGVGPNGLSKKQELMVRSAIKYWVERHKHVVRLVTAIGDAYGVALLLHMLTSTVMLTLLAYQATQIGGVNKYAATVIGYLVYSLAQVFHFCIFGNRLIEESSSVMEAAYSCHWYDGSEEAKTFVQIVCQQCQKAMSISGAKFFTISLDLFASVLGAVVTYFMVLVQLK

>XP_017785109.1_PREDICTED:_odorant_receptor_coreceptor_[Nicrophorus_vespilloides]

------MMEFKTQGLVTDLMPNIRLMQASGHFLFNYHADNS-GALHTLRIFYSCVHLILILLQFGCIFGNLVQEADDVNDLAANTITILFFTHCVTKFVYFAIRSKLFYRTLGIWNSPNSHPLFVESNNRYHSISLTKMRRVLMVVLATTLISAVSWTTLTFIGDSTHTKKDPNNENETITEEIPRLLVKAWYPWNAMSGMPYMLSLVYQVYYVLFSMLQSNLMDVLFCSWLIFACEQLKHLKAIMKPLMELSATLDTYV--PKSADLFRANSA----KSQDNLIENDYN----------NMKQDYKGVYNTQQEMGVNYRSG--ALQTFGPGG-------MNGGIGPNGLTKKQEMMVRSAIKYWVERHKHVVRLVTAIGDAYGVALLFHMLTSTITLTLLAYQATKIDGVTKYAASVIGYLLYALAQVFLFCIFGNQLIEESSSVMEAAYSCHWYDGSEEAKTFVQIVCQQCQKALSISGAKFFTISLDLFASVLGATVTYFMVLVQLK

>ACD40044.1_odorant_receptor_[Phyllotreta_striolata]

-----MMKKVKVTGLVADLMPNIRLMQASGHFMFNYHADNS-GVSHLLRKVYSCMHLVLVLIQYACILVNLALNPDDVNELTANTITVLFFTHCITKFVYFAVNVEFFYRTIAVWNQPNSHPLFVESDARYHCIGVMKMRRLLFLVNMSTSLDTIAWTTVTFFRESVANLQDNDTENGTINAHVPRLPIKAFYPWNAESGITYYVTLFFQINCFYFSTEIRNKLVPRFCSWLIFACEQLHHLQAIMKPLMELTASMDTYV--PNSAILFRAPSA----TSHEQVIDNNQ--K--------NEELDLKGVYNTRQEMGANFRSG--ALQNFGQGG---------GGVGPNGLTKKQELMVRSAIKYWVERHKHVVRLVTAIGDAYGVALLLHMLTSTVMLTLLAYQATKINGVDTYAATVIGYLVYALAQVFHFFIFGNRLIEESSSVMEAAYSCHWYDGSEEAKTFVQIVCQQCQKAMSISGAKFFTISLDLFASVLGATVTYFMVLVQLK

>XP_018568191.1_PREDICTED:_odorant_receptor_coreceptor_[Anoplophora_glabripennis]

------MMKFKVSGLVADLMPNIRLIQASGHFMFNYHADNS-GALHALRLGYSCAHLLFCLFQYGCIFGNLVVEKDDVNYLAANTITVLFFTHCITKFVYFALRSKLFYRTLGIWNQSNSHPLFVESNNRYHALALKKMRTLLICVTATTVLSAAAWTGITFVEESVHNIKDPDNENETITEEIPRLLIKSWYPWDAMSGMAYYGSLIFQIYYVLFSLAHANLMDSLFCSWLIFACEQLQHLKEIMKPLMELSASLDTYV--PKSADLFRAPSA----KSQDNYIENDYNAK--------NEELNLKGIYNTRQELGGNFRSG--ALQTFGQ-----------GGVGPNGLTKKQELMVRSAIKYWVERHKHVVRLVTAIGDAYGVALLLHMLTSTVMLTLLAYQATKINGVNTYAATTIGYLVYSLAQVFHFCIFGNRLIEESSSVMEAAYSCHWYDGSEEAKTFVQIVCQQCQKAMQISGAKFFTISLDLFASVLGAVVTYFMVLVQLK

>AIX97139.1_olfactory_receptor_4_[Rhyzopertha_dominica]

------MMTFKVQGLVADLMPNIRLMQAVGHFMLNYHADNS-GALHTLRLSYCFMHLFLLLTQYGFIFGNLVKERGDVNDLAANTITVLFFMHCITKFVYFAIRSKLFYRTLGIWNQSNSHPLFVESNNRFHAIALTKMRRVTIVVVAGTLVSAISWISVTFVGDSVHHIKDPNNVNETITEEIPRLPIKTWYPWNAMSGMPYYLSLGYQIYYVLFSMFHANLLDVLFCCWLIFACEQLMHLKEIMKPLMELSASLDTYH--PKSADLFRAVSA----NSQDNLVDHDYNTK--------NSEEDLKGYYNTHQELGGHFRSG--ALQMFGQGG---------GGIGPNGLTKKQELLVRSAIKYWVERHKHVVRLVTAIGDAYGIALLLHMLTSTIMLTLLAYQATKIDGVNTYAASVLGYLFYALAQVFLFCIFGNRLIEESSSVMEAAYSCHWYDGSEEAKTFVQIVCQQCQKAMSISGAKFFTISLDLFASVLGAVVTYFMVLVQLK

>XP_019869252.1_PREDICTED:_LOW_QUALITY_PROTEIN:_odorant_receptor_coreceptor-like_[Aethina_tumida]

------MMKFKVAGLVADLMPNIRLMQGAGHFMLNYHADNS-GALHTLRLGYCCVHLVLMLFQYGTILGNLVVDREDVNELAANTITTLFFAHCITKFIYFALRSKLFYRTLGIWNQANSHPIFLESNNRYHALALKKMRRLLVIIVCATLFSGIAWTAITFVGDSVKSKKDPENENETITVEIPRLLIKSWYPWDAMSGTAYYATLVFQVYYVFFALAQANLLDSLFCSWLIFACEQLQHLKEIMKPLMELSATLDTYV--PKSADLFRAPSA-----NND-IMENEYNDK--------NEDLNLKGIYSTRQELGGHFRSG--ALQTFGQGG---------GGVGPNGLTKKQELMVRSAIKYWVERHKHVVRLVTAIGDAYGVALLLHMLTSTVMLTLLAYQATKIDGVNTYAASTLGYLLYALGQVFHFCIFGNRLIEESSSVMEAAYSCHWYDGSEEAKTFVQIVCQQCQKAMSISGAKFFTISLDLFASVLGAVVTYFMVLVQLK

>AIX97092.1_olfactory_receptor_1_[Monochamus_alternatus]

--------------------------------MFNYHADNS-GALHTLRLLYSCMHLVFCLFQFGCIFGNLVVEKDDVNYLAANTITVLFFTHCITKFVYFALRSKLFYRTLGIWNQSNSHPLFVESNNRYHALSLKKMRTLLICVSATTVLSAAAWTGITFVEESVHNIKDPNNENETITEEIPRLLIKSWYPWDAMSGMAYYGSLVFQIYYVLFSLTHANLMDSLFCSWLIFACEQLQHLKEIMKPLMELSASLDTYV--PKSADLFRAPSA----KSQDNYIESDYNTK--------NEELNLKGIYNTRQELGGNFRSG--ALQTFGQ-----------GGVGPNGLTKKQELMVRSAIKYWVERHKHVVRLVTAIGDAYGVALLLHMLTSTVMLTLLAYQATKINGVNTYAATTIGYLVYSLAQVFHFCIFGNRLIEESSSVMEAAYSCHWYDGSEEAKTFVQIVCQQCQKAMQISGAKFFTISLDLFASVLGAVVTYFMVLVQLK

>AOO35283.1_olfactory_co-receptor_[Rhynchophorus_ferrugineus]

------MNTFKVAGLVADLMPNIRLIQASGHFMLNYHADNS-GALHGLRLGYCCMHLLFVLLQFGCIFGNLVKEKDNVNDLAANTITILFFTHCLTKFVYFAVRSKLFYRTLGIWNQANSHPIFIESNNRYHALALKKMRNLLYIIMIGTIFSASAWTGITFMGDSVHYIKDPNNENETISEEIPRLLIKSWYPFDAMSGMPYYIALVFQVYYVLFSLLHANLLDSLFCSWLIFACEQLQHLKEIMKPLMELSASLDTYV--PKSADLFKAPNSA---SSQDNLIENEYNSK--------NDELNLKGVYSTRQELGNLTFR-SGALQTFGQGG---------GGVGPNGLTKKQELMVRSAIKYWVERHKHVVRLVTAIGDAYGVALLLHMLTATIMLTLLAYEATKIDGVNVYAATTIGYLLYSLAQVFHFCIFGNRLIEESSSVMEAAYSCHWYDGSEEAKTFVQIVCQQCQKALSISGAKFFTISLDLFASVLGAVVTYFMVLVQLK

>AEG88961.1_odorant_receptor_Or83b_[Holotrichia_parallela]

------MMQFKPQGLVADLMPNIKLMKFAGHFMLNYYAENS-GAVHTLRLGFCFGHLFLMLLQFGFTFGNLVQESDDVNDLAANTITILFFTHCIVKFIYFGVRQKLFYRTLGIWNQSNSHPLFLESNNRYHQLALTKMRRLLIIVMVGTIGSWIAWTTITFLGDSVHTRKDPSNENETITEEIPRLLVRSWYPWDAMSGIPYYITLVYQVYYVGFSMLHSNLLDSLFCSWLIFACEQLQHLKEIMKPLMELSATLDTYV--PKSADLFRAPSA----SSQDRLMDSDYNAR--------NEDVHMKTMYSTHHEMGVTYRSGQ--LQDFS------------GGIGPNGLTKKQELMVRSAIKYWVERHKHVVRLVTAIGDAYGIALLLHTSASTITLTLLAYQATKIDGVNKYALTVLGYLFYALTQVFHFCIFGNRLIEESSSVMEAAYSCHWYDGSEEAKTFVQIVCQQCQKAMSISGAKFFTISLVLFASVLGATVTYFMVLVQLK

>AEE69033.1_olfactory_receptor_Or83b_[Holotrichia_oblita]

------MMKFKPQGLVADLMPNIKLMKFAGHFMLNYYAENS-GAVHTLRLGFCFGHLFLMLLQFGFTFGNLVQESDDVNDLAANTITVLFFTHCIVKFIYFGVRQKLFYRTLGIWNQSNSHPLFLESNNRYHQLALTKMRRLLIIVMIGTIGSWIAWTTITFFGDSVHNRKDPNNENETITEEIPRLLIRSWYPWDAMSGIPYYVSLIYQIYYVGFSMLHSNLLDSLFCSWLIFACEQLQHLKEIMKPLMELSATLDTYV--PKSADLFRAHSA----SSQDKLTESDYNAR--------NEDAHMRAMYSTHQEMGVTYRSGQ--LQEFSS-----------GGIGPNALTKKQELMVRSAIKYWVERHKHVVRLVTAIGDAYGIALLLHMLTSTITLTLLAYQATKIDGVNKYALTVLGYLFYALAQVFHFCIFGNRLIEESSSVMEAAYSCHWYDGSEEVKTFVQIVCQQCQKAMSISGAKFFTISLDLFASVLGATVTYFMVLVQLK

>AKC58535.1_odorant_co-receptor_[Anomala_corpulenta]

------MMQFKPQGLVADLIPNIKLMQFSGHFMLNYYAETT-GAVHTLRLGFCFGHLFLLLLQFGFTFGNLVQQSDDVNDLAANTITVLFFTHCITKFVYFAVRQKLFYRTLGIWNQSNSHPLFLESNNRYHQLALTKMRRLLIVIMIGTIGSWIAWTTITFFGDSVHTRKDPNNENETITEEVPRLLVRSWYPWDAMSGAAYYVSLVYQIYYVGFSMLHSNLLDSLFCSWLIFACEQLQHLKEIMKPLMELSATLDTYV--PKSADLFRAPSA----SSQDNLVDSDYNQS--------NEDANLRNLYTTHQEMGVTYRSGN--LQEFSS-----------GGIGPNGLSKKQELMVRSAIKYWVERHKHVVRLVTAIGDAYGIALLLHMLTSTIMLTLLAYQATKIDGVNKYALTVIGYLLYALAQVFHFCIFGNRLIEESSSVMEAAYSCHWYDGSEEAKTFVQIVCQQCQKAMSISGAKFFTISLDLFASVLGATVTYFMVLVQLK

>XP_018916513.1_PREDICTED:_odorant_receptor_coreceptor_[Bemisia_tabaci]

------MLEKCRRGLVQDLLPNIRLIHASGIYLSGYYDESS-SALDTLRGIYSWGTFTLMLFQYLLLVFHTATMSYTTEQLTASSITCLFFAHSIVKFIYFSVKGKSVCRVLDSWNQENSHPLFVESNKRHKVRALYRMRKLLYMIIGGSFFTYFAWILLTIAEEPYRMMPDPENRNNTVLTRVPKLLIQTWFPWNYESGVGYYVALGYQLYWLFMMIAHENLPDTLFCSTVIYACEQLKHLKEILKPLIEMSSNYDNGV--PPKPNILRMRSG----DSDMPLIDDEKVPE----------ENYQPVYNLIREFGPVYQRKNIHNMQN-----------------SFDALNDKDGNFVANAIKYWVERHKHVVRFVNDIGSMYGIALLLHMLISTVTLTLLAYQATQITGINVFGMSVIGYLGYSFIQVYAFCIYGNELIEESSSVMEAAYDCRWYDGSEEAKTFVQIVCQQCQKSLSISGAKFFTVSLDLFASVLGAVVTYFMVLVQLN

>AEX28371.1_olfactory_co-receptor,_partial_[Schistocerca_gregaria]

--------MQKPHGLVADLWPLIRMVQYSGHWMLEY---SG-G-LTALRAIYSSVVSVLVVTQFALMAVNPIQRSGDVNELAANTITVLFFLHPITKFAYFAVRSKAFYRTLATWNQSNNHPLFAESQARFHQLSVVRMRRLVMYVVSVTALSVVSWTSITFMGDSTREVADPDNANETITEEVPRLMISTWYPFDASSGMGYMLAFVYQLYWLTATLMHSNLMDVMFCCWLIYACEQLVYLKEIMKPLMELSATLDTVV--PHTSELFRAAST-------LPTN-EPLYGMGPDMSNGVTDGMTIRGIYSSQRDFSGFNRRS-AALSTVREADSGGAVTSA-GGIGPNGLSKRQEMLVRSAIKYWVERHKHVVRFVGNIGDAYGAALLLHMLTTTVTLTLLAYQATKIDSVDVYAASVLGYLFYTLGQVFLFCVFGNRLIEESSSVMEAAYSCHWYDGSEEAKTFVQIVCQQCQ------------------------------------

>ALD51504.1_odorant_receptor_co_[Locusta_migratoria]

--------MQKPHGLVADLWPLIRMVQYSGHWMLEY---SG-G--KALRAIYSSAVSLLVVTQFALMAVNLIQRSGDVNELAANTITVLFFLHPVTKFGYFAVRSKAFYRTLATWNQSNSHPLFAESQARFHQLSVVRMRRLVMYVVSVTALSVVSWTSITFMGDSTREVTDPDNANETITEEVPRLMISTWYPFDASSGMGYMLAFVYQLYWLTATLMHSNLMDVMFCCWLIYACEQLVHLKEIMKPLMELSATLDTVV--PHTSELFRAAST-------LPTN-EPLY----DAGNGAADGLTIRGIYSSQRDFSGFNRRSAA-LSTVREADAGGAVSSA-GGIGPNGLSKRQEMLVRSAIKYWVERHKHVVRFVGNIGDAYGAALLLHMLTTTVTLTLLAYQATKIDSVDVYAASVLGYLFYTLGQVFLFCVFGNRLIEESSSVMEAAYSCHWYDGSEEAKTFVQIVCQQCQKSLMISGAKFFTVSLDLFASVLGAVVTYFMVLVQLK

>KDR12002.1_Gustatory_and_odorant_receptor_7_[Zootermopsis_nevadensis]

------MYKFRLHGLVADMWPLIRIMQMTGFFLLDYHEDMS-FGWTSIRAGYAGSISGLMVIQFALIFINLMKQSDDVNDLTANTITVLFFVHSLVKFFYFALRRGKFYRTLATWNNANSHPLFSENHSRHHATAVGSMRRLVMYVGIGIIISGFAWTIITFVGDSVHEIPDPENANETIFEEVPRLMLRSWYPWNALSGGGYVVSFVIQILWLFLALSHAMMMDTMFCCWLIYTCQQLVHLKEIMKPLMELSASLDTLV--PHSAELFRAVSA----TTNNPITS--------------GDGDGIRAIYSNQHDF-SNFRLNTGALANINS-----------GSVGPNGLTKKQEVLVRSAIKYWVERHKHVVRFVSNIGDTYGSALLLHMLISTVTLTLLAYQATKIEAANVYACTVIGYLVYTLAQVFLFCFFGNRLIEESSSVMEAAYSCQWYDGSEEAKTFIQIVCQQCQKAMSISGAKFFTVSLDLFASVLGAVVTYFMVLVQLN

>ACT37280.1_atypical_seven-span_transmembrane_receptor_[Sitobion_avenae]

-------MGYKKDGLIKDLWPNIRLIQMSGLFISEYYEDYS-GLAVLFRKIYSWITTIIIYSQFIFIVMFMVTKSYDSDQLAAGVVTTLFFTHSMIKFMYFSTGTKSFYRTLSCWNNTSPHPLFAESHSRFHAKSLSRMRQLLIIVSIVTIFTTISWTTITFFGESVWKVPNPETFNQTMYVPVPRLMLHSWYPWDSSHGLGYIVAFVLQFYWIFITLSHSNLMELLFSSFLVHACEQLQHLKEILNPLIELSATLDSSVHNP--AEIFRASSA-----KNQSIN---------------GIDRDYNGSFVNEITEYGTKGENET------------------NRKGPNNLTSNQEVLVRSAIKYWVERHKHVVKYVSLITECYGSALLFHMLVSTVILTILAYQATKINGVNVFAFSTIGYLMYSFAQIFMFCIHGNELIEESSSVMEAAYGCHWYDGSEEAKTFVQIVCQQCQKPLIVSGAKFFNVSLDLFASVLGAVVTYFMVLVQLK

>XP_001951646.2_PREDICTED:_odorant_receptor_coreceptor_isoform_X1_[Acyrthosiphon_pisum]

MYTFSTNMGYKKDGLIKDLWPNIRLIQLSGLFISEYYDDYS-GLAVLFRKIYSWITAIIIYSQFIFIVIFMVTKSNDSDQLAAGVVTTLFFTHSMIKFVYFSTGTKSFYRTLSCWNNTSPHPLFAESHSRFHAKSLSRMRQLLIIVSIVTIFTTISWTTITFFGESVWKVPDPETFNQTMYVPVPRLMLHSWYPWDSSHGLGYIVAFVLQFYWIFITLSHSNLMELLFSSFLVHACEQLQHLKEILNPLIELSATLDSSVHNP--AEIFRANSA-----KNQSIN---------------GIDHDYNGSYVNEITEYGTKGENEP------------------NRKGPNNLTSNQEVLVRSAIKYWVERHKHVVKYVSLITECYGSALLFHMLVSTVILTILAYQATKINGVNVFAFSTIGYLMYSFAQIFMFCIHGNELIEESSSVMEAAYGCHWYDGSEEAKTFVQIVCQQCQKPLIVSGAKFFNVSLDLFASVLGAVVTYFMVLVQLK

>ALX17413.1_Orco_[Pediculus_humanus]

------MGKYKPHGLVADLWPNVRLMQLSGHFLFEYHDNNS-AINVLARKIYSWVHLVLILINYSAIIANLAMESDDVNALAANSITVLFFAHCVTKFLHFAIRRQKFYRVLGTWDTQNSHPLFAESHARHRALAIRKSRKLLMIAVVGTLMTIVGWTGSTFFGESTKTISDPESGNQTVTVEIPRLMLRAWYPWDSSSSSYYLLTFLFQLYWIIFTLFHSNLVDILFCSWLIFACEQLLHVKEIMKPLIDLSSSLDTFN--PNSADLFKMTNT--------NNDSENLYDSKNER------SMNIRGIYSNIRELKGN------RISVVNPVGTM-------NGIGPNGLTKKHEMLVRSAIKYWVERHKHIVRYVSSIGEAYGLALLMHMLTSTITLTLLAYQATKIDSFDLYAMAVLGYLIYSLAQVFVFCTYGNQLIEESSSVVEAAYSCQWYDGSEEAKTFVQIVCQQCQKALSISGAKFFTVSLDLFASVLGAVVTYFMVLVQLK

>XP_014279420.1_PREDICTED:_odorant_receptor_coreceptor_isoform_X2_[Halyomorpha_halys]

------MQKIKMHGLVGDLWPNIRLMQLTGHWLLEYHEETG-GMVRLIRLAYCWLTTFLVVMQFAFLACFLILDTYDADQMAAATITTLFFLHSITKFAYFAIRSKYFYRTFGAWNQVNSHPLFAESNARHRATALSRMRKLLMIIGVITIMSVMAWTTVTFLGDPHREITDPEDVNSTITVEMPQLMVDAWYPWDAKTGFCFFATFIYQLYWLFISLSHANLLDILFCSFVIFACEQLKHLKEILQPLMELSATLDSVV--PNSGELFRGGSG------------GSNMPLVEND----GNDFDIRGIYSNRGDFSGFGQTAVSTI-QTNG-----------NGIGPNGLTKKQELLVRSAIKYWVERHKHVVKFVSSIGDAYGSALLLHMLTSTVTLTLLAYQATKIEGVDVYASTVIGYLLYTLGQVFVFCIHGNELIEESSSVMEAAYSCHWYDGSEEAKTFVQIVCQQCQKSLTVSGAKFFTVSLDLFASVLGAVVTYFMVLVQLK

>NP_001303637.1_odorant_receptor_coreceptor_[Cimex_lectularius]_

-------------------------MQLTGHWLLEYHEENG-GIMRLLRLAYCWITTLLILVQFGFLVCFLILETYDADQMAAATITTLFFLHSVTKYLYFALRSKYFYRTLSAWNQVNSHPLFAESNARHRATALSRMRKLLMIIGVGTIFSVLAWTTVTFLDEPYRDITDPDDVNSTITVEVPQLMVDAWYPWDARNGMAYFLTFIYQLYWLIMSLSHANLLDILFCSFVIFSCEQLKHLKEILQPLMELSAALDSVV--PNSGDLFRASST------------SSNIPLIGND----VNEFDVRGIYSNQRDFSGFQGGAIP----TN------------GGIGPNGLTKKQELLVRSAIKYWVERHKHVVRFVTSIGDCYGSALLLHMLTSTVTLTLLAYQATKIEAVDVYASTVIGYLLYTLGQVFVFCIHGNELIEESSSVMEAAYSCHWYDGSEEAKTFVQIVCQQCQKSLTVSGAKFFTVSLDLFASVLGAVVTYFMVLVQLK

>AHC72291.1_olfactory_co-receptor_protein_[Lygus_pratensis]

------MQKVKMHGLVGDLWPNIRLMQLTGHWLLEYHEETG-GMARLIRIAYCWMTTFIVYLQYAFLVCFLTLETYNSDEMAAVTITTLFFLHSVTKFTYFAIRSKYFYRTLSAWNQVNSHPLFAESNARHRAAALSRMRKLLMIIGVVTILAVFGWTTVTFLDDPVWDKTDPDNVNETISVEIPQLMVYAWYPWDAKTGMTYFMTFALQLYWLFITLAHSNLLDVLFCCFVIFSCEQLKHLKEILQPLMELSAALDSVV--PNSGDLFKSGSA------------GSNIALISNGDG--GNDFDVRGIYSSQRDFSGFQGG------MTNG-----------TTVGPNGLTKRQELLVRSAIKYWVERHRHVVKFVTSIGDTYGTALLLHMLTSTVTLTLLAYQATKIEGVDVYASTTIGYLVYTLGQVFVFCIHGNELIEESSSVMEAAYSCHWYDGSEEAKTFVQIVCQQCQKSLTVSGAKFFTVSLDLFASVFGAVVTYFMVLVQLK

>AHC72294.1_olfactory_co-receptor_protein_[Adelphocoris_fasciaticollis]

------MQKVKMHGLVGDLWPNIRLMQLTGHWLLEYHEETG-GMARLIRIAYCWMTTFVVYLQYAFLVCFLILETYNSDEMAAVTITTLFFLHSVTKFTYFAIRSKYFYRTLSAWNQVNSHPLFAESNARHRAAALSRMRKLLMIIGVVTILAVFGWTTVTFLDDPVWDKTDPDNVNETISVEIPQLMVYAWYPWDAKTGMTYFMTFAPQLYWLFITLAHSNLLDVLFCCFVIFSCEQLKHLKEILQPLMELSAALDSVV--PNSGDLFKSGSA------------GSNVALISNGDG--GNDFDVRGIYSSQRDFSGFQGG------MTNG-----------TTVGPNGLTKRQELLVRSAIKYWVERHRHVVKFVTSIGDTYGTALLLHMLTSTVTLTLLAYQATKIEGVDVYASTTIGYLVYTLGQVFVFCIHGNELIEESSSVMEAAYSCHWYDGSEEAKTFVQIVCQQCQKSLTVSGAKFFTVSLDLFASVSLNKTHITILLY---

>AHC72292.1_olfactory_co-receptor_protein_[Adelphocoris_suturalis]

------MQKVKMHGLVGDLWPNIRLMQLTGHWLLEYHEETG-GMARLIRIAYCWMTTFIVYLQYAFLVCFLILETYNSDEMAAVTITTLFFLHSVTKFTYFAIRSKYFYRTLSAWNQVNSHPLFAESNARHRAAALSRMRKLLMIIGVVTILAVFGWTTVTFLDDPVWDKTDPDNVNETISVEIPQLMVYAWYPWDAKTGMTYFMTFALQLYWLFITLAHSNLLDVLFCCFVIFSCEQLKHLKEILQPLMELSAALDSVV--PNSGDLFKSGSA------------GSNIALISNGDG--GNDFDVRGIYSSQRDFSGFQGG------MTNG-----------TTVGPNGLTKRQELLVRSAIKYWVERHRHVVKFVTSIGDTYGTALLLHMLTSTVTLTLLAYQATKIEGVDVYASTTIGYLVYTLGQVFVFCIHGNELIEESSSVMEAAYSCHWYDGSEEAKTFVQIVCQQCQKSLTVSGAKFFTVSLDLFASVSLNKTHITILLY---

>AOH73455.1_olfactory_receptor_1_[Adelphocoris_lineolatus]

------MQKVKMHGLVGDLWPNIRLMQLTGHWLLEYHEETG-GMARLIRIAYCWMTTFVVYLQYAFLVCFLILETYNSDEMAAVTITTLFFLHSVTKFTYFAIRSKYFYRTLSAWNQVNSHPLFAESNARHRAAALSRMRKLLMIIGVVTILAVFGWTTVTFLDDPVWDKTDPDNVNETISVEIPQLMVYAWYPWDAKTGMTYFMTFALQLYWLFITLAHSNLLDVLFCCFVIFSCEQLKHLKEILQPLMELSAALDSVV--PNSGDLFKSGSA------------GSNIALISNGDG--GNDFDVRGIYSSQRDFSGFQGG------MTNG-----------TTVGPNGLTKRQELLVRSAIKYWVERHRHVVKFVTSIGDTYGTALLLHMLTSTVTLTLLAYQATKIEGVDVYASTTIGYLVYTLGQVFVFCIHGNELIEESSSVMEAAYSCHWYDGSEEAKTFVQIVCQQCQKSLTVSGAKFFTVSLDLFASVFGAVVTYFMVLVQLK

>AFX73448.1_olfactory_co-receptor_[Lygus_lineolaris]

------MQKVKMHGLVGDLWPNIRLMQLTGHWLLEYHEETG-GMARLLRLAYCWMTTFSVYIQYAFLVCFLILETYNADEMAAVTITTLFFLHSVTKFTYFAFRSKYFYRTLGAWNQVNSHPLFAESNARHRATALSRMRKLLMVIGCVTILAVFSWTTVTFLDDPVWDKTDPDNVNETISVEVPQLMVYAWYPWDAKYGMTYFMTFAFQLYWLFITLAHSNLLDVLFCCFVIFACEQLKHLKEILQPLMELSAALDSVV--PNSGDLFKAGSA------------GSDVALIGNGENGNGNDFDVRGIYSSQRDFSGFQGG------ITNG-----------GTVGPNGLTKRQELLVRSAIKYWVERHKHVVKFVSSIGDTYGSALLLHMLTSTVTLTLLAYQATKIEGVDVYAASTIGYLVYTLGQVFVFCIHGNELIEESSSVMEAAYSCHWYDGSEEAKTFVQIVCQQCQKSLTVSGAKFFTVSLDLFASVFGAVVTYFMVLVQLK

>AFX73447.1_olfactory_co-receptor_[Lygus_hesperus]_

------MQKVKMHGLVGDLWPNIRLMQLTGHWLLEYHEETG-GMARLLRLAYCWMTTFSVYIQYAFLVCFLILETYNADEMAAVTITTLFFLHSVTKFTYFAFRSKYFYRTLGAWNQVNSHPLFAESNARHRATALSRMRKLLMVIGCVTILAVFSWTTVTFLDDPVWDKTDPDNVNETISVEVPQLMVYAWYPWDAKYGMTYFMTFAFQLYWLFITLAHSNLLDVLFCCFVIFACEQLKHLKEILQPLMELSAALDSVV--PNSGDLFKAGSA------------GSDVALIGNGENGNGNDFDVRGIYSSQRDFSGFQGG------ITNG-----------GTVGPNGLTKRQELLVRSAIKYWVERHKHVVKFVSSIGDTYGSALLLHMLTSTVTLTLLAYQATKIEGVDVYAASTIGYLVYTLGQVFVFCIHGNELIEESSSVMEAAYSCHWYDGSEEAKTFVQIVCQQCQKSLTVSGAKFFTVSLDLFASVFGAVVTYFMVLVQLK

>AHC72290.1_olfactory_co-receptor_protein_[Apolygus_lucorum]

------MQKVKMHGLVGDLWPNIRLMQLTGHWLLEYHEENG-GMLRLLRMAYCWMTTFSIYIQYAFLVCFLILETYNADEMAAVTITTLFFLHSVTKFTYFAFRSSYFYRTLGAWNQVNSHPLFAESNARHRATALSRMRKLLMIIGTVTILAVFGWTTVTFLDEPVWDKTDPDNVNETISVEIPQLMVYAWYPWDARYGMTYFMTFVFQLYWLFITLAHSNLLDVLFCCFVIFACEQLKHLKEILQPLMELSAALDSVV--PNSGDLFKAGSA------------GSDIAL--IGNGENGNDFDVRGIYSSQRDFSGFQGG------VVNG-----------GTVGPNGLTKRQELLVRSAIKYWVERHKHVVKFVSSIGDTYGSALLLHMLTSTVTLTLLAYQATKIEAVDVYAASTIGYLVYTLGQVFVFCIHGNELIEESSSVMEAAYSCHWYDGSEEAKTFVQIVCQQCQKSLTVSGAKFFTVSLDLFASVFGAVVTYFMVLVQLK

>PbOr45PSE

-----------KKDIIDMLIW-NRLLRIFGIWPLIYT-----TIEKILATFLFASCWTVLSLFLILISIYTFTDQSIMNEKMKXGP-LNYVFFSILKYLFLLVRSTRISEMLQVVTIGWRMVQEKYHREIMIRD-AEKGHLLSXFCIMFMYYGDLSYNTVPFLSN------SPD-ANEZ-NFTIRPMVYGFDIIFNLQLMPIYVFAFTLQCFTGVVMFNITTSVCCLVTMFVAHACGQINIVIVRVEDLXEMH---------------------------------------------------------------------------------------------------------------YHVQHHVRPLRFSASIEKNFARXCLVK--GTMLIICLVEYSLIXEZRQYSDLHILFSIRFIRLQNIFIFCYIGKLLTKQXEHTYNTSYKIE-YNLPEKIALNLMLMINISRHSIQITAGRLINLSFANFGNAXKTSVAYLNLLRT--

>CfOr102

---------------EYAHGWNRYTMMFMGIWPENK------NFDRASSYKAVVPILTMFCFICAPQSANLLFIWDDFDLVIENLSMAITITISMLKTAIFWSKGRSMKILISSMKKDWNMTVDKRERKIMS-DIAKITRNLSIRSTIMAEIVVIAYVTYRYIVI-----------RYT----GRQLLFRAYFPYNVSNSPSYELTFFAQIIACMYAAVTYAAVDTFIATLVLHTCGQLANLRQELINLHNC------------------------------------------------------------------------------------------------------TKTRFQTKLRKIVRKHEYLNSFAETIEDCFNMMLLIQMVGCSLQLCFQCLQAFMANEFLAQIIFLMIYVVYILLQLYLYCYIGERLLVESTKIAYAAYDCSWYNLSAYEAKSLIIIMCRAQSPLQITAGRFCSFNRELFSEVLKKSVTYMSCLYA--

>CfOr103

--------------LHYAFTLSRQCMRIVGIWPDLDL-----NVCRRPKIAFIFATCIMTLYVLIPQVLNLLRTSGSVSQMVELFVAPNITFMAICKLIITKYHGDKLRILIASSMTDLMTSKNNWERNTMLNL-VRTGRKISITYFVIAIAIIIFACYIRL-EN----VFQNI-HQP-----RRYLVYRFDY---IQKSPNYEITCFIQICGAIYAIFGNYSVDSFISILLLHICAQLINLRTALNNLINKLNNKPI------------------------------------------------------------------------------------------LNNKPISSSKFKKGLAAIIVRHEYLIRNVKTINDCYSSVLCIHVLCGSFQLCLVAFQTSTMDNSNVKIIFLAIYISFILTQLYVYCYAAERLLMESTNMAFGMYECKWYNIPAKDAKDLMLIVYQSAISLKLTAGIFGNFSMELFGIAIKTTMGYLSALLT--

>CfOr100

--------ERWKDDIAYAMTPLKLITWPIGVWPLQVY-----NIYSLLRCVLCTFCACLVAILP---SLEIYMGCTDVGQNIDCLMLICCGFLGVLKTTWFRIYANSLIINYNSALRDYQTIDDTKERDIMR-KHAFIGRTIFSSLLIFAYFGCLMFGIVPILNYNMSNQIN-M-TNEDMTLE-YVIPSRCALKYFPTS--MYTIFCLIETVLITLASTTNFGNDALFLNITLHVCGQIKILRIRFINFDDTS--------------------------------------------------------------------------------------------------------RICDRFNALIERHRYLITLTRELANLISFVLQIQLFIISILLCIMGFQFIYAVNDTAMIGKSLVVQILFLTQLTLYSFIGNYLKSEMEEIGLSIYQSTWYNFPKKLARNVNFILLQTKSPIALQAGNFIVINLSTYVSILKSSFSYLSVLR---

>CfOr101

-------NERWKDDIAYAMTPFKLITWPIGVWPLQVY-----DIYSLLRCALSTFCASLVVILP---SMEIYMGCIDLKQNIDCLMLICCGFLGVLKMTWFRIYPNSLIISYNSALHDYQTIDDIKERDIMR-KHAFIGRIISSFLLSMAYFCCLTYGIISILDYNINNRIN-V-TNEDTTLE-YVIPSRCVLEYFNFPTSMYKICCLVETAIIILVSTANIGNDALFFNITLHICGQVNILRIHFINFD-----------------------------------------------------------------------------------------------------------RIYDRFNALIQRHQDLISLTRDLADLMSFVFLIELFIISILLCIMGFELILAANNIIRAGNKLFGLSGFLTQLTLYSFIGNYLKSEMEEIALSIYQSTWYNFPKKLANDVIFILMQTKSAVELQAGNFIAINLSTYVSILKTSFSYLSVLR---

>CfOr106

---------------NYAFTTSRQCMRLLGIWPDPNL-----NVFHRSKVGFMLAMCIMSLYVFTPQVINVIRAWGNVSRMVEFFVAANFSLMALCKLIITRYHGEKLRMIIASIMTDWMTSKSQLEQKTMLKL-ARSGRSLSFGYFVIVIGTLIAAYYAHI-GS----IFRNI-HQS-----RRYLIYRFDY---IQKSPNYEITYFIQLCGGTYAIFSNYSVDSFISILLLHMCAQLINLRTTLNNLIDELNNK-----------------------------------------------------------------------------------------------------TFRKGLAAIIIRHEYLIRRTKTIDDCYSPVLFVHMLSATFQLCLVTFQIFTMDVSFIKIMFFAFYILLVLIQLYIYCYAAERLLTESTNMAYGVYGCKWYNISAKNAKDLMFIVYRSAISLKLTAGKFGNFSLELFGIAVKTSMGYLSALLTIR

>HsOr70

-------SERWNDDIARAMTPFKLLMWPMGGWPLQ--------VYNIFSLIRCVAVTSGMSIIVILPSIEIYLGCNDAESNVDALMLIVCGLLAMAKMIWFRIHASNLVKTYKFAVNDYLAIESAEQRDIMLKH-AFIAKTLMCIMVSVAYSDSCILFLISILGN--SDNVTQA-TNVTQH-EGYTVPSRCALEYLNAPRNMHVTHCIIELIMLLLVCTSNYGNDSLFLHIALHICAQVKILKSNLIDF-----------------------------------------------------------------------------------------------------------PQINKRFNALIQRHGDLMEMTKILSNVVSFILLMQLFLSSILLCILGFQLILALNNFAMLVKSFMILNTFLAQITIYCVIGDYLKSQMEEVGTFIYQSTWYNLPAKLTKSLSFIIMRSQFPVQLQAGNFIVVNLATYMSILKTSISYLSVLRVM-

>CfOr104

---------------NYALQLSRQCLRLIGVWPDPHI-----SLSDFRRIRFIIAICAVSIYIFTPQAINLIRAWGNVNRMMECFVAANFSMMAISKLVVTKYHGKKLRTLIASIMTDWMTSNNNSERNKILKL-GKNSKNLSFGYFIATVGTTMLAVYVR-LEGVFRNIHKP----------RRHLPYRFDY---IQKTPNYEITCFIQICGGIYTIFGNYSVDNFISILILHICAQLINLQTTLNNLVDKLKNNPASS---------------------------------------------------------------------------------------KLKNNPASSSKFRKGLTAIIIRHEHLIRSAKTIDDCYSTVLVIHMMGASFQLCLVTFQIFTMDFSPIRTIYLIFFVSLVLMQLYIYCYASERLLTENINMAHTAYDCNWYNILAKDARDLMFIVYRSMIPLKLSAGIFGNFSLELFGIAIKTSMGYLSALLTIR

>CfOr105

---------------SYAFTMSRQCLWLLGVWPDPQV-----SLNIFRPNIFMIVTCILSLYVIVPQLTNMIRAWGDVGRMIEYVASANFGLMALCKLVATWYHGETLRTLMTSIVTDWRISRNNWERDAMLNI-ARRGRSLSFKCCLAATCTVTFYVSFNLIKF-YRNMYL----------PQRSLVYRFAYPYNIQKSPNYEITFFIQLSGGVYSAIINCSVDCFISILLLHVCAQLINLRTTLNNLVSELANRSIS-----------------------------------------------------------------------------------------------SSSKFKEGLTAIAIRHQHLIRYAXTSDEIKRYKYLFSLIMKYLNIKFNIFMITDKLDISVKITFLTFYITLVLTHLYIYCYSAERLLTESTNIAYGVYECKWYNIPAKDAKILMFIVHGSTIPLKLTAGKFGVFSIEMFGTTVKTSMGYLSALLTMK

>H_TcOr200FIX

------------------------------------------------HKITKTCNFLLVISQLFFYLIQIHFSRFSLELLARYSTIMMITTVALFGLILSFYLEEDIHELYKILTEIWPLDKASKKDQQDLRQKSRRINSLNLYFLGF--LAFMIVIFLPIFGD-------------------EENLFLCIQVFDEYFGDRAFIFYNLYFIGFPFLIYFSVQLCFMFLYAILHLHVQINLINHHICEM----------------------------------------------------------------------------------------------------LHSVVYQSAISQLLCQCIRQDIALKRFIVKLNETVQLGLPFFLPVSGLCGISVIFFLLNYTMSLVLWLRVSAFFICLVFVALIFSVSGQLLIDETGKIFDTLVKCPWHIWNVRNRKIYLICLTHCVRPNCISYAGI-TLNRIFLITVFYKTVSNAFILYQVR

>CfOr108

---------------SYAFALSRQCLRMLGVWPDPCI-----PLSNFRRPSIRFITVTCILSLYVIMLTNMIRAWGNVIHMVEYIASANFSLMALSKLIATWYHSETLRTLMTSVMIDWVNSWNNPERNTMLRL-ARRGRSLSSRYYAFATITVSFYMCFNLLK-FYRNIHQP----------QRRLVYHFVYPYNSQKSPNYEITFIIQLCGGLCTALINCTVDSFISTLLLHICAQLINLRMALNNLVDELANKSISS--------------------------------------------------------------------------------------------SISSSKFKEGLTAIAIRHIHLIRDARTIDNCYSAVLFAHMLAATFQLCFETFQVYTIDVSTFKMAFLLFYVILVLTQLYIYCYSAERLLTESSGMAHCVYECKWYNIPAKDAKNLIFIVHGSSIALKLTAGKFGNFSMEMFGTTVKTAMGYLSMLVTIK

>CfOr109

---------------SYAFALSRQCLRMLGIWPDPCL-----NDFHRPSIRFIIVTCILSLYVIMPQLTNMIRAWGNVIHMVEYIASANFSLMALSKLIATWYHSKTLRTLMTSVMIDWINSTNSPERNMMLR-LARRGRSLSSRYYALATTGVLFYICLNLLK-FYRNIHQP----------QRRLVYHFVYPYNSQKSPNYEITFVIQLCGGLCTGLINCTVDSFISILLLHVCAQLINLRTALNNLVDELANKSISS----------------------------------------------------------------------------------------------SSSKFKEGLAAIAIRHKHLIRDAKTIDNCYSAVLFVHMLAATFQLCFETFQVYTIDVSAFKMAFLLFYVILVLTQLYIYCYSAERLLTESSGMAHCVYECKWYNIPAKDAKNLIFIVHGSSIALKLTAGKFGIFSMEMF------------------

>McOr7PAR

------------------------------------------------------------------------------------LLYCTTEFAFLCKLMNFVLSKKEIIELEAILESRLFTVDTPEEEAI-IKTSTRQIRKLANIYKTLCFLSVTFYALFPLADG-----------GR----EAQKLPLPGWFPFNVCN--HYYEVFIFEAIGIGLCAWFNSALDLLVVIMMILGKAQFELLRHRLMNI--------------------------------------------------------------------------------------------------------------RRRVKMCAQHYKSILRFVMLTESIYSNGIFVQFMSSGIVICFTGFQMLIISLKSIQFVQRILYLSCMMYQIVMYCWYGQVLTDSSNKITEACYLADWINCNVILRKSLLIIMERAKYPAKIRAANIFTVNLETLLTILRSSYSYFALIYSI-

>DeugOr46aA

---------------------QKAILNLFSLWPQTER-----RWRIIHQVNYVHVMGFWVLFFDLLLVIHVVANLSYMSEVVRAIFVLATSAGHTTKLLSVKANNVELEKLFKRLDDEDFQPRGVEEE-LIFTEACERSKKLRDFYGALS-LAALSMILIPQFVLD-----------------WSQLPLGTYNPFDTSGSPGYWFLYCYQCLALSVSCFTNIGFDSLCSSLFIFIECQLDILVVRLDKMGRLNNDD-------------------------------------------------------------------------------------------------NDDSSVEHQLKENIRYHMTIVELTKTVERLLCNPISVQIFCSVLVLTANFYAIALVSSRLIFIINYITYHAESDFHLVLLCRVGSEVTQRSLDLPHELYKTSWVDWNRSNLRIVLLFLQRLHSTLRIRTLNPLGFDLMLF-SIVNCSYSYFALLKRV-

>383_Si_gnF.scaffold04171_663867-963062.pep

---------------DHYHKLNQIFLRILGLWPYG--------KTKYDHFRATCFFIILVSHTFV-QLAQLLIADFSIIVIIRILSEFLPILMCLVKFNMFFLETKKI---LKEMTNNRRILTEAQEIE-IIEHYANQGRIFTIIYTCNMTFILYILATIELFPSILDCFWPLN-ESRT-----HNMLFL--TQYHISEGIQYYSCFLYFSISVSIGCFSVICITTMFTVAMLHCCAIFKICSYRIKQSVDEK--------------------------------------------------------------------------------------------------CSNKRNVIVKRIIRTIELHQNAKRLFKLCIPNFGIYFFIMVVVGTCSCVANLYRLLYSMDNFPEIMLALGYIITHEIYLVATTYMGQNLVNHADELFNAIYMSLWYKAPVTIQKLLLFMMQVASKSVVPKVGGIYCSVMESFTTV---------------

>DeugOr46aB

----------------DFYKYQVWYFEILGVWKLPTT-----DHQRRFQSMRFGFILVILFIMLLLFALKLLDNISQVREILKVFFMFATEISCMTKLLYLKLKSRKLAGLVDMMLSTEFSVKTEQERQ-ILASTNVTVVHMRNFYGLMSFITAFMMLLIPCFGN------------------YEELPLTMYEVCEIEGRICYWLHYIFHAISLMPTCFLNITYDSMAFSLLCFLKVQLHILVLRLEKLGPVIDPQDNE-------------------------------------------------------------------------------------------IDPQDNERIARELRECAAYYNNIVQFKNLVELFIKVPGSVQLVCSVLVLISNLYDMSTMNGDAIFMAKVCIYQLVMLWQIFIICYASNEVTVQSSKLGHGIYSSEWTEWNKSNRRIILLMMQRFNSPMLLTFNPTFIFSLEAFASIVNCSYSYFALLKRV-

>DvirOr88a__scaffold_12855_residues_4334724_to_4337026_reverse_strand.[501_1803].sp

--------------------------------------------------------------------------------SHQNLPVLSISIYFTIRGIMMFIKRHDIVDFLNVLDREFPKDLVSQRV-LNVPQVFERYHRRHGYVGLYANYALPGFCLTPVVTYI---LTYED-RNAPIL--LDHQLLGGWLPYDLRQNLVYPLVWLYDVYCMLVGVTFFTSFDTLFNTMQAQVIMYLDCFCRQLEALNAVD-----------------------------------------------------------------------------------------------------DERQFHEHICGLIRRHQQLNLICDKFNDIFKLAILITDLVGATSICFHLYLITE-NQDPLMIIKYILPTLALVVFTFEICLRGTQLEEASSRLNEALYNQNWYMGSKMYRKLILIWIKYAQCTRKLTAYGLVEINMKHFSDIMQLAYRLFTFLKS--

>ApOr79F

------------------HIFNIKLARLTGLYQMLDP-----NTKCRVRNIYYVVMSCVLLYMCLISFSGLYYWTVNIPISIDYFWKSVSTFYTIYKTWIIIRHSNDIWNCLSITRHDFTTFSNRNRH--VLDRWRERVSWSTTIYAIIYTMSMVGYLVFTLAFDEGKTPVKNH--DGSIGY-YRQNVMNLYLIVDETYNAHYYTFYIIEALFVGFLGLFFFIFDFLLVTLCFSMCCQMQIVCSAFESVGHKS--------------------------------------------------------------------------------------------NGNTKITPNEHDLIWDELRTIIMDHQAVMEKYKDFLSLFRRVMLVHIFISSITVIALWFTFIMSKTSEVIVIKTFCSTPPVMFQIFMVCYLFGKLHEQKDSIIFALYSSNWTEMDMKCKKLILLTMNANQKKLKFTRTRI--VNLEMFFKTMGNCYTVISVLVN--

>DbipOr69aA

----------------------------------------------KLQTVCYYLGSFFMGYQNIGGVVYWCLYAKDIIETAQVFGSLMLTCVGFSKIWCFTRRRNQMEDVMAELHELYPTTRKEHYR---LQHHYDWADLIMKYANLFYFAFYIFYNGSPLVLLLWEYITDDQ--NL-----SYKTQANAWYPWKVRGSWGYALAIWVSTMAGNLGIFLTLAILNILCVCTVQLVMHFDGLATQLLNLDA-----------------------------------------------------------------------------------------------------------AHQELKHLIRYHRQLIDISDKTNEIFDSIFLTSLICSTLAICMTSVAVLL--LDLAPALKNINGLLAFLVYHFMLSYLGTQINLASEKILPAAFYNNWYEGDLPYRKMLLILMLRASKPYKWKTIKLSEVSILNYVDTLKTSYQMYACVRSM-

>DbipOr69aB

----------------------------------------------KLQTVCYYLGSFFMGYQNIGGVVYWCLYAKDIIETAQVFGSLMLTCVGFSKIWCFTRRRNQMEDVMAELHELYPTTRKEHYR---LQHHYDWADLIMKYANLFYFAFYIFYNGSPLVLLLWEYITDDQ--NL-----SYKTQANAWYPWKVRGSWGYALAIWVSTMAGNLGIFLTLAILNILCVCTVQLVMHFDGLATQLLNLDA-----------------------------------------------------------------------------------------------------------AHQELKHLIRYHRQLIDISDKTNEIFDSIFLTSLICSTLAICMTSVAVLL--LDLAPALKNINGLLAFLVYHFMLSYLGTQLLFQSEKILPAAFYNNWYEGDLPYRKMLLILMLRASKPYKWKTIKLSEVSILNYVDTLKTSYQMYACVRSM-

>DmojOr56a___scaffold_6496_residues_21038290_to_21042137_reverse_strand.[501_3348].sp

-----------------IFSLHLRCFRWYGYVASI-E-----QRHPWLSLIRCTIFTASIWVSCGMMLSRLFFSRGSINEDATSWATAVQYFAVSIATLNAYVQRERVIRMLRTAHADLQQLSDAQEL-NLLQDTQRYVRTITFLLWVPSVVAGMMAYLDCIYLPKTVFNVDAV-RRGEA---QPILLFK-LFPFNELY-DQFIVGYLGPCYALALGITTIPLWHTFIACLMKYVTLRLQVLKKRT---------------------------------------------------------------------------------------------------------------------KYFVQEQLKIREFVRQIERLIRIPVMADFIIFSILICFLFFALNVGVPSTMDYSFMFLYLFVMAAILWIYHWHATLIVESQNELCFAFYDVDWYQFGLPVQRMLLLMMMHTQRPLKIRAL-LVELNLKTFLDIMRGAYSYFNLLRST-

>DtakOr85b2

----------------SFLKYANVFYLSIGMLAYDHK-----DNGKRKEQLLHWIFVGQMLNLNAVLLIYVFLAISNFLEATMNLSFIGFVVVGDLKIWHIWRQRKRLTQVVNELEHLHPERLDQQSP-YNVKNHLSGYSRYSKFYFGMHLVLIWTYNLYWAVYYLVCDLWLGI-----RKF-ERMLPYYCWVPWNWSTGSSYYLMYVSQNLAGQACLSGQLAADMLMCALVTLVVMHFIRLSGNIE-------------------------------------------------------------------------------------------------------------------LQAVVAYHQRLLVLCQNINEIFGVSLLCNFVSSSFIICFVGFQMT-IGGKIDNLVMLVLFLFCAMVQVFMIATHAQRLIDAXXXXXXAVYNHDWFHADLRYRKMLILIVKRAQRPSRLKATIFLNVSLVTVSDLLQLSYKFFALLRTM-

>gi|350422520|ref|XP_003493189.1|

----------YAHDYEYSIQVNRWLMQPIGAWPKLTN-----RTQRLFAKLLNFICHSLIIFTIVPCILHIVYEAESSRMKMKIIGPVSHWLMGELNYCCLLSKTDDIIRCIKHVERDWQVVENASSRE-MMLKYAKVGRFIAFIAAFCMHSGVLAFSITKGFKK----MMFLV-GNDSY-F-MYPLPCPVYTNLDARFSPANEIVFVLQILSGFIVTSVTVGACGLAAVLTMHASGQLNMVVARLDNLVDTKVEEKQE---------------------------------------------------------------------------------------------QEAQTVAQRKLGIIVEHHLRTLSLIASIEKVMNMICLVELVGCTINMCIIKYSFLT-EKSKDMRIVYAIVYASMVFNIFIFCYIGEIVIEQGERVGKKVYMTEWYRLPHKTALGLVLVISRSSMVVKITAGKFIQISITTFGVVFKTSFAYLNMIRTM-

>DanaOr65aL3__scaffold_12916_residues_4974689_to_4977045_forward_strand.[501_1842].sp

----------------HTFAYNRGQMKAMGFYMSAEK-----R-HQPLMVAWHYFFLFQITISLASLGYGILESLDDIVRLGQDMAYTISTFYIYFKMVWFTIYADDIDEVIESLEKCNRRERKGPGLIRYMKRWIFLLLSLMALWLFLIVSFIMIMISAPFWLE------------------SQNLPFHVAYPFDPSKHPCHIIIFVSQSCIMLYALIWTLFAEQLSVTIYSELTSDLKTLCMELRIVQ----------------------------------------------------------------------------------------------------------------VYDFAKFHQRIISITDRSNEVFNGAFTMQLIVNFLLISLSVFEAMVFWHDPKVAAEYLVLMVMALSHLSYWSKFGDMLTQESLEVAVAAYEAYDPTGFRSTHTIIWLIIQRAQTPLSVKCRLFPPFNLENNLAVLKQCYGIFNFLLK--

>DyakOr47a__chr2R_residues_2003780_to_2008136_reverse_strand.[1501_2857].sp

----------------SFLQVQKSTIALLGFDLFS-------ENREMWKRPYRAMNVFGIAAIFPFIMAAVLHNWKNVMQLADAMVALLITILGLFKFSMILYLRRDFKRLIDKFRLLMSNEADQGEYAEILNAANKQDQRMCTLFRTCFLLAWALNSVLPFVRM----GFSYW-QSGHA---EPELPFPCLFPWNIHIIRNYVLSFVWSAFASTGVVLPAVSLDTIFCSFTSNLCAFFKIAQYKVVRFRGGSLKESQATLNKVF-------------------------------------------------------------------------------------------------LNKVFALYQTSLDMCSDLNQCYQPIICAQFFISSLQLCMLGYLFSI-TFAQSEGVYYASFIATIIIQAYIYCYCGENLKTESATFEWAIYDSPWHDSSTSICRSLLISMMRAHRGFRIT-GYFFEANMEAFSSIVRTAMSYITMLRSF-

>389_Si_gnF.scaffold00899_962711-1051965.pep

------------RDVTVTLSVHRFALSCVGIWPVR--------ERNIFMDLRWIIAIFLEASTAIPMFAEIYIHCNGAKRSFDWVTPGAAATLALTRLITPRIHRGELLEIVTSMVDDWTMQKEKKIR-WIMKKYATMSTRVTVLTFIMVAIILGVYIAMAISAV-TTKRQHPD-NEINVSVS-DEAESQ-SCVFRSES--SHQAFMVIQAMQMFTTCIITFGTTSFFFGLAMHLCAQFDALSIKLTEFR-----------------------------------------------------------------------------------------------------------NAHRVISEAVQRHCQLIRLAECMEESFNANVLMYLFVTSFLMCIDGYMLIASVGDVPTIVHSASILLLMLIQLSFYTFAGDYLETRSTALAYTTYNCDWYELPASRAKDFQIIIMRASIPHQLTAGKFVVMNMITFKDILKSTASYLSVLRVM-

>CfOr343

---------------EYYYKFNRFLLSVTGLWPYQ---------SKWSACFARTIITVIMLSAIFTQISSVFTSELNMDFVIESLPMFVPTVGNLCQLYSRIFYVDKIKELLEHMWNDWALEKTDIESKI-MHQYAKTTRLVMIYHSLLLYIIVTAMAISMFLPEIF-VILPVN-KSS-----QRLEAIHMEFYLDKER--YIYLIMSCVCIVLFLVPLVFLASSSLYLVLTQHVCSMCEVLGYRAERLFYIIED---------------------------------------------------------------------------------------------------KTKISCKNIVVLVQLHYNVIEFVNTIETCHTIPFLMDLVGVVITISFWLIQILTIFENVKRACASIGLIIASLCYLAIPNYMGQKVTDMTSSICEKVYNSAWYYASVSEQKSLLLIMGRRFRPLVLTACKFYAMSLPSFEMILQMGVSYCMFMRKV-

>gi|340710869|ref|XP_003394006.1|

-------GQHNDNDIHYTLQMCQWLLKLIGVWPLVNS-----KLEQLLSVVVMIICFCSIFFIILPSGHHFFFVEKNLYMKVKMLGPVGFCVFATVKYSYLALKGAFLQRCIRQLKNDWKRVQDPSHRQIML-KYAGISRKLITMCAVFIYTGGMSYHTVAQFLS-----TD-K-TRENYT--VRPLTYIGYDPFNTQSSPTYEIVFFLHCFAAMIMYSITTVAYGLAAVFVTHVCGQIQIQIVRLQNLVESK----------------------------------------------------------------------------------------------------------RDLFAVIVRDHVETLRFSKNIEDALYQICLTEIVECTMNMCMLEYYCLMESTDLIVTFTYITLLTSFTFNIFIFCYIGELLSEQCSEIGTVSYEIDWYNLPAKEAYDLILLISISQYPPKLTAGKIIELSLNTFSSVAKTSLVYLNLLQTV-

>AaOR55

--------------------ASLKLCRWLGLWHDV-------NLDKPCWQTVFISFCLLFWYI-LPGCLYITRGGRMLQYLLKSILEVFSMCVIVLRCVVHMINRKTVQNSFVELEDAISTFENSPYEVRQMLRHLKSADYLVKIYVSIVFIQASIYGLVPAILTTYRYC---N-SNETV--QLPSAVMEADYVFDHSTNWIWLLVTIISLLVEYLLLGTFSSQECLFWNLLHHVSSLFKVIRLEIARLDQYTDPK----------------------------------------------------------------------------------------------------KQYTERLASIVSTHEVCYRCARSLEIVLSPLLAVLYCTCIIQTCYLLFVISM-IDDLVVIASMIFVLQYIVFLIFSFSMLGAELTEESALVSEAIYNSNWYMRMPAERRLLLFMKMRADRPVGITAAKFFYVNRSTFAEAMKTAFSFFTIMQQF-

>DbipOr47a

----------------------KGTIALLGFDLFG-------ESRKMWLRPYRAVNVFGIATLFPFILAAVLHNIKNVMLMADAMVALLITILGLFKFSMILYLRKDFKGLIDRFRLVMANSGQGEEYAEIIRSANQQDQRVCSIFRTCFLIAWALNSVLPFVRM----GLSYW-LTGHY---EPELPFPCLFPWDIHILRNYAMSFVFCAFASTGVVLPAVSLDTIFCSFTSNLCAFFKIAQYKVLRFKGSTLEESQTTLNR-----------------------------------------------------------------------------------------------------------QTSLDMCSELNECYQPIICAQFFISSLQLCMLGYLFSI-TFAQTEGVYYASFIATIIIQAYIYCYCGENLKTESASFEWAIYDSPWHDSSAAICRSLLISMMRAHRGFRIT-GYFFEANMEAFSSIVRTAMSYITMLRSF-

>DbipOr85f

----------------------SSLFRLMGYDMLEAT-----RLQRIMMQLYRVLCLGSHGVCLGFMFFRLFETIDSVSLIMRYATLVTYVVNSDTKYG-TVLQRAAIQSLNNKLADLYPKTTLDRIY--YR-VNDHFWSRTLLYLIRFYIGSSIMVVVGPILTSLWLYFFHEQ-------F-SYMHCYP-YFIFDPEKHWIYVGIYALEWLHSTQMVVSNIATDMWLIYFQVQICMHFRAMIKSIE-----------------------------------------------------------------------------------------------------------------HFLSKIVDKHHYLVSLQTDLNSIFGSSLLLSLLSTASVLCTVSVYTL-IQGLTLEGVTYVIFIGTSVVQMYFVCYHGQQVLDLSAYISHAVYNHNFHNASLSYKKFLLIIIIRSQKPVELNAMGYLPISLDTFKQLMSVIYRAITMLRQM-

>DvirOr85d__scaffold_13047_residues_7545546_to_7547891_forward_strand.[501_1846].sp

-----TIKTRKTVPLDSFFRYVDVFYLSIGMVPYDRS-----KDSSWRRIILRTYFVFQMLNLNLVLLIYVFLSGQNFLEATMNLSFIGFVVVGDIKIQHIWGKRERITQVVRRLQELHPVCAEQQAI-YKIDEYLSGYKCVSIFYFGMHLVLIWTYNLYWAVYYLVWDFWLGM-R----RF-ERMLPYYCWTPWEWKNNWSYYVMYVSQNLGGQACLSGQLAADMFMCALVTLLIMHFKRLGSQIEQHVAG----------------------------------------------------------------------------------------------------------------ASIVYHQRLLLLCQDINEIFGVSLFCNFASSSFIICFVIFQMT-IGGNIDNLVMLVLFLFCAMVQVFMIGSYAQHLINATEQIGEAVYNHDWFLADLRYRKLMLVIIQRAQRPSYLKATNLLKVSLVTVTDLLQLSYKFFALLRTM-

>390_Si_gnF.scaffold02694_2287513-2447739.pep

-------------DFNYAVQVTRVILRAIGAWPIPNS-----NMERITTRLQNLICYFLFAFIIIPGLLRVFLKEHEFKRRIRLLAPLLNCGMGWLKYNLLVNHAREIKSCLKQARQDWSNTIDEDNR-KMMLSTAKIGRRFAIFSAAFMYIGGLSYRTLVPLSK-GRMLTP-M--NIT----VRALACPSYFIFDGEVSPAYEIVFTLQFFAGLITYSVRVGAAGLAAFFIMHVCGQLRIIIGKLQYLNNMP-------------------------------------------------------------------------------------------------------------LADIVEHQIKVKRFLSHLEVMMRQICFVELVGCTFNLCMLGYYTISENSDAVAILTYFILLISLTFNILIFCYIGELLMEESSQIGSMCYMINWYQLSPRSVRSLILIIAMSSHPIKLSAGRMVDLSLTTFGTVSHYVY----------

>DgriOr82a_scaffold_15074_residues_2505577_to_2508617_reverse_strand.[501_2541].sp

----------------RLFQLQENCLRLMGHNIESSG-----DGFKHLSL-KHIASLLFVISAVYPLLYYVIYNRDDMELITAGLSVVFTNLLTVIKISTFLVYKQNFWQMLHSFRQLYLKSHQKEEDAYLV-QANKLATNLGRAYCISCGFTGLYFMLGPIVKII-S---SSW-RGV---VYARELPMPMKFPFNDVNTPGYEVGFIYTMFVTIVVVLYASAVDGLFISFAINLRAHFQTLQQEIRELTF------------------------------------------------------------------------------------------------------------QQQLVKAVDYHVHILSLSKRLRVIYTPIVFGQFFITSLQVGVIIYQIVTHMNSIMALLVYLSFLFSIMLQLFMYCYGGEIIKVESLQVGIAVQTCNWHLAAPKQRRSLVFIMHRSQRQMLIKAG-FYEASLANFLSICRAAMSFITLIQSI-

>DperOr22aL3_super_8_residues_1247365_to_1251671_reverse_strand.[1501_2807].sp

----------------DAFVYLDRVQKLWGWRATEDE-----RWMVLYNTWAFIWNVLLLVLLPLSMSMEYVQRFKNFGEFFGSLEICVDMYGCSLKCVYTMFGYKRFQAARKLLDRLDLRCTSDEDRASVHR-SVALANRCYVTYHILYSGFVVINWTGYLLL--------------------GSHAWRMYLPLDSEK--NFLVTSFFELLLMSGVVTMNQCTDVSPLAHMIMARCHMGLLKDRLTKLHSD----------------------------------------------------------------------------------------------------SKTEKEHQEDLNRCIQDHSVILEYVNLLRPVYSVTIFVQFLLIGLVLGLSMIHIMFFSNFWTGIG-TMCFMFDVCLETFPFCYLCNIIIEDCRELSESLFQSDWLGASRKYKSTLVYFLHNLQQPIILTAGGVFPICMQTNLSV---------------

>DmojOr9a___scaffold_6473_residues_2079244_to_2081508_forward_strand.[501_1765].sp

---------------AYFLRVQTLTFRMMGFDLWE-S-----GSKNDRPQITFVVMILSIAFLA-PLLFSTVINVTIVSIMSDALGSVLAIVVSVVKYLVFVYHRKSFVHLIYRIRDILEKEMRAPEIIPIVEMENRNDQSLSLTYMRCFFAACVFAGVKPVIIML---TLRLR-TGHT----HLELPHAGVYPWSDQALLPYVPTYLWNLMASYGAVTMSLAMDTLLFAFTYNVCALFKIAQYRVRHLQPI-------------------------------------------------------------------------------------------------------------ELVRVLQLHQTGLDIGTQLGHSLRPLVFIQFLVSAVQLCFVGFQLAD-LFPTLECIYFIFFLGSIVIALFIYSHCGENMRQASVDFGTALYDSSWVDFAPATRQALIVAIMRAQLPCQLN-GYFFEPNMANFSAVVRSAISYVMMLRSF-

>gi|22293495|emb|CAD31853.1|

------------------------LFRFSGMNIKNKT-----PLDTIKYRWLYTLNFLVVFSAIIGSVYYVILGIKNFIEVTSVAPCLTFSILSMIKSLYHLMYEEHIQELIELLRELELRENNREKKEEIIASETGFLNKVINVLYVLNCSMIVVFDMTPMIMIAVK---YYK-TNE---F-EMLLPYLDVFSFIPYELKYWPFAYIHQIWSECVVLLDMAAADYLFFTCCTYIRVQFKLLQYDFERMI-------------------------------------------------------------------------------------------------------YEENELRNKFTELLKWHQDIIYSSTILEIIYSKSTLFNFLSSSLVICLTGFNVTI-VDDIVIIITFLTFLSMALMQVFFLCFFADLMMTASLEISNAVYNCRWYSANIKVGKQILFVQTRAQEPCKLTAAGFADVNLNAFMRVLSSAWSYFALLQTV-

>DpseOr85e__Ch2_residues_17228776_to_17231190_forward_strand.[501_2023].sp

------------------------------WWPTWLR-----PVGGLMAKAYCSMVILTSLHLLLFTTTLDVLPTGELQAITDALTMTIIYFFTAYANIYWCVRSQRLLAFMDHINREYRHHSLAGVTFVSSHAAHRWSRSFTTIWILSCLVGVITWGVSPLMLG------------------IRTLPLTCWYPFDALSPGTYTAVYATQLFGQISVGVTFGFGGSLFVTLCLLLLAQFDVLYCSLKNLDAHSKLLSGETIAGLGLLQRELLQ----------------------------------------------------------------------------SQNLAQVKVFHSALVECVRLHRFILYCCAELENLFSPYCLVKSMQITLQLCLLVFVGVSGTREFLRIVNQIQYLALTLFELLMFTYCGELLSRHSVRSGEAFWRGGWWKHAHLLRQDVLIFLVNSRRAVYVTAGKFYVMDVNRLRSVITQAFSFLTLLQK--

>HarmOR19p

--------------------------------------------------------------MTILEIMAFTIGNFPVEEKRECLSFASSHVVVMLKIFLFIFNKPLIKGLNHKIVSICEDY---DDSA-LMAKKYKTMKRNVISYFAIVYGTTLFYIAGGLRNMF-----R-----G-----SHFVTVVTYYPFDDNS-PLANFVRVLNTIILSMM------------------------------------------------------------------------------------------------------------------------------------------------------------------------------------------------------------------------------------------------------------------------------------------------------

>DkikOr7a

----------------EAFRNLFNCFYALGMQAPDGP-----TKSNAWQSIYRCFSAFMYLWQLLLVISYRYMGGMEITQVLTSLQVAIDAVILPAKIVALAWNLHLLRRAEHHLAELDGRCKRPEEFQ-MIMEAVQFCNRLVWFYQLSYGFYSFSTFVCAFLL--------------------RQPPYALYLPLDWKRSLQFCVQAWIEFFIMNVTCLHQASDDVYAVIYLYVVRLHVRLLAMRVRRLGKSANDE-------------------------------------------------------------------------------------ANDEATDGYPDERRQEEHCQELQRCIVDHQTLLKLLDCIGPVISGTIFVQFLITAAIMGTTMINIFIFANTNT-KIASIIYLSAVTLQTAPCCYQATSLKLDNEELALAIFQCRWLGQSARFRKMLLYYLHRAQQPITLTAMKLFPINLATYFSIAKFSFSLYTLIKGM-

>gi|379070016|gb|AFC91719.1|

-----------------FLNRPRNILLYLGIWLKP-------ANYVSLYVAYAIIVMLTQYSFVFFEFIYIALAWGDMDAVTEASFLLFTQASVCYKVTRFMINKDNLVFLLSFMEEEVFQAQNERHV-RCLLNQSIMIRRLCLFFLGSALTTCTLWGLMPVVDS----------TGG-----ERIFPFLIWMPVGPEKSPQYELGYFYQMVAIYISAFLFIAVDSVALSMIMFGCAQLEIIMDKVQQIKRVPMSGKVKKQDREQLIQEN-----------------------------------------------------------------------------------------KVLFVECLKHHQAVIRFIESAEDTYHANIFFQLSGSVAIICIIGLRITATTPGSVQFISMLNYMVTMLSQLFLYCWCGNELTIRSEILREVMYLCPWHEQSNSFRRLLWVAMERMKRPIIFKAGHYIPLSRPTFVAILRSSYSYFAVLNQTR

>AaOR35C

------------GELISSVRVILWIYRILGLSREN--------NQSVRYRIYRWVLNIPFLFAYLFAIISALHEENSEILWKDTIFIILTEASMFVKVVTTYYRFQDTFQLLQTSVSEEFSPRCPCEQER-HRRVLRHLNGALMGYLTVSVITACSTAIH-IFEG------------------MHKLPTFSWFPYGPDHGLNYLLIASYQVSGMVMHCALNVSGDIQITYLLAIAGIQLDFLKRRFEDLKEDYLVHLQRRNNRHMQLVEQFVQDIE-------------------------------------------------------------------------------------------------------------------------------------------------------------------------------------------------------------------------------------------

>BmOR-35

------------------------LLRAITLNIDSR------HTARIPFICYVMTVVITLSYFYVFLAWFVFVETRDYLAAMVVLSLGISSEIGTLKFFYTFIYIKKVQRIVREYLECDHMVVPESRFADNVLKTMRNVKKRAILYWVVVIGNGVVYVTKPLFMS------------------GRHHMEDRYIVYPMFESPNYEVAYFLMMFGLCFICYPPANVTVFLIVVVGYTEAQMIALGEEMLRIWEDAV------------------------------------------------------------------------------------YNNKYHTVGALTNSSEKNKIINQRLTEIIKMHTTNIQLLRQVEFVFRSAIAMGYVFLVLGLIAELLGGLE---------NTYLQIPFALIQVLVDCYTGQKVMDASSLFEQAVYDCKWENFDKSNMKTVLLILQNSQKSMRLSVGGITVLGFSCMMSVMKSIYSAYATLRT--

>DmirOr69aA

-----------------------IAFRYFGMAPRFEA-----PKQTVTRQIVFMVGSLCLGYQNLGMIIYWVLFNSQVAKIAEMGSVLALIFAGFLNICALTSKRPQIEAVLAELQEMYPEPRQRFYR---IRHYNDQAVGLMKFTVNFYVIFIIYYNIAPLVLLLCEHLMDSQ------DI-SYRAQSYTWYPWQVYGSPGYSAAYLCQAIGSILGVGFSMSSQQLICLFTTQLQLHFDVMANHLTAIDAKEPTAN----------------------------------------------------------------------------------------------------TANQQLRSLILYHRRILRLGDQVNRLFNFTFVVSLIVSTIAICLTSIATMLL--ELHKALLYISGLIAFVFYHFLICYRGSVVTLASDKVMPAAFYNNWYEGDLVYRKMLLILMMRSTKSYVWKTYNLAPVSIQTYMATLKFSYQMFTCVRSLK

>NvOr254PSE

--------------YKAYSSNIIWCLSFAGLWPHT---------HPVPKRIFSFVSFFSTLAILMSTTNFVFQNGRNVILVAKGLCPAVSFSSVFSKVLLFLLHQDDLVYLNKHLTSKFMIDMEVSEYRKEQLSIMRFFAKFVRAHEASLTAAMSMTSXVPIIIF-----LKHG---------LYVRTYPCLYPFSYEPGLIHWLLYTLEAVGAVCIWSTSIGVDCGFAMYTLQLCGELKILAKKFKEL-----------------------------------------------------------------------------------------------------------KDHKEKLRDCIERHHVIISAKNRLEEAFGLVSIWLAISGALVLCSLIFQISELKNSFFHMGHLCAQFLAKFLQIFMYAWYGNLVAEESLAFLDAMYSSHWPDCDTQFKNDILIVLV--QKPLVLVAKGCMNIKLDMFAKIVKSSMSYFFLLQTL-

>DanaOr22aL3__scaffold_12916_residues_14916600_to_14918400_forward_strand.[97_1467].sp

-------------QSRDAFIYLNRGMWVVGWTEPHEK-----RWTVLYRMWFMLTTLMIIILVPLSMVAEYVQRFRSFGEFLSSLEISVNIYGCSVKCVATIMGYRKFQEARKILDKLDESCQENAEK-VIVRRYVAWGNLVFMIFQVLYSFFVVTNCGGYMLM--------------------GSHAWRMYVPFNPNN--NFYMTNLLEFILMNFVVLMEQCTDVCALTYILLGRCHIILLKDRLTRLR------------------------------------------------------------------------------------------------------DKSEEEHCMELNKCIQDHRLILDYFNVLRPVFSKTIFIQFLLIGIVLGLSMIYIQFFATFWMG-IINLVFMFDVCLETFPFCYVCNLIIDDCQELADSLFQSNWMSADRRYKSTLIYFLHNLQQPIALTAGGVFPICMQTNLSMVKLAFSVVTVIKQF-

>CfOr229

------------------LPMPFALFTYSGYWRPVHF-----PVNSLKYRMYNIYSAFMFLLLFVFYIVDTFILSASLPEFVNKCYLFLTILGVSCKVIHVFICRGKIIELDKMLLEDNCVPRNIEEI-LIREKFDRYISRLTIACEIFNESSAMFGTLAQFYILL----------------KTRSLPVYNWAPFDLSSIYVFLPMLIFQCVALMLWANTSVAHETLTSGMMIQICAQFEILCHRARILPTLLMEAEKNSKSDEDLITRE-------------------------------------------------------------------------------KNSKSDEDLITREIRDLIYHHLYVYKFAHMVNATFTTMMFIQFSIISLVLCMSVYKLSTITSLFTNFAHKFSYLCSMLVQIFLYCWFGNEVILKSIDVSTAIYEMDFTKLRVRVMKDLMIIMMRASKPVKISTGYIVTLSTESFMSILKISYSTYNFLK---

>DbiaOr33a

----------------------WLYWRLLGV-----E-----GDYPFRWLLDLAITFFITTWYNVHLILGLY--NKPAVEVLRNLYFTTECIFCSFKFICFRWKLSDIREIEGYLQDLDSRADNEEERRYFNRSPRSVAQTLSKSYLVAAISSIITAIAAGLFSS------------------GRNLMYAGWFPYDVQASLVFWISFTYQAVGSSMLILENLANDSYPPITFCVVTGHVRLLAMRLSRIGQDEKISMAENKRK-----------------------------------------------------------------------------------------------NKRKLIEGIKDHRKLMRIISLLRRILHLTQLAQFLSSGINIAITLINILFFAENKFSMTYYAVFFAAMFIELFPSCYYGTLMKMEFDKLPYAIFSSNWIKKDNDYKRSLIILMQFMIAPVDIKAGGIVGIDMSAFFATCRLAYSFFTLALSFR

>gi|380014829|ref|XP_003691419.1|

---------------------------------------------------------------MIPTLMYVFLKEKNNKMRLKLMPPIINCSIQFFKYTIILWRRKEIQEGLYAIKHDWIKATE-EER-LIFRSKTKIGRRVVLIVAFTMYGGGLCYMILPLLKG---TIVTAN--NTT----IRALPCPSYFLLNEQQSPIYEILFVLQIIAGIAIYAVICGFCGIFALLVLHAWSMLRILVNKIKKLVDKSDMSEVVLQRKIMDIVEYQM--------------------------------------------------------------------------------------LQRKIMDIVEYQMKIREFLKNIETITEYICLIEMIGSTCMICLVGYCILMENTNTMAIVIYITIQISIIFCVFILCYIGQMLVDENYIVSQASSTVNWYRLSIKNMRCLILIIAMSNHPMKLKAAKMMEMSLITFTDIMKVSMGYLNILRE--

>PbOr158FIX

------MTDKQIKAYHRYQRFLRVLLLICGCWHAP--------SKSGKSTYYWSVCMLPLLFTYGVLLRISYIFRHHLAIMMKNIGVFISTSGTLLKIAIFLINRRYLINYHGTMSDLFEEELKRDEKVRRVMLALRGISTLAYTHSSIMLILIMTYVMPPFVV-IIRGIFHLY-LPT-----DYTLPYSRGYGYFWTNNFLRHFHLLYEWWVIITQAVTNASVDSAFGFYVYLLASTMRAMTFRLTN-------------------------------------------------------------------------------------------------------------KYSDVLRVCVTKHLKLMQCRDMLKRIYSVIILWHIVTNAVLLCAVIYEAMQLPEISIIAFHFMSYSVVKLLQTFIYAWYGTIITNADEDFRNGIYFSEWHNLDRHIRTNILLMMM--QKPMTIKVFSV-SIDVVIFTNLVNTTMSYLFLLE---

>DeugOr83c

---------------------------LLGVDVLA---------PKLEFNYRTWTTIFAIVNYTGFTVFSILNNGGDWGVGLKASLMGGGLFHGLGKFLTCLLKHQDMRRLILYSRSIYEEYENRGESHRTLNSNIDRLLGIMRIIRNGYVFAFCLMGILPLAMLMY---------DG-----TRVTAMQYLIPLPLENNFCYTVTYLIQLVTMVVQGVGFYAGDLFVFLGLTQILTFADMLQLKIDELNQALEQKADNR-AQVRVGARIYG--EEKRQYL-------------------------------------------------------------------------------------------------------------------------------------------------------------------DQVYESICNVTWYELSGDQRKLFGLMLRESQYPHTIRILGVMSLSVRTALQV---------------

>gi|383865815|ref|XP_003708368.1|

------------------VAVTAFFMKLIGLWFAG-G-----RGERWLRCVTLYFTMWMILFGIYLQTTIIYHSLDDVENLIFGLLNLLSILVPLIKILILLPRRKKLFRLIAYMVRNFLEADYDDFETSILTTCKRKCSFFVCSSVCFTELTIVSYVCAPLLVNLFK--------NES----ERVLPFKMYLNVPIQATPYFEIAYITQALASFSIGFSYFSMDNVLCIINLHIAGQFRILQYRLS------------------------------------------------------------------------------------------------QNGLDQKSDLYLKNNASDVFKSCIRQHQALITYCKQLQEVFGLIVLVQVLAFSMLICLDGFQVLL-VDLVQRKVIFFFHLLTTVCQLIMFSYSCDCIIRESVNLATAAFSGPWLQLPANLKKDFIMLIMRSNKPCCISGSGFFIVSLETCTRVITTAGSYFTLLQQ--

>376_Si_gnF.scaffold00330_12820-264637.pep9

-------------------------------WPDSA--------Y-PNLYWFSYVTTVAIVQYYQYTYIFVHFDSNNLWLLMDCLSLSLAYSLAFLKLLVLWWNRRIFHYIVKMIDQDWNEYIINDLHRSIMTSMAGLSRRFANITFSFYAFSAFFLTIGEHLIQSMDDGNQFS-NN------SRELPIKMEFPFDVSKSPIFECLLIGQFLYDMVIAFVVGLINALLVASILHVTGQIEIMQQDLIEISNGK-------------------------------------------------------------------------------------------------------------IKSLICKHQKIITLSENIETLFTHIALMQVLWNTLVMCCTGFVIVVIGEDTTNLIKSVSYYIAIIMEVFVYCFAGEFLSAKSKSIGDAVYESLWYNLPPSDSRIILFMMLRCQKRLTITAGRVFDLTLEGFTSVMKASASYMSVLHAM-

>376_Si_gnF.scaffold00330_12820-264637.pep8

-------------------------------WPDS-----------AYATLYWLIYMATMVIVQYYQYAYVVRHFDDIPLLMDCLGLTLAYTLAFLKLFALWWNRRTFYYIVKAMDEDWKECNVNDSYASTMVGMADLSRRCSNVMISINALAAFFLSIGEHMLQSMGDANKVD--NN-----PRELPIKMEFPFDVSESPIFECFLIGQFLYELVLASIVGMMNALLVSLILHVSGQIDIMRQDINEISYGKY------------------------------------------------------------------------------------------------------------IKGLICKHQKIITLSENIESLYTYIALMQLLWNTLVICCTGFVIIITNDSGTTSIKSVSFYMAITLEVFILCFAGEFLSAKSRSISDAVYESLWYDMPPTNSRILLFVILRSQKRLTITAGKVVDLTLEGFTSIMKASASYVSVLNAM-

>DsecOr46aA_super_1_residues_3641450_to_3647069_forward_strand.[1501_4120].sp

--------------------GQKAFLNIFSLWPQKER-----WWRIIHQVNYIHVIVFWVLLFDLLLVLHVIANLSYMSEVVKAIFILATSAGHTTKLLSIKANNVEMEELFRTLDNEEFRPRGANEE-LIFAAACEGSRKLRDFYGAL-SFAALSMILIPQFALD-----------------WSHLPLKTYNPLGENPGPAYWLLYCYQCLALSVSCITNIGFDSLCSSLFIFIKCQLDILAVRLDKIGRLSTT-------------------------------------------------------------------------------------------------------VEQQLKENIRYHMTIVELSKTVERLLCKPISVQIFCSVLVLTANFYAIAVLSDERLELFKYVTYQACMLIQIFILCYYAGEVTQRSLDLPHELYKTSWVDWDYRSRRIALLFMQRLQSTLRIRTLNPLGFDLMLF-SIVNCSYSYFALLKRV-

>gi|380014930|ref|XP_003691467.1|

--------QKFDNLSEYSIKLSKWYLKPLGAWPASST-----KIERITSQILIVICWCIILFTLIPGILYILFVKQDIYVKLKIFGPLSHWCVDAFNYAILLLRKNDILHCIEHLRADWKLITRTQDQQV-MLRNAKIGRYIAAFCAIFMQIVIFFTCFIGMFKRSI-----HV-DNRT--VELYNLPCPAYIPFDTDP-TIHDIMLGTQFLSAFVVSSSASASFTLATIFTCHVVGQLNIMVIWVNEFVDRLQKENKDN-------------------------------------------------------------------------------------------QKENKDNHINKISVIVEHHLRILSLIARIERITCPIYFMELFKCMMGMCMPSYYFL-AEHNIQNLTIYVMVALSMSFNILLVCCIGEILKEQCKKVGDMVYMTNWYQLPGKDILNLIMIISRSSMEVKITAGKIITMSIYTFGNIVKTVFAYLNMLRQI-

>DanaOr63a__scaffold_13337_residues_9141173_to_9143967_reverse_strand.[501_2295].sp

-----------KKRNYHRIREMIRLSYTVGFNLMDPS-----RCGQILRIWTIVLSLSSLASLYG-HWHMFRRYIHDIPRIVETVSTAFVFLTSIAKMWYFLYAHQQIYDLLRRARLPVAKLLRQKVE-ANMDRCWSSTRRQLLIYLYSCICVTSNYFISSFVTNLYRYFTLPQ-GSY-----DIMLPLPSLYPW-ERKGPYYHIQMYLETCSLYICGMCAVSFDGVFIVLCLHSLGLIKSLNQMVQ------------------------------------------------------------------------------------------------------------------YLRCCIIQYQRVASFAEEINDCYRHITFSQFMLSLFGWGLALFQMSIGSNSSITMIRMTMFLVSAGYQIAVYCYNGQRFTSASEQIGQAFYECEWWTESREFRQLIRMMLMRTNRGFRLDVSWFMQMSLPTLMAMVRTSGQYFLLLQNV-

>ApOr62C

-------------------FFNIKLAKIVGLYQMLDP-----KTKYRGRNIYHIGMACVLLYMCLFLMIYILYYWTNIPISMDYFWKAEITLYVIYKIWFVVQHSNDIWNCLFITRHDFTSFGNQNRD--ILDYWRDRLAWLTIVYATMYFMAMFSYLAITLVFSDEKSLVKNH--GGSIGY-YRQNAMNLYLIVDQTYNAHYYIFYFVEASFGIFIALLFFIFDFLLVTLCFSMCCQMQIICSAFESVGHKSLHDH--------------------------------------------------------------------------------------------------------------------------------------------------------------------------------------------------------------------------------------------------------------

>PbOr162

------MTDKQLQRYRRYQSFLRRMLVICGCWHTST--------KSGKTTQYYSFIVLILLVMFITILHILYQFRHNLVHLMKNLSLLMISLGSILKVSCFLINRRFLIAYHRTLNNLFEKELSQSEKVRTVMLSLRTIATLAYAYSAI-VITLSMTYFLPTYIIIIRGMIHLH-IPSNYTLP-YTRGHGYGYFWTVPPGILRHLHMFFESYLTVINSTTCTGVDSVFGFYIYLLTSTMRAMTFKLTN---------------------------------------------------------------------------------------------------------SPNDKFYDVLKINVAKHKKLMQCRDILTRVYSPIILWHIITNAVFLCSLIFDAVKLSNRSFKIVNFVTFSMLKLLQMFIYTWYGTIITTTDEDFRNGIYFSEWPNLNRHVRTNILLMMM--QKPMIIKVFTT-TVDVVLFTNFVNTTMSYFFLLQSF-

>1_383_Si_gnF.scaffold01629_62255-192132.pep

---------------NHYYYPNKILLSIIGQWPFQ---------SRLEGNIMFAVTFISFVSYAFIEIWGLIAGISDLNIIMENFSPFLVLCYLIINLLYCAFTKDKLKVFLENIEETWKMKPAGPEK-EILQCYAEKSRAFTIQFVIALYVTFVFYTMPSGVARWMYKLFPTN---ETY---RGKFMYRIEHVLDMDK--YFNLLMLHGTIAVFFMVSVAIGVTTVFTLCTLHICALFECIRYNVECIRSTDPVL--------------------------------------------------------------------------------------------------KDDEMYHDLIACIKSYKHALKLVSMLVFNIQERYSLTLRN--INTKINTFQVLMNLNDAKDIVAPLAIYVAQLTHLFFQFWQAQFLLDYSLAPYESM--SNWYYTSERCKKLLLLIMTRTVSPCRITVGKVATLSIESFGVVNKTTLAFISF-----

>gi|383859617|ref|XP_003705289.1|

-------------------------------------------KLSVSTRIYWLIVWLVQLSYLAVCVLGARYVPTEI-SLKDSTVAMAVSLEGIILSLYMNSRQNLLRRLIEQLNDTLNNGDKLLR--SVTTQTVQPLEKVLKLYTIGSIGPVTVWTLLQFIRV-----------TQTDEFYYRVPAVFSAEPFSL---KVFVAGGFFVCLGSTYAIARKVSLDLYMIHMIRLMTAQYRYLRIKFAEILRGKS-----------------------------------------------------------------------------------------------------EMEVKEEMRVLTSHYGTIIKMTSTLKKVLAPNIGVLYINNVFRFCFLSIMLVMTSTEDMEKYLITLYSIGALIQLYMLCFSIQELLEASIAVADDAFYEKWCVYDTSLLRATAIMSFGSKLECKLTSVRSVDLTLSSFMSILNQAYSVCLLFLKA-

>gi|383850520|ref|XP_003700843.1|

--------------SDYSLQLNRWFLKPIGVWPVLTS-----RNEKIVSLTLNVICYCSILVTVVPCLLRIFLEDDDLLAKLKMTGAMSHCFAGGLNYTTLLLRRKEIHYCIKQINSDWRTVKRPDAQQVMLKD-AKFGRSVAAFCAIFMQGGVFCFSVATVFTM----EIVQV-GNETRA--VHTFPCPAYRKIPVDNNFLCEIFLAVQFLCVFIVNSTIVGAFSLAIVFASHVYGQLDILMVWVTEYVNKSEEMHKNRWNQIGVFVE---------------------------------------------------------------------------------KSEEMHKNRWNQIGVFVENHVKILRFIAHIEQVMSPICLSELCQCTLSMCTLCYYIIMEEHDVQNLTSYAMILVSVTFNVFIMCYIGEVLMEQCNRVADVVYMTNWYCMPYRSILDLILIISRSNVVTKITAGKVIYMSIYTFGDVLKTAFTYLNILRQT-

>DbiaOr71a

-------------------RPVRYLTGILEWWRLWPK-----KGRPNWTNWRGYLLHIPFTMLFMVLLWVEAMMSRDIHHTADVLLIGLTTTALGAKMLNNWKYAHVAQGILNEWSSDLFELKSKQE-VDMWKFEHRRYSRVFIFYVMCSAGVIPFIVIQPLFDI------------------PNRLPFWMWIPFDWHQPNLFWYPFIYEAITIPIVCICNITMDGVNWYMMLHLSLCLRMLGQRLSSLRHDDRKLRE------------------------------------------------------------------------------------------------DDRKLREKFLELVHVHRRLKRQALDIEDFISKSTFTQILVSSLIICFTIYSMQMSMQDLPGFAAMIQYLLAMIMQIMLPSIYGNAVIDSANMLTDCMYNSDWPDMNPRIRRLILMFMVYLNRPMTLKAGGFFHVGLPLFTKVVE-------------

>CfOr107

---------------NYAFTTSRQCMRLLGIWPDPNL-----NVFRRPKIEFMLATCIMSVYVFTPQIINTIRAWGNISRVVELFVTANFSMMSIGKMIITRYHGEKLRLLISSMMTDWMTSTSNWERNIMLKL-AKTGRRLNFGYFIAAIGTITFA-FYVRLENV----LQTM-HQ-----PRRYLPYRFDY---IQKSPNYEITTFIQICGGAYAVLGNYSVDSFISILLLHICAQLINLQITLNNLIDKLDNKSI------------------------------------------------------------------------------------------------SSLTFRKGLTAIIIRHEHLIRILGYWYSCVSVIIYHIMYYFTILLLIKKYYFQMIDNFNISIIKLTIYIFLVLTQLYVYCYAAETLSTESINMAFGVYNCKWYNIPAKDAKDLMFIVYRSVISLKLTAGIFGNFSVELFGIAVKTSMGYLSALLTIR

>DereOr85a_scaffold_4770_residues_17426991_to_17429184_reverse_strand.[501_1813].sp

-------------------------IDQMGWRLPPRT-----KPYWWLYYIWTLVVIVLV-FIFIPYIMTGIKEFKNFTDLFTYVQVPVNTNASIMKGIIVLFMRRRFSTAQKMMDAMDTRCTKMEEK-VQVHRAAALCNRVVVIYHCIYFGYLSMALMGALVI--------------------GKTPFCLYNPVNPDE--HFYLATAIESVTMAGIILANLILDVYPIIYVVILRMHMELLSERIKKLRTEVDK---------------------------------------------------------------------------------------------------GDDQHYAELVDCVKDHKLIVEYGNILRPMISATMFIQLLSVGLLLGLAAVSMQF-YNTVMERFVSGVYTIAILSQTFPFCYVCEQLSSDCESLTNTLFHSKWIGAERRYRSTMLYFIHNVQQSILFTAGGIFPICLNTNIKMAKFAFSVVTIVNEM-

>DyakOr49b__chr2R_residues_8228542_to_8232896_reverse_strand.[1501_2855].sp

--------------------LIYMNIKILRFWALL-----------YDKNLKRYVCIGLASFHIFTQIVYMMSTNEGLTGIIRNSYMLVLWINTVLRAYLLLVDHDRYLALIQKLTEAYYDLLSLNDISEILGQVNNVGKLMARGNLFFGMLTSMGFGLYPLASS------------------ERVLPFGSRIPLNEYESPYYELWYVFQMLITPMGCCMYIPYTSLIVGLIMFGIVRCKALQHRLRQV---------------------------------------------------------------------------------------------------------DPQELREEIIACIRYQQSIIEYMDHINELTTMMFLFELMAFSALLCALLFMLIIVSGTS-QLIIVCMYINMILAQILALYWYANELREQNLAVATAAYETEWFTFDVPLRKNILFMMMRAQRPASILLGNIRPITLELFQNLLNTTYTFFTVLKR--

>DereOr33a__scaffold_4929_residues_13479185_to_13483391_reverse_strand.[1501_2707].sp

---------------ESLYKTYWLYWRLLGVEGD----------YPFRRLVDFTITFFITILFPVHLILGMYKKP--KVQVFRSLHFTSECLFCSYKFFCFRWKLKEIKAIEGLLQDLDNRAESEEERNYFDRNPSRVARMLSKSYLVAAISAIITATIAGLFSS------------------GRNLMYLGWLPYDFQATLTYWLSFTYQAVGSSLLILENLANDSYPPITFCVVTGHVRLLVMRLSRIGHDIRVSRSENTKK----------------------------------------------------------------------------------------------ENTKKLIEGIQDHRKLMQIVRLLRSVLRLTQLGQFLSSGINISITLINILFFAENHFAMIYYAVFFAAMLIELFPSCYYGTLMTMEFDKLPYALFSSNWLKMDKRYNRTLIILMQLTLVPVNIKAGGIVGIDMSAFFATVRMAYSFYTLALSFR

>DanaOr67d-1__scaffold_13337_residues_15189964_to_15193905_forward_strand.[501_1822].sp

-------SNKKLTPVDRYLRIVRVIRFFVGFCGNDVD-----PNFKMWWLTYLVLSAIGMFLACTGYIYRGVVIDGDLTVILQAMAMVGSALQGLTKLLVTANLAPLLRHIQYGYEDIYREYGAKSEYIKCLERIIKITWRIMISFFCPCLMNLIAVVSFPIFYL----VVYK----------KKIMVMQFLMPIDEKTDTGYMILSAVHVGLIFFGSFGNYGGDMYLFLFVTNLTLIKDIFCVKLKELNEVVLKKNEYEQMRVML-------------------------------------------------------------------------------------KKNEYEQMRVMLFDLVTWHQKYQNILLTTRRIYSFVLFVQLSTTCISMLCTIACIFLRVWPA-----APIYLLYSATTLYAFCGLGTAVEISNDVLIREIYSCLWYELPVKEEKIIILMLAKAQTEQHLTAANMCPLSMNTALQLTKGIYSFSMMLLT--

>DmelOr71a_3L_residues_15042000_to_15043999_reverse_strand.[294_1535].sp

------------------VRFLTGVLKWWRLWPRKES-----VSTPDWTNWQAYALHVPFTFLFVLLLWLEAIKSRDIQHTADVLLICLTTTALGGKVINIWKYAHVAQGILSEWSTWDLFELRSKQEVDMWRFEHRRFNRVFMFYCLCSAGVIPFIVIQPLFDI------------------PNRLPFWMWTPFDWQQPVLFWYAFIYQATTIPIACACNVTMDAVNWYLMLHLSLCLRMLGQRLSKLQHDDKDLR-------------------------------------------------------------------------------------------------DDKDLREKFLELIHLHQRLKQQALSIEIFISKSTFTQILVSSLIICFTIYSMQMSLQDLPGFAAMMQYLVAMIMQVMLPTIYGNAVIDSANMLTDSMYNSDWPDMNCRMRRLVLMFMVYLNRPVTLKAGGFFHIGLPLFTKTMNQAYSLLALLLNM-

>gi|379070012|gb|AFC91717.1|

-------------------------------------------------------------------------------------------------------------------------------------------------------------------------------------------------------------------------------------------------------------------------------------------------------------------------------------------------------------------KDGCYKKAVLCVKFHQRIIEYVEEVAKIFGLPIFCQCVTSSIVVCMTVYKIT-ITQEPVEMVTLVFYLICVMMELMMYCYPADVLLNKSLQVSDAAYP-EW-SGNIKTAQVLLLTTLRAQRALVVNAGGMFKISLPTAAAVVQTSYTYYALLQQ--

>CpluG2R504O04ELLZL

-----------------------------------------------------------------------------------------------------------------------------------------------------------------------------------------------------------------------------------------------------------------------------------------------------------------------------------------------------------------------------------------------------------------------------------------------------------------AVYSSNW---QGKKSFMVLIVMCLTQRPLAITACNFSIVSLRMFISVLK-------------

>AmOr127

--------------FDNQYRTYRIILKIVGLWPYD---------NSIYVRIQRICVLIYFLIGILIQIFSFVKSEISLRNCIVTFSTTFPTLLFCLRYIYCLTLFSYAKLLFDDICTEEHLLQDTTEIQIQ-TKYLDISSHIIYIFCWLSFICAAASCIFIVNPVILDVIMPLN---------KFRLHYSVIFLS-NDRRKCIDIFLVLNSIIIFFGLLSLICSELFTNIVSYYICRQFHIVSYRIRKIITNLSMSNL------------------------------------------------------------------------------------------------------------------------------------------------LYNALITMDNRVEIFCSTIVVTYHLMTALYNNHYGQLIINSNHDIFNELCASTWYRIPLKAQKLLLFMILRSSMGCEICLSGLFTPSYAGLTSMMSSSFSYCAVIYSI-

>gi|350397298|ref|XP_003484832.1|

----------------------------------------------------------------------------------------------------------------------------------------------------------------------------------------------------------------TQLLAVQQIGASYICPDNFLCVLNLHVVYQFRMLQNTLVNLWSNID-------------------------------------------------------------------------------------------IDERTDIVEYSNECYVMLEKCIPKHQSLIEFSAKLDDIYTLPILSHMVIFSVLMYFDTYEVILADVSPGTRLIFFFHMIGSFTHIIFFTYICNGLVEESTNISTASYSGWWMILPMKIRKDTRIMIMKSMRPCYLSAGGFFPVTLETSTALISSTMSYFTLMR---

>AmOr125

------------DVFDKQYRIYRIILKIVGLWPYD---------KSIYVWIQRICLSMYFLIGVIFQIIVLVKSEITLRNYIVTLSAIFPLLLFFIRYIYYITMFPYAKLLFDDIRTEEYLLEDETEIQI-QTRYLDISSHIIYIFCC-MTFAFIAAAIIFLVNLI---ILDLR-NSLN----EFRFYFDLLFFFDDQS-AYIKIFLILNFMNTLFGLLSITSTESLTNIFSYYVCRQFNIVNYRIRKIIEDLSTPNLSK---------------------------------------------------------------------------------------------------------------------------------------------LLYKALTAMDDRMEILGSTLIVIYHLMIAFYNNHCGQLIIDSNLGIFNELFASTWYRIPLKAQKLLLFMILRSSMDCELRLSGLFTPSYAGLTSMMSSSFSYCTVIYSI-

>DeleOr10a

----------DQQLSVYFFAVPRLSLDIMGYWPGGT------GDRLPRRSIVHFV-ILSIGVITEWHAGLRFLDQQQITLALETLCPAGTSAVTLLKMFLMLRYRRDLSTMSDRLRSLLFDLKSERSDQDIRLSHSVKAARINFWPLSTGFFTCTTYNLKPLLIALVLYLQDRF-DG----F-VWFTPFNMTMPTVLLRSPFFPLTYVFIAYTGYVTVFMFGGCDGFYFEFCAHLSSLFELLQAELRSIFR-----------------------------------------------------------------------------------------------------------LEQRMREVIMRQNTIIELTNFFRERYTVITLAHFVSAAMVIGFSMVNLLTAGGNSLGALLYVAYTVAALSQLLVYCYGGTLVAESSMELCRVMSTCPWQLFKPPQRRLVQLMILRSQRPISMA-VPFFSPSLATFA-----------------

>gi|350425961|ref|XP_003494288.1|

----------------LSITVTSFYMKFVGFWLAND------YVDKRWKNIAMSNTIFFIFVAITIELRDLYFTWGNFEDTIYTACNVATIVLVLLKTFVIFIHNDELLYLINYAKTNFWHSNYDSHEQMIINTSKRICTFLVCSFAFFAQGTVVSFILRPILVN-------YG-KNES----DRIHPFNMWFDDSLMLSPYFEIVFVIQTFSACLVGTCYHCFDNLLFVINLHTASQFRILQYRFSNMCNINDHEHYTTLERSSYTEDKY----------------------------------------------------------------------------------SYTEDKYATFKTYVKQHQMLIQYCNKLENVFSVIALVQVTLFSLIICLDGYLILMEEIAQIKRFTFIFHVMGCMCQLLMFTYSCNCLIQDSECVMNATYKSSWPPLPMMLRKDLMFVMMRSKAPCCLTACGFFAVSLETYTGILSSAVSYFTLLRN--

>AmOr121

---HTSESKKYSKDYEWAVRLNRFSLNVICLWPVEEN-----MRKQSWTKLHIMTCFMLITFVCTIPCLCALKQCNNLMEVTDNLAYSIPLIITTIKFIVVSSKKKVLSLIVNMVAKDWAKLKTDHEKDIMIRR-ARIARIINIFGYILICILIWLLMILPRFGI----TIRYV-TNETDA--KKLFPLPSYYIFDVSETPYFEIMYALQSISLLIAAFCYAGVDNFFGILILHICGQLTNLRFQLANIKESEA------------------------------------------------------------------------------------------------------------LIAIVKDHIRLIRVIELLKMFVEQIINLIITIFKYFFKCLKMYIEEEQFSLFRIIYLICNFTNTFLQTFLYFMAGQMLVTQSEEVHNAAYECEWVSLKYTKAKSLIIIMARSKKPLYLTAGKLFPVTMLTFCNILKISLSYISFLLT--

>AmOr120

-------NQTNTMNIRNYIFINQLVLKFVGFYPIN--------------ILRYVICISCIMFIVIPQIIMIYINWNDLNIVMETGSTLLTILLAALKSIVWIFNRKKLEFFIEFMLTDYWKIIETNVFE-YLQEYAIYAKNITKGYFFSMCNALLFFFSLPIIETL-----TKN-ENLN-NFTIKNFPFAASYPITFYKFPFYEIAYISQILATSICCLMMLAIDSLIATALLHTCGHFTVLKENLKNLDTYIYDLTKTNLKTNS---------------------------------------------------------------------------KTNLKTNSKYI-NKNLYEIKTQIIYIIKHHQLVLWFCDNMEKNFHLILFLQAITSSLIICFVGFQISIALTERSKFLESFSHLIVSLFQLLLFCFPGDILIRQSFNISIAAYSMQWYQLPTFIKDEICMIILRSQRPSFITAGKLYIMHLENFTAILSTAFSYFMMLQSF-

>gi|170045695|ref|XP_001850434.1|

----------------EAVDWIDRLNAFMGICYFD-P-----KVSRLQPRFIWGITTLFIYIYLAFESTYWY--RNDVEKLLLCITTHGFSVQMASKVYTFIINRSQVVEVNTMNRDYYEMETTKSVTQNSHKSSATIAYILLKMTAACYIILVGLIVLGPAIGGA---I-----------VSEKILPFGFELSHSN-AWPAYIMNLAFHVNCGFYVAFLTTSSDSTFILYLLTAVGQIDAISGLLNELNEMLEK-----------------------------------------------------------------------------------------------EKNASEEVISLQLRRIFQLHQHHFAYMRMFKNMFQYMMAITMLYFCMSICLAAFVLIN-------WYMGVLVFCFCSAQIFYMCFLGTALQSKTEFLMKEIDSFSWYRLSVPNQKFAILFLASAQNPILLSAA-MVKLNVATYLQVHKSVYSFLMLLLRIK

>H_TcOr63

-----------DYDLRNAFSLERKLMLVVGFYPKR-D-----NKHEILYWLSAFFNLLISYGQLTTMIIQMVFDRSDLSKLTESLLYFFTHFTFLCKLLNFQYYSKDLIEIENFLTDPIFYGYSFEQLD-IIKAKIRSCAFISNAFRICCTFTCSFYCLVPFIDE-----------SR-----KKILPLPGWFPYDTTN--YYYSTFFVQSLSLFISAYCNTAIDILTWKLITLASAQFEILKENLTKIDYE-----------------------------------------------------------------------------------------------------------KGALVRCITHHAKIVNYTERVEAIFSKGIFLQLFGSVIVICTTGFQLIVVPIPSVQFAVLGTYLCGMTTQVATYCYYGHEVMTTSDAIGMSLYLSNWYASHVKIRKIVMIFLEKTKKPTIVKAGNFITLSLATLTQILRSAYSYFAVLQR--

>H_TcOr65

-----------------YLRVHLTVLQILGIDILP--------VESVPQNLFYTYTALIISTMCLFTFLDMVLNYEDIYRLTFGLCYCVTHVLGTVKMFLMLYLRKKLWGNLTTLEEGIFKPNPPEEL-QIVNDAITMCNRQGYVFYTLVFLIILLYASLANWPYDKHNYFD---GNVTVIVNTKEMPYTTWMPFDYNDSPLYETIFAFQIFSTTVYGFYIGAADAVICGFMMLIKAQFLIVKRELETLIERAQ----------------------------------------------------------------------------------------------ELLDKRTQDYVAKYANECVYHHQELIALCDHAEEDFCYLMLLQFISSLLIVCFQLFQVSTLSPDSVEFFSMVCYLLLMLFQLLCYCWHGNEVQIVSGELSRYAFGINWIIMRESPKKTLLLLMMRAQRPCYFTAGKFSLLSLQTFMTIVRGAGSYFMFLRQM-

>gi|380014928|ref|XP_003691466.1|

---------HYENDIHYTLQMCQWLLKPIGVWPFVYD-----RTSRLISIVLMATCFSSLLFIILPSGHHMFFVEKDVHIKVKLLGPVGFCLSSTIKYCYLGVKGVFFEQCIKHVEKDWKMVQDPSYR-IIMLKYATISRKLITMCAVFLYTGGMSYHTIMQFLS------KER-VNNNYTF--KPLTYLGYDPFDTQSSPTYEIIFCMHCFAAMIMYSVTTVAYSLAAIFVTHICGQIQIQIARLQNLVENKDKKN-----------------------------------------------------------------------------------------------NKDKKNDCDLFALIVHDHVEILRFSKNVEEALREICLTEIIESTIIMCLLEYYCMTENSDAIAILTYFTLLISFTFNIFIFCYIGEILSEQCSQIGTISYEINWYKLPAKKAHNLILLISMSQYPPKLTAGKIIDLSFNTFSSVVKTSVIYLNLLRTV-

>H_TcOr67

-------------DLRNSFSLERKLLLVLGFYPIRDK-----EKHRILHQLSAFLNLLLYYGQLLTIIIQMVIDRNDLSKLTDSTLYFLTLFTFLCKLFNFQYYGKDLIEVEKSLTDPIFYGYSFHKLQ-IIKAKVRSCTLVCLAFRISCTCSCFIYSVVPFIDR-----------SG-----QKTLSIPGWFPYDTAK--HFYITFFLQSLSLFISAHCNSATDTLPCKLISLATAQFELLKDNLRTIDYENSF-----------------------------------------------------------------------------------------------------EETKHALVKCITHHRKIVNYTKRVETIFSKGIFLQLFASVLVICTTGFQLVIVPFGSLKFAIHGIYLCAMTAQIAIYCYYGHDVMITSDEIGTSLYMSNWYASHIKIRKIMVIFLEKTKKPTIVLAGNFITLSLVTLTQILRSAYSYFAVLRR--

>AmOr128

--------------FDNQYRTYRTVLKIVGLWPYD---------NSIYVRIQRICVLIYFLIVVLVQIFSLVKSEISLRNCIVTFSTTFPTLLFCLRYIYCLTLFSYAELLFDNVHTEEHLLEDTTEIQIQ-TKYLDISSHIIDIFCWMSFICVASTCIFMLNPVILDVIMPLN---------KFRLHFSLIFLSNDRR-TYIDIFMVLNLIILIFGLLSIVCSESLTNIFSYYIYRQFDIVSYRIQKIIADLSMPN-------------------------------------------------------------------------------------------------------------------------------------------------LYKALITMDNRMEIFGCILVVAYHLMIAFYSNYCGQLIIDSNLGIFNELYASTWYRIPLKAQKLLLLMMLRSTVGCELHLSGLFTPSYAGFTSMMSSSFSYCAVIYSI-

>AaOR11

------------------LRLTINGLKYYGILLYK-S-----QPFKELNCFRGVCFTASMLAFNVTQYVDLYQVWGNIAEMTANAATTLLFTTTIVRILHFYWNRARFNNAIKVADEGVQHLLRAPEKEI-FWDNVKYMNRLTAAFWICALVTANTMCVYALVQYQTLKSMDLF--NSTEPF-DPPLILRSWYPTDNIV-DSFATIYLIQLYIMYVGQLIVPCWHVFMVSLMLYARTALMALNYKLAHLEQYA------------------------------------------------------------------------------------------------------RCNVRKEIVECIQQLHKIFEYTRELEALTRGAMFMDFVVFSVLLCALLFEAS-STNSFVQIFIDICYIMTMTAILFLYYWHANEIHYQANLLSSSAFMNDWYNYPRSVNRHLITFICYSNKPLDMKAY-IVSMSLDTFLAILRASYSYFTILKQ--

>DeleOr56a

------------------FRNHLCGFRLYGYVAST-D-----QKRPWFSLVRCIFFTVSLWMCCALMLARVFRGYENLNDGATSWATAVQYFAMSIATLNAIVQRDSVIGLLRVAHADIQNLISEADDREMELLAQVYTRNITLTLCGPSFIAGSMAYLDCIY----RTVFLPK-SNSSAVQRGEEQPILMFFPLEVCD--NFVVGYIGAWYALSLGMTAIPMWHTFITCLMKYVNLRLQILNKRVEEMD-------------------------------------------------------------------------------------------------------------KQLFRDFVEEHIRIRNLVHEIQHLIRVPVMLDFIIFSISMCFLFYALALGVSSKMDYFFIGIYIFVMAAILWMYHWHATLIVECNDKLSFAYFSCGWYNFDVPLQRTLLFVMMHAQRPMNMRAL-LVELNLRTFIDIMRGAYSYFNLLRS--

>DvirOr74a_scaffold_13049_residues_5965447_to_5967813_forward_strand.[501_1867].sp

-------------------------------WPLEDS-----RRMVFIERLLIFVGFIIFCEHNEVDMHYVLAYRNDIDKMLTGVPTYLVLFEMQIRGFQLAATKDSFKQLLQKFYADIYVSQETEP---VRFARIQRQMLGTRLNSVVYLMALFNFSLVPVQNVIYH---------------RREMLYQQVYFFDNTKLQYYIPLICANYWVGIIITTMIFGELNILGELMMHLNARYELLGRDLKQV------------------------------------------------------------------------------------------------------------QYRCALIEILRRNAALNRFGQQMELEFSFRIFIMFAFSAALLCAMGFKAYT---NPAGNIVYIVWFTAKFCELLALGMLGSILYATTDQLSVMYYCCDWEQVNVKLMKLVTMAIEINSRPFYLTGLKYFRVTLVAVLKIIQGAFSYFTFLTSMR

>ApOr34C

------------------VAINLELMKRSRFYHFNPN-----GTKIFNCNAYRLLLFLYIVNCIVVFTLGFFVEMDDFTDLFVAIFVLINFFLCYWRICVFMYNVNAIYDVLSVSRFDLLKSKHCCKNVNVLNDYRDRTIKITNYFFLFSSTVMSQWIIYPLVVI--AFTMPED-EYG-----RFQNIMNLRYPVSTYTYQYYFIFYLMEVMVAIFTMYAMIFPDILLMSVCWAIIAQQEVLTQAFKNIGH--------------------------------------------------------------------------------------------------------------------------------------------------------------------------------------------------------------------------------------------------------------------

>DkikOr69aA

-----------------------------------------------GRKALLVLGIICAISQIIGVFMYWYIYERDTAAMAEMCGSLMLTMVGFSNIYALIKNHSQIEAVLEELQKLYPGPRERHYR---CQHYYEIAIFIMKCEFRFYVIFYIYYNSAPLFMLLWENF-KEG-QEL-----TYRTQTNTWFPWRVHGSLGFELAYLSIVMASFIGVGFSLVTQNLVCIFTFQLKLHYDAMASQLQNLDARHPQSKE------------------------------------------------------------------------------------------------------------------ILQIADQFNIILNFNFMSSLIGSTIAFCMTSVAVLL--LDSASAFKAFSGLVAFVFYQFIICYLGTEVN----------------------------------------------------------------------

>DereOr7a_scaffold_4690_residues_10433363_to_10435763_reverse_strand.[501_1901].sp

--------EEKAPESRRAFRNLFNCFYALGMQAPDGP-----TTSSTWRRIYCCFSVVMYVWQLLLVISYRYMGGMEITQVLTSAQVAIDAVILPAKIVALAWNLPLLRRAEHHLAALDARCKEQGEFQ-LVLDAVRFCNYLVWFYQICYAIYSSSTFVCAFLL--------------------GQPPYALYLPLDWQRSMQFWIQAWIEFLIMNWTCLHQASDDVYAVIYLYVVRTQVQLLARRVEKLGRDDT------------------------------------------------------------------------------------------------PDERRQEEHCAELQRCIVDHQTMLQLLGCISPVISRTIFVQFLITAAIMGTTMINIFIFANTNT-KIASIIYLMAVTLQTAPCCYQATSLMLDNEQLALAIFQCQWLGQSARFRKMQLYYLHRAQQPITLTAMKLFPINLATYFSIAKFSFSLYTLIKGM-

>DkikOr49a2

---------------------SHMLFKTLGYDILAT------NATKWPYIIYFYLCIVCNFYAAFYVTLRFLQWERSESKLMRHALHFFYMFSAEVKFATFVKYRKRLRSLNNQLKDLYPTDAKQQEV-YEVN--RYYLSRTTRIVLSFYYSVMALMMVTPHLQSCI---THFV-VKR--DF-PYLRIFPTQLSFSSETPWGYVFAYFVDFTYSHLIVNFTLGTDLWMICASSQICMHFGYLA--------------------------------------------------------------------------------------------------------------EREEEDCAFLSKFVKRHQLILXLHNEVDQIFGLLLASNLFTTASLLCCIAFYTV-VEGFNMEGMSYMTVFFSVVGLFYLVSSHGQNVIDLSTSIAVAAYDQNWYEGSVRYRRLLMLIMAKAQRPSVISASSVVIISLDTLKTLMTITYRVFAVLRQ--

>AmOr29

------TQDDYKRKTNLSIQWNRWLLTPIGAWPNLRS-----RIGKCYSLLISIICYSLIGFMLVSCSIFLMVEINNIYNKLKMVGPLSFFVMTIMKYYFLLFHENDIREGIERIEWDWKNVKHQEDRNIMI-TYANYGRKLAFICFFFMLCAFIFYFLIQPFGG--GKIVDGN---LT----FIQLPFPISILIDVRDSPYNEIMLSIQILTGIVMNAIRSAICSVAAVFAIHACGQMQVLMNWLNHLVEGRSDMS-----------------------------------------------------------------------------------------------SDMSKKIDDRIANIVIQHDRILKFLALTERALQQISFVEFLGCTANMCLLGYYLI-VEWNPIVSFTYIAIIASITFNIFIFCYIGELVAEQTEKVGEVAYMIEWYRIRGKKKLCCVLIIAMSNSSIKFTAGNMVELSIYTFSDVVKTSVAFLNMLRAL-

>AmOr75

-----------------SIIWTSFLMKIVGLWLAT-D-----RNEQRQRDFALIYTVGTLFISICIAFRDIYYSWGNFSNSVFICCNILYVAIVLLKISVLYAHREEFFNLIAFTQKNFWRLYDDPQELLIITG-CKKLCNFSIVLIFCAQGTCAGYMVTPLIENIGK--------NES----DRALPFNLWIDFPVGLSPYFELLFILQILCVYHVATCYICFDNLLCIVNLHVAGQFRILQHRLKNLGNAI---------------------------------------------------------------------------------------------------PRYEKCCYERLKDCVVQHQTLIEYCKRLEDIFTVMVLGQVMFLAVVICLVGFQLFLADTSASKKASLVLNLGGTFFQLLIFTYSCDNLIRQSVNVGNAVFSGPWVNLPMSVRKNLIIVIMRSQKICCLTAGKFFPVSLETSTAVLSTAISYFTLLKQ--

>LhOr233

--------------LNRYYSLNRLLLSIIGLWPYQ---------SAINAWILRVLTMAILIWFLIAQTAKIYMTELTTDFVLDVLPIIIPHVGIMVKFLNRIVNMNEMKDLLDRIKVDWERTSSQDEI-KIMQTYAAATRKMSISFSFYVFIGTSAYIFMTIIPQILDVFLPLN-ESR-----SREHPFHVEFFIDDKK--YFYVIRFQLYFILAFLMEVILANVIIFVIYMQHASGMFAVLGHRAEQLFDVPSPKARSV---------------------------------------------------------------------------------------------------------FIEDHRKVIQFVDVIQSCYSLLIVAEFLVLIILIGITLVQITKFSGT-DRPVRSIAYVIGQLIYLFMSSYMGQHLIDTSAQMSMKVYFAKWHNMSIWKQKIMLFIMMRCMRTVSLNLYSVCPVNLENFSAIVQQAISICMLLRHM-

>DpseOr85d__Ch2_residues_23351656_to_23353999_forward_strand.[501_1844].sp

------AEKSEPITTERFLRYANIFYLSIGMEAYDHQ-----GRRKMIELILRCIFIALILNLNAVLLIYVFLAIGNFLEATMNLSFIGFVIVGDLKVWHIWRKRDQLTNVVREMEKLHPKEGHHQK-AYDVESHLSGYSRYSKFYFGMHLVLIWTYNLYWAVYYLVCDFWLGI-RH----F-VRMLPYYCWVPWDWSTNSSYYLMYVSQNMAGQTCLSGQLAADLMMCALVTLLVMHFIRLGRGIEE------------------------------------------------------------------------------------------------------------------LQAAVVYHQRLLQLCHNINEIFGVSLLCNFVSSAFIICFVGFQMT-IGGKIDNLVMLVLFLFCALVQVFMITTYAQRLLDASEHIGEAVYNHDWFQADLPYRKMLIFMVRRSQQASRLKATIFLNVSLVTVSDLLQLSYKFFALLRTM-

>AmOr21

-----KIDQDYKSNVNLSIKYSRRISKMIGLWPIFDS-----TIHKFLRMLYNTICYCLLMFMIVLGWMYIAFEVKNIYDGLKFVSLMSFCMLSITKYHLINIHKDDVRECVKRIEWDWKNISYSEDREIMLMN-ANFGKRLIIVTTTVTYSGFVFFYIAIPMKI--GKIPAPD-ANIS--FIPTMFPFP-KYIADVRYSPINEIVFFFQFMCGFLVHGVTSSACSLAAIFTVHACGQIQVMMIWLEHLIEGRL--------------------------------------------------------------------------------------------------------VDQRIAKIVSQHVRILKFLSLIEKILQQVSYMEFLECTVNVCLLGYCAIIESNHLTEVVTYVIILITIIFNIFVFCYIGELLADQSRKIGEVTYMIEWYRLSGKKKLCCVLIIAMSNSSMKLTAGNLIELSMSTFSDVVKTSFAFLNVLRTL-

>AmOr20

---------EYQKNVNLSIQYNRWLLKPMGLWPNSY------TSKDYPYWLINIVCYCLISFLFIPCTLYLFLEIEDFYGKLKQFGPLIFCMMAFVKYYYLIFHKTDIRECVERIKWDWRNITYAKDREIMIM-YANFGRKLVMVCTFFMYSGFAFYYIAIPISV-GR-----V-KTDNLTF--VPLVFPFRFIVDTRYSPTNEIVFSIQLMAGALMHGITSAACSLVATFAVHACGQMQVLMNWLQHLIDGRLDMDE-------------------------------------------------------------------------------------------------DERLDGRIADVIRQHVRVLKFLALTEKTLQQISFTEFLGCTLDICLVGYYVIMESNDVTSVITYIILLISLTFNIFIFCYIGEIVAEECRKIGEISYMIEWYRLMGNKKLFCILIIAMSNSSIKLTAGNIVNLSISTFTDVVKTAVTYLNVLQK--

>AmOr23

-----KINQDIKNNINFSIKYSRLILKMIGLWPIFDS-----TIHKYLQWLYNVICYSLIMFIIISGWIYISLEVENIYDRLKFVSLMSFCMLSITKYHLINIHKDDVRECVKRIEWDWKNISYSEDREIMLMN-ANFGKRLIIVTTTVTYSGFVFFYIAVPMKI--GKIPAPD-ANIS--FIPTMFPFP-KYIADVRYSPINEIVFLAQFICGFLLHGITSSVCSLAAILTVHACGQIQVMMVWLKHLIDGRLD-----------------------------------------------------------------------------------------------------NSIDQRIATIVNQHVRILKFLSLIEKILQQVSYMEFLECTMNVCLLGYCAIMESNHLTEVITYLILLITIIFNIFIFCYIGELLANQSRNIGEVTYMIEWYQLFGKKKLCCVLIIAMSNSSTKLTAGNLIELSMSTFSDVIKTSFAFLNVLRTL-

>AmOr22

-----QTNHDYKRNVNLSIQWSRWILKPIGLWPNSSS-----TTGKYLYRLINVICYSLISFLSIPCSLYVILEVEDIYNRIKLFGPLSFCVMAFLKYHLLILHKDNISECIKRIEWDWKNITYSKDIEIMIT-NANFGRRLVIICTFFMYSGFAFYYIAVPISV--GKILAED-DNIT----FIPLVFPFRFIIDTRYSFINEIVFSIQLIAGALMHTITTAACSLAAIFAVHACGQMQVLSNWLKHLINGRSDMYN----------------------------------------------------------------------------------------------SDMYNNVDSRIASIVSQHVRILKFLALTEKALQQVSFVEFLGCMLNICLLGYYVITESSHLTSAITFFILLISLTFNIFIFCYIGELVAEQCKKIGEISYMVDWYRLEGNKKLCFVLIIAMSNSSIKLTAGNMVELCLTTFSDIVKTAVAFLNVLRTL-

>AmOr25

----------DGKKANLSIQWNRWLLTPIGAWPNLRS-----RIGKCYSLLISIICYGLIGFMLVSCSMFLMVEIKKVYNRIKMIGPLSFFLMTFMKYYLLLLHENDIREGIECIEWDWKNMKHQEDRNIMI-EYANYGRKLVLICTFFMYSAFAFYYLVLPFSV------GKI-EDGNLTF--IQLPFPSSSLADIRYSPYNEIVLSVQILTGVVMHAITSAACSIAAVFAVHACGQMQVLMNWLDHLVDGRSDM------------------------------------------------------------------------------------------------SDMSKAIDDRIANIVIQHDRILKFLALTEKALQQISFVEFLGCTANMCLLGYYLI-VEWNPKEIILYVALIISITFNIFIFCYIGDGVAEQCQKVGEMAYMIEWYRLTGKKKLCCILIIAMSNSSVKFTAGNMVELSIYTFSDVVKTSVAFLNMFRAL-

>AmOr24

----TKTDHDYKRNVNLSIQWSRWILKPIGLWPNSSS-----TTGKYLYRLINVICYSLISFLSIPCSLYVILEVEDIYNRIKLFGPLSFCVMAFLKYHLLILHKDNISECIKRIEWDWKNITYSKDREIMIT-NANFGRRLVVICTFFMYSGFAFYYIAVPISV--GKIPAED-DNIT----FIPLVFPFRFIIDTRYSFTNEIVFCIQLVAGVLLHTITTAACSLAAIFAVHACGQMQVLSSWLKHLINGRSDM-----------------------------------------------------------------------------------------------RSDMYNNVDSRIASIVNQHVRILKFLALTEKALQQVSFVEFLGCMLDICLLGYYVIMESSHLTSAITFFILLISLTFNIFIFCYIGELVAEQCKKVGEISYMVDWYRLEGNKKLCFVLIIAMSNSSIKLTAGNMVELCLTTFSDIVKTAVAFLNVLRTL-

>AmOr27

-----AITEEIKTNSDYSLQLNRWFLKPIGAWPLFST-----KFEKTVSLILNIICYAIVILCATPSLMQIILAEESFYLKLKTLGPVSHWFVSTVNYTALLMKSKDIRYCFEHMEADWQTIKRMEDQQTML-KNAKFGRYVAASCAIFMQGGILCFCFVTILTT----ETIQV-GNETRV--LHVLPCAVYKKVNVEENSINIFMLCFQFVAAAIANSSTVGIFSLAAVLAAHAYGQLSVVMVWITEFVNQSRNQKKT-------------------------------------------------------------------------------------------SRNQKKTDDFKEIGIIVERHLRVLNFITYLENIMNRIYFLELFRCTMIICIVGYYILTEEKNVQNLTTYFMMLLSICFNIFIICYIGEILTEQCMKIGEVVYMTDWYYLPDKTILNLILIILRSTVVVQITAGKLFNMSIYTFGDVLKTAFAYLNLLRQM-

>AmOr26

------MPVSYARDYEYSIQVNRWLLKPIGAWPNLTT-----RTEKLLVKLLNFICHSLIIFTVMPCIMYIFYEDESLKTRMKAIGPTSHWLMGELNYCCLLMRAKEIVYCIEHIKYDWKTVRRARDRELMIK-NAKLGRFIACIAALCMHSGIMSYTVITGFKK----ITFQI-GNDSYS--MYRLPCPFYTNLDVRFSPMNEIVFALQLLSGFISTSVTVGACGLAAVLAMHACGQFNVVMIRSDKLVKDNNEKKQD---------------------------------------------------------------------------------------KDNNEKKQDEQTLHKKLGFIVEHHLRTLSLVWYMEKVMNMICLVELVGCTMNMCILKYYFLT-EKSKTILGIYAIVYASMVFNIFIFCYIAEIVTEQGKKVGEKFYMTEWYQLPHKTALGLVLIISRSSMVIKITAGKLIQISIATFAAVFKASFAYLNMIRT--

>GPRor30

----------QIDDTREILPIGCRLLMLCGCPRSA----------RVNRRFW----LLGVFFFVFGQIPRFLIKIDEPIALVRVGAEIVYSTYMFLQLIALYARRSDLYRLIDTLQKCVENPYPDDVRAFI-LITRDTINKSSVMYSKCFFAVCISYIIMPFMAT-SAVVVRNR-RNQTGEREEYVMPTEMKYYLDIRFNLHYSLYFGAVSGLSVIGSLALCTKDVMDFSLIRTASMLFQATAQQIRNLP--------------------------------------------------------------------------------------------------------------AKLEAIIHSHRSTLKCAAQLQNALNPALLIQITFCTAIWCLMLFYILL-LGFTSKVMNVCLLLLVLTCETYSYCQLGTQFTSNAEEVLDELQQLARYDQSIPIQKQIYFMIHRSQTRIELTAGKLFPVNIAQFSEIVKKSYSYYLVLK---

>GPRor31

---------------VDFFRVQSICLRAIGIARTD----------SFRGRVLFAVSFFTVLVMMLGTVMFAFKHIDQIMLLCDCLGPTFTAYLGLVRQYNLLLHRSELWSIVDEFAALKHSLQSSEI--RIVQKYNRIDRFLAWAYLITAMSTGVLFVGVALVLVFL----SE----KS----DWKLPLLMDFPFDVKHPVTFTIFFVWCSVAIFWVVLDCVACDSTFGTFSSCLVAHFVIIQERFEGLRF------------------------------------------------------------------------------------------------------------NRELKKLIEHHKYILRISDRVINAYKNVILNQLLISSVLLCMLGFQLVISVGTNIMVV-YVAYGMAITIQVTYYCYYGSQLYYESTQVHDAVFKSKWYDASVATQKMLINCMMRAKKPVNAKSG-FTQASLPTLNAVLNSAGSYVALLMSL-

>DsimOr33a__chr2L_residues_11741184_to_11743384_forward_strand.[501_1701].sp

------------------YKTYWLYWRLLGVEGD----------YPFRRLVDFTITSFITILFPVHLILGMYKK--PKIQVFRSLHFTSECIFCTYKFFCFRWKLKEIKAIEGLLQDLDNRAESEEERNYFDQNPSRVARMLSKSYLVAAISAIITATVAGLFSS------------------GRNLMYLGWFPYDFQATPIYWISFSYQALGSSLLILENLANDSYPPITFCVVSGHVRLLLMRLSRIGHDIRITRSENT------------------------------------------------------------------------------------------------ENTRKLIEGIQDHRKLMEIIRQLRSTLHLSQLGQFLSSGINISITLINILFFAENNFAMLYYAVFFAAMLIELFPSCYYGTLMTMEFDKLPYAIFSSNWLKMDKRYNRSLIILMQLTLVPVNIKAGGIVGIDMSAFFATVRMAYSFYTLALSFR

>DyakOr43a__chr2L_residues_15816434_to_15821075_reverse_strand.[1501_3142].sp

--------------------INVRMWRHLAVLYPT-----------PGSSWRKFAFVLPVTAMNVMQFVYLLRMWGDLPAFILNMFFFSAIFNALMRTWLVIIKRGQFEEFLDQLSTLFHSLLDSTDERGILRRAEREARNLAILNLSASFLDIVGALVSPLFRE------------------ERAHPFGVALPVSMTSSPVYEVIYLAQLPTPLLLSMMYMPFVSLFAGLAIFGKAMLQILVHRLGQIGGAEQPEEERH-----------------------------------------------------------------------------------------------EEERHHRLTSCIAYHTQVMSYVWQLNKLVANIVAVEAIIFGSIICSLLFCLN-IITSPTQVTSIVMYILTMLYVLYTYYNRANEICLENNRVAEAVYNVPWYEAGTRFRKTLLIFLMQTQHPLEIRVGNVYPMTLAMFQSLLNASYSYFTMLR---

>gi|167876904|gb|EDS40287.1|

-------------RFESFIRVPEIFYGMIGITRYGEN-----TTKARLKQLFFWSSYANTIFCLIIEHIYFFRNFTNFLELTALAPCIGFTALSIVKIMTIKLNEAKLNGILDRLKELFPVTHLEQTRYRTH-QYNLESQMVMKSFSILYMILIWIFNLLPLVSMLVNYLMSGV-L-------VRELPYFMWYWYDWHREGLYEITFFHQNWGAFDSAVFNLCTDLMFCAVILLMCLQFDIIAVRLRAAKD-------------------------------------------------------------------------------------------------------------QELISCVQLHQTVLELGDQLESIFSPSILVNFLGSSVIICLVGFQAT-SQISAFDLFKFVLFLISSLVQ--------------SSQIPYAAFQGEWYLADVRYRKSLLFLMARAGKWQKLTAMKFSVVSLASFTGILSTAFSYFTLLKT--

>DanaOr92a__scaffold_13117_residues_595500_to_596900_reverse_strand.[10_1348].sp

------KEKREVRTFEDLTRFPMAFYKTIGEDLYSDR-----DPRRYLLRLYLVLGFLNFNAYVVGEIAYFIVHITTLLEATAVAPCIGFSFMADFKQFGLTVNRRRLVRLLDDLKAIFPTELE-QQRAYHVSYYQKHMNSVMTLFTILCMSYTSSFSFYPAIKSTIKYYFMG-----SEIFE-RNYGFHIQFPYDAETDTVYWFSYWGLAHCAYVAGVSYVCVDLLLITTITQLTMHFSYMADCLEAY-----------------------------------------------------------------------------------------------------------EENIKYLHDLVVYHARALDLSEEVNSIFSFLILWNFIAASLVICFAGFQIT--ASNVEDIVLYFIFFSASLVQVFVVCYYGDEMISSSSRIGHAAFNQNWLPCSSKYKRILKYIIMRSQKPASIRPPTFPPISFNTFMKVISMSYQFFALLRT--

>GPRor36

---------------------------------------------------------------------------------------------------------------------------------------------------------------------------------------------------------------------------------------------------------------------------------------------------------------------------------------------------------TKEKNGQADIWTSFERNTARAVRRHETFLWQLGQLKPFLQTTFLVMFYSAALFLAIGTFMIS-ANGTTTYGVILSGFLFALLLECYWCCQLVDRLNEVNTQIGILLYSLDWPVEYRQARSSLLIMMSKTQKSLGIRCGGMFEMSSEAFASLVKLTYTMLMFLRD--

>GPRor37

------------GDYIRPVRITVWTWKICGLYNGKP--------QTTRYRVYRAVFNIFLMVVYLFTLSLNVFVMQTFEQLLYIMYIVFTEIVMALKAIVTYYKFDQLCDLYRQTLGSDFKPLDAEEE-QLHRKGVASINRYLYPYLVTTNLAVASSCLYLL-QD------------------DYRLPYFPWILIGPTKRLNYGLLFAYQVIGMYLHMLINVAIDVQLSYLLGMISIQLDLIGKRFRSLHT-------------------------------------------------------------------------------------------------------TSEQFRESFVGLMNHHGKVQRMTAEIEQLFSAAYFAQFGSSGLVICASAFKTSSMFNLYETAIQNLLYMLSMIFQMFLPCRFGNEVTRKSHLLRTSIYSSRWYEMGLQERKTLRMLLQRMNKPLTLKAFYFFNYNLQAFTTTLNMAYSLYALLQ---

>GPRor38

------------------FKFQRKILLIFGCWPPD-R-----LTRRWYVKVLIAVNLITLAICIVGEFRYCLYRNGSLIETIESICPTVARFSGLLRMCWFLRNEHKIKSALNSVVHLIKNEHPRET-GY-TNRITARDQTITKVLFHSSFITAILYGIIPYFMMAYN-----W-FQGQYPL-VKLLPFKVVLPFDSQDPTLFVLTTIFLNYASVPTITAMTSTDALFSGVCLYMDGQFQAIRLELEALAETVDKYT----------------------------------------------------------------------------------------------------RINLELRRINKRHQTIIDVVSEVRHAFTPTILIVHICAAFMICVISMAMFLAEG--INKLTYMPYTFTVLMLLFMYSYGGTVVRESSEAIQTVAYGFPWYQFDRNTRHLVQMMMVRAKYGCNVDV-PFFRTSMATFSVIVRSAMSYITLMKSF-

>gi|380014590|ref|XP_003691310.1|

----------------YSLKLVYPLLKILGAWPKSTP-----PSSTILKWCLIFTCYLIQLMVLIPGILYIFLKEANLSGKIKMFVPHMNGITQVCKYTILLRQIKEFNIILKEVRRDYSLATDKNM--WIFTTRACIGHKMMIAIAITMYTSGVGYMILPFLKG--RILLPDN-TT------VRLLPCPGYYMFNEQVTPNYEIIFIIQVLGGFLNYTTLCGTTGISTMLCLHTCSLLKILINKMNDLTCQS------------------------------------------------------------------------------------------------------ETVVRKKLADIVEYQMKIIDFLNHVEQLTSYLYFCEILEYVCGACVIGYCLITENSNGAALIVYFILEFLCIFCTLTICYIGQLLIDESDRVRRISITLDWYRLPVNEARGLILIIIMSNYPIKVTAGKIVDISLITFTDIVKTSVGYLNILRTV-

>H_TcOr165

-----------------------------------------------------------------------------------------------------------------------------------------------------------------------------------------------------------------------------------------------------------------------------------------------------------------------------------------------------------------STQNEICGTLRLCISHHIALKRWMNKLANSVDTAMPVFILLGALSTIAVSFFVLNTSTSVILKIRLATITVCNLIVVATFAELGQIFSDQNNSLLEHLMDSPWYLWDVENRKTLLMFMANCMKPKTFSWGGI-TLDYSFALSIFKTSFSYALVLYQLR

>DficOr59b2

---------------------LYRAMKFIGWLPPK---------SGILRYIYLFWTLMTWSTTYLPLFLGSYMKSFSPGEFLTSLQVCFNAYGSSVKVAITYSMLWRLIKAKDLLDQLDLRCTTLEEREKIHRV-VARSNHAFLIFTFVYCGYAGSTYLSSVLS--------------------GRPPWQLYNPFDWREGLKLWVASTLEYLVMSGAVLQDQLSDTYPLIYTLILRAHLDMLRERIRRLRSDESLS---------------------------------------------------------------------------------------------------EAESYEELVKCVLDHKVIVRXCAIIRPVISWTIFTQFLLCGLVLGLTLINVFFFSDLWTG-IASFMFVITILLQTFPLCYTCNLIMEDCESLTHAIFQSNWVDASRRYKTTLLYFLQNVQQPIVFIAGGIFQISMSSN-IVAKFAFSVITITKQM-

>DficOr59b1

----------------------YRGMWIIGWIPPK---------SGILRYVYLFWTCVPFAFGVFYLIISYVQEFKNFGEFLTSLQVCINVYGASVKSTIFLWRLRKTEMLLDHLDKRLRNESDREK---I-HNMVARCNYAFLIYSFIYCGYAGSTFLSYALS--------------------GHPPWSVYNPFDWRDGLSLWIQAIFEYITMSFAVLQDQLADTYPLMFTIMFRAHMDILKDHVRNLRTDPDLSEEEN----------------------------------------------------------------------------------------------SEEENYQELVNCVMDHKMILRCCDMIRPMIGRTIFVQFALIGSVLGLTLVNVFFFSNFWKG-VASLFFVITILLQTFPFCYTCNLLIDDAMDLSNEIFQSNWVDAEPRYRSTLVHFMHHVQQPIIFIAGGIFPISMNSNISVAKFAFSIITIVRQM-

>gi|284009958|dbj|BAI66614.1|

----------------KYFKSIRTFMVAPGAWPAEIG-----EKISVLVIFHR-ATLPYHTSLIVVGFYYIWMHMDSFLDLGHMIITALLGTLTAVRSILPSLYHMLLLKFINVMHLMHSTNKGPYYN--QMNDTDKVCSYYTKFSLVITFISCLMFNLIPFYNNVTNVFIFKT-ENYT-----LEFALYYQYPIDPSD--YFTITSIYNVYLSYNCAIMVFGLDLILFLIIFQIIGHVYILRYNLENFR---------------------------------------------------------------------------------GDILKYKMNESIT---SEMFDAEENKEVRFKLAECIEHHKEIIGFPDELSALFGPILACTYLFHLVGCSLLLLECS--EGGYGAMLRYGPLTLLIYGQLIQMSVIFEMLGSETEKLPDSAYFLPWECMDNSNRRTACIMLHKMQYKISLKALGLAAVGVSTMTGILKTTFSYY-------

>gi|350422506|ref|XP_003493184.1|

------IEADSNSNSDYSLQLNRWFLKPIGAWPSSPT-----RLEKIISFLLNAICYSTVIITAIPSVLQMILEDESVNSKLKSLDFLSHLIVSIFTYSVLLLHNKDIRRCVEHMKADWRAVSRKEDQH-VMMKNAKFGRYVAAFCAIFVQGSVLCFCFVTALNTL-E---IQI-GNETRI--LHVLPCAVYKKVNVDESPTNEFMIFLQVWSSVIANFSTIGIFSLAAVLAAHACGQLNVVMLWIVEFVNEAA----------------------------------------------------------------------------------------------------------TKIGVIVERHLRTLNFISYIEGVMNKICFLEMLRCTMEICVIGYFIVSEEHDIRNLATYFMMFVTICFNIFIICYIGELLTEQCKKIGEAVYMTNWYYLPGKTILDLIMVIARSNVVVQITAGKLVHMSVYTFGSVVKTGFAYLNLLQQM-

>CpluG2R504O04D7PBQ

-------------------------------------------------------------------------------------------------------------------------------------------------------------------------------------------------------PKKFFFLYTHGAIVTPIALIVLVGFDSLYSGFVLHASSMFNILGRRLENLSESNNTKKNLINR---------------------------------------------------------------------------------------------------------------------------------------------------------------------------------------------------------------------------------------------------------

>DmojOr42a-2_scaffold_6496_residues_2666400_to_2668200_forward_strand.[207_1569].sp

---------------------LLNCIFLMGIRTPP--------RGCCLFLIYVAWSFALNLCSTFYQIGYVHLSEFSPGEFLTSLQVAFNACTCSTKVIIVWIFVKRFDQANALFDELDKQVLEPAERGRIHR-AVSQSNRIFFVFMSVYIIYATTTFISACIN--------------------GVPMFQNYYPFDWRASQQYWLQSCLEYFTMFGACFQDVCVDCYPINYILPLRAHMANFAERLRGLGHDPQESSEER---------------------------------------------------------------------------------------------ESSEERYEKLIECIKHHKIILSFCDTLRPIISGTIFVQLLVVGLVLGFTIINIVMFANF-ASRIASLCFMTAVLLETTPFCILCNYLADDCMKLADALFESNWIYQDERYKKTVLLFLQSLQKPIVFIAGNVFTISVATNLNATKFSFSVFTLVKQM-

>gi|380022248|ref|XP_003694963.1|

-----------------VISWSKRLLSLSGLWPDN------------RNDVRFFLYITYVVIFTWLEIVTLVQNIHDLERTLKNITLSFPTILIVLKAVMFRMNMHLVLPLLTVVKRDVNEYRSPEER-RTVVWYNVAATLFSTSSALSLFFVPTLFYAKPIIGC----LLSKY-NNCT--L-PFELPMKVNNVYEITKLQTYALFCVYLIPTSTLLTIGATGADSLLVTLTFHLCSQLSIVAYRMRNVD----------------------------------------------------------------------------------------------------------KIYFPKMKALVERHTELLRLANILAKTFSSLMFVQTLGLIFSLCIVVYQLLMTSGEDMNTIHFIIYSCAVILLAFCYCFLGECLINESSEVQMACYFTNWYDLPEQYTRSLIFCIARAQKPLYLTAGKFYVFSLETFGVIVKASMAYLSVLKS--

>LhOr203NTE

----------------WAIGLNRRMLKLVGLWPQNST-----ISEVIFSKFCLLFNIITIFVLTIPALASLIRVWGDMILMIDNLQYTLPLLITILKVSIMWCKREALSPLIDMVIKDWTKVKMEEERNVMLK-QARITRILAMCGGFMIFLTLLITFSSLFFGL----TLRHV-TNLTDP--GKPLPLQTYYLHDVSKSPQFELTFFTQGIAVTTSGLSYTAVDNFLGLLLLHICGQIENLHLRLLNLGKNSN-----------------------------------------------------------------------------------------------------NSNFIATLKFNVKDHIRLISSIQIIDNTFNLMLLGLIFFFGVLFCLQGFLIINVNLTLLHFIWYISANVCVLLHMCLYCAIGEFLVTQSEKIHSATYEYLWYTLEPKAAKHLILVMLRAKKPLYITAGKTFPMTMATFCNLLKTSAGYVSVLLA--

>GPRor21

------------EDPKAVMPFAKRLLRLSGFRQET---------EQLEKQIFFNL----FVYVAALLIPKVCSPYPDSEAIIRGLSELIFFTNVYVGYYCFVVQHRHYRDLLDEIQPTSQQPESPSER--TLIKLNVKINKISVLYCWYLAAAGLIYWSTPCLMT-YHSVLKAK-AGPNHPIRFYPNLEGSFYGLDNRTSVGYAAFSIVALLVFAFASYNNATKLLTILSTIKYCSTLLQLVGVEVDNLNHTSS------------------------------------------------------------------------------------------------LNHTSSEAIGRELKKVIQLHQLALRCVALLNQTLSFVMALQLALCILTWCFTLLYILI-VGFNAIATNGLLIMINMTLEMFGYCFFCTELDTTGKIVSRQMYEFRWEQHRPTVQKMVAMIIARSQTPLQITACGFIPINLELFTKVVKHSYTVLAVLK---

>376_Si_gnF.scaffold04648_643308-783672.pep

------------------LQFTLKLCTVSGCWQPLRT-----MSARIIYDSYRVLLICLISAFTMSQFINIALNIDNFNEISDNIYMMLTVFIATYKLISMWISKKHVTTIINIFTEKPFKPLESSEV-MIRQKYEKTIRQYAFWYYGLVQITVICIIINAFAMDFM----------------TGNLTYKAWVPFDYRPSVIFFFVFIHQLIGMIIAAAVNVACDSLVSGLLQEICCQLEILEYRLTKIFHDQNVL----------------------------------------------------------------------------------------------------------LHDCVRHHNRIYECAYMVNGKFAKIIAIQFAVSMLVVCANLYKLASISLAMIGLLTLILYTCCMLSQIFLYCWFGNELKLKSIGLANSIYNMKWADLENKNKKDLLLIMRRSMVPIEFSSAVIITLNLDSFGSLLKASYSAYNVLK---

>SlitOR23

---------------FYQIMVNFKVADLSNENPPS----------YFNQNAII-FMTGFVAKLLCISSFYHGLMTFNLRLATEAGTYIIVMAYALLISSCTRRNVPQYHNFLRAMKEDFQFICTSGEKYRVPYFSTWKICIFACIFTASIAVGMVSFAFLSLFYF-LA-TYKEE-IGG-----SRPLLFPFWLPVDFGETPVYEIAFMFSNICALLYAYNYIFMIQTQIVWIRQITSKVDIVIWSISDLL---------------------------------------------------------------------------------------------------EESVYFSYLIKARMRDILLHHQSMYSLMEDYAVVYKKMLMFEQKCCGSVVCLTAYCIAEAFDAGEFQAILLLLCIGTTVLHFVPCYFCTFLAVKVSSVCDACWNIPFWKAGPLIRPYMVLIMQRSLRHLPLQAAGFEDISIETFSKKMTNAYSLFNMLRQ--

>PbOr142FIX

------------------INLLKIILILFGVWPDA-------TCVTFCRMFWGSTAIIFIILQY--QYLMIHYRIDSIFELIDCISSFVWCIKLVCKFFLLWFNQRILKELLTIMAKDWKDCAKSDIEMRQAVNKAKISDRVANTVVIYQTVAVTLYGIGIVFGDV------DV-TQS-----NLPHIVKMEFPFQITTQRMYRSIVTIELIYIIMTGWCSALVNVLILTLSLHVGGQIDIVCCWLAKLMPKPNM--------------------------------------------------------------------------------------------------KENEFVAATISKIIQKHQKIIYFSQQIENMYSFIALILFLGNTIMICFLAFLFVTAPDTTDKIFRSLPFYGVTNIEAFIFCYAGEYIINKSKALELAAYNSAWYDLEPKFGRMLLFVILRSQKRMTLTVGKMIDLSLQCYARIMNSSGSYLTMLLAM-

>gi|383861646|ref|XP_003706296.1|

------MKKQSNNSIDYYILPNKILCTTLGISLSDKK-----RSGQIFAYLRLFVAVASISSFVVPQAMLLFIKWNDLKILSEVGGILTTLAQFEFKLIYLAIRREKTYKLYKEVRSLWNSTDDPEEKRSYEEF-AYWARRFTIIFYSFGTWTTVIYTASAAVDCI---IIQYS-NNDTI---TRSLPFDVWYGTNVSESPSFEIMFTVQTVSAIYNSAAVWGIETSCMTVILHVSGQFKLIKTWINNIGVKIKNEP----------------------------------------------------------------------------------------------------DIEDGLVRCIRHHQRLVNVVNELNDLLIPIIFIQLLTSGIKICLSGFAVM-NNNTNAELIKAVLYLFGMTTQLLLYCYPGEILIRESEEVGDAAYNVCWYKLPPSNRRQLLLTILRAQKCCSITAVTFQRLSFRTLTGVFNTAASYFTLLRQM-

>DvirOr85c_scaffold_13047_residues_7492516_to_7495526_reverse_strand.[501_2511].sp

----------------SFLKYANFFYKAVGIEPYTKR-----SHPHVGSIWVFWANIINLGLAGLGELAYVVITGEHIVEAIMVMSYIGFVLVGMSKMTFVWLEKPALSHIVWELEELFPGNKAAQSA-YRLDNYLRSCSRISFTFSMLYSVLIWTYNLFNLMQYF---IYELW--LQTRIVE-ETLPYRMYIPWQSTDNWTYYLLLFCENFAGYTSAAGQVSTDLLLCALATQIIMHFDYLSRRIEQ-----------------------------------------------------------------------------------------------------QQLSGNWSEDSRFLADIVRYHERLLKLSEQLNKVFGVPLILNLLISSFVLCFVSFQMS-VGVEPIMMIKHSLYLISALSQVYLICYHGQLIADASTGISLAAYKQNWSYADARYRRALAFIIMRSQNPTYLKATVFLQINRGTMTDLLQLSYKFFALLRTM-

>DperOr1a__super_17_residues_1115707_to_1117985_forward_strand.[501_1779].sp

--------------------TQRFTFARMGIQPMSAT-----GDGALRRPVLYGIMVLATSFELCTVCAFMVHHRHQIVLCSEALMHGLQMISSLLKMTIFLVKSGDLISLIHQLQEPVPSR--------V----VRRGQQLAAIYFLMCAGTSVSFLLMPLALTMVR----F---HQTDRFE-PVSSFRVLLPYDVTQPHIYALDCCLMGFVLTFFCCSTTGVDTLYGWFAVALSSHYRRLTTQLQ----------------------------------------------------------------------------------------------------------------------LFAQHARLLALVDRFNAAFREIAFVEVLVICVLYCSVICQYIM-PHTDQNIAFLGFFSMVVTTQLCIYLFGAEQVRLEAEGFSRQLYQVPWQGLSPNHRRLLLLPLQRAQGDMVLGAY-FFDLGRPLLVWIFRTAGSFTTLLNA--

>gi|170038169|ref|XP_001846925.1|

-------------------------------------------------------------SFLMYQLLRICFFLPDIDAFMAVFNMGFIWTVALSRGICFAIYRRELNQLKKYVNARSCQRDNPVAK-EFRKNTYRQNLKLATIFQLYTVINVSTWCFTEFRTN------------D-----NFDIPLPMHL----------------------------------------------------------------------------------------------------------------------------------------------------------------------------------------KQLKAASSTNFLIVFSSAMVLISMNMFLFII--DPSVKRIPLLVVSCQFAFEVFICCYLFDSLEQENNHIKHHVYAIDWMTRFRSIRQNALFLQLQTNAGLKIRAGGMFDLNLVSFSTLINLVYSMLTFLLRTR

>TcOr234

-------------------------------------------------RTMKIFTIVYFILYSGSLLLDLYYNNFSIAAMVRYGCMIMLISYVIAGMLFCFIFEKQLLNLLSEAETIFWPPEMITSE---LPK--FIHRTNVLNYFIIAWFGLLGVILFPVWGD------------------QSEWFLNVWAYKAYFGSWWYIPYNL-FYYSQPMAAWTCVRLPFIMMYFSLQIKLQIFLLNQQILEIPKGHNTNSETAPDDLSY--------------------------------------------------------------------------------------LSYQEAVSQKMCLCISHNVKIKRWTKSFLRKVIQAMPVFVLLGILGSIFVTFSVLYSSTSTILKIRLVVVVGCTILSVYMFVEGSQRLCDESSQMFEMLAYSPWYLYNKNNRRILLTFMTNTLEPITITWGGI-ILNYNFGLTMLRMSFSYALFLYNI-

>LhOr107FIX

-----------------------LMLTMFGVWPGI----------SCVLVIRVFWIITLAISQF-FHYRYFVTRADNLFNLMDCLSSFLAHVKLTAKLIVFSLQQQKFIEILSMMSEDWVKCGNRGVALSETIRKTKASNRICNGLIILHTIGAIAYVAGILLAD-----V-DV-TDRTT---ELPLMMKMEYPFVIDTRSKYDLVLATQFVYVMVCSWGAGLFNALFLTLTLHLGSQVNILLCWLAEMEPNK----------------------------------------------------------------------------------------------EPNKIKEENKSFVVSITNIIQKHQRVINLSENIESLYSYIVLLQFTSNTLQICSLGFLIVTAPNATEMIGRSLLFYALTNLETFIFCFAGEYLSNKSKAVGNAAYNSAWYDMKTKESNILLFIILRSQKQLKFTAGKMMDLSFESFTSIMRASASYLSVLLAMK

>DkikOr59b1

--------------------YLYRAMWLIGWIPPK---------EGLLRYVYLFWTCVPFAFGVFYLIISYVNEFKNFGEFLTSLQVCINVYGASVKSTITYIFLWRLRKTEMLLDNLDRRLKGDGDRKKIH-EMVARCNYAFLIYSFIYCGYAGSTFLSYALS--------------------GRPPWSVYNPFDWRDGLSLWIQAIFEYITMSFAVLQDQLSDTYPLMFTIIFRAHMDVLKDHVRNLRMD---------------------------------------------------------------------------------------------------PERSEADNYQDLVNCVIDHKMILRCCDMIRPMISRTIFVQFLLIGSVLGLTLINVFFFSNFWKG-VASFLFVITILLQTFPFCYTCNLLLDDAQDLANTIFQSNWVDAEPRYKATLVHFMHHVQQPIIFIAGGIFPISMNSNISVAKFAFSIITIVRQM-

>TcOr230

------------------------------------------------------------------------------------------------------------------------------------------------------------------------------------------------------------------------------------------------------------------------------------------------------------------------------------------------------LNQKIAHISDLGDQRLVFGTLCSCVSLQIKLRQMLNKVLQFVYLVMPVFLLLGALTAISVLFFLFYSLENPSLMIRLACFLGGNILVVFTFCESGQALSNDTGRIFDILLTCPWYKWDKKNKNILLMFLVNSLKPMSITIAGI-TLDYKLAVTLIRTCCSYALVLYQMK

>MsexOR-24

-------------------------------------------------------------------------------------------------------------------------------------------SLLTKYFFVLNAVLISVYNFSSPIIMLYQYIAKNK-------V-VFVLPYAVLLPFPTDGWLSWFLAFVYSATCGCICVLFFTTIDVLYCVLTSHVCNNFSIISDQLQHLQ----------------------------------------------------------------------------------------------------------------IGNIVKEHQYILKLADDLEDIFTAPNLFNVLVGSVEICALGFNLTT--GNLAQLPGTILFLTSVLLQILVMSVFGENIITESRKIGESAFLCKWYEMDEKSKKMILTIMIRSRKPQILTAYKFSIISYGSFSKIISTSWSYFTILQTV-

>CpomOR8

--------------------------------------------------------------------------------------------------------------------------------------------------------------------------------------------------------------------------------------------------------------------------------------------------------------------------------------------------------------------------------------------------------------------------------------------------------------------------------------------------------SLTSFTSIMNSSYSFFTLLRHM-

>395_Si_gnF.scaffold01629_62255-192132.pep

----------------QHMRINQMLMCLIGQWPYQ-E-----NWEKFLIQLVFVPAVFAQAVLQGGGMITAYF-AGDIDAFMESSSPFVISLMCVCKHINYTYNHDQMKRLAFTMVDDWKIYSKLSHEYDILCRNYAMGRKVTIAYAVSLYGSMTPFLVVPVVLNTASYMGLYN-ISD-----GRPLMFRTEYFIDSEK--YYYPLLVHSYIGTLGFVSIVVAIDSMLVFHVQHECSMCEILGYRLARIVAED-----------------------------------------------------------------------------------------------NLYPNKEEAISYDHIKNCVIIHNHIIEYAKRIENANTTSYFFQLGFNMMGMTFTIFQAVVKLSDPNEALRYASFTVCLISVLFLESWPGQQLSDYTDKIFAFITNGRWYQSSLRVRKVISIMLMRSYKPIKITAGKLYTLNLANFSAVVRTSFSYFTVLCSM-

>gi|167867977|gb|EDS31360.1|

---------RKQHHFDAMMAFNIRVLTALGFWGES-------GIE--LRATLQYLAY--TTWFIPPGVLFCVRQQPSSKMMLKSVYELVAAGSCIVRIGNLYVFNGTLQKAFYEVQFALGELSRDSSDRKVLNHLTISADYICKGYGGMLIIQCLLFGPAQGLVSILKYFVWGE-DP------KYSLLEADYLIYNQYSNNVWLLTMLASTCALYVLVFALLSHETFYWNILHHVSCLFKIIRLKILELDDCSTP-----------------------------------------------------------------------------------------------------KQFHEQLSIVVEMHERAFKSTRLLEQAISAMMSFLYLSFITIMCSMLLVFTVIQPDLGFLLMMSVALQYNVFLIFTFSMLGTELTEASLSVSDAVYSIRWYEKSPAERRLLLFVQMRAQKQAAITAAKFFYLTRASFATC---------------

>DgriOr45b__scaffold_15245_residues_15399810_to_15404865_reverse_strand.[1501_3556].sp

------------------FFVTRYSFGLLGLRFEL--------APTFWSMTWLVFNFVNLAHCCQAESFGWHYIRSSPVDAMDAFCPLACSLTTLFKMACMWRSRSEVASLMQRIRQLTEQQRH-GERVQLKRSYYRIATLVCMLIFTLGCINTGAFVLRSLWEMW---------MRRDLPF-KYDMPFRMFFPSIAHRFPMYPLAYIYSTWSGQVTVYAFVATDGFFFSFTLYITFLLKALQMDIQRVLR-----------------------------------------------------------------------------------------------------PRECEKCCQQLRNIIDRHNDIVGIVQRFSVIMATPTFVQFLSSSLVIATSVIDILLFSGYN--IIRYVVYAGTVSASLLQYCYGGTEMSIASLELGEAAYNSHWYQWNREVRQRVYLLILRAQRPVTVQV-PFFVPSLPTFTAVIKFTGSIVA------

>DpseOr10a__ChXL_group1e_residues_6051175_to_6053604_forward_strand.[501_1930].sp

----------------YFFAVPRLSLDVMGYWPMGD--------DLPVRAIVHFV-ILSIGVVTELHAGFMFLQNAQITLALETLCPAGTSAVTLLKMLLMLRYRRDLTNVWRQLQRMLFDGLNRPEQKAIIHDNSVLAARINFWPLSAGFFTCTTYNLKPLLIAFILYLQDPD-QEL-----PWNTPFNMTMPKVLLAAPFFPLTYAFIAYTGYVTIFMFGGCDGFYFEFCVHISSLFQSLQEETRAIFR------------------------------------------------------------------------------------------------------------ELQLRGLIIRQNSVFELISFFRKRYTVITLAHFVSAALVIGFSICNLLTVGNNGLGALLYVAYTVAALSQLLVYCYGGTLVAESSFELSRVAASCPWSLGAPRQRRVILLLILRSQRAPTM-AVPFFSPSLNTFASILQTSGSIIAL-----

>NvOr235PSE

---------RASKSFNASSRIQLSLFRLMGVLSFEK-------SRPLTSRLLSGWFYLCYCFFGSMFLNTCVHNCLRGYYNLEQISETVILLGSVGRFLLLSGSRREMERLLTSAEDLWRVLEQGERT--LVARFVDISRKVVYAYLVASCLMCSFYVGITPILQ----------GNG-----ZCVLPFE--FYVEVQSTPWYELVLILEGIAMFSLALVSSLVDITGPFLILMGCGHLRTLGHRLRNIDSRL---------------------------------------------------------------------------------------------------------YLAELTSCIRYHQMILSYCDQVQRVLGGVFVTQLISTTYNISLLGLKVI---GSDPDKSKYVMLIGVLMLQLLFLQWAPDMLVEESKRLASDSFLVPPIGENRQIGQLICIFAMRSQRPIEMKAAGYLELSMESFGAMLTNILSFFTVLHSI-

>DereOr33b__scaffold_4929_residues_13474925_to_13481762_reverse_strand.[1501_5338].sp

------------------------------------L-----ESHFFLNRLLDMVITAFVTIWYPIHLILGLFMERSLGDVCKGIPITAACFFASFKFICFRIKLSEIKEVEILFKELDQRALSQEECEFFNQNTRREANFIWKSFIAAYGLSNISAIASVLFGG------------------GHKLLYPAWFPYDVQASLIYWLSVTYQIAGVSLAILQNLANDSYPPMTFCVVAGHVRLLAMRLSIIGQGPEETK------------------------------------------------------------------------------------------------ETKYSIAKQLIEGIEDHRKLMKIVELLRNTMNISQLGQFISSGVNISISLINILFFAENHFAVIYYGVYFLSMVLELFPCCYYGTLISVEMNQLTYAIYSSNWMKMDRVYSRTLLIFMQLTLAEVQIKAGGMIGIGMNAFFATVRLAYSFFTLAMSLR

>gi|238623753|dbj|BAH66350.1|

------------------LGPNVKALKFWGLLLPE----------SRSKKYFYLFMHFAVTVFTATEYIDVWFVKSDLALLLNNLKITMLATVSVLKVTTFLLWQNAWRDLIGYVSADLEQRATSDSR-KLALINTGYCRKITYYYWFLMYTTVAIVTVQPIFKFFSSAAYRLD-VNG-----TYLQVVSSWIPWDKNTLPGYLLASIYQTYAAIYGGGWITSFDTNAIVIMVFFRAELELLRIDCAALFDDEKS-----------------------------------------------------------------------------------------------------MAFMRRLKECHRRHTELVKHSRLFDSCLSPIMLLYMFVCSVMLCVTAYQIT-IETNPMERFLMTEYLVFGVAQLFMYCWHSNDVLYASQDLSRGPYESAWWSRDVKYRKNLYILVAQFNKVIVFSAGPFTKLTVATFIRILKGAYSYYTLLSQ--

>DmelOr85d__3R_residues_4378320_to_4379697_forward_strand.[11_1368].sp

-------------PLHSFLKYANVFYLSIGMMAYDHK-----YSQKWKEVLLHWTFIAQMVNLNTVLLIYVFLAIGNFLEATMNLSFIGFVIVGDFKIWNISRQRKRLTQVVSRLEELHPQGLAQQEPY-NIGHHLSGYSRYSKFYFGMHMVLIWTYNLYWAVYYLVCDFWLGM-RQ----F-ERMLPYYCWVPWDWSTGYSYYFMYISQNIGGQACLSGQLAADMLMCALVTLVVMHFIRLSAHIES------------------------------------------------------------------------------------------------------------------LQATVAYHQSLIHLCQDINEIFGVSLLSNFVSSSFIICFVGFQMTI-GSKIDNLVMLVLFLFCAMVQVFMIATHAQRLVDASEQIGQAVYNHDWFRADLRYRKMLILIIKRAQQPSRLKATMFLNISLVTVSDLLQLSYKFFALLRTM-

>DbiaOr69aB

------------------------------------E-----NNRNLAKRMIFWFGAVNLVYHNFGCIMYAYYADRSIAELASVGAMLGFTIVGTLNLWKLWTLKPDIEKLMDDFEEMFQLTKRRPYRS---HHYYESYTRFIRNLLIFFTLTIAYYNALPIILM-TRELLKE-----SQKL-SYRLQSSTWYPWQFQGSPGFLVAVVCQGFSCQVNLCVLIFSQFLVSFFGIQLEIHFDGLARQLEAIDARH----------------------------------------------------------------------------------------------------------KDQLKSLIRYQKQIFIMADRVNEIFNFTFLISLSISVTCTSSLAFSVTMF--DLGPALKHTFGLLIFLIYNFCMCRNGTHLILQSDKVLPAAFYNNWYEGDLAYRKMLLILMMRATKPYIWRTYKLAPVSITTYMATLKFSYQMFTCVRSLK

>SlitOR13

-----------------YLRILKKIMWVIDGWPKE----------AHKSQFFRYYICILDMVSLIPGTLYLKINTGKIFELGHTYITVFMNAIAALRTVLVLTYNEIIFYFLKEVHLFNFRRKSKYAYETHILV-HKISHFFTMYVFMLMCCGILLFNLTPIYNSYAAGMFRDE-PPPNAT---FDHAVYFALPFDTSTSKGYAIVSLYNWYICITCSTYFCIIDLTIFIMVFHLWGHMRVLSYNLENFPKPASVLAAANDTHAYTPCEN-------------------------------------------------------------------------------------------------------------------------------------------------------------------------------------------------------------------------------------------------

>NvOr112PSE

----------------KTFPRTFALLIIGGLWAPTTZ-----RALFICYQIYAVFYFISALMMIITIVIDNILDDKSMEYLMENWZKLIVFFNGLQRITNLAVRRDKILHLLRIMSGRWQIIEK-----------SRISEIILKIWGSLIFVNGISSWLNPIVHE-----------NP-----ENKLMYECYSPRDRRTPTCFWIAYAYQLFGYLVLSAANVGTDCLIYNFIDRINAHMMIFLNRLLKLPTR------------------------------------------------------------------------------------------------------------KYIRECIEDHHDIYESIEELNRIFNELISIQFLSCISLLCMNIYFLSKQE-----------------------------------------------------------------------------------------------------

>gi|284448841|gb|ADB89178.1|

----------------KYFKSIRTVMVAPGVWPAEIG-----EKISVVAIVHRVTLPYHTSLIVFGELYYIWMHMHSFLDLGHMIITTLLGILTAVRSILLQNYHKLLLKFINVMHLMHSTNKGPYYNQ--MNDTDKVCSYYTKFSLGLIFISCSMFNVAPFCNNIANVFIFKT-ENYTL---EFSLYYQ--FPIDPTD--YFTTTSIYNFYLSYNCAMMVSGLDLILFLIIFQIIGHVYILRYNLENF--------------------------------------------------------------------------------KLGDILKYKINESIT---SEMFDAEENKEVRFKLAECIEYHKKIIGFTDEVSALFGPILACNYLFHLICCSLLLLECS--EGGYSAMLRYGPMTVLIYGQLIQMSVIFELLGSETEKLPDSAYFLPWECMDTSNRRTACIMLHKMQYKISLKALGLAAVGVSTMTGILKTTFSYYAFLQTM-

>gi|226510879|gb|ACO59966.1|

-----------------------------------------------------------------------------------------------------------------------------------------------------------------------------------------------------------------------------------------------------------------------------------------------------------------------------------------------------------------------------------------------------------------------------------------------------------------------------KNRKTVAFFLMNVQEPVHVRALGLADVGVTSMTAILKTSMSYFTFLRS--

>DeleOr1a

------------------LWTQRITFACMGLNLQPK------KGKVLQTPVLYGIMFLATGFELCTVCAFMVQHRNQIVLCSEALMHGLQMISSLLKMAIFLAKSHDLVALVQLIQAPFMNRGDLGVSE--WRSQNRWGQLMAAVYFMMCAGTSVSFLLMPVALTMIKYYS-------TGDF-APVSSFRVMLPYDVTQPNIYAMDCCLMIFVLSFFCCSTTGVDTLYGWCALGLSSQYRRLGLQLK------------------------------------------------------------------------------------------------------------------------AEHARLLKLVKHFNVSFMEIAFVEILVICVLYCAVICQFIM-PHTDQNFAFLGFFSMVVTTQLCIYLFGAEQVRLEAEGFSRQLYEIPWQKLSPQHRRLLIFPLQRAQRETVLGAY-FFELGRPLLVWIFRTAGSFTTLLNA--

>NvOr148PSE

----------------------------------------------------------SIIVLAVFITGFLSHVFGTALQCRRILVLHRHCSGIIAKFIVLMVSKEKLNELLSKISLDWNGITDPYER-SILEECSKSGRPKMWLYFVYCVLVGFALCQMFALASLMRIILPLN-ESR-----PKILAIKAECPFNY----YYELYSI----YCDVAVTSVSVTDQTYVMILQQSLGLFQIVKHKMQKTTM--------------------------------------------------------------------------------------------------MQKTTMYNNQDEVMVLAITLHKSVLQFLELTESTYQSAFLLFMVATVAFLSFGLVIMIKHSKEIIDLIRMTMIVXSHIF-----------------------------------------------------------------------------------

>DgriOr67d-3_scaffold_2580_residues_1_to_1700_forward_strand.[245_1498].sp

---------------RRVIYLLRLITKICGADIIE-------PNYKLNKLTYIMFVLTFLFYLGTFYIYTSVVTEGNFNVILKITTFLGAAILVNTKLFCFIRWAHLFRESQTFLDTTYMGFEKGGDYKKVMLDGIRAIRKTITILMLVYIACLVTFTGFPAVYYA----FYN----------KRILILQFLLPFDKNTTIGYWLLCGVHSVCLNFGGLGNFVGDAFFLTFIGNVPLLKDILACKFKHLNR----------------------------------------------------------------------------------------------------EKATSAQLRKAMVNIILWHKKYLSLQFRVRRIYFGVIAAQILSSFFSVLSSMYCLLT--GDWPA---APVYLIFSLLTLYLYCGLGTIVEKSNDDCVTIIYTCRWYDLPISEQHFLLLMLRMSQTTSSLSVAGMMPLNVNTALQLTKAAYSMGMMLMT--

>NvOr258

----------ELRKYARYSRALKCLLVLSGVWPDF---------HPVIQPMLGCFAVFVCSLTAMATLNFSIHHITNFVVMTKSLTIAIGLCLSTIKIVVCLRHHDGLVYLNSSLTASFDADNQNKSF---RRFTLAKVNIFANLFYTLTIAVGLATGMGVVFLILA--LLHG----------KYVMVWPSIFPFSYEPGRVYWILLLVELSANIFAWAVPSGVDSVFGWYTMQICGEFRVLAHKFQNL------------------------------------------------------------------------------------------------------NLKTSENYQDDLKECLERHYALMKSGEVLQDVFGFLAIMVAVTSAVIQCMLIFQAIQVFQQLSMMILIFAFITLKHVQAFIYAWYGQLIADESEDCIEAMYCAQWAGGDIRFMSDVLMVL--SQKPMVFRAKGCMSLKMDMFIKVLNTSVSYYFLLQT--

>gi|167865757|gb|EDS29140.1|

--------------FERITSANRWVLKLLGIDVFN---------PNFRYSTATWVIWSLASFFILVTGYDLYRFRNDVFNFAFALVTLGYAVIGVSRLGFFLGSPAAYSQIFAESKETYRQESSERSRE-VQQKYTIMLKQCVMLYSGCFLGGCIIAGMLPFAVYWWT--------------EQKVLPFGVILPFDPDTMEGYQLNYLYQVSCIAWTPPGLTATQNMYFALVFNICIQYDVLQLKLEDLDKLIEDTAE---------------------------------------------------------------------------------------------DTAEYSVIQQKLVEIIHCQQHLSAFVTEIERIFAVQMFIEISSMAMQIVVILFVI--SQ---DLWIPGYLAIIVATFQLLIFCALGTFISIKADLFAESVYNVSWHQIRIPEQQSIKFMMAKSQQSLLLTFGGMLPLDMNLFLSV---------------

>gi|350422511|ref|XP_003493186.1|

-----------------------------------------------------------------------FLEDESIYLKLKSLGPVSHWVVSGANYTTLLLRSKDIRQCMEHMEADWQTVTREKD-QWVMLKNAKFGRYVAASCAIFMQSGVMCFIFVTAMDT-VE---IQV-GNETRI--LHVLPCAVYNKINVDESPTNEIVLFLQAWCTIIANSSTVGMFSLAAVLAAHACGQLNVVMVWITEFVNEPKKTD--------------------------------------------------------------------------------------------------------GIGVIVERHLRTLNFIGYIEKLMNRVYFLEIFRCTMNICILGYYILSEDQNVQSLVTYIMIYISIGFNVFLICYIGEILTEQSKKVGEVVYMTNWYYLPDKTILDLILIIARSGVVVQITAGKMVHMSVYTFGDVVKTGFAYLNLLRQM-

>NvOr253

-------------KYNSYSNTIIWSLICSGLWPKG---------HYVLKKILSCISFLSITTIMTTAINFSFQNARNVQLMTKGMGTAVSFSSVFSKIVMVLYHQNDFIYLKKHLTTRFKRDLEQTENRQDLLFNVHIFTKFVNTHEASMAFAMFMYCIGPILAL-YR----H----G-----KYVRTFPCLYPFHYEPGVVHWVIYGLEVTGATVIWFITIGVDCGFCMYALELCGEFKVLGRKFRELR----------------------------------------------------------------------------------------------------------DDYKEKLRDCIERHHLIINAKNRLEDAFGIMAIWLALSGAFLLCSLIFQITEIHGSYLKIAHLCSHLLAKYLQIFMYAWYGNLIADESQSFLYSMYSSHWIDCDKRFKSDILIVLV--QEPLMLVAKGCMNIQLDMFLKIVKTAMSYFFLLQT--

>NvOr252

------------QTYKVCLQNVVICLIFSGVWPAT---------RPLLKRIAFFVTFFSTFSIMAHTLNFSLHNAQNVRILVRGLAAASSFLSISSKVFLFFQHQDDLVYLNDYLSKKFMSDMQNPENLPDLLSNVRTFAVFVRMYKTTAAFIASMYSVVPIIAF-----LKYG---------KYLRVYPCLYPFSYAPGVVHWLLYGWESAGALSAWAITVGTDCIFGMYAIQICGEQRILARKLKDLRVGSN------------------------------------------------------------------------------------------------------SNYKKQLRDCMERHHFIITVKNKFEDLYGLISIWLAISGAIVLCSLIFQVTEYDGGHVRAIIFFAHFSSKMMQVFMYAWYGNLINEESLAFPRAIYSSHWTDCDTRFKNDILIVL--AQRPLIVTALGCMNVQLDMFAKIVQSSISYFFLLQTLK

>NvOr251

-----------------------LCLIFSGVWSAT---------HPVLKKIAFFVTFFSTFSIMAHTLNFSLHNAQNVRILVRGLAAASSFLSISSKAFLFLQHQNDLNYLKDYLTEKFMSDMKNPENLPDLLSNMRMFAVFVTMYKTTIAFIMSMYCIVPLFSF-----LKYG---------KYLRVYPCLYPFSFVPGVVHWLLYGWESTGALSAWAISVGTDCAFGMYAIQICGEQRVLARKLKDLRVGS--------------------------------------------------------------------------------------------------------NYTRELRDCMERHHLIITAKNTFESLYGLISIWLAISGAIVLCSLIFQVTEYRGGYVRAIIFFAHFSGKMMQVFMYAWYGNLINEESLAFPRAIYSSHWTDCDTRFKNDILIVL--AQRPLIVTALGCMNVQLDMFAKIVKTSISYFFLLQTLK

>DtakOr85b1

------------------MKYASFFYTGVGIQPYSKS-----QKDMLRASIVFWANVINLTLVGICEYVYGAYKENKLLEAVTVMSYIGFDIVGLSKMFFIRWKKAAITEMMEELEEVYPRGKVQEDR-YNLSKYIGSCSRISLIYSSLYSVLIWTFNLFCIMEY---WVYEKW-LNIRVV--GKNLPYLMYIPWKWQDRWSYYPLLFSQNFAGYTAAAGQISTDLLLCAVATQLIMHFDYLSTTME----------------------------------------------------------------------------------------------------EDHKLSGNWQEDSRFLADTVRYHERILRLSDVVNDIFGIPLLLNFMVSSFVICFVGFQMT-VGVPPDVVIKLFLFLFSSMSQVYLICHYGQMVADASYGLSLATYNQDWNHADVRYKRALVIIMARAQKVTFLKATIFLDITRSTMTDLLQISYKFFALLRTM-

>NvOr257

----------ELRKYERYSRDLKWLLVLSGVWPDF---------HPVIQPMLGCFAVLVCSLTAIAVLNFSIHHITNFVVMTKSCSIAIGLCLSTLKLCACLWHHDDLVYLNTSLAASFNADNQNKSFRRFTLAKVNVFANLFYILTIAVGLVIVMGLVFMILS-----LLHG----------KYVLVWPSIFPFSYEPGWVYWILLTVQLLANFFAWTVPSGVDSVFGWYTLQICGEFRVLAHKFQNL------------------------------------------------------------------------------------------------------NLKISENYQDDLKECLERHYALMKSGEVLQDVFGFLAILVGLSSAIIQCMLIFQAIQVFQQLSMMILIFAFITLKHVQVFIYAWYGQLIADESEDCIEAMYNAQWAGGNIRFMRDVLVVL--SQKPMIFRAKGCMLLKMDMFIKVLNTSVSYFFLLQT--

>NvOr256

----------ELRKYERYSRDLKCLLVLSGIWPDF---------HPIIQPLLGCFAAFVCFVTVIAFLNFSIHHITNVVVLTKSFGLVISFFSSFLKICVFLWHHDDLVYLKAALTDRFNTDNLNKSFRRFTLAKVNVFANLFYILTIAVGLTTGMAVVLLIISL----------RHG-----KYVMLYPSIFPFSYEPGRVYWILLMVELFANLFVWAVTSGVDSVFGWYTLQICGEFRVLAHKFQNLKSSE---------------------------------------------------------------------------------------------------LKSSENYRDDLKECVERHYVLMKTRDVLQDVFGFLTILLALTSAIVQCMLVFQAIQVFKNLSMMVFLIAYITLKVVQAFIYAWYGQLIAEESEVCLGAIYNARWAGGDTRFMSDVVIVL--SQKPLIFRANGCMSLKMDIFIKILNTSVSYFFLLQT--

>NvOr255

-----------LKKYKRYYRDIKLLLVVSGIWPNF---------YPILDRVVSIVAAISTLLLTMALLNFCAHHVANIMILTKSMGIAISFFSSFLKICIFLSHHDDLVYLNDYLTSSHTSDLSNPDDRSHLLEKFSSFSKFFYTLTIAVALTFVLNTIAPFFAL----------KRG-----KYLHIYPVIFPFDYEPGSVYWSLISLELTAGFFVWSVTSGVDSVFGLYALQMCGELRVLAKRFEELRAT----------------------------------------------------------------------------------------------------------YRMRMRECMDRHHLLMRSRDILEKVFGFLAIWLAVTSALVQCSLVFQAK-VTLSPFKIGFFFFYILMKLVQAFTYAWYGNLIAEESALCLNAMYNAHWPGGDVRFMNDVLIVL--SQKPLIFKAKSCMSLHMDVFTKIMNTAVSYFFLLQT--

>gi|380016198|ref|XP_003692075.1|

--------------------------------------------------------------------------------------------------------------MLNVCETFWSNLKPHEKK--IVQSYTRKTTRLTRWYLASCVLTIAFYAFLVLFV------------------PKRHLPYA--FFLDVQKTPWYEIVYALQLIGMFNVGFTCVGVDTVGALFILITCGYFDTIRSRIENLHSFDTSLPSSSLSLLNILSRKIT---------------------------------------------------------------------ITTAKMSDTKTKTSNSAQMKNLRMCVIHHQLLLKFCEDIEHLTSGMFFIQVIASTYNISLVGFKLL---EDTPDKFKYITQLIILIIQLFLCNWPADLLLSKSVDISRATYSMPWYRYSYNLQKITNILMVRSQKAVRLTAGKFIGLSLETFASMISTAASFFTMVRSM-

>PbOr52

------------RDVTVTLSVHRFFLSCVGIWPVRE--------KNIFMDLRWIIAVVLEVIPVSIYLTEIYLHCNGAKKSFDWITPSAAGMLALTRLITPRIHREELVEIVTSMMDDWVMQKDKKIR-WVMKKYATMSTRVTVLTFILVGIIVAVYIAMAISAITAKIEQF---DNNNVST-SHKDGIQSCVFHSAS---SHQAFMIVQAMQMFVTGVLTFGTTSFFFGLAMYLCSQFDALSIKLSEF-------------------------------------------------------------------------------------------------------------AHRAIAEAVQRHCHLIRLAECMEESFNASVLVYLFVTSTLMCIDGYMLIASIGDLPMIIHSASVLLLMLIQLSFYTFAGDYLEMRSSALSYATYDCNWYELPASTAKNFQIILMRASIPHQLTAGKFVIMNMITFKDILKSTASYLSVLRIM-

>HsOr11

---------HYRRDNDYSLQLNRWVLKPIGAWPELPT-----NSRNMLKKLLRFTCHSLIAFTMVPSILYILFEEKDFRLKLKAVGPTSHFLMGSINYCSLLYQNERIRRSIEHMETDWRIAKREHDREVML-RNARIGRIIAGICALIMQGGVLCYNLARGMSP----IIMTI-GNETVA--IGRLPCPSYNKVDTRFSPVYEVVLVLQCLSTIIVNNTTIGACGLAAVFAMHACGQLNVVMFRLEELVDEKR----------------------------------------------------------------------------------------------------KRDILQLRLANIIERHLRALRFLSRMETIMRQICFVELVGCTFNLCMLGYYTITEEESTNTIITYIMILTAMMFNIFIFCFIGELITEQCSKVGEAAYMTNWYHLPHKTALGIILIILRSSIAVKITAGKIFHMSIATFGVVIKTSVAYLNMLRTL-

>LhOr65NP

--------------INYAFALSRLCLRILGVWPNPNH-----NQSNIRRPNLRFVIVAZSFYVLAPQLRNMIRAWGNIMGIMECLVSVNFTIMALCNLIATWYHGIALRX--ASVINDWVTWTSDWERNTMLKI-AKRERSLSFRCYVFYVSSAVFYVZFNFLKF------RRS-MHQT----QRILAYQSVYPYNTQKNPNYEITFLLQVSGGVYSSLINCTVDSFITILLLHVCAQLKNLRITLNNKVDELTNK---------------------------------------------------------------------------------------NKVDELTNKTISTLRFKKVLTAIVIRHEHRMKTAKTVIDCYSGVLFLQMLAITLLLCFESFQMYTINTPVIKIVFLFFYVGCVLTQFYNYCYSGEKLITKSTKMADGVYKCKWYDLLSXKTQDLMLIVYRSKIPLQLTAGKFGVFSMQMFGITLKTSIAYLSTLLTIK

>DanaOr98b__scaffold_13340_residues_16984316_to_16986520_forward_strand.[501_1705].sp

----------------NFLRLQSIFFSFLGFELQQDK-----E-VSHRYPWRIIFFLLSVATFLPLTIALGISNIQNVEHLTDALCSALVDLVAIFKIGFLLWLYADLRHLVKRFRCKLQSEGQYGDCEAIILAYNKRDLFISALYCLCFLLAGVSACLMPLLSIF---IIYLR-TGE---V-QPELPFPSVYPWDNKRPLNYLISYLWSVSAAVGVALPTVCVDTLFCALTHNLTALFEIAQLKLMNFMGKSL--------------------------------------------------------------------------------------------------------TRENLVHAFQLYGECLELGQSLNGYFRPLIFAKFVVASLHLCVLCFVLSTNLMEPAM-----LFYVAILGQVSIYCFCGSSVKEESQQFAQAIYESSWQQLDVKVARSLQFAMMRAQRGCRID-GYFFEANRQTLILIVRSAISSVALLRSL-

>TcOr128

-------------------ETSLTFFKVLGIHPLK-----------TLRKIPVLCLISSHLILFALVCLRFFYEKITFGLISDTFESTFTIVHVLAKFITLVIIKPTIKKLFETTNTFWLGKTFQKEFRHEVDADLRTLGLLLRLFLFFVSIVIVTFAIRPIFDD--------------------TLAIHCYVPFIPR-----AIFIVFNNAVFAVSAFAVGSFDMFVCVMVVLITVQFKILNYSI--------------------------------------------------------------------------------------------------------VTENDEKLCQKKIKIIVDYHDFLDRYLAKVSSIVSPALLVYFTIAPIVLCFELFFMSK-SSNFAEIAKSCVYILGIVIELFFFCLPATYLMVQTQLMITTVYNSGWENCYLSVRKSLIIMMQRMQKETLLTAGKMIKINLETCTNAFRMALSFYTSL----

>TcOr129

--------------------FPLKLYFLFGYHPDC---------SPKFRKLALLAGLWMYAFPFYLSIKGILFYRKDFLLMLECLEAAFLFGEPLFRHLAIHYHVSNLKNVLNLRQKAQIDGLEGES-----LFYYNLGKTFTFNYMIAGFVVLFGFCVQPFIS--------------------GKLPATTYLPE---G--YFVTFCIYYTLSGCYVVVTVVTTDALFCSLCTTAIVNFKILKRKIRNIKRTN-------------------------------------------------------------------------------------------------------TNLRHEVKKIVDQQNFLLRYCEALGEMYSDIFVVYFCFSIGAICMQTYISTNDQLESAIRIKTSIYAVGLFAQSILYSISAENVLSAASEIGDAAYDSPWYRFDAQYTKSLILVISKAQRKVIFSGCGLVTINFTTLTVIIKTAVSFYAYLNS--

>MsexOR-28

-------------------------------------------------------------------------HTGDLLAAMVVLSLGISVQIGTLKFFYTFVYINKETNIVKYYLECDSLIVPGGRFSGNLLRALRNVKKRAIVYWLVIIINGITYVTKPMFMR------------GRHHMEDRYVIYGLEP---MFESPNYEFAYFLMTAGLCFICYPPANVTVFLIVVVGYTEAQMLALSKELLHLWTDANEHYQKNINQHET------------------------------------------------------------------------------------KNKIINDYVRYRLKEIIKMHAFNIHLVRQVEFVFRGAIAIGYVFLTLGLIAELLGGLE---------NTYLQIPFALIQVAVDCYTGQKVTDASLIFERAVYDCKWENFDKMNMKTVLLLLQNSQKTMTISAGGITMLNFSCLMSVIKSIYSAYTTLRT--

>LhOr67NP

---------------NYAFALSRQSVRILGVZPDPHP------LSYFRRSNFVIVVCILSFYVIIPQLRNMIRAWGNIMDMVECLVSVNFSLMALCNLVAIWYHGKALKTLMASVTNDWITWTSDWERNTMLKI-AKRGRSLSFRCYVFYVSSAVFYVWFHSLKL--R---------RSMHQPQRILAYQFGYPYNTQKSPNYEITFFLQLSGGVYSGLINCIVNSFITMLLLHVCAQLKNLRTTLNNKVDELAK----------------------------------------------------------------------------------------NKVDELAKNSISTLRFKEGLAAIVIRHEHLMKNAKTVIDCYRGVLFLQMLATTFLLCFKSFEVYTIDTPIIKIVLLFCYTSVVLTQFYNYCYSSEKLITESTSMADGVYECKWYDLSENAGPNAHRISI--XTPLQLTAGKFGVFSMQMFEITLKTLIAYLFTLLTIK

>TcOr121

-------------------------LWLGHMHPFF----------PFRRSFAFLIINLTACFLMIALAIKGITYNNDIFFVAECLQTCNLMLHGVGKFLNLYFHRNGLQALLENRSKFWKIDDFKCENIYEDLSGTSTVKRGLRYYYCGALVVIFLFDLQPFAT--------------------GLLPTGCYVPEGWFK--GLTLTLW---LLSISFFLIIQSTDGFFCSLSVAIVIQFKLLSHRFKNM--------------------------------------------------------------------------------------------------------ESERKMWKELKGLVDYHNFLTNYCKQLNAAFAPIFLLQFLVSIVSASVSIFIFMQ-PGAWSNRIKFVLYYLAIMVETSFYCVPAEIIVNAASEIGNAVSDLDWYKIKNKVKKCFIIILARTQKTMVFTGYGLVNMNLQTFVIYVMTVFSFYTYLNSVR

>MsexOR-29

-----------------------------DFWYENKG-----DDKRILYRMYSCALFFIYGFMTVLEIMAALMGEFPTDEKRDSVTFAVSHAIVMFKIISVVFQKELVKTLNRKMVTICEHY---EEQ-ALMSEKYRIMKINVIVYFLIVYGSAACFVFEGLRK-----LFDG----------SHFVTVVTYYPYEDNSMFANSVRILATVILFLMLMNMIVCVDSFTMVYLIMYKYKFITLRHYFENLSVTIDKLNTPGN-----------------------------------------------------------------------------------------------KMLTDGLVEGVEMHSKLLRLSKDIDKAFGTVMALQLCQSSGSAVSLLLQIALSDQLTFASLKIVFFVMALFFLLGLFLCNAGEITYQAAQLSDAIFYSGWHVCSRHVRKIVLLAIMQAQQPLVMKAFKMLELTYGTFILVVRATYSVFALFY---

>gi|380016536|ref|XP_003692238.1|

---------------------------FLGQYPNQ---------SRWNKEFNTNVMICSLISFLIPGLLQVYTVEKNLNALMEIIPIVFATISCAIKLLNHRINKKNFDKLFDFMSEEWEMENDRNQTC-ILDEFTKQGNKFAEIYKNVLLSALLLFLLLPLFPSFLDVVFPLN-ETR-----QRQQLLRVNYIFDTND--YFFYVYLQLAWGSIILVLTIIAADWFYILIIHFNSGLFAVCGVQVLEATMN----------------------------------------------------------------------------------CGVQVLEATMNSNLVSKDAFSENSSYEKFRACVIMHNEVIEFYNILNESCQYSYLIQVGLNMLGMSTTAVQTVINLDRPDVAIRSAVFFAADQFHLFLLSLPGQVLLDHCADFANIIYDSTWYGTSLEIQKMLYMMQIRSKKLCALTAGGLYDMNIENFGITFKTCISYFTMIMSLK

>McOr25PAR

------------------LKYSLNMMNLVGLHPEK--------GNSIIQNIQCIGTIFLNFMVLILILLLLAKHRHTMTDITEVFESIFMIVHGSSKLILLYTNRSKLLAILEGTKQFWDMEKINDAQ--VKKSCDRSIKRLLCVYLLFFIFAVFVTVIFVF-RPVYQ---------------KGSLMFNSYVPKDVP----FSVIAILQTYVFIWGIFLTLGFDLFFTTIITLIEIQFKMLNLNIQRIYDFNESKK---------------------------------------------------------------------------------------------NESKKDLSTEKKLKKWVKHHNFMLDYVDLLNKTVSLCMLVYFGIIVLSMCMELYNML-IPETLSNKLRAALYIIAVFFQFVLYCIPSQILTNEADNICISIWSSTWYEFSTQMKFSMKQIMMRSQKTMYIMAGNIIYINLSTCLATLKTIVSYYMFLKTM-

>DficOr22b2

-------------KSRDAFIYLDRCMLFMGWTEPNDK-----RWRLPYFLWSVFWCALIYIYMPFAMTVEYVRGFKSFGEFLSSFEIGVNVYGSSIKCSCIMLGYKKMQKAKRLLDQLDESCVQDEEK-ITVRRYVALASLCYIIYHIL-YSSFVALNFFYFLTI-------------------RSHAWRMYYPVDSDD--MFFLSSIAEGIMMTVCVTMDQCSDVCPLMYTLMARCHITLLKKRLRNLRSVSEMT--------------------------------------------------------------------------------------------------TEDEHFVELTNCIKQHRLILEYFKASGSVFSRTVFVEFLLIGVVLGLSMINLMFFSTLWTGFV-TCLFMVDVCMETFPFCYVCNMIFDDCQELSDCVFQSNWVSAEPRYKSTMVHFLHNLQQPVVLLAGGVFPICLQTNLSMVKLAFSVVTIIKQF-

>DbipOr19a

--------------STRPLKLHWLIFRVMGVHAPK-------GRESLWGRHYRTYSIFWVTFHLCMSMVVNFLMSNSLESFCDSLSVAMPYTIYMLKLVNVWAARDRLLQTHHILRYLDTRLCSATERRIVRTKGIRRSHRVLWNIF-IGVAAVFICGIAYIILS-----------------SERTLMYPSWIPWQWKESRVFVSTVAAHTGCLAETAVLVYNVATYPCTYLILLSAHTRALACRFARLGHSARETQQQ----------------------------------------------------------------------------------------------ETQQQTHLRLLDYIWDHQMLIRILKSLERSLSKTCFMQFFCMACSQCTICYLILFEKIGFMPLVNMLCLLLAFTCETLLLCYSAELVHQAGNDLLSAVYSCNWLDQPVLFRRYLVLVLVRCQKPMILYSGIVVPLRMTSFXXXXKGAYSMLTLLSKMR

>PbOr53

-------TKEWNEKSHYALSVYKRVLSIIGVWPLT--------AGEFKSIARCSLAILLQISTIGSLSLEAYRQCLGTEDMMEAFLMDLSSVVSLSKLLVIRFTWRHTYVLVTSLIDDWSISRDSRQREIM-MRYTHVGRILTILYLGYASGVSFLFMAVPFDRLIPWLNMTKT-MNDNDTM-VSTYFLATYCVFGSLPMTVHICVLLLQVAQIFVNATSHCGNDGFFFGLAMHLCGQFEVLQMDFARIE---------------------------------------------------------------------------------------------------------ERACKRKIRMLINRHSHLISLADSFEYAFNMAIFAQLLMSVLLLCVEGMQLIISLNDNIAAIKHVVLILTMLVQLYLYCYAGDQLEYITGKIGYSVYNSPWYNFDVKIMKDMPMIMFRGRIPHQITAGKFLRMNLFSFKEILKATGSYLSVLRVM-

>395_Si_gnF.scaffold02814_302873-346762.pep

----------------REYHVNKIFLSHVGLWPLQ---------NKFIKNLIPISLFVLHLSNFPFEILMLYDHWEDKQLIFETFHFTTSLVMFIAKLFNEFWNYDKVQRLYQAMENHWNTFTNEFEV-RILKDYSSQSRKIIIIYSTI--TTYIMPSFTPILLD----IISPL--NE-----SRPRIFSMSFEWRIDMDKYYVPIVCYNTITLVTGIIICIGIDSMYITRIFHACSLFSIVSEQIENITSTQH---------------------------------------------------------------------------------------------EEHELSRKEQVLYQQYVMCVKKYQIALKFVDILNSTHQTVAVFFLLLICATLSLIGVRIVYVLGQLEEMIRFTFIIVGALLQLLIMCYSGQKLIDESENIFHRAYAAEWYNFSPRLKSLLIIILHKSIVPCKLTAGNLFPLSMAVFAAVIRTGISYFTAFLSIK

>gi|383859449|ref|XP_003705207.1|

-------RPFSEKEYNQLIKPIMLTAKIISIWPLAES-----TGTIMLRRFHLFSMFFLVVVMSLAVTADVVHNLDDLDEATECALICTAFYLCVVRLTVYTIHQKDMLYVVNTMKEDWITSSL-EDR-VVLEEKTMFAYRLAKYFISTVAITIILFMCVPLLEI-------YV-LGAS----EKILPFRGYFFINQTISPIFEILYLFNVTAGGFGGSMIAGATSFNLIVIMHGSAKFAVLRKRLEALNGA--------------------------------------------------------------------------------------------------------------MADCVLRHRRAIEYADALERIINVLALGQFMISTGLICFAGFQITSMMQDKGRLMKYSTFLNSAILELFMFSFSGNGLIDESEAVGESAYSSGWI--GSRLSKSLQIMMMRSQTPSKITAAKFYSMSLQSFSSVLSTSFSYFTVLKATK

>DtakOr7a

--------------SRRAFRNLFNCFYALGMQAPDGP-----TKSATWRRIYRCFSVVMYVWQLLVVISYRYMGGMEITQVLTSAQVAIDAVILPAKIVALAWNLPLLRRAEHHLASLDARCRDREEFQ-MILDAVRFCNRLVWFYQICYAIYSSSTFVCAFLL--------------------GQPPYALYLPLDWQRSLQFCIQAWIEFLIMNWTCLHQASDDVYAVIYLYVVRVQVQLLARRVEKLGRDGQA------------------------------------------------------------------------------------------------DARRQEEHCAELQRCIVDHQTMLRLLGCISPVISRTIFVQFLITAAIMGTTMINIFIFANTNT-KIASIIYLMAVTLQTAPCCYQATSLMLDNEKLALAIFQCQWLGQSARFRKMLLYYLHRAQQPIALTAMKLFPINLATYFSIAKFSFSLYTLIKGM-

>DgriOr30a__scaffold_15126_residues_3158383_to_3164100_reverse_strand.[1501_4218].sp

------------------------TLKLMRFWSYL-----------FVHNWRRYVALLPYALINVPQYVDIYLSTEPLDFIIRNVYLAVLFTNTIVRGILLCVHRKRYEQFLELLKVYYLQLLNDEHIGQLVSETTRLSISIGRINLLMGTCTCIGFVTYPIFGS------------------ERVLPYGMYVPIDKYASPYYEVFFAVQAIMAPMGCCMYIPYTSMMVTFTLFAILMCRVLQHKLSSM-------------------------------------------------------------------------------------------------------------VHDEIIWCIRYQIKLEGLVDAMNALMTNLHLVEFLCFGAMLCVLLFSLII-AQTIAQVIIVVFYIVMIFANSFVLYYVANELYFQSFHISIAAYESNWMDLDIDSQKTLKLLIMRSQKPLAILVGGVYPMNLKMLQSLLNVIYSFFTLLRR--

>HsOr303

---------------NHYYHLNKRLLSIIGQWPYQ---------SRLESNTMLGITLFFTGSLTFLEGWGLVSGITDLSIIMENASPMLVNIFIFVKLLNYFFNKYKMKELLDHVEETWKMMQVGPRN-EILRSYAEQNKVYTIRYTLALYTMWILYSTTPLIVSWTYKLLPIN-ATYT-----ARFLYRLEHVCDVDK--YFNLLMLHGFISVFYIVSVPIAVDTMFVLCIQHVCALFEIIKYDMEHIQGS-----------------------------------------------------------------------------------------------------------YHKIIECIKLHDQAFKFSELLSFAYATSYLFLLGNVIICLSFSLAELLMVDLQFDEIARFSASNIAQLLHIYYLSWMCQRLLDYSGDLHKVMY-----------------------------------------------------------

>gi|284010022|dbj|BAI66646.1|

----------------NYIKTVENFLGASGIWPSNID-----KLQPLVFRVHKQTLPYHTMLIVFGGYLSDNFHRMSFLDMGHIILSTFLAMVTAMRSVVLKVYAALVTKLGREIHLMHFAHKGPYYEEMNKMV-DKASYIYTKFIVVVMYLAMLMFNFAPMYNNAKNVLISKT-ENYT-----MEFALYYSYPFKPLN--YFPTTTLYNFYLSYNCGIMLCGLDLVLFLMIFQLIGHVYILRHNLENFPSPKN----------------------------------------------------------------------------------------KENCIVEMFDAKENEEVRVRLAECIEHHKIIIRFTDEISIVFGPLLAFNYMFHMVGCCLLLLECS-A---GNQIIRYGPLTTVVFGQLIQISVMFEMLGAETEKLKDSAYFVPWECMNTSNRRTAHIMLHKMQDKISIKALGLAAVGVNTMMGILKTTFSYY-------

>McOr46PAR

----------------------------------------------------------------------------------------------------------------------------------------------------------------------------------------------------------------------------------------------------------------------------------------------------------------------------------------------------QLLNWNAKDLFTDTDEGEIHSRVKHWVDYHNRLLDFVQLFNASFSLSTLFYFGIVVLSSCMELFIISI-QPPSSDYFKYILYIISLNFEFIAYCLPSQIVTNEAEEIGSHIWATDWNGLSTSVKTYMNMILMRSRQAVIIRAGTV-DANFETCLAVLKMTGSYYTFLKTM-

>DmelOr47a__2R_residues_6765885_to_6767926_forward_strand.[401_1742].sp

----------------SFLQVQKSTIALLGFDLFS-------ENREMWKRPYRAMNVFSIAAIFPFILAAVLHNWKNVLLLADAMVALLITILGLFKFSMILYLRRDFKRLIDKFRLLMSNEAEGEEYAEILNAANKQDQRMCTLFRTCFLLAWALNSVLPLVRMGL----SYW-LAGHA---EPELPFPCLFPWNIHIIRNYVLSFIWSAFASTGVVLPAVSLDTIFCSFTSNLCAFFKIAQYKVVRFKGGSLKESQATLNKV--------------------------------------------------------------------------------------------------LNKVFALYQTSLDMCNDLNQCYQPIICAQFFISSLQLCMLGYLFSI-TFAQTEGVYYASFIATIIIQAYIYCYCGENLKTESASFEWAIYDSPWHESSTSICRSLLISMMRAHRGFRIT-GYFFEANMEAFSSIVRTAMSYITMLRSF-

>gi|312385812|gb|EFR30221.1|

------CNTEEDRTITLNFRMLERILRFVAVWPTDYP--------ALLDALYLLFWLCICLHIATFHVISVTTVPVSYDELFLALITTSIYSIMAALYGYLRLYERKVRQLREYSDRLFRQRSAPGIYYLSISSCYRFANRCLGWWLIFCILGTMHWAIYPILSQ------------------NGQLPFPCWYPVDVQRSPVYELAYMCQVLGQLQVSLVFGLAGALFMVYVFIACNQFDMLSCSLVNVRHTAMLLNGGYGAELRHAQGHCS--VDLRDYFLQEAFLEDL-DKMADVVVLHH-------------------------------------------------------ELTVALDDCVRHHLVLLEFCERLEDCYHPYVLLKLFQILLLLCFLAFMATVVSN----------------------------------------------------------------------------------------------------

>NvOr108PSE

---------------------------------------------------------------------------------------------------------------------------------------------ITLAYATSVGTCVLIILLNPTVS-----------------ADAWATPVYSWIPCNINFSSCFWACYLHQSVGTATIAIVHVACETLVTGFMLQICAQLNVLNHRVLSIDLKIRNLTLREKDENRILMAETF--L-----------------------------------------------------------------------------------------ACVADHNGILKFSKLLSETFIQVLVIQFCASLTVFCTSIYMLTKIKVNTLDFLLMTTYLICMLNQILLYCWYGNEVMLNSQKLVQSIYNADWIALHGKTQKTLLLMMLVASSPIQLFEGAIIKVNLDAFLNILKFSYSAFNILHS--

>DyakOr98b__chr3R_residues_22322598_to_22324862_reverse_strand.[501_1765].sp

---------------DKFLRLQSVFFRLLGLELLHEP------DAGHRYPWRSICCILSVASFLPLTIGFGLQNIQNVEQLTDSLCSVLVDLLALCKIGVFLWLYKDFRFLIRQFYCVLQRETHCLAGELIVTRESRRDQFISAIYAHCFITAGLSACLMSPLAM----LISYQ---RTGEF-QPDFPFPSVYPWDNKKLPNYLISYVWNVSAALGVALPTVCVDTLFCFLSHNLSALFQIARHKMMHFEGRNAAETRENLRHV--------------------------------------------------------------------------------------------AETRENLRHVFQLYELCLQLGHSLNEYFRPLIFAQFVAASLHLCVLCYQLS-ANILQPALLFYAAFTAAIVGQVSIYCFCGSSVHSECQLFGQAIYESSWLHLNPLLVSSLKIAMMRSSLGCPID-GYFFAANRETLIRIVRSAISYVTLLRSL-

>gi|340710656|ref|XP_003393902.1|

------QLTKREEDMKYATRYVKPILGAIGAWPVSFS-----SSSKILLRTEHILTYFLFFLIIVPTIMYVFFKEKNNKIRLKLMGPIINCTMQFCKYTILLWRTNEVQKGLDVMKQDWITATDENRL--IFRSKAKIARRVVLTAAITMYGGGLCYTILPLLKG------PII-TPDNIT--IRPLPCPSYFVLDEQKSPNYEILFLVQILAGFVIYAVISGSCGLSALFVLHACSMLRILVDKMKALVDMR------------------------------------------------------------------------------------------------------DTMVQRRIMDIVEYQTKIKRFLKNIETITEYICLTEMMGGTCLVCLVGYYILMENNNIAAVLIYVTLQISCTFCVFILCYIGQLLIDENQIVGQASCMINWYHLSTKHMRSLILIIAMSNYPMKLMAGKMIEMSLATFTGVMKLSMGYLNILRE--

>gi|383865809|ref|XP_003708365.1|

-----------KYSTDIAVRLTFFYLKIIGFWFAR-S-----RLQLWFRHLMVIYTFIMNMFTMWLQCMGIYHFWGDFTMCTYILVNVSGIIIILVKLCFLSVKKKKFLRLIEFMQKNFWHSNYSQKEIQIFAGTKRICIYFVCSFTFVSQLTVVLYSVRPILLNIGK--------NES----ERVLLFHMWLDLPVSMSPYFEIMYVLQVLSLCQCNIGFTCFDNIFCIMCLHAAGQFRILQYRLKNMREMAGI-QEDNLKSASYFSSKYF-------------------------------------------------------------------------EDNLKSASYFSSKYFMVFKNCVRQHQMVIAFCTLFEEVFSAIVLCQVIMFSMLVCLLGYQLFLVDLNLPMLVSLISFISANLCQLWVFTYSCDTMTRESLNVGTAMYTAPWLQLPTMIRKDLQIVIMRSRRGCHITACGFFPISLETYTSIMSTAMSYFTLLK---

>DpseOr49a-1_Ch3_residues_7497548_to_7499893_forward_strand.[498_1846].sp

--------QEKQREYQDFTFLANIMFKTLGYDFLDSP-----SWQTGLLRCYFFVCIASSSYEAFFVALECLQVESSPSKIMRRALHFFYMLSAAVKFVTLMIYRKRLRTLILSLKELYPADESLRREYEVNKYYLPRSRYVFYSY----YCFMAVMAIGPLPQSFMMYFLKGH-------F-PFLRTFPTQLCFRSDTPVGYAVAYFMDLTYSQFVVNVSVGADLWMMCVSSQICMHFGYLAKKL-----------------------------------------------------------------------------------------------------------EREREDCEFLASLVQKHQLILRLHKEVNQIFGILLASNLFTTASLLCCIGFYTV-VEGRSEEGMSYMIIFVVVSAQFYMVSSFGQQLIDLSSSISMAAYSQYWYDGSLRYKKDLLLIMARAQRPAEISAKGIIIISLDTFKILMTITYRFFAAIRQ--

>NvOr184PSE

---------------------KWISLIVNILWPYQ----------STLRSISNYVIFTVTLVSMVIAMVAGIYSETKLLNILKGSLPIIFCSGALVAYMLLYSRKKEIKERTELMATDWMELADDSECKIMXNFFARSAINITIGYGIPVYGLFFGVTFMPRIFN-------KK-SLEQC---LRSFPYYFKLIIDENT--CDLQVCVHFALAILFSSFAFLTISSTYIISVKHICGLYGIACHRLRNA--------------------------------------------------------------------------------------------------LKKSSEDYDTTVIHSLIKVIDVHKEALRGIQIIEKHYSFGFFFLEIGAFVVLAILMFEINYHKEHISELLRFLPLLLLFTTYVFFMNWCGEQVIQSCDDPRISVYNMDWYRTSSRIRIFVLMIMQRTTKPVHLTAGTVMLLSIKNFATILKTASSFGMLLLTT-

>DbipOr43b

------------------------TLRMSGLNLGE--------KSGVAWKIWRVISYTYSMLILPISNYTIHLAEFPPDLLLQSLQLCLNTWCYAFKYFTLVIYTRHLELSNIQFDEMDKFCVKAEEKR-KIRDSVAAVNRLYMVFVVVY----ILYGTSTLMDALI----------------HDRVPYNTYFPFNWRLSTQLYIQCFLEYFIVGYAIYVATATDSYALIFVASLRTHITLLKERIISLGIINN---------------------------------------------------------------------------------------------------TDADSVFTLLVDCVKAHRTMLSFCDAIRPIISGTIFAQFIICGSILGVIMINMTLFADKSSQF-GTVTYIMAVLLQTFPLCFYCNAIVDECNELADALFHSAWWMQEKRYQRVVLQFLQKLQQPMTFTAMNIFNINLATNINVAKFAFTVYA------

>DbipOr43a

-------------------GINVRMWRYLAVLYPT-----------PGTNWRKYAFVLPVTAMNFMQFLYLLQSWGDLPAFILNMFFFSAIFNALMRTWLVIIKRVQFEKFLNQLSVLFHSIREDSDERDILRQAEHEARHLAIVNLSASFLDIVGALISPLFRE----------------------------------------------------------------------------------------------------------------------------------------------------------------------------------------------------------------------------------------------------------------------------------------XVAEAVYNVPWYRGSIRFRKTLLIFLMQTQHPLEVGVGNVYPMTLAMFRSLLNASYSYFTMLR---

>gi|238623742|dbj|BAH66345.1|

--------------------------------------------------------------------------------------------------------------------------------------------------------------------------------------------------------------------------------------------------------------------------------------------------------------------------------------------------------------------------------------KLINEVTDIFNPCLTFQFFTSSVAICMVIYKLSDTYIVSLEFVFLLNFIFVLLTQMFIYCYYGNVVSYESKYINTSLYLSDWSSASPGVRKMFLIVMPRWTRPLVVRIARVVPLSLDSFVSVRKYKCS---------

>DgriOr42b-5__scaffold_15245_residues_8727943_to_8732254_reverse_strand.[1501_2812].sp

------------------FIYLYRAMKFIGWIPPK---------EGLLRYVYFCWTLMTFGLCTFYMFLGSYITQISPGEFLTSLQVCINAYGCSTKVIIIYSQLWRLIKARELLDKLDVRCTSLEEREKIHSV-VARCNHVFFIFTIIYCSYGVSTYLSSVLS--------------------GHPPYQLYNPFDWHDGLNMWIVSTLEYLIMAGAVLKDQLSDTYTLVYGLTLRTHLELLNGRISKLRTN----------------------------------------------------------------------------------------------------EMTEDENYEELVNCVLDHKLILEYCALIRPVISGTIFIQFLLIGVVLGIALINLLFFSDVWTG-LACTVFIVAILLQTFPFCYICNLVVDDCEALAHAIFQSNWVGSGSRYQSTLFYFLHNVQQPIVFIAGGILPITVSSNISVAKLAFTVITIVKEM-

>gi|340718194|ref|XP_003397556.1|

---------------------------------------------------------------------------------------------------------------------------------------------------------------------------------------------------------------------------------------------------------------------------------------------------------------------------------------------------------------------------------------------------MFIQMLSSTSIICLSGFQAVVVGGQSSDVMKFGIYLSAAISQLLYICWIGNELNYATWTLDRSQWLSGWNNRLTNIVKMFTLSTMFTRRSITLKASVFYVLSLETFITIIRRSYSIYTLLNNM-

>DgriOr67c_scaffold_15110_residues_12664426_to_12666850_forward_strand.[522_1925].sp

----------TPRTFEDMMRIPVLFYKTIGEDIYAHR-----STNSLLLKIYLYAGFINFNLLVCGELVFFYKSIQDFETVIAVAPCIGFSLVADFKQITMALYRKTMIQLLDDLEEMHPKTLAAQSLY-QMADFERTMKRVIRIFTFLCLAYTTTFSFYPAFKATIKYNFLG-----YETF-DRNFGFLIWFPFDATTKWVYWITYWDIAHGAYLAGIAFLCADLLLVVVITQLCMHFNYISTCLES--------------------------------------------------------------------------------------------------------ADQDEENVRFLTTMIKHHNKCLMLCEQVNNMYSFSLLLNFLMASMQICFIAFQVT--ESTVEVILIYCIFLMTSMVQVFLVCYYGDALIAASLRVGDAAYNQNWFQCSKTYCIMLKMLILRSQKPAMIRPPTFPPISLVTYMKVISMSYQFFALLKT--

>DkikOr83c2

-----------------------KFVAFLGVDILA---------SCLKFNYRTWTTFFVGGNFMVFTIFSIFNNGGDLEKNLKATSMIGGLIHGLGKLLTCILKQQDMRRLTFFTRGIYEEYENRGCHYRTVLHKITRLLGLIRIIRNGYFVTIFIMMSLPLAMLMYD---------G-----TRVTAMQFDIPLSLESNIGFTATYLIHLVRIWIRNVGFYGGDLFVFLGLTQIITFADMLQLKIDELNEAL-------------------------------------------------------------------------------------------------------------LLDVIKWHQLFTSYCSTVNAIYHELIDTQVIAMALSMLLS-FCL-NLSGFNL---SLVVFFVVAAYSMSVYCFQGTIIEFGYDKVYESICNVAWYELGVNERKLFGLMLRESQHPHTIQILGIMSLSMRTALQIAKLIYSVSMMMMN--

>GPRor20

-------------DPKAVMPVVKRLLKLSGLTQET---------TRFGAI--NFL-NL-FIYIAAVLVPKVCFPYPNTEAMIRGLSELIFFTNVYVGFFCFISQHRHYRDLLNAIDPTAAHPSDSPSEQILIKF-NVKIQKLSVVFCWYIAVTATFYWLAPCLMTYRSIYMASV-SVENGSVQYYPNLEESFYWLDNRSSVYGYATFSIVALLVFTVASYNNATKLLILTTIKYCNTLLQLVIIEVDNLNHAT------------------------------------------------------------------------------------------------NLNHATSNAIDRELKQVIQLHQRAVRCVALLNQTLSFVMTVQLALCILTWCFTLLYILTV-GLDVTGMNGLLIMFNMTLEMFGYCFFCTELATTGTLIACQSYEFRWEEHDPKIQKMISTIVARSQLPLRITACGFITVNVELFAKVVKTTYSGFIVLK---

>CpluG2R504O01BDC83

-------------------------------------------------------------------------------------------------------------------------------------------------------------------------------------------------------------------------------------------------------------------------------------------------------------------------------------------------------------------------------------------------------------------------------------------------------------------------NASPIIRKLWLSVIHRTQKPMTINIPGFLELSNEYYANFLSTAFSVFTAMHA--

>gi|170057539|ref|XP_001864529.1|

---------------------------------------------------------------------FMLQHTDSFNLVFEGISVLVAGLDAWFGLVEIIVNRKAWVALMKDVSSRRSMYKSAKISA-LFDEYYERNIMFCKFLYGV-YMSTFSYFLLPAILP------DP----G-----KYNLPIPATIPLVPDTNKLYWITFLIQLLMVGIAQHVLIAQCSSLIIGIMSACCQIRALKIKLEDLNEQIK--------------------------------------------------------------------------------------------------------VHESLGEIIYLHASTKDYIRLLQRKAAIVYLSVFVTCGGIVCSCLNVIAEDLFNSAN-----ALMLAGTFSVLVHCFFGNTLLIENDSLPDAIYAIDWYKLPLADQKAFKFLLANAQP-----------------------------------

>CfOr220

------------------LKFSLTVLAVAGCWRPTS------WTKTIMYNTYSLSVILTLYTFAITQIMELILNADDADAFGDALFNVITSLLACYKAIVIQKSHESIITLINNLAETPFKPLDLNENMIQEK-FNKRITNNTLCYLVLILITNLYMILLSLFTDFK----------------NGTLMYKAWIPFNYSMSALFYPVYIHQLITLMFSGLVHSTCDSIICGLLLHICCQIEILEYRLSNIMNARENLRDCVHHHIHIFESYIY--TGSTKSKLLIQKFLT--------------------------------------------------------------------NARENLRDCVHHHIHIFESYIYTGSTKSKLLIQKFLTFSRIIEKI--RVTLIRHNSAKKIVQISFLICRDKSIF-YCNYNEICDKNSLQLSDNIYNTEWTILNNNVKKGLLMIMNRATIPIEFTSANIISMNLESFVMVLKTSYSLFNVLI---

>DficOr65b

-------------------------MKAMVLYTTS-K-----ERQLPYRSIWHTFVLIQTSVFFASVCYGLTESIGDNVQIGRDLAFIIGAFFIIFKMFYFLWFGDDLDDVINELVKHHPWAWKGPGC---V--DVRPLKRWSFCLGFFLASSWSFFLIIFLLLL---MTTPLW-------VQQQNLPFHAAFPFDRSTHPTHAIIYISQSFLMTYAIVWLMCIEGLSVCIYFELTFATEVLCLELKHLHRKCDD-----------------------------------------------------------------------------------------------------AKLRLETNRLIRFHQKIVEILNRTNHVFHGTLIMQMSVNFALVSLSVLEAMEARKDLKVVGQFGVLMVLALGHLSMWSMFGDMLSRESLKISQAAYEAYDPAGSKDVYKDLCFIIRSAQNPLIMRASPFPCFNLVNYAAILNQCYGILTFLLK--

>NvOr91PSE

------------------FNPAFEILTCFGFWKPTST-----PWSERFYDGIRVMLVSLLYYLALGQILRLLLDTISIDEMADTLFSMMSTVNACCKLTNMHLRNKQIDELMDMLRIEWTKPGNKEENVIYN------GFNDIIGYVTLVEPATLLDLSIPIIVGK-----------A-----EQSLPLEVWTPYNX----------------------------------------------------------------------------------------------------------------------------------------------------------------------------------------------------------------------SSPEFYMMLFYLSSVMTQLFLFCWFGNELMIRSQELETTILESDWTTLSLQSTRSILLISLRTSKPILISRGYFVPFSLETFKRILKVSYTAFNEL----

>DbipOr67c

------SEDNTARTFEDMMRVPVQFYRSIGEDIYAHR-----SKRSLLLKIYLYAGFINFNLLVIGELVFFYNSIQDFETVIAVAPCIGFSLVADFKQFAMVYHKQTLIQLLDELEDMHPKTLAKQKEYKMS-RFEQTMKRVINIFTFLCLAYTTTFSFYPAIKATVKFQFL-----GYDTF-DRNFGFLIWFPFDATSSVVYWIMYWDIAHGAYLAGIAFLCADLLLVVVITQICMHFEYISMRLEE------------------------------------------------------------------------------------------------------------KENKEFLISMIRYHDKCLRLCEHVNDLYSFSLLLNFLMASMQICFIAFQVT--ESTVEVIIIYCIFLMTSMVQVFLVCYYGDSLIAASLKVGDAAYNQKWFQCDKTYCGMLKLLIKRSQKPASIRPPTFPPISLVTYMKVISMSYQFFALLRT--

>AaOR119

------------------------LLLLGGIHSESTT-----WAHERKIRIICRCLFAYHAVAFVLQLNDALYEEKKAALVVWELMKVIFIFVAYLKVVLVVQLKGSIATLRQFIRSNHICSGDIEYD-ELEQNKFNKIVRITIQVVFVLIIIDTLILSVPNFSN-----------ND-----LLKLPHLLAL----TGMPSYILKILLTSCLGISVIPKYFACTACVGAVLIGMRTRLRILAHRFEHISQQDFTSEEK--------------------------------------------------------------------------------------------------CVNRDIQEALAQHLEYWSHLKAMKIMVSKTFLKVHYFSIVAIGSLIYVCC-AMGVNGVTFVIGAGTVSFLMEYYLLCHLVDLLQDEADSIGYHIYNSELRSEYVQLRTTLMIVWINTRNGISLNCLGLFEITTFTFVTLIDAAYSVLMFLIKM-

>LhOr252

---------------NRYYQLNKRLLSVIGQWPFQ---------PRREANVMFAITAFFIFSLTVLEFWGLISGITDLSIIMENVSPLLVNSLVIIKLVNCLFNKYKMKNLLEDVEETWKIIQVGPEN-KILRNYAEQCRTNTIEYAIGLYITSFSYSTMPIVVSGVYYFLPT---NETY---TARFLYRLEHVVDVNK--YFNLLMLHAIVSIFYIISVPIAIDTLYVLCTQHVCALFENIRYNMERIQDLHFD-----------------------------------------------------------------------------------------------------DKAYHIIIDCIKSYNRAIKFTDLLSATYATSFFFQLGNVIISLSFSAAELIMVDNQLDEIIRIVCANVAQLIHIFYLCLTSQRLIDYSSGLQRVIYSSNWYMISLRSRQLLKFMLLRTTKPCQIKAGKMYVMSLENFSSILQVSVSYFTMLTSL-

>cplu_012680_b1

-----------------------------------------------------------------------------------------------------------------------------------------------------------------------------------------ELPVRALFPFDTRTSPMHEVAFFIQIYSIAFALTNVVTLEFIGLGFIRWTTVQLFILTWNYKNCRTDI----------------------------------------------------------------------------------------------KNADHDGSKDCYLWRFQICIKHHQKLTWIVKELNSVFSSSMMMQLAASATMICLAGFQAVLGSNDKSSFMKFTVYLGATFTQLLYWC-----------------------------------------------------------------------------

>DbipOr67d

--------------VDRYLRIIKVIRFFVGFCGNDVN-----PNFKMWWLTYLVLSAIGLFYACTGYIYRGVVIDGDLTIILQAMAMVGSATQGLTKLLVTANLAPLLRHIQYSYEDIYREYGAKGEYTECLERRIRITWRLMLSFLCPCLMSMVSIVSFPIFYL----VVYK----------KKIMVMQFLMPIDENTDGGYMILSAVHVGLIAFGGFGNYGGDMYLFLFVTNLTLIKDIFCIKIKELNEVVLKRTEYEQMRLLLF------------------------------------------------------------------------------------------QMRLLLFDLVAWHQKYQKILLTTRRIYSIVLFVQLSTTCISMLCTIACVFL--HVWPAAP---IYLLYAGTMLYAFCGLGTAVEISNDALMREIYSCLWYELPVKEEKIIVLMLAKAQTESYLTAANMWPLSMNTALQLTKGMYSFTMMMLT--

>LhOr196NTE

---------------EWAVKLNRYCLEIAGLWPKSDT-----AWNKVICNLRTLLVFFSIFFLLIPTTHSLIKINGDIMLMIDNLQFTLPVIATLLKIAILWWKKEALISIIDMIANDWRTPKTSWEKMRMITR-AQTARTITKCAYCLMTISFFVLLVLPACNF----SVRYV-TNITDP--GRPLPIQSRYTYDITKSPHYELTFISQGISTFLCMIYYIGADSFFSFLVLHICGQLDILRNRILHL----------------------------------------------------------------------------------------------------------------NLKMCVTDHTRLLRSIVLVEHTFNIMMLALFLYFAILLSFYGFLILDLNDLSIRAAYLISVIINFVCHSCLYCAIGEILLLQCKRIHYAAYSNKWYNLEPRNSRNMILLMVRTNESVYLTVGRLFPLTMSTFSNLIKTSASYISVLLTTK

>AaOR27

------------------MSANVRLCRAVGLWYDL---------THWRFTWQPIFVILSVFWFMIPTVAFMIQREKIFAVQLKPILEIVEIGMIVFRTTAHWYGRRSLTNCFDDLHKAFEQFSVHEDIRRTLRHLQRSASYLVKIYVFVVLFQALSYGPLATFITIVRYCRSDE-TLV-----LTSPVLEADYVFDHLSSSAWLPSSLISVSVQFMMVISITASECLLWNLLHHVSCLFRIVRYEISRLD-------------------------------------------------------------------------------------------------------SDRKTFRKQFVEIISAHNTAYRCARRLESILSPVVGMLYCSCIFQTCYVLFVTS-VVDDPMLMASMIFILQYTTFLIFSFSMLGTELMGESALVSEAIYTTRWYEWSVDKRRLVLFVQMRADRITGITASKFFYLTRPTFGTAMKTAFSFFTIIRSL-

>HsOr42

---------NYRSDAEYVVKVAKFLLTPVGIWPLYGG-----STFSRVRTVQTSFIFSLMCFLLVPHIIYTFFDAENLTRYMKVIAAQVFSLLGIIKFWTMIINKNKIKRCLQQMEIQYRDVECEEDRMVMV-RNAKVGRQFTVMYLGLLYGGALPYIIMPLVAE---RIVK-E-DNTT----QLPLPYLSDYIFVVENSPFYEILFVTQILFSTIILSTNCGVYSLIATCVMHACCLFEITRRQLEAIVM---------------------------------------------------------------------------------------------------------DDLHERFGRIITQHMQALRFAEMIENSLNIIFLSEVVGCTIIICFLEYGVFKEDNQMFGMVIYTILMVSILVNIFTLSSIGDRLKEESEKIGETSYFIDWYTLPAKNVSNLIMVMIRSSRPSALTAGKMFDISLQGFCDVCKTSAAYFNFIR---

>gi|350422508|ref|XP_003493185.1|

---------DSNSNSDYSLQLNRWFLKPIGAWPSSST-----RLEKIVSFILNIICFTSVIVTAIPSLLLMILEDESIYMKLKTLGPVSHWFVSSANYTALLIRGRDIRHCVEHIEADWRTVTREEDQ-HVMMKNAKFGRYVAASCAVFMQGGVLCFCFVTALTT-TEI---QI-GNETRV--LHVLPCAVYKKVNVDESPANEIMLFLQIWAALIANSSTVGIFSLAAVLAAHACGQLDVIMMWITKFVKEAREQKETS--------------------------------------------------------------------------------------------EQKETSSFRKIGVIVERHLRTLNFISCIEDVMNRIYFLEMFRCTMDICVIGYYILSEDRDIQNLFTYFMMLISICFNIFVICYIGEILTEQCQKVGEVVYMTNWYYLPDKIILDLILIIARSSMVVHITAGKLVHMSVYTFGDVVKTGFAYLNLLRQM-

>PbOr49

---------NYQRDIQYIFKPGNWVLGSIGIWPLAIR-----GIGRHASKIAILLCNFALIFAIVPSVLHMIYDQKDLNIRLKLFGLLGFCSTALMKYCVLTMRRPKIMRCIEHVKSDWWQVKFNSDRE-MMLKYATTGRRLSMISVTSMYLAGFIFTILPFCTE------HKI-GNQT----IRPLVYPPYSKFNTQTSPVYEMVYLAHCLCGYTMYSVTAGSCGLAAIFATHACGQIQVIESRLEDLPRGK---------------------------------------------------------------------------------------------------------VNQRIAHIVKSHVRILRFSAAVQEVLQEVCLLEFASSIFTMCLPEYYCI-VDSDAVGLTTYFLLFISFCFNMYILCYIGEQLVQKSSQIGTKCFMTTWYQLPAKSIRGLVLLIAMSNHPIKFSAGRMVDLSLATFGNVLKTSLAYLSFLRTL-

>HsOr41

------------SDFHYATQVSLYLLKPVGIWPLKFE-----NTGQIAHISTIFLATFLQLFMIIPWIIYIFTAQCDLYEILRTACPLIFSITVFLRYMLLLFHRDEIKSCIDHIAEDWRNATLAEDREIMLV-NAKSGRLFGIVSVSFMFGSGLLYCIMPMVAP------PIV-STGNVT--LRPLPNPCELLLDSQASPVYETVYVMEFLSCFTLFTVFCGISSLTAKFVTHVCGQCEILKYFFDEIVDGSSR------------------------------------------------------------------------------------------------SSRNQGTIDQRISTAVMRHLRILKFVTDVDRIMNEICLAEFLNASCNICLLGYYVIMDEESILQISTYFLAFVSITFNIYIFCHIGEMLVEQCQMIGTRCYMIEWYRLPHNKARSLIFSIIMSNYPIELTAGKMLTMTMSSFSNILKTSMAYFNLLREV-

>HsOr46

------SDINHQQDMRYVFTPSSWFLGLIGIWPISFR-----DVGQHISKIAIVICNFVSSFTIVPCALHIIYDQKDLNIRLKVSGFLAFCATAMLKFFVLVIRRPKIRQCIEYVEDDWWQVKFKSDRE-LMLKYAGTSRKLSIISASSMYIAGFIYHLLPFCTE------HKI-GNQT----IKPLPYPTYPTYFIQTQVIYEIVYLAHCMCGYIMYTVTAGSCGLAAIFATHVCGQIDVITSRLEDLLQGKSYEQTTN---------------------------------------------------------------------------------------------EQTTNVNQRIAFIVKSHVRVLRFSAFVEEILQEICFLEFISSLFTICLLEYFCM-VDSDTIGLTTYLLLFVSFCFNIYILCYIGELLMEKSSQIGSMCFMIDWYKLPANSIRSLILVIAISSHPMKISAGRIVDLSLATFGSVLKTSVAYLSFLRTL-

>HsOr47

-------------DKERNIRVIRWLLKSICLWPRSSN-----ASDRVFSECLLVTCFFLLIITMVSCRVLFVKERADVDLLMTHIGPFLCYVMTIMKYICLVLHVDDIRSCVKYIEVDWNTVRSNEDYEVM-LRNAKIGGLMATSIAAFMHCAVQFYSVTRCLMK----NVVEV-DNVSVT--IRELPYPFYNELDVRFSPAYELVLVLHVVSAFVMSGVTSVTCGLMVIFVMHACGQLKILIIWLNDIVQDNDAINIS---------------------------------------------------------------------------------------------------VQRKMGFIVEHHLKVINFVSHIEEVIYLTCFVEVVGGSLTLCLLGYCSIMAQNKRESIATYCIAGCSFSFNIFIICYIAEILSEQYRRVGIATYMLEWYRLPPKTAIGLLLINLRSNFKINLTAGKMVDLSLYTFGNVLKASMTYLNMLRQV-

>HsOr44

------SDINHQQDMRYVFAPSSWFLGLIGIWPISFR-----GVGQHISKIAIVICNFVLSFAIVPCALHIIYDQKDLNIRLKLSGLLGFCTTAMMKFLVLVIRRPKIRQCIEHVKDDWWQVKFKSDRD-LMLKYADTGRKLSIISASSMYIAGFIYHLLPFCTE------HKI-GNQT----IRPLVYPTYSQFQTQVSPTYEIVYLAHCMCGYTMYTVTAGSCGLAAIFATHACGQIDIITSRLEDLPQDKSYE-----------------------------------------------------------------------------------------------SYEQSTDINQRIAIIVKSHVRVLRFAALVEEILQEVCLLEFASSIFTMCLPEYFCI-VDSDTIGLTTYFLLFISFCFNMYVLCYIGELLMEKSSQIGSICFMIDWYKLPAKSIRNLILVIAMSSHPIKISAGRIVDLSLATFGNVLKTSLAYLSVLRTL-

>HsOr45

-------------DKERNIRLIYWLLKSACLWPYSSK-----ASERVLSEFSIVTCFSMLSITMVSALFLFVEERGNTDLVMMHIGPFLCFLMTITKYICLVLHVDDIRSCVDCVELDWNIARSDEDHEVMVR-NAKIGRLTATSLAVFMHSAIQCYGISRCLIK------DVV-EVDDVNVSIRELPFPFYNKLDVRFSPAYEVVLFVHCSSAFFISGITSVNCGLMAIFVMHACGQLKILTMWLGNIVHDDDV-----------------------------------------------------------------------------------------------------NMMQKKLGFIIEHHLRVVNFVYHIEEVIYMTSLVELVGGSLTLCLLGYCSIMAQDKKERIPTYCIVGISFTLNIFIICYIGEILSEQYRQVGKATYMTEWYRLPPKTTLGLLLINIRSNYNINLTAGKMIDLSLYTFGNVLKASMTYLNMLRQM-

>PbOr43

--------VHYEHDIRYAMQLCRRILKPIGIWHMIYS-----QRQKFLSIILILISFSLLCFVLVPSGPYTLFREKDIDMKVKLFGPIGFCLTSAVKYCFLGMRGSAIGRCVEHVERDWRVVQHRDHRK-MMLRNALVGRRLTILSVLFLYTGGMSYHTIMPLSS------RTE-INGSYS--SRPLVYPGYLYVDPQISPAWEIIFCMHCLSAMIQYTATTAACSLAAIFATHACGQVQILMTLLDDLV------------------------------------------------------------------------------------------------------RNKDTTVEKRLSSIAKHHVRVLRFTANVEEVLREICLMELVAATLIICLLEYYCLKENSDAVAIFTYFILLISLTFNILIFCYIGELLVEEFSKVGSAAYELNWYDLPGHKAVDLILIITMSQYPPKLTAGKFCDLSLNTFSTVLKTSVVYLNLLRTV-

>PbOr42

------YNRHYEHDIRYAMQLCQWILKPIGIWHMIYS-----QSEKFLSIILIIISFFLLCFVLVPSGPYILFREKNIDVKVKLIAPIGFCLTSAIKYCFLGLRGLAIGRCVEHIERDWRVVRYWDHRK-MMLRNALVGRRLTILSVLFLYSGGMSYYAIMPFSS----RTKIN-GSYT----SRPLIYPGYDRVDPQVSPAWEIIFCMHCLCAIVQFTVTTAACSVVAIFATHACGQVQILMTLLDDLIV-----------------------------------------------------------------------------------------------------RNKDTTVEKRLSLIAKHHVRVLRFTANVEEVLREICLLELMSSTLIICLLEYYFLKENSDAVAIFTYFILVISLTFNILIFCYIGELIVEEFSKVDLAAYELNWYDLPGYKAVDLVLIIMMSHYPPKLTAGKICDLSLDTFSSVLKTSLAYLNLLRTV-

>PbOr41

----------TMDGWNYSIQLNRWFLKPIGAWPLTLT-----TAEKLGCVILTIISCFLICFLAIPCTLCTILVDTDLDTKVRMIGPVSFILRAAVKQYILISRSKNISECIREIRTDWDRVALNYVKREIMLDKAKFGRWLSSISALFMYSAGIFFTTVPICAK----RTEII-DNET----VRSLSFPIYRGFDPRTTPSFEIAQFMQAVAGYVIYTITVSVCSLAALFAMHACGQFRILMLKMEDFADGKERKSASTMHE---------------------------------------------------------------------------------------------TMHEGRLGDIVKYHIKILSFITRTEKLLNEVCLVDVVGGTLDICFLGFNMMTEEHREAGTITFSSLLISFIFNIFILCYIGELLAEQCTQIGIKSYMIDWYRIPNKGALGLILVMSMSNATIKLTAGKFMDLSLSSFCNIMKASLAYLNLLRTF-

>PbOr40

----------ADKYNEYSIQIMRWILRAISLWPLSVS-----IIEKICSDFTICLCYFLMIVIMVPNGLSIFISQESYETIIRNIGPITFWFIAMVNYSCLLMHIDDIRACVEHVKADWRVMSKTQNRQVML-KTARIGRFITGFCAVFMHSGVFSYNVAQGLSK---DIL-QV-GNSS--IEVRVLPYPFYSKLDTHYSPAYECMFFVQCLSSFVVNSVTVATCSIGAVFVMHACGQLRIMMSLLENLVDERNEEKTSVKQ--------------------------------------------------------------------------------------------EKTSVKQRFVIIVEHHLRVLSFVSRIEKIMNIVCLVELIGCTMHICLVGYYFILDGKDTESVVSYSMLLSSITFNIFIFCYIGETLSEQGGQIGKSVYMTNWYLLPGKTARGLILIILRSNADLKLTAGKIVQLSFSTFGDVIKSALAYLNILRT--

>PbOr47

------KDPNHQQDIRYVFKLNNWILGSIGIWPISIR-----GIGRHVSKVAIVFGNFTLSFAMVPCALHIIYDEKNIIMKLKLCGLMAFCLTAMTKYCILVIRRPKILRCIECIKNDWWQVMFTSDRE-MMLKYATIGRNLTIISASFMYTAGIIYILIPFFSE------TKI-NNQT----VRPFVYPTYSEFQSQISPIYEIVYGAHCICGYIMYSITVGACGLAALFATHACGQIQVIVSRLEELLNDESP-----------------------------------------------------------------------------------------------------PNIHQRIAVIVKDHVRIIRFSTVVEEVLQEVCLVEFASSVCTICLLEYYCIDWQDDNKSLAIYFMLFISFCFNIYILCYIGELLMEKSSQIGSICYMIKWYQLSPRSVRSLILIIVMSSHPIKLSAGRMADLSLSTFGNVLKTSVAYLSFLRTL-

>PbOr46

-----------ERDILNTLIWNRWILRVLGIWPLVYS-----TIEKILASISFAFCWSVLSFLLIPLTIFTFSKHSATYDRVKMLGPLVYVCISMLNYLFLVIRHKNIRQCIRVLSTDWRVVQQEDHRKIMIR-NAAKGHVLSKFCIVFMYCGGLSYHTLMPFLS---HAPTDE-QNGT----VRPLPWKGFSIFDLHFMPVYIFVFCAQCCSGIVMFNITTGVCCLAAMFVAHACGQIEIVMDHIENFIKSVQKSPKQR------------------------------------------------------------------------------------------------KSPKQRMAVIIKHHVQILRFSISIDSILREICLVEIVGSTICICTLEYYCMTENNDSIAILTYLFLLVSFVFNIFMFCYIGEQLTEQCSKIGYSSYEIEWYRLPGKIALDLTLIISISHRPIKITAGKLINLSFSSFGNAIKTSIAYFNLLRTF-

>PbOr44

-------NVHQENDIRYTMQLCRWVLKPIGMWHLIYS-----QSERLISIALIVTCFSALCFVLIPAGLYTLFREKNINIKVKLFGPVGFCLTSTIKYCYLGARAAAFGKCIRHVEDDWRVIRHQDHRE-MMLKNALMGRRLTTLCVLFLYTGGLSYHTIMPLSS----RRKIN-ENYT----IRLHTYPGYIFFDPGASPAYEIVFCIHCLFALITYNVTTAACSLAAIFVTHACGQIQILMTLLDDLVDGK--------------------------------------------------------------------------------------------------------TVKNRLKVIARHHVRLLRFSNNVEEVLREICLMEIVASTLIICLLEYYCLTENSDAVAILTYFILLISFTFNILIFCYIGELLVEQYSKIGLAVYGINWYNLSGNKALGLTLIIAMSHYPPKLTAGKIVDLSINTFGAVLKTSVVYLNLLRTV-

>cplu_016503_b1

----------------------------------------------------------------------------------------------------------------------------------------------------------------------------------------------------------------------------------------------------------------------------------------------------------------------------------------------------------------------------------------------------------------------------------------------------------EGMDLSVSIYKMNWYFLTNECKKDLLMMKKRTLKPIKYTSGTLIELSLDSFTNLVKFSYSVYNLLHQ--

>AaOR23

----------------------------------------------------IFLVCDLTLYIVVN-CWCLTVFWGQLTDVVFCLVTMGIAVQGFAKIAN--YTDDRLYELHVYNVARFDRVRDYPEARESLQTTAVLCKVFIKIFSYLFMMLTTFIPVYTIVYSI---------TSR-----SLQLPFGFFFPWDHTQLFGYIINLSYHFLQIYEASYGLLATDTCFLFFIIHAMGQLDVIIIYLKKLDELAL-------------------------------------------------------------------------------------------------KFKNDEELYQLLNDITEKHQEHVEYMSKMDSLLKPGFFVNFSCMIAETVASLYVQSE--TDGI-WYPGLIVVLLCIVQLFIACALGTIYSTKNDQLIDEIYNISWYAMPIPAQKSLALILNSSQHPVVLSDG-FDAIDLFAFVQIYKKIYTYFTMLQSF-

>gi|167875810|gb|EDS39193.1|

------------------------------------------------------------------------------------------------------------------------------------------------------------------------------------------------------------------------------------------------------------------------------------------------------------------------------------------------------------------------------------------------------------------------------------LVAIQFAFEAYMCCSMLSCIHAENERISKHLYVIDWLGTAKSVRQNGQLLQRQVHGDLKIRAGGMFPVDLQTYSQLMRFVYSLLTFFR---

>CpluG2R504O04EP4Q9

----------------------------------------------------------------------------------------------------------------------------------------------------------------------------------------------------------------------------------------------------------------------------------------------------------------------------------------------------QYTGGVGPNQLTKKQEMLVRSAIKYWVERHKHIVRLVTAIGXAYGVALLFHMLI---------------------------------------------------------------------------------------------------------------------

>HsOr202

---------------RNANSLNNRVNLLSGLLPIEDD-----SKFPLSWRVHNVVIWLIELIHTVALVFGLILVPRE-KALNDGTVCVVVLMEASFMLSRLYARRKLLSRVVDTLDSILREADETMK--DLVRSTLSPITKMFTVYCLVSVTTITIWTVQPVTIAFDKGAFFYV---------DYNLPAA--FTSEPFSRGVLIASSVVMTIGSVFLFLKKFGVDVYMMHIVLILTVQYRYIGRKLTLLFRDLQ-------------------------------------------------------------------------------------------------------RTKRELRALCQHHNTVLRISIMLKKLLSVNFSLLYVNSVFRFCFISILMSTVPSMNSEGISVLLFAFGSAMQFFLLCFSVQTLSDASTEITDTAFNENWYQYGSSIKRIFLLLIMTNNLECKIAAIEKFNLSLPSFMTIMNQSYSIALLFL---

>PbOr184NEW

-------------TLKKVIAVVKLSLLITWCWPLPKN-----TIKVICAGIYQYFCLTVTFSVAVGLMNAVWNHLDDPVIMAKSISVLCPALQVVCNIMYCKINFYRLKLVTFEMENFCELLKSHEET--IVQRYIHKCAYFYGGSMIWIYLSAVFIMSGPLTLD-------------------QSFPTNAEYPFNVDYQPLKSIIFIQQTIACMQGAAQ-LCMNIFIALLLWFTSARFEILIEKFREITN--------------------------------------------------------------------------------------------------------------ELKIYIQEHQNLLKYTEEVIILARPFALSTIYFSTIALIIVGLILIT-DQPLSMKIQCIGIIFSGLSVVFMFTWPAEHLIHISNEIGQAVFDTQWYEQSIGLRKDLQIIMLRAQKPVIISVPCLMPLSLRYYASYLSTIFSYFTTLR---

>AmOr67

------MKTTSNKDFTYAMIPLKFLSWPVGTWPFQ--------VHEIFSISRTIFSISLLLLMVVILQVELYLDRSNAENNLDALLLINCGILAVAKVMCFRIRPIGLVSNFSSAIKDYNELNSEENRV-IMRRHAYMSRVACASLISCSFIASTLFMTVPMLTGDKKDIINVT-EKS-----IIKYPIPSKNALAIINMPLSFMVFIVEYMMLLFTSTGNLGSDSLFFGIVFHLCGQVEILKLKYNKLSNTNERTME----------------------------------------------------------------------------------------------SNTNERTMEHIILLTKRHIYLLNLSKMLNETVSSILVIQLFSSCVLICTTGFQLILTFGNVVLTIKILAEISILLIQLFAYSYVGEYLKTQTEGIGNSVYFCTWYDMPKNVSKDIIFIIMKSQRPVLLTAGKFFVINMETYMSILKTSMSYLSVLRVM-

>LhOr198FN

---------------EWAVKLNRYCLEVAGLWPKSDT-----AWNKVMCNLRTLLVFFSIFFGLVIPTHSLIKVKGDIMLMIDNLQFTLPIIATLLKITILWWKKEALISIIDMIANDWRTPKTSWEKMRMITR-AQTARTITKCAYCLMTINFFVLLVLPACNLSV----RYV-TNITDP--GRLLPVQSRYTYDITKSPHYELTFISQSICLFLCMIHYIGIDSFFSFLVLHICGQLEILRNRILHL--------------------------------------------------------------------------------------------------------------AYNLKMCVMDHTRLLRSIVLIEHTFNIMMLALFLYFAILFSFYGFLILDLNDLSIRAAYLISIIINFVCHSCLYCAIGEILLLQCKRIHYAAYSHKWYNLEPQNSRNMILLMVRTNEPVYLTAGRLFPLTMSTFCNLIKTSVSYISVLLTTK

>DeugOr82a

-------------------ELQENCLRAMGHSDDMDS------TELRSLSFKHISSLLLVTSAQYPLISYAAYNRNEVEKVTACLSVMLTNFLTVIKITTFLVNRQAFWDMIRRFRKMQHQAAKQTPRLDYVTKANKQAAFLGKAYCVSCGLTGLYFMLGPIIKMLT-----S---SSHKTIYVRELPMPMKFPFSDLQSPGYELVFLYTVVVTVIVVAYASAVDGLFISFAINLRAHFRTLQDIIENS-------------------------------------------------------------------------------------------------------SSSEKKVQEGLTSIVRYHQLLILLSGKLRATYTATVFGQFVITSLQVGVIIYQLVKNMDSVMDLLLYASFFGSIMLQLFIYCYGGEIIKAESLQIDIAFRLSNWYLASPKLRRSLSVIIQQSQKEILIRAG-FFVASLANFVGICRTALSFITVIKSI-

>gi|380024720|ref|XP_003696140.1|

------MKTTSNKDFTYAMTPLKFLSWPVGTWPFQ--------VHEIFSISRTIFSISLLLLMIVILQVELYLDRSNAENNLDALLLINCGILAMAKVMCFRIRSIGLVSNFSSAIKDYNELNSEENRV-IMRRHAYMSRVACASLISCSFIASTLFMTVPMLAGDKKELINIT-EES-----VIKYPIPSKNALINMPENLGFMVFIVEYMMLLFTSTGNLGSDSLFFGIVFHLCGQVEILKLKYNKLSDANERTME------------------------------------------------------------------------------------------------------EHIILLTKRHIYLLNLSKMLNETVSFILVIQLFSSCVLICTTGFQLILTFGNVVLTIKILAEISILLIQLFAYSYVGEYLKTQTEGIGNSVYFCIWYNMPKNVLKDIIFIIMKTQRPVLLTAGKFFVVNMQTYMTILKTSMSYLSVLRVM-

>LhOr193NTE

---------------EWAVKLNRISLKLLGLWPKSEN-----LREKLICNLRVLVAFITLTGFIIPCLHSLIRIHSDIILAIDNLQTTLPGITCILRLVIFWWKKQALIPVVNMMVEDWKKSKNQTYERETMIRWALRARIVIICIYSIMGMAYILFVGMPIFGKAIR-LTP----NITDP--GRPLPLQTYYLYDVTKRPQHELTFIFQAISTFIAMLCYTGIDTFLGLLTFHICAQLDILKNRLMHLHE----------------------------------------------------------------------------------------------------------SFHDALKDVVMYHIRLLRMIFAVEDAYNIILLVLLSYFAILFAFYGFLLISLNGIPIRLLYLVIIVITVLLHMCLYCAVGEMLMTQCDGIYYAICNYKWYSLDPKKARHMIIFMIKASEPVYLTAGKVFPMTLSLFCNVIKTSAGYMSFLLTTR

>373_Si_gnF.scaffold00330_12820-264637.pep

-------------------SPLKIGLQLLGLWPGI-----------SYSIIYWSSFMLSMIVMQYFQYLYIFNHIKELLNLVDSMPAALDYSLTIFKLISLWLHRRVLHEILTAMDNDWRECINVDWQLYVMQVKANISHICCNAILSFNAIATLLYFLGNYIIHIM--FLTED-YNDT----LRQLPFKTQLPYETQQSPLFEFVFAILLLHVILHSSTVGIVNGLIFTLVLHVGGQIDIICQKFKNTSENTLLSKTSVP-------------------------------------------------------------------------------------------------------MLIERHNRIISASDNIEKLFSFIALMQVLWNTLVICSLGFAFTFFNGANFALVKTIFAYFGVIMEAFVICFAGEYLSHKGTSITNATYETLWYDMPPNQCKIIMFIMMRSQKQLAISAGKMLDMSFETFTSVI--------------

>DanaOr74a_scaffold_13337_residues_8277754_to_8280132_reverse_strand.[501_1879].sp

------------------------------SWPLE-E-----GSSRWAVWLDKFLIFLGFLVFCE-HFHYLIANWQDMDNMLAGMPTYLILVEMQIRSFQLAMHKDQFRVLLQRFYREIYVSEKEEPE---LFSRIQSQMLPTRINSVVYLLALLNFLVVPIQNVVF----H-----------RRDMLYKQVYPFDNTWLPVFIPLLVLNFWVGVIITSMLFGELNVLGELMMHLNARFVQLGQDLRRSAS----------------------------------------------------------------------------------------------------------KYRRALTHILRRNAALRDFGEQVEEEFSFRLFIMFLFSAALLCALFFKAYT---SPINNVAYIVWFLAKFMELLALGMLGSILLQTTDTMGMMYYTADWEQVNRRLMRLLTLAIQLNSKPFFVTGLKYFRITLTTVLKIIQGAFSYFTFLNSMR

>gi|238623751|dbj|BAH66349.1|

---------FKDYSLNGSLWIVNLLPRLMGFNLRA---------DKVGVFFWTIYILLLVYVFGIGIFVYLWKHVDTMSGLMKSYLNLSLILVIVNNSCWFLSKRSLLNKVLKKIHLIEDLSCESEH---ALAKYRRVFKIVTHLLLASYVLFYF----TEIYFMFLFRNYDLL--------EDYSLPCVGLE--PLSSSPNSEICLIIVLIHEFISTTVMMSFAALFLVLIAHTAVMFLVLAEDMTKLTDLIN----------------------------------------------------------------------------------------------------HRKMIRESLRSLIHRHSLLLQIVYELRLLYSVPLGINFISNAMSILVLLCLPI-HEWP-----SFLHIIGYCFFAFFLYCFLGQNVINASEKFIDAIYCCGWEHFGVAEKKLVHVMLRQAQKPVEIIALGMISVNMNTYVEALQLIYKFVTVL----

>DmojOr82a_scaffold_6540_residues_34000261_to_34003508_forward_strand.[501_2854].sp

----------------RLFQLQENCLRLMGHNMESS------GERSPPLGLRHIVSLVFVLSAEYPMLSYIVYNRDDMELITACLSVAFTNLVTVTKIWTFLAYKQSFIQMMQSFRQLDSKSQHGTVGYGYVEHGNKLATLLGRAYSLSCGFTGLYFMLGPIIKI----VTNNW-RGV--PY-ERELPMPMKFPFNDVESPGYEFGFIYILFVTIFVVLYASAVDGLFISFAINLRAHFQALQQDITL----------------------------------------------------------------------------------------------------------EEQLVDKQMADVVDYHVQLLSLSRQLRHIYTPIVFGQFFITSLEVGVIIYQIVTHFDSIMALLVYFSFFCSIMLQLFIYCYGGEVIKVEGLRVGVAIQTSNWHSASHTQRRFLIFIMHRSQREVLIKAG-FYEASLANFLGILRAAMSFITLIQSI-

>ApOr19C

-----------------------MLFKAIGLYQLLCR-----GGYSVRSRRALMTALGLSFALHSFQVPYLYYALNDLQRFAYMAAVIIYGMMCSFKGYVLVTNADRLWLVLNAADYGYTGCGHRDPS--RLRRCRATLSALLRTFVALSYGTLIVWIVLPFFVDEYTGITNSD---GTVTR-YRTTIHNMQYPIVYNSRPVWALIYVTELYVCIVNVFIWSLFDCYLVTMCFVLNAQFHTM----------------------------------------------------------------------------------------------------------------IESNHYSDLISHIQDNQNLIKMFDVFFEVVRPVVLVQIANGSYSVISLIFLTALMPVLSAAFLKFICGLISLTIELFIFCYGFNHIE----------------------------------------------------------------------

>gi|383861630|ref|XP_003706288.1|

--------KVAKDSIDYYVLPNKILCSTIGIWPPDEE-----RSFGLFVGFRVVFSMAAVCSIFVPEIMMIAVNWGDLRILTGVGCVLTTVAQLIFKMIYLTARKEKSYKLYKELRSLWDSSHDPKERRCYQ-DLAYIARNCTIIFYTTGMLTVAIFIVSAVSDYI-K-L--GQ-DNNTA---NRHLPFEVWYGTDVTDSPAFEIAFACQVVASAICCVGITGLDTTCAISIIHICGQFRLMCMWVSNIG-----------------------------------------------------------------------------------------------------------------------------------------------------------------------MFIAYAMSMMIQLILWCWPGEILIQASLEVGYAVYNVPWYKMAPACRRMLLLMILRSQNVCSLSALTFKSVSIRTLTTIFNTAASYFTLLRQM-

>DgriOr42b-4__scaffold_15245_residues_8725239_to_8729574_reverse_strand.[1501_2836].sp

------------------FIYLYRAMKFIGWIPPK---------AVLLRYVYFSWTLMAFGWHTVYLFLGSYITQITPGEFLTSLQVCISAYGCSTKVIIIYSQLWRLIKARELLDKLDVHCSSLEEREKIHHI-VARCNHVFLIFTIIYSSSGVSSYLSSVLS--------------------GHPPYQLYNPFDWHDGLNLWIVSTFEFMVLVGAAVQNLLSDTYTLVYGLILRTHLELLNGRISKLRTNP---------------------------------------------------------------------------------------------------EMTEDENYEELVNCVLDHKLILEYCALIRPVISGTIFIQFLLIGAILGIALINLFFFCDLWTG-FASVVYIVATLLQTFPFCYICNLVVDDCEALAHAIFQSNWIGSSPRYQSTLFYFLHNVQLPIVFIAGGIFPISMSSNISVTKFAFSVITIVQQ--

>gi|340726436|ref|XP_003401564.1|

----------------ISIIWASFLMKIVGLWLATN------RNEQRRRDFALIYTVGALFISICIAIRDIYHTWGNFSDSVFICCNILYVTIVFLKIGVIYKHKIEFFNLITFTQKNFWRPYHDPQEILVVADCKRICNIFIILMIFCTQGTCAGYMVTPLIANIGR--------NES----DRILPFNLWVDFPVGMSPYFEILFTIQILCVYHVGVCYICFDNLLCIVNLHVACQFRILQHRLRSIDNATKDQIEE------------------------------------------------------------------------------------------AKLSCYSNMCYTKLKNCVQQHQMLIEYCKKLENIFTLIVLAQVMFLAMVICLVGFQLLLVDTPTSKKASLVLNLCGVLCQLLMFTYSCDDLMRQSVNVGNASFSGPWPILPMTVRKNLLIIIMRSHKICCITAGKFFPVSLQTFTGVLSTAMSYFTLLRNT-

>AaOR38P

-----------------PFQLQKRLFRVLGYYPGD---------ERLVHWGM----LLVLFFHYWSQVMLIYIGEGDLQPALEGICPTPSRFGGILKCCILIWKRKQLKQLLDTLKGWFDRE-EPREKK-INQWATYWGYQFTYWELMFTHLTCVFYCLLPVAAMLFHFVKQPD-E-------PRIYILPFKLPFDYCKSPVFEITYIIMCYIAYPPIFMMAGGDGLFIGVCLLISSQYRIVQRELEALGQS---------------------------------------------------------------------------------------------------TAEENDHIFEQLK-LIARHNRTIDTTEEMSRLFLQNVFASFTIAAIKIGIACITVMKAEGLNK--LIFVWYSLGILTEIYLYSYGGTQLMEESEKLSRTAYDFPWYRYRKNVRQIIQMMMLRAQKPSRVDV-PFFEASVVTFSTILRTAGSYVALMQT--

>PbOr223JOI

------------------LQFTLKLCAVSGCWQPLS------WTSLSKYIIYSCYRILLITAFTISQMLHTGLNLDKHNEISDNLYMTLTVFIATYKIIIMWKTKKHVVRIINALTEEPFRPLESNEE-MIRQKYDKMIKKYAFLYYGLVQVTVICIILNAIFMDFM----------------KGNLTYKAWIPFDYTSPIIFGFVFTHQMIGMSITAAVNVACDSLVSGLLQEICCQLEILEYRLTKISHDQH------------------------------------------------------------------------------------------------------------LRNCIHHHNRIYEYAYIINRRFAKIIALQFAVSMLVVCANLYKLASIPSLMIGIITLILYTACMLSQIFLYCWFGNELKLKSTGVINSIYHMNWQNLDKASRKDLLLIMRRSMIPIEFTSAVIITLNLESFVSLLKASYSAYNLLKRF-

>AaOR68P

----------------------------------------------------GSYIMTQMVIYFWCNFWTVYKYRHDIIHVMEVLNCTGIAFQLSAKFFIAMNNKSKFRRLLQTIEDNLYTRYPDRSTEMVFMF-ARKYHILLRILTVLYCSTLFVFAILSLYYS----------EGE-----LIPLFMFEVSYVDWHTVWGYLLTNFVQN--------------------------------------------------------------------------------------------------------------------------------------ILQSENASQNKEKIIELWRECLVEYQTIIEYLIDIESFNGGMSLVLVFTGVFVMCDNLVLCAL-----TDWYASYLFLIICFIQLTIYFAMGNAVELKSDALDVCVVNFPWRLLKINYQKEYLFLICRMQN-----------------------------------

>DbipOr83c

---------------------------LLGVDILD---------PVLKFNYRTWTTIFAIINFTVFTIYSIVDNGGDWLVSLKAGLMIGGLTHGTAKFLTCIVRQKEMRSLTLFTQDIYDGYEKRNPSSSTLDANIDRLLRFMKGIGYGYMVTNFLMVFTPLAMFAY---------ND-----SRMTVLMYEIPLPIQKNFGYFLTFLIHLVTICVRGFGFYAGDLFVMLGLTQILTFSDILKLKIKELNSVLKLKEEKR---------------------------------------------------------------------------------------------------QRLLIEMIKWHQLFXXYCRRVNNLYNPLITTQVLAMAYEILAT-FCINL----NGLHVPSAINFLLAAYCMSVYCVMGTQIEFSYDEVYENICNVSWYELTGEQRKIFGMTLRESQSPHNIKLLGVWSLSVRTALQIIKLIYSASMLM----

>AmOr3

---------HYRSDAEYTVRVAKILLTMVGIWPRRNT-----FSNNVKFYVQTTIVFFLMCFLLLPHVIYTYFDCENLTKYMKVIAAQVFSLLAIIKIWTILINRNEIRFCLMEMEVQYRDVECEEDR-LVMMNTAKIGRIFTIVYLFLGYGGALPYVILPLISE---RIVKA--DNST----QIPLPYLSDYVFVIEDSPTYEITFVVQMFTSFLIMSLNYGIYSLIASITMHCCGLFEVTNRRIETILKN-------------------------------------------------------------------------------------------------------NRDLRGRIADIIQSHLKAIEYSALVGKSLSIVFLSEMLGCTIIICFLEFGVIVEDHKTFSMVTYFVLVTSMFVNVFILSFIGDRLKQESERIGQTSYFLPWYEFPTEIAKNIRIIILRASRPSSLSGAKMLDLSLRVFCDVFKTSAAYLNFLRTM-

>AmOr6

-----------EKDLKQAF-YAQPFLKIIGAWPILIS-----LSSKIQKWFIISFSISLQMCIVVPCILVMFLKEKNGRRKINLFMLLTNILNQVFKYVITLNRANELRIAIHEIKKDWLTATPEDRF-IFVT-NSRIGQRIMLIIAVITYSSGLGYMVLPLLKG--KIVLA---NNVT----IRLLPCPTYFTFNELVSPYYEMIFMLQILAGVFVYTVLSGTIGISLMLSLHMCSLLKILRRKMIDLADGSITS--------------------------------------------------------------------------------------------------SENTMQKRIVDIVEYQTKIKRFLGNTELITQYFCFYEISCNTCLICFIGYCIILENSNVVAIVVHFMLLGTCILVTYIVCYIGQLLIDESNNLARTCITLNWYHFPTRKARCLILIIIMSNYPVKLTAAKVVDVSLTTFTDVMKAAMGYLNMLRE--

>AmOr7

-----------EKDLKQAF-YAQPFLKIIGAWPIVIS-----LSSKIRKWFIISFSISLQMCIVVPCILVMFLKEKNGRRKINLFMLLTNILNQVFKYVITLNRANELRIAIHEIKKDWLTATPEDRF-IFVT-NSRIGQRIMLIIAVIMYSSGLGYMVLPLLKG--KIVLPN---NVT----IRLLPCPTYFTFNELVSPYYEIIFMLQILAGFFIYTVLSGTIGISLMLSLHMCSLLKILRRKMIDLADGSITS--------------------------------------------------------------------------------------------------SENTMQKRIVDIVEYQTKIKRFLGNTELITQYFCFYEISCNTCLICFIGYCIILENRNVIAIVVHFMLLGTCIFVTYIVCYIGQLLLDESNNLARTCITLNWYHFPTRKARCLILIIIMSNYPVKLTAAKVVDVSLTTFTDVMKAAMGYLNMLREV-

>AmOr4

--------KHTEKDLKQAF-YVQTFLKIIGAWPIAIE-----SSSKIQKWFIISFYLFLQICIVAPCILDVFLKEKNGSRRINLFMLLISTLNQVFKYVITLNRANELRIAIHEIKKDWLTATPEDR-FIFVMN-SRIGQRIMLIMAFIMYISGLGYMVLPLLKG--KIVLPN---NVT----IRLLPCPTYFTFNELVSPYYEMIFMLQLLARFFIYTVLNSTVGISLMLSLHMCSLLKILTRKMADLTDG-------------------------------------------------------------------------------------------------------EKIMQQRIVDIIEYQTRIKRFLSNTELITQYFCFYDIGCSTCLICFIGYSIIVENHNIASTVIYFSGLVTCTLMIYIICYIGQLLLDESNNLAQTCITLNWYRFPKKKARYLILMIIMSNYPIKLTAAKVVDVSLTTFTDVMKAAVGYLNMLRE--

>AmOr5

---------HTEKDLKQA-FYAQSFLKIVGVWPIPIP-----LSSKIRNWFITFFSLFLQICIVGPCILVMFLKEKNGKRKINLFKLLTNTLNQLFKYIITLNRANELAIAMNEIKNDWLTATS-EDR-WIFTANSKMGQKVMLIVAVTVYSSGLGYMLLPILKG--KIVLPN---NVT----IRLLPCPTYFTFNELVSPYYEMIFMLQLLAGFFSYTVLNGTVGISLMLSLHMCSLLKILTRKMANLTDRSIT---------------------------------------------------------------------------------------------------SENIIQEKIVEIVEYQTKIKRFLGNAELITEYFCFYDIGCNMCLMCFIGYSAILENHNIAAIVVHFMLLGTCIFIIYIVCYIGQLLLDESNNLAQQCITLSWYHFPTRKARCLILMIIMSNYPVKLTAAKVVDVSLTTFTDVMKAAMGYLNMLRE--

>DsimOr83c_chr3R_residues_1929197_to_1931667_reverse_strand.[501_1971].sp

-------------------------TNLLGVDFLA---------PKLEFNYRTWTTIFAIVNYTGFTVFTILNNGGDWKVGLKASLMTGGLFHGLGKFLTCLLKHQDMRRLVLYSQSIYEEYENRGDSHRTLNSNIDRLLGIMKIIRNGYVFAFCLMELLPLAMLMY---------DG-----TRVTAMQYLIPLPLENNYCYVVTYLIQTVTMLVQGVGFYSGDLFVFLGLTQILTFADMLQVKVKELNDALEQKAENR---------------------------------------------------------------------------------------------------QRLLLDVIKWHQLFTDYCRAINALYYELIATQVLSMALAMMLS-FCINL----SSFHMPSAIFFVVSAYSMSIYCILGTILEFGYDQVYETICNVTWYELSGDQRKLFGFLLRESQYPHNIQILGVMSLSVRTALQIVKLIYSVSMMMMN--

>AmOr8

---------NAREGINHTFWFAYPLSRMLGYWPLNVP-----SSSKILNSFTIFFSYLLPLIVLIPGLLYVFLKERNGRRKVKMLMPHINSIAQMTKYTIILRRTKELGKLLDEIKKDWSTATQENRR--IFSERASIEHKLTMIVAITIYGGGFLYRAILPLSK-GRIVLPN---NVT----IRLLPCPGYFGLDEQVTPNYEIIFTLQVLGGFVTHTAVCGIKSACLMVCMHMCGLLRILTNKLTDLTNDND-------------------------------------------------------------------------------------------------NDNDERVVQEKIVHIVEYQTRIKEFLNHVDQFVPYVYLIEIFVGVLITCILGYCIIVEDSDAMAIIAYVALQTTCVFGTFSICYVGQLLVDESESVRQACKTLKWYRLPTKKARSLILLIIMSNYPIKVTAGRLVDVSLVTFTSIIKSAVGYMNILQQV-

>AmOr9

----------AREGINHTLWFAYPLSKMVGCWPLNIS-----TFSKIFNAFIIFISYLLSLIVLVPGLLYLFLKEKNGRRKIKMLMPLMSTIAQMTKYTILLRRMKEFNKLLDEIKKDWSTATQENR--QIFSAKASIEHKLTTVIAITIYGGGIFYRMILPLSK-GRIVLPN---NVT----IRLLPCPGYFGLNVQITPNYEIIFTLQILGGFVIYTALCGVKSSCLMLCMHMCGLLRILTNKVMELTSDK-------------------------------------------------------------------------------------------NKVMELTSDKDEKVVQEKIVYIVQYQTRIKEFYNYVDQFVPYVYFIEMIVGVLITCVLGYCIIVEDSDAMAIIAYVVLQVTCVFGTFSICYAGQLLVDESENVRQACNTLKWYRLPTKKARSLILLIIMSNYPLKVTAGRIVDVSLVTFTSIIKSAVGYMNILQQI-

>TcOr100

----------------KICGITRKVLRYSLLWPVEND-----ELSPGIRYKLTILAFFSITGILVFSSVYSVLEIKQYDIDVEDVAILIAVYGTYYMVSAYLNNQHQIALLERDLSQFYKFGKPPGFE--QLNSQLNFAVKVLIIYSFL---GTFVYNGTKMLLREECKKNSQE-KGLSDN--HCGLIATFMFPFRVDYFPVFYIVLVITFLLAHTLIKLCMHISFNAYEIVNHIVLRIEHLKEMILSCFN-------------------------------------------------------------------TLIKLCMHISFNAYEIVNHIVLRIEHLKEMILSNERNQTIVQKKLRVCILYHIEILDMAARLDKNFFNTMFGHFALTGAICACLEKQIVL----GVNIVAGTLHFIGWIIALFVGCVAGQCLLNASEIIPNALWAAKWYHADLRTQKTLLFMLARSQKELTIKAGPFGILCFPLFVSVLKTSYSILCMLTS--

>gi|383848900|ref|XP_003700085.1|

---------------EKAIAFTRISVAPAVFLPLS-S-----RATKLLTTGYKVFKILVLLNSFLFLLINALYTYNDPANVSESICFLLAEVQFIVNTIISITHYDRFQCLSQPVIQTYCKNAKVRERA-ILQWYVNQFSTFYGVSATWFYTTAIVVILGTFFSS-------------------HPFPTNAVYPIAVDYQPLMSIVFLHQSVIGLQFSA-SVCVSVLCALLLLFASARFEILKMELREVK---------------------------------------------------------------------------------------------------------------ELIKCMEKYYTVRRYACDVVNTIKLLPLCTVILCGVILVFCGIKLIQ-PQPFTSRCQYLSIVWTALVEVFVCAWPADHLLDISQNVMEGIYESTWFDQDTSMQRDVRIMLL-PQPPVAIKIDCIIPLSLNYFCSFVSNVFSLFSVLR---

>DpseOr1a__ChXL_group1e_residues_4173766_to_4176048_forward_strand.[501_1783].sp

---------------------QRFTFARMGIQPMSAT-----GDGALRSPVLYGIMVLATSFELCTVCAFMVHHRHQIVLCSEALMHGLQMISSLLKMTIFLVKSGDLISLIHQLQEPVPSR--------V----VHRGQQLAAIYFLMCAGTSVSFLLMPLALTMVRFY-------QTDRFE-PVSSFRVLLPYDVTQPHIYALDCCLMGFVLTFFCCSTTGVDTLYGWFAVALSSHYRRLTTQLQ----------------------------------------------------------------------------------------------------------------------LFAQHARLLALVDRFNAAFREIAFVEVLVICVLYCSVICQYIM-PHTDQNIAFLGFFSMVVTTQLCIYLFGAEQVRLEAEGFSRQLYQVPWQGLSPNHRRLLLLPLQRAQGDMVLGAY-FFDLGRPLLVWIFRTAGSFTTLLNA--

>367_Si_gnF.scaffold04648_643308-783672.pep

-------------------ALTFKIMAICGCARLDST-----SYKRLVYHVYTIFVMLLIHTFMLSQLVDLIMIVDNSDDFTDNFYVLLAMIVSCCKMFALLVNRSNIKMLIEILTSKPFKPVEPDELK-IRQKFEKLIQSNTLHYTILVETTCLSVAVTSLLTE-YR---------------KGNLTFRGWLPFDYTSPQLFPLVYAHQLISFTMGSVHHVACDSLICGFLVHICCQIEILEYRLRKSA----------------------------------------------------------------------------------------------------------------LRECVLHHNHIFKFASIVNEKFRLTIFIQFVVSTLVMCFNLYQFTKSTALKTKYMQLILYTCSMLSQIFFYCWYGNEVKLRSRQLINNVFEMEWFKFNENGQKALLMIVRRAAVPIEFTSASVISMNLDSFVGLLKTSYSAYNILKQ--

>DpseOr19a__ChXL_group1e_residues_7187062_to_7189358_forward_strand.[501_1797].sp

--------------SMSAFRYHWQIWRVMGMHPADP--------QTLWGRHYTLYGIVWNAFFRLGMALSLVVNSTSLESFCESLSVAVPHTVANLKVFFLWRMRQQILQTHPILHHLDGRIGSLAEKQSILEG-IDRAYFTFISFLRAIIFILAVGILILCLSS------------------DRPLLYPSWMPWNYKDSTVYAMTVCLHSVGIIENALLVCNVDTYPGSYLNMLAAHTQALAHRVSRLGYD---------------------------------------------------------------------------------------------------------QACDRLRSCILDHQIIMNLFKSLEHSLSMSCFLQFASTAIAQCATCFFVIFVSVGTMQSVNMIFLFLVFTTQTLLLCSSAELVRHEGENLIKAIYDCNWLDQSVEFRRMLLLMLARSQRPMILRAGLIIPVQMSTFMVVCKGAYTMLTLLRE--

>gi|380024712|ref|XP_003696136.1|

------MKTTSNKDFAYAMTPLKFLSWPLGTWPLQ--------VFDTFSIIRAMFTTFLVLLMLAILQVELYLDRSNAENNLDALVLINGGILAVAKVMCFHVRPVGLISNFTSAVKDYNELNSEENRV-IMRRHAYMGRVACASLIFCSYVGSTLFMTVPMLAGDEEEVINVT-EES-----AIKYPMPSENTLINIPENMYFVIFIVEYLMLLLTSTGNLGSDSLFFGIAFHLCGQVEILRLEYNKLSNENERTTK---------------------------------------------------------------------------------------------LSNENERTTKHITLLTKRHIYLLKLSDMLNETISSILVVQLFSSCVLICTTGFEFILAIGNIVMTIKTFIVMCVLLIQLYLYAYAGDALESRTEEIAQAAFHSFWYQSRGRTARDLILIICRGNSSYHVTAGKFVFMNIFTFKEILKSSTSYLSVLRVM-

>NvOr4PSE

---------------------SRYFLRSIGLWSSSSS-----RSQAYLHKFLIVITYLLIMFILVPCALHTFIEEPNMAIKMKLIGPMSFALMAVTKYASLTQRTAEIAQCFRHVEEDWENGCAMEKKRVVAVIYAKIGRSLSIFSGIFMYGGGIFYHGIMPMD----NSLSLN-TSS----VPRILTFPTYNAWNIEISPIYEIVYLLQCFSGFVLDTVTVGTCSLAAVFVTHTCAQLELVMMLIRRYI-------------------------------------------------------------------------------------------------------------EARLAVIVTRHIRALKFAARVEKYLNGICFVEFIGCTMNICFIGYYCLT-EWERKEPISTTTYF------ILLAPFTTNILXEQCTRVGSIYHTIDWYNLPGKSASDLFFIIAVSRYPAKLTAGKFVDLRLVTFSNVMKSAFTYFNLIR---

>HsOr160

-------------------------LRLIGMWPYCV---------HADVNWWTYIASVAVVQYFQYSYVFAHFDMGNFSDTIDGLSITFGYSLAFFKLINLWFNRRKLYVILAAMEQDWSDEIAIDPNIATMTHHADLSRQCSNVMITTNALAVFFYTIGGPILRS---TINK-----GDHAATRELPLKMEFPFDVYKSPVFEVVRVAQLLHDLSVACIIAILNSLIVTLVLHVSGQIDIIRRGLLEISRNKHASK------------------------------------------------------------------------------------------------HASKSSLAAIKLLVGRHQRIIDLSDNIEDLFSSIALLQFVWNTLVICCIGFVIVISEEGATVITKSLIFYVAITLEAFVFCYAGEHLSAKSKSIGEAAYESLWYNLTPNECRILLFLILRSQKRLTITAGKVTDLSLESFTTIMKSSASYISVLRA--

>HsOr161

------------------------GLRYVGMWPDA-------PCALFCRCVWILTTTIVQTCQYWY--LILHFRTEDLLNLTDSLSVALEYTVMFSKLIILWLNSRIFNDVLASMAIDWREAALNDVQ--IMTGKASLSRYFSNLIIGLHSAAAFSYGLGVLVQS----SRSDE-TNAN-GVPIREFTLKLQLPFECNQSPRYEVVQCLEFLHQLSASAVTGMLNSLVVTFVLHTCGQIDILCDALKNLSPG----------------------------------------------------------------------------------------------------------------ELVVRHQKIIDFSDKIERIFCYIALMQFMSSTLVICCLGYMVVTSSVDSPALMKAIIFCMAATVEAFIFCFCGEYLSAKSKIIGDAAYKSLWYDLKPEQNRFILLIMLRSQKRLTITVGKMSDLSLEGFTTIIKASVSYISVLHAM-

>HsOr162

-------------------KPVKIGLHLTGVWPNT---------SVLFKLLWTLVMGTGLIFQY--QYLLNHFSTNDLPNLIDGLSTTLPYNLLFFKLVVLWINNRVFVDILRAMSKDWRKYSNMYA---MI-DKAVLSHRCSKLIIGIYSTAVLLYSTASIDLR-------NN--GGS----CREMLIKMELPFASCESPIYEIVMFVQFIHLLAVAWSIGVIDALIVTLMLHIGGQIDIIHQQLDEICPNDEHYD----------------------------------------------------------------------------------------------------------RSLVSKHHKIIAFSESIESLFSQIALMQFLSNTMIMCCIGFLVVTTT--DDMLIKTSFFYIAMTMESFIFCFAGEYLSNKSKTVGDAAYESLWYILKPRDGRILLLMIMRSQRRLTITAGKFMDLSLQGFTNSLKASASYISVLYAM-

>HsOr163

-----------------------FGLRAIGVWPGFP--------YTFLCRMLWTITLGVQTFQFR--YIIACAKANDFLNLVDSVSTTLPYSLLCLKLIILWLNQRLVNKILTLMSQDWRDGGAVAFNVRTMTDKARLSRRCSMLIIGVYSIAVVVYVSVIIEFN--HIHSDEF-SNK-----ERQFFLKMKFPFDYDVSPIHEIILFIQFLQLLSNASVIGMLDAFIITLMLHISGQVDIVCYNLCKLFSEKYEH-----------------------------------------------------------------------------------------------EKYEHKSYGEAIGMIIRKHQNLIALSNNIENLFTYIALMQFFTNTFVICCIAVVIVTSLESKILLLKSLFFYIAITLEAFIFCFSGEYLSNKSKSIANAAYEVLWYNAQPSKSRILLTLMLRSQKRLTLTIGKFNDLSLEVFANILKASASYVSVLLAM-

>LhOr213

----------------RSVSITRLFMKMVGLWDVETP-----R-ERLLLRAAFGYAVWQIVFAILVEGVDLYHCLGDFYAVTYNLCTTLLLIMILVKLGSFFFYRDTVMELIRFAEKNFWNVDYDETSARILEEYDKLGMSIIYTFTFIVYAATFNYIFAPLFEH-------DK-TNET----EKVLPFKLWIEFPYH-SPYYEVTYVIQSLSTFHSGICTFCFDNFVSTFNIHVAAQLKILAYKVEVIVEQCIDKVTDQK-------------------------------------------------------------------------------QCIDKVTDQKHLSETAALTFKKLQDCVQQHLKLIYYVRNLQRVFAIILLGQLLLSSVVICFGGFQFL-AADVFIRKCIFAFHFVGGVIQLLLYTWTCNDIIVQSTAICDAAYNSKWYLLPSALRKGLIMIMVRARRPCALTAGQFGVISLETFMGILSTAMSYFTLLRQM-

>LhOr212

-----------------SIRIARFLMKMIGFWPAESK-----A-EERLLNGLLIYTIVAVAGALWTETEFFYFSMGDFYAITYTACSTMPVVIILLKISFFLIHRKHMLNMLRYTQDHFWYAQYDEYGSKLLEEIDKKGKILMITFTFFVQGTVFTYMLTPIIEN-------RG-KNES----ERILPFNVWLGIPTNVTPIFEILFVFQILVLVHSGLCFCCFDNLLSLLNLHIAGQFKLLQHRLETILERINRVDT---------------------------------------------------------------------------------ERINRVDTVKSFDERKKREMYEEIKRCIVLHQELIWYCEQIQHIFMYSTLCQLLVSGIMLCVAGFQVFLAQGTFVRRLIFIAHTNGCFFQLFIITLTANDLMLESCAVGNAAYNADWQVLNRRVRNAILFIIMRCTRACSISAGGFFPVSLETFMAVLSTAASYFTLLRKF-

>HsOr166

-----------------------FTLRMIGIWPDS-----------LCKNLPPVLWTIVMLAWQIFQYWYLFTHIGTLIDLAYCLSQYLSNSLLFLKLSILWWNRRIIFDIFATMIEDWNVCTSVSSKTQMINK-AILCHRFSKCTIGAYTMNLLLFGITNIFVQ-KSVSFDQV-DE------ERQLLIKMKLPFMYNTSPIYEIVMITQFLLQYTLALMAGMLNVFIVTLILHIAGQIEIMCQGLEMLVSEDK----------------------------------------------------------------------------------------------------KNESHIATLRDLVIKHQRIITFADNIENVFCYAALLQFLSNTLVICFLGFLIVTQLDSNEVLMKTIPYYLIVNIEAFILCCTGEYLKSKSRFITRSAYTSLWYELKPAQSRILMLLILKSQRQLTVTAGKFVDLSLETFTNILKASASYVSVLHTM-

>LhOr210

-------ASSYLERFRKLSALHVTYLKYVGLGALGAS-----ALSKCLYFVYNKFILTTMLIFTITLLADICLSFDDLSIVTDDGCIFAGIIVVLFKVMIFQTRREQIIRLLRETIEGCDLLCKGGED-KILDKYLTLSRVTFYGFSTMAFFLVIALLFLVPVEN-------------------GELPVRARYPFDTTKYPWHGIGFFVEACTISVGLTGIIGMDSLHTNLCNLFLVQLEILNAHYKNCSNNDQ--------------------------------------------------------------------------------------------------------FTRQFRRSVRNHQRLLAIIDDFNEVFSAGMFVQMLSSTTMICLTGFQAALVRGQSSNTYKFSIYLAAAVSQLFYICWVGNEVMYQSMSLTQSQWLSKWSDLTVKTGRLLILSMIFSKRTLNLKAGVFYVLSMETFTAILKGSYSFFALLTTM-

>HsOr168

-----------------------------GIWPGL--------TYGLICKASWLIVMVVTSLQYT--FLFIHARTINFTIFMYALAASLAMSMKFIKIAIFWYNQRRFNEMWKTMSTNWELGCTSAID---SGGKMHVLRHLPNFIVAFNSVSVIMTSANTLSNA-----IHYD-EASD-T--ARSYILMMHLSFEINKQSVYLVVIFLQFFYLLIVSAGAATINSVLVTLMLYLSGQIDVICRCLTHMPQGE--------------------------------------------------------------------------------------------------------------KEIIRKHESVITFSEHIESLYTYIALVLLLLNTLITCGLGFILVTSVGSPIMIMKNIMFYCVINIESFVFCFAGEYISSKSRKIGEAAYNSPWYQ-SKFHGRTILFMIMRSQHQLTITMGKFMNLSLELFSTITKASASYMSVLLAM-

>HsOr169

--------------------TTKVILTLCGMWPGA--------CCVMVCRAYWIIALA---TDEFCHYRYFLLHWRDLFDLTDCFSSCIAQVKVITKFLVFWFNQRKFVNILTMMAEDWRDSADSDVDMRETTCKAKLSSRITNAMVTLHAITIVVYSSGVILAD-----VDIN-DREN----MPPLLLKVEVPIDIHSQYRYKVLLAAQFLYLFIAGCGAGLVNALLLTLILHVGGQMNILRCWLTKLVPKENERKR---------------------------------------------------------------------------------------------NERKRESIVIMTNKIIQKHQKIIIFSEYIEDLYTYIALVQFVLNTLLICTLGFIIVTSPDTKEQLRKSLLFYTVTTLEAFIFCFAGEYLKNKSKAVGTAAYDSSWYELKPENSRTLILVILRAQKQLTLTVGKIMDLSLESFTSIMKASGSYLSVLLAM-

>DficOr83a

--------------------FVRQTMCIAAMYPFGYY-----VQGSLIYELFNYFVSVHIAGLFIC-TIYLNYGQGDLDFFVNCLIQTIIYLWTIMKLYFRRFMPGLLNAILSDINDKYEPRSAVGFNFVTMAESYRMSRLWIKTYVYCCYIGTIFWLALPIAYR------------------DKSLPLACWYPFDYTQPIVYEVVFFLQAMGQIQVAASFASSSGLHMVLCVLISGQYDVLFCSLKNVLATTYVLMGANMAELSQLQAEQA--VSDAEYAYSLEE------------------------------------------------------------------------FKQSFKQCIRHHRYIVEALKKMERFYSPIWFVKIGEVTFLMCLVAFVSTKSANSFMRMVSLGQYLLLVLYELFIICYFADIVFQNSQRCGEALWRSPWQRHLKDVRSDYLFFILNSRRQFQLTAGKITHLNVERFRGTITTAFSFLTLLQKM-

>DereOr24a__scaffold_4929_residues_4230966_to_4235452_reverse_strand.[1501_2987].sp

---------------RHYFMVPKFALSLIGFYPEQ--------KRTVLVKLWSFCNFCILTYGCYAEAYYGIHYIPNIATALDALCPVASSILSLIKMVAIWWYRDELSSLIQRVRFLTEQQKSKRKLGYK-KRFYTLATRLTFLLLCCGFCTSTSYSVRHLMDNIL----RTA-HGKDW---IYETPFKMIFPDALLRLPLYPITYILVHWHGYITVVCFVGADGFFLGFCLYFTVLLHCLQDDVVDLLEVENM-----------------------------------------------------------------------------------------ENMEMSLSEADEIRIVREMENLVDRHNEIAELTERLSGVMVEITLAHFVTSSLIIGTSVVDILLFSGL--GIIVYVVYTCAVGVEIFLYCLGGSHIMEACSNLARCTFSSHWYGHSVRVQKMALLMVARAQRVLTIKI-PFFSPSLETLTSILRFTGSLIAL-----

>DmelOr23a__2L_residues_2653643_to_2655830_forward_strand.[501_1688].sp

----------------DYFRVQLNAWRICGALDLSE-----------GRYWSWSMLLCILVYLPTPMLLRGVYSFEDPVENNFSLSLTVTSLSNLMKFCMYVAQLTKMVEVQSLIGQLDARVSGESQS-ERHRNMTEHLLRMSKLFQITY-AVVFIIAAVPFVFE-------------T----ELSLPMPMWFPFDWKNSVAYIGALVFQEIGYVFQIMQCFAADSFPPLVLYLISEQCQLLILRISEIGYGYKTLEENE-------------------------------------------------------------------------------------------------ENEQDLVNCIRDQNALYRLLDVTKSLVSYPMMVQFMVIGINIAITLFVLIFYVETLYDRIYYLCFLLGITVQTYPLCYYGTMVQESFAELHYAVFCSNWVDQSASYRGHMLILAERTKRMQLLLAGNLVPIHLSTYVACWKGAYSFFTLM----

>LhOr218

-------------TLESAIVYIKWSLVLSLSWPLPIT-----KWQIIRFKILAVLSHINIVCFLVPLLILIQDCSDQRVVCIQSYPLICGCFHFWMSVGICHIQYKRFQSLIAEMESYCKHATDYEK--TVLQQYVDRCATFYAVVMISFYSTTCVAISAPLFTS-------------------APFPTYAKYPFDVNYQPLKTIIYAQQSLSGLQFSS-MLCVSLLVALLLWFTTARFDILCNELRKA----------------------------------------------------------------------------------------------------------------ELIQCIQKHQQLLRYANDVIENVRFLVLTVVGANAISVICTGAIFAT-RQPLIINVQFFLMTVISFCDVFTCCWPADSLLTASSDIAQATYESLWYYHNTDKQKNLIFILLKCRKPVILTVGCFIPLSLRYFSSYLSTGFSYMTTLHMM-

>LhOr27

----------YKRDSDYSLQLNRWFLKPIGAWPELSS-----TTRNILINVLRIMCHSLIAFAVIPSILYIFFEEKDFRLKLKAIGPTSHCLMGGINYCSLLYHNNRIGRSIEHMELDWRLANKEHNR-KMMLRNARVGRVIAGVCALIMQGGVFCYNLARGLSP----IIMTI-GNETIT--IGRLPCPSFNKVDTRFSPVYEVVLVLQCLSIVVVNNVTVSACGLAAVFTMHACGQLNVVILRLEELVDEKRE----------------------------------------------------------------------------------------------------------KLMDIVERHLRALKFLSRMEAIMRQICFVELVGSTFNLCMLGYYTITEEESINTIITYVMVLTGMMFNIFIFCFIGELVTEQCKKVGEAAYMTNWYYLPHKTVLGLILIILRSKTVIKITAGKIFHMSIPTFGVVIRTSVTYLNMLRAL-

>LhOr26

---------AYERDSEYSIQLNRWFLRPIGAWPSTSS-----NIEKILSRSIQLICHLLIAFTVIPCVLYIIFE-PDVHLKLKAFGPMIHWLMGGANYCSLLLRSYEIRKCINHMCADWRTVERTCDREVML-RNAKFGRFVSTFCAVFMQGGVCSYSVITAVTP----AIVQI-GNITIT--MHQLPCPFYMEVDARYSPMNEIVLGLQLLSTIIVNSITVGACSLAAVFAMHACGQLNILVMKLDELVDGTENKRETTHQK----------------------------------------------------------------------------------------TENKRETTHQKLVIIVEHHLRVLSFVSQIETVMHQICLIELLGCTTDMCMLGYYTITELHDTKNLLTYATIFIAMSCNVFIFCYIAEILTEQCRKIGDMVYMTKWYRLHHKTALDLILIISRSNAVIRITAGKMIQLSISTFGDVMKTSFAYLNILRTV-

>LhOr25

----------HEENMRLSVQLNRWILKPIGAWPKSAS-----SIEKLLYLLINIICIGLIGFLFIPCAAFVVLEVEDTYNTLKLTGPLNFCLMAVIKYSSLIFRENDIRKGIEHIKSDWMNTQHYSDRTIMIKN-AKFGRRLVVICAFFMYGGALFWYLALPFSN---GTVTDD-GNLT----YRPLVYPVAVIIDARHSPISEIFFWIQCLSGFLVHSITTGACSLAAVFAMHAYGRLEVLMQWIDHLVEGRE--------------------------------------------------------------------------------------------------------VDERVTMIVQQHVRILHFISLTDKVLREISFVEMIGCTLSMCFLGYFTITESKEPASFITYIVLLTSLTFNIFIFCYIGELVTEQCKKISEVSYMIDWHRLPDRKGLALVLIIAMANSSVKLTAGNLFELSLSTFSDVVKTSVAYLNMLRTL-

>LhOr23

----------HEEDMRLSVQLNRWILKPIGAWPKSAS-----SIEKLLYLLINIICTGLIGFLFIPCAAFIVLEVEDTYNTLKLSGPLSFCLMAVIKYSSLIFRENDIRKGIEHIESDWMNTRHYSDRTIMIKN-AKFGRRLVMICAFFMYGGAVFYYLAMPFGN---GTVTDD-ENLT----YRPLVYPVAVIVDARHSPISEIFFWLQCLSGFIAHSITTGACSLAAVFAMHAYGRLEVLMQWIDHLVEGR---------------------------------------------------------------------------------------------------------VDERLTMIVQQHVRILHFISLTDKVLREISLVEIVGCTLNMCFLGYYTIMESKEPASYITYIVLLISLTFNIFIFCYIGELVTEQCKKIGEVSYMIDWHRLPGKKGLALVLMIAMANSSVKLTAGNLFELSLSTFGDVVKTSVGYLNMLRTL-

>LhOr21

--------RNFKNDLQVSMQLNVWTLKPIGTWPKSLS-----RLETLWCRVSNITCYGLLVLILIPGGMYMVLEMKDFYSQLKLGSALSFFMTAVMKYCAFILRENDLRRCVEYIEGDWKNVKYTEDRKIMLK-NANIGRRLIVICGIFMYGAVLFYYVAVPFTR--AKIVEED-GNLT----YRRLVYPVKVLVDARRSPINEIFYFIQLLSGFVAHNITVAACGLAALLAMHACGQLQVLISWLNHLVDGREGIND--------------------------------------------------------------------------------------------------DTVDERLANIIELHVRILNFIAQTEELLHEISLIEVVGSTMNICFLGYHCMMEFQQPVSGLTYVILLVSLTFNIFMFCYIGELLTEQTMKVRESSYMIDWHRLPERKSLAIILIICMSDATTRLTAGNIIELSVSSFGNVIKSSVAYLNILRTL-

>LhOr20

------YNSNYKADIKYTVQVHKVIMGFIGVWPMLER-----CQKRLLEGLLRAACCFLLSFNLIPWALYMFVIVDTFSRRLRMLGALCFYSMVPAMYCTLMLRQDRIRECVKHVEEDWRNVRNVNDRKIML-DKARSGRFILICTILFLFTSGFTYRLIQPIVR-GKIV-----VNENVT--IRPLVQNYYIFFDPQHSPAYEIIFSLHLLTGIVIYIVTASVCGVTALFTMHACGQLEMLVTWLENLANENHWSKEYSTH--------------------------------------------------------------------------------------------KEYSTHARLALIIAHHVRVRRFLNQIQDVVGEMCFIEFIGSTLILCLLGYYVITGRNDALSFLTYAIMLVSFTFNIFILCYIGEVLSTQGSKVNITCCTTDWYCLPSKEARYLILVIAMASYPTKLTANKVIDLSFSSFGTVVRTAMAYLNLLRTV-

>DbiaOr24a

---------------RHYFAVPKFALSLIGFYPEQ--------KRTLLVKLWSFFNFFILTYGCYAEAYYGIHYIPNIATALDALCPVASSILSLVKMVAIWWYRDELKSLIERVRFLTEQQRSKRKLGYKMRF-YTLATRLTFLLLCCGFSTSTSYSVRHLMDNILR----RA-H-------GKDWVYE--TPFKMMX--XXXXTYILVHWHGYITVVCFVGADGFFLGFCLYFTVLLLCLRDDVGDLLE------------------------------------------------------------------------------------------------ERSPSEEQEARIVREMEKLVDRHNEVAELTERLSGVMVEITLAHFVTSSLIIGTSVVDILLFSGL--GIIVYVVYTCAVGVEIFLYCLGGSHIMEACSDLARYTFASHWYGHSVRVQKMTLLMVARAQKVLTIKI-PFFSPSLETLTSV---------------

>DbiaOr9a

------------QRQDQSLRIQILVYRCMGIDLWSP-------TRANDRTWLTLVTMGPLFLFMLPMFLAAHEYITQVSQLSDTLGSTFASMLTLVKFLLFCYHRRKFVGLIYHIRGILSAEIRPDARA-IVDVENESDQMLSLTYTRCFGLAGIFAAIKPFVGI----VVSLI-RGDEI---HLELPHNGVYPYDLQVVVWYVPTYLWNVMASYSAVTMALCVDSLLFFFTYNVCAIFKIAKHRMIHL-----------------------------------------------------------------------------------------------------------------LVQVLLLHQKGLRIADHIADKYRPLIFLQFFLSALQICFIGFQVAD-LFPNPQSLYFIAFVGSLLIALFIYSKCGENIKSASLDFGNGLYESNWTDFSPPTKRALLIAAMRAQRPCQMK-GYFFEASMATFSAV---------------

>LhOr29

---------HRNEYHEYSLQLNRWFLRPIGVWPQLHS-----NTERLLSKIIQITCYTLIAFTVFPCMLYFYFEEQDLDIKMDSVGPVSHWIMSGMNYSSLLWRNKDIRRCIEHIKSDWCMVSKVEDRE-LMLKYAKFGRFVAGFCAMFMHCGVFSYSVVYSLSPIT----IVI-GNQSVS--MRRLPCPFYSKLDTNHDPANHIVLATQFLSGFIANSITVGACSLAAVFATHACGQFAVLYSHLNKLVEKEEKLSA-----------------------------------------------------------------------------------------------------EHKLANIVEHHLRVLNFLSCFEKIMNQICMVELIGCTLNLCLLGFCSIKEARNTKTLTTYGILFVSLSFNIFIFCYIGELITEQCKKVGEAAYFTDWYHLPHKTALGMVLIISRSSAAIKITAGKLVQLSLVTFGDVIKTSAAYLNILRTL-

>NvOr208PSE

----------------------------NGTWPFQ---------NKRKNIILRLLHMGNITAMFIMFSIRLWQEYKNFHIIMENSLALIICFCVQVKIIMLCINEKQRYSFYKQVLALWKDTSDEEEI-MILKQHADFGFKTLKIYIII---SSTIYQVIPILSGVLRYLTDPN-ITY----YQKELPFYIEIYIDQEK--YYYHIFIAMFIMILAAAIITFSHELTFLQSVQHIIASFKIIEFSLIRLSKI----------------------------------------------------------------------------------------------------KDTDKIVLTSISKIVDLHNATLNNIKYIDQAFGTTYFIILLFNSLLFGSSLVLINNNLGDTLYVIRYALIFTGLTTHFFIIFWPSQRITDANESLFQVCCSCEWYKLSKRPINSLRIIMLKSSMQCEITAIGMFVLSLETFSKLFKTGLSFFAVF----

>CfOr263

------------------LPLNFRVLWFCGAWREE-S-----NNGLFVRFISFCYRYSIVILIYEFTVIELIRTHDHIEDLTEGLFLALTYVALCIKYGNFLARQDEVYTLLDCFRGETCQPKNFEEKM-ILIKYDRKAKWCVRAFMSISQATCIALVLAPIVGP-----------QDT----DRPLPFKTYLPYSIVGLYPYLATYLQHIGAIFYGVLLNVSFDSLVYGFTLHVCGQIELLCYRLSEIFKDYPDMAQYRLNS---------------------------------------------------------------------------------------------------ISQCVRHHLCVHEIVRRIQSLFVWTVMLLFMFSMVTLCTSIFQMSKKKILSVGFLSLILYLGSMLFQVFFYCWYGNELQLKSKSIGDAIYSSNWTTATIQDRRSLLFMMSISQKGLKLSYYGIFSLALGTFTWILKTSYSAFNVLQQT-

>CfOr262

------------------LPVNFKVLWFCGAWKERKN-----DNFVCLHFCYKYAIFFLIYVFTIFEIIEVIRTRDQIDELTEGLFLASTHITLCLKYTNFLLRKKDVSELLDCLRVKVCQPKNSTEKMIIKMH-IRKAKWSTLSFLIMSYTTAMGFVIAPIILGLS-----K---N------EWILPTKSYVPYSTSETLPYVATYLQQIASLFYAIMLNVSFDSLVYGFTIHACGQIELICCRLTNNIRGSVNFQKDSD----------------------------------------------------------------------------------------SVNFQKDSDSTASIEECVRHHILVHTLVKKVGALFIWTVMVLFFFSLIILCTSIFLISKTKLFSIEFLSLTLYFSSMMLQIFFYCWYGNELELKSKSIANSIYFSNWTLTTSYERRSLILIMINSQKGLTFSNNKIFALSLDTFTWIFKTSYSAFNLLQQ--

>CfOr261

------------------LPVNFKVLWFCGAWREQKN-----DNLGSLHFCYKYAIFFLIYEFTIFEVIELIRMRDRINELTEGLFLASTYITLCLKYANFLLRKNDVSELLDYLRVKMCQPRNLTEK-MIIETHSRKAKWSNLSFLIMCQATTVGFVIAPILGF-----------GKD----EWILPTKSYVPYSVSKILPYAATYLQQTAALFYAVMLNVSFDSLVYGFTIHACGQIELICCRLTN-------------------------------------------------------------------------------------------------NIRASVNFGKNSDVTASIEECVRHHILVHTLVKKIGELFIWTVMVLFFFSLIILCTSIFLISKTKLFSIEFLSLILYFSSIMLQIFFYCWYGNELELKSKNIGNSIYFSNWTLTTSHERRSLILIMINSQRGLTFSNNRMFALSLDTFTWIFKTSYSAFNLLQQ--

>CfOr260

------------------LPINFFVFRFCGIWKEHE------NSNLFAIFCHRYMIAVLIYHLTIFEIIELIRIRNSMEAVMEXFFXXXFTFVSLLKYLNFSMRQCELHALLSCFRVKICQPRDFAEK-LILKQYDRKAKGIVCFYMLMCQTTGLMFMIMPLLIP-----------------DEKSLPFKTYIPYSITTLLPYVLTYLQQSATLIYGILLNVSLDSLAYGFIIHTCGQIELLCYRLTEIFQFLQENNKKNAYAIE-------------------------------------------------------------------------------------------------IAECAKHHILVYDIIYRIESLFMWNVAALFFFSLINLCTNIYQMSKKELFGPEFFSFILYLGSMMFQIFTYCWYGNELDLKNKNISSAIYTSNWMTISTKQRKNLLLMMMMSQKGRILSFYGICALILSSFTWIIKTSYSAFNLLQQ--

>CfOr267

-----------QSDILRYYHVNKIFMSQIGVWPYQ---------NRAIRILIPTVLTFIDISYVAAEMIRMFDTWGDVDIAVECIISTIIVFACFTKLFNLSFRINEMRYLFSLIEYHWQVFNNSTDV-EILQNYVVFGRKVVIFYSIYVYVSMILYLLMPMSPQILDIMMPLN-ESR-----PRKFLFEVEYRIDREK--YYYLILFHSYVAVIGVMSIVVCADTTYIAYVQHGCSLFAAIGYRLEHIVSREYELSQ-----------------------------------------------------------------------------------------------VNQQTVYRELVICLRKHQLAIQYARLLESSFMLSTGIQLSCNMLALSLIGIQVISNLDSTEDLIRYLSLCAGAFFHLLWMSLPGQRLMDHSMKIFDKACRSHWYTFSAESKRLFRILLYRSNVACTLTAGKIYVMSMENYSMVVQTAMSYFMTFSS--

>CfOr266

-------------EMEEYYFINKFFLSRIGAWPYQ---------HKVLKVLLPCFLTIVQYSVIATEIILLHDTWGDVDIAVESVIIIIPIVGASTKLINIVVNNDKFRHLLRIMNEHWSIFNSESER-YILKYYAKIGRKVTKYYAVYCCTILVLYLFIPLSPRILDMVIPLN-ESR-----PLKYIYQAEYRVDKDK--YYYPILFHAYMSSVITVGIILSIDTMYVICVLHACSLFTAISHRLENIVGQTDVKTDDNE-----------------------------------------------------------------------------------------------NEDYRELMICLRKHQLALEHVRILDSTFTHAMFILLSLNVLIMSVIGLQLINKLGHTEEVIRYICVTVGAFTHLVCMCFPGQLLIDRSAEIFEKAYCSQWYTFSIKSRRLLKILLYRSLVPCTLTAGKMFVMSMTMCSSVMQTAMSYFTTLGSLK

>CfOr265

--------------MLKYYSRNQYFLSQMGIWPYQ---------PRMIKILLPCFLVGAEMSVLATQILLLYNTWGDLSMTIEGIITSILLVGATTKLVNVVTNNKKLQYLLQVMNEHWRLFHSECELH-ILRYYATIGQKVTKYYSVYINIFVVLFMLIPLMPKVLDVIIPLN---E-----SRPVIYVLEGDWGVDKDKYYFLILLHCYLAAVISTRCMVNVDTMYMMCVLHGCSLFNAIGMSLENILCKTKLVQD--------------------------------------------------------------------------------------MNIEQAVIKEYNSQDYCEMIACLRKHQLAIKYTQMLDSTFKSATFLILVLNIMILSLIGLQLINKFGQTQEVIRFGCIAMGSVTHLLSMCLPGQLLLDKSIEVFDKAYNAQWYMFSLKTTKLLSVLLYRSVVPCTLSAANMYIMSMTTFSSVMQTAMSYFTTFLSV-

>CfOr264

------------------LPVNFFVFRFCGIWKEHKD-----SNIRFAIFCYRYIIAILIYHLTIVEIIELIRIRNDVESVTESLFVVLTFMSLCLKYLNFSVRQCELRALLDCFRTKICQPKDFAEK-SILKQYNRKAKEISCVYMFMCQITGLLFLIMPLLIQ-----------------DKRSLPFKTYIPYSTTTLLPYVLTYLHQSIGLIYRILLNVSLDSIVYGFIIHTCGQIELLCHRLRQIFQFLQNNNEKNAI---------------------------------------------------------------------------------------NNNEKNAIAIESAIAECVRHHILVYNIMYKIQSLFMWNVAALFFFSLINLCTNIYQMSKKKVVSLEFFYFNMYLGSLMFQIFTFCWYGNELDLKNKNISSAIYTSNWMTISTKQRKNLLLMMMMSQKGRILSFYGICALILSSFTWIIKTSYSAFNLLQQ--

>CfOr269

------------IWQSRYYAIPRFYMTLAGLWPYH-S-----IRNRYLHFVPIFTICFFLLVXKLICVLYIFLYIFIVRKHLK---CFLISNFYNIHIYNVFFYFEIIKSCLKTIENDWLSLNTDNEKA-ILQRQTAYGRYLTIFYAIFMQLTGFLYILKSVVLI----MIDDT-SNSTKLA-VTKLPFRVEYGHKIDQ--YFYLILTHNYLTVFSHVTATVATDTFYFILIQHACGMFSVVGHSLERIGKDSNNSFDSKPDKINDVNYYKV--------------------------------------------------------------------------------------NYYKVLDCLRKHLHVIEFAELIESTFADILLISISLNMIGGSICGIQVLINLNDAKDIIAPLAIYVAQLTHMFLQFWQAQFLLDYSVLPYESICKANWYYTSERCRKLLLLIMNRTILPCRITAGRVVILSIESFGVVLKTSMSYFTMLRSF-

>gi|383863507|ref|XP_003707222.1|

----------------------------------------------------------------------------------------------------------------------------------------------------------------------------------------------MDLPVATKETPVYELVLTAQFLHQTSTAFSFGTFSALLLMVVLHVGCQVDIMCRTI---------------------------------------------------------------------------------------------------------------KNKEQLKFFINRHQDIILLTERIEKLFTYAALSQLLSNTLNTCCLGFLIVIANG-LPILIKSILFYVVVWLDAYLYCFAGEYLSTKSKLICETAYKCLWYDLHPNESQLLVLPILRSQKGLKLTFGKFSSLSLESFMSV---------------

>DmelOr65b__3L_residues_6299000_to_6301400_forward_strand.[560_1994].sp

----------------------------------------------Y-RSKWHTLVYIQMVIFFASMSFGLTESMGDHVQMGRDLAFILGAFFIIFKTYYFCWYGDELDQVISDLDALHPWAQKGPNPV---E--YQTGKRWYFVMAFFLATSWSFFLCILLLLLITSPM----------WVHQQNLPFHAAFPFQWHHPISHAIIYLFQSYFAVYCLTWLLCIEGLSICIYAEITFGIEVLCLELRQIHRHNYGLQEL--------------------------------------------------------------------------------------------------ELRMETNRLVKLHQKIVEILDRTNDVFHGTLIMQMGVNFSLVSLSVLEAVEARKDPKVVAQFAVLMLLALGHLSMWSYCGDQLSQKSLQISEAAYEAYDPTGSKDVYRDLCVIIRRGQDPLIMRASPFPSFNLINYSAILNQCYGILTFLLK--

>gi|380014835|ref|XP_003691422.1|

---------HYRSDTEYTVRVAKILLTMVGIWPRRNT-----FSNNVKFYVQTTIVFFLMCFLLLPHVIYTYFDCENLTKYMKVIAAQVFSLLAIIKIWTILINRKEIRFCLMEMEVQYRDVECEEDR-SVMMNTARIGRIFTIVYLCLGYGGALPYIILPLISE---RIVKA--DNST----QIPLPYLSDYVFVIEDSPTYEITFVVQMFTSFLIMSLNYGIYSLIASITMHCCGLFEVTNRRIETIL------------------------------------------------------------------------------------------------------------LRGRIAGIIQSHLKAIEYSALVGKSLSIVFLSEMLGCTIIICFLEFGVIVEDHKTFSMVTYFVLVTSMFVNVFILSFIGDRLKQESERIGQTSYFLPWYDFPTEEAKNIKIIILRASRPSSLSGAKILDLSLRVFCDVFKTSAAYLNFLRTM-

>cplu_013008_b1

------------------------------------------------------------------------------------------------------------------------------------------------------------------------------------------------------------------------------------------------------------------------------------------------------------------------------------------------------------------------------------------------------------------------------------------------------LLTAESDKCRDAMYAANWV-GDKPFMHSVIIML--SQQPLILQACSFTAVSMDVFMSVLNTTISYFLLLNT--

>PbOr298FI

--------------MTKYYDVNRLLLLICGLWPYE---------KSKFRYIQAIFYITIILSFIVFQFMTLIDADCSINLITKLLSIVLPMCVCLLKYGAFLYNNQKMKHLMDLIWYHWSIIQDKQEVA-ILKKYTKFSRRFTLFMLHIFSLSIFVIIMGHMLPMILDIIIPLN-SSR-----PRHFYMIMEYFIDQER--YFLWLLLHTSMTLLVAALMVLSIGTMLMSCVFHACAMFKIA-------------------------------------------------------------------------------------------------------------------------------------FTDLITCDFKISYFILLATGVASLALNIFQVFISTSNVNELFTTFTFVVAHFCYLYVGNHAGQIVTDHHVDVFNATCRSYWYTAPLRAQKLLLFIMQRTSKNFSFVFGGIFVVSLKGFSTLASMSISYFTVIYSM-

>LhOr170

----------WNNDVTYAMAPYKLLALPLGSWPLQKY-----DKFSLLRYILCCLGM---IAMLILMYVEIYHDCIDTSEKIDILMLTTCSIIALSKISIFRIYANNLTRNFTSAINDYLAIDTEEKRTIMLR-HAFMGRAIYYSIVLFGVMGILGLILTPFITS------DNN-AQLNVSMNEFTLVYPVPTPCHI-STNLYLLIFVTQCILIVMTCTANLGSDTLLFGIILHICGQIEVLRFEIANFGVENKNI---------------------------------------------------------------------------------------------------NKNIDQIFSKLTRRHSYLLTHAKLLIDAISFVLLVQLLVSCILICTIGLQFILATSDAMMIAKTVSALVTFMSQMFAYSFVGDYLKYQAEEIAKSIYGCNWHRFTAKLMKNILFVIARSHQPVQLTAGKFFVVNLETYMSILKTSFSYLSFLR---

>NvOr214PSE

-----------------YYRINKILLKAYGLWPETC-------NKTTKRTIFGFLNLATFGLIFIFAGMMHYVNENIWVKALENFVAILYLIAIVLEYNINYLLENKVKLLYEKIEYDWKMLTDQNELD-ILHKYAQFSRKLITIYIYYALVACILFLILPLAPIAMDYIIPLK-SGIRI---RIQTIHPYYFNFDVNK-YYYYVHVFHVSAVGFYVSAAYLTLNSMHVACIQHVRALFAVACYRIEKLGHHDNTSETS------------------------------------------------------------------------------------------------------------------------------------------------------------------------------------------------------------------------------------------------------------

>gi|167876334|gb|EDS39717.1|

----------------AVMPFTLRCLRLFGLRGDR-------------RNRVHFVLALLVVVIY---VPKLVFGFRDVDLVIRSISELFFECHIDLNAVLFAVKLDEFEELLCLLRKLYNKVKTLDANERMIIEANLAIDKRSKSYVVYVAIACTIFFWVPVAQTTGIWILSHG-SNITDR-PEFVTMMELNFYMDNRKDIVHYVIYAISGVAHYYAAVYFALSGIVIFGCVKSIAALFDMVSARLAALHE----------------------------------------------------------------------------------------------------------ELREELVDLIELHVDGLRCIELLENINNLAMMVQMINCVLIWISMFLSI--SSHFTPEVVSLMVLLLVTTGETYVLCQLATELS-QSLTITDSIWNSNWIGLPVDVQKGLAMMLQRAQKKAGLTAAKFCFMDIERFARVAQSSYSVFVILK---

>PbOr83NF

----------------WAIRLNRISMKFLGIWPENNT-----KQKIIMSNIRTGIILNMLWCLTVPTIHSLFRIWGDIMSMIDNLQYSLPLLMSTTKLSIIWQKKKDILPLLNMIKNDWLMLKTAKERDVMI-KYARIARTLTIFGYIIMLLSFILIMVPPLFGISI----RYL-TNKTDP--DKLLPMQTYYIYDQNKTPFYETTYILQTFAITTMGIAYSASDCFLGSLVFHICGQLENLKVRIIELDKFDN------------------------------------------------------------------------------------------------------DNFEKALICNVQNHIRLIRSVAIIENTFTLMLLVLFLYFGTMVSTYGFLVVIVNVSIKRATFLIMVICNAFTHMCIYCAVGELLVIKYEEIYNAVYTCKWYAVKTMEAKNLILLLVRTSKPLFITAGKILPMTMSTFCSILKTSSGYISVLLA--

>gi|340710686|ref|XP_003393917.1|

--------HSWDKDWMYSIQINRWLLKSIGVWPISLT-----PTEKINSIILTLISSFLISFLLVPCLCTLLDKTGDLDTKIKMIGPLSFCVMAAIKYYILVSRGAKIGKCIENIRSDWDRIHARQEDREIMKENARIGRSLAIFCAGFMYSGGFFYTTVPLCTE----RTEII-DNET----VRSQAFPIYRGLDPRTSPSFEIVQLMQCLAGFVIYSVTVGACSLAAVFVMHVCGQFGILVKKLQRLVGSLEE------------------------------------------------------------------------------------------------EEKNLNVHEQRLGDIVEHHLHILGFISQIEDLLNEICFVELIGCTMNICFLGYYLLTEQSETIGTLTYCTLLISFTFNIFILCYIGEILSEQCRNISLSAYMIDWYRLPQKKALGLILIFAVANTSTKLTAGKLVELSLASFCSVLKSAFAYLSLLRTL-

>gi|167865579|gb|EDS28962.1|

-------SQTEPSDFYQSVVIPNNAARFVGLDVFT---------EGYKFSWRTFCMIVSILVYIYCCVFTAYEVRCSVKELIFCLVTSGLAPHKTVHVWTFLMFRKELYWFHQYTKDLYREECKDRTK-SMLMKNVSLLSVTTKLMLVAYTCISSAMDIVPIISSAA---------SG-----EKTLPFGFYIPFDHTSSPGFTLYVLCDCIYLIAMASSFTQIDVLMS--------------------------------------------------------------------------------------------------------------------------IEAKKSDVGECLNQIIVRHQEHLKYLGTLESVFRIYFLVNFLS--------WYQGY-------------GFILFISYGLFFGCFLGTLLAMKSEQLERAIYDVPWYKMSLANQKSMKFLLNSAQQPVSMTFI-FYKLDVPTFLQMYKMIYSTFTMLLTVR

>PbOr242

-------TERKDIWRSRYYTIPRLYMTLAGLWPYH---------QISNRYLHFVPTFIICSIILIPMLLYVLIAMTGLEDLFECMPTILITIIFSFKLASLMANSEKIKACLKTIENDWLSLNTDSEKA-ILQRHAAYGQYLTMFYAVFMVMTGLLYMLKSIVLI----MMEDT-SNSTKL-AVTKLPFRVEYGQKIDQ--YFYPILIHCYLTVYSHVTATVAADTFYISLIQHACGMFSVVGHTLEHIGKDMN-------------------------------------------------------------------------------------------------PDKINDYNYNKALDCLRRHLHVMEFAELIESTFTNIFLVSVSLNMIGGSICGIQVLMNLNDAKDIIAPLAIYIAQLIHLFLQFWQAQFLLDYSNVPYESICRANWYYTSDRCRKLLLLIMNRTVSPCRITAGKVATLSIESFGVVLKTSMSYFTMLRSF-

>DgriOr71a_scaffold_15110_residues_14521578_to_14523870_forward_strand.[501_1793].sp

----------------KASRIVVRFLHFFAIWPNQGT-----PWIRRCNRYWPYLIHGVMTFMFTVLMWVEAFVTNDFERRAEVLFIMLTMTSLLAKIVNNWRRHHIARELLEEWSTSQQKVNFGQER-DIWELAHRRFALVTYVYIFCSAGSGVCILMSSLLNY------------------PNKLPFWMWVPYDWTQPVNFWCTFAFQVIAVPFSCLSNVGMDLLNCYLMLHISLCLELVGMRMAALAPTGNSLESEQQ------------------------------------------------------------------------------------------SLESEQQLLAQFVAIIALHRRIKMHTNRIQMFISSSTLIQILLSSIIICLTIYCLQMRLQDPFFFLMKSQYLFAMTWQIFLPCMYGNQIIDKANQLVNDLYTCQWPDMSVKMRRQIFTFMIYLNRPMELQAGSFFVIGLVLFGKIMNQAYSLLALLLNV-

>PbOr240

-----------KDIWQRQYYILKLYLTISGTWPYH---------GLYGRYIRFVLIFTLSFTILIPQLLYF-LVFLNLDNVFECLPTIMIGTVFSFKILTIMLNSEKVKICLNTIKEDWLLLNTSIEKD-ILQRHTKFGQYIATFYAVFMHMTTCLFVLKPVMLTLLA---EDV-LNVT----ESTIPFASRLPFHVEYGQYVYPIAVHCYAAILAHAFATIAIDSLYYTLIQHACGMFSAIGYVLENIGKNSDN------------------------------------------------------------------------------------------NNFDLMPDKMKDDNYIKVLNCLRRHLHVIEFAEHIESMYTKIFLLNLNFNMIGGSLAGIQVLMNDKNMNDIAGPITVYIAQFIHLFLHFWQAQFLLDYSVLPYESICRANWYYTSTRCQKLFLLIMNRTVSPCKITAGKIMVLSIESFAVVVKASLSYLTVFRSL-

>PbOr241

-------DERKNIWQSRFYTVPRAYMSLVGIWPYH---------AFRDRCLLFVPMFMFSITILVPQLLYLMIAAVDLDDVFSCTPSMWITIIFSFKLGWLMMHNKKLKTCLETMENDWLSLNTNVERDI-LRKHTAHGRYITLTYGVFMQFVGILLISKSLVVMLL----EDT-SEAT----VSSLVAESKLPLRIEYGIYLYPMAIHCYLAVFSHISITIAVDSCYIALIRHACGMFAIVGHTLEHIGKDS----------------------------------------------------------------------------------------------------KVKDDNYNRALGCLRRHLHVIQFAELIESTFTNIFLVSVCLNMIGGSMIGIQVVLNLNDAKDIVEPFAIYIAQLIHLFLQFWPAQFLLDYSILPYESICKSNWYYTSGRCRKLLFLIMNRSVLPCKITAGKVVPLTIENFGTVLKTMMSYFTMLRSF-

>PbOr246

--------------MEEYYAINKFFLSRIGGWPYQ---------RKVLKVLIPCLLTMVQYSVIATEVLLLYDTWGDIDIFVEVIITTIPILTSNLKLFNIVVNNGKFQCLLQLMNDHWKLFNSEFEH-HTLRYYANIGRKLTNYFGVYFIFVVIFYLLIPLIPRVLDIIIPLN-ESR-----SLTFVYQAEYRVDKEK--YYYSILCHSYISTSITLTILFTVDTTYIVCVLHACSLFTAIGQRLENITDRTNDKKPNTKKYYYLLKE----------------------------------------------------------------------------NDKKPNTKKYYYLLKESHGSVGSDYHELITHVQILDSMFTHVTLCVLTMNVLILSVIGIQLINNLDHIGEVIRYVFVTVAVFTHLICMCVPGQLLIDRSIEVFDKACNSEWYTFSLKTRNLLRILLYRSFVPCTLSAGKLFVMSMTMCSSVMQTAMSYFTAILAMK

>PbOr247

---------------TRYFKINKFLLSFIGLWPSQ---------TPFIKLLTLSFAIYGLTTMCLPQIVYLFKNANELDKMLELMPTLTGTVICIMKVISLTYNVEKFKMLLRQTCEDWDSLLTIEETQ-ILTRYAENSRKFTLLYSICVIGFVCCYAFLPLIEPILDIISPLN---ETR---PRKMQHSMDYVLDLEK--HYYLILLNTYLGYIVCLMIAVATDTMYVVMVVHICGMYNILCNRLEKITTYDN--------------------------------------------------------------------------------------------------TYQHDEIGRRVRHCIQLHKRIKLFIETMESTFALFLLFDIGGGFLLHTSSCIMIVIRIGS-SEIVRYVALVIMQSCRLFFNSWAGQQVIDHSLEVSIAAYKAVWYNISVKTQKLLILLIARSQKPTQITMAKLYIINLQGFSTVMRTSVSYCTVMISLR

>PbOr244

---------------TRYYSVTKLFASVVGMWPYQ---------SQLKRRIIQSISMFIMFTFFPPQVKRLYDVWGDIDAMCMCVPPIMTILVSMAKVITSARYDTEIKMLLRQIEVDWKINPNAEEY-KILSNYTSKARMLCIVYSTTLCNGLMCFIMTPMISPVLDIIVPHN-ETRT-----RSLAFDLDYGIDLQT--HWLWLWMHSSMTSMATIINIIGADLMYVTLTIHGCCLFAIVRYKLEHVADSIKKHMQSFENHNHNIKYYEY-------------------------------------------------------------------------------------------------------SVQLLHCIYTWGFLTILSLNLINTSISAVQLLSKLFQWDAMAMCIMFICGQLIHLFFLSLMAQYLLDQSSNVHESTYSGLWYNMPTKIQKDIVLILIRSRIPCKLMAGKLFVMSLENFCTIVQTAMSYFTVLASFR

>DyakOr46aA__chr2L_residues_18659200_to_18662927_forward_strand.[501_3228].sp

-------------------KGQKVFLNIFSLWSQNEP-----CWRIIHQVNYVHVIVFWVLLFDLLLVLHVVANLSYMSEVVKAIFILATSAGHTTKLLSIKANNVQMEELFKTLDDEEFRPRGAKEE-SIFASACEKSRKLRNFYGTLS-LAALGMILIPQFAVD-----------------WSHLPLKTYNPLGENPGPAFWLLYCYQCLALSASCITNICFDSLCSSLFIFIKCQLDILALRLDKIGRPSS--------------------------------------------------------------------------------------------------------VEQKLKENIRYHMAIVQLTRTVERLLCMPISVQIFCSVLVLTANFYAIAVLSEERLELFKYMTYQTCMLIQIFILCYYAGEVTQGSLDLPHELYKTSWVDWDYRSRRIALLFMQRLHSTLRIRTINSLGFDLMLF-SIVNCSYSYFALLKRV-

>DgriOr98aL2__scaffold_15110_residues_8655150_to_8657467_forward_strand.[501_1818].sp

---------------RDAFKYLERGMKCVGWIPPPKE-----KLYYWAYRFWSVVVFSLGIYTQVGLYATYIFDSFTVNTFLSSIQSVPDATTVPLKVLTLLPNIWRFHKAWDLLDLMDKRCTRMEERF-EVHRCVVRCNTAFIIYTAVYSIFLTLTYLSSVLM--------------------GSTPWGFYNPFDYRSSPNLWIACTFEYFVGSAASYLDEMADLYPLMFGLILRTHIKLLTERINRLRTNSDETE------------------------------------------------------------------------------------------LRTNSDETEDQSYEELVNCCKDYKLIVEFCNNFRPIISVTIFLQFISIGVCLGLTLFNLLFFATFWIGLA-TIAFIILLVFQTFPFCYVCDLVEKDCEMLSIAIFQSNWVDADKRYKQSLIYFLHRTQQPINIKAGGKFKICLQTNIEMAKIAFSVITF-----

>gi|388429169|gb|AFK30393.1|

----------------KYFKSIRTFMVAPGAWPAEIG-----EKISVLVIFHR-ATLPYHTSLIVVGFYYIWMHMDSFLDLGHMIITALLGTLTAVRSILLQNYHMLLLKFINVMHLMHSTNKGRYYN--QMNDTDKVCSYYTKFSLVITFFSCLMFNFIPFYNNVTNVFIFKT-ENYT-----LEFALYYQYPIDPSD--YFTITSIYNVYLSYNCAIMVFGLDLILFLIIFQIIGHVYILRYNLENFR----------------------------------------------------------------------------------DILKYKMNESIT---SEMFDAEENEEVRFKLAECIEHHKEIIGFTDELSALFGPILACTYLFHLVGCSLLLLECS--EGGYGAMLRYGPLTLLIYGQLIQMSVIFEMLGSETEKLPDSAYFLPWECMDNSNRRTACIMLHKMQYKISLKALGLAAVGVSTMTGILKTTFSYYAFLQTM-

>LhOr177NTE

----------------WAIKLSRYGFKLFGLWPENHT-----NKNMFVSNIQASFIFIMILVLIVPFIWSLVRVWSDMILMIENLQITLPIIISCLKIVIMRWKRSAILLIVNMMAEDWMHLSTEAERYVMIK-QMQIARLFTIFSYCIGAFGVTTVTVLPSFGLHF----RVI-TNLTDR--DRVLPMQAYYFYDTDKSPQFELTLATQIISMFFTLIIYMSVDAFFMLTIFHICSQLKNFRYRLLNLV----------------------------------------------------------------------------------------------------------NDFNNALRYIVETHLRLIRFADNIEDIFSLSTLAILLYFGVVFCIYGFLFVTIISSNETSYIVISGSISFLVFLLFFCIGGELVANECEAVYRALCDLEWYILKPTESKALILITMRASKSFSITAGKRFPLSMATFCNVLKTSVGYISFLLA--

>DeugOr23a2

-----------------YFRDQLFIWRICGAMNLSE--------GNFCSWA---LLFCVFMYLPTPMLLKVLFSFDSPLDNNFNLCMSITSLSNALKFSIYAAQLKKIVEIQALIAKLDDRVSGEDQE-LRHRQMSVHLRNISKVFVVSYSLLLINAAVPFLFNS------------------ERSLPIPMWFPFDWKTSTAYIAAVFFQEVALVFHTIQNFSGDSFPPLALFLVSEQCQLLILRISEIGYGSKTLKAN----------------------------------------------------------------------------------------------KTLKANEQELVNCIKDQNTLYRLLDIVHSLISYPMMVQFLVIGFDIAITLINIIFFVETMSDRIFHMSFLLAITLQTYPLCYYGTMVEESFADLHYAIFCSNWVDQSSTYRGLMLIMSERTKRRQLLLAGNVVPIHLSTFVACCKGAYSFFTLM----

>gi|383850508|ref|XP_003700837.1|

--------SEYERHVNLSIQWNRWLLKPIGIWPHSDS-----RTKKLYDWFTYVVCYGLISFLFVPCSMYVFLEVEDLYNKIKLFGPLIFFLMAFAKYYLLIYHGNDIRECIERIKWDWKNITYHDDKNIMLE-NANFGRRLVTVCTFFMYSGFASYYIIIPITI--GKVKSED-GNLT----FI----PLAFPFDTRYSPTNEIIFSVQLIAGALMHGITSAACGLGAMLAVHACGQMQVLMNWLKHLNDGRSDMGESV------------------------------------------------------------------------------------------------ESVDDRIACIVSQHVRILKYLTHTERTLQLISFAEFLGCTVDICLLGYYVIMESNDITNTVTYAILLTSLTFNIFIFCYIGELVAEQCKKIGEMTYMTEWYRLPGNKKLYCIMIIAMSNSSMKLTAGSMVELSIETFTNVVKTAFAFLNVLRTM-

>McOr29

-----------------FLKIHRFYMLILGKWKITP-------RNTLQNKLYKIYGWFIHIFFIGILMPYINRTKINWDVITKRGA--LYMICNNMFIISLISNSVNMQKLVEFILNYEKLL-YPSEKNFVQKTYDFYCKMNYNIVMIFIVIPSFFAYVYYGFEVILTFLKDPN-DPC---FTTKGLIFQFWLPFDTDK--YFYIAILFEFFLMSLAICFNTYNKLIPCSMSTFQLGQIKMLQEMLRHVDEEARELNASQCVEMDEAVDAF-------------------------------------------------------------------------------------------VTECIKKLQDILSLMNLLHKATRPVMLLAFFTNILETAFFMIRMLTAK-SDAEAITALGVSSVIFIQILCFFWQANEVQLESQNILDVIYNTNWVDYNISVRKKLLIMMTMVQKPMSFEALGIGSMTIETFKKILKSCYSIVTFFK---

>383_Si_gnF.scaffold00330_12820-264637.pep

-------------------RLVRFGLHVYGIRPYV--------TSTVVFRLYWIIMLS---TAQVFQYRYVVMHMDDFSEYMDGVSSAMASSLLYIKLIILWTHERIFSDLLQMMSTDWQDYISTRHSSRIMTNAANLARRTSRWIVGMQVASGTFYSVGVLASNA----NNPE-KL-----ETRELILKMELPFNISTEFIYTAVQSVQFYHLSLVCYGITIVNSLLVTLILHICGQIDILRECLLKVFSKNSAES---------------------------------------------------------------------------------------------------------MRSLIAKHQRIIIFAEHIETLYTYIALMMLLSDTIIICCLGFIIVISLDSPNILVKSMLFYISMNVEAFIYCFSGEYLSAKSKMIGNAAYDSLWYDFPAKESRTVLFLIVRSQKRLTITSGKIVDLSLERFTSVVKASLSYISVLLAM-

>cplu_015722_b1

--------------------------------------------------------------------------------------------------------------------------------------------------------------------------------------------------------------------------------------------------------------------------------------------------------------------------------------------------------------------------------------------------FLLLVLTVNAIGLSTIGFRFVAMASTMEDNIQCCAFLFGQVLYIYYLSSFGQKLMDHSAYVFESICAAK--------------------------------------------------------

>McOr26

---------------------PLGWLKTCGANPLQ--------KRKIGYMLVNISSTICLAALIV---LKMIFG-----PYLEAAELWITFMLMALKYLVLLQKKEKLQSLFKDFESFWVLESKDDKIS-QMLI---YLKRLTLCWTFILYVGLAVYLVKPIFLR-------------------DASVFFCYIPQHV----PFLCVYLVEVYYLFFVAHSFVGFNLLITTFIILTTTQFRQINMRLQRLDM------------------------------------------------------------------------------------------------ERIQDQSGWESCHNILRICVKHHNFLIRVVDDLNNTLYTAVGLLIGATTVVMCMHMYVLITIDLTSVEIARFLISFSAMVFEMFGYGLPSQMLMDEAGYMTEALYNCSWYLAPTSIKKEMLLMLMRSQRIVCISVKKVVIVNNQTFLMMLKTAYSFYTFLRTL-

>DmirOr69aB

----------------------------YGWSGRQSG-----AKQTLMKKVIFVLGALNMSCYFFSFITYGYHIERKVAELSEVGGMLWFTVLGICNMYTLLLYRPQIEELLEGLEQLFAPARQSPYCTRYF---YDESALKMKRLGINFVCSATYYNLLPLVKL-----LSEL-LTESQQV-SYQVQSKAWYPWQVHGSLGFWIAYASQAFASVMNLGMMMATECLVFVCTAQLELHFDGLARRLEALDARD----------------------------------------------------------------------------------------------------------KEQLQALISYHTRLFKVADRANGIFNFTFLISYCVSSIAVCSMGFSMIM--FDLGLALKYMVGMFLFMIYTFCICHNGTQVTMASDKVMPAAFYNNWYEGDLVYRKMLLILMMRSTKSYVWKTYNLAPVSIQTYMATLKFSYQMFTCVRSLK

>gi|380028686|ref|XP_003698022.1|

---VTSESKKYSKDYEWAVRLNRFSLNLICLWPVEEQ-----NIKQSWTKLHIIACFMLITFVCTIPCLCALKQCNTLMEVTDNLAYSIPLIITTIKFIVISSKKKVLSPIVNMIAKDWLKLKTDYEKDVMIQR-ARIARIINIFGYILMCILIWLLMILPRFGI----TIRYV-MNGTDA--KKLFPLPTYYIFDVSETPYFEITYTLQSISLLIAAFCYAGVDNFFGILILHICGQLTNLRFQLANMKESET----------------------------------------------------------------------------------------------------------FVLIAIVKDHIRLIRAANVIENTSTLLFLILLLNFGICTCIYGFLIITIQFSLLRIIYLICNFTNTFLQTFLYFMAGQMLVTQSEEIHNAAYECEWVSLKYTKAKSLIIIMARSKKPLYLTAGKLFPVTMLTFCNI---------------

>McOr22

----------------DYFGMIKIMYGIAAIWLFNPN-----GSVKCLKYSWTLILYSIVVTFVIFEYIMIEVMFKDIFTLIAQMGLLLCGHISLLKATVLIKNHKKLAEITDFLEDERYHYKSVGNFGKLVVDEKRFTNNFLKMLLVCFGLVGVSPHLAA--EKIIHQEVKGN-YSENVTC-YDYLPFIFYIPFPSETKHMCEVAVLFMDMSISGIAIIIACHDGFLVVLLNCVRVQFVIVGEAFSTLRERVLH-------------------------------------------------------------------------------------------------PQLEAELYKELNVVTRHYALLLKISEDLEEIFNKIILVQTLLCLLVFATCIYAGTTVPITSPTFGASVQCFSCVLAELALFCWFGNGVTTSSEGILLALYKSDWFSASKRFKSSMILTMTRVQKPVYITLGKFGPLTLVSLVSVCKASFSYYTLLKKM-

>gi|379070060|gb|AFC91741.1|

------------------------------------------------------------------------LKNNDFAAACTNGILSVLYIAVTFKYVVLLVKVEDITFAMNKVKGDFAAAKHSDE-QAITTEYAYKACWVTKVWLLTASSVFCVFPIQVIVLSIYNYAIGD--------F-QFVHMYQMTFPEETRETNMYLFLLILQTYFGVYVLLMFAGFTPLGLIFMLHVCGRIEIVKYRISKLFEGE-------------------------------------------------------------------------------------------------------REIHQRLKNIVTPLQDALDFVDLIKKTFRLVYEVYMKFTTIVIPIASYEVLESLKEGRLSIEFMTFIVAGAVLCFAPCYYSDLLMEKGLSLRMSVYTSGWEAYDSAMRRTLCIIMCRLERDVAIRTL-FQTVNLDAFSELCHQSYALFNVIN---

>McOr20

--------------------VSIKILQVCFLFPLKGK-----ELPNYLRGFIFFFLMGFSSLTVIGSFLHFIISIKNVYYHIDLMAIMISMFTTYSFSIVFFFNIKSAVRLYMTLSDFDEHGKPRNFDK-----RTKLIDKVVTYYYIYIEFLIIFMLSTSNVSSSGKCKKKNK-KNE-----VCGLFSYTWMPFEIDYYPVKQIYTICQLVGTHYLIILAGVVSCLMAETMEQIITRIHHARY---------------------------------------------------------------------------------------------------------AIKEKDYAKQRQMFNTAVRYHIGVLDLEDPLNETYGFFMLTHLAMTAPIIGTALYSILY-GGSGSSTF----ICLGWFIGVMKDCCCGQRLQSQSNTVPIAIYDSEWYTCNEEIKKDILFVLMRCRRPMYPKAISFGVLDHVMFLGVVKAAYSYIALLSQ--

>BmOR-22

----------------------------SGFWYQKTR-----NDKTLLYKIYSCVLFFTYGFMTVLEIMAATMGDFPDDEKRDSVTFASSHTLIMIKFISIIKNKELLKTLNRKMMMICEAH---EE-QTLMDEMYRIVKINVVAYCVAVYGSVTFFVFEGL-RKFY----DG----------SHFVTIVTYYPSKDDDTLASIFRIATTLVLLVMMLSMIISVDTYTMAYLIMYKYKFITLRHYFKRLRENVDEL-----------------------------------------------------------------------------------------------------KLAQGLVEGIKMHNELLSLSKDIHKAFGTVMALQLCQSSGSAVSLLLQIAV-----TMYL----------------------------------------------------------------------------------------------

>DgriOr67d-2_scaffold_15110_residues_12636465_to_12638667_reverse_strand.[501_1703].sp

-------AASASSQFSKIIGLVRKCTNVCGCDVWN---------PDYHTWAMTYFTITLVVFMYTSNIYVELVYNNDWTVILRVFSIAGSTMQAQAKIQSTLEYETSLRELMVTYDLMYKEYEAKGGYIKCLQKRVKNTWRMLIAFMLLYLIVAISMAVYPIYLYVV----H----N------EKTLVMQFLVPINHNTDRGHEILTVLHVIALGMGAFGHFGCDMFLFINIANLPLLTDLFKVKINEFN------------------------------------------------------------------------------------------VKINEFNELVVQSNKNEQIRDMFWELLAWHQKYSGILRKTEKVYNLIFFVQLSTSCINILCTISCIFLKVWPTA-----MLFLIYSVIVLYTYCGLGTQIEHSNDDF----------------------------------------------------------------

>gi|350405968|ref|XP_003487613.1|

------------------------------------------------------------------------------------------------------------------------------------------------------------------------------------------------------------------------------------------------------------------------------------------------------------------------------------------------------------------KEKLQKYIIDDIVGRHQRIIQFSKNIEKIFTYISLCQFVSNMLVICFISFILVSSDQATVIIMKCFPYYIAVNCEAFILCYTGEYLTSKSEDITKSVYNFLWYELKPQNARVILLMILRSQGKLTLTAGKFLCLSLEAFANMLKASASYVSVLYAM-

>DkikOr83a

----------------DLFVFVRQTMCIAAMYPFGYY-----LKGSVLYELFNYFVSVHIAGLFIC-TIYINYGQGDLDFFVNCLIQTIIYIWTIMKLYFRRLRPRLLDDILASINDKYEPRSAVGFSYVTMAEAYRISKLWIKTYVYCCYIGTIFWLALPIVYR------------------DKSLPLACWYPFDYTQPIVYEVVFFLQAMGQIQVAASFASSSGLHMVLCVLISGQYDVLFCSLKNVLATTYVLMGANMAELRLLQAEQS--VSDEESQYAYSLEE-------------------------------------------------------------------RKAFRQSFVHCVQHHRYIVAALKQMERFYSPIWFVKIGEVTFLMCLVAFVSTKSANSFMRMVSLGQYLLLVLYELFIICYFADIVYQNSQRCGEALWRSPWQRHLKEVRSDFLFFTLNSRRQFQLTAGKITNLNVERFRGTITTAFSFLTLLQKM-

>LhOr192NTE

--------------FERAVKLNRVSLELTGLWPKIQN-----FQQKLMCNIRVLVIFLAICSIIIPGVHSLIVAHSNLMLVVDNLHFTMPLLNCAIKIVIFWWKKEATATIINMMVEDWLRSKSAQERNVMM-RRAESARKIVAIEYCLMGLAYVFVVILPICGIPI----KYL-TNVTDPGRPTPGPIQTYYIYDVMKTPQYELTYITYSITLFFAILCYAGIDNFLGLVVFHICGQLDILRHRFTHLDK-------------------------------------------------------------------------------------------------------KYMNFHIDLKNCVMDHTRLLRAITIVDDLYNVLFLILFLCFAVLFAFYGFVIISLKVSIVRLIYLVSNVINLFAHMCLFCAVGEFLMAQCDTIYYAVYNQEWYTLGSNKAKNLIPLIIKSRKPVYLTAGKVFPMTLATFSNLLKTSAGYISVLFGM-

>DanaOr98a-2__scaffold_13340_residues_19024762_to_19027055_reverse_strand.[501_1785].sp

----------------------FRYFEYAMFWMGWTS-----KKFKVLYRIVSVFITAWVIYLPIGMLINLIIDTKTPKELLNTLQIFFNGLGTPIKVFFFRIYFWRFYKVKKILHEMDKRCQTEEEQ-IEVHRWVVLCNKVYLGYQAMYTGYSLTSFLSAILTG---------------------QLIGIYNPFDWRKSQSFWLAALHENALIMFSANHTMMSDIYPLLYALILKVHINLLRLRVKKLCEDPHKSDD-----------------------------------------------------------------------------------------------KSDDENHRDLIKCIQDHRLLKQYADLIRPVIGSTIFVQFLLIGILLGLSMINLLLFADIWF-GLSAAVYIMGLLLQTFPFCYVCDLIRYDCGRLSEAVFHSNWLTSSQKYKRTLRFFLQNSQKSIAFIAGNIFPISTSTNISVAKLAFTVATFLKQL-

>BmOR-28

-------------------------------------------------------------------------------------------------------------------------------------------------NLFFFCSSVIFKTMKYCVD--Y-KIVYKE-YNKTHDFK-RLMVHEMKLKYDIQSVPPFHCMFAYNFLQVCVLILNYSGFDGSFCIASIRLCMKLKLVVYKVQKAFAESKSVSE-------------------------------------------------------------------------------------------------------------------------------------------------------------------------------------------------------------------------------------------------------------

>385_Si_gnF.scaffold01629_62255-192132.pep

------------SEMEMYYATNKLFLYRIGVWPYQ---------RRVLKVMIPCFIVIVHISVVISEALLLYDTWGDINVAVDCIVNLILLFTADVKLINMVVNNRKFRRLLELMNKHWELLNNKIESH-ILKYYASISQKLTSYYAVYLIVIIIFYLLIPLTPKILDFVVPLN-ESR-----PLAYIYQGEYRVDKEK--YYYPILFHSYLATACTMTILFTCDTTYIICVLHACSLFTAIGEQLENITSKAGTTSNNDGEIHTEMQYHTF--IKK--------------------------------------------------------------------------------NDYKILITCLKKHQLAVENAQTLNSMFLHVTFILLSMNMLVLSIIGIQLINNLENTKETIRYICLTGATFIHLVCMCIPGQLLIDKSTEILDKTYGSEWYTFSNKTKKLLSVLLYKSLVPCTLTAGKMFVMSM---------------------

>5_389_Si_gnF.scaffold00899_962711-1051965.pep

----------RNDDVAYAMDPLKFLTVPLGIWPLQ--------KYGIFPLIRSIVSVFSLVVWLITLFLEVNYSNSDAYVKLDQLMLLSCAILSTLKIVFFRLYADNLICNFFSAVSDYLAIDTEEKRT-IMRRHAFIGRMISYSTISLAYVAATMFILLPMLSD----------ENAQVNVSAADLPLPMTWILEYHFSSLYYTIFIVQYYLLLLNANANVGNDSLFLAIVLHICGQMEFLKTEFTNYGVKSKNLNE------------------------------------------------------------------------------------------------KSKNLNEDFLVLISRHRYLMEHAERLVDVISFVLLVQVLISCIIICVIGLSFIVATHDMMMITKCGSVLSALLLQLFFYSFVSDYLKCQMEDVAHSIYSCDWYSFPLKLMKNVLFVIMRSQQPIQLLAGKFFVVNIETYMTILKTSMSYLSVLRVM-

>DbiaOr65a

------------------------------------------------RSFWHTLVAIKSFVFMATMLYGLTESIGDNVQMGRDLAFIIQIFYITFKIFYFLRYGDALDEVVNDLEAFHPWAQQGPHA---VD--YRSGKRWYFVLAFFVSTSWALFLVIFLGLL----VTSPM------WVQNQNLPFHAAFPFQWHTHPTHAIIYLFQCYFAAYALTWLLCMEGLSISIYVEITFAIEVLCLELLSLHR---------------------------------------------------------------------------------------------------------EQLRLETNRLVRLHQKVIRILDRINDVFHGTLIMQMGVNFSLVSLSVLEAMEARKDPKVVAQFAVLMLLALGHLSMWSLFGDLLSQTSLKVSEAAYEAYDPTGSKEVYRDLCLIIRRAQDPLIMRASPFPSFNFINYTAILNECFGILTFLLN--

>DbiaOr65c

-----------------------------------------------YRSFWHTLVAIKSFVFMATMLYGLTESIGDNVQMGRDLAFIIGIFYITFKIFYFLRYGDALDEVVNDLEAFHPWAQQGPHA---VD--YRSGKRWYFVLAFFVSTSWALFLVIFLGLL----VTSPM------WVQNQNLPFHAAFPFQWHTHPTHAIIYLFQCYFAAYALTWLLCMEGLSISIYVEITFAIEVLCLELLSLHRR---------------------------------------------------------------------------------------------------------QLRLETNRLVRLHQKVVEILDRINDVFHGTLIMQMGVNFSLVSLSVLEAMEARKDPKVVAQFAVLMLLALGHLSMWSLFGDLLSQTSLKVSEAAYEAYDPTGSKEVYRDLCLIIRRAQDPLIMRASPFPSFNFINYTAILNECFGILTFLLN--

>gi|380014579|ref|XP_003691305.1|

-----KIDQDYKSNVNFSIKYSRRISKMIGLWPTFDS-----TIHKYLRRLYNTICYSLLMFIIVLGWIYIAFEVKNIYDRLKFVSLMSFCMLSITKYHLINIHKDDVRECVKRMEWDWKNISYSQDREIMLMN-ASFGKRLIIVTTTVTYSGFVFFYIAVPMKI--GKIPAQD-AN--ISFIPTMFPFP-KYIADVRYSPINEIVFAFQFMCGFLVHGVTSSVCSLAAIFTVHTCGQIQVMMIWLEHLIEGR---------------------------------------------------------------------------------------------------------IDQRIAKIVSQHVRILKFLSLIEKILQQVSYMEFLECTVNVCLLGYCAIIESNHLTEVVTYVIILITIIFNIFVFCYIGELLADQSRKIGEVTYMIEWYRLSGKKKLCCVLIIAMSNSSMKLTAGNLIELSMSTFSDVVKTSFAFLNVLRTL-

>DpseOr56a-1__Ch3_residues_12782532_to_12786991_reverse_strand.[1501_2960].sp

------------------LRLHLRCFRLYGYVASK-Y-----QRRPWLSQARCILFTASIWMSCVLMLARVFQGYERLNDGATTCATALQYFTVSIATMNAIVRRERVVSMLREVHEDMQKLMKEADDQDLVLSTQKYTKTITLILWVSSIGAGLMCIYRTLFMPQTVFNLPAV-RRGE----ERPLLLFRLFPFGELY-DNFVVGFLCPWYALGLGVTTIPLWHTFIMCLMKYVHLKLMILNKRVPEM-----------------------------------------------------------------------------------------------------------------FTKFVTDHLKIRKFVKELELLICVPVMIDFIIFSILICFLFFALAVGSPTKMDYFFMCIYIFVMASILLIYHWHATLISECHDELSFAYYSTPWYEFERSAQRMILFMMIHSQRPLQIRAL-MIPVNLGTFLDIVRAAYSYSNLLRQI-

>DbipOr7a

-----------------CFRNLFNCFYALGMQAPDGP-----TRSSTWRRIYGCFSAVMYVWQLILVISYRYIEGMEMTQILTSIQVAIDAVILPAKIVALAWNLSLLRRAEYYLAKLDGRCKDAEEFH-MIAEAGRFCNRLVWFYQICYAIYSSSTFLCSFLL--------------------GQPPYALYLPLDWSRSVQFNIQAWIEFLIMNWTCLHQASDDVYAVIYLYVVRVQVQLLARRVRQLGSGDPPES----------------------------------------------------------------------------------------------DERRQEEHCQELQKCIVDHQTVLKLLGCISPVISRTIFVQFLITAAIMGTTMINIFIFANT-NTKISSIIYLMAVTLQTAPCCYQATSLMLDNEHLALAICQSRWLGQSARFRKMLLFYLHHAQQPITLTAMKLFPINLATYFSIAKFSFSLYTLIKGM-

>DpseOr94a__Ch2_residues_6066719_to_6068992_reverse_strand.[501_1774].sp

-----------HKERIESMHLILQVMRLFGLWPWSLS-----EGDHFVRRNYRFLLHLPITFTFIGLMWLEAFVSSNLEQAGQVLYMSITEMALVVKILSIWHHRSAAWQLMHDFQQAPELRLRSQEEQSFWQREQRYFKWFFYIYILISLGVVYSGCTGVLFLK------------------EYELPFAYYVPFEWHNERGYWFAYGYDMAGMTLTCISNITLDTLGCYFLFHISLLYRLLGMRLRALKSTAD--------------------------------------------------------------------------------------------------TADETYFGQELRQIFEMHQSVRRLTHTCQAIVSPYILSQIILSALIICFSGYRLQHVRANPGQFIAMLQFVSVMILQIYLPCYYGNEITVYAHQLTNEVYHTNWLHCRPPLRKLLNGYMEHLKKPVAIRAGNFFAVGLPIFVKTINNAYSFLALLLNV-

>HsOr354

-----------------------------------------------------------------------------------------------------------IKGLLEHLHSDWNVLHCEEEF-LIMKKYAETGRSYSFYYASIYSITFFIFGCTVLVSRFMDIVSPLN-ESR-----PIILPCPANYFVDEEE--YFYYIFVHLIIGAFVCITGLLAHDCNFFTFTEHVCGLFEIVGFRFEHLLYERNIKEK--------------------------------------------------------------------------------------NIKEKSLIEFSDNIYVKNIEFSIEAHRRALHFVALLENTFCLSFAVQLLIVTTGMSITLLYVK-LQGDMMEVVRFVPFVIGQLGHLLIYSYEGQKIINHSLELCQKIYNGLWYTIPVNSQRLLLFALRKTIEPSFVSAGKIYIFCLESFTSVLQCSLSYFTVLST--

>gi|383847311|ref|XP_003699298.1|

----------------------------------------------------------------------------------------------------------SFKILLDFIVQDWNVLTNDLLV---LDKVTKQGNKFATLYRLTFLMVFLYTYCIPLLPPTLDIIIPLN-DTRT----RRQLFAVNYIVMDVED--HFFIVYFHMTWTSIVCVLIVVTVDSLYMLVVHHASGLFDVCGYKV----------------------------------------------------------------------------------------------------------SRNHELNIEEFKKCVVAHHRALQFFDSLQECSQNMNLMLVGFNMTAISLTAIQIIMHFDRPADAMRFVLFLIAQNFHLFVISANGQILTNHAFLLPDKIFSSNWYEIPVKFQKLLYTMIIRCNRPCILSAGGLYDMNIENFGKTVKACMSYFTMFLSLR

>gi|380027252|ref|XP_003697343.1|

------------INPNKHLQNSLSIIYYVGLWPDR-------VKYKYLYNLYAICSLIFVGIIIVSEIIYIIINWGKIELMMIGLTILMTNSTYAAKVIYIICQYKRIKNLVDITNSEIFNRDNDKYKH-IISYYNWQGIFHYIAYQGFASISIFFYSCIPLQSV-----FSEK---------SKQLPLAGWYPYNVTSTPIFEITWLHQVLVILINCINNIAIDTLITGFIIITCCQLTILSYNISSIHYTVESIESS------------------------------------------------------------------------------------------------------------------------------------------------------------------------------------------------------------------------------------------------------------

>DpseOr63a__ChXR_group8_residues_3779517_to_3782247_forward_strand.[501_2231].sp

-------------RNYRSIREMIRLSYTVGFNLMRPR-----RWDVALRIWTVVLSLSSLLSLYGH-WQMFRHYVEDMPRIVETVSTALQVLTSVFKMWYFLFAHRRIYELLRQARLPVAQVLRRRV-AAIMQRYWGSTRRQLLIYLYSVIALTSNYFINSFARNLYRYLTQPP-GS----F-EIVLPLPALYPWEDK-GPYYHIQMYIETCALYICGMCAVSFDGVFIVVCLHGVGLMESLGEMI--------------------------------------------------------------------------------------------------------------------LRGCIYQYQRVASFAEEINDCFRHLTLSQFLLSLFGWGLALFQMSVGTSSAITMIRMTMYLTASGYQVAVYCYNGQRFATASEQIAGAFYGCEWYAECREFRQLIRMMLTRTGRCFRLDVSWFLAMSLPTLMSMVRTSGQYFLLLQNV-

>DereOr88a__scaffold_4770_residues_9481191_to_9483565_forward_strand.[501_1875].sp

----------------------------------------------------------------------------------QNPPVFSITIYFSIRGLMLFLKRREIVEFVNDLDRECPRDLVSQLDMQMDETYRNFWQRYRFI-RIYAHFGGPMFCVVPLALF----LLTHE--GKDTPVAQHEQLLGGWLPFGVRKKPYYLLVWFIDLMCTTCGVSFFITFDNLFNVMQGHLVMHLGHLARKFSAIDPRQSLTNENQ--------------------------------------------------------------------------------------------LTNENQFFADLRLLVQRQQFLNGLCRKYNDIFKVAFLVSNFVGAGSLCFYLFMLSE-TSDAVIIAQYILPTLVLVGFTFEICLRGTQLEEASEGLESSLRSQEWYLGSQRYRKFYLLWTQYCQRTQKLGAFGLIRVNMVHFTEIMQLAYRLFTFLKS--

>PbOr196JF

----------------------LRILEFLGVLSSTSS-----GRFIILKNVVWYLTFSNLIFQLIGETLYIY-HCQDTIMILKTIFVAACVTDAVLNLIICHTQRERLQHLLKEIENYLQNA-DENEI-NILQKHVDRYTLILVANTLLLSCAGLIICLRPLITK-------------------EYFPVDVWYPFMYSTSSRRCLIYISQIFSGIECVL-CFNTDISIAMFFCYSTAKLEVLQQKLQCTK-------------------------------------------------------------------------------------------------------------KDYIRKCIKQHQDIIRFVDLTQVAVQYLILLNVTMGTAAVCSL-FPLI-IDQPLVVKGQFIFTFLSACERFYISSWSATDLSEMSKLIAYSS--SSDDYISPRTVNDILLLIQRSQKPLTISMASFLPLSVEYFGNFLTSVLSYFMTMRA--

>LhOr81

---------------------LKIGLRLLGVWPDVS-----------YSTVYWVIYMSSLLIIQYFQYLYVFTHFKELSNLVDSLPMTLDYSLSIFKLTSLWLQRRIMQQILIAMDKDWRECMDVNQYLYVMTIKANVSHFFSNTILSFYGISGVFYVLGDYAIHIMHLVSD---NNDT----LRQLPMKVQLPFETEQSPIFEVLVVTLFLHVMANSFTIALLNGLIFSLVLHVSGQIDIICEEFRIISEKILL----------------------------------------------------------------------------------------------------------TLRTLIERHNKVILFSDNIEKLFSFIALMQVVWNTLVMCCLGFIIIISEGSLFVLIKTILAYIAMMIEAFIFCFAGEYLSLKSNAIAEAAYDVLWYNLPSNQSKIIIFVIMRSYRRLTITAGKIMDLSLETFASIMKASASYISVFHAM-

>8_385_Si_gnF.scaffold00330_12820-264637.pep

----------------------------------------------LITRLFWIVTTAFVEYCH-YLYFSTHLNSENFFNLVDCFCSFLAHAKVITKLVAFWVNQRKFEETLALITDDWSDYAKNDIGMRVMTGKAKVSDRITYIILILHTMTIVLYSMGIIIAD-------A---DVTETIE-LPFINKLVLPFSINTQHMYRFVLIAEFIHMMLSNFVAGVYNAILLAMVFHTGGQIDILQCWLAQLQP--------------------------------------------------------------------------------------------------KNIENKQKSIVVMANKIVLKHQKIIEFSENIESLYTYLAMLLFALNTLLICTIAFIIVTAADAMEQIIKCILFFTITNLEAFVFCYAGEYLSNKSREVGFATYNCEWYNLKSKDSQILLFIILRSQKQLTLTAGKIMDLTLQSFASIMNASGSYLSVILAM-

>gi|380025196|ref|XP_003696363.1|

-----------------------------------------------------------------------AIIAGDIESFLESFAPLAISLMCFVKYINFLYNFNQMKRLMDIMQEDWKFHAKLRDEYEILCEHYAIARKITTSFVAFLLGLITPFGAMPLLLNIGDALGLC---NISD---DRPLAFRVEYFVDVDK--YYYFLLIHSYIGTLGYTVIVLAINSMIIVYVLHECGLCEILRVKLENFVETDAMDVELRPNK----------------------------------------------------------------------------------------NKFKEDKWYQNARDCVLLHKRIIEFAKILEDANTTSYLLQLGFNMICISFTQFQAIINIQDTPKVLRYVSITISLLCDLLFISWTGQQLSNSTERIFQYTTNGKWYQSSINCRKLLTIMLSKSIKPLRLTACKLYTLNLESFTAIAKTSASYTMVLCSL-

>DvirOr42b__scaffold_12823_residues_1000155_to_1004473_forward_strand.[1501_2819].sp

----------------------YRAMKFIGWRPPK-A-----GPQRYLYFFWTLNTFLWSTIYLPLGFLGSYMTMITPGQFLTSLQVCINAYGSSVKVAVTYTKLSRLIKAKDLLDKLDSRCTKLEEREKIHLV-VSRANLVFAIYTFVYCVYAGSTYLTAVLS--------------------GRPPWQIYNPIDWHKNLNLWIVSTLEYVIMSGAVLQDQLSDAYPIIYALLLRTHLDILRERIRKLRTDNT----------------------------------------------------------------------------------------------RTDNTMTEDEHYEALVKCVIDHKMILDYCALIRPVISGTIFTQFLLIGLVLGLTLINVFFFSDSLPTAIASFIFVITTLLQTFPFCYLCNLIIDDCEALAHALFQSNWIDSGSRYQSTLLYFLHNVQQPIVFIAGGILPISMSSNISVAKFAFSVITITKQM-

>cplu_017206_b1

---------------------------------------------------------------------------------------------------------------------------------------------------------------------------------------------------------------------------------------------------------------------------------------------------------------------------------------------------------------------------------------------------------------------------------------------------------------------------PVNTRQYFNLIIIMSQQPQKISAGGVVDLSFVTYLQILKTGFAYLQLLRA--

>cplu_002078_b1

-------------------------------------------------------------------------------------------------------------------------------------------------------------------------VVD---SSETDIVEIPRLLVRSWYPFDARHGVAHIGMLIYQIYWLLVCTVDANSIDVLFCSWLLFACEQLQHLKAIMKPLMELSATLDTVVPNSGEASKS--------------------------------------------------------------------------------------------------------------------------------------------------------------------------------------------------------------------------------------------------

>DeugOr83a

----------------------------------------------LFYEFFNYFVSVHIAGLFIC-TIYINYGQGDLDFFVNCLIQTIIYLWTIMKLYFRRFRPGLLNAILANINENYEPRSAVGFSFVTMAGSYRMSKLWIKTYVYCCYIGTIFWLALPIAYR------------------DKSLPLACWYPFDYTQPVVYEVVFFLQAMGQIQVAASFASSSGLHMVLCVLISGQYDVLFCSLKNVLATTYVLMGANMAELRELQAEQS--VSDSQYAYSLEEQT---------------------------------------------------------------SKDFSTAFRQSFVHCIQHHRYIVAALKKMESFYSPIWFVKIGEVTFLMCLVAFVSTKSANSFMRMVSLGQYLLLVLYELFIICYFADIVFQNSQRCGEALWRSPWQRHLKAVRRDYLFFIMNSRRQFQLTAGKITNLNVERFRGTITTAFSFLTLLQKM-

>DsecOr65b__super_2_residues_6284685_to_6287117_forward_strand.[501_1933].sp

-----------------------------------------------YRSTWHTLVNIQIVIYFASMAFGLTESIGDHVQMGRDLAFILGAFFIMFKTYYFCWYGDELDQVISDLDALHPWAQKGPHPVEYQ--TGKRWYFVMAFFLATLWSFFLCIFLLLLITS-------P------MWVHQQNLPFHAAFPFQWHHPISHAMIYLFQSYFAVYCLTWLLCIEGLSVFIYAEITFGIEVLCLELRHIHRHN-------------------------------------------------------------------------------------------------------KELRMETNRLVKLHQKIVEILDRTNDVFHGTLIMQMGVNFSLVSLSVLEAVEARKDPKVVAQFAVLMLLALGHLSMWSYCGDLLSQKSLQISEAAYEAYDPTGSKDVYRDLCVIMTRGQKPMIMRANPFPSFNLINYSAILNQCYGILTFLLK--

>LhOr83

---------------------LRIGLRFLGLWPNE-----------SYPTLYIYMTSILIAQYFQYLYISTHFKFSEISNLVDGVMSTLFYSLMFLKLASLWIHRPVIHKILAAIDNDWRECINVEQHLYMMTNKANISHFYSNCMLGITIFAGILYGLGDYVIHVIHFIKDHN-ESG------RQLPLKVQLPFKTDQSPIFEFLFIILFLLMITTSLTMAIINATILSLVLHVSGQIDIMCQEFSIVSKQISIYNSSE------------------------------------------------------------------------------------------------------LKMLIKRHNRIIIFSDNIEKFFSFIALMQVVWNTLILCCIGIMIIISEAGIIALVKMSVSYFTVLLEIFILCFAGEYLSFQSESITDAAYNMLWYNMSSKQVKFIILIIMKSQSQLTITAGRFMNLSLETFTNIMKSSLSFMSVFHAM-

>gi|357621705|gb|EHJ73452.1|

---------------------------------------------------------------------------------------VIVGITVIFKHSILINCNDSIKQLLKILDEDYKAAELYGERKDIILKSAMSGVKICRFWLVSATLTCFMFPAKAIIEM-GNLYMR----GEFKLVPMFDFTYPNFIEVHKESTVAYTVLFLMCLSFDLFSLSIYIGFDPLVPIFMLHTCGQLELISNKLVNVFSKDASR--------------------------------------------------------------------------------------------------------------------------------------------------------------ISLEFIFFFSGACLHFYMPCYYSNLLMEKSESLRNAIYFSGWESRDIGIRKTILLMLTRTYVPLGIKTV-FYPICLDTFAEMVRQAYGIYNIMN---

>DmojOr67d_scaffold_6680_residues_20730840_to_20733105_reverse_strand.[516_1766].sp

-------AKTAVSRFATIIRLIRFCVKCCGTDVSN-------PNYRMWAWTYVTIVTIFAYFGFTGYTVYVAVQMREFATILQAIATAGSGAQGLVKLVCCVGKASLIRNIQHTYESMYREYEGRSGYTKYLHQRINNFYYLVLGFVFIYSTTVITMICFPLYELIV---LS-----------KKAMILHFLIPVDPNSDTGYVIMFVVHSMFLIVGAFGNFGGDMYLFLFIINIPLLKDIFSEKFKELNELVVQ---------------------------------------------------------------------------------------------------KYEEMQAVLWDLLTWHQKYATILRGTKEVYTYVMFVQLLSTCYGILFTICCIFMKNWPTA-----PFYLLYCFIVLYSFCGLGTIVETTNEQFTNEIYSCLWYEMPVREQKLIILMLAKSQDQRSLTAADMLPLSMTTALQLTKAIYSFGMMMIT--

>cplu_000774_b1

--------------------------------------------------------------------------------------------------------------------------------------------------------TEITWYLQPLTTP-----ASPI-ESDNETV-QFLLPYHFYVFYEMNGFHSYVLTYLSHGPHVVISGFGHMTSDCFLIILVFHLSGRLAVLTERINTLKEE-------------------------------------------------------------------------------------------------------------------------------------------------------------------------------------------------------------------------------------------------------------------

>LhOr82

--------------------PLKIGLRLLGVWPGV---------SHFVLYTYMSCILIVQYFQY--LYISTHLKISELSNLVDGLMSTLVYSLTFLKLFSLRIRCPVIHEILAAMDSDWHECIKFECHLYIMKNKANFSHFCSNCMLSLNVFAAIIYIVGNMAIHSSHIVE--D-YNST----WKQLPLKMQLPFKAEQSPIYEFLLVIQLLYIIINTSVMATFNGFISSLVLHISGQIDIMCQEFKIVSEQISVYQSSK----------------------------------------------------------------------------------------------------STLRMLIKRHKRIILFSYNIKKIFSFFALMQILWDALIICCLGIIIIITLHNEAMLMKGLLSYITILFEIFILCFAGEYLSIQSESIIDAAYGILWYNMPTKQSKIIIFIIMKSQSLLTLTAGKFMNLSLESFTNILKTSLSFTSVFN---

>McOr40PAR

-------------------------------------------------------------------------------------SYVLSMTSGFLLFTLFSFNITKMYMFLSEFKEFGKPPKFDKYNA-FLNKVAKFHVIYLNINITLFAAGSNVFKGAQCKK-------DNI-ELGYK--EICGLVTNTYLPFDIDYFPLKQIYVGLQFFSIYYVYTISGTITFMVMETMMHIGFRLDHVKQLFDEAISEKNV-----------------------------------------------------------------------------------------------ISEKNVERSRKRFNFAARYHARVLELEHEVNACFSYAMFSHMILTAAIIGCAAFGVMQSGS-----ANPFAVCIGWFNGISFVCLSDQHLINKSLEVGTAVYSSKWHQAHPSLQRDLVIVIMRCQKAMILRSAGFGVMNRATILAAVQASYSYITLL----

>DbiaOr46aB

---------------DDFYKYQVWYFQILGIWQLPTT-----DQQRRLQSMRFYLILVILCIMLLLFALELLNNISQVREILKVFFMFATEISCMTKLLHLKLESRKLGGLVEMMMSQDFAAKTEQER-SIMESARRAVVHMRNFYGITSWATASLILLVPCFAN------------------YEELPLAMFEWCSIQGRICYGMQYLFHSISLLPTCVLNITYDSVAFSLLCFLKVQLQMLVLRLEKLG---------------------------------------------------------------------------------------------------------NEKIARELRECAAYYNKIVQFKNLVELFIKVPGSVQLMCSVLVLVSNLYDMSTMNGDAIFMAKTCIYQLVMLWQIFIVCYASNDVTVQSSRLCHGIYSAQWTGWNRSNRRILLLMMQRFNSPMVLTFNPTFVFSLEAFGSIVNCSYSYFALLKRV-

>DgriOr24a__scaffold_15252_residues_12771501_to_12776159_reverse_strand.[1501_3159].sp

----------------HYFMVPKFALSLIGFYPEQ--------KRSWALLAWSFFNFSILTYGCYAEGAYGIQQIPNIGLALDALCPVASSILSLIKMLSVWYYRDEFKSLIDRIRQLTEEQHSDRKLG-YKKSYYTLATRLTALLLLCGTCTSTSYTVRHIFEN----MLRLS-HGK---VWIYETPFKMNFPDVFLRLPLYPWIYALVCWHGYITVACFVGADGFFLGFCLYLTVLLRSLRDDIKDLLQ-----------------------------------------------------------------------------------------------------VQEEKRIVREMVKLINRHNEIADLTERLSGVMVEITLAHFVTSSLIIGTSILDMLMFSG--VGIIVYLVYTLAVTTEIFLYCLGGTYIMEACLDLARSTFASHWYGHSVPVQKMTLLMIARAQRVLVVKI-PFFSPSMETLTSILRFTGSIIAL-----

>gi|284009968|dbj|BAI66619.1|

-------------------------------------------------------------LIVVGELYYMWMHMDEFLDLGHMIITTLLGILTVVRSIVLQSYHKLLLKFINVMHLMHSKNKGPYYNQ--MNDTDKICSYYTKFSLGLIFLSCSMFNFAPFCNNVANVFVFKT-ENYTL---EFSLYYQ--YPIDPTD--YFTTTSIYNFYLSYNCAMMVCGLDLILFLIIFQIIGHVYILRYNLENF-------------------------------------------------------------------------------FKLGDILKYKINESITFEMFG---AEENREVRFKLAECIEHHKEIIGFTDEVSALFGPILAFNYLFHLVCCSLLLLECS--EGGYSAMLRYGPMTVLIYGQLIQMSVIFEMLGSETEKLPDSAYFLPWECMDTSNRRAACIMLHKMQDKISLKALGLAAVGVSTMAGILKTTFSYY-------

>ApOr42

-----------------------------------------------GHNVFHVTVMVMIGFTVVCLPFGLYYWANDVTQCIFLLITIVNFSFGCFKAFTLVRHSDDICRCLDVTRFDFSSIMSDPDSARFFRKCRDASSTFTGWFAASSHFVLLVWTLLPFVVV-GKGVEINN-RDGSTSY-YHFNPYNMYFLVSSETYRLHLVFHLVEWAFGLCFVLIMVAFDTFMVTLCVAITCQMRGIGNAYSKLGHD--------------------------------------------------------------------------------------CATASNVCSDGGIESKSNNEYLRDLKLIIKDHQAVLGKMNDFYKIVGPVILPQLIVASFTIIFVSFIITRN-YFNGMLLTKMCCFPIFFYQIYYTCHAFGNLSHQKNVMNFALYSSDWTQMEIKFKKLLLLAMQMHANKLDMKLTDKLVINLELFTRVINMCYSIFSVLVN--

>cplu_007912_b1

------------------------------------------------------------------------------------------------------------------------------------------------------LLTTTAVILGPLILP-------------------QKFPTDAVYPFSVENRFVSRIVYLHQIIVGYQCSA-GMALDCQAAMYLWYLSARFEILSSEIRNVDSHN----------------------------------------------------------------------------------------------------------HNDLRNYIKKHQKTLMDAKELIRPTRLLALVTVMMTKIGMIFGGIVLI-SDEPVVIKIQFGILVISTTVNIYVCAWAADNLITVSTKLSNAIFETSW-RHEPKIRNLLLTVIYRTQKPIIIK------------------------------

>gi|226510881|gb|ACO59967.1|

-----------------------------------------------------------------------------------------------------------------------------------------------------------------------------------------------------------------------------------------------------------------------------------------------------------------------------------------------------------------------------------------------------------------------------------------------------------------------------KNRRIVAFFLMNVQEPVHVKALGLADVGVTSMTAILKTSMSYFAFLRSM-

>gi|340726442|ref|XP_003401567.1|

---------------DISITLSQFLLKLAGVWMTV-N-----NAEERRRRLTMAFTAVIHVYGLYLNLGDAYYTWNDLSHCTFLLSNTLCIVLAMFKLLILNFRRTEFKDLVLFAQQNFWHFKYDHDEKILFMKCRKLCKLWTITACSFTQASLAFYIITPICANIGK--------NKS----DRVLPFKMWVDLPLSVTPYYEIMFVIQLATVQQIGVTYLCSDNFLCILNMHVICQFRILHNRLLNLWKIIDQK------------------------------------------------------------------------------------------DQKTDKIDYADKCYIALKKCIRQHQLLIKFCEKLEYVNTLPIFGHVVVFSLLMCFDTYEILLANVSTGTRLIFVFHMVGSFIHIIFFTYTCHGLIEESSNISLATYSGWWTILPMMLREDVKVMMMKSMRPCCLTAGGFFPISLETSTALMSTTMSYFTLMR---

>CpluG2R504O03DDZ9O

----------------------------------------------------------------------------------------------------------------------------------------------------------------------------------------------SQVXFSVETHPNYEIIYVHQAFAGILCSS-IGSIDCQIAMLLWYSIARLDILSLEMKKINN--------------------------------------------------------------------------------------------------------------------------------------------------------------------------------------------------------------------------------------------------------------------

>DgriOr49a-1_scaffold_15245_residues_4301908_to_4306155_reverse_strand.[1501_2863].sp

----------ELRTYDDFIYVPNLIFKSMGYDFLDTP-----RWQKLMLNIYWTLCVTSHWYMVYYLILRTIELAGSADAIMRFAIIYFIVLNADVKFSLFMHHRRHLRELNDKLRALYPKEGKTRKEYRVN---EFYWPFIARCEIYIYYFVVGVVVLGPIIQAILMYLYQRY-TSDS----EVIFLYLSTFPMEVTSPMTYALSQCIEFIFSHSTMNIKLGTDIWMICFSGQLCMHFAHLGRKLA---------------------------------------------------------------------------------------------------------RQNHYEDCEFLVRLIRDHEQLLSLYKELNKIFGLMLAYNVFSTATILCCVAYYTIL-QGVTREGFGFLLYFFSDSVNFYMVCYYGQRLIDLSENIALAAYIHNWYNGSPTYQKYVLMIIQRAQRPAELSAKGVIIISRDSFKNMMSITYRFLAVVRR--

>LhOr175NTE

-----------------AIELCRFGLKVTGLWPENHT-----NKNNFISNIHATIVFIMITFVVIPLICSLVRIWGNMILMIDNLQVTITLTAIWAKFVIMRWKRSAISSIVNMIAEDWMYLKINAERNVMIR-QAQIARFVIISGYFMVIFGFSVVIILPSFGVHYR----LL-TNLTAG--VRALPMQGYYFYDTDKSPQFEFTLAAQAITTFLACVTYTSVDAFLALIIFHICGQLENFRYQLLNLASSDNF------------------------------------------------------------------------------------------------NLASSDNFANALRYNVETHLRLIRFADNIENVYAVLMLTLLFYFSSVFCLYSYPLVTVNKSFSRIGFSIIGVIMLLAHTFFYCGGGQLMAKECEAVYRALCDLEWYKWEPRESRALILLMIRASEPFRLTAGKIFPLSMVTFCSVLKTSAGYISFLLA--

>CpluG2R504O04EGOOH

-----------------YYKLAKSFASFIGQWPYQ---------SRFQRLVLEFLLWNLFIIQVVPQVVSLVDNFRDLDVVLELLSAFII-------------------------------------------------------------------------------------------------------------------------------------------------------------------------------------------------------------------------------------------------------------------------------------------------------------------------------------------------------------------------------------------------------------------------------------

>gi|383850714|ref|XP_003700921.1|

-------NTRYLQDYEYSIQLNRWFLKPIGVWPRAST-----ASEKILAIVLNVACHVLIIFTFVPCLLFILFEEASFQTRIKAIGPMSHWIMGELNYCFLTMRGQDILHCIEHIGSDWQTVKRASDREVMLK-NVKFGRLIACLAAVGMNVGVFSYIVIGFRK-----IVFHV-GNESFL--MYRLPCPVYTKLNVKLSPANEIVFALQVLSGFIVNSVTVGACGLAAILAMHACGQLNVMMSRMDELV-------------------------------------------------------------------------------------------------------EEQRVVKKKLADIVEHHLRTLSFVWYIEKIMNLICLVELVGCTVNICVLEYYIIT-EKSKDTLASYAIIYASMSFNIFIFCYIGERLTEQCKKVGEKAYSVEWYRLSHRTALSLVMIISRSSMVIKITAGKIIRISLATFGDVLKTSFTYFNMLRT--

>RprolixusORCO

------MQKVKMHGLVGDLWPNIRLMQMTGHWLLEYG-----GMVRLMRMAFCWFTTLIVFTQFAFITCFLILETYDADQMAAATITTLFFLHSVTKFMYFALRSKYFYRTLSAWNQVNSHPLFAESNARHRATALARMRKLLMIIGSGTIFSVLAWTTVTFLDDPHRQITDPE-ANETITVEKPMLMVDAWYPWDAKYGMTYFMSFIYQLYWLFISLSHANLLDILFCSFVLFACEQLKHLKEILQPLMELSAALDSVVPNSGDLFRASS----------------------------------------------------------------------------PNGLTKKQELMVRSAIKYWVERHKHVVRFVTSIGDCYGSALLLHMLTSTVTLTLLAYQATKVEGVDVYASTVIGYLVYTLGQVFVFCIHGNELIEESSSVMEAAYSCHWYDGSEEAKTFVQIVCQQCQKSLTVSGAKFFTVSLDLFASVLGAVVTYFMVLVQLK

>DbipOr2a

-------------DTHRAVRYHWRVWELTGLMQPT-------GISRFWYFIYSLVINASVTILFPLSLVARLFFTHNMQNMCENLTITITDIVANLKFLNVFLVRRQIRSLLKHLDERARQIHHPEELA-ALNEAVTIAQKGFQYFARIFTFGTILSCIRVAISS------------------KRQLLYPAWFGVDWENSWAYVICYAYQLFGLVVQAVQNCASDSYPPAYLCLLTGHMRALELRVRRIGYGRGRLNST--------------------------------------------------------------------------------------------------STHEELLDCIRDLMLVHQLKNIIQRILSVACMAQFACSAAVQCTVAMHFLYVDNDLSAMILSIVFFVAVTLEVFIICYFGERMRTQSEALCDGFYACNWVDQLPVFKRDLIFTLARTQKPSLIYAGGYIPLSLETFEQVMRFTYSAFTLLLRA-

>LhOr219IX

-------------TLESVIVYIKWSLFPSLSWPLPIT-----KWQIIRFKILAVLSHINVVCLLVPLLIFIQDCSGQRVICIQTYPFICGCFHFWTSVGVCHIQYKRFQSLIAEMENYCKHATDYEK--TVLQQYVDRCATFYAMVMISYYSTACVAISTPLFSS-------------------ASFPTYAKYPFDVNYQPLKTIIYAQQSLAGLQFSS-MLCVSLLVALLLWFTTARFDILCNELRKA----------------------------------------------------------------------------------------------------------------ELIQCIQKHQHLLRYANDVIENVRFVVLSVVGTNAVSVICTGAIFAT-RQPLIINLEFFMMTAMSFCDVFACCWPADSLLTASSDIAQATYESLWYYHNTDKQKNLIFILLKCRKPVILTVGCFIPLSLRYFSSYVSTGFSYMTTLH---

>ApOr47

--------------NEKHHVFNIRLANLIGLYQTLDP-----ETVKFRRNVYQIFVAFVALYLLVISFAGCLHLWTNTATSLLDLLITTNSFYASYKMWIVVYRSNEIWDCLSITRYGFTSLNNRKWNHDILDRWRARSVRYTSLLAGAYFLTIVFYVGCPLVFGAA--VIPI--KNQDGSIGYRLNVINLYLFVDETYNEYYNTFFFIEALFIVGLIITCLLFDTLLLTLCLGICCQIQMICSAFESVNHNSPS-------------------------------------------------------------------------------------SAIDNNDEKQIISNEHDLIHDELITIIINHQAVIKKFELFLTIFDRVMLSHIFVSSISLIILWFNLIMSSGD--TTLKTIVAIPSFLFQIFMVCYLFEDIHNQKDSIVYALYSSNWTEMDMKCKRLILLTMQLNQKKLRFTRTKI--VNLEMFFKTTGHCYTVVSVLMN--

>350_Si_gnF.scaffold04648_643308-783672.pep

------------------MKFTLTVLAVAGCWRPT-S-----WTSLFRYSMYNAYTALIILILYTFATMELI-VNADSDTFGDAFFNVVISLLACYKAIILRRNHDGITILIDSLVKKPFKPMDMSENM-IRQKFDKRITNNTLCYLILVFITAVYMIILSLFTDFK----------------NGILMYKAWLPFDCSISVLFYFAYAHQILSLICIGLVHPTCDNLICGLLLHICCQLEILEYRLSNIANEQKN--------------------------------------------------------------------------------------------------------QKNLRDCVDHHIHILQYVYTVNNMFAKIIAIQFAVSMLVVCSNLYRIAM-ATDYMSFIPLMMYTSAILVQIFILCWFGNEVKLKSLQLVNSIFDIEWPALSNSNKRNLLLIMKRAMTPVEFTSAYIITMNLDSFV-----------------

>gi|388429181|gb|AFK30399.1|

----------------RYMKMLRNLLHLISSWPYKLE------DVKPLPLRGTFYLFVEIVLVTGLIYVKTHINKLSFFEMGNTYVTVSLNVVGLQRITIFWFYRQAIKEFVLEVHLFHHRHKTEYSEHIYQYI-YKICAVFVVAIHAETFFGVLLFNVMPFVNNVRHGMFNEE-MPPDR---QFEHSINYSLPFNYHTDVGYIVIAIVNLILSYDCLLAFCGFDLALSVIVFHVWGHLKILDHDLRTF------------------------------------------------------------------------------------------------EDEMSYTKEENQRVRAMLKDIIDHHRHIMHFMTQASDAFGPMLCVYYMFHQVSGCILLLECS--KMDPESIGRYAALTVTLNQLLVQLSVIVELLGTQSETLKDAVYSMPWECMDTSNRRTVLFLLYNVQEPIRLKPMGVVTVGVTTMASILKTSFSYFMFLRTF-

>DsecOr67d_super_0_residues_2480503_to_2482908_forward_strand.[501_1906].sp

---------------CKVIRMIRFCVGFCGNDVAE-------PNFRMWWLTYAVMAAIAFFFACTGYTIYVVVINGDLTIILQALAMVGSAVQGLTKLLVTANNACHMREVQNTYEEIYREYGSKDEYAKCLEKRIRITWTLLIGFMLVYIILLGLVITFPIFYLL---ILH-----------QKVLVMQFLIPFDHTTDGGHLILTAAHVILITFGGFGNYGGDMYLFLFVTHVPLIKDIFFVKLTEFNEL----------------------------------------------------------------------------------------------------------VRAILCDLLAWHQLYTRMLQTTKKIYSIVLFVQLSTTCVGLLCTISCIFMKAWPAA-----PLYLLYAAITLYTFCGLGTLVENSNEDFLSVIYNCLWYELPVKEEKLIILMLAKAQNEVVLTAADMAPLSMNTALQLTKGIYSFSMMLMN--

>gi|170037997|ref|XP_001846840.1|

--------------------------------------------------------------------------------------------------------------------------------------------------------------------------------------------------------------------------------------------------------------------------------------------------------------------------------------------------------------------------------------------------------------------------TSNSTAIFFTSFSIIKRYQEHVNCFLGTILAVKSEQLQLAIYEVPWYKLSLPNQRSMQILLHASQKPVCLTLI-FYPIDMPTFLQMYKTIYSIFTMLLTVR

>DsimOr59a____chr2R_residues_17906415_to_17910609_reverse_strand.[1501_2695].sp

----------------EFFKSHWTAWRYLGVAHIRVE-----NWKN-LYVFYSILSNLLVTLCYPVHLGMSLFRNRTLTEDILNLTTFATCTACSVKCLLYAYNIKDVLEMERLLRLLDERVVGPEQRSIYGQV-RVQLRNVLYVFIGIYMPCALFAELSFLFKE------------------ERGLMYPAWFPFDWLHSRNYYIANAYQIVGITFQLLQNYVSDCFPAVVLCLISSHIKMLYKRFEKVG------------------------------------------------------------------------------------------------------------AEKDLEACITDHKHILELFRRIEAFISLPMLIQFTVTALNVCIGIAALVFFVSEPMARMYFIFYSLAMPLQIFPSCFFGTDNEYWFGRLHYAAFSCNWHTQNRSFKRKMMLFVEQSLKKSTAVAGGMMRIHLDTFFSTLKGAYSLFTIIIRMR

>ApOr45

-------------------IFNQKLAKIFGFYQILDT-----KTVTFLHNVYYRIFVFLIVYECLLSAIVILYNNNNIVQAMFYFGFVVNMLYGSYKMYIILSRSKVIWDCLSITKFDFTSYGVQGRH--TLNVWRNLSIKYTNIYAIFYLTISILCVASPVVFSNSFIIIKNH-DGLSNA--YRLGLINLYLFVSEETYAYFYVFHIVESLGLVINTLFIIIFDTIVNTLAFALIGQLQTISTAFESVGHKSLHFPN----------------------------------------------------------------------------------NNIDNKNKLPNENIKYMDHYKDLKTLIIDHQNILKKYDEFLSIFRPTMLLQVFVVSSSIIFLWFIFLTSEDDFTQYMAAIFGIPFCTFQMYMSCFVFNTLNIKKDSITFALYSSNWTEMDMKFKKLILLTMMNNAHHQKLQYTRTRIINMEIFFQTMRVCYTIVNVMISCK

>DmojOr22aL1___scaffold_6500_residues_3317700_to_3319300_forward_strand.[146_1447].sp

---------------RDSFVYIDRGMSLVGWMPPK---------HPCGRYIYRLWSAFTTLFIIILLDVSLLISYSTARQFLTSLQVAFNCFGCSIKASYTFAGVRRLKLAKKILDRLDSRCETSEHR-LQLQQTATLCNRIFMTYCAMYLLYSSSTFLGSMFF--------------------GRLAWNLYNPVDAEEGFSFWLAGILEFLWMTGAVMQDLMADVYPIVYFLALRAHIKMLKERLRNLRMDTNMSEEE---------------------------------------------------------------------------------------------NMSEEENYKQLIRCIENHRLILDYCNTLRPVVSATIFVQFVLIGVVMGLSAINVIFFSNFWTGLG-TGIFMFDLILQTFPFCYICDMIYNDCYEMANCLFHSNWLTAGRQYKTTLCFFLCNVQKPIIFTAGGIFVISTSSNIAVAKLAFSVVTFVRQL-

>PbOr369FNC

---------------------------------------------------------------------SIFMILGNVQVIFETSHHAIGMTMFIVKLFNEFWNRDKIQHLYEIMENHWNIFTNEFEI-NILKDYSIISRKFSIFYSTIISSMAAIFILVPFKPILLD-IVRPL--NE-----SRPRVFALSIKWRIDKDKYFVPLFCYNISAIVAGIIILTGTDSVYVTRIVHASSLFSIIRQQFEKITSKLVINEETNKH----------------------------------------------------------------------------------NKHGYYFKLTYEQMIYQEYIICIKKYQLALQFVDLLNSIYQTAVLILLFQITVIISLIGIRIVHCLDQLEEAIRYSFMMIGVLLQLLLLCYFGQMLIDESQNVFY--------------------------------------------------------------

>gi|167863513|gb|EDS26896.1|

----------------------------------------------------SYVLLLQMAVYFYSNFWTAYHYRDDIIMGMKVLNCTGIAIQLSAKFFIAVFRKKDFLALCVLIENELYQASTAEPEGQILYDYAKKYHILLRVIMFLYFASLFVFGLYPLYIF-----LDQG---------EIIPLFMFEIPYDWHTTGGLIATYAVQVITYVTGIFGIILADGLFIHYVVHAIVFMEIFEIHLHQLGIMLQNQD--------------------------------------------------------------------------------------------------------------------------------------------------------------------------------------------------------------------------------------------------------------

>DanaOr2a__scaffold_12929_residues_2038800_to_2041004_reverse_strand.[501_1705].sp

-----------QLDTHRAVRYHWRVWELTGLMQPD-------GISRLWYLAYSLALNASVTILFPLSLMARLLFTHNMQNLCENLTITITDIVANLKFLNVFLVRRKIRSLLKHLDERARQVNHPEELA-ALNEAVTTAQKGFQYFARIFTFGTILSCVRVAISS------------------KRQLLYPAWFGVDWENSWAYVICYGYQLFGLIVQAVQNCASDSYPPAYLCLLTGHMRALELRVRRIGYGTRR---------------------------------------------------------------------------------------------------------GELLDCIRDLMLVHRLKGIIQRILSVACMAQFACSAAVQCTVAMHFLYVDNDLSAMILSIVFFVAVTLEVFIICYFGERMRTQSEALCDGFYACNWVDQRPIFKRNLIFTLARTQKPSLIYAGGYIPLSLETFEQLMRFTYSAFTLLLRA-

>HarmOR13

----------------FYINLARKSMWILDSWPKTP----------NESVTYRYFVLALNVATLVGGAVYLRNNTGSSFELGHTYITVFMNCITCSRCIMILSYNEVMLSFVNKIHLFHHRHKSEYAYKTHIFI-HKISHFYTVYLLGLALNGLLLFNMIPFYNCYSRGMFRDV-IPANAT---YDHSVFYSVPFDYTTKKGYIAMTSFNCFISYTCTSYFCVVDLTVSLVIFHLWGHMRLLTYHLANFKKPASVLESNEN----------------------------------------------------------------------------------DAIKDHSYTQEELKEVFGKLREYIRHHNLILNFSSEMSNAFGPALLAYMVFHQVSGCILLLECSQ--LDMKTLVRYGPLTVVIFQQLIQISVIFELLGSSNDKLIDAVYLVPWEYMDTKNRKLVFVMLRQSQRSIDLKMMSMLTVGVQTMTAILKTSFSYFVMLKTV-

>HarmOR47p

-------------------------------------------------------------FYNFLLTLNLVYTPRSLELFLRELMFYFTEMPVTSKFLTVLLLRDEILEIFNFIDCDEFVGDYENKD-GMLYKTNMRYRLVWKLYFVLSHVAFTCDIILPIVFD----------------------------------------------------------------------------------------------------------------------------------------------------------------------------------------------------------------------------------------------------------------------------------------------------------------------------------------------------------

>DpseOr67c_ChXR_group6_residues_8631683_to_8634063_reverse_strand.[501_1881].sp

-------EPDTARTFKDMMRVPVQFYRTIGEDIYAHS-----PWRSLLLKVYLYGGFINFNLLVIGELVFFYKSIQDFETVIAVAPCIGFSLVSDFKQFAMAYYKGTLVRLLDELEEMHPKTLERQRA-YRMPDFERTMKRVISIFTFLCLAYTTTFSFYPALKASVK--FNLL-GYE--TF-DRNFGFLIWFPFDATSSLVYWIVYWDIAHGAYLAGIAFLCADLLLIVVITQICMHFDYVSRRLEE----------------------------------------------------------------------------------------------------------------EFLVWIVRYHNKCLTLCEHVNNLYSFSLLLNFLMASMQICFIAFQVT--ESTVEVIIIYCIFLMTSMVQVFLVCYYGDTLIATSLRVGDAAYNQKWFQCSKTYCQMLKMLIMRSQRPASIRPPTFPPISLVTYMKVISMSYQFFALLRT--

>NvOr97CTE

----------------HTIRLPFTILKYIGIWKPN-S-----WKSTWKGYQYDCWSLAVMMIMYSFVATELIAVSDHYDEISDTLFLLLTTAGVSIKSMNFLANRDNMAHLGDMLLKSCCIPKNLNELKIQ-KKFDDVNRFVTLMCITMVYGNIMTMMTIPLLQS-----------K-----EHRNLPFKTWLPYDTRSNLNYWLSYLHQGLGLACCGTMGVMIVNIITGFMQQACAQFEILDSRWRNLPKIVE-----------------------------------------------------------------------------------------------------------MLSQYIRHHIHVYEYMNEFVKTFNIIILVQFCVSSVVITISVYQMS-IKSLGLEWFMVFGYAVSMLTEFFLYCWFGNEVTLKSMDFALKIYDTEWNLLNIKSWKLILFVTHRTRKPIVVRCYNYIVLSLDTYVN----------------

>DsecOr33b__super_16_residues_133896_to_138199_forward_strand.[1501_2804].sp

------------------------------------L-----ESNFFLNRLLDLVITVFVTIWYPIHLILGLFMDRSLGDVCKGLPITAACFFASFKFICFRIKLSEIKEIEISFKELDQRALSQEECEFFNQNTRREANFIWKSFIVAYGLSNISAIASVLFAG------------------GHKLLYPAWFPYDVQATLIFWLSVTYQIAGVSLAILQNLANDSYPPMTFCVVAGHVRLLAMRLSRIGQGPEETKYSTGKQ-------------------------------------------------------------------------------------------TKYSTGKQLIESIEDHRKLMKIVELLRSTMNISQLGQFISSGVNISITLVNILFFADNKFAVTYYGVYFLSMVLEFFPCCYYGTLISVEMNQLTYAIYSSNWMSMDRSYSRILLIFMQLTLAEVQIKACGMIGIGMNAFFATVRLAYSFFTLAMSLR

>gi|167873238|gb|EDS36621.1|

---------KSYEMYDYNLLFIRWMADFSGVDIMV---------ENYRFNYRTGLCVAVIWMCAINATYSLVYYYPDYYKMCEVVLLFGILIQGIPKLYFGYIHREFYKLQYGRLRRLHYKYRDHEKLNANLLLLMERIHVLSKLLAIVFIFGGLGYSIYPMYVY--------W-ANN-----ERVLMIAMRLLWDADSYTGYIVTSAVQMVMIVITCTGLSAADTVILLFVANLIAYVDVFTNELDELNAM----------------------------------------------------------------------------------------------AMLNEEIRDETKIRQQVRTICTQHQDIIEYESDLDKRYIVISFAQIIGSVTTLSGYLFLVYM-----VNFIPGYGLILATICQLLEFCLLGTVLTVKNEEIIAAIYNAHWYRLQRPELACFALMLHKSQNFIEMTVGGFAPLNLETFVAIMNRIYSYFMMLISF-

>DmirOr22a

----------------DAFVYLDRVQKLWGWRATEDE-----RWMVLYNIWAFSWNVLLLVLLPMSMSMEYVQRFKNFGEFFGSLEICVDMYGCSLKCVYTMFGYKRFQAARKLLDRLDLRCTSDEERASVHR-SVALANRCYVTYHILYSGFVVINWTGYLLL--------------------GSHAWRMYLPLDSER--NFLVTSFFELLLMSGVVTMNQCTDVSPLAHMIMARCHMGLLKDRLTKLHSDSSKTEEEH------------------------------------------------------------------------------------------DSSKTEEEHQEDLNRCIHDHCVILEYVNLLRPVYSVTIFVQFLLIGLVLGLSMIHIMFFSNFWTGIG-TMCFMFDVCLETFPFCYLCNIIIEDCRELSESLFQSDWLGASRKYKSTLVYFLHNLQQPIILTAGGVFPICMQTNLSMVKLAFSVVTVIKQF-

>AmOr136

--------------FDKHYHTYRTLMKIVGLWPYN---------NSIYVWIQRLWFLMFFFGNIIFQIMSLLTSAITLQNCVLIFSTTCPLIIVLFRYIGLILFFPTIKLLFHHMCMEEAMIQDSIEA-QIRRKYIDDSCYMIDIFFWMTYVGIALCSILLLCPITLDFIMPLN-ES-------RTRIVHYVTIFSDKSIIYMDILCLNYMLLAILVILSATCTESILGLYSYHTSIMFKIIGHRIQKIVKYLTMFNLSSKQIDSKLAE----------------------------------------------------------------------------------------------------------------------------------------AIVIKKDELEILISFIFFTTQLVITFLNNNCNQILIDNSQELFIELYISMWYFVPLKVQKILLLIMIRSSTACMINILGVFTPCYIGFSKMLSTSFSYFTLMHSI-

>DmirOr22c

----------------HFYRIPRISGLIVGIWPQH--------PRPCHAQILFIFALAVVLMGAVGEILYGFVHLNNLVIALEAFCPGTTKAVCVLKLCVFFGSHRRWHEWVHRLRGMLWSNRQEKGQQMLV-GLSTIANRLSLLLLCSGTMTNTAFNLQPLIMGLYRWMSQLP---G-----QIELPFNIMLPTFVVQQPLFPFTYVLLTASGACTVFAFSFVDGFFLCSCMYICGGFRLVQQDIRRVFAD--------------------------------------------------------------------------------------------------FTESMNVDIRQRLGVIVERHNAIIDLCTDLTRQFTVIVLMHFLSAAFVLCSTILDIML-NTSSLSGLTYICYTVAALTQLFLYCYGGNHVSESSAAVADVLYDIEWYKCDARTRKMILMILRRSQRAKTIAV-PFFTPS----------------------

>NvOr8

-----------AEDFEYAIQILRWLFQPMGIWPLKSA-----AYPSFLRPISIVISFWSAAFLIIPGILSVIRVQNDFALRLRLIGPVSFCLVTSFKYFSFLVKNRQFYAYLINVALDWREMKKNNHNRIIMLRKTQISRFFMTSCSICMYLSGMSYNILLPLTK----APTQV-GNVT----FKNLPYIGYYIFDQYADPYYYVVFVMQCMSSFFCYSTCCGVCCISIQSVLHISGRCDITSIMIKNLNG------------------------------------------------------------------------------------------------------------EKALKAVVEFQLQSLKFAREIEKLLNQMFLVEFVGSTFNICLLVYYFMGDENDTVGTMTYVLLFISFTFNIFIFCYLGEHLTEQCASAGAAVYTMDWFRFSAKKSRDLFLIVLFCQRPVVITAGKMVNFSLLSFASLMKASAAYLNMLYKM-

>NvOr9

---------KNAQGYAYAVQLTRWLLLPLGLWPTKSI-----IYQKILRPVAILLCLFIMLFVIIPLCLFIFLVVKDLGIRLKLIGPLGFGLMSLFKYVVVIVKQRDVASCFLGMAVDWQELSSLSDRKV-MLRNAKTGRLLTIICVIFMIFGGMPYITVLPLTK------GPI-MRGNVS--LRPLAYPSYFVFNPQIRPIWDYVFVTHCMCGLVRYSVTCGVYSIAILCIMHICSQITITSSMLDRLVEN-------------------------------------------------------------------------------------------------------------LLGKIVTQHLRFLKFASKLEDLFNQICLVEVLGSTCIICFLGYYLITEQREPIATVTYFLLLCSFVFNIFILCYIGEILTEQCESIGTTAYMIRWYHLSGKEARNVVLIIASTQRPVVMTAGKMVNLSLQSFTNVIKASASYLNMLRTV-

>PbOr317FI

------------------------FLLSLGLWSND---------STCFKQIRVIFCILMFIFLIIAQLLKLFTMEYDLDFILADLSFAIPSLAYFLKYSTFYIQSHKIRQLMEYIRNDWNALQNEQEIN-IIRKYAVSAKRYMYAFVGVSYPGTIGFLLISLLPDILDIIVPLN-ESRT-----RHLPFMVEYFLNEQK--YFYLLFLHIAVTVILGITTVIATEALMLAYVYHICGMFKVIRYRIERALNASTS--------------------------------------------------------------------------------------------------------------------------------------------------------------------------------------------------HVYISQWYEAPLHAQKLLLFMMQQSIKGTAMSVGGIFVPSLEGFATLTSMSVSYFTVICSIK

>PbOr228PSE

----------------------------------------------------------------------------------------------------------------------WKENKDSNLIIRFVSFYXXEARRIAFMIISQMMGLLVLTVLLM--AQ------------------EKQLPLKMYVPYFMTTLLPYILIYLEQAVAVFYGVLLNVALDSLVYDLIIHTCGQ---LCYZLTEAFRYLZENNEEKK-------------------------------------------------------------------------------------------------------AIAEHHISVCNITYRIQFLFMWIIATLFFFSLVTLCSSIYQIXNKDLFTVEFFAFMLYLGSMLFQVFXYCWFGNELDLKYKSITHAIYASDWTVISINQRKSLSFVMMMSQRGRIIXFYGVCSLLLSTFTXIIKTPYSAFNF-----

>H_TcOr14

-----------------VIEFNLFLLKWIGLWPGED----------YQLNMYSFYGFSVIILILCGHGLTLILDSGDIDTFTETMFILNIEFMTAWKALNFALNRKKFMQLLDAIDKTTFQPRNGKQV-TLVLRNIDGWKVMFKMFGISLGLSFIFTGLLPIFSKTYK---------------DRKLPMEAWYPFDSTKSPFYQLCYVYQMAAVAVAVMVILNVDTLVAAMNICIGLQCDLLCDNLRNLHTNTSKSMN-------------------------------------------------------------------------------------------NLHTNTSKSMNQKLIECIKHHQNIISFAEKFRQAFNWSIFLQFFVSTTSLGIVMFKITRFSLYVSEYYRFISYACSVLVQVFIYCWFGNEVIVKSSKIPYALFESDWTQDSLEMKKNMIVFILRTQKTLKITVCHVFDLSLPTFLTILKTGWSYFAFMNR--

>H_TcOr15

----------------SVIKINIFLLKWVGLWPGE----------KYQLNVYSFYAFTVIILILCGQGLTLILGSGDVDTFTETLFVVNIEFMTAWKALNFALNRKKFIQLLNAIDKPMFQPRNDKQV-TLVLRNIDGWRVMFKMFAISLALSLIFTGLLPIFTKTYK---------------QRKFPYEAWYPFDSSKFPIYQLCYMYQMASASTLVVVILNVDTLVAAMNICIGLQCDLLCDNLRNLHFDTSKSMN-------------------------------------------------------------------------------------------------SKSMNQKLIECIKHHKSIIRFAEKFRQAFNWSIFLQFFISSTSLAIVMFKISRTTNYGSEYYRFISFACSVLVQVFIYCWFGNEVIVKSEKIPYALFECDWTPEPLEVKRSMIIFIIRTQRILKITVSYMFDLSLPTFLSILKTGWSYFAFMNQV-

>NvOr6

---------ENDRGFNYAVKLTRLLMISCGIWPAKFT-----SFQKCLRPILIIICFFIMFFQLIPFCLFMFLIIKDMRIRLKLLGPLGFSLTSLFKYVVVVIKNREIAKCVQIMVDDWHQLNSTEDRKA-MLINAKTGRVLTMVCMFLMYGGGMPYVTIVPLTK----GVTMV-GNVS----YRHLAYPSYYIFNPHVRPIYDVIFATHCICGFTRYTITCAVYSIVIICVMHICSRIAITSSMLQRLADDS-------------------------------------------------------------------------------------------------------------LGTAVKHHLDILKFATKLENIFKEIFLAEVLGSTYQICLLGYYFITEQRAGIATATYLFLFMSFVFNIFILCYIGQILTEQCESIATTAYTSKWYQLSGREARSIILIVHWNRRRVVLTAGKMLTLSLESFSSIVKAAGGYLNILRT--

>NvOr7

-------NLERNQGFDYAVQLTRLLLMPCGIWPAKFS-----RVQRFLRPFLIVACCFVMLFLLVPVCLFMFLIVRDVRIRIKLLGPLGFSLMSLFKYAVVIIRSREIEKCIQNMLDDWQQVASDEDRD-TMFENARTGRVLTMVCMFLMYGGGMPYVTIVPLAK----GATMV-GNVS----YRALAYPSYFIFNPYIRPVYDVVFLTQCLCGFTRYTITCGVYSVVIICVMHICSRITVTSSMLQRLADN------------------------------------------------------------------------------------------------------------KLMGTVVKHHLKFLNFAAKLDNIFREIFLVEVMGSTGVICLLGYYFITEQRESIATITYFFLLMSFVFNIFILCYIGQVLTEKCESIAKAAYTTKWYQLTGKEARSIVFIVSCNHRPVELTAGKLLKLSLNSFSSIIKAAAGYLNILRT--

>H_TcOr10

---------------EATLSQNINFLKVCGLWPPGDE-----AYKFNLYGIYAGFCVLGLCVHTGTQTFNVYFILDDLEAFTSSIFVTFSCVACVFKTYYLLKNMKLLKVLFININKEIFQPKNKEQQ-LLIQPSILFWKRFYLVFRILCYNTCFFWCAYPILDKR---------------IKQHKLPFLAWYPFDSSVSPLYEITYFYQAVAIWYIVIISFNIDMLIGALNMFVGAQCDILCDNLRNLGKSDI------------------------------------------------------------------------------------------------------NELNPDLIKCIQHHKAILSFVSKLNIFFNWIVLLQFFSSAVSVGFTMFELTLVAPFSGQFYSFICYGSAITTEMFIYCWFGNEIEIKSSKIPYAAFECNWVGTPLGVQKSLIIFTIRTQRPMQVSALNLFYLSLDTFKTVLRTSWSYFTVLNQV-

>NvOr2

------VNEQYLADTEYVVRVAKTLLMPIGIWPRYGS-----TLSNAIIYIRVCLIFCLMLFLLTPHFIWTWFKAEDLRKLMKIIAAQVFSSLAVLKFWTLILNKQDIRYCLEIMENDYRVVESEEDRQIMLK-NAKIGRFFTTAYLGLSYGGALPYIIMPLLQP---RVLRSD--NTT----MIPLPYPSEYVFIVEDSPLYEIVFVTQILISSIILSTNTGVYSLIACVVMHCCCLFEVTSNRAEKLL-------------------------------------------------------------------------------------------------MKYDKSKISPELGKKLSELIDFHVKAIQYAETMENALNIVMLSEMGGCTIIICFLEYGILQDLEDREGMVTYIMLMTSIFVNVFILSYVGDKVKEQSEAIGFSAYSMQWVDLPNEIMKDLKFVMARANQPTRLTAGKLFDLSLQGFCDVAKTSMAYLNFLRTL-

>NvOr3

-------NEKFEEDVKYATALNRRIANAIGIWPIFTS-----TGARCVKTLKNAAVYILLSFLLVPGILHIVVEEGKLKAKILKTGPMILNTMALLKYSVMLFRKSQIQECLKQLESDWRKAGNDELRA-LMRRNTAVGHRLSRVCVATFYVGGIFYRLIKTLLTPIRYTKDG--------LMIKPLPSPLYFRFNTSASPVYETIFATQMMSGFVVHSTTVTTCSYAVLLATHACGQLDIVVYLLKRLIEDD-------------------------------------------------------------------------------------------------------EAVDRKLRVIVQLHLKVLRFISSVEDLMNQICLVEILGGSTILCLTSFYFIVDSNDALGLFTYMVMITSLIALLFTYCYVGEIVSDKAKKVGAKTYMINWYDLPPKKGLCIGLIISVAHSPVQLTAGKMLELSMYNFGCIMKSTAGYLNLLRTI-

>AmOr14

----------AENGMRHTVWFAYPLLRILGAWPNRVS-----SSSKIFNWYLIFTCYTLQLIVLVPGFLHVFLKEKNGRKKMKMMIPQVNGYLQLCKYSLVLRWTNKLRVLLNEMKEDWLNTTEED--QLIFRAKASFGHRVMSMIAIVTYSAGLGYRTILPLSK-GRILLPNN-TT------KRLLPCPGYFVFNEQVSPIYEIIFIIQVLGGLLTYTIMCGTIGMCVMFCLHSSSLLRILLNKIYQLTKQ---------------------------------------------------------------------------------------------------------VVHEKIVDIVKYQTKVKGFLKNVEQLTTYLFLLEIMVETSIGCVIGYNVVTEDSNAAAMIIHLMMQVSTISCTFIMCYVGQTLIDEGNNVRRMSITLDWYRFPVKEARNLILVIIMSSYPVKLTAGKVVDISLATFTDIIKTTVGYLNMLQKV-

>AmOr15

----------AEDGMRHTIWFAYMLLGKLGAWPNRAT-----SFSRTRNCILIFMCYSVQLIILIPGLLHFFLKEKDSRKKVKILIPLINGYLQLCRYSLVLRSANKLCHLLNEMKKDWMNISEEDR--LIFRRKASIGHRLMSVVAIIMYSAGLGYRTFIPLSK-GRILLPDN-T--T----IRLLPCPGYYIFNEQITPNYEIVFTLQVIGGLLSYTIMCGTTSMCAMLCLHATSLLRILVKKINELTKQ--------------------------------------------------------------------------------------------------QPDINESAVHMKITDIVRYQTKIKQFLNDVEHITTYLFLLEIIDETGIGCVIGYCAITEDSDATAAIIYLLLEASVFGVTFTMCYVGQILIDEGNNVRRMSITIDWYRFPAKEARNLILVIIMSSYPVKLTAGKVVDISLSTYTDIIKATVGYLNMLRKV-

>AmOr16

---------KAEEDLKYATRFVKPILATIGAWPISSS-----TSLKALQRLGHIFTYFLFFLIMIPTLAYVFLKEKNSKVRLKLMGPIINCSMQFFKYTIIIWRRKEIQEGLHAIRHDWIQATE-EER-LIFRSKMKIGRRVVLIAAFTMYGGGLCYTILPLLKGT---VIT-A-DNIT----IRPLPCPSYFIINEQQSPIYEILFVLQVMAGMAIYAVISGTCGISALLVLHACSMLRILVNKIKKLVNKSDMSE-----------------------------------------------------------------------------------------------------LQRKIMDIVEYQMKIKRFLKNIETVTEYICLIEMIGGTCLMCLVGYCILMENTNTMAVVVYITLQISIIFCVFILCYIGQMLVDENYIVSQASSTINWYRLSIKNMRCLILIIAMSNYPMKLKAAKMMEMSLTTFTDVMKMSMGYLNILRE--

>AmOr17

---------KAEEDLKYATRFVKPIMGMIGAWPISPS-----TSLKVLQRLRHIFTYFLFFLIMIPTLMYVFLKEKNNKVRLKLMPPIINCSIQCFKYTIILWRRKEIQEGLYAIKHDWIKATE-EER-LIFRSKAKIGRRVVLVVAFTMYGGGLCYMILPLLKG---TIVTA---NNTM---IRALPCPSYFFLNEQQSPIYEILFVLQIIAGIAIYAVICGFCGIFALLVLHAWSMLRILVNKIKKLVDKSDMSE-----------------------------------------------------------------------------------------------------LQRKIMDIVEYQMKIKRFLKNIETITEYICLIEMIGSTCMICLVGYCILMENTNTMAIVIYITIQISIIFCIFILCYIGQLLVDENYIVSQASSTINWYRLSIKNMRCLILIIAMSNYPMKLKAAKMMEMSLITFTDIMKVSMGYLNILRE--

>AmOr10

---------NAKEGLRHTFWFAYPFSRMLGHWPLSVS-----SSSKILNSFIIFISYLLQMIVVIPSLLYVILKEKNPKKKIKLLMPHLNSIVQMIKYTILLRQMKLIDKLLDEIKKDWSIATEENR--RIFSRTASVEHKLTSIIAITIYSGGFFYRMILPFSK--NKIVSNN-M--T----IRLLPCPGYFGLDEQVSPNYEIIFILQVFGGFVIYTAVCSTKSICLMLCMHMCGLLRILTNKVMELTNDND------------------------------------------------------------------------------------------NKVMELTNDNDERVVQEKIVHIVEYQMKIKEFLKQIDQFVPTIYLFEVFIQVLIMCIIGYCIIMEESNGMGLITYVIVQMTCLIGSFSVCYVGQLLIDESENIRQAFIALKWYQLPVKKSRSLILLIIISNYPIKVTAGKIIDLSLVTFITIIKTAVSYMNMLQQI-

>AmOr11

---------NAKEGLKHTFWFAYPFSRTLGYWPLVSP-----SATKFFNSFTIFTLYFLELIVLIPGLLYV-LQVKNPRTKIKLLMPHLNSIAQMAKYTIILQRAKEFSKLLDEIKKDWLLATE-ENR-QIFSERASIEHKLTTVIVVTMYGGGFFYRTILPLSK-GKILLPNN---MT----VRLLPCPSYFGLNEQATPNYEIIFTLQVLGGFIIYTVLCGTKSACLMLCLHMCGLLKILTNKVMDLTNDSDE--------------------------------------------------------------------------------------------MDLTNDSDEQVVQEKIVHIVEYQTRIKEFLNQLDQFVPAIYLIEVVIQVLIICIIGYCIIMEDSNAMAMVIYVVFQVTCVIGTFSVCYVGQLLLDESENIRQAYNTLNWYRLPVKKARSLILLILMSHYPIKVTAGRIMDLSLVTFTSIIKSAVGYMNMLRTV-

>AmOr12

---------NAKDGIRHTFWFAYPFSRMLGYWPLSVS-----SSAKISNYFIIFLSYLLTLIFMVPGLLYIFLKVKNGRSRIKLLMSHINGIVQMAKYTILLRKTKEIAKLLDEIKKDWMTASE-ENR-QIFSTRASIEHKLTMVVVVTMYGGGFFYRAILPLSK-GKIVLSN---NVT----IRLLPCPGYFGFDEQVSPNYEIIFTLQVLGGFVIYTAVCGTKSICLMLCLHMCGLLKILTNKVMELTND-------------------------------------------------------------------------------------------TNKVMELTNDKDEKVVQEKIAHIVDYQTRIIEFLNDLNQFVPSVYFFEIILEVLIICIIGYCLITEDNNTMATVIFVIFQITCFIGTFAVCYAGQLLVDESENVRQACSTLNWYRLPVKKARSLILLILMSNYPIKVTAGRIVDVSLVTFTSIIKNSVGYMNILQQV-

>AmOr13

----------------YSLKLVYPLLKILGAWPKSSP-----SSSTILKCCLISICYLIQLMVLIPGILYIFLKEANLGGKIKMFVPHMNGITQVSKYTILLRQIKEFNIILKEVKRDYSLATDKNM--WIFTTRAYIGHKMMIAIAIAMYSSGVGYMILPFLKG--RILLPDN-TT------VRLLPCPGYYMFNEQVTPNYEIIFTIQVLGGFLNYTTLCGTTGITTMLCLHMCSLLEILINKMNDLTCQ-------------------------------------------------------------------------------------------------------EIIVRKKLADIVEYQMKIIDFLNHVEQLTSYLYFCEILEYVCGACVIGYCLITENSNAAALIVYFILEFLCIFCTLTICYIGQLLIDESDKVRQISVTLDWYRLPVNEARGLILVIIMSNYPIKVTAGKIVDISLITFTDIVKTSVGYLNILRTV-

>gi|332017493|gb|EGI58214.1|

------------------------------------------------------------------------------------------------------------------------------------------------------------------------------------------------------------------------------------------------------------------------------------------------------------------------------------------------------------------------------------------------------------------------------MITKTITVMSTLLLQLFFYSFVGDYLKCQMGEIGFSIYSCNWHCLPMKLMRNILFIIMRSQQPVQLLAGRFFVVNIETFMAILKSSLSYLSVLRVM-

>DtakOr46aA

--------------------GQKALLNVFSLWHQNER-----RWRIIHQVNYVHVMGFWVLLFDLLLMMHVVANLSYMSEIVRAIFVLATSAGHTTKLLSIKANNVELEQLFKRLDEDDFRPRGVEEE-LIVAAACERSRKLRDFYGALSLAALG-MVLVPQFVVD-----------------WSQLPLGTYNPFDNPGSPGYWLLYCYQCLALSVSCLTNIGFDSLCSSLFIFIKCQLDLMAVRLDKMGRLSSA------------------------------------------------------------------------------------------------------SVEQHLKQNIRYHMTIVELTKTVERLLCKPISVQIFCSVLVLTSNFYAIALLSDEKLALFKFITYQACMLSQIFILCYYAGEVTQRSLELPHELYKTSWVDWNKDSRRIVLLFMQRLHSTLKIRTLNSIGFDLMLF-SIVNCSYSYFALLKRV-

>DtakOr46aB

------------------YKYQVWYFQILGVWQLPIT-----NHQRRFQSMRFAFILVILCIMLLLFALELLNNISQVREILKVFFMFATEISCMTKLLHLKLESRKLVGLVEMMMSHEFAFKTEQERE-ILESARTAVVHMRNFYGIMSFGTASLILLVPCFTN------------------YEELPLAMFEVCSIQGRICYGLQYLFHSICLLPTCVLNITYDSVAFSLLCFLKVQIQMLVLRLEKLG-------------------------------------------------------------------------------------------------------RDNEKIARELRECAAYYNRIVQFKNLVELFIKVPGSVQLMCSVLVLVSNLYDMSTMNGDAIFMAKTCIYQLVMLWQIFIICYASNEVTVQSSRLCLGIYSSQWTGWNRSNRRILLLMMQRFNSPMLLTFNPTFVFSLEAFGSIVNCSYSYFALLKRV-

>gi|332026239|gb|EGI66379.1|

---------------------NSRVNMLSGNLLPIAN-----LRFSVIWRTYSAAIWLIQLTHTIALIFGIIMASKE-NGLKGSTHTILLTIEALFMLTRLYSHKRLMEEMIQKMNTILQNTDEIM--KDIIKSAIKPIIVPFIIYGVISMISIIIYHAKQPIILVFKFIFFYV--NC------NLLATFSSEPFSNN---AMISSTVLKIVGTIYLFLKKFGMDVYMMHLVLMLTAQYRYTATKLAILFRDLQIYNESRKEHYP-----------------------------------------------------------------------------------------ENRRIERELRKLCLHQNIVLNMSFILKKLLSVNFSLLYINNVLRFCFIGIILSTVLMNIAEGISVTIFGIGTLTQFFVLCSSVQTLSDASTKITDKAFDEGWYQLGPSLKRIFILLIISNNLECKIAAIGKFNLSLPSFMTV---------------

>AmOr18

--------ANYKNDLSFNVRLNVWTLRTIGTWPRSPS-----WLETLEHVCLNLFCYELLAFILIPCSIYIILEIKDFYNQLKLGSALSFFLMAVMKYCVFIIREDDIRKCVELIENDWKNVRYQEDRKIMLE-NASFSRRLIVICGTFMYGGVIFYYIALPLTR--AKIVEEG-GNLT----YRRLVYP--FPVDARHSPINEICYTIQLLSGFVAHNITVAACGLAALLAIHACGQLQILMSWLEKLVDGRKNDNEN---------------------------------------------------------------------------------------------KNDNENLDQRLANIVKQHVRIINFIALTEDLLHEISLIEVVGCTLNICFLGYYSMMESKQPVSGVTYIILLISVTFNIFIFCYIGQLLAEQTVKVGEKSYMIDWHRMPWKKSLAIPLMISMSHSTTKITAGNIIELSISSFGDVIKTSVAYLNMLRTF-

>AmOr19

-----SYNASYKNDLFFNVQLNVWTLRTIGTWPKSLS-----WLETIEHVCLCFLNYVLLAFILIPGVMYFLLEMKDFYDQMKLGSALSFFLMAVMKMCVFIIRENDIRKCIECIEDDWKNVKYQEDRKIMLE-NASFSRRLIVICGAFMYGGVVFYYIALPFTR--AKVVEEG-GNLT----YRRLVYP--FPKDARRTPANELLYTIQLLSGFVAHNITVAACGLAALLAMHACGQLQILMSWLEKLVDGREND------------------------------------------------------------------------------------------------ENDDENLDQRLVNIVEQHVRIINFITLTEDLLREISLVEVVGCTINICFLGYYSMM-EWDTEHLIRYIILLTSVTFNIFIFCYIGELLAEQTVKVGEKFYMIDWYRMPWKKSLAISLIISISRSTTKITAGNIIELSISSFGAIIKTSFAYLNILRTL-

>DanaOr85e__scaffold_13340_residues_18699561_to_18702841_reverse_strand.[501_2909].sp

----------------------MRLQLAMGMVPTPKP-----WWPKWLRSVYCSIVIFTSLHLGVLFTTLDVLPTGELQAITDALTMTIIYFFTGYGTIYWCVRSRRLLAYMELINRQYRHHSLAGVTFVSNYESYRLSRNFTAVWILACLLGVITWGVSPLILG------------------IRTLPLLCWYPFDALAPVTYSVVYATQLFGQILVGLTFGFGGSLFVTLCLLLLGQFDVLYCSLKNLEAHARLLSGDSVKDLSLLQGELL--LEDSTNQYAVLKEHPT-D---------------------------------------------------------------DNVLLEGLKECVRLHRFILHCSIELENLFSPYCLVKSLQITFQLCLLVFVGVSGTREVLRIVNQLQYLGLTVFELMMFTYCGELLSRHSIRSGDAFWRSGWWKYARHIRQDILIFLVNSRRAVHVTAGKFYVMDVNRLRSVITQAFSFLTLLQK--

>DanaOr67c_scaffold_13337_residues_15153178_to_15155557_reverse_strand.[501_1769].sp

--------DNTPRTFKDMMRVPVQFYRSIGEDIYAHR-----SKRSLLLKVYLYAGFINFNVLVIGEIVFFFKSIQNFETIIAVAPCIGFSLVADFKQFAMVYHKQTLIRLLDELEDMHPKTLEKQKEYKM-SNFEQTMKQVINIFTFLCLAYTTTFSFYPAIKATVK----FQ-LLGYDTF-DRNFGFLIWFPFDATSSLVYWITYWDIAHGAYLAGIAFLCADLLLVVVITQICMHFEYISMRLEE-------------------------------------------------------------------------------------------------------------ENKEFLIYMIRYHDKCLKLCEHVNDLYSFSLLLNFLMASMQICFIAFQVT--ESTVEVIIIYCIFLMTSMVQVFMVCYYGDTLIAASLKVGDAAYNQKWFQCDKSYCGMLKLLIKRSQKPASIRPPTFPPISLVTYMKVISMSYQFFALLRT--

>cplu_011994_b1

-------------------------------------------------------------------------------------------------------------------------------------------------------------------------------------------------------------------------------------------------------------------------------------------------------------------------------------------------------------------INCFQWRIRYCIRHHQRIVEIVNRLNDTLSSCLMAQFAVSTMIFCLNGFLQSHFKIDE-----VDFFLLVGFIQIFYWCRFGNELKFQADYLTTSQWMSGWENFNSELRNYITVAMIRTMKPVEIKAGGLFVLSMETFLSILKNSYSVFVLLTTV-

>DpseOr42b__Ch3_residues_11826570_to_11828588_forward_strand.[459_1831].sp

---------------------LFRAMKFIGWVPPK---------SGVLRYVYLFWTLMTWSTTYLPLFLGSYMKSFSPGEFLTSLQVCFNAYGSSVKTAITYSQLWRLIKAKDLLDKLDLRCTSVEEREKIHRV-VALSNHAFLIFTCVYCTYAGSTYLSSVLS--------------------GRPPWQLYNPYDWRDGRNLWLASTLEYVVMSGAVLQDQLSDTYPLVYTLILRVHLDMLRERIQRLRTDEAMS---------------------------------------------------------------------------------------------------EAENYEELVNCILDHKIIIMFCDTIKPVISGTIFTQFLLCGIVLGLTLINVFFFSDLWTG-IASFMFVITILLQTFPFCYTCNLIMEDCDALTHAIFQSKWVDASRRYKMTLLYFLQNVQRPIVFIAGGIFQISMSSNISVAKFAFSVITITKQM-

>DrhoOr47b

------------------FNYVRAFLSLLCQYPTK-K-----LASLALYRWLHVIIMCNMMSVLLT-LVFALPESTNVIQMGDDLVWISGMSLIFTKVFYMHWRCDEVDEVIWDFDYYNRELRPHQDDEEIL-GWQRQCYLVESGLYINCFVLVNLFSVAIFLQPLIS---------------EGALPFHSIWPFQWHHPYMFWFIYVWLAATSHHNLMTILMVDLLGISTFFQTALNLKLLCIELRKLGDLRA-----------------------------------------------------------------------------------------------------DARFHEEFCRLIRFHQHIIKLMTKANRVFNGSLNAQLLASFSLISISTFESVVASLDPKMAAKFVLLLMVAFVQLSLWCVSGNLVYTQSLEVAQAAFEINWHTKSPAIQRDIGFVILRAQKPLMYMAEPFMPFTLGTYMLVLKNCYRLLAVMQ---

>gi|170062194|ref|XP_001866562.1|

-------------NYHGTYAVYRVFMFISGVNFFDDD-----FMVGPLNIARFLGPAIAAILGTISCMIHLHRYLDEVDQVILSLAAFFSGTELLIKMCGMAVKRNKGAELLAVILNDRSYEDGPVERAIFVN-YHGLARTLMFITIFSYPFTALMLISYPVIAGKLG---------------EFLLPMGFSIPFISHKQPWYEINYLIEVVQMVWCAIAFIGLDGPFYIYVCYATCKMEVMKTYIEQIGDSEDVEQ---------------------------------------------------------------------------------------------------------LRKIIGIHTHVLQFLRDCSDFYQEIYLAQVFFSIAHICVSLFHVQLKLKNSS-----YGMLATNVAKMWIFCYCGELVVTKANEVCEAMYANRWYRWRKDDLRTVRFVLANTQQKVGFSIGGFRFLSYDAFTEIMKTAYSCNAFLHNM-

>gi|340721655|ref|XP_003399232.1|

-----------------YYNTIKKYLRFLGLDPHQK-----------YGLIIVIIMVISMTSGLVPMVLYGSLCTKNLDMVLECLPHLGALLTSVVKILNVHLNRENFKKLFDSITKEWQQLKLSQDL-YILEEVTIRGSKMAKLYRNTLLICMVLFLLVPLLPPMLDIVLPLN-ETR-----PRQQILNVNYVFDSDN--YFFYVYLQLSWTSIVVSIIIVTVDSLLMLIVHHNSGLFIVCGHQIQKSTRHLNSFTNEV-----------------------------------------------------------------------------------------------ERYTYKQIRNCVIMHNKAIDFYDILDENNRISYMIQIGLNMIGITTTAVQTVINLDRPGESIRSAVLCGANQFHLFMLSLPGQILIDHCTELTKQLYNSTWYGVPVKVQRMLYMMQIRTRRPCTLTACGLYEMNIENFGTTFKTCMSYITMIMSLK

>DeleOr59c

-------------------DYYYRITFVLGLTPPK---------EGLARWIYFLWSAIVMWLGIVYLTYVLHFDRFTPTEFLSSLQVDINCIGNVVKSFVTFSQMWRLRRMNELIAPLDARCDTPSQRQILHKV-VARVNLTVFIFVS-MYLGFGFLNVFTSVLA-------------------GKAPWQLYNPFDWQNGWQLWIASFLECFVVSIGTMQELISDAYPIVFVSLFRGHLAVLKNRIENLRQDPELSEEENYRQ--------------------------------------------------------------------------------------------EEENYRQLVACIQDHRTIVEXAQVIRPILSITIFAQFMLVGIDLGLAAISILYFPNTIWTIMANVSFILAICMESFPCCMLCEHLIDDCAHVSDALFHSNWISAEKRYKSTVIYFLHRVQQPIQFTAGSIFPISVQSNIAVAKFAFTIITIVNQM-

>DyakOr94a__chr3R_residues_19672571_to_19674787_forward_strand.[501_1717].sp

----------SHKDRIESMRLILRVMQLFGLWPWSLW-----TCTGFVKRHYRYLLHLPITFTFIGLMWLEAFISSNLEQAGQVLYMSITEMALVVKILSIWHHRTKAWRLMQEFQYAPDYQLHSQEEMDFWRREQRYFKWFFYIYILISLGVVYSGCTGVLFLE------------------DYELPFAYYVPFEWRNERRYWFAYGYDMAGMTLTCISNITLDTLGCYFLFHIALLYRLLGLRLRELKNVQ--------------------------------------------------------------------------------------------------NVQDDATFGQELRAIFILHQRIRRLTLTCESIVSPYILSQIVLSALIICFSGYRLQYVRDNPGQFIAMLQFVSVMILQIFLPCYYGNEITVYANQLTNEVYHTNWLECRPPIRKLLNAYMEHLKKPVTIRAGNFFAVGLPIFVKTINNAYSFLALLLNV-

>DficOr85b1

------------------TQYSTFFYTAVGIDPYVRS-----KQSTLRARIVFLANVINLTIVAVCEYAYSAFQEKKLLEAVTVMSYIGFVFVGMSKMFFIQWKKTVLNEILEDLEAIFPKGKVQEEK-YDLPKYLRTCSGICLTYASLYSLLIWTFNLFSVMEYL----VYEK-WLKTRVV-GKGLPYLMYVPWKWEDSWSYYPLLFSQNFAGYTAAAGQISTDLLFCAVATQLVMHFDFLSSRM-----------------------------------------------------------------------------------------------------MTHKLSGNWKKDSKFLSDMVRYHERILRLTDVVNDVFGIPLLLNFMVSSFVICFVGFQMT-VGVAPDMVVKLFLFLFSSMSQVYLICHYGQMVADASFGLSVATYSQNWVHADVRYKRALVIIISRAQNVTFLKATIFLNITRSSMTDLLQISYKFFALLRTM-

>DficOr85b2

------------------TQYSNFFYTAVGIDPYDKP-----GKSRLRALFLANVINLTIVAICESIYVYTAFQENKLLEAVTVMSYVGFDIVGISKMLFIRWKKTGLNEILEDLEAIFPKGKVQEEK-YDLPKYLRTCSGICLTYASLYSLLIWTFNLFSVMEYL----VYEK-WLKTRVV-GKGLPYLMYVPWKWEDSWSYYPLLFSQNFAGYTAAAGQISTDLLFCAVATQLVMHFDFLSSRM-------------------------------------------------------------------------------------------------------HKLSGNWKKDSKFLSDMVRYHERILRLTDVVNDVFGIPLLLNFMVSSFVICFVGFQMT-VGVAPDMVVKLFLFLFSSMSQVYLICHYGQMVADASFGLSVATYSQNWVHADVRYKRALVIIISRAQNVTFLKATIFLNITRSSMTDVLQISYKFFALLRTM-

>H_TcOr126FIX

---------------------PLRFFGFIGFHPIF---------NSLILKISFYSTTTLGLFIYTMAIIGIVKEETNSFFTLECLQTCILLSHTIGKQVNYYMNSNKIVKFLQMTTEFWEFETFQGTIHPESNFLFHTVRKMIRYYFLVTTFGFIFFLINPPVCY-----------------------------------------------------------------------------------------------------------------------------------------------------------------------------------------------------------KHQIFLHSCKLLNQIFAVVFLIQFLNSIAALAISIFIFSK-PGSWNNRFKTLFYLVVVLFENAFYCVPAELVSAEALKICDQLFASKWYESNVQFRKSLVIVLCCTQKVIKFSSFGLVEMNLQTFVLISKTALSFYAFLNQLK

>cplu_000252_b1

----------------------------------------------------------------------------------------------------------------------------------------------------------------------------------------------------------------------------------------------------------------------------------------------------------------------------------------------------------------------------------------------SYAAALLVYLVVGTIQLCIIGYHILVLMGKQHSLMPFFVFIITTYGLISIYCILSENLLAESKKCSEAFWCCQWYEMPQDCVKDIAYCIMRAQKPLGLTAGAFVVFSNSTLTDVTKTSMGYLSILRNF-

>DanaOr42b__scaffold_13266_residues_19110808_to_19113003_forward_strand.[501_1696].sp

----------------------YRAMKFIGWRPPK---------EGVLRYLYLFWTLMTSTTYLPLGFLGSYMKSFSPGEFLTSLQVCINAYGSSVKVAITYSMLWRLVKAKDLLDQLDLRCTSMEEREKIHRV-VARSNHAFLIFTFVYCGYAGSTYLSSVLS--------------------GRPPWQLYNPFDWHDGTKLWMASTLEYIVMSGAVLQDQLSDTYPLIYTLILRAHMDMLKERIRRLRTDETLS------------------------------------------------------------------------------------------------TLSESENYEELVKCVMDHKLILKYCALIKPVISGTIFTQFLLIGLVLGLTLINVFFFSDIWT-GIASFMFIITILLQTFPFCYTCNMIMEDCEALTHAIFQSNWVDANRPYKTTLLYFLQNVQQPIVFIAGGIFQISMSSN-----ISA----------

>DyakOr22c__chr2L_residues_2040048_to_2044539_forward_strand.[501_3992].sp

-----------------FYRIPRFSGRIVGLWPQRID-----GGRPWHAHLLFVFAFAVVLLGAAGEVSYGCVHLDNLVVALEAFCPGTTKAVCVLKLWVFFRSNRRWAELVQRLRVILWQSRRPEAQ-RMLVGLATTASRLSLLLLSSGTATNAAFNLQPLIMGLYRWIMQLP---G-----HIELPFNIILPFAVQ-PGLFPLTYVLLTASGACTVFAFSFVDGFFVCSCLYICGAFRLVQQDIRRIFAD---------------------------------------------------------------------------------------------------TAQMNAEVRHRLAQVVERHNAIIDFCTDLTRQFTVIVLMHFLSAAFVLCSTILDIML-NTSSLSGLTYICYIIAALTQLFLYCFGGNHVSESSAAVADVLYDIEWYKCDARTRKVILMILRRSQRAKTIA-VPFFTPSLPALRSILSTAGSYITLLKT--

>DsecOr59b____super_9_residues_2645391_to_2649692_reverse_strand.[1501_2802].sp

----------------------YRAMWLIGWIPPK---------EGILRYVYLFWTCVPFAFGVFYLIISYVQEFKNFGEFLTSLQVCINVYGASVKSTITYLFLWRLRKTEILLDSLDKRLANDNDRERIH-NMVARCNYAFLIYSFIYCGYAGSTFLSYALS--------------------GRPPWSVYNPFDWRDGMSLWIQAIFEYITMSFAVLQDQLSDTYPLMFTIMFRAHMEVLKDHVRSLRMD-------------------------------------------------------------------------------------------------MDPERSEADNYQDLVNCVLDHKTILKCCDMIRPMISRTIFVQFALIGSVLGLTLVNVFFFSNFWKG-VASLLFVITILLQTFPFCYTCNMLIDDAQDLSNEIFQSNWVDAEPRYKATLVLFMHHVQQPIIFIAGGIFPISMNSNISVAKFAFSIITIVRQM-

>DtakOr69aA

-----------------------------------DR-----KPYTWKRKALLVLGIICSIYQIFGVIIYWYRNGRKVTEISEMCGSLMLTVVGFANIYSLIKNRSEIEEMFKELQEIYPRSGDRHY---QSQHYYDMAIMIMKIEFWFYMVFYVYYNSAPLMLLLWENLQEE------PQL-SFKTQTNTWFPWKVHGSIGFGLAILSITMASFVGVGFSIATQNIVCIFAFQLKLHYDGMASQLVSLDS------------------------------------------------------------------------------------------------------------NRELRKLIAYHSRVLRIGDQFNEILNFVFGSSLVGSTIAICMTSVAVLL--LDLASAFKYISGLIAFVLYHFVICYMGTAVT----------------------------------------------------------------------

>TcOr88

---------------------SCFFLKWSFMWPTKSF-----RTSKGLYFRLLAFVIISGLTFTAMIVMHLLKSVEGDYDISEDIAILATNTGYILMMLLYIIRQKDLESLLVDLSSFKKYQKPPKFD--EVNR---KLEWCTRMVFGYCVFGSVFYNLVKILAIPSC-KKSRR-INE-----VCGVAIPYWVWFDTENWSIKLPLILHTFLVIIIVDKVTLLVSLQVLEIACNIKLRLDQLNCMLVSCFD-----------------------------------------------------------------------------------------------------------SRRRLNECIKYHKEIISYSEIFSKCFSIEMFTHLTTTGIICGCLENQVVQ-EHRPEA----ILHIGGWITAIFVSSFGGQILIDSSLSVAEAAYSSAWYEADVSLRKDLILVILRAQKALFVSTGPFNVLSFALFVSIMKMSYSILTILQ---

>gi|51127348|emb|CAG38119.1|

-------PEKKYKGFNETFKLCAFSLAFAFLYPNR--------TTALRRCITITLIVTFQLFWFIT-YTFKCLYTLDIYNFARNMTLAVVLVLFFIKTYYVIYATSKFAPLLDKISEDLLEANNLEEEFQVL--YDDHIKIAKVGEISWLLIPTIMSALFPIYAG---ALMTIE-SIQTDDYE-RRMVHDMELLFDIQSEPFFQCMFAYNCVQCVVLVPNYCGFDGSFCIATTHLRLKLKLMTLKVYKAFKYSKSRQE----------------------------------------------------------------------------------------------SKSRQELRVRLYESIKDHQDALDFYVQLQNVYGPWLFAVFLLTSFMISFNLYQIYLLQRID---PKYTSFGVVGVLHIYLPCRYASDLTRVSEEIPDDLYLAEWEAWDPSITKMLMFMITRAQKEMIVTGMGLVVFNMEMFKSILQTSYSFFTLI----

>TcOr80

---------------------ILTYLTFLGLWPRSRK-----S-----TKTVAYLIISSTSFLFFGSLFYLIAHRKFGSNEIDSIETVTSQFGILYYWVLFTLKREGTVEIVERLSDFSKFGKPRFFDQ----R-NRRLNYLLSYFVLVLMVAIGGVVALPVVYIDSCHKANER-LNLTK---TCGLIAPVWLPFDYNEYPRKNFVFAWEVYCCIMTYACCGIAALVLVGTMEHLIIRFEQLKLMFPEILDEPDR-------------------------------------------------------------------------------------------------------RQQKLKNWIEYHLTLFDIGKLMTSNYTYCLSVIVLCVGILFGCIGVSTMQSASSHN----SVFLFFGWFQSIGVLCIWGQRLLDTCLSVGIAVYSSRWYDMDVSFQKSVLMILIRSQKPILIYAGPFSYLSHLLILSVFQTAYSYINLL----

>TcOr86

---------------------SCFYLRYSFLWPEEA------PTRSFYAKFI-LVLILSFLTAFLPLFIHFLILVERGLDPSEDLFVIISYTGFALIMIIYVIHVKKTSYLIVQLSDFEKFGKPRGFD--YW---DKKFRLISSGVYYYVLIASSGLNLGRWVGM-------AE-CRKERDFQVCGIVIPYWLPWKVDSWLFFILLDLYVLKMTLVVNCALFLIIIQILEITTHLKLRIDHLKEMLVKCFDSDSQTNRKQ----------------------------------------------------------------------------------------------DSQTNRKQLVNCIRYHTYIINCSKLFKKCFTHAMFSLIVTMALSCGCLESQVVKFD------LWALPPISAWIFILFIACMAGQILMNASLSIGDAGYHSKWYQTDANFRKYLILVLMRSHKALVLSAGPFNILCFELFVAIMKFSYSVFMLLNQ--

>DpseOr67d_ChXR_group6_residues_8657414_to_8659681_forward_strand.[501_1768].sp

-------------PFERYCKINRAIRFCVGLCGNDVA-----EDYRMWWLTYAVIGAILFFFGCTGYTVYVVVLDGDLTVILQAFALVGSAVQGLAKLLVTARMAAVVRQIQATYEAIYREYARRGDYGRCLERRIKTTWHMLMSFMWVYVVLVGGLIAYPFFHLILH----H----------KKLLVMQFRVPWDESTDGGYLVLISIHVMLLSMGGFGNFGGDMFLFLFISNVPTLKDIFSAKLREFNEVA------------------------------------------------------------------------------------------------------YQRMRTLLWDLLAWHQQYVSILRDTERIYRIVLFVQLSTNCVSILCTISCIFIGAWPAA-----PIYLVYSFIVMYSFCGLGTIVETSNEDFSKEIYNCLWYELPVKEQRLVILMLAKSQHEISLTAADVMPLSMSTALQLTKGIYSFSMMLIT--

>DrhoOr85b

----------------KFMKYANFFYTAVGIEPYXXX-----XXXXXXXXXXFWANVTNLTLVAIGESIYFAYSSGKLLEAVTVLSYIGFDIVGMSKMCFIWWKKAAMNGMVRELEEIYPRGKMQEQR-YKLETYLKSCSRISFTYSLLYSLLIWTFNLFSIMQFL---VYEKW-LNIRE-V-GKTLPYFMYIPWNWEDNWTYYLVLFSQNFAGYTAAAGQISSDLLLSSVATQVVMHFDYLSESME---------------------------------------------------------------------------------------------------------TGDWEEDSVFLTNTVRYHERILRLSDVMNEIFGIPLLLNFMVSSFVICFVGFQMT-VGVSPDMITKLFLFLFSSMSQVYLICHYGQLVADASYGLSVAAYSQNWNHADIRYKRALVLIIARAQKLTYLKATIFLDISRSTMTDLLQISYKFFALLRTM-

>DrhoOr85c

------------------MKYAVFFYKSVGIEPYTKP-----KKISLWSNLLFWANVINLSVIVVGEFLHLALYSGKLIDAVTVLSYIGFVFVGMSKMLFIWWKKPDLSDMVQELEHIYPQTKDQEK-VYRLDSYLRSCSRISITYALLYSVLIWTFNLFSTMQFL---VYELW-LNIRV--VGLSLPYPVYFPWNWEAPWSYYLLLFCQNFAGHTSASGQISTDLLLCAVATQVTMHFDHLARVVEN---------------------------------------------------------------------------------------------------------EDWSENSRFLAEAVRYHQRILRLMDVLNDIFGIPLLLNFMVSTFVICFVGFQMT-VGVPPDIMVKLSLFLFSSLSQVYLICHYGQLIADAXXXXXIAAYKQNWTDADVRYRRALVFFIARPQRTTFLKATIFMNITRASMTDLLQISYKFFALLRTM-

>DeugOr33a

--------------------TYWRYWRFLGVEGEY-----------PFRRLWDLTMTIFITILYPVHLILGMYDKPK-VLIFRSLHFTIECLFCSFKFVFFRWKLAEIKEIEGLLQDLDKRAGSEEERSY-FKENSRVAKMLSKSYLVAAISSIITATVAGLFSS------------------GRNLMYLGWFPYDVQATLNFWTSFTYQAVGSSLMILENLANDSYPPITFCVVTGHVRLLAMRLSRIGHDEDISSNENTS---------------------------------------------------------------------------------------------SNENTSRLIEGVQDHRKLMRIIRLLRSILHLPQLGQFLSSGINISISLVNILFFAENNFTMIYYAVFFAAMLIELFPSCYYGTLMMMEFDKLPYAIFSSNWIKMDKGYNRSLIIFMQLTLIPVDIKAGGIVGIDMSAFFATVRMAYSFYTLAMSFR

>DeleOr63a

-----------KKRNYRSIREMIRIAYTVGFNLMDPS-----RCGQVLRMWTIVLSLSSLASLYG-HWQMLARNIHDIPRIVETVSTAFQFLSSIAKMWYFLFAHRQIYDLLRRARSDLPVIKTIREKESTMDRYWANTRRQLLVYLYCCICITTNYFINSLATTLYRHFTRPK-GSY-----EIVLALPSLYPGWAEKGPYYYIQMYLETCSLYICGMSAISFDGVFIVLCLHSVGLIESLNQMID------------------------------------------------------------------------------------------------------------------YLRCCIYQYQRVASFAEEINDCFRQVTFSQFLLSLFGFGLALFQMSVGSNSSIILFRMTLYLIAGGYQIVVYCYNGQRFTTASEQIGKAFYEVQWYGESREFRQLIRMVLMRTNRGFRLDVSWFMQMSLPTLMAMVRTSGQYFLLLQNV-

>DmirOr59b1

----------------------YRAMWLIGWIPPK---------EGVLRYVYLFWTCVPFAFGVFYLIISYVKEFKNFGEFLTSLQVCINVYGASVKSTITYLFLWRLRKTEQMLDTLDRRLQTESDRQKIH-NMVARCNYAFLIYSFIYCGYAGSTFLSYALS--------------------GRPPWSVYNPFDWRDGLSLWIQSIFEYITMSFAVLQDQLSDTYPLMFTIIFRAHMDVLKDHVRSLRMD---------------------------------------------------------------------------------------------------PDKSEAENYQDLVNCVIDHKIILRCCDMIRPMISRTIFVQFALIGSVLGLTLINVFFFSNFWKG-VASFLFVITILLQTFPFCYTCNLLIDDGDDLANTIFQSNWVDAEPRYKATLVHFIHNVQQPIIFIAGGIFPISMNSNISVAKFAFSIITIVRQM-

>DeugOr33b

-------------------------WRLLGL-----------ESSFILHRLLDILITVFVTVWYPIHLILGLFMERTLVDVCKGLPITAACFFASFKFICFRLKLSEIKTIEVLFKELDQRAVSHEECQFFNQNTRREANFIWKSFIVAYGLSNVSAISTVLFGG------------------GHKLLYPAWFPYDVQKSLRFWLSVTYQIAGVSLQILQNLANDSYPPMTFCVVAGHVRLLAMRLSRIGQDEKESQAS--------------------------------------------------------------------------------------------EKESQASIGKQLVESIEDHRKLMRIVELLRSTMNMSQLGQFISSGVNISITLVNILFFAENNFAVTYYGVYFVSMVLELFPCCYYGTLISVEMNRLTYAIYSSNWLGMDRGYCRTLLIFMQLTLAEVQIKAGGMIGIGMNAFFATVRLAYSFFTLAMSLR

>LhOr321PSE

--------------LDRYYYINRKLLTITGLWPYQ---------KWKISTIQATIFLSTISSFLIAQLTTFMTSEFNIDIALMIFPQALPFLLLILKYIFFLQNNATIKQCYELIHCEWIALKVPAEV-DILKKYADLSKLFTTVVTGLVLAFTIIFDLFHFLPDFLDIIIPLN-ESR-----EDYFIFVTEYFVDQKR--YIYFIITHCLLAVTLGGIVLAATGCMLMAWILHICGMLKIASYRFEHISQRSE-------------------------------------------------------------------------------------------------------------------------FAEIVVSNFTIITFFLTIVAILSISLNMFQFINFKTDIRKILICAVMIFIQFTYLFISTLCGQMITDHNIDVSNATYS-RWCTVSLQAQKMLLFILLKNTKNYYLVVGGIWTSSVETFASLANVSISYFMVIYST-

>gi|284010002|dbj|BAI66636.1|

-------------------------FRVVGAWPSKFG-----DVQTTSDAVVKYIQLVLNVVCQVAGILYLRENMDSFFELGHSYITVLMSLVSMSRIITHCTYQEIFSLYVRKIHLF--NVRNDSEYAMEMHTKHKLCYFLTFFIHAFMTLGILMFNLIPMYSNYINGKFNRE-TGAFSGVSNATMEHAVYYLWDTTHPIGYAIIVAFNWYISLVCSINFCTFDLFLYHLVFHIWGHLKILIHNLKTF--------------------------------------------------------------------------------------------AIRNEEQNDYTEEESKQIYERLKKLVQHHNLIIDFIARISDTLGLSLFVYLCYHQVCGCILLLECSTL--ELSALIRYGPLTAIIFQLLIQVSLVFELLGSMTESLMNAVYDLPWEYMEVRHRRTVHIMLRQSQVSLNTRALNMVDIGSRTMIAILKTSLSYFVMLRT--

>gi|332019954|gb|EGI60414.1|

--------------------------------------------------------------------------------------------------------------------TDWMTSTKEWERNTMLKI-TRSGRNLSFKCCALATSSITCHISLQLLR-FFKTIHQ----------PERNLVYRIET---IQKSPNYEITYFIQLFGGMYSAFANSIIDSFISMLILQVCAQLINLRTMLNNLVNKLANKS------------------------------------------------------------------------------------------------------------------------------------------------------------------------HLISLYAYCYSAEKLITESTRMAYGVYECKWYDLSSKDAKNLMFIVHRSRIPLKLTAGKFGTFSLEMFGIV---------------

>DmelOr82a_3R_residues_82690_to_84170_forward_strand.[5_1474].sp

------------------FQLQEYCLRAMGHKDDMDS-----TDST-ALSLKHISSLIFVISAQYPLISYVAYNRNDMEKVTACLSVVFTNMLTVIKISTFLANRKDFWEMIHRFRKMHEQSASHIPRYREGLDYVAEANKLARAYCVSCGLTGLYFMLGPIVKI----GVCRW-HGTTC---DKELPMPMKFPFNDLESPGYEVCFLYTVLVTVVVVAYASAVDGLFISFAINLRAHFQTLQRQIEN---------------------------------------------------------------------------------------------------------------QIRLKSIVEYHVLLLSLSRKLRSIYTPTVMGQFVITSLQVGVIIYQLVTNMDSVMDLLLYASFFGSIMLQLFIYCYGGEIIKAESLQVDTAVRLSNWHLASPKTRTSLSLIILQSQKEVLIRAG-FFVASLANFVGICRTALSLITLIKSI-

>CpluG2R504O02B94FA

----------------------------------------------------------------CPQFLEILLVRTSINELTNNMTMLLVMVTACGKIVGILFNRNEIVEILKSLEEKPFKPRDQFEE-DIRKKYIHISSLLTRSYSVYLTIGISGILLTRIPEA---------------KL-PDVLPFSSWLPYN---------------------------------------------------------------------------------------------------------------------------------------------------------------------------------------------------------------------------------------------------------------------------------------------------------------------------

>DsecOr85e__super_0_residues_17391563_to_17394012_forward_strand.[501_2084].sp

-----------------------------KWWPKRLE-----MIGQVLAKVYCSTVIFTSLHLGVLFTTLDVLPTGELQAITDALTMTIIYFFTGYGTIYWCLRSRRLLAYMEHMNREYRHHSLAGVTFVSSHAAFRMSRNFTVVWIMSCLLGVISWGVSPLMLG------------------IRMLPLQCWYPFDALGPITYTAVYATQLFGQILVGMTFGFGGSLFVTLSLLLLGQFDVLYCSLKNLDAHTKLLGGESVNGLSLLQEELL--LRDSNNQYVVLQEH--------------------------------------------------------------------NAFHNALVECVRLHRFILHCSQELENLFSPYCLVKSLQITFQLCLLVFVGVSGTREVLRIVNQLQYLGLTIFELLMFTYCGELLSRHSIRSGDAFWRGSWWKHAHFIRQDILIFLVNSRRAVHVTAGKFYVMDVNRLRSVITQAFSFLTLLQK--

>gi|383850710|ref|XP_003700919.1|

----AKTEQDYLKNVDLSIRLNRWILKPIAAWPESSS-----PIKKYFGWFVHVVCYFLVSFLFVPCLTFLILEVNDTYNRIKLIGPLSFFLMSYLKYYLLLLHKNDVLKCVKQIEWDWKNMKHLEEKRIMVMN-ANYARRIVIVCTFFMYSSYIFFYIAVPITV--GRIPAQD-GNFT----FIFLPFPSSMRIDYRQSPVNEIFYFLQCLAGVVLHIITVGACSLAATFAVHACGQMEILMNWLGYLVNGRADMSKS-----------------------------------------------------------------------------------------------MSKSLDGRIASIVEQHVRILKFLALTEKALQQISFVEFLGCTMNLCLLGYYSIMEPKDVMFSLTYMVLVTSFGFNIFIFCYIGELVAEQCRKVGEVSYMIDWYQLSGNKKLCCILFIAMSNSSIKLTAGNMVELSISTFSDVVKTATGFLNMLRAL-

>DsimOr88a__chr3R_residues_11492031_to_11494413_reverse_strand.[501_1883].sp

-----------------------------------------------------------------------------------NPPVLSITIYFSIRGLMLFLKRKEIVEFVNDLDRECPRDLVSQLDMQMDETYRNFWQRYRFI-RIYSHVGGPMFCVVPLALF----LLTHE--GKDTPVAQHEQLLGGWLPFGVRKDPFYLLVWFIDLMCTTCGVSFFITFDNLFNVMQGHLVMHLGHLARQFSSIDPRQSLTDEK---------------------------------------------------------------------------------------------LTDEKRFFADLRLLVQRQQLLNGLCRKYNDIFKVAFLVSNFVGAGSLCFYLFMLSE-TSDVLIIAQYILPTLVLVGFTFEICLRGTQLEAASEGLESSLRSQEWYLGSRRYRKFYLLWTQYCQRTQKLGAFGLIQVNMVHFTEIMQLAYRLFTFLKS--

>NvOr54PSE

-----------------SFCSLRFIGTCLGIWLTDNK-----KW-TCKNDYLFWFCILNYIVSILPLFYSLYLNRRNVSAALYTWIELSGVIKMMVITIYSKFQRHRLNARLIALFVPTEQLLAIDKV-KIVKKYANTYTLMFLAVLLLYMITMSIYLAIEQTSNW-----------------IRIRXMTAVYPFRFYCYAVKVLTCANZAFVQMHSVI-LPTFDGISILLIFMCTYRIKVLDHDFKNAZNF-----------------------------------------------------------------------------------------------------------FIELRKCIQEHENILXMVKEINLIIRFMIFKTVITFMSNVIACDLHILN-NVPVTQSTFQVSTIFLVCTEIILCAECADNMTTAGEDMEFTIYSTPWYEEEPKIMIIKSIILSKCQKAPVISINGVMSLDRKYLATIMYATVSYITTLRAV-

>NvOr200PSE

-------------------------MSILGFWPSR--------------IVLRCFWVFQHMSIMIPEAIKLYENRNNIDLVIEGTAPFTYNTTMLIKFLNGIFNLEKTKSVLEKVKNDWNTLLDDRZTE-ILSYNCSSGKFLNTVYIYMAWLTLVSYMFFPLTPMILDFISPLN---ETR---PKSPLYLVELYIDQDK--------------------------------------------------------------------------------------------------------------------------------------------------------------------------------YCDNINEIYNISFFIILIVELLEMSVTGTAXVSR--CQSHTAIFSTSHHTQIFHVFCYFFLGQNVLNYSENLRDTAYNLNWYAASTKTKYLIQFIIMKSLKPCLISVS-IFPLTLENFTTLMKTVFSYFTVMNSTR

>gi|380014823|ref|XP_003691416.1|

------IQKKFDNLNEYSVQFNKWFSKTIGVWPLPSS-----KFEKITTKILIFLCWIITLFVTISSLLHFALVKEDIIVKLKTLAPISYCFGGGLNYAVLLYHKNDILYCIEHMEVDWKAITKTADRQV-MFKNAKIGRIISASIAAFVQISAVCFCTVLVFKRT-----IKI-GNES--MEIHVLPSPTYIPVDT--NPGYGIVLGLQYITAYIMSATVVIAFSLATVFACHTVGQLTIMVTWIEEFINQPQEENKN---------------------------------------------------------------------------------------------EENKNVRIDEISVIIQHHMRILSFLERAEHLLSPICFMEMFKNILSICMFSYCIL-AEHDITILSTYIFNVMNLTFSTFLICYIGEVLTERCKEVGNMVYMTNWYQLHNKDILNLIMIIARSSVEYKMTAGKIMDMSVITFGNIIKTVFAYLNILRQT-

>NvOr249

---------------------IKCLIVASGIWPHY-E-----KHPHVLRKLLSFCSAFCSGSTFYCIVAFCFKYATNINIFTSCLGLMIGFFTTFIKIVILSMRQEDLQSLNEGVSKSFENNLKLPENQPHLLYHFPSFSRFFYLYAYVVGISFVFLASTPLSIM-----LRYG---------KYVRMYPQLMPFAYEPGSVHWAVFGFEMFTGFYLWSVTIGVDSIFGLYALHMVGQLRLLGSRFQNLKSSSN-------------------------------------------------------------------------------------------------NLKSSSNYDKELGECVRSHIQLMKSRHKLQRVFGFLAIWLAVTCAIALCSQVFQALHMNTTPVRALYLFGHWFIKIVQAYSYSWYGNIIAVESDLCLNSMYYSHWPGGDKRFMADVLIIL--SQKPLVFKAKQLMELRLDMFLKIVHTSLSYFFLLRTL-

>gi|167865758|gb|EDS29141.1|

----------AAAEFDRILAVQSWILRRLGCDVFD-----------LNYRFATWVIVFLASFYMVISAYDLYRFRNDVFNFAFSLVTLSYGVIGCTRIVLFLRNSRTYAQIVVEARRTYEQVSNEREQ-EVQERYTRMLKRCVTFYSVSFIGGCIMGGFFPLAVYWW---------TG-----LKVLPFGVILPFDPDTIEGYQLNYLYQVSCIVWTPPGLTATQNVYFALVFNLCIQYDVLKLKLEDLDKL------------------------------------------------------------------------------------------------------EYDTIHEKLVEIINWQRHLVDFIAEIDRNFTVQTFVEISSVAMQMVIVLFVLHIDVWLP-----GYMVIFVASFQLFVLCILGAMIEFKSDIFTEQIYDIAWHRMRTPEQKMVQFMLAKAQYTMQLTYGGMLPLNMNLFVTIYKKTYSVFMMLQNM-

>PbOr113PSE

---------------------------YIGIWPDA-------SHGTLRWVFYMATLFVMQYFQY--SYVYAHLDFNNLTKLMDGLGLVLDYTLTMLKLLSFWINRRXL----AAMEDDWKDCVTD-FRKYVMMDKANLTSNVVLTFWYPSMLFLLFYILLTATYV------------------DVCLPYGQFFPVQIQFSPVYXLIYMGVFFHVLETAIVIALLNALILTXVLHVSGQIEIMCQELKDIPF----------------------------------------------------------------------------------------------------------------KSLVARHQKIISLSKNIENFFSFVALLQFIWNTFIICALEFMAVISESKSGILIQFIMLYLAVTMEAFIFCFAGEYLSTKSKSIGDAVYEMVWYNLSTSECRILLFVILRSQKQLT-TAGKVMDLTLESFTTIMKASASYISVLHA--

>NvOr241

---------------EAYTHNVIWFLKSAGLWPEA---------HPVPRKILSMVTLCSTFVVMVTVSNFSFQNVGNVMVLTRGMSLAVSFSSAFSKVALFLLHHDDLVYLNKHLTGGFMRDMKEPENRPDLLNNVKTFNRFMFTHAISVAIAMSMYSIGPLLAL--------R-KHG-----KYIRAFPAIYPFAYESGLVHWILYALEVSGAASLWTVTVGVDCVFGLYALQVCGELRILAKKFRELRATEN------------------------------------------------------------------------------------------------------ENYREKLHDCIQRHHVLINAKNKLDNIFGLISIWLAISGALVLCSLIFQVTELLKTNNSYLRLCAYLLPKFLQIFTYAWYGNLIAEESGACLDAMYGSHWTDCDKNFKSDILIVL--AQEPLALVAMGCMVIQLDMFTKIVKTSVSYFFLLRTL-

>NvOr242

----------EVEKYKKYKSNLKFMIVSNGVWPDY-E-----KHPYCVRKFLNFCSISSISMTNYCMMLFVIATTTDVRSFTSFFGLLLGGFGNLFKVCALTMNQKELHALNEGISASFERNLRVPENRPHLLANFPMFSKFFNFLSYSTLGTIGFLTVIPLLHL----------RHG-----TYSRMWPILLPFSYEPGTIHWIIFVFELVVSFFAWITTCGVDCLFGLYSLHIVGEMRLLSSRFQKL----------------------------------------------------------------------------------------------------------SENYRKDIRSCVKSHLLLLKTLSQMQEAFGDLAVWFAFNSAASLCTLVFQFSQLVMNPARVLYLLCHTCIKLVQAYSYSWYGNIITVESEVCLNAAYNSHWPNGDKHFMRDVLIILL--QRPMVFKAKSFIALRLDLFARIANTTLSYFFLLQT--

>NvOr243

----------------AYTHNVIWFLKSAGLWPEG---------HPVSRKIRSMVTLFSTFVVMVTVSNFSFQNVSNVMVLTRGMSLAVSFSSAFSKVALFLLNYEDLVYLNEHLTGIFERDMKKPEYRPDLLKNVKTFHRFMYTHVASLTFTLIMYVIGPLLAL--------R-KHG-----KYVRVFPAIYPFAYEPGLVHWILYILEVLGATCLWSVTSGVDCVFGVYALQVCGELRILAKKFKELGAIEN------------------------------------------------------------------------------------------------------ENYREKLNDCIRRHHVLIKAKNKLDNIFGLISIWLAISGALVLCSLIFQITELKSSYLRVVHLSVYLLPKFLQIFSYAWYGNLIAEESTGCLEAMYDSHWTDLDKNFKSDILIVLV--QEPLTLIAMGCMVIQLDMFTKIVKTSVSYFFLLRTL-

>NvOr245

--------------YKAYERNVIWLLKSAGLWPEA---------HPVPRKILSLVTLFTSFVVMVTATNYSFQNVGNVRMLTKGMSLAVSFSSVFSKIAFFILHQEDLLYLNKHLTGGFMRDMKRPENGPALLSNVKTFNRFLYMHAVSVAIAMIMYSITPLLVL------R---KHG-----KYIRTFPSIYPFAYELGLVHWIIYAVEVSAAATLVTVSAGVDNLFGFYALQMCGELRMLAHRFRDLRAGNNY-----------------------------------------------------------------------------------------------------NNYKDNLKDCIERHQVLINAKNKLEDIFGLITIWLAISGSLVLCSLIFQVSELIKNHVRIAHVCAYLLPKFLQIFLYAWCGNLIAEESKICLYAMYDSHWPDHNTNSKRDILIVM--SQEPLSVVAMGCMVIQLDMFAKIVKTSVSYFFLLRTL-

>NvOr246

---------------KKYFKYIVFMLSASGVWPNY-T-----SHPAAVRLFLNICSALASGCMFY-CIVNFCLYATNINAFTSCLGLMIGFFSTFIKVIILPMQKEDLQSLNEGVSASYERNLRIVKFRHHLLAHFPMFSRFFYLYSYSVGMSVLLLTIMPLLAL----------RQG-----KYVRMYPQLVPFSYEPGSLHWSIYAFEVFCGFYLWSVTSGVDSVFGLYALHMVGELRLLNVRFQMLKSSN----------------------------------------------------------------------------------------------------KSSNNYAKDLKSCVDSHIMLMESRHKLQRIFGFLAIWLAITCAIALCALVFQALQAKHATIRIIYLCGHCFLKLLQAYFYAWYGNIIAIESDACQSAIYESQWPGGDKRFMNDVLVVL--SQTPMIFKAKQWMPLRLDMFSKVVHTSVSYFFLLRTL-

>NvOr247

---------------KSYARHVITRLIFAGIWPES---------NKTIKTILYFISFTSTLTVSVTSINFGIQNANNVILLTKGIGLASAFSSVFSKALLLPLHQEDIIFLKNRLTTKFMSDMETIEYRADLLSSVHVFSAFFNMHEAMVAFAMFMYCFVPLYVLFK---------HG-----TYLRTYPCLYPFSYTPGLVHWLIYALEVAGAISVWTITVGADCGFLMYALELCGEFKILARKFTELK------------------------------------------------------------------------------------------------------------YKRNLKECIERHHLIIEAKNRLEDSYGLIVIWLALSGAFLLCSLIFQITELHGSYVRIAHLCSHLVAKNLQIFMYAWYGNLIADESKAFLNAMYDSHWPECDKNFKNDILIVL--TQEPLVVVAKGCMYVQLDMFTKIVKTSMSYFFLIQT--

>ApOr5

------------------------------------------------------------------------------------------------------------------------------------------------------------------------------------------------------------------------------------------------------------------------------------------------------------------------------------------------------------QTIGEAFADNDEQKFKYAIIQHQKLLSYFNTMKKVFSKPILLSMSFNAIYFGLTTSFVIQARGYINQAIISICIASSAVINITIYTFYGSELMDLHDKILHVLFDNAFFYVSKSFKSSILIMITRVTIPLKFTVGYIFTINLNLLLKILKMSYTVLNVLLS--

>TcOr114

---------------------SLKLLKVCGLHPNS---------NNKLLNVYFVVNYSCFLAILTLAIIGISKTYDNVFEAIENLQTVFLYIHLLGKYPTLFFKKKTLKELLERQKQFWPIDNVEPKLAKKFDQILTNTTKFIRYFIIVTFLVIMNFFLQPILT--------------------GDLPVRVYVPSGWFH----YINSVY-WYLIPVIIGSIYGSDLIFCSLCVPVIIQFQLLAHKFEKF---------------------------------------------------------------------------------------------------------------QQLKKLVDYHNFLIKYCNDLNKYIEPIFLNQVIVATAIICMQLFIVSQKEFLLPNKLKCLGYCFGEVIETAIYCFNAEMISDAAEKVGIAVYNSQWYKVP---RKSVVLVIAKTQKKVVFNGLGLVAINLKTFTQIFKTALSFYSYLNTM-

>ApOr7

--------------------------------------------------------LVVTLFFAISAFLSIVYSEEDLSNRVYGFLWLFVEIHVFGLIVVRLYHQSQCRDMYDRSLLIEQG---------IPKNYRRTIAMVIAYYCIMSTVHVTVPMLYTISSDSA--------QVGD----PFAFPFADVLPIKTTNPTAYVCKYIVYAFPVYLTHLECCFMNVTFMYFTGVVKRHFQILDQQVQEAVANE--------------------------------------------------------------------------------------------------------EDEQKLKIAIEYHQESLKFFKEMETVYEKPLIMTIEFCGLYIGLTGYIMIQCIIHPIILGLCIASSTASLITISIYCICGSNMYDLHDGILNSLFEQSCYSRNKSLKHLILMMMKRATIPLELKAGSIFKINSNLLVKILKFTYTVFNLLLT--

>376_Si_gnF.scaffold05285_6032-497272.pep

---------------KNYYDIVCKISSLTGLWPYL---------KPRARIFRVGLLTVTMLTIFIPQIAYQFTCKTNLQCIFEAMTSYLLTVVAFVKVYTFQFNTHKIKGLTQHLFVDWKRLETPKEYE-IMKLYAKNSRRFCMVYAVA-TFTFMSMTFIPFVFD---VVWPLN-ESR-----PVLPPYPGYYFVDNRE--YFFKIYCHSLISWEIIMVGIVAHDCMFVTYVEHVCSKFALVGFHFENLFCN---------------------------------------------------------------------------------------------DEEIKITDNSDDTYRKRIKLFVHEHWEALKFAETLEDTFVVPFAVQILIVTVGISVTLLQITQQEGEVLESIRYVVYVIGQLIHLFFLSFEGQKLIDHSLQTGDKICNSAWYEVSVRSQRLIMLVMMKSTRPSFLSAGKIYVFSLESFTTVMR-------------

>ApOr1

----------KKDGLIKDLWPNIRLIQLSGLFISEYY-----DDYSLFRKIYSWITAIIIYSQFIFIVIFMVTKSNDSDQLAAGVVTTLFFTHSMIKFVYFSTGTKSFYRTLSCWNNTSPHPLFAESHSRFHAKSLSRMRQLLIIVSIVTIFTTISWTTITFFGESVWKVPDPE-TNQTMYVPVPRLMLHSWYPWDSSHGLGYIVAFVLQFYWIFITLSHSNLMELLFSSFLVHACEQLQHLKEILNPLIELSATLDSSVHNPAEIFRANSA--KNQS---------------------------------------------------------GTKGENEPNRKGPNNLTSNQEVLVRSAIKYWVERHKHVVKYVSLITECYGSALLFHMLVSTVILTILAYQATKINGVNVFAFSTIGYLMYSFAQIFMFCIHGNELIEESSSVMEAAYGCHWYDGSEEAKTFVQIVCQQCQKPLIVSGAKFFNVSLDLFASVLGAVVTYFMVLVQLK

>ApOr3

------------------YWLTLLLLNGINVYPGHGN-----RVVRIAAAAYPWMICAFWAFITTSVTTSLALRATSYQEAVEMLTYITGSSSTLALFAIGVHNRPGLHRMLDAVRRDFWDDGRRPAA-------DVLFSRFVRTYGAILPIANVMMCMTPVIWA--------A-RNGDIES-PAALIFRMWTPWTRLTTARYAVVYAAQFVVSLSVLTSISGMVFAMVLFVTEMQVQVDTLVDAVQDL----------------------------------------------------------------------------------------------------------RRAAFDGLVKCIKHHQALITYFNRFKSYFNLLFIVDILYIMVMTCLCASSVLMANGFSAFHIKMMSLLIIVVSQFFFYCLIGEQFSTMNQQIGDCVYFKLVKCKDPMLSRAGLLVILRTQKPLQLTAMGIYTASLFTFTVTMRSAYAGLNVLYN--

>DgriOr85d__scaffold_14830_residues_3409316_to_3411873_forward_strand.[525_2058].sp

--------------LERFFRYVNFFYLSIGMVAYDSS-----DRCSWQRILLRCFFIFQMVNLNAALLIYVFLRSNNFLEATMNLSFIGFVIVGDVKIRLIQQQRKRITRIVHQLDALHPLSIEQQAD-YKTAKYLSDYSRISKFYFIVHLILIWSYNLYWAIYYLICDFWLGI-R----RF-ERMLPYYCWVPWDWKRNWIYQFMYVSQNLGGQACLSGQLAADLFMCALATLLIMHFTHLATQIEHHVAGIL------------------------------------------------------------------------------------------------------------LSTSIIYHQQLLLLGKEINKIFGVSLLCNFASSSFIICFVIFHITI-GSTIDNVVMLMLFLFCAMVQLFMIGSYAQQLINASEQVGEAVYNHDWFGGELRYRKMMILILRRAQHPSYLRATSFLSVSMVTVTDLLQLSYKFFALLRTM-

>gi|350415227|ref|XP_003490572.1|

------MKTSTSKDFAYAMTPLKILSWPVGTWPLQD--------YNIFSAIRATIATFFLLLMVTVVQSEMYLDNSDAEKNLDGLVLITCGSLAASKIIQFRIRPAALISNFTSAVEDYMELRDEEKRL-IMRKHAYMARVASASVICFAYFSSILFITVPMLAEEEEKDIVNV-TEESIT--EYPLPSENVMAVIKMPDNLHFIVFIVEYLMLLFLSTGNIGSDSLFFGIIFHLCGQVEILRLEFNRLNNENEKAME-----------------------------------------------------------------------------------------------NENEKAMEHFISLTKRHIYLLKLAKMLSETISSILIVQLFTSCILICTSGLQFIIAVGNIVMTIKSFIVSSTLLLQLFAYSYVGEYLKRQMEAVGNSVYFCSWYDIPKCVAKDIIYVIMRTQDPVFLKAGKFFVVNMETYMSIIKTSMSYLSVLRVM-

>DpseOr22aL2_Ch4_group3_residues_10093594_to_10095937_forward_strand.[501_1844].sp

--------------SRDAFVYLDRVQKFFGWTAVEDK-----RW-RIPYILWGIFMNLLLIFFLPISLVAYIQMFKTAGEFLSSLEITVNMYGCVLKCIYTIWGFKGFTAARKVLDELDLRCTSDEERT-SVHRCVALGNLSYVLFHIFYSGFVVINWTGYVLM--------------------GRHAWMMYLPLDAEN--NFFVASLCEILLMSGVVTMDQCTDVSPLAHMLMARCHICLLKDRLTKLRTD--------------------------------------------------------------------------------------------------DPTKDEDEHYEELSNCVHDHRLILDYVKALRPTFSGTIFVQFLLIGIVLGLSMINVMFFSTLWTGLG-TVCFMFCVCLETFPFCYLCNMIIDDCQKLSDNLFQSDWTTASRRYKSTLVYFLQNLQKPIILTAGGVFPICMQTNLSMVKLAFSVVTVIKQF-

>TcOr119

----------------------LKILWYTGLHPAL---------SSRLINIYIYANLFLCSVLTILAIIGIVLSYTNIFFVAECLVTIILMVHAIGKFIALHWNKKSLINLLKKKSQFWKIGSFDGEIHNECLQISTFVKNIIRFYYALSFCGGVFFDLQPFTS--------------------GLMPSGCYVPEGWSN-----ILMGVMWYITFPVVFVVTGTDALFCSLSTSLIIQFKLLNHKFKTLKLTNKS----------------------------------------------------------------------------------------------KLTNKSQTQLWNDLKELVDYHNFLLSYCEELDATFSGVFLLQFIISIAPASVSIFIFMQ-PGAWANRMKFITYFLAVITETTFYCLPLDIVVNTASQVSDAIYESKWYEVDLHFKKCLTLVIARAQKSVRFTGFGMVYINLRTFLIIWKTVFTFYTYLNSAK

>ApOr8

----------------------------------------------------------------------------------------------------------------------------------------------------------------------------------------------------------------------------------------------------------------------------------------------------------------------------------------------------------------------DEQKLKIAIIHHQQVLKFFKDMKTVYEKPLLLTIEGFGLYIGLCCCAIIQVQGFVDQIILCMASCVAGFMTISLYCICASNMYDLQNGILNSLYEHRCYSRNKSFKRLNLIMMTRATIPLEFNVYSLFIVNLNLLVKILKLTYSVLNVLLT--

>HsOr114

----------------WTINLNRFMLRIAGLWPPDHD-----ASEAVKSKIQLLYNFITLIVLAIPSLLSLIRVWGDTILMIENMQYSIPYLSTIFKICIIWYRQADLLPLIHMIEKDWAKPKMKEERNIML-KYARTIRLIAMCGLSSTLLALISTCGYSCFEII----FRHV-ANLTN---PTKLPLPVHYLHDVSKSPQYELTLLAQITTLFICGFSYTAVDQLFGLLVLHVCGQLESLNLRLTRMEQ------------------------------------------------------------------------------------------------------EQYTNFDATLKYNVQDHVRLIKSIEIIDDAFDLMLLVLIFYFAIIFCLEGFLIVNIQLPLKQFIWFITGIVYILIHTCLYCAIGEIIVTQSEKISQATYEHPWYNLKPRAAKNLMLIMHRASKPLQITAGKIFPMTMIMFSNLLKTSASYISVLLAI-

>gi|242005719|ref|XM_002423665.1|

-------------NFNEFFFSYFNYLAYVGGWTYKTN-----NITNRIIIFYQILCLVSMFASIALQLADLWRVSGNLDKVTYNMCTSVIVIDTTSKVVVLLNKTKIINKLRLNLWNDFYKYQDEDIE-KLKNSITSELKRVSKMFHVAGLVFYPVWISVAYFSYLWG---------------PRELPFPAFLPINHDDSLSYNLCFLLEIVLGTIILTAAIETNLIVFSLVLQMVLQYKILYRRILKVSD-------------------------------------------------------------------------------------------------------KRKKTENYLKNIINRQLILSHYVKLFEFGFNRYFLVQITGGSTLVIMNGLHVIKMTYQLSKIGHQAAYLFFIIVNFFFNCFYTSKLVEEFNSIGEAAYATSWYKMDANLQKYFLMIIIQAQKQPKITAGRFILMNLNTFLTMLRNTYSWLMLIRQF-

>DmelOr46aB__2R_residues_5650740_to_5654344_forward_strand.[501_3105].sp

----------------DFYKYQVWYFQILGVWQLPTA-----DHQRRFQSMRFGFILVILFIMLLLFSFEMLNNISQVREILKVFFMFATEISCMAKLLHLKLKSRKLAGLVDAMLSPEFGVKSEQEMQ-MLELDRVAVVRMRNSYGIMSLGAASLILIVPCFDN------------------FGELPLAMLEVCSIEGWICYWSQYLFHSICLLPTCVLNITYDSVAYSLLCFLKVQLQMLVLRLEKLGPVIEPQDN--------------------------------------------------------------------------------------------IEPQDNEKIAMELRECAAYYNRIVRFKDLVELFIKGPGSVQLMCSVLVLVSNLYDMSTMNGDAIFMLKTCIYQLVMLWQIFIICYASNEVTVQSSRLCHSIYSSQWTGWNRANRRIVLLMMQRFNSPMLLSFNPTFAFSLEAFGSIVNCSYSYFALLKRV-

>gi|167873240|gb|EDS36623.1|

---------KSYEMYDYNLLFIRWLADFCGVDMMV---------EHYRFNYRTALCILALSLSVILSLYTFILYYPDIYKISEVLVLTGFILQGSVKFYYGYSHRKFYQVQYGRLRNLHYKYRNHEKINASLLTLMERIHMLSKLIWVVFLLGAMGYTIYPLYMHFA---------NG-----ERVLMIAMRIPIDTDLLSGYVATSVMQMAMLIVACTGISAADTVILLFVANLIAYVDVFTNELDELNA-----------------------------------------------------------------------------------------------AMLNAEVRDEAKIRQQVRVICSQHQDIIEYESDLDERYIMICFAHVIGSIVAMSTALFLVYM-----IQFVPGYPILLAVFCQLLEFCLLGTVLTVKNEEIIRAIYSAQWYLLREPERRSFALMLHKSQNFIEMTIGGFAPLNLETFVAV---------------

>367_Si_gnF.scaffold05285_6032-497272.pep

---------------------AKKMLSLAGLWPYQ---------EKRTRLFRVSLMTVITLSIMIPQLGKFIQCDGDVRCIVIVVPAILFIVVVFVKLYSCQFNSSKIKSSIDHLFNDWEKLKGAEEYK-IMKAHAARARLLSLIYCSFIYVSTALFSSMSLVPRILDVVFPLN-TSR-----RIILPYPAYYFVDDDQ--YFYYIYLHMLVASTVAMTGLIAHDSMFFVYIEHICGLFAVVGFCLKTVSRNDR----------------------------------------------------------------------------------------NDRNNVSNNSKNGDKIYNQKTAISIHIHWRAIWFAEHLEETFSISFVVQMMIVVVVMSITLLQIALQFEDVLETIKSISFIVGQLMHLFCYSMQGQKLMDHSIQLREEIYNSFWYEIPVKSQRLLLYVMQRSLEPNFLSAGKIFVFSLKSFTTV---------------

>gi|170037991|ref|XP_001846837.1|

--------------YDSLIVVPNRIAKIIGLNVFS---------ENYKVLTWNWFSLIMMISVYFYCAVTAYEVRFDTGDLIYCLVEGGIGIQGLAKIYTYLVYRKELVWIHQYTKQLYHEACNDQTK-SMLMDNIFLLKVAIIVMLMCYAFTSISLITAPMLFSIM--------T-G-----EKILPFGFYIPMDRTEWFGYLINYAVHLYDTIYVSAEDMAADTIYMITMLSAFTQIDLLMMSLKET-------------------------------------------------------------------------------------------------RMLDKDEKDDENLQAHLALVIKRHEEHLKYLRTVETVYRFYFFITFVSLASVLIMALFAVVTLSW-----YQGYIFVGFISYELFFGCFLGTLL-----------------------------------------------------------------------

>DsecOr59a____super_9_residues_2615133_to_2619327_reverse_strand.[1501_2695].sp

----------------EFFKSHWTAWRYLGVAHLRVE-----NWKN-LYVFYSILSNLLVTLCYPVHLGMSLFRNRTLTEDILNLTTFATCTACSVKCLLYAYNIKDVLEMERLLRLLDERVVGPEQRSIYGQV-RVQLRNVLYVFIGIYMPCALFAELSFLFKE------------------ERGLMYPAWFPFDWLHSRNYYIANAYQIVGITFQLLQNYVSDCFPAVVLCLISSHIKMLYKRFEKVG------------------------------------------------------------------------------------------------------------AEKDLEACITDHKHILELFRRIEAFISLPMLIQFTVTALNVCIGIAGLVFFVSEPMARMYFIFYSLAMPLQIFPSCFFGTDNEYWFGRLHYAAFSCNWHTKNRSFKRKMMLFVEQSLKKSTAVAGGMMRIHLDTFFSTLKGAYSLFTIIIRMR

>gi|340726311|ref|XP_003401503.1|

---------------DVSFSVAVFYLKIVGLWLST-N-----LVEKWFRNALVTYTVLAIIFNMWMQLRGLYFSWGDFSVSTYIACNSLGLFMDLFKLLIIFIHKKKFLYLVMYMQKNFWHFNYNQYEKSVLADAKRMCIYFVCVFSFLSQSTIFSYIFRPLISNIGK--------NES----DRVPIFHMYLDLPLNVSPYYEMTYLIQALTLYQVGVCYLCVDNIFCIMCLHVASQFRILQYRIANVLSLKDKVKFDQD---------------------------------------------------------------------------------------QDTNLDSSGEFYAIFKKCIQQHQALIGFCTTLEEIFTVIILGQVLTFSILICFVGYQALLVKLSLSWRISLVSFLTTNICQLWIFTYSCNALVQESMNTANAAYATPWIFLPMMARKDLQLVLMRSRRACYLTACGFFPISLETFTKIMSSAMSYFTILKQ--

>McOr56PAR

----------------------------------------------------------------------------------------------------------------------------------------------------------------------------------------DFMPYTFLYPFPSQTKRRCEIVALYMDIAISGVALGIACYDTFFAAILNCLTAQLTVVNKAFRTIRQRVLQ-----------------------------------------------------------------------------------------------ENPRLEKELYAELTFTTRHFVALLQIAEDLGKIYNLVILGQTVECLLIFAACVYIATTVPITSPEFAAASQYFAAVFVQLTLFCWFGNGVTTASEDILLALYECEWYSASRRFKSSLLITMIRMQRPVYLTLGKFGNLTLVSLVSVCQGSFSYYALFK---

>NvOr215PSE

-----------------YYGIIKTLHVSYGLWPEL-G-----KTRKFISLILYFTTT---LSLYIPIIAGIYLHRSEWVILLENVVGILYSLVTLLKYISCHMFEERIKRLYNRIVCDWENLTNKNEVD-ILHNYSKYSRTFAIFYIIYSAIGWIIILCLPFIPIIMDQLAPL--QNETRI--RIELYHPYYFGIDMNKYHXYYAYIFHGQVIYGISTIAIVTVDTMFAACMQHVCGLFAILCYRLKQIGSNTK-----------------------------------------------------------------------------------------------EMRVSDDKQVYRDIKFANEMYNNCRXFIDGIWNSFGITILNFVSSIIFATAVLLFDLLFYRIHLYERIILSILIMGILLLFLLLSWLFQHVSNSSEXVFKAVCCSCWYNLSVKEQKLMYLLMLRTFNSIKLRAGGLIQINLRQYAWVVKTAISYAMVMQS--

>gi|167881685|gb|EDS45068.1|

---------TSLQEFRNSFETVRKVTYMVGINVFT---------LEPDLNVRFFGSAMFMALIYVLCYTGWVLREAEGYRILEMVMILVLNAQGTNKMWIGFVHKPRYCRLFQSSEQIYESFDSDERNRPVLRDLVAKMNHLLKWIVIVYASSGLLIVVLVALYAIVA--------------REKFLALTIIVPFVDHTSTGYLIVYLTHMAMAAFCANGYLASDTAFIVTVVPIIAYANSLQNEIRNF--------------------------------------------------------------------------------------------------------------------------------------------------------------------------------------------------NDQMIHDIYEIGWHRLDKPQQQMVAFMLHRAQNAKELSVGGVAPLNLVTYVQ----------------

>PbOr73NTE

-----------------AIGWNRYNLNIVGIWPNPLN-----SKQKRLSLCFMIASFFMLGFMCIPQSANLVFIWGNTNLMTENLATAIPVATAFVKAFTMWRYRKVLKSLVDFFYQDWHSPKTSEERTIMLK-NAKLVRRISIWCTILTQVMVTIYIILKVSM-----VAKFS-RND--P--ARPMLYLAYFPFDITPRPIFELISTCQCLSAYTAAVSYTGSDSFISMLVLHTCGQFQNLHGRLKNLAN------------------------------------------------------------------------------------------------------KTSKEFKSELKQIVKRHEHLNWFAKTIEESFNMVLLIQMLSCTIQLCFQGFQVFRIGSVTFELIFLLSYIAFILVHLYIYCYIGEKLIVQSTNMGFAAYESNWFNVAGKEARDLLFIMRRSTRPLCLTAGKFSIFSLQMFSTIVRTSLGYLSVLLTV-

>DeugOr74a

-------------------------------WPLEAG-----RWTVFVDRLLIFMGFLVFC-----EFHYLIANRQDMGNLLTGMPTYLILMEMQIRCYQLAWHKDRFRALLQRFYAKIYVSEEMDPH---LFARIQKQMLATRANSTVYLLTLFNFLMVPVTNVIYH---------------RREMLYKQVYPFDNTKLYYFIPLLALNFWVGFIITSMLFGELNVMGEMMMHLNARYIQLGKDLRHSTNVLLE----------------------------------------------------------------------------------------------------------ALTQILRRNAVLRDFGERVEEEFSLRIFVMFAFSAGLLCALFFKAFTVCNPWGNVV-YIVWFLAKFMELLALGMLGSILLETTDELGMMYYTSGWEQVNVKLMKLMTLAIQLNSKPFFITGLNYFRVTLVAVLKIIQGAFSYFTFLNSMR

>MsexOR-40

----------------------------IGFFHGNSS-----YFKKYWRYSYIISCVVAYYSSLTVYALKIFLGQMELFELAYVVPVFVVCTQAILKAIIVIIHKGEIRALVLQLGETWRTDNLTTR---QLNKKNLLLKKLNFCYGV--------------------------------------------------------------------------------------------------------------------------------------------------------------------------------------------------------------------IELHDIVVKHQKLIKFSEQLNEIFNKMIFVNLSSVTITVCFFAFAT-KVARGPVDMANNFMAVMALILPIFNLCYYAEMLINASANKQESAYHSLWYVANKQYQMSIWFIIRRSQKPCCLTSLKFSPVALHTFTAVLSTTWSYFSL-----

>DkikOr74a

------------------------LYRILNHWPLEEG-----RWTVFLERVVIFLGFLVFCEHN--EFHYLIANRHDMENLLTGMPTYLILVEMQIRCYQLAWHKDRFRALLQRFYAEIYVTEEAEPQ---LFARIQRQMLAMRLNSTIYLLALFNFLVVPIQNVIYH---------------RREMLYKQVYPFDNTQLQYFIPLLGLNFWVGVIITSMLFGELNVMGELMMHLNARYVQLGQDLR---------------------------------------------------------------------------------------------------------------YRRALTHILRRNVALRDFADSVEEEFSFRIFVMFSFSAGLLCALFFKAYTX---PSGNVAYIVWFLAKFMELLALGMLGSILLKTTDELGMMYYTSDWEQINVRLMKLVTLAIELNSRPFFITGLKYFRVSLTAVLKIIQGAFSYFTFLNSMR

>394_Si_gnF.scaffold02694_2287513-2447739.pep

-------NKNYKRDINYVFELSRFVFRLLGIWPYARK------TETIERLVVILVSYILLTCELVPAILYMAIVQKETRARLKVVATVIFTMVAMAKYGQLVFSRDRVRSCLAQVEDDWRNVADSRNRDVMT-EKAKTGRRLLVICAIFMYSTGVSFRTIIPLSR-GK-IVT-E-QNIT----IRHLPCPNYFVFDVQLSPAYEIVFLMQFFSGVVKCTVTTAVCGLAGLCVMHVCAQLEILMVLMNNLVNERE---------------------------------------------------------------------------------------------NNLVNERELKNVNERLAIIVKHQIKARNFLQLVQNTIQYTSLLEVIGCTIIVCLLGYFVIMEDNNSIALCSYLIGLTSISFNIFIFCFIGEQLSTKGEKVALTACTLEWYRLPDAKARSLILIMIMSNLPTKIRGGKFMDLSLRTFGNVVKTAVTYFNML----

>gi|388429187|gb|AFK30402.1|

-----------------YIKTISFFLRPPGAWPSDVE-----GYLPLPIRIHRATLPFHTTIIVIGGLYYITDNFHSFLDMGHMIITTFLAMVTALRSILLQTYNSLLCKFLHEFHLMHHAYKGDYFE-EMNKTVDKISSYYTKFSTIIMYLGMLLFNITPTYNNIRHTLISKT-ENYS-----MEYSVYFSYPFNPLD--YFASTTIYNCYLSYNCSTLLCGFDLLLFLMIFQIIGHVYILRHNLENFQSPKNKIT--------------------------------------------------------------------------------------NTCTFEVFDAQENQEVRLQLAECIEHHKIIIGFTDDLSGVFGPILAFNYFFHMIACCLLLLECT--EGSYDAVLRYGPLTMIVFGQLIQMSVMFELLGSETEKLKDSVYCLPWEAMNTSNQRTAFIMLHKMQYKISLEALGLAAVGVNTMVGILKTTFSYYAFLQTM-

>DvirOr67c_scaffold_13049_residues_16030723_to_16033106_reverse_strand.[501_1884].sp

-----------PRTFGDMMRMPVRFYKTIGEDIYAHR-----SSRSLLLKIYLYAGFINFNLLVCGELVFFYKSIQDFETVIAVAPCIGFSLVADCKQLTMALYKNTLIRLLDDLEEMHPKTLVAQRA-YKLADFEHTMKRVISIFTFLCLAYTTTFTLYPAIKATVKYNFLG-----YETF-DRNFGFLIWFPYDATTKWVYWITYWDIAHGAYLAGTAFLCADLLLVVVITQLCMHFSYIATRLE------------------------------------------------------------------------------------------------------------DAENVRFLATLIKYHDKCLTLCELVDSMYSFSLLLNFLMASMQICFIAFQVT--ESTIEVILIYCIFLMTSMVQVFLVCYYGDALIAASLRVGDAAYNQNWFQCSKRYCIMLKMMILRSQKPAMIRPPTFPPISLVTYMKVISMSYQFFALLKT--

>gi|350412763|ref|XP_003489754.1|

---------HYENDIHYTLQMCQWLLKPIGVWPLVSS-----KLEQLVSVILMIMCFSSLLFIILPSFYHIFFVEKSVHVKVKLLGPVGFCLSSTIKYCYLGVKGAFFERCIQHVEKDWKMVQDPNHRTIML-KYATVSRKLITLCAIFLYTGGMSYHTVMQFLS----KERNK-KNYT----FRPLTYPGYSFLDTQSSPTYEVVFFLQCFAAMIMYSVTTVAYSLAAIFVTHICGQIQVQIARLHDLVENEKRKNNGR------------------------------------------------------------------------------------------ENEKRKNNGRDSMSVIVHDHVEVLRFSKNVEEALREICLTEIVESTIIMCLLEYYCMMENSDAIAILTYVTLLISFTFNIFIFCYIGEILTEQCSQIGITSYEIEWYQLPAKKAYDLILLISISHYPPKLTAGKIIELSLNTFSSVAKSSVIYLNLLRTV-

>5_386_Si_gnF.scaffold00330_12820-264637.pep

--------------------TLKFMLTLCGIWPGT--------PYVLLCRIFWVVSMTVILYFHY-RYFYWHVRSAEILDLMDCLSSFIAYSKIIIKFFVFWLNERKFVEILAKMAEDWSDCANNDVGLRETARKAKLSDRITNSIVILHTMTIVLYCIGIILTDV----------DVTDT--SKELPFKLDIPMDINTLFKYRIVLTVQFLYMMLSSWAAGITNSLLLTLILHTAGQIEIMRHWLAQLVPRKSEDK----------------------------------------------------------------------------------------------SEDKHKSITATASKIIQKHQKIISFTKNIENLYSYIALLQFVSNTIMICSLGFLIVTASNAVEQIMKSFLFYTITNLEAFIFCYAGEYLNNKSKEIGVAAYNCEWYDLKCTESRVLLFIILRSQKQLSLTVGKMMDLSLEAFTSIMNASGSYLSVLLAM-

>gi|383857329|ref|XP_003704157.1|

----------------LSITLSTFCMKYVGLWTAKDY-----A-QERRRKLGIAYSIYAVCLALFFEIRDLCFSWGDLNATLYVLCNLLSVSLTAFKVHVLVLYRTELAELIVYMQDHFWHCKYDEREKAILANCRKTCVFFTISVTCIGECAVVAYIVTPIVASL-------G-SNST----VRLHPFNMWYTTVLE-TPYFEFFFVVQVFILFHVGVCYFCFDNMFSIFSLHIATQFRILQYRFEIMCQIYEES--------------------------------------------------------------------------------------SDPANEKISPTNYAFEVYQKFKVYVQQHQALINYSKLLERVFTLITFGQVMVFSVLICLFGYQILLADSTPARRSVFICLTVGSTSLLFMFTYSCHGLMEQSDLLADAMYSTMWTRIPMMLRNDLMMVILRARRTCCITANGFFPVSLETFTTIFSTAASYFTLLRN--

>NvOr259PSE

---------------------KCLLCVLSGIWPDF---------HSIIQPMFGVFAAVVSLITVTAFLNFSIHHITNVNVLCKSFSLVISFFSTFLKICVFLZHHSDLVYLNTILNARFEEDTRNES---YRRFTLARVNIFTKTFYAVSISVGLATGTAFVLLII-----SLG--HG-----KYIMLYPSIFPFGYESGRLYWILLSIELFTNFFVCAVTSDXDSIFGMYTMQICREFRVFAHKFEIL-------------------------------------------------------------------------------------------------------LKTSENYRNDLKECVERHHELIRTKEVLHDVFGLLSIWLAIRSAVILCILIFQAIQVFKTMGGVIYIVGYIMLKLVQSFIYAWYGKLIADESENCIEAIYNARWAGGDARFMTDVIIVL--SQNPLVFRAKGCMSLKMDIFIKIFNTSVSYFFLLQTL-

>gi|383857771|ref|XP_003704377.1|

------------------------------------------------------FLCIIVSAITFLQLMDLVLIVENQAEFTDNIYISLSMIIACYKMYSILLSQKNIGVLTNILEDEPFQPKNEEEIEIRRKF-DNKAKQQAVLYTLLVESSVTLYVCVDILKF-----------------DDKKLLYRIWLPSELLP-SVHIFIYLQQSLSLIVGSIIHVACDSLIWGLLIHSCCQLEILEARLKAIKRDDDQSAKSS--------------------------------------------------------------------------------------------------------SSARYHNRVYRLVLMINQEFKMIIFVQFTASTLVVCFSLYIIATAKDMNDRLLVIAVYESCMLGQIFFYCWYGNEVKLKSVQLSDVIFGTDWMQFSRDTKKILSVIMRRATMSIEFTSVYIVTMNLESFVSLLKTSYSAYNMLQS--

>gi|332023365|gb|EGI63613.1|

-------------------PLNVRLNALSGLLPLRSD-----SRFPITWKIYSALIWLIEIIQTIALIPGLILVPRK-KALKDGTVLCVVTIEMFFMAVRIYSRRQLLNQLIQKLNDILRFS--DETMKNVVMTTLRPMENPLKFYWLSGWLGVFVWACLPFLFIFKKIFFFYE---------DYRMPAFSKQPFSLD---VFLLGSVLILIGNMYVFVKKVGMDVYMIHLVLLITAQYRYIAIKLAVIFRDGNSLSKLDESHQ----------------------------------------------------------------------------------SHQKYCSGINRWVKKEMIALCRHHSNIVHLSSMLKKLLSLNFSMIYVNSVLRFCFIGIMLSTTFVLDGGATRHVGIYLC--------VLVFYTLDSLSQQMTNKAFHEKWYQFGPSVKRTFMLMILGNNLECKLSMCDKFNLSLPSFMTILNQSYSIALLLLRV-

>gi|167866466|gb|EDS29849.1|

---------KSYEMYDYNLLFIRWMADFSGVDMLV-------DDYRFN--YRTAICVATVLMGAINCTYSLAYFYPNYYKMCEVVVLLGILIQGTPKLYFGYIHREFYKLQYGRLRLLHYRYRDHEKLNAKLLLLMERIHVLSKLLSIVFIFGGLGYSIYPLYMYWA----HH----------ELVLMVTMQVPWDADSYTGYTVTTAMQMVMIVIACTGLSASDTAILLFVANLSAYVEVFTDELNKLN-----------------------------------------------------------------------------------------------NAMLSAEDRDEEVIRCQVRKICSQHQDIIEYESDLDERYIVICFGLAIGAITTMTVSIFLAYMNS-----YIPGYALVVVGFFQLLEFCLLGTVLTVKNEEIIEAIYSTDWYLLAEPERRCFALMLHKSQNFTEMTIGGFAPLNLETFVAIMNRIYSYFMMLISF-

>381_Si_gnF.scaffold00330_12820-264637.pep

---------------------LKLGLRMIGMWPDS-------SCKTFFWLFYMITIVVMQYFQYLY--FFAQLGTNNFSKLMDGLSVTLDYTLTFLKLLSLWHNRRIFSDILSAMNDDWNDCSTDSHV-YVMTSKANLAHRCSNVMLVLNTLSTFFYFIGSFLSH---RTIS---KNGD----LREFPMQVQFPFDAATDPIFELIVLGLFLHVWETATVIALLNSLILTLVLHVSGQIDIMCQGLREISTTQKPL----------------------------------------------------------------------------------------------------------IRSLIVRHQRIISLSNNIDNFFSFVALIQFVWNTVVICSIGIMIMISEGKSGLLIQSIIPYIAVTLEAFVFCFAGEYLSTKSKSISDAAYDTIWYDLSISECRILLLIIIRSQKRLTITAGKVMDLTLEGFTTVMKASASYMSVLHAM-

>HsOr372

----------------YTLPLSFALLMYIGYRLVGC------SLTSIKYRPYNFYSAIMLLHSFAFCSLVDCILIEDLDIFIKRFSLFLSVLGVCCKVMNLXIRRDRIVDLTEILLNEICVPRNNYEANIQQRFDSAQSAYRRSLCEILNELAVFLATVSQ-FRYFA----------------NIHILLLSDVSYNITPMNNFWATMLH-TIGLMVAXNASVAHETLIFGFMLQTCSQLDILCHRARMLPNT-----------------------------------------------------------------------------------------------RRDCASKRNMKTHERIRKFVRHHRYIYGLAERINSVFAVMIFVQFAVNSTVLCLSIYKMST-KSLLSFEFXSLSYLGCVLMQIYLYRWFDNKISNSNAKIDTAVYEIDWEIFPVGLMKSLLLISKHSKKXSFRIMSHIIVLSNDSFMKIIKISYSTYNVLK---

>DeleOr69aA

------------------------------------R-----KPYRLEQKALFVLAVICAMYQIFGVIIYWYRNGRDVTEISEMCGSLMLTTVGFGNIYALIKPRNLIENMFEELEEIYPSQSDEHYR---CQLYYDLAMAIMKIEFMFYMVFYAYYNSAPVLLLLWENLQEG------QEL-SFKTQTNTWFPWKVHGSLGFGGAVLCLILGSFVGVGFSIATQNLIVIFTFQLKLHYDGLESKLLHLDSRQPNANQ-------------------------------------------------------------------------------------------------QPNANQQLRDLIAYHSRILHIGDQFNHILNFVFGTSLVGSTIAICMLSVAISL--LDAASAIKYVSGLTAFVLYHFVICYMGTEVT----------------------------------------------------------------------

>gi|383850691|ref|XP_003700915.1|

----AKTEQDYLKNVELSVQLNRWILKPIAAWPESSS-----PVKKYLGWFVHIICYSLLNFLFVPCFMYLILEVSDTYNRIKLVAPLSFFIMSYSKYTLLLLHKNDVLRCVKQIEWDWKNMKHLEEKRIMVMN-ANYARKFVIVCTFFMYSSFIFFYIAVPVAR--GRIEAPD-GNYT----YMSLPFPTSMRIDYRKSPVNEIFYFVQCVAGILLRVITVGACSLAATFAVHACGQMEILMNWLGYLVNERADMSKS----------------------------------------------------------------------------------------------DMSKSLDARITSIVEQHVRILKFLELIEKTLRYVSLVEFLGCTLMLCLCSYHAI-VEWSRNESISYAVLCVPFTFNIFIYCYIGELVADGCRQIAETSYMIDWYRLMGKKKLSMILIMQMSHSTNKLTAGNIVILSYTKFSDVVKSAFAFLNVLRTV-

>DeugOr1a

-------------------WTQRFTFARMGLDLVPKA-----KGRVLRSPALYGIMFVATGFELCTVCAFMVQHRNQIVLCSEALMHGLQMISSLLKMAIFLAKSHDLVALIQLIQQPFTVDSLGDSE---WKSQNRRGQLLAAIYFMMCAGTSVSFLLMPVALT----MLKYH-STG--DF-APVSSFRVXLPYDVTQPHIYALDCCLMVFVLSFFCCSTTGVDTLYGWCALGLSSQYRRLGQQLKWIQEHSDPS-------------------------------------------------------------------------------------------------------------LIVEHARLLRLVRRFNASFMEIAFVEVLVICVLYCSVICQYIM-PHTDQNFAFLGFFSMVVTTQLCIYLFGAEQVRLEAEGFSSQLYEIPWQSLSPQQRRFLLLPLQRAQRDTVLGAY-FFELGRPLLVWIFRTAGSFTTLLNA--

>CpluG2R504O03CUPD4

------------------------------------------------------------------------------------------------------------------------------------------------------------------------------------IV-KYPLIFPSKYPWQTSSNWAYKLTYLFESLATTSLVLITAGMDSLYLFYIFQI------------------------------------------------------------------------------------------------------------------------------------------------------------------------------------------------------------------------------------------------------------------------------------

>NvOr301FIX

----------------EYYHEVKLLLTYFGLWPNL---------SRFRKVVSFIAMVAMPISLVIPMGLKRAIRLKEPIQIIEDTIGILYFLAITTKYICIFIFEGRMIVVYEQIASDWKKIKDKNELEYL-HGRAKEGKIITILYLGYGAVGCTIFASTPYLPLFLDLVIPLN-VSR-----DKIYPYYADYVVDSEK-YFYTLYTLHGIFIIILVTMSAISIDCLFIMMVKHSVGLFQIVCYRLKKIGEEHNEKPH------------------------------------------------------------------------------------------------DDKIIHTRMKEIFDSHKSSIECVDAIQASFDVSFLFIMTMSGVGVSLILFDLLLNLDDLTQILRINSMMFGVYIAVFVICYAAQMTLNSSEIVFNDTYCGYWYNISPNARKYTQMVMVRSMKPCIITAGGLINMNLQSFFAILKTSVSYATVMLSM-

>TcOr64

------------DEYVKDVFINRWMLRCAGLWTPSTS-----KLVQIPYKIYAIVVFLFVNVYFTSTFLSLFYTHKNLYNFIKNVNFFLTHFMGAVKVIFWFFKGHVLRDLMRTLESPEFHYEPCEGFQLIWRKYRRIGFKYSLGFLALAHMTLSSSYIPPLLTK---------------------LPYFSWMPFSYSTPRSYLLALGYQAGPMFSYAYSIVGMDTLFMNIMNFIAAHLVILQGA-----------------------------------------------------------------------------------------------------------------MNNEMKRNCRHLQTILRVSEDLERVHRYLTLGQLTATLFILCTSLYLISTTPASSKQFYAELVYMVAMGFQLYLYCWFGNEVTLMASEIPVNVWKADWYDCDQSFKKSMIFTMTRMQKPIYMTVGKFAPLTLQTFVYILRTSYSIFAVIKNT-

>gi|332021179|gb|EGI61564.1|

------------------------MLKIIGLWPKEST-----RCKELLSKIQFLFNVIMIFVLTIPALMSLIRVWGDMILMIDNLQYTLPLLITMLKVFIMWYNKGALSPLIDMIVKDWIKVKMEEER-IVMLKQAKITRSLAICGVLMILSTLVITFGAFLFGK----TLRHV-TNFTDPV-GKHLPIQTYYPHDVSNSPNFELTYLIQVIGLTTSGLSYTAVDNFLGFLILHICGQMENLHLRLLNLGKNSN----------------------------------------------------------------------------------------------------KNSNFKALLKHNVKDHIRLIRSIETIDNTFNLMLLGLLFFFGILFCFDGFLIINVSKNNS--IAYIIF-----------------------------------------------------------------------------------------

>DficOr35a

------------------LAWPLALFRLNHIWPLD-P-----STGKCARYLDKFLAVLGCLIFMQHNLRYLRIRNRNLDDFLTGMPTYLILVEAQFRSLHILLHFEELRKFLQIFYANIYIDPRREPE--M-FKKVDGQMIINRLVSAMYGAVISGYLIAPVFSII----------NQ-----SKDFLYSMIFPFDSDPLHIFVPLLLSNVWVGIIIDSMMFGETSLLCELIVHLNGSYLLLKRDLEMAIQKI--------------------------------------------------------------------------------------------------RPQMAKQVKEQIIITIRKNVALNQFGQQLEAQYTVRVFIMFAFAAGLLCALSFKAYT---NPMANYIYAIWFGAKTVELLSLGQLGSNLAFTTDSLSSMYYLTHWEEINLRLLKLINLAIAMNSKPFYVTGLKYFRVSLQAGLKILQASFSYFTFLTSM-

>NvOr165JOI

------------------YKTYKDGMKLIGLWPFE---------NSTKRTLKRAFVLISMITTLIFMVRFVEELNHNIDVVLQSAGSIILSIGCIAKFITTFRAEDNIKLLFSRIAKQWASITDKTEC-KILADNIKVCHPLCTFYKVIAFFALVTYACLPSFGPVIMNILSPL--NETR---RKRLPAPAEYFVDEEK--YFYVLFSHGMICYMLVCALYVIIDCMYSCIVHHTVGLVGIVTYRLQNIIDLKVTSNQE-----------------------------------------------------------------------------------------NQEYQANNSEIRRRLRRAISLHKESLEFAQKIETTYNLCFIIVMSVNLLTMIFTAACAIRTYYDKVESFRWFILYGSIIFHLFFNSNPGQNLYDKSNEIINTLYFTEWYTLSRSNKRTMLIMMIRCLRPCQLTAGGLLVLNMINFGAIVKTSFSYITMLISL-

>gi|167868643|gb|EDS32026.1|

---------------------------LVGVCMFNA-------RVSRAQPA-FIWGIISLLLYVYLAFESTIWYRDNIDKLMMCITTHGFSLQMASKVYTFILNRNRVIEINE-INLKYFGTSGRKAQLECLKP-SAKIANILITMTIFGYVTLTSLIIAPVIYGL---IV-----------SKKVLPMGYEVV-HSTEWPGYIVNLLFQINCMIYVSLTTITSDGTFILFLMSAIGQIDAILGRLSEFSEQLKQ---------------------------------------------------------------------------------------------QLKQTDDDEQITGNLKQILRLHQHHQRYMRKLDDLFSTYFMIAIISLYFCMSVCLASF---------------------------------------------------------------------------------------------------------

>CfOr213

------------ITLEKVIAFLKVDLLFACCWPVSRT-----KFQIVCDRIFRVISSLHAILLMIELIYTIIYRTESIQMLMQSTCAVGILSEVPLQILLFTLQHDRLQVVIFQIENYYHQAKTEERN--VFQKYIDRYIYLYATTLGLITVGLFISFLDPLLRG------------------FDTFPLVIKYPFPIDRQLLRAIVYCHHMFGIYQIYC-QVSSNVFLAFLLWFTSARFEI----------------------------------------------------------------------------------------------------------------------YSDWKRCISEHQELLRFAQEISLSISYIILLSLGISTYSLVFGGVSIL-SRIPLSVKAKFFIVCVSSLLKVLLCAWPADYLMTISSDIGDAAYDSLWYKHGIDSQKMMLYILLRCQRPIIITVPGLLALTFQHYTSYISTAFSFLTTFR---

>DeleOr69aB

------------------------------------------VNRTLAQRFFFWFGALNLVYHNIGLIMCARFVDGSVSELTDIGAMLGFTTMGTLNLWKIMRCKPEIEKLMEEFEALFQQAKKRSYR---IQHYYEAHTRLIKKSVRFYAPSICYYNLLPIILMVVELLTDNK------QL-SYRIQSSAWYPWKVHGSSGFFAAVVCQAFSCQIDLGIIIFTQFLISFFGIQLEIQFDGLARQLEAIDA------------------------------------------------------------------------------------------------------------KEQLKHLLAYHIKLFNLADRVNHSLNFTFCVSFTVSILSMCFQGISVIM--GDLGPALKHMLGLFVFLVYNFSICRNGTHIXXXSDKVMPAAFYNNWYEGDLAYRKMILILMIRATKPYMWRTYKLAPVSITTYMAV---------------

>TcOr66

-----------------YLKVHLTVLQILGIDILPNE-----RIPQTLFYTYSVLLIATMVVFTTAECLDLVLNYEDIYKLTFGLCCCVTHVLGAAKMFLMLYLRKKLWGYFTTLENGIFKPNPAEEF-EIVTSAINMCKRQGYVFYVLTVGVTGLYAALANLPYDKHNYFD---GNVTVVVNTKQMPYATWTPFDYNDSPLYEIMFAFQIFSTTLYGFYIGAADAVICGFLMLIKAQFLIVKRELETLVERA--------------------------------------------------------------------------------------------------DDGTQVFVEKCANECVYHHQELIALCEHAEEDFCYLMLLQFISSLLIVCFQLFQLSTLSPGTFEFFSMACFLLFILFQLLCYCWHGNEVQFVSGELSRYAFSINWIIMRESPKKTLLLLMMRAQRPCYFTAGKFSLLSLQTFMTVVRGAGSYFMFLKQM-

>1_369_Si_gnF.scaffold04648_643308-783672.pep

-----------------VLQLTFKILTIVGCWRPQSS-----FYLSIIYDIYTVFMIILLYTFLVSQFLDIIWNVDNAEDFTENFYATLASVVSCSKMFSLLVNRKNINMLTNVLVERPYKPSEMDEM-RIRYKFDRHIYTNTLCYTILVETTCACITVTSLFTVF----------------KKGNLTYRAWLPYDYYSSIVFCLTYIHQLISLTAGSLVNVACDSLICGLLAHICCQIEILECRLSKVSNNHET------------------------------------------------------------------------------------------------------NNHETLRDCVRHHNSILEFAFKLNNKFRMTIAMQFVVSTLVVCSNLYQMTKSTDINASYLPLLLYMSCMLTQIFIYCWYGNEVKLKSTQLLTNIFAMDWVTMDRSLKRNLLLIMNRAVVPIEFTSAYVLSMNLDSFVGLLKTSYSAYNILKQV-

>NvOr278

---------FTKDDIEYYFDFIFKSLNTLDLKFSIST-----DEFKFRHKLPTIIGCLIGLIIFFLEIYFIRDALHNHTILIQIFSQVISNFQSISKVILIVYKVNKIQQILEKIGVLWKTYTPDEGNRAVLYNTLQRTLSICKIYYAVLIATVLIYYVQPIVNFVGQYGARNS-INHTYDY--SQTLVIIKLPFKVTQ-KRYFFVISQEAYLLYMSGVYWGCSDTFFACFTTQICYHFKILKYHTKAFFDEKNNNS------------------------------------------------------------------------------------------------EKNNNSRLNLVTLIKRHQELLRLCVLIEDVFSPIIFSTILFSAMNLCVNVIGVQETNGSYRQAGIYLFLFIITFSQILFYCAFAEATMEEAWSLADLAYNLEWTSKDYKLRYYIHVIILRAQKPFHFTAYGFFPIGIQKLTSIINASFSYYMMLQTV-

>6_378_Si_gnF.scaffold00330_12820-264637.pep

--------------------PVEIGLRLTGIWPNS---------LVLFRMLWTLVMGIVLIFQY--HYLLIHFSTEELPNLIDGLSTTLPYNLLFIKMIVLWVNNRIFNDVLKAMSNDWREYSGMYA---MI-DKAVLAHRCSKLTIGVYSTAVLLYSTASINF-------RKQ-SNDS----CRELLIKMELPFNFCESPVYEIVMWVQFVHLMAVASSIGMLDGLMVTLMLHIGGQIDLMRQEVEEICPNDD-------------------------------------------------------------------------------------------------------------RSLINKHQKIIAFTENIESLFSHIALMQFFSNTIIICCIGFLIVTSMGTDEMLIKTMFFYIAITLEAFIFCFAGEYLSNKSKTIGDAVYESVWYNLKPRDCRVLLFVIMRSQKRLTITAGKFMELSLQGFTNIVKASASYVSVLHAM-

>NvOr73PSE

-----------YNKNMQSIFITIILMRLLGMWYVSN------KREKIIADVLLGIVLASMIFSCMVQTNEFYHARKSLMLICSAAPTFFVLFSEQMKLLVFCKNRERVMKLNKYTYEHFWNGKYNRKETKIFNDCNSWCIKALACFLVTMQCISVHLMLAPYLEP--------S-DNSTSG--EKIFPFPVYVNYSIFETPTYEILYVLEVLGLYGVVLCVMSFPIFLLVTNMFTAVQFKMLNLRMRSLCQFPRNKND---------------------------------------------------------------------------------------NKNDDVNLQQQTNAYEKLKECIRKHQSLIHYVNEMENLYCYAMLGQILASIFQLSSTSITILLSEESLNKAVLRILILVASLMQFYFYAYSSHEILTESEKISEAIYSSDWYQTHPRYRKNFSLL-----------------------------------------

>PbOr235PSE

-------------WKSQYYLIPKTYMILTGLZPYY---------RLRNRYICFILTFISDTSILIPQLMYGL-AATNMNDIMENVPSVILSIIFSZKVLIIMFDTKKVKYCLQRIEKDWQLLXDIKR--IILQCHAEYGQYLMTSYAIFMHVVQICYLLKPIIPMLL---QNDI-TNSTKT--ISKLLYLVEYGMDIDQ--YFYPITIHCCLALFTHIFTSIAVDIXYI-----------------------------------------------------------------------------------------------------------YILEEVGKNNDANFQKLNKTDTDYKKALNCLRKHLQVIKFAESINSIFTKIFLISVNLNIICGSISDIQIILNLDNPGIILTPLALNTAQLXHLFIZFWQAQVLLDYSTVPWESXCRGNWYYISEKCRKLFLLIMSRTMSPCKITAGKIIILSIENFAAILKASMSZFAMLCSF-

>TcOr172

-------------------------------------------------------------------------------------------------------------------------------------------------------------------------------------------------------------------------------------------------------------------------------------------------------------------------------------------------------------------QDEVFKQLRDCISYHVALERWMARLIDLTKTAMPVFILLGALSSIAVSFFVLYSNTRFILKIRLTVVAICNVLIVATFAKAGQRFSDKTGLIFDAIATCPWYSWNVPNRKIVLIFMANCLKPKTFSWAGI-TLNYQFAIKIVRTSCSYALVLYKLR

>HsOr51

---------NHEKDVVDTLMWNRWLLRIIGIWPLVYT-----KIEKILATFAFALCWTVLALLLVLTSMYTFSDQSIMSEKMKMLGPLGYVFFSMLKYLFLVIRHKSIRGCVQVLSADWRMVQQGYHREIMIRE-AAKGHVLSKFCIMFMYCGGLSYNTVMPFLS---QTPESE-LNIT----VRPMAYGFDILFNLEFMPVYVFAFCLQCFTGVVMFNITTSVCCLAAMFVAHACGQIDIVIDRMENLMKGNEQ--------------------------------------------------------------------------------------------------CSRMKFDRCMAIIVQHHVRALRFSANIEDTLREICLVELVGTTLIMCLVEYSLITENSDRIAIFTYFFLLISFIFNIFVFCYIGELLTEQCTKIGHTSYMIEWYNLPGKAALDLMLMITMSRHPVHITAGRMISLSFANFGN----------------

>HsOr50

-----------EKDIVDALVWSKWILRILGIWPLVFS-----RVEKILATTSFALSWSALGFLLIPIAIFTLSDHTVTNDKVKMLGPLGHVLISMLKYLFLVVRHKSIRQCIRGLSFDWRAVQRENYRTIMMKD-SMKSHMLSKFCIAFMYCGGLSYVTVMPFLS---QKPDGE-KNAT----VGPVPYGFDIIFDLRFVPGYVFVFCMQCFSAVVMFNITTAVYCLAAMFVAHACGQIEIVMARVESFMKDVQSNRINS---------------------------------------------------------------------------------------------------EHCMAVIVKHHVKALRFSASIENILSEICLVEVVGSTLIICLLEYYFLMENSDSIAILAYSFLLTSFVFNIFIFCYISELLTDQYSKVGYTFYKIDWYLLPGKIALDFTLMISMSHHPIKITAGKLISLSFTSFGAVLKTSVAYLNLLRTV-

>HsOr53

--------VHYEHDIRYAMQICHWILKPIGIWHFVYS-----PSEKLFSSTLVFTCVSVLCFVLVPSGPYVLLYEKDIYMRVKLYGPVGFCLSCTIKYFLLGVRGTAIGRCIEQVEDDWQVIRQADHRKVML-KNAMVGRRLAILCVILLFSGGLSYHTIMPLSA----KIKIN-ENLT----IRSLVYPGYDRFNVQASPVYEIVFGMHCLSCLIQYTITTATCSLAAIFASHACGQVQILMTLLDDLVDGKK------------------------------------------------------------------------------------------------------STVGKRLRVIAKHHMRVLRFAADVEEVLREICLIELVAATLIICLLEYYFMMEKSDAIGIVTYFILLVSFAFNLLIFCYISELLVEEFRKIGSAAYNVNWYDLSGYKALDLTMIIMISHYPPRLTAGKFCDLSFNTFSTVLRTSLVYLNLLRTV-

>HsOr52

-------NVHHENDIHYTMQLCRWVLKPIGIWHLIYS-----HYDKLTSVILIVACLSALCFVLIPSGMHTLLREKDINVKVKLFGPVGFCLTSTIKYCYLGARSASLGKCIHHVEDDWRAVRDEDHRRIML-KNALTGRRLTTLCALFLYTGGLSYHTILPLSS------RRK-VNGSF--ISRPLTYPGYIFFDPEASPAYEIVFCIHCLFALITYNITTAACSLAAIFVTHACGQVQILMTLLDDLVEGDRNKGT--------------------------------------------------------------------------------------------------TTVDSRLGIIAKHHVRLLRFSTNVEEVLREICLMELVTSTLTICLLEYYCMTENSDAVAILTYFILLISFTFNILIFCYIGELLVEQCSKIGSAAYEVNWYDLSGNKAVDLVMIIAMSHYPPKLTAGKFVDLSINTFGVVLKSSVVYLNLLRTV-

>HsOr55

-------------DKDRNIRLIRWLLKSICLWPRSSN-----ASDRVFSELSRLTCFSLLFITVLSIGLDVFVEEHDVNSMMANIGPFITFLMALLKYTCLVLHVDDIRNCVHCIELDWNTVRSNKDHEVMLRD-GKIGRFMAIFIAAFMHSSVQSYNVSRCLKE----YIIEV-DNVNVS--LRELPYPFYSKLDARFSPAYEIVSIMHFVSSFIVSGVTSVNCGLMAIFVMHACGQLKILTLWLDDIVHDSDI---------------------------------------------------------------------------------------------------DDKIVQRKLGFIVEHHLRVISFVSYIEEAIYLTCLVELVGGSLTLCILGYSSITAQDETESILTYCVIALSFTLNIFIICYIAEILSEQYKRVGLATYMTEWYRLPPKTALGLLLINLRCNFNVNLNAGKMIELSLYTFGNVLKASVAYLNMLRQV-

>McOr2

--------NMSYDNFDYTVFFNILMYKIFGFWRPDDD-----MKREKLYNCYTLICTIIWLLFLASQYIFIITNIQNVDEVTATSFVTITFSINLIKMLAIYRNMNRIKQLIKDMNLPMFQAKCARHRDII--D---YTRIYTIFFYICLYFGNTFWTIVPFIGD------------------ERATLTHGWFPYNETKSVNYEITYVFQTTVSVWNTMLCLNLDTFTGSLLILIGLQCDLLCVTLENLGDFH----------------------------------------------------------------------------------------------LVNDKVKFSKTMTENLVVCIKHHKEIMRVSKDVEDIHRVSVFILFLGGALIMCCCLFQLSVVPIGSIEFFMLLFFLISILTEQFIYCWFGNEVIQKSSRILHSAYCTPWLDCDINFQKVLLQLMTQTYRPITLKAGGLFTISISVYISVIRTSYSYFTLLK---

>HsOr56
[truncated: 1,328,530 more chars]
